# Transcriptome analysis of rheumatoid arthritis uncovers genes linked to inflammation-induced pain

**DOI:** 10.1038/s41598-024-77212-0

**Published:** 2024-10-29

**Authors:** Bradford E. Hall, Khadijah Mazhar, Emma Macdonald, Margaret Cassidy, Megan Doty, Christian Judkins, Anita Terse, Stephanie Shiers, Saber Tadros, Sijung Yun, Michael D. Burton, Theodore J. Price, Ashok B. Kulkarni

**Affiliations:** 1 Functional Genomics Section, National Institute of Dental and Craniofacial Research, National Institutes of Health, 30 Convent Drive, Room 130, Bethesda, MD 20892 USA; 2 Department of Neuroscience and Center for Advanced Pain Studies, The University of Texas at Dallas, Dallas, TX 75080 USA; 3 Laboratory of Pathology, National Cancer Institute, National Institutes of Health, Bethesda, MD 20892 USA; 4 Predictiv Care, Inc, Mountain View, CA 94040 USA; 5 Neuroimmunology and Behavior Laboratory, Department of Neuroscience, Center for Advanced Pain Studies, School of Behavioral and Brain Sciences, University of Texas at Dallas, Richardson, TX USA; 6 Present Address: NIH Graduate Partnerships Program, Brown University, Providence, RI 02912 USA; 7 Present Address: U. Penn, Philadelphia, PA 19104 USA; 8 Present Address: Dartmouth, Hanover, NH 03755 USA; 9 Present Address: Millipore Sigma, Rockville, MD 20850 USA

**Keywords:** Rheumatoid arthritis, Pain, Transcriptomics, Inflammation, Neuroscience, Neurology, Rheumatology

## Abstract

Autoimmune diseases such as rheumatoid arthritis (RA) can promote states of chronic inflammation with accompanying tissue destruction and pain. RA can cause inflammatory synovitis in peripheral joints, particularly within the hands and feet, but can also sometimes trigger temporomandibular joint (TMJ) arthralgia. To better understand the effects of ongoing inflammation-induced pain signaling, dorsal root ganglia (DRGs) were acquired from individuals with RA for transcriptomic study. We conducted RNA sequencing from the L5 DRGs because it contains the soma of the sensory neurons that innervate the affected joints in the foot. DRGs from 5 RA patients were compared with 9 non-arthritic controls. RNA-seq of L5 DRGs identified 128 differentially expressed genes (DEGs) that were dysregulated in the RA subjects as compared to the non-arthritic controls. The DRG resides outside the blood brain barrier and, as such, our initial transcriptome analysis detected signs of an autoimmune disorder including the upregulated expression of immunoglobulins and other immunologically related genes within the DRGs of the RA donors. Additionally, we saw the upregulation in genes implicated in neurogenesis that could promote pain hypersensitivity. Overall, our DRG analysis suggests that there are upregulated inflammatory and pain signaling pathways that can contribute to chronic pain in RA.

Chronic pain affects about 20.5% of Americans^1^, where hyperalgesia and allodynia are often triggered by prolonged inflammatory responses. Inflammation is generally needed as part of the normal repair process to clear any cellular debris resulting from tissue injury, but aberrant immune responses can occur that produce chronic inflammation. Unresolved inflammatory signaling can promote adaptive changes in nociceptive neurons that lead to increased pain sensitivity. Chronic inflammatory disorders can include autoimmune diseases such as systemic lupus erythematosus, inflammatory bowel disease, and rheumatoid arthritis (RA), with pain being considered as a shared symptom amongst these conditions^2^. RA, in particular, is characterized as a prototype autoimmune disease with an overall prevalence in about 0.5–1% of Americans^3,4^.

In general, synovial tissues are richly innervated by sensory neurons, yet, despite the mechanical forces regularly encountered by our joints, normal movement is not perceived as painful unless there is injury^5,6^. The autoimmune character of RA, however, causes synovitis that leads to pain and swelling in the joints of the hands, wrists, feet, and knees^4^. The release of inflammatory cytokines along with acidification of the synovial fluid can then promote both peripheral sensitization of sensory neurons as well as the activation of mechanosensitive silent nociceptors^6–8^. The resulting joint pain affects not only mobility but can have an impact on quality of life as individuals with RA often experience depression and fatigue^9,10^. Interestingly, RA patients display increased pain hypersensitivity not only around the inflamed joints but also show hyperalgesia in non-inflamed tissues as well^11^. RA is mainly known to cause inflammation and pain in the small joints of the hands and feet but can also lead to TMJ arthralgia and higher orofacial pain intensity in some patients^12,13^. Pain can also occur despite the control of inflammation and joint damage using disease-modifying antirheumatic drugs (DMARDs)^14,15^. Along with typical reports of mechanical hypersensitivity, some RA patients additionally report symptoms akin to neuropathic pain including descriptions like burning, tingling, and electric shocks^10^.

To identify possible pain-related gene expression changes in the dorsal root ganglia (DRG) that occur with RA, we conducted RNA sequencing using the DRG of subjects with RA. The activity of nociceptors in the DRG play a key role in triggering pain, where the silencing of TRPV1^+^ neurons with resiniferatoxin in dogs and in patients with severe osteoarthritis (OA) has been shown to provide long-term analgesia, regardless of developing central sensitization^16,17^. Around 80–90% of RA patients report foot problems, so the L5 DRG was chosen for RNA-seq analysis as it contains the soma of the sensory neurons that innervate the joints in the ankle, and foot^18^. Overall, the DRG is a heterogeneous tissue comprised of not only sensory neurons, but also glial cells, perivascular cells, and resident macrophages. Bulk sequencing of the DRG was performed to identify gene expression changes in the primary afferent sensory neurons and non-neuronal cells that can influence pain sensitivity as well. In our RNA-seq analysis, we identified a total of 128 differentially expressed genes (DEGs) in the RA individuals versus the non-arthritic controls. We found changes in genes linked to immune activity that may reflect the autoimmune character of RA as well as pro-algesic neuroimmune signaling within the DRG. No signs of neuronal loss were histologically detected, while, instead, our transcriptome analysis uncovered the upregulation of genes linked to synaptic signaling and neurogenesis. Our RNA-seq analysis thereby looked specifically into the gene expression changes within the DRG of individuals with RA in order to provide initial insights into the mechanisms that can lead to pain hypersensitivity in sensory neurons.

## Results

### Neuroanatomical and metabolomic analysis of DRGs

Joint pain is a common symptom stemming from the inflammation and the autoimmune reactions that occur in RA patients, where arthralgia is typically an early indicator of RA and can often persist even with antirheumatic drug treatment that is used to prevent further joint destruction^14^. To examine the mechanisms involved in RA pain, we acquired DRGs from 5 individuals with RA along with 9 non-arthritic controls (Supplemental Tables 1 and 2). The DRG contains the soma of nociceptive neurons that relay pain from the periphery to the central nervous system (CNS). Until recently, there has been scarce availability of human DRGs for research, but now organ-donor networks can provide human DRGs for research^19,20^. Through these organ-donor networks, it is currently possible to acquire enough DRGs to statistically compare the gene expression changes between individuals with RA and non-arthritis controls.

**Fig. 1:**
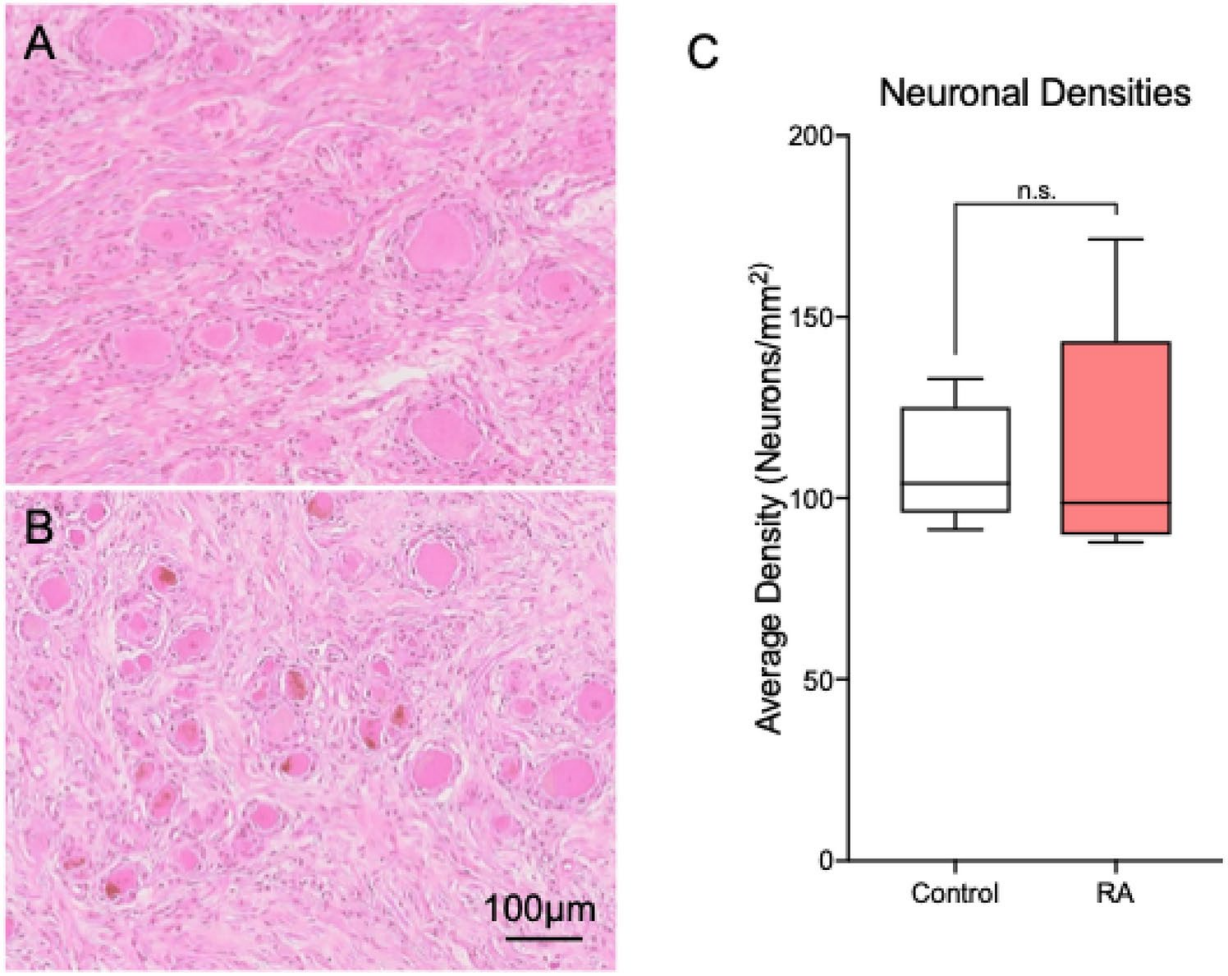
Histological examination of RA DRGs: Representative H&E images of (**A**) control and (**B**) RA L4 DRGs. No major pathological differences were identified between the controls and RA samples in terms of ganglionic cells nor inflammation (Supplemental Table 3). (**C**) No statistical differences were seen in DRG neuronal densities between the donors.

We examined the L4 DRGs histologically to check for any signs of neuronal pathology while performing transcriptomic analysis on L5 DRGs to identify gene expression changes that could lead to neuroplastic pain hypersensitivity (Fig. 1A,B).

Unlike our analysis of diabetic painful neuropathy (DPN), we did not see any indication of neuronal loss related to chronic joint pain (Supplemental Table S3)^21^. Overall, there were no significant differences in the average neuronal density between the RA subjects and the non-arthritic controls (Fig. 1C).

Along with the histological evaluation of the DRGs, we had a portion of the S1 DRG sent for metabolic analysis^22^ (Supplemental Tables S4–S6). DRGs from the donors were collected within 3 h post-mortem, and, as such, there were no clear indications of any tissue degradation such as autolysis in any of the DRG samples. Although control 1 and 9 did show the most variation amongst each detected metabolite (data not shown), none of the flagged metabolites were indicative of oxidation, degradation, or cell lysis. Our metabolomic analysis did, however, indicate that there may be increases in uric acid and 3-Aminoisobutyric acid (BAIBA). Increased uric acid has been associated with inflammation, metabolic disorders, and cardiovascular disease, yet a clear link between uric acid and these maladies is still considered debatable^23^. The increased BAIBA in the RA individuals, on the other hand, seemed counter to aspects of RA pathology as it is generally considered as an exercise-induced osteocyte survival factor^24^.

### Comparative transcriptomic analysis of DRGs from RA subjects and control

**Fig. 2:**
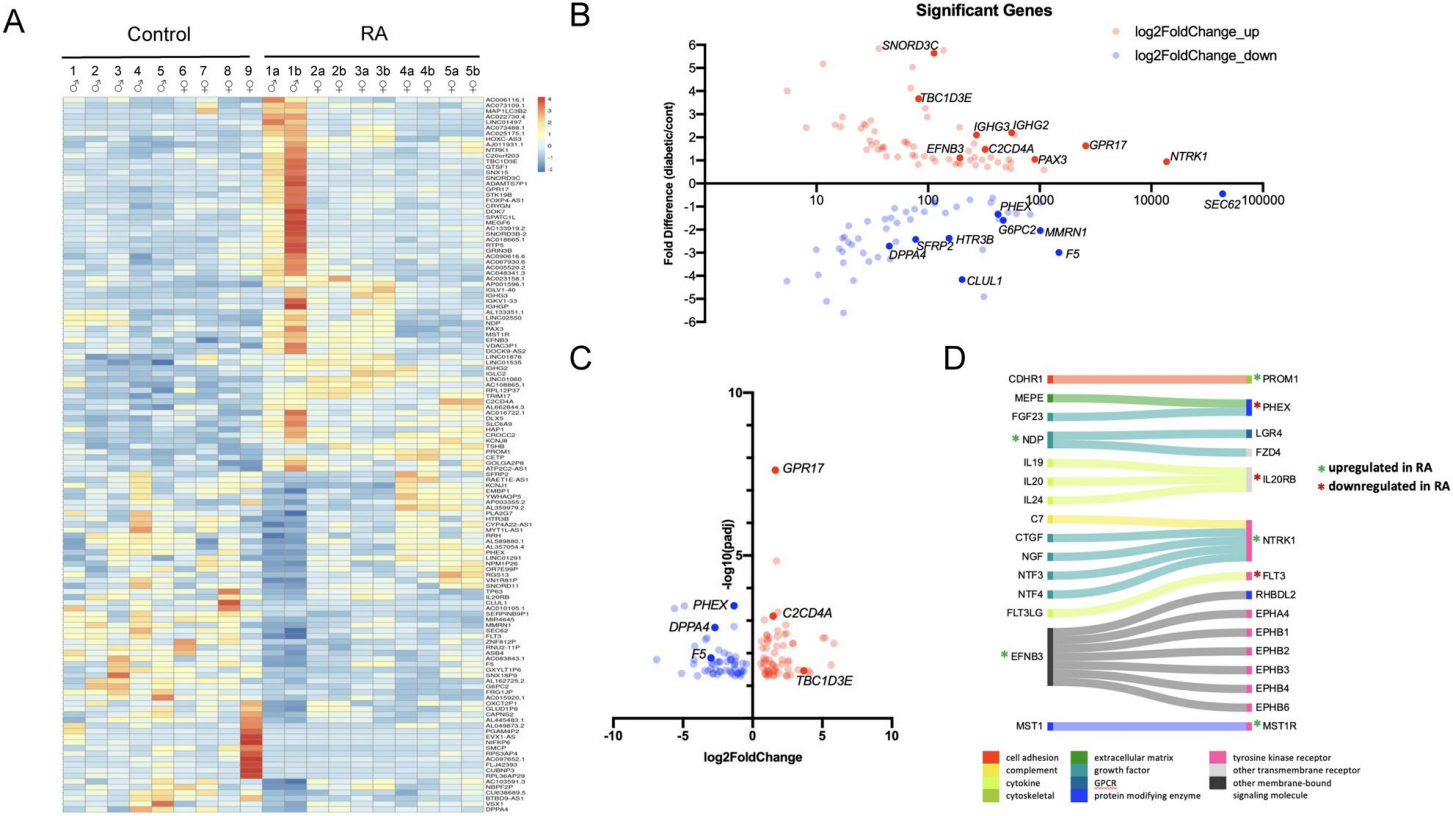
RNA-seq analysis identified 128 DEGs between RA and non-arthritic controls: (**A**) As seen in the heatmap (Z-score), 67 of these genes were upregulated while 61 were downregulated in the RA donors compared to the non-arthritic controls. Bilateral L5 DRGs (a & b) from the RA subjects were analyzed. Only one of the L5 DRGs from the non-arthritic controls was sequenced. (**B**) Scatterplot depicts base mean versus fold difference. (**C**) Plot of log2 Fold Change for each significant gene versus -log10 of the p adjusted value (see Supplemental Tables S7). (**D**). Ligand-receptor interactome data that depicts possible changes in cellular communication, particularly since our analysis showed that cell signaling seems most affected with RA.

For our transcriptome analysis, we purified RNA from bilateral L5 DRGs from the RA subjects to account for possible asymmetry in the presentation of symptoms^25^, while we only examined one of the L5 DRGs from the non-arthritic controls as no notable phenotypic dissymmetry in the DRG would be expected in those individuals. Gene expression changes were calculated with sex and age treated as covariates. Bulk RNA-seq of the L5 DRGs identified 128 differentially expressed genes (DEGs); 67 genes upregulated in the RA subjects while 61 genes were downregulated compared to the non-arthritic controls (Fig. 2A–C; Supplemental Tables S7–S11).

Of the DEGs, 41% (52/128) were protein-coding genes, 16% (20/128) were RNA genes (mostly long noncoding RNAs; Supplemental Fig. 1A), while the rest were either pseudogenes or have not been characterized.

Of the 52 protein-coding DEGs, almost half encode for either a secreted protein (n = 14) or a protein with a transmembrane helix (n = 10). Consequently, components of cell signaling (receptors and intracellular molecules) in the DRGs appear to be prominently impacted by RA, especially with the dysregulated expression of 9 cell surface receptors (Supplemental Fig. 1B–D). Cell signaling is vital for nociceptors in detecting tissue damage and/or cell stress. As such, we wanted to further explore ligand-receptor interactions within our data set. In Fig. 2D, we used ligand-receptor interactome data to map out potential changes in cellular communication^26^. Amongst the ligand-receptor interactions, the upregulation of *EFNB3* and *NTRK1* is noteworthy in that both have a role in axonogenesis, particularly as axonal degeneration has been seen in sural nerve biopsies of some RA patients^27,28^. Despite the overall increased expression in immune response genes in the RA donors (Supplemental Table S5), our interactome analysis additionally does show downregulation of *FLT3*, which is important for leukocyte homeostasis. Downstream of cell signaling, we identified 4 transcriptional regulators within our list of DEGs: *PAX3*, *DLX5, TP63,* and *VSX1*. *PAX3* and *DLX5* are both upregulated and are known to be involved in early neural crest cell specification, with *PAX3* implicated as a top transcription factor for at the least 3 of our DEGs (*SEC62*, *CLUL1*, and *HTR3B*; all downregulated)^29,30^.

### Upregulated neuronal genes in RA

**Fig. 3:**
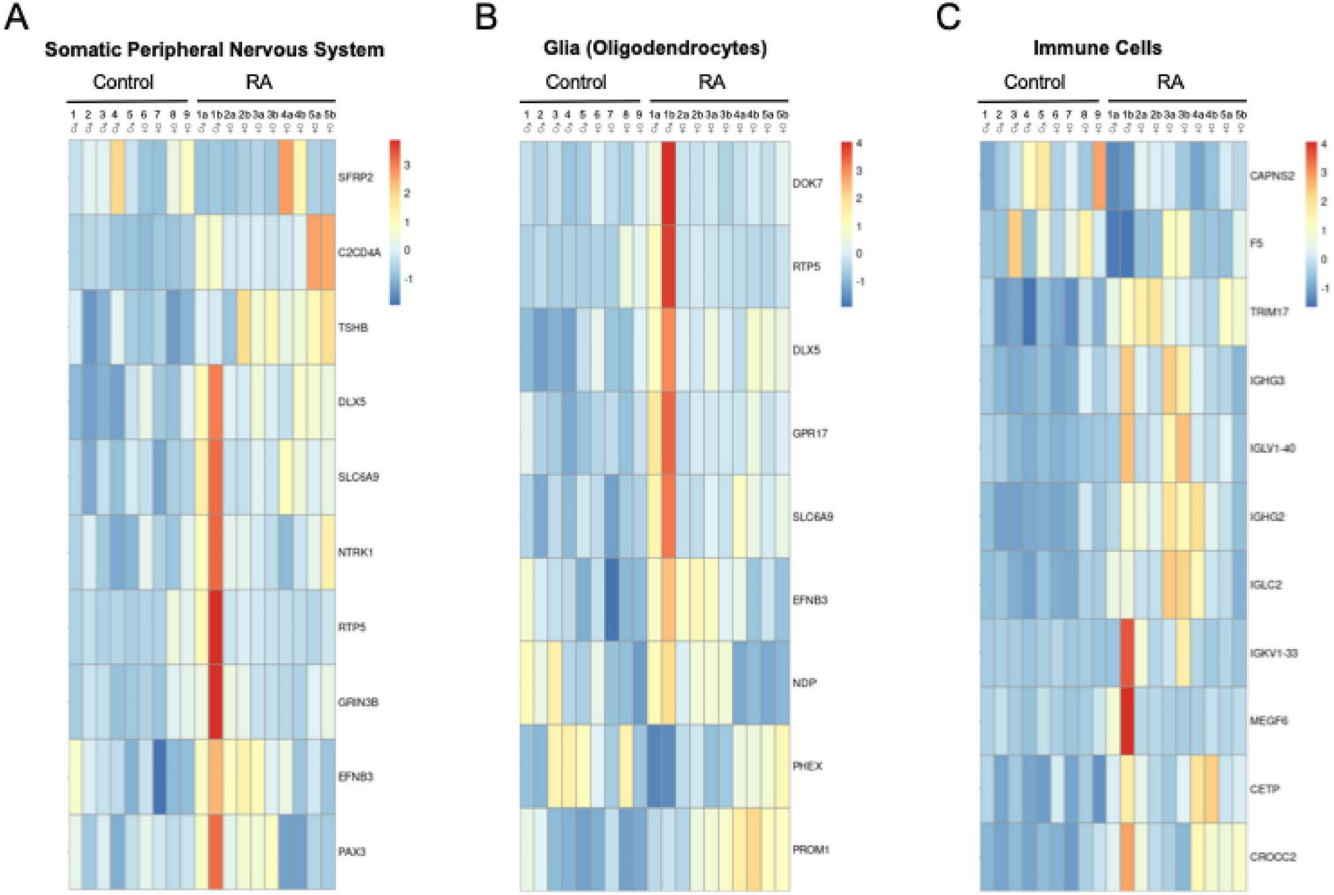
DEGs from bulk RNA-seq was analyzed using the ARCHS4 dataset to characterize how the different DRG cell types are affected by RA: Heatmaps were generated of genes associated with (**A**) peripheral neurons and (**B**) glial cells. Of note, there is some overlap in genes within each of these two cell types. (**C**) Signature genes for immune cells were also detected within the DRG in our transcriptomic analysis.

Next, we were able to break down our list of DEGs to identify signature genes related to the peripheral nervous system (PNS), glia (oligodendrocytes), and immune cells (Fig. 3A–C)^31^.

**Fig. 4:**
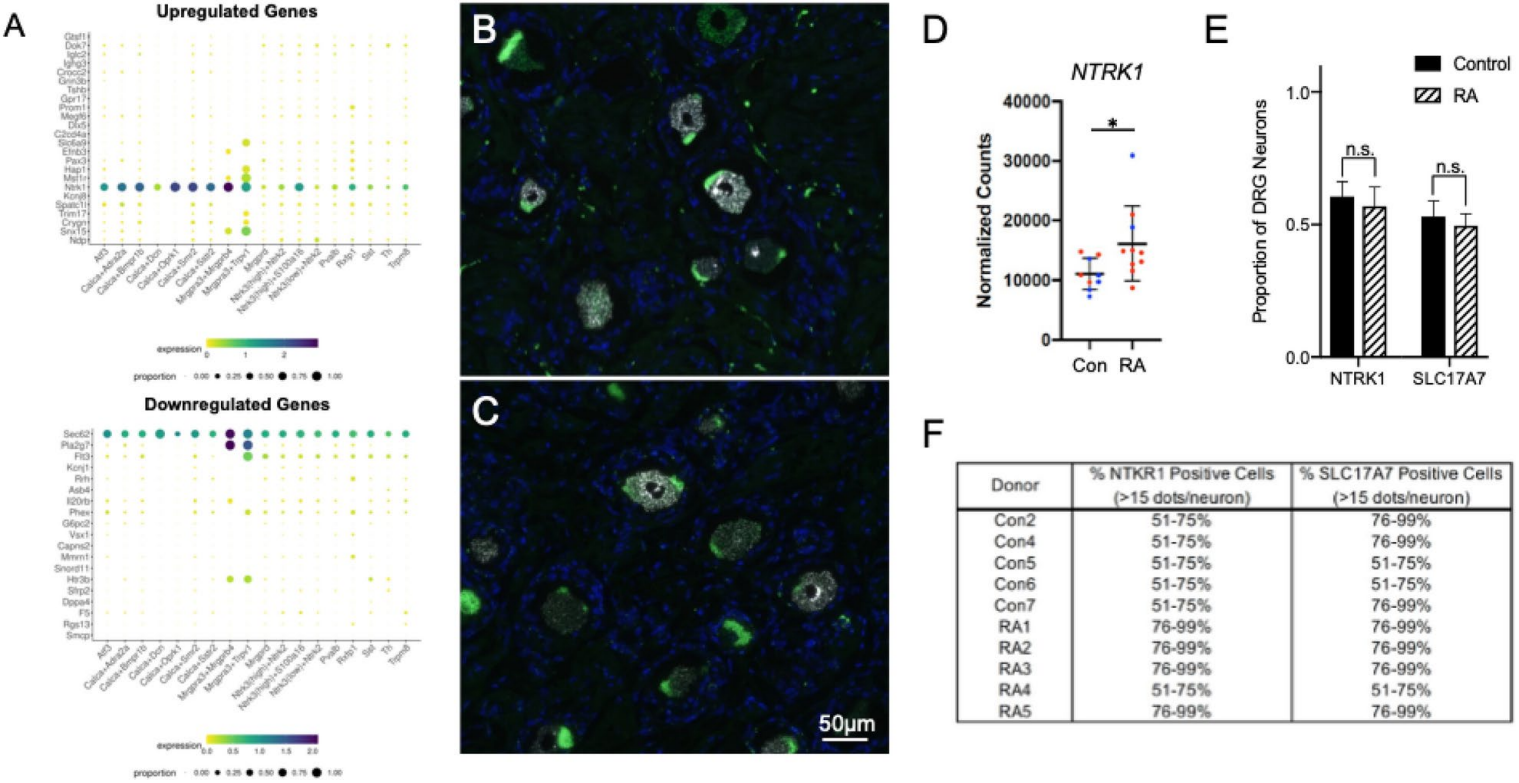
Characterization of dysregulated neuronal genes detected in the RNAseq analysis: (**A**) A harmonized cross-species DRG cell atlas was used to map out which neuronal subtypes could be most impacted with RA. RNAscope was performed on sections of (**B**) control and (**C**) RA DRGs to label NTRK1^+^ (White) and SLC17A7^+^ (Green) neurons (DAPI—Blue). *NTRK1* was upregulated in the DRGs of the RA donor and is generally expressed in unmyelinated C-fibers. *SLC17A7* was alternatively used as a marker for large, myelinated fibers. (**D**) Count plot for *NTRK1* (male donors—blue; female donors—red). Expression analysis of RNA-seq data shows that *NTRK1* was significantly upregulated in the RA donors. (**E**) While there was no significant difference in the proportion of either neuronal population, (**F**) semi-quantitative scoring shows that the percent of strongly expressing *NTRK1* cells (> 15 dots/neuron) is higher in the RA donors than the controls, which correlates with the transcriptomic data.

Unlike DPN, where neuronal loss consequently caused the decreased expression in neuronal markers^21^, most neuronal signature genes in the RA donors appear instead to be upregulated (Fig. 3A). For example, three genes related to neurogenesis were upregulated: *EFNB3*, *PAX3*, and *NTRK1*^32^. In addition, the glycine transporter *SLC6A9* was upregulated along with the neuronal gene for *HAP1*. *HAP1*, *SLC6A9*, *EFNB3*, and *NTRK1* are all linked with synaptic signaling, which again, differs from DPN where genes involved in synaptic function were predominantly downregulated. Lastly, we detected the upregulation of an uncharacterized protein-coding gene, *C20orf203* (FLJ33706), that is thought to be human specific and, more importantly, could be involved in neuronal functions^33^. Beyond these signs of altered neuronal activity, we also wanted to examine which specific neuronal subtypes could be impacted by RA using the harmonized cell atlas for the DRG (Fig. 4A)^34^.

The harmonized cell atlas dataset shows that RA-mediated joint pain would likely affect DRG neurons akin to the mouse non-peptidergic 2 (NP2) neuronal cluster. NP2 neurons are positive for both the chloroquine receptor Mrgpra3 (MRGPRX1 in humans) and TRPV1 and relay pain and itch, but Mrgpra3^+^ neurons are considered a rarer DRG subtype^35^.

The upregulated gene *NTRK1* codes for TrkA, the receptor for nerve growth factor (NGF) that is known to promote hyperalgesia^36^. Because of its clear role in nociception, we used RNAscope in situ hybridization to further characterize the expression of *NTRK1* in the DRG of individuals with RA. *NTRK1* is known to be mainly expressed in unmyelinated C-fibers^37^. As a contrast to *NTRK1*, we also wanted to label large diameter sensory neurons in the DRG using *SLC17A7,* mainly to serve as a control^21^ (Fig. 4B,C). There was no change in the actual number of NTRK1^+^ neurons, but semi-quantitative scoring shows that the percent of strongly expressing *NTRK1* cells (> 15 dots/neuron) was higher in RA, which is in line with its upregulated expression in the RNA-seq data (Fig. 4D–F). SLC17A7^+^ neuronal numbers were unchanged (Fig. 4E), while semi-quantitative counting shows possibly a slight difference in the percent of highly expressing *SLC17A7* neurons, (Fig. 4F).

### Immune response genes altered with RA

**Fig. 5:**
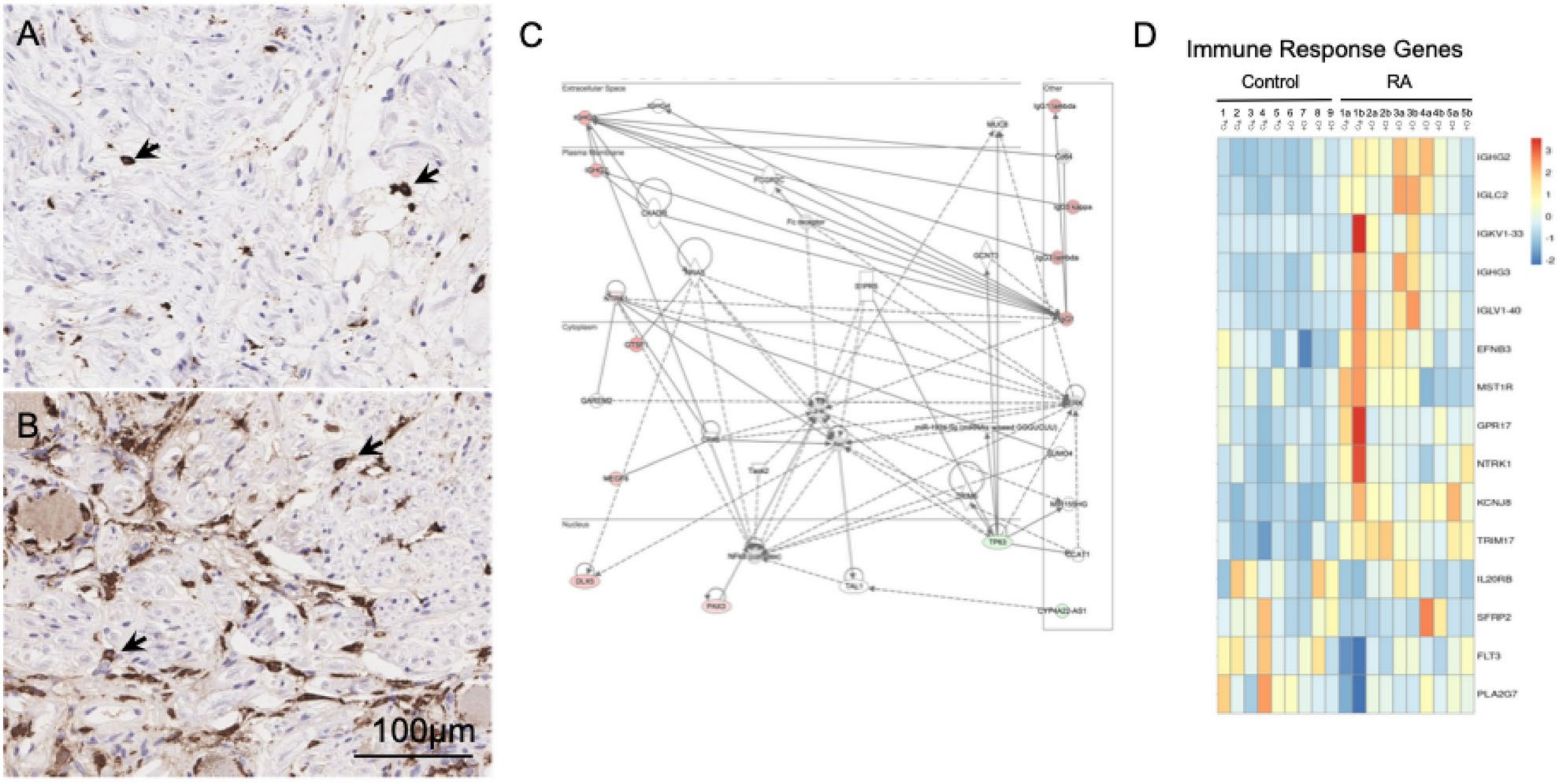
Indications of altered immune responses in the DRGs of the RA donors: CD163 immunostaining of (**A**) control and (**B**) RA DRGs. 3 out of 5 RA donors have > 50 CD163^+^ cells/high-power field (HPF; 40X) compared with just 2 out of 7 controls (Supplemental Table S3). (**C**) Network analysis on deregulated genes using Ingenuity Pathway Analysis (IPA) that identified immune response related networks are perturbed with RA, where IL-15 producing kinases such as MST1R and NTRK1 were upregulated. (**D**) Heatmap of DEGs involved in immune system processes (GO:0002376).

Sural nerve biopsies from some RA patients have shown indications of perivascular lymphomononuclear cell infiltrates and vasculitis^27^, so we decided to examine the DRGs from RA donors for any pathological signs of inflammation. While initial H&E staining showed lymphocytic foci within both the RA and control donors (Supplemental Table S3), we still stained for T cells and macrophages in the DRGs to test for any abnormal immune pathology. No differences were seen with immunostaining for CD3^+^, a pan T cell marker, between both groups. For macrophages, we performed immunohistochemistry for the scavenger receptor CD163. We focused on this receptor because CD163 labels a subset of peripheral macrophages known to reside in close proximity to sensory neurons, soluble CD163 is elevated in the sera of patients with RA, and synovial CD163 mRNA expression is linked to resting pain in patients with hip osteoarthritis^38–40^. With the CD163 staining, there is a trend toward increase numbers of CD163^+^ macrophages (Fig. 5A,B: 3 out of 5 RA donors have > 50 CD163^+^ cells/high-power field [HPF; 40X] compared with just 2 out of 7 controls, although the number of donors tested is too limited to be conclusive).

This increase may possibly be echoed by the upregulated expression of the *MST1R* (Recepteur d’Origine Nantais/Macrophage-Stimulating Protein Receptor) in the RA donors, which promotes M2 macrophage polarization^41^. Importantly, DRG macrophages are known to respond to damage-associated molecular patterns (DAMPs) and are linked with promoting neuropathic pain^42^. Additionally, there was also moderate fibrosis, possibly related to inflammation, in 2 RA individuals that concomitantly had higher numbers of CD163^+^ cells, but, again, our sample size is too small to determine if the fibrosis is linked with the increase in macrophages.

Due to the autoimmune nature of RA, we unsurprisingly identified genes related to immune responses in our transcriptomic analysis (Fig. 5C,D). For example, a total of 5 immunoglobin genes were detected to be upregulated in the DRGs of patients with RA. 4 of these 5 immunoglobulin genes, (*IGHG2*, *IGHG3*, *IGLC2*, and *IGKV1-33*) also showed increased expression in DPN as well^21^. Sensory neurons lack the machinery to secrete antibodies, which suggests that there could be activated B-cells within the DRG, yet only faint staining for CD20 and CD79a was seen^43^ (data not shown).

Other immune features detected in our transcriptome analysis include the upregulation of *C2CD4A*, which plays a role in vascular permeability during inflammation, and the glial gene *GPR17*, which may be indicative of damage to the myelin sheath^44–46^. Also of note, there was upregulation of *MEGF6*, a gene that has been associated with SAPHO syndrome (Synovitis, Acne, Pustulosis, Hyperostosis, and Osteitis)^47^. Lastly, *IL-20RB* was downregulated in the RA subjects even though IL-20R cytokines are typically upregulated in the synovial fluid of RA patients^48^. IL-20RB knock down in T-cells can lead to increased expression of IL-2 and IFN-γ^48^. IL-20Rβ (IL20RB) forms a heterodimer signaling complex with IL-20Rα (*IL20RA*), which is interestingly downregulated with DPN^21^.

The DRG is densely vascularized with capillaries that are fenestrated^49,50^. As seen in Fig. 3C, we detected signature genes indicative of immune cells within the DRG. For example, single cell sequencing in mice has identified macrophage, neutrophil, and B cell clusters within the DRG^38,51^. Other genes identified in our RNA-seq analysis can also be expressed by multiple cell types like immune cells^52^. We thereby wanted to clarify which DEGs identified in our bulk sequencing were localized to the DRG and which may originate from circulating immune cells. Unfortunately, blood samples were not collected during organ retrieval, so, we resorted to running a small bulk RNA-seq analysis using peripheral blood mononuclear cells (PBMCs) from a separate cohort of RA patients and healthy controls (6 individuals per group) and looked for any overlap in the DEG datasets (Supplemental Tables S12–S14, Supplemental Figs. 2 and 3). We decided to run this small bulk RNA-seq experiment as most reported transcriptomic data of PBMCs from RA patients were either generated using microarrays or used FACS analysis to look at specific immune cell subsets^53^. Only 8 genes were common to both datasets, even with a total of 1,468 DEGs uncovered in the PBMC RNA-seq analysis. Of the overlapping genes, only 3 out of 8 were dysregulated in the same direction, which suggests that the DEGs identified in the DRG of RA patients mostly likely reflect gene expression changes occurring within the resident DRG cell populations rather than from circulating immune cells. Although there was increased expression of immunoglobulin genes in both datasets, only *IGLV1-40*, which encodes a λ light variable region, was upregulated in both PBMCs and DRGs of RA subjects. The expression of *SNX15* was upregulated in both the DRG and PBMC data sets as well, which may be symptomatic of disrupted protein trafficking to recycling endosomes^54^. Lastly, Sec62, an ER-phagy receptor, was downregulated in both the DRG and PBMC data sets, where decreased expression may cause reduced ER stress tolerance^55^.

## Discussion

RA is considered an archetypal autoimmune disorder with pain often rated as one of the top therapeutic priorities by patients^56,57^. As such, we decided to perform transcriptomic analysis using DRGs from 5 RA donors and 9 non-arthritic controls in order to better understand the mechanisms that promote pain hypersensitivity. A key objective to our transcriptomic study was to identify potential disease mechanisms that promote peripheral sensitization of DRG neurons in autoimmune disorders, particularly since pain can persist in RA patients even while joint damage is being treated by DMARDS^2,57^. In our analysis of RA-mediated inflammatory pain, the upregulation of *NTRK1*, which encodes TrkA, is notable as NGF is known to promote hyperalgesia^36^. Inhibition of TrkA is also known to alleviate pain in rodent models of carrageenan and collagen-induced arthritis^58^, but clinical trials to treat osteoarthritis pain with tanezumab, a monoclonal antibody against NGF, were discontinued because of a higher risk of joint damage despite reduced pain^59^.

The overexpression of immunoglobulins seen in our transcriptomic analysis could impact pain hypersensitivity. Anticitrullinated protein antibodies (ACPA) are common in most patients with RA, and Wigerblad et al.^14^ showed that the injection of ACPA into mice causes mechanical and thermal hypersensitivity. ACPA did not act directly on sensory neurons to increase their excitability, rather, it caused hyperalgesia by prompting osteoclasts to release chemokines. Wang et al.^60^ also reported that FcγRI, a receptor for IgG immune complex (IgG-IC), was expressed on a subset of joint-innervating sensory neurons and that IgG-IC activated these sensory neurons via FcγRI signaling. These and many other reports^61,62^ suggest that autoantibodies can promote pain even despite controlling for joint damage using DMARDs.

Besides the immunoglobulin genes that are shared between RA and DPN, we identified 4 additional key genes that are dysregulated in the same direction in these chronic pain disorders as well (Supplemental Fig. 4). Of these, *G6PC2* of the glucose-6-phosphatase (G6Pase) catalytic enzyme system is downregulated and is interestingly a major target for cell-mediated autoimmunity in diabetes^63^, yet *G6PC2* is mostly known to be expressed in pancreatic islets and its role in the DRG needs to be further studied. The three remaining shared genes between RA and DPN are all upregulated. This includes *KCNJ8*, which codes for the potassium inwardly rectifying channel Kir6.1. Kir6.1 is one of two K_ATP_ channels considered to act as metabolic sensors to regulate responses to hyperglycemia, hypoglycemia, ischemia, and hypoxia^64,65^. *KCNJ8* is expressed in contractile cells including pericytes within the DRG^34^. *KNCJ8* may have a role as a “molecular rheostat”, possibly controlling blood flow to the DRG^66,67^. Increased expression of *TRIM17* is also of interest as it is considered a putative stress sensor^68^. Generally, *TRIM17* is expressed at low levels but it can be induced during cellular stress. Most studies on *TRIM17* have shown that it can initiate neuronal apoptosis^69^. This somewhat contrasts with some of our other findings, though, as we see upregulation in genes implicated in neurogenesis in the RA donors and a lack of any visible neuronal loss. Lastly, the gene for *HAP1* is upregulated, which codes for the Huntington-associated protein 1. HAP1 is found to be expressed in small and medium diameter DRG neurons and can be upregulated in the DRG of rodent models of neuropathic pain^70^. *HAP1* deficiency (HAP1 ± mice) reduces mechanical allodynia and heat hyperalgesia while also attenuating the hyperexcitability of DRG neurons in these neuropathic pain models.

In conclusion, here we provide a first of its kind study examining gene expression changes within the DRGs of humans with RA. We identified dysregulated genes in the RA subjects related to immune processes and neurological functions. Progress has been made in defining the human “nociceptome”^71^, but further research is also needed to identify and potentially target maladaptive transcriptomic changes that arise from inflammatory pain. While some of the gene expression changes in our RA study could be expected, we have still uniquely identified dysregulated genes of interest that warrant further investigation.

## Methods

### Human dorsal root ganglia preparation

DRGs from 5 individuals experiencing RA and 12 non-arthritis controls were acquired from Anabios (San Diego, CA), all obtained with the informed consent from all the participants (Table 1).

**Table 1:** Demographics on the DRG donors used in this study.

Donor	Sex	Age	Race	Height (cm	Weight (kg)	BMI	Diabetes	RNAseq	Metab	H&E
Control 1	M	29	Caucasian	185	56.5	17	Type I 5yr	✓		
Control 2	M	45	Caucasian	166	70.2	26		✓	✓	✓
Control 3	M	47	Caucasian	183	95.5	29		✓	✓	
Control 4	M	54	Caucasian	183	80.2	24		✓	✓	✓
Control 5	M	55	Caucasian	190.5	77.1	21		✓	✓	✓
Control 6	F	30	Hispanic	157	49	20		✓	✓	✓
Control 7	F	39	African American	159	63.1	25		✓	✓	✓
Control 8	F	40	Caucasian	161	65.8	25	Type II 4 yr	✓		
Control 9	F	49	Caucasian	167.6	103.5	37		✓	✓	
Control 10	F	51	African American	180	112	34				✓
Control 11	F	52	Caucasian	166	98.1	36				✓
Control 12	M	46	Hispanic	163	61.8	23			✓	
RA1	M	48	Hispanic	168	83.1	29		✓	✓	✓
RA2	F	53	Caucasian	160	105	41		✓	✓	✓
RA3	F	57	Caucasian	167	135.6	49		✓	✓	✓
RA4	F	61	Caucasian	165.1	88	32		✓	✓	✓
RA5	F	66	Caucasian	152	91	39	Type II 5yr	✓	✓	✓

BMI = Body Mass Index, COD = Cause of Death.

Approval for carrying out these studies was obtained from the National Institutes of Health (NIH) Office of Human Subjects Research Protection (OHSRP). All methods were performed in accordance with the guidelines and regulations approved by NIH Biosafety Committee, Bethesda, MD, USA. L4, L5, and S1 DRGs were chosen as these ganglia contain the soma of the primary afferent neurons innervating the joints within the foot (L5 DRGS were used for RNA sequencing, L4 DRGs were used for immunohistochemistry, and ~ 50 mg of the S1 DRGs were used for metabolomic analysis^22^ [see supplemental methods]). DRGs were collected under cold ischemic conditions, generally 3 h post cross-clamp of the aorta. Connective tissue and the dura mater were removed from the DRGs and the nerve roots and rami were trimmed. DRGs were then either snap frozen or stored in RNAlater (Ambion, Austin, TX).

### RNA-seq analysis

L5 DRGs from 5 RA patients were compared with 9 non-arthritis controls. The 5 RA donors include 4 females and 1 male (Age: 57 ± 3.1 SEM; BMI: 39.4 ± 3.4 SEM). RA is generally considered to cause symmetrical joint damage but can be asymmetrical early in the course of the disease^25^. We therefore opted to sequence both L5 DRGs from the RA donors to compensate for this probability. Left and right DRGs were each sequenced separately and were not pooled. A single DRG, however, was judged to be adequate for the 9 non-arthritis controls, as typically no major gene expression differences would be anticipated between adjacent L5 DRGs in the non-arthritic individuals. For the controls, there were 5 males and 4 females in total (Age: 47 ± 3.1 SEM; BMI: 21.1 ± 1.9 SEM). The controls were donors with no known history of chronic pain such as osteoarthritis, back pain, knee pain, etc. Since the incidence of chronic pain can often increase with age, the donors without pain were statistically younger than the RA donors, with about a 10-year age difference (unpaired t-test, *p* = 0.01). To account for this difference, we factored for age along with sex as a covariate during gene expression analysis with DESEQ2 (see below). The control donors were generally listed as either having a stroke (CVA) or head trauma. As organ donors, only a limited patient history was available. The medical history about the donors is obtained from the next of kin through an extensive interview with family members by a trained interviewer (Supplemental Table S1). RNA from the L5 DRGs was extracted using a RNeasy Midi Kit (Qiagen, Valencia, CA) as previously described^21^. RNA quality was scored using a Bioanalyzer (Agilent, Santa Clara, CA) and RINs between 7 and 9 were obtained (Supplemental Table S4), indicating some moderate degradation.

Bulk RNAseq of peripheral blood mononuclear cells (PBMCs) from RA patients was conducted in order compare the immune related gene expression changes within the DRG to the aberrant autoimmune reactions present in circulating immune cells. Blood samples from the DRG organ donors were not available, so the PBMCs from controls and RA patients were alternatively purchased from Precision For Medicine (Frederick, MD) and STEMCELL Technologies (Cambridge, MA). Mononuclear cells from whole blood were isolated using of a density gradient media. PBMCs were pelleted (400xg) and RNA was extracted using the RNeasy Plus Universal Mini Kit (Qiagen, Valencia, CA), according to the manufacturer’s protocol. Information about the PBMCs along with the RNA integrity number (RIN) from each sample can be found in Supplemental Table S12. Libraries were prepared and samples were sequenced at the NIH Intramural Sequencing Center (Rockville, MD) while RNA-seq analysis was performed as described for the DRGs and as detailed in Hall et al., 2022. PBMC transcriptomic data is available in Supplemental Tables S13 and S14.

Libraries were prepared and samples were sequenced at the NIH Intramural Sequencing Center (Rockville, MD) as previously described^21^. Briefly, libraries were generated using Illumina TruSeq mRNA Sample Prep Kit (polyA + method) (San Diego, CA) and sequenced with an Illumina NovaSeq-6000. For the human DRG RNA-seq, paired-end sequencing was performed with read length of the 151 bp. Read quality was checked using FASTQC version 0.11.6. Trimming was performed using BBTools version 38.42 to trim 20 bp off from 5’-end and 30 bp off from 3’-end. The alignments were performed using STAR version 2.7.2a to the hg38 reference human genome and Gencode release 27 for transcriptome annotation. Read counts per gene per sample were quantified using HTSeq version 0.9.1. Supplementary Table S11 shows alignment statistics and the total number of reads mapped to genes per sample. A list of differentially expressed genes (DEGs) between the RA and the control samples was generated using DESEQ2 version 1.24.0^72^. Sex and age were treated as covariates when building the generalized linear model for DESEQ2 analysis, hence, removing unwanted variation due to these factors. With an adjusted p-value cutoff of 0.05 by Benjamini Hochberg’s False Discovery Rate (FDR), differential expression analysis returned 67 upregulated genes and 61 downregulated genes in the RA subjects compared to the non-arthritic controls.

Functional data of the DEGs was analyzed using GeneCards^30^, and ToppGene^32^. Cell type expression data was determined using the harmonized cell atlas for the DRG^34^ and with the All RNA-seq and ChIP-seq sample and signature search (ARCHS4) dataset within Enrichr^31^. All 128 dysregulated genes were also entered into Ingenuity Pathway Analysis (IPA) (Qiagen, Valencia, CA) to identify enriched pathways and potential upstream regulators. Heatmaps were generated with the R package Pretty Heatmaps and show the Z-Score of normalized DESEQ2 gene counts. The interactome analysis was generated from data in Wangzhou et al.^26^ using SankeyMATIC.

### Histology, immunohistochemistry, and RNAscope analysis

For histological analysis, frozen L4 DRGs were temporarily held at −20 °C for cutting purposes, sliced in half, and immediately placed in fixative (10% buffered formalin). The DRGs were then paraffin embedded and 6 μm sections were cut. Slides were stained with H&E for general histological evaluation. Immunohistochemistry was conducted by Histoserv (Germantown, MD). Immunostaining for T cells using CD3 was performed as previously described^21^, while DRG macrophages were labeled with CD163 (MRQ-26; Millipore Sigma, St. Louis, MO) according to the manufacturer’s protocol. Formalin fixed paraffin embedded (FFPE) sections from the L4 DRG were used for in situ hybridization (5 controls and 5 RA donors). SLC17A7 and NTRK1 were labelled with RNAscope® technology by Advanced Cell Diagnostics, Inc. (ACD, Newark CA) as previously described^21,73^. Paired double-Z oligonucleotide probes were designed against Hs-NTRK1 (cat no. 402638) and Hs-SLC17A7 (cat no. 415618). Visual, semi-quantitative scoring of both NTRK1 and SLC17A was performed by ACD. Briefly, dots correlate to the number of individual RNA molecules and neurons were visually scored on a 0–4 scale (0 being no staining while 4 represents cells with > 15 dots).

## Supplementary Information

Supplementary Information.

## Data Availability

All datasets are available through the dbGaP with accession code—phs002548.v2.p1.

## References

[1] Yong, R. J., Mullins, P. M. & Bhattacharyya, N. Prevalence of chronic pain among adults in the United States. *Pain* **163**, e328–e332 (2022). 33990113 10.1097/j.pain.0000000000002291

[2] Lacagnina, M. J., Heijnen, C. J., Watkins, L. R. & Grace, M. Autoimmune regulation of chronic pain. *Pain Rep.* **6**, e905 (2021). 33981931 10.1097/PR9.0000000000000905 PMC8108590

[3] Trouw, L. A., Rispens, T. & Toes, R. E. M. Beyond citrullination: Other post-translational protein modifications in rheumatoid arthritis. *Nat. Rev. Rheumatol.* **13**, 331–339 (2017). 28275265 10.1038/nrrheum.2017.15

[4] Sparks, J. A. Rheumatoid arthritis. *Ann. Intern. Med.* **170**, ITC1–ITC16 (2019). 30596879 10.7326/AITC201901010

[5] Mapp, P. I. et al. Substance P-, calcitonin gene-related peptide- and C-flanking peptide of neuropeptide Y-immunoreactive fibres are present in normal synovium but depleted in patients with rheumatoid arthritis. *Neuroscience* **37**, 143–153 (1990). 1700840 10.1016/0306-4522(90)90199-e

[6] Bingham, B., Ajit, S. K., Blake, D. R. & Samad, T. A. The molecular basis of pain and its clinical implications in rheumatology. *Nat. Clin. Pract. Rheumatol.* **5**, 28–37 (2009). 19098926 10.1038/ncprheum0972

[7] McDougall, J. J. Arthritis and pain. Neurogenic origin of joint pain. *Arthritis Res. Ther.* **8**, 220 (2006). 17118212 10.1186/ar2069 PMC1794504

[8] Prato, V. et al. Functional and molecular characterization of mechanoinsensitive “silent” nociceptors. *Cell Rep.* **21**, 3102–3115 (2017). 29241539 10.1016/j.celrep.2017.11.066 PMC5751884

[9] Harth, M. & Nielson, W. R. Pain and affective distress in arthritis: Relationship to immunity and inflammation. *Expert Rev. Clin. Immunol.* **15**, 541–552 (2019). 30669892 10.1080/1744666X.2019.1573675

[10] Walsh, D. A. & McWilliams, D. F. Mechanisms, impact and management of pain in rheumatoid arthritis. *Nat. Rev. Rheumatol.* **10**, 581–592 (2014). 24861185 10.1038/nrrheum.2014.64

[11] Edwards, R. R. et al. Enhanced reactivity to pain in patients with rheumatoid arthritis. *Arthritis Res. Ther.* **11**, R61 (2009). 19413909 10.1186/ar2684 PMC2714104

[12] Mesic, V. F. et al. Characteristics of temporomandibular disorders and orofacial pain in individuals with rheumatoid arthritis. *Int. J. Prosthodont.* **36**, 630–636 (2023). 36484668 10.11607/ijp.8145

[13] Sodhi, A., Naik, S., Pai, A. & Anuradha, A. Rheumatoid arthritis affecting temporomandibular joint. *Contemp. Clin. Dent.* **6**, 124–127 (2015). 25684928 10.4103/0976-237X.149308 PMC4319332

[14] Wigerblad, G. et al. Autoantibodies to citrullinated proteins induce joint pain independent of inflammation via a chemokine-dependent mechanism. *Ann. Rheum. Dis.* **75**, 730–738 (2016). 26613766 10.1136/annrheumdis-2015-208094 PMC4819624

[15] Sarzi-Puttini, P., Zen, M., Arru, F., Giorgi, V. & Choy, E. A. Residual pain in rheumatoid arthritis: Is it a real problem?. *Autoimmun. Rev.* **22**, 103423 (2023). 37634676 10.1016/j.autrev.2023.103423

[16] Iadarola, M. J., Sapio, M. R., Raithel, S. J., Mannes, A. J. & Brown, D. C. Long-term pain relief in canine osteoarthritis by a single intra-articular injection of resiniferatoxin, a potent TRPV1 agonist. *Pain* **159**, 2105–2114 (2018). 30015705 10.1097/j.pain.0000000000001314 PMC8121156

[17] Szallasi, A. Resiniferatoxin: Nature’s precision medicine to silence TRPV1-positive afferents. *Int. J. Mol. Sci.* **24**, 15042 (2023). 37894723 10.3390/ijms242015042 PMC10606200

[18] Stolt, M., Suhonen, R. & Leino-Kilpi, H. Foot health in patients with rheumatoid arthritis—A scoping review. *Rheumatol. Int.* **37**, 1413–1422 (2017). 28324133 10.1007/s00296-017-3699-0

[19] Tavares-Ferreira, D. et al. Spatial transcriptomics of dorsal root ganglia identifies molecular signatures of human nociceptors. *Sci. Transl. Med.* **14**, eabj8186 (2022). 35171654 10.1126/scitranslmed.abj8186 PMC9272153

[20] Nguyen, M. Q., von Buchholtz, L. J., Reker, A. N., Ryba, N. J. & Davidson, S. Single-nucleus transcriptomic analysis of human dorsal root ganglion neurons. *Elife* **10**, e71752 (2021). 34825887 10.7554/eLife.71752 PMC8626086

[21] Hall, B. E. et al. Transcriptomic analysis of human sensory neurons in painful diabetic neuropathy reveals inflammation and neuronal loss. *Sci. Rep.* **12**, 4729 (2022). 35304484 10.1038/s41598-022-08100-8 PMC8933403

[22] Doty, M. et al. Integrative multiomic analyses of dorsal root ganglia in diabetic neuropathic pain using proteomics, phospho-proteomics, and metabolomics. *Sci. Rep.* **12**, 17012 (2022). 36220867 10.1038/s41598-022-21394-y PMC9553906

[23] Sharaf El Din, U. A. A., Salem, M. M. & Abdulazim, D. O. Uric acid in the pathogenesis of metabolic, renal, and cardiovascular diseases: A review. *J. Adv. Res.* **8**, 537–548 (2017). 28748119 10.1016/j.jare.2016.11.004 PMC5512153

[24] Kitase, Y. et al. Β-aminoisobutyric acid, l-BAIBA, is a muscle-derived osteocyte survival factor. *Cell Rep.* **22**, 1531–1544 (2018). 29425508 10.1016/j.celrep.2018.01.041 PMC5832359

[25] Zangger, P., Keystone, E. C. & Bogoch, E. R. Asymmetry of small joint involvement in rheumatoid arthritis: Prevalence and tendency towards symmetry over time. *Joint Bone Spine.* **72**, 241–247 (2005). 15850996 10.1016/j.jbspin.2004.08.013

[26] Wangzhou, A. et al. A ligand-receptor interactome platform for discovery of pain mechanisms and therapeutic targets. *Sci. Signal* **14**, eabe1648 (2021). 33727337 10.1126/scisignal.abe1648 PMC8097872

[27] Agarwal, V. et al. A clinical, electrophysiological, and pathological study of neuropathy in rheumatoid arthritis. *Clin. Rheumatol.* **27**, 841–844 (2008). 18084807 10.1007/s10067-007-0804-x

[28] Lanzillo, B. et al. Subclinical peripheral nerve involvement in patients with rheumatoid arthritis. *Arthritis Rheum.* **41**, 1196–1202 (1998). 9663475 10.1002/1529-0131(199807)41:7<1196::AID-ART8>3.0.CO;2-R

[29] Gou, Y., Zhang, T. & Xu, J. Transcription factors in craniofacial development: From receptor signaling to transcriptional and epigenetic regulation. *Curr. Top. Dev. Biol.* **115**, 377–410 (2015). 26589933 10.1016/bs.ctdb.2015.07.009

[30] Stelzer, G. et al. The GeneCards suite: From gene data mining to disease genome sequence analyses. *Curr. Protoc. Bioinformat.* **54**, 1–33 (2016). 10.1002/cpbi.5 27322403

[31] Xie, Z. et al. Gene set knowledge discovery with enrichr. *Curr. Protoc.* **1**, e90 (2021). 33780170 10.1002/cpz1.90 PMC8152575

[32] Chen, J., Bardes, E. E., Aronow, B. J. & Jegga, A. G. ToppGene Suite for gene list enrichment analysis and candidate gene prioritization. *Nucleic Acids Res.* **37**, W305–W311 (2009). 19465376 10.1093/nar/gkp427 PMC2703978

[33] Li, C. Y. et al. A human-specific de novo protein-coding gene associated with human brain functions. *PLoS Comput Biol.* **6**, e1000734 (2010). 20376170 10.1371/journal.pcbi.1000734 PMC2845654

[34] Bhuiyan, S.A. *et al.* Harmonized cross-species cell atlases of trigeminal and dorsal root ganglia. *bioRxiv.* 2023.07.04.547740 (2023). 10.1126/sciadv.adj9173 PMC11804847 38905344

[35] Sharif, B., Ase, A. R., Ribeiro-da-Silva, A. & Séguéla, P. Differential coding of itch and pain by a subpopulation of primary afferent neurons. *Neuron.* **106**, 940–951 (2020). 32298640 10.1016/j.neuron.2020.03.021

[36] Denk, F., Bennett, D. L. & McMahon, S. B. Nerve growth factor and pain mechanisms. *Annu. Rev. Neurosci.* **40**, 307–325 (2017). 28441116 10.1146/annurev-neuro-072116-031121

[37] Watson, J. J., Allen, S. J. & Dawbarn, D. Targeting nerve growth factor in pain: What is the therapeutic potential?. *BioDrugs* **22**, 349–359 (2008). 18998753 10.2165/0063030-200822060-00002

[38] Mecklenburg, J. et al. Transcriptional profiles of non-neuronal and immune cells in mouse trigeminal ganglia. *Front. Pain Res.* **4**, 1274811 (2023). 10.3389/fpain.2023.1274811 PMC10644122 38028432

[39] Matsushita, N. et al. Elevated levels of soluble CD163 in sera and fluids from rheumatoid arthritis patients and inhibition of the shedding of CD163 by TIMP-3. *Clin. Exp. Immunol.* **130**, 156–161 (2002). 12296867 10.1046/j.1365-2249.2002.01963.x PMC1906487

[40] Ohashi, Y. et al. Correlation between CD163 expression and resting pain in patients with hip osteoarthritis: Possible contribution of CD163+ monocytes/macrophages to pain pathogenesis. *J. Orthop. Res.* **40**, 1365–1374 (2022). 34370345 10.1002/jor.25157

[41] Chaudhuri, A. Regulation of macrophage polarization by RON receptor tyrosine kinase signaling. *Front. Immunol.* **5**, 546 (2014). 25400637 10.3389/fimmu.2014.00546 PMC4215628

[42] Yu, X. et al. Dorsal root ganglion macrophages contribute to both the initiation and persistence of neuropathic pain. *Nat. Commun.* **11**, 264 (2020). 31937758 10.1038/s41467-019-13839-2 PMC6959328

[43] Gunasekaran, M. et al. Immunization elicits antigen-specific antibody sequestration in dorsal root ganglia sensory neurons. *Front. Immunol.* **9**, 638 (2018). 29755449 10.3389/fimmu.2018.00638 PMC5932385

[44] Warton, K., Foster, N. C., Gold, W. A. & Stanley, K. K. A novel gene family induced by acute inflammation in endothelial cells. *Gene* **342**, 85–95 (2004). 15527968 10.1016/j.gene.2004.07.027

[45] Lecca, D., Raffaele, S., Abbracchio, M. P. & Fumagalli, M. Regulation and signaling of the GPR17 receptor in oligodendroglial cells. *Glia* **68**, 1957–1967 (2020). 32086854 10.1002/glia.23807

[46] Dziedzic, A., Miller, E., Saluk-Bijak, J. & Bijak, M. The GPR17 receptor-A promising goal for therapy and a potential marker of the neurodegenerative process in multiple sclerosis. *Int. J. Mol. Sci.* **21**, 1852 (2020). 32182666 10.3390/ijms21051852 PMC7084627

[47] Guo, C. et al. Copy number variation of multiple genes in SAPHO syndrome. *J. Rheumatol.* **47**, 1323–1329 (2020). 31615912 10.3899/jrheum.181393

[48] Chen, J., Caspi, R. R. & Chong, W. P. IL-20 receptor cytokines in autoimmune disease. *J. Leukoc. Biol.* **104**, 953–959 (2018). 30260500 10.1002/JLB.MR1117-471R PMC6298946

[49] Haberberger, R. V., Barry, C., Dominguez, N. & Matusica, D. Human dorsal root ganglia. *Front. Cell. Neurosci.* **13**, 271 (2019). 31293388 10.3389/fncel.2019.00271 PMC6598622

[50] Jimenez-Andrade, J. M. et al. Vascularization of the dorsal root ganglia and peripheral nerve of the mouse: Implications for chemical-induced peripheral sensory neuropathies. *Mol. Pain* **4**, 10 (2008). 18353190 10.1186/1744-8069-4-10 PMC2289805

[51] Renthal, W. et al. Transcriptional reprogramming of distinct peripheral sensory neuron subtypes after axonal injury. *Neuron* **108**, 128-144.e9 (2020). 32810432 10.1016/j.neuron.2020.07.026 PMC7590250

[52] Sjöstedt, E. et al. Integration of transcriptomics and antibody-based proteomics for exploration of proteins expressed in specialized tissues. *J. Proteome Res.* **17**, 4127–4137 (2018). 30272454 10.1021/acs.jproteome.8b00406

[53] Sumitomo, S. et al. Transcriptome analysis of peripheral blood from patients with rheumatoid arthritis: A systematic review. *Inflamm. Regen.* **38**, 21 (2018). 30410636 10.1186/s41232-018-0078-5 PMC6217768

[54] Phillips, S. A., Barr, V. A., Haft, D. H., Taylor, S. I. & Haft, C. R. Identification and characterization of SNX15, a novel sorting nexin involved in protein trafficking. *J. Biol. Chem.* **276**, 5074–5084 (2001). 11085978 10.1074/jbc.M004671200

[55] Zimmermann, J. S. M., Linxweiler, J., Radosa, J. C., Linxweiler, M. & Zimmermann, R. The endoplasmic reticulum membrane protein Sec62 as potential therapeutic target in *SEC62* overexpressing tumors. *Front. Physiol.* **13**, 1014271 (2022). 36262254 10.3389/fphys.2022.1014271 PMC9574383

[56] Heiberg, T. & Kvien, T. K. Preferences for improved health examined in 1,024 patients with rheumatoid arthritis: Pain has highest priority. *Arthritis Care Res.* **47**, 391–397 (2002). 10.1002/art.10515 12209485

[57] Lee, Y. C. et al. Pain persists in DAS28 rheumatoid arthritis remission but not in ACR/EULAR remission: A longitudinal observational study. *Arthritis Res. Ther.* **13**, R83 (2011). 21651807 10.1186/ar3353 PMC3218896

[58] Ashraf, S., Bouhana, K. S., Pheneger, J., Andrews, S. W. & Walsh, D. A. Selective inhibition of tropomyosin-receptor-kinase A (TrkA) reduces pain and joint damage in two rat models of inflammatory arthritis. *Arthritis Res. Ther.* **18**, 97 (2016). 27145816 10.1186/s13075-016-0996-z PMC4857260

[59] Katz, J. N. Tanezumab for painful osteoarthritis. *JAMA* **322**, 30–32 (2019). 31265081 10.1001/jama.2019.8250

[60] Wang, L. et al. Neuronal FcγRI mediates acute and chronic joint pain. *J. Clin. Invest.* **129**, 3754–3769 (2019). 31211699 10.1172/JCI128010 PMC6715360

[61] Bersellini Farinotti, A. et al. Cartilage-binding antibodies induce pain through immune complex-mediated activation of neurons. *J. Exp. Med.* **216**, 1904–1924 (2019). 31196979 10.1084/jem.20181657 PMC6683987

[62] Jurczak, A. et al. Insights into FcγR involvement in pain-like behavior induced by an RA-derived anti-modified protein autoantibody. *Brain Behav. Immun.* **113**, 212–227 (2023). 37437817 10.1016/j.bbi.2023.07.001

[63] Hutton, J. C. & Eisenbarth, G. S. A pancreatic beta-cell-specific homolog of glucose-6-phosphatase emerges as a major target of cell-mediated autoimmunity in diabetes. *Proc. Natl. Acad. Sci. U.S.A* **100**, 8626–8628 (2003). 12861077 10.1073/pnas.1633447100 PMC166361

[64] Du, R. H. et al. The pore-forming subunit Kir_6.1_ of the K-ATP channel negatively regulates the NLRP3 inflammasome to control insulin resistance by interacting with NLRP3. *Exp. Mol. Med.* **51**, 1–13 (2019). 31387986 10.1038/s12276-019-0291-6 PMC6802643

[65] Ray, P. R. et al. RNA profiling of human dorsal root ganglia reveals sex differences in mechanisms promoting neuropathic pain. *Brain* **146**, 749–766 (2023). 35867896 10.1093/brain/awac266 PMC10169414

[66] Olson, T. M. & Terzic, A. Human K(ATP) channelopathies: Diseases of metabolic homeostasis. *Pflugers Arch.* **460**, 295–306 (2010). 20033705 10.1007/s00424-009-0771-y PMC2883927

[67] Hosford, P. S. et al. A critical role for the ATP-sensitive potassium channel subunit K_IR_6.1 in the control of cerebral blood flow. *J. Cereb. Blood Flow Metab.* **39**, 2089–2095 (2019). 29862863 10.1177/0271678X18780602 PMC6775590

[68] Basu-Shrivastava, M., Kozoriz, A., Desagher, S. & Lassot, I. To Ubiquitinate or not to ubiquitinate: TRIM17 in cell life and death. *Cells* **10**, 1235 (2021). 34069831 10.3390/cells10051235 PMC8157266

[69] Lassot, I. et al. Trim17, a novel E3 ubiquitin-ligase, initiates neuronal apoptosis. *Cell Death Differ.* **17**, 1928–1941 (2010). 20559321 10.1038/cdd.2010.73 PMC3016602

[70] Pan, J. et al. Huntington associated protein 1 inhibition contributes to neuropathic pain by suppressing Cav1.2 activity and attenuating inflammation. *Pain* **164**, e286–e302 (2023). 36508175 10.1097/j.pain.0000000000002837

[71] Iadarola, M. J., Sapio, M. R. & Mannes, A. J. Be in it for the long haul: A commentary on human tissue recovery initiatives. *J. Pain* **23**, 1646–1650 (2022). 35504570 10.1016/j.jpain.2022.04.009 PMC9560952

[72] Love, M. I., Huber, W. & Anders, S. Moderated estimation of fold change and dispersion for RNA-seq data with DESeq2. *Genome Biol.* **15**, 550 (2014). 25516281 10.1186/s13059-014-0550-8 PMC4302049

[73] Wang, F. et al. RNAscope: A novel in situ RNA analysis platform for formalin-fixed, paraffin-embedded tissues. *J. Mol. Diagn.* **14**, 22–29 (2012). 22166544 10.1016/j.jmoldx.2011.08.002 PMC3338343

