# Supplementary material for: Transcriptome analysis of rheumatoid arthritis uncovers genes linked to inflammation-induced pain
Source: Sci Rep. 2024 Oct 29;14:25893. doi: 10.1038/s41598-024-77212-0 (PMC11522505; doi:10.1038/s41598-024-77212-0)
Supplement: Supplementary file 1 — Supplementary Information. [file 41598_2024_77212_MOESM1_ESM.pdf]

Supplemental Methods

Metabolomics:
Metabolite analysis was performed as described in Doty et al., 2022. Briefly, S1 DRG samples (30–50 mg) were analyzed by Human Metabolome Technologies-America, Boston, MA. Polar metabolites were detected using capillary electrophoresis time-of-flight mass spectrometry (carried out using Agilent CE-TOFMS system, Agilent Technologies Inc, Waldbronn, Germany), while non-polar metabolites were measured using liquid chromatography time-of-flight mass spectrometry LC-TOFMS (carried out with Agilent 1200 series RRLC system SL, Agilent Technologies Inc, Wadbronn, Germany). CE-TOFMS and LC-TOFMS data was processed with MasterHands (v2.17.1.11) for identification of peaks and quantification based on internal standards. Metabolites detected in fewer than 80% of samples were excluded from downstream analysis. Human Metabolome Database was used to identify metabolite class and directly related enzymes.

Supplemental Figure 1

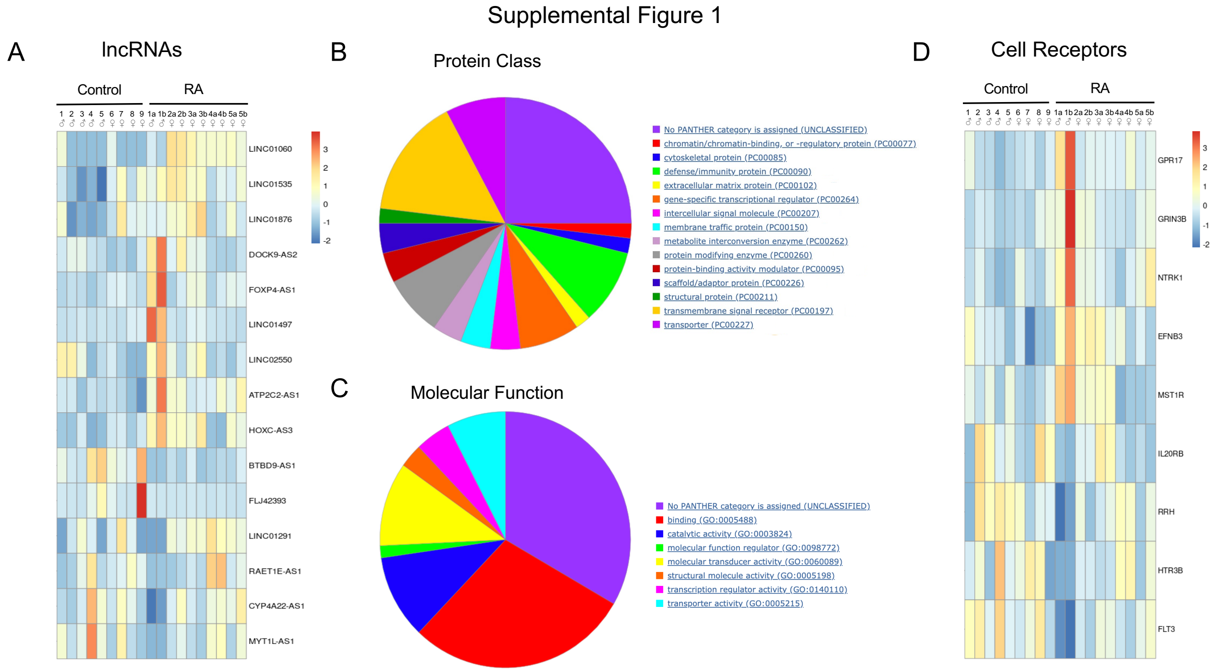

Characterization of DEGs: A) Long noncoding RNAs (lncRNAs) are a class of untranslated transcripts (more than 200 nucleotides) that are thought to regulate cellular gene expression. Heatmap shows about 15 discernable lncRNAs that were dysregulated between subjects with RA and controls. Notably, *DOCK9-AS2* is upregulated in RA patients and is implicated in atherosclerosis[4,2], while *LINC01876*, also upregulated, is a human-specific transcript that is linked to brain development[1]. Of the 128 significantly DEGs between the RA donors and the controls, 52 were protein-coding. PANTHER Analysis[3] was used to characterize these dysregulated protein-coding genes by B) protein class and C) molecular function. Of the characterized protein classes, transmembrane signaling receptors and intercellular signaling molecules together appear to be mostly affected with RA. D) Overall, 9 cell surface receptors (~17% of the protein-coding transcripts) were differentially expressed between RA and the controls.

1. Garza R, Atacho DAM, Adami A, Gerdes P, Vinod M, Hsieh P, Karlsson O, Horvath V, Johansson PA, Pandiloski N, Matas-Fuentes J, Quaegebeur A, Kouli A, Sharma Y, Jönsson ME, Monni E, Englund E, Eichler EE, Gale Hammell M, Barker RA, Kokaia Z, Douse CH, Jakobsson J. LINE-1 retrotransposons drive human neuronal transcriptome complexity and functional diversification. Sci Adv. 2023;9(44):eadh9543.

2. Shi J, Zhou B, Tian Z. DOCK9 antisense RNA2 interacts with LIN28B to stabilize Wnt5a and boosts proliferation and migration of oxidized low densitylipoprotein-induced vascular smooth muscle cells. Bioengineered. 2022;13(3):7564-7578.

3. Thomas PD, Ebert D, Muruganujan A, Mushayahama T, Albou LP, Mi H. PANTHER: Making genome-scale phylogenetics accessible to all. Protein Sci. 2022;31(1):8-22.

4. Wang CH, Shi HH, Chen LH, Li XL, Cao GL, Hu XF. Identification of Key lncRNAs Associated With Atherosclerosis Progression Based on Public Datasets. Front Genet. 2019;10:123

Supplemental Figure 2

Supplemental Figure 2

A

PBMC:

Gene	Base Mean	Log2FoldChange	padj
CAPNS2	16.21162315	2.146034209	0.003749995
F5	8845.794236	1.114750885	0.018293566
IGLV1-40	1635.09743	1.225317511	0.010234083
KCNJ1	132.430323	1.249187692	0.002577642
MMRN1	381.0272101	1.411650352	0.045220694
SEC62	26957.55599	-0.807514591	0.028393524
SNX15	192.9256473	1.478528787	0.033611113
TP63	53.3552703	1.328794454	0.038534494

DRG:

Gene	Base Mean	Log2FoldChange	padj
CAPNS2	19.49438331	-1.836991001	0.018443654
F5	1486.403356	-2.988420736	0.01389486
IGLV1-40	94.25275898	3.254732639	0.032378986
KCNJ1	75.76688136	-1.109852515	0.040406033
MMRN1	1010.013704	-2.038799918	0.009745558
SEC62	43484.94683	-0.445375145	0.021164539
SNX15	548.5219077	0.641759487	0.00430363
TP63	521.1796449	-1.247148452	0.049223562

B C

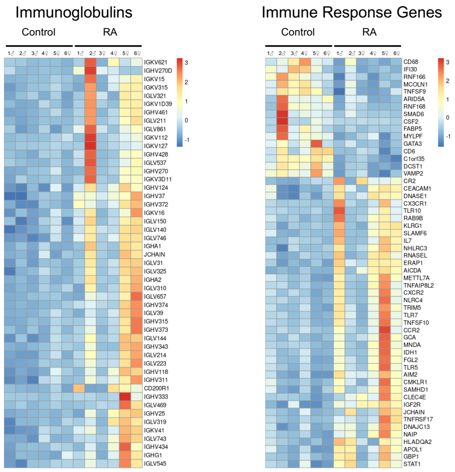

RNA-seq analysis of peripheral blood mononuclear cells (PBMCs) from patients with RA: A) 8 DEGS were common between the bulk sequencing performed on the DRGs and the PBMCs, of which only 3 were dysregulated in the same direction (*IGLV1-40, SNX15, and SEC62*). B) Nonetheless, both datasets show the overall upregulation in immunoglobulin genes. The heatmap shows genes primarily linked with immunoglobulin complexes (GO:0019814). C) Heatmap with predominately upregulated immune response genes (GO:0006955) in the PBMCs of patients with RA.

Supplemental Figure 3

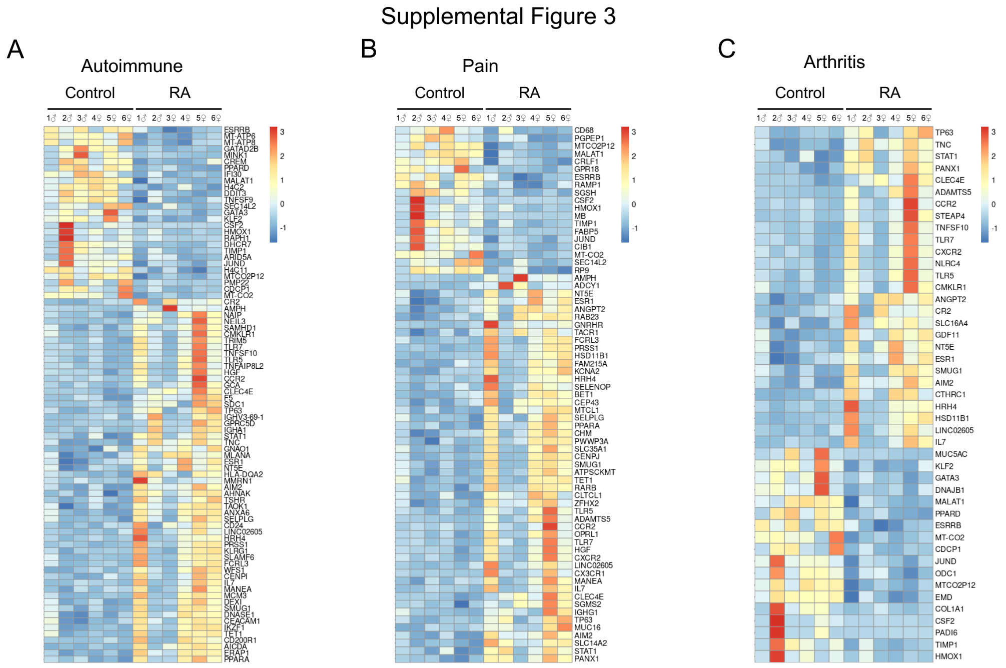

Characterization of PBMC RNA-seq data through DisGeNET: Heatmaps of genes linked with A) autoimmune diseases (C0004364), B) pain (C0030193), and C) arthritis (C0003864). Although upregulated inflammatory mediator signaling is a shared feature to these diseases, only about 12.7% of the selected DisGeNET genes (17 out of 134) were common to all 3 conditions.

Supplemental Figure 4

Supplemental Figure 4

A

Gene	Rheumatoid Arthritis			Diabetic Painful Neuropathy		
	baseMean	*log2FoldChange*	*padj*	*baseMean*	*log2FoldChange*	*padj*
HAP1	527.481	1.033	0.049	418.252	1.215	0.049
KCNJ8	420.886	0.916	0.015	286.455	1.141	0.018
TRIM17	194.616	0.841	0.017	111.050	1.012	0.008
G6PC2	470.507	-1.595	0.016	336.564	-1.437	0.012

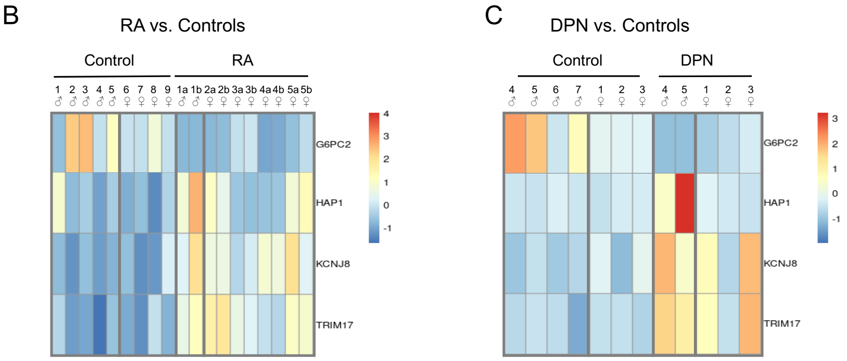

Overlap genes in both diabetic painful neuropathy (DPN) and RA: 10 gene are dysregulated in both DPN and RA, of which 5 are immunoglobulin related genes (*IGHG2*, *IGHG3*, *IGLC2*, and *IGKV1-33*, with *IGHGP* being a pseudogene). *CRYGN* is upregulated in RA while downregulated in DPN, but *G6PC2*, *HAP1*, *KCNJ8*, and *TRIM17* show fold changes in the same direction in both chronic pain disorders. A) Table with base mean, log2 fold change, and p adjusted value. B) Heatmap of RA donors and corresponding controls and C) heatmap of DPN donors with data from Hall et. al. 2022. As seen in the heatmaps, there may be gender differences related to dysregulated gene expression within the chronic pain donors.

Supplemental Tables

1 Donors
2 Demographics
3 Histology
4 Final Metabolites
5 Putative Metabolites
6 Quantitative Estimation
7 Significant Genes
8 Counts
9 Normalized Data
10 Raw Counts Data
11 RNA Quality
12 PBMC Demographics
13 PBMC Significant Genes
14 PBMC Normalized Data

Donor	Sex	Age	Race	Height (cm)	Weight (kg)	BMI	Diabetes	RNAseq	Metab	H&E
Control 1	M	29	Caucasian	185	56.5	16.5	Type I 5yr	✓		
Control 2	M	45	Caucasian	166	70.2	25.5		✓	✓	✓
Control 3	M	47	Caucasian	183	95.5	28.5		✓	✓	
Control 4	M	54	Caucasian	183	80.2	23.9		✓	✓	✓
Control 5	M	55	Caucasian	190.5	77.1	21.1		✓	✓	✓
Control 6	F	30	Hispanic	157	49	19.9		✓	✓	✓
Control 7	F	39	African American	159	63.1	25		✓	✓	✓
Control 8	F	40	Caucasian	161	65.8	25.4	Type II 4 yr	✓		
Control 9	F	49	Caucasian	167.6	103.5	36.7		✓	✓	
Control 10	F	51	African American	180	112	34.4				✓
Control 11	F	52	Caucasian	166	98.1	35.6				✓
Control 12	M	46	Hispanic	163	61.8	23.3			✓	
RA1	M	48	Hispanic	168	83.1	29.4		✓	✓	✓
RA2	F	53	Caucasian	160	105	41		✓	✓	✓
RA3	F	57	Caucasian	167	135.6	48.6		✓	✓	✓
RA4	F	61	Caucasian	165.1	88	32.3		✓	✓	✓
RA5	F	66	Caucasian	152	91	39.4	Type II 5yr	✓	✓	✓

ID	Storage	Condition	Sex	Age	Race	Height (cm)	Weight (kg)	BMI	Blood Type
Con1	RNA	Healthy (D)	M	29	Caucasian	185	56.5	16.5	O
Con2	RNA	Healthy	M	45	Caucasian	166	70.2	25.5	A
Con3	SF	Healthy	M	47	Caucasian	183	95.5	28.5	O
Con4	RNA	Healthy	M	54	Caucasian	183	80.2	23.9	B
Con5	SF	Healthy	M	55	Caucasian	190.5	77.1	21.1	
Con6	RNA	Healthy	F	30	Hispanic	157	49	19.9	O+
Con7	RNA	Healthy	F	39	African American	159	63.1	25	AB+
Con8	RNA	Healthy (D)	F	40	Caucasian	161	65.8	25.4	O
Con9	SF	Healthy	F	49	Caucasian	167.6	103.5	36.7	O
RA1	SF	RA	M	48	Hispanic	168	83.1	29.4	O
RA2	SF	RA	F	53	Caucasian	160	105	41	
RA3	RNA	RA	F	57	Caucasian	167	135.6	48.6	O+
RA4	SF	RA	F	61	Caucasian	165.1	88	32.3	
RA5	RNA	RA (D)	F	66	Caucasian	152	91	39.4	

COD	Respiratory Disease
Anoxia/Cardiovascular	Yes: Mild intermittent asthma, unknown timeframe
Anoxia	No
Head Trauma	No
Head Trauma/Blunt Injury	No
Head Trauma	No
CVA/Stroke	Yes: Asthma: used inhaler daily
CVA/Stroke	No
CVA/Stroke	Yes: Possibly asthma occational attack. Carried and inhaler, unknown specifics
MVA/Head Trauma	No
CVA/Stroke	No
Anoxia/Cardiovascular	Yes: COPD, Asthma, Asthmatic Bronchitis
Head Trauma	Yes: Exercise induced asthma (rarely used inhaler, no use in the last 10 years)
CVA/ICH/Stroke	No
Anoxia	No

Cardiac Disease
No
No
No
No
Yes: Coronary Artery Disease history, MI in 2013, heart stints placed
No
No
No
No
No
No
No
No
Yes: CAD, triple bypass surgery 02/2018

Hypertension
No
No
Yes: 5 years
No
No
Yes: Diagnosed several months ago, related to renal failure
No
Yes: HTN since she was 16 years old
No
Yes: High blood pressure for 20-25 years
Yes: Unknown Details
Yes: Managed without medications for past 2 years
Yes: x 5 years
Yes: Five years, compliant with treatment

Neurological Disease

No
Yes: Lennox-Gastaut Syndrome
No
No
No
No
No
No
No
No
Yes: PTSD, Anxiety, migraines, Bipolar disorder, major depressive disorder, suicidal ideation. Decline in mental processing 2017
No
No
Yes: Depression, anxiety

Cancer	Diabetes	Liver disease	GI Disease
No	Yes: 5 years, type I, non-compliant with insulin treatment, multiple DKA visits	No	No
No	No	No	No
No	No	No	No
No	No	No	No
No	No	No	No
No	No	No	No
No	No	No	No
No	Yes: 4 years ago	No	No
No	No	No	No
No	No	No	No
No	No	No	No
No	No	No	No
No	No	No	No
No	Yes: Type II x 5 years, treated with daily pill	No	No

Kidney Disease
No
No
No
No
No
Yes: Renal failure due to being born with small kidneys: kidney transplant done 10 years ago. Kidney failure post-transplant 1 year ago.
No
No
No
No
No
Yes: Kidney Stones (single episode in 2013)
No
No

Urinary Tract Disease
No
No
No
No
No
No
No
No
No
No
No
No
No
No

Tobacco
No
No
Yes: 0.5-1 pack per day since 1990's
Yes: Smoked cigarettes 1/2 ppd x 10 years
Yes: Cigarettes 1 pack per day x 30 years
No
Yes: Cigarettes (frequency of use, amount, and how often is unknown)
Yes: Smoked cigarettes 1/2 ppd until 4 yrs ago (started 13 years old). For the past 4 years a few cigarettes per day
Yes: Cigarettes 1/2 pack/day, for more than 18 years, stopped last year
No
Yes: Started smoking at age 12, 1/2-1 ppd x 41 years
No
No
Yes: Cigarettes - 1 ppd x 13 years until 11/2017, 1/4 pack since 2/2018

Alcohol
No
No
Yes: Unknown amount of beer and vodka daily, at least a 5th of vodka a day since 90's
Yes: Drank 6-12 pack of beer every other day x 15 years
Yes: 2-3 drinks daily x30 years (vodka, beer)
Yes: Wine/beer: 1-2 drinks every few months x5 years, stopped drinking 1 year ago
Yes: Beer and liquor (frequency of use, amount, and how often is unknown)
Yes: Drank aprox. 1 beer / month since 20's
Yes: 1/2 glass rum/beer, once every 3 months for more than 8 years
Yes: Beer 3-4 cans, daily for 10-15 years
Yes: Unknown details, alcoholic, drank daily for 10+ years
Yes: Wine (1/2 a glass once a month for 2 years)
Yes: Unknown details, has been sober for over 30 years
No

Drug Use
No
No
Yes: Smoked crack cocaine since 2011, unknown last use and no needles, MSM within the last 12 months
No
No
No
Yes: Methamphetamine (frequency of use, amount, and how often is unknown)
Yes: Used amphetamines intranasally for >10 years and last used was 6/25/16. Ecstasy
Yes: Smoked Methamphetamines, last used 10 years ago
No
Yes: Polysubstance abuse. Drug OD, Lorazepam, Oxycontin in 2016
No
No
No

Medications

Yes: Home: Lantus, Humalog, Insulin
Yes: Home: Benztropine (1mg), Clobazam (20mg), Diazepam (20mg), Diazepam (gel), Benadryl (25mg), Depakote sprinkle (125mg), Depakote tab. (500mg), Risperidone (2mg), Emollient (lotion)
Yes: Home: Unknown medication for hypertension, Antabuse
Yes: Home: Calcium Supplements
Yes: Home: Plavix
Yes: Home: Lisinopril, Sensipar, Renvela, Procardia, Protonix, Carvedilol, Albuterol, Vitamin D Supplement.
No
Yes: Home: Amlodipine, Labetalol, Catapres
Yes: Home: Simvastatin and Advil
Yes: Acetaminophen, Ibuprofen, Multivitamin
Yes: Norco, Sumatriptan, Seroquel, Promethazine, Meloxicam, Lyrica, Levothyroxine, Baclofen, Lipitor, Alendronate, Albuterol
Yes: Steroids, gabapentin
Yes: Humira, unknown blood pressure medication
Yes: Metformin, Ramipril, Sertraline, Diflucan, Alprazolam, Brilinta, Morphine, Torsemide, Rosuvastatin, Vitamin D3

Comments
None.
Allergies to Sulfa drugs, Vagus nerver stimulator 12yrs ago, Strabismus corrected at 4yrs old
None.
None.
None.
Surgeries done: kidney transplant, thoracentesis, port placement. Treated with peritoneal dialysis (age 16-20) and hemodialysis (for 1 year prior to death)
None.

Rheumatoid Arthritis
Rhumetoid Arthritis, Fibromyalgia, Osteoporosis, Chronic Pain, Cervicalgia, Thoracic sprain, Bowel resection and hysterectomy d/t Endometriosis, Restless leg syndrome, poor circulation in lower legs
Joint pain due to rhumatoid arthritis. Prescribed steroids for joint pain. Severe headaches every 2 weeks. Prescribed Gabapentin for migraines
Rhumetoid Arthritis
Rheumetoid Arthritis, Cellulitis, swelling in feet and ankles causing difficulty walking.

Donor	Sex	Age	Ganglion cells	Inflammation	Fibrosis	CD3	CD163*
**Con2**	M	45	Variably sized	5 lymphocytic foci	Minimal	Few, scattered	<50
**Con4**	M	54	Less variably sized, scattered single cells	Not seen	Minimal	Few, clustered	<50
**Con5**	M	55	Variably sized, clearing of nuclei	7 lymphocytic foci	Minimal	Few, scattered	<50
**Con6**	F	30	Variably sized, cytoplasmic vacuolization	Not seen	Minimal	Rare	>50
**Con7**	F	39	Variably sized, scattered single cells	Not seen	Minimal	Rare	<50
**Con 10**	F	51	Variably sized, scattered single cells	Diffuse interstitial infiltrate	Minimal	Few, scattered	<50
**Con 11**	F	52	Variably sized	Diffuse interstitial infiltrate	Minimal	Many, clustered	>50
**RA1**	M	48	Variably sized, clearing of nuclei	1 lymphocytic foci	Minimal	Few, scattered	<50
**RA2**	F	53	Variably sized	Not seen	Minimal	Few, scattered	<50
**RA3**	F	57	Variably sized, scattered single cells	2 lymphocytic foci	Moderate	Few, clustered	>50
**RA4**	F	61	Variably sized	Not seen	Minimal	Many, clustered	>50
**RA5**	F	66	Variably sized	Not seen	Moderate	Few, clustered	>50

** CD163+ cells/high-power field (HPF; 40X)*

ID	Compound name	PubChem CID	HMDB ID	*m/z*	MT/RT	Relative Area												control_avg	RA_avg	RA_log2fc	p	n_up_RA	n_down_RA
						Con2	Con9	Con5	Con3	Con12	Con7	Con4	Con6	RA3	RA4	RA2	RA1						
A_0019	Uric acid	1175	HMDB0000289	167.021	9.03	5.0E-04	1.3E-03	6.7E-04	8.0E-04	5.2E-04	3.9E-04	4.0E-04	1.7E-03	2.0E-03	2.0E-03	4.7E-03	1.8E-03	7.8E-04	2.7E-03	1.765644	0.07	4	0
C_0058	3-Aminoisobutyric acid	64956	HMDB0003911	104.071	7.28	1.4E-03	2.5E-03	1.6E-03	8.9E-04	2.0E-03	1.6E-03	1.6E-03	1.3E-03	4.5E-03	1.9E-03	5.4E-03	4.1E-03	1.6E-03	4.0E-03	1.30161	0.047	4	0

** samples from donors who had diabetes as a comorbidity were excluded from further analysis*

Putative Metabolites

	HMT DB †						Relative Area														
ID	Compound name	Pathway Label	PubChem CID	HMDB ID	*m/z*	MT/RT	Control										RA				
							Con2	Con8 (D)	Con1 (D)	Con9	Con5	Con3	Con12	Con7	Con4	Con6	RA5 (D)	RA3	RA4	RA2	RA1
C_0097	1-Methylhistamine	1-Methylhistamine	3614	HMDB0000898	126.104	4.60	N.D.	N.D.	N.D.	1.3E-04	N.D.	9.3E-05	N.D.	N.D.	N.D.	N.D.	N.D.	N.D.	N.D.	N.D.	N.D.
C_0055	1-Methylhistidine<br>3-Methylhistidine	1-Methylhistidine<br>3-Methylhistidine	92105<br>64969	HMDB000000<br>1<br>HMDB000047	170.094	6.95	N.D.	N.D.	N.D.	7.3E-04	N.D.	N.D.	N.D.	N.D.	N.D.	8.7E-04	N.D.	N.D.	6.5E-04	1.7E-03	1.3E-03
C_0098	1-Methylnicotinamide	1-Methylnicotinamide	457	HMDB0000699	137.071	6.83	4.5E-04	N.D.	N.D.	7.2E-04	3.0E-04	N.D.	2.5E-04	4.5E-04	2.3E-04	2.0E-04	N.D.	3.3E-04	3.4E-04	2.8E-04	2.3E-04
P_0087	1-Oleoyl-glycero-3-phosphocholine	1-Oleoyl-glycero-3-phosphocholir	5280634	HMDB0010385	522.361	13.33	N.D.	N.D.	N.D.	N.D.	N.D.	N.D.	N.D.	N.D.	N.D.	N.D.	5.9E-05	N.D.	N.D.	N.D.	N.D.
P_0084	1-Palmitoyl-glycero-3-phosphocholine-1	1-Palmitoyl-glycero-3-phosphoch	460602	HMDB0010382	496.338	13.14	N.D.	N.D.	4.7E-05	N.D.	N.D.	N.D.	N.D.	N.D.	N.D.	N.D.	4.9E-05	N.D.	N.D.	N.D.	N.D.
P_0085	1-Palmitoyl-glycero-3-phosphocholine-2	1-Palmitoyl-glycero-3-phosphoch	460602	HMDB0010382	496.343	13.31	4.2E-05	N.D.	N.D.	N.D.	N.D.	N.D.	N.D.	N.D.	N.D.	N.D.	N.D.	N.D.	N.D.	N.D.	N.D.
P_0078	1-Palmitoyl-glycero-3-phosphoethanolamine<br>LPE(16:0)	1-Palmitoyl-glycero-3-<br>phosphoethanolamine<br>LPE(16:0)	9547069<br>0	HMDB001150<br>3	454.292	13.27	N.D.	N.D.	2.7E-05	N.D.	N.D.	N.D.	N.D.	N.D.	N.D.	7.9E-05	N.D.	N.D.	N.D.	N.D.	N.D.
N_0036	15(S)-HETE	15(S)-HETE	5280724	HMDB0003876	319.226	12.67	N.D.	N.D.	1.6E-05	1.9E-05	N.D.	N.D.	1.0E-05	N.D.	N.D.	N.D.	1.2E-05	N.D.	N.D.	N.D.	N.D.
P_0022	17α-Hydroxyprogesterone<br>Deoxycorticosterone	17a-Hydroxyprogesterone<br>Deoxycorticosterone	6238<br>6166	HMDB000037<br>4<br>HMDB000001	331.228	10.17	3.3E-05	3.7E-05	N.D.	7.2E-05	N.D.	8.5E-05	2.2E-05	N.D.	N.D.	4.0E-05	5.1E-05	7.2E-05	1.4E-04	N.D.	1.0E-04
C_0056	1*H*-Imidazole-4-propionic acid	1H-Imidazole-4-propionic acid	10105257		141.067	7.51	N.D.	N.D.	N.D.	N.D.	N.D.	N.D.	N.D.	N.D.	N.D.	N.D.	N.D.	N.D.	N.D.	3.8E-04	N.D.
A_0047	2,3-Diphosphoglyceric acid	Diphosphoglycerate	186004	HMDB0001294	264.952	19.09	N.D.	N.D.	N.D.	N.D.	N.D.	N.D.	N.D.	N.D.	N.D.	N.D.	N.D.	N.D.	N.D.	N.D.	N.D.
C_0057	2-Aminoisobutyric acid<br>2-Aminobutyric acid	2-Aminoisobutyric acid<br>2-Aminobutyric acid	6119<br>6657	HMDB000190<br>6<br>HMDB000045	104.071	8.91	4.6E-03	2.9E-03	N.D.	9.6E-03	4.9E-03	1.7E-03	6.5E-04	2.5E-03	2.0E-03	7.0E-04	N.D.	4.4E-03	3.2E-03	4.9E-03	7.5E-03
P_0046	2-Arachidonoylglycerol-1	2-Arachidonoylglycerol	5282280	HMDB0004666	379.274	13.47	9.0E-05	1.2E-04	8.2E-05	1.1E-04	N.D.	2.2E-04	N.D.	8.9E-05	1.1E-04	1.4E-04	1.3E-04	1.2E-04	7.7E-05	N.D.	4.6E-05
P_0047	2-Arachidonoylglycerol-2	2-Arachidonoylglycerol	5282280	HMDB0004666	379.285	13.73	2.3E-03	3.0E-03	2.8E-02	5.4E-03	1.5E-03	2.9E-03	4.4E-03	2.4E-03	1.9E-03	1.5E-02	2.4E-02	1.1E-03	1.0E-03	2.3E-04	7.3E-04
A_0006	2-Hydroxybutyric acid	2-HBA	440864	HMDB0000008	103.041	9.90	N.D.	N.D.	N.D.	1.5E-03	6.7E-04	N.D.	N.D.	6.7E-04	N.D.	N.D.	N.D.	7.9E-04	N.D.	N.D.	N.D.
N_0007	2-Hydroxytetradecanoic acid-1<br>3-Hydroxytetradecanoic acid-4	2-Hydroxytetradecanoic acid<br>3-Hydroxytetradecanoic acid	656737<br>16064	HMDB000226<br>1	243.197	13.08	1.2E-05	1.6E-05	N.D.	8.5E-06	1.4E-05	1.8E-05	N.D.	N.D.	1.2E-05	N.D.	N.D.	2.3E-05	1.2E-05	1.4E-05	N.D.
N_0008	2-Hydroxytetradecanoic acid-2	2-Hydroxytetradecanoic acid	656737	HMDB0002261	243.198	13.22	N.D.	N.D.	N.D.	N.D.	N.D.	N.D.	N.D.	N.D.	N.D.	N.D.	8.9E-06	N.D.	N.D.	N.D.	N.D.
P_0033	20α-Hydroxycholesterol	20a-Hydroxycholesterol	121935	HMDB0006283	367.334	14.28	N.D.	5.4E-05	1.1E-04	1.0E-04	N.D.	2.2E-04	N.D.	6.1E-05	5.1E-05	7.2E-05	N.D.	N.D.	9.0E-05	6.4E-05	1.2E-04
P_0050	22-Hydroxycholesterol	22-Hydroxycholesterol	167685	HMDB0004035	385.346	13.71	2.0E-04	2.0E-04	2.2E-04	1.9E-04	1.9E-04	2.1E-04	1.9E-04	1.8E-04	1.3E-04	1.4E-04	N.D.	1.8E-04	N.D.	N.D.	N.D.
C_0058	3-Aminoisobutyric acid	3-Aminoisobutyric acid	64956	HMDB0003911	104.071	7.28	1.4E-03	2.3E-03	N.D.	2.5E-03	1.6E-03	8.9E-04	2.0E-03	1.6E-03	1.6E-03	1.3E-03	1.7E-03	4.5E-03	1.9E-03	5.4E-03	4.1E-03
C_0059	3-Hydroxy-2-methyl-4-pyrone	3-Hydroxy-2-methyl-4-pyrone	8369	HMDB0030776	127.039	20.16	1.7E-02	1.6E-02	5.5E-03	1.9E-02	1.8E-02	1.9E-02	8.1E-03	1.7E-02	1.5E-02	8.0E-03	6.3E-03	1.5E-02	2.5E-02	1.8E-02	2.3E-02
A_0005	3-Hydroxybutyric acid	3-HBA	441	HMDB0000011	103.040	9.65	N.D.	N.D.	N.D.	N.D.	5.4E-04	N.D.	N.D.	8.1E-04	N.D.	N.D.	N.D.	N.D.	N.D.	9.4E-04	1.2E-03
N_0006	3-Hydroxytetradecanoic acid-1	3-Hydroxytetradecanoic acid	16064	HMDB0061656	243.196	12.61	N.D.	1.1E-05	N.D.	N.D.	N.D.	N.D.	6.3E-06	N.D.	N.D.	1.5E-05	2.1E-05	8.7E-06	1.3E-05	N.D.	N.D.
N_0009	3-Hydroxytetradecanoic acid-2	3-Hydroxytetradecanoic acid	16064	HMDB0061656	243.198	12.72	N.D.	N.D.	9.9E-06	9.4E-06	1.2E-05	3.0E-05	N.D.	N.D.	N.D.	2.0E-05	N.D.	1.3E-05	N.D.	N.D.	N.D.
N_0005	3-Hydroxytetradecanoic acid-3	3-Hydroxytetradecanoic acid	16064	HMDB0061656	243.196	12.85	1.3E-05	1.8E-05	2.6E-05	2.0E-05	1.5E-05	3.6E-05	1.4E-05	2.3E-05	2.8E-05	3.1E-05	2.3E-05	1.9E-05	2.0E-05	1.1E-05	2.1E-05
A_0036	3-Indoxylsulfuric acid	3-Indoxylsulfuric acid	10258	HMDB0000682	212.002	9.70	N.D.	N.D.	N.D.	N.D.	N.D.	N.D.	N.D.	N.D.	N.D.	2.0E-03	N.D.	N.D.	N.D.	1.9E-03	N.D.
A_0029	3-Phosphoglyceric acid	3-PG	439183	HMDB0000807	184.987	20.36	N.D.	N.D.	N.D.	1.0E-03	N.D.	1.5E-03	N.D.	N.D.	N.D.	N.D.	N.D.	7.0E-04	N.D.	N.D.	9.0E-04
P_0010	4-Androstene-3,17-dione	4-Androstene-3,17-dione	6128	HMDB0000053	287.204	10.04	1.2E-04	1.7E-04	2.2E-04	1.7E-04	9.7E-05	1.7E-04	1.1E-04	8.6E-05	1.3E-04	1.2E-04	1.7E-04	1.3E-04	1.2E-04	1.2E-04	2.2E-04
C_0060	4-Oxopyrrolidine-2-carboxylic acid	4-Oxopyrrolidine-2-carboxylic aci	107541		130.051	10.10	2.9E-04	4.6E-04	N.D.	7.2E-04	4.2E-04	3.7E-04	N.D.	3.5E-04	2.8E-04	N.D.	N.D.	5.6E-04	3.5E-04	5.1E-04	5.4E-04
A_0010	5-Oxoproline	Oxoproline	7405	HMDB0000267	128.037	9.63	N.D.	N.D.	N.D.	1.2E-03	7.0E-04	N.D.	3.7E-04	N.D.	N.D.	N.D.	N.D.	6.6E-04	4.9E-04	1.5E-03	5.9E-04
P_0053	5α-Cholestan-3-one	5a-Cholestan-3-one	92128	HMDB0000871	387.368	16.27	1.0E-03	1.3E-03	1.5E-03	1.0E-03	1.1E-03	1.4E-03	8.4E-04	1.2E-03	1.0E-03	1.2E-03	1.4E-03	9.8E-04	1.1E-03	1.3E-03	1.3E-03
C_0061	6-Aminohexanoic acid	6-Aminohexanoic acid	564	HMDB0001901	132.102	7.76	1.0E-03	1.1E-03	N.D.	N.D.	N.D.	N.D.	9.4E-04	7.6E-04	N.D.	1.3E-03	5.3E-04	5.6E-04	N.D.	N.D.	N.D.
P_0036	7-Dehydrocholesterol-1<br>Desmosterol-1	7-Dehydrocholesterol<br>Desmosterol	439423<br>439577	HMDB000003	367.336	15.86	7.1E-03	7.8E-03	1.0E-02	6.3E-03	3.6E-03	4.5E-03	6.7E-03	4.3E-03	6.0E-03	1.1E-02	5.2E-03	2.5E-03	3.5E-03	2.9E-03	4.4E-03
P_0035	7-Dehydrocholesterol-2<br>Desmosterol-2	7-Dehydrocholesterol<br>Desmosterol	439423<br>439577	HMDB000003<br>2<br>HMDB000271	367.336	16.02	4.7E-04	4.7E-04	7.8E-04	2.2E-04	1.6E-04	3.0E-04	4.8E-04	5.1E-04	4.3E-04	8.9E-04	1.1E-03	3.3E-04	2.4E-04	1.7E-03	2.4E-04
P_0037	7-Dehydrocholesterol-3<br>Desmosterol-3	7-Dehydrocholesterol<br>Desmosterol	439423<br>439577	HMDB000003<br>2<br>HMDB000271	367.337	16.08	N.D.	2.3E-04	N.D.	2.5E-04	4.7E-04	4.4E-04	8.1E-05	1.6E-04	2.4E-04	5.6E-04	N.D.	1.9E-04	3.3E-04	3.7E-04	3.4E-04
P_0034	7-Dehydrocholesterol-4	7-Dehydrocholesterol	439423	HMDB0000032	367.336	16.26	6.9E-04	8.7E-04	9.5E-04	8.3E-04	9.0E-04	1.0E-03	6.5E-04	9.6E-04	8.0E-04	1.2E-03	9.4E-04	6.7E-04	7.5E-04	8.4E-04	8.5E-04
P_0015	AC(10:0)	AC(10:0)			316.248	9.12	6.5E-04	7.7E-04	8.8E-05	2.8E-03	1.6E-03	2.0E-03	3.2E-04	1.2E-03	5.0E-04	2.6E-04	6.7E-04	1.5E-03	4.2E-03	1.6E-03	1.8E-03
P_0028	AC(12:0)	AC(12:0)			344.280	10.13	4.4E-03	3.0E-03	6.8E-04	7.1E-03	7.1E-03	6.0E-03	1.4E-03	2.8E-03	1.8E-03	9.4E-04	3.1E-03	4.1E-03	6.1E-03	4.8E-03	5.0E-03
P_0025	AC(12:1)-1	AC(12:1)			342.261	9.62	4.0E-04	4.2E-04	1.3E-04	1.2E-03	8.7E-04	6.0E-04	2.1E-04	5.2E-04	3.8E-04	2.2E-04	4.3E-04	8.0E-04	1.2E-03	1.2E-03	8.8E-04
P_0027	AC(12:1)-2	AC(12:1)			342.262	9.77	5.6E-05	9.6E-05	4.1E-05	3.3E-04	1.0E-04	1.4E-04	N.D.	1.3E-04	7.7E-05	3.8E-05	8.2E-05	1.2E-04	3.6E-04	9.1E-05	1.6E-04
P_0026	AC(12:1)-3	AC(12:1)			342.261	9.89	7.1E-05	7.9E-05	4.2E-05	2.3E-04	1.1E-04	9.1E-05	5.6E-05	6.2E-05	5.5E-05	N.D.	9.4E-05	1.2E-04	2.9E-04	1.3E-04	2.0E-04
P_0030	AC(13:1)	AC(13:1)			356.281	10.06	9.6E-05	8.8E-05	5.0E-05	3.0E-04	1.2E-04	1.4E-04	5.6E-05	8.9E-05	5.5E-05	N.D.	1.0E-04	1.7E-04	4.9E-04	1.3E-04	1.3E-04
P_0044	AC(14:0)	AC(14:0)			372.313	10.96	1.4E-02	1.4E-02	3.4E-03	2.6E-02	2.9E-02	2.3E-02	7.9E-03	1.2E-02	9.1E-03	5.1E-03	1.1E-02	1.7E-02	2.1E-02	1.8E-02	1.8E-02
P_0039	AC(14:1)-1	AC(14:1)			370.293	10.43	8.7E-04	8.9E-04	1.7E-04	1.7E-03	2.0E-03	1.0E-03	3.7E-04	9.6E-04	6.9E-04	4.2E-04	7.3E-04	1.5E-03	1.4E-03	2.3E-03	1.1E-03
P_0040	AC(14:1)-2	AC(14:1)			370.293	10.52	2.9E-03	2.7E-03	4.6E-04	5.0E-03	3.7E-03	3.7E-03	1.1E-03	2.5E-03	1.3E-03	9.9E-04	1.3E-03	3.3E-03	4.7E-03	2.3E-03	4.0E-03
P_0041	AC(14:1)-3	AC(14:1)			370.293	10.76	1.3E-04	2.9E-04	6.1E-05	6.3E-04	3.1E-04	3.0E-04	1.2E-04	2.1E-04	1.1E-04	9.6E-05	1.1E-04	3.6E-04	7.8E-04	2.3E-04	7.7E-04
P_0038	AC(14:2)	AC(14:2)			368.280	10.03	6.6E-04	7.2E-04	1.3E-04	1.2E-03	5.4E-04	8.4E-04	2.8E-04	3.7E-04	2.3E-04	2.3E-04	4.2E-04	9.5E-04	1.0E-03	5.7E-04	9.4E-04
P_0032	AC(14:3)	AC(14:3)			366.261	9.54	N.D.	3.8E-05	N.D.	N.D.	2.9E-05	5.3E-05	N.D.	N.D.	N.D.	N.D.	N.D.	8.2E-05	5.4E-05	N.D.	N.D.
P_0052	AC(15:0)-1	AC(15:0)			386.325	11.22	2.6E-04	2.4E-04	1.3E-04	4.9E-04	3.1E-04	4.1E-04	1.4E-04	1.4E-04	1.8E-04	9.7E-05	3.0E-04	3.9E-04	3.7E-04	2.2E-04	1.7E-04
P_0051	AC(15:0)-2	AC(15:0)			386.325	11.36	7.7E-04	9.6E-04	2.9E-04	1.2E-03	1.3E-03	1.3E-03	5.3E-04	5.1E-04	4.4E-04	3.4E-04	6.7E-04	1.1E-03	1.0E-03	9.7E-04	8.4E-04
P_0060	AC(16:1)	AC(16:1)			398.328	11.19	1.3E-02	1.5E-02	4.5E-03	2.0E-02	2.6E-02	1.8E-02	9.5E-03	1.6E-02	1.0E-02	6.5E-03	7.9E-03	1.7E-02	1.6E-02	2.3E-02	1.6E-02
P_0056	AC(16:2)-1	AC(16:2)			396.313	10.95	3.7E-04	4.4E-04	1.4E-04	5.7E-04	5.3E-04	2.1E-04	2.7E-04	1.6E-04	2.7E-04	2.3E-04	1.0E-04	2.5E-04	4.0E-04	3.0E-04	3.7E-04
P_0057	AC(16:2)-2	AC(16:2)			396.313	10.75	7.5E-04	6.8E-04	1.6E-04	7.0E-04	5.8E-04	7.6E-04	4.9E-04	5.4E-04	2.6E-04	3.5E-04	5.2E-04	8.9E-04	7.9E-04	6.0E-04	7.5E-04
P_0067	AC(17:0)-1	AC(17:0)			414.358	11.99	7.4E-04	8.9E-04	2.7E-04	9.8E-04	9.0E-04	1.5E-03	3.9E-04	4.8E-04	3.6E-04	2.9E-04	5.7E-04	7.1E-04	7.2E-04	7.0E-04	7.1E-04
P_0068	AC(17:0)-2	AC(17:0)			414.358	12.12	9.1E-04	1.1E-03	2.7E-04	1.3E-03	1.7E-03	2.0E-03	5.0E-04	5.5E-04	3.7E-04	3.6E-04	5.7E-04	8.7E-04	1.0E-03	1.2E-03	1.1E-03

P_0066	AC(17:1)-1	AC(17:1)			412.340	11.56	5.0E-04	5.3E-04	2.2E-04	6.0E-04	7.2E-04	6.4E-04	3.7E-04	4.0E-04	2.6E-04	2.6E-04	2.7E-04	5.9E-04	4.3E-04	5.8E-04	4.8E-04
P_0065	AC(17:1)-2	AC(17:1)			412.339	11.74	1.0E-04	2.2E-04	1.3E-04	1.9E-04	2.3E-04	3.0E-04	N.D.	1.2E-04	1.6E-04	1.4E-04	2.2E-04	2.5E-04	2.3E-04	1.2E-04	3.0E-04
P_0073	AC(18:0)	AC(18:0)			428.375	12.49	1.9E-02	2.6E-02	6.9E-03	3.9E-02	5.2E-02	5.8E-02	1.1E-02	1.8E-02	9.4E-03	7.8E-03	1.0E-02	1.8E-02	2.8E-02	2.9E-02	3.4E-02
P_0072	AC(18:1)	AC(18:1)			426.360	11.93	6.4E-02	7.6E-02	2.7E-02	8.3E-02	1.1E-01	1.2E-01	5.6E-02	6.5E-02	4.4E-02	2.9E-02	2.9E-02	7.1E-02	7.8E-02	8.0E-02	7.2E-02
P_0071	AC(18:2)	AC(18:2)			424.344	11.45	1.0E-02	1.4E-02	4.2E-03	1.2E-02	1.5E-02	2.1E-02	1.3E-02	9.2E-03	5.5E-03	5.6E-03	7.3E-03	1.6E-02	1.4E-02	1.3E-02	1.3E-02
P_0080	AC(20:0)	AC(20:0)			456.403	13.19	2.3E-03	4.3E-03	6.0E-04	7.1E-03	7.4E-03	8.1E-03	1.1E-03	1.9E-03	1.0E-03	1.1E-03	1.9E-03	1.8E-03	3.4E-03	5.4E-03	6.9E-03
P_0079	AC(20:1)	AC(20:1)			454.389	12.60	1.0E-02	1.3E-02	3.7E-03	1.5E-02	1.2E-02	2.1E-02	7.3E-03	8.9E-03	5.0E-03	5.0E-03	4.1E-03	8.1E-03	1.1E-02	1.3E-02	1.5E-02
P_0081	AC(21:0)	AC(21:0)			470.421	13.53	4.9E-05	N.D.	4.3E-05	1.4E-04	2.9E-04	2.2E-04	3.7E-05	4.7E-05	N.D.	N.D.	5.6E-05	7.0E-05	1.4E-04	2.2E-04	1.2E-04
P_0082	AC(22:0)	AC(22:0)			484.435	13.85	4.6E-04	8.2E-04	1.3E-04	1.3E-03	2.0E-03	1.6E-03	2.4E-04	3.6E-04	1.8E-04	1.7E-04	4.2E-04	3.2E-04	7.1E-04	1.3E-03	1.6E-03
P_0086	AC(23:0)	AC(23:0)			498.451	14.17	5.6E-05	8.7E-05	5.6E-05	1.8E-04	4.2E-04	2.2E-04	N.D.	2.7E-05	N.D.	N.D.	N.D.	4.2E-05	1.5E-04	1.8E-04	1.9E-04
C_0028	Adenine	Adenine	190	HMDB0000034	136.062	7.08	N.D.	N.D.	N.D.	5.7E-04	N.D.	N.D.	N.D.	N.D.	N.D.	N.D.	N.D.	N.D.	N.D.	N.D.	3.3E-04
C_0049	Adenosine	Adenosine	60961	HMDB0000050	268.106	9.11	1.9E-03	8.2E-04	3.6E-03	7.6E-03	5.6E-03	9.6E-03	1.1E-03	1.1E-03	8.8E-04	1.6E-03	3.2E-03	2.1E-03	5.2E-03	1.1E-02	8.0E-03
A_0069	ADP	ADP	6022	HMDB0001341	426.024	11.06	7.4E-03	6.7E-03	1.5E-03	1.0E-03	1.4E-03	7.1E-04	8.4E-03	1.2E-02	1.2E-02	4.6E-03	1.1E-03	6.5E-03	N.D.	2.3E-03	6.7E-04
A_0080	ADP-ribose	ADP-Rib	445794	HMDB0001178	558.066	8.61	N.D.	N.D.	N.D.	4.8E-04	N.D.	N.D.	N.D.	N.D.	N.D.	4.1E-04	5.3E-04	N.D.	N.D.	N.D.	N.D.
P_0043	AEA(22:6)	AEA(22:6)			372.291	13.51	5.6E-05	6.3E-05	8.2E-05	1.0E-04	N.D.	N.D.	3.7E-05	6.4E-05	N.D.	4.7E-05	4.0E-05	5.8E-05	6.4E-05	N.D.	8.1E-05
C_0004	Ala	Ala	602	HMDB0000161	90.055	8.36	4.0E-02	4.9E-02	4.0E-03	6.9E-02	3.6E-02	3.5E-02	1.7E-02	2.9E-02	2.5E-02	1.1E-02	7.2E-03	6.8E-02	3.9E-02	3.4E-02	4.3E-02
A_0059	AMP	AMP	6083	HMDB0000045	346.057	9.41	1.8E-03	2.2E-03	9.1E-04	3.4E-03	6.0E-03	3.1E-03	2.4E-03	3.8E-03	3.6E-03	2.0E-03	1.1E-03	7.8E-03	2.3E-03	4.0E-03	3.3E-03
P_0029	Anandamide	Anandamide	5281969	HMDB0004080	348.293	13.57	6.6E-05	1.0E-04	1.4E-04	9.4E-05	N.D.	1.0E-04	6.8E-05	1.5E-04	7.8E-05	1.1E-04	2.1E-04	1.1E-04	N.D.	N.D.	1.1E-04
P_0007	Androsterone	Androsterone	5879	HMDB0000031	273.219	10.99	N.D.	N.D.	N.D.	N.D.	N.D.	N.D.	N.D.	3.0E-05	N.D.	4.4E-05	N.D.	N.D.	3.5E-05	N.D.	5.8E-05
C_0062	Anserine_divalent	Anserine	112072		121.070	6.39	N.D.	N.D.	N.D.	N.D.	N.D.	N.D.	N.D.	N.D.	N.D.	N.D.	N.D.	N.D.	N.D.	N.D.	N.D.
N_0035	Arachidic acid	Arachidic acid	10467	HMDB0002212	311.295	15.94	3.7E-05	3.4E-05	6.6E-05	3.7E-05	4.0E-05	4.9E-05	4.1E-05	4.1E-05	3.6E-05	4.3E-05	5.5E-05	4.6E-05	3.9E-05	5.1E-05	3.9E-05
N_0028	Arachidonic acid	Arachidonic acid	444899	HMDB0001043	303.235	14.35	3.7E-03	6.3E-03	2.0E-02	9.4E-03	3.3E-03	3.2E-03	5.1E-03	2.3E-03	1.2E-03	5.8E-03	1.7E-02	2.7E-03	2.9E-03	2.0E-03	2.7E-03
C_0040	Arg	Arg	6322	HMDB0000517	175.120	6.61	3.2E-02	2.7E-02	6.6E-03	4.3E-02	4.1E-02	4.1E-02	1.5E-02	3.0E-02	2.9E-02	1.2E-02	6.8E-03	3.2E-02	4.2E-02	3.7E-02	3.9E-02
C_0063	Argininosuccinic acid	ArgSuccinate	16950	HMDB0000052	291.131	8.71	N.D.	N.D.	N.D.	5.0E-04	N.D.	N.D.	N.D.	3.7E-04	3.1E-04	N.D.	N.D.	4.3E-04	N.D.	2.4E-04	N.D.
A_0026	Ascorbic acid	Ascorbic acid	54670067	HMDB0000044	175.026	8.58	N.D.	N.D.	N.D.	4.2E-03	2.2E-03	3.1E-03	N.D.	N.D.	5.2E-04	N.D.	N.D.	7.9E-04	6.9E-04	1.3E-03	2.4E-03
C_0025	Asn	Asn	236	HMDB0000168	133.061	9.67	4.5E-03	4.8E-03	N.D.	7.8E-03	4.1E-03	4.0E-03	2.8E-03	2.7E-03	3.3E-03	1.3E-03	N.D.	7.4E-03	4.6E-03	4.6E-03	4.7E-03
C_0027	Asp	Asp	424	HMDB0000191	134.045	10.65	2.2E-02	2.2E-02	3.6E-03	3.5E-02	1.9E-02	1.6E-02	6.1E-03	1.9E-02	1.5E-02	6.7E-03	3.2E-03	3.1E-02	1.4E-02	2.3E-02	2.8E-02
A_0078	ATP	ATP	5957	HMDB0000538	505.989	12.03	1.8E-03	1.7E-03	N.D.	8.2E-04	N.D.	N.D.	2.3E-03	2.3E-03	3.3E-03	7.6E-04	N.D.	1.6E-03	N.D.	1.9E-03	N.D.
N_0048	Behenic acid	Behenic acid	8215	HMDB0000944	339.329	16.41	2.0E-05	1.7E-05	2.7E-05	1.7E-05	2.4E-05	2.0E-05	2.3E-05	1.7E-05	1.8E-05	2.1E-05	2.7E-05	1.9E-05	1.4E-05	1.7E-05	1.6E-05
C_0015	Betaine	Betaine	247	HMDB0000043	118.087	10.40	3.9E-03	N.D.	N.D.	1.2E-02	4.8E-03	3.0E-03	N.D.	1.1E-02	3.7E-03	N.D.	N.D.	2.7E-03	N.D.	5.2E-03	1.0E-02
C_0018	Betaine aldehyde_+H_2O	BTL	249		120.104	6.93	N.D.	N.D.	N.D.	N.D.	N.D.	N.D.	N.D.	N.D.	2.2E-04	N.D.	N.D.	N.D.	N.D.	N.D.	N.D.
C_0064	Butyrylcarnitine	Butyrylcarnitine	439829	HMDB0002013	232.156	8.86	N.D.	1.4E-03	N.D.	2.7E-03	2.0E-03	1.6E-03	N.D.	N.D.	N.D.	N.D.	N.D.	1.5E-03	1.4E-03	1.6E-03	1.5E-03
P_0048	Campesterol-1	Campesterol	173183	HMDB0002869	383.366	16.53	1.0E-04	1.2E-04	2.0E-04	1.6E-04	1.3E-04	1.2E-04	1.1E-04	1.1E-04	1.0E-04	1.4E-04	9.8E-05	1.1E-04	8.2E-05	6.6E-05	2.1E-04
P_0049	Campesterol-2	Campesterol	173183	HMDB0002869	383.366	16.41	4.8E-04	3.4E-04	1.4E-03	1.1E-04	3.1E-04	2.3E-04	2.9E-04	9.9E-04	3.1E-04	3.3E-04	1.1E-03	2.6E-04	3.0E-04	7.3E-04	2.5E-04
C_0099	Carnitine	Carnitine	85	HMDB0000062	162.114	7.89	6.5E-03	1.1E-02	1.1E-03	1.9E-02	9.8E-03	5.4E-03	3.1E-03	6.8E-03	4.8E-03	3.1E-03	1.2E-03	1.3E-02	8.7E-03	9.5E-03	1.3E-02
A_0076	CDP-choline	CDP-choline	13804	HMDB0001413	487.102	6.90	N.D.	N.D.	N.D.	N.D.	N.D.	N.D.	N.D.	N.D.	N.D.	N.D.	N.D.	N.D.	N.D.	N.D.	3.9E-04
N_0056	Chenodeoxycholic acid	Chenodeoxycholic acid	10133	HMDB0000518	391.289	12.01	N.D.	N.D.	N.D.	N.D.	N.D.	N.D.	N.D.	1.0E-05	N.D.	N.D.	N.D.	8.9E-06	N.D.	N.D.	N.D.
N_0066	Cholesterol sulfate	Cholesterol sulfate	65076	HMDB0000653	465.305	16.93	3.6E-04	1.5E-04	2.7E-04	4.2E-05	9.2E-05	1.0E-04	4.1E-04	4.5E-04	1.3E-04	1.2E-04	1.8E-04	1.5E-04	5.4E-05	1.0E-04	1.7E-04
P_0042	Cholesterol	Cholesterol	5997	HMDB0000067	370.353	16.26	5.6E-02	6.6E-02	8.6E-02	5.2E-02	5.7E-02	7.3E-02	5.0E-02	6.1E-02	5.6E-02	6.6E-02	8.2E-02	5.0E-02	5.2E-02	5.9E-02	6.0E-02
N_0059	Cholic acid	Cholic acid	221493	HMDB0000619	407.280	11.10	N.D.	N.D.	N.D.	N.D.	N.D.	1.4E-05	N.D.	2.0E-05	N.D.	6.8E-06	N.D.	N.D.	N.D.	N.D.	N.D.
C_0008	Choline	Choline	305	HMDB0000097	104.107	6.38	2.6E-02	2.8E-02	1.5E-02	3.8E-02	3.1E-02	3.4E-02	1.7E-02	2.9E-02	2.1E-02	1.6E-02	1.3E-02	3.1E-02	2.2E-02	2.8E-02	2.9E-02
P_0013	Chrysoeriol	Chrysoeriol	5280666	HMDB0030667	301.075	9.24	3.8E-04	N.D.	N.D.	N.D.	N.D.	N.D.	N.D.	N.D.	N.D.	N.D.	N.D.	N.D.	N.D.	N.D.	N.D.
N_0031	cis-11,14-Eicosadienoic acid-1	cis-11,14-Eicosadienoic acid	6439848	HMDB0005060	307.257	14.79	N.D.	N.D.	2.6E-05	2.4E-05	1.5E-05	N.D.	N.D.	N.D.	7.9E-06	2.2E-05	1.6E-05	N.D.	N.D.	N.D.	N.D.
N_0032	cis-11,14-Eicosadienoic acid-2	cis-11,14-Eicosadienoic acid	6439848	HMDB0005060	307.266	15.03	2.7E-04	5.9E-04	1.2E-03	7.6E-04	1.6E-04	2.6E-04	5.0E-04	2.2E-04	1.6E-04	5.2E-04	6.2E-04	1.7E-04	1.9E-04	1.7E-04	2.8E-04
N_0033	*cis*-11-Eicosenoic acid	cis-11-Eicosenoic acid	5282768	HMDB0002231	309.283	15.45	3.2E-04	5.2E-04	1.4E-03	6.6E-04	2.3E-04	3.3E-04	4.1E-04	2.6E-04	2.2E-04	4.8E-04	6.2E-04	1.9E-04	2.5E-04	2.3E-04	3.4E-04
N_0034	*cis*-13-Eicosenoic acid	cis-13-Eicosenoic acid	5312518	HMDB0035159	309.285	15.24	N.D.	N.D.	1.2E-05	1.6E-05	2.7E-05	2.2E-05	N.D.	N.D.	N.D.	6.6E-06	1.2E-05	N.D.	N.D.	N.D.	1.2E-05
N_0038	cis-4,7,10,13,16,19-Docosahexaenoic acid-1	DHA	445580	HMDB0002183	327.233	14.11	N.D.	2.2E-05	1.8E-05	3.7E-05	N.D.	N.D.	N.D.	N.D.	1.5E-05	1.9E-05	2.0E-05	2.0E-05	1.4E-05	N.D.	2.1E-05
N_0037	cis-4,7,10,13,16,19-Docosahexaenoic acid-2	DHA	445580	HMDB0002183	327.232	14.26	6.3E-04	8.9E-04	3.9E-03	9.2E-04	3.5E-04	5.0E-04	6.6E-04	7.1E-04	3.2E-04	9.8E-04	4.2E-03	5.6E-04	5.1E-04	4.2E-04	5.3E-04
N_0027	*cis*-5,8,11,14,17-Eicosapentaenoic acid	EPA	446284	HMDB0001999	301.219	13.94	3.8E-05	4.5E-05	1.1E-04	2.6E-04	5.5E-05	6.4E-05	4.1E-05	4.0E-05	3.4E-05	7.2E-05	1.8E-04	5.4E-05	4.2E-05	6.5E-05	4.1E-05
N_0030	*cis*-8,11,14-Eicosatrienoic acid	cis-8,11,14-Eicosatrienoic acid	5280581	HMDB0002925	305.251	14.63	2.9E-04	9.6E-04	1.6E-03	1.4E-03	2.9E-04	3.2E-04	7.9E-04	2.7E-04	1.7E-04	5.7E-04	1.1E-03	2.8E-04	2.2E-04	1.9E-04	3.4E-04
A_0032	Citric acid	Citric acid	311	HMDB0000094	191.021	27.13	8.7E-01	1.0E+00	1.1E+00	N.D.	N.D.	6.9E-03	5.6E-01	5.1E-01	5.4E-01	7.6E-01	1.1E+00	2.8E-01	N.D.	6.3E-03	7.1E-03
C_0041	Citrulline	Citrulline	9750	HMDB0000904	176.103	10.20	7.9E-04	9.3E-04	N.D.	9.4E-04	N.D.	5.4E-04	N.D.	4.9E-04	N.D.	N.D.	N.D.	8.9E-04	N.D.	8.1E-04	7.5E-04
P_0100	Coenzyme Q10	Coenzyme Q10	5281915	HMDB0001072	863.691	19.61	6.8E-03	4.7E-03	6.9E-03	4.7E-04	7.8E-04	9.7E-04	4.0E-03	5.5E-03	4.3E-03	4.2E-03	4.6E-03	1.8E-03	7.9E-04	1.1E-03	1.1E-03
P_0031	Cortisol<br>18-Hydroxycorticosterone<br>Humulone	Cortisol<br>18-Hydroxycorticosterone<br>Humulone	5754<br>11222<br>442911	HMDB000006<br>3<br>HMDB000031<br>9	363.217	8.54	N.D.	N.D.	N.D.	N.D.	2.1E-04	N.D.	3.6E-05	3.7E-05	N.D.	2.6E-05	6.6E-04	3.7E-05	1.3E-04	3.8E-05	1.1E-04
C_0022	Creatine	Creatine	586	HMDB0000064	132.078	8.20	4.3E-02	3.8E-02	5.2E-03	8.1E-02	3.8E-02	3.2E-02	2.5E-02	5.0E-02	4.7E-02	9.7E-03	7.1E-03	6.2E-02	2.3E-02	5.5E-02	5.3E-02
C_0012	Creatinine	Creatinine	588	HMDB0000562	114.067	6.78	9.2E-04	1.2E-03	N.D.	1.4E-03	9.6E-04	1.1E-03	8.0E-04	7.6E-04	6.0E-04	6.0E-03	4.4E-04	2.6E-03	1.7E-03	1.1E-02	3.1E-03
P_0063	Cycloartenol<br>Lanosterol-1	Cycloartenol<br>Lanosterol	92110<br>246983	HMDB003659<br>1<br>HMDB000125	409.384	16.43	2.2E-04	1.9E-04	1.7E-04	1.4E-04	1.7E-04	2.7E-04	2.0E-04	3.3E-04	1.5E-04	1.6E-04	1.7E-04	1.5E-04	1.3E-04	2.0E-04	1.7E-04
C_0100	Cyclohexylamine	Cyclohexylamine	7965		100.112	7.14	1.5E-04	N.D.	N.D.	N.D.	N.D.	N.D.	N.D.	N.D.	N.D.	1.3E-04	N.D.	N.D.	N.D.	N.D.	1.7E-04
C_0019	Cys	Cys	594	HMDB0000574	122.028	10.42	3.0E-03	3.8E-03	5.3E-04	6.5E-03	8.3E-04	1.1E-03	2.4E-03	2.6E-03	1.2E-03	2.2E-03	6.9E-04	2.1E-03	1.7E-03	1.8E-03	3.0E-03
C_0065	Cys-Gly	Cys-Gly	439498	HMDB0000078	179.049	8.52	4.5E-04	4.3E-04	N.D.	N.D.	N.D.	N.D.	5.6E-04	9.7E-04	3.7E-04	1.0E-03	6.5E-04	N.D.	N.D.	N.D.	N.D.
C_0066	Cysteine glutathione disulfide	Cysteine glutathione disulfide	10455148	HMDB0000656	427.098	10.73	9.9E-04	1.4E-03	N.D.	2.9E-03	6.2E-04	7.4E-04	4.5E-04	9.1E-04	5.4E-04	2.9E-04	N.D.	2.2E-03	6.9E-04	1.4E-03	6.0E-04
C_0067	Cystine	Cystine	595	HMDB0000192	241.033	10.14	1.8E-04	2.7E-04	N.D.	3.0E-03	3.6E-04	4.6E-04	N.D.	1.2E-04	N.D.	N.D.	N.D.	3.5E-04	2.5E-04	8.9E-04	1.7E-04
C_0068	Daminozide<br>Ala-Ala	Daminozide<br>Ala-Ala	15331<br>5460362	HMDB000345<br>9	161.092	8.57	1.3E-03	N.D.	1.6E-03	9.2E-04	N.D.	N.D.	N.D.	1.6E-03	1.3E-03	2.2E-03	N.D.	N.D.	N.D.	N.D.	N.D.
N_0053	Dehydroisoandrosterone 3-sulfate	Dehydroisoandrosterone 3-sulfate	12594	HMDB0001032	367.158	11.41	1.5E-05	1.2E-05	N.D.	4.2E-05	1.5E-04	3.3E-05	N.D.	4.5E-05	2.5E-05	3.7E-05	2.4E-05	3.5E-05	1.9E-04	1.8E-05	4.1E-05
N_0055	Deoxycholic acid	Deoxycholic acid	222528	HMDB0000626	391.285	12.18	N.D.	N.D.	N.D.	N.D.	N.D.	1.5E-05	N.D.	1.3E-05	N.D.	1.1E-05	N.D.	3.0E-05	N.D.	9.2E-06	N.D.
C_0101	Diethanolamine	Diethanolamine	8113	HMDB0004437	106.087	7.10	4.2E-04	N.D.	N.D.	N.D.	2.0E-04	2.6E-04	N.D.	N.D.	N.D.	N.D.	N.D.	N.D.	3.6E-04	2.4E-04	2.7E-04
A_0021	Dihydroxyacetone phosphate	DHAP	668	HMDB0001473	168.993	12.92	N.D.	N.D.	N.D.	N.D.	N.D.	N.D.	N.D.	N.D.	N.D.	N.D.	N.D.	6.3E-04	N.D.	1.3E-03	N.D.

P_0069	Diosgenin	Diosgenin	99474		415.326	14.45	1.7E-04	1.3E-04	2.8E-04	1.8E-04	1.3E-04	2.3E-04	7.1E-05	1.4E-04	1.6E-04	2.5E-04	2.9E-04	1.2E-04	1.2E-04	1.3E-04	1.6E-04
C_0069	Dyphylline	Dyphylline	3182		255.109	20.15	5.2E-03	4.4E-03	3.1E-03	3.2E-03	4.9E-03	4.3E-03	3.1E-03	2.9E-03	4.3E-03	3.9E-03	3.6E-03	4.4E-03	6.9E-03	3.7E-03	6.5E-03
N_0047	Erucic acid	Erucic acid	5281116	HMDB0002068	337.314	15.94	5.0E-05	5.1E-05	1.1E-04	1.0E-04	5.2E-05	7.0E-05	6.7E-05	5.5E-05	5.6E-05	5.3E-05	8.2E-05	5.6E-05	5.5E-05	5.8E-05	6.5E-05
P_0005	Estrone	Estrone	5870	HMDB0000145	271.167	10.01	5.5E-05	3.5E-05	4.7E-05	N.D.	N.D.	N.D.	3.1E-05	5.2E-05	N.D.	5.4E-05	N.D.	5.8E-05	N.D.	5.4E-05	7.4E-05
C_0102	Ethanolamine	Ethanolamine	700	HMDB0000149	62.061	5.90	3.1E-03	2.9E-03	8.7E-04	6.1E-03	4.5E-03	4.1E-03	1.2E-03	3.9E-03	2.0E-03	9.9E-04	9.5E-04	5.4E-03	3.4E-03	4.0E-03	3.1E-03
A_0014	Ethanolamine phosphate	EAP	1015	HMDB0000224	140.012	8.13	1.1E-02	1.1E-02	1.5E-03	1.5E-02	9.6E-03	7.9E-03	4.2E-03	9.1E-03	8.7E-03	4.5E-03	1.8E-03	1.2E-02	6.4E-03	1.0E-02	1.2E-02
P_0023	Ethyl arachidonate	Ethyl arachidonate	5367369	HMDB0062725	333.282	15.22	6.7E-04	N.D.	8.5E-05	3.1E-05	N.D.	N.D.	N.D.	N.D.	N.D.	N.D.	N.D.	N.D.	N.D.	N.D.	N.D.
N_0065	Etiocholan-3α-ol-17-one glucuronide	Etiocholan-3a-ol-17-one glucuroni	443078	HMDB0004484	465.253	10.31	N.D.	N.D.	N.D.	N.D.	N.D.	N.D.	N.D.	N.D.	N.D.	N.D.	N.D.	N.D.	N.D.	N.D.	N.D.
N_0001	FA(12:0)	FA(12:0)			199.171	13.22	2.2E-05	2.8E-05	2.6E-05	2.8E-05	1.7E-05	3.0E-05	2.3E-05	3.0E-05	1.8E-05	3.1E-05	3.8E-05	1.2E-05	1.9E-05	2.4E-05	2.4E-05
N_0002	FA(14:3)	FA(14:3)			221.154	12.57	3.3E-03	8.4E-03	7.4E-03	8.2E-03	2.2E-03	5.2E-03	4.1E-03	2.8E-03	7.7E-03	5.4E-03	6.2E-03	6.7E-03	6.0E-03	5.7E-03	7.5E-03
N_0010	FA(16:2)	FA(16:2)			251.204	13.98	N.D.	1.2E-05	3.5E-05	1.1E-05	N.D.	N.D.	1.1E-05	6.8E-06	9.5E-06	2.4E-05	N.D.	N.D.	N.D.	N.D.	N.D.
N_0014	FA(17:1)-1	FA(17:1)			267.233	14.56	1.2E-05	3.1E-05	6.9E-05	4.9E-05	9.9E-06	2.4E-05	2.7E-05	1.9E-05	1.5E-05	3.7E-05	6.8E-05	1.5E-05	1.4E-05	2.6E-05	1.3E-05
N_0013	FA(17:1)-2	FA(17:1)			267.233	14.32	2.0E-05	2.8E-05	8.3E-05	3.4E-05	6.0E-05	6.6E-05	5.0E-05	7.0E-05	3.6E-05	7.6E-05	1.2E-04	4.4E-05	1.6E-05	4.3E-05	5.2E-05
N_0023	FA(19:1)-1	FA(19:1)			295.264	14.98	1.4E-05	1.2E-05	4.1E-05	1.7E-05	3.2E-05	3.6E-05	1.2E-05	3.4E-05	1.6E-05	2.8E-05	4.2E-05	2.5E-05	1.1E-05	2.1E-05	3.4E-05
N_0024	FA(19:1)-2	FA(19:1)			295.265	15.18	1.6E-05	2.8E-05	6.7E-05	3.8E-05	N.D.	2.1E-05	2.4E-05	N.D.	1.1E-05	2.5E-05	5.1E-05	N.D.	1.1E-05	N.D.	N.D.
N_0022	FA(19:2)	FA(19:2)			293.247	14.45	N.D.	N.D.	2.4E-05	7.9E-06	1.4E-05	2.0E-05	1.5E-05	1.2E-05	N.D.	2.0E-05	2.2E-05	1.4E-05	N.D.	1.7E-05	N.D.
N_0029	FA(20:3)	FA(20:3)			305.251	14.79	2.0E-04	3.7E-04	8.4E-04	7.7E-04	3.2E-04	1.0E-04	3.1E-04	8.4E-05	1.7E-04	6.3E-04	3.0E-04	9.9E-05	2.0E-04	1.0E-04	1.1E-04
N_0046	FA(22:2)	FA(22:2)			335.298	15.56	5.2E-05	5.4E-05	1.1E-04	5.9E-05	3.2E-05	6.1E-05	4.7E-05	2.5E-05	3.2E-05	6.6E-05	5.5E-05	3.7E-05	4.5E-05	3.7E-05	4.0E-05
N_0044	FA(22:3)-1	FA(22:3)			333.282	15.18	5.8E-05	8.1E-05	1.7E-04	7.7E-05	3.9E-05	5.4E-05	6.5E-05	5.2E-05	3.6E-05	7.7E-05	8.2E-05	4.5E-05	3.7E-05	4.7E-05	5.4E-05
N_0045	FA(22:3)-2	FA(22:3)			333.283	15.25	7.9E-05	1.0E-04	2.9E-04	1.4E-04	5.4E-05	3.7E-05	9.4E-05	3.2E-05	4.3E-05	1.8E-04	9.4E-05	2.8E-05	3.8E-05	2.4E-05	7.1E-05
N_0043	FA(22:3)-3	FA(22:3)			333.282	15.06	N.D.	2.7E-05	2.8E-05	2.1E-05	N.D.	1.2E-05	N.D.	N.D.	2.6E-05	N.D.	N.D.	N.D.	2.0E-05	1.7E-05	2.3E-05
N_0041	FA(22:4)-1	FA(22:4)			331.256	14.64	2.0E-05	2.0E-05	6.4E-05	N.D.	N.D.	1.2E-05	1.2E-05	N.D.	N.D.	1.4E-05	1.9E-05	N.D.	N.D.	N.D.	1.1E-05
N_0042	FA(22:4)-2	FA(22:4)			331.266	14.85	4.1E-04	7.9E-04	2.2E-03	8.6E-04	3.6E-04	4.7E-04	6.3E-04	3.7E-04	2.1E-04	6.1E-04	1.6E-03	2.7E-04	2.0E-04	2.7E-04	4.0E-04
N_0040	FA(22:5)-1	FA(22:5)			329.249	14.47	5.6E-05	1.2E-04	4.8E-04	2.9E-04	8.2E-05	6.4E-05	1.0E-04	5.1E-05	3.7E-05	1.1E-04	5.3E-04	8.5E-05	5.7E-05	8.3E-05	5.2E-05
N_0039	FA(22:5)-2	FA(22:5)			329.249	14.64	4.7E-04	4.5E-04	1.7E-03	3.4E-04	2.0E-04	2.6E-04	3.5E-04	2.3E-04	2.5E-04	5.0E-04	9.5E-04	1.6E-04	1.2E-04	1.2E-04	2.2E-04
N_0051	FA(24:2)	FA(24:2)			363.327	16.03	1.4E-05	N.D.	2.7E-05	1.3E-05	8.7E-06	N.D.	N.D.	N.D.	7.5E-06	1.8E-05	2.3E-05	N.D.	N.D.	1.3E-05	N.D.
N_0050	FA(24:4)	FA(24:4)			359.297	15.33	8.1E-05	8.2E-05	1.8E-04	1.1E-04	3.7E-05	6.3E-05	7.6E-05	6.8E-05	1.7E-05	9.9E-05	2.0E-04	3.0E-05	2.4E-05	3.1E-05	5.6E-05
N_0049	FA(24:5)	FA(24:5)			357.281	15.05	4.9E-05	6.7E-05	2.0E-04	9.0E-05	4.5E-05	5.5E-05	6.3E-05	4.0E-05	2.8E-05	9.5E-05	1.3E-04	2.9E-05	2.9E-05	3.3E-05	4.5E-05
N_0057	FA(26:2)	FA(26:2)			391.359	16.44	7.4E-06	N.D.	2.2E-05	N.D.	N.D.	N.D.	N.D.	N.D.	N.D.	1.3E-05	1.6E-05	N.D.	N.D.	N.D.	N.D.
P_0003	Flavanone	Flavanone	10251		225.092	10.80	N.D.	2.2E-04	N.D.	1.9E-04	1.5E-04	2.0E-04	N.D.	N.D.	1.3E-04	1.7E-04	1.8E-04	N.D.	1.2E-04	1.5E-04	2.1E-04
A_0057	Fructose 1,6-diphosphate	F1,6P	172313	HMDB0001058	338.989	14.99	1.3E-03	9.7E-04	N.D.	N.D.	N.D.	N.D.	N.D.	1.0E-03	1.2E-03	N.D.	N.D.	1.6E-03	N.D.	N.D.	N.D.
A_0043	Fructose 6-phosphate	F6P	603	HMDB0000124	259.024	10.13	N.D.	N.D.	N.D.	N.D.	N.D.	N.D.	N.D.	N.D.	N.D.	N.D.	N.D.	N.D.	N.D.	N.D.	4.3E-04
C_0006	GABA	GABA	119	HMDB0000112	104.071	7.14	3.5E-04	9.3E-04	N.D.	1.2E-03	4.9E-04	1.4E-03	N.D.	1.1E-03	N.D.	N.D.	N.D.	6.5E-04	N.D.	4.5E-04	1.1E-03
C_0103	Galactosamine<br>Glucosamine	Galactosamine<br>Glucosamine	24154<br>439213	HMDB000151<br>4	180.087	8.60	7.0E-04	N.D.	N.D.	N.D.	N.D.	N.D.	N.D.	N.D.	4.0E-04	N.D.	N.D.	N.D.	N.D.	N.D.	N.D.
A_0070	GDP	GDP	8977	HMDB0001201	442.018	10.85	1.3E-03	1.5E-03	9.6E-04	N.D.	N.D.	N.D.	1.6E-03	2.4E-03	2.6E-03	1.5E-03	8.3E-04	1.9E-03	N.D.	N.D.	N.D.
C_0033	Gln	Gln	738	HMDB0000641	147.077	9.93	5.9E-02	6.8E-02	3.8E-03	1.0E-01	5.2E-02	4.3E-02	1.6E-02	3.9E-02	3.4E-02	1.3E-02	7.2E-03	1.1E-01	5.5E-02	4.9E-02	5.6E-02
C_0035	Glu	Glu	611	HMDB0000148	148.061	10.10	3.4E-02	4.2E-02	6.1E-03	8.1E-02	3.6E-02	3.3E-02	1.6E-02	3.0E-02	2.8E-02	1.9E-02	6.7E-03	5.4E-02	3.2E-02	5.4E-02	4.8E-02
A_0033	Gluconic acid	Gluconic acid	10690	HMDB0000625	195.052	8.24	N.D.	N.D.	N.D.	N.D.	N.D.	N.D.	N.D.	N.D.	8.2E-04	N.D.	N.D.	N.D.	N.D.	1.5E-03	N.D.
C_0104	Gluconolactone	1,5-Gluconolactone	7027	HMDB0000150	179.056	20.61	N.D.	9.9E-03	N.D.	7.6E-02	2.7E-03	N.D.	N.D.	2.9E-03	N.D.	N.D.	N.D.	3.9E-03	2.1E-02	1.1E-01	9.4E-03
A_0045	Glucose 1-phosphate	G1P	65533	HMDB0001586	259.025	10.26	N.D.	4.9E-04	N.D.	9.1E-04	N.D.	N.D.	N.D.	7.3E-04	N.D.	N.D.	N.D.	7.3E-04	N.D.	8.5E-04	N.D.
A_0044	Glucose 6-phosphate	G6P	5958	HMDB0001401	259.024	10.00	5.9E-04	N.D.	N.D.	2.0E-03	N.D.	4.9E-04	N.D.	N.D.	N.D.	N.D.	N.D.	3.7E-04	N.D.	N.D.	1.2E-03
C_0053	Glutathione (GSH)	GSH	124886	HMDB0000125	308.093	12.17	2.0E-02	1.9E-02	2.3E-03	1.9E-02	8.4E-03	1.0E-02	1.5E-02	2.0E-02	2.0E-02	1.0E-02	3.5E-03	1.4E-02	6.1E-03	1.3E-02	2.0E-02
C_0052	Glutathione (GSSG)_divalent	GSSG	65359		307.085	11.22	5.4E-03	6.2E-03	6.5E-04	5.2E-03	4.4E-03	5.4E-03	2.7E-03	6.6E-03	7.5E-03	1.4E-03	7.2E-04	1.0E-02	4.1E-03	6.6E-03	5.1E-03
C_0001	Gly	Gly	750	HMDB0000123	76.040	7.71	2.3E-02	2.5E-02	4.1E-03	5.0E-02	3.5E-02	3.3E-02	1.1E-02	1.8E-02	1.6E-02	7.7E-03	4.3E-03	3.6E-02	3.0E-02	3.7E-02	3.9E-02
C_0070	Gly-Leu	Gly-Leu			189.124	8.98	6.9E-03	6.4E-03	4.0E-03	3.4E-03	1.0E-03	1.8E-03	N.D.	8.9E-03	4.6E-03	6.7E-03	4.3E-03	3.5E-03	4.6E-03	N.D.	1.9E-03
C_0105	Glycerol	Glycerol	753	HMDB0000131	93.055	20.13	5.0E-02	4.2E-02	5.7E-02	2.7E-02	4.8E-02	3.7E-02	4.6E-02	2.2E-02	4.0E-02	6.9E-02	7.2E-02	4.7E-02	5.0E-02	3.7E-02	5.4E-02
A_0022	Glycerol 3-phosphate	G3P	439162	HMDB0000126	171.007	12.37	6.4E-04	8.4E-04	N.D.	6.3E-04	4.9E-04	6.9E-04	5.9E-04	N.D.	6.2E-04	N.D.	N.D.	6.9E-04	5.1E-04	6.9E-04	N.D.
C_0106	Glycerophosphocholine	GPCho	439285	HMDB0000086	258.112	19.76	2.3E-02	3.0E-02	3.7E-03	2.1E-02	2.2E-02	1.7E-02	1.1E-02	1.6E-02	1.3E-02	1.4E-02	3.7E-03	3.1E-02	9.8E-03	1.5E-02	3.0E-02
N_0062	Glycochenodeoxycholic acid	Glycochenodeoxycholic acid	12544	HMDB0000637	448.306	11.23	7.2E-06	1.6E-05	1.7E-05	3.9E-05	5.5E-05	4.9E-05	4.1E-05	5.9E-05	6.0E-05	3.0E-05	5.4E-05	5.2E-05	2.4E-05	1.3E-05	5.8E-05
N_0064	Glycocholic acid	Glycocholic acid	10140	HMDB0000138	464.303	10.42	N.D.	N.D.	1.3E-05	1.3E-04	3.3E-05	N.D.	2.1E-05	3.8E-05	7.3E-05	1.5E-05	8.1E-05	7.2E-05	9.4E-06	N.D.	1.8E-05
N_0063	Glycodeoxycholic acid	Glycodeoxycholic acid	3035026	HMDB0000631	448.307	11.48	N.D.	N.D.	1.1E-05	N.D.	2.0E-05	1.4E-05	N.D.	1.3E-05	N.D.	N.D.	1.3E-05	3.3E-05	1.2E-05	N.D.	N.D.
N_0061	Glycoursodeoxycholic acid	Glycoursodeoxycholic acid	12310288	HMDB0000708	448.298	10.26	N.D.	N.D.	N.D.	N.D.	9.2E-06	N.D.	N.D.	N.D.	N.D.	N.D.	N.D.	N.D.	N.D.	N.D.	N.D.
A_0061	GMP	GMP	6804	HMDB0001397	362.052	9.31	N.D.	N.D.	N.D.	1.3E-03	1.2E-03	7.1E-04	N.D.	N.D.	N.D.	N.D.	N.D.	5.9E-04	5.4E-04	7.8E-04	9.1E-04
C_0071	Guanidoacetic acid	Guanidoacetic acid	763	HMDB0000128	118.061	7.64	N.D.	1.3E-03	N.D.	1.8E-03	1.0E-03	N.D.	N.D.	N.D.	N.D.	N.D.	N.D.	1.7E-03	N.D.	N.D.	6.3E-04
C_0037	Guanine	Guanine	764	HMDB0000132	152.057	7.71	N.D.	N.D.	N.D.	N.D.	N.D.	N.D.	4.0E-04	N.D.	N.D.	N.D.	N.D.	N.D.	N.D.	N.D.	3.6E-04
C_0051	Guanosine	Guanosine	6802	HMDB0000133	284.101	11.53	1.4E-03	1.2E-03	N.D.	3.6E-03	1.2E-03	1.3E-03	3.8E-04	4.5E-04	3.9E-04	6.3E-04	4.5E-04	7.7E-04	1.2E-03	1.5E-03	2.2E-03
P_0076	Hecogenin	Hecogenin	91453	HMDB0038824	431.316	12.31	1.2E-03	3.9E-04	7.4E-04	1.2E-03	3.9E-04	3.7E-04	2.8E-04	3.0E-04	5.4E-04	7.3E-04	5.9E-04	5.0E-04	7.9E-04	4.1E-04	4.4E-04
N_0015	Heptadecanoic acid-1<br>FA(17:0)-1	Heptadecanoic acid<br>FA(17:0)	10465<br>0	HMDB000225<br>9	269.249	15.00	3.6E-05	4.5E-05	6.0E-05	5.1E-05	5.3E-05	5.1E-05	4.3E-05	2.3E-05	2.7E-05	3.5E-05	7.0E-05	3.0E-05	4.1E-05	3.5E-05	5.7E-05
N_0016	Heptadecanoic acid-2<br>FA(17:0)-2	Heptadecanoic acid<br>FA(17:0)	10465<br>0	HMDB000225<br>9	269.249	15.11	5.8E-05	7.5E-05	1.1E-04	8.0E-05	5.3E-05	1.1E-04	7.1E-05	6.2E-05	5.9E-05	8.5E-05	1.1E-04	6.1E-05	6.9E-05	7.1E-05	8.3E-05
A_0027	Hippuric acid	Hippuric acid	464	HMDB0000714	178.051	8.54	N.D.	N.D.	N.D.	N.D.	N.D.	N.D.	N.D.	N.D.	N.D.	5.4E-04	N.D.	N.D.	N.D.	N.D.	N.D.
C_0038	His	His	773	HMDB0000177	156.078	6.77	1.2E-02	1.1E-02	2.1E-03	1.6E-02	1.7E-02	1.9E-02	8.3E-03	1.0E-02	9.0E-03	4.9E-03	8.4E-03	1.4E-02	1.9E-02	1.4E-02	1.7E-02
C_0072	Histamine	Histamine	774	HMDB0000870	112.088	4.47	4.3E-04	1.6E-03	8.8E-04	2.1E-03	1.2E-03	1.3E-03	5.4E-04	7.3E-04	1.3E-03	1.4E-03	9.5E-04	2.3E-03	1.3E-03	2.8E-03	2.1E-03
P_0074	Hydroxyprogesterone caproate-1	Hydroxyprogesterone caproate	169870		429.302	12.06	7.1E-05	N.D.	6.6E-05	4.4E-05	6.4E-05	N.D.	N.D.	N.D.	N.D.	N.D.	5.3E-05	N.D.	N.D.	N.D.	6.2E-05
P_0075	Hydroxyprogesterone caproate-2	Hydroxyprogesterone caproate	169870		429.307	12.49	4.2E-05	6.4E-05	N.D.	9.7E-05	N.D.	N.D.	3.7E-05	N.D.	N.D.	N.D.	N.D.	N.D.	7.1E-05	1.0E-04	N.D.
C_0021	Hydroxyproline	Hydroxyproline	5810	HMDB0000725	132.067	11.09	N.D.	7.8E-04	N.D.	1.1E-03	N.D.	N.D.	N.D.	N.D.	N.D.	N.D.	N.D.	1.3E-03	4.9E-04	2.0E-03	7.6E-04
C_0073	Hypotaurine	Hypotaurine	107812	HMDB0000965	110.027	16.61	5.7E-04	1.1E-03	N.D.	1.1E-03	3.1E-03	4.2E-03	1.3E-03	1.6E-03	1.4E-03	6.6E-04	N.D.	4.9E-03	3.0E-03	5.3E-04	2.3E-03
C_0029	Hypoxanthine	Hypoxanthine	790	HMDB0000157	137.047	10.25	3.2E-03	5.9E-03	5.9E-04	2.2E-02	3.5E-03	3.8E-03	3.7E-03	2.5E-03	1.2E-03	1.7E-03	8.0E-04	4.2E-03	5.2E-03	1.0E-02	5.8E-03
C_0023	Ile	Ile	791	HMDB0000172	132.102	9.43	7.4E-02	6.3E-02	1.7E-02	9.0E-02	1.1E-01	1.1E-01	3.6E-02	7.6E-02	6.1E-02	3.4E-02	1.8E-02	6.6E-02	1.2E-01	8.0E-02	1.1E-01

A_0060	IMP	IMP	8582	HMDB0000175	347.043	9.73	N.D.	N.D.	N.D.	N.D.	6.3E-04	N.D.	N.D.	N.D.	7.8E-04	N.D.	N.D.	4.9E-04	N.D.	5.2E-04	N.D.
P_0001	Indole-3-carboxaldehyde	Indole-3-carboxaldehyde	10256	HMDB0029737	146.060	7.00	N.D.	N.D.	8.3E-04	N.D.	N.D.	3.9E-04	N.D.	N.D.	3.6E-04	4.9E-04	7.7E-04	N.D.	N.D.	N.D.	4.1E-04
C_0050	Inosine	Inosine	6021	HMDB0000195	269.090	17.85	6.6E-03	5.7E-03	4.1E-03	2.5E-02	5.8E-03	4.4E-03	2.3E-03	3.0E-03	2.0E-03	4.1E-03	4.4E-03	3.8E-03	5.1E-03	2.0E-02	9.8E-03
C_0074	Isovalerylcarnitine	Isovalerylcarnitine	6426851	HMDB0000688	246.171	9.04	N.D.	N.D.	N.D.	N.D.	1.0E-03	N.D.	N.D.	N.D.	N.D.	N.D.	N.D.	N.D.	N.D.	5.6E-04	N.D.
A_0004	Lactic acid	Lactic acid	612	HMDB0000190	89.025	10.91	7.0E-02	9.7E-02	6.7E-03	2.0E-01	7.1E-02	7.8E-02	3.5E-02	6.1E-02	3.8E-02	2.2E-02	1.4E-02	1.0E-01	6.6E-02	1.2E-01	9.3E-02
P_0064	Lanosterol-2	Lanosterol	246983	HMDB0001251	409.385	16.25	1.0E-04	N.D.	N.D.	N.D.	N.D.	N.D.	N.D.	N.D.	N.D.	N.D.	N.D.	N.D.	9.7E-05	N.D.	9.1E-05
C_0024	Leu	Leu	857	HMDB0000687	132.102	9.53	1.2E-01	9.7E-02	3.1E-02	1.1E-01	1.2E-01	1.3E-01	6.4E-02	1.1E-01	8.5E-02	5.6E-02	3.2E-02	8.6E-02	1.3E-01	9.6E-02	1.3E-01
C_0075	Lidocaine	Lidocaine	3676		235.183	9.09	N.D.	N.D.	N.D.	N.D.	N.D.	N.D.	N.D.	N.D.	N.D.	N.D.	N.D.	N.D.	N.D.	N.D.	N.D.
N_0019	Linoleic acid	Linoleic acid	5280450	HMDB0000673	279.232	14.42	5.1E-04	1.1E-03	3.1E-03	2.3E-03	2.7E-04	5.8E-04	8.3E-04	5.9E-04	4.2E-04	1.2E-03	2.4E-03	5.3E-04	3.4E-04	4.1E-04	4.8E-04
N_0018	Linolenic acid-1	Linolenic acid	5280934	HMDB0001388	277.218	14.04	1.8E-05	2.0E-05	5.8E-05	2.6E-05	N.D.	9.9E-06	1.4E-05	1.0E-05	1.6E-05	3.3E-05	7.8E-05	1.7E-05	N.D.	1.3E-05	1.4E-05
N_0017	Linolenic acid-2	Linolenic acid	5280934	HMDB0001388	277.217	13.99	1.4E-05	3.3E-05	4.0E-05	7.9E-05	9.5E-06	1.8E-05	9.6E-06	1.0E-05	N.D.	3.5E-05	5.3E-05	1.4E-05	N.D.	1.2E-05	N.D.
P_0017	Linoleyl ethanolamide	Linoleyl ethanolamide	5283446	HMDB0012252	324.289	13.56	8.7E-05	1.1E-04	1.8E-04	1.6E-04	6.0E-05	1.2E-04	1.1E-04	6.2E-05	6.3E-05	8.6E-05	1.4E-04	7.9E-05	4.3E-05	5.4E-05	1.1E-04
N_0054	LPA(14:0)	LPA(14:0)			381.205	14.95	9.7E-05	2.1E-04	2.3E-04	3.4E-04	4.6E-05	8.7E-05	7.2E-05	7.3E-05	1.6E-04	2.0E-04	1.8E-04	2.0E-04	1.6E-04	9.5E-05	2.7E-04
N_0068	LPE(18:1)-1	LPE(18:1)			478.296	13.44	N.D.	N.D.	N.D.	N.D.	N.D.	N.D.	N.D.	N.D.	N.D.	N.D.	3.6E-05	2.4E-05	N.D.	N.D.	N.D.
N_0067	LPE(18:1)-2	LPE(18:1)			478.295	13.57	N.D.	N.D.	N.D.	N.D.	N.D.	N.D.	N.D.	N.D.	N.D.	N.D.	N.D.	N.D.	N.D.	N.D.	N.D.
N_0070	LPG(16:0)-1	LPG(16:0)			483.277	15.32	N.D.	6.9E-06	1.1E-05	N.D.	N.D.	N.D.	N.D.	N.D.	6.7E-06	N.D.	N.D.	N.D.	N.D.	N.D.	N.D.
N_0069	LPG(16:0)-2	LPG(16:0)			483.274	15.21	N.D.	N.D.	N.D.	N.D.	N.D.	N.D.	N.D.	N.D.	N.D.	6.6E-06	9.8E-06	N.D.	8.0E-06	N.D.	N.D.
N_0073	LPG(18:1)-1	LPG(18:1)			509.281	15.42	N.D.	9.5E-06	N.D.	N.D.	1.3E-05	2.9E-05	7.2E-06	N.D.	1.6E-05	1.2E-05	3.5E-05	1.4E-05	7.0E-06	1.4E-05	1.7E-05
N_0074	LPG(18:1)-2	LPG(18:1)			509.286	15.34	N.D.	9.6E-06	1.1E-05	1.2E-05	1.2E-05	N.D.	N.D.	N.D.	N.D.	N.D.	5.1E-05	2.3E-05	N.D.	N.D.	N.D.
N_0060	Lupulone	Lupulone	68051	HMDB0030041	413.265	14.17	4.1E-05	4.6E-05	7.2E-05	4.7E-05	9.4E-05	9.7E-05	4.7E-05	4.4E-05	4.4E-05	5.4E-05	7.4E-05	7.4E-05	4.3E-05	5.1E-05	8.6E-05
P_0088	Lutein	Lutein	5281243	HMDB0003233	551.428	15.07	N.D.	3.9E-05	7.5E-05	3.3E-05	N.D.	N.D.	2.7E-05	2.7E-05	5.4E-05	N.D.	N.D.	4.4E-05	3.0E-05	N.D.	N.D.
C_0034	Lys	Lys	866	HMDB0000182	147.113	6.39	4.7E-02	4.1E-02	1.0E-02	6.7E-02	6.2E-02	6.1E-02	2.5E-02	4.7E-02	4.5E-02	1.7E-02	1.1E-02	5.5E-02	7.0E-02	5.8E-02	6.2E-02
A_0011	Malic acid	Malic acid	525	HMDB0000156	133.015	22.07	4.0E-03	N.D.	N.D.	N.D.	4.2E-03	3.5E-03	N.D.	3.4E-03	4.0E-03	N.D.	N.D.	N.D.	N.D.	N.D.	N.D.
C_0036	Met	Met	876	HMDB0000696	150.059	9.90	6.3E-03	4.9E-03	1.4E-03	6.3E-03	7.7E-03	7.6E-03	3.5E-03	5.8E-03	4.6E-03	2.8E-03	1.2E-03	4.5E-03	8.7E-03	7.1E-03	6.8E-03
C_0076	Methionine sulfoxide	Methionine sulfoxide	158980	HMDB0002005	166.052	10.86	N.D.	N.D.	2.8E-04	N.D.	5.8E-04	N.D.	N.D.	N.D.	6.9E-04	N.D.	N.D.	7.1E-04	1.0E-03	N.D.	8.1E-04
C_0107	Metronidazole	Metronidazole	4173		172.072	8.75	N.D.	N.D.	N.D.	N.D.	N.D.	N.D.	N.D.	N.D.	N.D.	N.D.	N.D.	N.D.	9.2E-04	N.D.	N.D.
A_0042	*myo*-Inositol 1-phosphate<br>*myo*-Inositol 3-phosphate	Myoinositol 1-phosphate<br>Myoinositol 3-phosphate	107737<br>440194	HMDB000021<br>3<br>HMDB000681	259.024	10.44	2.9E-04	N.D.	N.D.	2.8E-03	2.8E-03	2.2E-03	N.D.	N.D.	N.D.	N.D.	N.D.	N.D.	7.6E-04	1.9E-03	1.8E-03
A_0041	*myo*-Inositol 2-phosphate	Myoinositol 2-phosphate	160886		259.024	10.69	N.D.	N.D.	N.D.	N.D.	6.0E-04	N.D.	N.D.	N.D.	N.D.	N.D.	N.D.	N.D.	N.D.	4.7E-04	N.D.
N_0003	Myristic acid	Myristic acid	11005	HMDB0000806	227.202	14.06	9.8E-05	1.3E-04	1.4E-04	1.2E-04	1.2E-04	1.2E-04	1.0E-04	7.4E-05	8.9E-05	1.1E-04	1.6E-04	9.6E-05	8.9E-05	7.5E-05	1.2E-04
C_0077	*N*-(1-Deoxy-1-fructosyl)valine	N-(1-Deoxy-1-fructosyl)valine	131752247	HMDB0037844	280.140	12.87	N.D.	N.D.	N.D.	N.D.	N.D.	N.D.	N.D.	N.D.	N.D.	N.D.	N.D.	N.D.	N.D.	N.D.	4.8E-04
A_0025	*N*-Acetylaspartic acid	N-Acetylaspartic acid	65065	HMDB0000812	174.041	14.94	1.1E-02	1.0E-02	2.0E-03	1.7E-02	5.1E-03	5.6E-03	3.8E-03	1.4E-02	1.5E-02	2.8E-03	1.7E-03	1.7E-02	7.4E-03	1.0E-02	8.9E-03
A_0051	*N*-Acetylglucosamine 1-phosphate	GlcNAc-P	440272	HMDB0001367	300.051	9.70	N.D.	N.D.	N.D.	3.6E-04	N.D.	N.D.	N.D.	N.D.	N.D.	N.D.	N.D.	N.D.	N.D.	N.D.	N.D.
A_0050	*N*-Acetylglucosamine 6-phosphate	NAcGlcNP	440996	HMDB0001062	300.048	9.31	N.D.	N.D.	N.D.	5.7E-04	N.D.	N.D.	N.D.	N.D.	N.D.	N.D.	N.D.	N.D.	N.D.	N.D.	N.D.
C_0078	*N*-Acetylhistidine	N-Acetylhistidine	75619		198.089	9.08	N.D.	N.D.	N.D.	N.D.	N.D.	5.3E-04	N.D.	N.D.	6.9E-04	N.D.	N.D.	7.1E-04	N.D.	5.0E-04	N.D.
A_0052	*N*-Acetylneuraminic acid	NeuNAc	439197	HMDB0000230	308.100	7.31	3.4E-04	3.3E-04	N.D.	1.5E-03	N.D.	2.8E-04	N.D.	6.3E-04	4.0E-04	N.D.	N.D.	4.6E-04	N.D.	4.7E-04	3.2E-04
P_0002	*N*-Acetylserotonin	N-Acetylserotonin	903	HMDB0001238	219.112	5.25	N.D.	N.D.	N.D.	N.D.	N.D.	N.D.	N.D.	N.D.	N.D.	6.9E-05	N.D.	N.D.	N.D.	2.3E-04	N.D.
A_0040	*N*-Acetyltryptophan	N-Acetyltryptophan	439917		245.095	7.83	N.D.	N.D.	N.D.	N.D.	N.D.	N.D.	N.D.	1.4E-03	1.3E-03	N.D.	N.D.	N.D.	N.D.	N.D.	N.D.
C_0079	*N*-Methylproline	N-Methylproline	557		130.087	11.49	N.D.	N.D.	N.D.	N.D.	N.D.	N.D.	N.D.	N.D.	N.D.	N.D.	N.D.	N.D.	N.D.	N.D.	N.D.
C_0108	N^1-Acetylspermidine	N1-Acetylspermidine	496	HMDB0001276	188.177	5.90	4.1E-04	5.0E-04	N.D.	4.2E-04	3.9E-04	2.5E-04	4.5E-04	3.9E-04	2.8E-04	2.4E-04	N.D.	3.6E-04	4.8E-04	3.2E-04	1.7E-04
A_0046	N^2-Phenylacetylglutamine	N2-Phenylacetylglutamine	92258	HMDB0006344	263.106	7.65	N.D.	N.D.	N.D.	N.D.	N.D.	N.D.	N.D.	N.D.	N.D.	5.4E-04	N.D.	N.D.	N.D.	1.3E-03	N.D.
C_0109	N^6,N^6,N^6-Trimethyllysine	Trimethyllysine	440120	HMDB0001325	189.159	6.68	N.D.	N.D.	N.D.	N.D.	N.D.	N.D.	N.D.	N.D.	N.D.	N.D.	N.D.	N.D.	N.D.	N.D.	N.D.
C_0110	N^8-Acetylspermidine	N8-Acetylspermidine	123689	HMDB0002189	188.177	5.97	N.D.	2.7E-04	N.D.	2.4E-04	N.D.	1.5E-04	N.D.	4.4E-04	N.D.	N.D.	N.D.	4.6E-04	N.D.	1.5E-04	2.0E-04
A_0083	NAD$^+$	NAD+	5893	HMDB0000902	662.102	6.67	4.2E-04	4.8E-04	N.D.	3.0E-04	4.8E-04	N.D.	5.6E-04	7.9E-04	6.6E-04	N.D.	N.D.	7.1E-04	N.D.	4.3E-04	N.D.
N_0052	Nervonic acid	Nervonic acid	5281120	HMDB0002368	365.342	16.38	7.0E-05	6.2E-05	1.1E-04	6.9E-05	4.1E-05	5.1E-05	6.2E-05	5.4E-05	4.9E-05	8.9E-05	7.0E-05	6.4E-05	5.3E-05	5.0E-05	5.1E-05
C_0080	Nicotinamide	Nicotinamide	936	HMDB0001406	123.056	6.87	2.9E-03	2.5E-03	1.4E-03	6.6E-03	3.1E-03	3.6E-03	2.0E-03	2.4E-03	2.0E-03	2.3E-03	1.7E-03	2.4E-03	3.7E-03	3.1E-03	4.2E-03
C_0081	*O*-Acetylcarnitine	ALCAR	439756	HMDB0000201	204.124	8.37	9.1E-03	1.1E-02	N.D.	2.0E-02	1.4E-02	1.0E-02	5.2E-03	1.3E-02	7.0E-03	3.0E-03	N.D.	1.8E-02	9.9E-03	1.1E-02	1.4E-02
C_0082	*O*-Acetylhomoserine<br>2-Aminoadipic acid	O-Acetylhomoserine<br>2-Aminoadipic acid	439389<br>92136	HMDB000051<br>0	162.078	10.12	4.7E-03	3.9E-03	N.D.	6.0E-03	3.2E-03	2.1E-03	2.0E-03	3.3E-03	3.6E-03	N.D.	N.D.	6.0E-03	4.2E-03	7.0E-03	2.8E-03
N_0020	Oleic acid	Oleic acid	445639	HMDB0000207	281.249	14.89	2.0E-03	4.5E-03	1.3E-02	6.8E-03	1.8E-03	2.3E-03	3.5E-03	2.7E-03	1.9E-03	4.0E-03	6.9E-03	1.6E-03	1.5E-03	1.6E-03	2.2E-03
P_0018	Oleoyl ethanolamide<br>AEA(18:1)	Oleoyl ethanolamide<br>AEA(18:1)	5283454<br>0	HMDB000208<br>8	326.304	14.06	1.2E-03	1.2E-03	1.8E-03	1.6E-03	7.9E-04	1.5E-03	1.1E-03	1.4E-03	1.0E-03	1.4E-03	8.7E-04	1.1E-03	9.3E-04	1.5E-03	8.1E-04
C_0083	Ophthalmic acid	Ophthalmic acid	7018721	HMDB0005765	290.135	12.16	N.D.	N.D.	N.D.	1.7E-03	N.D.	N.D.	N.D.	7.0E-04	N.D.	N.D.	N.D.	9.8E-04	N.D.	1.1E-03	N.D.
C_0026	Ornithine	Ornithine	389	HMDB0000214	133.098	6.34	4.2E-03	2.1E-03	4.3E-04	4.4E-03	1.3E-03	1.2E-03	1.1E-03	1.7E-03	1.9E-03	5.6E-04	4.7E-04	3.3E-03	3.3E-03	4.3E-03	1.6E-03
A_0016	*p*-Anisic acid<br>*o*-Hydroxyphenylacetic acid<br>Mandelic acid<br>Phenoxyacetic acid<br>2-(Hydroxymethyl)benzoic acid	p-Anisic acid<br>2-HPAA<br>Mandelic acid<br>Phenoxyacetic acid<br>2-(Hydroxymethyl)benzoic acid	7478<br>11970<br>11914<br>19188<br>11920	HMDB000110<br>1<br>HMDB000066<br>9<br>HMDB000070<br>3	151.041	9.24	N.D.	N.D.	N.D.	N.D.	N.D.	N.D.	N.D.	N.D.	N.D.	N.D.	N.D.	N.D.	N.D.	N.D.	N.D.
A_0013	*p*-Toluic acid<br>*m*-Toluic acid<br>*o*-Toluic acid	p-Toluic acid<br>m-Toluic acid<br>o-Toluic acid	7470<br>7418<br>8373		135.046	9.28	N.D.	N.D.	N.D.	N.D.	N.D.	7.3E-04	N.D.	N.D.	N.D.	N.D.	N.D.	N.D.	9.3E-04	6.2E-04	5.7E-04
N_0012	Palmitic acid	Palmitic acid	985	HMDB0000220	255.233	14.79	2.7E-03	3.5E-03	6.0E-03	3.9E-03	3.5E-03	4.9E-03	3.1E-03	3.1E-03	3.0E-03	3.6E-03	5.6E-03	2.7E-03	2.7E-03	3.0E-03	3.8E-03
N_0011	Palmitoleic acid	Palmitoleic acid	445638	HMDB0003229	253.218	14.21	1.2E-04	2.1E-04	6.3E-04	5.1E-04	9.8E-05	1.1E-04	1.8E-04	1.6E-04	1.6E-04	2.7E-04	4.6E-04	1.1E-04	9.1E-05	1.6E-04	1.2E-04
P_0062	Palmitoylcarnitine	Palmitoylcarnitine	11953816	HMDB0000222	400.344	11.75	6.1E-02	8.9E-02	2.3E-02	1.4E-01	1.8E-01	1.5E-01	4.6E-02	6.8E-02	4.5E-02	2.9E-02	3.8E-02	8.0E-02	8.9E-02	9.2E-02	9.8E-02
P_0011	Palmitoylethanolamide	Palmitoylethanolamide	4671	HMDB0002100	300.288	13.89	1.5E-03	1.4E-03	2.1E-03	1.7E-03	1.2E-03	2.1E-03	1.4E-03	2.0E-03	1.6E-03	2.0E-03	1.8E-03	1.6E-03	1.4E-03	1.8E-03	1.4E-03
A_0037	Pantothenic acid	Pantothenic acid	6613	HMDB0000210	218.105	7.77	3.8E-04	N.D.	N.D.	N.D.	4.4E-04	4.9E-04	N.D.	N.D.	4.2E-04	N.D.	N.D.	N.D.	5.7E-04	4.1E-04	6.5E-04
N_0004	Pentadecanoic acid	Pentadecanoic acid	13849	HMDB0000826	241.218	14.44	3.2E-05	3.4E-05	4.7E-05	3.8E-05	3.2E-05	5.0E-05	2.2E-05	2.3E-05	2.4E-05	4.5E-05	6.2E-05	3.9E-05	2.3E-05	4.0E-05	4.8E-05
C_0039	Phe	Phe	994	HMDB0000159	166.087	10.23	3.6E-02	3.1E-02	1.1E-02	4.2E-02	4.5E-02	4.5E-02	2.5E-02	3.7E-02	3.2E-02	2.0E-02	1.1E-02	3.1E-02	4.8E-02	3.5E-02	4.6E-02
A_0035	Phosphocreatine	Phosphocreatine	9548602	HMDB0001511	210.029	12.48	N.D.	5.9E-04	N.D.	N.D.	N.D.	N.D.	N.D.	9.0E-04	8.1E-04	N.D.	N.D.	2.2E-03	N.D.	N.D.	N.D.
C_0111	Phosphorylcholine	Phosphorylcholine	1014	HMDB0001565	184.074	18.78	6.9E-03	4.7E-03	N.D.	1.1E-02	7.0E-03	4.6E-03	3.8E-03	5.4E-03	5.9E-03	3.5E-03	N.D.	7.4E-03	4.5E-03	1.0E-02	8.9E-03

P_0016	Phytosphingosine	Phytosphingosine	122121	HMDB0004610	318.300	11.14	9.1E-05	N.D.	N.D.	2.3E-04	2.7E-04	1.6E-04	8.4E-05	N.D.	8.1E-05	N.D.	9.7E-05	6.9E-05	2.9E-04	1.8E-04	2.0E-04
C_0084	Pipecolic acid	Pipecolic acid	439227	HMDB0000070	130.087	9.50	N.D.	N.D.	N.D.	7.7E-04	N.D.	N.D.	N.D.	4.2E-04	N.D.	N.D.	N.D.	5.3E-04	N.D.	5.4E-04	N.D.
C_0112	Piperidine	Piperidine	8082		86.096	6.65	N.D.	N.D.	N.D.	N.D.	N.D.	N.D.	N.D.	N.D.	N.D.	N.D.	N.D.	N.D.	N.D.	N.D.	N.D.
P_0008	Piperine-1	Piperine	638024	HMDB0029377	286.142	10.50	N.D.	N.D.	N.D.	N.D.	N.D.	2.3E-04	N.D.	N.D.	N.D.	N.D.	N.D.	1.6E-04	1.1E-04	N.D.	7.6E-05
P_0009	Piperine-2	Piperine	638024	HMDB0029377	286.142	10.60	N.D.	N.D.	N.D.	N.D.	N.D.	1.7E-04	N.D.	N.D.	N.D.	N.D.	N.D.	1.1E-04	5.2E-05	N.D.	6.1E-05
N_0058	Pregnenolone sulfate	Pregnenolone sulfate	105074	HMDB0000774	395.189	12.43	1.3E-05	N.D.	N.D.	N.D.	2.8E-05	N.D.	7.3E-06	1.3E-05	N.D.	N.D.	N.D.	9.5E-06	2.7E-05	N.D.	N.D.
C_0013	Pro	Pro	614	HMDB0000162	116.071	9.97	2.2E-02	1.8E-02	2.7E-03	3.0E-02	1.5E-02	1.5E-02	1.0E-02	1.4E-02	1.4E-02	5.4E-03	4.3E-03	2.9E-02	2.9E-02	3.4E-02	1.7E-02
C_0085	Propionylcarnitine<br>XC0061	Propionylcarnitine<br>XC0061	188824<br>0	HMDB000082<br>4	218.139	8.62	N.D.	N.D.	N.D.	1.1E-03	N.D.	N.D.	N.D.	N.D.	N.D.	N.D.	N.D.	N.D.	N.D.	N.D.	1.0E-03
P_0024	Prostaglandin B2	Prostaglandin B2	5280881	HMDB0004236	335.226	10.83	5.5E-05	9.2E-05	4.4E-05	4.8E-05	6.2E-05	N.D.	7.5E-05	4.1E-05	6.2E-05	6.6E-05	1.3E-04	8.8E-05	6.9E-05	1.0E-04	N.D.
C_0002	Putrescine	Putrescine	1045	HMDB0001414	89.108	4.41	3.2E-04	3.7E-04	N.D.	1.4E-03	3.1E-04	4.5E-04	1.2E-03	1.2E-03	1.0E-03	2.0E-04	N.D.	7.6E-04	5.0E-04	5.8E-04	5.3E-04
C_0086	Pyridoxal	Pyridoxal	1050	HMDB0001545	168.067	8.13	3.9E-04	4.1E-04	N.D.	3.2E-04	9.9E-04	1.2E-03	N.D.	6.6E-04	N.D.	3.7E-04	N.D.	N.D.	7.5E-04	5.1E-04	4.5E-04
P_0004	Retinol	Retinol	445354	HMDB0000305	269.224	14.04	1.7E-04	8.9E-05	N.D.	1.2E-04	N.D.	9.2E-05	N.D.	1.7E-04	1.0E-04	2.3E-04	N.D.	2.6E-05	3.0E-04	4.8E-05	1.6E-04
P_0045	Riboflavin	Riboflavin	493570	HMDB0000244	377.147	6.00	5.6E-05	1.0E-04	7.9E-05	5.7E-05	9.0E-05	5.8E-05	7.0E-05	6.8E-05	7.0E-05	1.2E-04	8.6E-05	5.1E-05	5.8E-05	4.8E-05	6.0E-05
N_0026	Ricinoleic acid-1	Ricinoleic acid	643684	HMDB0034297	297.243	12.96	1.4E-05	3.1E-05	3.4E-05	1.8E-05	2.2E-05	7.3E-05	2.8E-05	2.5E-05	2.6E-05	3.7E-05	4.0E-05	2.2E-05	3.3E-05	3.3E-05	3.9E-05
N_0025	Ricinoleic acid-2	Ricinoleic acid	643684	HMDB0034297	297.243	12.78	3.7E-05	5.0E-05	6.9E-05	9.3E-05	8.4E-05	7.9E-05	7.9E-05	6.6E-05	4.1E-05	3.1E-05	7.8E-05	3.7E-05	6.8E-05	7.5E-05	9.7E-05
C_0087	*S*-Adenosylhomocysteine	SAHC	439155	HMDB0000939	385.133	8.10	N.D.	N.D.	N.D.	2.5E-04	1.5E-04	1.2E-04	1.1E-04	N.D.	N.D.	N.D.	N.D.	N.D.	N.D.	2.7E-04	1.9E-04
C_0054	*S*-Adenosylmethionine	SAM	34755	HMDB0001185	399.146	6.61	6.9E-04	7.1E-04	N.D.	6.8E-04	3.4E-04	4.7E-04	5.0E-04	8.0E-04	4.8E-04	3.6E-04	N.D.	5.4E-04	2.7E-04	5.6E-04	6.6E-04
C_0005	Sarcosine	Sarcosine	1088	HMDB0000271	90.055	8.76	N.D.	N.D.	N.D.	5.1E-04	N.D.	N.D.	N.D.	N.D.	N.D.	N.D.	N.D.	N.D.	N.D.	2.9E-04	N.D.
C_0088	SDMA	SDMA	169148	HMDB0003334	203.151	7.23	N.D.	N.D.	N.D.	N.D.	N.D.	N.D.	N.D.	N.D.	N.D.	3.4E-04	N.D.	N.D.	N.D.	6.4E-04	3.7E-04
A_0049	Sedoheptulose 7-phosphate	S7P	165007	HMDB0001068	289.035	9.75	N.D.	N.D.	N.D.	5.6E-04	N.D.	N.D.	N.D.	N.D.	N.D.	N.D.	N.D.	N.D.	N.D.	N.D.	N.D.
C_0009	Ser	Ser	617	HMDB0000187	106.050	9.23	1.9E-02	1.7E-02	4.2E-03	2.4E-02	2.2E-02	2.2E-02	8.2E-03	1.9E-02	1.6E-02	5.8E-03	3.8E-03	2.1E-02	2.1E-02	1.9E-02	2.5E-02
C_0089	Ser-Glu	Ser-Glu			235.094	9.59	6.6E-04	1.0E-03	1.3E-03	5.0E-04	N.D.	N.D.	7.6E-04	8.6E-04	7.0E-04	1.1E-03	N.D.	N.D.	N.D.	N.D.	2.7E-04
P_0058	Sitosterol-1	Sitosterol	222284	HMDB0000852	397.378	16.38	N.D.	N.D.	4.4E-05	N.D.	N.D.	N.D.	7.0E-05	N.D.	N.D.	3.4E-05	N.D.	3.6E-05	N.D.	N.D.	N.D.
P_0059	Sitosterol-2	Sitosterol	222284	HMDB0000852	397.385	16.57	1.3E-04	1.1E-04	3.4E-04	3.7E-05	1.9E-04	1.2E-04	1.6E-04	2.0E-04	1.3E-04	6.7E-05	2.5E-04	1.2E-04	9.3E-05	1.8E-04	1.5E-04
P_0061	Solanidine	Solanidine	65727	HMDB0003236	398.343	8.05	N.D.	N.D.	N.D.	N.D.	5.0E-04	N.D.	N.D.	N.D.	N.D.	3.3E-05	2.7E-04	N.D.	1.1E-04	1.4E-04	N.D.
C_0032	Spermidine	Spermidine	1102	HMDB0001257	146.166	4.24	2.2E-02	2.0E-02	3.5E-03	1.3E-03	9.3E-04	8.2E-04	1.2E-02	2.3E-02	1.9E-02	8.5E-03	3.4E-03	1.7E-02	1.7E-03	1.3E-03	1.3E-03
C_0043	Spermine	Spermine	1103	HMDB0001256	203.224	4.18	2.7E-03	3.4E-03	7.8E-04	N.D.	N.D.	N.D.	1.3E-03	2.5E-03	1.6E-03	1.6E-03	6.7E-04	1.6E-03	N.D.	N.D.	N.D.
P_0014	Sphinganine	Sphinganine	91486	HMDB0000269	302.308	11.42	5.8E-04	7.5E-04	4.7E-04	1.8E-03	2.1E-03	2.1E-03	5.8E-04	4.9E-04	5.9E-04	7.1E-04	7.2E-04	5.0E-04	2.5E-03	1.6E-03	2.5E-03
P_0021	Sphinganine(d20:0)	Sphinganine(d20:0)			330.334	11.82	4.8E-05	3.4E-05	N.D.	6.4E-05	1.5E-04	1.4E-04	N.D.	4.7E-05	4.3E-05	N.D.	N.D.	N.D.	1.7E-04	1.1E-04	1.6E-04
P_0096	Sphingomyelin(d18:1/16:0)-1	Sphingomyelin(d18:1/16:0)	5283590	HMDB0010169	703.578	16.29	1.9E-04	3.3E-04	3.0E-04	2.9E-04	3.2E-04	4.2E-04	2.1E-04	2.5E-04	2.8E-04	1.9E-04	2.9E-04	2.5E-04	3.0E-04	2.7E-04	3.9E-04
P_0097	Sphingomyelin(d18:1/16:0)-2	Sphingomyelin(d18:1/16:0)	5283590	HMDB0010169	703.591	16.45	1.6E-04	2.9E-04	2.6E-04	2.7E-04	4.0E-04	4.0E-04	2.8E-04	2.4E-04	2.6E-04	2.0E-04	3.3E-04	2.5E-04	2.4E-04	2.7E-04	3.8E-04
P_0098	Sphingomyelin(d18:1/18:0)	Sphingomyelin(d18:1/18:0)	5283588	HMDB0001348	731.624	16.79	4.4E-05	9.8E-05	8.6E-05	9.5E-05	9.1E-05	9.4E-05	1.0E-04	7.0E-05	6.4E-05	8.2E-05	1.4E-04	9.0E-05	6.3E-05	7.3E-05	1.0E-04
P_0012	Sphingosine	Sphingosine	5280335	HMDB0000252	300.288	11.23	7.5E-04	1.0E-03	1.3E-03	2.9E-03	2.2E-03	1.9E-03	1.4E-03	9.6E-04	9.1E-04	1.4E-03	1.7E-03	8.3E-04	2.8E-03	2.1E-03	2.1E-03
P_0019	Sphingosine(d20:1)	Sphingosine(d20:1)			328.321	11.96	3.7E-05	N.D.	N.D.	5.7E-05	8.4E-05	8.4E-05	N.D.	5.5E-05	6.8E-05	N.D.	N.D.	7.0E-05	5.4E-05	6.0E-05	1.5E-04
C_0113	Stachydrine	Stachydrine	115244	HMDB0004827	144.103	10.61	N.D.	N.D.	N.D.	N.D.	9.5E-04	N.D.	N.D.	N.D.	N.D.	N.D.	N.D.	N.D.	N.D.	N.D.	3.8E-04
N_0021	Stearic acid	Stearic acid	5281	HMDB0000827	283.264	15.41	4.6E-03	4.6E-03	6.8E-03	4.9E-03	5.5E-03	7.5E-03	4.6E-03	4.4E-03	4.4E-03	5.1E-03	7.3E-03	4.9E-03	4.8E-03	4.9E-03	5.8E-03
P_0020	Stearoyl ethanolamide	Stearoyl ethanolamide	27902	HMDB0013078	328.321	14.62	2.0E-03	1.2E-03	2.4E-03	2.1E-03	1.5E-03	2.7E-03	1.5E-03	1.8E-03	1.3E-03	1.6E-03	1.5E-03	2.1E-03	1.6E-03	2.3E-03	1.2E-03
P_0055	Stigmasterol-1	Stigmasterol	5280794	HMDB0000937	395.368	16.26	4.1E-04	2.3E-04	5.8E-04	1.3E-04	3.1E-04	2.1E-04	1.7E-04	4.2E-04	2.6E-04	2.3E-04	6.1E-04	2.5E-04	2.5E-04	3.4E-04	1.7E-04
P_0054	Stigmasterol-2	Stigmasterol	5280794	HMDB0000937	395.367	16.38	4.1E-04	4.2E-04	6.2E-04	3.2E-04	2.4E-04	3.1E-04	3.9E-04	3.8E-04	3.2E-04	3.3E-04	4.7E-04	2.8E-04	2.1E-04	3.2E-04	4.7E-04
C_0114	Streptomycin sulfate_+H_2O_divalent	Streptomycin sulfate_+H2O_divalı	19649		300.647	6.38	5.7E-03	6.1E-03	3.1E-03	9.5E-05	2.3E-04	2.6E-04	3.5E-03	5.6E-03	5.5E-03	3.5E-03	1.8E-03	2.4E-03	5.4E-04	N.D.	2.6E-04
C_0090	Taurine	Taurine	1123	HMDB0000251	126.022	20.12	7.0E-03	6.8E-03	8.9E-04	1.1E-02	6.3E-03	7.5E-03	3.5E-03	4.2E-03	3.9E-03	2.8E-03	1.6E-03	1.1E-02	6.7E-03	4.1E-03	8.0E-03
N_0072	Taurochenodeoxycholic acid	Taurochenodeoxycholic acid	387316	HMDB0000951	498.288	12.51	N.D.	N.D.	N.D.	8.4E-06	N.D.	N.D.	N.D.	1.3E-05	1.8E-05	8.2E-06	4.2E-05	2.9E-05	N.D.	N.D.	N.D.
N_0075	Taurocholic acid	Taurocholic acid	46783527	HMDB0000036	514.287	11.64	N.D.	N.D.	N.D.	1.8E-05	N.D.	N.D.	N.D.	1.1E-05	2.8E-05	N.D.	3.6E-05	3.3E-05	N.D.	N.D.	N.D.
N_0071	Taurodeoxycholic acid	Taurodeoxycholic acid	92042745	HMDB0000896	498.285	12.82	N.D.	N.D.	N.D.	N.D.	N.D.	N.D.	N.D.	N.D.	N.D.	N.D.	N.D.	2.3E-05	N.D.	N.D.	N.D.
N_0076	Taurolithocholic acid 3-sulfate	Taurolithocholic acid 3-sulfate	440071	HMDB0002580	562.254	14.23	N.D.	4.6E-06	N.D.	N.D.	N.D.	N.D.	1.5E-05	7.9E-06	N.D.	N.D.	N.D.	2.7E-05	N.D.	N.D.	N.D.
A_0017	Terephthalic acid	Terephthalic acid	7489	HMDB0002428	165.020	17.08	1.5E-03	1.9E-03	2.1E-03	1.8E-03	1.9E-03	1.8E-03	1.7E-03	1.8E-03	1.7E-03	1.9E-03	2.3E-03	1.7E-03	2.2E-03	1.6E-03	1.8E-03
C_0091	Theobromine	Theobromine	5429	HMDB0002825	181.072	20.14	9.4E-03	9.5E-03	N.D.	1.1E-02	5.7E-03	5.6E-03	5.1E-03	8.4E-03	7.2E-03	3.9E-03	N.D.	1.0E-02	4.1E-03	7.1E-03	7.6E-03
C_0115	Thiamine	Thiamine	1130	HMDB0000235	265.114	6.12	6.9E-04	6.6E-04	3.3E-04	8.6E-04	1.4E-03	1.4E-03	4.5E-04	8.3E-04	4.6E-04	4.3E-04	3.9E-04	6.8E-04	1.3E-03	7.5E-04	1.0E-03
C_0092	Thiaproline	Thiaproline	9934		134.028	12.85	N.D.	N.D.	N.D.	1.1E-03	5.0E-04	4.5E-04	1.8E-04	N.D.	N.D.	N.D.	N.D.	3.0E-04	5.6E-04	6.1E-04	2.2E-03
C_0017	Thr	Thr	6288	HMDB0000167	120.066	9.70	4.9E-02	4.3E-02	1.0E-02	5.2E-02	5.5E-02	5.5E-02	1.9E-02	4.8E-02	3.9E-02	1.5E-02	8.9E-03	6.6E-02	5.4E-02	4.8E-02	5.4E-02
A_0012	Threonic acid	Threonic acid	5460407	HMDB0000943	135.029	9.40	N.D.	N.D.	N.D.	N.D.	N.D.	N.D.	N.D.	N.D.	N.D.	N.D.	N.D.	N.D.	N.D.	N.D.	N.D.
P_0099	Thyroxine	Thyroxine	5819	HMDB0000248	777.691	9.78	N.D.	N.D.	N.D.	2.7E-05	N.D.	N.D.	N.D.	N.D.	N.D.	N.D.	N.D.	N.D.	N.D.	N.D.	N.D.
P_0093	Trilaurin-1	Trilaurin	10851	HMDB0011188	656.573	17.61	1.4E-04	3.2E-04	5.8E-04	1.2E-04	1.5E-04	7.2E-05	1.9E-04	3.0E-04	1.2E-04	2.5E-04	4.1E-04	8.6E-05	7.3E-05	8.1E-05	1.1E-04
P_0095	Trilaurin-2	Trilaurin	10851	HMDB0011188	656.598	17.86	N.D.	3.2E-04	N.D.	1.0E-04	N.D.	1.1E-04	2.8E-04	2.4E-04	9.5E-05	N.D.	N.D.	7.0E-05	N.D.	5.0E-05	1.2E-04
P_0094	Trilaurin-3	Trilaurin	10851	HMDB0011188	656.583	18.05	4.1E-05	2.1E-04	1.5E-04	6.7E-05	4.9E-05	5.5E-05	7.6E-05	4.3E-05	5.7E-05	N.D.	N.D.	6.3E-05	6.9E-05	4.8E-05	N.D.
C_0116	Trimethylamine *N*-oxide	Trimethylamine N-oxide	1145	HMDB0000925	76.076	6.14	N.D.	N.D.	N.D.	N.D.	N.D.	N.D.	N.D.	N.D.	N.D.	4.6E-04	N.D.	N.D.	N.D.	7.1E-04	N.D.
C_0044	Trp	Trp	1148	HMDB0000929	205.099	10.16	6.0E-03	4.9E-03	3.2E-03	6.5E-03	6.9E-03	6.2E-03	5.4E-03	6.5E-03	5.1E-03	3.8E-03	3.1E-03	5.0E-03	7.0E-03	5.0E-03	6.3E-03
C_0042	Tyr	Tyr	1153	HMDB0000158	182.082	10.47	1.8E-02	1.6E-02	5.6E-03	2.2E-02	2.4E-02	2.3E-02	1.2E-02	1.8E-02	1.6E-02	9.1E-03	5.8E-03	1.5E-02	2.5E-02	1.7E-02	2.3E-02
C_0093	Tyr-Arg_divalent	Tyr-Arg_divalent	123804		169.596	7.15	N.D.	N.D.	N.D.	N.D.	N.D.	N.D.	3.1E-04	N.D.	N.D.	N.D.	N.D.	N.D.	N.D.	N.D.	N.D.
A_0066	UDP	UDP	6031	HMDB0000295	402.996	11.89	1.2E-03	1.3E-03	N.D.	N.D.	N.D.	N.D.	1.5E-03	2.6E-03	3.2E-03	N.D.	N.D.	2.1E-03	N.D.	N.D.	N.D.
A_0081	UDP-glucose<br>UDP-galactose	UDP-Glc<br>UDP-Gal	8629<br>23724458	HMDB000028<br>6	565.052	8.77	N.D.	N.D.	N.D.	7.6E-04	N.D.	N.D.	N.D.	N.D.	N.D.	N.D.	N.D.	N.D.	N.D.	N.D.	N.D.
A_0082	UDP-*N*-acetylgalactosamine<br>UDP-*N*-acetylglucosamine	UDP-N-acetylgalactosamine<br>UDP-GlcNAc	23724461<br>445675	HMDB000030<br>4<br>HMDB000029	606.075	8.56	1.4E-03	1.6E-03	4.8E-04	2.3E-03	1.1E-03	1.2E-03	1.1E-03	1.9E-03	2.0E-03	8.5E-04	5.5E-04	2.2E-03	7.2E-04	1.3E-03	1.4E-03
A_0055	UMP	UMP	6030	HMDB0000288	323.030	9.96	N.D.	N.D.	N.D.	1.1E-03	1.4E-03	8.9E-04	N.D.	5.7E-04	5.0E-04	N.D.	N.D.	1.1E-03	6.1E-04	1.0E-03	5.9E-04
C_0117	Urea	Urea	1176	HMDB0000294	61.040	19.31	1.0E-02	N.D.	N.D.	6.6E-02	N.D.	N.D.	N.D.	N.D.	N.D.	8.5E-03	N.D.	3.8E-02	5.8E-02	1.1E-01	1.6E-02
A_0019	Uric acid	Uric acid	1175	HMDB0000289	167.021	9.03	5.0E-04	1.3E-03	N.D.	1.3E-03	6.7E-04	8.0E-04	5.2E-04	3.9E-04	4.0E-04	1.7E-03	N.D.	2.0E-03	2.0E-03	4.7E-03	1.8E-03
C_0048	Uridine	Uridine	6029	HMDB0000296	245.081	20.18	N.D.	N.D.	N.D.	2.3E-03	1.3E-03	1.2E-03	N.D.	N.D.	N.D.	N.D.	N.D.	N.D.	N.D.	1.7E-03	1.7E-03
A_0075	UTP	UTP	6133	HMDB0000285	482.962	12.85	N.D.	N.D.	N.D.	N.D.	N.D.	N.D.	N.D.	N.D.	1.0E-03	N.D.	N.D.	N.D.	N.D.	N.D.	N.D.
C_0014	Val	Val	1182	HMDB0000883	118.087	9.26	7.2E-02	5.8E-02	1.5E-02	8.4E-02	9.0E-02	8.9E-02	3.8E-02	6.8E-02	5.8E-02	3.0E-02	1.6E-02	6.5E-02	1.0E-01	7.5E-02	9.4E-02

A_0023	XA0013	XA0013			172.990	10.95	N.D.	N.D.	N.D.	N.D.	N.D.	N.D.	N.D.	N.D.	N.D.	8.9E-04	N.D.	N.D.	N.D.	N.D.	N.D.
A_0031	XA0019	XA0019			191.020	8.41	N.D.	N.D.	N.D.	N.D.	N.D.	N.D.	N.D.	8.5E-04	7.1E-04	N.D.	N.D.	N.D.	N.D.	N.D.	N.D.
A_0071	XA0065	XA0065			445.054	7.06	7.3E-04	7.0E-04	N.D.	1.1E-03	6.4E-04	4.4E-04	4.5E-04	1.1E-03	1.1E-03	N.D.	N.D.	9.6E-04	4.8E-04	6.0E-04	5.8E-04
C_0121	Xanthine	Xanthine	1188	HMDB0000292	153.042	17.83	N.D.	N.D.	N.D.	9.0E-04	N.D.	N.D.	N.D.	N.D.	N.D.	N.D.	N.D.	N.D.	N.D.	N.D.	N.D.
C_0118	XC0016	XC0016			129.066	8.13	1.6E-03	1.6E-03	N.D.	1.1E-03	1.3E-03	1.6E-03	N.D.	N.D.	N.D.	N.D.	N.D.	2.4E-03	4.0E-03	7.3E-04	1.0E-03
C_0119	XC0120	XC0120			298.054	8.76	N.D.	N.D.	N.D.	8.5E-04	1.8E-04	1.6E-04	N.D.	N.D.	N.D.	N.D.	N.D.	N.D.	N.D.	1.8E-04	N.D.
C_0120	XC0132	XC0132			325.159	8.04	N.D.	N.D.	N.D.	N.D.	N.D.	N.D.	N.D.	N.D.	N.D.	N.D.	N.D.	N.D.	N.D.	3.7E-04	N.D.
P_0092	Zeaxanthin	Zeaxanthin	5280899	HMDB0002789	569.427	15.06	2.3E-04	3.2E-04	1.9E-04	2.9E-04	3.4E-04	5.4E-04	1.2E-04	3.0E-04	2.8E-04	6.3E-05	N.D.	2.4E-04	2.3E-04	1.2E-04	3.1E-04
P_0077	α-Tocopherol	a-Tocopherol	14985	HMDB0001893	431.387	16.43	8.3E-03	1.0E-02	8.6E-05	1.1E-02	6.3E-03	8.9E-03	3.5E-03	8.6E-03	8.7E-03	6.3E-05	8.7E-05	1.1E-02	1.1E-02	7.0E-03	6.9E-03
P_0083	α-Tocopherol acetate	a-Tocopherol acetate	86472	HMDB0034227	490.425	16.69	5.6E-05	9.1E-05	1.1E-04	8.1E-05	9.8E-05	1.4E-04	5.5E-05	9.1E-05	8.5E-05	1.2E-04	1.1E-04	1.1E-04	5.1E-05	7.2E-05	9.2E-05
C_0003	β-Ala	b-Ala	239	HMDB0000056	90.055	6.82	3.8E-04	4.3E-04	N.D.	1.5E-03	5.7E-04	8.0E-04	N.D.	4.3E-04	N.D.	N.D.	N.D.	7.6E-04	6.0E-04	1.1E-03	9.7E-04
C_0094	β-Ala-Lys	b-Ala-Lys	440638		218.152	6.35	N.D.	N.D.	8.0E-04	6.7E-04	N.D.	N.D.	N.D.	N.D.	N.D.	1.2E-03	N.D.	N.D.	N.D.	N.D.	N.D.
P_0089	β-Cryptoxanthin-1	b-Cryptoxanthin	5281235	HMDB0033844	553.439	16.54	3.2E-04	3.4E-04	N.D.	1.2E-04	1.5E-04	4.2E-04	N.D.	2.4E-04	2.0E-04	N.D.	N.D.	1.1E-04	1.5E-04	1.6E-04	4.1E-04
P_0090	β-Cryptoxanthin-2	b-Cryptoxanthin	5281235	HMDB0033844	553.440	16.74	1.3E-03	1.7E-03	7.2E-04	6.2E-04	1.3E-03	1.2E-03	7.1E-04	1.2E-03	1.0E-03	N.D.	N.D.	5.9E-04	4.9E-04	4.2E-04	1.5E-03
P_0091	β-Cryptoxanthin-3	b-Cryptoxanthin	5281235	HMDB0033844	553.441	17.00	3.0E-04	3.6E-04	1.2E-04	N.D.	N.D.	1.7E-04	8.7E-05	2.2E-04	9.9E-05	N.D.	N.D.	1.2E-04	8.9E-05	1.2E-04	2.7E-04
P_0006	β-Estradiol<br>17α-Estradiol	b-Estradiol<br>17a-Estradiol	5757<br>68570	HMDB000015<br>1<br>HMDB000042	273.189	10.04	2.1E-04	3.7E-04	3.5E-04	2.9E-04	1.7E-04	3.1E-04	1.7E-04	1.8E-04	2.5E-04	2.6E-04	4.2E-04	2.4E-04	2.1E-04	2.2E-04	3.8E-04
C_0122	γ-Butyrobetaine	Actinine	134	HMDB0001161	146.118	7.50	7.8E-04	9.3E-04	N.D.	2.1E-03	7.5E-04	5.1E-04	6.1E-04	6.6E-04	6.4E-04	5.3E-04	N.D.	1.2E-03	7.2E-04	1.5E-03	1.0E-03
C_0095	γ-Glu-Cys	g-Glu-Cys	123938	HMDB0001049	251.071	11.95	N.D.	N.D.	N.D.	N.D.	N.D.	N.D.	N.D.	N.D.	N.D.	N.D.	N.D.	N.D.	N.D.	5.2E-04	N.D.
C_0096	γ-Glu-Gln	g-Glu-Gln	150914	HMDB0011738	276.122	11.97	N.D.	N.D.	N.D.	N.D.	N.D.	N.D.	N.D.	N.D.	N.D.	N.D.	N.D.	3.7E-04	N.D.	9.5E-04	N.D.
P_0070	γ-Tocopherol	g-Tocopherol	92729	HMDB0001492	417.370	16.23	1.2E-03	1.9E-03	9.4E-04	2.1E-03	2.3E-03	3.6E-03	2.2E-03	2.6E-03	1.2E-03	2.4E-04	1.2E-03	2.5E-03	2.9E-03	2.6E-03	4.3E-03

ID consists of analysis mode and number. 'C', 'A', 'P' and 'N' showed cation, anion, positive and negative modes, respectively.
N.D. (Not Detected): The target peak or metabolite was below detection limits.
N.A. (Not Available): The calculation was impossible because of insufficience of the data.
† Putative metabolites which were assigned on the basis of *m/z* and MT/RT. FA and AC means fatty acid and acylcarnitine with carbon chain (carbon number : unsaturated bond number).
¶ The ratio is of computed by using averaged detection values. The latter was used as denominator.
|| The p-value is computed by Welch's t-test. (*<0.05, **<0.01, ***<0.001)
Cholesterol: Peak intensity is saturated. Relative area was computed by using ^{13}C isotope ion peak.
The data are sorted by Compound name in ascending order.

Quantitative Estimation of Target Metabolites

ID	Metabolite	PubChem CID	HMDB ID	Concentration (nmol/g)														
				Control										RA				
				Con2	Con8 (D)	Con1 (D)	Con9	Con5	Con3	Con12	Con7	Con4	Con6	RA5 (D)	RA3	RA4	RA2	RA1
A_0006	2-Hydroxybutyric acid	440864	HMDB0000008	N.D.	N.D.	N.D.	54	24	N.D.	N.D.	24	N.D.	N.D.	N.D.	28	N.D.	N.D.	N.D.
A_0015	2-Oxoglutaric acid	51	HMDB0000208	N.D.	N.D.	N.D.	N.D.	N.D.	N.D.	N.D.	N.D.	N.D.	N.D.	N.D.	N.D.	N.D.	N.D.	N.D.
A_0008	2-Oxoisovaleric acid	49	HMDB0000019	N.D.	N.D.	N.D.	N.D.	N.D.	N.D.	N.D.	N.D.	N.D.	N.D.	N.D.	N.D.	N.D.	N.D.	N.D.
A_0028	2-Phosphoglyceric acid	439278	HMDB0003391	N.D.	N.D.	N.D.	N.D.	N.D.	N.D.	N.D.	N.D.	N.D.	N.D.	N.D.	N.D.	N.D.	N.D.	N.D.
A_0005	3-Hydroxybutyric acid	441	HMDB0000011	N.D.	N.D.	N.D.	N.D.	26	N.D.	N.D.	39	N.D.	N.D.	N.D.	N.D.	N.D.	45	60
A_0029	3-Phosphoglyceric acid	439183	HMDB0000807	N.D.	N.D.	N.D.	71	N.D.	105	N.D.	N.D.	N.D.	N.D.	N.D.	49	N.D.	N.D.	63
A_0048	6-Phosphogluconic acid	91493	HMDB0001316	N.D.	N.D.	N.D.	N.D.	N.D.	N.D.	N.D.	N.D.	N.D.	N.D.	N.D.	N.D.	N.D.	N.D.	N.D.
A_0067	Acetyl CoA_divalent	444493		N.D.	N.D.	N.D.	N.D.	N.D.	N.D.	N.D.	N.D.	N.D.	N.D.	N.D.	N.D.	N.D.	N.D.	N.D.
C_0028	Adenine	190	HMDB0000034	N.D.	N.D.	N.D.	6.6	N.D.	N.D.	N.D.	N.D.	N.D.	N.D.	N.D.	N.D.	N.D.	N.D.	3.9
C_0049	Adenosine	60961	HMDB0000050	22	9.1	40	85	62	107	12	13	9.8	18	36	23	58	121	89
A_0069	ADP	6022	HMDB0001341	294	268	61	40	57	28	337	469	475	183	44	261	N.D.	91	27
C_0004	Ala	602	HMDB0000161	606	743	60	1,042	534	520	257	437	375	161	108	1,014	584	516	646
A_0059	AMP	6083	HMDB0000045	72	89	37	138	244	126	96	152	146	81	45	315	91	161	132
C_0030	Anthranilic acid	227	HMDB0001123	N.D.	N.D.	N.D.	N.D.	N.D.	N.D.	N.D.	N.D.	N.D.	N.D.	N.D.	N.D.	N.D.	N.D.	N.D.
C_0040	Arg	6322	HMDB0000517	363	300	74	479	454	456	170	335	319	131	76	352	469	409	433
C_0025	Asn	236	HMDB0000168	83	88	N.D.	144	75	74	52	50	60	23	N.D.	136	84	84	87
C_0027	Asp	424	HMDB0000191	404	393	65	624	344	292	110	346	266	121	58	563	260	411	496
A_0078	ATP	5957	HMDB0000538	78	72	N.D.	35	N.D.	N.D.	97	99	139	33	N.D.	70	N.D.	82	N.D.
C_0015	Betaine	247	HMDB0000043	39	N.D.	N.D.	115	47	30	N.D.	109	36	N.D.	N.D.	27	N.D.	51	103
C_0018	Betaine aldehyde_+H_2O	249		N.D.	N.D.	N.D.	N.D.	N.D.	N.D.	N.D.	N.D.	2.5	N.D.	N.D.	N.D.	N.D.	N.D.	N.D.
A_0056	cAMP	6076	HMDB0000058	N.D.	N.D.	N.D.	N.D.	N.D.	N.D.	N.D.	N.D.	N.D.	N.D.	N.D.	N.D.	N.D.	N.D.	N.D.
C_0045	Carnosine	439224	HMDB0000033	N.D.	N.D.	N.D.	N.D.	N.D.	N.D.	N.D.	N.D.	N.D.	N.D.	N.D.	N.D.	N.D.	N.D.	N.D.
A_0065	CDP	6132	HMDB0001546	N.D.	N.D.	N.D.	N.D.	N.D.	N.D.	N.D.	N.D.	N.D.	N.D.	N.D.	N.D.	N.D.	N.D.	N.D.
A_0058	cGMP	24316	HMDB0001314	N.D.	N.D.	N.D.	N.D.	N.D.	N.D.	N.D.	N.D.	N.D.	N.D.	N.D.	N.D.	N.D.	N.D.	N.D.
C_0008	Choline	305	HMDB0000097	202	222	120	294	239	265	132	224	166	126	99	244	171	218	230
A_0024	*cis* -Aconitic acid	643757	HMDB0000072	N.D.	N.D.	N.D.	N.D.	N.D.	N.D.	N.D.	N.D.	N.D.	N.D.	N.D.	N.D.	N.D.	N.D.	N.D.
A_0032	Citric acid	311	HMDB0000094	25,813	30,305	32,239	N.D.	N.D.	205	16,654	15,175	16,068	22,644	32,325	8,294	N.D.	187	212
C_0041	Citrulline	9750	HMDB0000904	12	14	N.D.	14	N.D.	8.2	N.D.	7.3	N.D.	N.D.	N.D.	13	N.D.	12	11
A_0054	CMP	6131	HMDB0000095	N.D.	N.D.	N.D.	N.D.	N.D.	N.D.	N.D.	N.D.	N.D.	N.D.	N.D.	N.D.	N.D.	N.D.	N.D.
A_0062	CoA_divalent	87642		N.D.	N.D.	N.D.	N.D.	N.D.	N.D.	N.D.	N.D.	N.D.	N.D.	N.D.	N.D.	N.D.	N.D.	N.D.
C_0022	Creatine	586	HMDB0000064	436	389	53	824	387	321	255	508	476	99	72	631	231	556	539
C_0012	Creatinine	588	HMDB0000562	10	14	N.D.	16	11	13	9.0	8.6	6.7	68	4.9	29	20	126	35
A_0074	CTP	6176	HMDB0000082	N.D.	N.D.	N.D.	N.D.	N.D.	N.D.	N.D.	N.D.	N.D.	N.D.	N.D.	N.D.	N.D.	N.D.	N.D.
C_0019	Cys	594	HMDB0000574	58	72	10	123	16	22	46	50	23	41	13	40	32	34	57
C_0047	Cytidine	6175	HMDB0000089	N.D.	N.D.	N.D.	N.D.	N.D.	N.D.	N.D.	N.D.	N.D.	N.D.	N.D.	N.D.	N.D.	N.D.	N.D.
C_0010	Cytosine	597	HMDB0000630	N.D.	N.D.	N.D.	N.D.	N.D.	N.D.	N.D.	N.D.	N.D.	N.D.	N.D.	N.D.	N.D.	N.D.	N.D.
A_0077	dATP	15993	HMDB0001532	N.D.	N.D.	N.D.	N.D.	N.D.	N.D.	N.D.	N.D.	N.D.	N.D.	N.D.	N.D.	N.D.	N.D.	N.D.
A_0072	dCTP	65091	HMDB0000998	N.D.	N.D.	N.D.	N.D.	N.D.	N.D.	N.D.	N.D.	N.D.	N.D.	N.D.	N.D.	N.D.	N.D.	N.D.
A_0021	Dihydroxyacetone phosphate	668	HMDB0001473	N.D.	N.D.	N.D.	N.D.	N.D.	N.D.	N.D.	N.D.	N.D.	N.D.	N.D.	42	N.D.	88	N.D.
A_0064	dTDP	164628	HMDB0001274	N.D.	N.D.	N.D.	N.D.	N.D.	N.D.	N.D.	N.D.	N.D.	N.D.	N.D.	N.D.	N.D.	N.D.	N.D.
A_0053	dTMP	9700	HMDB0001227	N.D.	N.D.	N.D.	N.D.	N.D.	N.D.	N.D.	N.D.	N.D.	N.D.	N.D.	N.D.	N.D.	N.D.	N.D.
A_0073	dTTP	64968	HMDB0001342	N.D.	N.D.	N.D.	N.D.	N.D.	N.D.	N.D.	N.D.	N.D.	N.D.	N.D.	N.D.	N.D.	N.D.	N.D.
A_0034	Erythrose 4-phosphate	122357	HMDB0001321	N.D.	N.D.	N.D.	N.D.	N.D.	N.D.	N.D.	N.D.	N.D.	N.D.	N.D.	N.D.	N.D.	N.D.	N.D.
A_0057	Fructose 1,6-diphosphate	172313	HMDB0001058	53	39	N.D.	N.D.	N.D.	N.D.	N.D.	41	49	N.D.	N.D.	63	N.D.	N.D.	N.D.
A_0043	Fructose 6-phosphate	603	HMDB0000124	N.D.	N.D.	N.D.	N.D.	N.D.	N.D.	N.D.	N.D.	N.D.	N.D.	N.D.	N.D.	N.D.	N.D.	24
A_0007	Fumaric acid	444972	HMDB0000134	N.D.	N.D.	N.D.	N.D.	N.D.	N.D.	N.D.	N.D.	N.D.	N.D.	N.D.	N.D.	N.D.	N.D.	N.D.
C_0006	GABA	119	HMDB0000112	4.4	12	N.D.	16	6.2	18	N.D.	14	N.D.	N.D.	N.D.	8.1	N.D.	5.6	14
A_0070	GDP	8977	HMDB0001201	48	56	36	N.D.	N.D.	N.D.	60	90	97	55	31	70	N.D.	N.D.	N.D.
C_0033	Gln	738	HMDB0000641	968	1,119	63	1,705	859	700	259	634	552	214	118	1,787	900	814	926
C_0035	Glu	611	HMDB0000148	602	743	108	1,436	646	590	291	530	491	341	118	960	563	961	858

A_0033	Gluconic acid	10690	HMDB0000625	N.D.	N.D.	N.D.	N.D.	N.D.	N.D.	N.D.	N.D.	36	N.D.	N.D.	N.D.	N.D.	63	N.D.
A_0045	Glucose 1-phosphate	65533	HMDB0001586	N.D.	24	N.D.	45	N.D.	N.D.	N.D.	36	N.D.	N.D.	N.D.	36	N.D.	42	N.D.
A_0044	Glucose 6-phosphate	5958	HMDB0001401	32	N.D.	N.D.	106	N.D.	26	N.D.	N.D.	N.D.	N.D.	N.D.	20	N.D.	N.D.	64
C_0053	Glutathione (GSH)	124886	HMDB0000125	304	289	35	295	127	154	230	309	302	152	52	214	93	200	306
C_0052	Glutathione (GSSG)_divalent	65359		67	76	8.0	64	54	66	34	81	92	17	8.9	126	50	82	63
C_0001	Gly	750	HMDB0000123	538	599	97	1,188	820	774	262	436	390	182	103	845	720	866	921
A_0020	Glyceraldehyde 3-phosphate	729	HMDB0001112	N.D.	N.D.	N.D.	N.D.	N.D.	N.D.	N.D.	N.D.	N.D.	N.D.	N.D.	N.D.	N.D.	N.D.	N.D.
A_0022	Glycerol 3-phosphate	439162	HMDB0000126	42	55	N.D.	41	32	45	39	N.D.	41	N.D.	N.D.	45	33	45	N.D.
A_0002	Glycolic acid	757	HMDB0000115	N.D.	N.D.	N.D.	N.D.	N.D.	N.D.	N.D.	N.D.	N.D.	N.D.	N.D.	N.D.	N.D.	N.D.	N.D.
A_0001	Glyoxylic acid	760	HMDB0000119	N.D.	N.D.	N.D.	N.D.	N.D.	N.D.	N.D.	N.D.	N.D.	N.D.	N.D.	N.D.	N.D.	N.D.	N.D.
A_0061	GMP	6804	HMDB0001397	N.D.	N.D.	N.D.	73	67	40	N.D.	N.D.	N.D.	N.D.	N.D.	34	31	45	52
A_0079	GTP	6830	HMDB0001273	N.D.	N.D.	N.D.	N.D.	N.D.	N.D.	N.D.	N.D.	N.D.	N.D.	N.D.	N.D.	N.D.	N.D.	N.D.
C_0037	Guanine	764	HMDB0000132	N.D.	N.D.	N.D.	N.D.	N.D.	N.D.	6.7	N.D.	N.D.	N.D.	N.D.	N.D.	N.D.	N.D.	6.0
C_0051	Guanosine	6802	HMDB0000133	23	20	N.D.	57	19	20	6.1	7.1	6.1	10	7.1	12	19	24	34
C_0038	His	773	HMDB0000177	148	138	27	201	214	234	103	130	112	60	104	176	230	173	213
C_0016	Homoserine	12647	HMDB0000719	N.D.	N.D.	N.D.	N.D.	N.D.	N.D.	N.D.	N.D.	N.D.	N.D.	N.D.	N.D.	N.D.	N.D.	N.D.
C_0021	Hydroxyproline	5810	HMDB0000725	N.D.	12	N.D.	18	N.D.	N.D.	N.D.	N.D.	N.D.	N.D.	N.D.	20	7.6	30	12
C_0029	Hypoxanthine	790	HMDB0000157	62	114	11	422	67	74	72	48	23	33	15	82	99	200	112
C_0023	Ile	791	HMDB0000172	424	365	95	521	633	615	209	438	350	194	101	382	668	463	628
A_0060	IMP	8582	HMDB0000175	N.D.	N.D.	N.D.	N.D.	49	N.D.	N.D.	N.D.	61	N.D.	N.D.	39	N.D.	41	N.D.
C_0050	Inosine	6021	HMDB0000195	142	123	88	532	125	94	50	66	44	87	96	82	109	432	210
A_0030	Isocitric acid	1198	HMDB0000193	N.D.	N.D.	N.D.	N.D.	N.D.	N.D.	N.D.	N.D.	N.D.	N.D.	N.D.	N.D.	N.D.	N.D.	N.D.
A_0004	Lactic acid	612	HMDB0000190	3,899	5,416	375	11,016	3,966	4,350	1,954	3,425	2,111	1,199	771	5,644	3,670	6,660	5,201
C_0024	Leu	857	HMDB0000687	617	522	169	615	668	690	341	599	457	301	170	462	717	516	714
C_0034	Lys	866	HMDB0000182	668	579	148	950	887	873	351	672	640	235	156	779	1,003	825	877
A_0011	Malic acid	525	HMDB0000156	109	N.D.	N.D.	N.D.	112	94	N.D.	91	107	N.D.	N.D.	N.D.	N.D.	N.D.	N.D.
A_0068	Malonyl CoA_divalent	644066		N.D.	N.D.	N.D.	N.D.	N.D.	N.D.	N.D.	N.D.	N.D.	N.D.	N.D.	N.D.	N.D.	N.D.	N.D.
C_0036	Met	876	HMDB0000696	74	58	16	74	91	89	41	69	54	33	15	53	103	84	80
C_0007	*N*,*N*-Dimethylglycine	673	HMDB0000092	N.D.	N.D.	N.D.	N.D.	N.D.	N.D.	N.D.	N.D.	N.D.	N.D.	N.D.	N.D.	N.D.	N.D.	N.D.
A_0083	NAD^+	5893	HMDB0000902	38	43	N.D.	28	43	N.D.	50	71	60	N.D.	N.D.	64	N.D.	39	N.D.
A_0084	$NADP^+$	5886	HMDB0000217	N.D.	N.D.	N.D.	N.D.	N.D.	N.D.	N.D.	N.D.	N.D.	N.D.	N.D.	N.D.	N.D.	N.D.	N.D.
C_0026	Ornithine	389	HMDB0000214	56	28	5.7	58	17	16	15	22	25	7.4	6.2	43	44	57	21
C_0039	Phe	994	HMDB0000159	278	243	84	328	353	348	197	289	246	153	87	241	371	272	358
A_0018	Phosphoenolpyruvic acid	1005	HMDB0000263	N.D.	N.D.	N.D.	N.D.	N.D.	N.D.	N.D.	N.D.	N.D.	N.D.	N.D.	N.D.	N.D.	N.D.	N.D.
C_0013	Pro	614	HMDB0000162	219	181	27	301	149	151	101	137	140	54	42	289	287	339	173
A_0063	PRPP	7339	HMDB0000280	N.D.	N.D.	N.D.	N.D.	N.D.	N.D.	N.D.	N.D.	N.D.	N.D.	N.D.	N.D.	N.D.	N.D.	N.D.
C_0002	Putrescine	1045	HMDB0001414	5.5	6.4	N.D.	24	5.4	7.7	21	21	18	3.4	N.D.	13	8.6	10	9.2
A_0003	Pyruvic acid	1060	HMDB0000243	N.D.	N.D.	N.D.	N.D.	N.D.	N.D.	N.D.	N.D.	N.D.	N.D.	N.D.	N.D.	N.D.	N.D.	N.D.
A_0039	Ribose 5-phosphate	439167	HMDB0001548	N.D.	N.D.	N.D.	N.D.	N.D.	N.D.	N.D.	N.D.	N.D.	N.D.	N.D.	N.D.	N.D.	N.D.	N.D.
A_0038	Ribulose 5-phosphate	439184	HMDB0000618	N.D.	N.D.	N.D.	N.D.	N.D.	N.D.	N.D.	N.D.	N.D.	N.D.	N.D.	N.D.	N.D.	N.D.	N.D.
C_0054	*S*-Adenosylmethionine	34755	HMDB0001185	12	13	N.D.	12	6.1	8.3	8.9	14	8.5	6.4	N.D.	9.6	4.7	9.9	12
C_0005	Sarcosine	1088	HMDB0000271	N.D.	N.D.	N.D.	7.4	N.D.	N.D.	N.D.	N.D.	N.D.	N.D.	N.D.	N.D.	N.D.	4.2	N.D.
A_0049	Sedoheptulose 7-phosphate	165007	HMDB0001068	N.D.	N.D.	N.D.	30	N.D.	N.D.	N.D.	N.D.	N.D.	N.D.	N.D.	N.D.	N.D.	N.D.	N.D.
C_0009	Ser	617	HMDB0000187	386	343	86	485	452	457	168	391	319	117	78	428	425	392	510
C_0032	Spermidine	1102	HMDB0001257	226	206	37	13	9.7	8.6	125	241	195	89	36	182	17	13	14
C_0043	Spermine	1103	HMDB0001256	98	125	28	N.D.	N.D.	N.D.	46	90	59	59	24	58	N.D.	N.D.	N.D.
A_0009	Succinic acid	1110	HMDB0000254	N.D.	N.D.	N.D.	N.D.	N.D.	N.D.	N.D.	N.D.	N.D.	N.D.	N.D.	N.D.	N.D.	N.D.	N.D.
C_0017	Thr	6288	HMDB0000167	720	626	152	771	802	814	283	710	580	219	131	965	797	699	794
C_0046	Thymidine	5789	HMDB0000273	N.D.	N.D.	N.D.	N.D.	N.D.	N.D.	N.D.	N.D.	N.D.	N.D.	N.D.	N.D.	N.D.	N.D.	N.D.
C_0020	Thymine	1135	HMDB0000262	N.D.	N.D.	N.D.	N.D.	N.D.	N.D.	N.D.	N.D.	N.D.	N.D.	N.D.	N.D.	N.D.	N.D.	N.D.
C_0044	Trp	1148	HMDB0000929	64	52	34	69	74	66	57	69	54	40	32	53	74	53	66
C_0042	Tyr	1153	HMDB0000158	231	199	71	277	299	294	150	231	199	116	74	193	316	214	290
C_0031	Tyramine	5610	HMDB0000306	N.D.	N.D.	N.D.	N.D.	N.D.	N.D.	N.D.	N.D.	N.D.	N.D.	N.D.	N.D.	N.D.	N.D.	N.D.
A_0066	UDP	6031	HMDB0000295	47	50	N.D.	N.D.	N.D.	N.D.	59	104	128	N.D.	N.D.	86	N.D.	N.D.	N.D.
A_0055	UMP	6030	HMDB0000288	N.D.	N.D.	N.D.	56	73	46	N.D.	30	26	N.D.	N.D.	59	31	52	30
C_0011	Uracil	1174	HMDB0000300	N.D.	N.D.	N.D.	N.D.	N.D.	N.D.	N.D.	N.D.	N.D.	N.D.	N.D.	N.D.	N.D.	N.D.	N.D.
C_0048	Uridine	6029	HMDB0000296	N.D.	N.D.	N.D.	86	48	46	N.D.	N.D.	N.D.	N.D.	N.D.	N.D.	N.D.	65	63

A_0075	UTP	6133	HMDB0000285	N.D.	N.D.	N.D.	N.D.	N.D.	N.D.	N.D.	N.D.	44	N.D.	N.D.	N.D.	N.D.	N.D.	N.D.
C_0014	Val	1182	HMDB0000883	562	452	120	651	702	697	299	533	450	234	127	503	803	581	729
C_0003	β-Ala	239	HMDB0000056	6.3	7.1	N.D.	25	9.5	13	N.D.	7.1	N.D.	N.D.	N.D.	13	10	19	16

ID consists of analysis mode and number. 'C' and 'A' showed cation and anion modes, respectively.
N.D. (Not Detected): The target peak or metabolite was below detection limits.
N.A. (Not Available): The calculation was impossible because of insufficience of the data.
¶ The ratio is of computed by using averaged detection values. The latter was used as denominator.
|| The p-value is computed by Welch's t-test. (*<0.05, **<0.01, ***<0.001)
The data are sorted by Compound name in ascending order.

	baseMean	log2FoldChange	lfcSE	stat	pvalue	padj	weight
LINC01060	137.5329587	5.760481573	0.700711401	8.220904595	2.02E-16	2.66E-12	3.49149467
RPS3AP4	1.661017481	-18.39248115	2.23533094	-8.228079707	1.90E-16	1.65E-10	0.026403131
GPR17	2578.886915	1.630646347	0.222064155	7.343131735	2.09E-13	2.40E-08	0.133148884
AJ011931.1	65.9889914	1.710503155	0.292941451	5.839061527	5.25E-09	1.47E-05	3.281275518
AL049873.2	1.017841295	-18.3270299	2.770527425	-6.614996745	3.72E-11	1.47E-05	0.023940788
PHEX	424.0992767	-1.337036107	0.251198247	-5.322633117	1.02E-07	0.000347681	2.250665407
NBPF2P	315.7173395	-4.901745348	0.926170027	-5.292489723	1.21E-07	0.000351586	2.250665407
AC097652.1	17.43067512	-5.610804285	1.079271846	-5.198694201	2.01E-07	0.00042507	2.709268384
STK19B	60.92454468	1.804914704	0.354694684	5.088643241	3.61E-07	0.000550378	3.342205961
C2CD4A	324.063188	1.470564692	0.294509731	4.993263507	5.94E-07	0.000728214	3.742473548
DOCK9-AS2	176.1832186	0.841403867	0.168260584	5.000599933	5.72E-07	0.000959476	2.485852589
DOK7	25.10381898	2.383041941	0.487548309	4.887806803	1.02E-06	0.001344346	2.901552746
EMBP1	824.088141	-1.335835947	0.273861661	-4.877776393	1.07E-06	0.001524066	2.485852589
DPPA4	44.92461378	-2.71106033	0.565752685	-4.791953095	1.65E-06	0.00162042	3.342205961
GRIN3B	63.77552059	1.799231276	0.38029928	4.731093033	2.23E-06	0.002044849	3.342205961
GTSF1	191.8773863	2.433459894	0.496666445	4.89958587	9.60E-07	0.002480789	1.110725453
HOXC-AS3	33.15312858	2.257249368	0.478440531	4.717930906	2.38E-06	0.002618956	2.45660337
MST1R	239.0355028	1.014245614	0.211695891	4.791050079	1.66E-06	0.002735636	1.546724962
LINC01535	303.8054532	1.027556503	0.215346751	4.771636891	1.83E-06	0.002854459	1.546724962
FOXP4-AS1	34.57621694	1.771118564	0.385847638	4.590201905	4.43E-06	0.003141489	3.235404965
AC073488.1	11.43630688	5.177409483	1.124551764	4.603976133	4.14E-06	0.003344434	2.709268384
AC006116.1	92.57751579	1.179690146	0.251487096	4.690857556	2.72E-06	0.003375454	1.555807481
AC133919.2	72.3974864	5.032148215	1.10944377	4.535739754	5.74E-06	0.003375454	3.49149467
GXYLT1P6	45.77260779	-3.280914873	0.724965013	-4.525618218	6.02E-06	0.003375454	3.412409045
PAX3	904.760841	1.044197712	0.2294142	4.551582733	5.32E-06	0.003816447	2.561767538
CUBNP3	29.46242104	-3.387730983	0.751832087	-4.505967546	6.61E-06	0.003830771	3.045243497
SNX15	548.5219077	0.641759487	0.144903813	4.428865428	9.47E-06	0.00430363	3.742473548
ATP2C2-AS1	109.3349299	1.103624679	0.24022581	4.594113669	4.35E-06	0.004363393	1.632936437
AC022730.4	36.27191703	5.835965947	1.33058178	4.386025749	1.15E-05	0.005010288	3.411953508
IGHGP	98.11294764	2.888806314	0.656077438	4.403148389	1.07E-05	0.005010288	3.281275518
RTP5	17.09019539	2.887611309	0.650698701	4.437708733	9.09E-06	0.005010288	2.709268384
IGHG2	559.2880422	2.20057298	0.483788728	4.54862392	5.40E-06	0.005827769	1.329209903
CLUL1	201.1366329	-4.160896527	0.924399747	-4.501187437	6.76E-06	0.006077538	1.546724962
AC005520.2	257.4686014	1.73130389	0.382653332	4.52447096	6.05E-06	0.006150181	1.329209903
AC025175.1	115.5874972	0.970191616	0.223604343	4.338876437	1.43E-05	0.006817185	2.75536286
AC018665.1	494.7245589	0.718518912	0.165143712	4.350870541	1.36E-05	0.006955769	2.485852589
SFRP2	77.27033039	-2.423774496	0.571859584	-4.238408454	2.25E-05	0.007999282	3.49149467
AC067930.6	38.93298258	1.562826909	0.371994512	4.201209583	2.65E-05	0.009745558	3.245778424
MMRN1	1010.013704	-2.038799918	0.458402311	-4.447621378	8.68E-06	0.009745558	1.048696725
PGAM4P2	2.444573179	-18.89838091	4.108440155	-4.599891977	4.23E-06	0.010233776	0.474041304
AC015920.1	52.60327139	-1.707984707	0.42685046	-4.001365508	6.30E-05	0.011466818	6.149381994
RRH	92.49179605	-1.223988867	0.298519361	-4.100199273	4.13E-05	0.012922386	3.49149467
C20orf203	266.0362598	0.796124707	0.191792305	4.150973141	3.31E-05	0.01389486	2.485852589
F5	1486.403356	-2.988420736	0.640900717	-4.66284505	3.12E-06	0.01389486	0.237407751
KCNJ8	420.8861621	0.916429636	0.22776898	4.023505031	5.73E-05	0.015368598	3.742473548
SERPINB9P1	431.9466655	-1.541857797	0.374454613	-4.117609297	3.83E-05	0.015368598	2.485852589
G6PC2	470.507428	-1.594692529	0.390077199	-4.088145968	4.35E-05	0.015870807	2.485852589
IGLC2	598.3879186	2.111117826	0.528512162	3.994454582	6.48E-05	0.015870807	3.742473548
LINC01291	508.077139	-6.889532324	1.63733538	-4.207770998	2.58E-05	0.015870807	1.546724962
NPM1P26	41.10727146	-1.544418886	0.383163222	-4.030707533	5.56E-05	0.015870807	3.235404965
SMCP	21.46901684	-4.208329568	1.050386312	-4.006458881	6.16E-05	0.015870807	3.495696059
VDAC3P1	42.3177956	1.182078681	0.29405901	4.019868933	5.82E-05	0.015888693	3.235404965
AC108865.1	26.58330937	2.58460907	0.646315225	3.998991467	6.36E-05	0.017029842	3.235404965
TRIM17	194.615681	0.840506669	0.20750338	4.050568561	5.11E-05	0.017472884	2.485852589
ASB4	63.25062179	-1.231279771	0.32201118	-3.823717458	0.000131455	0.017842303	6.149381994
CAPNS2	19.49438331	-1.836991001	0.465240438	-3.948476641	7.87E-05	0.018443654	3.495696059
AL359979.2	19.31833421	-2.554290828	0.619842063	-4.120873652	3.77E-05	0.018452154	1.625478358
AL445483.1	23.66855851	-2.384129011	0.602283976	-3.958479897	7.54E-05	0.018452154	3.235404965
HTR3B	154.3612128	-2.371252715	0.602833057	-3.933514741	8.37E-05	0.018612907	3.49149467
LINC01497	27.64461311	2.524208421	0.63513323	3.974297519	7.06E-05	0.018612907	2.901552746
RAET1E-AS1	17.12161099	-2.961653027	0.740967445	-3.997008299	6.41E-05	0.019651154	2.45660337
NIFKP6	0.583787518	-18.01679631	4.117397053	-4.375773353	1.21E-05	0.020367742	0.432893501
VN1R81P	236.870545	-0.704518882	0.172146912	-4.092544403	4.27E-05	0.020367742	1.546724962
AP001596.1	81.8096637	0.828605042	0.212522356	3.898907656	9.66E-05	0.02060103	3.281275518

LINC02550	72.62469851	1.232563061	0.316180452	3.89828989	9.69E-05	0.02060103	3.281275518
NDP	1091.250555	0.590112109	0.149128677	3.957066627	7.59E-05	0.02060103	2.561767538
SEC62	43484.94683	-0.445375145	0.103440814	-4.305603625	1.67E-05	0.021164539	0.53911443
AL589880.1	307.3800414	-2.867022619	0.725948095	-3.949349324	7.84E-05	0.021281455	2.485852589
BTBD9-AS1	5.42475761	-4.234395416	0.989304043	-4.280175996	1.87E-05	0.021526129	0.573000757
IGHG3	270.8919096	2.098930432	0.533685314	3.932898988	8.39E-05	0.021526129	2.485852589
IL20RB	417.3287902	-1.291370471	0.328242189	-3.934200155	8.35E-05	0.021526129	2.485852589
MAP1LC3B2	231.324067	1.413121546	0.349198467	4.046757593	5.19E-05	0.021526129	1.546724962
SNORD3C	112.8038911	5.63899273	1.466363732	3.845562058	0.000120276	0.023050714	3.281275518
GOLGA2P8	35.47292922	1.469367774	0.380694675	3.85970141	0.000113526	0.02312649	3.045243497
ZNF812P	617.1211913	-1.306082932	0.317849471	-4.109124134	3.97E-05	0.023180702	1.048696725
TSHB	31.08630116	1.675854211	0.442650028	3.785957543	0.000153118	0.027106815	3.411953508
CRYGN	339.5522025	0.716640409	0.19083309	3.755325704	0.000173116	0.027577678	3.742473548
AC083843.1	20.74717419	-2.652410855	0.699820501	-3.790130258	0.000150568	0.030515964	2.901552746
OR7E99P	26.24602908	-3.008410448	0.800277887	-3.759207265	0.000170453	0.030515964	3.245778424
AP003355.2	142.7223582	-1.272214935	0.324170413	-3.924525136	8.69E-05	0.030537599	1.632936437
SNORD3B-2	14.69875422	2.539658543	0.66242229	3.833896565	0.000126129	0.030800501	2.320824397
IGLV1-40	94.25275898	3.254732639	0.831182626	3.915785214	9.01E-05	0.032378986	1.555807481
RPL12P37	40.27811323	1.580613424	0.424971559	3.719339312	0.000199745	0.032378986	3.411953508
CETP	154.3411891	1.053438627	0.270459827	3.894991127	9.82E-05	0.032865682	1.632936437
FRG1JP	364.7260317	-0.826203814	0.210135154	-3.931773426	8.43E-05	0.033946106	1.329209903
OXCT2P1	42.17918525	-1.956564495	0.529033062	-3.698378487	0.000216981	0.033946106	3.411953508
AL662844.3	28.10556709	1.463450305	0.391760792	3.735571131	0.00018729	0.034059123	2.901552746
AC048341.3	261.4811806	1.13491754	0.286908735	3.955674406	7.63E-05	0.034572776	1.110725453
NTRK1	13716.92369	0.943148433	0.226518414	4.163672238	3.13E-05	0.034572776	0.463299071
PLA2G7	379.3511187	-0.626949634	0.166584759	-3.76354739	0.00016752	0.034572776	2.485852589
RGS13	35.52058114	-3.189415658	0.868152771	-3.673795401	0.000238974	0.034572776	3.411953508
TBC1D3E	82.14359469	3.672452636	0.996221759	3.686380671	0.000227466	0.034572776	3.281275518
VSX1	28.60175022	-1.640202379	0.444298714	-3.691665826	0.00022279	0.034572776	3.235404965
AC073109.1	5.478174624	4.003698766	0.960000183	4.170518753	3.04E-05	0.034817193	0.424583088
SNX18P9	17.38054687	-3.43450605	0.912463161	-3.763994206	0.000167221	0.034817193	2.320824397
SLC6A9	364.3258087	1.128934347	0.292949219	3.853686146	0.000116353	0.035971761	1.546724962
MIR4645	17.1218495	-2.372751378	0.636320364	-3.728862867	0.000192346	0.036154061	2.507536703
PROM1	73.95299125	1.600144679	0.439238207	3.64299975	0.000269479	0.036154061	3.49149467
CYP4A22-AS1	113.8920086	-0.758768877	0.203633168	-3.726155636	0.000194422	0.036266459	2.485852589
SNORD11	17.80461647	-2.320698668	0.624599483	-3.715498863	0.000202803	0.037127603	2.507536703
AL162725.2	69.28420814	-1.556389774	0.430019418	-3.619347657	0.000295347	0.038447553	3.49149467
CROCC2	30.12915948	1.860540539	0.511942391	3.634277158	0.000278761	0.040406033	3.045243497
IGKV1-33	69.6995709	4.128353852	1.134607182	3.638575463	0.00027415	0.040406033	3.045243497
KCNJ1	75.76688136	-1.109852515	0.308238051	-3.600634351	0.000317442	0.040406033	3.49149467
AL357054.4	179.6609733	-1.010929036	0.281460014	-3.591732348	0.000328487	0.041132691	3.49149467
RPL36AP29	12.2727481	-5.107783371	1.324415292	-3.856632737	0.00011496	0.041797651	1.191124834
FLT3	1012.519201	-0.832665247	0.214626756	-3.879596669	0.00010463	0.042408336	1.048696725
MYT1L-AS1	15.95170041	-2.823281159	0.76662512	-3.682740216	0.00023074	0.042408336	2.320824397
AC090616.6	186.088413	0.894253691	0.244405855	3.658888166	0.000253312	0.04291638	2.485852589
AC023158.1	53.37934286	1.144699765	0.32055452	3.570998667	0.000355623	0.042965138	3.412409045
CU638689.5	9.926235169	-2.869415667	0.777542981	-3.690362767	0.000223934	0.042965138	2.136270133
SPATC1L	548.5491848	0.915623004	0.250724386	3.651910444	0.000260297	0.042965138	2.485852589
LINC01876	76.19758824	1.188385039	0.332732537	3.571592513	0.000354817	0.043128309	3.342205961
GLUD1P8	46.442793	-1.489036554	0.414900268	-3.588902369	0.000332073	0.043712077	3.045243497
RNU2-11P	50.02897538	-1.078269352	0.302589725	-3.563469817	0.000365985	0.043712077	3.342205961
FLJ42393	51.5906365	-2.522987598	0.711410844	-3.546456481	0.000390449	0.045280889	3.412409045
ADAMTS7P1	30.71185534	2.733939747	0.766986614	3.564520812	0.000364522	0.046966127	3.045243497
MEGF6	327.9600206	1.527720033	0.422732881	3.613913423	0.00030161	0.047201689	2.485852589
AC016722.1	8.075151571	2.425881801	0.669127841	3.625438446	0.000288472	0.049223562	2.243308896
AL133351.1	16.46779489	2.461032548	0.682106488	3.607988772	0.00030858	0.049223562	2.320824397
EFNB3	192.7467981	1.111347181	0.292702835	3.796844615	0.00014655	0.049223562	1.110725453
EVX1-AS	0.517667098	-16.60386714	4.123131204	-4.027004312	5.65E-05	0.049223562	0.432893501
TP63	521.1796449	-1.247148452	0.347073309	-3.593328617	0.00032648	0.049223562	2.485852589
YWHAQP5	10.34877085	-3.959591209	1.089101111	-3.635650693	0.00027728	0.049223562	2.136270133
DLX5	38.37113398	1.505196741	0.428226874	3.514951608	0.000439834	0.049389536	3.245778424
HAP1	527.4813507	1.033159231	0.275856017	3.745284373	0.00018019	0.049389536	1.329209903
AC010105.1	0.401374921	-17.14531442	4.132169546	-4.149228201	3.34E-05	0.049689363	0.242675482
AC103591.3	114.4216355	-0.864721853	0.241597949	-3.579177124	0.000344678	0.049727592	2.485852589

	Con1	Con2	Con3	Con4	Con5	Con6	Con7	Con8	Con9	RA1	RA1	RA2	RA2	RA3	RA3	RA4	RA4	RA5	RA5
LINC01060	196.986	3.001171	1.998267	4.157171	3.322455	147.3034	0.84968	1.852054	2.334575	109.5197	46.98544	376.312	352.6329	290.3847	215.1613	222.602	215.539	237.5448	184.6394
RPS3AP4	2.882723	0	1.998267	0	4.42994	0	0	3.704108	14.00745	0	0	0	0	0	0	0	0	3.640534	0.896308
GPR17	2908.667	1738.679	1572.636	717.112	1404.291	1819.026	2049.429	1314.032	2258.702	5304.021	8023.667	2018.652	2545.537	2451.517	2401.004	2285.38	3349.066	2187.051	2650.383
AJ011931.1	27.86632	70.02733	45.96015	27.02161	15.50479	23.97963	34.83689	34.263	49.02608	97.99132	178.3037	99.36845	66.82548	94.93347	85.08651	56.06272	63.63533	75.54107	107.557
AL049873.2	4.804538	0	0	2.078586	0	0	0	0.926027	7.003726	0	0	0	0	0	0	2.473355	2.052753	0	0
PHEX	291.155	261.1019	648.4377	670.3438	603.5793	435.0589	312.6823	661.1833	298.8257	145.0656	161.4372	368.0313	410.2057	274.5625	309.0498	507.0379	513.1881	546.08	640.8602
NBPF2P	197.947	441.1722	296.7427	242.1552	537.1302	730.8077	404.4478	152.7945	292.9892	0	0	334.9085	335.1555	551.9171	529.1012	140.9813	179.6158	279.411	351.3527
AC097652.1	0	0	5.994802	30.13949	8.859879	7.993209	0	23.15068	191.4352	0	0	4.600391	7.196591	9.307203	0.978006	8.244518	13.34289	14.56213	5.377848
STK19B	32.67086	37.01445	29.97401	16.62868	31.00958	39.96605	15.29424	51.85751	60.69896	151.7905	246.9747	34.04289	51.40422	73.5269	87.04252	42.8715	70.81996	40.956	43.02278
C2CD4A	179.6897	208.0812	228.8016	122.6365	125.1458	89.06719	144.4456	127.7917	165.7549	488.0352	516.8398	300.8656	302.2568	313.6527	277.7537	283.6114	363.3372	972.9326	946.5012
DOCK9-AS2	160.4716	185.0722	184.8397	150.6975	145.0805	116.4725	135.0992	111.1232	161.0857	275.7207	379.4978	172.0546	260.1053	185.2133	181.9091	136.859	127.2707	128.3288	150.5797
DOK7	17.29634	9.003514	18.98354	3.117878	5.537425	28.54718	15.29424	8.334243	25.68033	39.38867	146.9801	20.24172	18.50552	21.40657	9.780058	16.48904	24.63303	16.3824	31.37078
EMBP1	548.6782	947.3697	801.3052	1323.02	559.2799	897.5232	1132.624	1171.424	678.1942	369.8692	162.6419	705.7	764.8948	500.7275	484.1129	1356.223	1149.541	1063.036	1041.51
DPPA4	21.13997	55.02147	2.997401	130.9509	106.3186	42.24982	90.06611	32.41095	39.68778	4.803496	0	47.84407	62.71315	39.09025	33.2522	42.04704	36.94955	39.13574	26.88924
GRIN3B	24.02269	50.01952	41.96361	12.47151	19.93473	30.83095	16.9936	67.59997	80.54285	90.30573	301.1887	88.32751	80.19058	48.39746	42.05425	36.27588	30.79129	68.26001	81.56402
GTSF1	39.39721	84.0328	68.94022	141.3438	79.73891	83.35775	135.0992	68.526	23.34575	677.293	1119.217	128.811	71.96591	153.5689	186.7991	92.3386	79.03097	273.04	139.824
HOXC-AS3	13.45271	12.00469	28.97488	4.157171	8.859879	26.2634	28.03945	14.81643	26.84762	58.60265	87.94711	50.6043	51.40422	43.74385	69.43841	9.893422	7.184634	48.23707	39.43755
MST1R	252.7187	183.0715	263.7713	133.0295	272.4413	158.7223	130.8508	170.389	235.7921	467.8605	521.6589	292.5849	305.3411	317.3756	323.7199	92.3386	136.508	135.6099	147.8908
LINC01535	267.1323	200.0781	132.8848	175.6405	83.06137	291.1812	390.0032	249.1013	365.3611	320.8736	409.6167	472.9202	453.3852	378.8032	384.3563	247.3355	308.9393	286.692	354.9379
FOXP4-AS1	17.29634	22.00859	19.98267	11.43222	19.93473	30.83095	32.28785	25.00273	19.84389	84.54153	127.704	14.72125	28.78636	44.67457	43.03226	15.66458	28.73854	36.40534	34.0597
AC073488.1	2.882723	1.00039	0	0	0	0	2.549041	0	14.00745	56.68126	61.4425	11.04094	3.084253	34.43665	29.34017	0.824452	0	0	0
AC006116.1	107.6216	73.0285	117.8978	58.2004	54.26676	79.93209	96.86355	50.00546	38.5205	232.4892	156.6181	112.2495	77.10633	62.35826	47.92229	94.81196	99.5585	94.65388	104.868
AC133919.2	0	42.0164	1.998267	1.039293	2.21497	0	0	0.926027	0	276.6814	787.9097	3.680313	6.168506	3.722881	0.978006	7.420066	5.131881	113.7667	121.8979
GXYLT1P6	10.56998	39.01523	147.8718	134.0688	95.2437	86.78341	38.23561	19.44657	93.38302	8.646293	7.228529	7.360626	7.196591	22.33729	12.71408	7.420066	2.052753	68.26001	61.84525
PAX3	997.422	602.235	891.2272	443.778	784.0993	980.8809	837.7847	755.6381	818.2687	1227.774	2181.811	980.8034	1177.157	1138.271	1266.518	371.0033	385.9175	709.9041	639.9639
CUBNP3	8.648168	41.01601	22.98007	6.235757	45.40688	20.55397	30.58849	12.03835	298.8257	0	10.84279	6.440548	8.224675	5.584322	2.934017	9.893422	6.158258	7.281067	16.13354
SNX15	471.8056	459.1792	532.5382	444.8173	448.5314	504.7141	479.2197	570.4327	515.9412	906.9001	1114.398	532.7253	516.0984	476.5288	572.1334	428.715	526.531	485.1011	435.6057
ATP2C2-AS1	97.05166	98.03826	65.94282	93.53635	85.27634	92.49285	84.11834	64.82189	37.35321	128.7337	242.1557	137.0917	134.6791	98.65635	102.6906	103.8809	137.5344	113.7667	159.5428
AC022730.4	0.960908	0	1.998267	0	31.00958	13.70264	30.58849	0	0	229.6071	238.5415	24.84211	10.28084	51.18962	30.31818	15.66458	4.105505	5.4608	0.896308
IGHGP	19.21815	13.00508	8.992203	15.58939	16.61227	12.56076	6.797442	158.3506	47.8588	42.27077	798.7525	97.52829	68.88165	164.7375	158.4369	112.1254	48.23968	45.50667	28.68185
RTP5	1.921815	8.003124	0.999134	3.117878	0	2.283774	0.84968	30.55889	23.34575	48.03496	126.4993	11.04094	16.44935	13.03008	9.780058	8.244518	6.158258	3.640534	10.7557
IGHG2	245.0314	71.02772	66.94196	133.0295	138.4356	90.20907	146.9947	588.0272	297.6584	471.7033	1044.522	935.7196	740.2207	1473.33	1172.629	1507.098	722.5689	514.2254	267.0998
CLUL1	177.7679	285.1113	22.98007	507.1749	48.72934	240.9382	399.3497	1361.26	28.01491	13.44979	0	99.36845	136.7352	38.15953	55.74633	74.20066	69.79359	131.0592	131.7573
AC005520.2	310.3731	75.02928	195.8302	81.06484	102.9961	334.5729	120.6546	270.3999	287.1528	687.8607	1001.151	326.6278	382.4474	136.8159	140.8328	107.1787	143.6927	111.0363	76.18618
AC025175.1	122.9962	128.05	105.9082	80.02554	130.6832	105.0536	79.86994	64.82189	80.54285	224.8036	246.9747	105.809	82.24675	150.7767	163.327	100.5831	80.05735	73.72081	69.91202
AC018665.1	446.822	539.2104	431.6257	351.281	388.7272	365.4038	327.1269	458.3834	499.5991	724.3672	1110.784	510.6434	506.8456	441.1614	471.3988	335.5519	492.6606	451.4262	546.7478
SFRP2	55.73264	88.03436	89.92203	229.6837	58.6967	14.84453	13.59488	107.4191	147.0783	17.29259	20.48083	17.48149	28.78636	40.02097	20.53812	285.2603	174.484	34.58507	24.20031
AC067930.6	27.86632	29.01132	40.96448	4.157171	14.3973	29.68906	27.18977	34.263	63.03354	77.81664	116.8612	56.12477	26.73019	53.05106	27.38416	14.01568	42.08143	27.304	27.78555
MMRN1	1391.394	2319.905	1235.928	2016.228	1757.579	641.7405	666.1493	1518.684	337.3462	158.5154	146.9801	546.5265	659.0021	1412.833	1280.21	946.4707	1032.535	711.7243	410.509
PGAM4P2	15.37452	2.000781	0	0	1.107485	2.283774	0	0	25.68033	0	0	0	0	0	0	0	0	0	0
AC015920.1	48.04538	51.01991	102.9108	47.80747	178.3051	73.08077	58.62794	84.26846	57.1971	24.01748	13.2523	34.96297	26.73019	76.31906	43.03226	14.84013	37.97592	11.83173	15.23724
RRH	54.77173	148.0578	119.896	138.2259	125.1458	114.1887	67.12474	91.67668	88.71387	24.97818	44.57593	101.2086	59.62889	82.83411	98.77859	118.7211	112.9014	65.52961	100.3865
C20orf203	288.2723	239.0933	202.8241	169.4047	238.1093	317.4446	244.7079	248.1752	180.9296	440.0003	539.7302	247.501	290.9479	233.6108	206.3592	192.9217	225.8028	256.6576	292.1964
F5	1004.18	930.3631	3638.845	890.6739	2263.699	1222.961	2009.494	2888.278	1634.203	84.54153	43.37118	920.0782	925.2759	2576.234	2451.861	972.0287	1000.717	889.2003	1895.691
KCNJ8	310.3731	223.0871	311.7297	244.2338	285.7311	311.7352	215.8188	231.5068	448.2385	465.9391	760.2003	526.2847	513.0141	376.011	445.9707	561.4517	536.7948	759.0513	469.6654
SERPINB9P1	615.9417	647.2526	169.8527	1023.703	538.2377	614.3352	723.9276	970.4763	744.7296	262.2709	285.5269	113.1696	135.7071	336.9207	289.4897	204.4641	225.8028	146.5315	158.6465
G6PC2	257.5232	1270.496	1318.856	435.4637	869.3757	455.6129	441.8337	720.449	433.0637	209.4324	209.6273	218.0585	228.2347	447.6765	470.4208	124.4922	161.1411	244.8259	423.0574
IGLC2	247.9141	320.1249	126.89	42.611	311.2033	118.7562	88.36674	436.1587	367.6956	912.6643	972.2372	595.2906	551.0532	1853.064	1796.597	1325.719	623.0104	512.4051	167.6096
LINC01291	0.960908	386.1507	718.3771	443.778	0	437.3427	1105.434	509.3149	0	0.960699	0	667.0567	557.2217	732.4769	722.7463	1159.179	847.7868	740.8486	623.8303
NPM1P26	24.02269	21.0082	54.95235	40.53242	37.65449	36.54038	77.3209	64.82189	51.36066	10.56769	7.228529	43.24368	46.2638	20.47585	24.45015	50.29156	43.1078	53.69787	73.49725
SMCP	0.960908	27.01054	20.98181	5.196464	54.26676	44.53359	0	71.30408	121.3979	5.764196	0	0	4.112337	0	5.868035	4.946711	8.21101	12.74187	20.61508
VDAC3P1	27.86632	37.01445	51.95495	33.25737	24.36467	23.97963	26.34009	41.67122	10.50559	69.17035	109.6327	44.16376	51.40422	54.9125	48.90029	40.39814	39.0023	31.85467	37.64493
AC108865.1	52.84991	13.00508	18.98354	1.039293	1.107485	17.12831	2.549041	16.66849	18.6766	29.78168	31.32363	50.6043	46.2638	60.49682	49.8783	19.78684	29.76491	23.66347	21.51139
TRIM17	179.6897	127.0496	135.8822	96.65423	158.3703	140.4521	118.9552	188.9095	147.0783	226.725	263.8413	289.8246	307.3972	226.165	210.2713	190.4484	179.6158	260.2982	250.0699
ASB4	123.9571	72.02811	84.92636	71.7112	86.38382	137.0264	69.67378	65.74792	64.20083	33.62447	12.04755	49.68422	39.06721	51.18962	36.18622	28.85581	46.18693	71.90054	57.36371
CAPNS2	8.648168	14.00547	19.98267	33.25737	40.97694	15.98642	22.94137	13.89041	52.52795	3.842797	6.023774	23.92203	21.58977	21.40657	13.69208	7.420066	12.31652	20.93307	17.02985
AL359979.2	16.33543	20.00781	6.993936	16.62868	16.61227	7.993209	4.248401	18.52054	11.67288	0.960699	1.204755	6.440548	0	3.722881	6.846041	71.72731	59.52982	52.78774	44.8154
AL445483.1	2.882723	24.00937	41.96361	27.02161	38.76197	34.25661	24.64073	13.89041	75.8737	6.724895	1.204755	19.32164	25.70211	23.26801	7.824047	8.244518	15.39564	30.0344	28.68185
HTR3B	129.7225	204.0796	49.95668	385.5776	149.5105	181.56	293.9894	264.8437	4.669151	15.37119	14.45706	132.4913	128.5105	94.93347	108.5586	248.9845	271.9897	59.15867	194.4988
LINC01497	3.84363	8.003124	12.98874	4.157171	9.967364	5.709435	3.398721	21.29862	18.6766	204.6289	151.7991	11.04094	2.056169	22.33729	27.38416	9.06897	6.158258	2.7304	0
RAET1E-AS1	1.921815	5.001952	19.98267	43.6503	7.752394	12.56076	16.9936	29.63287	17.50932	0.960699	2.40951	2.760235	7.196591	13.03008	7.824047	50.29156	53.37157	12.74187	19.71877
NIFKP6	1.921815	0	0.999134	0	0	0	0	0	8.171014	0	0	0	0	0	0	0	0	0	0
VN1R81P	220.0478	217.0847	252.7808	366.8703	299.0209	198.6883	214.9691	223.1725	217.1155	122.9695	126.4993	228.1794	223.0943	229.8879	244.5015	191.2728	263.7787	355.8622	304.7447
AP001596.1	66.30262	95.03709	74.93502	67.55403	58.6967	57.09435	48.43177	54.6356	44.35693	122.9695	90.35662	119.6102	95.61185	108.8943	129.0968	89.86525	73.89909	70.99041	86.04556
LINC02550	119.1525	118.0461	82.92809	34.29666	55.37425	65.08756	45.88273	30.55889	30.34948	102.7948	157.8229	82.80704	52.4323	88.41843	109.5367	44.5204	54.39794	39.13574	66.32679
NDP	1408.69	1201.469	1493.705	961.3458	924.7499	1173.86	859.0267	840.8325	705.0418	1427.599	1669.79	1095.813	1280.993	1274.156	1263.584	761.7935	821.101	759.0513	811.1587

SEC62	48011.74	47342.48	47044.21	59985.9	45766.81	43363.16	51739.58	48234.9	42502.11	35110.68	29317.71	40177.98	42101.08	40737.63	42770.15	42361.16	38811.39	40948.72	39886.6
AL589880.1	34.59267	442.1726	449.6101	562.2574	447.4239	353.985	7.647122	431.5286	114.3942	21.13538	18.07132	411.275	454.4133	220.5807	277.7537	439.4328	371.5482	466.8984	315.5004
BTBD9-AS1	6.726353	4.001562	2.997401	15.58939	17.71976	7.993209	5.947762	4.630135	19.84389	0	0	2.760235	1.028084	4.653602	0	0.824452	2.052753	1.820267	4.48154
IGHG3	165.2761	82.03202	39.96535	63.39686	124.0383	28.54718	57.77826	335.2218	186.766	268.9958	851.7617	354.2301	233.3752	835.7868	661.1319	338.0253	228.8819	205.6902	86.04556
IL20RB	142.2143	890.3475	652.4343	365.8311	478.4335	246.6476	194.5768	890.838	626.8335	170.0438	113.247	395.6336	404.0372	761.3292	658.1979	293.5049	190.906	279.411	174.7801
MAP1LC3B2	185.4552	143.0558	161.8597	156.9332	112.9635	138.1683	621.9659	141.6821	106.2232	597.5549	584.3061	207.0176	202.5326	177.7676	166.261	169.0126	143.6927	207.5104	171.1948
SNORD3C	198.9079	10.0039	0	0	0	2.283774	0	1.852054	2.334575	670.5681	1251.74	0	2.056169	0	0	0.824452	0	0.910133	1.792616
GOLGA2P8	36.51449	34.01327	27.97574	36.37525	15.50479	30.83095	33.13753	8.334243	8.171014	51.87776	77.10431	47.84407	33.92678	44.67457	23.47214	34.62698	51.31881	20.02294	58.26002
ZNF812P	603.4499	878.3428	490.5746	724.3871	468.4661	1792.763	930.3999	1287.178	597.6513	336.2447	321.6696	221.7389	206.645	323.8907	355.0161	500.4423	345.8888	922.8753	417.6795
TSHB	29.78813	3.001171	8.992203	33.25737	17.71976	14.84453	21.24201	3.704108	10.50559	32.66377	28.91412	15.64133	69.90974	43.74385	53.79032	37.10033	43.1078	53.69787	69.01571
CRYGN	262.3278	318.1242	307.7332	175.6405	219.282	381.3903	389.1535	354.6684	414.3871	441.9217	796.343	271.4231	310.4815	326.6828	343.28	236.6177	245.3039	336.7494	319.9819
AC083843.1	36.51449	23.00898	56.95062	37.41454	27.68712	7.993209	19.54265	29.63287	12.84017	0.960699	0	11.96102	13.3651	17.68369	23.47214	5.771163	13.34289	31.85467	24.20031
OR7E99P	25.9445	1.00039	15.98614	25.98232	56.48173	7.993209	50.13113	23.15068	24.51304	0	0	28.52243	40.09529	16.75297	11.73607	21.43575	36.94955	56.42827	55.57109
AP003355.2	125.8789	136.0531	126.89	222.4087	215.9596	99.34417	67.12474	76.86024	285.9855	69.17035	36.14265	123.2905	83.27483	126.578	135.9428	229.1976	227.8555	190.2179	133.5499
SNORD3B-2	5.765445	11.00429	5.994802	10.39293	0	1.141887	4.248401	0	10.50559	48.03496	110.8374	6.440548	6.168506	10.23792	10.75806	9.06897	6.158258	7.281067	15.23724
IGLV1-40	61.49808	57.02225	10.99047	0	23.25718	5.709435	18.69297	37.96711	40.85507	51.87776	356.6074	80.96688	105.8927	245.7102	353.0601	107.1787	81.08372	121.9579	30.47447
RPL12P37	24.02269	19.00742	21.98094	29.1002	3.322455	62.80379	16.14392	15.74246	30.34948	48.03496	63.85201	72.68618	78.13441	69.80402	40.09824	43.69595	30.79129	46.4168	49.29694
CETP	135.488	87.03397	119.896	85.22201	76.41646	163.2898	85.8177	125.0136	70.03726	166.201	271.0698	183.0956	138.7914	120.9936	175.063	291.8559	313.0448	156.5429	167.6096
FRG1JP	572.7009	529.2065	769.3329	472.8782	763.0571	312.877	514.0565	323.1834	312.8331	236.332	325.2838	305.466	220.0101	290.3847	229.8314	188.7995	193.9851	196.5888	172.9874
OXCT2P1	37.47539	82.03202	25.97748	43.6503	40.97694	28.54718	54.37954	33.33697	103.8886	6.724895	3.614265	61.64524	56.54464	15.82225	29.34017	23.9091	60.5562	43.6864	49.29694
AL662844.3	14.41361	23.00898	26.97661	9.353635	4.42994	37.68227	31.43817	12.03835	21.01118	48.99566	48.1902	29.4425	23.64594	27.92161	23.47214	18.13794	31.81766	49.1472	52.88217
AC048341.3	128.7616	162.0633	268.767	100.8114	229.2494	258.0665	130.8508	226.8766	342.0153	605.2405	775.8621	335.8286	322.8185	272.701	214.1833	138.5079	113.9278	184.7571	156.8539
NTRK1	10817.9	13581.3	9703.586	7234.517	8343.791	14795.43	10896.3	9661.24	14288.77	18854.68	30922.44	14872.14	14612.16	13086.86	11588.39	8673.233	12638.8	15019.93	21030.07
PLA2G7	559.2482	366.1429	288.7496	612.1434	433.0266	437.3427	369.6109	350.9642	342.0153	239.2141	165.0514	385.5128	377.307	338.7822	338.39	453.4485	360.2581	407.7398	382.7235
RGS13	68.22443	4.001562	27.97574	59.23969	29.90209	53.66869	11.89552	50.00546	11.67288	0	0	27.60235	22.61786	35.36737	12.71408	28.03136	12.31652	121.9579	97.69757
TBC1D3E	7.68726	3.001171	5.994802	3.117878	19.93473	4.567548	189.4787	7.408216	12.84017	462.0963	738.5147	15.64133	21.58977	16.75297	12.71408	9.06897	4.105505	14.56213	11.652
VSX1	23.06178	25.00976	21.98094	45.72888	95.2437	7.993209	24.64073	30.55889	26.84762	10.56769	1.204755	41.40352	32.8987	23.26801	26.40616	13.19123	33.87042	26.39387	33.16339
AC073109.1	3.84363	1.00039	1.998267	0	0	2.283774	13.59488	0	4.669151	18.25329	20.48083	3.680313	8.224675	8.376483	8.802052	2.473355	1.026376	0	5.377848
SNX18P9	7.68726	7.002733	79.93069	20.78586	23.25718	30.83095	8.496802	20.37259	30.34948	0	1.204755	7.360626	7.196591	6.515042	19.56012	8.244518	0	22.75334	28.68185
SLC6A9	272.8977	139.0543	299.7401	188.112	345.5353	303.7419	124.0533	249.1013	270.8108	631.1794	972.2372	316.5069	305.3411	376.011	327.632	549.9094	451.6056	361.323	437.3983
MIR4645	32.67086	31.0121	6.993936	42.611	14.3973	41.10793	33.13753	30.55889	15.17474	1.921399	1.204755	8.280704	9.252759	12.09936	24.45015	9.06897	4.105505	6.370934	0.896308
PROM1	90.32531	80.03124	29.97401	25.98232	14.3973	23.97963	62.02666	13.89041	19.84389	56.68126	63.85201	52.44446	97.66802	104.2407	112.4707	146.7524	169.3521	123.7781	117.4163
CYP4A22-AS1	95.12985	88.03436	106.9073	192.2692	132.8982	103.9117	141.8966	116.6794	88.71387	47.07426	62.64725	108.5692	140.8476	107.0328	92.91055	125.3167	133.4289	120.1376	159.5428
SNORD11	3.84363	12.00469	12.98874	45.72888	18.82724	5.709435	23.79105	26.85478	19.84389	2.882098	0	15.64133	22.61786	24.19873	19.56012	17.31349	5.131881	26.39387	34.95601
AL162725.2	31.70995	164.064	145.8735	87.30059	136.2206	95.91851	122.354	98.15887	31.51677	43.23147	13.2523	61.64524	50.37613	46.53602	56.72434	28.03136	34.89679	30.94454	37.64493
CROCC2	10.56998	5.001952	11.9896	5.196464	12.18233	9.135096	1.69936	20.37259	29.18219	38.42797	101.1994	47.84407	26.73019	20.47585	10.75806	63.48279	54.39794	48.23707	55.57109
IGKV1-33	12.4918	22.00859	14.987	0	25.47215	38.82416	11.04584	12.96438	1.167288	0.960699	531.2969	173.8948	35.98295	76.31906	279.7097	37.92478	9.237386	37.31547	2.688924
KCNJ1	104.7389	58.02265	75.93416	129.9116	46.51437	83.35775	87.51706	98.15887	116.7288	58.60265	33.73314	37.72321	42.15146	37.22881	26.40616	132.7367	82.1101	102.8451	85.14926
AL357054.4	76.8726	161.0629	269.7661	254.6267	197.1323	220.3842	82.41898	207.4301	151.7474	79.73804	92.76613	156.4133	182.999	127.5087	99.75659	267.9468	276.0952	281.2312	227.6622
RPL36AP29	0	18.00703	12.98874	6.235757	29.90209	5.709435	0	0.926027	112.0596	0.960699	0	0	1.028084	1.861441	4.890029	0	5.131881	20.93307	12.54831
FLT3	1255.906	1363.532	992.1397	1532.957	972.3718	931.7798	1109.682	1401.079	846.2836	540.8737	444.5545	933.8794	807.0462	1098.25	1078.74	914.3171	810.8372	1036.642	1166.993
MYT1L-AS1	24.02269	10.0039	17.98441	55.08252	21.04221	5.709435	32.28785	9.26027	4.669151	0	0	10.12086	17.47743	9.307203	4.890029	24.73355	27.71216	6.370934	22.4077
AC090616.6	97.05166	142.0554	186.838	61.31827	147.2955	133.6008	156.3412	238.915	157.5838	298.7775	445.7593	230.0196	187.1114	154.4996	150.6129	201.9907	193.9851	155.6328	196.2914
AC023158.1	45.16265	72.02811	55.95149	34.29666	44.2994	25.12151	23.79105	37.96711	25.68033	93.18783	81.92333	84.6472	137.7633	60.49682	71.39443	23.08465	43.1078	34.58507	19.71877
CU638689.5	7.68726	14.00547	6.993936	19.74656	24.36467	15.98642	23.79105	7.408216	16.34203	1.921399	0	1.840156	8.224675	5.584322	5.868035	0	7.184634	9.101334	12.54831
SPATC1L	502.5546	523.2042	379.6708	348.1631	335.5679	550.3895	350.9179	336.1478	752.9006	684.9786	1531.243	493.1619	457.4975	600.3146	549.6393	441.0817	587.0872	493.2923	504.6214
LINC01876	97.05166	15.00586	29.97401	22.86444	32.11706	59.37812	134.2495	75.93422	80.54285	72.05244	75.89956	97.52829	102.8084	123.7858	151.5909	47.81821	63.63533	98.29441	67.2231
GLUD1P8	51.88901	39.01523	39.96535	48.84676	87.49131	34.25661	63.72602	40.74519	96.88488	13.44979	7.228529	65.32555	71.96591	49.32818	26.40616	30.50472	22.58028	31.85467	60.94894
RNU2-11P	53.81082	54.02108	80.92983	71.7112	57.58922	100.4861	37.38593	51.85751	53.69524	24.97818	21.68559	40.48344	25.70211	44.67457	43.03226	51.11601	46.18693	44.59654	46.60801
FLJ42393	23.06178	17.00664	14.987	58.2004	159.4778	55.95246	16.14392	10.1863	443.5693	24.01748	16.86657	18.40156	16.44935	27.92161	21.51613	11.54233	20.52753	12.74187	11.652
ADAMTS7P1	24.02269	12.00469	0.999134	5.196464	2.21497	15.98642	3.398721	39.81916	3.501863	119.1267	272.2746	20.24172	4.112337	7.445762	5.868035	4.122259	21.5539	8.191201	13.44462
MEGF6	192.1815	293.1144	151.8683	67.55403	290.161	176.9925	100.2623	140.7561	344.3499	689.7821	2099.888	230.9396	246.7402	157.2917	130.0748	171.486	281.2271	209.3307	257.2404
AC016722.1	5.765445	3.001171	3.996535	7.275049	8.859879	5.709435	6.797442	0	0	9.606993	28.91412	9.200782	5.140422	10.23792	7.824047	12.36678	6.158258	10.9216	11.652
AL133351.1	25.9445	31.0121	23.97921	2.078586	1.107485	4.567548	5.098081	7.408216	2.334575	23.05678	37.3474	23.00196	14.39318	42.81313	29.34017	8.244518	4.105505	10.9216	16.13354
EFNB3	250.7969	189.0738	172.8501	165.2476	109.641	172.4249	48.43177	119.4575	122.5652	255.546	374.6788	260.3821	287.8636	267.1167	197.5572	219.3042	135.4817	181.1165	132.6536
EVX1-AS	1.921815	0	0	0	0	0	0	0	7.003726	0	0	0	0	0	0	0	0	0.910133	0
TP63	514.0855	470.1835	699.3936	589.279	698.823	652.0175	328.8263	1422.378	387.5395	195.0219	159.0276	614.6123	562.3622	643.1277	626.9017	485.6021	431.078	176.5659	245.5884
YWHAQP5	0	10.0039	0.999134	29.1002	6.64491	5.709435	14.44456	3.704108	10.50559	0	0	10.12086	5.140422	0	0.978006	36.27588	20.52753	22.75334	19.71877
DLX5	17.29634	9.003514	14.987	9.353635	32.11706	45.67548	22.09169	19.44657	39.68778	61.48475	106.0184	39.56336	35.98295	50.2589	38.14223	31.32917	55.42432	52.78774	48.40063
HAP1	734.1334	374.146	436.6214	303.4735	433.0266	340.2823	390.0032	236.1369	462.2459	701.3105	1089.098	762.7449	629.1876	413.2398	384.3563	395.7369	413.6296	704.4433	818.3292
AC010105.1	2.882723	0	0	1.039293	0	0	0	3.704108	0	0	0	0	0	0	0	0	0	0	0
AC103591.3	128.7616	73.0285	106.9073	123.6758	188.2724	115.3306	184.3806	105.5671	99.21946	47.07426	30.11887	141.692	107.9489	127.5087	145.7229	103.0565	83.13648	117.4072	145.2019

	Con1	Con2	Con3	Con4	Con5	Con6	Con7	Con8	Con9	RA1	RA1	RA2	RA2	RA3	RA3	RA4	RA4	RA5	RA5
5S_rRNA	19.21815	33.01288	48.95755	36.37525	25.47215	27.40529	38.23561	25.92876	0	45.15286	12.04755	32.20274	32.8987	34.43665	33.2522	32.15362	36.94955	48.23707	41.23017
7SK	0.960908	0	2.997401	3.117878	3.322455	0	0.84968	0	2.334575	0	0	3.680313	3.084253	6.515042	0.978006	0	0	2.7304	3.585232
A1BG	122.0353	125.0488	157.8631	173.5619	159.4778	166.7155	139.3476	239.841	117.8961	146.987	180.7132	161.0137	129.5386	146.1231	141.8108	108.0032	139.5872	117.4072	136.2388
A1BG-AS1	610.1763	564.2202	335.7089	481.1926	594.7194	582.3624	459.677	343.556	667.6886	491.878	736.1052	681.778	600.4013	411.3784	398.0484	639.7746	730.7799	404.0992	739.4541
A1CF	14.41361	19.00742	6.993936	24.94303	1.107485	5.709435	51.83049	0.926027	2.334575	11.52839	1.204755	4.600391	2.056169	10.23792	6.846041	9.06897	13.34289	10.9216	12.54831
A2M	279775.9	143730.1	193570.2	322288.8	172911.6	194432.5	107061.4	178103.7	130010.2	153239.2	173522	186442.8	188099.3	204049.3	223408.8	517922.3	416656.4	445867.1	312700.3
A2M-AS1	1417.339	1394.544	1546.659	822.0806	1336.734	948.9081	902.3604	1343.665	1148.611	1512.141	1877.008	997.3648	1136.033	1259.265	1336.934	540.8404	611.7202	749.0398	947.3975
A2ML1	3480.407	1865.728	2778.591	795.059	1873.864	2181.004	1366.286	1698.334	1275.845	994.3237	690.3245	1351.595	1634.654	2203.946	2494.893	906.0726	1112.592	496.0227	556.6072
A2ML1-AS1	3.84363	5.001952	1.998267	5.196464	1.107485	0	2.549041	1.852054	0	9.606993	0	10.12086	5.140422	4.653602	0	2.473355	0	4.550667	3.585232
A2ML1-AS2	0	0	0	0	0	0	0	0	0	0	2.40951	0.920078	0	0	0	0	1.026376	0	0
A2MP1	286.3504	155.0605	236.7947	119.5187	341.1054	156.4385	167.387	338.9259	367.6956	256.5067	377.0883	225.4192	218.982	282.0083	282.6437	117.0722	162.1674	487.8315	387.205
A3GALT2	36.51449	56.02186	60.94715	15.58939	17.71976	11.41887	32.28785	14.81643	67.70269	21.13538	40.96167	32.20274	42.15146	22.33729	24.45015	56.06272	55.42432	27.304	35.85232
A4GALT	1425.987	2030.793	1294.877	1345.884	2422.07	1787.053	1023.015	1689.073	5277.308	2692.84	5249.117	2060.975	1996.54	1704.149	1448.427	2456.042	2867.695	1539.036	1538.064
A4GNT	82.63805	52.0203	50.95582	97.69352	29.90209	11.41887	11.04584	111.1232	0	2.882098	0	172.0546	137.7633	71.66546	49.8783	116.2477	120.086	204.78	138.9277
AAAS	9174.745	9891.861	10762.67	7597.23	10690.55	10008.64	6982.672	10332.61	18498.01	12818.61	17682.19	10185.27	10251.03	10126.24	10353.17	8709.509	9250.729	10062.44	9676.541
AACS	7082.849	9799.825	9285.948	9240.352	12965.33	10216.46	6495.805	8279.608	7544.181	13639.05	14208.88	7835.386	7777.458	8701.304	8123.316	9211.6	11287.06	7279.247	10139.93
AACSP1	0	14.00547	13.98787	0	11.07485	0	5.098081	1.852054	0	0	0	3.680313	6.168506	0.93072	3.912023	7.420066	18.47477	10.9216	0.896308
AADAC	2.882723	3.001171	0	1.039293	0	1.141887	1.69936	0	0	10.56769	0	0.920078	0	0	0	9.06897	0	0	0
AADACL2-AS1	1.921815	2.000781	0	0	2.21497	0	1.69936	0	0	0	1.204755	0.920078	0	0.93072	0	7.420066	3.079129	0	5.377848
AADACL4	0.960908	2.000781	0	0	0	0	0	0	0	0	0	0	1.028084	0	0	0.824452	2.052753	0.910133	0
AADACP1	36.51449	5.001952	8.992203	19.74656	8.859879	46.81737	14.44456	20.37259	1.167288	14.41049	36.14265	1.840156	27.75828	14.89152	15.64809	1.648904	15.39564	13.652	13.44462
AADAT	2345.575	2449.956	2891.493	2320.741	2612.557	2605.786	2613.616	2818.826	2971.915	2982.971	2875.75	2526.535	2799.474	2306.325	2422.52	2049.587	2031.199	2248.94	2408.379
AAED1	3469.837	3389.323	3464.996	4213.293	3823.038	4086.814	3097.934	3324.437	3564.897	3918.692	3728.716	3216.594	3245.662	3400.852	3650.896	4174.2	3947.443	3337.459	2749.873
AAGAB	9471.666	10465.08	8797.372	13241.63	8846.59	10506.5	11351.73	9933.492	6546.15	7789.35	6533.386	9660.821	9888.116	9317.441	8857.799	11518.42	11242.93	10066.08	9162.06
AAK1	27660.68	27635.79	24320.91	39509.75	21095.37	23532.01	36011.15	25051.81	18403.46	20212.15	16308.77	27694.35	27582.48	29055.23	28260.46	33824.79	32027.04	27780	30269.22
AAMDC	2000.609	2123.829	1908.345	1610.904	1815.168	1814.458	1707.857	1929.84	2275.044	1776.333	2107.116	1329.513	1457.824	1921.937	1921.781	1375.186	1597.041	1649.162	1417.063
AAMP	14276.2	14077.49	11901.68	12474.63	15031.89	15224.78	11985.59	13760.76	17991.41	13873.46	17911.09	14033.95	13880.17	13952.43	12780.58	14066.8	14211.21	14071.57	16150.57
AANAT	159.5106	206.0804	269.7661	176.6798	218.1745	360.8363	169.936	426.8985	368.8629	208.4717	245.77	201.4971	134.6791	224.3036	158.4369	331.4296	352.0471	663.4873	414.9906
AAR2	5479.095	5161.014	5019.648	4519.884	5964.914	5466.213	4739.516	5044.995	6401.406	5160.876	6210.511	5064.111	4911.159	5740.683	5159.959	5795.072	5511.64	5866.72	5184.245
AARD	588.0754	608.2374	530.54	1468.521	470.6811	926.0704	645.757	625.9943	430.7292	651.3541	568.6443	520.7643	532.5477	765.0521	696.3401	1097.345	979.1629	327.648	514.4808
AARS	34175.64	35675.92	32447.86	38773.93	30493.49	29851.21	40630.86	32738.76	24299.43	28578.88	29875.51	31200.77	31763.69	37054.77	35202.34	35386.3	35295.03	33925.22	38473.12
AARS2	2408.034	2253.88	2135.149	2366.47	2694.511	2715.407	1893.088	2562.317	3325.603	2570.831	3596.193	2640.625	2551.705	2729.803	2304.182	2868.268	2766.084	2485.574	2908.519
AARSD1	694.7361	862.3366	1050.089	970.6994	881.558	709.1118	1046.806	880.6517	855.6219	876.1577	940.9136	968.8424	1034.253	938.1661	909.5454	700.7841	695.8831	961.1009	852.3889
AASDH	3076.826	3311.292	3197.228	3516.967	3180.697	3195	3887.287	3636.508	1894.508	2523.757	2487.819	3189.911	3326.881	3389.683	3449.427	3120.55	2696.29	3022.553	2783.932
AASDHPPT	12631.13	15701.13	12293.34	16382.37	10405.93	13122.57	14560.12	12973.64	9563.588	11909.79	8616.407	10776.88	11724.27	12483.75	12794.27	13048.6	12795.83	10575.75	11099.88
AASS	12486.99	18299.14	28253.5	17119.23	22108.72	17715.24	28613.83	21723.67	8679.952	13905.16	10716.29	19747.64	18040.82	18963.43	21499.5	14469.95	12694.22	19683.46	20595.36
AATBC	133.5661	102.0398	93.91856	75.86837	128.4683	143.8778	69.67378	114.8274	45.52422	110.4804	122.885	151.8129	161.4092	87.48771	95.84457	149.2258	146.7718	227.5334	166.7133
AATF	11198.42	6483.53	6784.118	8783.063	8763.528	7060.287	6909.6	7475.816	10384.19	6511.62	7510.442	6949.351	7361.084	7358.275	7460.228	8322.841	8211.01	7848.991	7755.753
AATK	8285.906	8501.318	7610.401	3300.794	18094.09	10899.31	6099.854	7196.156	33792.98	25501.76	82807.62	13301.57	12105.69	6815.665	5972.682	3685.3	7469.966	7422.138	9455.153
AATK-AS1	19.21815	2.000781	7.993069	1.039293	11.07485	1.141887	6.797442	8.334243	8.171014	0	8.433284	6.440548	3.084253	0.93072	1.956012	4.946711	19.50115	3.640534	3.585232
AB015752.	27.86632	10.0039	16.98527	22.86444	6.64491	21.69585	29.73881	31.48492	0	25.93888	16.86657	17.48149	12.33701	29.78305	24.45015	15.66458	30.79129	20.93307	14.34093
ABALON	49.96719	24.00937	41.96361	29.1002	22.1497	30.83095	34.83689	12.96438	22.17847	17.29259	25.29985	28.52243	37.01104	35.36737	37.16422	40.39814	31.81766	43.6864	49.29694
ABAT	14085.94	18293.14	18583.89	16982.04	16502.63	18073.79	16945.17	20657.81	11100.91	14715.99	13327	15650.53	17536.04	15758.03	15953.23	14850.03	14738.76	12942.1	16337
ABBA0100(	7.68726	3.001171	3.996535	8.314342	3.322455	1.141887	0.84968	0.926027	0	0	1.204755	4.600391	3.084253	2.792161	0	0.824452	4.105505	7.281067	9.859387
ABBA0103	2.882723	6.002343	0	2.078586	1.107485	1.141887	0	0.926027	0	0.960699	3.614265	0	1.028084	0	0	0	0	1.820267	0
ABBA0103	12.4918	3.001171	4.995668	7.275049	22.1497	7.993209	1.69936	3.704108	5.836439	2.882098	1.204755	4.600391	13.3651	3.722881	11.73607	3.297807	1.026376	6.370934	11.652
ABBA0103	7.68726	5.001952	3.996535	9.353635	4.42994	10.27698	3.398721	3.704108	8.171014	1.921399	12.04755	6.440548	1.028084	3.722881	7.824047	3.297807	6.158258	9.101334	9.859387
ABBA0103	4.804538	21.0082	10.99047	2.078586	8.859879	6.851322	1.69936	4.630135	9.338302	1.921399	12.04755	5.520469	5.140422	6.515042	5.868035	1.648904	6.158258	5.4608	4.48154
ABCA1	35816.87	19647.67	16833.4	25165.44	13536.79	22174.3	28140.56	16456.43	10856.94	11250.75	5688.853	31102.32	29766.13	18793.1	20169.41	36814.25	24490.36	23060.05	18694.29
ABCA10	29002.11	21461.38	31931.31	19739.29	24253.92	14536.22	31771.24	23420.15	17412.43	25851.46	23293.94	25812.79	26589.35	27264.52	30044.34	15400.76	17678.3	17871.38	18368.94
ABCA11P	338.2395	358.1398	372.6769	407.4028	335.5679	227.2355	341.5715	358.3725	337.3462	352.5766	227.6987	333.9884	305.3411	349.0201	356.9721	300.9249	314.0711	353.1318	267.9961
ABCA12	91.28622	55.02147	56.95062	51.96464	19.93473	37.68227	67.97442	40.74519	26.84762	17.29259	18.07132	58.88501	67.85357	26.99089	47.92229	154.9969	125.2179	22.75334	57.36371
ABCA13	1497.094	644.2514	1001.132	312.8271	415.3068	876.9692	755.3657	724.1531	431.8965	582.1837	267.4556	654.1756	836.8607	794.8351	1007.346	826.9252	752.3338	570.6537	554.8146
ABCA17P	371.8712	505.1972	375.6743	307.6307	416.4143	432.7752	522.5533	403.7478	499.5991	428.4719	485.5162	509.7233	498.6209	393.6947	406.8504	276.1914	366.4163	427.7627	466.9764
ABCA2	29582.5	25315.88	24473.78	11659.83	32317.52	26611.68	18998	21000.44	50284.42	35965.7	72504.56	27742.2	28126.33	23429.02	21215.88	11034.46	16824.36	20316.91	27747.9
ABCA3	16993.65	17573.86	10435.95	12556.74	14468.18	15222.5	15604.38	11686.46	16352.53	13901.32	23574.64	23126.17	21184.71	16102.39	13284.25	18951.67	23795.51	16661.81	27319.47
ABCA4	92.24712	95.03709	114.9004	130.9509	83.06137	83.35775	128.3017	100.9369	79.37557	48.03496	66.26152	90.16767	127.4825	164.7375	121.2727	166.5393	215.539	141.0707	128.172
ABCA5	13536.3	14927.83	13344.43	22472.63	12804.74	13345.23	21694.04	15162.77	12856.51	11576.43	8847.72	15581.52	16538.79	14451.29	14126.32	18562.53	17784.02	14036.99	14728.13
ABCA6	18337.96	24597.6	31384.79	16377.18	17690.96	25505.19	26578	23206.24	12337.06	18206.21	15955.77	20984.22	20581.22	26102.05	28005.2	17698.51	16032	23059.14	22482.09
ABCA7	3295.913	3770.472	2561.779	2551.464	2892.751	4451.076	2303.483	2936.432	5877.294	5418.344	9598.282	3920.453	3654.84	3852.251	3068.982	2979.569	4064.45	2997.069	5269.394
ABCA8	181433.8	184727.1	185442.2	143581.4	170731	138799.8	204592.8	205992.9	43466.29	100990.6	95786.45	158892.9	166482.8	135169.4	144289.1	111485.7	112278.4	149977.2	156446.1

ABCA9	37600.31	33842.21	32965.42	26776.34	29442.49	25748.41	30536.66	34323.19	33177.82	21678.18	20171.21	38575.2	39092.91	31278.72	33335.33	26647.93	27353.95	31137.48	28948.06
ABCA9-AS	61.49808	34.01327	67.94109	36.37525	32.11706	26.2634	58.62794	62.04381	33.85134	35.54587	14.45706	46.00391	57.57272	38.15953	57.70234	44.5204	27.71216	20.02294	16.13354
ABCB1	12290.97	9399.669	6638.244	8660.427	8929.651	9742.58	7896.928	6110.852	4851.248	8300.442	8054.991	9921.204	11189.67	11217.04	12121.4	8346.75	9634.594	7058.085	7217.072
ABCB10	4139.59	3506.368	4037.499	5079.024	4048.965	4238.685	3590.749	4335.659	2261.036	3120.351	2414.329	3409.81	3652.784	3820.607	3801.509	4088.457	3546.13	4035.532	3263.457
ABCB10P1	73.02897	5.001952	28.97488	136.1474	3.322455	31.97284	82.41898	23.15068	4.669151	17.29259	9.638039	24.84211	31.87062	40.02097	54.76833	68.4295	51.31881	62.79921	48.40063
ABCB10P3	3.84363	3.001171	1.998267	4.157171	2.21497	0	10.19616	5.556162	0	2.882098	0	3.680313	8.224675	0	1.956012	4.122259	5.131881	7.281067	2.688924
ABCB10P4	0.960908	11.00429	22.98007	20.78586	0	6.851322	8.496802	0	9.338302	0.960699	2.40951	4.600391	2.056169	7.445762	6.846041	4.122259	16.42202	18.20267	0.896308
ABCB11	6.726353	0	0	2.078586	15.50479	4.567548	8.496802	0.926027	9.338302	0	14.45706	4.600391	8.224675	0	4.890029	14.01568	12.31652	10.01147	30.47447
ABCB4	400.6984	262.1023	502.5642	292.0413	325.6006	231.8031	418.8924	247.2492	385.205	519.7383	692.7341	334.9085	308.4253	253.1559	320.7859	234.1443	211.4335	415.931	475.0432
ABCB5	54.77173	15.00586	88.9229	9.353635	55.37425	20.55397	45.88273	12.96438	19.84389	25.93888	7.228529	27.60235	28.78636	49.32818	12.71408	16.48904	12.31652	15.47227	12.54831
ABCB6	591.919	825.3221	823.2861	865.7309	1366.636	1294.9	657.6525	1136.235	2301.891	1293.101	1492.691	1072.811	1222.392	911.1752	926.1715	929.9817	884.7363	441.4147	521.6512
ABCB7	3819.607	3710.448	3703.788	3686.371	3471.965	3727.119	3689.312	3586.503	2744.293	3034.849	2728.77	3064.781	3349.499	3649.354	3782.927	3646.55	3167.397	3777.964	3223.123
ABCB8	3937.799	4428.728	4236.327	4115.599	5045.701	5277.802	4152.387	4556.979	6387.398	7020.79	11976.47	5704.485	5250.427	4991.453	4468.509	5532.896	6689.92	5178.659	6923.083
ABCB9	2144.746	2912.137	2436.887	2039.092	3867.337	3064.825	2894.011	2879.944	3968.778	3716.945	5927.394	2849.482	2879.664	2987.612	2524.233	2453.569	2925.172	2029.598	2947.957
ABCC1	4203.97	4816.88	4182.374	5619.456	4779.905	4155.327	4745.464	4459.746	5005.33	4838.081	5428.625	5679.643	4970.788	5032.405	4757.998	7214.778	6374.823	5481.734	5521.257
ABCC10	4548.936	4587.791	4325.25	4250.707	5277.166	5664.901	3905.98	5122.782	6525.138	7021.751	10142.83	5353.015	5308	5314.413	5031.84	5340.799	5386.423	4729.053	5664.666
ABCC11	165.2761	124.0484	57.94975	117.4401	107.426	73.08077	159.7399	35.18903	39.68778	78.77734	61.4425	51.52438	71.96591	94.00275	84.1085	103.8809	89.29473	124.6883	172.9874
ABCC12	124.918	134.0523	98.91423	121.5973	155.0479	75.36454	130.8508	91.67668	126.0671	124.8909	174.6895	118.6901	186.0833	134.9544	148.6569	147.5769	159.0883	119.2275	233.9364
ABCC13	24.02269	135.0527	7.993069	5.196464	11.07485	14.84453	151.2431	12.96438	0	11.52839	8.433284	131.5712	113.0893	5.584322	1.956012	4.946711	0	0.910133	17.92616
ABCC2	344.0049	207.0808	421.6344	172.5226	271.3338	235.2287	271.8977	231.5068	228.7884	268.9958	261.4318	269.5829	231.319	260.6017	234.7214	213.533	159.0883	202.9598	206.1508
ABCC3	904.214	415.162	265.7696	1324.059	905.9227	485.302	659.3519	355.5944	3192.532	568.734	696.3483	1095.813	922.1917	946.5425	797.0747	1292.74	1124.908	937.4374	700.9128
ABCC4	4468.22	3654.426	3907.612	7655.431	4489.744	4720.561	4173.629	5247.795	1585.177	3291.356	2552.876	4163.354	3817.277	4404.168	4863.623	6191.633	4924.553	6299.033	6703.487
ABCC5	35971.57	33687.15	29241.64	35979.28	25618.34	31508.09	39345.29	30519.07	28040.59	30058.36	28506.91	35193.91	35841.08	36010.5	35400.88	38961.94	38927.37	28871.25	35538.61
ABCC5-AS	3.84363	5.001952	0.999134	7.275049	1.107485	6.851322	0.84968	1.852054	3.501863	1.921399	0	0.920078	4.112337	5.584322	4.890029	7.420066	3.079129	7.281067	0.896308
ABCC6	196.986	152.0593	298.741	136.1474	158.3703	260.3502	129.1514	137.978	326.8406	257.4674	324.0791	320.1872	277.5828	227.0958	149.6349	266.2979	302.781	151.9923	109.3496
ABCC6P1	7.68726	18.00703	14.987	5.196464	8.859879	33.11472	31.43817	24.0767	37.35321	22.09608	42.16642	12.8811	10.28084	2.792161	5.868035	18.96239	13.34289	9.101334	10.7557
ABCC6P2	8.648168	12.00469	6.993936	18.70727	2.21497	11.41887	10.19616	22.22465	2.334575	12.48909	9.638039	4.600391	4.112337	0.93072	1.956012	14.84013	2.052753	1.820267	5.377848
ABCC8	1606.637	1165.455	1390.794	730.6228	1156.214	1435.352	1294.063	1486.273	2854.019	3014.674	4325.07	2127.221	2311.134	1363.505	1092.433	1075.085	1338.395	1169.521	1999.663
ABCC9	1407.73	872.3405	1674.548	1490.346	1534.974	1164.725	1153.866	933.4353	682.8633	1332.49	1080.665	2368.281	2593.857	1461.231	1722.268	1424.653	1464.639	3071.7	2331.297
ABCD1	969.5557	586.2288	598.4811	490.5462	918.105	903.2326	610.0704	759.3422	2148.977	1098.079	1872.189	915.4778	902.6581	602.176	555.5073	1028.916	1239.863	913.774	765.447
ABCD1P4	0	1.00039	0	0	0	0	0	0	0	0	0	0	0	0	0	0	0	0	0.896308
ABCD1P5	0	1.00039	0.999134	0	0	1.141887	1.69936	1.852054	7.003726	1.921399	0	1.840156	0	2.792161	0	2.473355	5.131881	0	0
ABCD2	1945.838	2382.93	1888.363	3117.878	1342.272	1588.365	3080.091	1776.12	549.7925	1102.883	468.6496	1694.784	1716.901	2120.181	1955.034	2304.343	2266.239	2003.204	1916.306
ABCD3	19672.66	24082.4	23098.97	23252.1	21316.87	20460.33	27774.35	22749.71	15273.96	20438.88	14565.49	17243.19	19442.1	21456.83	22525.43	17297.82	17667.01	16241.33	17092.59
ABCD4	10140.46	9390.665	9973.352	7008.99	11044.95	9634.101	7488.232	9035.246	13024.6	11786.82	14496.82	9975.488	10355.89	10245.37	10078.35	8697.967	8863.785	10190.76	10495.77
ABCE1	11695.21	12222.77	11552.98	16804.32	11928.72	10555.6	13529.46	12889.37	6512.298	9418.695	7168.292	11211.15	11670.81	12305.98	12590.85	13363.54	11212.13	13342.56	11496.05
ABCF1	11604.88	12435.85	11097.38	12583.76	12450.35	12670.38	12294.87	12014.27	12163.14	10960.62	11986.11	12573.79	12432.62	11975.58	11110.15	13163.2	12771.2	12457.91	13172.14
ABCF2	12870.4	13601.31	13059.68	15057.27	11555.5	11890.47	13645.01	12643.97	11560.82	10445.68	10515.1	12463.38	12328.79	13410.75	12648.55	15454.35	14109.59	14269.98	12965.99
ABCF2P1	1.921815	0	19.98267	8.314342	0	9.135096	13.59488	6.482189	0	0	2.40951	8.280704	3.084253	8.376483	2.934017	5.771163	6.158258	0.910133	0
ABCF3	12071.88	12901.04	10441.95	11680.61	13053.92	13540.5	10683.88	11587.38	14605.1	12388.22	15803.97	12683.28	12727.68	12746.21	11517	13106.31	14660.76	11741.63	13656.15
ABCG1	5722.204	3184.243	3081.328	5831.472	2829.624	3785.355	4104.805	3291.1	3828.704	3239.478	3662.455	4624.313	4955.367	3940.67	3887.573	7247.756	6071.016	3890.82	4364.123
ABCG2	7049.218	8579.348	5712.047	8758.12	6235.14	8049.162	6502.603	5044.995	3764.503	6461.663	5603.315	5446.863	6128.411	9656.223	10509.65	7162.013	7901.044	4282.178	4483.332
ABCG4	2574.271	3497.365	2671.683	4098.971	2236.012	3745.389	3030.809	2763.265	4565.262	3730.395	4437.112	3041.779	2955.743	3409.228	3528.645	3865.03	3975.155	2239.838	4175.899
ABCG5	54.77173	38.01484	31.97228	41.57171	48.72934	38.82416	28.03945	66.67395	70.03726	50.91706	77.10431	11.04094	48.31997	36.29809	47.92229	32.15362	33.87042	36.40534	34.0597
ABCG8	50.9281	57.02225	34.96968	57.1611	26.57964	46.81737	60.3273	25.92876	30.34948	28.82098	6.023774	28.52243	44.20763	77.24979	60.63636	56.88718	45.16056	38.2256	78.8751
ABHD1	457.392	468.1827	483.5807	340.888	449.6389	671.4296	464.7751	494.4984	661.8521	796.4197	911.9994	519.8442	561.3341	447.6765	479.2229	298.4516	395.1549	460.5275	599.63
ABHD10	8787.499	10391.06	8229.864	11422.87	7757.932	9322.366	9879.232	8641.684	10917.64	9240.005	7911.625	7408.47	7821.666	8798.099	8849.975	8883.468	9191.199	7231.01	8098.142
ABHD11	1105.044	1403.548	1143.009	1136.986	1649.045	1611.203	956.74	1202.909	4670.318	2025.154	3335.966	1231.985	1203.887	1421.21	1160.893	1312.527	1555.986	1476.236	1538.961
ABHD11-AS	17.29634	19.00742	11.9896	15.58939	52.05179	6.851322	1.69936	30.55889	74.70641	23.05678	59.03299	1.840156	5.140422	32.57521	18.58211	33.80253	54.39794	9.101334	8.963079
ABHD12	14021.56	15490.05	13058.68	13087.81	14509.16	15382.36	12204.81	12998.64	25779.55	17229.18	20219.4	12331.81	12253.74	13636.91	13011.39	12206.83	13436.29	11870.87	13304.8
ABHD12B	367.0667	397.155	390.6613	614.222	497.2607	368.8295	374.709	410.23	506.6029	445.7645	407.2071	516.1639	596.2889	445.815	404.8944	404.8058	376.6801	594.3171	604.1116
ABHD13	4035.812	4182.632	3863.65	5438.619	3875.09	3566.113	4561.933	4185.642	1596.85	2938.779	2089.045	3788.882	3711.385	3654.008	3994.176	4312.708	3403.464	4828.258	3956.303
ABHD14A	454.5093	756.2952	683.4074	426.11	933.6098	1047.11	436.7356	754.712	2403.445	1354.586	2125.188	676.2575	634.3281	636.6127	611.2536	439.4328	613.773	559.7321	637.275
ABHD14A-/	23.06178	25.00976	18.98354	22.86444	14.3973	15.98642	11.89552	43.52327	24.51304	21.13538	4.81902	23.92203	19.5336	31.64449	24.45015	15.66458	22.58028	15.47227	13.44462
ABHD14B	4082.896	3503.367	3458.002	2791.54	3789.813	4349.448	2478.517	3889.314	10971.34	4961.051	7326.114	4107.229	4167.854	4003.028	3959.946	3553.387	3443.492	4868.304	3655.144
ABHD15	2653.066	2083.813	2698.66	1910.22	2364.48	3174.446	2201.522	2757.709	3783.18	2937.818	2781.779	2324.118	2432.448	2363.099	2289.512	1972.089	1941.904	2821.414	2512.351
ABHD16A	869.6213	816.3186	782.3217	763.8802	841.6885	755.9292	853.079	829.7202	804.2612	805.066	1039.703	938.4798	836.8607	905.5909	921.2815	745.3045	844.7077	1220.489	997.5907
ABHD17A	2136.097	1763.688	1720.508	1734.58	2663.501	1882.972	1545.568	1552.021	4527.909	3113.626	7318.886	2895.486	2417.026	2152.756	1913.957	1921.797	2330.9	3167.264	3268.835
ABHD17AF	0	1.00039	0.999134	0	3.322455	1.141887	0	1.852054	5.836439	0	8.433284	5.520469	5.140422	2.792161	1.956012	3.297807	2.052753	5.4608	2.688924
ABHD17AF	0	0	0	0	0	4.567548	0	1.852054	0	0	6.023774	2.760235	1.028084	0.93072	0	0	0	0	0.896308
ABHD17AF	0	0	0	0	0	0	0	0	0	0	0	0	1.840156	0	0	0	0	0	0
ABHD17AF	0	0	0	0	0	1.141887	0.84968	0	7.003726	0.960699	3.614265	0	0	0	1.956012	0.824452	0	0	0.896308

ABHD17AP	9.609075	16.00625	16.98527	7.275049	28.79461	38.82416	9.346483	6.482189	42.02236	38.42797	124.0898	39.56336	14.39318	20.47585	9.780058	12.36678	32.84404	14.56213	12.54831
ABHD17B	4300.061	4409.721	3845.665	4924.169	3636.98	4042.28	4879.714	4143.971	2108.122	3163.583	2322.767	3637.069	3934.479	3860.628	3870.947	4158.535	4304.622	3985.474	3475.882
ABHD17C	1868.004	1776.693	1504.695	1426.949	1581.488	1479.886	1569.359	1420.525	1309.697	1679.302	1504.739	1764.71	1808.4	1166.193	1511.997	1709.913	1905.981	2607.532	2020.278
ABHD18	3881.106	3383.32	3905.613	4553.142	3605.971	3415.384	4310.428	4360.661	2565.698	2946.465	2526.371	3918.613	4077.383	3545.114	3975.594	3970.56	3570.763	4415.057	3427.482
ABHD2	105933.3	118576.3	103189.5	154486.7	81751.21	99959.65	131489.7	89820.92	25606.79	62672.18	50184.06	118596.2	120743.4	105252.4	98533.11	135451.7	123973.9	94918.72	127374.3
ABHD3	10010.73	12883.03	13985.87	11827.15	10896.54	12312.97	9138.311	13095.87	9658.139	13780.27	12152.36	9136.377	9364.821	10336.58	10423.59	11865.51	10603.49	8341.373	9044.644
ABHD4	9166.097	7700.005	8826.347	6510.13	6839.827	7884.73	6930.842	7442.479	11752.25	7693.28	9362.15	8068.166	8101.305	9267.182	8894.963	8882.644	8370.098	6248.976	6301.941
ABHD5	1776.718	1888.737	1744.487	1993.364	2135.231	2193.565	2071.52	2020.591	1192.968	1741.748	1464.982	1961.607	1853.636	1964.751	1895.375	2495.616	2202.603	2090.576	1975.463
ABHD6	3165.229	3139.225	2862.518	3054.481	3697.892	3623.207	2589.825	3056.815	3113.156	3833.19	4088.938	2778.636	2918.732	3745.218	3290.012	3103.237	3176.634	2063.272	2211.192
ABHD8	222.9305	207.0808	154.8657	102.89	305.6658	286.6136	149.5437	259.2876	786.7519	470.7426	1075.846	261.3022	266.2739	167.5297	183.8651	280.3136	213.4863	261.2083	269.7887
ABI1	17253.09	15287.97	15211.81	18016.14	14615.48	15415.47	16369.09	16136.02	8339.104	11897.3	9368.174	14306.3	14323.27	14700.73	14760.06	17546.81	14859.88	18979.92	17281.71
ABI1P1	0.960908	4.001562	3.996535	2.078586	0	1.141887	4.248401	0.926027	4.669151	0	0	2.760235	2.056169	0	5.868035	5.771163	4.105505	5.4608	3.585232
ABI2	23201.11	24493.56	25275.08	28637.71	20986.84	22098.94	28321.54	23212.72	12251.85	18649.09	15104.01	22732.37	23555.47	21133.87	21456.47	21546.22	21011.97	18795.17	20028.9
ABI3	1275.124	1640.64	1579.63	1134.908	3066.626	2968.906	851.3796	2116.898	3602.25	1943.495	3049.235	2038.893	1771.389	2438.487	2261.149	3010.074	2565.941	5298.797	4055.793
ABI3BP	8807.678	7729.017	5549.188	7212.692	3196.201	3375.418	8970.924	8807.443	4093.678	1776.333	1721.595	6758.895	6064.67	5244.609	5972.682	3993.645	2504.358	5843.057	3451.682
ABL1	10601.69	6262.444	7982.079	6907.14	9164.438	6228.994	8179.022	6895.197	10335.17	7091.882	10016.33	9697.625	9107.799	8547.735	8149.723	8328.612	7405.305	11620.58	9957.085
ABL2	12889.61	10600.14	14608.33	16388.61	15355.28	9130.528	16644.39	11425.32	10470.57	10744.46	10887.37	14464.55	13346.59	13005.89	13470.07	15892.13	12726.04	20272.31	19746.56
ABLIM1	75209.27	78931.81	76004.1	88000.04	53761.75	66067.3	94520.13	64552.42	47886.81	50468.41	40421.94	71939.08	73210.92	75967.25	74129.91	76349.19	76834.53	65367.6	75957.62
ABLIM2	1865.122	2473.966	1632.584	1641.043	2435.359	2941.501	1751.191	1983.55	2494.494	2417.119	2931.169	2463.049	2483.852	2385.436	2060.658	2691.011	2842.036	2747.693	2659.346
ABLIM3	27510.78	35041.68	39925.38	35023.13	44952.81	40788.2	36682.4	35928.92	37815.45	41509.89	45451.79	29729.57	32244.84	34402.21	33181.78	35146.38	36673.45	33435.57	41972.31
ABO	1487.485	1832.715	1763.471	1414.477	2466.369	1581.514	1867.597	2404.892	2570.368	1759.04	1601.119	2076.617	1822.794	1880.055	1933.518	1711.562	1804.369	2107.869	1382.107
ABR	20315.51	16800.56	14113.76	19193.66	20223.78	20190.85	14466.66	15337.79	21364.87	17932.41	24554.11	22665.21	20556.55	20207.8	17722.44	23311.38	25445.92	27582.5	30781.9
ABRA	90.32531	189.0738	84.92636	624.615	197.1323	65.08756	146.145	71.30408	40.85507	79.73804	116.8612	66.24563	21.58977	127.5087	82.15249	231.671	234.0138	437.7742	362.1084
ABRACL	1370.254	1254.49	1594.617	2477.674	1767.546	1442.203	1351.841	1630.734	1404.247	1608.211	1431.249	1453.724	1210.055	1651.098	1724.224	3352.221	2567.993	2738.591	1495.938
ABRAXAS1	3001.875	2297.897	2255.045	2505.735	1893.799	1916.086	2299.235	2437.303	958.3432	1457.381	1273.426	2064.656	2072.618	2179.747	2191.711	2094.108	1952.168	2212.534	1629.488
ABRAXAS2	6725.392	6542.553	5657.095	9140.58	5610.519	6595.539	6924.044	6203.455	3555.558	5281.924	4235.918	5626.278	5741.851	6149.269	5996.154	7425.838	6489.777	6459.217	6206.036
ABT1	1276.085	1499.585	1166.988	1214.933	1564.876	1616.912	1004.322	1210.317	2813.163	2107.774	3432.347	1486.846	1347.819	1386.773	1321.286	1578.825	1557.013	1591.823	1466.36
ABT1P1	0	0	0	0	0	0	0	0	0	0	0	2.760235	0	0	0	0	1.026376	0	0
ABTB1	7057.866	5837.278	5834.941	3655.193	5448.826	6954.092	3883.039	5655.247	9925.448	7758.607	14285.98	6533.476	5748.02	7563.964	6571.221	5451.275	7341.669	5245.099	6107.442
ABTB2	2246.602	1803.704	1991.273	1347.963	1825.135	2220.97	1824.263	1462.197	1741.593	1700.438	2023.988	1972.648	1862.889	1829.796	1811.267	1798.954	1909.06	1884.886	2279.311
AC000032.	0	0	22.98007	12.47151	15.50479	0	4.248401	25.92876	0	67.24895	84.33284	0	0	0	0.978006	11.54233	17.4484	8.191201	1.792616
AC000036.	40.35812	40.01562	35.96881	42.611	11.07485	4.567548	60.3273	19.44657	28.01491	21.13538	46.98544	20.24172	19.5336	19.54513	40.09824	27.20691	34.89679	50.96747	69.01571
AC000058.	5.765445	3.001171	3.996535	1.039293	6.64491	5.709435	4.248401	1.852054	1.167288	4.803496	2.40951	5.520469	9.252759	16.75297	19.56012	5.771163	9.237386	4.550667	6.274156
AC000067.	3.84363	3.001171	1.998267	3.117878	2.21497	0	5.947762	5.556162	0	0.960699	24.0951	6.440548	0	13.9608	3.912023	4.122259	11.29014	7.281067	10.7557
AC000068.	50.9281	75.02928	99.91337	72.75049	56.48173	76.50643	134.2495	88.8986	72.37184	69.17035	53.00921	124.2106	110.005	90.27987	66.5044	80.79628	100.5849	94.65388	132.6536
AC000068.	8.648168	7.002733	15.98614	7.275049	24.36467	17.12831	12.7452	12.03835	4.669151	12.48909	7.228529	22.08188	19.5336	14.89152	10.75806	8.244518	15.39564	4.550667	10.7557
AC000078.	166.237	108.0422	101.9116	129.9116	99.67364	70.79699	87.51706	152.7945	51.36066	57.64196	19.27608	126.9708	165.5216	122.8551	138.8768	144.2791	188.8532	121.9579	165.817
AC000081.	0	0	0	0	0	1.141887	0	0	0	0	0	0	0	0	0	0	0	0.910133	0
AC000089.	16.33543	18.00703	10.99047	10.39293	31.00958	12.56076	14.44456	12.96438	7.003726	22.09608	16.86657	34.04289	50.37613	14.89152	22.49413	28.85581	31.81766	17.29253	18.82247
AC000095.	38.4363	61.02382	32.97141	50.92535	29.90209	60.52001	67.97442	41.67122	23.34575	28.82098	3.614265	30.36258	30.84253	49.32818	44.01026	39.57369	39.0023	29.12427	34.95601
AC000099.	0	2.000781	0	0	0	0	0	0	0	0	0	0	0	0	0	0	0	0	0
AC000120.	200.8297	231.0902	259.7748	287.8841	213.7446	158.7223	326.2772	242.6191	18.6766	129.6944	104.8137	235.54	272.4424	149.846	169.195	229.1976	181.6686	232.9942	286.8185
AC000123.	139.3316	183.0715	235.7955	247.3517	238.1093	205.5397	212.4201	239.841	124.8998	204.6289	178.3037	252.1014	300.2006	177.7676	179.9531	204.4641	211.4335	153.8125	171.1948
AC000123.	45.16265	76.02967	94.9177	90.41847	78.63143	108.4793	111.3081	123.1616	102.7213	93.18783	104.8137	126.0507	97.66802	91.21059	93.88856	100.5831	88.26836	98.29441	89.63079
AC000123.	561.17	401.1566	521.5478	596.554	632.3739	331.1472	841.1834	656.5532	191.4352	341.0482	246.9747	582.4095	531.5196	616.1368	681.6701	496.32	423.8934	796.3667	683.883
AC000124.	0.960908	4.001562	0.999134	1.039293	8.859879	2.283774	0	0	14.00745	0.960699	0	0.920078	3.084253	0.93072	0	1.648904	5.131881	0	0
AC000367.	0	0	0.999134	0	0	6.851322	3.398721	0	0	2.882098	0	0.920078	0	0	0.978006	3.297807	0	0	0
AC000372.	0	0	0	3.117878	0	1.141887	0	0.926027	0	0	0	0.920078	0	0.93072	0.978006	0.824452	0	0	0
AC000403.	472.7665	187.073	568.5071	245.2731	173.8751	260.3502	271.8977	232.4328	371.1975	201.7468	210.8321	337.6687	411.2337	325.7521	313.9399	200.3418	252.4886	315.8163	273.3739
AC001226.	27.86632	47.01835	54.95235	13.51081	31.00958	25.12151	18.69297	32.41095	56.02981	48.99566	59.03299	26.68227	39.06721	59.5661	49.8783	17.31349	15.39564	36.40534	36.74863
AC001226.	108.5826	86.03358	125.8908	163.169	129.5757	86.78341	157.1908	112.0493	82.87743	183.4936	59.03299	135.2515	111.0331	139.608	95.84457	86.56744	75.95184	76.45121	73.49725
AC002044.	2.882723	14.00547	14.987	3.117878	53.15928	25.12151	2.549041	11.11232	135.4054	42.27077	59.03299	11.96102	7.196591	17.68369	22.49413	18.13794	15.39564	16.3824	0.896308
AC002044.	14.41361	28.01093	31.97228	3.117878	52.05179	10.27698	6.797442	8.334243	136.5727	45.15286	81.92333	16.56141	9.252759	9.307203	29.34017	18.96239	11.29014	26.39387	6.274156
AC002044.	0.960908	0	15.98614	0	1.107485	5.709435	0.84968	22.22465	4.669151	27.86028	26.50461	1.840156	0	0.93072	0.978006	0	1.026376	4.550667	0.896308
AC002056.	9.609075	2.000781	0	0	9.967364	6.851322	2.549041	9.26027	7.003726	3.842797	0	1.840156	1.028084	0.93072	11.73607	15.66458	7.184634	0.910133	0.896308
AC002056.	12.4918	17.00664	11.9896	7.275049	5.537425	13.70264	18.69297	3.704108	2.334575	15.37119	38.55216	2.760235	11.30893	9.307203	10.75806	7.420066	11.29014	23.66347	25.09662
AC002059.	83.59896	77.03006	43.96188	203.7014	74.20149	75.36454	50.98081	92.6027	211.2791	152.7512	106.0184	103.0488	107.9489	116.34	140.8328	212.7086	232.9874	106.4856	86.94187
AC002064.	14.41361	27.01054	25.97748	11.43222	8.859879	4.567548	14.44456	13.89041	7.003726	8.646293	0	24.84211	25.70211	25.12945	19.56012	27.20691	15.39564	21.8432	1.792616
AC002064.	30.74904	56.02186	34.96968	76.90766	37.65449	15.98642	74.77186	32.41095	7.003726	31.70308	12.04755	62.56532	41.12337	41.88241	53.79032	46.99375	37.97592	91.92348	41.23017
AC002064.	0	0	0	0	0	1.141887	0	0	0	0	0	0	5.140422	0.93072	0	0.824452	0	0.910133	0.896308
AC002066.	73.98988	49.01913	62.94542	29.1002	16.61227	11.41887	24.64073	36.11505	91.04844	28.82098	22.89034	40.48344	34.95487	97.72563	82.15249	30.50472	28.73854	17.29253	9.859387
AC002069.	0	0	0	12.47151	0	0	0	0.926027	0	0	0	0	0	0	0	0.824452	0	0	0

AC002069.	0	0	0.999134	0	0	0	0	0	0	0	0	1.840156	1.028084	0.93072	0	0	4.105505	0	3.585232
AC002070.	171.0415	123.048	112.9021	165.2476	136.2206	183.8438	130.0011	101.863	253.3014	100.8734	131.3183	102.1287	101.7804	158.2225	136.9208	138.5079	151.9037	81.00187	129.0683
AC002075.	1.921815	6.002343	4.995668	3.117878	12.18233	9.135096	1.69936	7.408216	5.836439	10.56769	18.07132	8.280704	7.196591	13.9608	6.846041	6.595615	3.079129	12.74187	3.585232
AC002076.	0	0	0	0	0	2.283774	0	0	0	0	0	0	0	0	0	0	0	0	3.585232
AC002090.	10.56998	1.00039	0	0	0	0	7.647122	0	1.167288	0	7.228529	0	7.196591	0	0.978006	4.122259	2.052753	0	2.688924
AC002091.	94.16894	33.01288	7.993069	128.8723	31.00958	47.95925	36.53625	53.70957	37.35321	42.27077	21.68559	34.96297	29.81445	77.24979	44.98827	79.14738	24.63303	44.59654	28.68185
AC002091.	29.78813	6.002343	5.994802	36.37525	3.322455	23.97963	32.28785	29.63287	7.003726	2.882098	8.433284	25.76219	23.64594	13.9608	37.16422	42.04704	20.52753	26.39387	9.859387
AC002094.	8.648168	3.001171	25.97748	9.353635	3.322455	20.55397	22.09169	22.22465	18.6766	30.74238	19.27608	27.60235	7.196591	26.99089	4.890029	1.648904	13.34289	17.29253	14.34093
AC002094.	34.59267	16.00625	58.94889	29.1002	67.55658	19.41208	64.5757	14.81643	109.725	24.97818	9.638039	48.76415	51.40422	29.78305	52.81231	28.03136	28.73854	31.85467	25.99293
AC002115.	0.960908	1.00039	0.999134	0	4.42994	11.41887	4.248401	0	0	3.842797	0	0.920078	5.140422	7.445762	0	0	0	5.4608	15.23724
AC002115.	0	0	3.996535	0	0	0	0	0	0	0	0	0	0	0	0	0	0	0	0
AC002116.	23.06178	22.00859	4.995668	12.47151	8.859879	28.54718	12.7452	15.74246	33.85134	26.89958	38.55216	25.76219	15.42127	20.47585	16.6261	9.06897	23.60665	20.93307	14.34093
AC002127.	0	6.002343	1.998267	0	9.967364	0	6.797442	0	0	1.921399	0	0	0	6.515042	3.912023	1.648904	0	5.4608	0
AC002127.	24.02269	15.00586	28.97488	57.1611	31.00958	18.27019	16.9936	6.482189	17.50932	32.66377	9.638039	21.1618	33.92678	29.78305	28.36217	48.64266	17.4484	4.550667	23.30401
AC002128.	309.4122	190.0742	272.7635	203.7014	168.3377	206.6815	201.3742	184.2794	310.4985	324.7163	346.9694	184.9357	247.7683	290.3847	259.1715	243.2133	303.8074	330.3784	276.9592
AC002128.	64.3808	28.01093	58.94889	57.1611	40.97694	34.25661	42.48401	6.482189	15.17474	52.83846	15.66181	23.00196	43.17954	70.73474	80.19648	11.54233	29.76491	40.04587	51.08955
AC002306.	0.960908	5.001952	0.999134	1.039293	5.537425	4.567548	0.84968	7.408216	1.167288	1.921399	16.86657	8.280704	0	0	0	0.824452	0	0.910133	0.896308
AC002310.	88.40349	104.0406	126.89	77.94696	138.4356	149.5872	76.47122	123.1616	117.8961	124.8909	199.9893	103.9688	101.7804	136.8159	93.88856	117.0722	105.7168	112.8565	70.80833
AC002310.	5.765445	0	2.997401	1.039293	0	4.567548	2.549041	0	7.003726	1.921399	2.40951	4.600391	0	0.93072	0.978006	1.648904	6.158258	0	0
AC002310.	1.921815	1.00039	14.987	11.43222	0	5.709435	5.098081	18.52054	4.669151	16.33189	8.433284	8.280704	8.224675	14.89152	10.75806	11.54233	3.079129	4.550667	15.23724
AC002310.	79.75532	71.02772	57.94975	78.98625	67.55658	74.22266	87.51706	67.59997	162.253	88.38433	133.7278	71.7661	76.07824	54.9125	84.1085	65.13169	88.26836	79.18161	87.83818
AC002347.	5.765445	10.0039	15.98614	7.275049	39.86946	13.70264	6.797442	19.44657	24.51304	26.89958	25.29985	26.68227	11.30893	17.68369	12.71408	5.771163	5.131881	31.85467	21.51139
AC002347.	16.33543	10.0039	9.991337	2.078586	5.537425	1.141887	5.947762	22.22465	10.50559	2.882098	0	13.80117	15.42127	5.584322	7.824047	8.244518	7.184634	8.191201	5.377848
AC002350.	17.29634	14.00547	8.992203	3.117878	27.68712	4.567548	12.7452	6.482189	16.34203	19.21399	10.84279	0.920078	3.084253	2.792161	13.69208	18.96239	14.36927	22.75334	4.48154
AC002350.	41.31902	67.02616	79.93069	48.84676	34.33203	45.67548	42.48401	37.96711	102.7213	69.17035	67.46627	48.76415	28.78636	64.2197	53.79032	85.74299	76.97822	70.99041	54.67478
AC002351.	0	10.0039	0	14.5501	0	4.567548	3.398721	0	21.01118	0	0	0.920078	8.224675	1.861441	5.868035	5.771163	1.026376	2.7304	9.859387
AC002366.	0	0	0.999134	0	4.42994	0	8.496802	2.778081	0	0.960699	9.638039	1.840156	0	0	0.978006	3.297807	0	0	0.896308
AC002367.	0	1.00039	0	0	0	0	0.84968	3.704108	0	0	0	0	0	0	0	0	0	0	0.896308
AC002368.	5.765445	7.002733	5.994802	7.275049	13.28982	2.283774	4.248401	6.482189	0	0.960699	1.204755	2.760235	1.028084	7.445762	11.73607	2.473355	11.29014	15.47227	7.170464
AC002375.	0	0	0	0	0	0	0	0	1.167288	0	0	0	0	2.792161	0.978006	0	0	0.910133	0.896308
AC002378.	0	0	0	0	0	0	3.398721	0	0	0	0	0	0	0	0	0	0	0	0
AC002381.	0.960908	0	0	0	0	0	0	0	4.669151	0	0	0	0	0	0	0	0	0	0
AC002383.	3.84363	22.00859	11.9896	15.58939	12.18233	6.851322	20.39233	7.408216	9.338302	15.37119	14.45706	10.12086	11.30893	9.307203	2.934017	4.946711	6.158258	1.820267	17.92616
AC002384.	0	0	0	0	0	0	0	0	0	0	0	0	0	0	0	1.648904	0	0	0
AC002384.	1.921815	1.00039	2.997401	0	0	1.141887	0.84968	0	1.167288	1.921399	6.023774	2.760235	1.028084	0	1.956012	3.297807	0	0.910133	3.585232
AC002398.	11.53089	26.01015	29.97401	13.51081	52.05179	34.25661	11.04584	40.74519	124.8998	68.20965	42.16642	12.8811	24.67402	29.78305	15.64809	17.31349	22.58028	22.75334	17.92616
AC002400.	21.13997	5.001952	19.98267	24.94303	4.42994	7.993209	21.24201	38.89314	0	3.842797	4.81902	7.360626	3.084253	0	12.71408	11.54233	17.4484	8.191201	10.7557
AC002401.	1.921815	1.00039	3.996535	1.039293	0	5.709435	5.947762	6.482189	0	16.33189	13.2523	0.920078	0	2.792161	0	14.01568	1.026376	0	9.859387
AC002401.	30.74904	21.0082	66.94196	11.43222	36.547	20.55397	34.83689	40.74519	8.171014	31.70308	33.73314	23.92203	26.73019	39.09025	24.45015	9.06897	5.131881	24.5736	35.85232
AC002401.	41.31902	33.01288	59.94802	14.5501	33.22455	41.10793	61.17698	44.4493	88.71387	45.15286	51.80446	48.76415	50.37613	52.12034	53.79032	15.66458	28.73854	28.21414	23.30401
AC002401.	76.8726	187.073	116.8986	179.7976	151.7254	216.9585	72.22282	109.2712	175.0932	225.7643	239.7462	108.5692	86.35909	140.5388	125.1847	170.6615	135.4817	47.32694	124.5868
AC002407.	0.960908	4.001562	6.993936	0	1.107485	4.567548	0.84968	3.704108	5.836439	8.646293	16.86657	0	1.028084	1.861441	0.978006	4.946711	4.105505	0.910133	0.896308
AC002428.	7.68726	4.001562	5.994802	3.117878	0	1.141887	0	0.926027	0	0	1.204755	0.920078	1.028084	1.861441	9.780058	0	0	0	0
AC002428.	87.44259	79.03084	101.9116	77.94696	26.57964	31.97284	28.03945	57.41368	282.4836	116.2446	81.92333	90.16767	75.05016	62.35826	51.83431	48.64266	21.5539	42.77627	30.47447
AC002429.	11.53089	7.002733	22.98007	34.29666	27.68712	12.56076	5.098081	23.15068	21.01118	13.44979	18.07132	32.20274	27.75828	4.653602	20.53812	3.297807	2.052753	10.01147	17.92616
AC002451.	112.4262	321.1253	161.8597	28.0609	117.3934	147.3034	201.3742	136.126	80.54285	69.17035	42.16642	112.2495	106.9208	136.8159	163.327	86.56744	111.875	222.0726	196.2914
AC002451.	0.960908	0	0	7.275049	0	0	1.69936	7.408216	0	0	4.81902	0	0	0	1.956012	4.122259	0	3.640534	0
AC002454.	3.84363	9.003514	5.994802	6.235757	14.3973	13.70264	6.797442	19.44657	4.669151	8.646293	7.228529	14.72125	13.3651	3.722881	4.890029	4.122259	16.42202	11.83173	8.066772
AC002456.	138.3707	398.1554	116.8986	197.4656	98.56616	166.7155	103.661	151.8684	245.1304	118.166	122.885	95.68814	90.47142	116.34	111.4927	176.4327	251.4622	147.4416	121.0016
AC002456.	0	1.00039	0	0	0	0	0	0	0	0	0	0	0	0	1.956012	0	2.052753	0	0
AC002460.	0	0	0	0	0	0	0	0	0	0	0	0	0	0	5.868035	0	0	0	0
AC002463.	5.765445	0	6.993936	1.039293	2.21497	0	1.69936	0	0	6.724895	0	0.920078	5.140422	1.861441	1.956012	3.297807	0	0.910133	0.896308
AC002464.	10.56998	0	5.994802	21.82515	0	13.70264	11.89552	0	2.334575	26.89958	0	3.680313	6.168506	29.78305	8.802052	11.54233	5.131881	26.39387	5.377848
AC002467.	183.5333	203.0793	235.7955	119.5187	197.1323	215.8166	113.0075	213.9122	329.1751	249.7818	212.0369	191.3763	153.1846	201.0356	195.6012	183.8528	257.6204	164.7341	189.121
AC002470.	37.47539	38.01484	40.96448	43.6503	58.6967	22.83774	27.18977	3.704108	96.88488	54.75986	16.86657	27.60235	47.29188	26.99089	73.35044	61.83389	35.92317	31.85467	61.84525
AC002472.	6.726353	4.001562	1.998267	5.196464	8.859879	4.567548	14.44456	0	4.669151	1.921399	0	2.760235	4.112337	29.78305	12.71408	5.771163	5.131881	7.281067	3.585232
AC002472.	8.648168	3.001171	9.991337	6.235757	6.64491	1.141887	2.549041	6.482189	0	9.606993	2.40951	2.760235	14.39318	4.653602	11.73607	6.595615	9.237386	12.74187	7.170464
AC002480.	3.84363	2.000781	2.997401	1.039293	0	2.283774	0.84968	0	0	0	0	2.760235	0	0	0	2.473355	0	0.910133	0.896308
AC002480.	178.7288	123.048	105.9082	75.86837	54.26676	62.80379	48.43177	75.93422	60.69896	37.46727	42.16642	69.00587	57.57272	42.81313	53.79032	47.81821	42.08143	59.15867	50.19325
AC002480.	37.47539	28.01093	6.993936	9.353635	5.537425	10.27698	11.89552	8.334243	11.67288	0.960699	2.40951	6.440548	9.252759	10.23792	3.912023	12.36678	8.21101	29.12427	18.82247
AC002480.	0	0	0	0	0	0	0.84968	0	0	0	0	1.840156	7.196591	5.584322	0	4.122259	0	0	1.792616
AC002511.	0.960908	1.00039	0	0	0	6.851322	5.098081	5.556162	11.67288	1.921399	0	6.440548	12.33701	7.445762	6.846041	12.36678	7.184634	60.06881	27.78555

AC002511.	3.84363	1.00039	3.996535	6.235757	0	1.141887	0.84968	18.52054	1.167288	3.842797	6.023774	4.600391	14.39318	3.722881	0.978006	3.297807	12.31652	39.13574	21.51139
AC002519.	28.82723	22.00859	36.96795	77.94696	17.71976	20.55397	19.54265	20.37259	7.003726	58.60265	42.16642	20.24172	11.30893	26.99089	29.34017	55.23827	55.42432	35.4952	37.64493
AC002519.	4.804538	1.00039	4.995668	1.039293	0	39.96605	32.28785	3.704108	0	5.764196	9.638039	2.760235	3.084253	13.9608	30.31818	9.06897	7.184634	1.820267	1.792616
AC002524.	9.609075	6.002343	4.995668	7.275049	11.07485	2.283774	5.098081	0.926027	4.669151	6.724895	6.023774	9.200782	6.168506	7.445762	6.846041	6.595615	8.21101	9.101334	6.274156
AC002525.	36.51449	66.02577	45.96015	72.75049	43.19191	43.39171	13.59488	41.67122	37.35321	33.62447	26.50461	28.52243	34.95487	69.80402	42.05425	21.43575	25.65941	15.47227	11.652
AC002540.	17.29634	69.02694	56.95062	11.43222	27.68712	46.81737	32.28785	10.1863	46.69151	17.29259	4.81902	27.60235	24.67402	20.47585	15.64809	42.04704	57.47707	13.652	26.88924
AC002542.	2.882723	0	1.998267	0	2.21497	1.141887	3.398721	0	5.836439	0	0	0.920078	3.084253	0.93072	7.824047	4.122259	0	0	0
AC002543.	0	1.00039	0	4.157171	0	0	0	5.556162	8.171014	0	0	0	1.028084	5.584322	0.978006	6.595615	5.131881	6.370934	0
AC002546.	1402.925	1469.574	1780.456	2156.532	1264.748	2046.262	1734.197	1639.068	1820.969	2416.159	2120.369	1252.226	1344.734	2072.714	2265.061	1540.9	1481.061	1348.818	855.0778
AC002546.	1000.305	1938.757	2848.53	2389.334	1992.365	2194.707	2560.087	2599.358	1321.37	1291.18	873.4473	1067.291	1015.747	3195.163	3142.333	1888.819	1534.432	2714.018	1290.683
AC002550.	3.84363	1.00039	0	0	0	0	0.84968	0	0	0	0	0	1.028084	0	0	0.824452	1.026376	0.910133	0.896308
AC002550.	173.9243	199.0777	233.7973	168.3654	186.0575	107.3374	227.7143	233.3588	110.8923	193.1005	114.4517	194.1365	217.9539	195.4513	210.2713	155.8214	131.3762	139.2504	97.69757
AC002551.	0	2.000781	0	1.039293	1.107485	1.141887	0	3.704108	16.34203	9.606993	0	1.840156	1.028084	0	1.956012	0	0	0.910133	0.896308
AC002553.	340.1613	307.1199	398.6543	190.1906	252.5066	251.2151	294.839	298.1807	289.4874	360.2622	385.5216	343.1892	389.644	328.5443	371.6422	253.9312	206.3016	319.4568	246.4847
AC002553.	0	0	0.999134	1.039293	6.64491	1.141887	5.947762	0	0	0	0	0.920078	0	0	1.956012	0.824452	1.026376	0	0
AC002553.	149.9016	139.0543	153.8666	172.5226	166.1227	166.7155	194.5768	162.0547	58.36439	138.3407	78.30907	150.8928	186.0833	169.3911	188.7551	201.9907	131.3762	151.0821	131.7573
AC002558.	4.804538	5.001952	9.991337	10.39293	0	13.70264	5.098081	5.556162	0	5.764196	2.40951	6.440548	6.168506	13.03008	0.978006	16.48904	12.31652	6.370934	6.274156
AC002558.	4.804538	5.001952	14.987	3.117878	15.50479	2.283774	13.59488	4.630135	0	4.803496	0	8.280704	9.252759	10.23792	9.780058	4.122259	8.21101	10.9216	5.377848
AC002558.	8.648168	5.001952	9.991337	10.39293	4.42994	0	5.947762	12.03835	0	0	10.84279	11.96102	2.056169	2.792161	2.934017	1.648904	6.158258	3.640534	0.896308
AC002563.	45.16265	53.02069	66.94196	31.17878	49.83682	42.24982	63.72602	60.19176	19.84389	88.38433	66.26152	63.4854	62.71315	48.39746	62.59237	21.43575	33.87042	42.77627	34.95601
AC002984.	4.804538	17.00664	6.993936	47.80747	11.07485	13.70264	12.7452	22.22465	5.836439	15.37119	14.45706	4.600391	20.56169	14.89152	13.69208	17.31349	27.71216	10.9216	3.585232
AC002985.	42.27993	17.00664	20.98181	37.41454	46.51437	20.55397	33.13753	11.11232	38.5205	49.95636	45.78069	31.28266	20.56169	21.40657	19.56012	45.34485	34.89679	58.24854	14.34093
AC003001.	0	9.003514	1.998267	3.117878	0	0	2.549041	6.482189	0	0	1.204755	4.600391	1.028084	0	1.956012	0	0	5.4608	2.688924
AC003001.	0.960908	0	0	0	0	0	0.84968	0	0	0	0	0	0	1.861441	0	0	0	0.910133	0
AC003002.	735.0943	1002.391	573.5027	665.1474	635.6963	767.3481	615.1685	613.9559	1093.749	805.066	681.8913	570.4485	563.3902	718.5161	579.9575	730.4643	939.1343	789.9958	711.6685
AC003002.	35.55358	57.02225	78.93156	43.6503	56.48173	45.67548	45.03305	23.15068	35.01863	45.15286	19.27608	37.72321	55.51656	37.22881	36.18622	48.64266	37.97592	35.4952	43.91909
AC003002.	25.9445	45.01757	33.97054	23.90373	35.43952	12.56076	44.18337	16.66849	8.171014	13.44979	15.66181	21.1618	16.44935	27.92161	10.75806	44.5204	42.08143	15.47227	37.64493
AC003005.	42.27993	19.00742	44.96101	15.58939	17.71976	26.2634	45.03305	25.00273	2.334575	18.25329	21.68559	55.20469	16.44935	8.376483	10.75806	14.01568	2.052753	17.29253	29.57816
AC003009.	0	0	0	2.078586	1.107485	0	0	0	0	0	0	0.920078	0	0	1.956012	0	0	0	0
AC003029.	15.37452	13.00508	18.98354	5.196464	12.18233	3.425661	12.7452	4.630135	19.84389	13.44979	24.0951	19.32164	21.58977	5.584322	4.890029	0.824452	12.31652	4.550667	7.170464
AC003035.	30.74904	26.01015	45.96015	7.275049	36.547	43.39171	36.53625	42.59724	80.54285	21.13538	18.07132	27.60235	33.92678	17.68369	43.03226	27.20691	25.65941	80.09174	47.50432
AC003035.	18.25724	16.00625	45.96015	11.43222	26.57964	28.54718	18.69297	10.1863	51.36066	30.74238	50.5997	27.60235	16.44935	26.99089	31.29619	21.43575	17.4484	67.34987	48.40063
AC003043.	7.68726	0	0.999134	3.117878	4.42994	0	1.69936	1.852054	0	0	1.204755	0	0	0.93072	0	0.824452	0	0	0
AC003070.	186.4161	121.0472	108.9056	170.444	86.38382	125.6076	141.0469	219.4684	269.6435	150.8298	144.5706	192.2964	150.1003	167.5297	145.7229	200.3418	225.8028	378.6155	288.6112
AC003070.	0.960908	0	0	1.039293	0	0	0	0	0	0	0	0.920078	0	0	0	0	0	0	0
AC003071.	1.921815	0	0.999134	1.039293	2.21497	2.283774	0	0	0	2.882098	1.204755	0	1.028084	0.93072	1.956012	1.648904	3.079129	2.7304	0
AC003072.	93.20803	118.0461	101.9116	90.41847	183.8425	227.2355	107.0597	119.4575	436.5656	293.974	531.2969	131.5712	130.5667	135.8852	112.4707	137.6835	148.8246	93.74374	103.0754
AC003075.	48.04538	34.01327	96.91597	59.23969	90.81376	83.35775	50.13113	25.92876	29.18219	42.27077	44.57593	39.56336	62.71315	53.05106	49.8783	37.10033	24.63303	70.08027	62.74156
AC003080.	0	2.000781	3.996535	18.70727	0	5.709435	5.947762	2.778081	12.84017	0.960699	36.14265	1.840156	13.3651	0	0.978006	0	0	0.910133	4.48154
AC003084.	0	0	7.993069	2.078586	8.859879	4.567548	0.84968	0.926027	0	3.842797	6.023774	5.520469	0	0	0.978006	0	0	6.370934	4.48154
AC003087.	0	0	0	0	0	0	0	0	0	0	0	0.920078	0	0	0	0	0	0.910133	0
AC003092.	0	10.0039	0	0	0	0	0.84968	1.852054	7.003726	0	0	0	0	0	1.956012	0.824452	0	0	0
AC003092.	0	0	0	4.157171	0	3.425661	0	0	1.167288	0	4.81902	3.680313	2.056169	0	0	4.946711	0	1.820267	1.792616
AC003098.	0	4.001562	2.997401	1.039293	1.107485	0	1.69936	0	8.171014	10.56769	0	1.840156	0	0.93072	0	0	0	3.640534	0
AC003101.	0.960908	0	0	0	0	0	0.84968	0	0	0	0	4.600391	0	0.93072	0.978006	0	0	0	0
AC003101.	2.882723	1.00039	0.999134	2.078586	6.64491	0	0	0	0	2.882098	9.638039	0.920078	1.028084	3.722881	3.912023	0.824452	2.052753	1.820267	0
AC003102.	472.7665	418.1632	654.4325	440.6601	517.1955	395.0929	423.9904	493.5724	939.6666	564.8912	713.2149	573.2087	458.5256	616.1368	506.607	484.7777	495.7397	698.0723	451.7392
AC003102.	0	3.001171	0	1.039293	0	3.425661	8.496802	0.926027	0	0	8.433284	0.920078	1.028084	0.93072	4.890029	3.297807	4.105505	2.7304	0
AC003659.	0	0	0	0	0	0	0	6.482189	0	0	0	0	0	0	0	0	0	0	0.896308
AC003666.	0	0	0	5.196464	4.42994	9.135096	3.398721	1.852054	3.501863	1.921399	0	4.600391	10.28084	0.93072	0.978006	1.648904	0	2.7304	0.896308
AC003669.	6.726353	5.001952	4.995668	1.039293	8.859879	14.84453	7.647122	8.334243	37.35321	8.646293	26.50461	11.04094	13.3651	5.584322	3.912023	6.595615	29.76491	11.83173	7.170464
AC003681.	1618.168	1480.578	1876.373	1180.637	2200.573	1225.245	1789.427	2030.777	937.332	1375.721	1409.563	2475.01	2517.779	1497.529	1507.107	1794.007	1515.958	1497.169	1400.033
AC003682.	19.21815	39.01523	34.96968	41.57171	23.25718	12.56076	31.43817	47.22738	0	16.33189	6.023774	38.64329	32.8987	17.68369	25.42815	22.2602	18.47477	24.5736	28.68185
AC003684.	0	5.001952	0	0	0	1.141887	0.84968	0.926027	1.167288	1.921399	4.81902	1.840156	1.028084	7.445762	8.802052	3.297807	0	8.191201	8.963079
AC003686.	0	0	0	6.235757	0	0	0	0	0	0	0	0	0	0	0	0	0	0	0
AC003687.	0	0	0	0	0	0	0	5.556162	0	0	0	0	1.028084	0	0	0	0	0	0
AC003688.	0.960908	1.00039	0	1.039293	0	1.141887	0	0	1.167288	0	3.614265	1.840156	2.056169	0	0	1.648904	0	2.7304	0.896308
AC003956.	18.25724	39.01523	32.97141	25.98232	17.71976	11.41887	39.08529	38.89314	24.51304	41.31007	42.16642	20.24172	28.78636	29.78305	34.2302	40.39814	27.71216	17.29253	26.88924
AC003957.	19.21815	20.00781	25.97748	28.0609	6.64491	21.69585	11.89552	9.26027	16.34203	38.42797	19.27608	77.28657	44.20763	12.09936	18.58211	49.46711	74.92547	63.70934	77.08248
AC003958.	0	0	0	0	0	0	0	0	4.669151	0	0	0	0	0	0	0	0	0	0
AC003958.	0	0	2.997401	0	4.42994	0	0	0	0	1.921399	0	0.920078	0	1.861441	0	0	2.052753	0	3.585232
AC003965.	31.70995	39.01523	35.96881	19.74656	21.04221	3.425661	12.7452	62.04381	95.71759	23.05678	1.204755	27.60235	18.50552	37.22881	63.57038	47.81821	64.6617	57.33841	34.0597

AC003965.	0	0	0	0	0	0	0	0	0	0	0	1.840156	0	0	0	0	0	0	0
AC003973.	0	0	0	0	0	0	0	0	0	0	0	0.920078	0	0	0	1.648904	0	0	0.896308
AC003973.	0.960908	5.001952	0	1.039293	1.107485	2.283774	2.549041	0.926027	8.171014	0.960699	7.228529	2.760235	0	14.89152	10.75806	5.771163	1.026376	1.820267	5.377848
AC003973.	439.1347	345.1347	414.6405	387.6562	486.1859	424.782	525.1024	261.1396	498.4319	365.0657	280.7079	565.8481	504.7894	722.239	694.3841	563.1006	502.9244	455.9768	338.8044
AC003975.	0	1.00039	0.999134	0	0	0	0	0	0	0.960699	2.40951	0	0	0	0	0	1.026376	0	2.688924
AC003982.	0	4.001562	12.98874	4.157171	1.107485	1.141887	10.19616	0.926027	15.17474	21.13538	24.0951	0.920078	4.112337	1.861441	2.934017	1.648904	0	10.01147	4.48154
AC003985.	0	0	0	5.196464	0	0	0.84968	0	7.003726	0	3.614265	0	1.028084	0	0	6.595615	3.079129	0	0.896308
AC003986.	9.609075	10.0039	0	19.74656	0	18.27019	7.647122	28.70684	0	3.842797	2.40951	12.8811	1.028084	18.61441	13.69208	28.85581	14.36927	0.910133	25.09662
AC003986.	46.12356	19.00742	12.98874	11.43222	28.79461	12.56076	16.9936	9.26027	16.34203	23.05678	26.50461	19.32164	53.46039	9.307203	6.846041	79.97183	60.5562	37.31547	9.859387
AC003986.	10.56998	6.002343	0.999134	15.58939	1.107485	0	0.84968	15.74246	7.003726	3.842797	1.204755	0.920078	5.140422	2.792161	10.75806	18.13794	2.052753	0	0.896308
AC003988.	0	0	1.998267	0	0	0	0	0	0	0	0	2.760235	0	0	1.956012	0	1.026376	0	0
AC003989.	0	1.00039	3.996535	0	0	0	0	0.926027	0	0.960699	0	0	2.056169	0.93072	4.890029	4.946711	1.026376	0.910133	5.377848
AC003989.	0	6.002343	1.998267	2.078586	1.107485	1.141887	0.84968	0	0	0	0	0	0	2.792161	9.780058	2.473355	0	5.4608	0
AC003991.	69.18534	65.02538	93.91856	256.7053	107.426	55.95246	80.71962	90.75065	25.68033	70.13105	27.70936	172.0546	119.2578	159.1532	194.6232	762.6179	719.4898	729.0169	243.7958
AC003991.	15.37452	29.01132	6.993936	45.72888	23.25718	19.41208	38.23561	45.37532	3.501863	7.685594	15.66181	23.92203	14.39318	19.54513	30.31818	20.6113	19.50115	30.0344	38.54124
AC003992.	0.960908	0	12.98874	0	5.537425	4.567548	0.84968	0	0	7.685594	0	3.680313	8.224675	7.445762	5.868035	5.771163	0	4.550667	0.896308
AC004000.	44.20175	51.01991	54.95235	33.25737	198.2398	49.10114	22.09169	45.37532	486.759	129.6944	246.9747	29.4425	31.87062	69.80402	58.68035	31.32917	31.81766	38.2256	14.34093
AC004004.	0.960908	0	0	0	2.21497	9.135096	0.84968	6.482189	5.836439	0	0	3.680313	1.028084	0	4.890029	0	0	0.910133	0
AC004006.	0	1.00039	0	0	0	0	0	0	0	0	0	0	1.028084	0	0	0	0	0	0
AC004009.	0	0	0.999134	0	0	0	0	0	0	0	0	0	0	0	0	0	0	0.910133	0
AC004009.	0	0	0	0	0	0	0	3.704108	0	0	0	0	0	0	3.912023	0	0	0	0
AC004012.	4.804538	4.001562	19.98267	7.275049	5.537425	9.135096	5.947762	9.26027	14.00745	10.56769	22.89034	8.280704	3.084253	13.9608	24.45015	8.244518	7.184634	15.47227	9.859387
AC004014.	0	0	0	0	0	0	0	0	0	0	0	0	0	0	0	0	4.105505	1.820267	0
AC004022.	0.960908	1.00039	4.995668	1.039293	8.859879	2.283774	0	7.408216	0	0.960699	0	3.680313	1.028084	1.861441	0.978006	3.297807	1.026376	8.191201	1.792616
AC004022.	0.960908	1.00039	5.994802	1.039293	2.21497	0	0.84968	0	0	0	1.204755	8.280704	0	0	0	0	1.026376	0	0.896308
AC004023.	36.51449	25.00976	6.993936	46.76817	23.25718	28.54718	35.68657	25.00273	26.84762	14.41049	2.40951	29.4425	22.61786	27.92161	24.45015	28.85581	30.79129	21.8432	21.51139
AC004024.	7.68726	9.003514	10.99047	10.39293	12.18233	11.41887	22.09169	7.408216	3.501863	1.921399	10.84279	17.48149	19.5336	6.515042	10.75806	13.19123	8.21101	9.101334	22.4077
AC004034.	71.10716	36.01406	48.95755	92.49706	49.83682	55.95246	87.51706	71.30408	138.9072	115.2839	60.23774	65.32555	54.48847	66.08114	50.8563	61.00944	66.71446	63.70934	83.35664
AC004039.	92.24712	67.02616	101.9116	78.98625	86.38382	73.08077	84.96802	85.19449	37.35321	65.32755	33.73314	107.6492	87.38717	76.31906	73.35044	99.75867	80.05735	57.33841	75.28987
AC004052.	0	2.000781	0	0	0	0	0	0	0	0	0	1.840156	0	0	0	0	0	0	0
AC004053.	1.921815	4.001562	0.999134	0	1.107485	0	0.84968	3.704108	0	0	0	2.760235	1.028084	0	0.978006	0	0	0	1.792616
AC004054.	0	0	0	0	0	0	0.84968	0	0	0	0	0	1.028084	1.861441	0	0.824452	2.052753	0	0.896308
AC004057.	232.5396	229.0894	218.8103	219.2908	181.6275	141.594	124.903	250.9533	204.2754	118.166	228.9034	173.8948	158.325	290.3847	179.9531	242.3888	219.6445	128.3288	139.824
AC004063.	3.84363	1.00039	0	0	0	0	0	0	0	0	0	0	2.056169	0	0	0	2.052753	0	0
AC004066.	25.9445	3.001171	8.992203	12.47151	0	9.135096	22.09169	0	7.003726	0	0	9.200782	6.168506	3.722881	7.824047	34.62698	21.5539	6.370934	11.652
AC004067.	151.8234	122.0476	112.9021	151.7367	140.6506	103.9117	67.12474	128.7178	182.0969	149.8691	171.0752	134.3314	135.7071	88.41843	117.3607	179.7305	158.0619	138.3403	109.3496
AC004069.	82.63805	70.02733	76.93329	98.73281	96.35119	99.34417	39.93497	58.3397	172.7586	86.46293	124.0898	66.24563	81.21867	82.83411	80.19648	58.53608	72.87271	93.74374	96.80126
AC004069.	0	0	0	0	0	0	0	0	0	0.960699	0	0	0	0	0	0.824452	0	0	0
AC004076.	16.33543	17.00664	13.98787	27.02161	11.07485	6.851322	9.346483	20.37259	21.01118	15.37119	30.11887	11.96102	17.47743	26.99089	22.49413	18.13794	25.65941	18.20267	12.54831
AC004076.	330.5522	395.1542	419.6361	311.7878	325.6006	477.3088	393.402	343.556	353.6882	461.1356	344.5599	397.4738	393.7563	265.2553	257.2155	248.9845	254.5413	278.5008	266.2035
AC004080.	1.921815	0	1.998267	0	0	1.141887	0	7.408216	2.334575	0	0	1.840156	4.112337	0	0	0	0	3.640534	0
AC004080.	0	7.002733	11.9896	14.5501	4.42994	6.851322	3.398721	3.704108	15.17474	9.606993	16.86657	9.200782	8.224675	4.653602	15.64809	4.122259	8.21101	12.74187	0.896308
AC004112.	26.90541	52.0203	65.94282	46.76817	55.37425	43.39171	71.37314	70.37805	7.003726	37.46727	15.66181	49.68422	51.40422	30.71377	46.94428	53.58937	32.84404	25.48374	33.16339
AC004129.	0	0	0	0	1.107485	0	0	0	0	0	0	0	0	0.93072	0	0	0	0	0
AC004129.	0	5.001952	0	0	0	0	2.549041	0	2.334575	0	0	0.920078	4.112337	0	0.978006	0	0	0	0.896308
AC004130.	96.09075	60.02343	97.9151	53.00393	85.27634	50.24303	34.83689	50.00546	87.54658	100.8734	103.6089	48.76415	27.75828	95.86419	91.93255	124.4922	88.26836	27.304	31.37078
AC004134.	0	0	0.999134	0	0	0	0.84968	0.926027	0	0	16.86657	1.840156	0	0.93072	1.956012	0	0	0.910133	0
AC004147.	0	4.001562	0	0	0	0	9.346483	0	1.167288	1.921399	0	0.920078	3.084253	5.584322	0	0	0	0	0
AC004148.	3.84363	0	0	0	0	2.283774	0	0	0	0	0	0	0	0	0	0	0	0.910133	0
AC004148.	1681.588	1548.604	1831.412	1723.147	1760.901	2029.133	1780.08	1953.917	2565.698	2477.643	2639.618	1562.293	1742.603	1741.378	1745.74	1308.405	1444.111	1876.695	1429.611
AC004149.	1.921815	10.0039	0	13.51081	3.322455	5.709435	11.89552	3.704108	5.836439	0	4.81902	8.280704	21.58977	9.307203	2.934017	0.824452	0	4.550667	2.688924
AC004156.	205.6342	241.0941	142.8761	250.4696	167.2302	149.5872	155.4915	112.0493	349.019	219.0394	213.2416	165.6141	142.9037	250.3638	162.349	199.5173	175.5103	197.499	283.2333
AC004158.	18.25724	3.001171	22.98007	23.90373	3.322455	6.851322	5.098081	11.11232	0	20.17468	15.66181	30.36258	48.31997	40.02097	31.29619	19.78684	14.36927	12.74187	17.92616
AC004160.	62.45899	112.0437	149.87	109.1257	101.8886	49.10114	106.21	112.9753	25.68033	71.09174	61.4425	154.5731	117.2016	76.31906	127.1408	140.9813	98.53212	140.1605	114.7274
AC004160.	0.960908	2.000781	3.996535	5.196464	0	3.425661	0.84968	2.778081	0	0.960699	8.433284	9.200782	11.30893	0	1.956012	21.43575	5.131881	4.550667	2.688924
AC004217.	3.84363	4.001562	5.994802	6.235757	4.42994	2.283774	10.19616	1.852054	1.167288	8.646293	7.228529	0	0	0.93072	5.868035	4.946711	1.026376	2.7304	0
AC004217.	1.921815	0	2.997401	0	1.107485	0	0	0.926027	0	0	0	0	0	0	0.978006	0	0	0	0
AC004217.	0	0	0.999134	0	0	0	0.84968	0	0	0	0	0	1.028084	0	0.978006	0	1.026376	0	3.585232
AC004221.	22.10087	48.01874	36.96795	73.78979	66.4491	59.37812	44.18337	25.00273	51.36066	49.95636	59.03299	48.76415	49.34805	48.39746	35.20821	60.18498	46.18693	67.34987	107.557
AC004223.	13.45271	9.003514	4.995668	5.196464	5.537425	2.283774	5.947762	23.15068	1.167288	9.606993	0	3.680313	18.50552	3.722881	6.846041	4.122259	0	7.281067	6.274156
AC004223.	17.29634	4.001562	7.993069	5.196464	11.07485	22.83774	6.797442	14.81643	17.50932	2.882098	21.68559	22.08188	16.44935	14.89152	22.49413	6.595615	13.34289	0.910133	7.170464
AC004223.	0	0	0	0	0	0	0.84968	0	0	0	0	0	0	0	0	0	2.052753	0	0

AC004223.	30.74904	15.00586	21.98094	16.62868	31.00958	35.3985	27.18977	21.29862	19.84389	10.56769	12.04755	22.08188	18.50552	13.9608	27.38416	31.32917	34.89679	20.02294	22.4077
AC004224.	11.53089	4.001562	5.994802	2.078586	1.107485	10.27698	3.398721	0.926027	11.67288	8.646293	2.40951	9.200782	1.028084	6.515042	10.75806	3.297807	11.29014	1.820267	7.170464
AC004231.	6.726353	22.00859	16.98527	19.74656	14.3973	5.709435	10.19616	9.26027	4.669151	31.70308	6.023774	3.680313	12.33701	2.792161	17.6041	6.595615	7.184634	4.550667	11.652
AC004231.	2.882723	1.00039	0.999134	6.235757	6.64491	0	5.947762	5.556162	0	3.842797	0	0	1.028084	0.93072	0	0	0	1.820267	0
AC004232.	21.13997	51.01991	49.95668	49.88605	38.76197	57.09435	33.13753	50.00546	79.37557	52.83846	91.56137	40.48344	30.84253	43.74385	46.94428	22.2602	26.68578	42.77627	42.12647
AC004233.	34.59267	23.00898	12.98874	29.1002	4.42994	26.2634	30.58849	22.22465	9.338302	29.78168	66.26152	41.40352	46.2638	25.12945	22.49413	46.99375	50.29244	28.21414	24.20031
AC004233.	19.21815	4.001562	9.991337	11.43222	1.107485	3.425661	21.24201	18.52054	0	5.764196	8.433284	10.12086	6.168506	7.445762	8.802052	13.19123	14.36927	9.101334	9.859387
AC004233.	104.7389	42.0164	91.9203	101.8507	26.57964	61.6619	145.2953	200.9479	59.53167	138.3407	140.9563	92.9279	90.47142	90.27987	63.57038	135.2101	90.32111	105.5755	61.84525
AC004233.	9.609075	8.003124	7.993069	3.117878	2.21497	6.851322	11.89552	8.334243	1.167288	9.606993	20.48083	11.04094	18.50552	20.47585	4.890029	3.297807	11.29014	3.640534	6.274156
AC004241.	372.8321	152.0593	230.7999	245.2731	277.9787	343.708	252.355	208.3561	371.1975	256.5067	254.2033	261.3022	227.2066	256.8788	197.5572	232.4954	214.5126	192.9483	219.5954
AC004241.	35.55358	7.002733	19.98267	69.63262	34.33203	21.69585	48.43177	8.334243	39.68778	7.685594	8.433284	42.3236	27.75828	10.23792	29.34017	22.2602	56.45069	41.86614	31.37078
AC004241.	38.4363	33.01288	28.97488	19.74656	44.2994	35.3985	19.54265	22.22465	35.01863	36.50657	32.52838	8.280704	10.28084	35.36737	39.12023	23.9091	24.63303	45.50667	17.92616
AC004241.	31.70995	8.003124	30.97314	12.47151	26.57964	23.97963	17.84329	6.482189	11.67288	21.13538	26.50461	21.1618	15.42127	24.19873	26.40616	23.08465	11.29014	9.101334	12.54831
AC004241.	184.4942	53.02069	102.9108	172.5226	143.973	126.7495	284.6429	37.96711	170.424	42.27077	45.78069	166.5342	139.8195	80.97267	74.32844	161.5926	115.9805	118.3173	147.8908
AC004253.	286.3504	210.082	242.7895	174.6012	279.0862	189.5532	209.0213	242.6191	611.6588	287.2491	419.2547	182.1755	210.7573	239.1951	193.6452	163.2415	209.3808	189.3078	104.868
AC004253.	0	0	3.996535	0	0	0	0	0	0	0	0	0	0	0	0	0.824452	0	0	0
AC004257.	2.882723	4.001562	2.997401	1.039293	1.107485	1.141887	0.84968	12.96438	0	8.646293	0	0.920078	9.252759	1.861441	0.978006	0	0	3.640534	0
AC004263.	3.84363	1.00039	0	0	0	3.425661	3.398721	0	0	12.48909	9.638039	0.920078	3.084253	4.653602	5.868035	0	3.079129	4.550667	0
AC004263.	45.16265	30.01171	31.97228	40.53242	59.80419	5.709435	14.44456	21.29862	74.70641	29.78168	75.89956	33.12282	19.5336	40.95169	24.45015	32.97807	44.13418	43.6864	31.37078
AC004264.	0	0	0	0	0	0	0	0	0	0	4.81902	0.920078	0	0.93072	0	0	0	0	0
AC004264.	309.4122	1020.398	839.2723	417.7957	387.6197	703.4024	521.7037	310.2191	109.725	455.3714	372.2693	557.5674	520.2107	336.9207	303.1818	299.276	358.2053	254.8374	444.5687
AC004381.	6.726353	12.00469	28.97488	22.86444	12.18233	5.709435	11.04584	2.778081	1.167288	25.93888	8.433284	9.200782	15.42127	9.307203	6.846041	18.13794	15.39564	14.56213	15.23724
AC004381.	76.8726	52.0203	57.94975	118.4794	56.48173	42.24982	117.2559	45.37532	32.68406	37.46727	21.68559	70.84602	49.34805	49.32818	57.70234	60.18498	54.39794	56.42827	53.77848
AC004383.	58.61536	45.01757	49.95668	50.92535	53.15928	65.08756	56.0789	67.59997	35.01863	45.15286	25.29985	80.04681	48.31997	51.18962	62.59237	46.1693	84.16285	61.88907	47.50432
AC004386.	0.960908	0	0	1.039293	1.107485	0	0	0.926027	1.167288	0	0	0.920078	0	0	0	0	0	0.910133	0.896308
AC004408.	0	3.001171	0	1.039293	6.64491	2.283774	5.098081	0	17.50932	4.803496	0	3.680313	1.028084	4.653602	0.978006	0	2.052753	1.820267	0.896308
AC004408.	5.765445	11.00429	1.998267	5.196464	3.322455	0	7.647122	1.852054	3.501863	15.37119	15.66181	0.920078	4.112337	3.722881	1.956012	6.595615	7.184634	6.370934	5.377848
AC004448.	51.88901	43.01679	44.96101	31.17878	77.52394	70.79699	22.94137	32.41095	134.2381	117.2053	136.1373	51.52438	40.09529	67.01186	49.8783	60.18498	94.42662	91.92348	73.49725
AC004448.	13.45271	8.003124	7.993069	8.314342	0	0	1.69936	4.630135	26.84762	4.803496	1.204755	1.840156	3.084253	0.93072	0.978006	1.648904	8.21101	1.820267	1.792616
AC004448.	0	4.001562	0	0	1.107485	0	0	0	0	1.921399	0	0	0	0	0	0	0	0	3.585232
AC004448.	89.3644	233.091	132.8848	178.7584	84.16885	117.6144	98.56291	163.9068	189.1006	164.2796	254.2033	123.2905	134.6791	138.6773	112.4707	83.26963	86.21561	86.46267	103.9717
AC004448.	6.726353	1.00039	0	0	0	6.851322	2.549041	5.556162	0	0	3.614265	0	0	0	0.978006	0	3.079129	0	0
AC004449.	35.55358	27.01054	37.96708	10.39293	19.93473	25.12151	9.346483	22.22465	33.85134	49.95636	31.32363	26.68227	23.64594	39.09025	15.64809	18.13794	39.0023	23.66347	20.61508
AC004453.	92.24712	594.2319	63.94455	21.82515	87.49131	89.06719	57.77826	66.67395	108.5578	64.36685	89.15186	68.08579	81.21867	76.31906	64.54838	90.6897	82.1101	101.9349	55.57109
AC004453.	122.9962	118.0461	150.8692	128.8723	110.7485	127.8913	170.7857	75.00819	168.0894	98.95202	78.30907	91.08774	88.41526	99.58707	100.7346	89.86525	99.5585	72.81067	116.52
AC004461.	0.960908	3.001171	1.998267	2.078586	0	0	2.549041	0	0	5.764196	0	9.200782	12.33701	7.445762	4.890029	0.824452	2.052753	2.7304	0
AC004461.	25.9445	30.01171	66.94196	57.1611	56.48173	14.84453	45.03305	26.85478	17.50932	54.75986	44.57593	49.68422	52.4323	43.74385	51.83431	68.4295	53.37157	53.69787	43.91909
AC004466.	29.78813	28.01093	15.98614	19.74656	11.07485	14.84453	33.98721	25.92876	17.50932	24.97818	24.0951	29.4425	24.67402	22.33729	20.53812	34.62698	34.89679	29.12427	17.02985
AC004466.	13.45271	3.001171	35.96881	20.78586	21.04221	20.55397	26.34009	22.22465	4.669151	48.99566	42.16642	37.72321	26.73019	68.8733	19.56012	23.08465	42.08143	45.50667	53.77848
AC004466.	71.10716	87.03397	145.8735	38.45383	105.2111	107.3374	28.88913	148.1643	32.68406	101.8341	55.41872	118.6901	108.9769	84.69555	104.6466	50.29156	43.1078	102.8451	72.60094
AC004467.	5.765445	1.00039	1.998267	9.353635	0	1.141887	6.797442	3.704108	0	3.842797	2.40951	2.760235	2.056169	0.93072	0	11.54233	2.052753	4.550667	1.792616
AC004471.	7.68726	6.002343	2.997401	0	4.42994	3.425661	3.398721	2.778081	4.669151	2.882098	2.40951	9.200782	3.084253	13.03008	6.846041	0	6.158258	0.910133	1.792616
AC004471.	24.9836	19.00742	52.95408	33.25737	12.18233	28.54718	22.94137	46.30135	16.34203	13.44979	26.50461	38.64329	24.67402	31.64449	28.36217	17.31349	25.65941	24.5736	35.85232
AC004477.	129.7225	151.059	266.7687	58.2004	198.2398	134.7427	81.5693	119.4575	365.3611	283.4063	420.4595	152.733	139.8195	166.5989	203.4252	117.8966	167.2993	177.476	162.2317
AC004477.	218.126	399.1558	352.6942	468.721	243.6467	364.262	300.7868	333.3697	162.253	293.0133	226.4939	215.2983	214.8696	248.5023	238.6334	393.2635	416.7088	214.7915	381.8272
AC004485.	2.882723	17.00664	1.998267	3.117878	5.537425	3.425661	3.398721	3.704108	0	0.960699	3.614265	7.360626	2.056169	8.376483	3.912023	1.648904	6.158258	5.4608	12.54831
AC004486.	0.960908	1.00039	6.993936	6.235757	0	10.27698	7.647122	6.482189	0	0	1.204755	0.920078	3.084253	6.515042	2.934017	0.824452	8.21101	0.910133	8.066772
AC004490.	1.921815	2.000781	18.98354	10.39293	6.64491	5.709435	10.19616	10.1863	8.171014	17.29259	1.204755	13.80117	14.39318	12.09936	10.75806	13.19123	22.58028	23.66347	6.274156
AC004491.	0	1.00039	0.999134	2.078586	13.28982	0	0.84968	0	0	13.44979	12.04755	0	5.140422	9.307203	8.802052	0.824452	4.105505	0	4.48154
AC004492.	144.1361	173.0675	258.7756	180.8369	182.735	99.34417	346.6695	303.7369	98.05217	140.2621	86.74235	261.3022	179.9148	175.9061	224.9413	141.8057	102.6376	181.1165	246.4847
AC004494.	29.78813	29.01132	26.97661	72.75049	42.08443	27.40529	42.48401	36.11505	12.84017	52.83846	69.87578	69.00587	51.40422	42.81313	23.47214	56.06272	51.31881	56.42827	53.77848
AC004520.	526.5773	432.1687	690.4014	542.5108	652.3086	462.4642	693.3391	370.4108	784.4174	521.6597	456.6021	564.928	548.9971	353.6737	436.1906	425.4171	472.1331	283.9616	362.1084
AC004522.	0	0	0	0	0	0	0	0	0	1.921399	0	0	0	0	0	0	1.026376	0	0
AC004522.	57.65445	32.01249	48.95755	56.12181	42.08443	47.95925	53.52986	45.37532	63.03354	51.87776	22.89034	43.24368	74.02207	37.22881	44.98827	42.04704	51.31881	38.2256	42.12647
AC004528.	1.921815	0	0	5.196464	4.42994	0	5.947762	0	0	0.960699	1.204755	6.440548	4.112337	0.93072	0.978006	4.122259	5.131881	0	1.792616
AC004540.	244.0705	273.1066	178.8449	170.444	222.6045	174.7087	180.1322	228.7287	177.4277	160.4368	125.2945	263.1424	304.313	210.3428	164.305	190.4484	187.8269	238.455	307.4336
AC004540.	29.78813	45.01757	25.97748	47.80747	27.68712	45.67548	36.53625	46.30135	16.34203	33.62447	28.91412	35.88305	53.46039	34.43665	54.76833	42.04704	18.47477	43.6864	37.64493
AC004543.	4.804538	2.000781	7.993069	0	4.42994	0	0.84968	8.334243	2.334575	0	0	0.920078	1.028084	0	0	2.473355	3.079129	0	0
AC004551.	0.960908	10.0039	6.993936	5.196464	5.537425	7.993209	5.098081	10.1863	9.338302	5.764196	4.81902	7.360626	1.028084	8.376483	1.956012	1.648904	2.052753	11.83173	5.377848
AC004552.	20.17906	8.003124	13.98787	7.275049	8.859879	13.70264	8.496802	8.334243	19.84389	11.52839	9.638039	7.360626	8.224675	9.307203	4.890029	13.19123	13.34289	10.9216	8.066772
AC004554.	51.88901	33.01288	36.96795	122.6365	16.61227	52.5268	62.02666	37.96711	58.36439	39.38867	33.73314	55.20469	49.34805	63.28898	79.21847	51.94047	86.21561	58.24854	52.88217
AC004584.	48.04538	44.01718	41.96361	16.62868	34.33203	21.69585	73.0725	49.07943	21.01118	26.89958	31.32363	37.72321	47.29188	17.68369	61.61437	13.19123	17.4484	65.52961	45.71171

AC004584.	0	13.00508	0	0	5.537425	0	0	0	2.334575	0.960699	1.204755	1.840156	0	0	0.978006	0.824452	2.052753	0	0
AC004584.	58.61536	91.03553	114.9004	89.37918	46.51437	83.35775	151.2431	123.1616	21.01118	112.4018	40.96167	169.2944	94.58376	85.62627	134.9648	121.1944	105.7168	70.08027	63.63786
AC004585.	12.4918	3.001171	4.995668	15.58939	19.93473	11.41887	3.398721	32.41095	26.84762	8.646293	14.45706	24.84211	27.75828	16.75297	14.67009	23.08465	26.68578	26.39387	10.7557
AC004585.	0.960908	0	0	0	0	0	0	0	0	0	0	0	0	0	0	0	0	0	0.896308
AC004593.	0.960908	0	0	0	0	0	0	0	0	0	0	0	2.056169	0	0	0	0	0	0
AC004594.	28.82723	22.00859	39.96535	28.0609	37.65449	31.97284	79.02026	40.74519	11.67288	29.78168	30.11887	27.60235	41.12337	46.53602	47.92229	32.97807	33.87042	34.58507	33.16339
AC004596.	30.74904	26.01015	23.97921	45.72888	27.68712	34.25661	37.38593	39.81916	30.34948	17.29259	14.45706	34.04289	24.67402	39.09025	43.03226	57.71163	50.29244	66.43974	77.97879
AC004597.	0.960908	62.02421	59.94802	21.82515	100.7811	62.80379	71.37314	103.715	32.68406	136.4193	186.737	68.08579	76.07824	0.93072	4.890029	24.73355	39.0023	3.640534	9.859387
AC004623.	24.9836	5.001952	13.98787	4.157171	18.82724	11.41887	22.94137	41.67122	37.35321	42.27077	67.46627	19.32164	43.17954	20.47585	15.64809	6.595615	13.34289	22.75334	25.09662
AC004637.	31.70995	16.00625	52.95408	22.86444	45.40688	18.27019	26.34009	31.48492	39.68778	31.70308	57.82823	29.4425	49.34805	22.33729	34.2302	29.68027	17.4484	24.5736	17.92616
AC004656.	22519.83	25554.97	19050.48	31435.49	13902.26	22955.35	26384.27	18952.07	11684.55	17901.67	13160.74	20657.6	23165.83	27364.11	26722.05	25743.51	26178.75	22507.6	25534.92
AC004672.	0.960908	1.00039	1.998267	0	6.64491	3.425661	8.496802	38.89314	11.67288	4.803496	4.81902	0.920078	2.056169	1.861441	8.802052	6.595615	4.105505	1.820267	4.48154
AC004672.	3.84363	2.000781	1.998267	0	0	2.283774	2.549041	0.926027	0	0	0	0	0	3.722881	0	1.648904	0	0	1.792616
AC004674.	0.960908	0	3.996535	0	0	0	0	0	0	0	0	0	1.028084	0	0	0	0	0	0
AC004678.	3.84363	16.00625	5.994802	7.275049	2.21497	7.993209	9.346483	11.11232	16.34203	3.842797	10.84279	15.64133	12.33701	1.861441	11.73607	24.73355	13.34289	7.281067	32.26709
AC004678.	23.06178	30.01171	48.95755	30.13949	35.43952	53.66869	36.53625	59.26573	17.50932	59.56335	31.32363	23.00196	35.98295	25.12945	32.27419	33.80253	31.81766	25.48374	35.85232
AC004686.	78.79442	26.01015	69.93936	17.66798	46.51437	23.97963	37.38593	48.15341	17.50932	58.60265	33.73314	45.08383	41.12337	59.5661	62.59237	51.94047	28.73854	40.04587	45.71171
AC004687.	114.348	33.01288	8.992203	98.73281	99.67364	49.10114	9.346483	49.07943	477.4207	107.5983	150.5944	86.48735	55.51656	113.5479	81.17448	277.8403	275.0688	526.9672	249.1736
AC004690.	16.33543	13.00508	7.993069	22.86444	15.50479	17.12831	10.19616	12.96438	14.00745	8.646293	0	6.440548	1.028084	0	8.802052	9.893422	10.26376	7.281067	12.54831
AC004691.	14.41361	5.001952	3.996535	23.90373	9.967364	1.141887	4.248401	0.926027	4.669151	6.724895	0	3.680313	15.42127	6.515042	5.868035	6.595615	3.079129	3.640534	3.585232
AC004692.	1.921815	2.000781	3.996535	0	0	1.141887	5.098081	0	2.334575	0	0	0.920078	0	0	2.934017	13.19123	9.237386	6.370934	4.48154
AC004692.	0	5.001952	0	2.078586	1.107485	0	2.549041	0.926027	16.34203	0	0	6.440548	0	0.93072	0.978006	5.771163	1.026376	0.910133	4.48154
AC004696.	57.65445	80.03124	88.9229	102.89	42.08443	44.53359	101.9616	75.93422	29.18219	49.95636	20.48083	76.36649	58.60081	91.21059	88.02052	43.69595	65.68808	44.59654	65.43048
AC004696.	7.68726	3.001171	40.96448	17.66798	31.00958	2.283774	33.98721	3.704108	3.501863	4.803496	7.228529	30.36258	17.47743	13.9608	6.846041	8.244518	7.184634	8.191201	9.859387
AC004702.	0	0	0	0	0	0	0	0.926027	2.334575	0	16.86657	0.920078	0	0	0	0	0	0	0
AC004704.	0	0	0	5.196464	4.42994	5.709435	0	0	0	0	0	0.920078	2.056169	0	0	0	1.026376	0	1.792616
AC004706.	25.9445	13.00508	12.98874	56.12181	22.1497	45.67548	44.18337	37.96711	54.86252	36.50657	43.37118	39.56336	37.01104	15.82225	14.67009	22.2602	17.4484	31.85467	34.0597
AC004706.	7.68726	28.01093	27.97574	60.27898	6.64491	52.5268	34.83689	74.08216	22.17847	95.10923	69.87578	34.04289	35.98295	24.19873	18.58211	25.55801	27.71216	30.0344	29.57816
AC004706.	305.5686	300.1171	267.7678	353.3595	323.3856	298.0325	404.4478	324.1095	100.3867	151.7905	248.1795	442.5576	452.3571	253.1559	205.3812	300.1005	407.4714	294.8832	365.6936
AC004707.	0.960908	2.000781	3.996535	1.039293	0	0	2.549041	5.556162	0	0	0	0.920078	0	0	3.912023	2.473355	1.026376	10.01147	3.585232
AC004754.	33.63176	17.00664	38.96621	6.235757	22.1497	29.68906	19.54265	10.1863	71.20455	31.70308	50.5997	20.24172	24.67402	14.89152	11.73607	11.54233	15.39564	28.21414	26.88924
AC004771.	50.9281	64.02499	66.94196	37.41454	81.95388	85.64153	55.22922	80.56435	107.3905	135.4586	143.3658	86.6472	88.41526	64.2197	72.37243	64.30724	48.23968	51.8776	68.1194
AC004771.	54.77173	35.01367	40.96448	28.0609	25.47215	14.84453	35.68657	15.74246	39.68778	48.03496	26.50461	38.64329	33.92678	36.29809	29.34017	23.08465	35.92317	34.58507	27.78555
AC004771.	78.79442	72.02811	54.95235	40.53242	120.7159	50.24303	59.47762	38.89314	29.18219	78.77734	93.97088	66.24563	86.35909	36.29809	41.07624	49.46711	47.21331	102.8451	148.7871
AC004771.	176.807	129.0504	117.8978	177.7191	73.094	108.4793	162.2889	107.4191	56.02981	95.10923	62.64725	206.0975	220.0101	191.7284	124.2067	179.7305	153.9564	153.8125	170.2985
AC004775.	30.74904	18.00703	16.98527	5.196464	4.42994	7.993209	16.14392	9.26027	10.50559	10.56769	14.45706	8.280704	1.028084	9.307203	3.912023	6.595615	8.21101	3.640534	8.066772
AC004777.	0	6.002343	9.991337	2.078586	2.21497	1.141887	1.69936	6.482189	9.338302	12.48909	0	6.440548	2.056169	2.792161	0.978006	4.122259	0	4.550667	8.066772
AC004784.	4.804538	0	8.992203	1.039293	13.28982	2.283774	0.84968	1.852054	0	19.21399	1.204755	0.920078	7.196591	1.861441	0	2.473355	2.052753	6.370934	1.792616
AC004790.	0	0	0	0	0	0	0	0	0	0	3.614265	0	0	0	0	0	0	0	0
AC004801.	2.882723	9.003514	9.991337	0	4.42994	2.283774	1.69936	7.408216	7.003726	1.921399	4.81902	4.600391	4.112337	12.09936	2.934017	0	2.052753	3.640534	0
AC004801.	87.44259	74.02889	94.9177	87.30059	143.973	55.95246	50.98081	95.38078	262.6397	92.22713	92.76613	93.84798	103.8365	133.093	128.1188	95.63641	125.2179	104.6653	67.2231
AC004801.	0	8.003124	0	0	0	0	0	0	0	0	0	0	0	0	0.978006	0	0	0	0
AC004801.	0	0	0	0	0	1.141887	0	0	0	0	0	0.920078	0	0	0	0	0	0.910133	0.896308
AC004801.	0.960908	2.000781	0	0	1.107485	0	1.69936	8.334243	0	3.842797	0	1.840156	0	0	4.890029	1.648904	1.026376	0	0.896308
AC004801.	18.25724	31.0121	27.97574	40.53242	34.33203	20.55397	28.88913	31.48492	81.71014	26.89958	57.82823	23.00196	29.81445	24.19873	34.2302	28.85581	70.81996	33.67494	35.85232
AC004801.	0	0	0	0	0	0	0	0	0	0	0	0	0	0.93072	0.978006	0	0	0	0
AC004803.	822.5368	879.3432	1012.122	936.4028	794.0667	745.6522	960.9884	974.1804	861.4583	803.1446	809.5953	728.702	903.6862	1000.524	933.9956	703.2574	563.4806	868.2673	777.099
AC004805.	0.960908	0	0	0	4.42994	1.141887	4.248401	3.704108	0	0	1.204755	0	1.028084	0.93072	1.956012	0	0	0	0.896308
AC004805.	10.56998	10.0039	4.995668	3.117878	12.18233	5.709435	13.59488	22.22465	0	4.803496	22.89034	8.280704	1.028084	15.82225	4.890029	3.297807	2.052753	2.7304	3.585232
AC004808.	0.960908	0	0	0	0	0	3.398721	3.704108	0	0	1.204755	0	1.028084	3.722881	0	0	0	0	1.792616
AC004812.	0	0	0	0	0	0	0	0	0	0	0	0	0	0	0	1.648904	2.052753	0	0
AC004812.	445.8611	516.2015	609.4715	439.6208	611.3317	451.0454	513.2069	438.0108	815.9341	588.9086	718.0339	580.5694	650.7774	606.8296	519.3211	410.577	456.7374	655.2961	596.0448
AC004816.	761.9997	819.3198	625.4577	1680.536	573.6772	929.496	1121.578	875.0955	828.7743	978.9525	950.5516	902.5967	890.3211	900.9373	837.173	1115.483	1304.524	684.4203	1124.866
AC004816.	0	0	0.999134	2.078586	1.107485	1.141887	0	1.852054	12.84017	0	1.204755	17.48149	5.140422	0	0	1.648904	3.079129	0	6.274156
AC004817.	11.53089	0	7.993069	4.157171	6.64491	2.283774	0.84968	1.852054	9.338302	0.960699	0	4.600391	8.224675	3.722881	2.934017	0.824452	0	2.7304	6.274156
AC004817.	15.37452	0	11.9896	3.117878	6.64491	3.425661	1.69936	22.22465	7.003726	1.921399	2.40951	7.360626	3.084253	26.06017	32.27419	0	6.158258	10.9216	11.652
AC004817.	19.21815	15.00586	31.97228	1.039293	37.65449	14.84453	4.248401	59.26573	4.669151	25.93888	14.45706	23.00196	65.7974	24.19873	6.846041	0.824452	3.079129	80.09174	34.95601
AC004817.	7.68726	4.001562	19.98267	0	25.47215	6.851322	11.89552	11.11232	15.17474	0	21.68559	20.24172	9.252759	22.33729	13.69208	0.824452	7.184634	1.820267	0
AC004825.	55.73264	75.02928	79.93069	60.27898	27.68712	59.37812	70.52346	66.67395	84.04472	128.7337	78.30907	66.24563	58.60081	47.46674	32.27419	37.92478	44.13418	54.608	98.59387
AC004828.	18.25724	11.00429	47.95842	30.13949	17.71976	4.567548	81.5693	65.74792	8.171014	1.921399	12.04755	54.28462	71.96591	32.57521	61.61437	50.29156	71.84634	51.8776	41.23017
AC004830.	71.10716	119.0465	187.8371	141.3438	96.35119	162.148	107.9094	189.8355	116.7288	320.8736	352.9932	142.6121	128.5105	125.6472	96.82258	92.3386	92.37386	63.70934	78.8751
AC004832.	0	0	1.998267	0	4.42994	0	0	7.408216	2.334575	3.842797	3.614265	2.760235	1.028084	0	0.978006	0	0	0	4.48154

AC004832.	14.41361	25.00976	36.96795	13.51081	15.50479	29.68906	11.04584	25.00273	36.18592	27.86028	20.48083	16.56141	19.5336	27.92161	31.29619	7.420066	16.42202	17.29253	14.34093
AC004832.	42.27993	38.01484	53.95322	42.611	8.859879	52.5268	43.33369	36.11505	82.87743	79.73804	49.39495	38.64329	21.58977	62.35826	48.90029	50.29156	66.71446	27.304	34.0597
AC004832.	0	4.001562	2.997401	1.039293	6.64491	9.135096	5.947762	7.408216	15.17474	8.646293	0	3.680313	6.168506	4.653602	0.978006	1.648904	5.131881	2.7304	8.066772
AC004834.	49.96719	33.01288	54.95235	23.90373	15.50479	26.2634	43.33369	62.04381	59.53167	51.87776	53.00921	44.16376	31.87062	54.9125	58.68035	22.2602	83.13648	50.96747	67.2231
AC004835.	13.45271	2.000781	6.993936	5.196464	13.28982	3.425661	3.398721	12.96438	8.171014	0.960699	8.433284	2.760235	7.196591	4.653602	5.868035	4.122259	0	0.910133	2.688924
AC004835.	0	0	0	0	0	0	0	0	0	0	0	0	0	0	0	0	0	0	1.792616
AC004836.	83.59896	78.03045	87.92376	70.67191	163.9078	204.3978	99.41259	67.59997	123.7325	127.773	202.3988	70.84602	82.24675	50.2589	88.02052	67.60505	93.40024	56.42827	39.43755
AC004837.	85.52077	131.0511	120.8952	111.2043	70.87903	55.95246	274.4467	123.1616	46.69151	109.5197	40.96167	201.4971	122.342	79.11123	112.4707	86.56744	108.7959	111.9464	149.6834
AC004837.	0	0	0	0	0	0	0.84968	1.852054	0	0	0	0	0	0	0	0	0	0	0
AC004839.	17.29634	17.00664	17.98441	25.98232	18.82724	26.2634	5.098081	24.0767	7.003726	16.33189	30.11887	14.72125	16.44935	26.06017	9.780058	28.85581	19.50115	12.74187	20.61508
AC004840.	0	0	0.999134	0	1.107485	0	0	0	5.836439	0	9.638039	0.920078	1.028084	0	0.978006	0	0	0	0
AC004840.	0	0	6.993936	0	8.859879	2.283774	0.84968	1.852054	2.334575	0	0	0.920078	4.112337	0	4.890029	0.824452	0	0	0
AC004846.	371.8712	176.0687	296.7427	188.112	254.7215	259.2084	245.5576	274.104	238.1267	289.1705	248.1795	439.7974	509.9298	292.2462	306.1158	373.4767	347.9415	316.7264	287.7149
AC004846.	152.7843	169.066	128.8882	96.65423	108.5335	113.0468	144.4456	325.0355	172.7586	162.3582	156.6181	220.8188	171.6901	195.4513	194.6232	111.301	66.71446	87.37281	89.63079
AC004847.	15.37452	53.02069	81.92896	76.90766	49.83682	54.81058	56.92858	108.3452	63.03354	36.50657	46.98544	96.60821	121.314	114.4786	100.7346	211.0597	102.6376	559.7321	381.8272
AC004852.	0	1.00039	0	0	0	0	0	0	0	0	0	0	0	0	0	0	1.026376	0	0
AC004853.	0	1.00039	0	0	0	0	0	0	0	0	0	0	0	0	0	0	0	0	0.896308
AC004853.	0	0	0	1.039293	0	0	0	0	0	0	0	0	0	0	3.912023	0	0	0	0
AC004854.	19.21815	18.00703	27.97574	4.157171	9.967364	14.84453	4.248401	16.66849	17.50932	19.21399	24.0951	10.12086	18.50552	11.16864	6.846041	18.96239	6.158258	16.3824	16.13354
AC004854.	57.65445	66.02577	72.93676	49.88605	46.51437	75.36454	50.13113	39.81916	59.53167	88.38433	67.46627	88.32751	69.90974	86.55699	90.95454	52.76492	88.26836	58.24854	45.71171
AC004858.	2.882723	0	0	0	0	1.141887	3.398721	0	0	0	9.638039	0	1.028084	0	0	2.473355	2.052753	0	6.274156
AC004862.	5.765445	1.00039	3.996535	12.47151	0	9.135096	16.9936	1.852054	33.85134	1.921399	0	2.760235	2.056169	16.75297	5.868035	34.62698	34.89679	1.820267	3.585232
AC004865.	0	0	0.999134	2.078586	11.07485	1.141887	5.098081	9.26027	23.34575	0.960699	7.228529	4.600391	6.168506	0.93072	5.868035	4.946711	5.131881	0.910133	3.585232
AC004865.	22.10087	16.00625	24.97834	10.39293	44.2994	33.11472	28.88913	49.07943	54.86252	59.56335	60.23774	60.72516	40.09529	27.92161	14.67009	36.27588	17.4484	30.0344	13.44462
AC004870.	10.56998	12.00469	1.998267	14.5501	18.82724	21.69585	17.84329	0.926027	16.34203	5.764196	0	10.12086	7.196591	0.93072	13.69208	9.893422	13.34289	2.7304	5.377848
AC004870.	5.765445	2.000781	0	0	0	3.425661	1.69936	2.778081	0	0	0	0.920078	0	0	0.978006	0	0	0	0
AC004870.	0	0	0	1.039293	0	1.141887	1.69936	0	0	0	0	0	0	2.792161	0.978006	0.824452	0	0	0.896308
AC004870.	0	1.00039	0.999134	1.039293	0	0	2.549041	4.630135	0	0	0	0	0	0.93072	0	0	1.026376	0	3.585232
AC004875.	13.45271	2.000781	17.98441	4.157171	0	3.425661	5.098081	0	11.67288	3.842797	0	7.360626	4.112337	0	3.912023	3.297807	0	0.910133	1.792616
AC004877.	130.6834	156.0609	217.8111	145.501	137.3281	282.0461	146.9947	219.4684	178.595	285.3277	233.7224	180.3353	156.2688	196.382	230.8094	212.7086	177.5631	164.7341	153.2687
AC004877.	76.8726	117.0457	199.8267	155.8939	153.9404	101.6279	90.06611	167.6109	210.1118	178.6901	224.0844	153.6531	124.3982	144.2616	116.3827	103.0565	166.273	105.5755	97.69757
AC004882.	0	0	0	0	0	0	0.84968	0.926027	3.501863	0	0	0.920078	0	0	0.978006	0	0	1.820267	4.48154
AC004884.	0	0	0	0	0	0	0	2.778081	0	0	0	0.920078	0	0	0	0	0	0.910133	0
AC004884.	12.4918	24.00937	16.98527	17.66798	4.42994	4.567548	17.84329	7.408216	15.17474	9.606993	8.433284	17.48149	27.75828	10.23792	10.75806	6.595615	10.26376	11.83173	7.170464
AC004888.	0	0	4.995668	4.157171	0	1.141887	4.248401	0.926027	0	0	0	1.840156	1.028084	3.722881	1.956012	3.297807	0	0	0.896308
AC004889.	121.0743	129.0504	60.94715	94.57564	109.641	156.4385	62.87634	150.9424	25.68033	22.09608	71.08054	25.76219	38.03912	57.70466	59.65836	112.9499	87.24198	142.8909	132.6536
AC004890.	24.02269	3.001171	9.991337	10.39293	50.94431	1.141887	10.19616	15.74246	29.18219	15.37119	4.81902	11.04094	5.140422	10.23792	16.6261	1.648904	6.158258	20.02294	8.066772
AC004890.	1603.755	1489.581	1253.913	1016.428	1309.047	1261.785	1306.808	1482.569	1473.117	1682.184	2043.264	1929.404	1975.978	1241.581	1217.617	1067.665	1248.074	1266.906	1333.706
AC004890.	202.7515	219.0855	330.7132	277.4912	215.9596	148.4453	323.7282	205.578	58.36439	202.7075	130.1135	221.7389	280.667	270.8396	235.6994	164.8904	215.539	220.2523	205.2545
AC004893.	10.56998	7.002733	64.94369	16.62868	36.547	14.84453	21.24201	29.63287	0	7.685594	26.50461	27.60235	19.5336	9.307203	11.73607	17.31349	9.237386	13.652	8.066772
AC004893.	971.4775	700.2733	938.1865	509.2535	698.823	683.9903	860.7261	951.9558	436.5656	782.9699	781.8859	890.6357	834.8045	805.0731	807.8328	653.7903	608.6411	785.4451	914.2341
AC004895.	7.68726	18.00703	14.987	6.235757	17.71976	11.41887	28.88913	21.29862	0	36.50657	20.48083	13.80117	8.224675	3.722881	15.64809	3.297807	6.158258	4.550667	13.44462
AC004898.	0.960908	1.00039	0.999134	6.235757	16.61227	0	13.59488	2.778081	0	14.41049	7.228529	1.840156	9.252759	9.307203	10.75806	0	4.105505	0.910133	6.274156
AC004899.	5.765445	4.001562	8.992203	0	5.537425	7.993209	11.04584	1.852054	0	2.882098	0	0	4.112337	0	0	0	0	0.910133	2.688924
AC004899.	5.765445	3.001171	13.98787	0	1.107485	14.84453	0.84968	0	1.167288	19.21399	4.81902	11.04094	5.140422	2.792161	6.846041	0.824452	0	0	5.377848
AC004906.	13.45271	16.00625	5.994802	4.157171	8.859879	4.567548	1.69936	1.852054	11.67288	0	4.81902	4.600391	11.30893	0.93072	3.912023	4.122259	21.5539	10.01147	14.34093
AC004908.	304.6077	402.157	511.5564	278.5305	429.7041	296.8906	361.1141	548.208	155.2493	280.5242	260.2271	510.6434	400.9529	368.5652	411.7405	291.0315	260.6996	394.0878	418.5758
AC004908.	267.1323	387.1511	418.637	203.7014	341.1054	276.3367	381.5064	365.7807	109.725	234.4106	146.9801	509.7233	457.4975	396.4868	447.9267	243.2133	168.3257	374.0648	385.4124
AC004908.	106.6607	117.0457	103.9099	139.2652	76.41646	116.4725	158.8902	183.3534	78.20828	103.7555	74.6948	126.0507	100.7523	115.4093	127.1408	81.62073	66.71446	137.4301	95.90495
AC004910.	3.84363	1.00039	0	0	0	0	0	0	0	0	0	0	0.920078	0	0	0	0	2.052753	0.910133	0
AC004917.	9.609075	11.00429	19.98267	11.43222	19.93473	14.84453	22.09169	11.11232	7.003726	12.48909	6.023774	8.280704	11.30893	6.515042	11.73607	12.36678	7.184634	10.9216	14.34093
AC004918.	313.2559	273.1066	366.6821	432.3458	447.4239	364.262	286.3422	335.2218	575.4729	554.3235	665.0247	372.6317	361.8857	275.4932	273.8416	451.7996	400.2867	303.0744	276.9592
AC004918.	314.2168	301.1175	469.5928	782.5875	384.2973	269.4853	466.4745	454.6793	112.0596	293.0133	218.0606	602.6512	421.5146	347.1587	348.1701	811.2606	479.3177	521.5064	682.9867
AC004920.	0	0	0	0	0	0	0	0	0	0	0	0	1.028084	0	0	0	0	0.910133	0
AC004921.	179.6897	45.01757	48.95755	111.2043	22.1497	66.22945	40.78465	44.4493	135.4054	26.89958	30.11887	60.72516	45.23571	78.18051	90.95454	118.7211	59.52982	135.6099	50.19325
AC004922.	8.648168	9.003514	14.987	17.66798	2.21497	6.851322	15.29424	0	3.501863	4.803496	9.638039	11.96102	3.084253	7.445762	5.868035	5.771163	13.34289	17.29253	8.066772
AC004923.	0.960908	6.002343	15.98614	6.235757	0	5.709435	6.797442	3.704108	0	2.882098	7.228529	11.96102	8.224675	9.307203	21.51613	1.648904	2.052753	3.640534	2.688924
AC004923.	0	2.000781	0	0	0	0	0.84968	0	1.167288	0	6.023774	0	1.028084	0	2.934017	0	0	0	0
AC004923.	183.5333	136.0531	299.7401	190.1906	229.2494	252.357	127.452	212.9862	274.3126	296.8561	374.6788	282.464	244.6841	220.5807	286.5557	215.1819	208.3544	274.8603	224.077
AC004924.	0.960908	0	0	0	0	0	0.84968	0	4.669151	0	0	0	0	0.93072	2.934017	3.297807	0	0	0
AC004925.	74.95079	54.02108	82.92809	54.04322	17.71976	78.7902	92.61515	55.56162	37.35321	167.1617	132.523	44.16376	49.34805	45.60529	48.90029	80.79628	20.52753	48.23707	34.95601
AC004930.	0	0	0	0	1.107485	0	0	0	0	0	0	0	0	0	0	0	1.026376	0	0.896308

AC004941.	0.960908	4.001562	12.98874	9.353635	6.64491	0	4.248401	0	5.836439	0.960699	4.81902	4.600391	1.028084	0.93072	1.956012	5.771163	5.131881	0.910133	0
AC004941.	0	0	0	0	0	0	0	0	0	0.960699	0	0	0	0.93072	0	0	0	0.910133	0
AC004943.	62.45899	27.01054	49.95668	28.0609	52.05179	18.27019	43.33369	58.3397	19.84389	31.70308	37.3474	37.72321	53.46039	40.02097	60.63636	39.57369	42.08143	45.50667	66.32679
AC004943.	556.3655	462.1804	513.5547	487.4283	440.779	417.9306	610.0704	696.3723	525.2795	424.6291	351.7884	591.6103	591.1485	634.7512	645.4838	554.8561	479.3177	671.6785	604.1116
AC004943.	7.68726	4.001562	1.998267	0	1.107485	1.141887	4.248401	2.778081	28.01491	0	8.433284	9.200782	14.39318	17.68369	5.868035	0.824452	4.105505	0.910133	1.792616
AC004944.	0	0	0	0	0	0	0.84968	3.704108	0	0	0	0	0	0	0	0	0	0	0
AC004945.	0.960908	0	4.995668	2.078586	2.21497	0	5.947762	12.03835	0	1.921399	0	3.680313	2.056169	1.861441	3.912023	1.648904	9.237386	1.820267	3.585232
AC004947.	43.24084	62.02421	101.9116	31.17878	110.7485	33.11472	45.88273	69.45203	57.1971	78.77734	65.05676	140.772	119.2578	69.80402	84.1085	8.244518	42.08143	116.4971	64.53417
AC004947.	485.2583	424.1655	261.773	297.2377	495.0458	173.5668	335.6237	321.3314	374.6994	182.5329	134.9325	382.7525	553.1094	320.1678	317.8519	255.5801	257.6204	369.5142	509.9992
AC004948.	19.21815	14.00547	18.98354	41.57171	18.82724	44.53359	13.59488	22.22465	52.52795	25.93888	25.29985	13.80117	17.47743	11.16864	17.6041	32.15362	23.60665	15.47227	17.92616
AC004951.	70.14625	52.0203	95.91683	42.611	96.35119	58.23624	102.8113	75.93422	59.53167	53.79916	38.55216	95.68814	66.82548	107.0328	85.08651	93.16306	56.45069	108.3059	77.08248
AC004951.	38.4363	34.01327	47.95842	13.51081	68.66407	36.54038	22.09169	50.93149	108.5578	63.40615	75.89956	36.80313	40.09529	79.11123	47.92229	32.15362	41.05505	70.99041	55.57109
AC004951.	30.74904	26.01015	25.97748	5.196464	31.00958	26.2634	22.94137	24.0767	60.69896	24.01748	68.67103	46.00391	19.5336	28.85233	23.47214	23.08465	17.4484	29.12427	27.78555
AC004951.	66.30262	52.0203	51.95495	41.57171	76.41646	60.52001	53.52986	81.49038	135.4054	101.8341	104.8137	56.12477	27.75828	68.8733	54.76833	77.49847	42.08143	61.88907	43.91909
AC004967.	581.3491	451.1761	476.5868	495.7427	536.0227	226.0936	648.306	558.3943	303.4948	582.1837	599.9679	359.7506	403.0091	344.3665	339.368	398.2102	429.0253	545.1699	405.1312
AC004969.	19.21815	31.0121	14.987	21.82515	13.28982	2.283774	16.9936	18.52054	2.334575	21.13538	15.66181	9.200782	7.196591	5.584322	3.912023	20.6113	12.31652	4.550667	14.34093
AC004972.	0	0	1.998267	0	4.42994	0	0	0	0	0	0	0	0	0	0	0	0	0	0
AC004973.	3.84363	15.00586	8.992203	19.74656	14.3973	9.135096	5.098081	8.334243	0	3.842797	3.614265	11.04094	7.196591	13.9608	7.824047	9.893422	8.21101	20.02294	9.859387
AC004975.	25.9445	19.00742	24.97834	29.1002	16.61227	18.27019	22.94137	39.81916	0	11.52839	21.68559	37.72321	12.33701	23.26801	20.53812	27.20691	9.237386	50.96747	43.02278
AC004975.	1.921815	0	0	0	1.107485	1.141887	0	7.408216	0	0.960699	10.84279	0	0	0	0.978006	3.297807	1.026376	5.4608	0.896308
AC004980.	537.1473	635.2479	574.5019	456.2495	428.5967	405.3699	384.9051	463.0135	498.4319	433.2754	497.5638	465.5596	474.975	473.7366	499.761	342.972	329.4668	512.4051	440.0872
AC004980.	56.69354	32.01249	34.96968	58.2004	64.23413	25.12151	33.98721	56.48765	39.68778	57.64196	46.98544	52.44446	48.31997	92.14131	48.90029	71.72731	59.52982	50.96747	44.8154
AC004980.	6.726353	5.001952	7.993069	0	8.859879	7.993209	5.098081	24.0767	11.67288	20.17468	1.204755	2.760235	3.084253	9.307203	14.67009	0.824452	0	0	3.585232
AC004980.	6.726353	9.003514	9.991337	1.039293	7.752394	10.27698	1.69936	6.482189	7.003726	8.646293	19.27608	7.360626	6.168506	10.23792	15.64809	3.297807	3.079129	5.4608	11.652
AC004982.	419.9166	299.1167	417.6379	393.892	415.3068	400.8023	177.5832	189.8355	402.7143	342.9696	319.26	396.5537	442.0763	302.4841	318.8299	244.0377	288.4117	399.5486	366.59
AC004982.	1025.288	829.3237	1108.039	849.1022	926.9649	921.5028	616.0182	731.5614	1236.158	1216.245	1083.075	1071.891	1357.071	850.6784	978.9838	591.9564	714.3579	1010.248	1309.506
AC004986.	0	0	0	0	0	0	0	1.852054	0	0	0	0.920078	1.028084	0	0.978006	0.824452	2.052753	0	0
AC004987.	48.04538	58.02265	29.97401	29.1002	57.58922	35.3985	62.87634	51.85751	85.212	63.40615	62.64725	78.20665	44.20763	53.05106	41.07624	65.95615	67.74083	60.06881	80.66772
AC004987.	0	0	0	2.078586	0	0	0.84968	0	0	2.882098	7.228529	0	5.140422	4.653602	0	1.648904	1.026376	0.910133	1.792616
AC004987.	3.84363	5.001952	3.996535	0	1.107485	0	2.549041	2.778081	0	1.921399	1.204755	5.520469	1.028084	3.722881	2.934017	11.54233	4.105505	5.4608	0.896308
AC004988.	15.37452	1.00039	1.998267	2.078586	6.64491	0	4.248401	0	1.167288	11.52839	0	10.12086	0	16.75297	4.890029	21.43575	7.184634	6.370934	8.066772
AC004990.	7.68726	10.0039	7.993069	0	14.3973	6.851322	5.947762	0	1.167288	3.842797	0	16.56141	8.224675	9.307203	3.912023	5.771163	28.73854	3.640534	10.7557
AC004994.	0.960908	0	0	0	3.322455	0	0	5.556162	0	0	0	0	1.028084	0	0	0	0	0	0
AC004997.	9.609075	13.00508	4.995668	3.117878	11.07485	13.70264	1.69936	8.334243	33.85134	31.70308	81.92333	6.440548	13.3651	2.792161	2.934017	4.122259	3.079129	6.370934	8.066772
AC005000.	22.10087	15.00586	7.993069	16.62868	17.71976	15.98642	13.59488	29.63287	28.01491	9.606993	13.2523	15.64133	16.44935	13.9608	23.47214	10.71787	19.50115	22.75334	15.23724
AC005000.	0	0	0	0	2.21497	0	0	0	0	0.960699	2.40951	0	0	0	0	0	6.158258	0.910133	0
AC005005.	0	0	0.999134	0	6.64491	0	0	0	7.003726	0.960699	0	4.600391	0	0	0	0	0	0	0
AC005005.	0	0	0	0	0	3.425661	1.69936	10.1863	0	2.882098	0	1.840156	3.084253	4.653602	0.978006	1.648904	1.026376	0	1.792616
AC005005.	259.445	276.1078	316.7254	227.6051	197.1323	285.4718	229.4137	241.6931	670.0232	508.2099	448.1688	264.9825	230.2909	245.7102	231.7874	193.7462	179.6158	232.9942	271.5813
AC005005.	11.53089	15.00586	2.997401	10.39293	4.42994	12.56076	8.496802	12.96438	10.50559	9.606993	16.86657	1.840156	6.168506	4.653602	5.868035	0.824452	1.026376	11.83173	6.274156
AC005006.	22.10087	13.00508	7.993069	16.62868	34.33203	3.425661	8.496802	8.334243	10.50559	2.882098	0	14.72125	7.196591	21.40657	17.6041	15.66458	17.4484	15.47227	8.066772
AC005009.	40.35812	13.00508	17.98441	48.84676	13.28982	31.97284	21.24201	15.74246	3.501863	13.44979	14.45706	18.40156	21.58977	23.26801	16.6261	18.96239	21.5539	13.652	11.652
AC005009.	10.56998	4.001562	18.98354	22.86444	25.47215	28.54718	19.54265	18.52054	38.5205	24.97818	32.52838	23.00196	12.33701	24.19873	26.40616	18.13794	27.71216	26.39387	34.95601
AC005013.	15.37452	5.001952	24.97834	3.117878	1.107485	1.141887	9.346483	16.66849	2.334575	15.37119	26.50461	9.200782	8.224675	11.16864	19.56012	0	9.237386	10.9216	9.859387
AC005014.	3.84363	4.001562	1.998267	6.235757	0	2.283774	1.69936	5.556162	17.50932	12.48909	7.228529	9.200782	7.196591	3.722881	7.824047	6.595615	11.29014	2.7304	0
AC005014.	22.10087	45.01757	26.97661	76.90766	85.27634	90.20907	47.58209	35.18903	194.9371	129.6944	142.1611	74.52634	55.51656	44.67457	89.97654	56.88718	61.58258	35.4952	16.13354
AC005014.	7.68726	12.00469	24.97834	8.314342	2.21497	3.425661	10.19616	19.44657	30.34948	6.724895	12.04755	9.200782	10.28084	18.61441	13.69208	12.36678	3.079129	26.39387	14.34093
AC005019.	0.960908	1.00039	5.994802	0	1.107485	6.851322	0	0	5.836439	0	1.204755	0	1.028084	3.722881	0.978006	5.771163	0	2.7304	0.896308
AC005019.	0	0	0	0	0	0	0.84968	0	1.167288	0	0	1.840156	1.028084	0	0	0	0	0	0
AC005020.	0.960908	13.00508	0.999134	2.078586	5.537425	0	0	3.704108	0	1.921399	1.204755	7.360626	6.168506	0	1.956012	5.771163	0	1.820267	0.896308
AC005021.	15.37452	13.00508	29.97401	15.58939	50.94431	7.993209	38.23561	24.0767	5.836439	29.78168	22.89034	19.32164	33.92678	25.12945	24.45015	16.48904	16.42202	30.0344	40.33386
AC005033.	0	0	0	0	8.859879	0	0	0	0	0	0	0.920078	0	0	0	0	0	0	0.896308
AC005034.	43.24084	32.01249	26.97661	19.74656	46.51437	38.82416	28.88913	25.92876	64.20083	44.19217	65.05676	41.40352	19.5336	26.06017	22.49413	18.96239	16.42202	14.56213	30.47447
AC005034.	1180.955	1055.412	931.1926	1475.796	1213.803	1220.677	1017.067	1173.276	733.0567	990.4809	738.5147	1023.127	1037.337	985.6328	1098.301	1145.988	848.8132	981.1238	831.7738
AC005034.	36.51449	34.01327	33.97054	62.35757	43.19191	47.95925	48.43177	38.89314	29.18219	48.99566	30.11887	34.96297	52.4323	40.02097	71.39443	58.53608	40.02867	22.75334	59.15632
AC005034.	228.696	173.0675	166.8553	199.5442	152.8329	175.8506	211.5704	165.7588	39.68778	103.7555	51.80446	124.2106	182.999	141.4695	164.305	148.4013	105.7168	129.2389	155.9576
AC005035.	74.95079	55.02147	95.91683	23.90373	14.3973	46.81737	39.93497	18.52054	45.52422	2.882098	42.16642	40.48344	40.09529	126.578	96.82258	270.4202	118.0333	175.6557	153.2687
AC005040.	0	0	0	4.157171	0	0	0	0	0	0	0	0	0	0	0	0	0	0	0
AC005041.	88.40349	92.03592	82.92809	117.4401	50.94431	89.06719	113.8572	114.8274	81.71014	56.68126	54.21397	111.3295	117.2016	111.6864	69.43841	148.4013	153.9564	81.00187	85.14926
AC005041.	5.765445	14.00547	7.993069	14.5501	12.18233	2.283774	7.647122	5.556162	9.338302	12.48909	7.228529	11.96102	8.224675	5.584322	0.978006	6.595615	12.31652	5.4608	8.066772
AC005041.	34.59267	9.003514	34.96968	17.66798	43.19191	37.68227	16.9936	28.70684	10.50559	26.89958	54.21397	30.36258	22.61786	38.15953	24.45015	35.45143	25.65941	43.6864	27.78555
AC005042.	6.726353	7.002733	6.993936	3.117878	15.50479	1.141887	8.496802	0	2.334575	0	6.023774	2.760235	3.084253	18.61441	12.71408	9.06897	9.237386	1.820267	9.859387

AC005042.	0	0	0	0	1.107485	0	0	0	0	0	0	0	0	0	0	0.978006	0	0	0	0
AC005046.	372.8321	553.2159	943.1822	972.778	1053.218	448.7616	1275.37	315.7752	963.0124	726.2886	878.2663	489.4816	513.0141	578.908	683.6261	864.85	758.492	1002.057	1007.45
AC005050.	8.648168	15.00586	22.98007	11.43222	11.07485	20.55397	14.44456	13.89041	26.84762	23.05678	49.39495	8.280704	6.168506	28.85233	28.36217	19.78684	15.39564	5.4608	3.585232
AC005052.	23.06178	63.0246	19.98267	12.47151	37.65449	47.95925	45.03305	19.44657	38.5205	42.27077	39.75691	19.32164	19.5336	31.64449	30.31818	23.08465	29.76491	31.85467	61.84525
AC005056.	53.81082	19.00742	29.97401	27.02161	39.86946	45.67548	34.83689	51.85751	71.20455	61.48475	74.6948	31.28266	34.95487	37.22881	60.63636	20.6113	52.34519	64.61947	64.53417
AC005062.	50.9281	26.01015	37.96708	13.51081	15.50479	4.567548	14.44456	36.11505	49.02608	3.842797	7.228529	10.12086	8.224675	17.68369	29.34017	14.01568	14.36927	7.281067	1.792616
AC005064.	62.45899	85.03319	68.94022	195.387	39.86946	115.3306	65.42538	39.81916	42.02236	71.09174	67.46627	105.809	107.9489	87.48771	119.3167	122.8433	118.0333	30.0344	46.60801
AC005070.	0	0	7.993069	0	0	0	0	0	14.00745	0	0	0	1.028084	0	0	0	0	0	0.896308
AC005070.	2.882723	2.000781	0	4.157171	0	0	1.69936	0	1.167288	16.33189	6.023774	0.920078	2.056169	0	5.868035	1.648904	4.105505	11.83173	7.170464
AC005070.	698.5798	749.2924	1003.13	878.2024	780.7769	738.8009	1104.584	629.6984	605.8223	698.4284	427.688	747.1035	847.1415	636.6127	744.2624	592.7809	709.226	636.1833	839.8405
AC005072.	6.726353	4.001562	2.997401	2.078586	0	0	5.947762	0	7.003726	5.764196	4.81902	0.920078	6.168506	0	7.824047	0	4.105505	0.910133	1.792616
AC005076.	215.2433	213.0832	197.8285	133.0295	121.8233	95.91851	167.387	195.3917	270.8108	195.0219	181.918	221.7389	142.9037	188.0055	157.4589	168.1882	138.5608	161.0936	136.2388
AC005077.	0	1.00039	0	0	0	0	0.84968	0.926027	0	0	0	5.520469	1.028084	0.93072	0	0.824452	0	2.7304	0
AC005077.	27.86632	24.00937	3.996535	11.43222	38.76197	13.70264	22.94137	13.89041	1.167288	4.803496	12.04755	21.1618	27.75828	25.12945	26.40616	17.31349	23.60665	12.74187	14.34093
AC005077.	0	1.00039	3.996535	4.157171	0	1.141887	0	0	1.167288	0.960699	0	0.920078	0	0	1.956012	0.824452	0	0.910133	0
AC005077.	2.882723	10.0039	0	3.117878	6.64491	6.851322	8.496802	13.89041	37.35321	14.41049	28.91412	6.440548	10.28084	5.584322	1.956012	9.06897	11.29014	12.74187	29.57816
AC005082.	70.14625	72.02811	99.91337	97.69352	159.4778	129.0332	265.1002	74.08216	178.595	48.03496	16.86657	48.76415	49.34805	90.27987	122.2507	98.93422	105.7168	250.2867	112.9348
AC005083.	7.68726	11.00429	11.9896	19.74656	6.64491	4.567548	5.098081	6.482189	8.171014	17.29259	0	8.280704	2.056169	29.78305	18.58211	13.19123	17.4484	14.56213	13.44462
AC005084.	0	0	0	1.039293	0	0	0.84968	0	0	0	0	0	0	0.93072	0	0	0	0	7.170464
AC005086.	0	1.00039	4.995668	0	1.107485	0	1.69936	10.1863	3.501863	0	0	1.840156	1.028084	0.93072	0.978006	0	1.026376	2.7304	0.896308
AC005086.	3.84363	0	3.996535	3.117878	0	4.567548	0	3.704108	1.167288	1.921399	2.40951	0	0	0.93072	0	0	0	0	0
AC005086.	3.84363	0	0.999134	0	3.322455	3.425661	0	0	0	0	0	0	0	0	0	0	0	0.910133	0
AC005086.	0	0	12.98874	0	16.61227	0	2.549041	2.778081	0	2.882098	4.81902	6.440548	3.084253	1.861441	2.934017	2.473355	2.052753	0.910133	1.792616
AC005088.	0	1.00039	0.999134	0	1.107485	0	0	5.556162	0	0	0	0	4.112337	0	0.978006	0	1.026376	0	0
AC005089.	11.53089	14.00547	29.97401	62.35757	34.33203	44.53359	22.94137	75.93422	1.167288	38.42797	27.70936	11.96102	20.56169	33.50593	39.12023	21.43575	10.26376	16.3824	27.78555
AC005091.	10.56998	0	7.993069	9.353635	16.61227	3.425661	23.79105	16.66849	4.669151	9.606993	0	13.80117	22.61786	5.584322	1.956012	9.06897	2.052753	16.3824	5.377848
AC005096.	4.804538	4.001562	25.97748	4.157171	0	6.851322	6.797442	0	4.669151	8.646293	0	14.72125	8.224675	7.445762	0.978006	8.244518	6.158258	7.281067	0.896308
AC005099.	0	2.000781	0.999134	0	0	0	0	5.556162	0	0	0	0	1.028084	0	0	0	1.026376	0	0.896308
AC005102.	0	0	0	0	0	0	0	0	0	5.764196	0	0.920078	0	0	4.890029	0	0	1.820267	0
AC005104.	187.377	313.1222	364.6838	181.8762	269.1188	179.2763	282.9435	257.4355	105.0559	231.5285	218.0606	358.8305	330.0151	150.7767	211.2493	187.1506	188.8532	250.2867	194.4988
AC005104.	13.45271	9.003514	5.994802	15.58939	8.859879	20.55397	22.09169	11.11232	11.67288	8.646293	6.023774	27.60235	13.3651	5.584322	3.912023	7.420066	6.158258	3.640534	6.274156
AC005105.	0	0	0	1.039293	0	0	0	0	0	0	0	2.760235	1.028084	0	0	0	0	5.4608	0
AC005144.	0.960908	1.00039	0.999134	0	1.107485	6.851322	0	0	0	0.960699	0	0	0	0	0	0	0	0.910133	0
AC005150.	0	5.001952	0	0	0	0	0	1.852054	0	0	0	0	0	0	0.978006	2.473355	0	0	3.585232
AC005154.	12425.5	6876.684	9895.42	8845.421	13459.26	13556.48	9831.65	10846.55	10398.2	17451.1	17950.85	11736.52	11214.34	8218.26	9115.992	8724.349	8233.59	13504.56	12196.96
AC005154.	41.31902	10.0039	42.96275	36.37525	35.43952	17.12831	31.43817	21.29862	21.01118	17.29259	24.0951	34.96297	19.5336	31.64449	49.8783	15.66458	26.68578	23.66347	41.23017
AC005154.	708.1888	483.1886	782.3217	506.1356	676.6733	550.3895	582.031	691.7422	612.8261	723.4065	814.4143	657.8559	568.5307	565.8779	552.5733	424.5927	410.5505	495.1126	605.9042
AC005154.	44.20175	27.01054	43.96188	42.611	63.12664	42.24982	45.88273	49.07943	3.501863	53.79916	28.91412	52.44446	43.17954	53.05106	57.70234	33.80253	34.89679	59.15867	50.19325
AC005156.	2.882723	4.001562	3.996535	4.157171	2.21497	2.283774	0.84968	1.852054	0	1.921399	12.04755	5.520469	2.056169	13.9608	2.934017	7.420066	2.052753	2.7304	0.896308
AC005162.	0	0	3.996535	0	0	0	0.84968	0	0	0	0	4.600391	1.028084	1.861441	0.978006	0	0	8.191201	0.896308
AC005162.	0.960908	1.00039	2.997401	5.196464	0	4.567548	3.398721	0	0	0	0	1.840156	6.168506	0	0.978006	0	1.026376	0.910133	0.896308
AC005162.	295.9595	72.02811	177.8458	136.1474	134.0057	98.20228	101.1119	237.9889	213.6137	179.6508	204.8083	234.6199	253.9368	134.0237	163.327	110.4765	79.03097	88.28294	60.94894
AC005165.	361.3012	203.0793	384.6665	245.2731	364.3625	211.2491	185.2303	388.9314	269.6435	326.6377	181.918	222.6589	180.9428	263.3938	316.8739	212.7086	224.7764	359.5027	231.2475
AC005165.	0	0	0	0	0	0	0	0	0	0	0	0	1.028084	0	0.978006	0	0	0	0
AC005176.	0	1.00039	0	0	0	0	0	0	0	0	0	0	7.196591	0	0	0	1.026376	1.820267	0
AC005180.	34.59267	29.01132	7.993069	13.51081	23.25718	15.98642	22.09169	6.482189	17.50932	7.685594	26.50461	19.32164	6.168506	34.43665	41.07624	8.244518	31.81766	2.7304	8.963079
AC005180.	52.84991	14.00547	39.96535	35.33595	34.33203	18.27019	30.58849	31.48492	35.01863	23.05678	30.11887	18.40156	24.67402	46.53602	60.63636	14.84013	44.13418	18.20267	17.02985
AC005181.	0	0	0	0	0	0	0	0	0	0	0	0	0	0	0	0	0	0	5.377848
AC005183.	4.804538	8.003124	8.992203	23.90373	15.50479	3.425661	30.58849	12.96438	19.84389	13.44979	18.07132	11.96102	16.44935	16.75297	6.846041	23.9091	17.4484	8.191201	7.170464
AC005185.	0	0	10.99047	0	0	0	0	0	0	0	0	0	0	0	0	0	0	5.4608	0
AC005186.	45.16265	18.00703	8.992203	5.196464	4.42994	0	2.549041	6.482189	0	5.764196	2.40951	12.8811	19.5336	2.792161	0.978006	9.893422	4.105505	12.74187	20.61508
AC005189.	0.960908	1.00039	3.996535	0	0	0	0	2.778081	0	2.882098	0	0.920078	0	3.722881	0	0.824452	1.026376	0	0
AC005197.	94.16894	144.0562	317.7245	306.5914	490.6158	202.114	112.1578	267.6218	134.2381	413.1007	480.6972	219.8987	198.4203	160.0839	142.7889	211.8841	238.1193	395.908	627.4156
AC005208.	6.726353	2.000781	3.996535	3.117878	1.107485	10.27698	5.098081	0.926027	0	2.882098	0	7.360626	7.196591	6.515042	4.890029	8.244518	6.158258	0	0.896308
AC005209.	0.960908	0	0	2.078586	0	2.283774	0	0	4.669151	0	0	0	1.028084	0.93072	2.934017	1.648904	1.026376	0	0
AC005220.	31.70995	49.01913	29.97401	28.0609	49.83682	19.41208	48.43177	42.59724	8.171014	26.89958	3.614265	47.84407	52.4323	33.50593	35.20821	17.31349	10.26376	37.31547	57.36371
AC005224.	112.4262	83.03241	104.909	86.2613	138.4356	97.0604	107.0597	128.7178	225.2865	155.6333	120.4755	95.68814	106.9208	107.0328	102.6906	101.4076	108.7959	90.10321	73.49725
AC005224.	0.960908	1.00039	3.996535	0	1.107485	0	0	2.778081	7.003726	0	0	1.840156	6.168506	0	0	1.648904	6.158258	0	5.377848
AC005224.	1.921815	7.002733	0	13.51081	16.61227	7.993209	1.69936	6.482189	77.04099	10.56769	20.48083	1.840156	0	12.09936	23.47214	5.771163	13.34289	5.4608	1.792616
AC005224.	149.9016	204.0796	132.8848	341.9273	79.73891	235.2287	185.2303	101.863	50.19337	164.2796	132.523	166.5342	180.9428	141.4695	183.8651	312.4672	284.3062	98.29441	197.1877
AC005225.	169.1197	134.0523	122.8934	115.3615	40.97694	99.34417	165.6876	137.052	213.6137	157.5547	109.6327	131.5712	151.1284	93.07203	127.1408	79.14738	78.0046	120.1376	133.5499
AC005225.	15.37452	16.00625	15.98614	15.58939	12.18233	10.27698	16.9936	15.74246	29.18219	8.646293	12.04755	15.64133	17.47743	14.89152	16.6261	12.36678	14.36927	16.3824	17.92616

AC005225.	95.12985	51.01991	79.93069	62.35757	21.04221	81.07398	101.1119	25.92876	171.5913	73.01314	60.23774	79.12673	94.58376	80.97267	86.06451	42.04704	44.13418	104.6653	150.5797
AC005225.	0	0	0	0	3.322455	0	0	0	0	0	0	0	0	3.722881	0	0	0	0	0
AC005225.	461.2356	439.1714	522.5469	526.9214	344.4278	222.668	469.8732	518.5751	813.5995	588.9086	646.9534	472.9202	434.8797	349.0201	422.4985	398.2102	401.3131	413.2006	458.9097
AC005229.	0	1.00039	0	0	0	0	0	1.852054	0	0	0	0	0	0	0	0	0	0	0.896308
AC005229.	0	0	0	0	0	0	0	0	0	0	0	0	0	0	0	0	0	0	3.585232
AC005229.	195.0642	293.1144	263.7713	307.6307	237.0018	267.2016	319.4798	301.8848	568.4691	315.1094	345.7646	299.0254	252.9088	236.403	262.1056	222.602	241.1984	235.7246	276.0628
AC005230.	0.960908	7.002733	1.998267	2.078586	14.3973	1.141887	8.496802	6.482189	0	12.48909	2.40951	12.8811	14.39318	0	2.934017	0	1.026376	0.910133	3.585232
AC005234.	0	0	0	1.039293	1.107485	0	2.549041	0.926027	0	0	4.81902	1.840156	2.056169	0	0	3.297807	0	0	0.896308
AC005237.	9.609075	23.00898	5.994802	21.82515	0	4.567548	7.647122	8.334243	23.34575	3.842797	13.2523	12.8811	33.92678	15.82225	10.75806	22.2602	10.26376	46.4168	23.30401
AC005237.	0	0	0	0	0	0	0	0	0	2.882098	0	0	0	0	0	0	0	0	0
AC005242.	0	0	0	0	0	0	0	0	1.167288	0	0	0.920078	0	0	0	0	0	0	0
AC005244.	0.960908	16.00625	14.987	1.039293	9.967364	18.27019	13.59488	15.74246	16.34203	24.97818	24.0951	9.200782	26.73019	6.515042	11.73607	0.824452	12.31652	1.820267	7.170464
AC005244.	1.921815	0	0	4.157171	0	0	0	0	0	0	0	0	0	0	5.868035	0	0	1.820267	6.274156
AC005252.	86.48168	116.0453	68.94022	70.67191	49.83682	77.64832	172.4851	148.1643	108.5578	55.72056	65.05676	45.08383	86.35909	85.62627	61.61437	51.11601	48.23968	36.40534	32.26709
AC005253.	840.7941	612.2389	856.2575	1331.334	632.3739	612.0514	751.1173	724.1531	178.595	677.293	619.244	942.1601	930.4164	747.3684	660.1539	689.2417	655.8544	901.0321	826.3959
AC005261.	157.5888	183.0715	250.7825	56.12181	202.6697	172.4249	192.0277	191.6876	240.4613	262.2709	406.0024	219.8987	198.4203	176.8369	202.4472	131.9123	163.1938	200.2294	215.1139
AC005261.	11750.94	10593.13	10350.03	12112.96	8492.194	10416.29	10251.39	9837.185	6539.146	10735.81	9098.309	8072.766	9879.891	10411.97	11679.35	12490.45	12168.72	11126.38	8863.589
AC005261.	31.70995	63.0246	46.95928	45.72888	34.33203	9.135096	39.93497	37.96711	52.52795	60.52405	50.5997	41.40352	22.61786	50.2589	60.63636	57.71163	39.0023	44.59654	70.80833
AC005261.	658.2217	809.3159	797.3087	605.9077	823.9688	1004.861	588.8284	736.1915	1606.188	1252.752	1407.154	597.1308	607.5979	871.1542	777.5146	643.0724	644.5643	664.3974	468.7691
AC005261.	0	0	0	0	0	1.141887	0.84968	0	0	0	0	0	0	0	0	0	0	0	0
AC005261.	21.13997	22.00859	76.93329	33.25737	49.83682	41.10793	74.77186	67.59997	58.36439	36.50657	18.07132	96.60821	44.20763	67.94258	33.2522	43.69595	37.97592	40.04587	28.68185
AC005262.	0	0	0	0	0	0	0.84968	0	0	1.921399	0	0.920078	1.028084	0	0	0	0	0.910133	0
AC005264.	10.56998	12.00469	13.98787	30.13949	15.50479	7.993209	13.59488	24.0767	24.51304	14.41049	6.023774	21.1618	21.58977	13.03008	13.69208	44.5204	30.79129	103.7552	65.43048
AC005274.	36.51449	35.01367	31.97228	32.21808	23.25718	25.12151	32.28785	18.52054	63.03354	27.86028	33.73314	34.96297	31.87062	26.99089	44.01026	42.04704	27.71216	29.12427	25.09662
AC005277.	15.37452	27.01054	6.993936	48.84676	13.28982	41.10793	45.88273	18.52054	10.50559	14.41049	14.45706	22.08188	24.67402	23.26801	20.53812	23.9091	18.47477	29.12427	14.34093
AC005280.	4.804538	3.001171	5.994802	8.314342	1.107485	4.567548	4.248401	11.11232	2.334575	3.842797	4.81902	5.520469	10.28084	3.722881	0.978006	4.946711	7.184634	6.370934	5.377848
AC005281.	0.960908	3.001171	11.9896	0	5.537425	0	4.248401	8.334243	0	5.764196	27.70936	9.200782	15.42127	6.515042	7.824047	4.122259	1.026376	3.640534	0
AC005288.	1549.944	1365.533	1613.601	1569.332	1564.876	1363.413	1395.175	1518.684	1277.013	1435.285	1246.921	1322.152	1313.892	1588.74	1454.295	1583.772	1410.241	1622.768	1355.218
AC005291.	4.804538	0	0	0	1.107485	0	1.69936	5.556162	0	1.921399	1.204755	0	1.028084	0.93072	0.978006	0	1.026376	0	0.896308
AC005291.	0	0	0.999134	2.078586	2.21497	4.567548	6.797442	5.556162	14.00745	0.960699	4.81902	1.840156	4.112337	0	9.780058	2.473355	1.026376	1.820267	0.896308
AC005294.	0	0	0	0	0	0	0	0	0	0	0	1.840156	0	0	0	0	0	0	0
AC005296.	0	0	3.996535	0	0	0	0	0	0	1.921399	0	0	0	0	0.978006	0	0	0.910133	0
AC005300.	11.53089	4.001562	7.993069	9.353635	13.28982	1.141887	11.89552	3.704108	14.00745	35.54587	0	11.04094	11.30893	30.71377	9.780058	15.66458	5.131881	15.47227	7.170464
AC005303.	0	2.000781	0	0	0	0	2.549041	0.926027	0	0	0	0	0	0.93072	0	0	0	0	0
AC005304.	5.765445	9.003514	3.996535	0	16.61227	1.141887	0	7.408216	0	0	3.614265	0.920078	6.168506	2.792161	6.846041	20.6113	5.131881	17.29253	0.896308
AC005304.	3.84363	0	0	4.157171	0	0	2.549041	0.926027	0	4.803496	0	0.920078	0	0	1.956012	0	0	8.191201	0
AC005304.	1.921815	0	2.997401	1.039293	3.322455	0	1.69936	0.926027	0	0	0	0.920078	0	5.584322	1.956012	4.122259	0	0	4.48154
AC005306.	12.4918	8.003124	9.991337	5.196464	5.537425	13.70264	1.69936	4.630135	19.84389	8.646293	38.55216	4.600391	9.252759	6.515042	7.824047	4.946711	4.105505	7.281067	4.48154
AC005307.	12.4918	0	0	12.47151	6.64491	20.55397	2.549041	3.704108	0	5.764196	7.228529	4.600391	0	0	0	7.420066	7.184634	0	0
AC005324.	12.4918	7.002733	1.998267	29.1002	0	3.425661	0	3.704108	0	8.646293	0	0	0	3.722881	1.956012	0	0	0.910133	31.37078
AC005324.	78.79442	26.01015	27.97574	21.82515	39.86946	51.38492	11.04584	25.00273	42.02236	47.07426	26.50461	57.96493	12.33701	35.36737	25.42815	28.03136	9.237386	37.31547	37.64493
AC005324.	3.84363	4.001562	2.997401	5.196464	23.25718	4.567548	1.69936	15.74246	11.67288	4.803496	10.84279	7.360626	1.028084	6.515042	7.824047	4.946711	11.29014	4.550667	2.688924
AC005326.	1.921815	2.000781	4.995668	5.196464	7.752394	6.851322	1.69936	6.482189	18.6766	1.921399	13.2523	7.360626	7.196591	1.861441	1.956012	7.420066	2.052753	9.101334	3.585232
AC005329.	11.53089	7.002733	13.98787	0	5.537425	11.41887	3.398721	17.59451	92.21573	33.62447	87.94711	18.40156	27.75828	12.09936	10.75806	9.893422	5.131881	19.1128	15.23724
AC005329.	18.25724	24.00937	17.98441	15.58939	19.93473	23.97963	16.9936	29.63287	9.338302	27.86028	10.84279	44.16376	29.81445	37.22881	15.64809	17.31349	18.47477	30.0344	16.13354
AC005330.	119.1525	232.0906	129.8874	140.3045	139.5431	197.5465	150.3934	218.5424	333.8443	240.1748	392.7501	126.0507	162.4373	122.8551	103.6686	117.0722	176.5367	217.5219	308.3299
AC005332.	131.6443	159.0621	217.8111	240.0766	111.856	135.8846	226.0149	129.6438	248.6323	164.2796	272.2746	144.4523	164.4935	207.5506	218.0953	223.4264	242.2248	266.6691	185.5357
AC005332.	82.63805	87.03397	96.91597	65.47544	63.12664	75.36454	107.0597	106.4931	103.8886	83.58083	32.52838	82.80704	108.9769	53.05106	70.41642	42.04704	48.23968	38.2256	42.12647
AC005332.	0	0	0	0	0	1.141887	0	0	0	0	0	0	0	0	0	0	0	1.820267	0.896308
AC005332.	36.51449	39.01523	63.94455	35.33595	48.72934	61.6619	76.47122	34.263	16.34203	54.75986	45.78069	70.84602	75.05016	42.81313	65.52639	30.50472	26.68578	43.6864	49.29694
AC005332.	458.3529	403.1573	490.5746	301.3949	327.8155	298.0325	408.6962	488.9423	51.36066	513.9741	331.3076	400.234	473.9469	246.6409	314.9179	244.0377	236.0665	332.1987	289.5075
AC005332.	599.6063	741.2893	607.4733	694.2476	776.3469	669.1458	660.2015	537.0957	623.3316	695.5463	566.2348	526.2847	561.3341	530.5106	606.3636	568.0473	612.7466	485.1011	468.7691
AC005332.	1194.408	1017.397	1743.488	673.4617	1242.598	1180.711	841.1834	1235.32	2186.33	1408.385	1860.142	1092.133	1352.959	1479.845	1574.589	922.5616	981.2157	1504.451	1403.618
AC005332.	790.8269	673.2628	708.3858	554.9823	1003.381	911.2258	556.5406	844.5367	1098.418	1011.616	1614.372	925.5987	886.2087	658.95	660.1539	665.3326	1021.244	854.6153	789.6473
AC005332.	3629.348	3803.484	3609.87	4691.368	3675.742	3880.132	3748.789	3612.431	4030.645	3703.496	3726.307	3401.529	3681.57	3520.915	3725.224	4332.494	4034.685	3660.557	3518.009
AC005336.	255.6014	554.2163	302.7375	255.666	11.07485	33.11472	495.3636	590.8052	165.7549	400.6116	384.3168	46.92399	34.95487	291.3155	311.9839	273.718	157.0356	67.34987	71.70464
AC005336.	0	0	0	0	0	0	1.141887	0	0	4.803496	0	0	0	0	0	0	0	0	0
AC005336.	0	13.00508	0	1.039293	0	0	2.549041	8.334243	0	0.960699	14.45706	0	0	0	3.912023	0	1.026376	0	0
AC005342.	26.90541	5.001952	17.98441	2.078586	9.967364	2.283774	14.44456	0	8.171014	6.724895	1.204755	6.440548	8.224675	12.09936	18.58211	8.244518	5.131881	7.281067	3.585232
AC005342.	59.57627	39.01523	45.96015	53.00393	19.93473	61.6619	19.54265	56.48765	17.50932	29.78168	31.32363	53.36454	35.98295	23.26801	12.71408	24.73355	41.05505	178.3861	67.2231
AC005344.	0	0	0	0	6.64491	1.141887	0.84968	0.926027	0	5.764196	0	0	2.056169	0.93072	0	0	1.026376	0	1.792616
AC005355.	1.921815	1.00039	0	2.078586	0	6.851322	12.7452	10.1863	0	0	0	14.72125	7.196591	13.03008	0	4.122259	1.026376	3.640534	5.377848

AC005355.	0	6.002343	22.98007	9.353635	5.537425	7.993209	6.797442	22.22465	8.171014	1.921399	54.21397	1.840156	15.42127	7.445762	1.956012	3.297807	7.184634	17.29253	3.585232
AC005357.	0	2.000781	0.999134	0	6.64491	2.283774	2.549041	2.778081	0	1.921399	4.81902	1.840156	0	0	0.978006	0	4.105505	2.7304	0.896308
AC005357.	49.00628	30.01171	10.99047	17.66798	46.51437	31.97284	45.03305	31.48492	37.35321	30.74238	53.00921	10.12086	28.78636	21.40657	28.36217	13.19123	21.5539	36.40534	28.68185
AC005358.	83.59896	95.03709	78.93156	130.9509	60.91167	100.4861	169.936	76.86024	85.212	98.95202	54.21397	90.16767	161.4092	184.2826	129.0968	116.2477	80.05735	100.1147	157.7502
AC005363.	3.84363	1.00039	3.996535	0	7.752394	5.709435	0.84968	12.03835	17.50932	12.48909	24.0951	8.280704	4.112337	3.722881	0.978006	1.648904	9.237386	6.370934	8.066772
AC005377.	0.960908	1.00039	0.999134	4.157171	0	0	0.84968	0	0	0	4.81902	0.920078	0	0.93072	0	0.824452	3.079129	0.910133	0
AC005379.	0.960908	2.000781	0	4.157171	0	1.141887	0	0	1.167288	4.803496	3.614265	5.520469	0	0.93072	5.868035	5.771163	1.026376	14.56213	1.792616
AC005381.	1.921815	0	0	19.74656	4.42994	1.141887	1.69936	2.778081	0	0	2.40951	0	1.028084	0	0	2.473355	5.131881	0	0
AC005383.	142.2143	143.0558	156.864	170.444	277.9787	170.1412	218.3678	125.9397	673.525	365.0657	502.3828	145.3724	168.6058	85.62627	83.1305	41.22259	92.37386	178.3861	148.7871
AC005387.	36.51449	32.01249	13.98787	10.39293	33.22455	52.5268	29.73881	49.07943	191.4352	64.36685	62.64725	33.12282	41.12337	26.06017	35.20821	32.15362	40.02867	70.08027	34.95601
AC005387.	97.05166	100.039	94.9177	44.68959	69.77155	78.7902	104.5107	111.1232	91.04844	115.2839	124.0898	97.52829	88.41526	99.58707	96.82258	75.02512	75.95184	111.0363	62.74156
AC005391.	3.84363	3.001171	0.999134	3.117878	2.21497	6.851322	3.398721	2.778081	3.501863	6.724895	3.614265	8.280704	0	1.861441	0.978006	0.824452	3.079129	0.910133	4.48154
AC005392.	0	1.00039	0	0	0	1.141887	1.69936	0	0	0	0	0	2.056169	0	0	0.824452	0	0	0
AC005392.	0.960908	3.001171	7.993069	3.117878	0	3.425661	14.44456	4.630135	4.669151	0	0	4.600391	2.056169	22.33729	8.802052	28.85581	11.29014	0	0
AC005392.	0	10.0039	0.999134	8.314342	0	0	4.248401	7.408216	14.00745	0	3.614265	0.920078	4.112337	0.93072	4.890029	12.36678	9.237386	1.820267	0
AC005393.	98.01257	139.0543	143.8752	82.10413	170.5527	196.4046	112.1578	180.5753	394.5433	301.6596	269.8651	115.9299	123.3701	132.1623	129.0968	86.56744	72.87271	94.65388	108.4533
AC005394.	0	0	0	0	0	0	0	3.704108	0	0	4.81902	0	0	0	0	0	0	0	1.792616
AC005394.	43.24084	14.00547	22.98007	124.7151	15.50479	76.50643	9.346483	37.96711	31.51677	19.21399	53.00921	27.60235	33.92678	15.82225	23.47214	93.98751	91.34749	10.01147	34.0597
AC005410.	4.804538	2.000781	1.998267	9.353635	25.47215	1.141887	4.248401	12.03835	3.501863	0	0	6.440548	2.056169	4.653602	6.846041	1.648904	8.21101	5.4608	4.48154
AC005410.	39.39721	18.00703	24.97834	22.86444	16.61227	12.56076	26.34009	18.52054	26.84762	36.50657	7.228529	11.96102	13.3651	16.75297	23.47214	18.96239	20.52753	20.93307	19.71877
AC005412.	29.78813	53.02069	11.9896	30.13949	9.967364	17.12831	41.63433	26.85478	0	15.37119	7.228529	12.8811	17.47743	12.09936	25.42815	26.38246	40.02867	23.66347	7.170464
AC005412.	0	0	0	0	0	0	0	3.704108	0	0	0	0	0	0	0	0	0	0	0
AC005412.	4.804538	8.003124	4.995668	4.157171	0	2.283774	5.947762	0	2.334575	4.803496	6.023774	3.680313	5.140422	0.93072	0	0.824452	0	0.910133	3.585232
AC005476.	2.882723	1.00039	0	6.235757	39.86946	0	0	0	1.167288	0	0	0	3.084253	0.93072	0.978006	1.648904	0	0	6.274156
AC005476.	80.71623	36.01406	73.93589	43.6503	93.02873	61.6619	103.661	111.1232	108.5578	73.01314	67.46627	46.00391	84.30292	62.35826	55.74633	61.00944	60.5562	86.46267	81.56402
AC005479.	27.86632	6.002343	43.96188	73.78979	64.23413	23.97963	40.78465	19.44657	1.167288	17.29259	25.29985	28.52243	58.60081	34.43665	17.6041	95.63641	72.87271	101.9349	66.32679
AC005479.	118.1916	85.03319	49.95668	34.29666	107.426	73.08077	77.3209	134.2739	19.84389	64.36685	66.26152	147.2125	123.3701	122.8551	122.2507	29.68027	49.26606	80.09174	83.35664
AC005479.	38.4363	43.01679	33.97054	18.70727	57.58922	39.96605	14.44456	83.34243	98.05217	67.24895	69.87578	57.96493	88.41526	33.50593	66.5044	20.6113	19.50115	17.29253	25.99293
AC005480.	30.74904	47.01835	67.94109	21.82515	22.1497	28.54718	34.83689	38.89314	43.18965	43.23147	27.70936	35.88305	42.15146	55.84322	47.92229	28.85581	19.50115	34.58507	36.74863
AC005480.	27.86632	23.00898	8.992203	16.62868	11.07485	14.84453	14.44456	18.52054	11.67288	14.41049	14.45706	13.80117	9.252759	19.54513	13.69208	13.19123	23.60665	22.75334	17.02985
AC005481.	54.77173	32.01249	11.9896	30.13949	9.967364	36.54038	37.38593	29.63287	19.84389	9.606993	6.023774	69.92595	53.46039	73.5269	48.90029	34.62698	56.45069	43.6864	35.85232
AC005486.	2.882723	19.00742	2.997401	1.039293	1.107485	3.425661	1.69936	0.926027	7.003726	10.56769	1.204755	2.760235	3.084253	4.653602	1.956012	2.473355	2.052753	10.01147	20.61508
AC005487.	3.84363	0	0.999134	1.039293	0	5.709435	0.84968	0.926027	0	0	0	0	1.028084	0	0.978006	9.06897	2.052753	0	0.896308
AC005495.	196.0251	299.1167	697.3953	240.0766	233.6793	134.7427	470.7229	377.819	8.171014	162.3582	149.3896	363.4309	372.1665	412.3091	467.4868	263.0001	192.9587	202.9598	343.2859
AC005498.	37.47539	70.02733	43.96188	65.47544	45.40688	66.22945	62.87634	52.78354	96.88488	60.52405	67.46627	44.16376	45.23571	31.64449	31.29619	49.46711	43.1078	30.0344	39.43755
AC005498.	82.63805	108.0422	62.94542	57.1611	80.8464	97.0604	63.72602	85.19449	182.0969	85.50223	108.4279	57.04485	65.7974	46.53602	62.59237	76.67402	80.05735	41.86614	82.46033
AC005498.	230.6178	272.1062	257.7765	123.6758	214.8521	182.7019	110.4584	196.3177	405.0488	200.7861	297.5745	263.1424	252.9088	307.1377	241.5674	238.2666	246.3303	304.8947	245.5884
AC005514.	6.726353	0	2.997401	3.117878	2.21497	4.567548	5.947762	0	10.50559	3.842797	9.638039	5.520469	6.168506	4.653602	0	4.946711	1.026376	6.370934	2.688924
AC005517.	98.01257	87.03397	131.8856	95.61493	162.8003	92.49285	137.6482	204.652	66.5354	135.4586	74.6948	196.8967	171.6901	119.1322	115.4047	110.4765	105.7168	141.0707	136.2388
AC005517.	21.13997	13.00508	12.98874	16.62868	4.42994	5.709435	19.54265	18.52054	40.85507	4.803496	18.07132	13.80117	38.03912	1.861441	11.73607	9.06897	11.29014	8.191201	9.859387
AC005518.	0.960908	6.002343	4.995668	20.78586	4.42994	6.851322	6.797442	7.408216	2.334575	5.764196	3.614265	10.12086	14.39318	4.653602	7.824047	1.648904	6.158258	4.550667	1.792616
AC005519.	0	0	0	0	0	0	0	0	0	1.167288	0.960699	2.40951	0	0	0	0	0	0	1.792616
AC005519.	633.2381	672.2624	730.3667	590.3183	658.9535	649.7337	678.8945	656.5532	435.3983	639.8257	637.3153	682.698	847.1415	677.5644	763.8225	567.2229	587.0872	712.6345	644.4454
AC005520.	7.68726	10.0039	7.993069	2.078586	11.07485	0	3.398721	12.96438	0	3.842797	6.023774	1.840156	7.196591	4.653602	9.780058	0.824452	1.026376	13.652	9.859387
AC005520.	310.3731	75.02928	195.8302	81.06484	102.9961	334.5729	120.6546	270.3999	287.1528	687.8607	1001.151	326.6278	382.4474	136.8159	140.8328	107.1787	143.6927	111.0363	76.18618
AC005520.	18.25724	22.00859	15.98614	5.196464	11.07485	6.851322	5.098081	11.11232	11.67288	16.33189	1.204755	7.360626	10.28084	3.722881	1.956012	9.06897	12.31652	0.910133	9.859387
AC005520.	0	0	0	0	0	0	0	0	0	0	2.40951	0	1.028084	0	0	0	1.026376	0	0
AC005521.	0	0	0	1.039293	1.107485	0	0	0.926027	2.334575	0	2.40951	0.920078	0	0.93072	0.978006	0	0	0.910133	0
AC005522.	0	6.002343	0.999134	0	0	3.425661	6.797442	0	0	0	0	0	0	0.93072	0.978006	3.297807	0	3.640534	0
AC005522.	2.882723	1.00039	0	4.157171	1.107485	3.425661	0.84968	0.926027	5.836439	3.842797	2.40951	5.520469	3.084253	0	7.824047	3.297807	0	0	1.792616
AC005523.	0.960908	1.00039	1.998267	0	6.64491	4.567548	0	5.556162	1.167288	0.960699	0	6.440548	1.028084	0	3.912023	0	1.026376	4.550667	1.792616
AC005524.	0.960908	0	4.995668	1.039293	0	1.141887	0	2.778081	0	0	0	0.920078	4.112337	0	4.890029	0	0	1.820267	3.585232
AC005529.	1.921815	3.001171	1.998267	4.157171	2.21497	11.41887	6.797442	12.03835	1.167288	5.764196	1.204755	0	6.168506	9.307203	8.802052	6.595615	5.131881	4.550667	7.170464
AC005531.	42.27993	36.01406	32.97141	24.94303	24.36467	26.2634	45.03305	14.81643	44.35693	24.01748	1.204755	10.12086	6.168506	19.54513	16.6261	26.38246	27.71216	7.281067	31.37078
AC005532.	16.33543	44.01718	39.96535	40.53242	26.57964	59.37812	7.647122	42.59724	52.52795	79.73804	77.10431	44.16376	50.37613	33.50593	18.58211	28.85581	26.68578	10.01147	23.30401
AC005532.	1.921815	6.002343	0	0	2.21497	1.141887	1.69936	11.11232	0	1.921399	0	6.440548	1.028084	6.515042	0.978006	0.824452	1.026376	2.7304	0
AC005534.	9.609075	13.00508	23.97921	25.98232	26.57964	20.55397	5.098081	2.778081	15.17474	24.97818	34.93789	20.24172	23.64594	15.82225	21.51613	14.84013	6.158258	18.20267	19.71877
AC005534.	30.74904	65.02538	55.95149	20.78586	63.12664	61.6619	66.27506	74.08216	18.6766	76.85594	50.5997	34.04289	38.03912	46.53602	41.07624	17.31349	22.58028	22.75334	26.88924
AC005537.	26.90541	57.02225	16.98527	25.98232	32.11706	26.2634	32.28785	35.18903	47.8588	24.97818	93.97088	13.80117	17.47743	40.95169	26.40616	32.15362	25.65941	40.956	39.43755
AC005538.	0	0	0	0	0	6.851322	0.84968	0	0	0	0	0.920078	0	0	1.956012	0.824452	4.105505	0	0.896308
AC005540.	0	0	3.996535	0	6.64491	6.851322	2.549041	0.926027	0	0.960699	9.638039	3.680313	17.47743	1.861441	3.912023	4.122259	9.237386	10.01147	6.274156
AC005544.	1.921815	4.001562	6.993936	0	0	2.283774	2.549041	3.704108	0	10.56769	0	3.680313	8.224675	0.93072	0	4.122259	3.079129	4.550667	3.585232

AC005546.	24.02269	23.00898	19.98267	31.17878	17.71976	13.70264	25.49041	33.33697	4.669151	35.54587	20.48083	13.80117	24.67402	32.57521	15.64809	35.45143	24.63303	27.304	17.92616
AC005550.	2.882723	2.000781	25.97748	8.314342	9.967364	2.283774	5.098081	21.29862	4.669151	4.803496	1.204755	21.1618	15.42127	41.88241	20.53812	18.13794	13.34289	8.191201	8.066772
AC005550.	34.59267	40.01562	14.987	12.47151	28.79461	35.3985	20.39233	25.00273	35.01863	17.29259	46.98544	24.84211	10.28084	44.67457	9.780058	29.68027	13.34289	40.956	7.170464
AC005550.	131.6443	123.048	316.7254	172.5226	190.4874	86.78341	199.6749	274.104	70.03726	113.3625	84.33284	164.694	250.8526	90.27987	191.6891	169.0126	100.5849	157.4531	49.29694
AC005551.	0	4.001562	0	0	0	0	0	0	0	0	0	0	0	0	0	0	0	0	0
AC005552.	0	0	0.999134	0	14.3973	0	0.84968	0	0	0.960699	0	0.920078	6.168506	3.722881	0.978006	0	0	0	0
AC005562.	102.8171	56.02186	62.94542	53.00393	66.4491	53.66869	82.41898	53.70957	28.01491	29.78168	61.4425	74.52634	35.98295	56.77394	58.68035	56.06272	63.63533	77.36134	45.71171
AC005562.	0	0	0.999134	4.157171	0	0	0	0	0	0	0	0	0	0	0	0	0	0	0
AC005578.	15.37452	15.00586	12.98874	13.51081	31.00958	14.84453	6.797442	25.00273	46.69151	12.48909	14.45706	21.1618	27.75828	2.792161	8.802052	17.31349	17.4484	37.31547	22.4077
AC005580.	0.960908	0	0	0	0	0	0	0	0	0	0	0	0	0	0	0	0	0	0.896308
AC005586.	276.7414	191.0746	227.8025	371.0275	227.0344	213.5329	247.2569	166.6849	226.4538	262.2709	208.4226	188.616	211.7854	228.9572	248.4135	281.9625	274.0425	205.6902	195.3951
AC005586.	369.9494	269.105	220.8085	493.6641	220.3895	337.9986	393.402	262.0657	263.807	212.3145	92.76613	299.0254	295.0602	450.4686	448.9047	343.7964	363.3372	179.2963	303.8484
AC005587.	0	0	0	0	0	0	0	0	0	0	0	0	0	1.861441	0	0	0	0	0
AC005588.	0	0	3.996535	0	0	0	0.84968	3.704108	0	5.764196	0	0.920078	0	0	0	0	0	0	0.896308
AC005592.	7.68726	14.00547	4.995668	14.5501	19.93473	12.56076	13.59488	27.78081	21.01118	10.56769	4.81902	27.60235	22.61786	6.515042	15.64809	20.6113	11.29014	14.56213	5.377848
AC005593.	0.960908	10.0039	14.987	0	2.21497	6.851322	7.647122	12.96438	5.836439	13.44979	1.204755	5.520469	5.140422	4.653602	1.956012	6.595615	1.026376	11.83173	6.274156
AC005597.	10.56998	10.0039	3.996535	0	0	1.141887	5.098081	0	0	0	0	8.280704	1.028084	0	0.978006	0	0	0.910133	7.170464
AC005606.	0	0	0	0	1.107485	0	0	0	0	0	0	0	0	0	0	0	0	0.910133	0
AC005609.	0	0	0	2.078586	0	1.141887	0	0	0	0	0	0	0	0	3.912023	0	0	0.910133	0
AC005609.	0.960908	1.00039	2.997401	1.039293	0	0	0.84968	1.852054	0	0	0	2.760235	1.028084	0	0	0.824452	2.052753	0.910133	0.896308
AC005614.	6.726353	13.00508	8.992203	5.196464	13.28982	2.283774	8.496802	6.482189	0	16.33189	7.228529	7.360626	2.056169	11.16864	0.978006	12.36678	15.39564	6.370934	7.170464
AC005616.	0	1.00039	0	1.039293	0	0	3.398721	0	4.669151	0.960699	0	0	3.084253	0	0	1.648904	0	3.640534	0
AC005618.	363.223	348.1359	350.6959	200.5835	228.1419	159.8642	314.3817	284.2903	135.4054	130.6551	159.0276	274.1833	286.8355	404.8633	308.0718	266.2979	329.4668	363.1432	363.901
AC005618.	9.609075	4.001562	13.98787	6.235757	14.3973	12.56076	10.19616	25.00273	5.836439	9.606993	18.07132	24.84211	19.5336	18.61441	34.2302	29.68027	21.5539	9.101334	15.23724
AC005622.	12.4918	0	1.998267	3.117878	0	6.851322	16.14392	7.408216	9.338302	4.803496	0	11.96102	10.28084	0.93072	5.868035	4.946711	9.237386	16.3824	7.170464
AC005632.	7.68726	9.003514	7.993069	4.157171	0	0	11.04584	7.408216	1.167288	1.921399	3.614265	13.80117	9.252759	4.653602	6.846041	8.244518	18.47477	4.550667	12.54831
AC005632.	185.4552	83.03241	80.92983	73.78979	112.9635	81.07398	39.08529	201.8739	245.1304	107.5983	80.71858	73.60626	67.85357	99.58707	80.19648	65.13169	82.1101	90.10321	71.70464
AC005632.	0.960908	6.002343	1.998267	0	0	0	3.398721	0	0	0	0	0	5.140422	0	0	0	1.026376	0	0.896308
AC005632.	24.02269	7.002733	16.98527	20.78586	0	5.709435	2.549041	12.96438	14.00745	3.842797	12.04755	8.280704	4.112337	9.307203	8.802052	25.55801	30.79129	4.550667	13.44462
AC005670.	16.33543	28.01093	29.97401	6.235757	14.3973	9.135096	17.84329	28.70684	38.5205	43.23147	18.07132	23.92203	24.67402	25.12945	33.2522	26.38246	24.63303	22.75334	15.23724
AC005674.	37.47539	33.01288	50.95582	8.314342	5.537425	37.68227	6.797442	37.04108	2.334575	37.46727	18.07132	23.92203	30.84253	22.33729	19.56012	4.122259	14.36927	38.2256	13.44462
AC005674.	1432.713	1503.587	2392.925	1059.039	1656.797	1643.175	1244.782	1323.293	3423.655	1865.678	1997.484	2089.498	2290.572	1342.099	1421.042	1775.869	1743.813	1647.341	1375.833
AC005682.	723.5634	266.1039	617.4646	552.9038	459.6062	506.9978	424.8401	350.9642	497.2646	414.0614	537.3207	443.4777	412.2618	404.8633	423.4765	601.8498	662.0127	380.4358	371.0715
AC005691.	4.804538	13.00508	14.987	37.41454	6.64491	1.141887	28.88913	12.03835	0	0	4.81902	8.280704	17.47743	10.23792	2.934017	7.420066	15.39564	7.281067	11.652
AC005692.	0	4.001562	0.999134	0	0	1.141887	4.248401	0	4.669151	3.842797	7.228529	0	2.056169	0.93072	0	3.297807	1.026376	0	0
AC005692.	0	0	1.998267	9.353635	0	0	1.69936	0.926027	0	0	0	0.920078	3.084253	0	1.956012	4.946711	0	0	4.48154
AC005695.	34.59267	24.00937	25.97748	35.33595	40.97694	28.54718	67.12474	50.93149	15.17474	28.82098	36.14265	36.80313	34.95487	37.22881	14.67009	65.13169	78.0046	59.15867	46.60801
AC005695.	0	0	0.999134	0	13.28982	4.567548	0.84968	0	1.167288	3.842797	4.81902	0	0	0	0.978006	0	0	0	0
AC005695.	0	9.003514	0	3.117878	11.07485	2.283774	3.398721	1.852054	4.669151	0	0	11.96102	2.056169	0.93072	0.978006	4.122259	1.026376	1.820267	0
AC005696.	33.63176	93.03631	63.94455	30.13949	63.12664	73.08077	50.13113	72.23011	113.2269	108.559	142.1611	50.6043	51.40422	61.42754	71.39443	33.80253	60.5562	45.50667	96.80126
AC005696.	15.37452	7.002733	7.993069	16.62868	13.28982	11.41887	11.89552	8.334243	45.52422	15.37119	22.89034	4.600391	13.3651	18.61441	9.780058	4.946711	3.079129	13.652	25.99293
AC005696.	2375.363	3265.274	1795.443	2950.552	3232.748	3010.014	2423.288	2173.385	4349.314	3617.033	5573.196	2549.537	2606.194	2038.277	1992.198	3024.914	3530.734	2562.026	4244.018
AC005697.	0.960908	2.000781	2.997401	0	0	0	0.84968	0	8.171014	0	2.40951	0.920078	1.028084	1.861441	0	0.824452	0	0.910133	1.792616
AC005697.	59.57627	15.00586	38.96621	22.86444	28.79461	21.69585	40.78465	7.408216	7.003726	61.48475	36.14265	31.28266	16.44935	26.99089	37.16422	16.48904	13.34289	25.48374	56.4674
AC005699.	15.37452	8.003124	29.97401	8.314342	8.859879	10.27698	16.14392	21.29862	11.67288	1.921399	2.40951	26.68227	19.5336	18.61441	10.75806	3.297807	5.131881	7.281067	20.61508
AC005702.	13.45271	11.00429	1.998267	9.353635	15.50479	12.56076	21.24201	11.11232	7.003726	35.54587	8.433284	21.1618	19.5336	5.584322	10.75806	3.297807	3.079129	35.4952	17.02985
AC005702.	0	0	4.995668	3.117878	5.537425	2.283774	3.398721	6.482189	18.6766	13.44979	4.81902	1.840156	0	0.93072	6.846041	3.297807	0	7.281067	0
AC005703.	25.9445	17.00664	50.95582	23.90373	2.21497	12.56076	11.89552	46.30135	116.7288	31.70308	9.638039	23.00196	22.61786	8.376483	20.53812	25.55801	27.71216	21.8432	18.82247
AC005703.	0	0	0.999134	0	1.107485	1.141887	4.248401	1.852054	0	0	12.04755	0	2.056169	0	1.956012	3.297807	4.105505	0.910133	1.792616
AC005703.	71.10716	39.01523	103.9099	16.62868	29.90209	74.22266	28.03945	39.81916	40.85507	46.11356	65.05676	73.60626	49.34805	47.46674	51.83431	28.03136	28.73854	36.40534	25.09662
AC005703.	14.41361	2.000781	12.98874	19.74656	8.859879	10.27698	17.84329	12.03835	15.17474	5.764196	3.614265	9.200782	8.224675	1.861441	2.934017	20.6113	11.29014	2.7304	8.066772
AC005703.	0	0	0	0	0	0	0.84968	0	0	0	1.204755	0	0	0.93072	0	0	0	0	0
AC005703.	75.91169	43.01679	63.94455	51.96464	34.33203	98.20228	26.34009	60.19176	50.19337	68.20965	73.49005	81.88696	79.1625	60.49682	53.79032	61.00944	41.05505	94.65388	67.2231
AC005722.	0	0	0	1.039293	6.64491	0	0	0	0	0	0	0	1.028084	0	0	0	0	0.910133	0.896308
AC005722.	45.16265	28.01093	17.98441	15.58939	23.25718	26.2634	39.08529	35.18903	46.69151	50.91706	18.07132	38.64329	23.64594	11.16864	5.868035	27.20691	19.50115	43.6864	9.859387
AC005722.	0	1.00039	0	0	2.283774	0	0	0	0	0	1.204755	0.920078	2.056169	0	0	1.648904	0	15.47227	17.02985
AC005725.	2387.855	1740.679	1275.894	1278.33	1191.654	1073.374	1236.285	947.3257	1260.671	933.7997	897.5424	1679.143	1797.091	1502.183	1490.481	805.4894	711.2787	1145.858	1154.445
AC005726.	973.3993	1014.396	1139.012	510.2928	1354.454	976.3134	839.4841	889.912	1983.222	1220.088	1480.644	1108.694	1049.674	806.0038	727.6363	403.1569	456.7374	665.3075	699.1202
AC005726.	25.9445	12.00469	67.94109	21.82515	55.37425	28.54718	58.62794	40.74519	0	28.82098	19.27608	46.00391	48.31997	34.43665	41.07624	22.2602	19.50115	15.47227	32.26709
AC005726.	308.4513	365.1425	399.6535	240.0766	341.1054	358.5525	312.6823	327.8136	798.4248	344.891	304.803	248.4211	335.1555	270.8396	262.1056	187.1506	176.5367	203.8699	138.0314
AC005726.	0	2.000781	0	1.039293	2.21497	0	0	0	0	0	0	4.81902	1.840156	1.028084	2.792161	0	0	0	0
AC005726.	0	0	0	0	0	0	0.84968	0	0	0	0	0	1.028084	0	0	0	0	0.910133	0

AC005730.	2.882723	0	0.999134	0	1.107485	0	0	0	0	0	0	0.920078	2.056169	0	0	0	0	0	0
AC005730.	196.986	237.0925	128.8882	181.8762	290.161	224.9517	158.8902	187.0575	379.3685	330.4805	297.5745	174.8149	152.1565	266.186	259.1715	202.8152	267.8842	111.0363	191.8099
AC005730.	8.648168	12.00469	9.991337	8.314342	11.07485	13.70264	6.797442	36.11505	18.6766	23.05678	20.48083	7.360626	13.3651	2.792161	11.73607	5.771163	7.184634	8.191201	8.066772
AC005736.	233.5005	157.0613	257.7765	210.9764	167.2302	162.148	214.9691	241.6931	113.2269	185.415	181.918	233.6999	182.999	231.7494	208.3152	215.1819	162.1674	172.9253	158.6465
AC005736.	77.83351	70.02733	57.94975	84.18271	34.33203	78.7902	72.22282	112.9753	89.88116	80.69874	60.23774	75.44642	50.37613	63.28898	69.43841	82.44518	68.76721	39.13574	71.70464
AC005740.	45.16265	52.0203	22.98007	13.51081	16.61227	20.55397	26.34009	25.00273	26.84762	42.27077	31.32363	51.52438	53.46039	11.16864	6.846041	32.97807	54.39794	41.86614	28.68185
AC005740.	0	0	0	0	1.107485	0	1.69936	7.408216	0	0	0	0	1.028084	0	0	0	1.026376	0.910133	0.896308
AC005740.	37.47539	24.00937	34.96968	37.41454	32.11706	17.12831	25.49041	28.70684	7.003726	17.29259	16.86657	18.40156	26.73019	16.75297	24.45015	28.85581	11.29014	20.93307	22.4077
AC005740.	81.67714	56.02186	103.9099	84.18271	86.38382	50.24303	67.97442	105.5671	124.8998	96.06993	92.76613	128.811	110.005	60.49682	30.31818	103.8809	69.79359	103.7552	101.2828
AC005746.	137.4098	74.02889	111.903	80.02554	63.12664	98.20228	78.17058	112.0493	19.84389	89.34503	80.71858	98.44837	80.19058	71.66546	78.24047	81.62073	102.6376	113.7667	77.97879
AC005746.	83.59896	67.02616	61.94629	23.90373	102.9961	23.97963	44.18337	47.22738	86.37929	61.48475	65.05676	57.96493	83.27483	41.88241	60.63636	30.50472	40.02867	30.0344	32.26709
AC005746.	12.4918	4.001562	1.998267	10.39293	15.50479	4.567548	1.69936	7.408216	28.01491	7.685594	16.86657	6.440548	9.252759	8.376483	5.868035	1.648904	10.26376	1.820267	0.896308
AC005753.	48.04538	61.02382	37.96708	10.39293	17.71976	58.23624	38.23561	20.37259	114.3942	60.52405	86.74235	27.60235	33.92678	50.2589	32.27419	12.36678	44.13418	50.05734	43.91909
AC005757.	0.960908	4.001562	0	0	0	3.425661	8.496802	0.926027	0	2.882098	9.638039	0.920078	4.112337	2.792161	0	0	0	0	9.859387
AC005758.	1.921815	7.002733	0	2.078586	4.42994	5.709435	5.098081	10.1863	17.50932	16.33189	14.45706	9.200782	0	6.515042	1.956012	2.473355	3.079129	2.7304	5.377848
AC005759.	34.59267	2.000781	8.992203	18.70727	7.752394	22.83774	9.346483	8.334243	8.171014	25.93888	21.68559	12.8811	6.168506	26.06017	12.71408	20.6113	13.34289	10.9216	17.02985
AC005772.	0	0	0	0	0	0	4.248401	0	0	0	0	0	0	0	0	0	0	0	0
AC005774.	0	0	0.999134	0	0	0	0	0	0	0	0	1.840156	1.028084	0	0	0	0	0	0
AC005775.	42.27993	79.03084	45.96015	16.62868	34.33203	50.24303	31.43817	32.41095	40.85507	46.11356	55.41872	52.44446	47.29188	24.19873	20.53812	26.38246	63.63533	51.8776	60.05263
AC005776.	30.74904	20.00781	28.97488	44.68959	6.64491	21.69585	22.09169	20.37259	0	10.56769	10.84279	34.04289	27.75828	18.61441	28.36217	20.6113	30.79129	18.20267	14.34093
AC005776.	125.8789	124.0484	110.9036	153.8153	192.7024	135.8846	136.7985	133.3479	250.9669	129.6944	159.0276	199.657	149.0722	141.4695	203.4252	172.3104	177.5631	197.499	179.2616
AC005779.	6.726353	1.00039	6.993936	8.314342	13.28982	1.141887	13.59488	11.11232	1.167288	4.803496	0	16.56141	3.084253	6.515042	4.890029	4.122259	11.29014	20.93307	5.377848
AC005779.	0	0	1.998267	1.039293	0	0	0	0	0	0	0	0.920078	2.056169	0	0	0.824452	0	0	0.896308
AC005785.	381.4803	315.123	329.7141	305.5521	458.4988	374.5389	242.1589	366.7067	1182.462	682.0965	787.9097	224.4991	260.1053	381.5953	387.2903	264.649	225.8028	302.1643	225.8696
AC005785.	270.9759	264.1031	239.7921	322.1808	169.4452	250.0733	324.5779	272.2519	219.4501	349.6945	209.6273	205.1774	213.8415	300.6227	286.5557	196.2195	174.484	273.04	195.3951
AC005786.	7.68726	15.00586	9.991337	1.039293	9.967364	4.567548	2.549041	5.556162	17.50932	6.724895	31.32363	9.200782	7.196591	0	5.868035	0.824452	2.052753	6.370934	5.377848
AC005786.	0	0	0.999134	0	2.21497	1.141887	0.84968	0.926027	3.501863	0.960699	16.86657	5.520469	3.084253	0	0.978006	0.824452	0	1.820267	1.792616
AC005786.	9.609075	23.00898	13.98787	3.117878	13.28982	2.283774	5.098081	2.778081	5.836439	2.882098	7.228529	21.1618	5.140422	3.722881	6.846041	0	0	5.4608	8.066772
AC005790.	0.960908	0	0	0	0	2.283774	0	0	11.67288	0	3.614265	8.280704	9.252759	0.93072	0	0	0	0.910133	5.377848
AC005796.	0	0	0	0	0	0	0	0	0	0	0	0.920078	0	0	0	0	0	2.7304	0
AC005798.	0	0	0	1.039293	0	0	0	4.630135	0	3.842797	0	0	8.224675	1.861441	0	0	0	0.910133	0
AC005808.	0.960908	0	0	0	0	0	0	0	0	0	4.81902	0	0	0	1.956012	0.824452	0	1.820267	1.792616
AC005821.	484.2974	780.3045	868.2472	241.1159	531.5928	694.2673	618.5672	623.2162	832.2762	657.1183	654.1819	246.581	328.987	482.1131	514.4311	169.8371	227.8555	212.0611	286.8185
AC005822.	12.4918	14.00547	17.98441	4.157171	33.22455	18.27019	10.19616	5.556162	18.6766	16.33189	14.45706	6.440548	9.252759	9.307203	10.75806	16.48904	14.36927	24.5736	12.54831
AC005823.	0	4.001562	0	0	0	1.141887	0.84968	0	0	0	0	4.600391	0	0.93072	0	0	6.158258	0	0
AC005823.	0	0	0	0	0	0	0	0	0	0	0	0	2.056169	0	0	0	0	0	0
AC005828.	1.921815	2.000781	7.993069	5.196464	3.322455	0	18.69297	0	7.003726	4.803496	2.40951	2.760235	9.252759	4.653602	2.934017	10.71787	3.079129	4.550667	17.02985
AC005828.	0.960908	5.001952	4.995668	2.078586	0	5.709435	2.549041	9.26027	0	2.882098	0	5.520469	5.140422	1.861441	6.846041	1.648904	2.052753	0.910133	0
AC005828.	0	0	0	1.039293	0	4.567548	2.549041	0	0	1.921399	0	1.840156	2.056169	6.515042	0	0.824452	3.079129	0	6.274156
AC005828.	35.55358	35.01367	26.97661	27.02161	29.90209	7.993209	41.63433	42.59724	4.669151	14.41049	15.66181	34.04289	44.20763	21.40657	20.53812	13.19123	39.0023	40.956	56.4674
AC005828.	47.08447	21.0082	47.95842	59.23969	9.967364	79.93209	89.21643	27.78081	25.68033	24.97818	39.75691	50.6043	88.41526	21.40657	31.29619	51.94047	56.45069	82.82214	37.64493
AC005828.	6.726353	1.00039	8.992203	1.039293	7.752394	7.993209	9.346483	0.926027	14.00745	5.764196	0	1.840156	8.224675	9.307203	0.978006	2.473355	4.105505	0	3.585232
AC005828.	2.882723	3.001171	5.994802	0	0	1.141887	3.398721	0	1.167288	6.724895	1.204755	6.440548	2.056169	0	1.956012	0	2.052753	0	0
AC005829.	39.39721	41.01601	55.95149	33.25737	27.68712	42.24982	13.59488	25.00273	23.34575	18.25329	28.91412	32.20274	37.01104	35.36737	45.96627	48.64266	43.1078	44.59654	97.69757
AC005829.	165.2761	100.039	87.92376	44.68959	9.967364	67.37133	0	156.4986	1.167288	1.921399	1.204755	6.440548	3.084253	120.0629	83.1305	96.46086	133.4289	126.5085	127.2757
AC005831.	203.7124	144.0562	159.8614	274.3733	134.0057	106.1955	248.1066	198.1698	92.21573	80.69874	67.46627	211.618	133.651	151.7074	183.8651	354.5143	275.0688	335.8392	234.8327
AC005832.	8.648168	9.003514	10.99047	6.235757	2.21497	5.709435	0.84968	12.96438	4.669151	9.606993	0	6.440548	9.252759	1.861441	2.934017	11.54233	11.29014	10.01147	6.274156
AC005832.	2.882723	5.001952	0.999134	8.314342	1.107485	0	16.9936	0	0	3.842797	0	0.920078	1.028084	0	0.978006	2.473355	2.052753	1.820267	0.896308
AC005832.	0	0	0	9.353635	8.859879	0	0	0	0	0	1.204755	2.760235	10.28084	0	0	0.824452	1.026376	1.820267	8.066772
AC005832.	0	2.000781	0	0	0	2.283774	0.84968	0	0	0.960699	0	0	0	2.792161	0.978006	0	1.026376	0	0.896308
AC005833.	31.70995	31.0121	20.98181	12.47151	34.33203	27.40529	16.14392	49.07943	56.02981	38.42797	74.6948	31.28266	40.09529	22.33729	10.75806	41.22259	30.79129	22.75334	27.78555
AC005833.	4.804538	2.000781	4.995668	15.58939	0	1.141887	17.84329	0.926027	0	2.882098	3.614265	10.12086	8.224675	12.09936	17.6041	4.946711	5.131881	6.370934	22.4077
AC005837.	177.7679	209.0816	195.8302	237.998	148.403	121.04	239.6098	176.8712	113.2269	97.99132	74.6948	242.9007	244.6841	150.7767	185.8211	133.5612	141.6399	257.5678	287.7149
AC005837.	6.726353	18.00703	13.98787	4.157171	21.04221	17.12831	14.44456	3.704108	24.51304	4.803496	21.68559	3.680313	13.3651	1.861441	5.868035	1.648904	6.158258	6.370934	7.170464
AC005838.	5.765445	30.01171	11.9896	13.51081	4.42994	4.567548	19.54265	13.89041	14.00745	2.882098	2.40951	20.24172	14.39318	6.515042	5.868035	4.122259	8.21101	19.1128	13.44462
AC005839.	569.8182	774.3022	813.2948	901.0668	728.7251	550.3895	1113.081	885.2818	170.424	593.7121	328.8981	860.2731	828.636	817.1724	815.6569	666.9815	595.2982	916.5044	810.2624
AC005840.	11.53089	17.00664	10.99047	14.5501	13.28982	1.141887	9.346483	24.0767	7.003726	1.921399	9.638039	12.8811	14.39318	14.89152	17.6041	28.85581	18.47477	29.12427	29.57816
AC005840.	2.882723	7.002733	2.997401	1.039293	4.42994	9.135096	1.69936	1.852054	9.338302	0.960699	3.614265	12.8811	5.140422	0.93072	5.868035	0	0	6.370934	4.48154
AC005840.	365.1449	369.1441	747.352	229.6837	693.2856	675.9971	384.0555	564.8765	804.2612	727.2493	778.2717	500.5226	502.7333	679.4258	635.7038	534.2448	470.0803	597.0475	493.8657
AC005840.	9.609075	4.001562	1.998267	4.157171	17.71976	0	17.84329	14.81643	10.50559	18.25329	30.11887	16.56141	19.5336	5.584322	0.978006	10.71787	7.184634	5.4608	7.170464
AC005840.	90.32531	128.05	150.8692	159.0118	176.0901	140.4521	108.7591	135.1999	224.1192	151.7905	185.5323	84.6472	114.1174	129.3701	149.6349	192.9217	154.9828	152.9024	103.9717
AC005841.	0	0	0.999134	0	3.322455	0	0	0.926027	0	1.921399	0	0	1.028084	0	0	0	3.079129	0	0

AC005841.	469.8838	190.0742	242.7895	262.9411	199.3473	222.668	177.5832	244.4711	166.9221	235.3713	248.1795	427.8364	382.4474	210.3428	277.7537	273.718	297.6491	261.2083	249.1736
AC005842.	8.648168	23.00898	19.98267	6.235757	28.79461	4.567548	19.54265	4.630135	0	4.803496	1.204755	16.56141	8.224675	1.861441	27.38416	7.420066	0	7.281067	22.4077
AC005845.	9.609075	9.003514	11.9896	4.157171	7.752394	7.993209	6.797442	12.03835	2.334575	6.724895	7.228529	6.440548	9.252759	9.307203	15.64809	4.946711	7.184634	14.56213	9.859387
AC005856.	70.14625	66.02577	81.92896	15.58939	24.36467	27.40529	58.62794	50.00546	17.50932	55.72056	62.64775	43.24368	34.95487	45.60529	73.35044	20.6113	29.76491	21.8432	38.54124
AC005858.	19.21815	9.003514	1.998267	2.078586	2.21497	0	1.69936	0	11.67288	0	1.204755	3.680313	2.056169	0	0.978006	6.595615	1.026376	10.01147	0.896308
AC005865.	25.9445	12.00469	18.98354	18.70727	1.107485	12.56076	44.18337	11.11232	18.6766	14.41049	12.04755	20.24172	18.50552	29.78305	27.38416	11.54233	14.36927	23.66347	31.37078
AC005865.	24.9836	23.00898	39.96535	33.25737	19.93473	18.27019	62.02666	35.18903	12.84017	25.93888	27.70936	64.40548	52.4323	44.67457	20.53812	42.04704	49.26606	27.304	36.74863
AC005884.	15.37452	8.003124	8.992203	2.078586	9.967364	11.41887	14.44456	9.26027	5.836439	16.33189	4.81902	5.520469	1.028084	2.792161	17.6041	0.824452	6.158258	5.4608	5.377848
AC005885.	134.5271	179.0699	127.8891	32.21808	90.81376	6.851322	70.52346	108.3452	189.1006	34.58517	9.638039	129.731	112.0612	48.39746	55.74633	54.41382	63.63533	70.99041	133.5499
AC005899.	68.22443	69.02694	53.95322	59.23969	66.4491	94.77662	107.0597	80.56435	53.69524	36.50657	49.39495	76.36649	111.0331	80.04195	44.01026	59.36053	42.08143	81.91201	68.1194
AC005899.	21.13997	8.003124	8.992203	27.02161	21.04221	13.70264	9.346483	12.96438	0	19.21399	3.614265	31.28266	22.61786	10.23792	1.956012	9.893422	5.131881	7.281067	14.34093
AC005899.	52.84991	62.02421	56.95062	51.96464	26.57964	30.83095	36.53625	48.15341	8.171014	54.75986	49.39495	68.08579	58.60081	31.64449	30.31818	42.04704	65.68808	40.956	40.33386
AC005899.	82.63805	88.03436	108.9056	77.94696	128.4683	111.9049	66.27506	101.863	231.123	119.1267	175.8942	101.2086	86.35909	71.66546	103.6686	86.56744	82.1101	81.91201	79.77141
AC005899.	77.83351	74.02889	63.94455	70.67191	116.2859	83.35775	75.62154	49.07943	168.0894	110.4804	127.704	97.52829	80.19058	69.80402	89.97654	76.67402	114.9541	88.28294	69.01571
AC005899.	91.28622	81.03163	74.93502	94.57564	65.34161	82.21586	95.16419	99.08489	93.38302	71.09174	103.6089	123.2905	90.47142	32.57521	66.5044	70.90286	63.63533	70.99041	85.14926
AC005901.	0.960908	0	0	0	0	2.283774	0	0	0	0	0	0	0	0	0	0	0	0	0
AC005906.	938.8067	1115.435	910.2108	877.1631	866.0532	1189.846	1070.597	653.7751	1252.5	778.1664	791.524	897.0763	794.7092	809.7267	800.0088	916.7904	1070.51	542.4395	795.9215
AC005908.	0	1.00039	0	0	0	0	0	0	9.338302	0	7.228529	0	0	0	0	0	0	0	0
AC005909.	0.960908	0	0	0	0	0	0.84968	0	0	0	0	0	0	0	0	0	0	0	0
AC005911.	29.78813	19.00742	51.95495	46.76817	26.57964	23.97963	44.18337	33.33697	77.04099	17.29259	46.98544	48.76415	32.8987	32.57521	45.96627	24.73355	50.29244	50.05734	35.85232
AC005912.	476.6101	573.2237	320.7219	230.723	431.9191	423.6401	236.2111	401.8957	336.1789	227.6857	345.7646	337.6687	286.8355	455.1222	401.9604	392.4391	399.2604	459.6174	266.2035
AC005912.	0	0	4.995668	1.039293	1.107485	3.425661	2.549041	0.926027	0	6.724895	16.86657	2.760235	0	7.445762	2.934017	1.648904	1.026376	12.74187	0.896308
AC005915.	0	0	0	0	0	0	0	0	0	0	0	0	0	0.93072	0	3.297807	0	0	0.896308
AC005920.	10.56998	37.01445	31.97228	12.47151	19.93473	4.567548	28.03945	28.70684	29.18219	13.44979	19.27608	27.60235	19.5336	48.39746	36.18622	18.96239	18.47477	23.66347	25.99293
AC005920.	7.68726	4.001562	0	13.51081	0	11.41887	18.69297	0	4.669151	1.921399	2.40951	0	2.056169	0.93072	5.868035	4.122259	8.21101	4.550667	6.274156
AC005920.	0	1.00039	0	0	4.42994	0	0	4.630135	0	0	13.2523	0	2.056169	1.861441	0	1.648904	1.026376	0	0
AC005920.	34.59267	30.01171	50.95582	28.0609	33.22455	52.5268	21.24201	38.89314	61.86625	20.17468	56.62348	56.12477	35.98295	49.32818	44.01026	23.08465	27.71216	56.42827	35.85232
AC005921.	0	0	0	0	0	0	1.69936	0	0	0	0	0	0	0	0	0	0	0	0
AC005921.	11.53089	7.002733	15.98614	3.117878	11.07485	6.851322	3.398721	0	11.67288	5.764196	1.204755	5.520469	8.224675	6.515042	1.956012	4.122259	9.237386	7.281067	5.377848
AC005954.	9.609075	16.00625	17.98441	28.0609	11.07485	18.27019	14.44456	19.44657	16.34203	17.29259	21.68559	27.60235	14.39318	13.9608	15.64809	8.244518	41.05505	28.21414	8.963079
AC005954.	5.765445	4.001562	5.994802	7.275049	2.21497	3.425661	16.14392	13.89041	7.003726	8.646293	4.81902	5.520469	8.224675	8.376483	2.934017	9.06897	8.21101	2.7304	3.585232
AC005962.	251.7578	263.1027	186.838	351.281	168.3377	229.5193	229.4137	201.8739	241.6286	227.6857	175.8942	247.501	287.8636	147.9845	154.5249	267.1224	205.2753	174.7456	230.3511
AC005972.	3.84363	3.001171	8.992203	9.353635	2.21497	2.283774	17.84329	14.81643	7.003726	1.921399	3.614265	11.04094	1.028084	9.307203	0.978006	4.946711	1.026376	9.101334	0
AC005972.	0	0	0	0	2.21497	0	0.84968	0	0	0	0	0.920078	0	0	0	0	0	0	0
AC005972.	125.8789	143.0558	136.8813	160.0511	114.0709	73.08077	142.7463	126.8657	121.3979	94.14853	45.78069	99.36845	137.7633	79.11123	133.9868	79.14738	85.18923	117.4072	134.4462
AC005993.	0	0	0	0	0	0	0	0	0	0	0	0	1.028084	0	0.978006	0	0	0.910133	0
AC005996.	6.726353	6.002343	11.9896	5.196464	7.752394	2.283774	35.68657	8.334243	1.167288	5.764196	9.638039	19.32164	8.224675	23.26801	23.47214	14.84013	5.131881	23.66347	10.7557
AC005996.	5.765445	4.001562	7.993069	1.039293	29.90209	23.97963	5.098081	17.59451	16.34203	11.52839	0	12.8811	2.056169	4.653602	17.6041	4.122259	12.31652	6.370934	2.688924
AC005999.	16.33543	22.00859	17.98441	0	7.752394	12.56076	8.496802	0	3.501863	6.724895	0	15.64133	3.084253	25.12945	1.956012	1.648904	2.052753	3.640534	4.48154
AC006001.	4.804538	0	0.999134	0	0	0	0	0.926027	0	1.921399	0	3.680313	0	0	0	0	1.026376	0	0
AC006001.	228.696	138.0539	210.8172	130.9509	282.4087	162.148	152.9424	161.1287	351.3536	220.9608	355.4027	219.8987	235.4313	235.4722	280.6877	192.0973	174.484	177.476	211.5287
AC006001.	7927.487	7596.965	8870.309	6221.207	7104.516	7424.549	6770.252	7890.676	7618.887	8832.669	9041.685	8382.833	8374.775	8251.766	8621.121	6375.486	6774.083	7704.279	6887.23
AC006003.	0	4.001562	0.999134	0	2.21497	1.141887	0	0.926027	0	0	0	0	1.028084	0.93072	0	8.244518	0	4.550667	1.792616
AC006004.	0	0	0	2.078586	1.107485	0	0.84968	0	0	0	0	0	6.168506	0	0	0	1.026376	0	0
AC006006.	0	0	0	0	0	0	0	0	0	0	0	0	0	0	0	0	2.052753	0	0
AC006011.	0.960908	4.001562	9.991337	2.078586	3.322455	5.709435	0	5.556162	4.669151	0.960699	4.81902	3.680313	4.112337	15.82225	2.934017	4.946711	7.184634	7.281067	3.585232
AC006012.	25.9445	14.00547	18.98354	6.235757	6.64491	7.993209	18.69297	24.0767	8.171014	30.74238	9.638039	9.200782	24.67402	21.40657	12.71408	31.32917	8.21101	15.47227	20.61508
AC006013.	0	0	0.999134	4.157171	0	4.567548	2.549041	0	0	2.882098	0	5.520469	5.140422	0.93072	0	3.297807	5.131881	0	6.274156
AC006017.	89.3644	122.0476	103.9099	104.9686	116.2859	132.4589	96.86355	91.67668	89.88116	147.9477	138.5468	125.1306	141.8756	132.1623	113.4487	109.6521	29.76491	66.43974	69.91202
AC006019.	10.56998	5.001952	4.995668	9.353635	24.36467	4.567548	8.496802	0	1.167288	18.25329	40.96167	19.32164	14.39318	3.722881	2.934017	11.54233	32.84404	16.3824	20.61508
AC006019.	5.765445	8.003124	5.994802	0	9.967364	13.70264	1.69936	19.44657	0	16.33189	19.27608	10.12086	15.42127	10.23792	19.56012	5.771163	11.29014	7.281067	9.859387
AC006019.	0	0	0	3.117878	0	1.141887	0	1.852054	0	0.960699	0	0	1.028084	0	0.978006	0	0	0.910133	0.896308
AC006020.	21.13997	28.01093	49.95668	10.39293	31.00958	11.41887	32.28785	45.37532	31.51677	15.37119	38.55216	17.48149	31.87062	41.88241	35.20821	12.36678	16.42202	45.50667	24.20031
AC006022.	0.960908	0	0	1.039293	0	0	0	1.852054	3.501863	0	0	0	0	0	0	0	0	0.910133	0
AC006023.	1.921815	1.00039	8.992203	8.314342	4.42994	1.141887	5.098081	0.926027	2.334575	0.960699	0	0.920078	6.168506	0.93072	3.912023	1.648904	2.052753	7.281067	1.792616
AC006023.	9.609075	8.003124	12.98874	7.275049	7.752394	31.97284	7.647122	3.704108	7.003726	11.52839	36.14265	5.520469	3.084253	5.584322	9.780058	14.84013	18.47477	3.640534	3.585232
AC006026.	0	0	1.039293	0	0	0	0.84968	1.852054	0	0	4.81902	0	1.028084	0	0	1.648904	0	3.640534	0.896308
AC006026.	16.33543	44.01718	22.98007	29.1002	48.72934	23.97963	11.04584	18.52054	12.84017	29.78168	15.66181	34.04289	30.84253	11.16864	15.64809	15.66458	15.39564	35.4952	34.0597
AC006028.	0	2.000781	0	0	0	0	0.84968	0.926027	0	0	0	0	1.028084	4.653602	6.846041	0	0	0	0.896308
AC006030.	40.35812	74.02889	68.94022	100.8114	31.00958	29.68906	106.21	50.00546	17.50932	9.606993	6.023774	64.40548	68.88165	61.42754	50.8563	64.30724	45.16056	43.6864	58.26002
AC006033.	0	0	0	0	1.107485	0	1.69936	1.852054	10.50559	9.606993	7.228529	2.760235	0	0	5.868035	0	4.105505	0.910133	0
AC006033.	95.12985	79.03084	53.95322	95.61493	31.00958	76.50643	44.18337	83.34243	26.84762	45.15286	34.93789	101.2086	44.20763	84.69555	60.63636	135.2101	86.21561	95.56401	57.36371

AC006041.	3.84363	6.002343	3.996535	6.235757	3.322455	3.425661	0.84968	0.926027	11.67288	7.685594	15.66181	2.760235	3.084253	4.653602	3.912023	1.648904	2.052753	1.820267	3.585232
AC006042.	198.9079	155.0605	133.8839	184.9941	197.1323	139.3102	214.9691	167.6109	199.6062	269.9565	280.7079	211.618	195.336	223.3729	225.9193	179.7305	223.75	199.3192	154.165
AC006042.	24.02269	15.00586	15.98614	30.13949	27.68712	14.84453	11.89552	17.59451	11.67288	5.764196	7.228529	11.96102	10.28084	14.89152	18.58211	8.244518	9.237386	9.101334	11.652
AC006042.	135.488	54.02108	116.8986	37.41454	42.08443	55.95246	87.51706	92.6027	14.00745	71.09174	27.70936	117.77	112.0612	54.9125	87.04252	61.00944	33.87042	40.04587	60.05263
AC006042.	34.59267	31.0121	44.96101	28.0609	18.82724	9.135096	46.73241	62.04381	23.34575	20.17468	34.93789	40.48344	13.3651	26.99089	30.31818	35.45143	25.65941	40.04587	23.30401
AC006055.	0	2.000781	0	2.078586	0	3.425661	0	3.704108	0	1.921399	1.204755	0.920078	0	0	4.890029	5.771163	4.105505	0.910133	1.792616
AC006058.	21866.41	22836.91	18986.54	27993.35	18988.94	24559.71	20610.69	18049.19	26097.05	24420.97	23457.78	20703.6	20065.12	20586.6	20523.45	26747.69	29674.59	16174.89	23532.57
AC006058.	23.06178	79.03084	20.98181	27.02161	22.1497	35.3985	29.73881	39.81916	45.52422	73.01314	73.49005	34.04289	38.03912	49.32818	49.8783	29.68027	25.65941	35.4952	42.12647
AC006058.	125.8789	255.0996	146.8726	112.2436	166.1227	299.1744	121.5043	112.9753	646.6774	323.7556	786.7049	184.9357	145.988	131.2316	140.8328	122.8433	241.1984	201.1395	312.8115
AC006058.	3785.015	3763.469	3892.625	5853.297	2754.315	3775.078	4860.171	3133.675	1464.946	3307.688	2219.158	5088.033	4901.906	4494.448	4388.312	5863.501	5987.879	3712.434	5495.264
AC006059.	76.8726	68.02655	28.97488	31.17878	48.72934	35.3985	56.0789	27.78081	71.20455	55.72056	65.05676	61.64524	46.2638	55.84322	29.34017	28.85581	35.92317	73.72081	67.2231
AC006063.	14.41361	36.01406	30.97314	75.86837	25.47215	29.68906	28.03945	37.04108	23.34575	41.31007	42.16642	13.80117	15.42127	23.26801	29.34017	19.78684	20.52753	19.1128	27.78555
AC006063.	12.4918	21.0082	10.99047	14.5501	7.752394	1.141887	23.79105	15.74246	1.167288	13.44979	22.89034	14.72125	13.3651	13.9608	10.75806	27.20691	13.34289	5.4608	23.30401
AC006064.	175.8461	113.0441	111.903	221.3694	104.1036	190.6951	160.5896	134.2739	52.52795	113.3625	90.35662	243.8207	151.1284	134.0237	130.0748	215.1819	204.2489	159.2733	120.1053
AC006064.	63.4199	50.01952	88.9229	116.4008	55.37425	94.77662	87.51706	70.37805	74.70641	96.06993	57.82823	70.84602	126.4544	68.8733	60.63636	70.90286	78.0046	65.52961	74.39356
AC006064.	51.88901	57.02225	104.909	41.57171	84.16885	53.66869	39.08529	97.23284	121.3979	112.4018	153.0039	60.72516	70.93782	53.05106	64.54838	46.1693	46.18693	52.78774	70.80833
AC006065.	3.84363	0	0.999134	0	1.107485	1.141887	9.346483	0	0	0.960699	1.204755	0	3.084253	4.653602	0.978006	0	0	0.910133	0.896308
AC006065.	0	0	2.997401	0	0	0	0	0	0	0	0	0	0	0	0	0	0	0	0
AC006065.	0	3.001171	0	2.078586	1.107485	0	0	0	0	0	0	1.840156	1.028084	0.93072	0	2.473355	1.026376	1.820267	4.48154
AC006065.	0	0	0	0	0	0	0	0	2.334575	0	0	0	0	0	0	0	0	0	0
AC006077.	0	0	0	1.039293	4.42994	0	0	0	0	0	0	0	1.028084	0	0	0	0	0	0
AC006077.	11.53089	3.001171	5.994802	6.235757	18.82724	4.567548	1.69936	2.778081	11.67288	15.37119	42.16642	11.96102	5.140422	2.792161	1.956012	3.297807	4.105505	1.820267	0.896308
AC006111.	23.06178	66.02577	50.95582	33.25737	81.95388	52.5268	28.03945	24.0767	72.37184	63.40615	72.28529	42.3236	29.81445	42.81313	29.34017	28.85581	34.89679	20.93307	19.71877
AC006111.	15.37452	39.01523	20.98181	1.039293	29.90209	9.135096	11.89552	7.408216	22.17847	30.74238	16.86657	13.80117	9.252759	12.09936	12.71408	14.01568	11.29014	24.5736	12.54831
AC006115.	40.35812	19.00742	20.98181	32.21808	29.90209	14.84453	26.34009	30.55889	77.04099	33.62447	48.1902	22.08188	11.30893	29.78305	60.63636	14.84013	19.50115	15.47227	15.23724
AC006115.	0.960908	0	0	0	0	0	0	0	0	0.960699	7.228529	0	0	2.792161	0	0	3.079129	3.640534	2.688924
AC006115.	0	0	2.997401	4.157171	1.107485	4.567548	0.84968	0	0	0	0	0	0	0	1.956012	0	1.026376	0	1.792616
AC006116.	107.6216	73.0285	117.8978	58.2004	54.26676	79.93209	96.86355	50.00546	38.5205	232.4892	156.6181	112.2495	77.10633	62.35826	47.92229	94.81196	99.5585	94.65388	104.868
AC006116.	36.51449	26.01015	50.95582	30.13949	40.97694	35.3985	31.43817	39.81916	49.02608	43.23147	44.57593	15.64133	20.56169	33.50593	34.2302	44.5204	28.73854	29.12427	22.4077
AC006116.	24.02269	18.00703	13.98787	6.235757	0	6.851322	8.496802	16.66849	1.167288	12.48909	1.204755	6.440548	18.50552	6.515042	8.802052	37.92478	22.58028	16.3824	8.066772
AC006116.	0	0	0.999134	0	0	0	0.84968	0	0	0	0	0	0	3.722881	0	0	0	0	0
AC006116.	11.53089	14.00547	29.97401	22.86444	31.00958	25.12151	27.18977	24.0767	15.17474	27.86028	14.45706	25.76219	20.56169	19.54513	18.58211	29.68027	31.81766	19.1128	19.71877
AC006116.	1.921815	3.001171	7.993069	1.039293	3.322455	1.141887	14.44456	1.852054	0	1.921399	0	5.520469	12.33701	1.861441	12.71408	3.297807	12.31652	4.550667	7.170464
AC006116.	0	3.001171	14.987	2.078586	0	4.567548	5.947762	0.926027	28.01491	32.66377	4.81902	8.280704	6.168506	7.445762	6.846041	9.06897	0	1.820267	3.585232
AC006116.	18.25724	16.00625	40.96448	23.90373	13.28982	12.56076	34.83689	27.78081	14.00745	33.62447	56.62348	25.76219	12.33701	25.12945	33.2522	18.13794	48.23968	31.85467	22.4077
AC006116.	7.68726	3.001171	20.98181	0	16.61227	10.27698	4.248401	10.1863	0	15.37119	9.638039	4.600391	7.196591	4.653602	5.868035	5.771163	4.105505	5.4608	12.54831
AC006122.	0	5.001952	0.999134	4.157171	0	0	0.84968	3.704108	0	0	4.81902	9.200782	0	5.584322	0.978006	0.824452	0	2.7304	0
AC006128.	217.1651	155.0605	212.8155	76.90766	325.6006	235.2287	105.3603	197.2438	977.0198	696.507	1534.858	344.1093	221.0381	213.1349	208.3152	150.8747	289.4381	322.1872	365.6936
AC006130.	0	0	0	0	0	0	0	0.926027	0	0	0	0.920078	0	0	0	0	0	0.910133	3.585232
AC006130.	0	0	0	0	6.64491	0	0.84968	0	0	0	0	0	0	0	0	0	0	0	0.896308
AC006141.	3.84363	6.002343	0.999134	6.235757	0	1.141887	0.84968	0.926027	14.00745	6.724895	6.023774	5.520469	5.140422	6.515042	0.978006	5.771163	4.105505	2.7304	1.792616
AC006144.	10.56998	9.003514	5.994802	14.5501	3.322455	9.135096	8.496802	4.630135	0	3.842797	0	11.04094	11.30893	6.515042	8.802052	4.946711	11.29014	4.550667	14.34093
AC006145.	5.765445	5.001952	2.997401	10.39293	0	1.141887	10.19616	9.26027	10.50559	7.685594	6.023774	8.280704	1.028084	4.653602	5.868035	7.420066	5.131881	2.7304	0.896308
AC006148.	35.55358	47.01835	39.96535	73.78979	46.51437	49.10114	24.64073	52.78354	44.35693	25.93888	4.81902	34.96297	32.8987	29.78305	8.802052	19.78684	10.26376	17.29253	12.54831
AC006148.	11.53089	6.002343	1.998267	3.117878	6.64491	0	2.549041	9.26027	0	0.960699	0	0.920078	3.084253	0	0	1.648904	0	1.820267	0
AC006157.	21.13997	38.01484	64.94369	18.70727	52.05179	0	0	0	0	51.87776	51.80446	0	0	0	0	0	0	0	0
AC006159.	0.960908	0	1.998267	1.039293	2.21497	1.141887	5.098081	1.852054	0	4.803496	0	1.840156	3.084253	0	6.846041	0.824452	1.026376	0	0
AC006159.	0	0	0	0	0	3.425661	0	0.926027	5.836439	0	0	0.920078	0	0	0.978006	0	0	0.910133	2.688924
AC006160.	107.6216	63.0246	123.8926	53.00393	94.13622	98.20228	73.92218	100.0109	47.8588	114.3232	40.96167	102.1287	89.44334	123.7858	98.77859	89.0408	87.24198	111.0363	77.97879
AC006195.	13.45271	12.00469	7.993069	12.47151	0	17.12831	14.44456	23.15068	1.167288	22.09608	18.07132	7.360626	14.39318	7.445762	11.73607	7.420066	4.105505	4.550667	2.688924
AC006197.	0	0	0.999134	0	0	0	0	0	0	0	0	3.680313	1.028084	0	0	0.824452	1.026376	0.910133	0
AC006199.	0	0	0	1.039293	0	0	2.549041	0	0	0	0	0	0	0	0	0	0	0	0
AC006205.	0	0	0	0	0	0	0	0	0	0	0	0	1.028084	0	0	0.824452	0	0	0
AC006205.	0	0	0	0	0	0	0	0	0	0	15.66181	0	0	1.861441	0.978006	0	0	0	1.792616
AC006206.	0.960908	0	0	1.039293	0	1.141887	8.496802	14.81643	0	0.960699	7.228529	1.840156	1.028084	0	0	0.824452	0	0	0
AC006207.	10.56998	29.01132	5.994802	0	4.42994	3.425661	6.797442	0	16.34203	8.646293	12.04755	8.280704	1.028084	9.307203	3.912023	13.19123	9.237386	0	1.792616
AC006210.	2.882723	0	0	0	0	0	0	0	0	0	0	0	0	0.93072	0	0	1.026376	0	0
AC006210.	3.84363	1.00039	0.999134	0	2.21497	3.425661	0	0.926027	1.167288	1.921399	2.40951	1.840156	0	0.93072	2.934017	0.824452	0	3.640534	0.896308
AC006213.	92.24712	118.0461	111.903	223.4479	85.27634	101.6279	119.8049	129.6438	77.04099	69.17035	104.8137	123.2905	76.07824	106.1021	130.0748	127.79	167.2993	72.81067	93.21603
AC006213.	553.4827	333.13	321.721	619.4185	246.9691	401.9442	450.3305	455.6053	92.21573	165.2403	133.7278	350.5498	332.0713	364.8424	386.3123	502.0912	521.3991	350.4014	432.0204
AC006213.	40.35812	27.01054	37.96708	19.74656	25.47215	60.52001	45.88273	68.526	11.67288	33.62447	19.27608	34.04289	30.84253	40.02097	28.36217	29.68027	51.31881	45.50667	31.37078
AC006213.	100.8953	160.0625	137.8804	165.2476	129.5757	99.34417	94.31451	158.3506	58.36439	125.8516	71.08054	139.8519	125.4263	94.93347	113.4487	103.0565	111.875	56.42827	101.2828

AC006213.	30.74904	47.01835	57.94975	47.80747	23.25718	22.83774	28.88913	58.3397	11.67288	31.70308	34.93789	25.76219	39.06721	48.39746	18.58211	36.27588	37.97592	20.93307	32.26709
AC006213.	387.2457	248.0968	227.8025	336.7309	258.044	339.1404	371.3103	279.6602	281.3163	296.8561	219.2654	332.1482	354.6891	275.4932	253.3035	286.0848	239.1457	311.2656	354.9379
AC006237.	52.84991	65.02538	43.96188	43.6503	84.16885	133.6008	74.77186	117.6054	120.2306	81.65944	56.62348	46.92399	46.2638	45.60529	43.03226	18.13794	29.76491	19.1128	20.61508
AC006238.	11.53089	23.00898	37.96708	14.5501	18.82724	2.283774	8.496802	16.66849	29.18219	8.646293	7.228529	11.04094	12.33701	14.89152	10.75806	8.244518	11.29014	52.78774	15.23724
AC006249.	13.45271	34.01327	18.98354	30.13949	43.19191	37.68227	5.947762	23.15068	12.84017	19.21399	22.89034	9.200782	9.252759	4.653602	20.53812	9.893422	11.29014	6.370934	18.82247
AC006252.	48.04538	40.01562	57.94975	54.04322	28.79461	55.95246	23.79105	35.18903	49.02608	46.11356	84.33284	41.40352	48.31997	46.53602	43.03226	37.92478	44.13418	44.59654	51.08955
AC006254.	313.2559	342.1335	423.6327	489.5069	459.6062	465.8899	395.951	345.4081	1024.879	695.5463	915.6137	492.2419	555.1656	422.547	580.9355	473.2354	507.0299	607.9691	639.9639
AC006254.	20.17906	9.003514	2.997401	12.47151	3.322455	13.70264	16.9936	14.81643	0	3.842797	1.204755	11.04094	12.33701	20.47585	0.978006	6.595615	15.39564	14.56213	1.792616
AC006254.	3.84363	1.00039	4.995668	3.117878	0	0	5.947762	6.482189	0	12.48909	0	0.920078	3.084253	3.722881	1.956012	2.473355	0	1.820267	1.792616
AC006262.	260.4059	419.1636	294.7444	183.9548	230.3569	282.0461	429.0885	226.8766	285.9855	341.0482	591.5346	151.8129	230.2909	178.6983	197.5572	87.39189	90.32111	147.4416	110.2459
AC006262.	10.56998	14.00547	4.995668	0	9.967364	7.993209	18.69297	12.96438	0	7.685594	0	9.200782	2.056169	4.653602	10.75806	2.473355	0	2.7304	2.688924
AC006262.	0	1.00039	0.999134	1.039293	2.21497	3.425661	0.84968	0	0	1.921399	1.204755	3.680313	1.028084	0	0	4.122259	2.052753	1.820267	0
AC006262.	2.882723	9.003514	8.992203	2.078586	4.42994	5.709435	1.69936	4.630135	0	0.960699	1.204755	5.520469	6.168506	3.722881	1.956012	2.473355	16.42202	0	1.792616
AC006269.	0	0	3.996535	0	1.107485	0	0	0	0	0	0	0	0	0	0	0	0	0	0
AC006270.	5.765445	14.00547	6.993936	4.157171	34.33203	20.55397	6.797442	5.556162	60.69896	18.25329	33.73314	8.280704	4.112337	0.93072	12.71408	10.71787	1.026376	1.820267	3.585232
AC006270.	0	4.001562	0.999134	4.157171	0	0	9.346483	0.926027	1.167288	7.685594	6.023774	8.280704	2.056169	0	4.890029	0	0	0	1.792616
AC006270.	0	0	0	0	0	0	0	0	0	3.842797	0	0	0	0	0	0	0	0.910133	0
AC006272.	14.41361	8.003124	3.996535	7.275049	23.25718	20.55397	14.44456	10.1863	24.51304	12.48909	9.638039	34.96297	30.84253	26.06017	14.67009	17.31349	18.47477	40.04587	25.09662
AC006273.	2.882723	1.00039	10.99047	4.157171	0	1.141887	0	3.704108	10.50559	5.764196	6.023774	18.40156	4.112337	4.653602	3.912023	8.244518	9.237386	5.4608	4.48154
AC006277.	94.16894	184.0718	221.8077	111.2043	168.3377	176.9925	145.2953	151.8684	159.9184	249.7818	186.737	121.4503	112.0612	238.2644	185.8211	129.4389	141.6399	158.3632	238.4179
AC006288.	0	0	0	0	4.42994	0	0	0	0	0	0	0.920078	1.028084	0	1.956012	0	2.052753	0	0
AC006296.	0	1.00039	0	0	0	0	0	0	0	0	0	3.680313	0	0	0	0	0	0	0
AC006299.	49.00628	42.0164	29.97401	24.94303	29.90209	20.55397	22.94137	22.22465	15.17474	22.09608	16.86657	15.64133	30.84253	17.68369	34.2302	35.45143	27.71216	21.8432	12.54831
AC006305.	24.02269	15.00586	58.94889	11.43222	38.76197	5.709435	13.59488	55.56162	54.86252	21.13538	8.433284	11.04094	42.15146	11.16864	18.58211	4.122259	6.158258	25.48374	2.688924
AC006305.	1.921815	2.000781	1.998267	0	0	0	0	0.926027	0	0	0	0	0	0	5.868035	0	0	2.7304	0
AC006326.	0	0	0	0	0	0	0	0	0	0	0	0	0	0	0	0	0	0	3.585232
AC006329.	30.74904	29.01132	19.98267	17.66798	27.68712	33.11472	31.43817	44.4493	11.67288	24.97818	13.2523	11.96102	17.47743	24.19873	16.6261	25.55801	14.36927	17.29253	10.7557
AC006329.	2.882723	5.001952	0	2.078586	3.322455	12.56076	0	1.852054	42.02236	0	4.81902	0.920078	5.140422	0	5.868035	2.473355	1.026376	0.910133	8.963079
AC006330.	77.83351	86.03358	121.8943	71.7112	99.67364	62.80379	91.76547	121.3095	21.01118	66.28825	102.4042	135.2515	92.52759	80.04195	83.1305	93.98751	87.24198	121.9579	110.2459
AC006333.	0	0	0	0	0	0	0	0.84968	7.408216	7.003726	0	3.614265	0	0	0	0.978006	0.824452	0	0
AC006333.	917.6667	936.3655	1141.011	1009.153	1005.596	757.0711	1017.917	789.9011	622.1644	853.1009	712.0101	847.3921	913.967	1083.358	1013.214	860.7277	833.4175	1009.338	794.1288
AC006349.	0	9.003514	0	6.235757	2.21497	7.993209	8.496802	3.704108	0	0.960699	7.228529	3.680313	1.028084	1.861441	0	5.771163	2.052753	2.7304	6.274156
AC006355.	0.960908	0	0.999134	0	0	0	1.69936	0	0	0.960699	0	0	0	0	1.956012	0.824452	1.026376	0.910133	0.896308
AC006355.	0	0	0.999134	1.039293	0	0	3.398721	0	0	0.960699	0	0.920078	0	0.93072	0.978006	1.648904	0	0.910133	3.585232
AC006357.	1.921815	4.001562	3.996535	0	0	0	7.647122	7.408216	2.334575	0	6.023774	0.920078	5.140422	7.445762	0.978006	21.43575	13.34289	4.550667	5.377848
AC006362.	0	0	0	0	0	0	0	0	0	0	0	0	0	0	1.956012	0	0	0	0
AC006369.	5.765445	4.001562	1.998267	1.039293	0	1.141887	11.89552	7.408216	25.68033	0.960699	8.433284	10.12086	2.056169	1.861441	0	20.6113	8.21101	6.370934	0
AC006369.	0	0	0	0	0	0	0.84968	0	0	0	0	0	0	0	0	0	0	0.910133	0
AC006372.	0	4.001562	5.994802	6.235757	1.107485	0	5.098081	7.408216	4.669151	0	1.204755	11.04094	10.28084	0.93072	0.978006	0	3.079129	0	3.585232
AC006372.	1.921815	3.001171	6.993936	2.078586	5.537425	7.993209	5.098081	1.852054	23.34575	2.882098	16.86657	8.280704	7.196591	0	1.956012	3.297807	4.105505	0.910133	3.585232
AC006372.	1.921815	6.002343	8.992203	0	8.859879	0	22.09169	3.704108	18.6766	5.764196	6.023774	12.8811	11.30893	6.515042	6.846041	9.893422	3.079129	0.910133	8.066772
AC006372.	0.960908	0	7.993069	4.157171	17.71976	0	2.549041	0.926027	7.003726	5.764196	0	5.520469	11.30893	0.93072	3.912023	0	8.21101	5.4608	1.792616
AC006378.	107.6216	69.02694	58.94889	119.5187	91.92125	76.50643	80.71962	112.0493	51.36066	79.73804	44.57593	77.28657	68.88165	62.35826	81.17448	71.72731	68.76721	80.09174	83.35664
AC006378.	192.1815	108.0422	204.8224	142.3831	275.7637	266.0597	95.16419	192.6136	194.9371	208.4717	179.5085	137.0917	134.6791	107.9636	160.393	128.6145	142.6663	150.172	146.9945
AC006387.	12.4918	47.01835	22.98007	12.47151	38.76197	21.69585	11.89552	14.81643	94.55031	28.82098	83.12809	7.360626	12.33701	21.40657	23.47214	4.946711	12.31652	17.29253	14.34093
AC006390.	0	0	0	0	0	0	0	0	0.926027	0	0	0	0	0	0	0	0	0	0.896308
AC006427.	0	0	1.998267	1.039293	0	0	1.69936	0	0	1.921399	0	1.840156	1.028084	0	0.978006	0	4.105505	0.910133	0
AC006435.	532.3428	715.2792	564.5105	426.11	530.4853	779.9088	597.3252	400.9697	1229.154	819.4765	1058.98	535.4855	485.2558	496.0739	569.1994	429.5394	594.2718	460.5275	663.2679
AC006435.	1.921815	7.002733	3.996535	1.039293	3.322455	4.567548	3.398721	1.852054	4.669151	5.764196	2.40951	2.760235	0	6.515042	1.956012	1.648904	4.105505	0	0.896308
AC006435.	0	0	0	1.039293	4.42994	0	0	0	0	2.882098	0	0	0	0	0	0	0	4.550667	0
AC006441.	17.29634	40.01562	40.96448	18.70727	23.25718	22.83774	79.02026	31.48492	5.836439	18.25329	34.93789	57.04485	17.47743	20.47585	36.18622	32.15362	42.08143	15.47227	15.23724
AC006441.	0	4.001562	0.999134	0	0	0	0	0	0	0	0	0.920078	0	0	0	0	0	0	0.896308
AC006441.	45.16265	64.02499	42.96275	24.94303	57.58922	37.68227	47.58209	55.56162	79.37557	51.87776	60.23774	43.24368	45.23571	44.67457	75.30645	40.39814	50.29244	60.97894	56.4674
AC006441.	0	0	0	0	0	0	0	0.926027	2.334575	0	0	0	0	0	0	0	0	0	0
AC006445.	1.921815	1.00039	0	0	1.107485	0	1.69936	0	4.669151	0	0	0	0	0	0	0.824452	0	0	1.792616
AC006445.	33.63176	32.01249	48.95755	35.33595	6.64491	5.709435	54.37954	10.1863	2.334575	17.29259	14.45706	23.00196	14.39318	12.09936	37.16422	11.54233	11.29014	60.97894	61.84525
AC006449.	5.765445	0	0	1.039293	0	0	0	0	0	0	0	0	0	0	0	0.824452	0	0	0
AC006449.	183.5333	117.0457	115.8995	93.53635	79.73891	91.35096	93.46483	69.45203	80.54285	135.4586	113.247	178.4952	137.7633	124.7165	102.6906	96.46086	91.34749	129.2389	127.2757
AC006449.	1.921815	7.002733	10.99047	0	2.21497	5.709435	3.398721	3.704108	7.003726	17.29259	1.204755	7.360626	2.056169	3.722881	11.73607	3.297807	3.079129	7.281067	8.066772
AC006449.	34.59267	56.02186	73.93589	51.96464	69.77155	65.08756	31.43817	45.37532	103.8886	62.44545	114.4517	70.84602	46.2638	38.15953	52.81231	40.39814	75.95184	50.05734	58.26002
AC006449.	122.0353	172.0672	136.8813	103.9293	178.3051	142.7359	72.22282	105.5671	328.0079	273.7993	480.6972	188.616	150.1003	194.5205	168.217	183.0283	141.6399	197.499	173.8837
AC006450.	0	0	0	0	0	0	0.84968	0	0	0	4.81902	0	0	0	0	0	0	0	0

AC006453.	0	0	0	0	0	0	0.84968	0	0	0	0	0	0	0	0.978006	0	0	0	0.896308
AC006453.	1970.821	2059.804	1536.668	1514.25	1953.603	1822.452	1057.852	1176.98	2025.244	1449.695	1452.934	1955.166	1914.293	1546.857	1478.745	1684.355	2103.045	1357.009	1436.782
AC006453.	0	0	0	4.157171	0	0	1.69936	0	0	0	0	0	0	0	5.868035	0	1.026376	0	0.896308
AC006453.	14.41361	17.00664	0.999134	9.353635	6.64491	3.425661	5.947762	0	23.34575	5.764196	0	5.520469	2.056169	5.584322	7.824047	12.36678	16.42202	5.4608	10.7557
AC006453.	0	0	0	1.039293	0	0	0	0	0	1.921399	0	0.920078	0	0.93072	3.912023	0	0	0	1.792616
AC006458.	0	0	0	6.235757	1.107485	0	0.84968	4.630135	2.334575	2.882098	0	0	2.056169	1.861441	0.978006	1.648904	3.079129	3.640534	0.896308
AC006460.	12.4918	1.00039	8.992203	13.51081	13.28982	0	10.19616	5.556162	2.334575	3.842797	1.204755	5.520469	7.196591	23.26801	3.912023	1.648904	5.131881	8.191201	9.859387
AC006460.	335.3567	326.1273	439.6188	246.3124	314.5257	301.4582	333.9243	375.0409	213.6137	309.3452	232.5177	355.1502	455.4414	366.7038	398.0484	304.2227	292.5172	357.6824	389.894
AC006463.	0.960908	0	0	0	6.64491	4.567548	4.248401	0.926027	0	1.921399	12.04755	4.600391	0	0	0.978006	0	0	0	0
AC006466.	0.960908	20.00781	7.993069	5.196464	1.107485	3.425661	3.398721	1.852054	2.334575	10.56769	0	11.04094	7.196591	8.376483	2.934017	10.71787	6.158258	0.910133	3.585232
AC006480.	11.53089	2.000781	6.993936	27.02161	2.21497	3.425661	24.64073	3.704108	8.171014	1.921399	2.40951	11.04094	13.3651	9.307203	21.51613	25.55801	10.26376	14.56213	14.34093
AC006480.	385.3239	432.1687	590.488	193.3085	436.3491	270.6272	363.6631	475.0519	360.6919	296.8561	260.2271	351.4699	307.3972	336.9207	348.1701	200.3418	181.6686	253.0171	313.7078
AC006483.	5.765445	0	0.999134	0	0	3.425661	6.797442	0	0	3.842797	0	0	0	0	0	1.648904	1.026376	0	0.896308
AC006483.	9.609075	7.002733	9.991337	1.039293	16.61227	5.709435	3.398721	0.926027	5.836439	3.842797	0	20.24172	1.028084	21.40657	6.846041	9.06897	7.184634	3.640534	5.377848
AC006486.	66.30262	48.01874	98.91423	78.98625	43.19191	67.37133	86.66738	44.4493	53.69524	81.65944	66.26152	59.80509	46.2638	80.97267	51.83431	104.7054	114.9541	71.90054	89.63079
AC006487.	199.8688	154.0601	296.7427	165.2476	262.4739	140.4521	264.2506	182.4273	144.7437	224.8036	273.4794	443.4777	332.0713	327.6135	312.9619	251.4578	257.6204	221.1624	136.2388
AC006487.	3.84363	0	0.999134	7.275049	1.107485	6.851322	1.69936	0.926027	10.50559	6.724895	8.433284	0.920078	6.168506	0	5.868035	1.648904	3.079129	12.74187	3.585232
AC006504.	681.2834	779.3042	1276.893	2693.847	549.3125	842.7126	506.4094	669.5175	595.3167	691.7035	771.0431	2463.049	2694.609	866.5006	861.6231	2000.12	1930.614	678.0494	847.011
AC006504.	0	4.001562	0	0	5.537425	0	5.947762	0	0	0	0	1.840156	3.084253	0	0.978006	0	0	0	0
AC006504.	126.8398	208.0812	116.8986	186.0334	165.0153	187.2695	139.3476	99.08489	228.7884	162.3582	149.3896	108.5692	110.005	196.382	203.4252	82.44518	94.42662	117.4072	106.6606
AC006504.	1.921815	1.00039	1.998267	1.039293	9.967364	3.425661	1.69936	9.26027	0	1.921399	0	0.920078	2.056169	0.93072	0	0	0	5.4608	5.377848
AC006504.	1106.005	1177.46	1008.126	1075.668	994.5215	962.6107	1174.258	1062.153	758.737	872.3149	1026.451	1056.25	1075.376	1161.539	1001.478	878.8657	937.0815	982.9441	1182.23
AC006504.	5.765445	2.000781	1.998267	3.117878	14.3973	1.141887	3.398721	0	0	0	0	0.920078	1.028084	0	0	0.824452	2.052753	2.7304	0.896308
AC006504.	11.53089	9.003514	9.991337	11.43222	0	12.56076	5.947762	3.704108	15.17474	8.646293	6.023774	5.520469	4.112337	1.861441	0.978006	0	4.105505	0.910133	1.792616
AC006504.	241.1878	270.1054	241.7903	225.5265	403.1245	407.6537	205.6226	268.5478	207.7772	401.5723	368.655	186.7759	248.7964	335.99	281.6657	248.9845	223.75	289.4224	313.7078
AC006509.	5.765445	0	0	0	0	0	0	6.482189	0	0	2.40951	0	1.028084	0	0	0.824452	0	0	0.896308
AC006509.	0	1.00039	0.999134	0	0	2.283774	0	0.926027	9.338302	0	0	0	4.600391	4.112337	0.93072	0.978006	0	0	0	0.896308
AC006511.	0	0	1.998267	0	0	0	0	0	0	2.882098	0	0	1.028084	0	0	0.824452	0	0	0
AC006511.	0.960908	7.002733	0	1.039293	0	0	0.84968	7.408216	0	0	0	0.920078	0	0.93072	0	0	4.105505	0.910133	0.896308
AC006511.	86.48168	188.0734	273.7626	118.4794	111.856	108.4793	134.2495	125.9397	123.7325	171.9652	167.4609	116.8499	131.5948	138.6773	152.5689	80.79628	114.9541	136.52	152.3724
AC006511.	0	0	0	0	0	0	0.84968	0	0	0	0	0	0	0.93072	0	0	0	0	1.792616
AC006511.	50.9281	21.0082	54.95235	20.78586	38.76197	22.83774	69.67378	12.03835	52.52795	13.44979	13.2523	43.24368	25.70211	7.445762	33.2522	26.38246	21.5539	15.47227	23.30401
AC006512.	4.804538	0	0	0	2.21497	0	3.398721	7.408216	2.334575	5.764196	0	4.600391	5.140422	4.653602	3.912023	5.771163	17.4484	0	0
AC006512.	0	0	0	0	0	0	0	0	0	0.960699	0	0	0	0	0	0.824452	0	0	0
AC006518.	9.609075	18.00703	12.98874	28.0609	14.3973	19.41208	26.34009	13.89041	0	20.17468	1.204755	14.72125	16.44935	7.445762	12.71408	17.31349	17.4484	2.7304	17.02985
AC006518.	7.68726	16.00625	5.994802	10.39293	16.61227	10.27698	43.33369	25.92876	21.01118	7.685594	10.84279	15.64133	5.140422	13.9608	12.71408	12.36678	9.237386	17.29253	21.51139
AC006529.	0	0	0	0	0	0	0.84968	3.704108	0	0	0	1.840156	1.028084	5.584322	0	2.473355	0	1.820267	0
AC006530.	0	0	0	0	3.322455	0	0	4.630135	0	0	0	3.680313	2.056169	0	0	0	0	0	0
AC006538.	99.93438	173.0675	106.9073	101.8507	224.8194	214.6748	103.661	107.4191	454.0749	251.7032	559.0063	166.5342	127.4825	150.7767	146.7009	143.4546	165.2466	114.6768	183.7431
AC006538.	7.68726	14.00547	20.98181	3.117878	13.28982	12.56076	22.94137	0	47.8588	33.62447	15.66181	20.24172	2.056169	14.89152	13.69208	14.84013	10.26376	10.9216	5.377848
AC006539.	0	0	0	0	0	0	0	0	0	0	0	0	0	3.722881	0	0	0	0	0
AC006539.	12.4918	13.00508	6.993936	5.196464	6.64491	1.141887	6.797442	19.44657	2.334575	3.842797	12.04755	9.200782	1.028084	22.33729	18.58211	16.48904	11.29014	20.93307	24.20031
AC006539.	3.84363	3.001171	2.997401	6.235757	0	4.567548	0.84968	7.408216	0	3.842797	1.204755	4.600391	1.028084	1.861441	4.890029	0.824452	0	2.7304	8.066772
AC006547.	1132.91	1243.485	933.1908	1467.481	1260.318	1197.839	966.9361	1075.117	1048.224	1080.787	1240.898	1278.909	1340.622	1235.997	1227.397	1449.386	1755.103	1070.317	1206.43
AC006548.	0	0	0	0	0	0	0.84968	0	0	0.960699	0	0	0	0	0	0.824452	0	0	0
AC006548.	320.9431	579.2261	783.3208	428.1886	759.7347	235.2287	714.5811	701.0025	670.0232	495.7208	883.0853	575.969	558.2498	458.8451	397.0704	218.4797	220.6709	300.344	503.7251
AC006557.	35.55358	13.00508	15.98614	25.98232	19.93473	21.69585	28.03945	39.81916	50.19337	39.38867	124.0898	20.24172	25.70211	30.71377	37.16422	23.9091	40.02867	18.20267	34.95601
AC006557.	0	8.003124	2.997401	6.235757	0	0	0	6.482189	3.501863	9.606993	18.07132	10.12086	1.028084	5.584322	0	8.244518	0	12.74187	8.066772
AC006557.	14.41361	3.001171	14.987	7.275049	14.3973	11.41887	12.7452	22.22465	14.00745	7.685594	16.86657	1.840156	11.30893	5.584322	12.71408	7.420066	6.158258	7.281067	8.066772
AC006557.	1.921815	2.000781	17.98441	0	9.967364	13.70264	13.59488	9.26027	84.04472	0	0	16.56141	4.112337	13.03008	10.75806	6.595615	10.26376	7.281067	11.652
AC006566.	11.53089	1.00039	4.995668	22.86444	3.322455	12.56076	10.19616	17.59451	5.836439	8.646293	20.48083	2.760235	1.028084	9.307203	23.47214	10.71787	2.052753	5.4608	12.54831
AC006581.	0.960908	0	0	1.039293	1.107485	0	0	0	0	0	0	0	4.112337	0.93072	0.978006	0	1.026376	0	0.896308
AC006581.	73.02897	86.03358	72.93676	80.02554	23.25718	46.81737	67.12474	59.26573	39.68778	48.99566	33.73314	100.2885	63.74123	71.66546	58.68035	72.55176	70.81996	62.79921	58.26002
AC006600.	0	0	0	3.117878	0	0	0	0	0	0	0	0	0	0	0	0	0	0.910133	0
AC006927.	0	0	0	0	0	9.135096	1.69936	0	0	0	1.204755	4.600391	0	0.93072	0	6.595615	0	0	0
AC006927.	0	1.00039	0.999134	1.039293	0	0	0.84968	0	0	0	0	1.840156	0	0.93072	0	0	0	0	0.896308
AC006942.	17.29634	10.0039	11.9896	10.39293	12.18233	11.41887	6.797442	10.1863	30.34948	23.05678	12.04755	7.360626	10.28084	14.89152	8.802052	9.893422	15.39564	10.01147	11.652
AC006946.	4.804538	17.00664	43.96188	23.90373	62.01916	19.41208	44.18337	51.85751	0	24.97818	4.81902	74.52634	53.46039	5.584322	1.956012	23.08465	21.5539	25.48374	12.54831
AC006946.	1101.2	1224.478	1399.786	887.556	1573.736	1015.138	1100.336	1384.41	1273.511	1850.307	1692.681	975.2829	1232.673	915.8288	917.3695	743.6556	871.3934	625.2617	700.0165
AC006946.	273.8586	339.1324	371.6777	187.0727	480.6485	294.6068	249.806	335.2218	267.3089	436.1575	489.1305	223.579	277.5828	161.0146	169.195	155.8214	123.1652	205.6902	160.4391
AC006947.	12.4918	7.002733	163.8579	12.47151	33.22455	26.2634	11.04584	89.82462	50.19337	79.73804	62.64725	35.88305	35.98295	73.5269	58.68035	4.946711	3.079129	31.85467	17.92616
AC006960.	16.33543	4.001562	13.98787	12.47151	2.21497	9.135096	5.098081	6.482189	0	0	3.614265	5.520469	14.39318	3.722881	6.846041	14.01568	4.105505	10.9216	10.7557

AC006960.	0	1.00039	0	2.078586	0	0	0	0.926027	0	0	0	1.840156	0	3.722881	1.956012	1.648904	3.079129	0.910133	11.652
AC006960.	29.78813	67.02616	76.93329	119.5187	56.48173	166.7155	84.96802	22.22465	148.2455	97.03062	128.9088	24.84211	52.4323	29.78305	35.20821	84.91854	78.0046	52.78774	58.26002
AC006963.	16.33543	23.00898	26.97661	43.6503	38.76197	20.55397	26.34009	14.81643	5.836439	41.31007	25.29985	9.200782	33.92678	18.61441	8.802052	14.84013	23.60665	10.9216	10.7557
AC006965.	2.882723	8.003124	12.98874	6.235757	2.21497	0	14.44456	9.26027	4.669151	0.960699	0	9.200782	8.224675	4.653602	0.978006	4.946711	2.052753	3.640534	6.274156
AC006967.	0	0	0	5.196464	0	0	0	0	5.836439	0	0	0	1.028084	0	0	0.824452	0	0	3.585232
AC006967.	0	0	0	0	0	0	0	0	0	0	0	0	4.112337	0	0	0	0	0	0
AC006970.	3.84363	1.00039	0	3.117878	12.18233	2.283774	8.496802	2.778081	1.167288	6.724895	19.27608	2.760235	1.028084	0.93072	0.978006	2.473355	0	0.910133	0.896308
AC006970.	54.77173	73.0285	61.94629	81.06484	83.06137	93.63473	95.16419	65.74792	11.67288	23.05678	13.2523	129.731	145.988	76.31906	78.24047	62.65834	66.71446	36.40534	86.04556
AC006971.	1.921815	1.00039	3.996535	7.275049	4.42994	1.141887	1.69936	0	0	0	0	1.840156	1.028084	1.861441	0	4.946711	6.158258	0	0.896308
AC006974.	3.84363	0	0.999134	2.078586	0	0	0	0	0	0	0	0	0	1.861441	0.978006	0	0	0	8.066772
AC006974.	0	7.002733	5.994802	10.39293	4.42994	5.709435	0.84968	25.00273	0	2.882098	0	0.920078	4.112337	0.93072	1.956012	21.43575	19.50115	0.910133	20.61508
AC006978.	2005.414	1348.526	2125.157	1946.595	2008.978	1685.425	2166.685	1704.816	2776.978	1980.001	2144.464	2070.176	2169.258	1719.971	1872.881	1066.016	1027.403	2273.513	2045.375
AC006978.	26.90541	22.00859	6.993936	20.78586	2.21497	13.70264	11.04584	15.74246	38.5205	3.842797	6.023774	9.200782	17.47743	16.75297	15.64809	18.96239	10.26376	8.191201	9.859387
AC006994.	22.10087	35.01367	16.98527	31.17878	21.04221	13.70264	22.94137	26.85478	2.334575	17.29259	6.023774	16.56141	20.56169	12.09936	23.47214	23.08465	12.31652	35.4952	29.57816
AC006994.	139.3316	89.03475	175.8475	87.30059	69.77155	47.95925	142.7463	106.4931	112.0596	62.44545	49.39495	90.16767	87.38717	173.114	122.2507	60.18498	68.76721	86.46267	73.49725
AC007000.	181.6115	135.0527	196.8293	49.88605	114.0709	153.0129	89.21643	132.4219	189.1006	123.9302	145.7753	115.0098	127.4825	108.8943	178.9751	114.5988	85.18923	151.9923	158.6465
AC007000.	55.73264	44.01718	51.95495	50.92535	31.00958	27.40529	32.28785	79.63833	14.00745	35.54587	13.2523	48.76415	54.48847	40.02097	82.15249	27.20691	17.4484	50.96747	76.18618
AC007000.	113.3871	56.02186	82.92809	58.2004	63.12664	30.83095	48.43177	127.7917	7.003726	48.03496	22.89034	111.3295	138.7914	52.12034	61.61437	33.80253	26.68578	86.46267	45.71171
AC007001.	27.86632	74.02889	51.95495	12.47151	25.47215	5.709435	13.59488	25.92876	1.167288	3.842797	3.614265	10.12086	4.112337	80.97267	85.08651	16.48904	6.158258	9.101334	7.170464
AC007012.	0	0	0	0	0	0	0	0	0	0	0	0	1.028084	0	0	4.122259	0	0	0
AC007014.	0	2.000781	0.999134	0	8.859879	0	0	0	0	0.960699	0	0	0	0.93072	0	0.824452	1.026376	0.910133	0
AC007014.	0.960908	17.00664	11.9896	0	19.93473	4.567548	2.549041	7.408216	0	10.56769	8.433284	15.64133	8.224675	10.23792	2.934017	5.771163	1.026376	11.83173	3.585232
AC007016.	0	0	0	0	0	0	3.398721	1.852054	0	0	9.638039	0.920078	1.028084	0.93072	0.978006	0	0	0.910133	0
AC007029.	15.37452	9.003514	7.993069	7.275049	4.42994	5.709435	16.14392	15.74246	8.171014	12.48909	13.2523	13.80117	7.196591	13.9608	18.58211	7.420066	9.237386	11.83173	4.48154
AC007032.	0.960908	8.003124	3.996535	3.117878	1.107485	0	1.69936	3.704108	1.167288	2.882098	15.66181	4.600391	1.028084	10.23792	0	1.648904	0	4.550667	1.792616
AC007036.	24.02269	20.00781	21.98094	44.68959	29.90209	25.12151	58.62794	24.0767	5.836439	7.685594	6.023774	63.4854	43.17954	14.89152	12.71408	30.50472	14.36927	14.56213	24.20031
AC007036.	11.53089	10.0039	27.97574	7.275049	15.50479	6.851322	21.24201	19.44657	40.85507	24.97818	40.96167	27.60235	7.196591	25.12945	30.31818	5.771163	4.105505	23.66347	28.68185
AC007038.	303.6468	312.1218	452.6075	334.6523	355.5027	247.7895	376.4083	393.5615	381.7031	373.712	303.5982	474.7604	384.5036	343.4358	430.3226	272.0691	289.4381	332.1987	260.8256
AC007038.	274.8196	286.1117	310.7306	287.8841	341.1054	292.3231	330.5256	353.7423	169.2567	385.2404	339.7409	373.5518	390.6721	307.1377	313.9399	260.5268	193.9851	282.1414	202.5656
AC007040.	0	4.001562	0	1.039293	2.21497	1.141887	5.098081	4.630135	0	1.921399	0	0.920078	1.028084	0	0	0	0	0	0
AC007040.	50.9281	18.00703	35.96881	6.235757	50.94431	15.98642	44.18337	10.1863	112.0596	8.646293	15.66181	39.56336	31.87062	16.75297	24.45015	8.244518	31.81766	14.56213	24.20031
AC007041.	0.960908	0	0	0	2.21497	5.709435	0	0.926027	4.669151	3.842797	1.204755	3.680313	4.112337	4.653602	3.912023	2.473355	4.105505	0	0
AC007064.	0	0	1.998267	0	2.21497	2.283774	0.84968	0.926027	0	2.882098	0	5.520469	0	0	17.6041	0.824452	3.079129	0.910133	3.585232
AC007064.	0	0	0.999134	0	0	0	0	0	0	0	0	0	0	0	1.956012	0	0	0	0
AC007066.	1.921815	7.002733	4.995668	1.039293	1.107485	1.141887	3.398721	2.778081	4.669151	0.960699	7.228529	6.440548	7.196591	9.307203	0	9.06897	5.131881	4.550667	1.792616
AC007066.	860.9731	979.3822	1186.971	671.3831	858.3008	915.7934	818.2421	1176.98	922.1573	1220.088	1090.303	956.8814	875.9279	892.5608	961.3797	713.1508	753.3602	1045.743	847.011
AC007068.	0	0	0.999134	0	0	0	0	0	1.167288	0	0	0	4.112337	0	0	0	2.052753	0	0.896308
AC007091.	8.648168	7.002733	4.995668	10.39293	0	4.567548	5.947762	3.704108	0	6.724895	0	2.760235	4.112337	7.445762	0	12.36678	13.34289	10.01147	15.23724
AC007098.	8.648168	11.00429	6.993936	9.353635	15.50479	3.425661	23.79105	3.704108	0	12.48909	6.023774	20.24172	14.39318	2.792161	4.890029	17.31349	5.131881	14.56213	11.652
AC007099.	1.921815	4.001562	0	0	0	0	1.69936	3.704108	0	0.960699	0	3.680313	1.028084	0	0.978006	0.824452	0	1.820267	3.585232
AC007099.	0	0	0	2.078586	1.107485	0	0	0	0	0.960699	0	0	0	0	0	0	0	0	0
AC007106.	0	1.00039	0	9.353635	2.21497	0	0	3.704108	0	5.764196	0	0	1.028084	0	4.890029	0.824452	5.131881	0	0.896308
AC007114.	166.237	124.0484	172.8501	118.4794	59.80419	92.49285	39.93497	94.45476	276.6472	188.2971	374.6788	133.4113	111.0331	133.093	140.8328	166.5393	156.0092	133.7896	196.2914
AC007114.	10.56998	7.002733	15.98614	17.66798	33.22455	3.425661	26.34009	8.334243	7.003726	6.724895	16.86657	27.60235	31.87062	1.861441	11.73607	24.73355	3.079129	13.652	20.61508
AC007126.	0.960908	0	0	0	0	0	0	0	0	0	2.40951	0	0	0	0	0	8.21101	0	0.896308
AC007128.	4.804538	4.001562	0.999134	8.314342	0	3.425661	11.04584	2.778081	4.669151	0	0	4.600391	10.28084	13.03008	0.978006	4.946711	5.131881	0.910133	7.170464
AC007136.	0	0	0.999134	0	0	0	0	0	0	0	0	0	0	0	0.978006	7.420066	4.105505	3.640534	0
AC007151.	0	0	0	0	4.42994	0	0.84968	0.926027	0	0.960699	0	5.520469	0	7.445762	1.956012	0	1.026376	0.910133	0
AC007159.	0	0	0	0	0	0	0	0	0	0	0	0	0	3.722881	0	0	0	0	0
AC007160.	0.960908	0	0	0	0	0	0	0	0	0	2.40951	0	0	0.93072	0	0	2.052753	1.820267	0
AC007161.	5.765445	1.00039	5.994802	2.078586	6.64491	2.283774	11.04584	4.630135	1.167288	2.882098	1.204755	5.520469	12.33701	3.722881	3.912023	3.297807	1.026376	2.7304	6.274156
AC007161.	0	0	0	1.039293	0	1.141887	1.69936	7.408216	0	0.960699	4.81902	1.840156	2.056169	3.722881	7.824047	3.297807	3.079129	0	0
AC007163.	0	0	1.998267	0	0	0	0	0	0	0	0	0	0	0	0	0	0	0.910133	0
AC007179.	0	0	0	0	4.42994	0	0	0	1.167288	0	0	0	0	0	0	0	0	0	0
AC007182.	1.921815	16.00625	7.993069	11.43222	25.47215	1.141887	11.04584	11.11232	9.338302	0	1.204755	1.840156	5.140422	7.445762	6.846041	14.84013	13.34289	3.640534	14.34093
AC007182.	30.74904	6.002343	14.987	14.5501	23.25718	7.993209	33.98721	22.22465	53.69524	14.41049	6.023774	4.600391	22.61786	0.93072	6.846041	47.81821	20.52753	20.93307	22.4077
AC007191.	898.4485	530.2069	607.4733	526.9214	465.1437	476.1669	569.2858	488.9423	131.9035	336.2447	237.3367	563.0879	530.4915	592.8688	592.6715	583.7119	539.8739	432.3134	347.7675
AC007192.	0	0	5.994802	1.039293	0	1.141887	2.549041	0.926027	1.167288	0	0	0.920078	0	0	0	0.824452	3.079129	8.191201	4.48154
AC007193.	309.4122	709.2768	578.4984	403.2456	368.7925	589.2137	599.8742	638.9587	336.1789	490.9173	440.9403	244.7408	323.8466	507.2426	443.0366	183.8528	277.1216	145.6213	155.9576
AC007204.	0	4.001562	0.999134	4.157171	0	0	0	2.778081	5.836439	0.960699	0	8.280704	1.028084	2.792161	3.912023	2.473355	0	4.550667	8.066772
AC007204.	1.921815	1.00039	0.999134	1.039293	0	1.141887	5.947762	1.852054	0	0	0	1.840156	2.056169	0	4.890029	0	0	4.550667	3.585232
AC007207.	0	0	0	1.039293	0	4.567548	0	0	2.334575	0.960699	0	1.840156	10.28084	5.584322	0.978006	0.824452	3.079129	0	0

AC007207.	0	0	0	0	0	0	0	0	0	0	0	0	0	0	0.93072	0	0.824452	0	0	3.585232
AC007216.	147.9798	67.02616	137.8804	53.00393	137.3281	63.94567	38.23561	76.86024	18.6766	65.32755	45.78069	103.9688	101.7804	166.5989	159.4149	56.06272	54.39794	185.6672	258.1367
AC007216.	12.4918	14.00547	16.98527	8.314342	24.36467	3.425661	11.04584	0	9.338302	7.685594	7.228529	14.72125	19.5336	9.307203	5.868035	9.06897	9.237386	11.83173	5.377848
AC007216.	133.5661	186.0726	185.8389	92.49706	137.3281	115.3306	129.1514	193.5397	85.212	118.166	126.4993	175.7349	117.2016	116.34	168.217	86.56744	114.9541	171.1051	118.3126
AC007216.	69.18534	70.02733	88.9229	78.98625	78.63143	55.95246	120.6546	96.30681	28.01491	51.87776	72.28529	97.52829	132.6229	105.1714	88.02052	73.37621	62.60895	76.45121	97.69757
AC007216.	0	0	0.999134	1.039293	0	0	0.84968	1.852054	0	0	0	0.920078	1.028084	0.93072	1.956012	0	4.105505	0.910133	0
AC007218.	0	0	2.997401	0	1.107485	1.141887	1.69936	0.926027	3.501863	0.960699	0	0.920078	1.028084	0	0	0	1.026376	0	0
AC007218.	0	1.00039	0	0	0	0	5.947762	3.704108	0	1.921399	1.204755	0	4.112337	0	0.978006	4.122259	2.052753	0	0
AC007220.	65.34171	60.02343	89.92203	69.63262	53.15928	102.7698	84.11834	37.96711	135.4054	80.69874	140.9563	76.36649	88.41526	76.31906	101.7126	70.07841	52.34519	47.32694	63.63786
AC007221.	0	2.000781	3.996535	2.078586	3.322455	6.851322	2.549041	1.852054	1.167288	3.842797	0	4.600391	2.056169	2.792161	6.846041	7.420066	5.131881	8.191201	2.688924
AC007221.	1.921815	0	2.997401	0	6.64491	2.283774	18.69297	0	0	1.921399	1.204755	0.920078	1.028084	4.653602	1.956012	0.824452	1.026376	1.820267	0.896308
AC007223.	0	0	0	0	1.107485	1.141887	3.398721	0	0	0	0	0	1.028084	2.792161	0	0	2.052753	0.910133	0
AC007224.	0.960908	0	0	0	0	0	0	0	0	0	0	1.840156	1.028084	0	0	0	0	0	0.896308
AC007224.	35.55358	17.00664	43.96188	18.70727	62.01916	61.6619	12.7452	20.37259	101.554	33.62447	20.48083	17.48149	42.15146	21.40657	37.16422	17.31349	18.47477	43.6864	13.44462
AC007225.	5.765445	5.001952	3.996535	1.039293	2.21497	5.709435	10.19616	15.74246	1.167288	11.52839	0	8.280704	6.168506	11.16864	18.58211	4.946711	4.105505	3.640534	17.02985
AC007228.	49.96719	93.03631	43.96188	48.84676	228.1419	141.594	48.43177	65.74792	588.313	313.188	299.984	49.68422	58.60081	115.4093	125.1847	52.76492	43.1078	68.26001	53.77848
AC007229.	2.882723	6.002343	1.998267	2.078586	8.859879	9.135096	1.69936	1.852054	0	5.764196	14.45706	5.520469	8.224675	1.861441	6.846041	3.297807	7.184634	3.640534	3.585232
AC007238.	319.9822	346.1351	299.7401	327.3772	343.3203	359.6944	292.29	287.0684	535.7851	383.319	434.9165	292.5849	326.9308	370.4267	358.9281	362.7588	365.3899	305.8048	283.2333
AC007240.	1.921815	2.000781	13.98787	6.235757	5.537425	0	0	5.556162	0	13.44979	9.638039	3.680313	11.30893	11.16864	1.956012	28.03136	28.73854	135.6099	49.29694
AC007249.	24.02269	27.01054	44.96101	7.275049	25.47215	18.27019	59.47762	41.67122	43.18965	26.89958	56.62348	54.28462	62.71315	39.09025	55.74633	18.13794	28.73854	70.08027	56.4674
AC007249.	209.4778	276.1078	249.7834	248.391	190.4874	187.2695	424.8401	154.6465	267.3089	328.5591	187.9418	271.4231	347.4925	251.2945	266.9956	211.0597	242.2248	329.4683	305.641
AC007250.	0	1.00039	0	0	6.64491	0	0	0.926027	0	5.764196	0	5.520469	0	0	3.912023	0	0	0.910133	0.896308
AC007255.	62.45899	89.03475	144.8744	82.10413	56.48173	53.66869	104.5107	91.67668	16.34203	138.3407	109.6327	63.4854	82.24675	101.4485	69.43841	109.6521	111.875	69.17014	93.21603
AC007255.	0.960908	0	0	0	0	0	0	0.926027	1.167288	0	0	0.920078	0	0	0	0	0	0	0
AC007256.	35.55358	11.00429	27.97574	42.611	14.3973	20.55397	70.52346	12.03835	1.167288	16.33189	1.204755	13.80117	14.39318	24.19873	32.27419	18.13794	16.42202	26.39387	32.26709
AC007262.	0	0	0	0	3.322455	0	0	0	0	3.842797	0	0	0	0	0	0.824452	5.131881	6.370934	0
AC007271.	0	2.000781	7.993069	2.078586	0	1.141887	2.549041	15.74246	0	0.960699	0	4.600391	5.140422	0.93072	2.934017	4.946711	7.184634	2.7304	1.792616
AC007272.	24.02269	31.0121	34.96968	37.41454	35.43952	11.41887	30.58849	39.81916	40.85507	52.83846	34.93789	45.08383	51.40422	20.47585	30.31818	21.43575	23.60665	40.04587	8.963079
AC007277.	5.765445	15.00586	18.98354	0	15.50479	5.709435	14.44456	11.11232	8.171014	1.921399	6.023774	6.440548	13.3651	16.75297	9.780058	11.54233	8.21101	8.191201	5.377848
AC007278.	15.37452	21.0082	5.994802	8.314342	3.322455	1.141887	84.11834	7.408216	17.50932	6.724895	7.228529	2.760235	17.47743	19.54513	29.34017	12.36678	11.29014	8.191201	19.71877
AC007278.	3.84363	6.002343	0.999134	1.039293	0	0	24.64073	3.704108	0	0.960699	0	0	0	11.16864	4.890029	8.244518	0	3.640534	13.44462
AC007279.	24.9836	12.00469	22.98007	25.98232	16.61227	31.97284	22.09169	18.52054	7.003726	21.13538	7.228529	23.00196	41.12337	11.16864	22.49413	11.54233	33.87042	32.7648	37.64493
AC007283.	19.21815	10.0039	17.98441	8.314342	4.42994	18.27019	6.797442	22.22465	3.501863	11.52839	19.27608	25.76219	20.56169	11.16864	18.58211	9.893422	16.42202	18.20267	7.170464
AC007285.	126.8398	84.0328	129.8874	54.04322	68.66407	70.79699	71.37314	118.5315	50.19337	45.15286	103.6089	102.1287	86.35909	108.8943	68.46041	93.16306	74.92547	115.5869	93.21603
AC007285.	24.02269	27.01054	20.98181	43.6503	14.3973	30.83095	34.83689	23.15068	42.02236	26.89958	12.04755	13.80117	42.15146	41.88241	34.2302	20.6113	24.63303	18.20267	32.26709
AC007292.	36.51449	93.03631	36.96795	33.25737	87.49131	75.36454	29.73881	63.89587	229.9557	97.03062	185.5323	46.92399	57.57272	66.08114	52.81231	25.55801	68.76721	40.956	60.94894
AC007292.	77.83351	31.0121	43.96188	71.7112	64.23413	85.64153	50.13113	48.15341	154.082	63.40615	75.89956	59.80509	62.71315	61.42754	45.96627	65.95615	61.58258	71.90054	64.53417
AC007292.	9.609075	14.00547	5.994802	1.039293	2.21497	5.709435	8.496802	11.11232	11.67288	2.882098	8.433284	2.760235	2.056169	19.54513	8.802052	2.473355	11.29014	10.9216	2.688924
AC007298.	0	0	0	0	0	0	0	6.482189	16.34203	0.960699	0	0	0	0	0	0	0	0.910133	0
AC007298.	0	2.000781	0	0	0	0	1.69936	0	0	0	0	0	0	2.792161	0.978006	0	0	0	0.896308
AC007312.	5.765445	0	0	0	0	0	0	0	0	0	3.614265	0.920078	1.028084	3.722881	0	0	0	0	0
AC007314.	0	2.000781	6.993936	0	0	4.567548	16.9936	4.630135	0	14.41049	8.433284	9.200782	8.224675	5.584322	3.912023	5.771163	2.052753	3.640534	2.688924
AC007318.	189.2988	184.0718	196.8293	178.7584	122.9308	166.7155	129.1514	172.241	88.71387	167.1617	151.7991	170.2145	116.1735	150.7767	148.6569	191.2728	292.5172	151.0821	135.3425
AC007318.	9.609075	25.00976	44.96101	25.98232	26.57964	19.41208	26.34009	14.81643	46.69151	24.97818	42.16642	21.1618	5.140422	35.36737	19.56012	18.13794	6.158258	5.4608	17.02985
AC007319.	55.73264	40.01562	108.9056	67.55403	85.27634	27.40529	175.0341	32.41095	4.669151	60.52405	56.62348	15.64133	22.61786	56.77394	70.41642	46.1693	47.21331	35.4952	29.57816
AC007326.	24.02269	25.00976	45.96015	43.6503	7.752394	23.97963	50.98081	16.66849	8.171014	3.842797	28.91412	22.08188	25.70211	24.19873	11.73607	26.38246	39.0023	16.3824	34.0597
AC007326.	0	0	0	0	0	0	0	0.926027	0	0	0	0	1.028084	0.93072	0	0	0	0.910133	0
AC007326.	4.804538	1.00039	0.999134	3.117878	0	0	0	7.408216	0	6.724895	0	11.04094	2.056169	1.861441	1.956012	0	3.079129	0	5.377848
AC007326.	0.960908	1.00039	0	3.117878	0	2.283774	3.398721	0	0	0.960699	0	0	0	1.861441	4.890029	0	0	0.910133	0
AC007327.	0	0	0	4.157171	0	1.141887	5.098081	0.926027	0	0.960699	0	1.840156	1.028084	0	0	0	1.026376	0	3.585232
AC007327.	3.84363	0	0	4.157171	0	0	6.797442	0	0	0	0	0	2.056169	1.861441	0	0.824452	0	0.910133	6.274156
AC007336.	1324.131	1155.451	1542.662	633.9686	1767.546	696.5511	1834.46	1583.506	315.1677	509.1706	333.7171	1420.601	1531.846	2003.841	2343.302	403.1569	258.6468	629.8123	572.7408
AC007336.	12.4918	37.01445	12.98874	19.74656	7.752394	0	21.24201	16.66849	95.71759	1.921399	10.84279	1.840156	4.112337	27.92161	17.6041	0	0	0	0
AC007336.	6.726353	20.00781	28.97488	60.27898	40.97694	33.11472	75.62154	38.89314	4.669151	9.606993	1.204755	69.92595	59.62889	13.9608	9.780058	9.06897	21.5539	28.21414	5.377848
AC007342.	7.68726	6.002343	0.999134	4.157171	0	1.141887	4.248401	9.26027	3.501863	0.960699	0	0	7.196591	0	7.824047	0	0	0.910133	5.377848
AC007342.	0	0	0.999134	0	0	0	0	0	7.003726	0	0	0	0	0	0	0	0	0	0
AC007342.	0.960908	7.002733	14.987	6.235757	1.107485	2.283774	11.04584	4.630135	1.167288	4.803496	7.228529	6.440548	4.112337	3.722881	11.73607	4.946711	10.26376	31.85467	34.0597
AC007342.	0.960908	4.001562	0	0	1.107485	0	0.84968	0	0	0	0	0	0	0	0	0	0	0.910133	0
AC007342.	0.960908	0	19.98267	0	0	0	20.39233	0	0	0	0	5.520469	0	0	0.978006	0.824452	0	0.910133	4.48154
AC007342.	121.0743	129.0504	103.9099	103.9293	125.1458	73.08077	176.7335	139.8301	53.69524	95.10923	99.99465	44.16376	57.57272	74.45762	75.30645	61.00944	63.63533	133.7896	150.5797
AC007342.	723.5634	965.3768	988.1432	579.9254	727.6176	923.7866	1006.021	1116.789	436.5656	904.9787	686.7103	735.1425	755.642	851.6091	862.6011	521.878	571.6916	584.3057	537.7848
AC007342.	0	0	0.999134	0	0	4.567548	0	0	0	0	0	0	0	0	0	1.648904	1.026376	0	0

AC007342.	0	0	0	0	0	0	0.84968	0	0	0	0	3.680313	0	0	0	0	0	0	0
AC007346.	0.960908	0	0.999134	0	2.21497	0	0.84968	0	16.34203	0	7.228529	1.840156	0	1.861441	0.978006	0.824452	0	0.910133	0.896308
AC007346.	0	0	0	0	0	0	0	0	0	0	0	0	0	0	0	0	0	0.910133	0.896308
AC007347.	9.609075	13.00508	6.993936	5.196464	3.322455	1.141887	8.496802	10.1863	14.00745	0	4.81902	1.840156	10.28084	0.93072	6.846041	4.122259	0	2.7304	0
AC007349.	0.960908	8.003124	1.998267	0	1.107485	0	5.947762	0	11.67288	3.842797	0	2.760235	1.028084	13.9608	3.912023	6.595615	2.052753	3.640534	2.688924
AC007349.	0	8.003124	1.998267	4.157171	0	0	0	0	0	0.960699	7.228529	0.920078	7.196591	1.861441	3.912023	0.824452	1.026376	5.4608	0
AC007349.	0	0	0	0	0	0	0	8.334243	0	0	0	0	0	0	0	0.824452	0	0	0
AC007349.	14.41361	5.001952	8.992203	11.43222	18.82724	10.27698	10.19616	0	11.67288	22.09608	16.86657	6.440548	14.39318	9.307203	7.824047	9.06897	15.39564	12.74187	6.274156
AC007362.	24.02269	10.0039	34.96968	83.14342	44.2994	6.851322	48.43177	17.59451	38.5205	19.21399	20.48083	38.64329	28.78636	17.68369	19.56012	101.4076	66.71446	46.4168	62.74156
AC007363.	0	0	0	0	0	0	0	0	0	0	0	3.680313	0	0	0	0	0	0	0
AC007364.	31.70995	24.00937	34.96968	41.57171	39.86946	43.39171	34.83689	59.26573	45.52422	27.86028	93.97088	26.68227	49.34805	36.29809	23.47214	21.43575	21.5539	21.8432	27.78555
AC007365.	22.10087	16.00625	27.97574	9.353635	13.28982	33.11472	21.24201	29.63287	0	11.52839	21.68559	6.440548	22.61786	35.36737	37.16422	32.97807	17.4484	22.75334	10.7557
AC007365.	0	0	0	0	0	0	1.69936	0	0	0	0	0	0	0	0	0.824452	0	0	0
AC007365.	0.960908	0	3.996535	1.039293	8.859879	0	0	0.926027	0	0	1.204755	0	0	0	0.978006	1.648904	7.184634	6.370934	0.896308
AC007368.	5.765445	2.000781	0	4.157171	0	2.283774	0.84968	5.556162	0	5.764196	0	11.04094	2.056169	0	1.956012	0	0	5.4608	0.896308
AC007370.	1.921815	0	0	0	3.322455	0	0.84968	6.482189	0	0	1.204755	2.760235	0	1.861441	0	0.824452	0	0	0
AC007370.	3.84363	5.001952	0	0	4.42994	4.567548	1.69936	5.556162	8.171014	2.882098	8.433284	5.520469	0	0	3.912023	0	7.184634	0.910133	3.585232
AC007375.	0	0	0	2.078586	0	0	3.398721	0	0	5.764196	0	0	0	0	1.956012	0	0	0	4.48154
AC007375.	2.882723	1.00039	5.994802	11.43222	1.107485	4.567548	10.19616	0.926027	1.167288	2.882098	0	3.680313	4.112337	8.376483	10.75806	22.2602	14.36927	9.101334	6.274156
AC007376.	0	5.001952	5.994802	1.039293	0	0	0	0	0	0	8.433284	1.840156	1.028084	0	0	0	0	0	0
AC007376.	44.20175	16.00625	10.99047	17.66798	15.50479	22.83774	16.9936	30.55889	30.34948	22.09608	27.70936	28.52243	21.58977	27.92161	19.56012	9.893422	17.4484	26.39387	22.4077
AC007378.	0	0	2.997401	10.39293	2.21497	0	1.69936	0.926027	0	0	0	0.920078	4.112337	0	0.978006	0.824452	2.052753	0	0
AC007382.	1042.585	802.3131	1087.057	1552.703	949.1146	1042.543	932.0992	931.5832	324.506	684.9786	538.5254	918.2381	907.7985	709.2089	677.758	1075.91	914.5012	918.3246	932.1603
AC007383.	388.2066	336.1312	433.624	323.2201	324.4931	411.0793	354.3167	342.63	609.3242	480.3496	632.4963	321.1073	398.8967	357.3966	394.1363	342.1475	339.7305	364.0534	413.198
AC007384.	30.74904	6.002343	8.992203	7.275049	2.21497	20.55397	19.54265	20.37259	21.01118	5.764196	6.023774	36.80313	40.09529	14.89152	7.824047	19.78684	8.21101	75.54107	41.23017
AC007386.	14.41361	27.01054	8.992203	39.49313	16.61227	11.41887	33.98721	25.92876	1.167288	13.44979	2.40951	32.20274	41.12337	18.61441	8.802052	16.48904	19.50115	16.3824	8.963079
AC007386.	23.06178	42.0164	13.98787	24.94303	13.28982	22.83774	35.68657	36.11505	61.86625	46.11356	54.21397	17.48149	26.73019	26.06017	17.6041	22.2602	18.47477	28.21414	25.99293
AC007387.	0	0	0	2.078586	0	0	3.398721	0	0	0	0	0	0	0	0	0	0	1.820267	0.896308
AC007388.	1299.147	1287.502	1292.879	1791.741	1200.514	1213.826	1774.132	1263.101	1342.381	1271.005	1207.164	1423.361	1598.671	1301.147	1254.781	1125.377	1155.7	1154.049	1286.202
AC007389.	58.61536	66.02577	67.94109	46.76817	33.22455	90.20907	75.62154	112.9753	117.8961	89.34503	83.12809	47.84407	55.51656	67.94258	65.52639	65.95615	86.21561	82.82214	96.80126
AC007389.	0	0	0	4.157171	0	0	0	0	0	1.921399	9.638039	6.440548	0	0	0	0	0	7.281067	6.274156
AC007389.	0	0	0	1.039293	2.21497	1.141887	0	0	1.167288	0	0	0	0	0.93072	0.978006	0	0	0	0.896308
AC007389.	16.33543	2.000781	2.997401	6.235757	0	7.993209	5.947762	9.26027	0	1.921399	3.614265	2.760235	7.196591	0	0	12.36678	5.131881	8.191201	6.274156
AC007389.	11.53089	21.0082	34.96968	38.45383	40.97694	50.24303	40.78465	43.52327	137.74	85.50223	119.2707	26.68227	28.78636	51.18962	39.12023	32.97807	23.60665	57.33841	55.57109
AC007390.	0	0	0	0	0	0	0.84968	0	0	0.960699	1.204755	0.920078	0	3.722881	0.978006	0	1.026376	0	0.896308
AC007390.	290.1941	335.1308	377.6725	325.2986	447.4239	215.8166	462.2261	508.3888	170.424	226.725	180.7132	411.275	377.307	325.7521	363.8182	274.5425	247.3567	257.5678	304.7447
AC007391.	2.882723	8.003124	6.993936	0	17.71976	9.135096	2.549041	27.78081	14.00745	9.606993	14.45706	4.600391	6.168506	2.792161	8.802052	6.595615	8.21101	8.191201	2.688924
AC007394.	0	0	0	0	0	0	0	0	0	1.921399	0	0	0	0	0	0	0	0	0
AC007395.	0	3.001171	8.992203	0	1.107485	6.851322	0	2.778081	10.50559	6.724895	2.40951	0	0	3.722881	21.51613	0.824452	0	0	1.792616
AC007402.	3.84363	2.000781	5.994802	5.196464	0	0	4.248401	2.778081	0	1.921399	0	0	1.028084	0	3.912023	0.824452	2.052753	6.370934	2.688924
AC007405.	83.59896	88.03436	42.96275	96.65423	42.08443	68.51322	60.3273	81.49038	45.52422	38.42797	44.57593	106.7291	84.30292	91.21059	58.68035	71.72731	85.18923	84.64241	63.63786
AC007405.	0	0	0	0	0	0	0	0	8.171014	0	0	0	0	0	0	3.297807	0	0	0
AC007405.	304.6077	319.1245	186.838	478.0747	269.1188	400.8023	336.4734	232.4328	234.6248	324.7163	337.3314	286.1443	299.1726	293.1769	299.2698	330.6052	371.5482	260.2982	266.2035
AC007406.	8.648168	6.002343	0.999134	5.196464	3.322455	6.851322	8.496802	2.778081	5.836439	7.685594	6.023774	7.360626	13.3651	3.722881	5.868035	3.297807	8.21101	7.281067	6.274156
AC007406.	2.882723	11.00429	10.99047	1.039293	15.50479	1.141887	9.346483	8.334243	1.167288	5.764196	3.614265	8.280704	5.140422	7.445762	4.890029	0.824452	2.052753	5.4608	3.585232
AC007406.	60.53717	104.0406	77.93243	188.112	141.7581	55.95246	174.1844	53.70957	30.34948	97.03062	126.4993	101.2086	98.6961	133.093	151.5909	192.0973	193.9851	151.9923	152.3724
AC007406.	21.13997	12.00469	0	20.78586	7.752394	3.425661	6.797442	29.63287	84.04472	20.17468	27.70936	19.32164	14.39318	7.445762	10.75806	2.473355	15.39564	3.640534	2.688924
AC007406.	3744.657	3157.232	3602.876	3252.986	3289.23	3193.858	3397.022	3483.714	1263.005	2990.657	2110.731	2802.558	3037.989	2874.995	3036.708	2806.434	2847.168	3308.335	2468.432
AC007422.	2.882723	3.001171	5.994802	0	6.64491	2.283774	3.398721	1.852054	0	0	0	0.920078	1.028084	5.584322	0.978006	0.824452	6.158258	5.4608	2.688924
AC007423.	0	0	5.994802	1.039293	0	0	0	0	9.338302	0	0	0	0	0	0	0	0	0	0.896308
AC007424.	104.7389	61.02382	82.92809	40.53242	88.59879	73.08077	30.58849	42.59724	54.86252	51.87776	45.78069	69.92595	63.74123	123.7858	162.349	28.85581	29.76491	37.31547	28.68185
AC007431.	1.921815	6.002343	2.997401	1.039293	2.21497	0	0	4.630135	7.003726	3.842797	0	1.840156	0	0.93072	0.978006	4.122259	1.026376	3.640534	2.688924
AC007431.	6.726353	0	5.994802	0	0	2.283774	0	0	0	0	0	0.920078	0	0	0.978006	0	0	0	0.896308
AC007431.	0	1.00039	3.996535	0	1.107485	0	0	0	0	0.960699	0	0	0	0.93072	1.956012	0	1.026376	0	0
AC007435.	0	2.000781	0	0	0	1.141887	1.69936	0	0	0	0	0	0	0	0	0	0	0.910133	0
AC007448.	0.960908	0	0	4.157171	0	0	0	0	1.167288	0	0	0.920078	0	0	0	4.946711	0	0.910133	0
AC007448.	0	0	0	0	0	0	0.84968	0	0	0	0	0	0	0	0	3.297807	0	0	3.585232
AC007448.	2.882723	3.001171	0.999134	0	0	6.851322	0.84968	1.852054	4.669151	3.842797	16.86657	0	2.056169	0.93072	7.824047	0	1.026376	0	1.792616
AC007450.	0	0	3.996535	1.039293	0	0	1.69936	0	0	0	0	0.920078	0	0.93072	0	0	0	0	2.688924
AC007450.	2.882723	2.000781	2.997401	7.275049	0	0	8.496802	6.482189	0	1.921399	0	0	4.112337	2.792161	0.978006	5.771163	1.026376	0.910133	9.859387
AC007450.	1.921815	2.000781	1.998267	0	0	0	8.496802	0	0	2.882098	3.614265	0	1.028084	0	0	0.824452	6.158258	1.820267	0
AC007450.	0	1.00039	0	0	0	0	0	0.926027	0	0	9.638039	0	0	0	0	0	0	0	0.896308

AC007485.	7.68726	5.001952	12.98874	6.235757	0	7.993209	11.04584	2.778081	2.334575	0	0	5.520469	4.112337	1.861441	5.868035	9.06897	7.184634	7.281067	5.377848
AC007485.	3465.033	4104.602	4192.365	4477.273	3243.823	3672.309	4165.982	3897.648	4004.964	3976.334	3582.941	3940.695	3991.024	4432.09	4328.654	4029.096	4083.951	3402.989	3744.775
AC007490.	28.82723	20.00781	15.98614	45.72888	17.71976	58.23624	25.49041	27.78081	32.68406	29.78168	19.27608	21.1618	8.224675	11.16864	27.38416	18.96239	15.39564	3.640534	17.02985
AC007491.	0.960908	4.001562	0	1.039293	0	2.283774	0	0	0	0	0	0	0	0	4.890029	0	1.026376	2.7304	0
AC007493.	1.921815	0	0	0	0	0	0.84968	0.926027	0	0	0	0.920078	1.028084	0	0	3.297807	4.105505	0	0
AC007493.	0	0	0	2.078586	0	0	3.398721	7.408216	0	0	0	0	0	0	0	0	0	0.910133	0
AC007494.	13.45271	1.00039	8.992203	2.078586	11.07485	17.12831	7.647122	0.926027	0	14.41049	0	16.56141	13.3651	0.93072	17.6041	10.71787	6.158258	14.56213	25.99293
AC007494.	3.84363	4.001562	0.999134	2.078586	1.107485	2.283774	5.098081	0.926027	3.501863	1.921399	3.614265	2.760235	8.224675	3.722881	7.824047	11.54233	4.105505	8.191201	1.792616
AC007494.	0.960908	3.001171	28.97488	1.039293	1.107485	5.709435	15.29424	20.37259	0	11.52839	2.40951	5.520469	13.3651	3.722881	5.868035	2.473355	1.026376	7.281067	3.585232
AC007495.	300.7641	270.1054	274.7618	508.2142	249.1841	306.0257	511.5075	368.5588	112.0596	199.8254	216.8559	292.5849	335.1555	330.4057	265.0396	502.9156	445.4473	273.04	368.3826
AC007495.	0	0	0	2.078586	0	0	1.69936	2.778081	0	0	0	0	1.028084	0.93072	0	5.771163	7.184634	0.910133	1.792616
AC007496.	8.648168	1.00039	0	0	7.752394	1.141887	0	1.852054	0	1.921399	0	0.920078	5.140422	0	3.912023	0	6.158258	0	0
AC007496.	0	1.00039	1.998267	0	8.859879	4.567548	0.84968	0	7.003726	0.960699	0	0.920078	1.028084	3.722881	0	0	2.052753	4.550667	1.792616
AC007496.	1.921815	5.001952	6.993936	4.157171	4.42994	6.851322	11.04584	9.26027	7.003726	4.803496	16.86657	11.04094	7.196591	3.722881	5.868035	12.36678	2.052753	5.4608	3.585232
AC007497.	15.37452	16.00625	15.98614	8.314342	21.04221	13.70264	16.9936	11.11232	56.02981	15.37119	38.55216	12.8811	13.3651	12.09936	13.69208	4.946711	7.184634	7.281067	4.48154
AC007513.	0	0	0	0	0	0	0.84968	0	0	0	0	3.680313	0	0	0	0	0	0.910133	0
AC007527.	0	3.001171	0	0	0	0	0	0	0	0	0	0	0	0.93072	0	0.824452	0	0	0
AC007529.	0	0	0	0	0	0	0	0	0	0	0	0	0	0	0	0.824452	1.026376	0	0
AC007529.	10.56998	0	7.993069	7.275049	5.537425	12.56076	6.797442	3.704108	2.334575	0.960699	4.81902	0.920078	1.028084	0.93072	0	7.420066	7.184634	1.820267	11.652
AC007533.	0	1.00039	6.993936	7.275049	1.107485	1.141887	1.69936	0.926027	4.669151	0.960699	0	12.8811	1.028084	5.584322	0.978006	0	1.026376	0	2.688924
AC007537.	25.9445	29.01132	38.96621	19.74656	21.04221	36.54038	8.496802	9.26027	35.01863	17.29259	24.0951	34.04289	16.44935	35.36737	50.8563	42.8715	36.94955	9.101334	20.61508
AC007537.	5.765445	6.002343	9.991337	1.039293	6.64491	2.283774	7.647122	0	1.167288	2.882098	0	2.760235	5.140422	0	5.868035	1.648904	6.158258	0.910133	11.652
AC007540.	0	0	0	0	0	1.141887	0	1.852054	4.669151	1.921399	2.40951	0	0	0	0	0.824452	1.026376	0	0
AC007541.	312.2949	321.1253	343.702	236.9588	403.1245	382.5321	330.5256	321.3314	314.0004	326.6377	289.1412	326.6278	259.0773	416.032	373.5982	238.2666	218.6181	222.9827	195.3951
AC007546.	58.61536	66.02577	105.9082	95.61493	78.63143	49.10114	84.11834	88.8986	63.03354	72.05244	66.26152	79.12673	79.1625	69.80402	78.24047	51.11601	45.16056	87.37281	80.66772
AC007546.	160.4716	100.039	181.8423	107.0472	165.0153	73.08077	118.9552	138.9041	94.55031	136.4193	144.5706	181.2554	229.2628	93.07203	105.6246	79.14738	82.1101	170.1949	203.4619
AC007547.	0	0	0	0	0	0	1.69936	0	4.669151	0	0	0	0	0	0	0	2.052753	0	0
AC007547.	0	7.002733	20.98181	0	0	0	3.398721	3.704108	4.669151	0.960699	9.638039	6.440548	2.056169	0.93072	1.956012	3.297807	3.079129	0	2.688924
AC007551.	0	8.003124	3.996535	1.039293	6.64491	0	0	3.704108	2.334575	3.842797	0	3.680313	1.028084	2.792161	0.978006	0	1.026376	0	0.896308
AC007551.	0	0	0	2.078586	0	0	3.398721	6.482189	0	12.48909	9.638039	1.840156	1.028084	3.722881	0.978006	3.297807	1.026376	7.281067	0
AC007552.	0	1.00039	0	0	1.107485	0	0.84968	3.704108	0	1.921399	1.204755	1.840156	0	3.722881	0	4.946711	0	5.4608	0.896308
AC007552.	4.804538	23.00898	7.993069	14.5501	16.61227	5.709435	37.38593	5.556162	11.67288	8.646293	4.81902	19.32164	3.084253	32.57521	22.49413	7.420066	6.158258	9.101334	14.34093
AC007556.	10.56998	0	0	0	0	0	0	12.7452	0	0	0	0	1.840156	0	0.93072	1.956012	0	0	0
AC007557.	0.960908	6.002343	6.993936	2.078586	0	5.709435	4.248401	10.1863	0	0	0	6.440548	4.112337	0	0	2.473355	0	0	0
AC007557.	0	2.000781	0.999134	0	0	0	3.398721	0	0	0	0	0.920078	0	0.93072	0	3.297807	1.026376	0.910133	0
AC007557.	0	0	0	0	0	0	0	0	0	0	0	5.520469	0	0	0.978006	0	1.026376	0.910133	0.896308
AC007559.	0	0	0	0	0	0	0.84968	0	0	0	0	0	0	0	0	1.648904	0	0	0
AC007560.	8.648168	1.00039	1.998267	4.157171	0	2.283774	0.84968	0.926027	0	14.41049	0	4.600391	0	1.861441	1.956012	1.648904	2.052753	2.7304	2.688924
AC007562.	0.960908	0	0	3.117878	0	0	1.69936	0	0	0	0	0.920078	1.028084	0	0	0	0	0	3.585232
AC007563.	0	1.00039	3.996535	0	0	4.567548	0	0	0	2.882098	0	0	11.30893	0	2.934017	2.473355	13.34289	1.820267	0
AC007563.	1.921815	2.000781	2.997401	2.078586	2.21497	0	5.947762	0	0	2.882098	4.81902	2.760235	1.028084	7.445762	0.978006	0	3.079129	0	1.792616
AC007563.	0	0	1.998267	0	0	0	5.947762	0.926027	0	0	0	0	0	2.792161	0	0	1.026376	0.910133	0
AC007566.	517.9292	394.1538	391.6604	376.224	382.0823	310.5933	412.9446	323.1834	201.9408	281.4849	255.408	385.5128	466.7503	322.0292	328.61	253.9312	303.8074	313.996	348.6638
AC007568.	7.68726	1.00039	0.999134	0	0	0	4.248401	3.704108	0	0.960699	1.204755	0	1.028084	0	0	0	0	3.640534	1.792616
AC007569.	21.13997	17.00664	29.97401	24.94303	22.1497	34.25661	27.18977	33.33697	30.34948	33.62447	30.11887	33.12282	47.29188	33.50593	29.34017	57.71163	39.0023	21.8432	31.37078
AC007570.	2.882723	6.002343	3.996535	4.157171	6.64491	0	4.248401	12.03835	7.003726	0	3.614265	4.600391	9.252759	2.792161	2.934017	15.66458	7.184634	8.191201	8.066772
AC007595.	0	4.001562	0	2.078586	1.107485	0	2.549041	2.778081	3.501863	5.764196	0	4.600391	9.252759	0.93072	3.912023	4.946711	3.079129	9.101334	0
AC007598.	0	2.000781	0	5.196464	0	0	2.549041	0	3.501863	2.882098	0	0.920078	6.168506	5.584322	0.978006	3.297807	0	2.7304	0
AC007598.	5.765445	1.00039	3.996535	0	8.859879	0	0.84968	0.926027	18.6766	0	2.40951	0	0	0	3.912023	1.648904	0	0	0.896308
AC007598.	0	5.001952	7.993069	3.117878	7.752394	0	3.398721	0	29.18219	2.882098	22.89034	0.920078	7.196591	3.722881	5.868035	0.824452	6.158258	2.7304	3.585232
AC007599.	0	0	0.999134	0	0	1.141887	0.84968	0	0	0	0	0	0	0.93072	0	0	0	0	0
AC007599.	0	0	0	0	0	0	0	0	0	0	0	0	1.028084	12.09936	0	0	0	0	0
AC007601.	13.45271	11.00429	11.9896	1.039293	15.50479	1.141887	0.84968	14.81643	28.01491	1.921399	6.023774	1.840156	1.028084	13.9608	2.934017	2.473355	11.29014	11.83173	4.48154
AC007601.	4.804538	0	0.999134	4.157171	0	0	1.69936	0	0	0	0	0	4.112337	0	0	0.824452	2.052753	0	0
AC007603.	0	0	0	0	0	0	0.84968	0	0	0	0	0	1.028084	4.653602	0	0	0	0	0
AC007608.	8.648168	5.001952	11.9896	5.196464	7.752394	9.135096	10.19616	6.482189	5.836439	13.44979	1.204755	7.360626	6.168506	8.376483	22.49413	5.771163	2.052753	1.820267	7.170464
AC007608.	0	0	7.993069	1.039293	2.21497	0	0	0	0	1.921399	0	0	3.084253	5.584322	0	4.122259	4.105505	0	0
AC007608.	77.83351	23.00898	72.93676	48.84676	60.91167	47.95925	37.38593	38.89314	171.5913	70.13105	42.16642	34.04289	47.29188	44.67457	32.27419	60.18498	46.18693	103.7552	82.46033
AC007608.	30.74904	29.01132	43.96188	1.039293	46.51437	19.41208	9.346483	10.1863	161.0857	26.89958	48.1902	11.96102	2.056169	32.57521	22.49413	10.71787	1.026376	14.56213	0.896308
AC007610.	1.921815	2.000781	0	2.078586	0	6.851322	2.549041	0	0	3.842797	0	1.840156	2.056169	0.93072	4.890029	4.122259	0	5.4608	2.688924
AC007610.	27.86632	23.00898	33.97054	37.41454	33.22455	25.12151	33.13753	48.15341	16.34203	43.23147	15.66181	20.24172	27.75828	25.12945	22.49413	43.69595	35.92317	99.20454	86.94187
AC007610.	0.960908	6.002343	0.999134	0	0	0	0	0	0	0.960699	0	0.920078	1.028084	0	1.956012	0	0	0	0

AC007610.	103.778	124.0484	60.94715	216.1729	162.8003	76.50643	152.0928	116.6794	59.53167	143.1442	114.4517	194.1365	173.7463	110.7557	165.283	184.6772	195.0115	369.5142	337.9081
AC007611.	196.0251	76.02967	114.9004	122.6365	130.6832	85.64153	99.41259	88.8986	50.19337	87.42363	127.704	91.08774	61.68506	120.0629	133.9868	127.79	132.4025	101.0248	93.21603
AC007613.	13.45271	40.01562	36.96795	37.41454	33.22455	21.69585	26.34009	25.00273	9.338302	35.54587	43.37118	33.12282	23.64594	15.82225	22.49413	32.15362	19.50115	26.39387	44.8154
AC007614.	9.609075	13.00508	3.996535	4.157171	8.859879	10.27698	9.346483	0.926027	0	16.33189	9.638039	2.760235	5.140422	9.307203	8.802052	6.595615	9.237386	8.191201	9.859387
AC007614.	0	0	0	0	0	0	0	0	0	4.803496	14.45706	4.600391	3.084253	0	0	0.824452	3.079129	0.910133	4.48154
AC007614.	32.67086	38.01484	14.987	16.62868	19.93473	90.20907	23.79105	16.66849	15.17474	35.54587	45.78069	26.68227	33.92678	40.95169	12.71408	30.50472	30.79129	17.29253	22.4077
AC007614.	20.17906	41.01601	42.96275	33.25737	71.98652	110.763	28.88913	48.15341	42.02236	64.36685	89.15186	34.96297	41.12337	40.95169	40.09824	55.23827	54.39794	40.04587	63.63786
AC007614.	23.06178	21.0082	11.9896	17.66798	69.77155	22.83774	42.48401	17.59451	18.6766	37.46727	34.93789	29.4425	27.75828	26.99089	20.53812	29.68027	19.50115	29.12427	15.23724
AC007614.	0	0	0	0	0	0	0	0	0	0	0	0	0	3.722881	0	0	0	0	0
AC007618.	0.960908	0	0.999134	0	6.64491	0	3.398721	0	0	0	0	0	0	0	0	0	1.026376	0	0
AC007619.	190.2597	135.0527	151.8683	222.4087	99.67364	125.6076	250.6557	135.1999	8.171014	103.7555	42.16642	198.7369	166.5497	136.8159	156.4809	151.6991	136.508	135.6099	179.2616
AC007619.	0.960908	1.00039	0	0	0	3.425661	0.84968	1.852054	0	0	1.204755	5.520469	3.084253	0	0.978006	0.824452	0	0	0
AC007620.	30.74904	44.01718	46.95928	49.88605	39.86946	27.40529	49.28145	75.93422	19.84389	69.17035	42.16642	34.04289	43.17954	45.60529	47.92229	22.2602	27.71216	41.86614	54.67478
AC007621.	0	0	0	0	0	0	0	0	7.003726	0.960699	0	1.840156	0	0	0	0	1.026376	0	0
AC007622.	16.33543	15.00586	19.98267	48.84676	17.71976	35.3985	30.58849	16.66849	49.02608	35.54587	13.2523	14.72125	19.5336	35.36737	17.6041	14.01568	6.158258	8.191201	24.20031
AC007622.	49.00628	102.0398	104.909	102.89	13.28982	52.5268	104.5107	80.56435	26.84762	87.42363	67.46627	57.04485	84.30292	71.66546	52.81231	102.232	88.26836	111.9464	92.31972
AC007637.	221.0087	217.0847	304.7358	223.4479	366.5775	197.5465	329.6759	209.2821	63.03354	146.0263	165.0514	248.4211	338.2398	148.9152	201.4692	139.3324	178.5895	163.824	226.7659
AC007638.	0	6.002343	0	0	0	3.425661	0	8.334243	0	0	0	4.600391	3.084253	2.792161	0	0	4.105505	2.7304	0
AC007638.	5.765445	17.00664	11.9896	22.86444	22.1497	14.84453	16.14392	10.1863	4.669151	22.09608	31.32363	10.12086	23.64594	10.23792	13.69208	22.2602	3.079129	0	8.963079
AC007639.	30.74904	12.00469	7.993069	3.117878	36.547	34.25661	19.54265	24.0767	9.338302	6.724895	18.07132	11.96102	18.50552	9.307203	20.53812	15.66458	9.237386	17.29253	7.170464
AC007655.	0	1.00039	0.999134	1.039293	0	1.141887	0	0	0	2.882098	0	0	6.168506	0	0	3.297807	1.026376	0	0
AC007656.	1.921815	9.003514	0	12.47151	3.322455	2.283774	5.947762	3.704108	1.167288	0	2.40951	3.680313	9.252759	1.861441	4.890029	17.31349	4.105505	8.191201	4.48154
AC007656.	0	0	0	0	0	0	0	0	9.338302	0	1.204755	0	1.028084	0	0	0	0	0	0
AC007663.	35.55358	73.0285	57.94975	35.33595	95.2437	247.7895	94.31451	102.789	186.766	248.8211	410.8214	129.731	89.44334	71.66546	49.8783	39.57369	74.92547	83.73227	95.90495
AC007663.	556.3655	533.2081	491.5738	641.2436	312.3107	1113.34	592.2271	871.3914	657.183	508.2099	673.458	545.6064	487.312	696.1788	570.1774	887.1102	984.2948	337.6595	620.2451
AC007663.	20.17906	32.01249	23.97921	14.5501	33.22455	2.283774	6.797442	13.89041	53.69524	37.46727	53.00921	28.52243	24.67402	17.68369	11.73607	22.2602	23.60665	10.01147	35.85232
AC007663.	183.5333	139.0543	150.8692	98.73281	171.6602	142.7359	124.903	179.6492	133.0708	102.7948	92.76613	157.3334	216.9258	137.7466	183.8651	127.79	181.6686	143.8011	72.60094
AC007666.	85.52077	340.1327	311.7297	153.8153	136.2206	182.7019	332.225	199.0958	136.5727	197.904	219.2654	70.84602	65.7974	134.9544	181.9091	37.10033	61.58258	96.47414	89.63079
AC007671.	4.804538	2.000781	8.992203	4.157171	15.50479	31.97284	9.346483	4.630135	24.51304	42.27077	44.57593	9.200782	9.252759	21.40657	25.42815	11.54233	11.29014	20.93307	25.09662
AC007671.	1.921815	0	1.998267	3.117878	3.322455	2.283774	5.947762	0.926027	0	0	0	4.600391	1.028084	12.09936	0.978006	4.122259	0	0	1.792616
AC007673.	0.960908	0	0	0	5.537425	0	0	0	0	0	0	1.840156	0	0	0	0	0	0	0
AC007679.	0.960908	0	0	0	0	0	0	0	1.167288	0	0	0	0	0	0.978006	0	0	0	0
AC007679.	0	1.00039	0	0	0	0	0.84968	0	0	0	0	0.920078	1.028084	0	0	0.824452	0	1.820267	1.792616
AC007681.	133.5661	133.0519	702.391	301.3949	1407.613	396.2348	98.56291	194.4657	134.2381	823.3193	695.1436	1072.811	1129.865	424.4085	481.1789	189.6239	220.6709	600.6881	336.1155
AC007683.	3.84363	4.001562	14.987	6.235757	14.3973	5.709435	0.84968	12.03835	4.669151	3.842797	8.433284	19.32164	7.196591	2.792161	8.802052	0.824452	0	2.7304	5.377848
AC007683.	1.921815	0	0.999134	0	0	0	0.84968	1.852054	0	4.803496	4.81902	0	3.084253	0.93072	0	0	0	0	0.896308
AC007684.	40.35812	31.0121	64.94369	38.45383	87.49131	35.3985	52.68017	37.96711	25.68033	20.17468	33.73314	15.64133	33.92678	37.22881	21.51613	30.50472	23.60665	29.12427	15.23724
AC007685.	0	3.001171	2.997401	0	0	2.283774	0	1.852054	4.669151	0	0	0	1.028084	2.792161	2.934017	5.771163	1.026376	0	6.274156
AC007686.	0	2.000781	0	0	0	1.141887	0	0	0	0.960699	0	0.920078	2.056169	0	0	1.648904	1.026376	0	0.896308
AC007686.	0	4.001562	0.999134	4.157171	0	3.425661	0.84968	0	5.836439	0	0	1.840156	0	0	0	3.297807	0	1.820267	1.792616
AC007686.	800.436	919.3588	891.2272	522.7643	761.9496	658.8688	788.5033	811.1997	797.2575	883.8433	1005.97	941.24	936.5849	755.7449	893.8973	567.2229	590.1663	958.3705	995.7981
AC007687.	94.16894	113.0441	114.9004	81.06484	122.9308	99.34417	179.2825	129.6438	45.52422	135.4586	75.89956	122.3704	120.2859	136.8159	165.283	50.29156	49.26606	52.78774	121.0016
AC007688.	17.29634	0	1.998267	1.039293	2.21497	0	0.84968	3.704108	9.338302	3.842797	3.614265	4.600391	1.028084	2.792161	0	3.297807	0	0.910133	6.274156
AC007688.	3.84363	1.00039	0	8.314342	0	2.283774	1.69936	3.704108	0	0	0	3.680313	7.196591	1.861441	0	0.824452	1.026376	1.820267	0.896308
AC007690.	0	0	0	0	0	0	0	0	0	0	0	0	0	0	0.978006	0	0	0	0.896308
AC007728.	3.84363	10.0039	37.96708	7.275049	6.64491	6.851322	11.89552	25.00273	0	5.764196	7.228529	17.48149	11.30893	11.16864	10.75806	4.122259	0	3.640534	17.02985
AC007728.	149.9016	83.03241	176.8467	212.0157	222.6045	134.7427	100.2623	187.9835	109.725	66.28825	80.71858	169.2944	144.9599	116.34	124.2067	237.4421	192.9587	425.9424	288.6112
AC007728.	37.47539	15.00586	41.96361	50.92535	97.45867	28.54718	16.9936	77.78627	14.00745	14.41049	22.89034	69.92595	34.95487	30.71377	15.64809	106.3543	41.05505	194.7686	146.9945
AC007731.	0	0	0	4.157171	0	0	0	0	0	0	0	0	0	0	1.956012	0	1.026376	0	0
AC007731.	126.8398	190.0742	361.6864	151.7367	317.8482	190.6951	180.1322	220.3944	40.85507	270.9172	245.77	394.7136	384.5036	262.4631	245.4795	192.0973	193.9851	138.3403	182.8468
AC007731.	0	0	2.997401	0	0	0	0	0	0	0	0	0	0	0	0	0	0	0	0
AC007731.	11.53089	1.00039	0.999134	4.157171	0	0	0.84968	6.482189	7.003726	0	12.04755	3.680313	1.028084	1.861441	0	0	4.105505	3.640534	2.688924
AC007738.	0	0	0	0	0	0	0	0	0	0	0	0	0	3.722881	1.956012	0	0	0	0
AC007741.	4.804538	0	0.999134	6.235757	0	1.141887	1.69936	7.408216	7.003726	4.803496	0	0	1.028084	0.93072	1.956012	0.824452	2.052753	0	1.792616
AC007743.	2236.993	2399.937	2156.13	3314.305	1558.231	1572.378	2001.847	1320.515	570.8037	776.245	530.0921	1978.168	2264.87	1676.227	1664.566	3024.089	3279.272	1721.062	2109.909
AC007750.	7.68726	6.002343	4.995668	4.157171	14.3973	11.41887	10.19616	11.11232	39.68778	30.74238	21.68559	17.48149	11.30893	5.584322	12.71408	18.13794	9.237386	7.281067	3.585232
AC007751.	0	0	0	5.196464	0	1.141887	0	0	0	0	0	0.920078	0	0	0.978006	0	0	0	0.896308
AC007773.	49.00628	64.02499	32.97141	19.74656	28.79461	30.83095	16.14392	50.00546	81.71014	24.97818	51.80446	26.68227	31.87062	26.06017	45.96627	18.13794	16.42202	12.74187	25.09662
AC007780.	57.65445	55.02147	67.94109	100.8114	45.40688	66.22945	95.16419	56.48765	26.84762	58.60265	45.78069	33.12282	70.93782	55.84322	63.57038	67.60505	86.21561	69.17014	45.71171
AC007785.	12.4918	25.00976	53.95322	36.37525	16.61227	7.993209	23.79105	15.74246	2.334575	13.44979	6.023774	17.48149	18.50552	12.09936	11.73607	8.244518	15.39564	3.640534	5.377848
AC007785.	0.960908	0	17.98441	0	0	0	0.84968	0	0	0	0	0	1.028084	0.93072	0	0	0	0	0
AC007785.	32.67086	79.03084	44.96101	50.92535	38.76197	34.25661	210.7207	54.6356	33.85134	38.42797	36.14265	64.40548	51.40422	46.53602	51.83431	46.99375	65.68808	27.304	69.01571

AC007786.	91.28622	99.03865	88.9229	46.76817	49.83682	60.52001	87.51706	75.00819	96.88488	93.18783	49.39495	62.56532	82.24675	64.2197	94.86656	56.88718	63.63533	73.72081	119.209
AC007786.	34.59267	34.01327	33.97054	32.21808	23.25718	18.27019	43.33369	43.52327	56.02981	25.93888	46.98544	21.1618	42.15146	13.03008	49.8783	29.68027	65.68808	30.0344	35.85232
AC007786.	8.648168	2.000781	14.987	1.039293	0	2.283774	8.496802	8.334243	22.17847	5.764196	19.27608	6.440548	1.028084	8.376483	0.978006	3.297807	13.34289	5.4608	4.48154
AC007787.	0	0	0	0	5.537425	4.567548	0.84968	1.852054	7.003726	4.803496	4.81902	0.920078	1.028084	3.722881	1.956012	0	0	4.550667	3.585232
AC007787.	21.13997	2.000781	32.97141	6.235757	19.93473	26.2634	17.84329	32.41095	0	46.11356	61.4425	19.32164	12.33701	11.16864	20.53812	12.36678	9.237386	35.4952	19.71877
AC007790.	0	5.001952	1.998267	1.039293	0	3.425661	1.69936	0	0	1.921399	0	5.520469	1.028084	1.861441	1.956012	4.122259	0	1.820267	0
AC007792.	6.726353	32.01249	18.98354	8.314342	23.25718	15.98642	3.398721	13.89041	15.17474	17.29259	21.68559	16.56141	14.39318	8.376483	23.47214	3.297807	2.052753	23.66347	12.54831
AC007823.	85.52077	40.01562	75.93416	70.67191	90.81376	90.20907	50.98081	69.45203	23.34575	87.42363	30.11887	47.84407	70.93782	39.09025	49.8783	108.0032	127.2707	43.6864	64.53417
AC007834.	0.960908	1.00039	0.999134	0	0	2.283774	2.549041	0	0	0	0	1.840156	2.056169	1.861441	0.978006	0	1.026376	2.7304	0.896308
AC007834.	34.59267	25.00976	48.95755	27.02161	21.04221	38.82416	67.12474	44.4493	5.836439	44.19217	15.66181	37.72321	51.40422	17.68369	38.14223	30.50472	24.63303	29.12427	21.51139
AC007842.	147.0189	132.0515	242.7895	282.6876	193.8099	153.0129	231.113	207.4301	180.9296	220.9608	115.6565	254.8617	289.9198	159.1532	246.4575	248.9845	205.2753	224.803	233.0401
AC007848.	0	2.000781	0	0	0	0	0	10.1863	0	8.646293	3.614265	0.920078	0	0	0	1.648904	0	0	0
AC007849.	0	1.00039	5.994802	4.157171	12.18233	1.141887	0	3.704108	8.171014	0	0	0	0	0	0.978006	0	1.026376	0	0
AC007849.	0	0	0	2.078586	0	2.283774	2.549041	0.926027	0	0	0	0	1.028084	0	1.956012	3.297807	1.026376	1.820267	0
AC007861.	1.921815	1.00039	0	0	1.107485	0	1.69936	0.926027	0	0	0	0	1.028084	1.861441	1.956012	0.824452	0	0.910133	0
AC007878.	132.6052	120.0469	119.896	273.334	222.6045	107.3374	305.8849	166.6849	72.37184	88.38433	46.98544	204.2574	182.999	195.4513	196.5792	130.2634	162.1674	171.1051	169.4022
AC007879.	6.726353	3.001171	3.996535	10.39293	18.82724	7.993209	12.7452	1.852054	3.501863	6.724895	9.638039	9.200782	2.056169	3.722881	6.846041	4.122259	2.052753	4.550667	5.377848
AC007879.	44.20175	50.01952	18.98354	58.2004	15.50479	19.41208	22.94137	29.63287	96.88488	46.11356	36.14265	29.4425	32.8987	38.15953	30.31818	15.66458	41.05505	42.77627	50.19325
AC007879.	35.55358	34.01327	48.95755	64.43615	23.25718	45.67548	39.93497	42.59724	49.02608	45.15286	30.11887	43.24368	40.09529	11.16864	15.64809	15.66458	22.58028	33.67494	37.64493
AC007879.	2.882723	9.003514	4.995668	16.62868	9.967364	10.27698	11.89552	0.926027	12.84017	0.960699	1.204755	11.96102	7.196591	3.722881	4.890029	4.946711	9.237386	12.74187	6.274156
AC007881.	96.09075	141.0551	108.9056	77.94696	178.3051	182.7019	56.92858	118.5315	550.9598	211.3538	406.0024	120.5302	108.9769	102.3792	103.6686	121.1944	142.6663	139.2504	115.6237
AC007881.	0	39.01523	6.993936	51.96464	0	30.83095	33.13753	34.263	0	8.646293	15.66181	44.16376	39.06721	6.515042	6.846041	1.648904	3.079129	1.820267	5.377848
AC007881.	4.804538	7.002733	23.97921	18.70727	6.64491	10.27698	4.248401	16.66849	22.17847	21.13538	13.2523	13.80117	8.224675	14.89152	13.69208	7.420066	10.26376	17.29253	14.34093
AC007899.	0.960908	13.00508	3.996535	4.157171	1.107485	6.851322	1.69936	5.556162	0	7.685594	3.614265	7.360626	0	0	0.978006	2.473355	2.052753	3.640534	3.585232
AC007906.	24.02269	5.001952	15.98614	11.43222	2.21497	1.141887	6.797442	5.556162	2.334575	9.606993	22.89034	4.600391	7.196591	9.307203	5.868035	14.84013	7.184634	19.1128	6.274156
AC007906.	1505.742	908.3545	1684.539	1008.114	1492.89	717.105	901.5107	1739.079	1558.329	1171.092	1403.539	1518.129	1699.423	1584.086	1610.776	1046.229	1046.904	913.774	625.6229
AC007907.	3.84363	6.002343	3.996535	0	1.107485	0	0	0	0	4.803496	0	0.920078	1.028084	0	0	0	0	0	0
AC007919.	2.882723	0	7.993069	0	0	0	1.69936	9.26027	0	1.921399	0	8.280704	0	0	0	0	0	0.910133	0.896308
AC007919.	0	0	0	0	0	0	0.84968	0	0	0	0	0	0	0	0	0.824452	0	0	0
AC007920.	0	0	0	0	0	0	0	0.926027	0	0	0	0	0	1.861441	0	0	0	4.550667	0
AC007920.	27.86632	16.00625	69.93936	1.039293	21.04221	19.41208	9.346483	25.00273	24.51304	26.89958	3.614265	15.64133	15.42127	32.57521	29.34017	9.06897	1.026376	1.820267	3.585232
AC007922.	0	1.00039	0	1.039293	5.537425	2.283774	2.549041	0	0	6.724895	1.204755	1.840156	6.168506	3.722881	0	2.473355	0	13.652	7.170464
AC007922.	0.960908	0	3.996535	7.275049	0	0	7.647122	1.852054	0	0	1.204755	3.680313	5.140422	1.861441	4.890029	4.122259	1.026376	2.7304	8.066772
AC007923.	13.45271	3.001171	3.996535	5.196464	3.322455	9.135096	2.549041	5.556162	14.00745	4.803496	0	6.440548	2.056169	6.515042	2.934017	12.36678	6.158258	3.640534	3.585232
AC007938.	94.16894	64.02499	41.96361	90.41847	40.97694	49.10114	73.0725	82.41641	51.36066	49.95636	49.39495	42.3236	57.57272	69.80402	51.83431	42.8715	43.1078	21.8432	20.61508
AC007938.	36.51449	13.00508	25.97748	27.02161	6.64491	7.993209	41.63433	21.29862	9.338302	0.960699	0	60.72516	42.15146	7.445762	25.42815	18.13794	8.21101	30.0344	22.4077
AC007938.	131.6443	155.0605	196.8293	106.0079	183.8425	135.8846	189.4787	157.4246	274.3126	217.118	185.5323	166.5342	152.1565	180.5597	175.063	89.86525	55.42432	164.7341	95.90495
AC007938.	69.18534	50.01952	89.92203	64.43615	48.72934	21.69585	59.47762	125.9397	32.68406	32.66377	22.89034	63.4854	79.1625	94.00275	69.43841	46.99375	51.31881	32.7648	35.85232
AC007948.	0	0	0	0	0	0	0	0	0	0	0	0	0	1.861441	0	0	0	0	0
AC007950.	0.960908	0	0	2.078586	0	0	2.549041	0	0	0	0	0.920078	0	0	0	0	0	3.640534	1.792616
AC007950.	5.765445	10.0039	10.99047	3.117878	11.07485	14.84453	7.647122	12.03835	29.18219	14.41049	7.228529	4.600391	3.084253	10.23792	3.912023	7.420066	15.39564	4.550667	3.585232
AC007952.	45.16265	9.003514	45.96015	4.157171	13.28982	54.81058	20.39233	9.26027	119.0633	60.52405	144.5706	62.56532	37.01104	25.12945	20.53812	25.55801	39.0023	15.47227	17.02985
AC007952.	9.609075	1.00039	1.998267	0	0	4.567548	0	0	0	2.882098	7.228529	0	0	0	0.978006	6.595615	7.184634	3.640534	1.792616
AC007952.	2.882723	2.000781	0.999134	8.314342	1.107485	3.425661	2.549041	1.852054	21.01118	0	0	6.440548	2.056169	4.653602	2.934017	22.2602	5.131881	14.56213	3.585232
AC007952.	15.37452	7.002733	4.995668	22.86444	8.859879	1.141887	0	3.704108	0	0	16.86657	11.96102	2.056169	26.99089	20.53812	30.50472	50.29244	40.956	27.78555
AC007952.	8.648168	2.000781	0	12.47151	0	1.141887	4.248401	0	8.171014	1.921399	0	5.520469	11.30893	6.515042	1.956012	4.122259	7.184634	7.281067	1.792616
AC007952.	0	0	0	0	0	0	0	0	0	0	0	0.920078	0	0	0.978006	0	0	0	0
AC007953.	6.726353	0	0	6.235757	2.21497	7.993209	0	0	4.669151	0	0	0	1.028084	0	0.978006	14.01568	26.68578	0	2.688924
AC007953.	45.16265	78.03045	58.94889	45.72888	76.41646	65.08756	60.3273	28.70684	78.20828	67.24895	33.73314	37.72321	21.58977	107.9636	47.92229	93.16306	136.508	103.7552	77.97879
AC007954.	3.84363	0	0	0	0	1.141887	1.69936	4.630135	0	0	7.228529	0	5.140422	0	0.978006	5.771163	1.026376	5.4608	0
AC007956.	77.83351	56.02186	60.94715	136.1474	104.1036	19.41208	91.76547	11.11232	9.338302	43.23147	6.023774	57.96493	50.37613	50.2589	62.59237	63.48279	68.76721	41.86614	49.29694
AC007966.	27.86632	75.02928	128.8882	131.9902	39.86946	62.80379	98.56291	98.15887	110.8923	88.38433	27.70936	34.04289	74.02207	96.79491	100.7346	26.38246	42.08143	33.67494	28.68185
AC007967.	0	1.00039	0.999134	0	0	0	0	0	0	0	0	0	0	0	0	0	0	0	0
AC007969.	18.25724	8.003124	15.98614	5.196464	8.859879	21.69585	11.04584	5.556162	16.34203	32.66377	19.27608	13.80117	14.39318	33.50593	7.824047	14.01568	27.71216	18.20267	13.44462
AC007969.	0	0	0	2.078586	1.107485	1.141887	0	3.704108	4.669151	0	0	2.760235	0	0.93072	6.846041	3.297807	0	4.550667	0
AC007991.	0.960908	0	0	1.039293	7.752394	1.141887	0.84968	9.26027	0	0	0	0	1.028084	3.722881	0.978006	0	1.026376	5.4608	0
AC007991.	3.84363	0	0	7.275049	1.107485	4.567548	3.398721	5.556162	2.334575	0	0	0.920078	1.028084	2.792161	1.956012	5.771163	2.052753	13.652	24.20031
AC007991.	0	0	0	0	1.107485	0	0	5.556162	0	0	0	0	1.028084	1.861441	1.956012	4.122259	0	1.820267	3.585232
AC007993.	4.804538	3.001171	0.999134	4.157171	0	1.141887	2.549041	0	0	0.960699	0	6.440548	2.056169	3.722881	2.934017	5.771163	3.079129	1.820267	3.585232
AC007993.	7.68726	4.001562	1.998267	6.235757	1.107485	2.283774	3.398721	0	0	1.921399	2.40951	3.680313	2.056169	5.584322	9.780058	3.297807	6.158258	2.7304	1.792616
AC007993.	9.609075	2.000781	0.999134	3.117878	3.322455	1.141887	4.248401	18.52054	0	9.606993	6.023774	11.96102	0	7.445762	10.75806	2.473355	3.079129	7.281067	1.792616
AC007996.	644.769	704.2749	624.4585	530.0393	724.2951	607.4839	723.0779	849.1668	772.7445	951.0923	751.767	507.8832	662.0863	741.7841	845.975	502.9156	611.7202	426.8526	422.161

AC007998.	15.37452	12.00469	49.95668	14.5501	11.07485	2.283774	10.19616	12.96438	3.501863	3.842797	13.2523	13.80117	26.73019	23.26801	35.20821	42.8715	22.58028	26.39387	14.34093
AC007998.	4.804538	3.001171	15.98614	5.196464	13.28982	12.56076	6.797442	15.74246	11.67288	9.606993	3.614265	11.04094	4.112337	6.515042	8.802052	10.71787	6.158258	4.550667	5.377848
AC007998.	17.29634	10.0039	8.992203	17.66798	8.859879	5.709435	7.647122	15.74246	0	6.724895	7.228529	6.440548	3.084253	4.653602	12.71408	2.473355	12.31652	4.550667	16.13354
AC008011.	0	0	0	2.078586	0	0	0	0	0	0	0	0	0	0	0	0	0	0	0
AC008011.	1.921815	2.000781	0	0	0	1.141887	0.84968	1.852054	4.669151	1.921399	0	0.920078	1.028084	0	0	0	4.105505	0.910133	0
AC008013.	87.44259	82.03202	173.8493	78.98625	137.3281	71.93888	157.1908	80.56435	89.88116	81.65944	114.4517	130.6511	164.4935	116.34	95.84457	67.60505	88.26836	93.74374	84.25295
AC008013.	62.45899	25.00976	84.92636	44.68959	84.16885	13.70264	93.46483	43.52327	79.37557	67.24895	89.15186	35.88305	54.48847	24.19873	31.29619	26.38246	16.42202	30.94454	69.01571
AC008013.	0	1.00039	0	0	7.752394	1.141887	2.549041	0	0	7.685594	1.204755	0.920078	0	5.584322	0	4.946711	0	0.910133	3.585232
AC008014.	229.6569	318.1242	354.6925	215.1336	233.6793	188.4114	223.4659	202.7999	393.376	140.2621	237.3367	239.2203	301.2287	272.701	333.5	328.1318	286.359	298.5238	246.4847
AC008015.	0	0	0	1.039293	1.107485	0	0	0	0	0	0	0	0	0	0	0	0	0	0
AC008021.	0.960908	6.002343	8.992203	5.196464	2.21497	5.709435	0.84968	0.926027	11.67288	4.803496	10.84279	2.760235	4.112337	5.584322	7.824047	0.824452	1.026376	0.910133	4.48154
AC008026.	7.68726	2.000781	4.995668	0	2.21497	1.141887	1.69936	3.704108	0	0	4.81902	3.680313	1.028084	3.722881	1.956012	6.595615	5.131881	14.56213	0
AC008026.	0	0	0	0	0	0	0	0	0	3.842797	0	0.920078	0	0	0	0	0	0	0
AC008026.	22.10087	3.001171	30.97314	4.157171	32.11706	6.851322	16.9936	16.66849	32.68406	12.48909	1.204755	4.600391	18.50552	29.78305	22.49413	7.420066	11.29014	17.29253	9.859387
AC008033.	0	2.000781	0.999134	0	0	3.425661	0.84968	0.926027	2.334575	0.960699	0	0.920078	1.028084	0.93072	0	0.824452	2.052753	0.910133	0
AC008033.	0	0	0	0	0	6.851322	0	0	0	0	0	0	0	0	0	0	0	0	0
AC008033.	0	2.000781	0	13.51081	0	2.283774	0.84968	12.96438	4.669151	7.685594	0	8.280704	7.196591	7.445762	0.978006	14.01568	3.079129	16.3824	2.688924
AC008035.	36.51449	39.01523	50.95582	28.0609	32.11706	28.54718	35.68657	43.52327	141.2418	38.42797	27.70936	38.64329	50.37613	50.2589	61.61437	29.68027	44.13418	44.59654	20.61508
AC008038.	479.4929	389.1519	328.715	276.4519	378.7598	296.8906	281.2442	515.7971	366.5283	352.5766	402.3881	299.9455	244.6841	456.0529	381.4223	438.6084	531.6629	486.0112	403.3386
AC008040.	8.648168	12.00469	19.98267	24.94303	8.859879	7.993209	16.9936	22.22465	36.18592	14.41049	9.638039	7.360626	3.084253	31.64449	17.6041	27.20691	12.31652	25.48374	41.23017
AC008050.	47.08447	24.00937	15.98614	27.02161	31.00958	43.39171	42.48401	12.96438	26.84762	26.89958	92.76613	43.24368	33.92678	40.02097	45.96627	56.88718	78.0046	19.1128	30.47447
AC008055.	0	0	0	0	0	0	0	0	0	0	0	0	0	0	0	0	0	0.910133	0.896308
AC008056.	0	5.001952	7.993069	11.43222	13.28982	3.425661	16.9936	7.408216	3.501863	5.764196	7.228529	4.600391	11.30893	11.16864	12.71408	6.595615	5.131881	6.370934	4.48154
AC008056.	7.68726	7.002733	46.95928	4.157171	1.107485	22.83774	8.496802	41.67122	1.167288	38.42797	57.82823	8.280704	2.056169	0.93072	9.780058	4.122259	4.105505	5.4608	3.585232
AC008060.	114.348	198.0773	99.91337	126.7937	53.15928	258.0665	223.4659	175.9451	115.5615	92.22713	112.0422	134.3314	132.6229	150.7767	140.8328	155.8214	130.3498	82.82214	160.4391
AC008060.	0	0	0	0	0	0	0	0	3.501863	0	0	0	0	0	0	0	0	0	0
AC008060.	240.2269	417.1628	264.7704	268.1375	233.6793	687.416	388.3039	200.0218	494.93	529.3453	610.8107	287.0644	310.4815	522.1341	401.9604	366.0566	409.5241	286.692	363.0047
AC008063.	23.06178	5.001952	10.99047	9.353635	1.107485	3.425661	1.69936	13.89041	5.836439	7.685594	1.204755	8.280704	4.112337	21.40657	12.71408	3.297807	4.105505	0.910133	0
AC008063.	0	0	0	0	0	0	0	0	0	0	0	0	0	0	0	0	0	3.640534	0
AC008065.	0.960908	0	0	0	0	0	0.84968	0.926027	0	0	1.204755	1.840156	0	0	0	0.824452	0	0	0
AC008066.	18.25724	15.00586	8.992203	13.51081	21.04221	19.41208	16.9936	15.74246	15.17474	15.37119	4.81902	33.12282	23.64594	13.9608	9.780058	4.122259	5.131881	8.191201	5.377848
AC008067.	22.10087	5.001952	0.999134	6.235757	0	18.27019	14.44456	11.11232	0	8.646293	1.204755	7.360626	2.056169	13.9608	13.69208	14.01568	14.36927	5.4608	8.963079
AC008069.	0	0	0	0	0	0	0	0	0	0	0	0	0	0	0	0.824452	4.105505	0	3.585232
AC008073.	43.24084	7.002733	36.96795	27.02161	47.62185	14.84453	26.34009	3.704108	40.85507	27.86028	7.228529	16.56141	17.47743	16.75297	10.75806	14.84013	15.39564	7.281067	15.23724
AC008073.	120.1134	98.03826	118.8969	101.8507	106.3186	63.94567	86.66738	52.78354	108.5578	133.5372	130.1135	72.68618	72.99399	84.69555	91.93255	115.4233	90.32111	78.27147	80.66772
AC008073.	7.68726	9.003514	19.98267	14.5501	9.967364	5.709435	14.44456	3.704108	0	9.606993	1.204755	8.280704	8.224675	11.16864	9.780058	14.01568	3.079129	6.370934	13.44462
AC008074.	62.45899	69.02694	94.9177	24.94303	45.40688	49.10114	49.28145	87.97257	32.68406	77.81664	48.1902	52.44446	62.71315	36.29809	57.70234	53.58937	49.26606	53.69787	60.94894
AC008074.	15.37452	7.002733	16.98527	13.51081	4.42994	2.283774	13.59488	15.74246	15.17474	12.48909	16.86657	17.48149	4.112337	24.19873	7.824047	9.06897	2.052753	8.191201	15.23724
AC008074.	128.7616	136.0531	111.903	91.45776	75.30897	122.1819	177.5832	104.6411	175.0932	113.3625	130.1135	107.6492	96.63993	139.608	147.6789	165.7148	165.2466	258.4779	219.5954
AC008079.	230.6178	225.0878	390.6613	263.9804	305.6658	355.1269	273.597	328.7396	281.3163	284.367	318.0553	297.1853	298.1445	239.1951	233.7434	347.0942	296.6227	322.1872	263.5145
AC008080.	8.648168	17.00664	16.98527	4.157171	19.93473	9.135096	11.04584	1.852054	16.34203	7.685594	26.50461	23.92203	18.50552	13.9608	20.53812	14.84013	32.84404	19.1128	13.44462
AC008080.	3.84363	2.000781	1.998267	8.314342	11.07485	2.283774	9.346483	3.704108	0	6.724895	0	5.520469	4.112337	0	8.802052	12.36678	7.184634	3.640534	5.377848
AC008080.	0	11.00429	5.994802	4.157171	8.859879	1.141887	5.098081	0.926027	4.669151	0.960699	0	5.520469	0	0.93072	3.912023	3.297807	2.052753	0.910133	0.896308
AC008080.	21.13997	27.01054	27.97574	30.13949	39.86946	10.27698	39.93497	42.59724	26.84762	32.66377	33.73314	29.4425	31.87062	34.43665	25.42815	31.32917	27.71216	20.02294	20.61508
AC008083.	0.960908	0	0	0	0	0	3.398721	0	7.003726	0	1.204755	0	0	0	0	0	0	0.910133	0
AC008083.	44.20175	13.00508	77.93243	34.29666	18.82724	12.56076	42.48401	28.70684	26.84762	8.646293	18.07132	17.48149	17.47743	29.78305	25.42815	61.00944	37.97592	82.82214	73.49725
AC008083.	0	1.00039	0	0	0	5.709435	0	0.926027	0	0	0	0	0	0.93072	0	0	0	3.640534	1.792616
AC008088.	0	0	0	0	0	0	0	1.852054	0	0	0	0	0	0	0	0	0	0	0
AC008105.	119.1525	106.0414	81.92896	148.6189	57.58922	61.6619	118.9552	67.59997	259.1379	55.72056	85.5376	115.9299	74.02207	69.80402	88.02052	80.79628	86.21561	143.8011	178.3653
AC008105.	1.921815	1.00039	1.998267	1.039293	1.107485	0	0	0.926027	10.50559	4.803496	22.89034	6.440548	2.056169	7.445762	1.956012	0.824452	0	0	5.377848
AC008105.	706.267	638.2491	702.391	742.055	565.9248	1049.394	464.7751	952.8818	1488.292	1139.389	1178.25	882.355	928.3602	658.0193	701.2302	676.0505	616.8521	722.6459	626.5193
AC008109.	3.84363	2.000781	0	2.078586	0	0	0.84968	0	0	0	0	0	0	0	0	2.473355	1.026376	0	0
AC008114.	5.765445	2.000781	9.991337	1.039293	2.21497	4.567548	5.098081	6.482189	9.338302	0	2.40951	9.200782	4.112337	4.653602	0.978006	1.648904	5.131881	6.370934	4.48154
AC008115.	0	4.001562	0.999134	1.039293	0	0	0.84968	0.926027	1.167288	0	0	0	0	0	0	0	0	0	0.896308
AC008115.	5.765445	13.00508	8.992203	0	6.64491	0	6.797442	0.926027	7.003726	0.960699	0	2.760235	2.056169	5.584322	1.956012	3.297807	0	9.101334	2.688924
AC008115.	442.9784	196.0765	411.6431	88.33989	235.8943	122.1819	202.2239	233.3588	50.19337	222.8822	127.704	244.7408	276.5547	245.7102	329.588	86.56744	90.32111	297.6136	185.5357
AC008115.	72.06806	69.02694	93.91856	75.86837	77.52394	29.68906	52.68017	56.48765	151.7474	36.50657	102.4042	45.08383	28.78636	38.15953	42.05425	28.03136	59.52982	55.51814	53.77848
AC008121.	2.882723	2.000781	11.9896	4.157171	5.537425	5.709435	10.19616	5.556162	1.167288	0.960699	16.86657	5.520469	9.252759	0.93072	0.978006	9.893422	11.29014	4.550667	0.896308
AC008121.	7.68726	24.00937	27.97574	19.74656	23.25718	36.54038	23.79105	38.89314	53.69524	32.66377	7.228529	32.20274	19.5336	16.75297	16.6261	25.55801	8.21101	10.01147	2.688924
AC008121.	1.921815	9.003514	15.98614	14.5501	0	0	17.12831	7.647122	15.74246	3.501863	2.882098	7.228529	1.840156	3.084253	0.93072	6.846041	5.771163	2.052753	1.820267	0.896308
AC008124.	4229.915	4169.627	4934.721	4038.692	4922.77	3482.755	4791.347	4225.461	2957.907	3789.959	3407.047	4321.607	4639.745	4379.97	4687.582	3315.121	3157.133	4395.944	4149.009
AC008125.	0	3.001171	4.995668	0	3.322455	0	3.398721	3.704108	0	0	0	0.920078	1.028084	4.653602	0	1.648904	0	0	0

AC008126.	0	0	0	0	0	1.141887	0	0	0	0	0	0	2.056169	0	0	0	1.026376	0	0
AC008127.	2.882723	1.00039	0.999134	0	0	1.141887	0	3.704108	0	3.842797	0	0	0	1.861441	0.978006	0	0	0.910133	5.377848
AC008127.	0	0	0	0	0	0	0	0	0	0.960699	0	0	0	0.93072	0	0	0	0	0
AC008132.	0	2.000781	3.996535	4.157171	4.42994	0	0	1.852054	5.836439	2.882098	0	0.920078	0	3.722881	0	1.648904	1.026376	0.910133	0
AC008133.	0	0	0	0	0	1.141887	1.69936	0	0	0	0	4.600391	0	0	0.978006	0	0	0	0.896308
AC008133.	0	0	3.996535	1.039293	0	1.141887	0.84968	0	0	0	0	0	0	0	0	0	1.026376	0	0
AC008147.	9.609075	4.001562	22.98007	3.117878	1.107485	0	9.346483	2.778081	10.50559	1.921399	3.614265	2.760235	2.056169	7.445762	12.71408	6.595615	0	0.910133	6.274156
AC008147.	73.98988	87.03397	216.812	91.45776	137.3281	82.21586	130.8508	183.3534	11.67288	128.7337	57.82823	209.7778	196.3641	146.1231	199.5132	79.97183	73.89909	84.64241	91.42341
AC008147.	0.960908	2.000781	0	1.039293	0	0	0	0.926027	1.167288	8.646293	10.84279	5.520469	1.028084	9.307203	1.956012	3.297807	0	0.910133	0.896308
AC008147.	20.17906	16.00625	21.98094	9.353635	11.07485	29.68906	22.94137	39.81916	22.17847	2.882098	16.86657	22.08188	21.58977	17.68369	33.2522	22.2602	3.079129	31.85467	17.02985
AC008149.	0.960908	0	2.997401	2.078586	0	0	1.69936	7.408216	8.171014	0.960699	3.614265	2.760235	2.056169	0	3.912023	3.297807	7.184634	0	0
AC008154.	0	0	0	0	0	0	0.84968	0	0	0	0	0	0	0	0	3.297807	1.026376	1.820267	0
AC008154.	0	0	0	0	0	0	0.84968	0	0	4.803496	10.84279	1.840156	0	0.93072	1.956012	2.473355	1.026376	0.910133	0
AC008155.	0.960908	3.001171	0.999134	1.039293	1.107485	0	2.549041	0.926027	1.167288	1.921399	0	4.600391	0	0.93072	0.978006	1.648904	0	4.550667	0.896308
AC008158.	104.7389	38.01484	3.996535	20.78586	159.4778	143.8778	43.33369	53.70957	222.952	40.34937	49.39495	54.28462	48.31997	97.72563	133.0088	12.36678	17.4484	121.9579	129.9647
AC008163.	1.921815	1.00039	2.997401	1.039293	1.107485	1.141887	3.398721	0	0	0	0	3.680313	5.140422	0.93072	3.912023	0	1.026376	0.910133	5.377848
AC008164.	0	0	0	0	0	0	0	0	0	0	0	0	1.028084	0	0.978006	0	0	1.820267	0
AC008171.	0	0	4.995668	0	0	4.567548	0.84968	3.704108	0	3.842797	1.204755	0	2.056169	9.307203	3.912023	0	0	5.4608	0
AC008243.	12.4918	28.01093	9.991337	27.02161	11.07485	19.41208	33.13753	43.52327	33.85134	49.95636	38.55216	15.64133	6.168506	21.40657	26.40616	17.31349	24.63303	13.652	17.02985
AC008250.	0.960908	4.001562	1.998267	0	0	0	0	0.926027	2.334575	0	4.81902	2.760235	1.028084	0	0	0.824452	0	0	2.688924
AC008250.	0.960908	0	0.999134	0	1.107485	0	0.84968	0	0	2.882098	0	6.440548	2.056169	0	0	4.946711	0	0	0
AC008264.	6.726353	4.001562	8.992203	6.235757	1.107485	5.709435	8.496802	4.630135	18.6766	3.842797	14.45706	6.440548	6.168506	1.861441	3.912023	0.824452	7.184634	4.550667	1.792616
AC008264.	171.0415	140.0547	136.8813	183.9548	208.2072	93.63473	231.113	173.1671	78.20828	106.6376	145.7753	177.5751	168.6058	150.7767	133.9868	92.3386	132.4025	94.65388	69.91202
AC008267.	6.726353	8.003124	0	0	7.752394	7.993209	0	0	4.669151	11.52839	13.2523	5.520469	0	1.861441	0	0	1.026376	3.640534	0.896308
AC008267.	9.609075	13.00508	25.97748	7.275049	9.967364	10.27698	11.89552	27.78081	22.17847	17.29259	7.228529	11.04094	9.252759	12.09936	10.75806	11.54233	14.36927	10.9216	23.30401
AC008267.	412.2293	382.1491	330.7132	220.3301	327.8155	400.8023	298.2378	268.5478	347.8517	408.2972	399.9786	453.5986	421.5146	326.6828	380.4443	267.1224	266.8578	251.1968	316.3967
AC008267.	0.960908	0	12.98874	2.078586	7.752394	0	0.84968	1.852054	0	3.842797	0	3.680313	4.112337	0	5.868035	7.420066	12.31652	0	0
AC008267.	0	0	1.998267	3.117878	2.21497	1.141887	2.549041	1.852054	0	1.921399	3.614265	2.760235	3.084253	0.93072	9.780058	0.824452	0	0	1.792616
AC008267.	0	0	0	2.078586	0	0	0.84968	0	0	0	0	0	1.028084	1.861441	0	0	0	0	0
AC008267.	2.882723	0	1.998267	6.235757	4.42994	5.709435	5.947762	0.926027	8.171014	0.960699	6.023774	3.680313	6.168506	0.93072	6.846041	3.297807	0	0	0
AC008268.	0	0	0	0	0	0	0.84968	0	16.34203	0	0	0	1.028084	0	0.978006	6.595615	1.026376	0.910133	0
AC008269.	9.609075	25.00976	21.98094	11.43222	8.859879	10.27698	22.09169	6.482189	15.17474	10.56769	15.66181	11.04094	9.252759	26.99089	13.69208	6.595615	14.36927	8.191201	10.7557
AC008269.	5.765445	0	0.999134	15.58939	7.752394	0	5.098081	3.704108	10.50559	9.606993	6.023774	0.920078	1.028084	9.307203	5.868035	10.71787	4.105505	7.281067	8.066772
AC008277.	1.921815	5.001952	1.998267	11.43222	0	1.141887	9.346483	11.11232	1.167288	7.685594	3.614265	2.760235	1.028084	0	1.956012	4.122259	2.052753	4.550667	6.274156
AC008277.	0	3.001171	4.995668	0	4.42994	0	2.549041	12.96438	0	0	1.204755	0	4.112337	0	1.956012	0	1.026376	0	0
AC008278.	0.960908	0	10.99047	1.039293	1.107485	0	0.84968	0	2.334575	0.960699	3.614265	0.920078	1.028084	0	4.890029	0	1.026376	3.640534	0.896308
AC008278.	4.804538	4.001562	9.991337	7.275049	0	0	5.947762	2.778081	8.171014	7.685594	0	5.520469	5.140422	4.653602	10.75806	8.244518	2.052753	5.4608	5.377848
AC008280.	2.882723	4.001562	4.995668	2.078586	1.107485	1.141887	4.248401	0	0	4.803496	0	11.04094	5.140422	0.93072	1.956012	4.122259	5.131881	0	3.585232
AC008280.	119.1525	55.02147	78.93156	45.72888	78.63143	153.0129	84.96802	114.8274	150.5801	142.1835	181.918	122.3704	126.4544	148.9152	158.4369	65.13169	51.31881	93.74374	116.52
AC008378.	0	0	0	1.039293	0	0	0	0	0	0	0	0	1.028084	0	0.978006	0.824452	0	3.640534	0
AC008379.	0	1.00039	1.998267	0	6.64491	4.567548	0	6.482189	0	0	0	0.920078	6.168506	0	5.868035	0	0	0.910133	0.896308
AC008385.	40.35812	15.00586	26.97661	5.196464	8.859879	25.12151	7.647122	32.41095	23.34575	31.70308	27.70936	19.32164	19.5336	30.71377	26.40616	3.297807	7.184634	1.820267	1.792616
AC008392.	0	0	0	0	0	1.141887	0	1.852054	0	0	0	0	1.028084	0	0	0	0	13.652	17.92616
AC008393.	256.5623	311.1214	343.702	234.8802	188.2724	215.8166	276.9958	214.8383	349.019	301.6596	368.655	270.503	228.2347	254.0866	371.6422	192.0973	234.0138	381.3459	343.2859
AC008395.	0	0	1.998267	1.039293	0	0	0	0.926027	0	0.960699	0	0	0	0.93072	0	0.824452	0	0	0
AC008403.	3.84363	1.00039	0	0	11.07485	6.851322	0	0.926027	1.167288	7.685594	4.81902	0.920078	6.168506	0	0.978006	0	1.026376	10.9216	0.896308
AC008406.	0	0	0	0	4.42994	0	0	0	0	0	0	0	0	0	0	0	0	0	0
AC008412.	0	0	0	0	0	0	0	0	0	0	1.204755	0	0	0	0	0.824452	0	0.910133	0
AC008413.	0	0	0	2.078586	0	0	0	0	1.167288	3.842797	0	0	0	0	0	0	0	0	0
AC008415.	0.960908	0	0.999134	1.039293	1.107485	7.993209	5.098081	0.926027	0	0.960699	0	5.520469	4.112337	0	1.956012	2.473355	5.131881	3.640534	5.377848
AC008417.	7.68726	4.001562	2.997401	18.70727	1.107485	10.27698	2.549041	21.29862	0	1.921399	0	8.280704	2.056169	0.93072	0.978006	8.244518	1.026376	2.7304	5.377848
AC008427.	0	2.000781	0	1.039293	0	0	0	1.852054	1.167288	0	1.204755	0	1.028084	0	0	0.824452	1.026376	0.910133	0.896308
AC008429.	191.2206	168.0656	131.8856	160.0511	136.2206	130.1751	185.2303	164.8328	123.7325	109.5197	144.5706	138.9318	162.4373	173.114	177.9971	117.0722	149.8509	142.8909	164.9207
AC008429.	24.9836	20.00781	25.97748	27.02161	23.25718	25.12151	15.29424	20.37259	14.00745	18.25329	14.45706	14.72125	22.61786	29.78305	20.53812	33.80253	32.84404	14.56213	15.23724
AC008429.	1.921815	0	5.994802	4.157171	7.752394	9.135096	1.69936	1.852054	0	0	3.614265	3.680313	5.140422	6.515042	4.890029	3.297807	9.237386	5.4608	1.792616
AC008429.	0.960908	6.002343	2.997401	2.078586	13.28982	0	7.647122	0	3.501863	0	0	4.600391	6.168506	13.9608	7.824047	3.297807	1.026376	4.550667	1.792616
AC008434.	51.88901	45.01757	23.97921	45.72888	63.12664	41.10793	29.73881	76.86024	8.171014	89.34503	49.39495	57.96493	69.90974	51.18962	51.83431	47.81821	35.92317	70.08027	56.4674
AC008438.	165.2761	302.1179	324.7184	128.8723	326.7081	161.0061	257.4531	237.0629	205.4426	329.5198	415.6404	247.501	275.5266	217.7886	204.4032	172.3104	196.0379	273.9502	266.2035
AC008438.	16.33543	12.00469	10.99047	18.70727	29.90209	12.56076	37.38593	11.11232	2.334575	18.25329	6.023774	30.36258	47.29188	14.89152	11.73607	23.9091	23.60665	52.78774	30.47447
AC008440.	0.960908	3.001171	0	4.157171	0	5.709435	3.398721	0	0	0.960699	0	1.840156	0	0	0	0	0	12.74187	0.896308
AC008440.	13.45271	26.01015	0.999134	3.117878	0	19.41208	16.14392	12.03835	23.34575	7.685594	4.81902	4.600391	13.3651	18.61441	38.14223	9.06897	8.21101	38.2256	35.85232
AC008443.	309.4122	262.1023	450.6093	152.776	367.685	310.5933	245.5576	349.1122	162.253	322.7949	452.9878	448.0781	401.981	358.3273	311.0059	236.6177	275.0688	542.4395	560.1925

AC008443.	24.02269	22.00859	41.96361	18.70727	42.08443	20.55397	24.64073	26.85478	29.18219	38.42797	12.04755	60.72516	43.17954	29.78305	25.42815	26.38246	21.5539	39.13574	38.54124
AC008443.	47.08447	20.00781	20.98181	80.02554	59.80419	12.56076	44.18337	56.48765	40.85507	40.34937	74.6948	33.12282	31.87062	35.36737	25.42815	23.08465	37.97592	48.23707	47.50432
AC008443.	26.90541	34.01327	32.97141	25.98232	17.71976	38.82416	30.58849	31.48492	78.20828	53.79916	71.08054	31.28266	18.50552	40.02097	32.27419	34.62698	29.76491	49.1472	38.54124
AC008443.	20.17906	21.0082	30.97314	36.37525	17.71976	10.27698	17.84329	32.41095	25.68033	25.93888	44.57593	28.52243	29.81445	5.584322	22.49413	15.66458	20.52753	24.5736	34.95601
AC008443.	17.29634	47.01835	29.97401	30.13949	33.22455	12.56076	25.49041	25.00273	5.836439	79.73804	56.62348	59.80509	38.03912	24.19873	39.12023	53.58937	35.92317	33.67494	59.15632
AC008448.	3.84363	1.00039	5.994802	1.039293	0	6.851322	2.549041	11.11232	3.501863	0.960699	3.614265	0	3.084253	8.376483	9.780058	3.297807	0	4.550667	0
AC008456.	0.960908	5.001952	29.97401	10.39293	13.28982	5.709435	14.44456	12.03835	8.171014	37.46727	0	22.08188	37.01104	13.03008	19.56012	2.473355	6.158258	12.74187	10.7557
AC008464.	68.22443	122.0476	73.93589	66.51474	90.81376	46.81737	39.08529	134.2739	25.68033	28.82098	36.14265	59.80509	56.54464	45.60529	47.92229	37.10033	52.34519	51.8776	49.29694
AC008467.	179.6897	154.0601	133.8839	107.0472	114.0709	332.2891	154.6418	362.0766	349.019	197.904	206.0131	399.314	352.6329	257.8095	338.39	117.0722	153.9564	94.65388	114.7274
AC008474.	0	0	0	0	0	1.141887	0	0	0	0	0	0	0	0	0	1.648904	0	0	0.896308
AC008481.	16.33543	11.00429	14.987	25.98232	5.537425	9.135096	15.29424	14.81643	1.167288	12.48909	12.04755	13.80117	31.87062	10.23792	4.890029	16.48904	12.31652	12.74187	7.170464
AC008481.	20.17906	13.00508	21.98094	27.02161	15.50479	5.709435	19.54265	34.263	11.67288	21.13538	30.11887	18.40156	20.56169	26.06017	20.53812	21.43575	33.87042	48.23707	21.51139
AC008494.	4.804538	26.01015	8.992203	9.353635	33.22455	12.56076	13.59488	18.52054	26.84762	20.17468	32.52838	23.92203	7.196591	12.09936	18.58211	23.9091	5.131881	4.550667	28.68185
AC008494.	21.13997	33.01288	36.96795	63.39686	14.3973	44.53359	32.28785	37.04108	0	15.37119	26.50461	32.20274	19.5336	44.67457	17.6041	40.39814	34.89679	38.2256	17.92616
AC008494.	38.4363	22.00859	60.94715	116.4008	23.25718	53.66869	35.68657	38.89314	25.68033	38.42797	15.66181	33.12282	31.87062	34.43665	17.6041	41.22259	62.60895	46.4168	54.67478
AC008496.	7.68726	0	2.997401	2.078586	0	2.283774	0	3.704108	0	1.921399	0	0	1.028084	3.722881	12.71408	4.122259	0	0	9.859387
AC008498.	76.8726	25.00976	60.94715	49.88605	50.94431	33.11472	65.42538	60.19176	28.01491	40.34937	32.52838	39.56336	38.03912	41.88241	27.38416	39.57369	52.34519	77.36134	51.08955
AC008498.	0	1.00039	0	1.039293	1.107485	1.141887	7.647122	1.852054	9.338302	0	0	1.840156	2.056169	1.861441	2.934017	4.122259	4.105505	0	4.48154
AC008505.	9.609075	10.0039	0.999134	5.196464	0	0	5.098081	7.408216	0	10.56769	0	0.920078	5.140422	17.68369	3.912023	6.595615	10.26376	0.910133	1.792616
AC008507.	203.7124	234.0914	217.8111	263.9804	237.0018	186.1276	174.1844	165.7588	368.8629	301.6596	286.7317	205.1774	191.2237	205.6892	188.7551	194.5706	218.6181	196.5888	170.2985
AC008507.	5.765445	21.0082	24.97834	53.00393	16.61227	3.425661	61.17698	41.67122	1.167288	10.56769	4.81902	33.12282	12.33701	21.40657	21.51613	37.10033	20.52753	26.39387	21.51139
AC008507.	0	0	0	0	0	0	0	0	0	0	0	1.840156	2.056169	0	0.978006	0	0	0	0
AC008507.	39.39721	47.01835	28.97488	43.6503	26.57964	39.96605	43.33369	25.00273	32.68406	32.66377	62.64725	28.52243	34.95487	27.92161	48.90029	30.50472	27.71216	30.0344	32.26709
AC008507.	0	0	0	0	0	3.425661	0.84968	0	0	0	3.614265	0	0	0	0	0	0	0	3.585232
AC008514.	11.53089	4.001562	10.99047	8.314342	0	1.141887	0.84968	1.852054	0	0.960699	4.81902	8.280704	8.224675	5.584322	8.802052	4.946711	8.21101	8.191201	16.13354
AC008514.	9.609075	0	7.993069	16.62868	4.42994	9.135096	9.346483	1.852054	1.167288	1.921399	4.81902	4.600391	11.30893	6.515042	5.868035	9.893422	2.052753	5.4608	1.792616
AC008517.	0	0	0	0	1.107485	0	0	0	4.669151	0	0	0	0	0	0.978006	0	0	0	0
AC008521.	0	4.001562	0	0	0	0	0.84968	0	0	0	0	0	0	0	0	0	0	0	0
AC008522.	7.68726	8.003124	15.98614	9.353635	21.04221	6.851322	28.88913	35.18903	23.34575	64.36685	33.73314	20.24172	8.224675	10.23792	17.6041	11.54233	13.34289	10.01147	17.92616
AC008525.	0	0	0	0	4.42994	0	0	0.926027	0	0	0	0	6.168506	0	0	0.824452	0	0	0
AC008526.	0	0	0.999134	2.078586	0	9.135096	3.398721	0.926027	0	0	0	0	1.028084	0.93072	0	0.824452	4.105505	1.820267	0
AC008534.	0.960908	0	0.999134	13.51081	0	7.993209	6.797442	11.11232	8.171014	12.48909	13.2523	8.280704	7.196591	19.54513	10.75806	5.771163	1.026376	3.640534	4.48154
AC008536.	337.2785	444.1734	394.6578	228.6444	429.7041	443.0522	457.128	564.8765	221.7847	408.2972	381.9073	370.7915	345.4363	383.4568	352.0821	305.0472	217.5918	299.4339	275.1665
AC008536.	71.10716	59.02304	71.93762	68.59332	66.4491	45.67548	90.06611	74.08216	53.69524	46.11356	93.97088	62.56532	74.02207	85.62627	74.32844	54.41382	54.39794	37.31547	29.57816
AC008536.	265.2105	312.1218	297.7418	259.8232	302.3434	316.3027	453.7292	438.9368	261.4725	401.5723	320.4648	362.5108	265.2458	270.8396	299.2698	197.8684	225.8028	303.0744	265.3072
AC008537.	230.6178	209.0816	208.8189	258.7839	237.0018	174.7087	241.3092	150.0164	8.171014	82.62014	72.28529	185.8558	203.5607	198.2434	148.6569	213.533	223.75	158.3632	197.1877
AC008537.	452.5874	345.1347	354.6925	392.8527	533.8077	334.5729	373.0096	325.9615	316.335	260.3495	274.6841	404.8344	389.644	344.3665	351.1041	394.088	410.5505	245.736	251.8625
AC008537.	123.9571	177.0691	233.7973	177.7191	188.2724	264.9178	209.871	244.4711	374.6994	241.1355	390.3406	159.1735	187.1114	138.6773	172.129	216.0064	165.2466	194.7686	150.5797
AC008543.	123.9571	146.057	101.9116	188.112	146.188	150.7291	111.3081	103.715	66.5354	186.3757	154.2086	109.4893	89.44334	85.62627	127.1408	88.21635	101.6112	104.6653	80.66772
AC008543.	0.960908	2.000781	0	0	1.107485	1.141887	0.84968	3.704108	2.334575	0	0	1.840156	0	0.93072	0.978006	0.824452	0	0	0
AC008543.	14.41361	9.003514	9.991337	6.235757	16.61227	1.141887	19.54265	14.81643	9.338302	5.764196	9.638039	10.12086	12.33701	14.89152	7.824047	16.48904	11.29014	3.640534	3.585232
AC008543.	14.41361	14.00547	10.99047	9.353635	16.61227	10.27698	4.248401	17.59451	26.84762	9.606993	15.66181	3.680313	5.140422	5.584322	13.69208	7.420066	3.079129	5.4608	2.688924
AC008543.	1.921815	5.001952	1.998267	1.039293	21.04221	0	14.44456	4.630135	1.167288	7.685594	7.228529	21.1618	9.252759	12.09936	5.868035	3.297807	7.184634	10.01147	21.51139
AC008549.	0	0	0	0	0	0	0	0	0	0	0	0	0	0.93072	0	0	0	0.910133	0
AC008549.	0	0	0	0	0	0	0	1.852054	0	0	0	0	0	1.861441	0	0	0	0	0
AC008549.	1.921815	1.00039	0	0	0	0	0	0	0	0.960699	0	0	0	0	0	0	0	0	0
AC008550.	0	0	0.999134	0	0	2.283774	1.69936	0	0	0	0	0.920078	0	0	0.978006	0	0	0	0
AC008551.	10.56998	8.003124	8.992203	13.51081	0	25.12151	6.797442	20.37259	11.67288	1.921399	9.638039	11.96102	19.5336	18.61441	0.978006	20.6113	24.63303	0.910133	11.652
AC008554.	1.921815	0	0	0	0	1.141887	0	0	12.84017	0	0	0	0	0	0	0.824452	0	0	1.792616
AC008555.	72.06806	46.01796	30.97314	23.90373	53.15928	17.12831	18.69297	42.59724	15.17474	35.54587	28.91412	23.00196	21.58977	82.83411	64.54838	38.74924	24.63303	7.281067	6.274156
AC008555.	73.02897	20.00781	28.97488	44.68959	63.12664	97.0604	24.64073	18.52054	86.37929	28.82098	42.16642	100.2885	52.4323	73.5269	72.37243	34.62698	66.71446	54.608	32.26709
AC008555.	0.960908	0	0	7.275049	4.42994	0	0	0	0	0.960699	0	0.920078	0	0	0	0	1.026376	0	0
AC008555.	0	0	0	0	6.64491	0	0	0	0	4.803496	4.81902	0	0	0.93072	3.912023	0.824452	3.079129	0	0
AC008555.	37.47539	23.00898	14.987	20.78586	52.05179	31.97284	6.797442	21.29862	68.86998	9.606993	36.14265	34.96297	35.98295	31.64449	47.92229	18.96239	30.79129	36.40534	12.54831
AC008555.	0	0	0	0	0	0	1.69936	0	0	0	0	0	0	0	3.912023	0.824452	0	0	0.896308
AC008555.	352.6531	278.1085	261.773	345.0452	242.5392	220.3842	169.936	212.9862	207.7772	268.0351	224.0844	217.1385	234.4032	269.9089	198.5352	286.9092	259.6732	244.8259	162.2317
AC008556.	154.7061	177.0691	143.8752	131.9902	233.6793	227.2355	83.26866	164.8328	421.3909	366.9871	440.9403	120.5302	174.7743	180.5597	139.8548	191.2728	127.2707	129.2389	174.7801
AC008560.	417.0339	350.1367	316.7254	435.4637	271.3338	436.2008	365.3625	426.8985	412.0526	429.4326	475.8782	359.7506	302.2568	317.3756	355.9941	460.8686	366.4163	364.0534	224.077
AC008565.	0	0	0	0	0	0	0	0	0	0	0	0	0	0	0.978006	0	0	0	0.896308
AC008567.	0.960908	0	0	1.039293	1.107485	1.141887	2.549041	5.556162	0	0	12.04755	0.920078	1.028084	4.653602	3.912023	0.824452	0	0	0.896308
AC008567.	24.9836	25.00976	13.98787	17.66798	2.21497	11.41887	14.44456	14.81643	9.338302	17.29259	42.16642	3.680313	10.28084	12.09936	13.69208	7.420066	6.158258	3.640534	10.7557
AC008567.	395.8939	365.1425	272.7635	460.4067	397.5871	421.3563	361.1141	215.7643	787.9192	274.76	289.1412	394.7136	480.1154	292.2462	223.9633	434.4861	443.3945	425.0323	571.8845

AC008569.	59.57627	62.02421	54.95235	40.53242	55.37425	62.80379	52.68017	71.30408	116.7288	71.09174	71.08054	102.1287	45.23571	40.02097	41.07624	54.41382	61.58258	56.42827	54.67478
AC008574.	0	0	0	1.039293	1.107485	0	0	0	0	0	0	0	0	0	0	0	0	0	0
AC008575.	0	0	0.999134	0	0	0	1.69936	3.704108	0	0	0	2.760235	0	1.861441	0	1.648904	0	0	0.896308
AC008575.	58.61536	52.0203	21.98094	45.72888	22.1497	37.68227	45.03305	46.30135	7.003726	24.01748	12.04755	36.80313	44.20763	25.12945	43.03226	31.32917	28.73854	25.48374	17.92616
AC008588.	0	0	0	0	0	0	0	0	1.167288	0	3.614265	0.920078	0	0	0	3.297807	0	0	1.792616
AC008591.	3.84363	1.00039	10.99047	13.51081	4.42994	7.993209	9.346483	15.74246	7.003726	13.44979	3.614265	16.56141	7.196591	7.445762	17.6041	1.648904	5.131881	0	8.963079
AC008592.	19.21815	5.001952	8.992203	7.275049	2.21497	12.56076	3.398721	9.26027	7.003726	8.646293	0	1.840156	3.084253	3.722881	13.69208	0.824452	5.131881	8.191201	12.54831
AC008592.	0	0	0.999134	0	2.21497	0	0	0	0	0	0	0.920078	0	0	0	0	0	0	0
AC008592.	16.33543	10.0039	30.97314	7.275049	14.3973	33.11472	11.04584	29.63287	16.34203	24.97818	25.29985	27.60235	16.44935	39.09025	31.29619	25.55801	19.50115	62.79921	18.82247
AC008592.	11.53089	6.002343	25.97748	9.353635	8.859879	13.70264	3.398721	1.852054	8.171014	1.921399	3.614265	5.520469	14.39318	1.861441	7.824047	9.893422	7.184634	20.93307	3.585232
AC008592.	4.804538	14.00547	4.995668	5.196464	12.18233	12.56076	5.947762	7.408216	10.50559	5.764196	4.81902	10.12086	20.56169	4.653602	1.956012	12.36678	20.52753	6.370934	18.82247
AC008601.	0	0	0	0	0	0	0	0	0	0	0	0	0	0	0	0	0	0	4.48154
AC008608.	0.960908	0	0	0	0	0	0	0	0	0	1.204755	0	2.056169	0	0	0	0	1.820267	0.896308
AC008608.	1.921815	4.001562	5.994802	1.039293	12.18233	1.141887	3.398721	3.704108	5.836439	16.33189	22.89034	4.600391	8.224675	10.23792	3.912023	6.595615	3.079129	9.101334	10.7557
AC008609.	11.53089	4.001562	10.99047	10.39293	2.21497	4.567548	5.098081	0.926027	1.167288	2.882098	0	22.08188	14.39318	5.584322	2.934017	6.595615	3.079129	4.550667	8.963079
AC008610.	267.1323	279.1089	222.8068	145.501	310.0958	310.5933	163.9883	249.1013	468.0824	329.5198	406.0024	298.1053	283.7513	252.2252	169.195	141.8057	218.6181	343.1203	289.5075
AC008619.	36.51449	57.02225	23.97921	43.6503	45.40688	17.12831	27.18977	37.96711	4.669151	22.09608	8.433284	18.40156	19.5336	66.08114	38.14223	20.6113	17.4484	38.2256	60.05263
AC008620.	0	0	0	0	1.107485	4.567548	0	0	0	0	0	0	0	0	0	0	0	0.910133	0
AC008622.	0.960908	1.00039	0	1.039293	7.752394	6.851322	4.248401	5.556162	0	0.960699	2.40951	3.680313	0	0	3.912023	4.946711	1.026376	0	0
AC008622.	61.49808	63.0246	33.97054	55.08252	87.49131	71.93888	48.43177	41.67122	117.8961	76.85594	56.62348	55.20469	50.37613	50.2589	50.8563	65.95615	66.71446	73.72081	35.85232
AC008626.	0	0	0.999134	0	0	0	0	0	0	0.960699	0	0	0	0.93072	0.978006	0	1.026376	1.820267	0
AC008627.	0	6.002343	0	0	0	0	3.398721	0.926027	0	0	0	0	0	0	0	0	2.052753	0	0
AC008628.	0	0	0	0	0	1.141887	0.84968	0	0	0	0	0	0	0	2.934017	0.824452	0	0	0
AC008629.	53.81082	56.02186	71.93762	18.70727	97.45867	49.10114	68.8241	53.70957	58.36439	32.66377	36.14265	37.72321	47.29188	43.74385	31.29619	34.62698	32.84404	36.40534	56.4674
AC008629.	22.10087	24.00937	33.97054	8.314342	13.28982	25.12151	16.14392	35.18903	3.501863	13.44979	19.27608	22.08188	18.50552	5.584322	1.956012	0.824452	12.31652	0	2.688924
AC008629.	2.882723	6.002343	4.995668	3.117878	0	1.141887	11.04584	4.630135	0	0	0	3.680313	11.30893	0.93072	0.978006	1.648904	0	0	1.792616
AC008632.	9.609075	2.000781	5.994802	4.157171	0	4.567548	5.947762	6.482189	0	2.882098	0	0.920078	6.168506	0	2.934017	0	0	3.640534	0.896308
AC008635.	11.53089	9.003514	31.97228	10.39293	11.07485	11.41887	10.19616	10.1863	2.334575	18.25329	13.2523	14.72125	31.87062	16.75297	7.824047	9.893422	8.21101	20.93307	10.7557
AC008638.	0	0	0	0	0	0	0.84968	0	0	0.960699	0	0	0	0	3.912023	0	0	0	0
AC008641.	5.765445	8.003124	21.98094	32.21808	3.322455	22.83774	24.64073	11.11232	9.338302	7.685594	0	15.64133	29.81445	17.68369	15.64809	9.893422	8.21101	28.21414	10.7557
AC008649.	10.56998	6.002343	1.998267	6.235757	2.21497	4.567548	1.69936	1.852054	16.34203	6.724895	3.614265	0.920078	4.112337	2.792161	1.956012	3.297807	3.079129	3.640534	3.585232
AC008649.	0	1.00039	10.99047	7.275049	0	0	10.19616	4.630135	0	4.803496	0	1.840156	4.112337	0.93072	0	0.824452	6.158258	0.910133	0.896308
AC008652.	5.765445	6.002343	4.995668	7.275049	2.21497	2.283774	7.647122	8.334243	8.171014	0	0	0.920078	1.028084	0.93072	0	0.824452	10.26376	0	2.688924
AC008655.	98.97348	40.01562	70.93849	71.7112	49.83682	66.22945	73.0725	82.41641	61.86625	66.28825	75.89956	69.00587	58.60081	86.55699	99.75659	50.29156	50.29244	70.99041	60.94894
AC008659.	0	0	0	0	0	0	3.398721	0	0	0	0	0	12.33701	2.792161	4.890029	0.824452	0	1.820267	0.896308
AC008663.	0.960908	1.00039	0	6.235757	0	2.283774	3.398721	4.630135	0	0	2.40951	2.760235	1.028084	1.861441	0.978006	0.824452	4.105505	0.910133	0.896308
AC008663.	111.4653	212.0828	210.8172	248.391	263.5814	116.4725	249.806	130.5698	128.4017	237.2927	210.8321	192.2964	177.8586	131.2316	125.1847	143.4546	161.1411	231.1739	199.8767
AC008663.	28.82723	40.01562	14.987	12.47151	36.547	12.56076	18.69297	25.00273	37.35321	33.62447	48.1902	25.76219	23.64594	19.54513	9.780058	7.420066	10.26376	7.281067	8.066772
AC008667.	114.348	78.03045	92.91943	59.23969	57.58922	36.54038	197.1258	112.9753	36.18592	20.17468	27.70936	73.60626	114.1174	80.97267	59.65836	113.7744	100.5849	205.6902	174.7801
AC008667.	55.73264	54.02108	24.97834	31.17878	22.1497	42.24982	39.08529	38.89314	67.70269	62.44545	102.4042	21.1618	40.09529	24.19873	25.42815	40.39814	48.23968	31.85467	60.05263
AC008667.	25.9445	7.002733	11.9896	9.353635	12.18233	10.27698	5.947762	2.778081	0	2.882098	3.614265	10.12086	8.224675	20.47585	16.6261	9.06897	9.237386	28.21414	36.74863
AC008667.	3.84363	0	0	0	0	1.141887	0.84968	0	0	0.960699	0	0	0	0.93072	0	1.648904	0	3.640534	0
AC008669.	374.7539	430.1679	372.6769	686.9725	481.7559	351.7012	399.3497	419.4902	199.6062	351.6159	291.5507	360.6707	419.4584	422.547	413.6965	371.0033	255.5677	299.4339	226.7659
AC008676.	28.82723	22.00859	61.94629	28.0609	32.11706	14.84453	50.13113	13.89041	42.02236	20.17468	6.023774	34.96297	32.8987	32.57521	29.34017	22.2602	25.65941	25.48374	32.26709
AC008676.	22.10087	8.003124	15.98614	13.51081	5.537425	5.709435	7.647122	3.704108	40.85507	7.685594	7.228529	11.04094	26.73019	13.03008	9.780058	14.01568	24.63303	21.8432	22.4077
AC008677.	0	0	0.999134	0	1.107485	0	0	0	0	0	0	0	0	0	0	0	0	0	1.792616
AC008680.	1.921815	7.002733	4.995668	1.039293	7.752394	5.709435	1.69936	27.78081	0	13.44979	0	6.440548	8.224675	7.445762	2.934017	0	11.29014	0.910133	0
AC008686.	0	0	0	0	0	0	0	0	0	0	0	0	2.056169	0	0	0	0	0	0
AC008687.	0	0	15.98614	0	0	1.141887	0	0	0	0	8.433284	3.680313	1.028084	0	0	0	0	0.910133	0.896308
AC008687.	0	1.00039	0	0	0	0	0.84968	0	0	1.921399	0	0	0	0	0	1.648904	4.105505	0.910133	0
AC008687.	65.34171	19.00742	46.95928	24.94303	37.65449	54.81058	34.83689	16.66849	33.85134	72.05244	59.03299	37.72321	28.78636	21.40657	58.68035	51.11601	54.39794	34.58507	25.99293
AC008691.	36.51449	63.0246	41.96361	72.75049	18.82724	29.68906	25.49041	22.22465	57.1971	53.79916	19.27608	41.40352	14.39318	23.26801	22.49413	17.31349	12.31652	4.550667	7.170464
AC008694.	0.960908	0	0	0	0	0	0.84968	0.926027	0	0	0	0.920078	0	0.93072	0	0	0	0.910133	0
AC008694.	0	0	0	2.078586	0	0	0	0	0	0	0	0	0	0	0	0	0	0	0
AC008696.	0.960908	4.001562	7.993069	2.078586	0	1.141887	1.69936	2.778081	12.84017	0.960699	2.40951	2.760235	0	6.515042	5.868035	0	0	0.910133	3.585232
AC008696.	0	6.002343	0	4.157171	0	0	9.346483	0	7.003726	0	0	0	0	0	1.956012	0	0	0	0
AC008700.	7.68726	1.00039	8.992203	0	4.42994	2.283774	0.84968	1.852054	3.501863	0	14.45706	0	2.056169	0	0	0	2.052753	0	0.896308
AC008703.	0	4.001562	1.998267	0	0	0	0	0	0	0	0	2.760235	0	0	7.824047	3.297807	0	0	0
AC008705.	4.804538	7.002733	12.98874	5.196464	15.50479	4.567548	5.947762	12.03835	19.84389	14.41049	0	9.200782	8.224675	6.515042	8.802052	8.244518	13.34289	9.101334	8.066772
AC008708.	246.9532	134.0523	135.8822	112.2436	215.9596	51.38492	118.9552	89.82462	127.2344	107.5983	73.49005	119.6102	89.44334	264.3246	244.5015	104.7054	94.42662	133.7896	76.18618
AC008708.	84.55986	139.0543	73.93589	82.10413	141.7581	137.0264	95.16419	143.5342	222.952	126.8123	81.92333	83.72712	71.96591	109.825	133.9868	118.7211	132.4025	64.61947	83.35664
AC008716.	0.960908	0	0.999134	5.196464	0	0	0.84968	0	0	0	0	0	0	1.028084	3.722881	0	0.824452	2.052753	0	0

AC008725.	0	4.001562	0	0	0	0	0.84968	0	0	0	0	0	0	0	1.956012	0.824452	0	0	0
AC008728.	7.68726	0	3.996535	0	18.82724	0	9.346483	0	0	1.921399	0	3.680313	1.028084	5.584322	2.934017	2.473355	1.026376	0.910133	3.585232
AC008731.	97.05166	89.03475	260.7739	139.2652	150.6179	98.20228	185.2303	186.1314	45.52422	144.1049	95.17564	223.579	192.2518	191.7284	168.217	112.9499	113.9278	135.6099	197.1877
AC008734.	71.10716	85.03319	121.8943	70.67191	146.188	60.52001	113.0075	122.2356	42.02236	102.7948	83.12809	158.2535	119.2578	108.8943	125.1847	97.28532	86.21561	100.1147	111.1422
AC008734.	14.41361	5.001952	15.98614	0	0	14.84453	2.549041	6.482189	10.50559	0	7.228529	9.200782	4.112337	0.93072	4.890029	5.771163	4.105505	8.191201	1.792616
AC008735.	136.4489	111.0433	151.8683	112.2436	86.38382	132.4589	161.4392	152.7945	87.54658	187.3364	133.7278	126.9708	80.19058	84.69555	103.6686	126.9656	175.5103	248.4664	178.3653
AC008735.	350.7312	346.1351	317.7245	273.334	364.3625	412.2212	269.3486	357.4464	560.2981	535.1095	621.6535	440.7175	469.8346	403.0019	446.9487	300.9249	274.0425	425.0323	336.1155
AC008735.	3.84363	0	0	0	0	0	1.69936	0	0	0	3.614265	0.920078	0	0	0	3.297807	1.026376	0	0
AC008735.	192.1815	138.0539	153.8666	187.0727	155.0479	190.6951	177.5832	215.7643	241.6286	217.118	186.737	139.8519	159.3531	170.3218	216.1393	326.4829	267.8842	212.9712	217.8028
AC008735.	0	0	0	0	0	1.141887	0.84968	0	0	0	0	0	0	0.93072	0	0.824452	0	0	0
AC008735.	0	0	0	1.039293	0	0	0	0	0	3.842797	0	0	1.028084	0	0	0	0	0	0
AC008736.	116.2698	153.0597	109.9047	116.4008	177.1976	113.0468	110.4584	87.04654	256.8033	79.73804	120.4755	102.1287	110.005	123.7858	143.7669	162.417	146.7718	116.4971	145.2019
AC008736.	1.921815	9.003514	19.98267	10.39293	25.47215	14.84453	1.69936	7.408216	21.01118	20.17468	16.86657	4.600391	4.112337	8.376483	16.6261	15.66458	5.131881	2.7304	8.066772
AC008737.	132.6052	72.02811	106.9073	91.45776	69.77155	26.2634	87.51706	56.48765	7.003726	86.46293	91.56137	116.8499	141.8756	96.79491	94.86656	84.09409	110.8486	91.01334	86.94187
AC008737.	2.882723	5.001952	0	2.078586	0	2.283774	0.84968	0	0	0	7.228529	0	0	2.792161	5.868035	0	1.026376	0.910133	0.896308
AC008737.	0.960908	0	0.999134	0	0	0	0	0	0	0	3.614265	1.840156	5.140422	0.93072	0	1.648904	1.026376	0	0
AC008738.	9.609075	13.00508	34.96968	16.62868	19.93473	14.84453	25.49041	11.11232	0	14.41049	8.433284	16.56141	16.44935	19.54513	31.29619	14.01568	12.31652	20.93307	13.44462
AC008738.	18.25724	33.01288	27.97574	16.62868	17.71976	20.55397	17.84329	37.04108	15.17474	7.685594	16.86657	22.08188	16.44935	17.68369	12.71408	21.43575	9.237386	13.652	14.34093
AC008738.	1.921815	0	2.997401	0	6.64491	1.141887	0	0	0	3.842797	3.614265	0.920078	2.056169	0	0.978006	0	4.105505	0	0
AC008738.	0	0	0	0	0	0	0	0	7.003726	0	0	0.920078	0	0	0	0	2.052753	0.910133	0
AC008739.	0	0	0	0	0	0	0	0	0	0	0	0	6.168506	0	0	0	0	0	0
AC008739.	1.921815	9.003514	11.9896	8.314342	4.42994	19.41208	2.549041	10.1863	28.01491	9.606993	16.86657	21.1618	11.30893	10.23792	15.64809	5.771163	7.184634	5.4608	24.20031
AC008739.	5.765445	0	1.998267	6.235757	0	0	5.947762	4.630135	0	0	0	0	0	0	0	0	0	0	2.688924
AC008739.	0.960908	0	0.999134	8.314342	0	0	2.549041	0.926027	0	1.921399	0	0.920078	0	0.93072	0	1.648904	0	3.640534	3.585232
AC008740.	0	0	4.995668	0	2.21497	1.141887	0	0	0	0	2.40951	1.840156	1.028084	1.861441	0	1.648904	1.026376	0	3.585232
AC008740.	0	0	0	0	0	0	1.69936	0	0	0.960699	0	0.920078	5.140422	0	0	0	0	0	0
AC008741.	868.6604	1112.434	1498.7	450.0138	1307.94	1252.65	942.2954	1501.09	1166.12	1802.272	1849.299	1198.862	1101.078	1320.692	1258.693	582.063	631.2214	1160.42	890.9301
AC008742.	43.24084	84.0328	108.9056	67.55403	192.7024	102.7698	84.11834	77.78627	141.2418	163.3189	137.3421	131.5712	128.5105	63.28898	89.97654	59.36053	72.87271	80.09174	52.88217
AC008743.	5.765445	7.002733	0	19.74656	0	2.283774	11.04584	3.704108	0	13.44979	8.433284	1.840156	7.196591	1.861441	0.978006	4.122259	3.079129	2.7304	13.44462
AC008745.	0	0	0	0	0	0	1.69936	0	0	0	0	0	0	1.861441	0	0	0	0	0.896308
AC008747.	0.960908	0	0.999134	2.078586	0	0	0	0.926027	9.338302	0	7.228529	0	1.028084	1.861441	0.978006	0.824452	0	1.820267	2.688924
AC008750.	26.90541	67.02616	81.92896	99.77211	84.16885	82.21586	33.98721	93.52873	89.88116	48.03496	109.6327	62.56532	30.84253	106.1021	144.7449	267.9468	209.3808	168.3747	200.773
AC008750.	20.17906	16.00625	15.98614	39.49313	40.97694	22.83774	31.43817	38.89314	28.01491	6.724895	45.78069	15.64133	22.61786	26.06017	53.79032	112.9499	85.18923	84.64241	74.39356
AC008750.	6.726353	11.00429	12.98874	13.51081	12.18233	22.83774	25.49041	15.74246	45.52422	9.606993	30.11887	11.96102	30.84253	8.376483	11.73607	4.122259	15.39564	27.304	27.78555
AC008750.	0	3.001171	0	0	0	0	0	0	0	0	0	0	0	0	0	0	0	0	0
AC008750.	21.13997	45.01757	55.95149	42.611	24.36467	37.68227	52.68017	45.37532	36.18592	37.46727	20.48083	46.92399	46.2638	65.15042	48.90029	48.64266	23.60665	52.78774	43.91909
AC008750.	0	4.001562	1.998267	3.117878	0	1.141887	2.549041	0	0	0	0	2.760235	1.028084	0	0.978006	7.420066	1.026376	6.370934	5.377848
AC008750.	0	0	0	0	0	0	0	0	0	0	6.023774	0	0	0	0	0	0	1.820267	0.896308
AC008751.	0	4.001562	0	0	0	1.141887	0	0	0	0	0	0	0	0	0	0	0	0	0
AC008751.	0.960908	3.001171	0.999134	4.157171	6.64491	0	0	0.926027	1.167288	0	0	0	1.028084	4.653602	3.912023	0.824452	3.079129	0	0
AC008752.	1.921815	3.001171	5.994802	0	0	4.567548	0.84968	5.556162	1.167288	3.842797	0	3.680313	1.028084	0.93072	0.978006	0.824452	0	0	0.896308
AC008752.	32.67086	6.002343	31.97228	10.39293	8.859879	7.993209	4.248401	32.41095	22.17847	28.82098	39.75691	28.52243	33.92678	25.12945	33.2522	9.893422	24.63303	16.3824	18.82247
AC008752.	494.8674	446.1741	532.5382	628.7721	454.0688	527.5518	505.5597	643.5888	236.9594	562.9698	383.1121	609.0918	646.6651	471.8752	563.3314	417.1726	454.6847	425.0323	381.8272
AC008753.	0	0	0	0	0	0	0	0	0	0	0	0.920078	0	0	0	0	1.026376	0	0
AC008753.	0.960908	4.001562	0	0	6.64491	0	0	0	0	0	0	3.680313	2.056169	0	0	0.824452	1.026376	0	0.896308
AC008753.	0	0	0	4.157171	0	0	0	1.852054	0	0	0	0	3.084253	0	0	0	0	0	0
AC008758.	17.29634	20.00781	19.98267	19.74656	7.752394	34.25661	20.39233	36.11505	11.67288	17.29259	4.81902	26.68227	28.78636	16.75297	20.53812	14.01568	19.50115	14.56213	19.71877
AC008758.	15.37452	32.01249	11.9896	8.314342	39.86946	38.82416	9.346483	42.59724	79.37557	37.46727	37.3474	7.360626	10.28084	21.40657	19.56012	15.66458	16.42202	13.652	22.4077
AC008758.	15.37452	19.00742	37.96708	21.82515	24.36467	20.55397	17.84329	6.482189	19.84389	20.17468	14.45706	19.32164	13.3651	19.54513	17.6041	16.48904	25.65941	25.48374	24.20031
AC008759.	20.17906	25.00976	11.9896	34.29666	14.3973	28.54718	14.44456	29.63287	29.18219	23.05678	15.66181	26.68227	30.84253	22.33729	19.56012	20.6113	4.105505	12.74187	19.71877
AC008759.	190.2597	147.0574	147.8718	81.06484	114.0709	187.2695	61.17698	227.8027	438.9002	169.0831	101.1994	122.3704	155.2407	132.1623	118.3387	108.8276	151.9037	100.1147	86.04556
AC008759.	165.2761	208.0812	222.8068	170.444	2.21497	263.7759	63.72602	348.1862	429.5619	53.79916	49.39495	138.0117	129.5386	224.3036	213.2053	331.4296	230.9347	192.9483	130.861
AC008760.	62.45899	133.0519	108.9056	129.9116	110.7485	126.7495	94.31451	101.863	61.86625	97.03062	113.247	139.8519	110.005	99.58707	120.2947	93.16306	88.26836	222.9827	150.5797
AC008760.	97.05166	58.02265	102.9108	116.4008	316.7407	102.7698	28.88913	285.2163	84.04472	161.3975	175.8942	174.8149	129.5386	125.6472	159.4149	319.8873	246.3303	331.2886	292.1964
AC008761.	49.96719	29.01132	18.98354	91.45776	52.05179	49.10114	50.13113	36.11505	12.84017	48.03496	39.75691	29.4425	68.88165	42.81313	25.42815	81.62073	118.0333	36.40534	65.43048
AC008761.	47.08447	34.01327	20.98181	82.10413	34.33203	54.81058	57.77826	50.93149	17.50932	45.15286	4.81902	57.04485	44.20763	69.80402	62.59237	127.79	138.5608	56.42827	78.8751
AC008763.	14.41361	9.003514	4.995668	27.02161	18.82724	10.27698	14.44456	48.15341	46.69151	6.724895	18.07132	14.72125	10.28084	20.47585	16.6261	14.84013	4.105505	19.1128	9.859387
AC008763.	0	0	0	2.078586	0	0	0.84968	0	0	0	0	0	0	0	0	0.824452	0	0	0.896308
AC008764.	0	0	0.999134	0	0	0	0	0	0	0	0	0	0	0	0	4.946711	0	0.910133	0
AC008764.	0	1.00039	2.997401	2.078586	0	0	9.346483	0	0	4.803496	12.04755	2.760235	3.084253	1.861441	0	0.824452	1.026376	0.910133	8.066772
AC008764.	1098.317	721.2815	901.2186	1275.212	796.2817	798.179	1170.01	782.4928	288.3201	705.1533	540.9349	916.3979	941.7253	835.7868	889.0073	803.0161	859.0769	868.2673	786.0621
AC008764.	0	0	1.998267	0	2.21497	1.141887	0.84968	0.926027	0	3.842797	0	4.600391	1.028084	0	0.978006	0	2.052753	0	0

AC008764.	199.8688	283.1105	237.7938	107.0472	170.5527	236.3706	150.3934	286.1424	340.848	305.5024	299.984	225.4192	180.9428	194.5205	245.4795	173.1349	183.7213	249.3766	198.0841
AC008764.	304.6077	394.1538	550.5226	188.112	331.138	339.1404	289.741	461.1615	494.93	556.2449	712.0101	479.3608	495.5367	497.9354	410.7624	199.5173	258.6468	452.3363	387.205
AC008764.	38.4363	53.02069	46.95928	30.13949	32.11706	26.2634	29.73881	51.85751	115.5615	39.38867	78.30907	46.00391	26.73019	25.12945	25.42815	21.43575	73.89909	56.42827	65.43048
AC008770.	722.6025	682.2663	703.3901	870.9273	695.5005	666.862	665.2996	912.1366	931.4956	845.4153	712.0101	715.8209	576.7553	780.8743	726.6583	733.7621	822.1274	688.971	663.2679
AC008770.	13.45271	12.00469	12.98874	22.86444	21.04221	4.567548	23.79105	21.29862	9.338302	3.842797	16.86657	25.76219	28.78636	29.78305	19.56012	29.68027	12.31652	30.0344	18.82247
AC008770.	109.5435	109.0426	124.8917	86.2613	111.856	78.7902	85.8177	137.978	28.01491	105.6769	55.41872	115.9299	100.7523	71.66546	114.4267	146.7524	92.37386	120.1376	94.11233
AC008771.	303.6468	266.1039	366.6821	455.2102	289.0536	256.9246	411.2452	332.4437	311.6658	289.1705	274.6841	317.427	294.0321	289.454	341.324	319.0629	370.5218	302.1643	281.4407
AC008780.	12.4918	13.00508	20.98181	10.39293	1.107485	15.98642	24.64073	61.11778	4.669151	0.960699	10.84279	12.8811	12.33701	9.307203	7.824047	10.71787	3.079129	5.4608	2.688924
AC008780.	13.45271	37.01445	13.98787	4.157171	9.967364	11.41887	10.19616	104.6411	31.51677	4.803496	24.0951	9.200782	15.42127	18.61441	23.47214	1.648904	6.158258	13.652	8.066772
AC008781.	21.13997	23.00898	13.98787	29.1002	16.61227	18.27019	21.24201	19.44657	59.53167	16.33189	22.89034	20.24172	15.42127	26.99089	31.29619	12.36678	26.68578	30.0344	18.82247
AC008781.	6.726353	14.00547	3.996535	18.70727	14.3973	0	10.19616	5.556162	5.836439	6.724895	0	3.680313	6.168506	5.584322	10.75806	11.54233	4.105505	0.910133	7.170464
AC008781.	16.33543	4.001562	10.99047	7.275049	8.859879	17.12831	12.7452	1.852054	50.19337	35.54587	15.66181	23.92203	21.58977	18.61441	9.780058	16.48904	10.26376	24.5736	10.7557
AC008794.	0	0	0	0	0	1.141887	0	0	0	0	1.204755	0	0	0	0	0	0	0	0
AC008794.	0	0	0	0	1.107485	1.141887	0	0	1.167288	0	0	0	0	0	0.978006	0	3.079129	0	1.792616
AC008799.	0	0	0	1.039293	0	0	0	5.556162	11.67288	0	0	0.920078	0	0	0	1.648904	1.026376	6.370934	0
AC008799.	0	0	8.992203	0	0	0	0	0	1.167288	0	0	0	6.168506	0	0.978006	0	0	5.4608	0
AC008802.	545.7955	482.1882	415.6396	320.1022	966.8343	899.807	248.1066	650.997	1226.819	869.4328	1138.493	417.7155	447.2167	661.7421	624.9457	479.0065	541.9267	501.4835	270.685
AC008805.	0	0	0	4.157171	0	2.283774	0	0.926027	0	0	0	0	4.112337	0	0	0.824452	0	0	4.48154
AC008805.	3.84363	0	0	0	4.42994	17.12831	0.84968	3.704108	2.334575	0	3.614265	0	0	1.861441	4.890029	0	0	0	8.963079
AC008806.	10.56998	34.01327	36.96795	38.45383	27.68712	25.12151	35.68657	25.00273	28.01491	42.27077	32.52838	30.36258	18.50552	16.75297	13.69208	33.80253	14.36927	36.40534	39.43755
AC008808.	0	0	0	1.039293	0	0	0.84968	0	0	1.921399	6.023774	3.680313	2.056169	0	1.956012	0	2.052753	1.820267	0.896308
AC008808.	170.0806	155.0605	135.8822	68.59332	77.52394	86.78341	127.452	74.08216	170.424	145.0656	63.85201	134.3314	112.0612	92.14131	76.28445	142.6302	125.2179	150.172	145.2019
AC008813.	0.960908	4.001562	9.991337	6.235757	2.21497	13.70264	4.248401	3.704108	2.334575	14.41049	10.84279	1.840156	4.112337	5.584322	6.846041	7.420066	16.42202	4.550667	3.585232
AC008814.	0	1.00039	0.999134	1.039293	1.107485	0	0.84968	0.926027	0	2.882098	6.023774	5.520469	0	0	3.912023	0	0	4.550667	0
AC008817.	0	0	0	0	0	0	0	0	1.167288	0	0	0	0	0	0	0.824452	4.105505	0	0
AC008825.	0	0	0	3.117878	0	0	0.84968	0	0	0	0	0	0	0.93072	0	0	0	0	0
AC008833.	9.609075	0	0	0	0	0	0	0.926027	0	0	0	0	0	0	0	0.824452	0	0	0
AC008840.	30.74904	47.01835	50.95582	53.00393	15.50479	44.53359	34.83689	64.82189	37.35321	18.25329	18.07132	23.92203	45.23571	31.64449	29.34017	42.8715	23.60665	40.04587	17.92616
AC008850.	0	0	1.998267	6.235757	0	0	2.549041	2.778081	1.167288	0	1.204755	1.840156	4.112337	0	0	0.824452	2.052753	0.910133	1.792616
AC008852.	1.921815	1.00039	0.999134	1.039293	0	1.141887	9.346483	0	0	2.882098	0	0	1.028084	2.792161	5.868035	0	1.026376	0	0
AC008868.	0.960908	0	0	1.039293	0	0	0	0	1.167288	0	0	0	0	3.722881	1.956012	0	0	0	0
AC008870.	202.7515	246.096	417.6379	441.6994	338.8904	180.4181	455.4286	324.1095	250.9669	295.8954	227.6987	448.9982	355.7172	266.186	288.5117	366.0566	331.5195	357.6824	296.6779
AC008870.	0.960908	4.001562	0.999134	3.117878	2.21497	3.425661	5.947762	7.408216	7.003726	7.685594	16.86657	3.680313	0	6.515042	0	2.473355	9.237386	13.652	13.44462
AC008870.	36.51449	37.01445	66.94196	32.21808	39.86946	41.10793	62.02666	42.59724	4.669151	62.44545	43.37118	44.16376	35.98295	24.19873	55.74633	28.03136	32.84404	50.05734	47.50432
AC008871.	3.84363	3.001171	0	1.039293	6.64491	4.567548	2.549041	5.556162	23.34575	0	0	0.920078	2.056169	0.93072	0.978006	0.824452	2.052753	2.7304	0
AC008873.	0	0	1.998267	0	0	0	0	0	0	0	0	0	0	0	0	0	0	0	0
AC008875.	81.67714	86.03358	83.92723	53.00393	137.3281	95.91851	45.03305	103.715	169.2567	117.2053	172.2799	70.84602	66.82548	52.12034	81.17448	41.22259	67.74083	84.64241	74.39356
AC008878.	0	3.001171	0	1.039293	1.107485	0	0.84968	0	0	0.960699	0	1.840156	3.084253	3.722881	1.956012	1.648904	8.21101	0.910133	0
AC008883.	0	0	0.999134	1.039293	4.42994	1.141887	3.398721	0.926027	7.003726	0	0	0	1.028084	0	3.912023	0	0	5.4608	3.585232
AC008883.	0.960908	6.002343	4.995668	0	4.42994	1.141887	0.84968	3.704108	0	5.764196	1.204755	0.920078	1.028084	7.445762	5.868035	4.122259	1.026376	0.910133	0.896308
AC008894.	4.804538	0	0	6.235757	6.64491	0	0	0	0	0	0	1.840156	0	0	0	4.946711	0	0.910133	0
AC008894.	20.17906	9.003514	20.98181	36.37525	0	2.283774	11.89552	12.96438	17.50932	16.33189	20.48083	5.520469	5.140422	3.722881	6.846041	10.71787	58.50345	30.0344	25.99293
AC008894.	112.4262	117.0457	182.8415	82.10413	120.7159	74.22266	105.3603	102.789	211.2791	107.5983	161.4372	116.8499	105.8927	123.7858	111.4927	105.5298	75.95184	193.8584	100.3865
AC008895.	116.2698	136.0531	116.8986	187.0727	108.5335	59.37812	190.3284	150.0164	14.00745	71.09174	60.23774	121.4503	151.1284	100.5178	74.32844	151.6991	121.1124	77.36134	84.25295
AC008897.	131.6443	114.0445	163.8579	134.0688	208.2072	105.0536	164.838	234.2848	14.00745	144.1049	101.1994	172.0546	181.9709	161.9453	126.1628	98.93422	112.9014	107.3957	98.59387
AC008897.	5.765445	3.001171	15.98614	3.117878	3.322455	3.425661	10.19616	0.926027	8.171014	8.646293	13.2523	2.760235	0	2.792161	7.824047	5.771163	1.026376	4.550667	8.066772
AC008897.	164.3152	426.1663	425.6309	182.9155	668.9209	372.2552	158.8902	262.0657	349.019	509.1706	685.5055	144.4523	174.7743	188.9362	177.9971	153.348	268.9106	174.7456	275.1665
AC008906.	255.6014	233.091	179.8441	279.5698	219.282	182.7019	255.7538	327.8136	156.4166	232.4892	150.5944	237.3802	266.2739	109.825	108.5586	173.1349	136.508	99.20454	126.3794
AC008906.	12.4918	4.001562	15.98614	17.66798	12.18233	23.97963	11.89552	9.26027	29.18219	14.41049	8.433284	11.96102	13.3651	26.06017	14.67009	27.20691	28.73854	15.47227	7.170464
AC008915.	18.25724	11.00429	18.98354	5.196464	12.18233	14.84453	21.24201	36.11505	38.5205	23.05678	13.2523	32.20274	12.33701	18.61441	4.890029	20.6113	31.81766	25.48374	15.23724
AC008915.	47.08447	33.01288	30.97314	28.0609	35.43952	27.40529	32.28785	29.63287	60.69896	25.93888	67.46627	16.56141	21.58977	17.68369	27.38416	33.80253	12.31652	29.12427	43.02278
AC008937.	11.53089	7.002733	3.996535	2.078586	24.36467	0	5.098081	16.66849	2.334575	4.803496	1.204755	11.96102	7.196591	4.653602	8.802052	12.36678	7.184634	6.370934	7.170464
AC008937.	1.921815	0	4.995668	2.078586	3.322455	0	5.947762	0.926027	0	1.921399	2.40951	0.920078	1.028084	2.792161	6.846041	4.946711	4.105505	8.191201	8.963079
AC008937.	75.91169	59.02304	84.92636	90.41847	97.45867	84.49964	98.56291	127.7917	61.86625	45.15286	39.75691	89.24759	91.49951	77.24979	75.30645	70.07841	69.79359	41.86614	97.69757
AC008938.	0	0	0	0	0	0	1.69936	0	0	0	0	5.520469	1.028084	0.93072	0	0	0	0	0
AC008940.	6.726353	7.002733	3.996535	8.314342	0	1.141887	3.398721	16.66849	10.50559	1.921399	7.228529	1.840156	7.196591	3.722881	8.802052	9.06897	1.026376	3.640534	5.377848
AC008945.	100.8953	132.0515	181.8423	77.94696	105.2111	52.5268	158.0405	133.3479	214.7809	139.3014	119.2707	67.16571	119.2578	85.62627	116.3827	14.01568	47.21331	65.52961	51.98586
AC008946.	69.18534	13.00508	76.93329	40.53242	62.01916	33.11472	50.98081	74.08216	53.69524	93.18783	102.4042	74.52634	91.49951	37.22881	49.8783	48.64266	74.92547	49.1472	41.23017
AC008953.	0	1.00039	0	2.078586	0	1.141887	2.549041	0	0	0.960699	3.614265	0.920078	0	0	4.890029	1.648904	1.026376	0.910133	0
AC008957.	92.24712	167.0652	40.96448	346.0845	138.4356	26.2634	190.3284	315.7752	47.8588	36.50657	18.07132	220.8188	150.1003	123.7858	147.6789	102.232	54.39794	533.3382	458.0134
AC008957.	16.33543	5.001952	2.997401	27.02161	36.547	5.709435	5.947762	18.52054	19.84389	1.921399	12.04755	10.12086	4.112337	23.26801	17.6041	7.420066	11.29014	8.191201	9.859387
AC008957.	943.6112	815.3182	554.5192	1970.499	887.0954	375.6808	1452.104	1224.208	131.9035	382.3583	168.6657	641.2945	564.4183	630.0976	765.7786	878.8657	599.4037	1065.766	904.3747

AC008958.	0	6.002343	0	4.157171	4.42994	0	2.549041	3.704108	0	0.960699	0	7.360626	2.056169	0	0	0	0	0	0.896308
AC008966.	614.9808	661.2581	700.3927	685.9332	552.635	582.3624	595.6258	820.46	685.1979	577.3803	587.9204	601.7312	607.5979	893.4915	933.9956	775.8092	717.437	452.3363	570.0519
AC008966.	104.7389	97.03787	80.92983	65.47544	80.8464	76.50643	150.3934	127.7917	96.88488	143.1442	116.8612	68.08579	113.0893	53.05106	88.02052	54.41382	70.81996	53.69787	52.88217
AC008967.	5.765445	0	0	0	0	0	0	0	0	0	0	0	1.840156	1.028084	0	0	0	0	2.688924
AC008969.	4450.924	4828.885	4545.059	2392.452	3318.025	3773.937	4136.243	3633.73	2469.981	3967.688	3378.133	4926.099	5063.316	3233.322	3599.061	3954.895	4210.195	4259.424	4724.439
AC008972.	40.35812	24.00937	29.97401	28.0609	37.65449	18.27019	31.43817	21.29862	45.52422	13.44979	33.73314	31.28266	25.70211	29.78305	31.29619	65.13169	56.45069	42.77627	47.50432
AC008972.	28.82723	6.002343	14.987	8.314342	0	10.27698	15.29424	0	15.17474	4.803496	21.68559	6.440548	10.28084	32.57521	21.51613	36.27588	24.63303	38.2256	31.37078
AC008982.	128.7616	104.0406	91.9203	438.5815	91.92125	47.95925	215.8188	76.86024	89.88116	57.64196	26.50461	78.20665	77.10633	86.55699	126.1628	240.7399	201.1697	200.2294	212.425
AC008985.	1.921815	15.00586	10.99047	9.353635	16.61227	12.56076	9.346483	20.37259	5.836439	35.54587	13.2523	10.12086	17.47743	10.23792	26.40616	13.19123	27.71216	8.191201	13.44462
AC008991.	0	0	0	0	0	0	0	0	0	0	0	0.920078	0	0	0	0.824452	0	0	0
AC008992.	37.47539	21.0082	36.96795	21.82515	17.71976	18.27019	22.09169	37.04108	112.0596	27.86028	25.29985	16.56141	20.56169	29.78305	15.64809	29.68027	13.34289	17.29253	19.71877
AC008993.	0.960908	2.000781	3.996535	0	1.107485	0	0	0.926027	0	5.764196	0	17.48149	12.33701	3.722881	3.912023	9.893422	0	0	0
AC008993.	0	0	0.999134	4.157171	1.107485	0	0.84968	0	1.167288	0	0	3.680313	2.056169	0.93072	0.978006	0.824452	1.026376	0	0.896308
AC008993.	0	0	1.998267	0	0	0	0	0	0	0	0	0	0	0	0	0	0	0	0
AC009005.	20.17906	10.0039	14.987	11.43222	15.50479	10.27698	15.29424	26.85478	12.84017	14.41049	33.73314	15.64133	10.28084	10.23792	12.71408	36.27588	9.237386	42.77627	17.02985
AC009014.	0	0	0	0	0	0	0.84968	0	0	3.842797	4.81902	0	0	0.93072	0	0.824452	0	3.640534	2.688924
AC009019.	62.45899	67.02616	41.96361	68.59332	64.23413	57.09435	57.77826	37.96711	67.70269	67.24895	40.96167	49.68422	45.23571	66.08114	64.54838	42.04704	62.60895	76.45121	38.54124
AC009021.	0.960908	0	1.998267	0	1.107485	2.283774	0	0	4.669151	0	0	0.920078	0	0	0.978006	0	0	0.910133	0
AC009021.	1.921815	6.002343	0	0	0	199.8302	4.248401	0	1.167288	0	0	0	4.112337	0	4.890029	139.3324	129.3234	0	0
AC009027.	0	0	0.999134	0	0	1.141887	0.84968	0	3.501863	0	0	0.920078	0	0.93072	0	0	0	0.910133	0.896308
AC009032.	0.960908	5.001952	6.993936	6.235757	1.107485	5.709435	4.248401	12.03835	3.501863	9.606993	36.14265	3.680313	1.028084	2.792161	8.802052	8.244518	5.131881	0	1.792616
AC009034.	0	1.00039	0	0	1.107485	0	0	0	0	0	0	0	0	0	0	0	0	0	0
AC009035.	0	0	0	0	0	0	0	0	0	0	0	0	0	0.93072	0	0.824452	1.026376	0	0
AC009041.	3.84363	0	0	1.039293	0	1.141887	0.84968	0.926027	0	0	3.614265	0.920078	0	2.792161	0	0	0	0	5.377848
AC009041.	5157.191	4613.801	4668.952	1132.829	8559.751	5860.164	2695.186	4713.478	17023.72	9409.088	20583.24	4659.276	4930.693	4362.286	4038.186	1755.258	2506.411	3224.603	3326.199
AC009041.	15.37452	12.00469	20.98181	35.33595	25.47215	21.69585	22.94137	33.33697	14.00745	10.56769	24.0951	38.64329	49.34805	31.64449	26.40616	35.45143	35.92317	29.12427	37.64493
AC009044.	0	0	0	1.039293	0	0	0	0	0	0	7.228529	0	1.028084	0	0	0	0	0	0
AC009053.	960.9075	976.3811	1397.788	927.0492	1475.17	1161.299	968.6355	1696.482	887.1387	913.625	993.9228	1072.811	1086.685	1065.675	1266.518	1282.023	1105.407	1278.737	1316.676
AC009053.	10.56998	41.01601	33.97054	42.611	17.71976	46.81737	20.39233	59.26573	24.51304	7.685594	45.78069	19.32164	21.58977	32.57521	23.47214	45.34485	26.68578	31.85467	64.53417
AC009053.	2.882723	10.0039	14.987	5.196464	15.50479	6.851322	9.346483	20.37259	7.003726	16.33189	0	6.440548	4.112337	17.68369	26.40616	23.9091	17.4484	9.101334	27.78555
AC009053.	3.84363	0	0	3.117878	0	1.141887	10.19616	3.704108	0	8.646293	3.614265	0.920078	0	1.861441	1.956012	7.420066	2.052753	0.910133	3.585232
AC009054.	6.726353	10.0039	1.998267	10.39293	7.752394	1.141887	5.947762	0	0	2.882098	0	9.200782	1.028084	1.861441	1.956012	1.648904	5.131881	1.820267	0.896308
AC009054.	7.68726	18.00703	18.98354	12.47151	8.859879	7.993209	6.797442	15.74246	31.51677	13.44979	3.614265	7.360626	9.252759	26.06017	31.29619	5.771163	6.158258	6.370934	11.652
AC009055.	0	0	0	1.039293	2.21497	0	0.84968	0	0	0.960699	0	0.920078	0	0	0	4.122259	2.052753	0	0
AC009055.	7.68726	2.000781	0	0	0	0	1.69936	0	2.334575	0	0	0.920078	0	0	0.978006	0.824452	0	1.820267	8.066772
AC009060.	0	5.001952	2.997401	6.235757	3.322455	9.135096	0.84968	21.29862	10.50559	0.960699	6.023774	3.680313	1.028084	3.722881	4.890029	4.946711	5.131881	16.3824	1.792616
AC009060.	7.68726	4.001562	5.994802	0	6.64491	0	3.398721	4.630135	1.167288	5.764196	0	2.760235	4.112337	1.861441	7.824047	6.595615	9.237386	7.281067	17.92616
AC009061.	625.5508	637.2487	688.4031	393.892	426.3817	503.5722	476.6706	750.0819	489.0936	617.7296	665.0247	716.7409	798.8216	697.1095	699.2742	427.066	390.023	599.7779	549.4368
AC009063.	0	1.00039	3.996535	0	0	0	1.69936	0	0	0	0	0	0	2.792161	0	0	0	5.4608	0
AC009063.	147.9798	72.02811	59.94802	19.74656	86.38382	45.67548	48.43177	32.41095	22.17847	48.03496	16.86657	54.28462	57.57272	25.12945	34.2302	37.10033	39.0023	116.4971	69.01571
AC009063.	16.33543	41.01601	26.97661	8.314342	32.11706	34.25661	61.17698	11.11232	37.35321	10.56769	27.70936	38.64329	48.31997	47.46674	23.47214	0.824452	5.131881	7.281067	3.585232
AC009065.	117.2307	106.0414	145.8735	102.89	217.067	165.5736	110.4584	199.0958	89.88116	267.0744	262.6366	230.9396	171.6901	159.1532	220.0513	79.97183	92.37386	120.1376	164.0244
AC009065.	31.70995	69.02694	10.99047	23.90373	23.25718	23.97963	6.797442	3.704108	0	21.13538	4.81902	9.200782	13.3651	17.68369	9.780058	15.66458	20.52753	23.66347	24.20031
AC009065.	121.0743	112.0437	111.903	51.96464	151.7254	139.3102	20.39233	75.00819	431.8965	285.3277	389.1358	80.96688	126.4544	125.6472	130.0748	136.0346	148.8246	102.8451	70.80833
AC009065.	387.2457	431.1683	423.6327	447.9352	417.5218	481.8763	461.3764	527.8354	102.7213	432.3147	395.1596	610.0119	485.2558	428.1313	507.585	429.5394	341.7833	316.7264	268.8924
AC009065.	0	0	0	0	1.107485	0	0	0	0	0	4.81902	0.920078	0	0	1.956012	0	1.026376	0	0
AC009065.	173.9243	213.0832	191.8337	154.8546	251.3991	210.1072	203.0736	172.241	306.9967	285.3277	307.2125	151.8129	212.8135	141.4695	170.173	150.0502	141.6399	179.2963	170.2985
AC009075.	9.609075	8.003124	0	2.078586	0	1.141887	16.9936	15.74246	0	0.960699	1.204755	5.520469	10.28084	0.93072	15.64809	16.48904	7.184634	3.640534	3.585232
AC009075.	0	2.000781	0	1.039293	0	0	3.398721	0	9.338302	0	1.204755	0	0	0	0	4.122259	1.026376	1.820267	0.896308
AC009078.	0.960908	1.00039	0	2.078586	0	0	1.69936	3.704108	0	0	1.204755	0	0	0	0.978006	0	0	0	0.896308
AC009078.	22.10087	34.01327	19.98267	78.98625	32.11706	106.1955	28.03945	5.556162	7.003726	4.803496	22.89034	31.28266	38.03912	47.46674	33.2522	97.28532	70.81996	22.75334	27.78555
AC009078.	126.8398	136.0531	110.9038	147.5796	310.0958	142.7359	117.2559	152.7945	144.7437	75.89524	195.1703	161.0137	213.8415	149.846	187.7771	208.5863	182.695	90.10321	153.2687
AC009084.	1.921815	1.00039	2.997401	17.66798	6.64491	3.425661	8.496802	4.630135	0	2.882098	0	7.360626	0	8.376483	6.846041	3.297807	4.105505	11.83173	2.688924
AC009084.	587.1145	1189.464	1518.683	585.1218	1163.967	946.6243	1091.839	1440.898	1126.433	1339.215	1454.139	815.1893	774.1475	877.6692	902.6994	535.8937	477.265	673.4987	726.9057
AC009086.	15.37452	25.00976	30.97314	5.196464	12.18233	9.135096	9.346483	29.63287	25.68033	29.78168	12.04755	14.72125	21.58977	13.9608	28.36217	8.244518	19.50115	16.3824	25.99293
AC009087.	47.08447	52.0203	141.877	27.02161	76.41646	68.51322	54.37954	106.4931	47.8588	60.52405	65.05676	57.04485	70.93782	62.35826	88.99853	35.45143	47.21331	35.4952	54.67478
AC009088.	54.77173	44.01718	77.93243	92.49706	90.81376	58.23624	69.67378	19.44657	140.0745	109.5197	131.3183	61.64524	65.7974	86.55699	59.65836	49.46711	42.08143	60.06881	73.49725
AC009088.	2.882723	2.000781	0.999134	0	0	1.141887	0	0	1.167288	0	1.204755	1.840156	1.028084	0	0	0	0	0.910133	0.896308
AC009090.	186.4161	178.0695	239.7921	173.5619	274.6563	200.9721	259.1525	213.9122	264.9743	251.7032	297.5745	183.0956	177.8586	147.9845	184.8431	129.4389	143.6927	170.1949	155.0613
AC009090.	2.882723	0	0	0	0	0	1.69936	0	0	0	0	0	1.028084	0	0	0	0	0.910133	0.896308
AC009090.	135.488	163.0636	182.8415	126.7937	166.1227	146.1615	170.7857	159.2767	180.9296	226.725	169.8704	145.3724	188.1394	127.5087	134.9648	136.859	148.8246	159.2733	104.868
AC009090.	651.4953	637.2487	561.5131	552.9038	611.3317	413.3631	536.1482	566.7285	170.424	674.4109	625.2678	452.6785	659.0021	369.496	513.4531	447.6773	503.9507	536.0686	553.9183

AC009090.	130.6834	107.0418	92.91943	81.06484	90.81376	110.763	125.7527	125.0136	108.5578	108.559	118.066	104.8889	104.8646	93.07203	119.3167	91.51415	66.71446	88.28294	111.1422
AC009093.	65.34171	147.0574	82.92809	34.29666	44.2994	49.10114	118.1056	134.2739	107.3905	102.7948	132.523	106.7291	94.58376	60.49682	61.61437	55.23827	26.68578	53.69787	19.71877
AC009093.	7.68726	18.00703	17.98441	13.51081	12.18233	21.69585	11.04584	16.66849	7.003726	5.764196	24.0951	23.92203	8.224675	13.9608	9.780058	9.06897	11.29014	8.191201	24.20031
AC009093.	72.06806	107.0418	110.9038	28.0609	118.5009	129.0332	117.2559	223.1725	133.0708	130.6551	156.6181	103.9688	123.3701	130.3008	102.6906	91.51415	80.05735	69.17014	62.74156
AC009093.	54.77173	58.02265	74.93502	149.6582	60.91167	83.35775	124.0533	102.789	17.50932	31.70308	72.28529	108.5692	86.35909	40.02097	87.04252	112.9499	111.875	32.7648	35.85232
AC009093.	17.29634	15.00586	41.96361	11.43222	16.61227	9.135096	9.346483	12.96438	11.67288	19.21399	14.45706	23.92203	20.56169	14.89152	23.47214	23.08465	12.31652	29.12427	15.23724
AC009093.	0	0	19.98267	11.43222	15.50479	4.567548	19.54265	0.926027	30.34948	7.685594	7.228529	18.40156	13.3651	18.61441	15.64809	3.297807	28.73854	1.820267	11.652
AC009093.	47.08447	35.01367	39.96535	27.02161	43.19191	47.95925	45.03305	69.45203	121.3979	89.34503	84.33284	33.12282	30.84253	60.49682	30.31818	35.45143	44.13418	19.1128	33.16339
AC009093.	18.25724	33.01288	12.98874	11.43222	49.83682	28.54718	21.24201	62.04381	58.36439	19.21399	33.73314	46.00391	29.81445	20.47585	34.2302	13.19123	23.60665	23.66347	13.44462
AC009093.	9.609075	25.00976	26.97661	11.43222	17.71976	11.41887	13.59488	18.52054	17.50932	35.54587	16.86657	16.56141	16.44935	11.16864	14.67009	3.297807	9.237386	7.281067	7.170464
AC009093.	5.765445	9.003514	21.98094	6.235757	12.18233	12.56076	18.69297	7.408216	14.00745	15.37119	20.48083	11.96102	20.56169	7.445762	17.6041	11.54233	6.158258	6.370934	4.48154
AC009094.	0	6.002343	0	0	0	0	0	0	0	0	0	0	0	0	0	0	0	0	0.896308
AC009095.	51.88901	73.0285	66.94196	118.4794	93.02873	46.81737	114.7068	76.86024	25.68033	111.4411	86.74235	59.80509	52.4323	64.2197	81.17448	59.36053	69.79359	106.4856	100.3865
AC009097.	5.765445	2.000781	3.996535	11.43222	1.107485	17.12831	11.04584	7.408216	0	5.764196	1.204755	0.920078	8.224675	0.93072	7.824047	9.893422	15.39564	0.910133	8.066772
AC009097.	10.56998	32.01249	25.97748	9.353635	15.50479	35.3985	18.69297	34.263	60.69896	63.40615	55.41872	26.68227	42.15146	23.26801	44.01026	9.893422	7.184634	27.304	15.23724
AC009097.	3.84363	5.001952	0	3.117878	1.107485	0	0.84968	0	1.167288	7.685594	14.45706	11.96102	0	8.376483	0.978006	5.771163	20.52753	6.370934	0.896308
AC009097.	0	2.000781	0	1.039293	0	0	0.84968	0	0	4.803496	0	0	1.028084	0.93072	0.978006	0	0	0	5.377848
AC009101.	3.84363	1.00039	7.993069	3.117878	26.57964	0	1.69936	4.630135	0	0	7.228529	0.920078	0	6.515042	2.934017	3.297807	4.105505	7.281067	14.34093
AC009101.	26.90541	16.00625	96.91597	1.039293	169.4452	10.27698	16.9936	98.15887	16.34203	6.724895	8.433284	19.32164	2.056169	103.31	132.0308	286.0848	227.8555	193.8584	181.9505
AC009102.	2.882723	10.0039	13.98787	7.275049	0	10.27698	5.098081	10.1863	25.68033	7.685594	1.204755	8.280704	11.30893	0.93072	0	5.771163	0	1.820267	0.896308
AC009102.	42.27993	68.02655	42.96275	86.2613	18.82724	39.96605	62.87634	29.63287	11.67288	41.31007	56.62348	60.72516	37.01104	41.88241	40.09824	14.67009	96.47937	55.51814	95.90495
AC009102.	0.960908	9.003514	0	0	0	3.425661	4.248401	0	9.338302	3.842797	0	5.520469	1.028084	0	0.978006	0.824452	1.026376	0.910133	0
AC009107.	0	0	0	0	0	0	0	0.926027	0	0	0	0.920078	0	0.93072	0	0.824452	0	0.910133	0.896308
AC009107.	111.4653	91.03553	130.8865	96.65423	86.38382	101.6279	132.5501	148.1643	123.7325	132.5765	148.1848	119.6102	102.8084	106.1021	111.4927	113.7744	108.7959	115.5869	99.49018
AC009108.	116.2698	78.03045	98.91423	80.02554	58.6967	52.5268	96.86355	101.863	51.36066	48.03496	78.30907	68.08579	82.24675	73.5269	119.3167	90.6897	86.21561	59.15867	79.77141
AC009108.	0	0	0.999134	0	0	0	0.84968	0	0	0	0	0.920078	1.028084	0.93072	0	0	0	0	0
AC009108.	186.4161	166.0648	82.92809	172.5226	148.403	206.6815	115.5565	84.26846	345.5172	142.1835	131.3183	34.04289	44.20763	102.3792	133.0088	126.9656	150.8773	24.5736	39.43755
AC009108.	0	0	4.995668	5.196464	0	0	4.248401	0	1.167288	12.48909	4.81902	4.600391	15.42127	0	3.912023	2.473355	0	1.820267	3.585232
AC009110.	0	0	0	2.078586	0	0	0.84968	0.926027	0	0	0	0	1.028084	0	0.978006	0	0	0	0
AC009113.	1169.424	1064.415	1552.654	880.281	1109.7	1052.82	1018.767	1123.271	1126.433	1344.018	1390.287	1173.1	1245.01	1218.313	1364.318	798.8938	868.3143	926.5158	1005.658
AC009117.	0.960908	1.00039	0	0	1.107485	0	0	0	0	0	0	0	0	0	0	0	0	0	0
AC009118.	65.34171	80.03124	109.9047	116.4008	71.98652	43.39171	73.0725	95.38078	21.01118	86.46293	80.71858	85.56728	79.1625	45.60529	59.65836	56.06272	96.47937	50.96747	95.00864
AC009118.	0.960908	5.001952	3.996535	3.117878	1.107485	2.283774	3.398721	3.704108	7.003726	3.842797	0	1.840156	0	2.792161	1.956012	0	2.052753	9.101334	0.896308
AC009118.	254.6405	277.1082	327.7158	262.9411	318.9557	300.3163	214.9691	258.3615	550.9598	362.1836	413.2309	209.7778	226.1786	273.6318	236.6774	181.3794	148.8246	164.7341	136.2388
AC009119.	80.71623	113.0441	87.92376	114.3222	80.8464	124.4657	79.86994	142.6082	21.01118	143.1442	72.28529	46.00391	32.8987	62.35826	61.61437	73.37621	70.81996	263.9387	216.9065
AC009119.	53.81082	43.01679	85.9255	55.08252	59.80419	29.68906	79.02026	59.26573	88.71387	107.5983	153.0039	75.44642	83.27483	67.94258	53.79032	39.57369	26.68578	93.74374	56.4674
AC009119.	20.17906	18.00703	32.97141	23.90373	18.82724	5.709435	12.7452	5.556162	7.003726	23.05678	45.78069	17.48149	29.81445	23.26801	24.45015	11.54233	12.31652	27.304	20.61508
AC009120.	442.0175	545.2128	655.4317	660.9902	589.182	280.9042	726.4766	703.7805	158.7511	390.0439	277.0936	612.7721	633.3	499.7968	606.3636	554.8561	522.4255	493.2923	482.2137
AC009120.	2272.546	2312.903	2803.569	2266.698	2558.29	2326.024	2153.09	2650.289	2824.836	2837.906	2799.85	1997.49	2324.499	2297.018	2596.605	2091.634	2318.584	2332.672	2177.132
AC009120.	0.960908	0	0	0	1.107485	1.141887	0.84968	0.926027	0	0	0	1.840156	0	0	0	0	0	0	0
AC009120.	291.155	306.1195	497.5686	404.2849	511.658	178.1344	394.2516	448.1971	67.70269	319.9129	157.8229	466.4797	415.3461	352.743	369.6862	407.2792	420.8143	346.7608	510.8955
AC009120.	0.960908	1.00039	0.999134	0	2.21497	3.425661	0.84968	0	0	3.842797	0	3.680313	6.168506	6.515042	3.912023	1.648904	1.026376	1.820267	0.896308
AC009120.	26.90541	42.0164	54.95235	23.90373	26.57964	43.39171	33.98721	39.81916	57.1971	54.75986	22.89034	35.88305	22.61786	30.71377	37.16422	29.68027	28.73854	31.85467	47.50432
AC009120.	0.960908	0	0	1.039293	0	0	0	7.408216	0	0	0	0	1.028084	0	0	0	0	0	2.688924
AC009121.	0.960908	0	7.993069	15.58939	11.07485	1.141887	7.647122	14.81643	0	1.921399	0	16.56141	5.140422	7.445762	4.890029	27.20691	7.184634	133.7896	97.69757
AC009121.	2.882723	15.00586	18.98354	6.235757	6.64491	6.851322	23.79105	17.59451	10.50559	10.56769	0	14.72125	25.70211	15.82225	20.53812	8.244518	18.47477	13.652	8.963079
AC009122.	4.804538	4.001562	3.996535	2.078586	1.107485	2.283774	4.248401	1.852054	3.501863	5.764196	0	7.360626	0	0	2.934017	4.946711	3.079129	1.820267	0.896308
AC009123.	0	4.001562	15.98614	12.47151	8.859879	25.12151	2.549041	3.704108	1.167288	22.09608	31.32363	17.48149	11.30893	12.09936	11.73607	20.6113	36.94955	2.7304	18.82247
AC009126.	238.3051	253.0988	283.754	231.7623	234.7868	218.1004	305.0352	169.4629	282.4836	284.367	163.8467	253.9416	257.0211	205.6892	210.2713	302.5738	297.6491	235.7246	340.597
AC009127.	0	0	0	1.039293	0	0	0	0	0	0	0	0	1.028084	0	0	0	0	0	0
AC009127.	3.84363	17.00664	2.997401	7.275049	9.967364	6.851322	1.69936	6.482189	17.50932	25.93888	4.81902	23.92203	6.168506	4.653602	12.71408	0.824452	2.052753	10.9216	4.48154
AC009127.	34.59267	18.00703	16.98527	42.611	17.71976	29.68906	10.19616	7.408216	19.84389	40.34937	16.86657	14.72125	7.196591	26.06017	11.73607	17.31349	17.4484	11.83173	14.34093
AC009132.	9.609075	1.00039	14.987	15.58939	9.967364	1.141887	2.549041	10.1863	2.334575	1.921399	9.638039	1.840156	0	3.722881	2.934017	9.06897	3.079129	1.820267	2.688924
AC009133.	752.3906	570.2225	605.475	486.389	662.276	799.3209	536.9979	550.0601	854.4546	855.983	1236.079	751.7039	852.2819	543.5407	633.7478	639.7746	732.8326	696.2521	802.1956
AC009133.	0	0	0	0	0	0	0	0.926027	0	0	0	0	0	0.93072	0	0	0	0	0
AC009133.	13.45271	1.00039	4.995668	19.74656	0	19.41208	0	7.408216	8.171014	1.921399	8.433284	14.72125	5.140422	4.653602	0	14.84013	1.026376	14.56213	2.688924
AC009134.	0.960908	1.00039	0	0	2.21497	1.141887	0.84968	9.26027	0	0	1.204755	1.840156	3.084253	0	2.934017	0.824452	7.184634	0.910133	4.48154
AC009135.	0	1.00039	0.999134	10.39293	1.107485	2.283774	11.89552	0	18.6766	0	1.204755	0.920078	3.084253	7.445762	0	5.771163	3.079129	0.910133	0
AC009135.	0	4.001562	4.995668	5.196464	5.537425	0	0	9.26027	15.17474	5.764196	7.228529	2.760235	5.140422	0	0.978006	1.648904	0	0	0
AC009137.	0	0	0	0	0	0	0	0	0	0	0	0	1.028084	5.584322	0	0	0	0	0
AC009137.	6.726353	0	3.996535	6.235757	7.752394	0	6.797442	1.852054	4.669151	1.921399	1.204755	1.840156	11.30893	11.16864	4.890029	2.473355	1.026376	1.820267	1.792616
AC009139.	7.68726	0	4.995668	2.078586	9.967364	0	9.346483	2.778081	0	0	1.204755	1.840156	1.028084	2.792161	0.978006	0.824452	4.105505	3.640534	0

AC009142.	0	4.001562	0	0	0	0	0	0	0	5.764196	0	0	0	0	0	0.824452	0	0	0.896308
AC009145.	8.648168	8.003124	26.97661	6.235757	3.322455	22.83774	6.797442	8.334243	5.836439	0	4.81902	11.04094	23.64594	8.376483	2.934017	3.297807	4.105505	13.652	3.585232
AC009145.	0	2.000781	0.999134	0	1.107485	1.141887	0	0.926027	0	3.842797	4.81902	0	1.028084	1.861441	0.978006	0	0	0.910133	1.792616
AC009145.	0	0	0	0	0	0	3.398721	0	0	0	0	0	0	0	0	0	0	0	0
AC009145.	16.33543	11.00429	13.98787	10.39293	37.65449	25.12151	0.84968	1.852054	151.7474	4.803496	63.85201	5.520469	9.252759	11.16864	13.69208	2.473355	6.158258	10.9216	13.44462
AC009148.	66.30262	125.0488	159.8614	68.59332	93.02873	87.9253	79.02026	141.6821	67.70269	183.4936	239.7462	142.6121	84.30292	95.86419	122.2507	41.22259	51.31881	87.37281	83.35664
AC009152.	6.726353	5.001952	4.995668	0	1.107485	0	6.797442	0.926027	1.167288	0	0	0	1.028084	0	0.978006	2.473355	0	0.910133	0
AC009153.	0	0	0	0	1.107485	0	0	0	0	0.960699	0	0	6.168506	0	0	5.771163	0	0	0
AC009154.	8.648168	2.000781	11.9896	4.157171	0	7.993209	5.947762	6.482189	8.171014	9.606993	1.204755	9.200782	19.5336	6.515042	12.71408	6.595615	0	2.7304	2.688924
AC009154.	10.56998	7.002733	15.98614	2.078586	14.3973	1.141887	43.33369	22.22465	57.1971	15.37119	30.11887	20.24172	29.81445	14.89152	8.802052	1.648904	9.237386	8.191201	6.274156
AC009158.	0	0	5.994802	0	0	4.567548	1.69936	0	0	0	0	0	0	0	3.912023	0.824452	4.105505	0	0
AC009159.	5.765445	14.00547	2.997401	11.43222	13.28982	1.141887	11.04584	16.66849	9.338302	11.52839	20.48083	4.600391	2.056169	0.93072	0.978006	3.297807	2.052753	5.4608	2.688924
AC009159.	73.98988	24.00937	62.94542	58.2004	34.33203	33.11472	33.13753	40.74519	0	49.95636	19.27608	57.96493	79.1625	25.12945	37.16422	54.41382	28.73854	39.13574	27.78555
AC009159.	464.1183	244.0953	484.5798	259.8232	407.5545	300.3163	232.8124	287.0684	624.4989	398.6902	501.178	386.4329	381.4193	293.1769	226.8974	267.1224	205.2753	283.9616	243.7958
AC009159.	29.78813	25.00976	42.96275	28.0609	18.82724	22.83774	35.68657	29.63287	61.86625	37.46727	62.64725	17.48149	19.5336	13.03008	26.40616	17.31349	11.29014	6.370934	8.963079
AC009163.	19.21815	8.003124	21.98094	8.314342	9.967364	12.56076	22.94137	24.0767	0	8.646293	2.40951	30.36258	27.75828	21.40657	11.73607	7.420066	12.31652	14.56213	16.13354
AC009163.	128.7616	96.03748	126.89	122.6365	96.35119	108.4793	130.0011	180.5753	116.7288	107.5983	122.885	118.6901	125.4263	105.1714	132.0308	76.67402	93.40024	103.7552	129.9647
AC009163.	5.765445	18.00703	5.994802	10.39293	8.859879	14.84453	0.84968	10.1863	3.501863	11.52839	10.84279	0.920078	1.028084	4.653602	3.912023	6.595615	7.184634	0.910133	4.48154
AC009163.	509.281	437.1706	647.4386	351.281	343.3203	409.9374	493.6642	455.6053	382.8704	522.6204	395.1596	477.5206	458.5256	537.0256	489.0029	274.5425	342.8097	326.7379	376.4493
AC009166.	0.960908	3.001171	0	11.43222	0	1.141887	0.84968	0	0	0.960699	14.45706	0	0	0.93072	0	6.595615	1.026376	3.640534	0
AC009166.	13.45271	37.01445	27.97574	47.80747	3.322455	34.25661	11.89552	32.41095	10.50559	24.01748	25.29985	53.36454	30.84253	16.75297	8.802052	11.54233	21.5539	12.74187	14.34093
AC009167.	1.921815	1.00039	0	3.117878	3.322455	3.425661	0	1.852054	2.334575	0	0	1.840156	4.112337	3.722881	4.890029	0	1.026376	0	1.792616
AC009171.	0	0	0	1.039293	1.107485	6.851322	7.647122	1.852054	2.334575	0.960699	8.433284	2.760235	8.224675	2.792161	6.846041	8.244518	4.105505	3.640534	22.4077
AC009185.	12.4918	20.00781	10.99047	18.70727	22.1497	21.69585	34.83689	14.81643	9.338302	12.48909	7.228529	20.24172	23.64594	30.71377	18.58211	15.66458	16.42202	16.3824	33.16339
AC009220.	3.84363	19.00742	22.98007	5.196464	14.3973	13.70264	18.69297	5.556162	38.5205	21.13538	8.433284	17.48149	11.30893	3.722881	6.846041	10.71787	17.4484	19.1128	4.48154
AC009220.	9.609075	8.003124	15.98614	18.70727	15.50479	10.27698	20.39233	10.1863	1.167288	20.17468	6.023774	21.1618	14.39318	14.89152	24.45015	11.54233	8.21101	13.652	20.61508
AC009220.	58.61536	31.0121	54.95235	39.49313	53.15928	39.96605	52.68017	56.48765	9.338302	33.62447	13.2523	43.24368	38.03912	42.81313	41.07624	37.92478	24.63303	45.50667	39.43755
AC009226.	28.82723	23.00898	8.992203	36.37525	24.36467	17.12831	36.53625	23.15068	24.51304	18.25329	4.81902	14.72125	20.56169	17.68369	8.802052	10.71787	14.36927	29.12427	21.51139
AC009227.	43.24084	60.02343	49.95668	74.82908	44.2994	22.83774	52.68017	35.18903	5.836439	26.89958	24.0951	32.20274	24.67402	43.74385	60.63636	63.48279	64.6617	33.67494	51.08955
AC009227.	0	1.00039	0.999134	0	0	1.141887	0.84968	6.482189	0	0	0	1.840156	1.028084	2.792161	0	0	1.026376	0	0
AC009228.	34.59267	18.00703	34.96968	21.82515	29.90209	18.27019	30.58849	15.74246	7.003726	13.44979	20.48083	15.64133	22.61786	28.85233	15.64809	18.96239	19.50115	13.652	15.23724
AC009229.	37.47539	41.01601	68.94022	68.59332	79.73891	68.51322	60.3273	87.97257	19.84389	34.58517	26.50461	81.88696	55.51656	78.18051	85.08651	47.81821	32.84404	94.65388	64.53417
AC009229.	30.74904	41.01601	72.93676	44.68959	125.1458	34.25661	82.41898	144.4602	106.2232	164.2796	140.9563	142.6121	94.58376	241.9873	317.8519	53.58937	24.63303	251.1968	126.3794
AC009229.	3.84363	4.001562	29.97401	13.51081	11.07485	13.70264	6.797442	14.81643	14.00745	4.803496	7.228529	40.48344	32.8987	36.29809	28.36217	16.48904	3.079129	23.66347	27.78555
AC009229.	33.63176	15.00586	96.91597	37.41454	63.12664	18.27019	34.83689	97.23284	24.51304	111.4411	90.35662	159.1735	112.0612	135.8852	177.0191	57.71163	46.18693	158.3632	106.6606
AC009234.	0.960908	8.003124	3.996535	17.66798	0	13.70264	37.38593	7.408216	0	8.646293	0	16.56141	3.084253	9.307203	9.780058	10.71787	13.34289	16.3824	17.92616
AC009237.	0.960908	0	0	0	1.107485	2.283774	0.84968	0	0	0	0	5.520469	5.140422	0	0	4.946711	2.052753	1.820267	0.896308
AC009237.	0.960908	0	0	0	0	0	0	0	0	0	0	1.840156	1.028084	0.93072	0	0	0	1.820267	0
AC009237.	162.3934	115.0449	143.8752	95.61493	116.2859	172.4249	103.661	139.8301	143.5764	175.808	140.9563	130.6511	134.6791	148.9152	147.6789	98.93422	131.3762	116.4971	145.2019
AC009237.	115.3089	120.0469	172.8501	128.8723	98.56616	115.3306	186.9297	100.0109	58.36439	87.42363	49.39495	153.6531	155.2407	146.1231	144.7449	143.4546	152.9301	98.29441	160.4391
AC009237.	0	0	3.996535	0	4.42994	0	0.84968	4.630135	0	0	0	3.680313	0	0	2.934017	0	0	0.910133	5.377848
AC009237.	239.266	169.066	237.7938	284.7662	170.5527	205.5397	330.5256	156.4986	108.5578	110.4804	118.066	350.5498	265.2458	206.6199	229.8314	267.1224	151.9037	180.2064	345.9749
AC009237.	0	0	0	0	0	0	0.84968	0	0	0	0	0	0	0	1.956012	0	0	0	0
AC009237.	5.765445	0	0.999134	0	1.107485	1.141887	0.84968	3.704108	0	0	0	11.04094	1.028084	1.861441	0.978006	0.824452	3.079129	4.550667	5.377848
AC009237.	3.84363	1.00039	2.997401	2.078586	1.107485	2.283774	1.69936	0	0	0.960699	0	1.840156	1.028084	0.93072	1.956012	0	0	0.910133	0
AC009237.	88.40349	80.03124	101.9116	117.4401	77.52394	47.95925	123.2036	80.56435	10.50559	33.62447	18.07132	117.77	117.2016	102.3792	90.95454	96.46086	69.79359	109.216	78.8751
AC009237.	0	2.000781	8.992203	5.196464	0	0	0	5.556162	0	1.921399	9.638039	0	4.112337	1.861441	1.956012	0	0	10.9216	0.896308
AC009238.	0	0	0	0	0	0	0	1.852054	0	0	0	2.760235	5.140422	2.792161	1.956012	0	1.026376	0	0
AC009238.	1.921815	0	11.9896	1.039293	7.752394	1.141887	2.549041	0	0	0.960699	0	1.840156	1.028084	1.861441	0	4.946711	1.026376	0	5.377848
AC009242.	84.55986	91.03553	149.87	82.10413	158.3703	90.20907	139.3476	67.59997	130.7362	85.50223	193.9655	132.4913	185.0552	86.55699	81.17448	37.92478	100.5849	94.65388	138.9277
AC009244.	43.24084	41.01601	110.9038	70.67191	65.34161	50.24303	34.83689	6.482189	1.167288	35.54587	38.55216	10.12086	9.252759	65.15042	45.96627	66.7806	70.81996	50.05734	47.50432
AC009245.	24.02269	24.00937	26.97661	27.02161	14.3973	19.41208	20.39233	22.22465	33.85134	35.54587	9.638039	27.60235	21.58977	31.64449	23.47214	28.85581	35.92317	25.48374	10.7557
AC009248.	0	0	0	0	0	0	0	0	14.00745	0	1.204755	0	0	0	0	0	0	0	0
AC009248.	4.804538	0	0.999134	0	0	0	1.69936	0	0	4.803496	0	0	0	1.861441	0	0	0	0	0
AC009262.	10.56998	1.00039	0	0	0	0	2.549041	5.556162	0	2.882098	0	2.760235	0	5.584322	3.912023	4.122259	2.052753	10.9216	0.896308
AC009263.	1.921815	0	0	2.078586	0	0	1.69936	3.704108	0	0	0	0	0	0	0.978006	4.122259	0	0	0
AC009264.	1.921815	4.001562	8.992203	3.117878	3.322455	4.567548	3.398721	0	0	0.960699	0	9.200782	6.168506	0	0	1.648904	5.131881	0	0
AC009268.	0	3.001171	14.987	0	0	12.56076	5.947762	1.852054	0	8.646293	0	5.520469	3.084253	2.792161	7.824047	0.824452	0	0.910133	4.48154
AC009268.	12.4918	9.003514	24.97834	10.39293	22.1497	21.69585	2.549041	20.37259	17.50932	12.48909	16.86657	7.360626	21.58977	6.515042	0.978006	8.244518	18.47477	12.74187	14.34093
AC009269.	6.726353	0	3.996535	0	5.537425	1.141887	0	14.81643	4.669151	0	8.433284	3.680313	1.028084	5.584322	7.824047	3.297807	1.026376	1.820267	5.377848
AC009269.	0	0	3.996535	0	0	0	0	0	0	0	0	0	0	0	0.978006	0	0	0	0
AC009269.	10.56998	3.001171	8.992203	7.275049	14.3973	0	5.947762	6.482189	1.167288	7.685594	3.614265	2.760235	5.140422	9.307203	18.58211	0.824452	5.131881	0	0

AC009269.	0	1.00039	0	0	0	1.141887	0	0	0	0	0	0.920078	2.056169	0.93072	0.978006	1.648904	5.131881	0	0.896308
AC009269.	123.9571	29.01132	44.96101	101.8507	29.90209	18.27019	106.21	24.0767	53.69524	30.74238	39.75691	57.04485	51.40422	72.59618	68.46041	86.56744	58.50345	61.88907	56.4674
AC009269.	126.8398	47.01835	66.94196	15.58939	98.56616	38.82416	43.33369	87.97257	60.69896	55.72056	59.03299	72.68618	80.19058	80.97267	93.88856	7.420066	8.21101	28.21414	20.61508
AC009271.	17.29634	14.00547	5.994802	4.157171	6.64491	7.993209	16.14392	25.92876	9.338302	5.764196	0	9.200782	4.112337	19.54513	15.64809	5.771163	4.105505	1.820267	11.652
AC009275.	877.3086	1052.411	701.3918	1162.969	605.7943	1023.131	872.6216	770.4545	910.4844	833.887	784.2954	646.815	671.3391	759.4678	764.8006	908.5459	1126.961	611.6097	745.7282
AC009276.	0.960908	0	0.999134	0	13.28982	0	1.69936	0	1.167288	0	0	0	0	0	0.978006	0	0	0	0
AC009283.	719.7197	657.2565	642.4429	549.7859	678.8883	776.4832	543.7954	622.2902	1344.715	841.5725	1131.265	637.6142	722.7433	698.9709	622.9897	534.2448	526.531	599.7779	470.5617
AC009292.	0	10.0039	6.993936	1.039293	5.537425	2.283774	0	2.778081	0	1.921399	1.204755	6.440548	0	5.584322	12.71408	9.06897	12.31652	13.652	9.859387
AC009292.	8.648168	2.000781	2.997401	1.039293	6.64491	1.141887	8.496802	3.704108	5.836439	4.803496	7.228529	1.840156	1.028084	5.584322	3.912023	11.54233	36.94955	4.550667	4.48154
AC009299.	100.8953	43.01679	0	0	29.90209	0	11.04584	0	0	0	0	0	0	3.722881	0	0	0	44.59654	78.8751
AC009299.	21.13997	21.0082	12.98874	16.62868	9.967364	7.993209	28.88913	8.334243	1.167288	5.764196	4.81902	19.32164	9.252759	17.68369	13.69208	9.06897	24.63303	29.12427	18.82247
AC009299.	35.55358	35.01367	49.95668	57.1611	40.97694	14.84453	91.76547	45.37532	0	43.23147	6.023774	76.36649	41.12337	34.43665	72.37243	56.88718	43.1078	73.72081	48.40063
AC009302.	10.56998	4.001562	0	3.117878	2.21497	6.851322	0.84968	6.482189	5.836439	7.685594	2.40951	0	2.056169	4.653602	0	5.771163	8.21101	7.281067	6.274156
AC009303.	0.960908	1.00039	2.997401	1.039293	11.07485	2.283774	7.647122	4.630135	0	0	4.81902	11.96102	9.252759	6.515042	6.846041	9.893422	8.21101	15.47227	16.13354
AC009303.	0.960908	3.001171	3.996535	3.117878	6.64491	1.141887	3.398721	9.26027	0	4.803496	1.204755	7.360626	6.168506	9.307203	12.71408	5.771163	2.052753	2.7304	0
AC009303.	0	0	0	0	0	0	0	0	0	0	0	0.920078	2.056169	0	0	0	0	0	0.896308
AC009303.	702.4234	885.3455	766.3355	878.2024	718.7577	679.4228	974.5832	733.4134	859.1238	796.4197	812.0048	946.7605	1012.663	912.1059	865.5352	912.6682	1048.957	970.2022	1227.046
AC009305.	0	0	0	0	0	0	0	6.482189	0	0	0	1.840156	3.084253	0	4.890029	0	0	0	0.896308
AC009309.	53.81082	92.03592	58.94889	51.96464	75.30897	60.52001	45.03305	64.82189	130.7362	64.36685	27.70936	55.20469	55.51656	61.42754	69.43841	41.22259	52.34519	54.608	48.40063
AC009312.	29.78813	5.001952	14.987	29.1002	3.322455	7.993209	17.84329	9.26027	0	0.960699	26.50461	15.64133	6.168506	10.23792	2.934017	35.45143	12.31652	7.281067	12.54831
AC009313.	2.882723	11.00429	0	1.039293	2.21497	0	3.398721	0.926027	0	0	0	7.360626	0	1.861441	0	0.824452	0	0.910133	0.896308
AC009313.	0	0	0	0	1.107485	0	0	0	0	0	0	0	0	0	0.978006	0	0	0	0
AC009314.	0.960908	0	0	1.039293	0	0	0.84968	0	0	6.724895	0	3.680313	0	0	0	0	1.026376	0	0
AC009318.	550.6	865.3377	552.5209	968.6209	729.8326	404.228	1656.027	615.808	228.7884	380.4369	285.5269	994.6046	1007.523	722.239	804.8988	854.9565	855.9978	1218.669	1292.476
AC009318.	173.9243	203.0793	246.786	223.4479	191.5949	223.8099	135.9488	194.4657	110.8923	260.3495	198.7846	158.2535	191.2237	246.6409	220.0513	145.928	168.3257	121.9579	129.0683
AC009318.	101.8562	162.0633	121.8943	194.3477	84.16885	106.1955	112.1578	154.6465	145.911	176.7687	178.3037	111.3295	114.1174	105.1714	113.4487	104.7054	119.0596	81.00187	58.26002
AC009318.	60.53717	209.0816	139.8787	96.65423	160.5853	101.6279	150.3934	150.9424	186.766	167.1617	145.7753	136.1716	181.9709	123.7858	149.6349	98.93422	119.0596	192.0382	178.3653
AC009319.	5.765445	11.00429	3.996535	12.47151	0	1.141887	1.69936	4.630135	0	11.52839	10.84279	10.12086	4.112337	4.653602	8.802052	12.36678	12.31652	5.4608	5.377848
AC009320.	59.57627	51.01991	33.97054	39.49313	18.82724	61.6619	40.78465	57.41368	33.85134	37.46727	25.29985	57.04485	47.29188	38.15953	43.03226	46.99375	55.42432	55.51814	69.01571
AC009336.	37.47539	88.03436	53.95322	46.76817	48.72934	74.22266	48.43177	54.6356	44.35693	30.74238	51.80446	42.3236	51.40422	72.59618	90.95454	37.92478	69.79359	63.70934	70.80833
AC009336.	31.70995	41.01601	32.97141	14.5501	74.20149	46.81737	21.24201	25.00273	49.02608	117.2053	218.0606	57.96493	67.85357	46.53602	55.74633	8.244518	26.68578	60.06881	60.94894
AC009336.	21.13997	2.000781	6.993936	55.08252	11.07485	31.97284	26.34009	26.85478	23.34575	16.33189	20.48083	4.600391	13.3651	3.722881	24.45015	23.08465	32.84404	5.4608	13.44462
AC009362.	0	0	0	0	0	0	0.84968	0	0	0.960699	0	0	0	0.93072	0	0	0	0	0
AC009365.	2.882723	3.001171	0	5.196464	5.537425	0	16.14392	1.852054	0	1.921399	0	0	1.028084	0.93072	5.868035	3.297807	1.026376	7.281067	3.585232
AC009396.	0	2.000781	0	0	0	0	5.098081	0	0	0	0	0.920078	1.028084	0	0	0.824452	1.026376	0	0
AC009396.	0	6.002343	0	1.039293	0	0	0	0.926027	0	0	7.228529	1.840156	1.028084	0	0	0	0	0	0.896308
AC009403.	316.1386	220.0859	409.6448	299.3163	330.0305	261.4921	279.5448	389.8574	388.7068	347.7731	333.7171	290.7447	318.7062	377.8724	341.324	326.4829	290.4645	364.0534	296.6779
AC009403.	13.45271	15.00586	10.99047	24.94303	25.47215	15.98642	17.84329	12.03835	12.84017	12.48909	24.0951	13.80117	17.47743	8.376483	17.6041	8.244518	4.105505	27.304	5.377848
AC009404.	474.6883	446.1741	544.5278	541.4715	398.6946	646.308	448.6312	532.4655	791.4211	555.2842	581.8966	501.4426	544.8847	473.7366	577.0234	606.7965	692.804	369.5142	490.2804
AC009407.	17.29634	21.0082	28.97488	21.82515	23.25718	35.3985	28.03945	32.41095	46.69151	14.41049	28.91412	12.8811	14.39318	17.68369	15.64809	11.54233	11.29014	17.29253	42.12647
AC009413.	1.921815	0	0	0	0	0	0	0	1.167288	1.921399	0	0.920078	0	0.93072	0.978006	0.824452	2.052753	5.4608	0
AC009414.	0	0	0	0	1.107485	0	0	0	9.338302	0.960699	0	0	0	0	0	0	0	0	0
AC009414.	91.28622	161.0629	129.8874	65.47544	69.77155	113.0468	85.8177	123.1616	331.5097	203.6682	143.3658	107.6492	92.52759	116.34	111.4927	70.07841	125.2179	92.83361	75.28987
AC009432.	0	0	3.996535	0	0	0	0	0	0	0	0	0	0	0	0	0	0	0	0
AC009435.	3.84363	2.000781	2.997401	1.039293	0	0	0.84968	1.852054	0	0	1.204755	0	2.056169	0.93072	3.912023	0	9.237386	0	1.792616
AC009452.	0.960908	0	0	0	0	0	0	0	0	0	0	0.920078	0	0.93072	0	0.824452	1.026376	0	3.585232
AC009464.	7.68726	0	0	8.314342	7.752394	2.283774	9.346483	20.37259	9.338302	6.724895	0	10.12086	8.224675	0	8.802052	4.946711	1.026376	4.550667	2.688924
AC009480.	0	3.001171	1.998267	8.314342	0	2.283774	3.398721	2.778081	0	1.921399	0	2.760235	1.028084	2.792161	1.956012	0.824452	1.026376	1.820267	1.792616
AC009486.	76.8726	63.0246	113.9012	99.77211	56.48173	133.6008	113.8572	77.78627	47.8588	43.23147	34.93789	142.6121	136.7352	107.9636	94.86656	122.8433	107.7695	116.4971	112.9348
AC009487.	80.71623	63.0246	149.87	43.6503	93.02873	29.68906	92.61515	67.59997	35.01863	56.68126	57.82823	88.32751	83.27483	50.2589	66.5044	77.49847	80.05735	45.50667	77.08248
AC009487.	0	0	0	0	0	1.141887	0	0	0	0	0	0.920078	0	0	0	0	0	0	0
AC009487.	0	1.00039	0	0	0	1.141887	0.84968	0	0	0	0	0	0	0	0	0	0	0	0
AC009495.	10.56998	15.00586	17.98441	14.5501	14.3973	2.283774	13.59488	8.334243	4.669151	4.803496	7.228529	7.360626	9.252759	7.445762	6.846041	5.771163	8.21101	9.101334	9.859387
AC009495.	19.21815	45.01757	32.97141	46.76817	77.52394	18.27019	49.28145	80.56435	12.84017	46.11356	28.91412	25.76219	38.03912	13.03008	17.6041	22.2602	24.63303	15.47227	29.57816
AC009495.	20.17906	20.00781	24.97834	21.82515	37.65449	15.98642	12.7452	23.15068	31.51677	12.48909	25.29985	25.76219	17.47743	20.47585	31.29619	22.2602	13.34289	11.83173	17.92616
AC009498.	0	2.000781	0.999134	18.70727	1.107485	6.851322	7.647122	4.630135	0	1.921399	0	17.48149	10.28084	0.93072	1.956012	7.420066	3.079129	0	7.170464
AC009501.	1327.013	1077.421	1524.678	850.1415	957.9745	992.2998	1188.703	1008.443	1449.771	1260.437	1185.479	1457.404	1513.34	1275.087	1468.965	866.4989	1028.429	1386.133	1469.049
AC009501.	9.609075	3.001171	8.992203	4.157171	4.42994	7.993209	0.84968	2.778081	17.50932	6.724895	12.04755	5.520469	2.056169	8.376483	0	3.297807	3.079129	2.7304	3.585232
AC009501.	0	0	0	0	0	0	0.84968	0	0	0	0	0	0	5.584322	3.912023	0	0	0	0
AC009502.	0	0	0	0	0	0	0	0	0	0	0	0.920078	0	0	0	1.648904	0	0	0
AC009506.	145.097	118.0461	190.8345	104.9686	137.3281	157.5804	110.4584	164.8328	183.2642	175.808	222.8797	101.2086	133.651	155.4303	151.5909	125.3167	150.8773	150.172	156.8539
AC009509.	67.26353	41.01601	57.94975	38.45383	33.22455	75.36454	44.18337	106.4931	52.52795	104.7162	79.51382	55.20469	44.20763	45.60529	31.29619	40.39814	50.29244	30.0344	38.54124

AC009509.	8.648168	4.001562	0.999134	3.117878	0	3.425661	1.69936	2.778081	21.01118	2.882098	4.81902	0	0	0	2.934017	0	0	0	0.896308
AC009509.	0	0	0	0	0	0	0	0	2.334575	0.960699	0	0	0	0	0	0	0	0.910133	0
AC009509.	115.3089	96.03748	130.8865	70.67191	170.5527	184.9857	137.6482	158.3506	292.9892	269.9565	265.0461	93.84798	96.63993	140.5388	115.4047	80.79628	120.086	40.04587	61.84525
AC009511.	0	0	0	1.039293	0	0	0	0	0	0	0	4.600391	0	0	0	0.824452	0	1.820267	0.896308
AC009518.	28.82723	23.00898	9.991337	31.17878	24.36467	18.27019	31.43817	15.74246	30.34948	23.05678	38.55216	19.32164	30.84253	12.09936	15.64809	17.31349	25.65941	11.83173	24.20031
AC009518.	0	4.001562	1.998267	0	1.107485	0	1.69936	8.334243	0	0	0	0.920078	0	0	0.978006	0	1.026376	0	0
AC009533.	1248.219	727.2838	447.6119	273.334	1915.949	390.5254	238.7601	1314.032	1506.968	2042.447	3250.429	1040.608	962.287	1579.432	1641.094	910.1948	991.4795	1903.089	1800.683
AC009533.	22.10087	27.01054	3.996535	0	27.68712	4.567548	17.84329	33.33697	8.171014	63.40615	39.75691	11.96102	19.5336	41.88241	52.81231	42.04704	47.21331	24.5736	30.47447
AC009541.	0	4.001562	0	0	4.42994	0	0	0	0	0	0	0	0	0	0	0	0	0	0
AC009542.	12.4918	48.01874	97.9151	75.86837	37.65449	17.12831	63.72602	49.07943	25.68033	36.50657	10.84279	72.68618	57.57272	40.02097	107.5806	25.55801	20.52753	23.66347	35.85232
AC009542.	1.921815	6.002343	15.98614	7.275049	19.93473	4.567548	9.346483	31.48492	3.501863	8.646293	2.40951	14.72125	4.112337	8.376483	18.58211	51.11601	60.5562	13.652	10.7557
AC009549.	626.5117	863.3369	636.4481	651.6366	692.1781	723.9564	627.064	491.7204	1188.299	1140.35	1634.852	445.3179	488.3401	565.8779	564.3094	638.9502	818.0219	795.4566	1000.28
AC009554.	0	0	0	1.039293	2.21497	0	0.84968	0	0	0	0	0.920078	0	0	0.978006	0	0	0.910133	1.792616
AC009558.	2.882723	9.003514	1.998267	10.39293	1.107485	11.41887	3.398721	2.778081	0	0.960699	2.40951	8.280704	4.112337	9.307203	9.780058	4.122259	19.50115	10.9216	2.688924
AC009558.	0	0	0	0	0	1.141887	0	0	0	0	0	0	0	0	0	0	1.026376	0	5.377848
AC009560.	2.882723	3.001171	10.99047	3.117878	6.64491	0	0.84968	4.630135	21.01118	21.13538	0	4.600391	5.140422	0	6.846041	4.122259	1.026376	6.370934	6.274156
AC009562.	0	1.00039	0	3.117878	0	0	0.84968	0	1.167288	0	0	3.680313	1.028084	0	0	0	0	0	0.896308
AC009563.	8.648168	10.0039	19.98267	18.70727	21.04221	9.135096	23.79105	17.59451	11.67288	23.05678	1.204755	16.56141	12.33701	13.9608	15.64809	1.648904	22.58028	17.29253	16.13354
AC009570.	22.10087	39.01523	40.96448	24.94303	22.1497	19.41208	22.09169	14.81643	24.51304	25.93888	22.89034	17.48149	17.47743	19.54513	11.73607	24.73355	9.237386	30.94454	25.09662
AC009597.	0	0	0	1.039293	0	2.283774	0	0	0	0	0	0	1.028084	0	4.890029	0	0	0.910133	0
AC009597.	0	0	0	0	0	1.141887	0	0	0	0	0	0.920078	0	0	0	0	0	0	0
AC009623.	0	5.001952	13.98787	0	0	0	0	0	0	7.685594	0	0	0	0	0	0	0	1.820267	0.896308
AC009623.	0	0	0	0	0	0	0.84968	0	0	0	0	0	1.028084	0	0.978006	0	0	0	0
AC009630.	2.882723	18.00703	5.994802	12.47151	19.93473	21.69585	10.19616	4.630135	7.003726	15.37119	6.023774	11.96102	13.3651	18.61441	10.75806	19.78684	13.34289	10.9216	11.652
AC009630.	4.804538	0	5.994802	1.039293	12.18233	1.141887	0	3.704108	2.334575	3.842797	3.614265	4.600391	8.224675	6.515042	2.934017	0.824452	1.026376	15.47227	2.688924
AC009643.	0	0	0	0	0	0	0	0	0	0	2.40951	0	0	0	0	0	0	0	0
AC009646.	2.882723	6.002343	1.998267	1.039293	4.42994	1.141887	0	14.81643	2.334575	9.606993	6.023774	8.280704	6.168506	3.722881	9.780058	0.824452	1.026376	2.7304	7.170464
AC009652.	0.960908	21.0082	20.98181	6.235757	35.43952	9.135096	10.19616	20.37259	19.84389	15.37119	7.228529	2.760235	3.084253	20.47585	10.75806	2.473355	1.026376	3.640534	4.48154
AC009652.	0.960908	0	1.998267	1.039293	8.859879	5.709435	3.398721	0.926027	0	0	0	0.920078	1.028084	2.792161	0.978006	0.824452	12.31652	2.7304	5.377848
AC009654.	14.41361	32.01249	3.996535	9.353635	23.25718	14.84453	11.04584	12.03835	23.34575	0	10.84279	28.52243	14.39318	11.16864	15.64809	5.771163	2.052753	6.370934	14.34093
AC009656.	0	0	0	0	0	0	0.84968	0	0	0	0	0	0	0.93072	0	0	0	0	0
AC009660.	0.960908	1.00039	4.995668	13.51081	0	1.141887	4.248401	0	0	0.960699	0	2.760235	0	0	8.802052	4.122259	6.158258	6.370934	1.792616
AC009686.	2.882723	3.001171	1.998267	5.196464	1.107485	2.283774	5.947762	0.926027	0	0	0	3.680313	0	11.16864	4.890029	2.473355	1.026376	4.550667	3.585232
AC009686.	42.27993	70.02733	41.96361	65.47544	26.57964	68.51322	49.28145	32.41095	44.35693	47.07426	113.247	45.08383	62.71315	59.5661	44.01026	67.60505	70.81996	28.21414	47.50432
AC009690.	94.16894	94.0367	91.9203	68.59332	98.56616	124.4657	114.7068	75.93422	149.4128	154.6726	199.9893	124.2106	99.72418	61.42754	66.5044	75.84957	79.03097	99.20454	167.6096
AC009690.	55.73264	36.01406	61.94629	49.88605	62.01916	55.95246	82.41898	67.59997	39.68778	93.18783	71.08054	78.20665	77.10633	96.79491	77.26246	70.90286	42.08143	62.79921	51.98586
AC009704.	3.84363	0	13.98787	7.275049	0	0	8.496802	10.1863	0	0.960699	0	4.600391	0	1.861441	0.978006	0.824452	5.131881	0.910133	0.896308
AC009704.	22.10087	24.00937	32.97141	15.58939	42.08443	23.97963	28.03945	35.18903	17.50932	32.66377	9.638039	33.12282	27.75828	18.61441	60.63636	40.39814	33.87042	12.74187	26.88924
AC009716.	41.31902	43.01679	97.9151	37.41454	29.90209	22.83774	56.92858	67.59997	4.669151	14.41049	4.81902	71.7661	47.29188	39.09025	36.18622	23.08465	24.63303	43.6864	22.4077
AC009716.	5.765445	6.002343	0.999134	2.078586	5.537425	1.141887	4.248401	9.26027	0	7.685594	12.04755	7.360626	17.47743	16.75297	9.780058	18.96239	15.39564	4.550667	1.792616
AC009720.	0	4.001562	0	0	0	0	0	0	0	0	0	0	0	0	0	0	0	0	0
AC009729.	1.921815	0	0	0	0	0	0.84968	0	0	0	0	0	0	0	0.978006	8.244518	0	0	6.274156
AC009743.	0	0	0	0	0	0	0	0	0	3.842797	0	0	0	1.861441	0	0	0	0	0.896308
AC009754.	13.45271	35.01367	38.96621	12.47151	16.61227	31.97284	24.64073	21.29862	21.01118	19.21399	20.48083	24.84211	19.5336	38.15953	56.72434	11.54233	18.47477	10.01147	12.54831
AC009779.	6.726353	26.01015	23.97921	2.078586	8.859879	9.135096	6.797442	14.81643	43.18965	11.52839	16.86657	12.8811	16.44935	5.584322	10.75806	1.648904	10.26376	9.101334	39.43755
AC009779.	1239.571	1436.561	1196.962	1284.566	1520.577	1520.993	939.7463	1717.78	1670.389	1640.874	2089.045	1473.045	1360.156	1508.698	1215.661	1389.201	1375.344	1590.913	1647.414
AC009779.	723.5634	577.2253	497.5686	495.7427	406.447	443.0522	512.3572	696.3723	839.2799	701.3105	745.7433	446.2379	562.3622	432.7849	490.9589	732.9377	709.226	409.56	492.0731
AC009779.	0.960908	0	8.992203	0	8.859879	1.141887	0.84968	0.926027	11.67288	6.724895	9.638039	5.520469	2.056169	0.93072	0	0.824452	4.105505	1.820267	8.066772
AC009779.	0	0	0	0	0	0	0	0	0	0	9.638039	2.760235	0	0	0.978006	0	0	0	0.896308
AC009802.	117.2307	194.0757	158.8623	180.8369	126.2533	126.7495	62.02666	112.9753	332.677	216.1573	202.3988	120.5302	136.7352	137.7466	106.6026	165.7148	111.875	120.1376	161.3354
AC009804.	1.921815	0	0	0	0	0	0.84968	0	0	0	0	0	3.084253	0	0	0	0	0	0
AC009806.	87.44259	81.03163	105.9082	88.33989	81.95388	54.81058	84.11834	80.56435	115.5615	92.22713	72.28529	81.88696	71.96591	52.12034	105.6246	71.72731	75.95184	91.92348	69.91202
AC009812.	453.5484	402.157	471.5911	417.7957	416.4143	396.2348	493.6642	416.7122	476.2534	601.3977	434.9165	414.9553	526.3792	469.083	435.2126	341.3231	432.1044	475.9998	429.3315
AC009812.	0	0	2.997401	1.039293	0	3.425661	0.84968	0	0	0	7.228529	0	0	0	0	0	0	0	0
AC009812.	255.6014	263.1027	222.8068	606.947	199.3473	176.9925	522.5533	250.9533	14.00745	107.5983	75.89956	298.1053	279.6389	185.2133	240.5894	312.4672	330.4932	327.648	268.8924
AC009812.	320.9431	462.1804	489.5755	632.9293	377.6524	440.7684	515.7559	406.5259	566.1346	583.1444	415.6404	276.9435	374.2227	334.1286	357.9501	375.1256	429.0253	437.7742	340.597
AC009831.	78.79442	101.0394	106.9073	24.94303	84.16885	75.36454	37.38593	75.00819	151.7474	142.1835	213.2416	53.36454	52.4323	100.5178	61.61437	51.94047	81.08372	81.00187	65.43048
AC009831.	0.960908	3.001171	3.996535	0	4.42994	0	5.947762	2.778081	3.501863	0	0	2.760235	0	0.93072	2.934017	0	6.158258	0.910133	0
AC009831.	5.765445	13.00508	10.99047	6.235757	3.322455	3.425661	1.69936	3.704108	12.84017	5.764196	0	4.600391	12.33701	9.307203	6.846041	13.19123	0	9.101334	0.896308
AC009831.	0	1.00039	5.994802	0	0	0	7.647122	4.630135	0	3.842797	0	0	0	1.861441	4.890029	4.946711	0	0	1.792616
AC009869.	2.882723	2.000781	0	2.078586	0	0	0.84968	0	0	0	0	0	0	0.93072	0.978006	0	0	0	0
AC009878.	81.67714	63.0246	60.94715	161.0904	48.72934	115.3306	87.51706	56.48765	31.51677	61.48475	38.55216	47.84407	48.31997	50.2589	102.6906	61.83389	54.39794	43.6864	50.19325

AC009879.	0	1.00039	5.994802	2.078586	2.21497	6.851322	1.69936	5.556162	15.17474	0	1.204755	6.440548	5.140422	0	0	0	0	2.7304	12.54831
AC009884.	0	0	0	8.314342	0	0	0	0	1.167288	0	0	0	0	0	2.934017	0	1.026376	0	0
AC009884.	0	0	0	1.039293	0	0	0	0	0	0	0	0	0	0	0	0.824452	0	0	0
AC009901.	0	0	0	0	0	0	0	0	0	0	0	0	1.028084	0.93072	0	0	0	0	0
AC009901.	0	1.00039	0	0	0	0	0	0	0	0	0	0	1.028084	0	0	0	0	0	0
AC009902.	51.88901	20.00781	44.96101	36.37525	47.62185	37.68227	24.64073	21.29862	45.52422	52.83846	18.07132	74.52634	57.57272	47.46674	72.37243	49.46711	46.18693	49.1472	112.9348
AC009902.	185.4552	219.0855	232.7981	182.9155	222.6045	245.5057	161.4392	146.3123	226.4538	251.7032	226.4939	218.0585	172.7182	248.5023	196.5792	145.928	202.1961	293.063	319.9819
AC009908.	0	9.003514	2.997401	5.196464	0	1.141887	0	0.926027	9.338302	2.882098	4.81902	7.360626	0	8.376483	4.890029	0	3.079129	5.4608	10.7557
AC009927.	13.45271	9.003514	6.993936	46.76817	37.65449	13.70264	49.28145	6.482189	0	9.606993	0	23.92203	20.56169	18.61441	11.73607	25.55801	8.21101	19.1128	58.26002
AC009927.	0	2.000781	0	2.078586	0	4.567548	0	0	0	0	0	0.920078	0	0	0	0	0	0	0.896308
AC009948.	3075.865	3143.227	3071.337	4082.342	2725.52	3231.54	3731.796	3993.955	1579.34	2591.006	1944.474	3641.67	3685.682	3371.069	3383.9	3944.178	3410.648	4228.48	3327.991
AC009948.	12.4918	18.00703	26.97661	5.196464	22.1497	25.12151	21.24201	15.74246	10.50559	28.82098	57.82823	46.92399	37.01104	12.09936	20.53812	17.31349	24.63303	20.02294	31.37078
AC009948.	19.21815	22.00859	9.991337	12.47151	40.97694	7.993209	16.9936	16.66849	28.01491	14.41049	25.29985	26.68227	14.39318	19.54513	22.49413	18.96239	17.4484	10.9216	12.54831
AC009948.	34.59267	2.000781	15.98614	20.78586	28.79461	10.27698	32.28785	29.63287	10.50559	12.48909	8.433284	21.1618	27.75828	19.54513	20.53812	23.08465	22.58028	20.93307	18.82247
AC009948.	0	2.000781	0	0	0	0	10.19616	5.556162	0	0	9.638039	1.840156	2.056169	6.515042	0.978006	5.771163	4.105505	10.01147	0
AC009949.	14.41361	29.01132	11.9896	14.5501	35.43952	11.41887	32.28785	14.81643	2.334575	16.33189	12.04755	26.68227	28.78636	16.75297	21.51613	14.01568	2.052753	21.8432	43.02278
AC009950.	334.3958	402.157	542.5296	429.2279	396.4796	415.6469	503.0107	588.9532	187.9333	380.4369	346.9694	560.3276	551.0532	376.9417	403.9164	527.6492	399.2604	663.4873	626.5193
AC009950.	0	0	0	0	0	0	0	0	0	2.882098	0	0	0	0	0	0	1.026376	0.910133	0
AC009951.	17.29634	35.01367	50.95582	55.08252	24.36467	30.83095	33.98721	50.93149	49.02608	43.23147	19.27608	57.96493	34.95487	32.57521	24.45015	53.58937	53.37157	29.12427	7.170464
AC009951.	8.648168	12.00469	13.98787	3.117878	1.107485	3.425661	9.346483	12.03835	3.501863	11.52839	16.86657	11.04094	6.168506	0.93072	2.934017	16.48904	0	9.101334	0
AC009955.	3.84363	0	0.999134	4.157171	9.967364	3.425661	8.496802	5.556162	4.669151	0	4.81902	2.760235	2.056169	1.861441	0.978006	1.648904	4.105505	5.4608	3.585232
AC009955.	7.68726	23.00898	28.97488	18.70727	52.05179	30.83095	12.7452	16.66849	47.8588	18.25329	40.96167	23.00196	12.33701	19.54513	33.2522	10.71787	27.71216	20.93307	14.34093
AC009955.	24.9836	26.01015	31.97228	44.68959	35.43952	25.12151	27.18977	16.66849	43.18965	27.86028	7.228529	35.88305	38.03912	22.33729	15.64809	57.71163	50.29244	23.66347	47.50432
AC009955.	21.13997	15.00586	16.98527	21.82515	18.82724	33.11472	33.98721	31.48492	35.01863	41.31007	34.93789	42.3236	40.09529	17.68369	24.45015	31.32917	22.58028	25.48374	32.26709
AC009961.	46.12356	95.03709	89.92203	72.75049	66.4491	49.10114	63.72602	130.5698	35.01863	31.70308	34.93789	101.2086	83.27483	75.38834	90.95454	94.81196	107.7695	87.37281	123.6905
AC009961.	1.921815	11.00429	2.997401	3.117878	1.107485	2.283774	1.69936	4.630135	0	0	0	3.680313	11.30893	7.445762	4.890029	0.824452	0	1.820267	9.859387
AC009961.	6.726353	7.002733	4.995668	10.39293	43.19191	2.283774	9.346483	2.778081	0	5.764196	1.204755	2.760235	14.39318	23.26801	7.824047	14.01568	5.131881	0	6.274156
AC009962.	215.2433	155.0605	294.7444	252.5481	126.2533	125.6076	146.9947	204.652	79.37557	156.594	157.8229	183.0956	208.7011	161.0146	203.4252	244.0377	159.0883	223.8928	162.2317
AC009963.	0	0	0	0	0	0	0	5.556162	0	0	0	0	0	0	0	0	0	0	0
AC009963.	0.960908	0	0	0	0	0	0	0	0	0	0	0	0	0	0	0	0	3.640534	0
AC009963.	0	5.001952	0	6.235757	0	0	0.84968	0	0	0	0	0	4.112337	3.722881	0	0	2.052753	0.910133	0.896308
AC009963.	0	0	0	0	0	1.141887	0	0	0	0.960699	0	0	0	0	0.978006	0.824452	0	0	0
AC009967.	2.882723	9.003514	8.992203	12.47151	28.79461	4.567548	6.797442	17.59451	16.34203	21.13538	12.04755	3.680313	2.056169	9.307203	18.58211	4.122259	22.58028	6.370934	8.066772
AC009970.	1.921815	0	2.997401	6.235757	0	6.851322	2.549041	0	0	0	0	0.920078	2.056169	1.861441	1.956012	9.06897	3.079129	0.910133	1.792616
AC009971.	0	0	0	3.117878	0	0	0	0	0	0	0	0	0	0	0	0.824452	0	1.820267	0
AC009974.	94.16894	89.03475	69.93936	81.06484	80.8464	77.64832	82.41898	70.37805	117.8961	76.85594	90.35662	76.36649	90.47142	79.11123	91.93255	115.4233	84.16285	70.08027	45.71171
AC009975.	141.2534	115.0449	69.93936	190.1906	202.6697	106.1955	62.87634	10.1863	77.04099	135.4586	169.8704	173.8948	166.5497	184.2826	254.2815	10.71787	13.34289	44.59654	49.29694
AC009975.	2.882723	2.000781	0	2.078586	2.21497	0	9.346483	7.408216	0	2.882098	10.84279	1.840156	9.252759	0	0	0.824452	8.21101	0	4.48154
AC009977.	84.55986	54.02108	75.93416	56.12181	73.094	0	0	0	0	68.20965	28.91412	0	0	0	0	0	0	0	0
AC009987.	8.648168	0	4.995668	5.196464	6.64491	0	2.549041	3.704108	0	0.960699	0	8.280704	6.168506	1.861441	0.978006	1.648904	1.026376	1.820267	0.896308
AC009994.	6.726353	12.00469	23.97921	1.039293	11.07485	0	11.04584	1.852054	0	0.960699	0	1.840156	10.28084	7.445762	19.56012	4.122259	4.105505	2.7304	4.48154
AC009996.	70.14625	113.0441	106.9073	80.02554	145.0805	71.93888	121.5043	112.0493	31.51677	88.38433	45.78069	119.6102	112.0612	91.21059	88.02052	102.232	136.508	155.6328	95.00864
AC009997.	0	0	0	0	0	0	0	0	0	1.921399	0	0	0	0	0.978006	0	0	0	0
AC010082.	1.921815	1.00039	0	1.039293	0	2.283774	0	1.852054	0	0	0	0	1.028084	0.93072	0	0	1.026376	0.910133	0
AC010096.	0	0	0	0	0	0	0	0	0	1.921399	0	0	0	0	0	0	0	0	0.896308
AC010099.	0	0	0	0	3.322455	1.141887	0.84968	0.926027	0	0.960699	0	0.920078	0	0.93072	1.956012	0.824452	1.026376	0	0.896308
AC010099.	0	0	0	0	0	0	0	3.704108	0	0.960699	0	0	0	3.722881	0	0	0	0	0
AC010099.	0	1.00039	0	0	0	0	0	0.926027	0	7.685594	0	0	0	0.93072	0	1.648904	1.026376	0	0
AC010099.	3.84363	12.00469	22.98007	17.66798	4.42994	12.56076	21.24201	3.704108	3.501863	25.93888	7.228529	4.600391	10.28084	9.307203	7.824047	12.36678	10.26376	11.83173	10.7557
AC010105.	2.882723	0	0	1.039293	0	0	0	3.704108	0	0	0	0	0	0	0	0	0	0	0
AC010127.	74.95079	139.0543	76.93329	157.9725	128.4683	67.37133	207.322	82.41641	95.71759	98.95202	74.6948	49.68422	52.4323	90.27987	100.7346	176.4327	168.3257	161.0936	84.25295
AC010132.	2.882723	6.002343	0.999134	7.275049	0	1.141887	7.647122	2.778081	0	5.764196	7.228529	3.680313	6.168506	2.792161	3.912023	4.122259	18.47477	0.910133	5.377848
AC010132.	14.41361	2.000781	7.993069	7.275049	3.322455	6.851322	2.549041	0	0	1.921399	0	5.520469	1.028084	10.23792	4.890029	4.122259	4.105505	13.652	17.02985
AC010132.	70.14625	153.0597	169.8527	162.1297	167.2302	84.49964	171.6354	143.5342	255.636	134.4979	140.9563	113.1696	131.5948	90.27987	146.7009	120.37	148.8246	42.77627	58.26002
AC010136.	34.59267	36.01406	25.97748	14.5501	9.967364	47.95925	6.797442	15.74246	16.34203	30.74238	33.73314	23.00196	11.30893	19.54513	16.6261	18.96239	8.21101	17.29253	16.13354
AC010148.	24.9836	1.00039	27.97574	20.78586	7.752394	15.98642	3.398721	3.704108	9.338302	4.803496	1.204755	21.1618	13.3651	37.22881	32.27419	14.01568	6.158258	18.20267	22.4077
AC010149.	9.609075	6.002343	0.999134	15.58939	2.21497	2.283774	20.39233	16.66849	16.34203	1.921399	0	18.40156	7.196591	0.93072	7.824047	9.06897	10.26376	2.7304	9.859387
AC010149.	0	0	0	1.039293	0	0	0	1.852054	0	0	0	0	0	0	0	1.648904	0	0	0
AC010157.	0	3.001171	1.998267	0	0	0	1.69936	1.852054	0	0.960699	1.204755	0	0	3.722881	3.912023	0	0	0.910133	2.688924
AC010163.	1.921815	8.003124	3.996535	4.157171	0	11.41887	0.84968	0	1.167288	4.803496	4.81902	8.280704	3.084253	5.584322	3.912023	3.297807	1.026376	10.9216	0.896308
AC010163.	2.882723	2.000781	0.999134	1.039293	0	4.567548	2.549041	0	0	10.56769	0	0.920078	1.028084	0.93072	0	3.297807	0	0	0
AC010168.	16.33543	10.0039	6.993936	2.078586	5.537425	6.851322	8.496802	14.81643	2.334575	7.685594	6.023774	11.96102	26.73019	42.81313	17.6041	28.85581	20.52753	18.20267	17.92616

AC010168.	977.243	1138.444	1250.915	734.78	899.2778	1037.975	1218.441	1202.909	860.2911	1041.398	933.685	1028.647	1155.567	1328.138	1483.635	831.0474	825.2065	1487.158	1547.924
AC010173.	46.12356	51.01991	48.95755	42.611	39.86946	65.08756	36.53625	58.3397	107.3905	69.17035	118.066	52.44446	53.46039	53.98178	83.1305	72.55176	46.18693	66.43974	77.97879
AC010175.	85.52077	76.02967	68.94022	78.98625	86.38382	41.10793	90.06611	67.59997	194.9371	124.8909	97.58515	80.04681	70.93782	78.18051	86.06451	45.34485	94.42662	86.46267	123.6905
AC010181.	3.84363	0	0	0	0	1.141887	0	0	0	0	0	0	1.028084	0.93072	1.956012	0	0	3.640534	0.896308
AC010181.	0	0	0	0	0	0	0.84968	0	0	0	0	0.920078	0	0	0	0	0	0.910133	0
AC010182.	0.960908	1.00039	0	5.196464	0	0	3.398721	5.556162	7.003726	0.960699	8.433284	0	0	0	3.912023	4.122259	0	2.7304	0
AC010185.	0	0	0	3.117878	0	0	0.84968	0	0	0	0	0	2.056169	0	4.890029	2.473355	1.026376	0.910133	0.896308
AC010186.	2.882723	8.003124	6.993936	0	1.107485	5.709435	6.797442	0	7.003726	0	6.023774	0	0	0.93072	7.824047	3.297807	4.105505	2.7304	10.7557
AC010186.	557.3264	247.0964	372.6769	291.002	330.0305	358.5525	266.7996	363.9286	288.3201	209.4324	189.1465	412.195	341.324	393.6947	347.1921	467.4642	332.5459	469.6288	326.2561
AC010186.	602.489	386.1507	465.5963	454.1709	417.5218	505.8559	459.677	564.8765	437.7329	323.7556	320.4648	543.7662	466.7503	690.5945	652.3299	592.7809	433.1308	587.9462	456.2207
AC010197.	5.765445	1.00039	7.993069	107.0472	18.82724	14.84453	26.34009	4.630135	0	1.921399	0	1.840156	14.39318	2.792161	4.890029	51.11601	59.52982	6.370934	12.54831
AC010198.	30.74904	44.01718	69.93936	45.72888	57.58922	3.425661	19.54265	29.63287	115.5615	33.62447	32.52838	50.6043	47.29188	32.57521	39.12023	24.73355	40.02867	46.4168	51.98586
AC010200.	7.68726	40.01562	42.96275	16.62868	54.26676	53.66869	22.09169	21.29862	9.338302	48.03496	40.96167	41.40352	21.58977	24.19873	30.31818	18.13794	14.36927	28.21414	51.08955
AC010201.	110.5044	182.0711	242.7895	99.77211	187.165	247.7895	121.5043	174.0931	305.8294	282.4456	357.8122	190.4562	143.9318	125.6472	137.8988	87.39189	82.1101	157.4531	131.7573
AC010201.	963.7903	1278.499	1849.396	704.6405	1434.193	1172.718	1028.963	1507.572	849.7855	1582.272	1242.102	1433.482	1348.847	1189.461	1140.355	568.0473	672.2764	1141.307	1089.91
AC010203.	50.9281	16.00625	53.95322	35.33595	33.22455	35.3985	67.97442	63.89587	1.167288	21.13538	13.2523	46.00391	61.68506	45.60529	30.31818	43.69595	26.68578	17.29253	24.20031
AC010203.	48.04538	41.01601	38.96621	20.78586	56.48173	39.96605	69.67378	36.11505	7.003726	25.93888	2.40951	33.12282	52.4323	42.81313	64.54838	26.38246	66.71446	35.4952	53.77848
AC010205.	35.55358	29.01132	44.96101	29.1002	42.08443	37.68227	46.73241	39.81916	21.01118	27.86028	42.16642	26.68227	42.15146	72.59618	32.27419	23.08465	31.81766	44.59654	41.23017
AC010207.	122.9962	137.0535	275.7609	70.67191	153.9404	191.837	175.0341	113.9013	408.5507	319.9129	225.2892	137.0917	182.999	161.9453	183.8651	82.44518	76.97822	138.3403	146.9945
AC010210.	245.9923	389.1519	484.5798	412.5992	315.6332	372.2552	510.6578	513.019	385.205	357.3801	425.2785	387.3529	348.5206	407.6555	371.6422	362.7588	302.781	597.0475	481.3174
AC010223.	14.41361	8.003124	2.997401	22.86444	7.752394	4.567548	15.29424	7.408216	0	0	0	17.48149	19.5336	13.9608	9.780058	26.38246	16.42202	5.4608	17.02985
AC010226.	97.05166	91.03553	136.8813	75.86837	104.1036	131.317	118.1056	82.41641	100.3867	141.2228	113.247	88.32751	106.9208	59.5661	121.2727	65.95615	75.95184	71.90054	93.21603
AC010230.	6.726353	0	12.98874	11.43222	8.859879	3.425661	8.496802	3.704108	0	10.56769	3.614265	2.760235	5.140422	4.653602	6.846041	4.122259	0	1.820267	7.170464
AC010234.	0	0	0.999134	0	0	0	0	0.926027	0	0.960699	1.204755	0	0	0	0.978006	1.648904	0	0.910133	0.896308
AC010235.	0	0	0	0	0	0	0	0	0	5.764196	4.81902	3.680313	0	2.792161	0.978006	0	0	0	0
AC010240.	0	0	0	1.039293	2.21497	0	0	0	1.167288	0	0	1.840156	0	0	0	0	4.105505	0.910133	0
AC010240.	8.648168	4.001562	4.995668	3.117878	1.107485	9.135096	0	0.926027	11.67288	21.13538	6.023774	3.680313	10.28084	11.16864	0.978006	0.824452	5.131881	3.640534	4.48154
AC010240.	5.765445	4.001562	7.993069	4.157171	5.537425	0	8.496802	5.556162	11.67288	0.960699	2.40951	7.360626	8.224675	3.722881	21.51613	8.244518	1.026376	7.281067	10.7557
AC010245.	316.1386	359.1402	406.6474	314.9057	454.0688	516.1329	256.6034	447.2711	412.0526	445.7645	521.6589	396.5537	414.318	300.6227	281.6657	331.4296	304.8337	314.9062	280.5444
AC010247.	146.0579	48.01874	31.97228	58.2004	60.91167	44.53359	39.08529	79.63833	77.04099	30.74238	55.41872	88.32751	60.65698	55.84322	31.29619	47.81821	30.79129	77.36134	52.88217
AC010247.	1108.887	1607.627	811.2965	651.6366	995.6289	1139.603	1151.317	938.0654	2340.412	1461.224	1660.152	1180.46	1179.213	1291.84	1248.913	806.3139	1017.139	920.1449	1194.778
AC010255.	0.960908	0	5.994802	5.196464	0	1.141887	5.098081	2.778081	0	3.842797	0	0	0	0.93072	2.934017	0	0	0	1.792616
AC010255.	0	0	0	0	2.21497	0	1.69936	5.556162	1.167288	2.882098	0	1.840156	1.028084	0	0.978006	0	0	0	1.792616
AC010255.	2.882723	5.001952	4.995668	1.039293	0	1.141887	4.248401	9.26027	1.167288	1.921399	1.204755	0.920078	10.28084	0.93072	2.934017	6.595615	0	7.281067	5.377848
AC010260.	108.5826	142.0554	180.8432	118.4794	115.1784	99.34417	195.4265	186.1314	38.5205	120.0874	154.2086	173.8948	115.1454	124.7165	176.041	103.8809	79.03097	97.38428	101.2828
AC010261.	11.53089	10.0039	29.97401	19.74656	15.50479	1.141887	16.9936	22.22465	16.34203	6.724895	12.04755	24.84211	12.33701	26.99089	19.56012	11.54233	15.39564	17.29253	26.88924
AC010261.	35.55358	40.01562	41.96361	40.53242	40.97694	41.10793	45.88273	31.48492	28.01491	33.62447	13.2523	55.20469	35.98295	53.05106	52.81231	56.88718	40.02867	25.48374	46.60801
AC010265.	0	0	0	0	0	0	0	0	0	1.167288	0.960699	0	0	0	0	1.648904	0	0	0
AC010266.	10.56998	8.003124	27.97574	7.275049	39.86946	22.83774	14.44456	40.74519	1.167288	18.25329	15.66181	42.3236	34.95487	6.515042	7.824047	9.893422	11.29014	3.640534	7.170464
AC010267.	0	0	0.999134	0	0	1.141887	0	0	0	0	0	0	0	0	0.978006	0	0	0.910133	0
AC010271.	52.84991	25.00976	30.97314	77.94696	22.1497	46.81737	54.37954	33.33697	21.01118	22.09608	27.70936	26.68227	45.23571	34.43665	20.53812	108.0032	74.92547	52.78774	43.91909
AC010271.	17.29634	6.002343	0.999134	1.039293	1.107485	10.27698	0	5.556162	14.00745	17.29259	4.81902	4.600391	7.196591	7.445762	1.956012	3.297807	7.184634	0	5.377848
AC010273.	19.21815	21.0082	21.98094	13.51081	27.68712	26.2634	17.84329	11.11232	39.68778	5.764196	3.614265	10.12086	14.39318	24.19873	15.64809	4.946711	12.31652	24.5736	11.652
AC010273.	24.02269	53.02069	21.98094	19.74656	9.967364	9.135096	14.44456	18.52054	4.669151	33.62447	3.614265	23.00196	8.224675	15.82225	24.45015	32.97807	33.87042	25.48374	7.170464
AC010280.	0	0	0	1.039293	0	4.567548	0	0	2.334575	0	0	0.920078	0	0	0	6.595615	1.026376	0	0
AC010280.	15.37452	7.002733	6.993936	16.62868	1.107485	11.41887	9.346483	10.1863	21.01118	0	16.86657	5.520469	10.28084	3.722881	3.912023	4.122259	12.31652	30.0344	7.170464
AC010280.	0	0	0	0	0	3.425661	0	0	0	0	0	0	1.028084	0	0	0	0	0	0
AC010285.	0	4.001562	0	4.157171	1.107485	4.567548	1.69936	0	0	0	21.68559	0.920078	1.028084	0	4.890029	0	0	1.820267	3.585232
AC010285.	30.74904	38.01484	96.91597	34.29666	45.40688	41.10793	62.87634	75.93422	7.003726	20.17468	7.228529	82.80704	83.27483	51.18962	32.27419	39.57369	12.31652	40.956	50.19325
AC010285.	0.960908	4.001562	0.999134	4.157171	1.107485	18.27019	0.84968	0	31.51677	5.764196	2.40951	3.680313	6.168506	1.861441	0.978006	4.946711	4.105505	1.820267	5.377848
AC010287.	0	0	5.994802	0	1.107485	1.141887	0.84968	0.926027	0	3.842797	0	0.920078	0	0	0.978006	3.297807	0	0	5.377848
AC010300.	463.1574	469.1831	414.6405	896.9097	606.9017	806.1722	605.822	449.1231	1277.013	511.092	644.5439	503.2828	484.2277	432.7849	420.5425	257.229	419.7879	531.5179	517.1697
AC010301.	5.765445	1.00039	0	0	1.107485	0	0.84968	0	0	0	0	0	4.112337	0	0.978006	0	0	0.910133	0
AC010306.	11.53089	10.0039	21.98094	3.117878	18.82724	11.41887	13.59488	7.408216	3.501863	0.960699	2.40951	27.60235	13.3651	9.307203	6.846041	4.946711	8.21101	4.550667	7.170464
AC010307.	0	4.001562	0	0	0	0	0	0	0	0	0	0	1.028084	3.722881	0	0	0	0.910133	0
AC010307.	3.84363	14.00547	9.991337	4.157171	16.61227	3.425661	7.647122	0	3.501863	0.960699	0	9.200782	12.33701	3.722881	4.890029	8.244518	4.105505	5.4608	8.963079
AC010307.	0	0	0	0	0	1.141887	0	0	0	0	4.81902	0	0	3.722881	0	0	1.026376	3.640534	0
AC010307.	0	4.001562	0	12.47151	8.859879	7.993209	0.84968	0	2.334575	0	14.45706	7.360626	8.224675	0	7.824047	1.648904	8.21101	3.640534	2.688924
AC010319.	251.7578	201.0785	124.8917	236.9588	66.4491	153.0129	131.7004	198.1698	54.86252	112.4018	89.15186	133.4113	186.0833	164.7375	221.0293	240.7399	195.0115	125.5984	95.00864
AC010319.	97.05166	88.03436	101.9116	87.30059	101.8886	73.08077	64.5757	78.7123	227.6211	123.9302	125.2945	80.96688	105.8927	64.2197	67.4824	90.6897	63.63533	78.27147	60.05263
AC010319.	31.70995	8.003124	30.97314	15.58939	45.40688	29.68906	22.09169	22.22465	23.34575	11.52839	8.433284	13.80117	16.44935	6.515042	11.73607	16.48904	10.26376	15.47227	4.48154
AC010320.	14.41361	9.003514	2.997401	6.235757	4.42994	9.135096	11.04584	6.482189	25.68033	7.685594	6.023774	7.360626	4.112337	9.307203	19.56012	7.420066	4.105505	8.191201	14.34093

AC010320.	15.37452	29.01132	16.98527	3.117878	8.859879	14.84453	32.28785	10.1863	19.84389	8.646293	4.81902	18.40156	14.39318	11.16864	14.67009	19.78684	5.131881	9.101334	10.7557
AC010320.	33.63176	37.01445	37.96708	35.33595	118.5009	30.83095	39.08529	29.63287	29.18219	27.86028	44.57593	27.60235	28.78636	58.63538	40.09824	41.22259	32.84404	55.51814	58.26002
AC010320.	8.648168	18.00703	17.98441	6.235757	19.93473	1.141887	10.19616	22.22465	0	3.842797	16.86657	15.64133	17.47743	8.376483	20.53812	11.54233	23.60665	8.191201	7.170464
AC010320.	3.84363	0	0.999134	0	0	0	0.84968	0	0	0	0	0	0	0	0	0	1.026376	2.7304	0.896308
AC010323.	1.921815	0	2.997401	4.157171	0	2.283774	3.398721	0	0	2.882098	2.40951	0	6.168506	1.861441	0	2.473355	0	3.640534	1.792616
AC010323.	22.10087	21.0082	28.97488	25.98232	5.537425	51.38492	27.18977	34.263	0	20.17468	15.66181	48.76415	24.67402	26.99089	20.53812	35.45143	22.58028	39.13574	29.57816
AC010325.	1.921815	9.003514	18.98354	9.353635	15.50479	17.12831	3.398721	66.67395	9.338302	20.17468	8.433284	23.92203	22.61786	8.376483	7.824047	12.36678	20.52753	12.74187	18.82247
AC010325.	0	5.001952	0.999134	0	1.107485	0	2.549041	10.1863	0	0.960699	0	0.920078	0	0	0	4.946711	2.052753	0.910133	3.585232
AC010325.	1.921815	21.0082	9.991337	9.353635	18.82724	6.851322	28.03945	27.78081	4.669151	10.56769	6.023774	18.40156	10.28084	12.09936	11.73607	18.96239	11.29014	8.191201	15.23724
AC010326.	14.41361	13.00508	22.98007	1.039293	23.25718	19.41208	4.248401	3.704108	32.68406	8.646293	10.84279	10.12086	27.75828	14.89152	12.71408	19.78684	14.36927	23.66347	9.859387
AC010326.	660.1435	481.1878	672.417	470.7996	653.4161	574.3692	466.4745	600.0655	562.6327	823.3193	675.8675	524.4446	669.2829	478.3902	627.8797	626.5834	656.8808	522.4166	366.59
AC010326.	351.6922	431.1683	473.5894	346.0845	347.7503	337.9986	306.7346	360.2245	305.8294	500.5243	524.0684	399.314	392.7282	330.4057	359.9061	474.8843	405.4186	293.063	243.7958
AC010327.	24.02269	16.00625	21.98094	21.82515	16.61227	7.993209	4.248401	21.29862	0	16.33189	13.2523	8.280704	6.168506	20.47585	9.780058	5.771163	28.73854	24.5736	13.44462
AC010327.	27.86632	52.0203	10.99047	29.1002	19.93473	34.25661	64.5757	9.26027	5.836439	24.01748	7.228529	32.20274	32.8987	5.584322	21.51613	37.10033	67.74083	20.93307	17.92616
AC010327.	49.00628	53.02069	51.95495	70.67191	42.08443	63.94567	53.52986	53.70957	52.52795	51.87776	39.75691	57.96493	52.4323	97.72563	87.04252	63.48279	73.89909	77.36134	85.14926
AC010327.	20.17906	29.01132	12.98874	7.275049	50.94431	9.135096	8.496802	29.63287	59.53167	39.38867	90.35662	26.68227	24.67402	26.06017	32.27419	29.68027	32.84404	35.4952	34.0597
AC010328.	12.4918	1.00039	5.994802	0	17.71976	5.709435	4.248401	0	0	0	0	5.520469	11.30893	8.376483	11.73607	0	1.026376	8.191201	16.13354
AC010328.	4.804538	8.003124	0	1.039293	1.107485	0	0	0	1.167288	0.960699	2.40951	0.920078	1.028084	0	0	0.824452	2.052753	0	0
AC010328.	0	0	0	0	8.859879	0	0.84968	0	0	0	0	0.920078	3.084253	0	0.978006	0	0	0.910133	0.896308
AC010329.	0	0	3.996535	1.039293	0	3.425661	0	2.778081	0	0	0	0	0	0.93072	0.978006	0	0	0.910133	0
AC010329.	6.726353	0	9.991337	0	0	0	2.549041	0.926027	3.501863	5.764196	7.228529	2.760235	1.028084	8.376483	0.978006	3.297807	1.026376	1.820267	0
AC010329.	0.960908	1.00039	3.996535	6.235757	0	3.425661	0	1.852054	0	4.803496	0	0	1.028084	0	0.978006	2.473355	7.184634	1.820267	0.896308
AC010331.	0.960908	1.00039	3.996535	0	1.107485	1.141887	0	0.926027	5.836439	11.52839	46.98544	1.840156	8.224675	0.93072	4.890029	0	1.026376	1.820267	0
AC010332.	85.52077	91.03553	81.92896	49.88605	68.66407	81.07398	86.66738	97.23284	46.69151	114.3232	162.6419	113.1696	127.4825	72.59618	85.08651	78.32292	76.97822	100.1147	69.01571
AC010332.	5.765445	2.000781	8.992203	4.157171	5.537425	4.567548	9.346483	2.778081	1.167288	11.52839	15.66181	6.440548	11.30893	15.82225	3.912023	9.893422	7.184634	10.01147	2.688924
AC010333.	20.17906	3.001171	25.97748	4.157171	5.537425	0	24.64073	5.556162	0	3.842797	2.40951	23.92203	7.196591	13.03008	6.846041	12.36678	8.21101	2.7304	14.34093
AC010333.	4.804538	12.00469	29.97401	8.314342	17.71976	1.141887	11.89552	0.926027	0	3.842797	4.81902	14.72125	13.3651	5.584322	1.956012	11.54233	16.42202	10.01147	1.792616
AC010333.	4.804538	1.00039	0	2.078586	0	0	0.84968	2.778081	19.84389	0	1.204755	0	1.028084	0	1.956012	1.648904	0	0	0
AC010335.	12.4918	13.00508	3.996535	9.353635	9.967364	13.70264	14.44456	4.630135	7.003726	4.803496	8.433284	18.40156	20.56169	11.16864	9.780058	23.9091	24.63303	20.02294	16.13354
AC010335.	0	0	0	0	0	0	0	0	0	2.882098	0	0	0	0	0	0	0	0	0
AC010336.	6.726353	19.00742	5.994802	15.58939	8.859879	23.97963	14.44456	5.556162	5.836439	22.09608	15.66181	20.24172	10.28084	12.09936	1.956012	0.824452	1.026376	7.281067	13.44462
AC010336.	3.84363	4.001562	0.999134	7.275049	5.537425	3.425661	5.947762	11.11232	7.003726	25.93888	14.45706	19.32164	17.47743	26.99089	18.58211	6.595615	13.34289	15.47227	16.13354
AC010336.	0	0	0	7.275049	11.07485	27.40529	0	23.15068	10.50559	0.960699	0	5.520469	3.084253	11.16864	7.824047	16.48904	14.36927	0.910133	0.896308
AC010342.	17.29634	19.00742	15.98614	23.90373	9.967364	9.135096	21.24201	17.59451	4.669151	11.52839	10.84279	11.96102	18.50552	21.40657	10.75806	22.2602	45.16056	11.83173	21.51139
AC010343.	27.86632	11.00429	18.98354	12.47151	11.07485	11.41887	11.89552	8.334243	22.17847	16.33189	32.52838	20.24172	17.47743	18.61441	24.45015	18.96239	40.02867	15.47227	16.13354
AC010343.	1.921815	0	0.999134	10.39293	0	5.709435	5.947762	2.778081	0	0.960699	0	7.360626	8.224675	14.89152	12.71408	14.84013	1.026376	0.910133	6.274156
AC010359.	37.47539	33.01288	66.94196	19.74656	60.91167	21.69585	56.92858	24.0767	65.36811	82.62014	121.6802	53.36454	14.39318	28.85233	44.01026	45.34485	27.71216	52.78774	36.74863
AC010368.	0	0	0	0	0	0	0.84968	1.852054	0	0	0	0	0	0	0	0	0	0	0
AC010374.	0	0	0.999134	0	0	0	0	0	0	3.842797	0	0	0	0	0	0	0	0	0
AC010378.	24.9836	22.00859	20.98181	11.43222	26.57964	20.55397	24.64073	41.67122	0	7.685594	4.81902	25.76219	15.42127	13.03008	38.14223	24.73355	7.184634	10.9216	25.99293
AC010385.	1.921815	0	0.999134	1.039293	1.107485	0	5.098081	3.704108	0	1.921399	0	0	0	1.861441	5.868035	4.122259	0	0	0
AC010385.	1.921815	18.00703	0.999134	1.039293	0	0	0.84968	0	0	0	0	0	0	4.653602	0.978006	0.824452	0	0	0
AC010387.	0	0	3.996535	2.078586	0	1.141887	0.84968	0	3.501863	0	0	0.920078	1.028084	0.93072	2.934017	0	10.26376	0	0.896308
AC010395.	5.765445	1.00039	1.998267	3.117878	2.21497	1.141887	3.398721	0.926027	0	3.842797	0	10.12086	8.224675	5.584322	11.73607	0	3.079129	0.910133	6.274156
AC010395.	0	0	0	0	0	0	0	0	0	3.842797	0	0	0	0	0	0	0	0	0
AC010401.	7.68726	6.002343	13.98787	2.078586	8.859879	9.135096	13.59488	0.926027	0	7.685594	2.40951	0.920078	0	3.722881	1.956012	1.648904	0	2.7304	0
AC010401.	5.765445	25.00976	31.97228	14.5501	27.68712	6.851322	25.49041	35.18903	17.50932	10.56769	28.91412	21.1618	37.01104	12.09936	18.58211	15.66458	4.105505	29.12427	18.82247
AC010401.	0	6.002343	0	0	0	1.141887	0	0	0	0	0	0	0	0	0	0.824452	0	0	6.274156
AC010406.	1.921815	2.000781	0	2.078586	0	0	5.098081	1.852054	0	0	0	0	2.056169	6.515042	0.978006	6.595615	0	1.820267	0
AC010409.	0	0	0	0	1.107485	3.425661	7.647122	0	0	0	0	0.920078	0	0	0	0.824452	7.184634	1.820267	4.48154
AC010420.	16.33543	22.00859	64.94369	9.353635	15.50479	15.98642	28.03945	67.59997	12.84017	19.21399	9.638039	22.08188	22.61786	38.15953	32.27419	6.595615	8.21101	8.191201	18.82247
AC010422.	25.9445	19.00742	26.97661	21.82515	9.967364	11.41887	30.58849	37.96711	17.50932	19.21399	48.1902	38.64329	38.03912	12.09936	8.802052	40.39814	35.92317	28.21414	34.0597
AC010422.	45.16265	12.00469	15.98614	7.275049	27.68712	36.54038	23.79105	7.408216	36.18592	32.66377	77.10431	32.20274	30.84253	25.12945	39.12023	27.20691	27.71216	11.83173	18.82247
AC010422.	2.882723	8.003124	1.998267	1.039293	0	2.283774	3.398721	4.630135	7.003726	5.764196	8.433284	3.680313	3.084253	2.792161	3.912023	2.473355	0	0.910133	2.688924
AC010422.	49.00628	37.01445	47.95842	5.196464	12.18233	45.67548	16.9936	60.19176	44.35693	62.44545	38.55216	13.80117	17.47743	60.49682	58.68035	29.68027	43.1078	23.66347	20.61508
AC010422.	182.5724	161.0629	168.8536	194.3477	146.188	204.3978	152.9424	181.5013	117.8961	258.4281	185.5323	161.9338	160.3812	184.2826	193.6452	209.4108	197.0642	170.1949	188.2247
AC010424.	315.1777	362.1413	390.6613	355.4381	301.2359	176.9925	235.3614	276.8821	61.86625	429.4326	340.9456	205.1774	189.1675	225.2343	172.129	154.9969	216.5654	131.0592	162.2317
AC010424.	1.921815	3.001171	10.99047	16.62868	11.07485	7.993209	4.248401	20.37259	1.167288	14.41049	8.433284	5.520469	9.252759	25.12945	9.780058	6.595615	11.29014	0	1.792616
AC010425.	10.56998	32.01249	30.97314	29.1002	42.08443	15.98642	23.79105	27.78081	11.67288	28.82098	13.2523	27.60235	29.81445	10.23792	14.67009	24.73355	25.65941	31.85467	22.4077
AC010435.	9.609075	2.000781	12.98874	16.62868	7.752394	7.993209	16.14392	6.482189	0	0.960699	1.204755	10.12086	5.140422	6.515042	5.868035	0.824452	3.079129	4.550667	2.688924
AC010442.	0	4.001562	0.999134	2.078586	0	0	0.84968	0	0	0	0	0	1.028084	3.722881	0	0	0	0	0
AC010442.	0	0	6.993936	1.039293	11.07485	0	0	0.926027	0	5.764196	0	3.680313	8.224675	0	0.978006	32.15362	43.1078	0.910133	1.792616

AC010445.	5.765445	0	0	0	0	0	0	0	0	0	0	0	0	0	0	0	0	0	0
AC010457.	106.6607	243.0949	230.7999	170.444	202.6697	258.0065	221.7665	179.6492	117.8961	304.5417	296.3697	189.5361	220.0101	164.7375	226.8974	185.5017	164.2202	397.7283	295.7816
AC010460.	0.960908	0	0	0	0	0	0	0	0	0	0	2.760235	0	1.861441	0	0	0	0	0
AC010463.	22.10087	28.01093	3.996535	27.02161	66.4491	1.141887	34.83689	5.556162	23.34575	9.606993	0	17.48149	37.01104	34.43665	15.64809	4.946711	2.052753	10.9216	8.066772
AC010463.	124.918	89.03475	137.8804	138.2259	108.5335	105.0536	116.4062	80.56435	147.0783	86.46293	103.6089	113.1696	99.72418	87.48771	114.4267	98.10977	82.1101	147.4416	152.3724
AC010468.	29.78813	59.02304	35.96881	19.74656	45.40688	49.10114	20.39233	40.74519	47.8588	57.64196	33.73314	22.08188	15.42127	48.39746	55.74633	36.27588	40.02867	43.6864	22.4077
AC010469.	0	4.001562	3.996535	1.039293	3.322455	7.993209	0.84968	0	4.669151	5.764196	8.433284	0	2.056169	0	0	0	0	6.370934	0.896308
AC010469.	0	0	0.999134	0	0	0	0	0	0	0	0	0	1.028084	0	0	0.824452	0	0	0
AC010476.	0	0	1.998267	0	0	0	0.84968	0	0	0	0	0	0	0	0	0	1.026376	0.910133	0
AC010478.	759.1169	974.3803	870.2454	1123.475	576.9996	1273.204	1338.246	720.449	433.0637	701.3105	612.0155	750.7838	865.647	724.1004	705.1422	1103.117	1025.35	859.1659	1104.251
AC010480.	13.45271	10.0039	15.98614	9.353635	2.21497	19.41208	48.43177	5.556162	1.167288	6.724895	16.86657	21.1618	29.81445	5.584322	25.42815	12.36678	7.184634	16.3824	20.61508
AC010487.	473.7274	515.2011	724.3719	612.1434	433.0266	529.8356	531.8998	484.3121	377.0339	536.0702	543.3444	518.004	576.7553	428.1313	477.2668	476.5332	475.2122	547.9003	508.2066
AC010487.	4.804538	11.00429	11.9896	1.039293	1.107485	29.68906	8.496802	8.334243	25.68033	10.56769	13.2523	10.12086	6.168506	11.16864	11.73607	6.595615	10.26376	14.56213	12.54831
AC010487.	6.726353	13.00508	5.994802	3.117878	0	1.141887	3.398721	2.778081	1.167288	4.803496	2.40951	3.680313	5.140422	0.93072	0	0.824452	1.026376	2.7304	2.688924
AC010491.	86.48168	141.0551	262.7722	54.04322	73.094	85.64153	95.16419	133.3479	255.636	136.4193	187.9418	93.84798	173.7463	154.4996	114.4267	61.00944	78.0046	147.4416	123.6905
AC010501.	270.9759	330.1288	196.8293	269.1768	169.4452	320.8702	373.8593	245.3972	231.123	268.9958	189.1465	225.4192	259.0773	241.0566	267.9736	238.2666	254.5413	233.9043	379.1383
AC010503.	0	0	0	0	0	0	0	0.926027	0	0	0	0.920078	0	0	0	0	0	0	0
AC010503.	19.21815	30.01171	33.97054	30.13949	31.00958	61.6619	29.73881	62.96984	33.85134	41.31007	21.68559	35.88305	34.95487	32.57521	32.27419	34.62698	25.65941	27.304	80.66772
AC010504.	121.0743	122.0476	132.8848	85.22201	79.73891	70.79699	87.51706	119.4575	66.5354	81.65944	125.2945	128.811	95.61185	84.69555	106.6026	101.4076	134.4553	85.55254	114.7274
AC010507.	0.960908	1.00039	0	0	1.107485	0	0.84968	0.926027	0	0	2.40951	0	0	0	1.956012	0.824452	1.026376	2.7304	0
AC010511.	96.09075	25.00976	22.98007	94.57564	34.33203	79.93209	64.5757	21.29862	3.501863	21.13538	27.70936	37.72321	54.48847	30.71377	26.40616	70.90286	53.37157	39.13574	69.01571
AC010519.	11.53089	10.0039	2.997401	13.51081	21.04221	9.135096	7.647122	11.11232	18.6766	22.09608	18.07132	14.72125	17.47743	20.47585	9.780058	0.824452	10.26376	4.550667	0.896308
AC010524.	33.63176	17.00664	12.98874	108.0864	48.72934	29.68906	45.88273	26.85478	35.01863	26.89958	43.37118	44.16376	39.06721	33.50593	52.81231	67.60505	93.40024	61.88907	69.91202
AC010525.	9.609075	11.00429	5.994802	1.039293	21.04221	18.27019	7.647122	6.482189	35.01863	20.17468	42.16642	4.600391	6.168506	1.861441	9.780058	15.66458	15.39564	20.02294	14.34093
AC010528.	0	0	0	0	0	0	1.69936	0	0	0	0	0	0	0	0	0	0	0	0
AC010530.	24.02269	28.01093	67.94109	49.88605	14.3973	37.68227	79.02026	71.30408	1.167288	47.07426	46.98544	44.16376	82.24675	62.35826	52.81231	61.00944	62.60895	44.59654	21.51139
AC010531.	0	0	1.998267	0	0	0	0	0	0	0	0	0	0	3.722881	0	0	0	0	0
AC010531.	26.90541	20.00781	42.96275	48.84676	28.79461	17.12831	39.93497	30.55889	3.501863	33.62447	46.98544	36.80313	23.64594	26.06017	35.20821	23.9091	25.65941	28.21414	51.08955
AC010531.	5.765445	2.000781	13.98787	0	2.21497	0	2.549041	2.778081	0	5.764196	4.81902	5.520469	3.084253	1.861441	0	0	7.184634	0.910133	0
AC010531.	20.17906	20.00781	15.98614	81.06484	44.2994	7.993209	6.797442	22.22465	9.338302	6.724895	3.614265	34.04289	42.15146	42.81313	55.74633	46.1693	48.23968	38.2256	50.19325
AC010531.	1.921815	0	4.995668	2.078586	6.64491	1.141887	0.84968	3.704108	4.669151	5.764196	12.04755	6.440548	1.028084	5.584322	2.934017	2.473355	4.105505	4.550667	0
AC010531.	0	0	0	0	0	0	0	0	11.67288	0	0	0	0	0	0	0	0	0	0
AC010533.	43.24084	39.01523	71.93762	19.74656	32.11706	12.56076	24.64073	40.74519	46.69151	17.29259	44.57593	61.64524	94.58376	44.67457	44.98827	41.22259	29.76491	40.04587	21.51139
AC010536.	38.4363	80.03124	39.96535	56.12181	57.58922	69.65511	17.84329	62.96984	102.7213	122.9695	165.0514	82.80704	52.4323	68.8733	57.70234	28.03136	80.05735	45.50667	42.12647
AC010536.	37.47539	27.01054	28.97488	36.37525	52.05179	11.41887	30.58849	75.00819	44.35693	35.54587	50.5997	106.7291	79.1625	40.95169	29.34017	23.9091	33.87042	11.83173	15.23724
AC010536.	2.882723	2.000781	3.996535	2.078586	24.36467	4.567548	0.84968	0	10.50559	0.960699	21.68559	1.840156	2.056169	1.861441	4.890029	9.06897	9.237386	8.191201	4.48154
AC010538.	50.9281	33.01288	22.98007	39.49313	62.01916	66.22945	27.18977	52.78354	86.37929	103.7555	92.76613	39.56336	66.82548	26.06017	54.76833	60.18498	45.16056	40.04587	36.74863
AC010542.	0	4.001562	6.993936	6.235757	3.322455	0	5.098081	0	0	0	0	0.920078	4.112337	5.584322	1.956012	3.297807	5.131881	2.7304	4.48154
AC010542.	4.804538	0	0	0	0	1.141887	1.69936	0	2.334575	0	0	1.840156	0	1.861441	0	2.473355	0	0	0
AC010542.	1.921815	0	3.996535	10.39293	5.537425	5.709435	8.496802	1.852054	2.334575	0	1.204755	5.520469	5.140422	0.93072	0	2.473355	8.21101	3.640534	1.792616
AC010542.	122.9962	125.0488	136.8813	75.86837	109.641	103.9117	115.5565	173.1671	177.4277	175.808	212.0369	157.3334	162.4373	81.90339	134.9648	94.81196	129.3234	154.7227	105.7643
AC010546.	0	0	5.994802	0	0	0	4.248401	0	0	0.960699	0	4.600391	0	0	2.934017	0	0	0	0
AC010547.	79.75532	136.0531	127.8891	58.2004	58.6967	83.35775	84.96802	107.4191	108.5578	122.0088	132.523	85.56728	74.02207	71.66546	90.95454	74.20066	85.18923	71.90054	97.69757
AC010547.	0	0	0	0	0	0	0	5.556162	0	1.921399	0	0	0	0	0	0	1.026376	0	0
AC010547.	0	0	0	0	0	1.141887	1.69936	0	0	0	0	0	0	0.93072	0	0.824452	0	0	0
AC010547.	4.804538	0	0	0	0	0	0	0.926027	0	0	0	0.920078	0	0	0	0	0	0	0
AC010551.	0.960908	0	0.999134	1.039293	0	0	0	0	0	3.842797	1.204755	3.680313	0	0	0.978006	3.297807	0	0	0
AC010551.	7.68726	7.002733	23.97921	51.96464	3.322455	19.41208	16.9936	29.63287	0	1.921399	12.04755	10.12086	3.084253	5.584322	14.67009	50.29156	20.52753	10.9216	9.859387
AC010551.	11.53089	13.00508	12.98874	32.21808	5.537425	28.54718	19.54265	10.1863	21.01118	14.41049	13.2523	13.80117	10.28084	13.03008	15.64809	57.71163	35.92317	1.820267	8.066772
AC010598.	16.33543	17.00664	6.993936	16.62868	28.79461	12.56076	9.346483	23.15068	10.50559	7.685594	10.84279	12.8811	23.64594	7.445762	14.67009	8.244518	27.71216	9.101334	10.7557
AC010601.	11.53089	6.002343	4.995668	5.196464	0	1.141887	0.84968	0	0	1.921399	3.614265	11.04094	0	7.445762	4.890029	5.771163	9.237386	12.74187	0
AC010605.	0.960908	4.001562	11.9896	0	7.752394	6.851322	4.248401	20.37259	0	14.41049	0	0.920078	5.140422	4.653602	6.846041	0	6.158258	0.910133	5.377848
AC010608.	0.960908	1.00039	1.998267	0	0	0	0.84968	0	0	0.960699	0	0.920078	0	0	0	0	0	0	0
AC010608.	14.41361	29.01132	16.98527	3.117878	9.967364	4.567548	4.248401	9.26027	23.34575	6.724895	7.228529	15.64133	2.056169	15.82225	10.75806	2.473355	7.184634	6.370934	8.066772
AC010609.	0.960908	2.000781	0.999134	1.039293	6.64491	0	4.248401	11.11232	1.167288	0	0	0	7.196591	0.93072	0.978006	0	0	3.640534	0
AC010615.	226.7742	211.0824	207.8198	445.8566	306.7733	286.6136	311.8326	410.23	96.88488	262.2709	134.9325	174.8149	170.662	228.0265	222.9853	283.6114	227.8555	303.9846	236.6253
AC010615.	199.8688	221.0863	190.8345	218.2515	120.7159	226.0936	271.048	139.8301	186.766	138.3407	159.0276	338.5888	301.2287	229.8879	234.7214	258.8779	253.5149	143.8011	184.6394
AC010615.	0	0	2.997401	0	0	3.425661	0	9.26027	0	29.78168	19.27608	0	0	0.93072	5.868035	5.771163	2.052753	1.820267	8.963079
AC010616.	62.45899	34.01327	32.97141	22.86444	70.87903	23.97963	40.78465	31.48492	117.8961	55.72056	127.704	57.96493	41.12337	28.85233	47.92229	29.68027	34.89679	27.304	56.4674
AC010618.	8.648168	14.00547	3.996535	1.039293	0	14.84453	1.69936	4.630135	19.84389	1.921399	9.638039	12.8811	2.056169	0	13.69208	4.122259	10.26376	10.01147	3.585232
AC010618.	32.67086	35.01367	54.95235	24.94303	68.66407	28.54718	27.18977	34.263	53.69524	77.81664	66.26152	51.52438	51.40422	67.01186	28.36217	48.64266	33.87042	52.78774	26.88924
AC010618.	741.8206	751.2932	1068.074	609.0256	987.8766	757.0711	584.58	1014	902.3134	1005.852	940.9136	924.6786	973.5959	822.7567	823.4809	630.7056	488.5551	826.4011	701.8091

AC010619.	0	2.000781	1.998267	2.078586	2.21497	2.283774	0	10.1863	10.50559	13.44979	26.50461	11.04094	3.084253	0.93072	0.978006	5.771163	2.052753	2.7304	8.963079
AC010620.	0	5.001952	1.998267	0	5.537425	7.993209	0	19.44657	1.167288	2.882098	2.40951	0.920078	5.140422	1.861441	0	2.473355	8.21101	1.820267	0.896308
AC010620.	0.960908	0	0	2.078586	0	0	2.549041	2.778081	0	0.960699	0	0	2.056169	0	0	0	1.026376	3.640534	2.688924
AC010624.	14.41361	29.01132	56.95062	33.25737	58.6967	39.96605	38.23561	25.00273	36.18592	53.79916	60.23774	27.60235	39.06721	40.02097	55.74633	23.9091	30.79129	51.8776	60.05263
AC010624.	89.3644	201.0785	119.896	72.75049	162.8003	188.4114	129.1514	79.63833	449.4058	192.1399	351.7884	73.60626	62.71315	120.9936	112.4707	52.76492	138.5608	101.9349	170.2985
AC010624.	8.648168	9.003514	3.996535	17.66798	13.28982	18.27019	11.89552	3.704108	19.84389	5.764196	2.40951	4.600391	16.44935	13.03008	8.802052	13.19123	1.026376	24.5736	14.34093
AC010624.	2.882723	1.00039	5.994802	9.353635	0	1.141887	2.549041	0	0	6.724895	6.023774	8.280704	6.168506	7.445762	0	1.648904	5.131881	20.02294	25.99293
AC010627.	25.9445	18.00703	27.97574	60.27898	15.50479	13.70264	18.69297	19.44657	3.501863	9.606993	12.04755	18.40156	19.5336	9.307203	29.34017	56.06272	26.68578	30.0344	42.12647
AC010631.	0	0	0	0	0	0	2.549041	0	0	0	0	0	0	0	0	0	0	0	0
AC010632.	6.726353	8.003124	11.9896	1.039293	12.18233	5.709435	11.89552	2.778081	4.669151	3.842797	3.614265	4.600391	0	8.376483	5.868035	1.648904	1.026376	7.281067	5.377848
AC010632.	12.4918	19.00742	27.97574	21.82515	16.61227	10.27698	28.03945	19.44657	33.85134	38.42797	49.39495	13.80117	23.64594	32.57521	16.6261	23.9091	16.42202	14.56213	25.99293
AC010632.	0	0	0	0	0	0	0	0	0	0	0	0	0	0	0	0	0	3.640534	0
AC010636.	0	1.00039	0	2.078586	0	1.141887	0	0	0	0	0	0	4.112337	0	0	0	0	0	0
AC010636.	0	1.00039	0	0	1.107485	3.425661	0	0	0	0	0	1.840156	0	0	0	0	0	0.910133	0.896308
AC010641.	4.804538	5.001952	21.98094	7.275049	0	1.141887	7.647122	6.482189	0	7.685594	27.70936	0.920078	9.252759	14.89152	10.75806	9.893422	15.39564	5.4608	14.34093
AC010642.	25.9445	17.00664	74.93502	25.98232	19.93473	35.3985	28.03945	27.78081	1.167288	47.07426	44.57593	28.52243	19.5336	20.47585	19.56012	29.68027	37.97592	20.93307	17.92616
AC010642.	485.2583	463.1808	571.5045	514.4499	503.9056	623.4703	544.645	495.4245	636.1718	684.0179	636.1106	493.1619	484.2277	445.815	510.519	488.0755	510.109	532.428	496.5546
AC010643.	124.918	73.0285	54.95235	108.0864	135.1132	50.24303	66.27506	17.59451	60.69896	103.7555	74.6948	39.56336	31.87062	48.39746	44.01026	103.8809	109.8223	15.47227	42.12647
AC010650.	18.25724	27.01054	17.98441	18.70727	23.25718	5.709435	16.14392	12.96438	25.68033	29.78168	10.84279	10.12086	13.3651	11.16864	6.846041	3.297807	11.29014	11.83173	7.170464
AC010653.	44.20175	37.01445	40.96448	38.45383	44.2994	37.68227	53.52986	49.07943	45.52422	40.34937	46.98544	42.3236	40.09529	32.57521	53.79032	51.94047	21.5539	47.32694	24.20031
AC010653.	0	0	0	0	0	0	0	3.704108	0	0	0	0	0	0	0.978006	0	0	0	0
AC010653.	5.765445	10.0039	1.998267	0	0	1.141887	0	3.704108	7.003726	20.17468	22.89034	5.520469	6.168506	4.653602	0	0	5.131881	5.4608	0.896308
AC010654.	28.82723	67.02616	84.92636	31.17878	80.8464	54.81058	50.13113	57.41368	130.7362	61.48475	131.3183	61.64524	69.90974	77.24979	64.54838	18.13794	46.18693	40.956	97.69757
AC010655.	0	5.001952	3.996535	0	0	1.141887	0.84968	4.630135	10.50559	0	7.228529	0.920078	0	0.93072	0	0	4.105505	4.550667	0
AC010655.	29.78813	21.0082	21.98094	29.1002	42.08443	30.83095	21.24201	37.96711	31.51677	29.78168	32.52838	39.56336	24.67402	50.2589	30.31818	78.32292	58.50345	50.96747	39.43755
AC010677.	0	0	0.999134	0	0	0	0	0	0	0.960699	0	0.920078	0	0	0.978006	0	0	0	0
AC010680.	11.53089	26.01015	8.992203	42.611	21.04221	10.27698	45.03305	41.67122	12.84017	21.13538	0	27.60235	25.70211	22.33729	21.51613	11.54233	11.29014	4.550667	17.92616
AC010680.	21.13997	25.00976	22.98007	42.611	29.90209	12.56076	20.39233	40.74519	21.01118	21.13538	31.32363	21.1618	21.58977	23.26801	27.38416	26.38246	24.63303	22.75334	25.99293
AC010680.	0.960908	0	0.999134	1.039293	9.967364	0	1.69936	0.926027	0	0	2.40951	1.840156	1.028084	0	0	0	0	0.910133	2.688924
AC010680.	69.18534	48.01874	48.95755	101.8507	66.4491	65.08756	65.42538	54.6356	31.51677	48.99566	43.37118	40.48344	39.06721	55.84322	57.70234	42.04704	43.1078	33.67494	41.23017
AC010683.	0	0	0.999134	1.039293	0	0	0	0	0	0	0	0	0	0	0	0	0	0	0
AC010719.	41.31902	44.01718	69.93936	38.45383	86.38382	31.97284	33.13753	63.89587	159.9184	115.2839	98.7899	45.08383	81.21867	108.8943	81.17448	75.02512	41.05505	60.97894	34.0597
AC010722.	5.765445	3.001171	7.993069	7.275049	4.42994	0	0	0	0	17.29259	0	1.840156	0	0	0	0.824452	3.079129	3.640534	7.170464
AC010729.	3.84363	7.002733	15.98614	17.66798	0	14.84453	22.09169	4.630135	9.338302	14.41049	39.75691	22.08188	20.56169	5.584322	5.868035	23.9091	17.4484	1.820267	6.274156
AC010731.	0	0	0	5.196464	0	0	3.398721	0	0	0.960699	0	0.920078	0	0	0.978006	0	0	0	0
AC010731.	52.84991	105.041	96.91597	136.1474	163.9078	86.78341	270.1983	143.5342	43.18965	79.73804	87.94711	246.581	171.6901	121.9244	119.3167	115.4233	101.6112	165.6443	259.033
AC010731.	3.84363	1.00039	5.994802	0	0	9.135096	0.84968	0	0	1.921399	0	2.760235	2.056169	0	5.868035	3.297807	1.026376	0	0.896308
AC010733.	106.6607	79.03084	124.8917	193.3085	138.4356	103.9117	129.1514	119.4575	24.51304	51.87776	48.1902	102.1287	83.27483	81.90339	96.82258	173.1349	131.3762	305.8048	345.0786
AC010735.	4.804538	5.001952	10.99047	7.275049	7.752394	2.283774	6.797442	8.334243	0	4.803496	4.81902	12.8811	13.3651	3.722881	5.868035	9.06897	7.184634	5.4608	7.170464
AC010735.	34.59267	52.0203	11.9896	46.76817	50.94431	21.69585	56.92858	13.89041	61.86625	12.48909	22.89034	42.3236	43.17954	40.02097	25.42815	15.66458	18.47477	32.7648	32.26709
AC010737.	78.79442	54.02108	63.94455	54.04322	119.6084	0	0	0	0	36.50657	15.66181	0	0	0	0	0	0	0	0
AC010740.	0	0	0.999134	0	1.107485	0	0	0.926027	0	0	0	1.840156	2.056169	7.445762	5.868035	0	0	0.910133	0
AC010746.	5.765445	3.001171	0.999134	5.196464	1.107485	2.283774	0	0	0	0	4.81902	0.920078	1.028084	0	0	0	4.105505	0	0.896308
AC010754.	117.2307	49.01913	126.89	102.89	90.81376	26.2634	58.62794	43.52327	17.50932	29.78168	37.3474	45.08383	84.30292	67.01186	59.65836	116.2477	118.0333	61.88907	95.90495
AC010760.	0.960908	0	0	0	0	0	0	0	0	0	0	1.840156	1.028084	0	0	0	0	0	0
AC010761.	222.9305	150.0586	171.851	231.7623	303.4509	190.6951	204.7729	214.8383	316.335	297.8168	157.8229	200.5771	158.325	197.3127	251.3475	322.3607	249.4094	354.0419	248.2773
AC010761.	0	0	5.994802	0	0	0	0	0	0	0	3.614265	0	0	0	0	0	0	0	0
AC010761.	73.98988	44.01718	95.91683	169.4047	104.1036	27.40529	135.0992	41.67122	32.68406	67.24895	42.16642	87.40743	112.0612	60.49682	77.26246	157.4703	135.4817	126.5085	98.59387
AC010761.	4.804538	6.002343	2.997401	0	7.752394	10.27698	6.797442	3.704108	5.836439	23.05678	14.45706	10.12086	2.056169	5.584322	0.978006	9.893422	3.079129	7.281067	23.30401
AC010761.	3.84363	1.00039	4.995668	0	12.18233	0	0.84968	5.556162	3.501863	2.882098	0	0	0	0	0	4.122259	2.052753	0.910133	9.859387
AC010768.	1.921815	8.003124	3.996535	3.117878	7.752394	11.41887	7.647122	12.03835	0	25.93888	10.84279	1.840156	3.084253	0.93072	3.912023	3.297807	2.052753	3.640534	0.896308
AC010768.	2.882723	16.00625	25.97748	4.157171	8.859879	5.709435	12.7452	6.482189	0	4.803496	1.204755	17.48149	10.28084	5.584322	8.802052	9.06897	8.21101	5.4608	7.170464
AC010768.	0	3.001171	0.999134	0	0	2.283774	8.496802	0	0	0.960699	0	0	0	1.861441	0	0	0	0	0.896308
AC010776.	0	0	0.999134	0	0	0	0	0	0	0.960699	0	0.920078	0	0	0	0	0	0	0
AC010776.	0	0	0	0	1.107485	0	0	0	0	0	0	0	0	0	0	0	4.105505	0	0
AC010789.	0	1.00039	0.999134	0	4.42994	0	1.69936	0	0	0	0	1.840156	0	0	1.956012	3.297807	1.026376	1.820267	0
AC010809.	149.9016	86.03358	299.7401	161.0904	98.56616	214.6748	238.7601	102.789	65.36811	110.4804	138.5468	161.9338	131.5948	144.2616	132.0308	109.6521	144.7191	172.9253	250.9662
AC010809.	229.6569	116.0453	379.6708	189.1513	125.1458	211.2491	269.3486	143.5342	128.4017	182.5329	114.4517	157.3334	136.7352	157.2917	205.3812	145.928	158.0619	200.2294	252.7588
AC010809.	31.70995	33.01288	60.94715	35.33595	43.19191	42.24982	56.0789	25.00273	71.20455	34.58517	20.48083	26.68227	42.15146	42.81313	42.05425	22.2602	26.68578	22.75334	28.68185
AC010834.	13.45271	23.00898	8.992203	14.5501	33.22455	14.84453	26.34009	0	24.51304	19.21399	18.07132	2.760235	9.252759	13.03008	11.73607	4.122259	15.39564	12.74187	21.51139
AC010834.	602.489	600.2343	793.3121	663.0688	479.541	601.7745	802.9478	776.9367	180.9296	536.0702	402.3881	778.3862	693.957	555.64	555.5073	573.8185	463.9221	664.3974	702.7054
AC010857.	10.56998	47.01835	5.994802	28.0609	16.61227	41.10793	21.24201	17.59451	47.8588	24.01748	9.638039	16.56141	14.39318	26.06017	44.98827	21.43575	24.63303	23.66347	22.4077

AC010864.	56.69354	89.03475	98.91423	38.45383	83.06137	43.39171	46.73241	57.41368	129.5689	83.58083	97.58515	66.24563	61.68506	75.38834	72.37243	32.97807	71.84634	89.19307	124.5868
AC010867.	0	0	0	1.039293	0	0	1.69936	0	0	0	0	0	0	1.861441	0	0	4.105505	0	0
AC010878.	0	4.001562	4.995668	8.314342	2.21497	0	4.248401	0.926027	0	0	0	0.920078	1.028084	2.792161	2.934017	6.595615	0	1.820267	3.585232
AC010880.	0	0	0	0	0	4.567548	0	0	0	0	0	0	0	0	3.912023	0	1.026376	0	5.377848
AC010883.	49.96719	48.01874	95.91683	40.53242	37.65449	39.96605	58.62794	70.37805	217.1155	94.14853	132.523	44.16376	59.62889	66.08114	81.17448	45.34485	58.50345	86.46267	49.29694
AC010884.	84.55986	100.039	55.95149	112.2436	55.37425	68.51322	121.5043	100.0109	22.17847	47.07426	13.2523	69.00587	66.82548	88.41843	71.39443	98.10977	100.5849	59.15867	63.63786
AC010886.	0	0	0.999134	6.235757	2.21497	0	0.84968	5.556162	5.836439	0	0	5.520469	0	0.93072	0	16.48904	5.131881	4.550667	0
AC010889.	646.6908	548.214	628.4551	627.7328	383.1898	0	0	0	0	445.7645	342.1504	0	0	0	0	0	0	0	0
AC010890.	109.5435	92.03592	96.91597	94.57564	145.0805	78.7902	57.77826	183.3534	77.04099	137.38	116.8612	133.4113	144.9599	109.825	95.84457	109.6521	86.21561	134.6997	150.5797
AC010891.	1.921815	0	0.999134	2.078586	0	0	0	0	0	2.882098	0	0	0	0	0	0	0	0	0
AC010894.	147.0189	201.0785	121.8943	181.8762	149.5105	188.4114	184.3806	163.9068	212.4464	181.5722	151.7991	174.8149	112.0612	147.0538	159.4149	159.1192	145.7454	163.824	130.861
AC010894.	109.5435	75.02928	151.8683	269.1768	148.403	178.1344	166.5373	152.7945	176.2604	189.2578	119.2707	137.0917	150.1003	253.1559	186.7991	279.4892	203.2225	90.10321	121.8979
AC010894.	12.4918	22.00859	20.98181	28.0609	29.90209	15.98642	39.93497	15.74246	10.50559	15.37119	27.70936	21.1618	24.67402	16.75297	31.29619	16.48904	22.58028	23.66347	24.20031
AC010894.	5.765445	16.00625	23.97921	13.51081	28.79461	17.12831	16.14392	37.04108	16.34203	6.724895	4.81902	11.96102	9.252759	27.92161	16.6261	8.244518	1.026376	6.370934	1.792616
AC010900.	13.45271	2.000781	2.997401	1.039293	11.07485	13.70264	4.248401	41.67122	14.00745	28.82098	7.228529	9.200782	4.112337	12.09936	2.934017	16.48904	5.131881	0	7.170464
AC010900.	0	1.00039	0	0	0	1.141887	0.84968	0	0	1.921399	1.204755	0.920078	0	0	0.978006	0	0	0	0
AC010904.	0	0	0	0	0	0	0	2.778081	0	0	0	0	0	0	0	0	0	0	0
AC010904.	22.10087	15.00586	10.99047	11.43222	9.967364	19.41208	28.88913	14.81643	53.69524	17.29259	20.48083	0	11.30893	3.722881	7.824047	17.31349	11.29014	1.820267	14.34093
AC010907.	129.7225	165.0644	172.8501	36.37525	150.6179	76.50643	169.936	128.7178	185.5987	116.2446	69.87578	110.4094	112.0612	319.2371	239.6114	40.39814	42.08143	62.79921	56.4674
AC010907.	0	3.001171	0	0	0	0	0	1.852054	5.836439	0	0	1.840156	2.056169	0	0	0	0	0	3.585232
AC010913.	31.70995	39.01523	17.98441	39.49313	5.537425	12.56076	33.98721	15.74246	45.52422	34.58517	14.45706	36.80313	39.06721	51.18962	31.29619	54.41382	71.84634	44.59654	48.40063
AC010928.	17.29634	12.00469	0	0	5.537425	0	12.7452	0	0	6.724895	0	5.520469	4.112337	3.722881	0.978006	1.648904	11.29014	3.640534	9.859387
AC010928.	0.960908	0	0	0	1.107485	0	15.29424	0	0	0	0	2.760235	4.112337	0.93072	0.978006	0.824452	7.184634	2.7304	0.896308
AC010931.	0.960908	3.001171	1.998267	0	1.107485	5.709435	3.398721	0	1.167288	0	2.40951	0	1.028084	1.861441	0	0.824452	3.079129	0	0.896308
AC010931.	197.947	145.0566	63.94455	208.8978	128.4683	134.7427	137.6482	104.6411	144.7437	122.9695	120.4755	173.8948	162.4373	148.9152	138.8768	79.14738	191.9324	42.77627	134.4462
AC010931.	35.55358	20.00781	21.98094	17.66798	28.79461	10.27698	31.43817	16.66849	28.01491	15.37119	13.2523	37.72321	6.168506	31.64449	35.20821	196.2195	125.2179	320.367	176.5727
AC010947.	0	0	0	0	0	0	0	0	0	0	0	0	0	0	0	0	3.079129	0	0
AC010967.	29.78813	30.01171	13.98787	1.039293	19.93473	9.135096	19.54265	0.926027	2.334575	5.764196	2.40951	21.1618	20.56169	9.307203	11.73607	7.420066	29.76491	27.304	9.859387
AC010969.	0	0	0	0	0	1.141887	0	0	14.00745	6.724895	2.40951	3.680313	0	0.93072	0	0	0	0	0
AC010969.	903.2531	972.3795	791.3139	1126.593	1067.615	1059.671	725.6269	939.9174	1230.321	1118.254	1087.894	718.5811	828.636	745.507	775.5586	895.3547	954.5299	683.5102	566.4666
AC010973.	42.27993	42.0164	33.97054	63.39686	60.91167	31.97284	94.31451	49.07943	0	45.15286	46.98544	65.32555	64.76932	80.04195	54.76833	108.8276	122.1388	61.88907	57.36371
AC010973.	79.75532	101.0394	70.93849	37.41454	170.5527	184.9857	59.47762	128.7178	267.3089	214.2359	265.0461	132.4913	121.314	97.72563	79.21847	81.62073	92.37386	128.3288	77.08248
AC010974.	0	0	1.998267	0	0	3.425661	1.69936	0.926027	0	8.646293	0	0	2.056169	0	2.934017	0	0	0.910133	2.688924
AC010975.	6.726353	6.002343	7.993069	16.62868	7.752394	9.135096	33.98721	7.408216	16.34203	9.606993	0	6.440548	6.168506	2.792161	3.912023	7.420066	19.50115	5.4608	19.71877
AC010975.	0	0	0	0	0	0	0	0	0	5.764196	0	0	0	0	0	0	0	0	0
AC010975.	2.882723	0	4.995668	1.039293	0	0	4.248401	0	0	3.842797	0	0	2.056169	0	0	3.297807	0	0	0
AC010976.	509.281	472.1843	669.4196	417.7957	637.9113	439.6265	594.7762	663.0354	301.1602	570.6554	531.2969	610.9319	465.7222	476.5288	621.0337	382.5456	319.203	445.9654	439.1909
AC010976.	102.8171	355.1386	113.9012	172.5226	275.7637	380.2484	127.452	127.7917	808.9304	319.9129	462.6259	234.6199	236.4594	178.6983	177.0191	219.3042	258.6468	282.1414	268.8924
AC010978.	5.765445	10.0039	12.98874	2.078586	11.07485	1.141887	25.49041	19.44657	5.836439	4.803496	3.614265	13.80117	14.39318	5.584322	9.780058	10.71787	1.026376	0.910133	12.54831
AC010980.	710.1107	879.3432	568.5071	862.613	656.7386	808.456	903.2101	558.3943	870.7966	692.6642	781.8859	740.663	784.4284	675.7029	605.3856	780.7559	837.523	567.0131	828.1885
AC010980.	2670.362	3841.499	2498.833	4429.466	2444.219	3567.255	3901.732	3091.078	1598.017	2635.198	1620.395	2646.145	3084.253	2636.731	2931.083	3511.34	3539.972	1687.387	2359.083
AC010982.	4.804538	12.00469	0	14.5501	0	19.41208	11.04584	14.81643	4.669151	0	0	0	1.028084	3.722881	8.802052	22.2602	9.237386	0	0
AC010997.	0	0	0	1.039293	4.42994	0	0	0	4.669151	1.921399	0	0	0	0	0	0	0	0.910133	5.377848
AC010997.	0.960908	17.00664	7.993069	7.275049	2.21497	18.27019	18.69297	31.48492	0	10.56769	1.204755	13.80117	13.3651	13.03008	7.824047	5.771163	8.21101	34.58507	34.95601
AC010997.	1.921815	2.000781	5.994802	2.078586	4.42994	0	4.248401	0	7.003726	16.33189	2.40951	4.600391	2.056169	3.722881	1.956012	4.946711	0	7.281067	3.585232
AC010997.	141.2534	197.0769	270.7652	91.45776	151.7254	161.0061	111.3081	118.5315	221.7847	273.7993	263.8413	143.5322	175.8024	146.1231	176.041	133.5612	173.4576	172.0152	212.425
AC010997.	0	2.000781	0	0	0	0	0	0	0	0	6.023774	0.920078	0	0	0	0	0	0	0
AC010997.	0	0	1.998267	0	3.322455	2.283774	0.84968	3.704108	4.669151	3.842797	0	0	0	2.792161	2.934017	0.824452	5.131881	1.820267	0.896308
AC010998.	11.53089	66.02577	75.93416	82.10413	58.6967	66.22945	86.66738	82.41641	77.04099	56.68126	43.37118	103.0488	105.8927	82.83411	90.95454	86.56744	99.5585	50.96747	71.70464
AC010998.	3.84363	0	3.996535	2.078586	9.967364	0	0	0	5.836439	12.48909	0	0.920078	4.112337	0	0	0	0	0	3.585232
AC010999.	22.10087	15.00586	45.96015	13.51081	38.76197	13.70264	14.44456	32.41095	10.50559	16.33189	38.55216	12.8811	14.39318	15.82225	22.49413	23.9091	25.65941	26.39387	12.54831
AC010999.	5.765445	7.002733	13.98787	4.157171	16.61227	11.41887	4.248401	7.408216	15.17474	5.764196	8.433284	7.360626	2.056169	12.09936	11.73607	3.297807	4.105505	4.550667	7.170464
AC011005.	19.21815	26.01015	3.996535	14.5501	13.28982	34.25661	10.19616	14.81643	14.00745	21.13538	12.04755	5.520469	14.39318	24.19873	14.67009	23.9091	14.36927	17.29253	7.170464
AC011005.	162.3934	133.0519	173.8493	209.9371	318.9557	218.1004	145.2953	337.9999	381.7031	361.2229	316.8505	182.1755	204.5888	127.5087	154.5249	90.6897	120.086	102.8451	89.63079
AC011008.	18.25724	20.00781	24.97834	22.86444	16.61227	12.56076	10.19616	25.92876	30.34948	26.89958	6.023774	12.8811	5.140422	8.376483	9.780058	10.71787	17.4484	18.20267	6.274156
AC011008.	910.9403	818.3194	907.2134	896.9097	656.7386	898.6651	927.0011	595.4354	1120.596	1207.599	1138.493	782.0665	865.647	718.5161	620.0557	813.734	897.0528	725.3763	881.967
AC011037.	0	0	0	0	0	0	0	0	1.167288	0	0	0	0	0.93072	0	0	2.052753	0	0
AC011092.	0.960908	0	0	0	1.107485	0	4.248401	0	0	0	0	0	1.028084	1.861441	0	3.297807	0	0	0.896308
AC011095.	0	1.00039	0	0	3.322455	0	0	0	0	0	0	0	0	2.792161	0	0	0	0	0
AC011120.	0	0	2.997401	1.039293	2.21497	4.567548	1.69936	12.96438	2.334575	0	2.40951	0	2.056169	7.445762	4.890029	0.824452	0	2.7304	1.792616
AC011124.	0	1.00039	0	1.039293	1.107485	2.283774	4.248401	0	7.003726	5.764196	0	0	6.168506	0	2.934017	0.824452	0	0	3.585232
AC011124.	0	4.001562	2.997401	1.039293	2.21497	0	0.84968	0	4.669151	0	0	0.920078	1.028084	3.722881	1.956012	0	1.026376	0	0

AC011131.	0	0	0	0	0	0	0	0.926027	1.167288	0	0	0	0	0	0	1.648904	0	0	0
AC011139.	42.27993	101.0394	214.8137	70.67191	94.13622	176.9925	77.3209	62.96984	311.6658	218.0787	198.7846	98.44837	64.76932	43.74385	22.49413	74.20066	86.21561	60.97894	138.9277
AC011193.	0	1.00039	1.998267	0	2.21497	0	7.647122	0	0	0	0	0.920078	0	1.861441	0	2.473355	2.052753	0	0
AC011193.	0	0	0	0	0	0	0	0	0	0.960699	9.638039	0	0	0	0	4.946711	0	0	0
AC011195.	4.804538	1.00039	0	0	4.42994	4.567548	0	3.704108	0	3.842797	9.638039	0.920078	1.028084	13.9608	2.934017	0	2.052753	0	0
AC011196.	0	0	0	0	0	0	5.947762	3.704108	0	0	0	0	1.028084	0	0	0	2.052753	0	0
AC011199.	40.35812	23.00898	20.98181	28.0609	23.25718	31.97284	7.647122	43.52327	39.68778	33.62447	49.39495	21.1618	23.64594	26.06017	24.45015	34.62698	20.52753	9.101334	25.09662
AC011228.	2.882723	10.0039	2.997401	3.117878	13.28982	5.709435	11.04584	1.852054	2.334575	9.606993	0	6.440548	6.168506	15.82225	15.64809	6.595615	0	6.370934	5.377848
AC011239.	292.1159	186.0726	199.8267	140.3045	74.20149	77.64832	215.8188	140.7561	155.2493	48.03496	43.37118	187.696	191.2237	195.4513	161.371	108.8276	158.0619	137.4301	145.2019
AC011239.	37.47539	72.02811	64.94369	51.96464	29.90209	75.36454	27.18977	20.37259	95.71759	42.27077	74.6948	11.96102	30.84253	69.80402	69.43841	28.03136	66.71446	13.652	17.92616
AC011242.	0	0	5.994802	0	0	0	0.84968	0	0	0	0	0	0	0	0	0	0	0	0
AC011243.	0	0	0	0	0	0	1.69936	0	0	0	0	0	0	0	0	0	2.052753	0	0
AC011247.	42.27993	34.01327	51.95495	41.57171	47.62185	27.40529	39.93497	17.59451	81.71014	42.27077	20.48083	54.28462	45.23571	73.5269	68.46041	50.29156	21.5539	14.56213	9.859387
AC011247.	7.68726	2.000781	3.996535	7.275049	8.859879	3.425661	4.248401	3.704108	0	2.882098	0	4.600391	5.140422	0.93072	12.71408	7.420066	0	6.370934	8.066772
AC011247.	8.648168	1.00039	3.996535	5.196464	4.42994	7.993209	1.69936	2.778081	4.669151	6.724895	12.04755	10.12086	1.028084	0.93072	2.934017	6.595615	10.26376	0.910133	1.792616
AC011257.	0	3.001171	2.997401	2.078586	0	7.993209	2.549041	1.852054	0	0.960699	0	1.840156	2.056169	0.93072	0.978006	3.297807	1.026376	3.640534	0.896308
AC011260.	0	0	0	1.039293	3.322455	0	0.84968	0	4.669151	0	3.614265	0.920078	0	7.445762	0	4.122259	0	0	7.170464
AC011266.	0.960908	0	0	0	6.64491	0	0	0	0	0	0	0	1.028084	3.722881	7.824047	0	4.105505	0	4.48154
AC011270.	0	0	0	0	0	0	0	0	16.34203	0	0	0	0	0	3.912023	0	2.052753	0.910133	0
AC011276.	5.765445	5.001952	2.997401	3.117878	11.07485	2.283774	3.398721	5.556162	11.67288	12.48909	3.614265	6.440548	2.056169	0.93072	4.890029	3.297807	3.079129	15.47227	5.377848
AC011284.	3.84363	0	4.995668	14.5501	1.107485	7.993209	3.398721	3.704108	0	0	0	2.760235	2.056169	3.722881	2.934017	13.19123	2.052753	0	3.585232
AC011287.	47.08447	37.01445	44.96101	66.51474	15.50479	20.55397	28.03945	18.52054	1.167288	29.78168	33.73314	26.68227	17.47743	21.40657	16.6261	19.78684	19.50115	20.02294	35.85232
AC011287.	0	0	0	0	0	0	0	0	0	0	0	0	0	0	0	0.824452	0	0.910133	0
AC011290.	6.726353	17.00664	20.98181	16.62868	5.537425	6.851322	13.59488	3.704108	25.68033	11.52839	15.66181	8.280704	6.168506	10.23792	0.978006	12.36678	9.237386	20.02294	6.274156
AC011290.	172.9634	159.0621	282.7548	134.0688	156.1554	242.08	198.8252	297.2547	227.6211	274.76	315.6458	240.1404	228.2347	174.0447	160.393	159.9437	148.8246	236.6347	181.9505
AC011294.	24.02269	0	15.98614	7.275049	34.33203	34.25661	17.84329	6.482189	29.18219	12.48909	20.48083	27.60235	15.42127	9.307203	19.56012	3.297807	8.21101	6.370934	5.377848
AC011297.	214.2824	259.1011	176.8467	364.7918	209.3147	0	0	0	0	449.6072	431.3022	0	0	0	0	0	0	0	0
AC011298.	0	2.000781	0.999134	0	0	126.7495	53.52986	42.59724	10.50559	0	3.614265	0	1.028084	8.376483	8.802052	10.71787	14.36927	20.02294	29.57816
AC011306.	32.67086	27.01054	56.95062	39.49313	29.90209	25.12151	40.78465	14.81643	0	18.25329	20.48083	46.00391	61.68506	37.22881	53.79032	74.20066	46.18693	35.4952	36.74863
AC011330.	233.5005	232.0906	262.7722	262.9411	300.1284	199.8302	209.871	345.4081	128.4017	197.904	189.1465	367.1112	314.5938	241.9873	237.6554	247.3355	220.6709	308.5352	328.0487
AC011330.	49.96719	76.02967	47.95842	73.78979	83.06137	74.22266	38.23561	130.5698	81.71014	75.89524	110.8374	59.80509	60.65698	67.94258	81.17448	61.83389	59.52982	62.79921	46.60801
AC011337.	481.4147	265.1035	359.6881	483.2711	231.4643	298.0325	570.1354	383.3752	330.3424	364.105	330.1028	417.7155	418.4303	343.4358	439.1246	592.7809	516.2673	473.2694	320.8782
AC011346.	13.45271	9.003514	9.991337	6.235757	0	6.851322	0	21.29862	11.67288	0	0	2.760235	3.084253	5.584322	0.978006	3.297807	0	1.820267	0
AC011363.	10.56998	7.002733	9.991337	14.5501	3.322455	0	22.09169	6.482189	9.338302	5.764196	10.84279	17.48149	16.44935	13.9608	10.75806	3.297807	5.131881	8.191201	7.170464
AC011365.	6.726353	16.00625	21.98094	6.235757	9.967364	3.425661	10.19616	15.74246	14.00745	20.17468	13.2523	29.4425	30.84253	28.85233	15.64809	32.15362	62.60895	22.75334	12.54831
AC011365.	5.765445	13.00508	15.98614	13.51081	14.3973	7.993209	12.7452	4.630135	16.34203	18.25329	22.89034	31.28266	6.168506	18.61441	2.934017	15.66458	14.36927	31.85467	19.71877
AC011369.	16.33543	14.00547	24.97834	2.078586	22.1497	14.84453	19.54265	4.630135	11.67288	9.606993	26.50461	9.200782	29.81445	4.653602	2.934017	14.01568	1.026376	13.652	8.963079
AC011369.	23.06178	13.00508	8.992203	9.353635	38.76197	19.41208	20.39233	22.22465	1.167288	7.685594	4.81902	11.04094	8.224675	18.61441	6.846041	23.08465	21.5539	14.56213	4.48154
AC011373.	25.9445	28.01093	25.97748	19.74656	24.36467	43.39171	32.28785	47.22738	65.36811	21.13538	74.6948	25.76219	23.64594	17.68369	17.6041	8.244518	26.68578	8.191201	16.13354
AC011374.	82.63805	31.0121	44.96101	75.86847	44.2994	25.12151	75.62154	17.59451	166.9221	62.44545	37.3474	30.36258	16.44935	32.57521	43.03226	93.98751	76.97822	68.26001	77.08248
AC011374.	97.05166	75.02928	128.8882	92.49706	152.8329	163.2898	70.52346	97.23284	319.8368	245.939	156.6181	103.0488	93.55568	145.1924	106.6026	87.39189	89.29473	97.38428	95.00864
AC011377.	16.33543	29.01132	12.98874	24.94303	42.08443	3.425661	16.14392	16.66849	0	15.37119	21.68559	18.40156	16.44935	51.18962	51.83431	10.71787	6.158258	11.83173	11.652
AC011379.	17.29634	21.0082	43.96188	18.70727	22.1497	10.27698	38.23561	42.59724	29.18219	12.48909	18.07132	11.04094	30.84253	2.792161	17.6041	8.244518	25.65941	2.7304	9.859387
AC011379.	492.9456	399.1558	596.4828	1349.002	300.1284	125.6076	1038.309	219.4684	249.7996	276.6814	202.3988	553.8871	449.2729	392.764	528.1231	610.0944	578.8762	1047.564	847.9073
AC011383.	4.804538	1.00039	5.994802	2.078586	6.64491	9.135096	0	11.11232	8.171014	1.921399	0	7.360626	1.028084	12.09936	7.824047	14.01568	6.158258	3.640534	4.48154
AC011389.	0	7.002733	0	0	8.859879	11.41887	3.398721	0.926027	0	0.960699	1.204755	4.600391	2.056169	0.93072	2.934017	5.771163	0	3.640534	0
AC011389.	19.21815	26.01015	13.98787	7.275049	7.752394	15.98642	3.398721	7.408216	43.18965	20.17468	22.89034	25.76219	12.33701	26.99089	18.58211	19.78684	6.158258	4.550667	1.792616
AC011389.	0	0	0	1.039293	2.21497	0	0.84968	2.778081	14.00745	0	0	0	0	0	0	0	0	0	0
AC011396.	0	0	7.993069	1.039293	0	2.283774	1.69936	1.852054	0	0.960699	2.40951	1.840156	0	0.93072	3.912023	4.946711	0	0	0
AC011396.	118.1916	164.064	154.8657	303.4735	56.48173	293.465	163.1386	148.1643	43.18965	112.4018	125.2945	66.24563	76.07824	186.1441	161.371	234.1443	217.5918	81.00187	94.11233
AC011396.	0.960908	0	0	0	0	0	0.84968	0	0	0	0	0	0	0	0	0.824452	0	0	0
AC011405.	25.9445	25.00976	36.96795	33.25737	45.40688	17.12831	64.5757	24.0767	36.18592	22.09608	26.50461	23.92203	19.5336	37.22881	22.49413	33.80253	6.158258	10.01147	25.99293
AC011406.	0.960908	4.001562	0	2.078586	1.107485	0	5.947762	1.852054	0	0	0	0.920078	0	0	0.978006	0	0	0	3.585232
AC011410.	0	0	0	0	0	0	0	0	0	0	0	0	0	2.792161	0	0	0	0	0.896308
AC011411.	17.29634	31.0121	24.97834	22.86444	25.47215	20.55397	42.48401	25.00273	0	28.82098	8.433284	39.56336	57.57272	20.47585	16.6261	23.08465	18.47477	20.93307	21.51139
AC011416.	0.960908	0	0.999134	0	0	0	0	0	0	0	0	3.680313	1.028084	0	0.978006	4.946711	0	0.910133	1.792616
AC011416.	86.48168	23.00898	36.96795	121.5973	54.26676	54.81058	89.21643	42.59724	57.1971	55.72056	119.2707	103.9688	95.61185	72.59618	94.86656	78.32292	80.05735	113.7667	96.80126
AC011442.	17.29634	7.002733	49.95668	41.57171	34.33203	1.141887	16.9936	16.66849	4.669151	34.58517	22.89034	23.00196	10.28084	34.43665	15.64809	18.96239	55.42432	29.12427	34.0597
AC011443.	2.882723	0	0	3.117878	4.42994	0	5.947762	0	0	0.960699	0	0	4.112337	7.445762	9.780058	2.473355	0	0	0
AC011444.	44.20175	36.01406	34.96968	27.02161	31.00958	57.09435	14.44456	17.59451	93.38302	19.21399	81.92333	30.36258	10.28084	19.54513	12.71408	14.84013	40.02867	40.04587	30.47447
AC011444.	0	0	0	0	1.107485	0	1.69936	0	0	0.960699	0	0	0	0.93072	0	0	0	0	1.792616
AC011444.	1.921815	5.001952	10.99047	4.157171	19.93473	1.141887	24.64073	4.630135	21.01118	18.25329	22.89034	9.200782	10.28084	0.93072	7.824047	4.122259	6.158258	8.191201	8.066772

AC011444.	0	9.003514	0	0	0	1.141887	1.69936	0	0	0	2.40951	3.680313	0	3.722881	2.934017	3.297807	0	0	3.585232
AC011445.	17.29634	33.01288	12.98874	19.74656	12.18233	12.56076	31.43817	23.15068	18.6766	15.37119	9.638039	37.72321	51.40422	28.85233	15.64809	15.66458	22.58028	20.02294	51.08955
AC011445.	41.31902	45.01757	7.993069	28.0609	45.40688	66.22945	28.03945	25.92876	56.02981	49.95636	159.0276	29.4425	39.06721	37.22881	24.45015	39.57369	55.42432	60.06881	35.85232
AC011447.	0	5.001952	0	2.078586	0	3.425661	3.398721	0	0	3.842797	3.614265	2.760235	2.056169	3.722881	0.978006	0.824452	1.026376	0	1.792616
AC011447.	53.81082	57.02225	75.93416	101.8507	33.22455	71.93888	78.17058	73.15614	84.04472	73.97384	89.15186	65.32555	50.37613	62.35826	52.81231	84.09409	42.08143	75.54107	78.8751
AC011447.	2.882723	0	0	0	1.107485	1.141887	0.84968	1.852054	0	0	0	2.760235	0	0	0	0	1.026376	0.910133	0
AC011447.	0	0	0.999134	0	0	2.283774	1.69936	0	0	0	0	0.920078	2.056169	0	0	2.473355	0	0.910133	0
AC011447.	12.4918	17.00664	57.94975	19.74656	21.04221	5.709435	16.9936	14.81643	12.84017	25.93888	43.37118	18.40156	14.39318	12.09936	30.31818	19.78684	17.4484	12.74187	7.170464
AC011447.	72.06806	55.02147	97.9151	152.776	49.83682	124.4657	47.58209	93.52873	172.7586	145.0656	97.58515	64.40548	98.6961	79.11123	98.77859	109.6521	103.664	75.54107	90.5271
AC011448.	24.02269	27.01054	19.98267	5.196464	26.57964	29.68906	11.89552	12.03835	46.69151	34.58517	86.74235	22.08188	31.87062	12.09936	10.75806	12.36678	21.5539	20.93307	6.274156
AC011450.	186.4161	250.0976	238.7929	140.3045	187.165	206.6815	201.3742	160.2027	190.2679	166.201	150.5944	233.6999	279.6389	216.8578	188.7551	161.5926	202.1961	208.4206	169.4022
AC011451.	367.0667	290.1132	296.7427	284.7662	238.1093	291.1812	322.0288	240.767	281.3163	289.1705	207.2178	279.7038	302.2568	327.6135	313.9399	281.9625	294.57	378.6155	253.6551
AC011451.	61.49808	61.02382	71.93762	50.92535	56.48173	57.09435	46.73241	89.82462	29.18219	31.70308	63.85201	54.28462	69.90974	62.35826	76.28445	48.64266	92.37386	34.58507	26.88924
AC011451.	46.12356	49.01913	37.96708	27.02161	21.04221	35.3985	30.58849	68.526	43.18965	41.31007	40.96167	71.7661	59.62889	37.22881	47.92229	18.13794	26.68578	45.50667	56.4674
AC011453.	0	5.001952	2.997401	0	0	10.27698	5.098081	0	0	0	2.40951	1.840156	0	0.93072	0.978006	0	1.026376	1.820267	0.896308
AC011455.	1.921815	3.001171	3.996535	6.235757	0	6.851322	0.84968	3.704108	12.84017	5.764196	2.40951	8.280704	10.28084	4.653602	6.846041	4.122259	1.026376	8.191201	16.13354
AC011455.	363.223	533.2081	378.6717	448.9745	397.5871	350.5593	403.5981	396.3396	824.1051	498.6029	420.4595	448.9982	432.8235	349.9508	344.2581	361.1099	331.5195	339.4798	305.641
AC011455.	0	0	0	1.039293	0	0	0	0	0	0	1.204755	0	1.028084	0.93072	0	1.648904	1.026376	0	0
AC011458.	6.726353	13.00508	27.97574	29.1002	0	28.54718	26.34009	20.37259	3.501863	17.29259	7.228529	22.08188	23.64594	4.653602	14.67009	18.96239	16.42202	13.652	14.34093
AC011458.	7.68726	7.002733	12.98874	11.43222	5.537425	5.709435	10.19616	0.926027	8.171014	16.33189	20.48083	13.80117	13.3651	3.722881	2.934017	6.595615	6.158258	13.652	17.92616
AC011460.	10.56998	29.01132	9.991337	30.13949	3.322455	15.98642	19.54265	10.1863	36.18592	8.646293	21.68559	16.56141	12.33701	6.515042	17.6041	18.13794	9.237386	39.13574	5.377848
AC011461.	30.74904	23.00898	20.98181	66.51474	12.18233	38.82416	34.83689	24.0767	43.18965	34.58517	22.89034	23.92203	35.98295	27.92161	22.49413	73.37621	54.39794	38.2256	37.64493
AC011462.	5.765445	2.000781	1.998267	3.117878	0	1.141887	1.69936	9.26027	0	0.960699	1.204755	0.920078	4.112337	2.792161	0	0	0	0.910133	2.688924
AC011462.	92.24712	71.02772	50.95582	55.08252	101.8886	76.50643	58.62794	70.37805	106.2232	110.4804	196.375	81.88696	53.46039	70.73474	80.19648	74.20066	50.29244	91.01334	65.43048
AC011465.	4.804538	2.000781	20.98181	7.275049	13.28982	1.141887	6.797442	9.26027	0	11.52839	1.204755	9.200782	12.33701	20.47585	1.956012	12.36678	2.052753	4.550667	4.48154
AC011466.	22.10087	59.02304	58.94889	30.13949	45.40688	52.5268	28.88913	71.30408	26.84762	29.78168	39.75691	69.00587	45.23571	43.74385	24.45015	47.81821	28.73854	46.4168	43.91909
AC011466.	5.765445	1.00039	2.997401	9.353635	2.21497	3.425661	22.94137	0.926027	0	0.960699	1.204755	11.96102	7.196591	0.93072	3.912023	7.420066	0	0.910133	5.377848
AC011466.	19.21815	14.00547	21.98094	14.5501	14.3973	9.135096	19.54265	28.70684	0	17.29259	13.2523	15.64133	28.78636	17.68369	32.27419	18.96239	30.79129	40.956	16.13354
AC011466.	26.90541	20.00781	33.97054	30.13949	57.58922	13.70264	28.88913	101.863	5.836439	20.17468	42.16642	45.08383	49.34805	27.92161	37.16422	22.2602	33.87042	40.956	29.57816
AC011467.	1.921815	10.0039	1.998267	0	6.64491	0	5.098081	8.334243	1.167288	0	1.204755	6.440548	2.056169	0	0	0	0	4.550667	6.274156
AC011467.	0	0	0	0	0	0	0	0	0	1.921399	0	0	1.028084	0	0	0	0	0	0
AC011467.	0	0	0	0	0	0	0	0	0	0	4.81902	0	0	0	0	0	0	0	0
AC011468.	229.6569	230.0898	238.7929	155.8939	199.3473	226.0936	219.2175	269.4739	447.0712	353.5373	244.5652	204.2574	176.8305	241.0566	258.1935	219.3042	172.4312	198.4091	142.513
AC011468.	0.960908	0	0	0	0	0	0	0	0	0	0	0	0	0	0	0	1.026376	0	0
AC011468.	7.68726	15.00586	2.997401	14.5501	9.967364	17.12831	9.346483	14.81643	24.51304	4.803496	14.45706	15.64133	11.30893	24.19873	28.36217	8.244518	24.63303	8.191201	7.170464
AC011468.	54.77173	23.00898	37.96708	57.1611	44.2994	27.40529	46.73241	15.74246	11.67288	40.34937	50.5997	23.00196	37.01104	40.95169	28.36217	37.92478	36.94955	21.8432	23.30401
AC011468.	44.20175	75.02928	87.92376	86.2613	87.49131	25.12151	38.23561	54.6356	79.37557	32.66377	48.1902	67.16571	59.62889	40.02097	52.81231	44.5204	34.89679	27.304	41.23017
AC011471.	0	0	0	6.235757	7.752394	1.141887	0	0.926027	0	0	0	0	0.920078	1.028084	0	5.868035	0	0	0
AC011471.	0	0	0	0	0	0	0	0	0	0	0	3.680313	0	0	0	0	0	0	0
AC011472.	183.5333	195.0761	205.8215	101.8507	258.044	155.2966	141.8966	187.9835	288.3201	210.3931	349.3789	267.7428	223.0943	242.918	253.3035	155.8214	127.2707	252.107	214.2176
AC011472.	114.348	259.1011	200.8259	122.6365	343.3203	340.2823	151.2431	192.6136	535.7851	486.1138	569.8491	133.4113	117.2016	185.2133	203.4252	142.6302	136.508	102.8451	130.861
AC011472.	74.95079	47.01835	90.92116	56.12181	107.426	66.22945	56.92858	112.9753	193.7698	95.10923	166.2562	55.20469	84.30292	93.07203	56.72434	39.57369	80.05735	80.09174	54.67478
AC011472.	21.13997	36.01406	37.96708	15.58939	29.90209	10.27698	25.49041	32.41095	14.00745	29.78168	24.0951	39.56336	54.48847	41.88241	49.8783	19.78684	33.87042	31.85467	22.4077
AC011473.	0	0	0	0	2.21497	0	0	6.482189	0	0	0	0	0	0	0	0	0	0	0.896308
AC011473.	0	0	0	0	7.752394	0	0.84968	4.630135	0	0	0	0.920078	0	0	0	0	0	5.4608	0
AC011474.	97.05166	115.0449	77.93243	176.6798	53.15928	89.06719	164.838	88.8986	42.02236	12.48909	50.5997	53.36454	41.12337	150.7767	130.0748	169.8371	186.8005	78.27147	111.1422
AC011474.	7.68726	8.003124	1.998267	11.43222	14.3973	19.41208	12.7452	3.704108	15.17474	3.842797	0	6.440548	5.140422	13.9608	5.868035	13.19123	10.26376	2.7304	21.51139
AC011474.	17.29634	9.003514	7.993069	1.039293	4.42994	6.851322	4.248401	0	10.50559	3.842797	0	10.12086	2.056169	1.861441	7.824047	5.771163	2.052753	9.101334	12.54831
AC011475.	0.960908	0	0	0	0	0	0	0	0	0	0	0	0	0	0	0.824452	0	0	0
AC011476.	0	0	0	0	0	0	0	0	0	0	0	1.840156	0	0	0	0.824452	1.026376	0	0.896308
AC011476.	10.56998	15.00586	32.97141	10.39293	1.107485	13.70264	11.89552	33.33697	74.70641	45.15286	10.84279	18.40156	28.78636	46.53602	37.16422	20.6113	29.76491	24.5736	24.20031
AC011476.	276.7414	184.0718	226.8033	273.334	229.2494	179.2763	289.741	321.3314	96.88488	174.8473	163.8467	305.466	317.6781	384.3875	425.4325	275.3669	234.0138	308.5352	206.1508
AC011477.	340.1613	214.0836	280.7566	207.8586	261.3664	270.6272	275.2964	386.1533	298.8257	246.8997	230.1082	344.1093	308.4253	290.3847	264.0616	247.3355	250.4358	276.6806	244.6921
AC011477.	1020.484	742.2897	1178.978	781.5482	757.5197	790.1858	910.8572	1023.26	420.2236	801.2232	642.1343	1006.566	893.4053	794.8351	1043.532	886.2857	930.9233	862.8065	759.1728
AC011477.	343.044	291.1136	266.7687	358.556	203.7772	324.2959	367.9115	332.4437	155.2493	188.2971	106.0184	312.8266	298.1445	230.8186	277.7537	306.6961	322.2821	281.2312	272.4776
AC011477.	15.37452	16.00625	10.99047	16.62868	2.21497	10.27698	5.947762	22.22465	0	2.882098	8.433284	6.440548	9.252759	13.03008	13.69208	10.71787	20.52753	14.56213	3.585232
AC011477.	0	1.00039	0	0	11.07485	1.141887	4.248401	11.11232	0	0	0	0	6.168506	1.861441	1.956012	3.297807	4.105505	0	5.377848
AC011477.	11.53089	22.00859	9.991337	2.078586	6.64491	2.283774	15.29424	1.852054	0	3.842797	0	19.32164	13.3651	28.85233	20.53812	28.03136	18.47477	13.652	12.54831
AC011477.	57.65445	62.02421	51.95495	58.2004	66.4491	55.95246	60.3273	29.63287	37.35321	56.68126	39.75691	58.88501	58.60081	78.18051	48.90029	57.71163	82.1101	75.54107	101.2828
AC011478.	7.68726	4.001562	35.96881	21.82515	1.107485	17.12831	12.7452	3.704108	0	0	1.204755	9.200782	7.196591	18.61441	9.780058	14.01568	18.47477	8.191201	18.82247
AC011479.	15.37452	28.01093	21.98094	16.62868	8.859879	15.98642	16.9936	19.44657	16.34203	18.25329	13.2523	23.92203	18.50552	20.47585	13.69208	14.01568	15.39564	30.94454	26.88924
AC011479.	2.882723	8.003124	7.993069	0	6.64491	18.27019	30.58849	7.408216	1.167288	17.29259	15.66181	4.600391	11.30893	5.584322	2.934017	5.771163	13.34289	3.640534	0.896308

AC011481.	113.3871	143.0558	196.8293	70.67191	110.7485	121.04	91.76547	108.3452	142.4091	171.9652	174.6895	128.811	117.2016	130.3008	162.349	77.49847	60.5562	118.3173	115.6237
AC011481.	11.53089	0	3.996535	13.51081	17.71976	0	16.9936	14.81643	19.84389	11.52839	9.638039	15.64133	7.196591	4.653602	10.75806	15.66458	8.21101	11.83173	10.7557
AC011481.	177.7679	179.0699	268.767	229.6837	150.6179	397.3767	92.61515	161.1287	306.9967	273.7993	328.8981	163.7739	166.5497	168.4604	120.2947	331.4296	310.992	182.9368	282.337
AC011483.	0	0	0	0	0	3.425661	0	0	0	0	2.40951	0	1.028084	0	1.956012	1.648904	0	0	8.963079
AC011483.	0.960908	1.00039	0	1.039293	0	2.283774	0	0	0	1.921399	0	0	0	0	0	0	0	0.910133	0
AC011483.	0	0	0	2.078586	0	0	0	0	0	0	0	0	0	0	0	0	0	0.910133	0
AC011484.	0.960908	0	0	0	0	0	0	0	0	0	0	0	0	0	0	2.473355	0	0	0
AC011487.	42.27993	40.01562	48.95755	47.80747	47.62185	53.66869	37.38593	50.93149	39.68778	20.17468	33.73314	38.64329	44.20763	56.77394	71.39443	31.32917	30.79129	33.67494	39.43755
AC011487.	8.648168	14.00547	10.99047	5.196464	0	7.993209	3.398721	2.778081	1.167288	10.56769	7.228529	4.600391	4.112337	2.792161	2.934017	0.824452	10.26376	4.550667	7.170464
AC011487.	0	0	3.996535	0	0	0	0	0	0	0	0	0	0	0.93072	0.978006	0	0	0	0
AC011487.	6.726353	3.001171	3.996535	3.117878	2.21497	5.709435	3.398721	7.408216	1.167288	6.724895	3.614265	8.280704	7.196591	11.16864	4.890029	2.473355	1.026376	3.640534	8.066772
AC011489.	127.8007	113.0441	116.8986	163.169	65.34161	121.04	102.8113	110.1972	105.0559	148.9084	99.99465	111.3295	137.7633	105.1714	100.7346	100.5831	135.4817	113.7667	66.32679
AC011491.	3.84363	4.001562	0	1.039293	0	1.141887	0.84968	0	0	3.842797	0	0.920078	0	7.445762	0.978006	2.473355	2.052753	3.640534	8.963079
AC011491.	4.804538	17.00664	9.991337	8.314342	11.07485	2.283774	12.7452	7.408216	7.003726	16.33189	8.433284	16.56141	9.252759	18.61441	17.6041	4.946711	7.184634	18.20267	12.54831
AC011491.	0	5.001952	5.994802	5.196464	6.64491	5.709435	2.549041	2.778081	4.669151	1.921399	6.023774	1.840156	1.028084	0	1.956012	10.71787	4.105505	2.7304	2.688924
AC011492.	0	0	0	0	1.107485	0	0	0.926027	0	0	0	0	0	0	0	0.824452	0	0	0
AC011493.	36.51449	47.01835	52.95408	42.611	77.52394	113.0468	39.93497	41.67122	10.50559	64.36685	36.14265	47.84407	27.75828	30.71377	31.29619	70.07841	84.16285	47.32694	21.51139
AC011494.	0	2.000781	0.999134	0	0	0	0	2.549041	0	0	4.81902	1.840156	1.028084	1.861441	2.934017	0.824452	5.131881	4.550667	0
AC011495.	94.16894	62.02421	101.9116	29.1002	58.6967	52.5268	71.37314	99.08489	58.36439	96.06993	103.6089	98.44837	103.8365	67.94258	135.9428	65.13169	51.31881	128.3288	92.31972
AC011497.	5.765445	8.003124	5.994802	11.43222	18.82724	3.425661	4.248401	11.11232	0	21.13538	7.228529	6.440548	7.196591	2.792161	6.846041	9.06897	8.21101	9.101334	9.859387
AC011498.	41.31902	17.00664	34.96968	46.76817	17.71976	15.98642	41.63433	24.0767	15.17474	17.29259	30.11887	25.76219	23.64594	26.06017	34.2302	45.34485	52.34519	49.1472	30.47447
AC011498.	2.882723	5.001952	16.98527	3.117878	1.107485	2.283774	8.496802	1.852054	0	0.960699	2.40951	3.680313	0	0.93072	10.75806	3.297807	1.026376	21.8432	4.48154
AC011498.	18.25724	116.0453	118.8969	53.00393	78.63143	90.20907	92.61515	200.9479	10.50559	89.34503	84.33284	70.84602	44.20763	67.94258	86.06451	67.60505	52.34519	55.51814	50.19325
AC011498.	0	0	0.999134	2.078586	1.107485	6.851322	0.84968	0	0	0	0	1.840156	2.056169	0	3.912023	0	1.026376	0	13.44462
AC011498.	50.9281	104.0406	51.95495	38.45383	80.8464	95.91851	87.51706	51.85751	91.04844	89.34503	102.4042	112.2495	134.6791	57.70466	70.41642	69.25395	40.02867	132.8795	80.66772
AC011498.	414.1511	329.1285	393.6587	457.2888	317.8482	368.8295	417.193	364.8547	259.1379	415.0221	481.902	482.121	397.8687	379.7339	471.3988	374.3011	400.2867	594.3171	430.2278
AC011500.	0	0	2.997401	0	0	0	0	0	0	0	3.842797	0	0	0	0	0	0.824452	0	0	0
AC011500.	4.804538	2.000781	1.998267	0	1.107485	3.425661	3.398721	0	5.836439	0.960699	3.614265	9.200782	0	5.584322	0.978006	1.648904	0	5.4608	1.792616
AC011503.	24.9836	15.00586	23.97921	14.5501	16.61227	4.567548	33.98721	0.926027	9.338302	3.842797	0	1.840156	6.168506	3.722881	7.824047	9.893422	13.34289	9.101334	3.585232
AC011503.	65.34171	76.02967	61.94629	151.7367	68.66407	82.21586	80.71962	101.863	85.212	131.6158	132.523	92.9279	85.331	84.69555	83.1305	99.75867	145.7454	133.7896	119.209
AC011504.	1011.836	847.3307	1267.901	822.0806	1144.032	522.9842	1161.513	856.575	671.1904	723.4065	787.9097	876.8346	1108.275	999.5936	1114.927	715.6242	635.3269	873.7281	842.5295
AC011506.	0	4.001562	4.995668	4.157171	2.21497	1.141887	1.69936	1.852054	5.836439	5.764196	7.228529	2.760235	2.056169	3.722881	4.890029	2.473355	2.052753	0.910133	0.896308
AC011507.	4.804538	4.001562	2.997401	7.275049	0	1.141887	2.549041	0	0	0	0	6.440548	5.140422	0	0.978006	0.824452	0	0.910133	0
AC011509.	4.804538	0	2.997401	1.039293	8.859879	3.425661	2.549041	7.408216	21.01118	3.842797	1.204755	4.600391	5.140422	0.93072	0.978006	0	7.184634	0.910133	2.688924
AC011509.	20.17906	5.001952	14.987	17.66798	24.36467	11.41887	9.346483	17.59451	24.51304	6.724895	16.86657	11.96102	17.47743	6.515042	8.802052	16.48904	10.26376	23.66347	11.652
AC011510.	60.53717	69.02694	34.96968	91.45776	71.98652	63.94567	85.8177	69.45203	73.53913	28.82098	31.32363	92.9279	71.96591	72.59618	68.46041	76.67402	64.6617	90.10321	101.2828
AC011511.	20.17906	17.00664	5.994802	13.51081	18.82724	13.70264	17.84329	25.00273	43.18965	31.70308	39.75691	29.4425	20.56169	13.03008	21.51613	12.36678	15.39564	17.29253	10.7557
AC011511.	1.921815	1.00039	0	0	0	0	0	0	0	5.836439	0	0	0	0	0	0	0.824452	0	0	0
AC011511.	0	2.000781	0	0	3.322455	1.141887	5.098081	0	0	0	1.204755	0.920078	0	2.792161	0	4.122259	2.052753	0	0
AC011511.	0	0	0	0	0	0	0	0	0	0	1.204755	0	0	7.445762	0	1.648904	6.158258	0	0
AC011525.	8.648168	0	0	0	4.42994	0	0.84968	3.704108	0	0	0	0	0	0.93072	0	6.595615	1.026376	0	0.896308
AC011551.	6.726353	11.00429	0.999134	0	15.50479	4.567548	3.398721	7.408216	1.167288	13.44979	8.433284	14.72125	16.44935	3.722881	5.868035	15.66458	4.105505	13.652	8.963079
AC011586.	0.960908	0	0	0	4.42994	5.709435	3.398721	0	0	0	4.81902	0	1.028084	0.93072	2.934017	0	2.052753	0	0
AC011591.	9.609075	3.001171	0	0	9.967364	0	4.248401	3.704108	1.167288	5.764196	12.04755	1.840156	6.168506	0.93072	3.912023	1.648904	1.026376	5.4608	1.792616
AC011591.	9.609075	0	22.98007	1.039293	3.322455	0	5.947762	0.926027	0	11.52839	3.614265	8.280704	8.224675	0.93072	0	9.893422	6.158258	7.281067	6.274156
AC011592.	0	0	0	0	0	0	0.84968	5.556162	7.003726	0	0	0	0	0.93072	0	0.824452	0	0	0
AC011595.	5.765445	15.00586	24.97834	14.5501	26.57964	14.84453	18.69297	5.556162	77.04099	4.803496	9.638039	10.12086	10.28084	0	0.978006	27.20691	13.34289	5.4608	6.274156
AC011595.	8.648168	2.000781	12.98874	12.47151	3.322455	4.567548	6.797442	4.630135	2.334575	3.842797	3.614265	9.200782	1.028084	0	9.780058	3.297807	8.21101	10.9216	8.066772
AC011595.	16.33543	25.00976	16.98527	29.1002	17.71976	45.67548	51.83049	10.1863	46.69151	20.17468	27.70936	23.92203	27.75828	52.12034	33.2522	18.96239	33.87042	13.652	32.26709
AC011601.	0	0	0	0	1.107485	0	0	4.630135	4.669151	0.960699	1.204755	0	1.028084	1.861441	1.956012	0	0	0	0
AC011603.	122.0353	150.0586	176.8467	102.89	215.9596	158.7223	209.0213	129.6438	208.9445	171.0045	122.885	145.3724	141.8756	119.1322	108.5586	51.11601	67.74083	107.3957	160.4391
AC011603.	35.55358	32.01249	45.96015	12.47151	33.22455	41.10793	46.73241	44.4493	56.02981	67.24895	54.21397	40.48344	52.4323	38.15953	62.59237	33.80253	29.76491	47.32694	36.74863
AC011603.	152.7843	147.0574	124.8917	128.8723	155.0479	205.5397	129.1514	107.4191	100.3867	265.153	245.77	172.0546	162.4373	77.24979	122.2507	152.5236	156.0092	141.0707	132.6536
AC011604.	0	0	0.999134	0	0	0	0	0	0	0	0	0	0	0	1.956012	0	0	0	0
AC011611.	0	0	0	0	0	0	0	0	0	0	0	0	0	0	0	2.473355	0	0	0
AC011611.	0	0	0	0	0	0	3.398721	0	0	0	0	0	0	0	0	0	0	0	0
AC011611.	2.882723	9.003514	1.998267	0	12.18233	6.851322	2.549041	3.704108	7.003726	0.960699	2.40951	16.56141	20.56169	7.445762	4.890029	0.824452	2.052753	0.910133	1.792616
AC011611.	20.17906	20.00781	25.97748	25.98232	14.3973	23.97963	44.18337	18.52054	7.003726	33.62447	10.84279	46.92399	16.44935	20.47585	44.98827	13.19123	12.31652	20.93307	25.99293
AC011625.	0	0	0	3.117878	0	0	0.84968	0	0	6.724895	0	0	1.028084	0.93072	0	0	0	0	0
AC011626.	0	1.00039	0	0	0	1.141887	0	0	0	0	0	0	0	0	0	0	0	0	0
AC011632.	0.960908	5.001952	7.993069	8.314342	5.537425	6.851322	7.647122	1.852054	0	1.921399	0	5.520469	7.196591	13.9608	0.978006	9.06897	7.184634	1.820267	12.54831
AC011632.	0	0	0	1.039293	0	0	0	0	0	0.960699	3.614265	0	0	0	0	0	1.026376	0	0

AC011676.	10.56998	19.00742	23.97921	25.98232	9.967364	38.82416	28.88913	11.11232	56.02981	33.62447	8.433284	11.04094	23.64594	35.36737	31.29619	20.6113	16.42202	20.93307	33.16339
AC011676.	0	1.00039	1.998267	0	0	0	0	0	4.669151	1.921399	1.204755	0.920078	0	0	0	0.824452	1.026376	0	0
AC011676.	0	0	0.999134	0	8.859879	1.141887	1.69936	0	7.003726	4.803496	0	0	0	0	0	0	0	0	0
AC011676.	16.33543	8.003124	6.993936	18.70727	8.859879	5.709435	16.14392	8.334243	0	12.48909	10.84279	4.600391	6.168506	8.376483	18.58211	27.20691	12.31652	8.191201	14.34093
AC011700.	2.882723	14.00547	2.997401	0	0	4.567548	8.496802	9.26027	0	5.764196	34.93789	10.12086	2.056169	1.861441	0	3.297807	7.184634	9.101334	15.23724
AC011716.	3.84363	8.003124	14.987	8.314342	1.107485	1.141887	1.69936	0	3.501863	1.921399	1.204755	0.920078	14.39318	3.722881	0	1.648904	2.052753	1.820267	3.585232
AC011726.	2.882723	1.00039	2.997401	0	6.64491	3.425661	12.7452	0	4.669151	8.646293	4.81902	11.96102	5.140422	3.722881	3.912023	4.122259	6.158258	9.101334	4.48154
AC011726.	8.648168	0	0	2.078586	0	1.141887	0	0.926027	0	3.842797	4.81902	1.840156	9.252759	0	6.846041	2.473355	1.026376	0.910133	0.896308
AC011726.	33.63176	29.01132	53.95322	12.47151	16.61227	22.83774	38.23561	52.78354	19.84389	19.21399	22.89034	42.3236	64.76932	80.97267	82.15249	21.43575	11.29014	46.4168	32.26709
AC011731.	1.921815	4.001562	1.998267	0	0	4.567548	5.098081	0	0	2.882098	0	0.920078	0	2.792161	4.890029	2.473355	8.21101	5.4608	4.48154
AC011742.	0.960908	7.002733	5.994802	3.117878	1.107485	9.135096	0.84968	0	7.003726	0.960699	0	1.840156	0	3.722881	6.846041	6.595615	5.131881	3.640534	1.792616
AC011742.	10.56998	10.0039	1.998267	3.117878	5.537425	1.141887	8.496802	10.1863	9.338302	3.842797	0	15.64133	3.084253	7.445762	3.912023	4.122259	0	14.56213	0.896308
AC011742.	2.882723	45.01757	77.93243	16.62868	9.967364	18.27019	16.14392	73.15614	11.67288	47.07426	32.52838	49.68422	40.09529	12.09936	34.2302	1.648904	6.158258	20.93307	6.274156
AC011747.	6.726353	44.01718	11.9896	16.62868	0	20.55397	20.39233	12.96438	4.669151	1.921399	13.2523	4.600391	6.168506	9.307203	5.868035	15.66458	9.237386	10.01147	8.963079
AC011747.	0	0	0	9.353635	0	1.141887	0	0.926027	0	0	1.204755	0	1.028084	0	0	3.297807	0	0.910133	0.896308
AC011752.	1.921815	2.000781	4.995668	3.117878	0	3.425661	2.549041	0	4.669151	5.764196	0	0	1.028084	6.515042	5.868035	0.824452	2.052753	0.910133	0
AC011753.	0.960908	0	1.998267	2.078586	2.21497	0	6.797442	0	1.167288	3.842797	1.204755	0	2.056169	0	0	0.824452	3.079129	6.370934	0.896308
AC011753.	5.765445	0	0	0	0	0	0.84968	0.926027	0	0	0	0.920078	1.028084	1.861441	0.978006	0	1.026376	0	0
AC011753.	40.35812	19.00742	71.93762	70.67191	75.30897	36.54038	101.1119	38.89314	0	27.86028	16.86657	84.6472	67.85357	38.15953	69.43841	46.99375	24.63303	66.43974	100.3865
AC011755.	0	8.003124	0.999134	6.235757	0	11.41887	8.496802	10.1863	11.67288	5.764196	0	4.600391	6.168506	10.23792	0.978006	3.297807	1.026376	7.281067	0
AC011767.	396.8548	281.1097	313.728	692.169	196.0248	328.8635	609.2207	312.9971	52.52795	213.2752	137.3421	369.8714	330.0151	341.5744	327.632	527.6492	490.6078	254.8374	326.2561
AC011773.	0	4.001562	2.997401	0	0	10.27698	2.549041	0	0	1.921399	0	1.840156	2.056169	0	0	0	1.026376	0	1.792616
AC011773.	0	0	0.999134	1.039293	0	0	0.84968	0	0	0.960699	0	0	1.028084	0	0	0	2.052753	0.910133	0.896308
AC011773.	10.56998	9.003514	14.987	0	15.50479	6.851322	6.797442	2.778081	4.669151	30.74238	15.66181	1.840156	26.73019	4.653602	8.802052	3.297807	2.052753	1.820267	5.377848
AC011773.	31.70995	24.00937	83.92723	30.13949	39.86946	36.54038	38.23561	58.3397	65.36811	47.07426	42.16642	54.28462	40.09529	36.29809	55.74633	18.13794	34.89679	55.51814	34.95601
AC011815.	350.7312	211.0824	463.598	298.277	273.5488	124.4657	202.2239	240.767	157.5838	248.8211	233.7224	344.1093	291.976	326.6828	376.5322	258.0534	209.3808	406.8296	267.0998
AC011815.	76.8726	155.0605	169.8527	40.53242	176.0901	171.2831	61.17698	168.5369	142.4091	172.9259	222.8797	177.5751	184.0271	159.1532	129.0968	81.62073	86.21561	102.8451	105.7643
AC011815.	201.7906	275.1074	287.7505	263.9804	347.7503	252.357	406.1472	300.0328	121.3979	293.0133	265.0461	384.5927	345.4363	228.0265	343.28	224.2509	357.1789	252.107	372.8641
AC011816.	0.960908	6.002343	3.996535	1.039293	0	0	0	0	0	7.685594	1.204755	3.680313	2.056169	0	0.978006	11.54233	7.184634	7.281067	0
AC011816.	31.70995	24.00937	15.98614	9.353635	38.76197	27.40529	11.89552	12.96438	64.20083	15.37119	9.638039	26.68227	21.58977	20.47585	24.45015	18.96239	2.052753	31.85467	12.54831
AC011824.	0	0	0	1.039293	1.107485	0	0.84968	2.778081	1.167288	0	0	0	1.028084	0	0.978006	0.824452	2.052753	0	0
AC011824.	0	0	0	0	0	0	0	0	0	0	0	0.920078	0	0	0	0.824452	0	0	0
AC011825.	3.84363	1.00039	13.98787	15.58939	4.42994	11.41887	0.84968	0.926027	0	2.882098	2.40951	7.360626	7.196591	4.653602	12.71408	9.893422	1.026376	1.820267	6.274156
AC011825.	97.05166	110.0429	97.9151	74.82908	139.5431	63.94567	187.7793	179.6492	5.836439	83.58083	37.3474	160.0936	150.1003	67.01186	120.2947	81.62073	90.32111	142.8909	62.74156
AC011825.	10.56998	9.003514	35.96881	15.58939	13.28982	5.709435	20.39233	26.85478	22.17847	32.66377	25.29985	23.00196	14.39318	18.61441	19.56012	8.244518	5.131881	20.93307	29.57816
AC011840.	0	0	0.999134	0	0	1.141887	0	24.0767	0	13.44979	33.73314	0	0	0	0	0.824452	0	0	0
AC011840.	8.648168	5.001952	11.9896	2.078586	0	9.135096	0.84968	4.630135	21.01118	9.606993	18.07132	0.920078	2.056169	2.792161	3.912023	6.595615	15.39564	12.74187	8.963079
AC011840.	6.726353	7.002733	10.99047	1.039293	0	5.709435	0.84968	5.556162	4.669151	7.685594	2.40951	9.200782	13.3651	18.61441	3.912023	5.771163	1.026376	6.370934	9.859387
AC011840.	26.90541	42.0164	76.93329	17.66798	59.80419	25.12151	40.78465	29.63287	25.68033	42.27077	68.67103	27.60235	30.84253	9.307203	31.29619	29.68027	18.47477	30.0344	42.12647
AC011840.	20.17906	7.002733	29.97401	1.039293	4.42994	4.567548	9.346483	31.48492	0	12.48909	32.52838	10.12086	23.64594	12.09936	10.75806	3.297807	6.158258	14.56213	25.09662
AC011853.	0	0	0	0	0	0	0	0	0	0	0	0.920078	0	0	0	0	0	0	0.896308
AC011890.	0	0	0.999134	2.078586	13.28982	0	1.69936	0	0	0.960699	0	0	0	1.861441	0	9.893422	10.26376	10.9216	15.23724
AC011891.	0	0	0	0	0	0	0	9.26027	0	0	1.204755	0	0	0.93072	0	0	0	3.640534	0
AC011893.	0	0	0	0	0	0	0	0	7.003726	0	0	0	0	0	0	0	0	0	0
AC011897.	0	0	0	0	2.21497	0	0.84968	0	0	0	0	0	0	0	0	0	0	0	0.896308
AC011899.	0	7.002733	0	4.157171	0	0	6.797442	0	0	9.606993	1.204755	0.920078	8.224675	9.307203	13.69208	4.946711	4.105505	2.7304	5.377848
AC011899.	143.1752	90.03514	163.8579	197.4656	91.92125	192.9789	76.47122	119.4575	249.7996	121.0481	173.4847	138.0117	80.19058	172.1833	108.5586	563.1006	349.9943	752.6803	512.6881
AC011899.	14.41361	17.00664	4.995668	38.45383	5.537425	17.12831	14.44456	2.778081	38.5205	24.97818	34.93789	1.840156	5.140422	24.19873	16.6261	32.97807	34.89679	40.04587	27.78555
AC011900.	1.921815	5.001952	1.998267	3.117878	0	2.283774	1.69936	12.96438	0	0	0	5.520469	1.028084	7.445762	0.978006	0	19.50115	0	0
AC011904.	9.609075	15.00586	0	0	3.322455	0	5.098081	5.556162	2.334575	3.842797	0	0	1.028084	0	0.978006	0	4.105505	0.910133	4.48154
AC011912.	475.6492	455.1776	410.6439	958.2279	295.6985	280.9042	662.7506	425.9724	46.69151	179.6508	131.3183	392.8734	365.998	367.6345	363.8182	384.1946	395.1549	324.9176	448.154
AC011921.	78.79442	40.01562	65.94282	43.6503	32.11706	76.50643	46.73241	134.2739	68.86998	54.75986	72.28529	57.96493	46.2638	80.97267	21.51613	53.58937	49.26606	60.97894	60.94894
AC011921.	0.960908	0	0	0	1.107485	0	0	0	0.960699	0	3.680313	0	6.515042	0.978006	0	4.105505	1.820267	0	
AC011933.	0	4.001562	2.997401	2.078586	6.64491	3.425661	9.346483	2.778081	21.01118	0	4.81902	5.520469	5.140422	11.16864	3.912023	1.648904	1.026376	2.7304	8.963079
AC011933.	4.804538	4.001562	0	11.43222	12.18233	4.567548	5.098081	11.11232	0	6.724895	1.204755	5.520469	7.196591	3.722881	9.780058	11.54233	2.052753	5.4608	1.792616
AC011933.	2.882723	10.0039	15.98614	20.78586	12.18233	20.55397	16.9936	0.926027	28.01491	16.33189	16.86657	17.48149	10.28084	13.03008	6.846041	16.48904	17.4484	17.29253	30.47447
AC011933.	0	2.000781	0	3.117878	0	0	0	0.926027	0	0	0	0	0	0	0	1.648904	1.026376	0	0.896308
AC011939.	32.67086	9.003514	29.97401	10.39293	23.25718	6.851322	16.9936	15.74246	21.01118	22.09608	49.39495	13.80117	24.67402	10.23792	28.36217	12.36678	19.50115	6.370934	6.274156
AC011939.	1.921815	0	1.998267	2.078586	0	0	20.39233	9.26027	0	0	19.27608	0	7.196591	0.93072	6.846041	0.824452	8.21101	0.910133	0
AC011944.	11.53089	16.00625	26.97661	10.39293	14.3973	15.98642	62.87634	16.66849	5.836439	15.37119	15.66181	39.56336	39.06721	14.89152	15.64809	4.946711	24.63303	27.304	17.02985
AC011944.	12.4918	14.00547	19.98267	19.74656	5.537425	1.141887	27.18977	0.926027	1.167288	6.724895	8.433284	28.52243	22.61786	6.515042	36.18622	11.54233	19.50115	11.83173	25.99293
AC011978.	3.84363	6.002343	5.994802	6.235757	4.42994	3.425661	0.84968	1.852054	0	1.921399	12.04755	9.200782	6.168506	0.93072	3.912023	1.648904	6.158258	7.281067	2.688924

AC011978.	373.793	504.1968	426.6301	489.5069	314.5257	443.0522	384.0555	425.0464	296.4911	523.5811	343.3551	237.3802	304.313	375.0803	372.6202	463.3419	592.2191	334.9291	320.8782
AC011979.	2.882723	13.00508	20.98181	7.275049	0	5.709435	4.248401	7.408216	0	3.842797	2.40951	18.40156	12.33701	5.584322	7.824047	7.420066	4.105505	11.83173	10.7557
AC011979.	0	1.00039	0	0	0	0	0	0	0	0	0	0	0	0	0.978006	0	0	0	0
AC011995.	120.1134	113.0441	78.93156	78.98625	83.06137	75.36454	126.6024	38.89314	31.51677	78.77734	34.93789	97.52829	74.02207	99.58707	82.15249	86.56744	90.32111	57.33841	59.15632
AC011997.	0	3.001171	0	3.117878	5.537425	0	0	0	0	0	0	0.920078	0	1.861441	6.846041	0	0	6.370934	0.896308
AC011998.	0	0	0	3.117878	0	0	0	0	0	0	0	0	0	20.47585	8.802052	0	0	0	0
AC011998.	0.960908	0	0.999134	2.078586	0	0	3.398721	3.704108	0	0	0	0	0	0	0	0.824452	0	0	0.896308
AC011998.	0.960908	1.00039	0	1.039293	0	0	0	0	0	0	0	0	0	0	0	0	0	0	0
AC011999.	6.726353	0	0.999134	0	0	0	1.69936	0	9.338302	0.960699	0	0.920078	2.056169	0.93072	0.978006	0	0	1.820267	1.792616
AC012005.	0.960908	0	1.998267	0	0	0	0	0	0	0	0	0	0	0	0	0	0	0	0
AC012005.	0	0	0	0	2.21497	0	0.84968	0	0	0	2.40951	0.920078	0	0	0	0	0	0.910133	1.792616
AC012020.	126.8398	188.0734	220.8085	275.4126	193.8099	211.2491	186.9297	254.6574	82.87743	244.9783	266.2508	280.6239	276.5547	160.0839	182.8871	248.9845	245.3039	257.5678	225.8696
AC012038.	0	0	0	0	0	0	0.84968	0	0	3.842797	0	0	0	0	0	0	0	0	0
AC012038.	0	0	4.995668	1.039293	0	1.141887	0	0	0	0	0	1.840156	0	0	0	0.824452	0	0	0
AC012044.	0	0	0	0	0	0	0	0	0	0	0	0.920078	0	0	0.978006	0	0	0	0
AC012048.	0	0	0	0	1.107485	1.141887	0	0	0	3.842797	0	0	0	0	0	0.824452	0	0	0
AC012050.	0	1.00039	0	1.039293	4.42994	2.283774	2.549041	0	9.338302	0	0	1.840156	3.084253	0	0	0	0	0.910133	1.792616
AC012055.	0	13.00508	3.996535	1.039293	8.859879	1.141887	6.797442	0.926027	11.67288	0	1.204755	5.520469	1.028084	24.19873	10.75806	2.473355	0	12.74187	8.066772
AC012065.	0	0	3.996535	0	6.64491	0	2.549041	0	0	0	0	0	0	0	0.978006	0	0	0	0
AC012065.	24.9836	10.0039	9.991337	12.47151	11.07485	4.567548	18.69297	8.334243	0	8.646293	0	15.64133	18.50552	3.722881	5.868035	12.36678	4.105505	3.640534	10.7557
AC012065.	88.40349	75.02928	134.883	48.84676	194.9173	81.07398	98.56291	60.19176	170.424	98.95202	59.03299	24.84211	42.15146	66.08114	86.06451	28.03136	34.89679	30.0344	26.88924
AC012065.	28.82723	47.01835	34.96968	27.02161	37.65449	35.3985	32.28785	37.96711	38.5205	31.70308	21.68559	23.92203	22.61786	13.03008	26.40616	17.31349	33.87042	19.1128	10.7557
AC012066.	7.68726	10.0039	95.91683	11.43222	34.33203	31.97284	15.29424	41.67122	64.20083	86.46293	171.0752	24.84211	16.44935	4.653602	19.56012	35.45143	28.73854	15.47227	12.54831
AC012073.	173.9243	222.0867	162.8588	109.1257	149.5105	212.391	175.8838	135.1999	179.7623	205.5896	214.4464	258.542	246.7402	166.5989	166.261	255.5801	218.6181	303.0744	266.2035
AC012074.	116.2698	122.0476	92.91943	209.9371	52.05179	103.9117	111.3081	110.1972	138.9072	106.6376	80.71858	142.6121	149.0722	113.5479	123.2287	150.8747	173.4576	127.4187	145.2019
AC012078.	0	0	0	0	0	0	0	0	0	0	4.81902	0.920078	0	0	0.978006	0	0	0	0
AC012081.	0	1.00039	1.998267	0	0	1.141887	0	0	0	0	0	0	0	0	0	0.824452	0	0	0
AC012085.	25.9445	44.01718	73.93589	64.43615	58.6967	33.11472	50.13113	59.26573	38.5205	43.23147	43.37118	40.48344	28.78636	54.9125	55.74633	47.81821	76.97822	61.88907	62.74156
AC012085.	80.71623	70.02733	166.8553	84.18271	168.3377	151.871	90.06611	137.978	203.1081	124.8909	67.46627	62.56532	76.07824	88.41843	119.3167	114.5988	106.7431	87.37281	87.83818
AC012100.	8.648168	5.001952	4.995668	2.078586	1.107485	7.993209	5.098081	4.630135	1.167288	4.803496	0	5.520469	2.056169	0	1.956012	5.771163	1.026376	0.910133	5.377848
AC012100.	2.882723	0	0	0	0	1.141887	0	0	3.501863	0	2.40951	4.600391	0	0	0	0	0	0	2.688924
AC012100.	0	0	2.997401	0	4.42994	0	3.398721	3.704108	4.669151	5.764196	0	0.920078	2.056169	0	5.868035	4.122259	1.026376	0.910133	0
AC012103.	15.37452	4.001562	3.996535	19.74656	2.21497	10.27698	15.29424	7.408216	0	6.724895	4.81902	11.04094	13.3651	1.861441	7.824047	7.420066	12.31652	10.01147	19.71877
AC012123.	17.29634	1.00039	0	0	0	7.993209	10.19616	3.704108	0	1.921399	4.81902	1.840156	8.224675	19.54513	3.912023	1.648904	3.079129	5.4608	0
AC012123.	0.960908	0	0.999134	0	0	0	0	0.926027	0	0	0	0	0	2.792161	3.912023	0	0	0	0
AC012146.	135.488	210.082	657.43	151.7367	219.282	277.4785	107.0597	328.7396	492.5954	778.1664	650.5676	158.2535	204.5888	275.4932	236.6774	196.2195	259.6732	162.0037	171.1948
AC012146.	0	1.00039	0	1.039293	4.42994	7.993209	0.84968	3.704108	11.67288	0	0	4.600391	1.028084	0	0.978006	0	1.026376	1.820267	1.792616
AC012146.	20.17906	4.001562	21.98094	16.62868	8.859879	14.84453	20.39233	21.29862	10.50559	13.44979	1.204755	5.520469	13.3651	33.50593	19.56012	2.473355	9.237386	11.83173	25.99293
AC012146.	1.921815	3.001171	52.95408	0	6.64491	0	0	0	22.17847	102.7948	159.0276	0.920078	2.056169	1.861441	0	0	1.026376	0.910133	2.688924
AC012150.	82.63805	54.02108	48.95755	175.6405	89.70628	82.21586	96.01387	195.3917	33.85134	65.32755	61.4425	35.88305	27.75828	57.70466	56.72434	46.99375	45.16056	29.12427	14.34093
AC012150.	63.4199	113.0441	44.96101	60.27898	63.12664	190.6951	59.47762	181.5013	36.18592	20.17468	65.05676	35.88305	54.48847	94.00275	55.74633	25.55801	20.52753	1.820267	8.963079
AC012157.	26.90541	14.00547	27.97574	9.353635	7.752394	9.135096	18.69297	4.630135	3.501863	15.37119	0	6.440548	10.28084	19.54513	15.64809	4.946711	4.105505	8.191201	8.066772
AC012158.	93.20803	63.0246	108.9056	70.67191	122.9308	75.36454	101.9616	72.23011	116.7288	77.81664	71.08054	85.56728	88.41526	66.08114	108.5586	75.84957	73.89909	84.64241	112.0385
AC012170.	11.53089	1.00039	2.997401	2.078586	29.90209	11.41887	10.19616	15.74246	19.84389	12.48909	15.66181	4.600391	10.28084	4.653602	7.824047	9.893422	4.105505	4.550667	14.34093
AC012170.	172.9634	164.064	247.7851	170.444	170.5527	189.5532	237.9105	220.3944	46.69151	178.6901	191.556	196.8967	177.8586	150.7767	234.7214	134.3856	130.3498	204.78	161.3354
AC012170.	51.88901	36.01406	81.92896	57.1611	57.58922	50.24303	56.0789	46.30135	39.68778	78.77734	60.23774	75.44642	55.51656	52.12034	68.46041	46.1693	45.16056	54.608	38.54124
AC012173.	7.68726	13.00508	5.994802	9.353635	7.752394	18.27019	14.44456	7.408216	7.003726	5.764196	7.228529	9.200782	12.33701	11.16864	15.64809	22.2602	16.42202	16.3824	11.652
AC012174.	0	0	0	0	0	1.141887	1.69936	0	0	0	7.228529	1.840156	0	2.792161	0	4.122259	0	0	0
AC012181.	3.84363	9.003514	6.993936	11.43222	6.64491	22.83774	11.89552	20.37259	40.85507	3.842797	7.228529	11.04094	9.252759	9.307203	8.802052	7.420066	10.26376	7.281067	10.7557
AC012181.	337.2785	313.1222	449.6101	125.7544	434.1341	180.4181	346.6695	343.556	101.554	252.6639	130.1135	388.273	541.8005	248.5023	367.7302	192.0973	163.1938	249.3766	113.8311
AC012181.	509.281	513.2003	562.5123	195.387	559.2799	275.1948	485.1674	476.9039	115.5615	270.9172	178.3037	565.8481	741.2488	414.1705	608.3196	317.414	283.2798	475.0896	252.7588
AC012182.	0.960908	0	0	0	0	0	0	0	0	0	0	0	0	0	0	0	1.026376	0	0
AC012183.	0	1.00039	0	2.078586	2.21497	0	1.69936	7.408216	3.501863	0	2.40951	0.920078	0	0	3.912023	3.297807	9.237386	1.820267	0
AC012184.	122.0353	118.0461	176.8467	121.5973	162.8003	149.5872	130.8508	189.8355	157.5838	153.7119	125.2945	141.692	108.9769	144.2616	159.4149	110.4765	94.42662	121.9579	161.3354
AC012186.	0.960908	0	1.998267	10.39293	0	0	5.098081	0.926027	0	1.921399	0	0.920078	5.140422	0.93072	0	2.473355	2.052753	1.820267	1.792616
AC012186.	72.06806	84.0328	98.91423	78.98625	71.98652	51.38492	130.8508	47.22738	28.01491	87.42363	42.16642	85.56728	92.52759	67.01186	74.32844	50.29156	51.31881	109.216	75.28987
AC012186.	0	1.00039	0	0	11.07485	3.425661	0	0.926027	0	8.646293	3.614265	2.760235	0	0	0.978006	2.473355	0	0.910133	0.896308
AC012213.	307.4904	181.0707	137.8804	229.6837	55.37425	194.1208	130.8508	141.6821	36.18592	87.42363	98.7899	172.9747	169.6339	156.361	170.173	270.4202	245.3039	113.7667	204.3582
AC012213.	43.24084	74.02889	47.95842	112.2436	5.537425	39.96605	37.38593	12.03835	24.51304	30.74238	46.98544	31.28266	42.15146	9.307203	46.94428	42.8715	69.79359	47.32694	72.60094
AC012213.	12.4918	38.01484	41.96361	27.02161	31.00958	44.53359	34.83689	29.63287	24.51304	48.03496	43.37118	28.52243	32.8987	32.57521	17.6041	14.01568	17.4484	13.652	26.88924
AC012213.	84.55986	94.0367	69.93936	73.78979	83.06137	68.51322	64.5757	37.96711	89.88116	67.24895	39.75691	45.08383	45.23571	41.88241	59.65836	103.0565	85.18923	43.6864	75.28987
AC012229.	0	0	0	0	3.322455	0	0	0	0	0	0	0	0	0	0	0	0	0	0

AC012236.	182.5724	166.0648	256.7774	184.9941	214.8521	182.7019	164.838	149.0904	242.7958	246.8997	313.2363	265.9026	316.65	176.8369	232.7654	231.671	149.8509	445.9654	474.1469
AC012254.	1.921815	14.00547	7.993069	10.39293	39.86946	1.141887	16.9936	31.48492	43.18965	13.44979	20.48083	12.8811	18.50552	16.75297	17.6041	7.420066	13.34289	13.652	22.4077
AC012254.	72.06806	71.02772	124.8917	152.776	170.5527	25.12151	166.5373	196.3177	32.68406	50.91706	62.64725	129.731	117.2016	83.76483	96.82258	129.4389	82.1101	126.5085	122.7942
AC012254.	23.06178	12.00469	4.995668	29.1002	16.61227	10.27698	14.44456	12.96438	5.836439	21.13538	16.86657	7.360626	16.44935	7.445762	16.6261	37.10033	37.97592	15.47227	4.48154
AC012254.	48.04538	63.0246	86.92463	85.22201	190.4874	68.51322	64.5757	111.1232	178.595	62.44545	136.1373	63.4854	63.74123	78.18051	76.28445	79.14738	79.03097	57.33841	29.57816
AC012291.	3.84363	0	0	0	4.42994	0	0.84968	0	0	0	0	0.920078	0	7.445762	0	0	1.026376	0	0
AC012301.	0.960908	1.00039	0	1.039293	0	0	2.549041	0	0	0	0	2.760235	0	2.792161	0	0.824452	0	3.640534	0
AC012306.	64.3808	92.03592	71.93762	62.35757	57.58922	42.24982	65.42538	50.93149	70.03726	45.15286	38.55216	56.12477	102.8084	58.63538	60.63636	75.02512	60.5562	80.09174	70.80833
AC012307.	0	0	0	0	0	3.425661	2.549041	0	0	0.960699	0	0.920078	0	1.861441	0	0.824452	5.131881	0.910133	0.896308
AC012308.	1.921815	2.000781	3.996535	0	4.42994	2.283774	5.098081	0	12.84017	0	4.81902	0	0	3.722881	0	0.824452	6.158258	3.640534	2.688924
AC012309.	3.84363	0	1.998267	4.157171	1.107485	4.567548	2.549041	1.852054	1.167288	0.960699	0	0.920078	7.196591	3.722881	3.912023	0	11.29014	5.4608	1.792616
AC012309.	1.921815	3.001171	1.998267	0	0	0	2.549041	0	8.171014	1.921399	0	0.920078	2.056169	0	0.978006	0	1.026376	0.910133	2.688924
AC012312.	0	0	0	0	0	0	0	0.926027	0	8.646293	0	0	0	0	0	0	0	0	0
AC012313.	1351.036	1113.435	1382.801	1279.369	799.6041	1326.873	1391.776	1232.542	862.6256	953.9744	1107.17	1460.164	1359.128	1114.072	1176.541	1503.8	1524.169	1331.525	1549.716
AC012313.	42.27993	22.00859	33.97054	18.70727	11.07485	25.12151	19.54265	9.26027	42.02236	57.64196	72.28529	39.56336	29.81445	5.584322	14.67009	28.85581	33.87042	32.7648	40.33386
AC012313.	136.4489	148.0578	115.8995	172.5226	165.0153	211.2491	163.1386	121.3095	214.7809	185.415	186.737	230.9396	224.1224	91.21059	99.75659	163.2415	218.6181	172.0152	237.5216
AC012313.	603.4499	744.2905	482.5816	680.7368	665.5984	520.7005	543.7954	507.4628	839.2799	537.0309	616.8345	507.8832	501.7052	503.5197	414.6745	543.3138	530.6365	525.147	442.7761
AC012313.	66.30262	81.03163	95.91683	159.0118	134.0057	74.22266	115.5565	106.4931	71.20455	72.05244	86.74235	80.04681	102.8084	98.65635	95.84457	92.3386	93.40024	102.8451	86.94187
AC012313.	690.8925	620.2421	727.3693	784.666	652.3086	641.7405	633.0118	668.5915	417.889	647.5113	534.9112	580.5694	660.0302	533.3027	591.6935	713.1508	643.5379	566.103	472.3543
AC012313.	143.1752	75.02928	179.8441	47.80747	48.72934	98.20228	135.0992	131.4958	123.7325	194.0612	122.885	104.8889	124.3982	147.0538	165.283	68.4295	107.7695	145.6213	157.7502
AC012313.	145.097	92.03592	253.78	141.3438	137.3281	62.80379	113.8572	100.0109	105.0559	118.166	131.3183	179.4153	123.3701	134.0237	178.9751	152.5236	174.484	212.9712	177.469
AC012313.	41.31902	110.0429	101.9116	74.82908	46.51437	42.24982	79.86994	75.93422	50.19337	43.23147	30.11887	91.08774	90.47142	53.98178	96.82258	60.18498	57.47707	67.34987	98.59387
AC012317.	11.53089	15.00586	1.998267	4.157171	0	18.27019	13.59488	10.1863	31.51677	6.724895	28.91412	18.40156	15.42127	7.445762	14.67009	5.771163	6.158258	4.550667	10.7557
AC012322.	0	0	0	0	0	0	0	0	0	0	0	0	0	0	0.978006	0	1.026376	0.910133	0
AC012339.	0	0	0.999134	0	0	0	0	0	0	0	0	0.920078	0	0	0	0	0	0.910133	0
AC012349.	18.25724	1.00039	4.995668	10.39293	21.04221	1.141887	13.59488	2.778081	0	0	2.40951	18.40156	9.252759	10.23792	1.956012	6.595615	1.026376	0.910133	9.859387
AC012354.	10.56998	0	2.997401	0	4.42994	19.41208	5.098081	0.926027	2.334575	0	9.638039	4.600391	16.44935	10.23792	8.802052	3.297807	6.158258	7.281067	8.066772
AC012354.	0.960908	7.002733	5.994802	1.039293	6.64491	3.425661	2.549041	0.926027	0	0.960699	0	3.680313	0	0.93072	5.868035	0	2.052753	3.640534	0.896308
AC012358.	41.31902	25.00976	64.94369	59.23969	67.55658	22.83774	57.77826	31.48492	29.18219	58.60265	45.78069	31.28266	43.17954	41.88241	49.8783	75.84957	41.05505	116.4971	137.1351
AC012358.	100.8953	109.0426	107.9064	65.47544	43.19191	77.64832	77.3209	95.38078	103.8886	131.6158	224.0844	101.2086	60.65698	85.62627	106.6026	115.4233	112.9014	84.64241	138.9277
AC012358.	99.93438	113.0441	88.9229	125.7544	140.6506	111.9049	124.903	153.7205	44.35693	128.7337	168.6657	107.6492	98.6961	119.1322	133.0088	78.32292	90.32111	60.97894	60.94894
AC012360.	7.68726	6.002343	2.997401	9.353635	2.21497	13.70264	0.84968	0	15.17474	20.17468	1.204755	6.440548	3.084253	2.792161	5.868035	4.946711	5.131881	1.820267	4.48154
AC012360.	26.90541	48.01874	25.97748	34.29666	76.41646	22.83774	14.44456	54.6356	44.35693	43.23147	48.1902	42.3236	16.44935	34.43665	30.31818	32.15362	25.65941	15.47227	17.92616
AC012360.	871.5431	1055.412	1219.942	1145.301	993.414	873.5436	1068.048	922.3229	999.1983	783.9306	650.5676	992.7644	1040.421	941.8889	867.4912	948.1196	845.734	1045.743	916.0267
AC012363.	14.41361	9.003514	0.999134	27.02161	2.21497	5.709435	3.398721	5.556162	0	2.882098	1.204755	14.72125	1.028084	9.307203	6.846041	7.420066	9.237386	0	0.896308
AC012366.	0	0	3.996535	0	0	1.141887	8.496802	0	0	7.685594	2.40951	1.840156	6.168506	6.515042	0	0	5.131881	4.550667	8.066772
AC012368.	4398.074	4140.616	4698.926	3157.371	5024.659	4068.543	4137.943	3602.245	7373.757	4714.151	6363.515	2855.003	2863.215	4245.946	4325.72	2094.108	2538.228	3000.71	2960.505
AC012368.	19.21815	44.01718	29.97401	28.0609	34.33203	23.97963	40.78465	33.33697	31.51677	9.606993	1.204755	27.60235	18.50552	43.74385	58.68035	25.55801	14.36927	10.9216	17.02985
AC012368.	1.921815	1.00039	0	1.039293	1.107485	0	1.69936	7.408216	1.167288	2.882098	3.614265	2.760235	0	12.09936	4.890029	3.297807	0	0.910133	1.792616
AC012370.	0	0	2.997401	1.039293	0	1.141887	2.549041	0.926027	0	0	2.40951	0.920078	9.252759	1.861441	2.934017	4.946711	0	0.910133	1.792616
AC012370.	0	1.00039	4.995668	3.117878	5.537425	10.27698	1.69936	12.96438	19.84389	10.56769	28.91412	8.280704	5.140422	2.792161	12.71408	7.420066	8.21101	22.75334	10.7557
AC012377.	23.06178	2.000781	10.99047	4.157171	4.42994	6.851322	12.7452	4.630135	18.6766	10.56769	8.433284	14.72125	4.112337	8.376483	6.846041	4.946711	9.237386	11.83173	2.688924
AC012378.	0	1.00039	0	0	0	1.141887	0	0	0	0	0	0	0	0	0	0	0	0	0
AC012400.	0.960908	0	3.996535	0	5.537425	0	0.84968	2.778081	2.334575	4.803496	4.81902	0.920078	0	0	0	0	1.026376	0.910133	0
AC012404.	0.960908	0	0.999134	9.353635	1.107485	6.851322	0.84968	0	0	0.960699	4.81902	7.360626	1.028084	0	0	0	11.29014	0.910133	2.688924
AC012404.	5.765445	0	0	7.275049	0	14.84453	1.69936	3.704108	8.171014	5.764196	0	7.360626	1.028084	4.653602	0.978006	7.420066	5.131881	0.910133	1.792616
AC012409.	22.10087	16.00625	34.96968	12.47151	22.1497	25.12151	33.98721	3.704108	85.212	19.21399	48.1902	20.24172	7.196591	13.9608	10.75806	15.66458	9.237386	19.1128	6.274156
AC012409.	25.9445	25.00976	10.99047	3.117878	12.18233	12.56076	8.496802	8.334243	7.003726	20.17468	18.07132	30.36258	20.56169	32.57521	17.6041	14.84013	5.131881	30.0344	10.7557
AC012409.	289.2332	225.0878	168.8536	164.2083	344.4278	318.5865	237.9105	153.7205	867.2948	493.7994	665.0247	379.9923	432.8235	233.6108	314.9179	119.5455	149.8509	142.8909	172.9874
AC012409.	48.04538	26.01015	24.97834	15.58939	24.36467	10.27698	11.89552	31.48492	1.167288	37.46727	19.27608	18.40156	18.50552	12.09936	10.75806	12.36678	11.29014	22.75334	20.61508
AC012409.	78.79442	184.0718	188.8363	78.98625	450.7464	198.6883	79.02026	112.0493	766.908	454.4107	708.3959	143.5322	140.8476	163.8068	90.95454	48.64266	56.45069	35.4952	40.33386
AC012413.	0	2.000781	1.998267	0	4.42994	10.27698	1.69936	0.926027	0	2.882098	0	0	2.056169	2.792161	1.956012	0	0	0	0
AC012414.	0	0	0	0	0	0	0	0	0	0	0	0	0	0	0	0	0	0	1.792616
AC012414.	0	0	0	0	0	222.668	0	0	0	0	0	0	0	0	0	386.6679	468.0276	0	0
AC012414.	0.960908	0	0.999134	0	0	1.141887	0	1.852054	0	0	0	0.920078	1.028084	0	0	0.824452	0	0	0.896308
AC012414.	143.1752	329.1285	317.7245	168.3654	208.2072	82.21586	186.08	206.504	123.7325	72.05244	59.03299	266.8227	297.1164	137.7466	146.7009	43.69595	69.79359	142.8909	232.1438
AC012414.	0.960908	2.000781	6.993936	7.275049	0	0	0	0	22.17847	0	0	1.840156	0	13.03008	14.67009	1.648904	0	1.820267	0
AC012414.	3.84363	3.001171	15.98614	11.43222	2.21497	3.425661	10.19616	0	0	3.842797	9.638039	7.360626	4.112337	6.515042	17.6041	11.54233	3.079129	8.191201	21.51139
AC012417.	12.4918	2.000781	1.998267	9.353635	0	7.993209	3.398721	1.852054	0	0	7.228529	1.840156	3.084253	12.09936	0	1.648904	4.105505	3.640534	0.896308
AC012435.	65.34171	40.01562	63.94455	88.33989	48.72934	41.10793	53.52986	54.6356	40.85507	61.48475	59.03299	77.28657	49.34805	51.18962	67.4824	53.58937	66.71446	75.54107	62.74156
AC012435.	20.17906	9.003514	2.997401	8.314342	7.752394	6.851322	1.69936	22.22465	32.68406	35.54587	21.68559	10.12086	17.47743	9.307203	5.868035	9.06897	17.4484	8.191201	5.377848
AC012441.	12.4918	17.00664	17.98441	5.196464	18.82724	15.98642	8.496802	13.89041	70.03726	43.23147	84.33284	29.4425	17.47743	24.19873	11.73607	12.36678	18.47477	29.12427	18.82247

AC012441.	12.4918	17.00664	17.98441	4.157171	18.82724	15.98642	8.496802	13.89041	73.53913	43.23147	84.33284	28.52243	17.47743	23.26801	11.73607	11.54233	18.47477	29.12427	20.61508
AC012442.	34.59267	35.01367	36.96795	23.90373	33.22455	36.54038	29.73881	36.11505	23.34575	40.34937	42.16642	43.24368	34.95487	40.95169	52.81231	47.81821	19.50115	41.86614	40.33386
AC012442.	40.35812	36.01406	9.991337	41.57171	53.15928	59.37812	33.13753	44.4493	94.55031	52.83846	103.6089	28.52243	23.64594	31.64449	41.07624	48.64266	56.45069	50.05734	37.64493
AC012442.	0	0	4.995668	9.353635	7.752394	1.141887	14.44456	4.630135	0	5.764196	1.204755	12.8811	17.47743	7.445762	5.868035	10.71787	10.26376	1.820267	14.34093
AC012443.	0.960908	4.001562	0	0	0	0	0.84968	0	0	0	0	0	0	1.861441	0.978006	0	0	0.910133	0
AC012445.	0	0	8.992203	0	6.64491	1.141887	0.84968	0	0	0	0	2.760235	2.056169	0	0.978006	4.122259	0	0	0
AC012447.	9.609075	21.0082	23.97921	22.86444	16.61227	43.39171	13.59488	45.37532	2.334575	27.86028	25.29985	10.12086	20.56169	9.307203	8.802052	32.97807	31.81766	13.652	16.13354
AC012454.	3.84363	5.001952	0.999134	1.039293	0	2.283774	1.69936	0	0	7.685594	0	0.920078	0	3.722881	10.75806	0.824452	6.158258	1.820267	1.792616
AC012456.	0	0	0	5.196464	0	2.283774	5.098081	0	0	3.842797	0	1.840156	0	3.722881	0	0	1.026376	0	5.377848
AC012456.	54.77173	28.01093	52.95408	24.94303	42.08443	18.27019	21.24201	36.11505	28.01491	24.01748	18.07132	26.68227	33.92678	23.26801	36.18622	19.78684	37.97592	30.0344	26.88924
AC012462.	0	0	0.999134	0	0	0	0	0	0	0	0.960699	2.40951	0.920078	0	0	0	0	0	0
AC012462.	7.68726	9.003514	12.98874	2.078586	2.21497	5.709435	2.549041	1.852054	3.501863	10.56769	9.638039	5.520469	7.196591	8.376483	6.846041	4.122259	7.184634	10.9216	7.170464
AC012464.	71.10716	41.01601	65.94282	27.02161	76.41646	54.81058	73.0725	50.93149	31.51677	27.86028	9.638039	35.88305	45.23571	40.95169	35.20821	23.08465	33.87042	36.40534	52.88217
AC012464.	12.4918	9.003514	17.98441	2.078586	9.967364	19.41208	11.04584	4.630135	19.84389	9.606993	3.614265	7.360626	19.5336	12.09936	7.824047	14.01568	5.131881	10.01147	2.688924
AC012464.	63.4199	94.0367	114.9004	11.43222	49.83682	98.20228	125.7527	71.30408	124.8998	58.60265	91.56137	54.28462	74.02207	46.53602	60.63636	45.34485	22.58028	50.96747	34.0597
AC012467.	35.55358	39.01523	73.93589	18.70727	18.82724	11.41887	41.63433	33.33697	0	49.95636	7.228529	47.84407	35.98295	26.99089	41.07624	34.62698	48.23968	42.77627	26.88924
AC012467.	1229.962	1057.413	1002.131	1425.91	822.8613	1433.068	1227.788	975.1065	1394.909	1368.996	1156.565	989.0841	1057.899	1038.684	976.0498	1178.966	1276.812	914.6841	924.0935
AC012468.	0	1.00039	0	0	0	0	0	0	0	0	0	0	0	0	0	0	0	3.640534	0
AC012485.	4.804538	23.00898	2.997401	10.39293	24.36467	15.98642	12.7452	14.81643	4.669151	25.93888	15.66181	3.680313	9.252759	8.376483	4.890029	10.71787	20.52753	5.4608	11.652
AC012485.	0	1.00039	0	0	0	0	0.84968	0	0	0	0	0.920078	2.056169	0	0	0	0	0.910133	0
AC012485.	7.68726	14.00547	7.993069	0	6.64491	7.993209	4.248401	0.926027	16.34203	0.960699	0	5.520469	11.30893	17.68369	10.75806	15.66458	4.105505	8.191201	2.688924
AC012486.	12.4918	19.00742	1.998267	10.39293	19.93473	7.993209	10.19616	15.74246	16.34203	8.646293	9.638039	7.360626	14.39318	4.653602	7.824047	12.36678	8.21101	8.191201	16.13354
AC012488.	4.804538	0	0	0	0	0	0	0	0	0	0	0	0	0	0	0	0	0	0
AC012493.	0	0	0.999134	0	0	0	2.549041	0	0	0	0	0	0	0	0	0	0	0	8.963079
AC012498.	0.960908	0	0	0	0	1.141887	1.69936	3.704108	0	0	0	0	1.028084	0	0.978006	0.824452	0	7.281067	0
AC012498.	2.882723	0	0	0	0	0	0	0	1.167288	0	0	0.920078	0	0	0.978006	0	0	0	0
AC012499.	0.960908	11.00429	5.994802	7.275049	13.28982	11.41887	17.84329	7.408216	2.334575	3.842797	1.204755	1.840156	2.056169	40.95169	25.42815	3.297807	0	2.7304	3.585232
AC012501.	3.84363	17.00664	3.996535	2.078586	4.42994	15.98642	23.79105	6.482189	7.003726	23.05678	13.2523	0.920078	2.056169	0	7.824047	12.36678	9.237386	1.820267	6.274156
AC012506.	0	0	0	0	0	1.141887	0	0	0	0	0	0.920078	0	0	0	0	0	0	0.896308
AC012506.	0.960908	4.001562	0	1.039293	0	1.141887	3.398721	3.704108	0	3.842797	0	0	1.028084	0	5.868035	0.824452	0	0.910133	0.896308
AC012506.	0	4.001562	0	0	3.322455	6.851322	5.098081	4.630135	2.334575	0	0	0.920078	4.112337	0	0	0.824452	2.052753	0.910133	0.896308
AC012506.	2.882723	0	0	2.078586	14.3973	5.709435	5.098081	3.704108	4.669151	5.764196	6.023774	1.840156	4.112337	1.861441	1.956012	4.122259	9.237386	4.550667	2.688924
AC012507.	109.5435	22.00859	52.95408	9.353635	58.6967	37.68227	23.79105	20.37259	121.3979	35.54587	34.93789	28.52243	35.98295	31.64449	41.07624	12.36678	1.026376	2.7304	5.377848
AC012508.	8.648168	43.01679	25.97748	90.41847	52.05179	69.65511	16.9936	11.11232	18.6766	20.17468	40.96167	14.72125	15.42127	14.89152	1.956012	6.595615	28.73854	18.20267	26.88924
AC012508.	9.609075	51.01991	36.96795	51.96464	64.23413	36.54038	52.68017	18.52054	26.84762	71.09174	163.8467	50.6043	32.8987	48.39746	46.94428	29.68027	31.81766	55.51814	97.69757
AC012510.	7.68726	11.00429	12.98874	11.43222	19.93473	17.12831	9.346483	9.26027	58.36439	31.70308	49.39495	21.1618	12.33701	12.09936	7.824047	10.71787	19.50115	12.74187	6.274156
AC012511.	12.4918	35.01367	32.97141	19.74656	6.64491	14.84453	29.73881	23.15068	30.34948	41.31007	24.0951	44.16376	21.58977	29.78305	13.69208	38.74924	15.39564	24.5736	48.40063
AC012512.	8.648168	21.0082	24.97834	8.314342	17.71976	18.27019	7.647122	24.0767	4.669151	6.724895	12.04755	14.72125	11.30893	5.584322	14.67009	10.71787	11.29014	6.370934	15.23724
AC012513.	0	4.001562	0.999134	10.39293	1.107485	2.283774	2.549041	0.926027	0	0	3.614265	4.600391	5.140422	1.861441	4.890029	4.122259	3.079129	0	0
AC012513.	0	0	2.997401	0	0	9.135096	0	5.556162	0	3.842797	1.204755	0	2.056169	0	0.978006	0.824452	0	8.191201	0
AC012513.	1411.573	1310.511	1588.623	2612.782	1488.46	881.5368	2835.383	1503.868	748.2314	617.7296	604.7869	1691.104	1667.553	1364.436	1299.77	1627.468	1258.337	1444.382	1561.368
AC012519.	0	3.001171	3.996535	0	4.42994	0	0.84968	9.26027	0	0	0	0	0	1.861441	0	0	1.026376	0	0
AC012531.	900.3704	670.2616	908.2125	485.3497	573.6772	389.3835	814.8434	642.6628	175.0932	541.8344	373.474	1015.766	1033.225	608.6911	676.78	548.2605	525.5046	701.7129	842.5295
AC012531.	0	0	0	0	0	0	0	0	0	0	0	2.760235	0	1.861441	4.890029	0	0	0.910133	0
AC012531.	0	0	0	0	0	0	0	0	7.003726	0	0	0.920078	0	5.584322	0.978006	0	0	0	0
AC012555.	0	0	0	0	0	1.141887	3.398721	0	0	2.882098	0	0	0	0.93072	2.934017	0	0	1.820267	1.792616
AC012557.	178.7288	258.1007	251.7817	344.0059	210.4221	221.5261	282.0938	195.3917	168.0894	255.546	138.5468	261.3022	217.9539	128.4394	156.4809	222.602	240.172	180.2064	191.8099
AC012557.	46.12356	20.00781	25.97748	32.21808	45.40688	43.39171	23.79105	55.56162	95.71759	43.23147	42.16642	34.96297	42.15146	20.47585	40.09824	25.55801	71.84634	42.77627	30.47447
AC012557.	8.648168	19.00742	14.987	13.51081	18.82724	5.709435	12.7452	26.85478	18.6766	17.29259	8.433284	16.56141	20.56169	16.75297	11.73607	14.84013	29.76491	15.47227	35.85232
AC012558.	5.765445	9.003514	17.98441	29.1002	48.72934	13.70264	3.398721	18.52054	11.67288	27.86028	21.68559	7.360626	2.056169	10.23792	0.978006	4.946711	4.105505	6.370934	9.859387
AC012560.	7.68726	2.000781	15.98614	2.078586	5.537425	1.141887	2.549041	0.926027	0	0	0	28.52243	30.84253	7.445762	11.73607	8.244518	14.36927	3.640534	1.792616
AC012568.	0	0	5.994802	0	0	0	0.84968	0	0	0	0	0	1.028084	0	0.978006	3.297807	6.158258	0.910133	0
AC012569.	24.9836	19.00742	34.96968	39.49313	12.18233	21.69585	49.28145	49.07943	10.50559	15.37119	9.638039	31.28266	42.15146	42.81313	49.8783	36.27588	47.21331	29.12427	47.50432
AC012574.	0	0	0	1.039293	0	0	0.84968	0	0	0.960699	0	0	0	0	0	0	5.131881	0.910133	0
AC012574.	0	0	0	0	0	4.567548	0	0	0	0	0	0	0	0	0	0	0	0	0
AC012593.	0	9.003514	1.998267	2.078586	0	0	0	5.556162	1.167288	0	0	0.920078	1.028084	9.307203	0.978006	1.648904	0	0	1.792616
AC012594.	5.765445	0	5.994802	5.196464	0	2.283774	0.84968	0	9.338302	0	3.614265	1.840156	1.028084	0	2.934017	0	0	4.550667	0.896308
AC012603.	33.63176	19.00742	51.95495	16.62868	16.61227	33.11472	31.43817	14.81643	17.50932	34.58517	33.73314	35.88305	32.8987	24.19873	47.92229	41.22259	15.39564	63.70934	49.29694
AC012613.	0	6.002343	3.996535	3.117878	4.42994	3.425661	0	0	4.669151	2.882098	2.40951	3.680313	1.028084	0	7.824047	0.824452	0	1.820267	0.896308
AC012613.	13.45271	2.000781	45.96015	18.70727	34.33203	9.135096	13.59488	39.81916	14.00745	12.48909	7.228529	31.28266	17.47743	7.445762	17.6041	27.20691	18.47477	20.93307	24.20031
AC012615.	171.0415	239.0933	211.8163	138.2259	245.8617	253.4989	162.2889	162.0547	473.9188	353.5373	404.7976	166.5342	246.7402	217.7886	193.6452	122.8433	149.8509	222.0726	218.6991
AC012615.	0	0	0	0	0	0	0	0	3.501863	0	0	0	0	0	0	0	0	0	0

AC012615.	24.9836	2.000781	5.994802	7.275049	23.25718	5.709435	14.44456	13.89041	1.167288	14.41049	6.023774	12.8811	9.252759	8.376483	0	14.01568	17.4484	30.0344	18.82247
AC012615.	327.6695	360.1406	288.7496	296.1984	439.6715	319.7284	390.0032	248.1752	381.7031	452.4893	665.0247	368.9514	387.5878	336.9207	348.1701	324.834	343.836	438.6843	452.6355
AC012617.	206.5951	222.0867	192.8328	111.2043	217.067	248.9314	174.1844	192.6136	434.231	284.367	337.3314	112.2495	149.0722	332.2671	356.9721	192.9217	255.5677	144.7112	116.52
AC012618.	0	0	0.999134	0	0	0	0.84968	0	1.167288	4.803496	6.023774	0	0	0	0	0	1.026376	0	1.792616
AC012618.	0.960908	4.001562	2.997401	1.039293	21.04221	12.56076	10.19616	5.556162	11.67288	11.52839	8.433284	43.24368	7.196591	11.16864	7.824047	16.48904	7.184634	22.75334	18.82247
AC012618.	115.3089	147.0574	87.92376	92.49706	172.7676	150.7291	111.3081	196.3177	72.37184	190.2185	189.1465	110.4094	81.21867	128.4394	107.5806	105.5298	123.1652	83.73227	121.0016
AC012625.	23.06178	27.01054	14.987	14.5501	28.79461	58.23624	0.84968	61.11778	35.01863	24.97818	25.29985	11.04094	22.61786	30.71377	33.2522	22.2602	14.36927	10.01147	10.7557
AC012629.	0	0	0	2.078586	0	0	0	0	0	0.960699	0	0	0	0	0	0	0	0	0.896308
AC012629.	0.960908	0	0.999134	0	0	6.851322	2.549041	0	1.167288	0	1.204755	0	0	0.93072	3.912023	2.473355	0	0.910133	2.688924
AC012629.	0	4.001562	0	0	0	0	0	0	3.501863	0	0	0	0	5.584322	0	0	0	0	0
AC012640.	4.804538	1.00039	0.999134	2.078586	0	5.709435	0.84968	5.556162	1.167288	7.685594	0	13.80117	2.056169	3.722881	3.912023	3.297807	5.131881	0.910133	0.896308
AC012640.	46.12356	70.02733	63.94455	74.82908	118.5009	101.6279	79.86994	71.30408	151.7474	103.7555	134.9325	80.96688	91.49951	90.27987	106.6026	66.7806	100.5849	89.19307	121.0016
AC012640.	0.960908	1.00039	4.995668	0	13.28982	1.141887	2.549041	8.334243	0	11.52839	0	1.840156	5.140422	8.376483	1.956012	0	5.131881	0	3.585232
AC012640.	12.4918	21.0082	17.98441	8.314342	9.967364	21.69585	3.398721	12.96438	50.19337	67.24895	18.07132	4.600391	11.30893	11.16864	8.802052	18.96239	7.184634	3.640534	8.963079
AC012640.	1.921815	4.001562	3.996535	6.235757	11.07485	0	10.19616	5.556162	35.01863	9.606993	3.614265	5.520469	4.112337	7.445762	3.912023	4.122259	12.31652	2.7304	6.274156
AC012645.	213.3215	165.0644	216.812	155.8939	180.52	190.6951	186.9297	170.389	305.8294	190.2185	275.8889	168.3743	189.1675	247.5716	206.3592	167.3637	199.117	182.9368	144.3056
AC012645.	45.16265	48.01874	16.98527	28.0609	16.61227	20.55397	24.64073	34.263	21.01118	15.37119	30.11887	37.72321	27.75828	29.78305	20.53812	37.92478	71.84634	22.75334	26.88924
AC012645.	32.67086	41.01601	23.97921	70.67191	57.58922	55.95246	43.33369	114.8274	42.02236	69.17035	78.30907	60.72516	59.62889	45.60529	29.34017	65.13169	79.03097	137.4301	104.868
AC012645.	165.2761	94.0367	84.92636	25.98232	107.426	134.7427	56.0789	142.6082	288.3201	269.9565	392.7501	109.4893	120.2859	84.69555	103.6686	85.74299	137.5344	91.01334	95.90495
AC012651.	735.0943	675.2635	937.1874	671.3831	747.5523	582.3624	755.3657	765.8244	494.93	720.5244	846.9427	874.9944	961.2589	601.2453	740.3504	641.4235	672.2764	640.7339	862.2482
AC012653.	0	0	0.999134	0	0	0	0.84968	0	11.67288	0	0	0.920078	0	7.445762	0	0	0	0.910133	0.896308
AC012668.	3.84363	0	0	0	0	1.141887	0	0	1.167288	0.960699	0	1.840156	1.028084	0	0	1.648904	0	0	0
AC012668.	0	0	0	1.039293	0	0	0	0	0	0	0	0.920078	0	0	0	0	0	0	0
AC012668.	8.648168	1.00039	10.99047	4.157171	7.752394	2.283774	3.398721	3.704108	0	0	0	4.600391	0	5.584322	1.956012	6.595615	6.158258	0.910133	8.066772
AC012676.	36.51449	28.01093	35.96881	21.82515	28.79461	27.40529	16.9936	24.0767	57.1971	62.44545	98.7899	37.72321	15.42127	32.57521	40.09824	34.62698	36.94955	38.2256	54.67478
AC012676.	4.804538	1.00039	13.98787	1.039293	3.322455	5.709435	3.398721	1.852054	9.338302	6.724895	0	10.12086	4.112337	7.445762	6.846041	9.06897	4.105505	4.550667	5.377848
AC012676.	19.21815	11.00429	12.98874	39.49313	9.967364	5.709435	22.94137	32.41095	16.34203	13.44979	13.2523	6.440548	14.39318	22.33729	24.45015	13.19123	1.026376	22.75334	24.20031
AC012676.	15.37452	9.003514	7.993069	22.86444	35.43952	14.84453	25.49041	15.74246	51.36066	20.17468	30.11887	11.04094	12.33701	4.653602	17.6041	27.20691	18.47477	13.652	10.7557
AC012676.	1230.923	787.3073	1094.051	1090.218	757.5197	554.9571	879.419	847.3147	379.3685	628.2973	457.8069	1076.492	1127.809	1073.121	945.7316	1009.129	998.6641	1301.491	1068.399
AC013244.	27.86632	11.00429	35.96881	13.51081	19.93473	18.27019	16.14392	28.70684	25.68033	17.29259	18.07132	23.00196	26.73019	29.78305	43.03226	9.893422	25.65941	33.67494	15.23724
AC013244.	1.921815	2.000781	0.999134	0	5.537425	0	0	1.852054	0	0	0	0.920078	3.084253	0	0	0	0	0	0.896308
AC013248.	0	2.000781	0	5.196464	0	0	0	0	0	3.842797	0	2.760235	0	0	0	0	0	0	0
AC013248.	51.88901	33.01288	19.98267	21.82515	18.82724	29.68906	16.14392	25.00273	16.34203	13.44979	13.2523	22.08188	14.39318	31.64449	22.49413	28.03136	32.84404	38.2256	12.54831
AC013264.	0	1.00039	0	1.039293	0	0	0	8.334243	0	0	0	0	0	0	0	4.122259	0	3.640534	1.792616
AC013267.	0	0	0	0	0	0	0	0.926027	1.167288	0	0	0.920078	2.056169	0	0	0.824452	1.026376	0	3.585232
AC013268.	4.804538	1.00039	1.998267	3.117878	3.322455	0	1.69936	9.26027	3.501863	4.803496	2.40951	1.840156	4.112337	13.9608	14.67009	9.06897	9.237386	8.191201	2.688924
AC013268.	0	0	0.999134	4.157171	1.107485	0	6.797442	0	0	14.41049	19.27608	1.840156	2.056169	11.16864	1.956012	0.824452	2.052753	0	2.688924
AC013268.	10.56998	4.001562	1.998267	1.039293	23.25718	0	0	5.556162	5.836439	4.803496	0	5.520469	11.30893	0.93072	0	5.771163	2.052753	0	4.48154
AC013268.	0	0	0	0	0	0	0	0	0	0	0	0	0	0.93072	0.978006	0	1.026376	0.910133	0
AC013270.	3.84363	4.001562	0	1.039293	0	0	1.69936	2.778081	18.6766	0	14.45706	1.840156	0	0	0.978006	4.946711	8.21101	7.281067	0
AC013271.	3.84363	2.000781	0.999134	3.117878	2.21497	3.425661	7.647122	2.778081	2.334575	0	3.614265	10.12086	3.084253	7.445762	4.890029	6.595615	2.052753	8.191201	0.896308
AC013275.	17.29634	13.00508	17.98441	46.76817	1.107485	49.10114	19.54265	22.22465	14.00745	9.606993	30.11887	8.280704	13.3651	2.792161	4.890029	29.68027	11.29014	15.47227	17.02985
AC013283.	4.804538	3.001171	9.991337	4.157171	11.07485	4.567548	5.947762	1.852054	7.003726	0	8.433284	1.840156	2.056169	4.653602	0.978006	0	1.026376	4.550667	1.792616
AC013286.	0	2.000781	0	4.157171	6.64491	0	0	5.556162	0	0	0	0	0	0	0	0	3.079129	4.550667	0
AC013290.	0	0	13.98787	5.196464	1.107485	0	2.549041	0	0	0	0	0	0	3.722881	5.868035	0.824452	1.026376	0	0
AC013355.	0	6.002343	0.999134	0	1.107485	1.141887	1.69936	11.11232	0	2.882098	0	0	1.028084	3.722881	7.824047	0	1.026376	0.910133	1.792616
AC013356.	1.921815	3.001171	0.999134	0	1.107485	2.283774	0.84968	1.852054	1.167288	4.803496	6.023774	0	0	0	0.978006	0	0	0	0.896308
AC013356.	33.63176	54.02108	60.94715	9.353635	32.11706	71.93888	15.29424	52.78354	23.34575	55.72056	43.37118	77.28657	41.12337	28.85233	32.27419	21.43575	25.65941	49.1472	34.95601
AC013356.	12.4918	3.001171	1.998267	0	1.107485	6.851322	5.947762	1.852054	0	0	0	3.680313	1.028084	8.376483	0.978006	15.66458	4.105505	2.7304	3.585232
AC013391.	0	0	0	0	0	0	0	0	0	1.921399	0	0	0	0	0	0	0	0	0
AC013391.	0	0	2.997401	0	0	1.141887	0	0	0	0	0	0	0	0	1.956012	0	1.026376	0	0.896308
AC013391.	0.960908	12.00469	3.996535	0	0	0	5.098081	5.556162	11.67288	13.44979	4.81902	0.920078	0	7.445762	3.912023	6.595615	1.026376	3.640534	1.792616
AC013394.	128.7616	200.0781	376.6734	73.78979	238.1093	264.9178	227.7143	504.6847	30.34948	328.5591	244.5652	292.5849	461.6099	145.1924	185.8211	79.97183	59.52982	49.1472	73.49725
AC013399.	0	0	0	0	0	0	0	0	0	0	0	0	0	3.722881	0	0	1.026376	0	0
AC013400.	12.4918	18.00703	26.97661	2.078586	6.64491	22.83774	7.647122	12.96438	16.34203	20.17468	19.27608	24.84211	12.33701	12.09936	17.6041	22.2602	17.4484	10.9216	26.88924
AC013402.	0.960908	1.00039	0	0	0	0	0	0	0	0	0	0	1.028084	0	0	0	0	0	0
AC013403.	4.804538	13.00508	14.987	5.196464	13.28982	9.135096	3.398721	3.704108	19.84389	21.13538	19.27608	13.80117	11.30893	10.23792	6.846041	8.244518	13.34289	10.9216	6.274156
AC013403.	245.9923	242.0945	208.8189	378.3026	297.9134	372.2552	396.8007	231.5068	246.2977	244.0176	234.9272	277.8636	284.7794	251.2945	206.3592	230.8465	337.6778	189.3078	224.077
AC013417.	0	0	0	0	0	2.283774	0.84968	0	4.669151	0	0	0	0	0	0	0	0	0	0
AC013429.	11.53089	7.002733	2.997401	11.43222	6.64491	6.851322	3.398721	0.926027	0	10.56769	31.32363	5.520469	10.28084	1.861441	5.868035	1.648904	11.29014	0	0
AC013429.	0	1.00039	0	0	0	0	0	0	0	0	0	0	0	0.93072	0	6.595615	0	0	0
AC013436.	0	2.000781	0	0	0	0	0	7.408216	1.167288	0	0	0	0	0	0	0	0	0	0.896308

AC013437.	6.726353	9.003514	0	0	7.752394	0	1.69936	4.630135	1.167288	0.960699	0	0.920078	2.056169	5.584322	3.912023	2.473355	0	1.820267	0.896308
AC013437.	0	0	0	0	0	0	0	0	0	0	0	0	0	0	0	0	0	10.9216	3.585232
AC013439.	4.804538	0	0	12.47151	12.18233	2.283774	0.84968	0.926027	35.01863	3.842797	14.45706	2.760235	3.084253	21.40657	16.6261	0	2.052753	0.910133	0
AC013444.	6.726353	1.00039	7.993069	4.157171	0	4.567548	12.7452	6.482189	16.34203	15.37119	0	0.920078	4.112337	17.68369	2.934017	0.824452	0	0.910133	1.792616
AC013448.	1.921815	5.001952	2.997401	7.275049	4.42994	20.55397	15.29424	1.852054	0	22.09608	13.2523	5.520469	9.252759	3.722881	3.912023	2.473355	1.026376	4.550667	2.688924
AC013448.	0	0	0	0	0	0	0	0	0	0	0	0	1.028084	0	0	0	1.026376	0	0
AC013451.	16.33543	111.0433	67.94109	21.82515	4.42994	19.41208	21.24201	12.96438	50.19337	42.27077	46.98544	34.04289	34.95487	24.19873	36.18622	24.73355	48.23968	18.20267	26.88924
AC013451.	10.56998	19.00742	13.98787	13.51081	16.61227	7.993209	4.248401	11.11232	21.01118	8.646293	1.204755	11.96102	6.168506	1.861441	2.934017	9.893422	7.184634	16.3824	5.377848
AC013452.	0.960908	0	3.996535	0	1.107485	1.141887	0.84968	0.926027	5.836439	0	0	0	3.084253	0	0.978006	0.824452	0	0.910133	1.792616
AC013452.	27.86632	44.01718	53.95322	66.51474	34.33203	22.83774	80.71962	21.29862	17.50932	11.52839	20.48083	43.24368	32.8987	27.92161	53.79032	37.92478	40.02867	36.40534	35.85232
AC013457.	0.960908	0	0.999134	0	6.64491	0	0.84968	0	0	0	0	0	0	0	0	6.595615	5.131881	0	0
AC013460.	27.86632	46.01796	33.97054	23.90373	49.83682	28.54718	48.43177	15.74246	11.67288	30.74238	12.04755	29.4425	24.67402	13.9608	23.47214	19.78684	24.63303	45.50667	30.47447
AC013467.	0	0	0	0	1.107485	2.283774	0	0	0	4.803496	0	6.440548	0	0	0	3.297807	0	0	0
AC013468.	81.67714	81.03163	65.94282	119.5187	88.59879	79.93209	63.72602	60.19176	96.88488	82.62014	54.21397	67.16571	56.54464	48.39746	63.57038	84.09409	92.37386	48.23707	62.74156
AC013470.	0	0	0	0	0	2.283774	0	0	0	0	0	0	0	0	0	0	0	0	0
AC013470.	7.68726	5.001952	4.995668	14.5501	0	17.12831	0.84968	0	9.338302	0	0	4.600391	5.140422	3.722881	1.956012	0	1.026376	0	4.48154
AC013470.	3.84363	3.001171	0.999134	0	0	0	0.84968	0	0	0	0	0.920078	0	0	0	0.824452	3.079129	3.640534	4.48154
AC013470.	0	0	0	6.235757	0	0	0	0	0	0	0	0	0	0	0	0	0	0	0
AC013472.	5.765445	8.003124	23.97921	11.43222	19.93473	22.83774	17.84329	12.96438	22.17847	16.33189	1.204755	27.60235	19.5336	13.9608	13.69208	27.20691	13.34289	16.3824	28.68185
AC013472.	18.25724	68.02655	15.98614	51.96464	26.57964	57.09435	32.28785	29.63287	30.34948	16.33189	24.0951	20.24172	26.73019	26.06017	12.71408	42.8715	47.21331	19.1128	29.57816
AC013472.	134.5271	197.0769	104.909	134.0688	134.0057	170.1412	132.5501	152.7945	324.506	155.6333	237.3367	120.5302	101.7804	161.9453	129.0968	108.0032	182.695	98.29441	116.52
AC013474.	2.882723	1.00039	17.98441	5.196464	25.47215	0	11.04584	8.334243	7.003726	9.606993	0	16.56141	7.196591	8.376483	6.846041	6.595615	11.29014	7.281067	14.34093
AC013476.	3.84363	10.0039	8.992203	9.353635	16.61227	7.993209	16.14392	4.630135	3.501863	1.921399	3.614265	5.520469	8.224675	3.722881	8.802052	6.595615	10.26376	25.48374	5.377848
AC013476.	1.921815	12.00469	0	1.039293	0	0	0	0	4.669151	0.960699	0	0	0	0.93072	6.846041	0	1.026376	0.910133	1.792616
AC013480.	4.804538	3.001171	1.998267	9.353635	0	0	5.947762	4.630135	0	0	0	0.920078	6.168506	0	0.978006	0.824452	6.158258	0	0
AC013488.	0	4.001562	0	1.039293	0	1.141887	0	0	0	0	0	0.920078	0	0.93072	6.846041	0	3.079129	0	0.896308
AC013489.	0	1.00039	0	0	0	0	1.69936	0	0	0	0	0.920078	0	0	0	0	0	0.910133	0
AC013489.	1.921815	0	0.999134	0	1.107485	0	1.69936	1.852054	0	2.882098	1.204755	1.840156	0	1.861441	0.978006	0	0	0	0.896308
AC013546.	0	1.00039	8.992203	10.39293	6.64491	9.135096	0.84968	0	0	0	0	0	5.140422	0	10.75806	3.297807	1.026376	5.4608	21.51139
AC013549.	2.882723	2.000781	5.994802	3.117878	12.18233	5.709435	1.69936	5.556162	40.85507	0.960699	14.45706	1.840156	0	3.722881	6.846041	0.824452	2.052753	3.640534	1.792616
AC013549.	0	0	0	0	0	1.141887	10.19616	0	0	0	0	0	0	1.861441	0	0	0	0.910133	0
AC013549.	0	5.001952	2.997401	0	6.64491	0	1.69936	0	0	0	0	0	1.028084	0	0	0	0	0	0
AC013553.	13.45271	20.00781	15.98614	45.72888	3.322455	3.425661	14.44456	12.96438	0	16.33189	14.45706	17.48149	19.5336	10.23792	14.67009	17.31349	12.31652	24.5736	32.26709
AC013553.	1.921815	4.001562	10.99047	2.078586	0	6.851322	0	17.59451	0	9.606993	1.204755	0.920078	2.056169	1.861441	3.912023	8.244518	0	2.7304	1.792616
AC013553.	123.9571	233.091	217.8111	212.0157	423.0592	228.3774	154.6418	118.5315	798.4248	600.437	1319.207	184.0156	208.7011	81.90339	88.02052	176.4327	218.6181	212.0611	251.8625
AC013553.	11.53089	12.00469	7.993069	22.86444	25.47215	13.70264	3.398721	10.1863	36.18592	23.05678	51.80446	11.96102	12.33701	11.16864	6.846041	20.6113	21.5539	13.652	31.37078
AC013562.	1.921815	1.00039	0.999134	7.275049	0	0	1.69936	0	0	0	0	1.840156	2.056169	7.445762	0	2.473355	1.026376	0.910133	3.585232
AC013564.	3.84363	0	2.997401	1.039293	0	4.567548	0	0	0	0	0	0.920078	0	0	0	12.36678	1.026376	0	1.792616
AC013565.	441.0566	1597.624	845.2671	436.503	1220.448	516.1329	780.0065	783.4189	818.2687	935.7211	880.6758	792.1874	820.4113	1189.461	1265.54	260.5268	290.4645	901.0321	1350.736
AC013565.	3.84363	1.00039	0	2.078586	4.42994	0	0.84968	0	0	0	0	0	0	0	0	0.824452	0	0	0.896308
AC013565.	46.12356	92.03592	61.94629	55.08252	46.51437	21.69585	57.77826	48.15341	92.21573	45.15286	59.03299	43.24368	45.23571	42.81313	40.09824	28.03136	51.31881	41.86614	39.43755
AC013640.	0	0	2.997401	2.078586	1.107485	0	0.84968	3.704108	0	2.882098	0	0.920078	1.028084	3.722881	3.912023	0	0	0	3.585232
AC013643.	0.960908	1.00039	0	0	0	1.141887	0.84968	0	0	3.842797	0	0.920078	0	0	0	0	1.026376	0	0
AC013643.	106.6607	42.0164	47.95842	37.41454	38.76197	27.40529	66.27506	45.37532	82.87743	41.31007	21.68559	51.52438	57.57272	45.60529	23.47214	11.54233	7.184634	30.94454	28.68185
AC013643.	240.2269	127.0496	198.8276	81.06484	228.1419	127.8913	226.0149	217.6164	117.8961	172.9259	121.6802	281.5439	228.2347	142.4002	184.8431	78.32292	85.18923	140.1605	132.6536
AC013644.	0	2.000781	0.999134	0	0	0	0.84968	0	0	5.764196	0	2.760235	0	3.722881	0.978006	0.824452	0	0	2.688924
AC013652.	13.45271	27.01054	16.98527	2.078586	14.3973	1.141887	11.04584	1.852054	16.34203	0	21.68559	14.72125	4.112337	14.89152	11.73607	9.893422	20.52753	10.9216	8.066772
AC013652.	0	0	0	0	6.64491	0	0	0	0	0	0	0	0	0	0	0	0	0	0
AC013714.	0.960908	2.000781	5.994802	1.039293	1.107485	0	1.69936	9.26027	0	2.882098	0	4.600391	2.056169	0	1.956012	4.946711	1.026376	0.910133	2.688924
AC013724.	6.726353	1.00039	0.999134	0	11.07485	9.135096	24.64073	0.926027	5.836439	6.724895	3.614265	7.360626	2.056169	0.93072	3.912023	10.71787	11.29014	10.01147	13.44462
AC013726.	24.02269	31.0121	24.97834	25.98232	53.15928	17.12831	17.84329	7.408216	39.68778	32.66377	30.11887	22.08188	14.39318	19.54513	16.6261	26.38246	32.84404	43.6864	50.19325
AC013727.	1.921815	0	0	0	0	0	0	0	0	0	0	0	0	0	0	0.824452	0	0	0
AC013731.	24.02269	54.02108	102.9108	28.0609	54.26676	90.20907	45.03305	78.7123	151.7474	119.1267	162.6419	79.12673	42.15146	62.35826	55.74633	43.69595	23.60665	60.97894	35.85232
AC013734.	0	11.00429	0	0	0	0	0	0	0	0	0	0	0	0	0	0	0	0	0
AC013762.	0	1.00039	2.997401	0	3.322455	9.135096	2.549041	0	0	0	0	3.680313	1.028084	0.93072	2.934017	0	1.026376	0	0.896308
AC013787.	61.49808	66.02577	103.9099	73.78979	91.92125	66.22945	99.41259	81.49038	30.34948	100.8734	55.41872	65.32555	101.7804	60.49682	79.21847	43.69595	49.26606	47.32694	77.97879
AC013791.	0.960908	0	0	0	1.107485	0	0	0	0	3.842797	0	0	0	0	0	0	0	0	0
AC013799.	0	0	0	2.078586	2.21497	1.141887	0	0	0	0	0	0	0	0	0	0	0	0	0.896308
AC013828.	0	0	0	0	0	0	0	0	0	5.764196	0	0	0	0	0	0	0	0	0
AC015468.	0	0	2.997401	0	0	3.425661	0	0	0	0.960699	0	0	1.028084	0	0	9.06897	9.237386	1.820267	0.896308
AC015468.	0	0	0	0	0	0	1.69936	0	0	3.842797	0	0	2.056169	0	0	3.297807	2.052753	0.910133	0
AC015468.	2.882723	0	0	0	1.107485	0	0	0	0	0	0	9.200782	4.112337	0.93072	0.978006	7.420066	8.21101	4.550667	5.377848

AC015522.	0	0	0	0	0	0	0	1.852054	0	0	0	0	0	0	0	0	0	0	0
AC015524.	0	0	0	0	0	0	0.84968	0	0	0	0	0.920078	0	0	0	0	0	0	0
AC015540.	129.7225	233.091	124.8917	181.8762	100.7811	138.1683	163.9883	128.7178	194.9371	200.7861	115.6565	96.60821	87.38717	133.093	145.7229	105.5298	89.29473	113.7667	113.8311
AC015563.	10.56998	15.00586	36.96795	9.353635	13.28982	10.27698	16.9936	24.0767	18.6766	14.41049	25.29985	9.200782	19.5336	14.89152	12.71408	7.420066	8.21101	10.01147	6.274156
AC015563.	0	0	0	6.235757	0	0	1.69936	0.926027	0	0	0	0	1.028084	0	0	0	0	0	0
AC015574.	1.921815	11.00429	1.998267	1.039293	3.322455	9.135096	14.44456	0.926027	0	3.842797	0	5.520469	3.084253	0.93072	1.956012	3.297807	4.105505	1.820267	0
AC015656.	134.5271	175.0683	120.8952	130.9509	157.2629	97.0604	279.5448	103.715	131.9035	146.0263	133.7278	352.39	238.5156	174.9754	115.4047	269.5957	259.6732	235.7246	236.6253
AC015660.	13.45271	20.00781	33.97054	86.2613	22.1497	45.67548	38.23561	39.81916	1.167288	31.70308	24.0951	25.76219	26.73019	48.39746	41.07624	49.46711	66.71446	8.191201	28.68185
AC015660.	7.68726	8.003124	11.9896	18.70727	23.25718	2.283774	7.647122	11.11232	0	15.37119	2.40951	6.440548	5.140422	6.515042	11.73607	19.78684	15.39564	8.191201	3.585232
AC015660.	1.921815	12.00469	0.999134	5.196464	24.36467	2.283774	6.797442	18.52054	4.669151	15.37119	7.228529	7.360626	3.084253	9.307203	13.69208	7.420066	15.39564	3.640534	5.377848
AC015660.	6.726353	5.001952	31.97228	14.5501	21.04221	13.70264	2.549041	8.334243	23.34575	16.33189	30.11887	8.280704	10.28084	1.861441	5.868035	17.31349	7.184634	0.910133	0
AC015660.	93.20803	52.0203	65.94282	64.43615	59.80419	43.39171	12.7452	102.789	88.71387	26.89958	72.28529	106.7291	77.10633	87.48771	32.27419	89.86525	89.29473	103.7552	61.84525
AC015688.	8.648168	15.00586	7.993069	12.47151	11.07485	4.567548	3.398721	2.778081	4.669151	7.685594	6.023774	3.680313	7.196591	4.653602	6.846041	6.595615	5.131881	4.550667	1.792616
AC015688.	0	0	0	0	0	0	0	1.852054	0	0	0	0	0	0	0	0	0	0.910133	0
AC015688.	6.726353	4.001562	8.992203	15.58939	8.859879	1.141887	20.39233	9.26027	0	4.803496	1.204755	17.48149	10.28084	6.515042	0.978006	9.06897	20.52753	19.1128	19.71877
AC015689.	14.41361	27.01054	12.98874	19.74656	28.79461	5.709435	13.59488	25.00273	14.00745	14.41049	15.66181	12.8811	8.224675	13.03008	6.846041	17.31349	28.73854	38.2256	18.82247
AC015689.	0	1.00039	3.996535	0	0	0	4.248401	0	0	0	0	0	0	0	0.978006	0	0	0.910133	0
AC015691.	0	5.001952	30.97314	2.078586	0	3.425661	16.14392	10.1863	14.00745	12.48909	6.023774	13.80117	18.50552	1.861441	9.780058	15.66458	17.4484	11.83173	1.792616
AC015712.	122.9962	84.0328	118.8969	56.12181	96.35119	109.6212	33.13753	101.863	82.87743	123.9302	132.523	95.68814	107.9489	95.86419	76.28445	79.14738	70.81996	135.6099	86.04556
AC015712.	662.0653	558.2179	598.4811	665.1474	717.6502	534.4031	908.3082	663.0354	688.6998	502.4457	533.7064	1029.568	948.9219	669.1879	569.1994	1271.305	1040.746	912.8638	941.1233
AC015712.	0	0	2.997401	0	0	0	0	0	0	0	0	0	0	0	0	0	0	0	0
AC015712.	123.9571	62.02421	113.9012	92.49706	129.5757	89.06719	409.5459	99.08489	16.34203	118.166	115.6565	109.4893	161.4092	32.57521	49.8783	108.0032	58.50345	168.3747	166.7133
AC015712.	443.9393	131.0511	454.6058	188.112	300.1284	170.1412	398.5	265.7698	403.8816	286.2884	255.408	237.3802	209.7292	214.0657	174.085	333.903	463.9221	362.2331	284.1296
AC015712.	53.81082	31.0121	56.95062	57.1611	45.40688	63.94567	124.903	62.96984	32.68406	67.24895	122.885	97.52829	91.49951	63.28898	56.72434	52.76492	91.34749	45.50667	80.66772
AC015720.	0	0	0	1.039293	0	0	0	0	0	1.921399	0	0	0	0	0.978006	0	0	0	0
AC015720.	0	0	0	0	13.28982	0	0.84968	5.556162	7.003726	3.842797	4.81902	0.920078	1.028084	0	0	4.946711	0	2.7304	3.585232
AC015722.	362.2621	482.1882	407.6465	479.114	586.967	668.0039	333.9243	455.6053	564.9673	506.2885	601.1727	305.466	384.5036	361.1195	364.7962	321.5362	356.1526	199.3192	288.6112
AC015726.	273.8586	265.1035	281.7557	306.5914	271.3338	443.0522	192.0277	199.0958	710.8782	320.8736	545.754	201.4971	179.9148	173.114	180.9311	256.4045	295.5964	212.9712	209.7361
AC015726.	8.648168	0	9.991337	2.078586	1.107485	1.141887	2.549041	5.556162	2.334575	1.921399	1.204755	0.920078	1.028084	6.515042	0	4.946711	3.079129	4.550667	1.792616
AC015726.	9.609075	4.001562	3.996535	0	4.42994	1.141887	3.398721	2.778081	0	7.685594	14.45706	1.840156	0	1.861441	0.978006	2.473355	1.026376	6.370934	0
AC015727.	36.51449	31.0121	112.9021	55.08252	99.67364	62.80379	79.86994	53.70957	25.68033	60.52405	43.37118	49.68422	55.51656	53.05106	49.8783	47.81821	45.16056	50.96747	66.32679
AC015743.	1.921815	5.001952	3.996535	3.117878	0	18.82724	0	1.69936	0	29.18219	0.960699	4.81902	6.440548	6.168506	1.861441	1.956012	0	7.184634	10.01147	0
AC015795.	0	0	3.996535	3.117878	0	6.851322	0	0	0	0.960699	0	0	0	1.861441	0	0	3.079129	0	0
AC015795.	6.726353	7.002733	9.991337	3.117878	6.64491	1.141887	5.098081	12.03835	0	16.33189	7.228529	4.600391	8.224675	17.68369	16.6261	9.06897	9.237386	8.191201	10.7557
AC015799.	133.5661	121.0472	179.8441	94.57564	161.6928	108.4793	122.354	210.2081	66.5354	85.50223	165.0514	113.1696	121.314	88.41843	90.95454	66.7806	35.92317	66.43974	52.88217
AC015801.	0.960908	0	0	2.078586	0	0	1.69936	1.852054	0	2.882098	0	0	1.028084	0	0.978006	2.473355	0	0	3.585232
AC015802.	165.2761	218.0851	164.8571	77.94696	134.0057	188.4114	194.5768	184.2794	211.2791	256.5067	325.2838	241.9806	319.7342	91.21059	140.8328	157.4703	156.0092	111.0363	96.80126
AC015802.	36.51449	10.0039	23.97921	10.39293	4.42994	20.55397	11.04584	17.59451	7.003726	11.52839	40.96167	12.8811	20.56169	15.82225	17.6041	4.946711	14.36927	12.74187	16.13354
AC015802.	201.7906	201.0785	207.8198	136.1474	217.067	256.9246	219.2175	230.5807	302.3275	261.3102	165.0514	344.1093	309.4534	320.1678	255.2595	179.7305	192.9587	256.6576	190.0173
AC015802.	121.0743	75.02928	86.92463	71.7112	139.5431	101.6279	99.41259	111.1232	75.8737	110.4804	68.67103	151.8129	164.4935	103.31	130.0748	95.63641	118.0333	118.3173	50.19325
AC015802.	30.74904	20.00781	15.98614	13.51081	17.71976	17.12831	22.94137	24.0767	24.51304	31.70308	21.68559	33.12282	28.78636	21.40657	19.56012	18.13794	18.47477	23.66347	12.54831
AC015802.	745.6642	959.3744	818.2905	248.391	1237.061	1370.264	587.9787	1023.26	2101.118	1833.014	3476.923	1547.572	1788.867	536.0949	657.2199	448.5018	614.7994	671.6785	1000.28
AC015804.	2.882723	1.00039	7.993069	5.196464	1.107485	1.141887	0	4.630135	0	9.606993	1.204755	1.840156	3.084253	6.515042	1.956012	1.648904	0	0	3.585232
AC015813.	5970.118	5642.202	6144.672	9088.615	6600.61	5672.895	7344.636	5855.269	5391.702	6326.205	5335.859	6369.702	6997.142	4314.819	4540.881	7337.621	7436.096	7214.628	6375.438
AC015813.	6.726353	1.00039	4.995668	1.039293	1.107485	0	5.098081	0	0	0	1.204755	11.96102	8.224675	5.584322	1.956012	3.297807	6.158258	3.640534	2.688924
AC015813.	2.882723	15.00586	6.993936	10.39293	16.61227	1.141887	1.69936	3.704108	18.6766	14.41049	31.32363	5.520469	5.140422	9.307203	4.890029	9.893422	11.29014	5.4608	11.652
AC015813.	174.8852	134.0523	146.8726	116.4008	146.188	129.0332	153.7921	127.7917	89.88116	183.4936	173.4847	168.3743	165.5216	153.5689	170.173	128.6145	169.3521	220.2523	212.425
AC015813.	5129.324	3801.484	4154.398	4546.906	4831.957	4367.718	4745.464	3634.656	3389.804	3863.932	3487.765	3278.239	3366.976	2922.462	3022.038	4185.742	4350.809	3149.062	3517.112
AC015813.	3.84363	12.00469	4.995668	0	8.859879	2.283774	7.647122	5.556162	0	0.960699	0	0	1.028084	4.653602	6.846041	4.122259	1.026376	4.550667	3.585232
AC015818.	2.882723	51.01991	74.93502	39.49313	14.3973	44.53359	25.49041	38.89314	9.338302	22.09608	9.638039	11.04094	10.28084	7.445762	19.56012	9.893422	2.052753	5.4608	2.688924
AC015818.	0	0	0	2.078586	0	0	0	0	0	0	0	0	5.140422	0	0	0	0	0	0
AC015818.	0	5.001952	11.9896	3.117878	3.322455	0	8.496802	16.66849	2.334575	0	7.228529	0.920078	1.028084	12.09936	9.780058	0	0	2.7304	0
AC015819.	36.51449	22.00859	8.992203	25.98232	7.752394	13.70264	21.24201	3.704108	45.52422	48.99566	15.66181	12.8811	30.84253	25.12945	28.36217	35.45143	25.65941	35.4952	15.23724
AC015819.	14.41361	3.001171	11.9896	2.078586	3.322455	12.56076	5.947762	13.89041	25.68033	7.685594	0	11.96102	17.47743	5.584322	10.75806	17.31349	19.50115	11.83173	13.44462
AC015819.	0	1.00039	5.994802	0	8.859879	4.567548	0.84968	0.926027	1.167288	1.921399	0	0	0	0	7.824047	1.648904	0	0	0
AC015820.	0.960908	0	0	1.039293	0	0	0	0	0	0	0	0	0	0	0	0.824452	2.052753	1.820267	0
AC015845.	4.804538	2.000781	11.9896	1.039293	3.322455	1.141887	0.84968	1.852054	7.003726	0.960699	1.204755	8.280704	0	1.861441	7.824047	4.946711	0	0.910133	0.896308
AC015845.	15.37452	14.00547	14.987	12.47151	19.93473	26.2634	14.44456	15.74246	26.84762	41.31007	18.07132	36.80313	24.67402	32.57521	26.40616	21.43575	50.29244	15.47227	12.54831
AC015849.	17.29634	26.01015	22.98007	24.94303	43.19191	65.08756	27.18977	43.52327	81.71014	48.99566	112.0422	24.84211	40.09529	16.75297	16.6261	24.73355	26.68578	9.101334	20.61508
AC015849.	3.84363	0	0	0	0	0	0	0	0	0	0	0	0	0	0	0	0	0	0
AC015849.	236.3833	214.0836	320.7219	448.9745	421.9518	189.5532	493.6642	361.1505	81.71014	276.6814	208.4226	494.082	379.3631	286.6619	368.7082	362.7588	271.9897	407.7398	394.3755
AC015849.	6.726353	4.001562	6.993936	12.47151	9.967364	2.283774	27.18977	13.89041	3.501863	0.960699	6.023774	9.200782	7.196591	7.445762	15.64809	7.420066	4.105505	3.640534	8.066772

AC015849.	8.648168	12.00469	12.98874	12.47151	1.107485	4.567548	14.44456	20.37259	4.669151	5.764196	12.04755	9.200782	3.084253	10.23792	11.73607	12.36678	7.184634	39.13574	19.71877
AC015849.	3.84363	2.000781	8.992203	4.157171	0	2.283774	5.947762	0	0	0	0	4.600391	3.084253	0.93072	0	2.473355	3.079129	3.640534	2.688924
AC015853.	20.17906	11.00429	36.96795	10.39293	24.36467	14.84453	60.3273	25.92876	11.67288	45.15286	39.75691	19.32164	32.8987	17.68369	34.2302	13.19123	16.42202	25.48374	27.78555
AC015853.	38.4363	27.01054	41.96361	69.63262	44.2994	42.24982	78.17058	20.37259	5.836439	15.37119	42.16642	52.44446	55.51656	36.29809	38.14223	35.45143	51.31881	32.7648	70.80833
AC015853.	2.882723	15.00586	19.98267	1.039293	9.967364	6.851322	16.9936	19.44657	0	10.56769	8.433284	6.440548	4.112337	11.16864	2.934017	5.771163	5.131881	2.7304	5.377848
AC015864.	0	0	0.999134	1.039293	0	0	0	0	0	0	1.204755	0.920078	0	0	0	0	0	0	0
AC015871.	0	1.00039	0	0	2.21497	0	0	0	0	0	0	0	0	0	0	0	0	0	0
AC015871.	1362.567	1145.447	1552.654	1162.969	1290.22	996.8674	1350.992	1216.8	811.265	1067.337	971.0324	1259.587	1296.414	1147.578	1293.902	1198.753	984.2948	1659.173	1472.634
AC015871.	3.84363	1.00039	0	4.157171	1.107485	4.567548	2.549041	0	0	7.685594	2.40951	1.840156	4.112337	0.93072	1.956012	3.297807	2.052753	1.820267	0.896308
AC015878.	0	0	0	0	0	0	0	0	0	0	0	0	0	0	0	1.648904	0	0.910133	0
AC015883.	2.882723	15.00586	11.9896	7.275049	17.71976	15.98642	23.79105	0	21.01118	9.606993	28.91412	5.520469	1.028084	1.861441	8.802052	13.19123	7.184634	1.820267	13.44462
AC015908.	4.804538	3.001171	5.994802	7.275049	1.107485	12.56076	14.44456	9.26027	5.836439	3.842797	7.228529	3.680313	7.196591	3.722881	3.912023	9.893422	5.131881	5.4608	3.585232
AC015908.	24.02269	48.01874	35.96881	34.29666	59.80419	36.54038	19.54265	42.59724	57.1971	56.68126	31.32363	31.28266	16.44935	21.40657	29.34017	53.58937	68.76721	32.7648	36.74863
AC015908.	113.3871	125.0488	149.87	122.6365	62.01916	172.4249	151.2431	108.3452	151.7474	139.3014	192.7608	173.8948	127.4825	132.1623	146.7009	136.859	147.7982	124.6883	139.824
AC015908.	8.648168	4.001562	0.999134	5.196464	8.859879	3.425661	1.69936	9.26027	0	3.842797	15.66181	0	2.056169	2.792161	10.75806	0.824452	0	3.640534	1.792616
AC015908.	791.7878	950.3709	456.6041	1096.454	513.873	1160.157	933.7986	708.4107	691.0343	741.6598	911.9994	720.4213	816.299	709.2089	650.3739	804.665	866.2616	506.9443	759.1728
AC015909.	3.84363	3.001171	16.98527	2.078586	2.21497	0	4.248401	3.704108	15.17474	6.724895	4.81902	11.04094	9.252759	13.03008	12.71408	6.595615	7.184634	22.75334	9.859387
AC015909.	83.59896	75.02928	58.94889	47.80747	91.92125	75.36454	61.17698	62.96984	121.3979	61.48475	86.74235	30.36258	55.51656	34.43665	22.49413	8.244518	36.94955	12.74187	15.23724
AC015909.	765.8433	1042.407	689.4022	376.224	723.1877	783.3345	768.1109	757.4901	1203.474	1228.734	1621.6	484.8812	634.3281	647.7813	703.1862	260.5268	293.5436	471.4491	734.9725
AC015909.	0	0	0	0	0	0	0	0.926027	0	0.960699	0	4.600391	1.028084	0.93072	0.978006	0.824452	0	6.370934	0
AC015909.	238.3051	324.1265	224.8051	235.9195	121.8233	246.6476	346.6695	172.241	353.6882	224.8036	236.132	302.7057	223.0943	190.7977	233.7434	272.8936	307.9129	146.5315	231.2475
AC015910.	4.804538	5.001952	3.996535	6.235757	0	2.283774	2.549041	6.482189	1.167288	0.960699	3.614265	0	1.028084	0.93072	0	7.420066	4.105505	7.281067	4.48154
AC015911.	0	1.00039	0.999134	0	0	0	0	0	0	0.960699	0	0	0	0	0	0	0	0.910133	1.792616
AC015911.	0	0	0	0	1.107485	0	0	0	0	0	0	0	0	1.861441	0	0	2.052753	0	0
AC015911.	62.45899	22.00859	61.94629	88.33989	36.547	105.0536	19.54265	52.78354	25.68033	59.56335	18.07132	102.1287	93.55568	83.76483	74.32844	117.0722	49.26606	52.78774	30.47447
AC015911.	0	1.00039	5.994802	11.43222	5.537425	0	0.84968	0.926027	0	1.921399	3.614265	1.840156	4.112337	2.792161	1.956012	8.244518	2.052753	7.281067	1.792616
AC015911.	0	0	0	0	0	0	0.84968	0	0	0	0	0.920078	0	0	0	0.824452	0	0	0
AC015911.	0.960908	1.00039	1.998267	0	0	1.141887	0	2.778081	0	0	0	0	2.056169	0	0	2.473355	2.052753	0	0
AC015911.	98.01257	41.01601	112.9021	70.67191	57.58922	140.4521	35.68657	125.9397	330.3424	160.4368	114.4517	100.2885	151.1284	149.846	112.4707	104.7054	67.74083	124.6883	52.88217
AC015911.	37.47539	21.0082	27.97574	66.51474	43.19191	47.95925	19.54265	79.63833	31.51677	42.27077	32.52838	69.00587	39.06721	39.09025	44.01026	54.41382	61.58258	48.23707	38.54124
AC015912.	0	4.001562	1.998267	1.039293	5.537425	2.283774	2.549041	0.926027	0	14.41049	3.614265	1.840156	0	3.722881	0.978006	0	4.105505	0.910133	7.170464
AC015912.	0.960908	0	0	1.039293	0	2.283774	0	0	5.836439	7.685594	12.04755	1.840156	0	0.93072	0	0	2.052753	7.281067	1.792616
AC015914.	24.9836	8.003124	63.94455	44.68959	27.68712	3.425661	56.0789	20.37259	2.334575	22.09608	24.0951	31.28266	20.56169	19.54513	20.53812	56.06272	53.37157	13.652	16.13354
AC015914.	3.84363	6.002343	5.994802	4.157171	9.967364	0	0.84968	0	0	1.921399	0	0	0	0	0	0.824452	7.184634	0	0
AC015917.	0	0	0	0	0	0	0	0	0	5.764196	0	0	0	0	0	0	0	0	0
AC015920.	48.04538	51.01991	102.9108	47.80747	178.3051	73.08077	58.62794	84.26846	57.1971	24.01748	13.2523	34.96297	26.73019	76.31906	43.03226	14.84013	37.97592	11.83173	15.23724
AC015921.	12.4918	12.00469	4.995668	7.275049	12.18233	7.993209	5.098081	1.852054	0	3.842797	7.228529	8.280704	4.112337	1.861441	5.868035	9.893422	10.26376	28.21414	8.066772
AC015922.	0	0	0.999134	0	0	4.567548	0	0	0	0	7.228529	0.920078	1.028084	0.93072	0	0.824452	0	0.910133	0.896308
AC015922.	24.02269	14.00547	15.98614	14.5501	48.72934	22.83774	4.248401	9.26027	10.50559	16.33189	26.50461	17.48149	37.01104	10.23792	18.58211	14.84013	17.4484	10.01147	11.652
AC015922.	284.4286	254.0992	253.78	318.0236	191.5949	319.7284	286.3422	181.5013	66.5354	220.9608	395.1596	296.2652	363.9419	194.5205	227.8574	385.8435	340.7569	263.9387	320.8782
AC015922.	773.5306	1076.42	1193.965	1115.161	1070.938	1581.514	866.6738	911.2106	1277.013	1346.9	1287.883	919.1582	1012.663	973.5334	977.0278	1158.355	1162.884	795.4566	753.795
AC015923.	2.882723	16.00625	32.97141	21.82515	4.42994	13.70264	28.88913	30.55889	0	19.21399	10.84279	5.520469	6.168506	4.653602	11.73607	3.297807	8.21101	5.4608	4.48154
AC015936.	0	4.001562	0.999134	0	1.107485	14.84453	5.947762	0	0	0.960699	0	1.840156	0	4.653602	0	1.648904	0	0	0
AC015936.	0.960908	4.001562	0.999134	4.157171	0	2.283774	2.549041	3.704108	0	0	0	0.920078	0	0	0.978006	1.648904	0	0	0
AC015959.	0	0	0	0	0	0	0	0	0	0	0	0.920078	0	0	0.978006	0	4.105505	0	0
AC015961.	204.6733	197.0769	172.8501	189.1513	84.16885	101.6279	229.4137	137.978	91.04844	100.8734	74.6948	182.1755	229.2628	128.4394	145.7229	166.5393	181.6686	172.0152	196.2914
AC015961.	48.04538	80.03124	68.94022	78.98625	118.5009	97.0604	50.13113	79.63833	173.9259	148.9084	197.5798	64.40548	116.1735	83.76483	52.81231	52.76492	48.23968	57.33841	69.01571
AC015967.	180.6506	321.1253	222.8068	308.67	177.1976	230.6612	260.0022	171.315	196.1043	268.0351	222.8797	174.8149	233.3752	201.9663	190.7111	171.486	195.0115	141.0707	192.7062
AC015967.	0	1.00039	0	0	3.322455	0	0	0	0	0.960699	3.614265	0	0	0	2.934017	0	0	0	1.792616
AC015969.	0	0	0	0	0	0	1.69936	0	0	0	0	1.840156	1.028084	0.93072	0.978006	4.946711	12.31652	0	0
AC015971.	136.4489	154.0601	240.7912	289.9627	120.7159	41.10793	466.4745	186.1314	11.67288	59.56335	13.2523	209.7778	156.2688	174.0447	217.1173	178.0816	130.3498	185.6672	305.641
AC015977.	0	0	0	2.078586	0	0	0	8.334243	7.003726	0	0	0.920078	5.140422	3.722881	0	0	1.026376	0.910133	0
AC015977.	1.921815	6.002343	13.98787	3.117878	13.28982	10.27698	6.797442	0.926027	9.338302	0	18.07132	10.12086	6.168506	6.515042	13.69208	8.244518	6.158258	13.652	18.82247
AC015982.	0	0	0	0	0	0	0.84968	0	0	0	0	0.920078	0	0	0.978006	0	0	0	0
AC015983.	0	1.00039	0	0	3.322455	1.141887	0	5.556162	3.501863	0	0	0.920078	0	0	0	0	0	0	0
AC015983.	72.06806	98.03826	149.87	56.12181	73.094	41.10793	112.1578	119.4575	51.36066	44.19217	27.70936	61.64524	66.82548	41.88241	66.5044	42.8715	44.13418	50.05734	60.05263
AC015987.	16.33543	22.00859	59.94802	19.74656	18.82724	12.56076	40.78465	46.30135	4.669151	40.34937	4.81902	33.12282	22.61786	19.54513	45.96627	21.43575	2.052753	23.66347	34.95601
AC016026.	63.4199	86.03358	86.92463	70.67191	128.4683	106.1955	100.2623	125.9397	105.0559	148.9084	159.0276	134.3314	115.1454	87.48771	150.6129	68.4295	84.16285	76.45121	132.6536
AC016027.	103.778	109.0426	135.8822	127.833	131.7907	77.64832	135.0992	118.5315	210.1118	146.987	207.2178	104.8889	111.0331	120.0629	144.7449	142.6302	110.8486	196.5888	166.7133
AC016027.	5.765445	4.001562	6.993936	2.078586	16.61227	15.98642	5.098081	1.852054	32.68406	10.56769	24.0951	10.12086	7.196591	14.89152	2.934017	5.771163	0	12.74187	8.963079
AC016027.	0	0	0	4.157171	0	0	3.398721	0	4.669151	8.646293	0	4.600391	1.028084	0	0.978006	0.824452	4.105505	8.191201	0
AC016027.	20.17906	2.000781	13.98787	3.117878	25.47215	11.41887	9.346483	5.556162	2.334575	1.921399	12.04755	24.84211	12.33701	13.9608	17.6041	15.66458	14.36927	8.191201	3.585232

AC016044.	127.8007	135.0527	87.92376	84.18271	95.2437	87.9253	162.2889	124.0876	79.37557	106.6376	96.38039	133.4113	97.66802	115.4093	78.24047	104.7054	144.7191	86.46267	144.3056
AC016065.	185.4552	189.0738	213.8146	169.4047	207.0997	286.6136	171.6354	179.6492	455.2422	363.1443	568.6443	217.1385	206.645	198.2434	222.9853	164.0659	231.961	265.759	392.5829
AC016065.	18.25724	6.002343	3.996535	2.078586	3.322455	12.56076	8.496802	10.1863	0	14.41049	0	3.680313	0	8.376483	10.75806	1.648904	10.26376	6.370934	0
AC016134.	0.960908	12.00469	6.993936	2.078586	18.82724	10.27698	6.797442	9.26027	9.338302	33.62447	26.50461	11.04094	1.028084	6.515042	4.890029	0.824452	0	8.191201	3.585232
AC016144.	3.84363	0	0	1.039293	0	0	0.84968	0	4.669151	0	0	0.920078	5.140422	0	0.978006	0	0	0	0
AC016152.	5.765445	4.001562	5.994802	2.078586	15.50479	2.283774	1.69936	2.778081	0	5.764196	8.433284	1.840156	3.084253	8.376483	6.846041	2.473355	0	2.7304	11.652
AC016168.	14.41361	3.001171	13.98787	6.235757	2.21497	11.41887	5.947762	0	0	0	0	0.920078	2.056169	9.307203	9.780058	9.893422	8.21101	9.101334	4.48154
AC016168.	3.84363	9.003514	12.98874	5.196464	5.537425	4.567548	5.947762	12.96438	18.6766	7.685594	6.023774	4.600391	4.112337	16.75297	3.912023	7.420066	9.237386	5.4608	11.652
AC016168.	0.960908	4.001562	0.999134	3.117878	0	0	0.84968	0	9.338302	3.842797	3.614265	0.920078	1.028084	0	0	0	2.052753	0.910133	0
AC016205.	9.609075	5.001952	6.993936	1.039293	6.64491	28.54718	12.7452	5.556162	1.167288	18.25329	0	2.760235	11.30893	14.89152	7.824047	12.36678	15.39564	14.56213	2.688924
AC016229.	0	0	0	0	0	2.283774	1.69936	0.926027	0	0	0	0.920078	0	0.93072	0.978006	0	0	0.910133	1.792616
AC016245.	11.53089	4.001562	0	0	6.64491	0	0.84968	0	10.50559	8.646293	26.50461	9.200782	1.028084	1.861441	0.978006	0.824452	0	7.281067	17.02985
AC016251.	49.00628	14.00547	75.93416	16.62868	56.48173	17.12831	71.37314	50.00546	5.836439	42.27077	54.21397	33.12282	52.4323	39.09025	37.16422	8.244518	4.105505	33.67494	43.02278
AC016251.	15.37452	9.003514	33.97054	6.235757	12.18233	29.68906	27.18977	33.33697	16.34203	46.11356	21.68559	29.4425	45.23571	12.09936	14.67009	8.244518	7.184634	15.47227	5.377848
AC016252.	12.4918	15.00586	4.995668	19.74656	1.107485	9.135096	13.59488	11.11232	22.17847	18.25329	13.2523	17.48149	10.28084	14.89152	4.890029	10.71787	8.21101	3.640534	7.170464
AC016257.	43.24084	37.01445	35.96881	28.0609	19.93473	35.3985	38.23561	37.96711	9.338302	28.82098	8.433284	36.80313	22.61786	16.75297	51.83431	31.32917	39.0023	39.13574	34.0597
AC016292.	0.960908	4.001562	5.994802	3.117878	0	1.141887	2.549041	5.556162	0	0	0	0	1.028084	3.722881	6.846041	2.473355	5.131881	6.370934	2.688924
AC016292.	5.765445	8.003124	3.996535	3.117878	11.07485	3.425661	5.947762	19.44657	2.334575	2.882098	0	2.760235	9.252759	0	1.956012	14.01568	0	10.9216	0
AC016355.	120.1134	97.03787	188.8363	84.18271	55.37425	86.78341	74.77186	97.23284	99.21946	151.7905	151.7991	80.96688	102.8084	89.34915	136.9208	97.28532	81.08372	142.8909	122.7942
AC016383.	0	4.001562	0	0	1.107485	0	0	0	0	5.764196	1.204755	0	2.056169	0.93072	0	0.824452	1.026376	0	3.585232
AC016394.	96.09075	147.0574	150.8692	130.9509	120.7159	95.91851	105.3603	128.7178	44.35693	119.1267	149.3896	110.4094	147.0161	102.3792	152.5689	139.3324	114.9541	82.82214	93.21603
AC016395.	7.68726	2.000781	15.98614	12.47151	0	14.84453	5.098081	5.556162	17.50932	0	1.204755	15.64133	10.28084	3.722881	6.846041	9.893422	15.39564	6.370934	5.377848
AC016396.	12.4918	3.001171	1.998267	5.196464	15.50479	2.283774	5.947762	5.556162	5.836439	2.882098	19.27608	9.200782	0	9.307203	16.6261	9.06897	12.31652	10.01147	6.274156
AC016396.	1.921815	14.00547	5.994802	0	2.21497	9.135096	14.44456	0	23.34575	6.724895	0	7.360626	1.028084	6.515042	11.73607	3.297807	14.36927	11.83173	0.896308
AC016397.	2.882723	4.001562	0	4.157171	12.18233	2.283774	0.84968	2.778081	3.501863	7.685594	8.433284	2.760235	10.28084	0.93072	5.868035	0.824452	4.105505	0	0.896308
AC016397.	158.5497	206.0804	155.8649	161.0904	174.9826	205.5397	178.4329	188.9095	143.5764	200.7861	180.7132	166.5342	124.3982	157.2917	173.107	115.4233	133.4289	124.6883	138.9277
AC016405.	4.804538	13.00508	27.97574	7.275049	19.93473	7.993209	8.496802	10.1863	23.34575	2.882098	14.45706	13.80117	9.252759	0	12.71408	0.824452	7.184634	5.4608	0.896308
AC016405.	9.609075	1.00039	10.99047	4.157171	0	10.27698	2.549041	0.926027	0	0.960699	0	4.600391	3.084253	0.93072	2.934017	0	5.131881	0	2.688924
AC016405.	9.609075	2.000781	0.999134	6.235757	1.107485	3.425661	0	5.556162	0	3.842797	12.04755	0	1.028084	0	0.978006	0	10.26376	2.7304	4.48154
AC016493.	3.84363	0	0	3.117878	0	0	3.398721	3.704108	0	0.960699	0	1.840156	0	0.93072	0	2.473355	5.131881	0	0
AC016526.	0	0	0	1.039293	0	0	0	0	0	0	0	0	2.056169	0	0.978006	0	0	0	0
AC016526.	6.726353	9.003514	13.98787	17.66798	33.22455	10.27698	14.44456	12.96438	33.85134	33.62447	44.57593	14.72125	6.168506	14.89152	19.56012	37.92478	29.76491	21.8432	13.44462
AC016526.	8.648168	10.0039	2.997401	66.51474	1.107485	2.283774	39.93497	36.11505	18.6766	5.764196	3.614265	27.60235	10.28084	17.68369	32.27419	32.15362	24.63303	19.1128	10.7557
AC016526.	3.84363	0	0	1.039293	0	0	0.84968	0	0	0	0	0	2.056169	0.93072	0	0	0	0	0.896308
AC016542.	11.53089	9.003514	1.998267	6.235757	25.47215	9.135096	12.7452	11.11232	9.338302	2.882098	21.68559	12.8811	12.33701	2.792161	24.45015	5.771163	5.131881	3.640534	3.585232
AC016542.	0	1.00039	7.993069	0	0	0	0.84968	3.704108	0	0	7.228529	0	1.028084	3.722881	0	0	0	0	3.585232
AC016542.	0	1.00039	0	0	0	0	0.84968	0	0	0	0	0.920078	0	0	0	0	0	0	0
AC016550.	0	0	7.993069	0	0	0	0.84968	0	0	0	0	0	0	0	0	0.824452	1.026376	0	0
AC016559.	2.882723	1.00039	2.997401	0	2.21497	0	0	0	0	5.764196	0	0	0	0	0	0	0	0	0
AC016559.	5.765445	0	1.998267	2.078586	5.537425	3.425661	5.098081	3.704108	0	3.842797	0	0	4.112337	0.93072	5.868035	0	0	0	0.896308
AC016573.	7.68726	9.003514	5.994802	18.70727	6.64491	4.567548	10.19616	4.630135	0	0	0	0.920078	1.028084	0	0.978006	0	0	5.4608	3.585232
AC016575.	0.960908	1.00039	1.998267	1.039293	8.859879	0	2.549041	0.926027	5.836439	3.842797	21.68559	6.440548	1.028084	0.93072	1.956012	3.297807	1.026376	2.7304	5.377848
AC016576.	0.960908	0	0	0	0	0	0	0	0	0	0	0.920078	1.028084	2.792161	0	3.297807	0	1.820267	0
AC016582.	25.9445	22.00859	47.95842	43.6503	23.25718	46.81737	33.98721	26.85478	42.02236	57.64196	22.89034	65.32555	59.62889	38.15953	86.06451	67.60505	40.02867	32.7648	42.12647
AC016582.	72.06806	46.01796	80.92983	51.96464	38.76197	59.37812	26.34009	75.93422	80.54285	45.15286	28.91412	76.36649	55.51656	43.74385	41.07624	75.84957	74.92547	41.86614	34.0597
AC016582.	440.0956	316.1234	414.6405	442.7387	261.3664	489.8695	355.1663	469.4957	366.5283	330.4805	275.8889	404.8344	429.7393	415.1013	394.1363	475.7087	451.6056	276.6806	285.0259
AC016583.	69.18534	65.02538	50.95582	67.55403	44.2994	84.49964	57.77826	76.86024	105.0559	78.77734	95.17564	81.88696	102.8084	31.64449	49.8783	71.72731	40.02867	10.01147	31.37078
AC016586.	49.96719	41.01601	48.95755	15.58939	119.6084	65.08756	36.53625	60.19176	21.01118	75.89524	59.03299	98.44837	75.05016	65.15042	46.94428	40.39814	46.18693	79.18161	83.35664
AC016588.	115.3089	18.00703	81.92896	84.18271	114.0709	22.83774	161.4392	157.4246	77.04099	68.20965	48.1902	156.4133	125.4263	144.2616	107.5806	90.6897	89.29473	130.1491	83.35664
AC016590.	228.696	155.0605	117.8978	114.3222	160.5853	142.7359	307.5842	131.4958	427.2273	253.6246	336.1266	134.3314	196.3641	317.3756	303.1818	142.6302	210.4071	167.4645	186.4321
AC016590.	23.06178	8.003124	106.9073	23.90373	23.25718	9.135096	48.43177	26.85478	8.171014	35.54587	13.2523	76.36649	48.31997	17.68369	34.2302	32.97807	16.42202	23.66347	8.963079
AC016590.	134.5271	138.0539	171.851	88.33989	117.3934	153.0129	58.62794	121.3095	187.9333	158.5154	138.5468	102.1287	140.8476	113.5479	129.0968	80.79628	129.3234	111.0363	95.90495
AC016596.	24.9836	26.01015	17.98441	19.74656	21.04221	23.97963	37.38593	50.93149	11.67288	20.17468	12.04755	27.60235	22.61786	36.29809	44.98827	24.73355	27.71216	30.94454	16.13354
AC016597.	52.84991	61.02382	147.8718	3.117878	117.3934	91.35096	113.8572	75.00819	99.21946	61.48475	61.4425	46.00391	58.60081	87.48771	81.17448	12.36678	29.76491	14.56213	24.20031
AC016597.	0	4.001562	0	0	2.21497	2.283774	0	0.926027	0	0.960699	0	0	0	0	0	0	0	0	0
AC016598.	0	1.00039	0	0	0	0	0	0	0	0	0	0	0	0	0.978006	0	0	0	0.896308
AC016598.	0	1.00039	0	0	0	0	2.549041	5.556162	0	0	0	0	0	0	0	0.824452	0	0	0
AC016629.	6.726353	32.01249	45.96015	18.70727	15.50479	33.11472	39.08529	24.0767	9.338302	30.74238	18.07132	17.48149	21.58977	25.12945	4.890029	24.73355	18.47477	32.7648	27.78555
AC016638.	0	0	0	0	0	0	0	0	0	5.764196	0	0	0	0	0	0	0	0	0
AC016642.	0	0	0	0	3.322455	0	3.398721	0	0	0	0	0	0	0	0	2.473355	3.079129	0	12.54831
AC016644.	6.726353	1.00039	1.998267	0	0	6.851322	7.647122	0	0	0	0	0	0	5.584322	0	4.122259	2.052753	1.820267	0.896308
AC016650.	0.960908	0	0	6.235757	0	0	0	0	0	0	0	0.920078	1.028084	3.722881	0	0.824452	0	0	0.896308

AC016683.	0	0	0	0	0	0	0	1.852054	0	3.842797	0	0.920078	0	0	0	0.824452	0	2.7304	1.792616
AC016687.	0	1.00039	0.999134	1.039293	0	1.141887	0	0	0	0	0	0	1.028084	0	0.978006	0.824452	1.026376	0	0
AC016687.	0	0	0	0	4.42994	0	0	0	0	0	0	0	0	0	0	0	0	0	1.792616
AC016687.	0	0	0	0	0	1.141887	0	0	0	0	0	0	1.028084	0	0	0	0	0	0
AC016691.	2.882723	2.000781	0.999134	1.039293	1.107485	1.141887	2.549041	0	0	0.960699	0	0	0	0	0	0	0	0	3.585232
AC016696.	0	0	0	1.039293	0	0	1.69936	0	0	0	0	0	0	0	0	0	0	0	0
AC016700.	0	0	0.999134	0	0	0	0	0	0	0	0	0	0	0	0	0	0	0.910133	0
AC016700.	7.68726	3.001171	0.999134	6.235757	4.42994	4.567548	1.69936	1.852054	11.67288	0	6.023774	0.920078	1.028084	2.792161	1.956012	6.595615	7.184634	5.4608	0.896308
AC016700.	8.648168	4.001562	11.9896	3.117878	19.93473	9.135096	1.69936	0	9.338302	13.44979	0	0.920078	3.084253	2.792161	12.71408	4.122259	2.052753	4.550667	1.792616
AC016705.	26.90541	22.00859	18.98354	11.43222	17.71976	27.40529	35.68657	27.78081	40.85507	25.93888	40.96167	17.48149	17.47743	6.515042	8.802052	8.244518	10.26376	20.02294	12.54831
AC016705.	1585.497	1757.686	1661.559	1306.391	1739.859	1597.5	2040.082	1722.41	528.7813	1156.682	1015.608	1624.858	1772.417	1264.849	1389.746	987.6933	937.0815	1215.028	1464.567
AC016708.	18.25724	15.00586	14.987	43.6503	11.07485	19.41208	17.84329	13.89041	0	18.25329	1.204755	11.96102	24.67402	16.75297	30.31818	27.20691	17.4484	70.99041	43.02278
AC016710.	1.921815	0	2.997401	2.078586	0	0	0	0	0	0	0	0	1.028084	5.584322	0	0	0	2.7304	1.792616
AC016712.	0	0	3.996535	0	0	4.567548	2.549041	5.556162	0	0.960699	9.638039	2.760235	1.028084	0	2.934017	3.297807	1.026376	3.640534	0
AC016716.	3.84363	1.00039	0	1.039293	0	0	3.398721	1.852054	0	1.921399	1.204755	0.920078	2.056169	4.653602	0	2.473355	1.026376	0	0
AC016716.	10.56998	12.00469	4.995668	4.157171	33.22455	13.70264	0	16.66849	37.35321	29.78168	30.11887	5.520469	5.140422	0.93072	2.934017	0.824452	1.026376	0.910133	1.792616
AC016717.	0.960908	3.001171	4.995668	1.039293	0	0	8.496802	1.852054	0	0.960699	0	0.920078	2.056169	4.653602	4.890029	14.01568	0	1.820267	11.652
AC016717.	167.1979	87.03397	108.9056	342.9666	89.70628	105.0536	202.2239	112.9753	9.338302	88.38433	36.14265	145.3724	111.0331	69.80402	96.82258	188.7995	198.0906	142.8909	152.3724
AC016722.	5.765445	3.001171	3.996535	7.275049	8.859879	5.709435	6.797442	0	0	9.606993	28.91412	9.200782	5.140422	10.23792	7.824047	12.36678	6.158258	10.9216	11.652
AC016722.	60.53717	79.03084	170.8519	160.0511	165.0153	69.65511	152.9424	117.6054	31.51677	111.4411	69.87578	183.0956	142.9037	131.2316	129.0968	131.0878	111.875	172.0152	213.3213
AC016722.	204.6733	181.0707	324.7184	195.387	191.5949	226.0936	194.5768	208.3561	400.3797	166.201	271.0698	241.0605	238.5156	233.6108	209.2932	216.0064	196.0379	161.0936	153.2687
AC016723.	0	11.00429	0.999134	6.235757	13.28982	2.283774	6.797442	4.630135	0	10.56769	0	9.200782	1.028084	13.03008	0.978006	4.122259	0	5.4608	6.274156
AC016727.	369.9494	415.162	471.5911	281.6483	302.3434	464.748	302.4862	490.7943	277.8145	510.1313	387.9311	321.1073	246.7402	419.7549	474.3328	248.16	312.0184	331.2886	227.6622
AC016734.	2.882723	10.0039	0.999134	3.117878	1.107485	15.98642	3.398721	1.852054	17.50932	1.921399	7.228529	12.8811	1.028084	16.75297	1.956012	15.66458	13.34289	21.8432	9.859387
AC016734.	0.960908	0	0	0	0	1.141887	0	0.926027	0	0	0	0	1.028084	0	0.978006	0.824452	0	0	0.896308
AC016735.	7.68726	2.000781	20.98181	27.02161	23.25718	7.993209	16.14392	9.26027	46.69151	36.50657	14.45706	34.96297	20.56169	9.307203	21.51613	14.01568	14.36927	9.101334	4.48154
AC016735.	5.765445	5.001952	15.98614	19.74656	3.322455	7.993209	30.58849	5.556162	8.171014	8.646293	1.204755	3.680313	11.30893	0.93072	1.956012	0.824452	1.026376	9.101334	5.377848
AC016737.	89.3644	56.02186	104.909	38.45383	70.87903	90.20907	49.28145	108.3452	114.3942	118.166	114.4517	89.24759	51.40422	88.41843	86.06451	61.83389	59.52982	109.216	51.98586
AC016737.	0	8.003124	11.9896	0	11.07485	3.425661	5.098081	23.15068	19.84389	7.685594	7.228529	8.280704	16.44935	7.445762	6.846041	2.473355	1.026376	3.640534	2.688924
AC016738.	118.1916	77.03006	143.8752	109.1257	87.49131	75.36454	115.5565	92.6027	232.2903	117.2053	174.6895	70.84602	72.99399	111.6864	109.5367	130.2634	156.0092	265.759	241.1068
AC016738.	0.960908	2.000781	3.996535	8.314342	1.107485	6.851322	0.84968	0	2.334575	1.921399	0	1.840156	0	1.861441	1.956012	4.946711	1.026376	0.910133	0.896308
AC016739.	54.77173	41.01601	30.97314	50.92535	52.05179	42.24982	33.98721	28.70684	70.03726	34.58517	40.96167	37.72321	35.98295	51.18962	46.94428	41.22259	64.6617	64.61947	69.01571
AC016745.	2.882723	3.001171	9.991337	28.0609	12.18233	1.141887	5.098081	16.66849	4.669151	22.09608	12.04755	6.440548	12.33701	20.47585	5.868035	11.54233	8.21101	24.5736	5.377848
AC016745.	1.921815	4.001562	0	7.275049	4.42994	9.135096	4.248401	6.482189	0	0.960699	0	0.920078	1.028084	0.93072	6.846041	0	1.026376	0	0
AC016746.	6.726353	1.00039	1.998267	2.078586	2.21497	5.709435	6.797442	9.26027	0	0	0	2.760235	2.056169	2.792161	0.978006	0	7.184634	6.370934	7.170464
AC016747.	2867.348	2291.894	2074.201	3247.79	1680.055	2274.639	2007.794	1985.402	1596.85	1723.494	1413.177	1692.024	1752.884	2336.108	2427.41	2662.155	2417.116	2268.963	1873.284
AC016747.	98.97348	102.0398	120.8952	140.3045	80.8464	92.49285	130.0011	90.75065	92.21573	81.65944	61.4425	132.4913	102.8084	43.74385	43.03226	185.5017	143.6927	161.0936	87.83818
AC016747.	51.88901	66.02577	50.95582	97.69352	91.92125	85.64153	108.7591	101.863	80.54285	72.05244	65.05676	111.3295	70.93782	81.90339	91.93255	90.6897	53.37157	91.01334	77.08248
AC016747.	61.49808	37.01445	34.96968	82.10413	60.91167	76.50643	90.91579	48.15341	10.50559	64.36685	34.93789	65.32555	53.46039	37.22881	43.03226	50.29156	73.89909	51.8776	80.66772
AC016757.	244.0705	189.0738	277.7592	107.0472	339.9979	134.7427	149.5437	301.8848	444.7366	434.2361	643.3391	83.72712	98.6961	164.7375	153.5469	85.74299	79.03097	112.8565	172.9874
AC016766.	2.882723	3.001171	0	0	0	0	0	5.098081	5.556162	0	0	4.81902	0	1.028084	2.792161	0	0	0	2.688924
AC016769.	1.921815	14.00547	14.987	1.039293	13.28982	15.98642	12.7452	16.66849	11.67288	19.21399	1.204755	35.88305	5.140422	4.653602	18.58211	13.19123	17.4484	10.01147	21.51139
AC016769.	0	0	0	0	0	0	1.69936	0	0	0	0	0	0	0	0.978006	0	0	0	0
AC016769.	34.59267	60.02343	37.96708	8.314342	42.08443	47.95925	35.68657	38.89314	42.02236	24.01748	20.48083	22.08188	31.87062	32.57521	27.38416	23.08465	19.50115	13.652	16.13354
AC016772.	0	5.001952	0	4.157171	19.93473	2.283774	12.7452	5.556162	1.167288	0.960699	0	0	1.028084	7.445762	1.956012	1.648904	3.079129	0.910133	6.274156
AC016773.	26.90541	64.02499	51.95495	21.82515	90.81376	74.22266	20.39233	47.22738	158.7511	174.8473	408.4119	76.36649	52.4323	69.80402	51.83431	52.76492	59.52982	86.46267	95.90495
AC016813.	0	4.001562	0.999134	0	4.42994	0	0.84968	0	0	0	4.81902	3.680313	0	0	11.73607	0	0	3.640534	0.896308
AC016820.	6.726353	2.000781	10.99047	4.157171	5.537425	7.993209	18.69297	8.334243	12.84017	5.764196	21.68559	21.1618	27.75828	9.307203	6.846041	18.13794	17.4484	5.4608	17.02985
AC016822.	0.960908	6.002343	1.998267	0	0	0	4.248401	0.926027	0	2.882098	0	0.920078	0	2.792161	0	0	3.079129	0	0
AC016825.	0	2.000781	0	0	1.107485	0	3.398721	5.556162	0	1.921399	0	1.840156	0	0	0	0.824452	0	0	0.896308
AC016831.	11496.3	20341.94	15246.78	19205.09	15412.87	18948.47	12164.87	15358.16	15749.05	18120.71	16209.98	13102.83	12884.98	14880.36	14915.57	15737.14	17903.08	9897.701	14021.84
AC016831.	0	3.001171	1.998267	9.353635	9.967364	0	3.398721	12.03835	3.501863	3.842797	0	3.680313	1.028084	3.722881	1.956012	0	2.052753	0	5.377848
AC016831.	6.726353	5.001952	17.98441	3.117878	7.752394	3.425661	9.346483	13.89041	14.00745	14.41049	9.638039	7.360626	10.28084	11.16864	8.802052	6.595615	17.4484	6.370934	6.274156
AC016831.	1.921815	3.001171	7.993069	0	5.537425	4.567548	0	8.334243	5.836439	3.842797	2.40951	4.600391	2.056169	0	6.846041	0	0	0	0
AC016831.	7.68726	21.0082	13.98787	2.078586	6.64491	9.135096	5.098081	1.852054	10.50559	14.41049	14.45706	22.08188	29.81445	16.75297	10.75806	10.71787	13.34289	16.3824	15.23724
AC016831.	20.17906	20.00781	21.98094	9.353635	4.42994	9.135096	16.14392	11.11232	11.67288	3.842797	10.84279	3.680313	6.168506	15.82225	12.71408	13.19123	5.131881	10.01147	3.585232
AC016866.	44.20175	37.01445	42.96275	40.53242	35.43952	45.67548	49.28145	28.70684	33.85134	56.68126	32.52838	60.72516	59.62889	53.05106	55.74633	33.80253	28.73854	43.6864	70.80833
AC016866.	0	0	0	1.039293	0	0	2.549041	0	0	0	0	0	0	0	0	0	0	0	0
AC016876.	252.7187	185.0722	247.7851	87.30059	207.0997	149.5872	100.2623	177.7972	497.2646	301.6596	696.3483	167.4542	174.7743	188.9362	177.9971	110.4765	153.9564	188.3976	209.7361
AC016876.	26.90541	57.02225	57.94975	27.02161	37.65449	55.95246	29.73881	64.82189	117.8961	62.44545	149.3896	28.52243	43.17954	30.71377	48.90029	23.9091	42.08143	13.652	8.963079
AC016876.	2.882723	4.001562	9.991337	2.078586	3.322455	18.27019	0	12.96438	26.84762	0.960699	36.14265	20.24172	6.168506	2.792161	3.912023	9.893422	16.42202	10.9216	14.34093
AC016877.	8.648168	4.001562	19.98267	8.314342	50.94431	9.135096	23.79105	22.22465	60.69896	12.48909	24.0951	18.40156	9.252759	18.61441	45.96627	4.122259	5.131881	17.29253	7.170464

AC016877.	0	0	0	0	0	0	0	0	0	0	0	0	2.056169	0.93072	7.824047	0	1.026376	0	0
AC016877.	34.59267	29.01132	33.97054	25.98232	37.65449	37.68227	41.63433	20.37259	51.36066	24.97818	7.228529	30.36258	35.98295	63.28898	61.61437	20.6113	17.4484	17.29253	34.0597
AC016885.	0.960908	0	0.999134	4.157171	0	0	0.84968	6.482189	0	5.764196	0	7.360626	6.168506	6.515042	6.846041	0.824452	2.052753	0	5.377848
AC016885.	6.726353	0	0	0	4.42994	0	4.248401	11.11232	0	0	0	0.920078	0	0	1.956012	0	0	0	0
AC016885.	0	1.00039	0.999134	0	0	0	0	3.704108	14.00745	0	4.81902	2.760235	0	0	3.912023	0	0	0	0
AC016888.	1.921815	2.000781	1.998267	4.157171	0	2.283774	0	0	7.003726	0	4.81902	7.360626	1.028084	0.93072	5.868035	0.824452	13.34289	0.910133	0
AC016894.	3.84363	0	9.991337	0	13.28982	1.141887	2.549041	14.81643	5.836439	1.921399	6.023774	0	5.140422	2.792161	3.912023	0.824452	0	0.910133	0.896308
AC016903.	0	0	2.997401	0	0	0	0	0	0	0	0	0	0	0	0	0	0	0	0
AC016907.	30.74904	46.01796	57.94975	56.12181	42.08443	38.82416	53.52986	30.55889	63.03354	21.13538	12.04755	61.64524	24.67402	49.32818	47.92229	24.73355	35.92317	26.39387	59.15632
AC016909.	0	1.00039	0	0	0	0	0	0	0	0	0	0	0	0.93072	0	0	1.026376	0	0
AC016909.	0	0	1.998267	0	1.107485	0	3.398721	1.852054	0	0	0	0.920078	7.196591	0.93072	15.64809	0.824452	0	0	5.377848
AC016910.	13.45271	13.00508	20.98181	12.47151	24.36467	3.425661	25.49041	15.74246	7.003726	14.41049	3.614265	28.52243	24.67402	8.376483	17.6041	19.78684	13.34289	11.83173	20.61508
AC016924.	209.4778	306.1195	442.6162	266.0589	358.8251	373.3971	322.0288	248.1752	645.5101	337.2054	319.26	231.8597	230.2909	276.4239	256.2375	169.0126	205.2753	243.9158	261.7219
AC016930.	1.921815	0	1.998267	0	1.107485	0	5.098081	0	0	0	0	0	5.140422	4.653602	0.978006	0	0	0	0
AC016933.	0.960908	0	0	0	0	0	0	0	0	0	0	0	0	0.93072	0	0.824452	0	0	0
AC016949.	9.609075	14.00547	89.92203	36.37525	74.20149	30.83095	57.77826	29.63287	67.70269	52.83846	45.78069	47.84407	38.03912	39.09025	49.8783	23.08465	11.29014	75.54107	51.98586
AC016954.	0.960908	5.001952	7.993069	8.314342	1.107485	3.425661	9.346483	12.96438	1.167288	6.724895	7.228529	3.680313	5.140422	15.82225	5.868035	9.06897	11.29014	7.281067	11.652
AC016956.	0.960908	1.00039	0	0	0	0	3.398721	0.926027	0	0	0	5.520469	0	0	5.868035	0	0	0	0
AC016957.	2.882723	1.00039	4.995668	2.078586	3.322455	9.135096	1.69936	0.926027	5.836439	8.646293	0	3.680313	0	3.722881	0.978006	0	2.052753	1.820267	4.48154
AC016957.	75.91169	64.02499	60.94715	78.98625	75.30897	101.6279	60.3273	19.44657	141.2418	77.81664	84.33284	104.8889	68.88165	62.35826	40.09824	53.58937	66.71446	52.78774	51.98586
AC016987.	0	0	0	2.078586	0	0	0	0	0	0	0	0	0	0	0	0	0	0	0
AC016999.	3.84363	4.001562	0	0	2.21497	2.283774	0.84968	3.704108	8.171014	7.685594	4.81902	14.72125	1.028084	4.653602	0	4.946711	1.026376	9.101334	0.896308
AC016999.	7.68726	1.00039	3.996535	4.157171	6.64491	0	5.098081	18.52054	5.836439	0.960699	0	0.920078	2.056169	3.722881	0.978006	1.648904	2.052753	1.820267	0.896308
AC017000.	0	1.00039	0	2.078586	0	4.567548	0	0	0	3.842797	0	0.920078	1.028084	0.93072	0.978006	0	3.079129	0.910133	0
AC017002.	11.53089	26.01015	5.994802	8.314342	4.42994	1.141887	22.94137	10.1863	7.003726	4.803496	19.27608	26.68227	18.50552	17.68369	10.75806	28.03136	27.71216	19.1128	7.170464
AC017002.	9.609075	1.00039	5.994802	10.39293	12.18233	9.135096	1.69936	4.630135	9.338302	8.646293	9.638039	1.840156	5.140422	9.307203	0.978006	4.946711	0	0.910133	2.688924
AC017002.	0	0	2.997401	0	0	0	3.398721	0	0	0.960699	0	2.760235	6.168506	0.93072	0.978006	9.06897	4.105505	2.7304	6.274156
AC017002.	0	0	0.999134	1.039293	4.42994	0	0	0	0	7.685594	0	0.920078	1.028084	0	0	0	1.026376	5.4608	0.896308
AC017002.	0	0	0	3.117878	5.537425	0	3.398721	2.778081	0	0	0	0.920078	1.028084	0	0	0.824452	0	0.910133	0
AC017006.	0.960908	2.000781	3.996535	8.314342	0	4.567548	16.14392	3.704108	0	0	0	6.440548	10.28084	0.93072	0.978006	7.420066	5.131881	3.640534	0.896308
AC017006.	0	0	4.995668	0	0	2.283774	6.797442	1.852054	0	0	6.023774	0.920078	0	0.93072	1.956012	0.824452	0	0	3.585232
AC017007.	1.921815	1.00039	0.999134	8.314342	3.322455	0	1.69936	0	4.669151	2.882098	0	0.920078	1.028084	1.861441	0	0	0	0	0
AC017007.	8.648168	19.00742	4.995668	35.33595	0	13.70264	8.496802	18.52054	4.669151	58.60265	44.57593	14.72125	24.67402	10.23792	17.6041	22.2602	25.65941	3.640534	14.34093
AC017007.	3.84363	0	0	1.039293	2.21497	0	3.398721	3.704108	5.836439	0.960699	0	0	0	0	0	0.824452	2.052753	3.640534	0
AC017007.	0	1.00039	0	8.314342	6.64491	3.425661	0.84968	4.630135	0	5.764196	7.228529	0	3.084253	0	0.978006	1.648904	0	0	0.896308
AC017007.	28.82723	11.00429	31.97228	13.51081	0	11.41887	21.24201	21.29862	28.01491	6.724895	30.11887	7.360626	23.64594	13.03008	15.64809	17.31349	12.31652	9.101334	8.066772
AC017013.	0	0	0	2.078586	0	0	0	0.926027	0	0	0	0	1.028084	0	0	0.824452	0	0	0
AC017015.	0	2.000781	0	0	4.42994	0	0	1.852054	0	0	0	0	1.028084	1.861441	0	0	0	0	0
AC017015.	12.4918	10.0039	38.96621	5.196464	1.107485	6.851322	11.04584	9.26027	10.50559	8.646293	2.40951	18.40156	9.252759	8.376483	23.47214	3.297807	8.21101	20.02294	24.20031
AC017033.	10.56998	15.00586	11.9896	0	29.90209	5.709435	11.04584	9.26027	7.003726	11.52839	28.91412	19.32164	17.47743	17.68369	5.868035	6.595615	12.31652	27.304	9.859387
AC017035.	8.648168	0	0.999134	4.157171	15.50479	0	3.398721	5.556162	1.167288	4.803496	4.81902	2.760235	11.30893	10.23792	7.824047	0	0	0.910133	0.896308
AC017037.	0	0	0.999134	0	5.537425	0	3.398721	0	0	0	0	0.920078	1.028084	0	0	3.297807	0	0	0
AC017037.	1.921815	2.000781	11.9896	1.039293	11.07485	6.851322	9.346483	0	5.836439	0.960699	9.638039	2.760235	5.140422	0	0	1.648904	4.105505	0	0.896308
AC017037.	0	0	0	1.039293	0	0	0	0	0	0	0	0	1.028084	0	0.978006	0	0	0	0
AC017037.	9.609075	15.00586	3.996535	14.5501	4.42994	6.851322	12.7452	19.44657	1.167288	3.842797	7.228529	18.40156	20.56169	11.16864	19.56012	0	14.36927	7.281067	8.066772
AC017048.	0.960908	1.00039	0.999134	0	0	0	0	0	8.171014	0	0	0.920078	0	3.722881	0	0	0	0	0
AC017048.	1.921815	11.00429	24.97834	1.039293	1.107485	7.993209	1.69936	0.926027	2.334575	8.646293	3.614265	8.280704	5.140422	5.584322	1.956012	1.648904	2.052753	0.910133	6.274156
AC017048.	41.31902	31.0121	60.94715	7.275049	77.52394	47.95925	16.9936	12.03835	127.2344	80.69874	73.49005	46.00391	50.37613	55.84322	34.2302	9.06897	31.81766	50.05734	43.02278
AC017053.	0	0	0	0	0	1.141887	0	0	0	0	0	0	0	0	0.978006	0	0	0	0
AC017067.	0	1.00039	10.99047	1.039293	13.28982	0	6.797442	0	11.67288	3.842797	0	7.360626	12.33701	2.792161	0	0	0	21.8432	12.54831
AC017071.	44.20175	52.0203	34.96968	77.94696	62.01916	54.81058	67.12474	89.82462	57.1971	102.7948	78.30907	56.12477	54.48847	57.70466	107.5806	31.32917	18.47477	29.12427	31.37078
AC017074.	30.74904	26.01015	24.97834	16.62868	35.43952	31.97284	11.04584	12.96438	24.51304	21.13538	43.37118	23.92203	27.75828	44.67457	36.18622	60.18498	42.08143	48.23707	36.74863
AC017074.	0	0	0	0	0	2.283774	0	3.704108	0	0	7.228529	0.920078	6.168506	0.93072	3.912023	1.648904	4.105505	1.820267	1.792616
AC017076.	32.67086	69.02694	124.8917	110.165	58.6967	179.2763	25.49041	97.23284	142.4091	162.3582	102.4042	11.04094	19.5336	40.02097	32.27419	50.29156	71.84634	30.94454	41.23017
AC017078.	0	0	3.996535	1.039293	0	2.283774	3.398721	0	0	3.842797	0	0	0	0	0.978006	1.648904	2.052753	0	0
AC017079.	1.921815	1.00039	3.996535	3.996535	0	1.107485	8.496802	0	0	0	8.433284	3.680313	8.224675	0	0	3.297807	0	0	8.963079
AC017079.	0	2.000781	23.97921	5.196464	7.752394	10.27698	10.19616	7.408216	4.669151	4.803496	2.40951	9.200782	16.44935	0	6.846041	9.06897	5.131881	22.75334	12.54831
AC017083.	6.726353	3.001171	0.999134	2.078586	5.537425	3.425661	4.248401	8.334243	24.51304	3.842797	8.433284	1.840156	9.252759	9.307203	1.956012	6.595615	6.158258	3.640534	6.274156
AC017083.	98.97348	106.0414	134.883	50.92535	128.4683	91.35096	28.88913	179.6492	289.4874	226.725	222.8797	102.1287	74.02207	95.86419	115.4047	42.8715	41.05505	81.00187	61.84525
AC017083.	6.726353	1.00039	6.993936	9.353635	11.07485	7.993209	5.947762	7.408216	1.167288	0	6.023774	17.48149	6.168506	2.792161	5.868035	1.648904	3.079129	0	3.585232
AC017099.	0	1.00039	0	0	0	0	6.797442	0	0	0.960699	0	0	0	0	0	0	0	0	0
AC017100.	149.9016	206.0804	87.92376	184.9941	74.20149	200.9721	175.8838	183.3534	166.9221	188.2971	95.17564	195.0566	162.4373	190.7977	158.4369	183.0283	175.5103	151.0821	193.6025

AC017101.	0.960908	4.001562	7.993069	6.235757	4.42994	1.141887	16.14392	0.926027	0	2.882098	0	8.280704	7.196591	9.307203	4.890029	2.473355	2.052753	1.820267	6.274156
AC017104.	49.96719	52.0203	60.94715	61.31827	70.87903	85.64153	28.03945	44.4493	186.766	87.42363	139.7516	46.00391	61.68506	55.84322	21.51613	50.29156	68.76721	31.85467	70.80833
AC017104.	0.960908	4.001562	7.993069	10.39293	4.42994	0	1.69936	0	0	0.960699	0	0.920078	0	0.93072	0	0.824452	1.026376	0	1.792616
AC017104.	181.6115	361.1409	190.8345	129.9116	158.3703	106.1955	152.9424	200.9479	173.9259	383.319	286.7317	201.4971	202.5326	141.4695	146.7009	136.0346	97.50574	161.0936	93.21603
AC017104.	122.9962	150.0586	134.883	120.558	60.91167	97.0604	101.9616	163.9068	21.01118	114.3232	65.05676	184.9357	163.4654	93.07203	68.46041	108.0032	118.0333	115.5869	128.172
AC018358.	0.960908	0	0	0	8.859879	0	0	11.11232	0	0	0	4.600391	0	0	0	6.595615	0	0	0
AC018371.	0	2.000781	0	0	1.107485	0	0	0	0	0	0	0	0	0	0	0	1.026376	0	3.585232
AC018410.	21.13997	28.01093	43.96188	68.59332	27.68712	27.40529	37.38593	21.29862	19.84389	17.29259	12.04755	41.40352	37.01104	33.50593	44.01026	31.32917	14.36927	22.75334	21.51139
AC018410.	32.67086	33.01288	112.9021	87.30059	43.19191	41.10793	74.77186	42.59724	33.85134	38.42797	10.84279	92.9279	84.30292	44.67457	37.16422	55.23827	68.76721	122.868	42.12647
AC018413.	139.3316	154.0601	188.8363	164.2083	157.2629	105.0536	141.0469	155.5725	148.2455	120.0874	185.5323	150.8928	140.8476	164.7375	168.217	126.1411	153.9564	137.4301	143.4093
AC018437.	0	0	3.996535	1.039293	4.42994	0	0.84968	0	0	0	0	1.840156	0	0.93072	0	0	0	0	0
AC018437.	1.921815	0	0	4.157171	0	4.567548	0.84968	0	0	0	0	0	0	0	4.890029	8.244518	1.026376	0	1.792616
AC018442.	0.960908	3.001171	1.998267	0	9.967364	4.567548	0	1.852054	4.669151	15.37119	37.3474	8.280704	1.028084	5.584322	5.868035	1.648904	1.026376	0	5.377848
AC018442.	0.960908	0	3.996535	4.157171	6.64491	0	0	0	11.67288	5.764196	0	0.920078	5.140422	0	4.890029	0	1.026376	2.7304	1.792616
AC018445.	14.41361	1.00039	5.994802	0	9.967364	1.141887	1.69936	11.11232	1.167288	0	1.204755	2.760235	11.30893	0	0	3.297807	8.21101	0	3.585232
AC018445.	5.765445	1.00039	4.995668	2.078586	3.322455	4.567548	6.797442	0	1.167288	0	0	5.520469	5.140422	5.584322	0	2.473355	11.29014	3.640534	1.792616
AC018445.	2.882723	8.003124	1.998267	1.039293	12.18233	9.135096	2.549041	0	1.167288	10.56769	0	1.840156	3.084253	11.16864	1.956012	11.54233	1.026376	10.9216	5.377848
AC018445.	0	7.002733	5.994802	5.196464	11.07485	0	5.098081	0	0	1.921399	2.40951	17.48149	11.30893	9.307203	2.934017	23.9091	5.131881	9.101334	11.652
AC018448.	0	0	0	0	0	1.141887	0	0	0	0	0	0	0	0	0	0	0	0.910133	0
AC018448.	0	0	0	3.117878	0	0	0.84968	0	0	0	0	0	0	0	0	0	0	0	0
AC018450.	120.1134	58.02265	53.95322	43.6503	67.55658	134.7427	46.73241	87.04654	150.5801	63.40615	61.4425	52.44446	51.40422	84.69555	62.59237	43.69595	69.79359	56.42827	34.95601
AC018450.	0	0	0	0	0	1.141887	0	0	0	0	0	0	0	0	0	0.824452	0	0	0
AC018462.	36.51449	36.01406	52.95408	76.90766	79.73891	31.97284	56.0789	55.56162	21.01118	10.56769	42.16642	51.52438	41.12337	31.64449	57.70234	32.97807	22.58028	28.21414	42.12647
AC018462.	0.960908	2.000781	1.998267	8.314342	1.107485	0	2.549041	0.926027	0	0	0	8.280704	5.140422	0	2.934017	7.420066	1.026376	1.820267	3.585232
AC018463.	0	6.002343	0	13.51081	0	2.283774	2.549041	10.1863	0	5.764196	0	0	0	2.792161	7.824047	0.824452	5.131881	3.640534	2.688924
AC018467.	9.609075	3.001171	12.98874	23.90373	22.1497	4.567548	12.7452	3.704108	5.836439	8.646293	10.84279	12.8811	8.224675	8.376483	3.912023	22.2602	24.63303	0.910133	4.48154
AC018470.	0	0	0.999134	0	1.107485	0	0	5.556162	0	0	0	3.680313	0	0	0	0	0	0	0
AC018475.	43.24084	9.003514	13.98787	15.58939	15.50479	14.84453	16.9936	28.70684	7.003726	12.48909	18.07132	7.360626	11.30893	15.82225	11.73607	32.15362	13.34289	20.93307	9.859387
AC018511.	3.84363	5.001952	5.994802	1.039293	1.107485	4.567548	5.947762	8.334243	0	6.724895	1.204755	6.440548	1.028084	4.653602	5.868035	2.473355	1.026376	0.910133	6.274156
AC018511.	0	0	1.998267	0	0	0	0	0	0	0	0	5.520469	0	0.93072	0	0	0	0	0
AC018521.	0.960908	1.00039	0.999134	0	0	0	6.797442	0	16.34203	1.921399	2.40951	0.920078	9.252759	0.93072	3.912023	2.473355	0	1.820267	2.688924
AC018521.	0	0	0	0	0	0	0	0	0	0	1.204755	0	1.028084	0	0	0	0	0	2.688924
AC018521.	2024.632	1661.649	1386.798	2147.179	1738.751	1739.094	1958.513	1522.388	925.6592	1057.73	1257.764	1651.54	1737.463	1610.146	1571.655	2331.55	2533.097	2048.71	1537.168
AC018521.	91.28622	89.03475	124.8917	62.35757	109.641	113.0468	126.6024	71.30408	105.0559	106.6376	93.97088	155.4932	125.4263	97.72563	106.6026	63.48279	46.18693	112.8565	112.9348
AC018521.	24.9836	22.00859	42.96275	20.78586	18.82724	33.11472	10.19616	30.55889	11.67288	24.01748	25.29985	21.1618	31.87062	16.75297	24.45015	26.38246	2.052753	55.51814	12.54831
AC018523.	8.648168	3.001171	16.98527	6.235757	16.61227	12.56076	5.098081	8.334243	0	12.48909	1.204755	15.64133	16.44935	6.515042	9.780058	5.771163	13.34289	27.304	10.7557
AC018529.	3.84363	1.00039	0	0	0	0	0	0	10.50559	0	2.40951	0	0	0	3.912023	0	2.052753	0	0.896308
AC018529.	179.6897	30.01171	74.93502	35.33595	75.30897	50.24303	24.64073	75.00819	47.8588	69.17035	112.0422	119.6102	95.61185	70.73474	97.80058	65.95615	51.31881	75.54107	44.8154
AC018529.	13.45271	11.00429	0.999134	1.039293	0	14.84453	4.248401	24.0767	0	7.685594	1.204755	14.72125	10.28084	4.653602	10.75806	7.420066	2.052753	11.83173	1.792616
AC018552.	1.921815	0	3.996535	0	0	0	9.346483	2.778081	0	3.842797	0	0.920078	0	0	6.846041	2.473355	2.052753	6.370934	7.170464
AC018552.	8.648168	5.001952	0	5.196464	6.64491	11.41887	0	12.03835	0	9.606993	0	2.760235	4.112337	12.09936	8.802052	12.36678	8.21101	1.820267	0.896308
AC018552.	3.84363	3.001171	7.993069	15.58939	17.71976	12.56076	8.496802	4.630135	1.167288	10.56769	2.40951	11.04094	17.47743	5.584322	3.912023	23.08465	23.60665	27.304	15.23724
AC018553.	0	5.001952	1.998267	0	1.107485	12.56076	6.797442	4.630135	5.836439	3.842797	0	8.280704	10.28084	0	0.978006	0	0	6.370934	1.792616
AC018553.	0	0	0	0	7.752394	0	0	0.926027	7.003726	0	0	1.840156	0	0	0	0	6.158258	0	3.585232
AC018554.	0	0	0	1.039293	2.21497	0	1.69936	0	0	1.921399	0	0	0	5.584322	0	0.824452	0	0	0
AC018554.	0	0	0	0	0	0	0	0	0	0	0	0.920078	0	0	0	0	0	0.910133	0
AC018557.	7.68726	9.003514	21.98094	5.196464	24.36467	2.283774	11.89552	3.704108	11.67288	9.606993	3.614265	12.8811	20.56169	7.445762	2.934017	7.420066	3.079129	0	4.48154
AC018607.	0	0	0	8.314342	0	0	1.69936	0	2.334575	0	0	0.920078	0	0	0	0	0	0	0
AC018616.	0	0	0	0	0	1.141887	0	1.852054	0	0	0	4.600391	1.028084	0	2.934017	0	0	0	0
AC018618.	0	0	0.999134	12.47151	4.42994	2.283774	1.69936	1.852054	0	4.803496	3.614265	17.48149	15.42127	52.12034	62.59237	37.10033	33.87042	10.9216	0.896308
AC018618.	0	0	0	0	0	0	0	0	0	0	0	0	0	0	0	3.297807	0	0	0
AC018620.	0.960908	0	10.99047	3.117878	0	0	4.248401	0	0	2.882098	4.81902	15.64133	6.168506	0	0	6.595615	1.026376	0.910133	0
AC018628.	340.1613	279.1089	266.7687	298.277	270.2263	238.6544	454.5789	351.8903	200.7735	226.725	172.2799	275.1034	426.655	298.7612	307.0938	231.671	250.4358	330.3784	246.4847
AC018629.	5.765445	0	0	13.51081	1.107485	0	0.84968	0	0	0	0	1.840156	0	0	0	5.771163	0	7.281067	3.585232
AC018630.	24.02269	20.00781	18.98354	35.33595	2.21497	28.54718	30.58849	15.74246	11.67288	10.56769	36.14265	34.96297	43.17954	32.57521	40.09824	27.20691	20.52753	24.5736	12.54831
AC018630.	118.1916	41.01601	15.98614	42.611	52.05179	59.37812	12.7452	3.704108	19.84389	48.99566	30.11887	83.72712	103.8365	53.98178	64.54838	56.88718	42.08143	27.304	17.92616
AC018630.	0	0	0	2.078586	0	0	0	0	0	2.882098	0	2.760235	0	3.722881	1.956012	0	0	0	0
AC018634.	0	1.00039	0.999134	0	0	0	0.84968	0	0	0	0	0	1.028084	0	0.978006	0	0	5.4608	2.688924
AC018635.	0	1.00039	2.997401	0	9.967364	4.567548	0.84968	7.408216	12.84017	2.882098	12.04755	0	3.084253	0.93072	0	1.648904	2.052753	1.820267	8.066772
AC018638.	200.8297	118.0461	114.9004	178.7584	107.426	125.6076	173.3348	195.3917	198.4389	121.0481	199.9893	297.1853	241.5998	188.0055	159.4149	208.5863	222.7236	206.6003	64.53417
AC018638.	185.4552	153.0597	134.883	89.37918	228.1419	247.7895	113.8572	158.3506	581.3093	335.284	1113.194	395.6336	327.9589	125.6472	205.3812	145.928	301.7546	366.7838	339.7007
AC018638.	49.96719	29.01132	21.98094	18.70727	28.79461	39.96605	44.18337	24.0767	1.167288	41.31007	36.14265	36.80313	46.2638	37.22881	34.2302	34.62698	40.02867	36.40534	25.09662

AC018638.	1755.578	1406.549	1707.519	1421.753	1379.926	1640.892	1763.086	1765.934	2154.813	1861.835	2260.12	2807.159	2604.138	1821.42	1892.441	1948.18	2055.832	2413.674	2578.678
AC018638.	40.35812	31.0121	30.97314	13.51081	70.87903	59.37812	23.79105	37.96711	454.0749	225.7643	557.8015	163.7739	96.63993	55.84322	54.76833	43.69595	101.6112	96.47414	98.59387
AC018638.	402.6203	404.1577	285.7522	473.9175	313.4182	556.099	310.983	400.0437	717.882	423.6684	631.2916	272.3432	336.1836	283.8697	257.2155	505.389	538.8475	225.7131	252.7588
AC018638.	394.933	203.0793	463.598	256.7053	393.1571	568.6597	215.8188	307.441	518.2758	513.0134	440.9403	305.466	440.0201	284.8004	223.9633	315.7651	312.0184	310.3555	224.9733
AC018638.	294.9986	189.0738	266.7687	422.9922	181.6275	150.7291	488.5661	342.63	47.8588	148.9084	139.7516	420.4758	397.8687	245.7102	295.3578	412.2259	343.836	328.5582	307.4336
AC018639.	0	0	0	0	1.107485	0	0	0	0	0	0	0	0	0	0.978006	0	0	0	0
AC018641.	0	11.00429	4.995668	4.157171	6.64491	10.27698	0	12.03835	9.338302	2.882098	0	0	1.028084	2.792161	25.42815	3.297807	4.105505	0	0
AC018643.	0	0	0	0	2.21497	0	0	0	0	0	1.204755	0	0	0	0	0	0	0	0
AC018644.	9.609075	33.01288	38.96621	34.29666	5.537425	9.135096	11.04584	24.0767	9.338302	32.66377	15.66181	24.84211	6.168506	13.9608	6.846041	25.55801	26.68578	36.40534	31.37078
AC018645.	274.8196	170.0664	216.812	351.281	172.7676	202.114	282.0938	201.8739	224.1192	175.808	153.0039	187.696	249.8245	219.65	224.9413	225.0753	263.7787	301.2542	227.6622
AC018645.	638.0426	543.212	610.4707	725.4264	565.9248	607.4839	570.9851	474.1258	839.2799	613.8868	638.5201	563.0879	712.4625	509.104	529.1012	555.6805	641.4852	557.0017	579.9112
AC018647.	669.7525	1171.457	1043.096	877.1631	1433.085	951.1919	741.7708	921.3969	468.0824	903.0573	1143.312	689.1386	797.7935	688.733	785.3387	578.7652	674.3292	639.8238	611.282
AC018647.	3068.178	2896.13	3292.145	3165.686	2701.156	2582.948	3145.516	2907.725	2349.75	2166.377	1956.522	2797.038	2781.996	2826.598	2641.594	2767.685	2662.42	2853.268	2938.097
AC018648.	59.57627	74.02889	79.93069	66.51474	98.56616	75.36454	41.63433	66.67395	151.7474	147.9477	59.03299	47.84407	38.03912	24.19873	48.90029	53.58937	75.95184	55.51814	51.98586
AC018653.	4.804538	3.001171	14.987	6.235757	0	1.141887	3.398721	25.92876	0	3.842797	7.228529	9.200782	8.224675	12.09936	4.890029	9.06897	9.237386	1.820267	7.170464
AC018653.	189.2988	181.0707	388.663	579.9254	355.5027	302.6001	276.1461	205.578	184.4315	228.6464	307.2125	690.0587	632.2719	308.0684	302.2038	582.8874	486.5023	386.8067	371.0715
AC018659.	5.765445	9.003514	1.998267	15.58939	1.107485	12.56076	16.14392	10.1863	7.003726	0	4.81902	2.760235	4.112337	0	0	7.420066	6.158258	3.640534	2.688924
AC018665.	446.822	539.2104	431.6257	351.281	388.7272	365.4038	327.1269	458.3834	499.5991	724.3672	1110.784	510.6434	506.8456	441.1614	471.3988	335.5519	492.6606	451.4262	546.7478
AC018680.	0	0	2.997401	0	0	1.141887	0	0	9.338302	0.960699	0	0	0	0	0	0	0	0	0
AC018682.	210.4387	180.0703	283.754	113.2829	261.3664	176.9925	197.1258	242.6191	98.05217	137.38	115.6565	310.9864	271.4143	197.3127	230.8094	192.0973	138.5608	247.5563	222.2844
AC018682.	4.804538	8.003124	0.999134	2.078586	3.322455	0	3.398721	0.926027	4.669151	2.882098	2.40951	2.760235	1.028084	1.861441	3.912023	3.297807	4.105505	6.370934	1.792616
AC018685.	0.960908	0	0	0	0	0	0	0	0	0	0	0	1.028084	0	0	0	0	0	3.585232
AC018685.	0	7.002733	0.999134	2.078586	0	1.141887	3.398721	1.852054	0	0	1.204755	3.680313	1.028084	0	6.846041	0	1.026376	0.910133	6.274156
AC018688.	262.3278	364.1421	282.7548	288.9234	410.8769	198.6883	223.4659	81.49038	155.2493	136.4193	148.1848	161.9338	119.2578	188.0055	205.3812	33.80253	45.16056	143.8011	154.165
AC018690.	188.3379	125.0488	237.7938	169.4047	178.3051	179.2763	181.8316	118.5315	200.7735	200.7861	201.1941	193.2164	162.4373	158.2225	144.7449	146.7524	139.5872	138.3403	105.7643
AC018692.	0	1.00039	0	0	0	0	0	0	0	0.960699	0	0	0	0	0	0	1.026376	0	0
AC018692.	0	0	0.999134	0	0	0	0	0	0	0	0	0	1.028084	0	1.956012	0	0	0	0
AC018693.	2.882723	0	3.996535	1.039293	3.322455	0	0	7.408216	0	2.882098	0	0	1.028084	0.93072	0.978006	0.824452	4.105505	8.191201	0.896308
AC018693.	0	0	0	0	0	0	0	1.852054	0	0	0	0	0	0	0	0	0	0	0
AC018695.	0	0	0	0	0	0	0.84968	0	0	0	7.228529	0.920078	0	0.93072	0.978006	0	0	0	0
AC018695.	0.960908	14.00547	8.992203	3.117878	13.28982	1.141887	5.947762	4.630135	0	13.44979	9.638039	1.840156	7.196591	8.376483	2.934017	5.771163	4.105505	0.910133	8.066772
AC018695.	118.1916	127.0496	133.8839	87.30059	142.8656	99.34417	149.5437	154.6465	12.84017	92.22713	80.71858	162.8538	201.5045	146.1231	158.4369	138.5079	126.2443	170.1949	155.0613
AC018695.	1.921815	1.00039	3.996535	3.117878	6.64491	18.27019	8.496802	3.704108	56.02981	24.01748	39.75691	2.760235	8.224675	15.82225	18.58211	14.01568	4.105505	3.640534	8.963079
AC018695.	34.59267	8.003124	23.97921	0	5.537425	25.12151	1.69936	21.29862	21.01118	3.842797	15.66181	72.68618	79.1625	13.03008	7.824047	33.80253	40.02867	0	4.48154
AC018697.	7.68726	21.0082	27.97574	85.22201	4.42994	42.24982	52.68017	29.63287	3.501863	4.803496	19.27608	7.360626	22.61786	36.29809	38.14223	14.01568	13.34289	13.652	13.44462
AC018712.	11.53089	2.000781	0	0	0	0	0.84968	0	0	0	0	4.600391	0	0	1.956012	7.420066	5.131881	0	7.170464
AC018714.	1.921815	3.001171	5.994802	1.039293	0	2.283774	7.647122	0	0	0	0	0	0	7.445762	3.912023	0	4.105505	0	0
AC018716.	0.960908	0	0	0	0	0	0	0	0	0	0	0	1.028084	0.93072	0	0	0	0	0
AC018730.	0	2.000781	0	0	0	0	0.84968	0	0	0	0	0	0	0	0	0	0	0	0
AC018730.	0.960908	2.000781	0.999134	0	7.752394	0	9.346483	2.778081	1.167288	0.960699	1.204755	0.920078	1.028084	3.722881	0.978006	0.824452	1.026376	7.281067	14.34093
AC018731.	2.882723	1.00039	0	0	2.21497	0	0.84968	0	0	0	0	1.840156	1.028084	1.861441	0	0.824452	0	0	0
AC018735.	0	0	0	0	2.21497	1.141887	0	0	0	0	0	2.760235	0	0.93072	1.956012	0.824452	5.131881	1.820267	0
AC018737.	0	0	0	0	0	1.141887	0.84968	0	0	0	0	1.840156	0	0	0	0	0	0	0
AC018737.	6.726353	11.00429	17.98441	16.62868	8.859879	12.56076	6.797442	32.41095	14.00745	14.41049	9.638039	18.40156	12.33701	26.99089	4.890029	22.2602	26.68578	28.21414	13.44462
AC018738.	0.960908	4.001562	5.994802	1.039293	8.859879	2.283774	0	0	15.17474	2.882098	9.638039	0	1.028084	3.722881	0	0	0	3.640534	0.896308
AC018742.	97.05166	92.03592	71.93762	92.49706	95.2437	62.80379	22.09169	39.81916	410.8853	219.0394	240.951	26.68227	27.75828	195.4513	168.217	46.99375	71.84634	30.0344	24.20031
AC018752.	32.67086	43.01679	79.93069	134.0688	50.94431	50.24303	141.0469	58.3397	2.334575	24.01748	14.45706	59.80509	54.48847	80.97267	61.61437	73.37621	86.21561	40.956	66.32679
AC018754.	76.8726	68.02655	87.92376	34.29666	69.77155	53.66869	67.97442	74.08216	64.20083	67.24895	61.4425	63.4854	50.37613	79.11123	84.1085	48.64266	42.08143	65.52961	41.23017
AC018755.	20.17906	18.00703	4.995668	0	2.21497	4.567548	6.797442	11.11232	11.67288	23.05678	43.37118	1.840156	1.028084	11.16864	5.868035	4.946711	4.105505	2.7304	0.896308
AC018755.	16.33543	41.01601	8.992203	37.41454	34.33203	38.82416	30.58849	44.4493	66.5354	22.09608	37.3474	36.80313	42.15146	5.584322	24.45015	21.43575	26.68578	14.56213	25.99293
AC018755.	90.32531	85.03319	159.8614	201.6228	223.712	216.9585	104.5107	162.9808	331.5097	63.40615	30.11887	58.88501	79.1625	0	0	295.9782	205.2753	540.6192	185.5357
AC018761.	3.84363	1.00039	0	0	0	1.141887	0	0	9.338302	0	0	0	0	1.861441	5.868035	0.824452	0	0	4.48154
AC018761.	0	6.002343	0	14.5501	0	15.98642	11.04584	7.408216	12.84017	7.685594	6.023774	2.760235	11.30893	16.75297	19.56012	4.946711	4.105505	15.47227	7.170464
AC018766.	66.30262	64.02499	72.93676	62.35757	27.68712	35.3985	96.01387	64.82189	63.03354	54.75986	62.64725	60.72516	85.331	43.74385	82.15249	56.06272	100.5849	79.18161	54.67478
AC018767.	0.960908	3.001171	3.996535	1.039293	0	1.141887	0.84968	0	0	0	0	0	1.028084	0.93072	1.956012	0	0	0	0
AC018767.	0	0	0	0	2.21497	0	0	0	0	0	0	0	0	0	0	3.297807	0	0	3.585232
AC018793.	0	0	1.998267	3.117878	0	0	0	0	0	0	0	3.680313	0	3.722881	0	0.824452	6.158258	0	0
AC018793.	0	2.000781	0	0	0	0	0	0	0	0	0	0	0	0	0	1.648904	0	0	0
AC018793.	0	0	0	0	1.107485	0	0	0	0	0	0	0	0	0	0	0	0	0.910133	0
AC018797.	2.882723	4.001562	5.994802	17.66798	1.107485	2.283774	4.248401	2.778081	0	11.52839	0	8.280704	12.33701	0	6.846041	10.71787	7.184634	3.640534	7.170464
AC018797.	326.7086	297.116	441.6171	188.112	314.5257	364.262	316.9307	353.7423	498.4319	335.284	354.1979	346.8695	354.6891	329.475	377.5102	282.787	340.7569	349.4912	305.641
AC018797.	0.960908	2.000781	10.99047	13.51081	18.82724	1.141887	3.398721	23.15068	2.334575	8.646293	10.84279	0.920078	8.224675	7.445762	0.978006	4.946711	8.21101	14.56213	9.859387

AC018804.	5.765445	6.002343	74.93502	47.80747	7.752394	26.2634	6.797442	36.11505	19.84389	12.48909	12.04755	20.24172	47.29188	8.376483	0	28.85581	28.73854	14.56213	22.4077
AC018809.	123.9571	163.0636	138.8796	95.61493	136.2206	199.8302	130.0011	211.1342	239.294	216.1573	219.2654	124.2106	137.7633	130.3008	158.4369	115.4233	122.1388	129.2389	78.8751
AC018809.	1211.704	1332.52	1502.697	1495.542	1328.982	1367.981	1477.594	1393.671	1062.232	1610.132	1568.591	1335.034	1314.92	1511.49	1470.921	1325.719	1257.311	1323.334	1412.581
AC018816.	51.88901	72.02811	149.87	42.611	120.7159	47.95925	67.12474	92.6027	80.54285	69.17035	63.85201	80.04681	69.90974	85.62627	99.75659	66.7806	42.08143	137.4301	70.80833
AC018816.	0	1.00039	0	0	4.42994	1.141887	0	0	0	0	0	0	0	0	0	1.648904	4.105505	0	0
AC018832.	0.960908	0	0	0	1.107485	3.425661	3.398721	7.408216	2.334575	0	3.614265	0	6.168506	1.861441	1.956012	0	1.026376	0.910133	0
AC018845.	9.609075	0	13.98787	2.078586	1.107485	1.141887	0.84968	0	0	0.960699	0	1.840156	1.028084	1.861441	0	2.473355	3.079129	2.7304	0.896308
AC018845.	21.13997	31.0121	28.97488	16.62868	22.1497	20.55397	12.7452	13.89041	59.53167	55.72056	14.45706	25.76219	15.42127	20.47585	17.6041	28.85581	13.34289	20.93307	8.963079
AC018861.	3.84363	0	0	0	6.64491	0	0.84968	0	0	4.803496	0	0.920078	1.028084	0.93072	0	0.824452	0	3.640534	0
AC018861.	0	1.00039	0	0	0	0	0	0	0	0	0	0	0	0.93072	0	0	2.052753	0	0.896308
AC018866.	0	10.0039	17.98441	1.039293	15.50479	12.56076	0	0	4.669151	41.31007	32.52838	1.840156	1.028084	4.653602	22.49413	0	0	0.910133	0
AC018868.	5.765445	10.0039	4.995668	7.275049	7.752394	1.141887	12.7452	2.778081	4.669151	11.52839	31.32363	11.96102	14.39318	11.16864	6.846041	11.54233	9.237386	3.640534	3.585232
AC018868.	16.33543	20.00781	27.97574	29.1002	36.547	17.12831	16.9936	8.334243	0	17.29259	16.86657	17.48149	24.67402	15.82225	17.6041	26.38246	17.4484	8.191201	5.377848
AC018878.	0	0	0.999134	0	0	2.283774	0	0	0	0.960699	13.2523	0	0	0	0	0	0	0	2.688924
AC018889.	0	2.000781	0	0	1.107485	1.141887	0	7.408216	0	3.842797	2.40951	6.440548	0	8.376483	0.978006	0	1.026376	0	9.859387
AC018892.	0	0	0	0	0	0	3.398721	0	0	0	0	0	0	0	0	0	0	0	0
AC018892.	0	3.001171	2.997401	2.078586	0	0	1.69936	0	0	0	0	0.920078	0	0	0	0.824452	0	0.910133	3.585232
AC018892.	20.17906	18.00703	52.95408	41.57171	27.68712	28.54718	12.7452	33.33697	86.37929	24.01748	8.433284	44.16376	27.75828	42.81313	38.14223	13.19123	33.87042	35.4952	51.08955
AC018904.	478.5319	334.1304	517.5512	231.7623	369.9	446.4778	234.5117	261.1396	789.0865	596.5942	679.4818	339.5089	332.0713	318.3063	288.5117	349.5676	304.8337	259.388	201.6693
AC018904.	1.921815	0	0	1.039293	1.107485	3.425661	3.398721	9.26027	0	0	0	0	0	1.861441	0	0	0	0.910133	0.896308
AC018926.	71.10716	52.0203	54.95235	57.1611	58.6967	33.11472	76.47122	70.37805	51.36066	64.36685	69.87578	69.00587	88.41526	29.78305	54.76833	59.36053	36.94955	37.31547	55.57109
AC018926.	7.68726	3.001171	2.997401	5.196464	16.61227	4.567548	7.647122	18.52054	37.35321	53.79916	83.12809	23.00196	24.67402	12.09936	17.6041	13.19123	10.26376	13.652	13.44462
AC018926.	70.14625	97.03787	112.9021	120.558	87.49131	82.21586	115.5565	144.4602	60.69896	101.8341	55.41872	131.5712	95.61185	89.34915	101.7126	88.21635	99.5585	92.83361	97.69757
AC018943.	0	1.00039	0	3.117878	0	0	0.84968	0	0	0.960699	0	0	0	0	0	0	0	0	0
AC018946.	0.960908	0	0	0	2.21497	3.425661	0.84968	0	10.50559	10.56769	6.023774	3.680313	3.084253	0	0	5.771163	1.026376	0.910133	1.792616
AC018953.	0	0	0	0	0	0	0	0	0	0	0	0	0	0	5.868035	0	0	3.640534	0
AC018992.	20.17906	6.002343	28.97488	10.39293	5.537425	11.41887	14.44456	12.96438	9.338302	10.56769	1.204755	25.76219	9.252759	26.06017	14.67009	14.01568	8.21101	1.820267	16.13354
AC019050.	0	3.001171	1.998267	0	0	0	1.69936	0	0	0	0	0.920078	0	0	0	0.824452	0	0	0
AC019068.	0	0	0	0	0	0	0	3.704108	0	0	0	0	0	9.307203	6.846041	0.824452	6.158258	0	0.896308
AC019069.	64.3808	65.02538	67.94109	148.6189	86.38382	156.4385	50.13113	121.3095	226.4538	136.4193	103.6089	116.8499	75.05016	117.2708	64.54838	156.6458	138.5608	232.9942	129.0683
AC019070.	0.960908	1.00039	0.999134	0	0	0	0	7.408216	2.334575	0	0	1.840156	11.30893	0	2.934017	0	1.026376	0.910133	0.896308
AC019072.	3.84363	0	0.999134	6.235757	1.107485	0	11.04584	6.482189	0	0.960699	0	2.760235	0	1.861441	0.978006	0.824452	6.158258	3.640534	5.377848
AC019077.	0	1.00039	0.999134	7.275049	7.752394	1.141887	0	0.926027	3.501863	0	3.614265	3.680313	0	1.861441	5.868035	0	1.026376	1.820267	3.585232
AC019080.	1471.149	1576.615	2076.2	1886.316	1320.122	1300.609	2023.089	2040.964	1079.741	1741.748	1266.197	1788.632	1886.535	1682.742	1649.896	1699.195	1611.411	1541.766	1430.507
AC019080.	0.960908	2.000781	1.998267	6.235757	5.537425	6.851322	15.29424	8.334243	3.501863	1.921399	1.204755	12.8811	5.140422	0.93072	3.912023	2.473355	7.184634	8.191201	9.859387
AC019080.	19.21815	40.01562	60.94715	43.6503	100.7811	36.54038	26.34009	41.67122	264.9743	64.36685	134.9325	41.40352	38.03912	12.09936	35.20821	22.2602	22.58028	24.5736	15.23724
AC019080.	5.765445	7.002733	20.98181	16.62868	13.28982	4.567548	12.7452	4.630135	5.836439	5.764196	6.023774	11.96102	6.168506	5.584322	10.75806	14.01568	22.58028	5.4608	12.54831
AC019080.	172.9634	97.03787	177.8458	186.0334	127.3608	226.0936	175.0341	212.0602	182.0969	211.3538	131.3183	211.618	195.336	200.1049	179.9531	182.2039	166.273	124.6883	96.80126
AC019084.	0	4.001562	11.9896	4.157171	0	1.141887	4.248401	1.852054	0	2.882098	2.40951	1.840156	2.056169	4.653602	4.890029	1.648904	2.052753	3.640534	0.896308
AC019097.	35.55358	67.02616	86.92463	69.63262	6.64491	58.23624	71.37314	114.8274	19.84389	55.72056	22.89034	58.88501	35.98295	53.98178	53.79032	56.06272	46.18693	51.8776	35.85232
AC019109.	0	0	0	1.039293	0	0	0	0	7.003726	0	0	0	0	0	0	3.297807	0	0	0
AC019117.	9.609075	13.00508	45.96015	11.43222	23.25718	21.69585	16.14392	36.11505	10.50559	19.21399	57.82823	33.12282	39.06721	13.9608	30.31818	14.84013	12.31652	33.67494	34.95601
AC019117.	0	1.00039	0	3.117878	0	1.141887	1.69936	2.778081	1.167288	2.882098	1.204755	6.440548	6.168506	4.653602	5.868035	1.648904	0	10.01147	1.792616
AC019118.	3.84363	3.001171	1.998267	2.078586	8.859879	2.283774	3.398721	5.556162	1.167288	8.646293	4.81902	1.840156	0	4.653602	2.934017	0.824452	2.052753	1.820267	2.688924
AC019118.	3.84363	3.001171	0	0	4.42994	9.135096	0.84968	3.704108	4.669151	0.960699	1.204755	0	2.056169	2.792161	0	2.473355	6.158258	0.910133	0.896308
AC019129.	1.921815	8.003124	0.999134	1.039293	7.752394	13.70264	12.7452	0.926027	4.669151	10.56769	7.228529	4.600391	1.028084	0	7.824047	2.473355	0	8.191201	6.274156
AC019130.	0.960908	0	0	0	0	0	0	0	0	1.921399	0	0	0	0	0.978006	0	0	0	0
AC019131.	0.960908	0	13.98787	3.117878	8.859879	1.141887	3.398721	3.704108	2.334575	2.882098	14.45706	2.760235	7.196591	17.68369	1.956012	4.122259	0	0.910133	7.170464
AC019131.	248.8751	267.1042	298.741	267.0982	258.044	277.4785	275.2964	175.0191	814.7668	406.3758	348.1742	405.7545	470.8626	179.629	275.7976	230.8465	242.2248	192.9483	157.7502
AC019155.	61.49808	53.02069	41.96361	45.72888	37.65449	46.81737	60.3273	53.70957	5.836439	27.86028	16.86657	50.6043	44.20763	44.67457	42.05425	42.04704	31.81766	26.39387	35.85232
AC019155.	0	0	0.999134	1.039293	1.107485	1.141887	0.84968	0	0	1.921399	1.204755	0	0	0	0	0	0	0	0
AC019155.	2.882723	0	0	4.157171	0	4.567548	4.248401	0	0	0	0	4.600391	1.028084	1.861441	3.912023	1.648904	1.026376	0.910133	4.48154
AC019159.	0	0	1.998267	1.039293	0	0	2.549041	0	0	0	0	1.840156	0	0	2.934017	0	0	0	1.792616
AC019163.	3.84363	2.000781	4.995668	0	8.859879	15.98642	6.797442	2.778081	8.171014	1.921399	0	1.840156	1.028084	0	0.978006	8.244518	6.158258	3.640534	5.377848
AC019171.	18.25724	16.00625	15.98614	15.58939	14.3973	44.53359	8.496802	12.03835	42.02236	23.05678	78.30907	5.520469	11.30893	25.12945	16.6261	13.19123	26.68578	17.29253	6.274156
AC019176.	0	7.002733	0	0	0	0	0	0	0	0	0	0	0	0	0	0	0	0	0
AC019178.	0	0	0	0	0	0	0.84968	0	0	0	0	0	1.028084	0	0	0	0	0	0
AC019181.	48.04538	54.02108	83.92723	7.275049	63.12664	17.12831	63.72602	59.26573	24.51304	72.05244	50.5997	68.08579	55.51656	55.84322	58.68035	10.71787	4.105505	12.74187	28.68185
AC019185.	0	0	0	1.039293	0	0	0	3.704108	0	0	0	0	0	0	3.912023	0.824452	0	0	0
AC019185.	3.84363	0	4.995668	3.117878	4.42994	2.283774	0.84968	0.926027	0	0.960699	0	1.840156	1.028084	1.861441	0.978006	5.771163	2.052753	5.4608	3.585232
AC019186.	17.29634	29.01132	34.96968	12.47151	33.22455	28.54718	22.09169	37.96711	19.84389	24.97818	25.29985	24.84211	40.09529	16.75297	22.49413	14.84013	9.237386	48.23707	9.859387
AC019193.	0	0	0	0	0	0	0	0	0	0	0.960699	0	0	1.028084	0	0	3.297807	0	0	0

AC019193.	60.53717	38.01484	66.94196	23.90373	25.47215	31.97284	18.69297	48.15341	46.69151	58.60265	37.3474	27.60235	37.01104	38.15953	40.09824	33.80253	17.4484	21.8432	20.61508
AC019193.	7.68726	6.002343	1.998267	8.314342	4.42994	1.141887	12.7452	0	10.50559	14.41049	9.638039	2.760235	10.28084	3.722881	0.978006	1.648904	3.079129	7.281067	10.7557
AC019197.	61.49808	85.03319	67.94109	46.76817	75.30897	37.68227	48.43177	91.67668	73.53913	53.79916	63.85201	44.16376	60.65698	60.49682	98.77859	59.36053	66.71446	11.83173	14.34093
AC019205.	0	2.000781	3.996535	23.90373	7.752394	0	3.398721	0	0	14.41049	0	0	4.112337	0.93072	0	0.824452	0	7.281067	4.48154
AC019205.	693.7752	616.2405	704.3892	637.0865	538.2377	560.6665	691.6397	784.3449	564.9673	634.0615	739.7195	734.2224	743.305	656.1578	674.824	634.0035	631.2214	502.3936	544.0589
AC019205.	510.2419	379.148	285.7522	293.0806	364.3625	348.2755	242.1589	296.3287	456.4095	325.677	359.017	310.0664	224.1224	377.8724	364.7962	376.7745	422.867	499.6632	172.9874
AC019206.	26.90541	21.0082	20.98181	15.58939	25.47215	14.84453	39.08529	29.63287	1.167288	6.724895	2.40951	51.52438	40.09529	26.06017	29.34017	28.85581	14.36927	26.39387	22.4077
AC019209.	0	0	0	0	0	1.141887	0	0	0	0	0	0	0	0	5.868035	0	4.105505	0	0
AC019209.	6.726353	15.00586	20.98181	3.117878	15.50479	18.27019	20.39233	32.41095	12.84017	7.685594	0	20.24172	13.3651	9.307203	10.75806	4.122259	8.21101	14.56213	12.54831
AC019211.	0.960908	0	0	2.078586	0	0	0	2.778081	0	0	0	10.12086	1.028084	0	0	7.420066	9.237386	2.7304	2.688924
AC019226.	20.17906	17.00664	53.95322	18.70727	39.86946	21.69585	16.9936	32.41095	75.8737	23.05678	30.11887	23.92203	22.61786	17.68369	30.31818	20.6113	18.47477	33.67494	6.274156
AC019226.	13.45271	4.001562	0.999134	7.275049	1.107485	2.283774	3.398721	0.926027	0	11.52839	8.433284	8.280704	18.50552	2.792161	10.75806	1.648904	0	4.550667	0
AC019227.	1.921815	2.000781	1.998267	3.117878	1.107485	1.141887	4.248401	1.852054	7.003726	2.882098	1.204755	3.680313	12.33701	0	3.912023	0	0	6.370934	1.792616
AC019235.	0.960908	0	1.998267	6.235757	0	0	0	0	1.167288	0	3.614265	3.680313	0	0	2.934017	0	0	0	0
AC019254.	22.10087	24.00937	25.97748	8.314342	17.71976	31.97284	22.94137	37.04108	25.68033	34.58517	49.39495	44.16376	47.29188	24.19873	27.38416	43.69595	39.0023	44.59654	34.0597
AC019254.	2.882723	0	0.999134	0	1.107485	11.41887	2.549041	7.408216	0	4.803496	0	0.920078	3.084253	0.93072	2.934017	13.19123	3.079129	1.820267	3.585232
AC019257.	68.22443	122.0476	88.9229	87.30059	80.8464	134.7427	87.51706	42.59724	134.2381	118.166	167.4609	62.56532	89.44334	107.9636	92.91055	76.67402	102.6376	89.19307	183.7431
AC019257.	27.86632	28.01093	47.95842	6.235757	4.42994	39.96605	50.98081	11.11232	1.167288	36.50657	28.91412	25.76219	18.50552	26.06017	31.29619	32.15362	20.52753	30.94454	77.97879
AC019270.	7.68726	0	0	1.039293	1.107485	0	4.248401	6.482189	1.167288	7.685594	0	1.840156	3.084253	5.584322	10.75806	5.771163	2.052753	1.820267	0
AC019278.	0	0	0	2.078586	0	0	5.098081	0	0	0	0	0	0	2.792161	0.978006	0	0	0	0
AC019288.	0	0	0	0	0	0	0	0	0	0	0	2.760235	0	0	0	0	0	0	0
AC019294.	0	1.00039	0	0	0	0	0	0	0	0	0	2.760235	1.028084	0	0	0.824452	4.105505	0	0
AC019294.	33.63176	44.01718	47.95842	20.78586	49.83682	33.11472	36.53625	58.3397	37.35321	30.74238	62.64725	60.72516	32.8987	48.39746	48.90029	33.80253	41.05505	76.45121	60.94894
AC019294.	3.84363	5.001952	0	3.117878	5.537425	3.425661	6.797442	6.482189	3.501863	9.606993	9.638039	7.360626	13.3651	1.861441	15.64809	11.54233	1.026376	6.370934	8.963079
AC019294.	0	0	0	0	0	0	0	0	0	0	0	0	0	0	3.912023	4.122259	1.026376	0	0
AC019322.	2.882723	2.000781	5.994802	2.078586	7.752394	0	3.398721	2.778081	0	1.921399	0	4.600391	8.224675	0	4.890029	4.122259	1.026376	1.820267	2.688924
AC019322.	0	1.00039	0	3.117878	3.322455	0	11.04584	3.704108	0	4.803496	0	1.840156	7.196591	3.722881	6.846041	6.595615	6.158258	5.4608	7.170464
AC019330.	0	0	0	0	1.107485	0	0.84968	0	0	0	0	0	0	0	0	0	0	0	0
AC020549.	13.45271	1.00039	0.999134	9.353635	9.967364	5.709435	22.09169	16.66849	4.669151	18.25329	9.638039	8.280704	16.44935	5.584322	10.75806	4.946711	15.39564	6.370934	13.44462
AC020550.	0.960908	1.00039	3.996535	4.157171	0	0	2.549041	0.926027	4.669151	9.606993	7.228529	4.600391	7.196591	0	0.978006	2.473355	8.21101	0	0
AC020558.	0	0	0	3.117878	0	1.141887	1.69936	0.926027	0	0	0	0	0	0.93072	0	1.648904	3.079129	1.820267	3.585232
AC020558.	122.9962	123.048	98.91423	41.57171	118.5009	107.3374	74.77186	118.5315	143.5764	166.201	244.5652	146.2924	129.5386	126.578	111.4927	51.94047	117.0069	107.3957	136.2388
AC020558.	17.29634	3.001171	23.97921	24.94303	14.3973	23.97963	17.84329	19.44657	47.8588	8.646293	7.228529	9.200782	13.3651	4.653602	13.69208	23.9091	28.73854	6.370934	17.92616
AC020559.	24.02269	24.00937	33.97054	48.84676	28.79461	30.83095	28.03945	38.89314	8.171014	20.17468	7.228529	43.24368	40.09529	13.9608	26.40616	30.50472	16.42202	23.66347	21.51139
AC020571.	11.53089	13.00508	19.98267	1.039293	27.68712	6.851322	3.398721	5.556162	0	0	0	6.440548	5.140422	4.653602	14.67009	3.297807	10.26376	10.9216	11.652
AC020593.	0.960908	0	0	0	0	0	0	0	9.338302	3.842797	0	1.840156	0	0	0	0	0	0.910133	0
AC020594.	112.4262	61.02382	52.95408	56.12181	54.26676	52.5268	56.0789	37.04108	117.8961	87.42363	89.15186	49.68422	61.68506	62.35826	79.21847	53.58937	43.1078	44.59654	50.19325
AC020595.	0	0	0	0	0	0	0	7.408216	0	0	0	0	0	0	0	0	0	0	0
AC020604.	71.10716	47.01835	74.93502	41.57171	43.19191	68.51322	26.34009	53.70957	89.88116	118.166	91.56137	67.16571	81.21867	87.48771	78.24047	114.5988	141.6399	40.956	23.30401
AC020611.	0	1.00039	3.996535	0	0	0	1.69936	11.11232	0	0	0	3.680313	5.140422	0.93072	1.956012	0	0	1.820267	0
AC020611.	2.882723	6.002343	9.991337	0	9.967364	5.709435	11.89552	17.59451	4.669151	2.882098	1.204755	3.680313	9.252759	6.515042	4.890029	0	0	0.910133	5.377848
AC020612.	31.70995	38.01484	27.97574	31.17878	19.93473	13.70264	22.94137	42.59724	19.84389	28.82098	18.07132	57.04485	48.31997	29.78305	40.09824	14.01568	27.71216	9.101334	25.09662
AC020612.	1.921815	2.000781	0	0	0	1.141887	1.69936	1.852054	1.167288	4.803496	0	0.920078	0	0	0	0	0	0	0
AC020612.	19.21815	24.00937	33.97054	21.82515	3.322455	21.69585	29.73881	18.52054	26.84762	45.15286	24.0951	12.8811	30.84253	26.06017	29.34017	14.84013	13.34289	12.74187	18.82247
AC020629.	0	0	0	0	0	1.141887	0.84968	0	0	0	0	0.920078	1.028084	6.515042	0	0.824452	1.026376	0	1.792616
AC020633.	8.648168	0	7.993069	11.43222	2.21497	3.425661	15.29424	12.96438	0	2.882098	9.638039	16.56141	18.50552	4.653602	3.912023	24.73355	2.052753	70.08027	23.30401
AC020634.	8.648168	6.002343	29.97401	7.275049	5.537425	11.41887	29.73881	5.556162	17.50932	4.803496	13.2523	3.680313	9.252759	13.03008	8.802052	9.06897	13.34289	1.820267	1.792616
AC020636.	0	0	0	0	0	0	18.69297	1.852054	0	0	0	0	1.028084	0	0	0.824452	0	0	1.792616
AC020636.	0	0	0	1.039293	0	0	0	0	0	0	0	0	0	0	3.912023	0	0	1.820267	0
AC020637.	16.33543	12.00469	3.996535	5.196464	0	34.25661	15.29424	6.482189	37.35321	20.17468	9.638039	5.520469	17.47743	0.93072	3.912023	12.36678	5.131881	3.640534	6.274156
AC020656.	9.609075	5.001952	31.97228	7.275049	26.57964	15.98642	17.84329	15.74246	15.17474	24.97818	3.614265	22.08188	34.95487	8.376483	9.780058	28.03136	11.29014	33.67494	32.26709
AC020658.	0.960908	0	0.999134	1.039293	2.21497	0	0	0.926027	0	0	0	0	1.028084	0	0.978006	2.473355	1.026376	0	0.896308
AC020658.	0	0	0	1.039293	0	0	0	3.704108	0	0	7.228529	0	1.028084	0	0.978006	0.824452	4.105505	17.29253	0
AC020658.	0	0	0	8.314342	0	0	0	0	4.669151	1.921399	0	0	0	0	0	6.595615	1.026376	10.01147	0.896308
AC020658.	32.67086	58.02265	60.94715	36.37525	83.06137	94.77662	33.13753	69.45203	242.7958	83.58083	109.6327	64.40548	52.4323	46.53602	32.27419	37.10033	34.89679	91.01334	51.08955
AC020658.	0	0	0	0	0	0	2.549041	0.926027	4.669151	0	0	0	0	3.722881	0	4.122259	14.36927	4.550667	0.896308
AC020658.	0	0	0	4.157171	0	0	0	0	0	0	0	0	0	0.93072	3.912023	0	4.105505	0.910133	0.896308
AC020659.	737.0161	416.1624	554.5192	179.7976	541.5601	509.2816	270.1983	313.9232	1411.251	649.4327	873.4473	506.9631	650.7774	453.2608	410.7624	160.7681	262.7523	189.3078	253.6551
AC020659.	4.804538	1.00039	0	0	0	2.283774	0	11.11232	0	7.685594	6.023774	4.600391	8.224675	3.722881	0.978006	1.648904	3.079129	0.910133	7.170464
AC020661.	39.39721	55.02147	52.95408	68.59332	50.94431	79.93209	38.23561	50.93149	63.03354	70.13105	112.0422	53.36454	37.01104	54.9125	23.47214	30.50472	46.18693	37.31547	76.18618
AC020661.	3.84363	0	5.994802	8.314342	1.107485	1.141887	4.248401	5.556162	14.00745	7.685594	3.614265	2.760235	0	0	6.846041	2.473355	3.079129	0	10.7557
AC020661.	0.960908	0	0.999134	0	13.28982	0	0	0	0	1.921399	0	4.600391	1.028084	0	0	0	0	0	0

AC020661.	1.921815	4.001562	0.999134	1.039293	0	1.141887	11.04584	3.704108	7.003726	0.960699	1.204755	11.96102	12.33701	4.653602	5.868035	3.297807	1.026376	9.101334	6.274156
AC020661.	0	0	0	1.039293	4.42994	0	5.098081	1.852054	4.669151	0	0	0	1.028084	0.93072	0.978006	1.648904	1.026376	0	3.585232
AC020663.	7.68726	2.000781	1.998267	0	4.42994	4.567548	4.248401	1.852054	8.171014	0.960699	2.40951	2.760235	1.028084	3.722881	1.956012	1.648904	6.158258	4.550667	7.170464
AC020663.	0	2.000781	1.998267	0	0	3.425661	3.398721	1.852054	18.6766	11.52839	9.638039	2.760235	3.084253	0.93072	2.934017	6.595615	6.158258	5.4608	9.859387
AC020663.	22.10087	56.02186	56.95062	18.70727	28.79461	11.41887	34.83689	41.67122	94.55031	34.58517	32.52838	23.92203	27.75828	27.92161	14.67009	38.74924	37.97592	36.40534	19.71877
AC020687.	0	0	0	0	0	0	0	0	0	0	0	1.840156	1.028084	0	0.978006	0	0	0	0
AC020699.	0	0	0	2.078586	0	0	0	0	0	0	0	0	0	0	0	0	0	0	0
AC020703.	8.648168	4.001562	6.993936	2.078586	5.537425	13.70264	11.04584	6.482189	9.338302	0.960699	2.40951	25.76219	8.224675	7.445762	6.846041	20.6113	23.60665	3.640534	8.066772
AC020704.	4.804538	3.001171	13.98787	0	6.64491	2.283774	0	0	4.669151	1.921399	0	6.440548	0	5.584322	2.934017	0	4.105505	0	5.377848
AC020728.	7.68726	7.002733	48.95755	0	15.50479	11.41887	16.14392	31.48492	26.84762	14.41049	43.37118	11.96102	13.3651	30.71377	14.67009	6.595615	22.58028	0.910133	0.896308
AC020741.	4.804538	8.003124	0	2.078586	0	5.709435	5.947762	0.926027	1.167288	0.960699	1.204755	15.64133	7.196591	0.93072	0.978006	0.824452	12.31652	4.550667	7.170464
AC020743.	0	0	3.996535	0	0	0	0	0	1.167288	0	0	0	0	3.722881	0	0	0	0	0
AC020743.	0	0	0	1.039293	0	0	0	0.926027	0	0	0	0	0	0	0.978006	4.946711	0	9.101334	6.274156
AC020763.	0	2.000781	0	4.157171	0	1.141887	2.549041	4.630135	0	1.921399	0	0.920078	1.028084	2.792161	0.978006	0.824452	0	4.550667	0
AC020763.	54.77173	66.02577	76.93329	49.88605	52.05179	70.79699	90.91579	37.04108	56.02981	70.13105	44.57593	126.9708	128.5105	107.0328	88.99853	77.49847	96.47937	95.56401	112.9348
AC020763.	130.6834	107.0418	203.8233	75.86837	159.4778	180.4181	121.5043	192.6136	245.1304	210.3931	436.1213	143.5322	199.4484	98.65635	128.1188	96.46086	106.7431	156.5429	162.2317
AC020763.	0	5.001952	24.97834	0	0	0	0	3.704108	0	0	0	0.920078	2.056169	0	2.934017	4.946711	7.184634	0	3.585232
AC020765.	0	0	2.997401	3.117878	0	0	0	0	0	0	0	5.520469	0	0.93072	0	0	0	0	0
AC020765.	8.648168	1.00039	10.99047	2.078586	1.107485	2.283774	0.84968	11.11232	4.669151	4.803496	16.86657	3.680313	2.056169	15.82225	5.868035	12.36678	8.21101	14.56213	7.170464
AC020765.	48.04538	33.01288	27.97574	43.6503	21.04221	27.40529	47.58209	31.48492	14.00745	51.87776	46.98544	57.04485	111.0331	49.32818	27.38416	20.6113	17.4484	20.02294	23.30401
AC020765.	37.47539	49.01913	69.93936	49.88605	65.34161	23.97963	79.02026	45.37532	44.35693	51.87776	40.96167	61.64524	54.48847	28.85233	57.70234	26.38246	30.79129	17.29253	27.78555
AC020779.	8.648168	11.00429	8.992203	15.58939	13.28982	11.41887	14.44456	12.03835	9.338302	18.25329	10.84279	11.96102	12.33701	17.68369	24.45015	4.946711	4.105505	4.550667	8.066772
AC020779.	0.960908	6.002343	7.993069	1.039293	2.21497	0	3.398721	6.482189	0	0	0	1.840156	8.224675	6.515042	2.934017	0.824452	0	4.550667	3.585232
AC020890.	8.648168	15.00586	10.99047	24.94303	7.752394	19.41208	30.58849	10.1863	35.01863	16.33189	14.45706	11.04094	16.44935	9.307203	1.956012	13.19123	21.5539	36.40534	14.34093
AC020891.	0.960908	2.000781	0.999134	4.157171	14.3973	0	4.248401	8.334243	1.167288	0	0	0.920078	3.084253	8.376483	1.956012	12.36678	4.105505	3.640534	5.377848
AC020891.	0	0	0	0	4.42994	0	0	13.89041	0	0	0	0	1.028084	0	3.912023	0	0	0	0
AC020891.	0	0	0	0	0	0	0	0	0	0	0	0	0	0	1.956012	0	0	0	0
AC020891.	0.960908	2.000781	0.999134	0	7.752394	1.141887	1.69936	0	0	10.56769	2.40951	8.280704	5.140422	1.861441	5.868035	4.946711	19.50115	1.820267	2.688924
AC020892.	10.56998	16.00625	13.98787	30.13949	15.50479	19.41208	11.89552	7.408216	22.17847	16.33189	10.84279	8.280704	9.252759	12.09936	7.824047	15.66458	10.26376	3.640534	15.23724
AC020892.	29.78813	24.00937	7.993069	41.57171	18.82724	21.69585	13.59488	5.556162	28.01491	11.52839	13.2523	11.04094	20.56169	28.85233	13.69208	20.6113	27.71216	14.56213	29.57816
AC020893.	2.882723	0	0	4.157171	1.107485	0	1.69936	3.704108	0	0.960699	0	0.920078	3.084253	3.722881	0.978006	0.824452	0	0	0
AC020893.	4.804538	1.00039	7.993069	2.078586	5.537425	1.141887	0.84968	8.334243	4.669151	0.960699	7.228529	1.840156	4.112337	1.861441	7.824047	6.595615	1.026376	1.820267	3.585232
AC020895.	0	2.000781	0	1.039293	7.752394	0	6.797442	0	0	0	0	2.760235	0	0	0.978006	4.122259	1.026376	10.01147	4.48154
AC020895.	1.921815	17.00664	17.98441	16.62868	11.07485	0	2.549041	12.03835	0	6.724895	4.81902	11.04094	9.252759	0	0.978006	22.2602	8.21101	50.96747	73.49725
AC020897.	0	0	0	0	0	0	0	0	0	0	0	0	0	0	0	0.824452	0	0	0.896308
AC020898.	553.4827	493.1925	421.6344	326.3379	979.0167	902.0907	252.355	661.1833	1237.325	875.197	1157.769	429.6765	458.5256	663.6036	637.6598	480.6554	551.164	507.8544	274.2702
AC020899.	0	0	8.992203	0	1.107485	1.141887	0	0	2.334575	0	1.204755	0	3.084253	1.861441	0.978006	2.473355	0	50.96747	25.09662
AC020900.	6.726353	8.003124	22.98007	18.70727	15.50479	14.84453	13.59488	16.66849	11.67288	9.606993	13.2523	17.48149	12.33701	23.26801	10.75806	5.771163	12.31652	14.56213	11.652
AC020904.	0.960908	20.00781	1.998267	0	0	2.283774	9.346483	5.556162	0	0	0	0	0	0	1.956012	3.297807	0	9.101334	0
AC020904.	48.04538	61.02382	108.9056	40.53242	40.97694	59.37812	59.47762	85.19449	37.35321	50.91706	36.14265	78.20665	44.20763	64.2197	77.26246	52.76492	99.5585	69.17014	87.83818
AC020905.	4.804538	0	0.999134	0	0	0	0.84968	0	0	0	1.204755	0	2.056169	0	0.978006	0	0	3.640534	0.896308
AC020907.	101.8562	172.0672	142.8761	35.33595	191.5949	149.5872	93.46483	176.8712	149.4128	209.4324	337.3314	247.501	242.6279	149.846	160.393	178.0816	199.117	255.7475	301.1595
AC020907.	8.648168	18.00703	0.999134	4.157171	4.42994	6.851322	1.69936	1.852054	0	7.685594	0	2.760235	0	0.93072	2.934017	0	0	6.370934	7.170464
AC020907.	183.5333	134.0523	176.8467	106.0079	139.5431	180.4181	164.838	141.6821	229.9557	247.8604	254.2033	199.657	123.3701	176.8369	189.7331	230.0221	174.484	308.5352	249.1736
AC020907.	120.1134	109.0426	113.9012	48.84676	58.6967	134.7427	103.661	58.3397	93.38302	82.62014	179.5085	115.0098	122.342	84.69555	84.1085	52.76492	82.1101	108.3059	93.21603
AC020908.	6.726353	9.003514	15.98614	3.117878	13.28982	5.709435	5.098081	13.89041	0	4.803496	3.614265	7.360626	5.140422	8.376483	10.75806	3.297807	0	11.83173	10.7557
AC020908.	0	0	0	0	0	0	0.84968	0	0	0	0	0	1.028084	0	0	0	0	0	0
AC020908.	0.960908	10.0039	3.996535	0	11.07485	2.283774	4.248401	0	12.84017	5.764196	7.228529	3.680313	6.168506	9.307203	19.56012	4.122259	1.026376	6.370934	3.585232
AC020910.	8.648168	4.001562	10.99047	3.117878	16.61227	4.567548	3.398721	2.778081	1.167288	2.882098	4.81902	2.760235	4.112337	13.9608	2.934017	0.824452	12.31652	0.910133	4.48154
AC020910.	0	0	2.997401	8.314342	8.859879	1.141887	0.84968	0	0	2.882098	14.45706	0	8.224675	0.93072	0.978006	0	0	0	0.896308
AC020910.	23.06178	17.00664	57.94975	7.275049	14.3973	21.69585	11.04584	49.07943	7.003726	23.05678	22.89034	17.48149	24.67402	11.16864	12.71408	14.84013	17.4484	22.75334	25.09662
AC020910.	197.947	251.098	252.7808	181.8762	199.3473	205.5397	169.936	170.389	416.7217	297.8168	449.3736	167.4542	169.6339	295.0383	243.5235	164.8904	224.7764	212.9712	206.1508
AC020910.	987.8129	774.3022	774.3286	1024.743	414.1994	761.6386	790.2026	820.46	723.7184	552.4021	450.5783	695.5791	822.4675	785.5279	756.9765	986.0444	1066.405	749.9499	742.143
AC020910.	6.726353	1.00039	14.987	4.157171	35.43952	5.709435	12.7452	4.630135	0	13.44979	0	10.12086	8.224675	3.722881	9.780058	0.824452	10.26376	1.820267	15.23724
AC020911.	16.33543	4.001562	8.992203	12.47151	17.71976	11.41887	13.59488	11.11232	11.67288	7.685594	13.2523	9.200782	10.28084	5.584322	14.67009	8.244518	14.36927	22.75334	20.61508
AC020911.	1.921815	2.000781	1.998267	0	0	5.709435	2.549041	5.556162	14.00745	1.921399	0	17.48149	7.196591	9.307203	3.912023	0.824452	2.052753	5.4608	8.963079
AC020912.	178.7288	45.01757	129.8874	205.78	21.04221	58.23624	86.66738	63.89587	82.87743	39.38867	62.64725	68.08579	69.90974	130.3008	103.6686	328.9563	182.695	334.9291	190.9136
AC020913.	0	0	9.991337	0	0	4.567548	1.69936	0	3.501863	0.960699	0	0	2.056169	0	2.934017	0	2.052753	0.910133	0
AC020913.	6.726353	5.001952	8.992203	21.82515	11.07485	2.283774	13.59488	3.704108	1.167288	18.25329	6.023774	10.12086	4.112337	13.9608	9.780058	12.36678	11.29014	26.39387	4.48154
AC020913.	14.41361	12.00469	6.993936	27.02161	24.36467	5.709435	22.94137	27.78081	4.669151	10.56769	21.68559	25.76219	18.50552	4.653602	21.51613	28.03136	17.4484	22.75334	24.20031
AC020914.	0	4.001562	0	2.078586	0	1.141887	0	0	0	0	0	11.04094	1.028084	0	3.912023	0.824452	0	0	4.48154
AC020914.	8.648168	6.002343	1.998267	13.51081	7.752394	1.141887	1.69936	14.81643	4.669151	4.803496	2.40951	0	0	3.722881	3.912023	4.946711	1.026376	5.4608	0

AC020914.	5.765445	1.00039	12.98874	6.235757	8.859879	7.993209	0.84968	1.852054	109.725	0	1.204755	4.600391	4.112337	4.653602	7.824047	5.771163	6.158258	6.370934	1.792616
AC020915.	1115.614	1390.543	1501.698	1723.147	1526.114	1217.252	1861.649	1347.369	1222.15	1541.922	1539.677	1485.926	1454.739	1368.159	1494.393	1497.205	1510.826	1811.165	1664.444
AC020915.	55.73264	59.02304	67.94109	94.57564	49.83682	53.66869	66.27506	74.08216	11.67288	52.83846	46.98544	69.92595	56.54464	69.80402	76.28445	59.36053	60.5562	73.72081	72.60094
AC020915.	101.8562	175.0683	226.8033	115.3615	108.5335	192.9789	176.7335	127.7917	100.3867	104.7162	210.8321	165.6141	162.4373	134.0237	160.393	113.7744	107.7695	149.2619	135.3425
AC020915.	444.9002	421.1644	473.5894	334.6523	400.9095	415.6469	333.0747	551.9121	461.0787	452.4893	455.3973	379.0722	350.5768	510.9654	564.3094	461.693	406.445	465.0782	448.154
AC020915.	0.960908	4.001562	5.994802	0	0	0	0	12.03835	12.84017	1.921399	1.204755	1.840156	1.028084	0.93072	2.934017	0	1.026376	5.4608	13.44462
AC020915.	0	0	0	0	0	0	0.84968	0.926027	0	0	0	0	0	0	0.978006	0	0	0	0
AC020916.	1681.588	1877.733	1556.65	1406.163	2986.887	2111.349	1291.514	1402.931	3324.435	2803.32	4227.485	2358.161	1883.451	1959.166	1985.352	1744.54	1792.053	3166.354	2427.202
AC020916.	2.882723	7.002733	15.98614	12.47151	2.21497	2.283774	5.947762	0	0	3.842797	0	2.760235	3.084253	0.93072	1.956012	1.648904	0	3.640534	6.274156
AC020917.	4.804538	1.00039	3.996535	8.314342	3.322455	0	6.797442	0	0	1.921399	30.11887	3.680313	1.028084	0	11.73607	1.648904	0	1.820267	0.896308
AC020917.	6.726353	1.00039	11.9896	0	2.21497	2.283774	3.398721	0	21.01118	3.842797	7.228529	3.680313	4.112337	4.653602	0.978006	0	1.026376	0.910133	3.585232
AC020917.	0.960908	2.000781	0.999134	7.275049	7.752394	5.709435	1.69936	4.630135	5.836439	0.960699	1.204755	1.840156	2.056169	9.307203	0.978006	4.122259	1.026376	2.7304	0
AC020917.	1015.679	1068.417	1095.05	442.7387	1093.088	1221.819	635.5608	939.9174	986.3581	1360.35	1743.28	948.6007	932.4725	988.425	925.1935	633.179	671.2501	866.447	986.8351
AC020922.	0	0	0	0	0	2.283774	0	0	0	0	0	0	0	0	0	0	0	0	0
AC020922.	0	0	1.998267	0	0	0	0	0	0	0	4.81902	0	0	0	0	0	0	0	0.896308
AC020928.	97.05166	53.02069	74.93502	107.0472	65.34161	69.65511	108.7591	75.93422	66.5354	61.48475	56.62348	69.92595	46.2638	72.59618	72.37243	69.25395	99.5585	81.00187	59.15632
AC020928.	22.10087	5.001952	4.995668	4.157171	2.21497	7.993209	9.346483	0.926027	0	5.764196	13.2523	5.520469	3.084253	0.93072	8.802052	2.473355	2.052753	1.820267	1.792616
AC020931.	53.81082	38.01484	34.96968	82.10413	45.40688	66.22945	49.28145	64.82189	35.01863	45.15286	72.28529	63.4854	61.68506	54.9125	46.94428	48.64266	72.87271	91.92348	56.4674
AC020934.	0.960908	0	3.996535	3.117878	1.107485	0	1.69936	0	0	0.960699	7.228529	0.920078	5.140422	3.722881	2.934017	2.473355	4.105505	1.820267	1.792616
AC020934.	0	0	0	0	0	0	0	4.630135	0	0	0	0	0	0	0	0	1.026376	0	0
AC020937.	4.804538	1.00039	24.97834	3.117878	15.50479	15.98642	20.39233	44.4493	7.003726	22.09608	27.70936	22.08188	10.28084	5.584322	19.56012	15.66458	23.60665	6.370934	0.896308
AC020951.	130.6834	117.0457	302.7375	252.5481	225.9269	62.80379	390.0032	248.1752	9.338302	78.77734	24.0951	322.0274	269.3581	135.8852	190.7111	159.9437	159.0883	137.4301	206.1508
AC020978.	4.804538	2.000781	3.996535	2.078586	9.967364	0	3.398721	12.03835	17.50932	0	0	9.200782	7.196591	0	6.846041	1.648904	1.026376	2.7304	0
AC020978.	394.933	395.1542	579.4975	688.0118	474.0035	237.5125	676.3455	733.4134	168.0894	273.7993	179.5085	934.7995	798.8216	575.1851	667.978	1038.809	849.8395	587.0361	370.1752
AC020978.	47.08447	38.01484	58.94889	55.08252	74.20149	33.11472	85.8177	50.93149	4.669151	24.01748	18.07132	64.40548	89.44334	40.95169	44.98827	37.92478	59.52982	58.24854	57.36371
AC020978.	24.02269	11.00429	10.99047	32.21808	1.107485	11.41887	15.29424	7.408216	17.50932	20.17468	10.84279	13.80117	16.44935	10.23792	7.824047	48.64266	24.63303	13.652	25.09662
AC020978.	49.00628	53.02069	35.96881	30.13949	43.19191	37.68227	58.62794	45.37532	157.5838	84.54153	120.4755	39.56336	59.62889	41.88241	50.8563	24.73355	15.39564	36.40534	34.0597
AC020978.	0	4.001562	1.998267	0	0	0	3.398721	0	0	0	0	0	8.224675	0	0	3.297807	2.052753	0	0
AC020978.	264.2496	230.0898	215.8129	144.4617	261.3664	184.9857	281.2442	319.4793	218.2828	210.3931	197.5798	248.4211	288.8917	248.5023	226.8974	134.3856	156.0092	192.0382	154.165
AC020980.	5.765445	8.003124	20.98181	14.5501	7.752394	28.54718	7.647122	2.778081	1.167288	12.48909	22.89034	6.440548	18.50552	9.307203	10.75806	20.6113	25.65941	15.47227	8.066772
AC020983.	0	2.000781	0.999134	0	1.107485	2.283774	0.84968	1.852054	1.167288	0	0	0.920078	0	0	0.978006	0.824452	0	0	0
AC021001.	2.882723	4.001562	7.993069	0	1.107485	1.141887	13.59488	4.630135	0	9.606993	0	0	0	7.445762	0.978006	0.824452	2.052753	3.640534	4.48154
AC021006.	0	0	0	0	0	0	0	0	0	0	0	0	1.028084	0	0	0.824452	0	0	0
AC021006.	4.804538	2.000781	7.993069	0	3.322455	9.135096	4.248401	1.852054	0	0.960699	9.638039	0	0	3.722881	2.934017	0.824452	2.052753	0	0
AC021006.	0	0	0	0	0	0	0.84968	0	0	0	0	0	0	0	1.956012	0	0	0	0
AC021016.	0.960908	2.000781	0.999134	0	0	1.141887	0	0	4.669151	0.960699	0	4.600391	0	0	2.934017	0	0	0	0
AC021016.	962.8293	1089.425	833.2775	1711.715	748.6598	1181.853	625.3647	837.1284	952.5068	1223.931	1079.46	498.6824	463.6661	1128.033	1038.642	1353.75	1649.387	795.4566	881.0707
AC021028.	7.68726	25.00976	23.97921	8.314342	3.322455	3.425661	3.398721	6.482189	21.01118	0	18.07132	25.76219	37.01104	24.19873	42.05425	14.84013	8.21101	36.40534	40.33386
AC021054.	1358.723	979.3822	1378.804	1107.886	1067.615	1644.317	1085.891	1353.852	1693.734	1190.306	1071.027	1137.217	1135.005	1690.188	1634.248	1260.587	1224.467	1135.847	1067.503
AC021072.	15.37452	8.003124	3.996535	9.353635	18.82724	5.709435	4.248401	4.630135	9.338302	5.764196	0	14.72125	14.39318	5.584322	4.890029	5.771163	8.21101	26.39387	25.09662
AC021074.	6.726353	4.001562	0	1.039293	7.752394	3.425661	0.84968	6.482189	5.836439	0.960699	7.228529	6.440548	1.028084	5.584322	4.890029	9.06897	5.131881	11.83173	1.792616
AC021074.	69.18534	76.02967	48.95755	129.9116	52.05179	53.66869	109.6088	73.15614	3.501863	15.37119	0	73.60626	86.35909	84.69555	88.99853	104.7054	126.2443	74.63094	103.9717
AC021074.	4.804538	0	0	1.039293	0	1.141887	3.398721	0	7.003726	0	0	0.920078	1.028084	0	2.934017	0	0	0.910133	0
AC021078.	4015.633	4429.729	5716.044	4653.953	6258.397	4640.629	5734.492	6778.518	2340.412	4261.662	3675.707	6441.468	5837.463	5341.404	5505.195	4743.896	3936.153	5666.491	4932.383
AC021079.	0	8.003124	3.996535	8.314342	2.21497	0	0.84968	0	0	7.685594	4.81902	0	0	2.792161	9.780058	8.244518	0	0	0
AC021086.	24.02269	19.00742	29.97401	25.98232	21.04221	27.40529	17.84329	14.81643	4.669151	6.724895	1.204755	30.36258	37.01104	18.61441	18.58211	16.48904	23.60665	15.47227	25.99293
AC021087.	63.4199	61.02382	101.9116	47.80747	87.49131	46.81737	71.37314	80.56435	89.88116	78.77734	104.8137	80.96688	54.48847	120.0629	111.4927	42.04704	30.79129	65.52961	42.12647
AC021087.	35.55358	26.01015	50.95582	59.23969	69.77155	15.98642	75.62154	42.59724	7.003726	32.66377	0	69.92595	55.51656	25.12945	44.01026	60.18498	32.84404	50.96747	43.02278
AC021087.	135.488	222.0867	322.7202	227.6051	149.5105	147.3034	376.4083	222.2465	151.7474	143.1442	124.0898	361.5907	410.2057	171.2525	168.217	216.0064	209.3808	288.5123	288.6112
AC021087.	0	1.00039	0	0	0	0	0.84968	0.926027	0	0	12.04755	0	0	0	0	0	0	0	0
AC021088.	1.921815	0	6.993936	4.157171	0	0	5.098081	5.556162	0	6.724895	0	0	2.056169	0	4.890029	0	0	0	0
AC021092.	115.3089	170.0664	90.92116	101.8507	60.91167	101.6279	95.16419	85.19449	74.70641	79.73804	63.85201	70.84602	68.88165	95.86419	117.3607	71.72731	92.37386	85.55254	64.53417
AC021106.	178.7288	117.0457	124.8917	219.2908	161.6928	172.4249	135.9488	182.4273	19.84389	113.3625	83.12809	184.9357	140.8476	141.4695	145.7229	171.486	86.21561	183.847	131.7573
AC021106.	3.84363	4.001562	0.999134	17.66798	0	3.425661	0.84968	0	0	0.960699	0	1.840156	3.084253	7.445762	5.868035	4.122259	4.105505	5.4608	1.792616
AC021106.	30.74904	36.01406	35.96881	81.06484	28.79461	59.37812	57.77826	75.00819	18.6766	18.25329	28.91412	80.04681	74.02207	26.99089	55.74633	61.00944	29.76491	41.86614	51.08955
AC021127.	221.0087	140.0547	152.8675	729.5835	132.8982	540.1126	322.8785	456.5313	33.85134	54.75986	49.39495	193.2164	166.5497	191.7284	153.5469	887.1102	434.1572	94.65388	277.8555
AC021146.	3.84363	0	0	0	0	0	0	0	0	0	0	0	1.028084	0	0	0	0	0	0
AC021146.	0	0	0	0	0	0	0	0	0	0	0	0	0	0	0	0	2.052753	0	0
AC021146.	0	0	0.999134	8.314342	14.3973	3.425661	0	0	0	0	9.638039	5.520469	4.112337	0	0	0	0	0	0
AC021146.	0	0	0	0	3.322455	0	0	0	0	0	0	0	0	0	0	0	0	0	0
AC021146.	0	0	0	0	1.107485	0	0	0	0	0	0	0	1.028084	0	0	0	0	0	0
AC021146.	0	0	0	0	3.322455	0	0	0	0	0	0	0	0	0	0	0	0	0	0

AC021148.	56.69354	106.0414	82.92809	80.02554	64.23413	98.20228	67.97442	90.75065	116.7288	134.4979	173.4847	43.24368	50.37613	86.55699	67.4824	54.41382	78.0046	44.59654	51.98586
AC021151.	0.960908	5.001952	4.995668	2.078586	0	0	1.69936	7.408216	4.669151	0.960699	3.614265	0.920078	2.056169	1.861441	1.956012	3.297807	0	4.550667	5.377848
AC021171.	0	0	13.98787	6.235757	3.322455	0	16.14392	19.44657	4.669151	6.724895	0	6.440548	5.140422	0	0.978006	6.595615	0	0	1.792616
AC021171.	0	0	2.997401	0	0	0	0.84968	0	0	0	0	0.920078	0	0	0.978006	0.824452	0	0	7.170464
AC021188.	50.9281	24.00937	20.98181	16.62868	26.57964	36.54038	10.19616	26.85478	32.68406	13.44979	26.50461	19.32164	29.81445	23.26801	32.27419	14.84013	32.84404	32.7648	41.23017
AC021205.	0	0	2.997401	1.039293	3.322455	0	0.84968	0	0	0	0	0	0	3.722881	0	3.297807	0	0	0
AC021205.	0	0	0	0	2.21497	0	0	0	0	0	1.204755	0	0	0	0	0	0	0	0
AC021205.	253.6796	268.1046	326.7167	202.6621	400.9095	229.5193	210.7207	338.9259	419.0563	270.9172	331.3076	196.8967	262.1615	250.3638	254.2815	182.2039	172.4312	182.0267	131.7573
AC021218.	13.45271	2.000781	13.98787	6.235757	1.107485	18.27019	5.947762	0	14.00745	5.764196	1.204755	4.600391	11.30893	18.61441	14.67009	4.946711	1.026376	38.2256	15.23724
AC021224.	6.726353	5.001952	10.99047	4.157171	5.537425	1.141887	9.346483	11.11232	0	13.44979	6.023774	5.520469	7.196591	6.515042	3.912023	2.473355	0	2.7304	2.688924
AC021231.	20.17906	26.01015	17.98441	20.78586	11.07485	5.709435	16.14392	12.96438	3.501863	27.86028	7.228529	28.52243	18.50552	4.653602	7.824047	12.36678	5.131881	18.20267	2.688924
AC021231.	11.53089	16.00625	8.992203	16.62868	21.04221	6.851322	26.34009	7.408216	8.171014	10.56769	9.638039	16.56141	15.42127	8.376483	31.29619	18.96239	21.5539	2.7304	14.34093
AC021231.	14.41361	38.01484	35.96881	12.47151	13.28982	10.27698	8.496802	18.52054	57.1971	12.48909	20.48083	18.40156	18.50552	19.54513	18.58211	18.13794	27.71216	10.9216	8.066772
AC021237.	0.960908	0	0	0	0	0	0	0	0	0	0	0.920078	0	0	0	0	1.026376	0	0
AC021242.	0	1.00039	5.994802	0	0	0	0.84968	0	0	0.960699	0	6.440548	1.028084	0	0	0	0	0	1.792616
AC021242.	20.17906	43.01679	68.94022	39.49313	62.01916	52.5268	46.73241	42.59724	64.20083	64.36685	49.39495	40.48344	23.64594	36.29809	34.2302	33.80253	40.02867	74.63094	37.64493
AC021305.	5.765445	7.002733	23.97921	5.196464	3.322455	5.709435	3.398721	0	14.00745	6.724895	7.228529	10.12086	7.196591	3.722881	0	9.06897	8.21101	9.101334	0
AC021321.	139.3316	146.057	209.8181	118.4794	93.02873	140.4521	203.9233	149.0904	98.05217	185.415	145.7753	157.3334	119.2578	243.8487	185.8211	184.6772	145.7454	137.4301	172.0911
AC021351.	4.804538	0	2.997401	1.039293	0	1.141887	0.84968	0.926027	0	0	4.81902	0	4.112337	0	0.978006	0	0	1.820267	0.896308
AC021382.	0	0	3.996535	2.078586	2.21497	0	5.947762	0	0	8.646293	1.204755	0	4.112337	0.93072	0.978006	7.420066	1.026376	4.550667	8.066772
AC021422.	1.921815	3.001171	0.999134	0	0	2.283774	1.69936	2.778081	14.00745	2.882098	0	8.280704	5.140422	2.792161	0.978006	0.824452	2.052753	6.370934	6.274156
AC021439.	73.98988	103.0402	50.95582	112.2436	81.95388	110.763	98.56291	33.33697	19.84389	16.33189	33.73314	80.96688	75.05016	120.9936	90.95454	106.3543	117.0069	98.29441	94.11233
AC021443.	3.84363	4.001562	0.999134	3.117878	3.322455	0	4.248401	1.852054	0	4.803496	0	1.840156	8.224675	0.93072	9.780058	0.824452	0	0.910133	3.585232
AC021451.	0	0	0	4.157171	0	0	0	0	0	0	0	0	0	0	0	0	1.026376	0	0
AC021483.	25.9445	11.00429	9.991337	23.90373	8.859879	6.851322	11.04584	10.1863	11.67288	4.803496	3.614265	33.12282	18.50552	15.82225	22.49413	24.73355	14.36927	6.370934	10.7557
AC021483.	16.33543	1.00039	7.993069	13.51081	12.18233	17.12831	9.346483	4.630135	8.171014	4.803496	0	14.72125	3.084253	14.89152	5.868035	4.946711	15.39564	5.4608	7.170464
AC021491.	14.41361	30.01171	2.997401	12.47151	22.1497	27.40529	18.69297	27.78081	37.35321	25.93888	14.45706	21.1618	22.61786	22.33729	8.802052	17.31349	25.65941	16.3824	21.51139
AC021491.	44.20175	58.02265	44.96101	35.33595	38.76197	51.38492	30.58849	54.6356	224.1192	57.64196	42.16642	81.88696	70.93782	78.18051	66.5044	16.48904	27.71216	54.608	48.40063
AC021491.	1.921815	11.00429	5.994802	11.43222	8.859879	30.83095	4.248401	16.66849	12.84017	25.93888	15.66181	11.04094	3.084253	9.307203	21.51613	11.54233	2.052753	14.56213	9.859387
AC021517.	15.37452	16.00625	28.97488	8.314342	2.21497	11.41887	12.7452	16.66849	7.003726	2.882098	16.86657	5.520469	4.112337	22.33729	6.846041	4.122259	7.184634	6.370934	6.274156
AC021534.	0	0	0	1.039293	0	0	0.84968	0	0	1.921399	0	0	3.084253	0	0	3.297807	0	0	0
AC021546.	0	0	0	0	0	2.283774	0	0	0	0	0	0	0	0	0	0	0	0	0
AC021549.	0	1.00039	0	0	0	0	3.398721	0	0	0	0	0	0	0	4.890029	0	0	0	3.585232
AC021594.	5.765445	4.001562	7.993069	11.43222	3.322455	7.993209	5.947762	1.852054	0	12.48909	0	11.04094	16.44935	6.515042	6.846041	14.84013	14.36927	13.652	17.02985
AC021594.	0.960908	0	3.996535	13.51081	6.64491	3.425661	1.69936	10.1863	1.167288	5.764196	0	17.48149	0	5.584322	2.934017	9.06897	9.237386	27.304	9.859387
AC021660.	0	0	0	0	0	0	0	0	0	0	0	0	0	0	0	0	0	5.4608	0
AC021660.	0	0	1.998267	1.039293	3.322455	0	0	0	2.334575	0.960699	0	0	0	0	2.934017	0	0	5.4608	0
AC021678.	0	12.00469	3.996535	5.196464	5.537425	6.851322	4.248401	0	0	0	0	0.920078	6.168506	7.445762	1.956012	1.648904	2.052753	3.640534	2.688924
AC021678.	0	5.001952	0	0	0	0	0.84968	0	0	0	0	0.920078	0	0	0	0	0	0	1.792616
AC021683.	56.69354	95.03709	65.94282	186.0334	124.0383	179.2763	108.7591	105.5671	145.911	112.4018	118.066	80.04681	76.07824	121.9244	138.8768	144.2791	147.7982	13.652	73.49725
AC021683.	108.5826	105.041	116.8986	295.1591	160.5853	142.7359	166.5373	130.5698	178.595	144.1049	175.8942	151.8129	193.2799	176.8369	131.0528	172.3104	197.0642	21.8432	120.1053
AC021683.	218.126	190.0742	179.8441	289.9627	126.2533	327.7216	245.5576	211.1342	325.6733	227.6857	196.375	240.1404	217.9539	241.9873	288.5117	269.5957	454.6847	71.90054	124.5868
AC021683.	4.804538	0	3.996535	7.275049	0	7.993209	0	0	14.00745	0	4.81902	0.920078	7.196591	0.93072	2.934017	4.122259	16.42202	5.4608	5.377848
AC021698.	0	2.000781	0	0	0	0	0	0	2.334575	3.842797	15.66181	0	0	0	0.978006	0	1.026376	0	0
AC021713.	0	0	0	0	0	0	2.549041	0	0	0	0	0	0	0	0	0	0	0	0
AC021723.	4.804538	0	9.991337	10.39293	5.537425	0	17.84329	2.778081	7.003726	24.01748	14.45706	0.920078	8.224675	14.89152	24.45015	1.648904	39.0023	5.4608	8.963079
AC021733.	5.765445	0	10.99047	2.078586	1.107485	10.27698	8.496802	11.11232	0	3.842797	0	2.760235	0	4.653602	7.824047	1.648904	7.184634	9.101334	8.066772
AC021733.	1.921815	7.002733	1.998267	0	0	0	1.69936	0.926027	0	0.960699	3.614265	2.760235	5.140422	1.861441	0	0.824452	1.026376	1.820267	0
AC021739.	1.921815	6.002343	6.993936	13.51081	6.64491	12.56076	1.69936	9.26027	15.17474	8.646293	16.86657	5.520469	5.140422	0.93072	3.912023	2.473355	5.131881	2.7304	17.02985
AC021739.	242.1487	584.228	456.6041	749.3301	965.7269	456.7548	337.3231	632.4765	880.1349	710.9174	981.8752	364.351	440.0201	274.5625	274.8196	267.9468	333.5723	253.0171	311.9152
AC021739.	100.8953	146.057	139.8787	206.8193	323.3856	77.64832	124.903	124.0876	200.7735	377.5548	403.5929	135.2515	147.0161	96.79491	119.3167	79.97183	94.42662	162.0037	128.172
AC021739.	0.960908	16.00625	13.98787	6.235757	24.36467	9.135096	11.04584	12.03835	0	10.56769	6.023774	11.96102	21.58977	22.33729	12.71408	9.893422	7.184634	8.191201	12.54831
AC021739.	64.3808	252.0984	128.8882	234.8802	327.8155	213.5329	101.1119	240.767	371.1975	367.9478	563.8253	139.8519	166.5497	107.0328	96.82258	118.7211	133.4289	144.7112	188.2247
AC021744.	0.960908	5.001952	1.998267	0	2.21497	0	2.549041	0	2.334575	1.921399	0	2.760235	3.084253	0.93072	1.956012	3.297807	0	2.7304	1.792616
AC021752.	132.6052	54.02108	101.9116	36.37525	107.426	84.49964	83.26866	75.93422	84.04472	69.17035	97.58515	132.4913	121.314	70.73474	83.1305	38.74924	54.39794	54.608	79.77141
AC021755.	0	0	3.996535	0	0	0	0	0	0	0	0	0	0	0	0	0	0	0	0
AC021755.	54.77173	14.00547	19.98267	11.43222	43.19191	9.135096	15.29424	6.482189	11.67288	1.921399	0	15.64133	4.112337	18.61441	24.45015	20.6113	14.36927	27.304	20.61508
AC021755.	24.9836	5.001952	8.992203	2.078586	5.537425	5.709435	0	8.334243	5.836439	9.606993	14.45706	6.440548	10.28084	6.515042	9.780058	19.78684	11.29014	8.191201	10.7557
AC021766.	0	0	7.993069	0	0	0	4.248401	10.1863	0	0	0	2.760235	3.084253	8.376483	5.868035	1.648904	0	3.640534	0
AC021818.	0	0	0	0	0	0	0.84968	0	1.167288	0	0	1.840156	0	0	0	0	0	0.910133	0
AC021820.	0	4.001562	0.999134	0	6.64491	1.141887	2.549041	0	0	0	0	0	1.028084	0.93072	0	0	1.026376	0.910133	0
AC021851.	79.75532	96.03748	65.94282	64.43615	50.94431	97.0604	57.77826	125.0136	133.0708	93.18783	79.51382	75.44642	78.13441	94.93337	101.7126	65.95615	97.50574	97.38428	51.08955

AC021860.	0	1.00039	1.998267	0	5.537425	0	6.797442	10.1863	0	1.921399	0	0.920078	0	2.792161	0	0.824452	0	1.820267	0
AC021915.	0	0	0	0	0	0	0	0	0	0	0	0	0	0	0.978006	0	0	0	3.585232
AC021945.	91.28622	151.059	127.8891	129.9116	158.3703	29.68906	86.66738	114.8274	87.54658	126.8123	102.4042	55.20469	77.10633	144.2616	207.3372	126.1411	102.6376	145.6213	136.2388
AC021979.	0.960908	4.001562	0	2.078586	0	0	2.549041	0	0	0	0	0	0	0	0	0	0	0	0.896308
AC022001.	0.960908	0	0	1.039293	4.42994	0	0.84968	1.852054	0	3.842797	0	0.920078	2.056169	0	0	0	0	1.820267	0
AC022001.	15.37452	5.001952	1.998267	5.196464	5.537425	3.425661	11.89552	9.26027	0	2.882098	0	10.12086	1.028084	2.792161	10.75806	7.420066	9.237386	5.4608	3.585232
AC022001.	3.84363	4.001562	6.993936	32.21808	17.71976	3.425661	21.24201	7.408216	38.5205	8.646293	4.81902	6.440548	14.39318	20.47585	11.73607	2.473355	2.052753	15.47227	12.54831
AC022007.	428.5648	354.1382	384.6665	303.4735	449.6389	512.7073	244.7079	384.3012	790.2538	599.4763	715.6244	443.4777	470.8626	414.1705	363.8182	361.1099	457.7638	494.2024	446.3614
AC022017.	0	6.002343	0	2.078586	0	7.993209	0	0	0	0	0	0	0	0	0	0	0	0	0.896308
AC022018.	0	4.001562	2.997401	2.078586	0	0	7.647122	0.926027	11.67288	0	0	6.440548	4.112337	0	3.912023	4.122259	1.026376	0.910133	0
AC022021.	0	0	0.999134	0	4.42994	0	5.098081	0	0	5.764196	4.81902	0	0	3.722881	0	0	1.026376	0.910133	5.377848
AC022022.	0	0	0	0	0	0	0	1.852054	0	0	0	0.920078	0	0	0	0.824452	0	0	0.896308
AC022022.	0	5.001952	0	0	0	0	0	0	0	0	0	0	1.028084	0.93072	3.912023	0	0	0.910133	0
AC022028.	0	0	0	0	0	0	0	0	0	0	9.638039	0	0	0	0	0	0	0	0
AC022028.	0	4.001562	0	0	6.64491	0	6.797442	6.482189	1.167288	0.960699	0	9.200782	9.252759	11.16864	1.956012	1.648904	1.026376	10.9216	6.274156
AC022031.	0	0	11.9896	1.039293	0	0	0	0	0	0	0	0.920078	0	0	0	0	0	3.640534	0
AC022031.	0	0	1.998267	0	7.752394	0	0.84968	0	0	0	0	1.840156	0	0	0.978006	0	0	0.910133	1.792616
AC022034.	8.648168	1.00039	9.991337	0	0	1.141887	0	0	0	0	0	0	0	0	0	0	0	0	0
AC022034.	2685.737	2307.901	3438.019	3174	2711.123	1703.695	2597.472	3687.44	748.2314	881.9219	903.5662	1800.593	1983.175	2699.089	2665.066	1848.421	1693.521	2147.005	2351.016
AC022034.	0.960908	1.00039	0	0	0	0	0	0	9.338302	0	0	0	0	0	0	0	0	0	0
AC022035.	0.960908	0	2.997401	1.039293	3.322455	2.283774	1.69936	1.852054	0	2.882098	1.204755	0	0	2.792161	1.956012	0.824452	0	0.910133	0.896308
AC022039.	0	0	0	0	0	0	0	0	0	0	0	0	0	0	0	0	0	0.910133	0.896308
AC022069.	0	0	0	0	0	0	0	0	0	0	0	0	0	0.93072	0	6.595615	0	0	0
AC022075.	452.5874	157.0613	264.7704	146.5403	209.3147	186.1276	262.5512	291.6985	276.6472	144.1049	151.7991	383.6726	369.0823	307.1377	431.3006	216.8308	156.0092	238.455	179.2616
AC022075.	6.726353	1.00039	10.99047	10.39293	6.64491	0	1.69936	0	1.167288	3.842797	7.228529	3.680313	1.028084	3.722881	1.956012	0.824452	10.26376	4.550667	7.170464
AC022075.	3.84363	6.002343	0	0	0	0	2.549041	0	0	2.882098	3.614265	0	6.168506	1.861441	2.934017	9.06897	8.21101	12.74187	15.23724
AC022079.	0.960908	12.00469	10.99047	0	7.752394	5.709435	0	6.482189	4.669151	5.764196	0	7.360626	3.084253	0.93072	1.956012	10.71787	11.29014	9.101334	2.688924
AC022079.	33.63176	34.01327	81.92896	32.21808	21.04221	25.12151	45.03305	25.92876	32.68406	48.99566	37.3474	61.64524	35.98295	57.70466	79.21847	37.92478	28.73854	12.74187	20.61508
AC022080.	1.921815	3.001171	0	1.039293	13.28982	5.709435	5.098081	0	0	0.960699	1.204755	3.680313	7.196591	9.307203	3.912023	0	2.052753	4.550667	3.585232
AC022080.	0	2.000781	0	0	0	2.283774	1.69936	0	1.167288	0.960699	0	0	0	0	3.912023	0	0	0.910133	0.896308
AC022080.	0	0	0	0	0	0	1.69936	0	0	0	0	3.680313	5.140422	0	4.890029	0	0	0	0.896308
AC022081.	0	0	3.996535	0	0	0	0	0	18.6766	0	0	0	0	0	0	0	0	0	0
AC022092.	6.726353	0	5.994802	4.157171	0	1.141887	0	9.26027	14.00745	9.606993	0	1.840156	0	0	0	0	0	0	0
AC022098.	1653.722	1200.469	1472.723	831.4342	1469.632	1270.92	975.4329	1353.852	1377.4	1417.992	1901.103	1150.098	1239.87	1244.373	1131.553	1015.725	1059.22	1074.868	1058.54
AC022098.	24.02269	11.00429	7.993069	13.51081	6.64491	12.56076	6.797442	11.11232	67.70269	3.842797	13.2523	2.760235	6.168506	21.40657	21.51613	32.15362	16.42202	2.7304	3.585232
AC022098.	9.609075	5.001952	2.997401	0	0	2.283774	13.59488	5.556162	17.50932	16.33189	6.023774	7.360626	5.140422	21.40657	7.824047	0.824452	8.21101	4.550667	10.7557
AC022098.	0	0	0	8.314342	1.107485	1.141887	0	9.26027	0	0	0	11.04094	0	0.93072	0	1.648904	4.105505	0.910133	0.896308
AC022106.	0	5.001952	0	0	5.537425	1.141887	0.84968	0	0	0	0	0.920078	2.056169	5.584322	4.890029	10.71787	1.026376	0.910133	0
AC022106.	0	0	0	4.157171	0	0	5.098081	0.926027	0	0	0	0	0	0	1.956012	0.824452	0	0	3.585232
AC022107.	1023.367	872.3405	1076.067	1060.079	571.4622	616.619	1115.63	845.4627	529.9486	609.0833	483.1067	812.4291	853.31	792.043	885.0953	1087.452	1031.508	827.3113	830.8775
AC022113.	50.9281	53.02069	74.93502	33.25737	40.97694	20.55397	60.3273	69.45203	40.85507	27.86028	67.46627	67.16571	62.71315	78.18051	52.81231	55.23827	44.13418	50.96747	46.60801
AC022113.	19.21815	20.00781	30.97314	15.58939	16.61227	11.41887	29.73881	24.0767	53.69524	39.38867	40.96167	11.04094	16.44935	26.06017	23.47214	34.62698	30.79129	60.06881	39.43755
AC022118.	0	0	0	0	0	0	5.947762	0	1.167288	0	0	0	0	0	0	0.824452	1.026376	3.640534	0
AC022121.	5.765445	6.002343	10.99047	1.039293	6.64491	2.283774	6.797442	3.704108	15.17474	5.764196	10.84279	5.520469	8.224675	9.307203	0.978006	7.420066	5.131881	0	8.066772
AC022126.	15.37452	10.0039	5.994802	19.74656	3.322455	3.425661	27.18977	3.704108	15.17474	2.882098	0	8.280704	9.252759	2.792161	3.912023	13.19123	16.42202	10.9216	21.51139
AC022140.	0	0	0	0	0	0	0	0	0	0	0	0.920078	0	0	0	0	0	0	2.688924
AC022144.	16.33543	18.00703	34.96968	22.86444	64.23413	19.41208	30.58849	20.37259	31.51677	67.24895	51.80446	35.88305	35.98295	26.06017	12.71408	14.84013	12.31652	25.48374	16.13354
AC022146.	3.84363	24.00937	9.991337	11.43222	32.11706	30.83095	5.947762	25.92876	66.5354	24.01748	27.70936	0.920078	8.224675	9.307203	7.824047	10.71787	14.36927	1.820267	13.44462
AC022148.	39.39721	87.03397	55.95149	124.7151	81.95388	111.9049	91.76547	86.12051	102.7213	89.34503	59.03299	65.32555	78.13441	47.46674	49.8783	112.9499	125.2179	82.82214	102.1791
AC022148.	3.84363	2.000781	10.99047	3.117878	1.107485	0	3.398721	12.96438	3.501863	5.764196	3.614265	7.360626	9.252759	2.792161	2.934017	7.420066	5.131881	3.640534	2.688924
AC022149.	305.5686	341.1331	354.6925	383.499	227.0344	268.3434	370.4606	293.5506	119.0633	183.4936	202.3988	243.8207	255.993	358.3273	331.544	313.2917	279.1743	359.5027	241.1068
AC022150.	336.3176	373.1456	379.6708	248.391	363.2551	398.5186	333.9243	432.4546	379.3685	400.6116	426.4832	411.275	442.0763	306.207	375.5542	374.3011	368.4691	367.6939	304.7447
AC022150.	6.726353	13.00508	12.98874	24.94303	18.82724	17.12831	28.88913	21.29862	5.836439	6.724895	2.40951	15.64133	31.87062	13.9608	20.53812	28.03136	20.52753	15.47227	20.61508
AC022150.	395.8939	512.1999	894.2246	593.4362	763.0571	292.3231	823.3402	744.5257	172.7586	231.5285	266.2508	871.3141	857.4224	581.7002	721.7683	725.5176	632.2478	689.8811	629.2082
AC022154.	71.10716	61.02382	110.9038	96.65423	63.12664	50.24303	62.02666	90.75065	38.5205	78.77734	48.1902	59.80509	70.93782	75.38834	92.91055	81.62073	76.97822	65.52961	72.60094
AC022164.	14.41361	15.00586	5.994802	10.39293	5.537425	17.12831	18.69297	9.26027	4.669151	4.803496	1.204755	27.60235	10.28084	12.09936	17.6041	42.8715	25.65941	11.83173	8.066772
AC022165.	9.609075	14.00547	10.99047	14.5501	19.93473	4.567548	15.29424	15.74246	4.669151	6.724895	1.204755	20.24172	12.33701	7.445762	6.846041	15.66458	5.131881	12.74187	5.377848
AC022166.	0	0	0	1.039293	0	0	1.69936	0	0	0	0	0	0	3.722881	0	2.473355	0	0.910133	0
AC022167.	571.74	520.203	372.6769	554.9823	407.5545	491.0114	379.8071	371.3368	561.4654	433.2754	480.6972	597.1308	548.9971	383.4568	406.8504	1039.634	1022.271	451.4262	638.1713
AC022167.	4.804538	10.0039	0	0	0	1.141887	0	0	4.669151	1.921399	0	2.760235	0	0.93072	11.73607	4.946711	0	2.7304	7.170464
AC022167.	0.960908	0	0	2.078586	5.537425	1.141887	0	0	3.501863	4.803496	0	0	1.028084	0	1.956012	4.122259	12.31652	0.910133	0.896308
AC022167.	0.960908	19.00742	4.995668	9.353635	0	7.993209	4.248401	5.556162	5.836439	13.44979	18.07132	0.920078	8.224675	3.722881	6.846041	5.771163	9.237386	1.820267	0.896308

AC022173.	0	0	0	5.196464	5.537425	0	0.84968	0	0	6.724895	1.204755	0.920078	3.084253	6.515042	9.780058	1.648904	0	0.910133	0.896308
AC022182.	21.13997	31.0121	11.9896	39.49313	13.28982	23.97963	24.64073	29.63287	12.84017	23.05678	34.93789	32.20274	16.44935	20.47585	8.802052	26.38246	32.84404	11.83173	29.57816
AC022182.	53.81082	114.0445	117.8978	195.387	56.48173	114.1887	75.62154	121.3095	128.4017	66.28825	55.41872	145.3724	95.61185	63.28898	82.15249	108.8276	100.5849	106.4856	101.2828
AC022182.	1.921815	5.001952	0.999134	7.275049	8.859879	5.709435	6.797442	9.26027	8.171014	0.960699	3.614265	9.200782	12.33701	7.445762	5.868035	2.473355	6.158258	22.75334	11.652
AC022188.	17.29634	6.002343	0.999134	1.039293	6.64491	10.27698	16.9936	14.81643	4.669151	0.960699	8.433284	4.600391	6.168506	0.93072	0.978006	12.36678	8.21101	2.7304	17.02985
AC022196.	0	0	0	0	0	0	0	0	0	0	0	0	0	1.861441	0	0	1.026376	0.910133	0
AC022206.	1.921815	0	0	0	0	0	0	0	0	0	0	0	0	0	0	0.824452	0	0	0
AC022210.	0	0	1.998267	3.117878	3.322455	2.283774	0.84968	5.556162	0	0	6.023774	3.680313	3.084253	0.93072	0.978006	4.946711	4.105505	3.640534	0
AC022211.	49.96719	19.00742	25.97748	49.88605	55.37425	18.27019	51.83049	39.81916	23.34575	27.86028	8.433284	51.52438	34.95487	37.22881	31.29619	87.39189	49.26606	99.20454	70.80833
AC022211.	72.06806	93.03631	93.91856	46.76817	129.5757	69.65511	51.83049	101.863	131.9035	81.65944	137.3421	127.8909	98.6961	107.9636	98.77859	78.32292	106.7431	136.52	148.7871
AC022211.	65.34171	44.01718	29.97401	138.2259	49.83682	54.81058	57.77826	61.11778	29.18219	51.87776	43.37118	61.64524	81.21867	52.12034	57.70234	115.4233	92.37386	113.7667	84.25295
AC022211.	34.59267	14.00547	24.97834	27.02161	2.21497	29.68906	14.44456	19.44657	56.02981	11.52839	7.228529	7.360626	16.44935	21.40657	12.71408	16.48904	29.76491	14.56213	11.652
AC022215.	0.960908	0	7.993069	0	1.107485	4.567548	7.647122	8.334243	0	6.724895	0	4.600391	0	0	3.912023	1.648904	0	0.910133	0.896308
AC022217.	0	0	0	1.039293	0	0	0	0	0	0	1.204755	0	0	1.861441	5.868035	0	0	0.910133	0
AC022217.	0	0	0	0	0	0	0	0	9.338302	0	0	0	0	0.93072	0	0	0	0	0
AC022217.	10.56998	6.002343	1.998267	14.5501	11.07485	3.425661	6.797442	0	3.501863	8.646293	2.40951	6.440548	2.056169	2.792161	7.824047	0.824452	13.34289	0.910133	10.7557
AC022239.	12.4918	7.002733	8.992203	30.13949	4.42994	23.97963	5.098081	12.96438	30.34948	14.41049	8.433284	22.08188	4.112337	5.584322	1.956012	14.01568	3.079129	2.7304	4.48154
AC022239.	24.02269	17.00664	11.9896	15.58939	0	19.41208	10.19616	11.11232	10.50559	22.09608	0	5.520469	4.112337	15.82225	4.890029	14.01568	6.158258	6.370934	19.71877
AC022239.	7.68726	18.00703	14.987	19.74656	7.752394	10.27698	14.44456	19.44657	0	11.52839	10.84279	10.12086	13.3651	3.722881	10.75806	16.48904	19.50115	16.3824	24.20031
AC022239.	2.882723	17.00664	5.994802	28.0609	19.93473	30.83095	15.29424	25.92876	29.18219	28.82098	18.07132	14.72125	5.140422	6.515042	12.71408	19.78684	21.5539	15.47227	1.792616
AC022240.	0.960908	0	0	0	0	0	2.549041	0	0	0	0	0	0	0	0	0	0	0.910133	0
AC022254.	0	0	0	0	0	0	0	0	0	0	0	0	0	0	0	0	4.105505	1.820267	0
AC022272.	24.9836	18.00703	41.96361	22.86444	17.71976	15.98642	51.83049	46.30135	4.669151	25.93888	19.27608	32.20274	33.92678	15.82225	39.12023	30.50472	20.52753	40.04587	45.71171
AC022274.	9.609075	4.001562	3.996535	4.157171	27.68712	6.851322	3.398721	1.852054	1.167288	11.52839	9.638039	10.12086	0	0	7.824047	0	6.158258	0	5.377848
AC022294.	82.63805	46.01796	92.91943	65.47544	183.8425	14.84453	150.3934	65.74792	151.7474	92.22713	89.15186	31.28266	60.65698	40.02097	64.54838	14.84013	24.63303	26.39387	50.19325
AC022296.	0	0	0	0	0	0	4.248401	0	0	0	0	0	0	0	0.978006	0	1.026376	0	0
AC022296.	0.960908	0	0.999134	8.314342	0	1.141887	0	0.926027	0	0	1.204755	0	8.224675	0.93072	0	0.824452	7.184634	7.281067	0
AC022296.	8.648168	11.00429	15.98614	1.039293	11.07485	5.709435	2.549041	6.482189	21.01118	3.842797	10.84279	7.360626	9.252759	15.82225	13.69208	8.244518	4.105505	6.370934	6.274156
AC022306.	18.25724	37.01445	52.95408	11.43222	17.71976	37.68227	29.73881	20.37259	4.669151	36.50657	37.3474	17.48149	16.44935	26.06017	58.68035	18.96239	28.73854	19.1128	17.02985
AC022306.	338.2395	401.1566	483.5807	381.4204	466.2512	371.1133	353.467	410.23	727.2203	625.4152	773.4526	370.7915	410.2057	443.0229	385.3343	225.0753	279.1743	294.8832	326.2561
AC022306.	49.96719	120.0469	63.94455	47.80747	89.70628	83.35775	35.68657	76.86024	50.19337	118.166	59.03299	53.36454	65.7974	34.43665	47.92229	39.57369	32.84404	32.7648	34.0597
AC022335.	0	0	0	0	0	0	0	2.778081	0	0	0	0	0	0	0	0	0	0	0.896308
AC022336.	0	15.00586	9.991337	4.157171	0	9.135096	0.84968	10.1863	4.669151	3.842797	7.228529	8.280704	6.168506	1.861441	4.890029	7.420066	0	11.83173	4.48154
AC022336.	24.02269	12.00469	10.99047	11.43222	6.64491	11.41887	1.69936	5.556162	8.171014	7.685594	25.29985	23.92203	11.30893	16.75297	12.71408	17.31349	1.026376	10.01147	6.274156
AC022360.	0	0	0	0	0	0	0	0	0	0	0	1.840156	0	0	0.978006	0.824452	0	0	0
AC022360.	0.960908	0	3.996535	1.039293	0	0	0.84968	0	3.501863	0	0	0.920078	0	0.93072	1.956012	0	1.026376	0	6.274156
AC022364.	512.1637	592.2311	513.5547	430.2672	382.0823	419.0725	695.8881	429.6765	270.8108	415.0221	361.4265	516.1639	483.1997	467.2216	563.3314	521.878	455.7111	547.9003	567.3629
AC022364.	0	0	0.999134	0	0	0	0.84968	0	0	0	0	0	0.920078	0	0	1.956012	0	0	0.896308
AC022367.	0	0	0	0	0	0	0.84968	0	0	0	0	0	0	0	0	0	0	0.910133	0
AC022382.	5.765445	6.002343	13.98787	6.235757	4.42994	12.56076	11.89552	2.778081	9.338302	11.52839	15.66181	7.360626	8.224675	0	2.934017	4.122259	5.131881	9.101334	6.274156
AC022387.	19.21815	15.00586	4.995668	8.314342	1.107485	2.283774	11.89552	0	0	1.921399	4.81902	8.280704	10.28084	9.307203	1.956012	7.420066	16.42202	3.640534	6.274156
AC022390.	5.765445	5.001952	0.999134	9.353635	14.3973	5.709435	4.248401	0.926027	0	0.960699	2.40951	2.760235	1.028084	1.861441	3.912023	9.06897	6.158258	0	0.896308
AC022392.	1.921815	1.00039	0	0	1.107485	0	5.947762	0.926027	1.167288	0.960699	2.40951	0	3.084253	5.584322	0	0	0	0	9.859387
AC022395.	10.56998	32.01249	22.98007	36.37525	33.22455	7.993209	42.48401	13.89041	1.167288	12.48909	0	44.16376	10.28084	7.445762	8.802052	33.80253	24.63303	37.31547	64.53417
AC022398.	0.960908	0	2.997401	1.039293	0	0	2.549041	4.630135	0	4.803496	0	0.920078	1.028084	1.861441	0.978006	0.824452	4.105505	0.910133	4.48154
AC022400.	333.4349	478.1866	349.6968	442.7387	259.1515	382.5321	400.1994	387.0793	141.2418	263.2316	244.5652	405.7545	403.0091	482.1131	460.6407	408.1037	490.6078	415.931	515.3771
AC022400.	5.765445	3.001171	21.98094	6.235757	7.752394	2.283774	26.34009	18.52054	0	10.56769	7.228529	11.96102	16.44935	0	5.868035	9.893422	4.105505	4.550667	8.066772
AC022400.	0.960908	0	0	0	0	0	0	0	0	0	0	2.760235	1.028084	3.722881	1.956012	0	0	0	1.792616
AC022400.	24.9836	20.00781	21.98094	12.47151	32.11706	14.84453	11.04584	23.15068	1.167288	14.41049	14.45706	19.32164	18.50552	47.46674	6.846041	45.34485	37.97592	23.66347	34.95601
AC022400.	234.4614	166.0648	414.6405	312.8271	282.4087	205.5397	324.5779	237.0629	189.1006	217.118	156.6181	307.3061	319.7342	216.8578	243.5235	230.0221	227.8555	278.5008	205.2545
AC022400.	2.882723	8.003124	3.996535	4.157171	0	2.283774	7.647122	3.704108	0	3.842797	0	4.600391	8.224675	5.584322	4.890029	5.771163	3.079129	8.191201	4.48154
AC022400.	3248.828	2208.862	3618.862	2347.762	2621.417	1957.194	2509.955	2290.065	5361.353	3171.268	3393.794	2721.591	2955.743	3008.088	2855.777	2359.581	2330.9	3362.943	2549.996
AC022400.	179.6897	165.0644	183.8406	247.3517	183.8425	200.9721	266.7996	266.6958	328.0079	288.2098	238.5415	207.0176	182.999	171.2525	214.1833	245.6866	204.2489	224.803	227.6622
AC022400.	3.84363	2.000781	3.996535	0	8.859879	7.993209	0.84968	0	0	3.842797	3.614265	7.360626	4.112337	1.861441	0	2.473355	1.026376	1.820267	3.585232
AC022404.	0	0	0	0	0	0	0.84968	0	0	0	0	0.920078	0	0	0	0	0	1.820267	0
AC022405.	0	0	1.998267	2.078586	0	0	1.69936	3.704108	0	0	0	0	1.028084	0	0.978006	4.122259	0	9.101334	10.7557
AC022413.	41.31902	28.01093	40.96448	22.86444	37.65449	33.11472	32.28785	36.11505	50.19337	43.23147	65.05676	21.1618	38.03912	20.47585	27.38416	23.9091	31.81766	45.50667	43.02278
AC022415.	184.4942	185.0722	174.8484	198.5049	186.0575	212.391	186.08	180.5753	347.8517	248.8211	319.26	162.8538	160.3812	190.7977	198.5352	187.1506	216.5654	219.3422	202.5656
AC022417.	106.6607	144.0562	110.9038	210.9764	180.52	140.4521	77.3209	176.8712	314.0004	271.8779	267.4556	123.2905	191.2237	195.4513	167.239	134.3856	152.9301	121.9579	130.861
AC022424.	491.0237	511.1995	578.4984	380.3812	527.1628	415.6469	365.3625	438.0108	595.3167	834.8476	1439.682	457.2789	502.7333	382.526	338.39	187.1506	246.3303	236.6347	289.5075
AC022424.	0	1.00039	0.999134	0	0	0	0.84968	0	0	0.960699	0	0	0	0	0	0	0	0	0
AC022431.	49.96719	49.01913	48.95755	31.17878	40.97694	47.95925	53.52986	58.3397	25.68033	34.58517	39.75691	32.20274	41.12337	48.39746	43.03226	38.74924	64.6617	62.79921	34.0597

AC022431.	0	3.001171	6.993936	3.117878	1.107485	18.27019	8.496802	1.852054	0	21.13538	8.433284	5.520469	13.3651	4.653602	4.890029	7.420066	6.158258	12.74187	8.066772
AC022432.	14.41361	24.00937	37.96708	37.41454	40.97694	39.96605	28.03945	18.52054	29.18219	10.56769	38.55216	23.92203	53.46039	20.47585	19.56012	40.39814	43.1078	32.7648	25.09662
AC022441.	20.17906	16.00625	2.997401	10.39293	4.42994	12.56076	13.59488	0	0	18.25329	0	5.520469	2.056169	2.792161	5.868035	0.824452	4.105505	4.550667	2.688924
AC022445.	4.804538	3.001171	18.98354	0	7.752394	3.425661	16.14392	11.11232	4.669151	7.685594	0	11.96102	6.168506	13.9608	20.53812	9.06897	1.026376	15.47227	5.377848
AC022447.	0	4.001562	0	0	0	0	0	0	0	0	0	0	0	0.93072	0	0	0	0	0
AC022447.	2.882723	1.00039	6.993936	2.078586	1.107485	4.567548	2.549041	0	22.17847	6.724895	1.204755	4.600391	2.056169	4.653602	9.780058	2.473355	3.079129	1.820267	1.792616
AC022447.	0.960908	0	0	0	4.42994	0	0	0.926027	0	0	0	0	0.920078	0	0	0	0.824452	0	0
AC022447.	0	0	0	0	0	0	0	0	0	0	0	2.40951	0	0	0	0	0	0	0
AC022447.	0	0	0	0	0	0	0.84968	0	1.167288	0	0	0	0	0	0	0	0	0	0
AC022447.	0	0	0	0	0	0	0	0	0	0	0	3.680313	1.028084	0	0	0	0	0	0
AC022447.	0	0	0	0	0	0	0.84968	0	0	0	0	0	1.028084	0	0	0.824452	0	0	0
AC022467.	5.765445	27.01054	20.98181	4.157171	37.65449	18.27019	13.59488	12.03835	40.85507	23.05678	44.57593	18.40156	12.33701	7.445762	31.29619	15.66458	7.184634	25.48374	14.34093
AC022469.	2.882723	2.000781	0	1.039293	0	2.283774	0.84968	5.556162	0	1.921399	0	0	0	0	0	0	1.026376	0	0
AC022469.	0	4.001562	2.997401	1.039293	4.42994	2.283774	0	0	0	4.803496	2.40951	0.920078	7.196591	4.653602	0	0	0	4.550667	3.585232
AC022483.	0	1.00039	0	0	0	1.141887	0.84968	1.852054	0	0	0	0	0	0.93072	0	4.122259	0	0	0
AC022483.	230.6178	128.05	143.8752	182.9155	190.4874	212.391	98.56291	177.7972	93.38302	94.14853	110.8374	118.6901	96.63993	158.2225	138.8768	169.8371	84.16285	167.4645	78.8751
AC022486.	0	2.000781	0.999134	1.039293	0	0	0	0	0	1.921399	1.204755	0	0	0	0	0	0	0	0
AC022493.	0	4.001562	0	0	0	0	0	1.852054	0	0	0	0.920078	1.028084	0.93072	5.868035	2.473355	0	0	2.688924
AC022493.	3.84363	57.02225	2.997401	0	0	1.141887	0	0	0	0.960699	0	3.680313	0	0	0	3.297807	1.026376	0.910133	0.896308
AC022494.	3.84363	2.000781	0	0	0	2.283774	0	7.408216	1.167288	0	0	0	0	0	3.912023	3.297807	0	2.7304	0
AC022497.	221.9696	172.0672	209.8181	216.1729	314.5257	158.7223	268.499	237.0629	375.8666	194.0612	220.4701	228.1794	266.2739	197.3127	236.6774	247.3355	264.8051	127.4187	117.4163
AC022498.	0	18.00703	18.98354	1.039293	11.07485	9.135096	15.29424	2.778081	0	4.803496	7.228529	0	0	1.861441	3.912023	0.824452	0	0	0
AC022509.	2.882723	4.001562	0.999134	10.39293	0	0	4.248401	0	10.50559	0	0	0.920078	0	0.93072	0	0.824452	2.052753	10.01147	0
AC022509.	56.69354	69.02694	61.94629	59.23969	65.34161	59.37812	53.52986	86.12051	80.54285	89.34503	65.05676	56.12477	60.65698	40.95169	48.90029	46.99375	44.13418	42.77627	48.40063
AC022509.	237.3442	132.0515	160.8605	131.9902	116.2859	83.35775	158.0405	118.5315	33.85134	66.28825	45.78069	239.2203	202.5326	164.7375	147.6789	122.0189	186.8005	133.7896	229.4548
AC022509.	0.960908	9.003514	9.991337	0	0	1.141887	7.647122	0.926027	18.6766	4.803496	6.023774	14.72125	9.252759	7.445762	8.802052	8.244518	14.36927	2.7304	7.170464
AC022517.	1.921815	1.00039	0	0	8.859879	0	0	0	0	3.842797	0	0	0	7.445762	3.912023	0.824452	0	1.820267	0.896308
AC022523.	65.34171	27.01054	29.97401	66.51474	24.36467	59.37812	77.3209	26.85478	5.836439	30.74238	20.48083	44.16376	41.12337	38.15953	35.20821	63.48279	78.0046	54.608	59.15632
AC022532.	122.9962	69.02694	136.8813	115.3615	50.94431	84.49964	115.5565	93.52873	120.2306	68.20965	153.0039	122.3704	97.66802	65.15042	70.41642	91.51415	88.26836	132.8795	114.7274
AC022535.	0	0	0	1.039293	0	0	0	0	0	0	0	0	0	0	0	0	1.026376	0	0
AC022537.	0	0	0	4.157171	0	0	0	7.408216	0	0	0	0	2.056169	0	1.956012	1.648904	0	0	0
AC022540.	2.882723	3.001171	11.9896	5.196464	8.859879	1.141887	7.647122	23.15068	0	15.37119	7.228529	11.96102	8.224675	9.307203	4.890029	9.06897	4.105505	7.281067	5.377848
AC022558.	98.97348	102.0398	115.8995	97.69352	99.67364	77.64832	142.7463	121.3095	8.171014	43.23147	24.0951	138.0117	83.27483	104.2407	89.97654	92.3386	70.81996	119.2275	135.3425
AC022558.	6.726353	0	4.995668	0	21.04221	6.851322	1.69936	1.852054	7.003726	0.960699	0	1.840156	3.084253	5.584322	5.868035	0	0	3.640534	0
AC022558.	56.69354	99.03865	108.9056	47.80747	98.56616	107.3374	115.5565	153.7205	29.18219	136.4193	121.6802	98.44837	98.6961	120.9936	89.97654	78.32292	58.50345	78.27147	69.91202
AC022568.	0	0	0	0	0	0	0	0	0	5.764196	0	0	0	0	0	0	0	0	0.896308
AC022601.	2.882723	1.00039	2.997401	1.039293	0	0	5.098081	4.630135	0	0.960699	6.023774	4.600391	1.028084	6.515042	2.934017	0.824452	1.026376	0.910133	5.377848
AC022613.	142.2143	98.03826	42.96275	189.1513	59.80419	208.9653	126.6024	141.6821	15.17474	70.13105	69.87578	116.8499	69.90974	104.2407	122.2507	176.4327	160.1147	69.17014	91.42341
AC022613.	304.6077	237.0925	285.7522	204.7407	169.4452	111.9049	192.0277	238.915	294.1565	178.6901	202.3988	184.9357	188.1394	309.9299	308.0718	178.0816	214.5126	216.6118	175.6764
AC022613.	46.12356	57.02225	98.91423	63.39686	39.86946	27.40529	90.91579	62.96984	36.18592	52.83846	39.75691	85.56728	59.62889	87.48771	109.5367	64.30724	67.74083	72.81067	75.28987
AC022634.	0	3.001171	0	0	0	0	2.549041	6.482189	0	0	0	0	2.056169	4.653602	0.978006	0	0	1.820267	0.896308
AC022634.	0	0	0.999134	0	0	0	0	0	0	0	0	0.920078	1.028084	0	0.978006	0	0	0.910133	0
AC022639.	22.10087	14.00547	15.98614	18.70727	2.21497	12.56076	8.496802	5.556162	21.01118	9.606993	1.204755	15.64133	2.056169	13.9608	14.67009	22.2602	14.36927	11.83173	11.652
AC022662.	0	0	0	3.117878	0	0	0	0	0	0	0	0	0	0	0	0	0	0	0
AC022679.	5.765445	3.001171	1.998267	1.039293	7.752394	2.283774	0.84968	2.778081	9.338302	15.37119	0	0	1.028084	3.722881	9.780058	0.824452	0	0	2.688924
AC022679.	0	1.00039	1.998267	6.235757	8.859879	2.283774	0.84968	1.852054	0	3.842797	0	1.840156	5.140422	0	0.978006	0	2.052753	4.550667	0.896308
AC022695.	0	0	1.998267	0	0	0	0	0	0	0	0	0	0	0	0	0	0	0	0
AC022695.	0	3.001171	7.993069	1.039293	0	0	0	2.778081	5.836439	0	0	0	0	0.93072	0.978006	1.648904	0	3.640534	2.688924
AC022695.	0	0	0	0	0	0	0	0	0	0	0	0.920078	1.028084	0	0	0.824452	2.052753	0	0
AC022706.	211.3997	149.0582	199.8267	159.0118	160.5853	228.3774	107.0597	332.4437	266.1416	187.3364	315.6458	173.8948	226.1786	261.5324	319.8079	248.16	172.4312	130.1491	67.2231
AC022709.	0	0	1.998267	0	0	0	0	0	0	0	0	0	0	0	0	0	0	0	0
AC022710.	0	1.00039	4.995668	5.196464	0	0	0.84968	0	4.669151	0	7.228529	0.920078	0	0	0.978006	3.297807	6.158258	0.910133	0
AC022726.	0.960908	5.001952	3.996535	8.314342	0	0	5.947762	4.630135	0	3.842797	0	1.840156	1.028084	3.722881	0.978006	6.595615	5.131881	3.640534	0.896308
AC022726.	4.804538	3.001171	18.98354	1.039293	5.537425	2.283774	2.549041	12.03835	14.00745	7.685594	4.81902	2.760235	9.252759	3.722881	1.956012	5.771163	4.105505	0.910133	0.896308
AC022730.	0.960908	0	0	0	0	0	0	0	1.167288	0	0	0.920078	0	0	0	0	1.026376	0.910133	0
AC022730.	0	0	0	0	1.107485	0	1.69936	0	0	10.56769	10.84279	0	0	2.792161	5.868035	4.946711	0	0	0
AC022730.	0.960908	0	1.998267	0	31.00958	13.70264	30.58849	0	0	229.6071	238.5415	24.84211	10.28084	51.18962	30.31818	15.66458	4.105505	5.4608	0.896308
AC022733.	0	0	0	1.039293	0	0	0	0.926027	0	0	0	0	0	4.112337	0	0.978006	0	0	0.896308
AC022733.	0	3.001171	0.999134	1.039293	0	0	5.098081	0.926027	0	0	0	0.920078	4.112337	0.93072	0.978006	3.297807	3.079129	0	10.7557
AC022748.	0	0	0	2.078586	1.107485	0	0	0	0	0	0	0	1.028084	0	0	0	0	0	0
AC022748.	2.882723	0	0	4.157171	0	0	0	0.926027	0	0	0	0.920078	0	0	0	0	2.052753	0	0
AC022762.	0	1.00039	0.999134	0	0	0	5.098081	0.926027	0	4.803496	0	9.200782	4.112337	0	0	1.648904	1.026376	0	0

AC022762.	207.556	355.1386	387.6639	337.7702	356.6101	514.991	228.564	288.9204	409.718	658.079	579.4871	241.0605	306.3691	195.4513	230.8094	194.5706	227.8555	164.7341	138.9277
AC022778.	5.765445	2.000781	7.993069	8.314342	13.28982	2.283774	4.248401	5.556162	0	2.882098	0	6.440548	4.112337	0	0	2.473355	5.131881	3.640534	3.585232
AC022784.	22.10087	2.000781	11.9896	10.39293	6.64491	4.567548	8.496802	22.22465	18.6766	20.17468	7.228529	12.8811	8.224675	6.515042	9.780058	4.946711	2.052753	9.101334	3.585232
AC022784.	0	0	0	0	0	0	0	0	0	0	0	3.680313	0	0	0	0	0	0	0.896308
AC022784.	27.86632	27.01054	47.95842	23.90373	37.65449	23.97963	11.89552	56.48765	33.85134	43.23147	31.32363	32.20274	17.47743	20.47585	21.51613	32.15362	19.50115	27.304	29.57816
AC022784.	7.68726	1.00039	1.998267	0	13.28982	1.141887	0	0	1.167288	0	0	0	0	0	4.890029	0.824452	1.026376	0	0
AC022784.	12.4918	8.003124	42.96275	12.47151	25.47215	15.98642	19.54265	12.03835	12.84017	17.29259	45.78069	30.36258	17.47743	11.16864	9.780058	14.84013	8.21101	26.39387	17.92616
AC022784.	11.53089	6.002343	8.992203	8.314342	25.47215	1.141887	0.84968	6.482189	14.00745	0.960699	9.638039	12.8811	1.028084	4.653602	0	0.824452	1.026376	6.370934	0.896308
AC022795.	0	5.001952	2.997401	2.078586	1.107485	2.283774	3.398721	2.778081	4.669151	0.960699	3.614265	0.920078	3.084253	0	0.978006	3.297807	0	0.910133	0
AC022796.	0	0	0	1.039293	0	0	3.398721	0.926027	0	0.960699	0	0	0	0	2.934017	0	1.026376	0	2.688924
AC022809.	16.33543	19.00742	8.992203	27.02161	2.21497	15.98642	16.14392	25.00273	23.34575	16.33189	32.52838	11.96102	6.168506	25.12945	23.47214	20.6113	20.52753	22.75334	20.61508
AC022816.	0	5.001952	0	0	2.21497	0	0	0	0	0	0	0	0	0	0	4.122259	1.026376	0	0
AC022819.	70.14625	81.03163	130.8865	32.21808	102.9961	58.23624	92.61515	56.48765	89.88116	115.2839	71.08054	117.77	81.21867	57.70466	83.1305	41.22259	61.58258	33.67494	60.94894
AC022826.	8.648168	2.000781	7.993069	25.98232	5.537425	14.84453	22.09169	14.81643	0	4.803496	14.45706	11.04094	17.47743	7.445762	22.49413	11.54233	19.50115	36.40534	9.859387
AC022832.	0	1.00039	1.998267	0	17.71976	5.709435	15.29424	2.778081	23.34575	6.724895	9.638039	4.600391	11.30893	3.722881	5.868035	6.595615	0	8.191201	11.652
AC022858.	3.84363	3.001171	0.999134	25.98232	6.64491	4.567548	2.549041	2.778081	4.669151	19.21399	7.228529	24.84211	13.3651	13.03008	24.45015	15.66458	9.237386	9.101334	17.92616
AC022868.	5.765445	5.001952	14.987	2.078586	11.07485	12.56076	5.947762	30.55889	7.003726	13.44979	13.2523	20.24172	11.30893	7.445762	19.56012	13.19123	11.29014	37.31547	29.57816
AC022872.	4.804538	20.00781	5.994802	3.117878	12.18233	10.27698	2.549041	9.26027	0	8.646293	2.40951	9.200782	3.084253	3.722881	1.956012	8.244518	6.158258	6.370934	0.896308
AC022880.	0	0	0	0	0	2.283774	0	0	0	0	0	0	0	0	0	0	0	0	0
AC022884.	0	0	0.999134	5.196464	0	1.141887	0	0	0	0	0	6.440548	0	1.861441	1.956012	0	2.052753	0	0.896308
AC022893.	2249.485	1161.453	1786.451	1081.904	2370.018	1017.421	1218.441	1658.514	1872.33	1399.739	1413.177	1835.556	2039.719	1288.117	1371.164	901.9503	823.1538	1253.254	1192.986
AC022893.	5238.868	6751.635	4622.991	8881.796	3582.714	6920.977	5955.409	5098.705	2347.416	4205.941	2413.124	4177.155	4800.126	5690.424	5440.646	6239.451	6409.72	3768.862	4617.779
AC022893.	117.2307	81.03163	66.94196	39.49313	137.3281	69.65511	104.5107	43.52327	81.71014	85.50223	69.87578	86.48735	127.4825	75.38834	88.99853	35.45143	18.47477	104.6653	70.80833
AC022898.	1.921815	2.000781	7.993069	0	0	3.425661	9.346483	0.926027	16.34203	3.842797	10.84279	0.920078	0	0.93072	0.978006	0.824452	1.026376	0.910133	3.585232
AC022898.	11.53089	7.002733	13.98787	1.039293	0	7.993209	14.44456	11.11232	7.003726	20.17468	22.89034	4.600391	11.30893	0.93072	17.6041	3.297807	2.052753	2.7304	5.377848
AC022903.	0.960908	0	0	6.235757	0	0	2.549041	2.778081	0	0	0	0	0	0.93072	0	0	0	0	0.896308
AC022905.	168.1588	116.0453	59.94802	513.4106	109.641	187.2695	214.1194	130.5698	50.19337	142.1835	114.4517	170.2145	126.4544	124.7165	81.17448	470.762	342.8097	73.72081	114.7274
AC022915.	11.53089	14.00547	3.996535	3.117878	11.07485	0	5.098081	0	5.836439	11.52839	2.40951	8.280704	13.3651	3.722881	4.890029	4.946711	0	0	3.585232
AC022915.	4.804538	6.002343	0.999134	6.235757	0	0	0	12.96438	7.003726	0	0	1.840156	0	2.792161	5.868035	2.473355	6.158258	0.910133	1.792616
AC022915.	0	0	0	0	0	0	0	0	4.669151	0.960699	0	0	0	0.93072	0	0.824452	0	0	0
AC022916.	297.8813	265.1035	259.7748	229.6837	241.4317	178.1344	274.4467	214.8383	283.6509	214.2359	256.6128	296.2652	333.0993	271.7703	299.2698	195.3951	198.0906	339.4798	413.198
AC022916.	0	0	2.997401	0	0	0	0	0	4.669151	0	0	0	0	0	0	0	4.105505	0.910133	0
AC022919.	5.765445	1.00039	3.996535	3.117878	8.859879	4.567548	9.346483	0	0	0.960699	1.204755	0	1.028084	0.93072	1.956012	11.54233	2.052753	1.820267	1.792616
AC022929.	0	0	0	0	0	0	0	0	0	0	0	0	0	0	0.978006	0.824452	2.052753	0	0
AC022929.	0	9.003514	1.998267	1.039293	0	1.141887	0	0	0	0	1.204755	0	1.028084	0.93072	0.978006	0	1.026376	7.281067	0.896308
AC022960.	6.726353	22.00859	14.987	43.6503	7.752394	1.141887	45.03305	25.00273	0	3.842797	7.228529	30.36258	13.3651	10.23792	37.16422	15.66458	16.42202	30.0344	14.34093
AC022960.	24.02269	41.01601	49.95668	40.53242	6.64491	15.98642	38.23561	12.96438	18.6766	39.38867	15.66181	22.08188	48.31997	39.09025	37.16422	28.85581	36.94955	60.06881	27.78555
AC022960.	0	0	0.999134	0	0	1.141887	0	0	0	0	0	0	1.028084	0	0	0	0	0.910133	0
AC022966.	104.7389	115.0449	178.8449	49.88605	130.6832	111.9049	107.9094	81.49038	200.7735	173.8866	183.1227	117.77	99.72418	181.4905	158.4369	75.02512	106.7431	81.91201	90.5271
AC022968.	36.51449	32.01249	6.993936	23.90373	23.25718	33.11472	23.79105	19.44657	29.18219	31.70308	36.14265	22.08188	26.73019	36.29809	21.51613	27.20691	19.50115	36.40534	32.26709
AC022973.	2.882723	2.000781	0	4.157171	2.21497	0	22.09169	4.630135	0	0	0	0.920078	6.168506	6.515042	12.71408	4.122259	2.052753	17.29253	2.688924
AC022973.	8.648168	7.002733	10.99047	15.58939	14.3973	3.425661	6.797442	4.630135	10.50559	0	2.40951	18.40156	3.084253	6.515042	3.912023	5.771163	3.079129	9.101334	4.48154
AC022973.	3.84363	3.001171	0.999134	19.74656	5.537425	0	11.04584	4.630135	2.334575	0	0	3.680313	9.252759	9.307203	1.956012	11.54233	11.29014	29.12427	8.963079
AC022973.	107.6216	118.0461	214.8137	131.9902	168.3377	121.04	146.145	225.9506	17.50932	108.559	49.39495	199.657	166.5497	144.2616	171.151	101.4076	86.21561	232.084	147.8908
AC023024.	234.4614	237.0925	310.7306	331.5344	199.3473	227.2355	326.2772	212.9862	106.2232	197.904	210.8321	547.4465	513.0141	208.4813	239.6114	309.1694	339.7305	379.5256	432.9167
AC023024.	373.793	448.1749	440.6179	437.5423	199.3473	427.0657	559.9393	272.2519	149.4128	405.4151	371.0645	736.0626	664.1425	316.4449	324.6979	337.2008	429.0253	516.0456	622.934
AC023034.	47.08447	16.00625	8.992203	31.17878	8.859879	7.993209	37.38593	6.482189	16.34203	11.52839	9.638039	24.84211	22.61786	18.61441	13.69208	19.78684	41.05505	22.75334	29.57816
AC023034.	0	0	4.995668	7.275049	1.107485	0	21.24201	0	0	3.842797	4.81902	0	0	0	7.824047	5.771163	1.026376	1.820267	3.585232
AC023043.	37.47539	15.00586	29.97401	17.66798	28.79461	30.83095	16.9936	26.85478	21.01118	30.74238	56.62348	10.12086	19.5336	26.06017	17.6041	28.03136	43.1078	33.67494	32.26709
AC023043.	0	0	9.991337	5.196464	21.04221	1.141887	0	0	0	0	0	1.840156	0	0	0.978006	1.648904	0	0	0
AC023043.	3.84363	7.002733	9.991337	3.117878	4.42994	1.141887	0	0.926027	0	0	0	0.920078	8.224675	0.93072	2.934017	5.771163	4.105505	1.820267	0.896308
AC023043.	19.21815	8.003124	55.95149	33.25737	15.50479	25.12151	8.496802	16.66849	17.50932	40.34937	33.73314	6.440548	16.44935	26.06017	9.780058	17.31349	30.79129	15.47227	12.54831
AC023050.	0	1.00039	0	0	0	0	0	0	0	0	0	0	1.028084	0	0	0	0	0	0
AC023050.	0	0	0	0	0	0	0	0	0	0	0	0	0	3.722881	0	0	0	0	0
AC023050.	0.960908	0	0	0	0	0	0	0	0	0	0	1.840156	0	0	0	0	0	0	0
AC023050.	8.648168	0	10.99047	9.353635	13.28982	11.41887	5.947762	12.96438	0	0.960699	3.614265	5.520469	11.30893	4.653602	0.978006	4.122259	5.131881	4.550667	8.066772
AC023051.	17.29634	12.00469	9.991337	7.275049	13.28982	1.141887	10.19616	18.52054	22.17847	5.764196	31.32363	10.12086	20.56169	4.653602	16.6261	12.36678	8.21101	18.20267	11.652
AC023051.	0	1.00039	0	0	1.107485	0	1.69936	0	0	0	0	6.440548	0	0	1.956012	0.824452	0	0	0
AC023055.	7.68726	6.002343	16.98527	9.353635	23.25718	25.12151	6.797442	9.26027	9.338302	2.882098	4.81902	15.64133	7.196591	1.861441	5.868035	18.13794	19.50115	10.01147	8.963079
AC023078.	2.882723	1.00039	0	0	6.64491	4.567548	0.84968	0	0	0	6.023774	0	0	1.861441	7.824047	2.473355	2.052753	5.4608	3.585232
AC023078.	1.921815	0	3.996535	4.157171	0	5.709435	0.84968	1.852054	0	0	0	0	1.028084	0	0	4.946711	2.052753	0	0.896308
AC023078.	40.35812	24.00937	2.997401	14.5501	25.47215	49.10114	16.9936	8.334243	5.836439	15.37119	0	19.32164	6.168506	26.06017	9.780058	47.81821	42.08143	2.7304	16.13354

AC023078.	1.921815	4.001562	0.999134	11.43222	16.61227	18.27019	8.496802	3.704108	0	0	1.204755	0.920078	5.140422	5.584322	1.956012	9.06897	13.34289	0.910133	8.963079
AC023078.	10.56998	6.002343	12.98874	27.02161	6.64491	15.98642	16.9936	27.78081	0	6.724895	1.204755	5.520469	18.50552	5.584322	15.64809	21.43575	27.71216	0.910133	9.859387
AC023078.	0	0	3.996535	5.196464	0	1.141887	3.398721	4.630135	9.338302	0	0	0	1.028084	0	0.978006	3.297807	10.26376	0	3.585232
AC023078.	4.804538	1.00039	0.999134	13.51081	11.07485	17.12831	2.549041	10.1863	0	0	2.40951	2.760235	4.112337	1.861441	0.978006	10.71787	4.105505	0	1.792616
AC023090.	16.33543	27.01054	10.99047	6.235757	8.859879	6.851322	16.9936	9.26027	10.50559	14.41049	25.29985	11.04094	14.39318	1.861441	9.780058	1.648904	3.079129	3.640534	5.377848
AC023090.	13.45271	21.0082	24.97834	27.02161	34.33203	3.425661	33.13753	38.89314	0	8.646293	25.29985	47.84407	41.12337	3.722881	30.31818	14.01568	7.184634	20.93307	19.71877
AC023115.	15.37452	22.00859	22.98007	14.5501	12.18233	19.41208	11.89552	21.29862	1.167288	2.882098	4.81902	9.200782	21.58977	31.64449	7.824047	31.32917	32.84404	19.1128	14.34093
AC023128.	0	0	0	0	0	0	0	0	0	3.842797	0	0	0	0	0	0	0	0.910133	0
AC023128.	0	1.00039	0	0	0	0	0	0	0	0	0	0	0	0	0	0	0	0.910133	0
AC023136.	0	0	0	0	0	0	0.84968	3.704108	1.167288	0	1.204755	0.920078	4.112337	0	0	0.824452	0	0	0.896308
AC023137.	15.37452	129.0504	191.8337	41.57171	109.641	63.94567	99.41259	76.86024	108.5578	76.85594	43.37118	31.28266	40.09529	125.6472	79.21847	8.244518	32.84404	14.56213	27.78555
AC023141.	0	0	0.999134	1.039293	0	0	0	0	0	0	0	0	0	0	0	0	0	0	0
AC023141.	0	4.001562	0	0	0	0	0	0	0	0	0	0	0	0	0	0	0	0	0
AC023141.	0	0	1.998267	0	0	0	0	0	0	0	6.023774	0	0	0	0	0	0	0	0
AC023141.	0.960908	0	0	0	0	0	0	0	0	0	0	1.840156	0	0	0	0	0	0	0.896308
AC023141.	0	0	0	0	0	0	0	0	0	0	0	0	0	0	3.912023	0	0	0	0
AC023154.	14.41361	12.00469	30.97314	19.74656	17.71976	5.709435	31.43817	30.55889	25.68033	13.44979	14.45706	2.760235	18.50552	26.06017	23.47214	9.06897	2.052753	10.01147	8.963079
AC023157.	7.68726	6.002343	0.999134	2.078586	6.64491	10.27698	2.549041	3.704108	0	20.17468	44.57593	1.840156	2.056169	9.307203	4.890029	4.946711	8.21101	6.370934	4.48154
AC023157.	437.2129	507.198	563.5114	410.5206	368.7925	448.7616	432.4872	510.2409	484.4244	423.6684	540.9349	446.2379	478.0592	642.197	633.7478	391.6146	490.6078	595.2273	497.4509
AC023158.	45.16265	72.02811	55.95149	34.29666	44.2994	25.12151	23.79105	37.96711	25.68033	93.18783	81.92333	84.6472	137.7633	60.49682	71.39443	23.08465	43.1078	34.58507	19.71877
AC023158.	29.78813	14.00547	41.96361	34.29666	54.26676	21.69585	16.14392	37.04108	19.84389	27.86028	24.0951	41.40352	50.37613	42.81313	59.65836	4.946711	24.63303	32.7648	37.64493
AC023161.	0	0	1.998267	0	1.107485	0	1.69936	0	0	0	0	0	0	0	0	0	0	0	0
AC023161.	0	2.000781	13.98787	2.078586	7.752394	3.425661	5.947762	0.926027	2.334575	1.921399	0	1.840156	1.028084	7.445762	3.912023	0	0	8.191201	3.585232
AC023162.	0.960908	0	0	2.078586	0	0	0	0.926027	0	0.960699	0	0.920078	2.056169	0	0	0	0	4.550667	0
AC023200.	0	0	0	0	0	0	0	0	0	9.606993	0	0	0	0	0	0	0	0	0
AC023202.	12.4918	19.00742	19.98267	29.1002	12.18233	19.41208	38.23561	21.29862	0	13.44979	15.66181	7.360626	6.168506	12.09936	18.58211	17.31349	12.31652	24.5736	24.20031
AC023206.	3.84363	11.00429	2.997401	3.117878	3.322455	33.11472	7.647122	7.408216	5.836439	3.842797	0	4.600391	6.168506	15.82225	8.802052	4.946711	6.158258	20.02294	8.963079
AC023232.	0	0	0.999134	0	0	0	0.84968	0	0	0	2.40951	0.920078	3.084253	0.93072	0.978006	0.824452	6.158258	0.910133	0
AC023245.	10.56998	0	2.997401	16.62868	1.107485	9.135096	5.947762	11.11232	1.167288	6.724895	2.40951	1.840156	1.028084	7.445762	10.75806	20.6113	6.158258	0	3.585232
AC023271.	24.9836	32.01249	28.97488	5.196464	36.547	21.69585	25.49041	10.1863	4.669151	35.54587	24.0951	27.60235	32.8987	33.50593	18.58211	14.84013	5.131881	10.9216	15.23724
AC023274.	0.960908	2.000781	0	0	0	0	0	0	0	0	0	0	0	0	0	0	0	0	0
AC023283.	733.1724	697.2721	612.4689	702.5619	505.0131	585.788	753.6664	919.5448	513.6066	523.5811	513.2256	808.7488	785.4565	725.9618	873.3592	677.6994	683.5666	752.6803	800.403
AC023300.	3.84363	0	3.996535	0	0	5.709435	0	7.408216	0	0	24.0951	0.920078	0	0	1.956012	11.54233	1.026376	2.7304	3.585232
AC023300.	0	0	0	1.039293	0	1.141887	0	0	3.501863	0	0	0	1.028084	0	0.978006	0	1.026376	0	0
AC023301.	67.26353	39.01523	40.96448	97.69352	285.7311	180.4181	123.2036	106.4931	149.4128	133.5372	50.5997	161.0137	141.8756	120.9936	89.97654	106.3543	71.84634	37.31547	21.51139
AC023302.	6.726353	13.00508	12.98874	4.157171	12.18233	9.135096	12.7452	2.778081	15.17474	18.25329	15.66181	4.600391	5.140422	6.515042	10.75806	18.96239	12.31652	13.652	17.92616
AC023310.	8.648168	13.00508	23.97921	7.275049	11.07485	2.283774	8.496802	10.1863	3.501863	0.960699	0	3.680313	3.084253	3.722881	1.956012	6.595615	20.52753	5.4608	6.274156
AC023310.	0	0	0	0	0	0	1.69936	0	0	0	0	0	1.028084	0	0	0.824452	0	0	0
AC023310.	200.8297	370.1445	303.7366	149.6582	197.1323	303.7419	291.4403	61.11778	366.5283	126.8123	138.5468	166.5342	138.7914	147.0538	151.5909	172.3104	186.8005	148.3517	140.7203
AC023347.	0.960908	0	0	0	2.21497	0	0	0	0	0	0	0	0	0	0	0	0	0	0
AC023355.	142.2143	155.0605	194.8311	123.6758	183.8425	94.77662	182.6813	152.7945	96.88488	122.9695	55.41872	211.618	144.9599	107.9636	153.5469	122.0189	122.1388	287.6022	234.8327
AC023389.	1.921815	7.002733	2.997401	5.196464	2.21497	2.283774	1.69936	13.89041	10.50559	9.606993	1.204755	9.200782	7.196591	2.792161	15.64809	8.244518	2.052753	8.191201	3.585232
AC023389.	0.960908	0	0	0	1.107485	0	0	0	0	0	0	5.520469	1.028084	2.792161	0	3.297807	7.184634	0	0
AC023394.	1.921815	5.001952	0	2.078586	8.859879	9.135096	1.69936	0	15.17474	15.37119	1.204755	0.920078	1.028084	11.16864	7.824047	4.122259	1.026376	3.640534	4.48154
AC023403.	29.78813	34.01327	108.9056	8.314342	52.05179	61.6619	25.49041	166.6849	23.34575	64.36685	24.0951	31.28266	30.84253	51.18962	53.79032	100.5831	53.37157	30.0344	48.40063
AC023421.	23.06178	23.00898	16.98527	38.45383	6.64491	15.98642	19.54265	24.0767	0	9.606993	0	17.48149	13.3651	15.82225	12.71408	16.48904	28.73854	9.101334	17.02985
AC023421.	20.17906	9.003514	10.99047	22.86444	5.537425	12.56076	24.64073	15.74246	0	14.41049	3.614265	11.04094	6.168506	21.40657	10.75806	22.2602	14.36927	6.370934	14.34093
AC023424.	0	0	0	0	0	0	0	0	0	0	0	0	0	5.584322	0	0	0	0	0
AC023424.	33.63176	68.02655	81.92896	24.94303	60.91167	33.11472	34.83689	60.19176	11.67288	86.46293	51.80446	40.48344	63.74123	40.02097	68.46041	32.15362	37.97592	37.31547	43.02278
AC023449.	98.97348	67.02616	53.95322	24.94303	31.00958	111.9049	81.5693	60.19176	85.212	44.19217	72.28529	49.68422	38.03912	88.41843	56.72434	35.45143	46.18693	18.20267	47.50432
AC023469.	6.726353	0	0	0	0	0	1.69936	3.704108	0	1.921399	0	1.840156	0	0	2.934017	0.824452	0	0	1.792616
AC023469.	0	0	0	0	0	0	0.84968	0	0	0	1.204755	0	0	0	0	0	0	0	0
AC023481.	13.45271	61.02382	36.96795	0	21.04221	6.851322	20.39233	26.85478	8.171014	24.97818	15.66181	15.64133	8.224675	24.19873	11.73607	14.84013	8.21101	21.8432	10.7557
AC023490.	0	0	0.999134	6.235757	0	0	5.098081	9.26027	0	1.921399	0	1.840156	0	0	0	0	4.105505	0	0
AC023490.	12.4918	9.003514	12.98874	1.039293	8.859879	10.27698	6.797442	8.334243	3.501863	6.724895	3.614265	0.920078	8.224675	14.89152	3.912023	4.122259	11.29014	9.101334	2.688924
AC023509.	34.59267	41.01601	40.96448	42.611	8.859879	5.709435	49.28145	39.81916	0	12.48909	4.81902	46.92399	26.73019	30.71377	26.40616	18.13794	29.76491	21.8432	34.0597
AC023509.	358.4185	369.1441	313.728	209.9371	411.9844	461.3224	209.871	413.9341	426.06	646.5506	750.5623	385.5128	336.1836	397.4176	338.39	237.4421	271.9897	281.2312	188.2247
AC023509.	162.3934	128.05	146.8726	166.2868	212.6371	119.8981	160.5896	208.3561	373.5321	203.6682	232.5177	217.1385	180.9428	182.4212	184.8431	192.9217	229.9083	188.3976	183.7431
AC023509.	8.648168	0	3.996535	0	3.322455	0	2.549041	4.630135	4.669151	0.960699	12.04755	0.920078	10.28084	0.93072	10.75806	3.297807	2.052753	0	3.585232
AC023510.	3.84363	3.001171	4.995668	14.5501	8.859879	7.993209	8.496802	36.11505	0	9.606993	0	14.72125	1.028084	6.515042	1.956012	7.420066	3.079129	20.93307	25.09662
AC023510.	153.7452	95.03709	176.8467	227.6051	127.3608	139.3102	154.6418	133.3479	102.7213	81.65944	57.82823	190.4562	189.1675	122.8551	193.6452	138.5079	76.97822	307.6251	238.4179
AC023511.	92.24712	86.03358	65.94282	209.9371	89.70628	254.6408	170.7857	125.9397	59.53167	123.9302	60.23774	103.9688	93.55568	124.7165	97.80058	154.9969	141.6399	56.42827	113.8311

AC023511.	1.921815	0	0.999134	3.117878	3.322455	2.283774	0.84968	3.704108	0	0	0	2.760235	1.028084	7.445762	0.978006	6.595615	9.237386	1.820267	5.377848
AC023512.	5.765445	7.002733	0.999134	6.235757	1.107485	4.567548	11.89552	0.926027	0	6.724895	0	5.520469	2.056169	2.792161	4.890029	8.244518	9.237386	2.7304	11.652
AC023513.	0	0	0	0	0	1.141887	0	0	0	0	0	0	0	0	0	0	0	0	0.896308
AC023538.	0	1.00039	0	1.039293	0	0	0	0	0	0	0	0	0	0	0	0	0	0	0
AC023575.	0	1.00039	0	0	0	1.141887	2.549041	0	0	0	0	1.840156	1.028084	0	4.890029	0	0	0	0
AC023590.	1.921815	0	7.993069	12.47151	1.107485	5.709435	5.098081	4.630135	0	5.764196	2.40951	4.600391	4.112337	8.376483	0.978006	0.824452	0	0.910133	0.896308
AC023595.	0	0	0	0	0	0	0	1.69936	0	0	0	0	0	0	0	0	0	0	0.896308
AC023598.	15.37452	10.0039	21.98094	28.0609	44.2994	19.41208	28.88913	6.482189	12.84017	14.41049	30.11887	23.00196	27.75828	20.47585	34.2302	28.03136	7.184634	11.83173	24.20031
AC023632.	11.53089	12.00469	20.98181	10.39293	21.04221	27.40529	13.59488	35.18903	28.01491	9.606993	37.3474	34.04289	15.42127	11.16864	12.71408	10.71787	7.184634	14.56213	10.7557
AC023632.	197.947	153.0597	252.7808	166.2868	186.0575	168.9993	197.9755	226.8766	309.3312	230.5678	167.4609	162.8538	201.5045	198.2434	218.0953	150.8747	148.8246	123.7781	115.6237
AC023632.	0.960908	0	2.997401	0	3.322455	1.141887	0	0	2.334575	0	1.204755	0	0	0.93072	0.978006	0.824452	2.052753	0	0
AC023632.	0	0	4.995668	1.039293	7.752394	5.709435	5.947762	24.0767	1.167288	6.724895	2.40951	3.680313	3.084253	2.792161	0	4.122259	0	7.281067	0.896308
AC023632.	1643.152	1063.415	1577.632	1439.42	1423.118	762.7805	1564.261	1319.589	884.8041	1307.512	892.7234	1197.022	1355.015	1250.888	1509.063	925.035	1004.822	990.2252	790.5436
AC023644.	6.726353	0	0.999134	0	0	3.425661	0.84968	2.778081	0	7.685594	0	0.920078	1.028084	0.93072	0	0	0	7.281067	0
AC023669.	0	0	0	0	0	0	0	0	0	0	0	0	1.028084	0	0	0	0	0	1.792616
AC023762.	0	0	0.999134	4.157171	0	1.141887	0.84968	0	0	1.921399	0	4.600391	0	0	0	0	0	1.820267	0
AC023790.	0	6.002343	2.997401	1.039293	0	6.851322	0	7.408216	0	0	1.204755	0.920078	2.056169	0	0.978006	0.824452	1.026376	0.910133	0.896308
AC023790.	38.4363	29.01132	50.95582	101.8507	34.33203	36.54038	6.797442	49.07943	22.17847	37.46727	32.52838	68.08579	71.96591	55.84322	35.20821	55.23827	43.1078	47.32694	54.67478
AC023794.	27.86632	11.00429	16.98527	5.196464	8.859879	6.851322	24.64073	24.0767	28.01491	17.29259	10.84279	36.80313	30.84253	1.861441	18.58211	9.893422	7.184634	9.101334	10.7557
AC023794.	29.78813	15.00586	30.97314	15.58939	16.61227	20.55397	17.84329	5.556162	39.68778	18.25329	22.89034	23.00196	21.58977	13.03008	1.956012	13.19123	13.34289	10.01147	4.48154
AC023794.	206.5951	155.0605	251.7817	146.5403	111.856	208.9653	119.8049	156.4986	269.6435	163.3189	225.2892	176.655	175.8024	177.7676	171.151	120.37	115.9805	179.2963	122.7942
AC023794.	105.6998	11.00429	38.96621	32.21808	22.1497	17.12831	17.84329	61.11778	25.68033	32.66377	45.78069	70.84602	92.52759	21.40657	30.31818	22.2602	41.05505	45.50667	54.67478
AC023794.	85.52077	61.02382	87.92376	73.78979	84.16885	26.2634	60.3273	54.6356	81.71014	121.0481	59.03299	101.2086	71.96591	41.88241	34.2302	17.31349	14.36927	52.78774	62.74156
AC023796.	0.960908	6.002343	1.998267	2.078586	0	0	2.549041	6.482189	0	0	1.204755	3.680313	2.056169	2.792161	6.846041	3.297807	0	6.370934	0.896308
AC023813.	0	0	0	0	0	0	0.84968	0	0	0	0	1.840156	0	0	0	0.824452	0	3.640534	0
AC023813.	28.82723	8.003124	9.991337	10.39293	17.71976	9.135096	11.04584	17.59451	7.003726	13.44979	16.86657	8.280704	2.056169	12.09936	3.912023	12.36678	17.4484	13.652	8.066772
AC023813.	0	1.00039	0	0	0	0	0	0	0	0	0	0	1.028084	0	0	0	0	0	0
AC023818.	22.10087	42.0164	21.98094	11.43222	33.22455	34.25661	38.23561	29.63287	30.34948	14.41049	18.07132	36.80313	53.46039	34.43665	14.67009	24.73355	55.42432	11.83173	36.74863
AC023818.	249.836	253.0988	485.579	428.1886	323.3856	208.9653	427.3892	437.0848	173.9259	306.4631	199.9893	394.7136	381.4193	299.6919	313.9399	319.0629	247.3567	272.1299	347.7675
AC023824.	0	0	0	0	0	0	0	0	0	0	0	0	0	0	0	0	0	0	3.585232
AC023824.	0.960908	0	3.996535	1.039293	1.107485	0	0	0	0	0	0	0	0	0.93072	0	0	0	0	0
AC023824.	0	0	1.998267	0	0	0	0.84968	2.778081	9.338302	2.882098	0	0	0	0	0	5.771163	0	0	0
AC023825.	3.84363	4.001562	0	6.235757	0	2.283774	0	3.704108	4.669151	0	0	0.920078	0	5.584322	1.956012	3.297807	0	0	4.48154
AC023825.	14.41361	25.00976	41.96361	24.94303	31.00958	35.3985	11.89552	41.67122	15.17474	24.97818	68.67103	61.64524	26.73019	36.29809	27.38416	72.55176	33.87042	22.75334	38.54124
AC023830.	17.29634	10.0039	14.987	16.62868	18.82724	10.27698	2.549041	6.482189	0	6.724895	8.433284	0	13.3651	13.03008	1.956012	14.01568	2.052753	6.370934	8.963079
AC023830.	0	0	0.999134	2.078586	0	1.141887	1.69936	0	0	0	0	0	4.112337	1.861441	1.956012	0.824452	0	0	0.896308
AC023830.	57.65445	72.02811	65.94282	43.6503	78.63143	57.09435	55.22922	91.67668	164.5876	153.7119	178.3037	80.96688	54.48847	87.48771	62.59237	39.57369	47.21331	76.45121	72.60094
AC023855.	0	1.00039	3.996535	1.039293	11.07485	1.141887	6.797442	0	0	0	1.204755	11.96102	6.168506	1.861441	0.978006	0.824452	1.026376	3.640534	4.48154
AC023866.	0	2.000781	0	2.078586	0	0	8.496802	0	0	0.960699	0	0	0	0	1.956012	0	0	4.550667	1.792616
AC023866.	0	1.00039	0	0	0	11.41887	3.398721	1.852054	0	3.842797	0	0.920078	0	0	1.956012	9.06897	1.026376	1.820267	0.896308
AC023886.	30.74904	49.01913	58.94889	47.80747	25.47215	42.24982	45.03305	58.3397	56.02981	67.24895	75.89956	35.88305	59.62889	28.85233	42.05425	18.13794	22.58028	31.85467	26.88924
AC023886.	0	6.002343	2.997401	1.039293	1.107485	2.283774	0	1.852054	7.003726	2.882098	14.45706	1.840156	4.112337	0	2.934017	0.824452	0	14.56213	0.896308
AC023905.	2.882723	9.003514	3.996535	0	0	1.141887	6.797442	3.704108	0	0	6.023774	7.360626	1.028084	7.445762	0	0	8.21101	8.191201	6.274156
AC023906.	0	0	0.999134	0	1.107485	0	0.84968	0.926027	0	0	0	0	0	0	0.978006	0	1.026376	0	0
AC023906.	0	0	0	0	0	0	0	0	0	0	0	0	0	0	0	3.297807	0	0	0
AC023906.	26.90541	26.01015	31.97228	55.08252	26.57964	26.2634	40.78465	37.04108	28.01491	10.56769	46.98544	139.8519	115.1454	23.26801	42.05425	22.2602	32.84404	23.66347	34.0597
AC023906.	26.90541	40.01562	50.95582	28.0609	36.547	49.10114	96.86355	50.00546	22.17847	26.89958	8.433284	104.8889	76.07824	33.50593	57.70234	42.8715	36.94955	15.47227	37.64493
AC023908.	0	0	0	0	0	6.851322	0	3.704108	0	0	0	0	3.084253	0	0	0	1.026376	0	0
AC023908.	205.6342	333.13	438.6197	128.8723	315.6332	245.5057	339.8721	358.3725	273.1453	473.6247	442.145	430.5966	268.33	306.207	370.6642	193.7462	177.5631	374.0648	249.1736
AC023934.	0	0	1.998267	0	0	0	0	0	0	0	0	0	0	0	0	0	0	0	0
AC023968.	0	0	0	0	0	1.141887	0	0	0	0	0	0	0	5.584322	0.978006	1.648904	0	0	0
AC023983.	34.59267	25.00976	24.97834	14.5501	27.68712	29.68906	12.7452	16.66849	0	11.52839	32.52838	21.1618	23.64594	32.57521	24.45015	11.54233	5.131881	16.3824	25.09662
AC023983.	0.960908	19.00742	14.987	18.70727	25.47215	34.25661	6.797442	4.630135	53.69524	39.38867	25.29985	5.520469	5.140422	27.92161	7.824047	7.420066	28.73854	20.93307	25.09662
AC024022.	12.4918	14.00547	4.995668	13.51081	26.57964	4.567548	9.346483	15.74246	39.68778	0.960699	4.81902	2.760235	14.39318	12.09936	11.73607	12.36678	15.39564	8.191201	3.585232
AC024023.	0	0	0	0	0	0	0	0.926027	0	0	0	0.920078	2.056169	0	0	7.420066	1.026376	15.47227	12.54831
AC024028.	0	0	0.999134	0	0	0	0	0	0	0	0	0	0	0	0	3.297807	0	0	0.896308
AC024060.	451.6265	548.214	718.3771	517.5678	674.4583	753.6454	586.2794	636.1806	1236.158	842.5332	766.2241	639.4544	583.9519	554.7093	582.8915	521.878	526.531	614.3401	514.4808
AC024075.	1000.305	771.301	933.1908	1385.377	732.0475	694.2673	1350.992	679.7038	65.36811	384.2797	209.6273	985.4038	806.0181	756.6756	810.7668	838.4675	795.4416	873.7281	759.1728
AC024075.	3105.653	2820.101	2817.557	3327.815	3058.873	3091.088	3120.876	2465.084	5022.839	3494.063	3816.663	2842.122	2967.052	2978.305	3024.972	2580.534	2827.667	2594.79	2207.606
AC024075.	500.6328	428.1671	491.5738	698.4047	495.0458	345.9918	614.3188	356.5204	84.04472	296.8561	209.6273	600.8111	521.2388	492.351	422.4985	413.0504	449.5528	497.843	360.3158
AC024082.	2.882723	0	0.999134	2.078586	0	0	0	0	0.926027	0	5.764196	0	0	0	0	0	0	0	0	0
AC024084.	10.56998	5.001952	3.996535	7.275049	1.107485	1.141887	6.797442	10.1863	4.669151	0.960699	9.638039	0.920078	2.056169	27.92161	35.20821	9.893422	3.079129	15.47227	17.02985

AC024132.	21.13997	20.00781	1.998267	17.66798	4.42994	25.12151	11.89552	20.37259	3.501863	13.44979	18.07132	12.8811	6.168506	15.82225	19.56012	0.824452	11.29014	2.7304	8.963079
AC024145.	144.1361	126.0492	194.8311	240.0766	264.6889	145.0196	252.355	153.7205	164.5876	218.0787	150.5944	260.3821	230.2909	228.9572	175.063	152.5236	133.4289	143.8011	185.5357
AC024153.	0	1.00039	19.98267	1.039293	0	0	0	0	0	2.882098	0	0.920078	1.028084	0	0	0	0	0	0
AC024220.	1.921815	1.00039	0	0	0	0	3.398721	0	1.167288	0	1.204755	0.920078	4.112337	1.861441	0.978006	0.824452	0	0	0
AC024224.	0	0	0	0	0	1.141887	0	0	0	0	0	0	0	0.93072	0	0	0	0	0
AC024224.	7.68726	2.000781	4.995668	6.235757	2.21497	3.425661	5.947762	8.334243	0	0	3.614265	3.680313	2.056169	3.722881	5.868035	0	8.21101	5.4608	4.48154
AC024230.	0	0	0	0	0	0	0	0	5.836439	0	0	0	0	0	0	1.648904	3.079129	0	0
AC024243.	25.9445	16.00625	42.96275	13.51081	25.47215	23.97963	20.39233	4.630135	23.34575	11.52839	7.228529	36.80313	27.75828	24.19873	32.27419	24.73355	10.26376	11.83173	11.652
AC024257.	27.86632	13.00508	7.993069	3.117878	11.07485	9.135096	5.098081	7.408216	9.338302	3.842797	12.04755	12.8811	9.252759	14.89152	4.890029	6.595615	9.237386	4.550667	9.859387
AC024257.	0	0	10.99047	8.314342	0	0	0.84968	0	1.167288	0	12.04755	3.680313	0	0.93072	0	0	0	0.910133	0.896308
AC024257.	133.5661	164.064	306.734	111.2043	243.6467	142.7359	160.5896	318.5533	109.725	251.7032	227.6987	271.4231	214.8696	251.2945	212.2273	152.5236	151.9037	221.1624	205.2545
AC024257.	24.9836	61.02382	57.94975	17.66798	37.65449	21.69585	22.94137	44.4493	92.21573	33.62447	68.67103	23.00196	38.03912	42.81313	52.81231	55.23827	42.08143	34.58507	37.64493
AC024257.	4.804538	10.0039	12.98874	23.90373	1.107485	1.141887	22.09169	1.852054	14.00745	7.685594	3.614265	11.04094	10.28084	13.03008	2.934017	4.946711	19.50115	20.93307	14.34093
AC024267.	0	0	0	0	4.42994	0	0	0	0	0	0	3.680313	2.056169	0.93072	0	0	0	5.4608	0
AC024267.	134.5271	157.0613	126.89	213.055	118.5009	108.4793	193.7271	208.3561	32.68406	116.2446	44.57593	203.3373	171.6901	217.7886	195.6012	244.0377	251.4622	270.3096	265.3072
AC024267.	27.86632	28.01093	43.96188	31.17878	28.79461	30.83095	19.54265	38.89314	32.68406	42.27077	30.11887	34.04289	44.20763	27.92161	36.18622	19.78684	17.4484	26.39387	25.09662
AC024267.	1.921815	10.0039	24.97834	7.275049	5.537425	1.141887	4.248401	22.22465	9.338302	6.724895	10.84279	5.520469	29.81445	3.722881	11.73607	10.71787	5.131881	11.83173	14.34093
AC024270.	18.25724	28.01093	39.96535	16.62868	17.71976	12.56076	24.64073	17.59451	18.6766	7.685594	63.85201	19.32164	26.73019	19.54513	11.73607	11.54233	12.31652	32.7648	16.13354
AC024270.	23.06178	48.01874	35.96881	55.08252	27.68712	38.82416	41.63433	35.18903	23.34575	48.99566	38.55216	42.3236	46.2638	42.81313	35.20821	28.85581	62.60895	38.2256	34.95601
AC024270.	41.31902	49.01913	62.94542	27.02161	38.76197	27.40529	57.77826	46.30135	14.00745	35.54587	28.91412	45.08383	48.31997	32.57521	43.03226	46.99375	23.60665	36.40534	33.16339
AC024293.	52.84991	79.03084	48.95755	41.57171	117.3934	78.7902	16.9936	59.26573	164.5876	141.2228	181.918	64.40548	76.07824	61.42754	44.01026	42.8715	71.84634	99.20454	50.19325
AC024337.	49.00628	25.00976	67.94109	32.21808	65.34161	42.24982	14.44456	18.52054	0	13.44979	8.433284	57.96493	28.78636	35.36737	31.29619	33.80253	27.71216	70.08027	28.68185
AC024337.	1672.94	967.3776	2039.232	1209.737	1714.387	1296.042	1906.682	781.5668	2395.274	2319.128	2296.263	1649.7	1972.894	1005.178	920.3035	523.5269	701.015	685.3305	549.4368
AC024361.	331.5131	296.1156	417.6379	357.5167	320.0631	368.8295	345.8199	325.9615	330.3424	334.3233	271.0698	387.3529	349.5487	376.011	433.2566	265.4735	264.8051	265.759	241.1068
AC024361.	14.41361	32.01249	19.98267	35.33595	18.82724	43.39171	36.53625	28.70684	58.36439	18.25329	48.1902	35.88305	33.92678	26.99089	20.53812	23.9091	12.31652	20.02294	53.77848
AC024361.	46.12356	17.00664	26.97661	29.1002	26.57964	53.66869	25.49041	34.263	39.68778	44.19217	67.46627	29.4425	44.20763	23.26801	44.98827	16.48904	25.65941	28.21414	17.92616
AC024382.	0	0	0	0	0	0	0	0	0	0	0	1.840156	0	0	0	0	2.052753	5.4608	0
AC024405.	0	0	0	0	2.21497	4.567548	3.398721	0	0	0	1.204755	0	0	0	0.978006	0	0	0	0
AC024451.	0.960908	1.00039	0	0	0	0	0	0	0	0	0	8.280704	4.112337	0	0.978006	2.473355	0	0	0
AC024451.	55.73264	54.02108	71.93762	39.49313	40.97694	30.83095	68.8241	62.96984	57.1971	33.62447	16.86657	88.32751	81.21867	100.5178	88.02052	147.5769	97.50574	144.7112	152.3724
AC024451.	9.609075	12.00469	17.98441	10.39293	4.42994	3.425661	15.29424	14.81643	1.167288	7.685594	9.638039	4.600391	8.224675	0.93072	27.38416	3.297807	5.131881	2.7304	8.066772
AC024451.	16.33543	14.00547	18.98354	7.275049	14.3973	50.24303	19.54265	25.00273	43.18965	9.606993	14.45706	21.1618	18.50552	14.89152	14.67009	30.50472	15.39564	48.23707	30.47447
AC024475.	2.882723	2.000781	0.999134	2.078586	1.107485	4.567548	0.84968	2.778081	0	0	0	3.680313	1.028084	0.93072	0.978006	4.946711	0	1.820267	1.792616
AC024475.	0	0	0	0	0	0	3.398721	0	0	0	0	0.920078	0	0	0	0	0	0	0
AC024475.	5.765445	7.002733	4.995668	1.039293	2.21497	0	0.84968	3.704108	2.334575	0	0	0	3.084253	0.93072	3.912023	7.420066	2.052753	1.820267	7.170464
AC024558.	168.1588	107.0418	151.8683	104.9686	167.2302	102.7698	130.8508	181.5013	182.0969	136.4193	109.6327	138.0117	139.8195	94.93347	171.151	75.84957	82.1101	89.19307	104.868
AC024559.	73.02897	112.0437	79.93069	21.82515	81.95388	92.49285	66.27506	93.52873	51.36066	121.0481	68.67103	72.68618	67.85357	97.72563	77.26246	23.08465	33.87042	73.72081	96.80126
AC024559.	0	3.001171	0	3.117878	0	0	5.098081	0.926027	0	0	0	0.920078	0	1.861441	0	0	0	5.4608	0
AC024560.	19.21815	11.00429	26.97661	13.51081	34.33203	36.54038	6.797442	9.26027	60.69896	40.34937	55.41872	11.96102	9.252759	12.09936	6.846041	29.68027	20.52753	7.281067	4.48154
AC024560.	240.2269	274.107	371.6777	175.6645	253.614	237.5125	180.1322	299.1067	219.4501	267.0744	233.7224	256.7018	238.5156	250.3638	243.5235	277.8403	227.8555	208.4206	160.4391
AC024560.	42.27993	34.01327	124.8917	60.27898	77.52394	44.53359	62.87634	68.526	32.68406	40.34937	46.98544	65.32555	72.99399	57.70466	37.16422	60.18498	44.13418	43.6864	73.49725
AC024563.	14.41361	9.003514	6.993936	9.353635	16.61227	5.709435	5.098081	9.26027	5.836439	7.685594	3.614265	7.360626	2.056169	9.307203	8.802052	11.54233	16.42202	5.4608	0
AC024568.	6.726353	27.01054	10.99047	10.39293	5.537425	23.97963	39.08529	18.52054	5.836439	17.29259	6.023774	13.80117	19.5336	18.61441	14.67009	11.54233	11.29014	20.93307	2.688924
AC024575.	26.90541	18.00703	31.97228	41.57171	32.11706	18.27019	18.69297	30.55889	17.50932	31.70308	55.41872	28.52243	40.09529	37.22881	39.12023	32.97807	33.87042	22.75334	27.78555
AC024579.	0	0	0	0	0	0	0	0	0	0	0	0	0	0	0	0	0	2.7304	0
AC024580.	211.3997	129.0504	217.8111	113.2829	186.0575	123.3238	140.1972	136.126	102.7213	81.65944	104.8137	166.5342	207.673	192.6591	183.8651	131.9123	117.0069	127.4187	135.3425
AC024580.	1.921815	1.00039	0	1.039293	0	1.141887	0	0	1.167288	0	6.023774	0	0	0	0	0	0	0	0
AC024581.	0	0	0	0	0	3.425661	0.84968	3.704108	0	0	1.204755	2.760235	0	3.722881	0.978006	0	1.026376	0.910133	0.896308
AC024581.	0	0	0	0	0	0	0	0	0	0	0	0	0	0	0	0	0	0	1.792616
AC024587.	0.960908	0	0.999134	0	1.107485	1.141887	0	0	0	0.960699	0	0	0	0	0	0	0	0	3.585232
AC024587.	0	1.00039	0	1.039293	0	0	0	11.11232	4.669151	0.960699	0	0	0	3.722881	6.846041	4.946711	1.026376	0.910133	0.896308
AC024592.	12.4918	1.00039	1.998267	5.196464	0	2.283774	0	0.926027	10.50559	2.882098	1.204755	3.680313	2.056169	2.792161	7.824047	7.420066	4.105505	10.01147	1.792616
AC024592.	3.84363	1.00039	0	2.078586	1.107485	1.141887	4.248401	0	1.167288	0	0	3.680313	3.084253	0	0	2.473355	6.158258	0	4.48154
AC024597.	1.921815	0	1.998267	9.353635	1.107485	1.141887	0.84968	0	0	2.882098	0	0	0	0	0	9.893422	2.052753	3.640534	8.066772
AC024600.	4.804538	1.00039	0	2.078586	0	6.851322	2.549041	0	0	0	0	0.920078	3.084253	0.93072	0	0	4.105505	2.7304	4.48154
AC024601.	31.70995	32.01249	37.96708	40.53242	47.62185	35.3985	33.98721	72.23011	47.8588	26.89958	60.23774	48.76415	31.87062	38.15953	35.20821	37.10033	45.16056	24.5736	43.02278
AC024603.	0	3.001171	0	1.039293	0	3.425661	9.346483	0	7.003726	0	0	13.80117	13.3651	0	1.956012	0	1.026376	6.370934	3.585232
AC024610.	1.921815	4.001562	10.99047	0	0	1.141887	5.947762	0	2.334575	0.960699	0	0.920078	2.056169	2.792161	9.780058	2.473355	9.237386	0.910133	0
AC024614.	0	0	0	0	0	0	0	0	1.167288	0	0	0.920078	0	0	0	0	2.052753	0	0
AC024614.	0	1.00039	0	0	0	0	0.84968	0.926027	0	0	0	0	0	0	0	0	0	0	0
AC024619.	1.921815	0	0	8.314342	0	0	0.84968	0	0	0	0	0	0	0.93072	0	0	0	0	0
AC024619.	0	0	0	0	0	0	0	0	0	0	0	0	2.056169	0	0	0	0	0	0

AC024619.	10.56998	6.002343	1.998267	1.039293	6.64491	0	1.69936	3.704108	2.334575	1.921399	0	6.440548	5.140422	1.861441	0.978006	3.297807	8.21101	6.370934	3.585232
AC024619.	0.960908	1.00039	2.997401	0	1.107485	1.141887	0.84968	0	4.669151	0	0	0	0	0	0	0	1.026376	0.910133	0
AC024651.	5.765445	14.00547	37.96708	13.51081	15.50479	10.27698	18.69297	7.408216	4.669151	7.685594	10.84279	11.96102	19.5336	9.307203	3.912023	5.771163	9.237386	12.74187	13.44462
AC024681.	0	0	1.998267	0	0	0	0	0	0	0	0	0	0.920078	2.056169	0.93072	0	0	0	0
AC024884.	6.726353	0	0	6.235757	1.107485	4.567548	4.248401	0	7.003726	3.842797	7.228529	3.680313	3.084253	0	5.868035	0	0	3.640534	8.963079
AC024884.	0	5.001952	0.999134	3.117878	2.21497	5.709435	0	0	0	0	0	0.920078	3.084253	8.376483	10.75806	4.122259	5.131881	0.910133	7.170464
AC024896.	4107.88	4544.774	5422.298	5097.731	5136.515	3735.112	4783.7	5428.37	5828.268	6012.056	5367.183	3760.36	4104.113	4465.596	4674.868	3125.497	3319.301	3953.62	3907.006
AC024901.	10.56998	14.00547	18.98354	32.21808	18.82724	13.70264	22.94137	20.37259	7.003726	6.724895	12.04755	21.1618	11.30893	16.75297	13.69208	8.244518	11.29014	19.1128	14.34093
AC024908.	0	0	0	0	0	0	0	0.926027	0	0	0	0.920078	0	0	0	0.824452	0	0	0.896308
AC024909.	5.765445	1.00039	0.999134	0	0	0	6.797442	1.852054	4.669151	0	0	1.840156	2.056169	2.792161	1.956012	3.297807	1.026376	0.910133	0
AC024909.	0	0	0	0	0	1.141887	0	0	0	0	0	5.520469	0	0	0	0	0	0	0.896308
AC024909.	63.4199	54.02108	62.94542	67.55403	76.41646	36.54038	67.12474	67.59997	82.87743	87.42363	85.5376	61.64524	71.96591	27.92161	38.14223	89.86525	56.45069	51.8776	57.36371
AC024933.	5.765445	20.00781	15.98614	49.88605	14.3973	7.993209	45.03305	14.81643	7.003726	25.93888	7.228529	19.32164	15.42127	20.47585	33.2522	9.893422	16.42202	13.652	7.170464
AC024937.	0	0	7.993069	0	11.07485	1.141887	0.84968	0	0	2.882098	0	8.280704	0	0.93072	0	0.824452	1.026376	3.640534	5.377848
AC024937.	102.8171	109.0426	115.8995	99.77211	146.188	159.8642	101.1119	148.1643	85.212	88.38433	91.56137	118.6901	127.4825	128.4394	105.6246	94.81196	111.875	119.2275	107.557
AC024940.	35.55358	8.003124	21.98094	28.0609	32.11706	17.12831	27.18977	26.85478	44.35693	85.50223	99.99465	35.88305	32.8987	37.22881	28.36217	113.7744	93.40024	83.73227	60.05263
AC024940.	13.45271	23.00898	4.995668	10.39293	15.50479	12.56076	4.248401	3.704108	21.01118	12.48909	14.45706	16.56141	3.084253	7.445762	9.780058	8.244518	6.158258	9.101334	9.859387
AC024940.	0	0	0	0	2.21497	0	2.549041	0	0	0	9.638039	0.920078	0	0	0.978006	0	0	0	0
AC024940.	0	0	1.998267	1.039293	0	0	0.84968	0	0	0	0	0	0	0	0	2.473355	0	0	0
AC024941.	8.648168	5.001952	3.996535	4.157171	9.967364	2.283774	6.797442	7.408216	2.334575	10.56769	7.228529	14.72125	18.50552	2.792161	13.69208	3.297807	11.29014	11.83173	13.44462
AC024941.	50.9281	72.02811	21.98094	27.02161	57.58922	36.54038	28.88913	35.18903	22.17847	34.58517	48.1902	64.40548	75.05016	31.64449	34.2302	26.38246	19.50115	40.956	36.74863
AC024958.	3.84363	2.000781	8.992203	0	1.107485	0	0	1.852054	0	0	0	3.680313	1.028084	0	0.978006	4.122259	2.052753	0	0
AC024995.	0	0	0	0	0	1.141887	0	1.852054	0	0	0	0	0	0	0	0	1.026376	0	0
AC025030.	1.921815	0	1.998267	1.039293	0	0	1.69936	4.630135	9.338302	5.764196	1.204755	0	0	0	0	0.824452	2.052753	3.640534	11.652
AC025030.	0	3.001171	0	6.235757	3.322455	0	2.549041	0	0	0	0	3.680313	6.168506	12.09936	0.978006	0	1.026376	0.910133	0
AC025031.	24.02269	11.00429	27.97574	15.58939	26.57964	19.41208	16.14392	43.52327	30.34948	12.48909	4.81902	20.24172	9.252759	9.307203	34.2302	9.893422	14.36927	10.01147	11.652
AC025031.	8.648168	10.0039	0.999134	2.078586	6.64491	1.141887	6.797442	3.704108	3.501863	6.724895	3.614265	7.360626	5.140422	8.376483	2.934017	8.244518	12.31652	0.910133	6.274156
AC025031.	1.921815	0	1.998267	0	0	3.425661	0	0	0	0	1.204755	0	1.028084	0	0	0	0	0	0
AC025031.	49.00628	110.0429	147.8718	44.68959	81.95388	66.22945	87.51706	104.6411	47.8588	50.91706	38.55216	69.92595	65.7974	135.8852	99.75659	28.85581	59.52982	81.00187	74.39356
AC025031.	4.804538	22.00859	23.97921	17.66798	15.50479	6.851322	17.84329	25.92876	53.69524	15.37119	1.204755	17.48149	28.78636	28.85233	25.42815	16.48904	12.31652	16.3824	19.71877
AC025033.	0	1.00039	0	0	0	0	2.549041	0	0	0	0	0	0	0	0	0	0	0	0
AC025034.	229.6569	155.0605	273.7626	207.8586	126.2533	231.8031	214.9691	277.8081	85.212	139.3014	122.885	262.2223	240.5717	158.2225	196.5792	240.7399	179.6158	213.8814	158.6465
AC025035.	15.37452	27.01054	14.987	14.5501	8.859879	3.425661	6.797442	4.630135	59.53167	12.48909	19.27608	34.96297	33.92678	31.64449	46.94428	13.19123	12.31652	8.191201	6.274156
AC025038.	0	1.00039	0.999134	1.039293	0	0	0.84968	2.778081	0	0	0	1.840156	0	4.653602	2.934017	0	0	0	0
AC025040.	13.45271	13.00508	14.987	48.84676	22.1497	11.41887	23.79105	6.482189	37.35321	3.842797	9.638039	35.88305	19.5336	19.54513	25.42815	40.39814	56.45069	64.61947	35.85232
AC025040.	0	2.000781	0	1.039293	1.107485	0	0.84968	3.704108	0	0	1.204755	4.600391	1.028084	0	0	9.893422	2.052753	11.83173	1.792616
AC025043.	31.70995	35.01367	67.94109	56.12181	25.47215	43.39171	78.17058	37.96711	0	45.15286	14.45706	60.72516	65.7974	43.74385	44.01026	56.88718	40.02867	42.77627	36.74863
AC025048.	12.4918	4.001562	8.992203	3.117878	4.42994	11.41887	2.549041	14.81643	7.003726	9.606993	0	0.920078	7.196591	4.653602	6.846041	9.06897	9.237386	8.191201	15.23724
AC025048.	308.4513	382.1491	366.6821	309.7092	343.3203	467.0318	309.2836	410.23	427.2273	434.2361	538.5254	311.9065	400.9529	339.7129	350.1261	335.5519	380.7856	348.5811	360.3158
AC025048.	0	0	0	0	0	0	0	0	9.338302	0	7.228529	0	2.056169	0	0	0	0	0.910133	0.896308
AC025048.	8.648168	13.00508	12.98874	11.43222	14.3973	17.12831	6.797442	16.66849	16.34203	20.17468	36.14265	14.72125	26.73019	24.19873	20.53812	4.946711	8.21101	27.304	30.47447
AC025048.	53.81082	32.01249	43.96188	37.41454	31.00958	30.83095	35.68657	27.78081	15.17474	28.82098	6.023774	46.00391	33.92678	63.28898	45.96627	14.84013	18.47477	29.12427	23.30401
AC025062.	0.960908	0	3.996535	10.39293	0	1.141887	1.69936	14.81643	0	0.960699	4.81902	4.600391	1.028084	9.307203	2.934017	4.946711	10.26376	5.4608	0.896308
AC025062.	0	0	0	0	0	0	0	0	0	1.921399	0	0.920078	1.028084	0	0	0	1.026376	0	0
AC025125.	96.09075	106.0414	277.7592	81.06484	177.1976	161.0061	175.8838	75.00819	187.9333	243.0569	220.4701	259.4621	299.1726	116.34	158.4369	35.45143	42.08143	49.1472	80.66772
AC025154.	17.29634	2.000781	9.991337	35.33595	6.64491	15.98642	10.19616	10.1863	0	6.724895	15.66181	14.72125	12.33701	8.376483	21.51613	35.45143	40.02867	6.370934	8.066772
AC025161.	0	0	0	0	0	1.141887	0	0	1.167288	0	0	0	0	0	0	0.824452	0	0	0
AC025162.	22.10087	40.01562	47.95842	19.74656	42.08443	52.5268	27.18977	62.04381	81.71014	97.99132	40.96167	36.80313	24.67402	60.49682	41.07624	33.80253	21.5539	63.70934	22.4077
AC025164.	0.960908	3.001171	1.998267	0	0	0	2.549041	0	0	0	0	0	1.028084	0.93072	0	0	0	0	0.896308
AC025164.	672.6353	340.1327	522.5469	390.7741	407.5545	256.9246	352.6173	315.7752	298.8257	359.3015	361.4265	374.4718	346.4644	432.7849	447.9267	432.0128	374.6273	590.6766	456.2207
AC025165.	926.3149	751.2932	800.3061	342.9666	730.94	616.619	856.4777	727.8573	1224.485	953.9744	783.0907	1279.829	1460.908	997.7322	883.1393	550.7338	642.5115	551.5409	583.4965
AC025165.	87.44259	111.0433	133.8839	131.9902	93.02873	147.3034	162.2889	176.8712	99.21946	179.6508	221.6749	163.7739	132.6229	146.1231	128.1188	149.2258	175.5103	92.83361	142.513
AC025165.	820.615	987.3854	680.41	1253.387	603.5793	984.3066	1016.218	1069.561	674.6923	801.2232	540.9349	712.1406	831.7203	698.9709	669.934	1153.408	1072.563	486.0112	628.3119
AC025166.	0	0	8.992203	1.039293	0	1.141887	1.69936	0	9.338302	6.724895	1.204755	5.520469	0	0	3.912023	7.420066	3.079129	3.640534	0
AC025171.	759.1169	664.2593	849.2636	619.4185	868.2682	615.4771	683.1429	582.471	583.6439	667.686	739.7195	770.1055	808.0743	702.6938	826.4149	660.3859	589.14	902.8523	854.1815
AC025171.	351.6922	398.1554	615.4663	376.224	481.7559	310.5933	548.8934	549.134	306.9967	447.6859	399.9786	697.4193	641.5246	542.6099	505.629	332.2541	274.0425	605.2387	554.8146
AC025171.	19.21815	22.00859	35.96881	10.39293	11.07485	7.993209	2.549041	33.33697	10.50559	9.606993	43.37118	23.00196	17.47743	26.06017	12.71408	9.06897	5.131881	36.40534	33.16339
AC025171.	245.9923	167.0652	234.7964	111.2043	165.0153	186.1276	152.9424	212.9862	583.6439	206.5503	210.8321	161.9338	196.3641	198.2434	128.1188	140.9813	109.8223	188.3976	121.0016
AC025171.	27.86632	10.0039	58.94889	12.47151	62.01916	17.12831	15.29424	25.00273	14.00745	16.33189	24.0951	16.56141	27.75828	28.85233	26.40616	28.85581	36.94955	10.9216	22.4077
AC025174.	0	0	10.99047	0	0	0	3.398721	0.926027	0	7.685594	4.81902	1.840156	1.028084	0	1.956012	0.824452	4.105505	0	0
AC025175.	122.9962	128.05	105.9082	80.02554	130.6832	105.0536	79.86994	64.82189	80.54285	224.8036	246.9747	105.809	82.24675	150.7767	163.327	100.5831	80.05735	73.72081	69.91202
AC025176.	1.921815	10.0039	0	7.275049	11.07485	0	5.098081	4.630135	11.67288	16.33189	0	0	1.028084	5.584322	4.890029	5.771163	2.052753	23.66347	4.48154

AC025178.	147.9798	285.1113	272.7635	182.9155	170.5527	266.0597	311.8326	250.9533	197.2716	293.974	274.6841	169.2944	136.7352	251.2945	227.8754	183.8528	273.0161	206.6003	233.9364
AC025180.	9.609075	2.000781	15.98614	0	2.21497	9.135096	3.398721	0.926027	0	5.764196	2.40951	1.840156	6.168506	2.792161	0	9.06897	1.026376	25.48374	7.170464
AC025181.	0	0	0	1.039293	0	0	0	0	0	0	0	0.920078	1.028084	0	0	0	0	1.820267	1.792616
AC025181.	147.9798	196.0765	200.8259	107.0472	259.1515	135.8846	113.0075	125.9397	380.5358	272.8386	289.1412	138.0117	139.8195	134.9544	118.3387	138.5079	178.5895	232.9942	158.6465
AC025183.	0	0	0	0	0	0	0	0	0	0	0	0	0	0	0	0	1.026376	0.910133	0
AC025186.	0	2.000781	15.98614	0	0	1.141887	16.14392	1.852054	0	10.56769	2.40951	5.520469	4.112337	10.23792	10.75806	1.648904	0	7.281067	0.896308
AC025187.	0	0	0	0	0	0	0	0	0	0	0	0.920078	0	0	0	0	0	1.820267	0
AC025188.	25.9445	20.00781	16.98527	18.70727	0	21.69585	17.84329	5.556162	21.01118	28.82098	10.84279	30.36258	51.40422	18.61441	32.27419	1.648904	2.052753	13.652	14.34093
AC025198.	1.921815	0	0.999134	0	0	1.141887	2.549041	9.26027	0	0	0	2.760235	5.140422	0.93072	0	1.648904	6.158258	0.910133	0
AC025211.	0	3.001171	0	6.235757	0	2.283774	3.398721	0	0	0	1.204755	3.680313	0	0	0	0	2.052753	0	1.792616
AC025219.	1.921815	2.000781	1.998267	0	1.107485	2.283774	38.23561	0	1.167288	0.960699	0	0.920078	2.056169	1.861441	1.956012	0	0	4.550667	2.688924
AC025244.	3.84363	0	0.999134	0	0	0	2.549041	0	0	0.960699	0	3.680313	0	1.861441	0	3.297807	1.026376	0	3.585232
AC025244.	0	0	4.995668	0	0	0	0	0.926027	0	0	0	0	1.028084	0	0	1.648904	0	0	0
AC025252.	17.29634	36.01406	10.99047	4.157171	7.752394	23.97963	28.03945	5.556162	4.669151	3.842797	1.204755	0	2.056169	15.82225	5.868035	14.01568	13.34289	8.191201	14.34093
AC025253.	5.765445	1.00039	1.998267	7.275049	1.107485	3.425661	5.098081	1.852054	3.501863	6.724895	1.204755	0.920078	3.084253	9.307203	5.868035	2.473355	10.26376	4.550667	0.896308
AC025254.	2.882723	1.00039	1.998267	1.039293	1.107485	1.141887	1.69936	0	0	0.960699	0	0.920078	2.056169	0	0	0	3.079129	1.820267	1.792616
AC025259.	66.30262	8.003124	46.95928	15.58939	24.36467	13.70264	22.94137	11.11232	28.01491	30.74238	18.07132	47.84407	47.29188	37.22881	37.16422	12.36678	14.36927	18.20267	25.09662
AC025259.	0	0	0	0	0	0	0	0	0	0	0	0.920078	1.028084	0.93072	0	0	0	0	0
AC025259.	3160.425	1277.499	616.4655	363.7525	1996.795	602.9163	2520.152	702.8545	2370.761	557.2056	899.9519	1204.382	3013.315	2201.154	2097.822	411.4015	304.8337	192.0382	153.2687
AC025260.	0	0	2.997401	0	8.859879	0	0	0	0	3.842797	0	0.920078	1.028084	0	0.978006	0	0	0	0
AC025262.	30.74904	25.00976	49.95668	37.41454	15.50479	21.69585	37.38593	26.85478	4.669151	13.44979	26.50461	24.84211	24.67402	20.47585	23.47214	18.13794	11.29014	15.47227	31.37078
AC025262.	2.882723	8.003124	11.9896	13.51081	1.107485	2.283774	4.248401	0.926027	17.50932	0.960699	4.81902	3.680313	3.084253	4.653602	4.890029	21.43575	15.39564	4.550667	2.688924
AC025263.	4.804538	24.00937	22.98007	7.275049	2.21497	6.851322	7.647122	0.926027	22.17847	14.41049	4.81902	12.8811	19.5336	14.89152	10.75806	11.54233	12.31652	15.47227	16.13354
AC025263.	0	0	0	0	1.107485	0	0	0	7.003726	0	0	0	1.028084	3.722881	0	0	0	0	0
AC025265.	168.1588	36.01406	148.8709	93.53635	155.0479	156.4385	102.8113	150.0164	381.7031	108.559	150.5944	86.48735	98.6961	151.7074	173.107	67.60505	80.05735	100.1147	96.80126
AC025265.	0.960908	0	0	8.314342	0	0	0.84968	0	0	0	0	0	0	1.861441	0	0.824452	0	0	0
AC025268.	44.20175	24.00937	69.93936	89.37918	85.27634	33.11472	96.86355	41.67122	0	43.23147	16.86657	107.6492	92.52759	80.04195	69.43841	73.37621	85.18923	86.46267	69.01571
AC025271.	0	0	0	0	0	0	0	0.926027	1.167288	0.960699	0	1.840156	0	0	0	0	0	0	0.896308
AC025271.	4.804538	2.000781	24.97834	1.039293	21.04221	1.141887	5.947762	7.408216	0	1.921399	7.228529	12.8811	19.5336	10.23792	11.73607	0.824452	5.131881	2.7304	2.688924
AC025271.	0	1.00039	9.991337	0	6.64491	0	4.248401	0	2.334575	0.960699	1.204755	0.920078	3.084253	3.722881	5.868035	0	0	0	1.792616
AC025271.	26.90541	12.00469	1.998267	1.039293	19.93473	18.27019	6.797442	12.03835	31.51677	26.89958	30.11887	20.24172	15.42127	17.68369	22.49413	14.84013	15.39564	9.101334	3.585232
AC025277.	0	1.00039	0.999134	0	0	0	1.69936	0	0	8.646293	0	0	0	0.93072	0	5.771163	1.026376	0	0
AC025278.	0.960908	9.003514	11.9896	50.92535	14.3973	28.54718	12.7452	8.334243	4.669151	17.29259	15.66181	17.48149	8.224675	5.584322	4.890029	19.78684	6.158258	9.101334	8.066772
AC025279.	74.95079	60.02343	153.8666	83.14342	54.26676	13.70264	83.26866	54.6356	36.18592	24.97818	25.29985	73.60626	87.38717	69.80402	99.75659	48.64266	57.47707	62.79921	60.94894
AC025279.	1.921815	1.00039	1.998267	7.275049	3.322455	0	0	1.852054	7.003726	0	0	0.920078	0	4.653602	0	0	0	0.910133	0.896308
AC025280.	73.98988	22.00859	52.95408	16.62868	37.65449	5.709435	33.13753	11.11232	21.01118	27.86028	13.2523	34.04289	74.02207	49.32818	54.76833	24.73355	12.31652	26.39387	10.7557
AC025280.	221.0087	179.0699	270.7652	83.14342	654.5236	180.4181	401.8988	73.15614	290.6546	354.498	221.6749	478.4407	487.312	492.351	450.8607	112.9499	92.37386	182.0267	188.2247
AC025283.	5.765445	7.002733	6.993936	14.5501	7.752394	3.425661	5.947762	6.482189	33.85134	11.52839	4.81902	5.520469	7.196591	2.792161	24.45015	10.71787	1.026376	0.910133	8.066772
AC025283.	10.56998	37.01445	31.97228	22.86444	14.3973	13.70264	51.83049	12.03835	29.18219	17.29259	18.07132	33.12282	24.67402	26.99089	25.42815	10.71787	29.76491	19.1128	20.61508
AC025287.	0.960908	0	0	0	1.107485	0	1.69936	0	0	0	2.40951	0	0	0	0	0	0	0	0
AC025287.	85.52077	116.0453	103.9099	49.88605	87.49131	100.4861	55.22922	83.34243	233.4575	119.1267	99.99465	51.52438	59.62889	93.07203	47.92229	72.55176	83.13648	71.90054	101.2828
AC025287.	259.445	261.1019	369.6795	231.7623	245.8617	178.1344	306.7346	293.5506	59.53167	206.5503	163.8467	396.5537	388.6159	305.2763	340.346	212.7086	195.0115	268.4894	254.5515
AC025287.	10.56998	11.00429	25.97748	11.43222	25.47215	13.70264	16.14392	12.96438	26.84762	16.33189	16.86657	10.12086	10.28084	3.722881	7.824047	16.48904	12.31652	7.281067	8.066772
AC025366.	6.726353	7.002733	3.996535	4.157171	5.537425	7.993209	6.797442	1.852054	0	9.606993	0	1.840156	7.196591	4.653602	4.890029	8.244518	9.237386	1.820267	7.170464
AC025419.	3.84363	5.001952	21.98094	2.078586	1.107485	3.425661	3.398721	0	24.51304	2.882098	4.81902	14.72125	2.056169	1.861441	7.824047	5.771163	4.105505	6.370934	9.859387
AC025423.	4.804538	7.002733	4.995668	6.235757	11.07485	1.141887	10.19616	1.852054	14.00745	9.606993	14.45706	18.40156	13.3651	16.75297	13.69208	6.595615	19.50115	6.370934	0.896308
AC025423.	7.68726	1.00039	4.995668	4.157171	2.21497	2.283774	0	2.778081	0	9.606993	7.228529	3.680313	2.056169	1.861441	1.956012	1.648904	0	4.550667	2.688924
AC025423.	1.921815	0	0	0	0	1.141887	0.84968	0.926027	0	0.960699	0	0.920078	6.168506	0.93072	10.75806	0	5.131881	0	3.585232
AC025430.	34.59267	65.02538	75.93416	22.86444	32.11706	18.27019	36.53625	55.56162	14.00745	24.97818	26.50461	48.76415	28.78636	42.81313	47.92229	33.80253	36.94955	49.1472	21.51139
AC025431.	0	0	0	0	0	0	0	0	0	0	0	0	0	0.93072	0	0	0	0	0.896308
AC025434.	0	0	0	0	3.322455	0	0	0	0	0	0	4.600391	0	0	0	0	0	0	0
AC025437.	1.921815	0	1.998267	2.078586	0	0	0	0	0	2.882098	0	2.760235	1.028084	0	0	0	0	0	2.688924
AC025437.	0	0	0	0	2.21497	1.141887	0.84968	0	0	0	0	0.920078	0	0	0.978006	0.824452	0	0	0
AC025437.	2.882723	0	0	5.196464	0	0	1.69936	3.704108	0	0	0	0	0	3.722881	0	3.297807	0	0	0
AC025437.	0.960908	0	0	0	0	1.141887	0.84968	0	0	3.842797	0	0	0	0	3.912023	0	0	0.910133	0
AC025437.	0	1.00039	0	4.157171	0	0	0	0	0	0	0	4.600391	4.112337	0	0	4.946711	0	0	0
AC025437.	0	0	0	0	0	0	1.69936	0	0	0	0	0	1.028084	0	0	0	0	0	0
AC025442.	0	0	0	0	0	1.141887	0	0	0	0	0	1.840156	0	0	0	0.824452	0	0	0.896308
AC025445.	0	1.00039	0	0	0	0	1.69936	0.926027	0	0	0	5.520469	1.028084	0.93072	0	0	0	0	0
AC025449.	113.3871	132.0515	139.8787	151.7367	107.426	147.3034	104.5107	211.1342	384.0377	242.0962	263.8413	108.5692	122.342	140.5388	147.6789	123.6678	117.0069	104.6653	69.01571
AC025458.	0	5.001952	2.997401	0	5.537425	3.425661	0.84968	0	1.167288	0	1.204755	4.600391	0	4.653602	2.934017	11.54233	0	3.640534	4.48154
AC025459.	1.921815	0	0	0	0	3.425661	0	3.704108	1.167288	0	1.204755	0	0	0	0	1.648904	0	0	0

AC025465.	3.84363	0	0	0	0	2.283774	4.248401	0	0	0	3.614265	1.840156	1.028084	0	0	2.473355	3.079129	0.910133	3.585232
AC025465.	0	0	0	0	0	0	0	0	0	0	13.2523	0	0	0	0	0	0	0	0
AC025470.	0	0	0.999134	0	0	4.567548	0.84968	1.852054	0	0	0	0.920078	4.112337	0	0	0.824452	0	0	0
AC025470.	103.778	66.02577	106.9073	43.6503	45.40688	26.2634	72.22282	60.19176	18.6766	12.48909	1.204755	49.68422	48.31997	124.7165	58.68035	15.66458	15.39564	7.281067	18.82247
AC025518.	0.960908	5.001952	5.994802	0	0	4.567548	0	3.704108	1.167288	0.960699	3.614265	0.920078	2.056169	0.93072	0	4.122259	0	1.820267	0
AC025518.	0	6.002343	1.998267	0	0	4.567548	3.398721	5.556162	0	0.960699	0	2.760235	0	0	4.890029	0	1.026376	0.910133	0.896308
AC025521.	5.765445	6.002343	0.999134	1.039293	1.107485	1.141887	0.84968	0.926027	2.334575	0.960699	4.81902	2.760235	5.140422	0	5.868035	2.473355	10.26376	0.910133	3.585232
AC025524.	3.84363	0	2.997401	4.157171	1.107485	0	0.84968	0	0	0	0	0.920078	2.056169	0	7.824047	0	0	0	2.688924
AC025524.	1.921815	2.000781	0.999134	0	0	0	0	0	0	0	1.204755	0	0	0.93072	3.912023	0	0	3.640534	0.896308
AC025539.	24.02269	4.001562	26.97661	21.82515	28.79461	7.993209	27.18977	12.03835	33.85134	21.13538	18.07132	17.48149	17.47743	13.9608	9.780058	32.97807	41.05505	50.96747	69.91202
AC025569.	519.851	542.2116	632.4516	467.6817	509.4431	461.3224	436.7356	416.7122	260.3052	647.5113	526.4779	389.1931	428.7112	745.507	757.9545	371.0033	420.8143	431.4032	429.3315
AC025572.	0.960908	1.00039	3.996535	1.039293	0	0	2.549041	0	0	0	1.204755	0	1.028084	0.93072	0	0.824452	0	3.640534	2.688924
AC025574.	0	1.00039	0	0	0	0	0	0.926027	0	0	0	0.920078	0	0	0	0.824452	1.026376	0	0
AC025575.	0	4.001562	0	0	0	1.141887	0	0	0	3.842797	1.204755	1.840156	1.028084	0	3.912023	0.824452	0	1.820267	2.688924
AC025575.	39.39721	107.0418	122.8934	103.9293	67.55658	49.10114	116.4062	62.96984	63.03354	96.06993	112.0422	57.04485	58.60081	73.5269	102.6906	68.4295	89.29473	52.78774	100.3865
AC025576.	0.960908	9.003514	11.9896	6.235757	3.322455	10.27698	15.29424	19.44657	0	4.803496	0	13.80117	12.33701	9.307203	5.868035	9.893422	7.184634	10.01147	16.13354
AC025578.	0	0	0	0	0	0	0	0	0	0	0	0	0	0	0	4.946711	0	0.910133	0
AC025580.	9.609075	4.001562	2.997401	0	0	1.141887	9.346483	0	0	4.803496	6.023774	3.680313	1.028084	7.445762	18.58211	22.2602	16.42202	60.06881	51.98586
AC025580.	13.45271	8.003124	22.98007	13.51081	11.07485	21.69585	15.29424	24.0767	21.01118	26.89958	40.96167	18.40156	29.81445	20.47585	6.846041	14.84013	16.42202	4.550667	5.377848
AC025580.	61.49808	24.00937	69.93936	16.62868	34.33203	19.41208	22.94137	46.30135	23.34575	39.38867	33.73314	43.24368	14.39318	35.36737	31.29619	12.36678	21.5539	10.9216	19.71877
AC025588.	49.00628	97.03787	84.92636	20.78586	56.48173	82.21586	60.3273	87.04654	36.18592	72.05244	57.82823	79.12673	72.99399	80.04195	91.93255	53.59397	76.97822	40.04587	57.36371
AC025594.	329.5913	327.1277	216.812	279.5698	410.8769	324.2959	207.322	328.7396	714.3801	361.2229	389.1358	211.618	278.6109	321.0985	253.3035	337.2008	371.5482	237.5448	381.8272
AC025594.	57.65445	60.02343	9.991337	93.53635	36.547	57.09435	39.08529	52.78354	94.55031	41.31007	45.78069	20.24172	24.67402	44.67457	17.6041	42.8715	28.73854	71.90054	28.68185
AC025627.	6.726353	9.003514	11.9896	23.90373	0	11.41887	5.947762	12.96438	12.84017	3.842797	8.433284	11.04094	4.112337	4.653602	8.802052	14.84013	5.131881	2.7304	11.652
AC025627.	2.882723	1.00039	2.997401	1.039293	0	0	0	0	0	0	0	0	2.056169	0	0.978006	0	0	0	3.585232
AC025627.	0	0	0.999134	0	1.107485	0	0	0	0	0	0	0	0	0	0	0.824452	0	0	0
AC025627.	0	0	0.999134	0	0	1.141887	0	0.926027	0	0	0	0	0	0	0	0.824452	0	0	0
AC025647.	0	3.001171	0	6.235757	0	1.141887	3.398721	0.926027	0	4.803496	0	2.760235	1.028084	1.861441	0.978006	0.824452	0	3.640534	4.48154
AC025674.	0	0	0.999134	1.039293	1.107485	0	0	0	0	0	0	0	2.056169	0	0	0.824452	0	0	0
AC025674.	5.765445	0	0	0	0	0	0	0	0	0	0	0	0	0	0	0	0	0	0
AC025678.	0	0	0	0	0	0	0	0	0	0	0	0	0	1.861441	0	0	0	0	0
AC025678.	0	2.000781	5.994802	2.078586	2.21497	5.709435	0.84968	2.778081	2.334575	0.960699	1.204755	7.360626	2.056169	2.792161	0.978006	0.824452	6.158258	1.820267	11.652
AC025678.	28.82723	40.01562	25.97748	59.23969	7.752394	15.98642	62.02666	26.85478	78.20828	40.34937	50.5997	307.3061	290.9479	34.43665	42.05425	44.5204	70.81996	39.13574	22.4077
AC025682.	55.73264	51.01991	94.9177	58.2004	80.8464	67.37133	73.0725	99.08489	128.4017	124.8909	130.1135	69.00587	51.40422	100.5178	70.41642	56.88718	60.5562	90.10321	52.88217
AC025741.	9.609075	24.00937	4.995668	15.58939	2.21497	5.709435	21.24201	4.630135	9.338302	8.646293	12.04755	11.96102	28.78636	8.376483	8.802052	26.38246	14.36927	24.5736	22.4077
AC025750.	0.960908	0	0	0	0	0	0	4.630135	0	0	1.204755	0.920078	0	2.792161	0.978006	0	0	0	0
AC025750.	0	0	0	6.235757	0	0	0.84968	0	0	7.685594	1.204755	0	1.028084	0	7.824047	0.824452	1.026376	0	0
AC025754.	1.921815	1.00039	0	0	0	2.283774	0	0	0	0	1.204755	3.680313	0	2.792161	0	0	0	0	0.896308
AC025754.	10.56998	18.00703	21.98094	35.33595	42.08443	36.54038	13.59488	34.263	29.18219	44.19217	49.39495	9.200782	6.168506	21.40657	29.34017	12.36678	57.47707	10.01147	13.44462
AC025755.	0	0	0	0	0	0	0	0	0	0	0	0	0	0	3.912023	0	0	0	0
AC025766.	31.70995	54.02108	48.95755	20.78586	39.86946	20.55397	62.02666	70.37805	12.84017	29.78168	31.32363	48.76415	46.2638	33.50593	58.68035	38.74924	21.5539	45.50667	31.37078
AC025773.	0	0	0.999134	0	0	0	0.84968	0	0	0	0	0	0	0	0	0.824452	0	3.640534	1.792616
AC025809.	79.75532	78.03045	101.9116	92.49706	59.80419	45.67548	112.1578	75.93422	24.51304	38.42797	50.5997	79.12673	110.005	101.4485	101.7126	53.58937	65.68808	61.88907	69.01571
AC025809.	221.0087	164.064	374.6751	269.1768	244.7542	115.3306	333.9243	287.9994	213.6137	222.8822	201.1941	267.7428	308.4253	344.3665	397.0704	211.8841	221.6973	234.8144	240.2105
AC025810.	0	0	1.998267	0	4.42994	0	0	3.704108	0	0	0	0	0	5.584322	1.956012	0.824452	0	0	0
AC025811.	8.648168	1.00039	5.994802	5.196464	5.537425	2.283774	10.19616	5.556162	3.501863	0	0	10.12086	4.112337	8.376483	4.890029	2.473355	1.026376	2.7304	8.066772
AC025822.	31.70995	8.003124	24.97834	22.86444	5.537425	11.41887	16.9936	5.556162	0	0.960699	0	8.280704	7.196591	23.26801	34.2302	15.66458	2.052753	29.12427	30.47447
AC025857.	0	1.00039	0	0	0	1.141887	0	0	7.003726	0	0	0	1.028084	1.861441	0	0	0	0	0
AC025857.	1216.509	1546.604	1668.553	949.9136	1884.939	1375.974	800.3988	1126.049	2257.534	1956.944	1675.814	1358.035	1428.009	1597.116	1813.223	938.2262	976.0838	1469.865	1221.668
AC025871.	0	0	1.998267	5.196464	11.07485	0	1.69936	0.926027	0	2.882098	2.40951	0	0	1.861441	7.824047	3.297807	4.105505	0.910133	0
AC025871.	13.45271	13.00508	11.9896	23.90373	8.859879	10.27698	18.69297	15.74246	16.34203	11.52839	27.70936	4.600391	19.5336	13.9608	13.69208	16.48904	10.26376	6.370934	4.48154
AC025881.	0	8.003124	0	0	0	0	0.84968	0	7.003726	0	0	0	0	0	0	0	0	0	0
AC025884.	21.13997	36.01406	24.97834	12.47151	4.42994	4.567548	57.77826	0	0	11.52839	36.14265	86.48735	49.34805	1.861441	1.956012	0.824452	1.026376	40.956	23.30401
AC025884.	2.882723	0	0.999134	0	0	0	0	0	0	0.960699	6.023774	5.520469	1.028084	0	0	0	0	0	1.792616
AC025884.	0	6.002343	0	0	0	0	0.84968	0	0	1.921399	0	0	0	0	0	0	0	0	0
AC025887.	1.921815	2.000781	1.998267	0	0	1.141887	3.398721	1.852054	0	4.803496	0	3.680313	1.028084	2.792161	0.978006	1.648904	2.052753	4.550667	0
AC025917.	324.7867	308.1203	495.5703	341.9273	173.8751	259.2084	548.8934	256.5095	40.85507	187.3364	95.17564	248.4211	275.5266	298.7612	412.7185	218.4797	239.1457	253.9272	312.8115
AC025918.	20.17906	33.01288	48.95755	29.1002	27.68712	13.70264	41.63433	48.15341	4.669151	31.70308	16.86657	34.96297	24.67402	21.40657	20.53812	28.03136	6.158258	41.86614	30.47447
AC025918.	0	0	0	1.039293	0	0	0.84968	0	0	0	0	0.920078	0	0	0	0	0	0	0
AC025946.	0.960908	0	0	0	0	0	0.84968	0	0	0	0	0	0	0	0	0.824452	0	2.7304	0.896308
AC026108.	0	0	0	0	0	0	0	0	0	3.842797	0	0	0	0	0.978006	0	0	0.910133	0
AC026111.	3.84363	2.000781	0	7.275049	0	0	6.797442	10.1863	0	4.803496	21.68559	0	4.112337	2.792161	5.868035	1.648904	2.052753	0.910133	3.585232

AC026117.	0	0	0	0	0	0	0	0	0	0	0	0.920078	0	0	0	0	0	0	0	0.896308
AC026120.	16.33543	5.001952	1.998267	3.117878	24.36467	4.567548	2.549041	1.852054	35.01863	6.724895	8.433284	8.280704	7.196591	8.376483	8.802052	4.946711	7.184634	2.7304	0.896308
AC026120.	1.921815	0	0.999134	3.117878	0	1.141887	5.947762	0	0	0.960699	7.228529	1.840156	6.168506	0	0	0	2.052753	0	0
AC026124.	0	1.00039	0.999134	2.078586	11.07485	1.141887	1.69936	11.11232	12.84017	2.882098	1.204755	7.360626	2.056169	7.445762	1.956012	9.893422	6.158258	4.550667	0.896308
AC026124.	10.56998	6.002343	7.993069	20.78586	5.537425	5.709435	11.89552	6.482189	10.50559	0	6.023774	3.680313	6.168506	5.584322	0.978006	18.96239	9.237386	4.550667	6.274156
AC026150.	51.88901	138.0539	73.93589	181.8762	97.45867	51.38492	116.4062	104.6411	31.51677	59.56335	44.57593	105.809	99.72418	156.361	109.5367	140.9813	156.0092	124.6883	123.6905
AC026150.	41.31902	11.00429	12.98874	34.29666	31.00958	71.93888	10.19616	93.52873	84.04472	21.13538	61.4425	18.40156	26.73019	20.47585	44.98827	44.5204	37.97592	17.29253	20.61508
AC026150.	25.9445	17.00664	64.94369	44.68959	31.00958	4.567548	47.58209	48.15341	16.34203	24.97818	18.07132	43.24368	34.95487	42.81313	49.8783	29.68027	23.60665	28.21414	23.30401
AC026167.	2.882723	0	1.998267	0	0	0	0	0	2.334575	4.803496	7.228529	0	0	3.722881	0.978006	0	1.026376	0	1.792616
AC026191.	19.21815	9.003514	24.97834	7.275049	6.64491	7.993209	16.14392	6.482189	32.68406	10.56769	0	3.680313	1.028084	5.584322	3.912023	4.122259	3.079129	6.370934	11.652
AC026202.	0	0	0	0	0	0	0	0	0	0	0	0	0	0	0	0	0	1.820267	0
AC026202.	26.90541	48.01874	40.96448	22.86444	37.65449	20.55397	43.33369	49.07943	46.69151	56.68126	34.93789	48.76415	29.81445	76.31906	65.52639	22.2602	13.34289	65.52961	53.77848
AC026250.	59.57627	35.01367	69.93936	56.12181	31.00958	45.67548	45.88273	77.78627	9.338302	24.97818	30.11887	68.08579	35.98295	112.6172	104.6466	59.36053	67.74083	108.3059	43.91909
AC026254.	0	0	0	0	0	0	0	0	0	0	0	0	0	0	0	0	4.105505	0	0
AC026254.	0	6.002343	0.999134	0	5.537425	5.709435	0	0	0	0.960699	0	0.920078	0	0.93072	3.912023	0	1.026376	2.7304	0.896308
AC026254.	54.77173	49.01913	50.95582	30.13949	31.00958	36.54038	38.23561	37.04108	68.86998	24.97818	37.3474	58.88501	87.38717	39.09025	41.07624	41.22259	37.97592	39.13574	59.15632
AC026271.	434.3302	492.1921	459.6015	537.3144	475.111	461.3224	262.5512	516.7231	806.5958	801.2232	861.3997	349.6297	382.4474	430.9235	426.4105	356.1632	344.8624	354.0419	248.2773
AC026271.	0.960908	0	0	0	2.21497	0	1.69936	0	0	0	4.81902	2.760235	1.028084	0.93072	0.978006	0.824452	1.026376	2.7304	2.688924
AC026271.	0	0	0	5.196464	0	0	0	0	0	1.921399	0	0	0	0	0	0	0	0	0
AC026271.	332.474	323.1261	339.7054	430.2672	334.4604	282.0461	390.0032	400.9697	200.7735	237.2927	204.8083	368.0313	310.4815	434.6464	284.5997	277.0158	213.4863	309.4454	346.8712
AC026304.	0	2.000781	3.996535	1.039293	0	0	0	0	0	0.960699	2.40951	2.760235	1.028084	0	1.956012	0	0	1.820267	5.377848
AC026310.	0	0	0	0	4.42994	0	0	0	0	0	0	0	0	0	0	0	0	0.910133	0
AC026310.	81.67714	77.03006	79.93069	95.61493	55.37425	67.37133	81.5693	54.6356	4.669151	24.01748	13.2523	92.9279	76.07824	55.84322	71.39443	68.4295	71.84634	196.5888	138.9277
AC026316.	0.960908	0	0	0	0	2.283774	0.84968	0	0	0	0	0	0	0	0	0	1.026376	0	0
AC026316.	0	3.001171	26.97661	2.078586	12.18233	2.283774	1.69936	0.926027	10.50559	9.606993	0	1.840156	0	5.584322	1.956012	0	2.052753	1.820267	0.896308
AC026316.	6.726353	24.00937	5.994802	2.078586	14.3973	1.141887	22.09169	45.37532	31.51677	1.921399	19.27608	22.08188	15.42127	14.89152	25.42815	18.13794	7.184634	8.191201	25.09662
AC026320.	0	0	0	0	0	0	0	0	0	0	0	0	0	0	5.868035	0	0	0	0
AC026333.	3.84363	1.00039	0	0	0	0	0	0	0	0	0	3.680313	8.224675	0.93072	6.846041	0	1.026376	0	1.792616
AC026333.	35.55358	18.00703	17.98441	8.314342	15.50479	46.81737	13.59488	21.29862	137.74	38.42797	33.73314	24.84211	31.87062	25.12945	28.36217	23.08465	24.63303	26.39387	11.652
AC026336.	12.4918	7.002733	20.98181	1.039293	11.07485	0	0.84968	0	1.167288	19.21399	8.433284	2.760235	7.196591	0	0.978006	5.771163	8.21101	3.640534	4.48154
AC026336.	0.960908	0	0	0	0	0	0.84968	4.630135	7.003726	0	0	0	0	0	0.978006	0	0	0	0
AC026336.	0	1.00039	0	0	0	5.709435	0	0	0	0	0	0	0	0	0	0	0	0	0
AC026347.	0	0	0	0	1.107485	1.141887	0	1.852054	0	0	0	0	0	0.93072	0	0	0	0	0
AC026348.	47.08447	46.01796	56.95062	69.63262	60.91167	23.97963	60.3273	60.19176	7.003726	83.58083	51.80446	54.28462	56.54464	39.09025	52.81231	34.62698	23.60665	48.23707	54.67478
AC026355.	8.648168	7.002733	1.998267	10.39293	0	0	1.69936	16.66849	10.50559	5.764196	7.228529	3.680313	2.056169	0.93072	12.71408	32.15362	21.5539	10.9216	7.170464
AC026355.	1.921815	0	0	0	1.107485	1.141887	0	0	0	0	0	1.840156	0	0	0	35.45143	22.58028	5.4608	0.896308
AC026355.	0	0	0	0	0	0	0	0	0	0	0	3.680313	0	0	3.912023	0	0	0.910133	0
AC026356.	424.7211	347.1355	428.6283	327.3772	470.6811	349.4174	389.1535	511.1669	454.0749	569.6947	508.4066	350.5498	514.0422	529.5799	576.0454	259.7023	259.6732	361.323	350.4564
AC026356.	324.7867	339.1324	428.6283	221.3694	435.2416	253.4989	388.3039	450.9752	368.8629	400.6116	349.3789	351.4699	322.8185	387.1796	467.4868	169.8371	189.8796	261.2083	274.2702
AC026358.	53.81082	57.02225	85.9255	94.57564	37.65449	84.49964	81.5693	42.59724	12.84017	34.58517	32.52838	48.76415	65.7974	65.15042	54.76833	81.62073	80.05735	81.00187	65.43048
AC026362.	13.45271	36.01406	19.98267	10.39293	15.50479	18.27019	16.9936	22.22465	36.18592	27.86028	34.93789	22.08188	28.78636	16.75297	20.53812	25.55801	22.58028	20.02294	31.37078
AC026362.	4.804538	7.002733	17.98441	14.5501	38.76197	18.27019	9.346483	19.44657	52.52795	18.25329	20.48083	31.28266	32.8987	13.03008	8.802052	5.771163	25.65941	9.101334	18.82247
AC026366.	5855.77	3531.378	1889.362	3488.906	4844.139	4360.866	3106.431	4041.182	2469.981	4063.758	5047.923	2323.198	1996.54	2732.595	2679.736	5534.545	4797.283	9121.357	6629.99
AC026367.	12.4918	11.00429	17.98441	10.39293	21.04221	9.135096	5.947762	9.26027	17.50932	16.33189	27.70936	0.920078	6.168506	6.515042	17.6041	13.19123	12.31652	4.550667	10.7557
AC026367.	94.16894	60.02343	72.93676	57.1611	58.6967	83.35775	38.23561	42.59724	119.0633	80.69874	139.7516	80.04681	84.30292	53.98178	56.72434	41.22259	45.16056	54.608	25.99293
AC026367.	297.8813	190.0742	130.8865	247.3517	155.0479	154.1547	196.2761	154.6465	212.4464	278.6028	191.556	192.2964	212.8135	129.3701	139.8548	93.16306	137.5344	107.3957	130.861
AC026368.	14.41361	13.00508	28.97488	19.74656	18.82724	14.84453	12.7452	14.81643	38.5205	9.606993	13.2523	11.96102	18.50552	26.06017	20.53812	28.03136	4.105505	12.74187	9.859387
AC026369.	1524.96	900.3514	544.5278	1171.283	846.1185	1318.879	515.7559	910.2846	1728.753	888.6468	956.5754	576.8891	696.0131	743.6455	710.0322	728.8154	719.4898	637.0934	771.7211
AC026369.	22.10087	28.01093	53.95322	21.82515	53.15928	14.84453	26.34009	34.263	88.71387	57.64196	59.03299	32.20274	29.81445	66.08114	42.05425	43.69595	54.39794	45.50667	40.33386
AC026369.	0	7.002733	5.994802	25.98232	15.50479	1.141887	0	0	4.669151	0	0	11.04094	4.112337	12.09936	22.49413	86.56744	47.21331	65.52961	8.963079
AC026391.	0	0	0	0	1.107485	0	0	0	0	0	0	0	0	0.93072	0	0	0	0	0
AC026396.	0	0	0	2.078586	0	0	0	0	0	0	0	0	0	0	0	0	0	0	0
AC026398.	0	0	0.999134	1.039293	0	2.283774	0	0	0	0	4.81902	0	0	0	0.978006	0.824452	1.026376	0	0
AC026401.	1.921815	4.001562	9.991337	20.78586	2.21497	3.425661	10.19616	9.26027	0	14.41049	10.84279	2.760235	6.168506	0.93072	12.71408	5.771163	1.026376	0	0
AC026401.	10.56998	7.002733	21.98094	9.353635	3.322455	4.567548	32.28785	9.26027	7.003726	10.56769	2.40951	28.52243	9.252759	4.653602	6.846041	10.71787	2.052753	15.47227	10.7557
AC026401.	360.3403	311.1214	406.6474	130.9509	402.017	424.782	206.4723	394.4875	820.6033	407.3365	609.606	261.3022	249.8245	308.0684	355.9941	281.1381	222.7236	307.6251	218.6991
AC026402.	0	1.00039	0	0	0	0	1.69936	3.704108	0	0	2.40951	0	0	0	0.978006	0	0	0	0
AC026403.	17.29634	22.00859	8.992203	4.157171	17.71976	12.56076	4.248401	3.704108	37.35321	24.97818	38.55216	24.84211	8.224675	12.09936	21.51613	21.43575	28.73854	24.5736	5.377848
AC026407.	0.960908	0	0	4.157171	0	1.141887	0.84968	4.630135	0	4.803496	0	5.520469	4.112337	0.93072	0.978006	1.648904	4.105505	0	1.792616
AC026410.	0	1.00039	0	0	0	0	7.647122	0	0	0.960699	2.40951	0.920078	0	2.792161	3.912023	0	0	0	6.274156
AC026410.	2.882723	2.000781	7.993069	6.235757	8.859879	3.425661	5.098081	0	2.334575	3.842797	16.86657	5.520469	4.112337	11.16864	2.934017	4.946711	12.31652	14.56213	16.13354
AC026412.	2228.345	1547.604	2129.154	1966.342	1783.051	1492.446	2098.71	2055.78	1478.954	1879.128	1720.39	2035.213	2334.78	1702.287	1607.842	1447.737	1602.173	1741.995	1644.725

AC026412.	7.68726	19.00742	35.96881	3.117878	6.64491	27.40529	41.63433	23.15068	32.68406	9.606993	1.204755	31.28266	24.67402	38.15953	48.90029	47.81821	55.42432	36.40534	43.02278
AC026412.	133.5661	184.0718	123.8926	154.8546	127.3608	127.8913	134.2495	116.6794	119.0633	40.34937	84.33284	137.0917	157.2969	152.6381	156.4809	166.5393	172.4312	161.0936	279.6481
AC026422.	0	1.00039	0	0	0	0	0.84968	0.926027	4.669151	0	0	3.680313	0	0	0	0	0	0	0
AC026427.	1.921815	1.00039	0.999134	1.039293	4.42994	2.283774	5.098081	1.852054	7.003726	1.921399	0	4.600391	5.140422	0.93072	2.934017	4.946711	1.026376	0	0.896308
AC026427.	0	2.000781	2.997401	0	0	1.141887	0	0.926027	0	0	0	0	1.028084	3.722881	0	0	0	3.640534	1.792616
AC026434.	0.960908	0	2.997401	0	1.107485	4.567548	0	0	0	4.803496	0	0	1.028084	0	0	0	0	0	0
AC026436.	49.00628	44.01718	56.95062	59.23969	125.1458	30.83095	130.8508	42.59724	72.37184	74.93454	72.28529	67.16571	47.29188	133.093	129.0968	81.62073	97.50574	56.42827	39.43755
AC026458.	4.804538	0	2.997401	0	0	5.709435	0.84968	0	9.338302	0	4.81902	1.840156	1.028084	11.16864	1.956012	0	1.026376	0.910133	0
AC026458.	3.84363	12.00469	12.98874	1.039293	1.107485	7.993209	14.44456	5.556162	2.334575	7.685594	0	1.840156	7.196591	5.584322	4.890029	0.824452	2.052753	3.640534	0.896308
AC026461.	5.765445	0	1.998267	0	0	0	0	0	0	1.921399	0	0	3.084253	3.722881	0.978006	16.48904	0	7.281067	0
AC026461.	2.882723	0	3.996535	6.235757	3.322455	2.283774	0	1.852054	10.50559	4.803496	2.40951	3.680313	1.028084	0.93072	0	1.648904	4.105505	4.550667	0.896308
AC026462.	10.56998	2.000781	0.999134	1.039293	4.42994	0	0.84968	0.926027	2.334575	5.764196	8.433284	2.760235	7.196591	4.653602	7.824047	3.297807	3.079129	10.01147	13.44462
AC026462.	1.921815	5.001952	6.993936	6.235757	0	15.98642	0.84968	0	7.003726	3.842797	0	4.600391	8.224675	7.445762	0.978006	4.122259	0	1.820267	0.896308
AC026464.	16.33543	1.00039	0	17.66798	17.71976	10.27698	9.346483	0	0	19.21399	4.81902	9.200782	0	21.40657	14.67009	4.946711	3.079129	7.281067	3.585232
AC026468.	1.921815	0	5.994802	0	0	0	0.84968	5.556162	0	0	0	0.920078	1.028084	0	0	1.648904	0	0.910133	0.896308
AC026469.	24.02269	15.00586	5.994802	67.55403	11.07485	6.851322	36.53625	25.92876	44.35693	8.646293	46.98544	16.56141	18.50552	30.71377	14.67009	37.10033	61.58258	39.13574	22.4077
AC026470.	0	1.00039	1.998267	0	0	0	0.84968	0	0	5.764196	0	0	1.028084	0	0	0.824452	0	3.640534	0.896308
AC026470.	13.45271	10.0039	15.98614	13.51081	6.64491	4.567548	35.68657	15.74246	28.01491	13.44979	0	19.32164	8.224675	15.82225	8.802052	9.893422	12.31652	18.20267	20.61508
AC026471.	1025.288	808.3155	1271.897	985.2495	852.7634	616.619	1154.715	858.4271	290.6546	732.0528	612.0155	1122.495	1230.617	913.0366	1052.334	902.7748	908.343	1107.632	932.1603
AC026471.	42.27993	46.01796	97.9151	31.17878	45.40688	57.09435	39.93497	61.11778	143.5764	83.58083	118.066	75.44642	47.29188	88.41843	48.90029	29.68027	43.1078	54.608	76.18618
AC026471.	0	0	0	0	0	0	0	0	5.836439	0	6.023774	0	0	0	0	0	0	0	0
AC026471.	320.9431	268.1046	462.5989	295.1591	415.3068	328.8635	356.016	303.7369	499.5991	468.8212	383.1121	472.9202	367.0261	386.2489	318.8299	302.5738	271.9897	350.4014	345.0786
AC026471.	0	0	0	0	0	1.141887	0	0	1.167288	0	0	0.920078	0	3.722881	0	0	0	0	1.792616
AC026471.	176.807	151.059	258.7756	130.9509	296.806	215.8166	161.4392	279.6602	475.0861	270.9172	274.6841	199.657	299.1726	161.0146	186.7991	130.2634	144.7191	258.4779	347.7675
AC026474.	0.960908	0	0	1.039293	5.537425	0	0	0	11.67288	0	4.81902	0.920078	0	0	0	1.648904	0	0.910133	0
AC026477.	0	0	0.999134	0	0	1.141887	0	0	4.669151	0	0	0	0	0.93072	0	0	0	3.640534	0.896308
AC026495.	12.4918	28.01093	22.98007	13.51081	3.322455	3.425661	24.64073	0	12.84017	7.685594	96.38039	24.84211	6.168506	0	0	0.824452	0	16.3824	7.170464
AC026523.	33.63176	39.01523	38.96621	33.25737	42.08443	47.95925	28.88913	35.18903	47.8588	24.97818	66.26152	31.28266	34.95487	19.54513	12.71408	14.01568	18.47477	11.83173	8.066772
AC026523.	28.82723	70.02733	49.95668	65.47544	78.63143	57.09435	39.08529	50.93149	80.54285	32.66377	74.6948	80.04681	93.55568	47.46674	36.18622	49.46711	28.73854	23.66347	48.40063
AC026583.	0.960908	0	0	1.039293	0	0	0	0	0	0	0	0	0	0	0	0.824452	0	0.910133	0
AC026585.	41.31902	122.0476	70.93849	70.67191	60.91167	82.21586	63.72602	71.30408	144.7437	82.62014	81.92333	46.00391	40.09529	48.39746	43.03226	32.97807	51.31881	75.54107	67.2231
AC026616.	0	0	0	2.078586	0	4.567548	0	0	0	0	0	0.920078	0	0	0	0	0	0	0
AC026620.	0	1.00039	0	0	0	0	0	3.704108	0	0	0	0	1.028084	0	0	0	6.158258	0	0
AC026634.	0	1.00039	8.992203	0	0	1.141887	0	0	0	0	7.228529	0	0	0	0	0	0	0	0
AC026688.	0	1.00039	0	0	1.107485	1.141887	0	0	0	3.842797	0	0	0	0	3.912023	0	0	0	0.896308
AC026691.	313.2559	312.1218	406.6474	336.7309	215.9596	244.3638	249.806	300.9588	372.3648	233.4499	268.6603	366.1911	360.8576	307.1377	335.456	220.9531	246.3303	271.2198	276.9592
AC026700.	0	0	0	1.039293	0	0	4.248401	0	0	0	1.204755	0.920078	0	0.93072	3.912023	0	2.052753	0.910133	5.377848
AC026704.	8.648168	1.00039	9.991337	8.314342	18.82724	14.84453	11.04584	12.03835	1.167288	3.842797	0	10.12086	9.252759	15.82225	15.64809	4.946711	6.158258	2.7304	4.48154
AC026719.	0	0	0	0	0	0	0.84968	0	0	0	0	1.840156	0	0	0	0	0	0	0
AC026725.	24.9836	47.01835	47.95842	27.02161	28.79461	23.97963	32.28785	34.263	29.18219	42.27077	54.21397	49.68422	47.29188	5.584322	29.34017	37.10033	11.29014	26.39387	61.84525
AC026726.	0.960908	9.003514	18.98354	8.314342	8.859879	1.141887	16.9936	0	9.338302	22.09608	1.204755	12.8811	10.28084	0.93072	11.73607	7.420066	0	16.3824	9.859387
AC026740.	3.84363	10.0039	0.999134	3.117878	14.3973	2.283774	5.947762	5.556162	0	4.803496	10.84279	1.840156	1.028084	0.93072	0.978006	12.36678	10.26376	1.820267	8.066772
AC026741.	14.41361	21.0082	27.97574	3.117878	17.71976	6.851322	22.09169	6.482189	23.34575	29.78168	22.89034	4.600391	6.168506	10.23792	13.69208	4.946711	4.105505	22.75334	17.02985
AC026746.	1.921815	5.001952	21.98094	2.078586	11.07485	2.283774	31.43817	16.66849	0	5.764196	0	9.200782	2.056169	9.307203	9.780058	5.771163	3.079129	7.281067	7.170464
AC026748.	67.26353	98.03826	51.95495	50.92535	48.72934	51.38492	99.41259	75.93422	183.2642	130.6551	104.8137	91.08774	112.0612	70.73474	63.57038	65.95615	100.5849	134.6997	207.9434
AC026765.	0	1.00039	0.999134	1.039293	0	1.141887	0.84968	0	0	0	0	0	0	0.93072	0.978006	2.473355	0	0	0
AC026765.	1.921815	3.001171	6.993936	2.078586	1.107485	1.141887	1.69936	6.482189	7.003726	9.606993	0	9.200782	0	0.93072	0.978006	5.771163	13.34289	0	8.963079
AC026770.	10.56998	9.003514	11.9896	9.353635	13.28982	5.709435	21.24201	4.630135	15.17474	7.685594	7.228529	21.1618	15.42127	13.03008	13.69208	17.31349	15.39564	5.4608	16.13354
AC026771.	32.67086	25.00976	40.96448	42.611	34.33203	33.11472	49.28145	44.4493	23.34575	36.50657	72.28529	25.76219	28.78636	24.19873	31.29619	24.73355	30.79129	46.4168	48.40063
AC026774.	101.8562	128.05	47.95842	73.78979	118.5009	79.93209	98.56291	100.9369	64.20083	107.5983	110.8374	104.8889	127.4825	95.86419	73.35044	120.37	133.4289	104.6653	158.6465
AC026774.	21.13997	44.01718	27.97574	68.59332	9.967364	28.54718	17.84329	20.37259	9.338302	14.41049	4.81902	27.60235	8.224675	7.445762	38.14223	30.50472	12.31652	14.56213	16.13354
AC026780.	4.804538	16.00625	11.9896	5.196464	19.93473	3.425661	5.098081	6.482189	8.171014	1.921399	2.40951	3.680313	4.112337	9.307203	8.802052	12.36678	10.26376	4.550667	6.274156
AC026780.	24.9836	44.01718	47.95842	42.611	59.80419	18.27019	14.44456	80.56435	11.67288	3.842797	27.70936	25.76219	19.5336	21.40657	57.70234	37.10033	21.5539	31.85467	37.64493
AC026782.	0	0	0	0	4.42994	0	0	0	0	0	0	0	0	0	0	0	0	0	0
AC026782.	0	0	0	1.039293	4.42994	1.141887	0.84968	0	0	0.960699	7.228529	0	0	1.861441	3.912023	1.648904	1.026376	0	0
AC026784.	0	0	0.999134	0	0	0	0	0	4.669151	0	0	0	0	0	0.978006	0	3.079129	0	0
AC026785.	0	1.00039	0	0	0	5.709435	0	0.926027	0	1.921399	0	0	0	0	0	0	2.052753	0.910133	0
AC026786.	0	0	0.999134	0	2.21497	0	2.549041	0	0	0.960699	12.04755	8.280704	1.028084	0.93072	0.978006	0	0	3.640534	5.377848
AC026787.	0.960908	4.001562	0	8.314342	8.859879	0	1.69936	0	0	3.842797	1.204755	0	0	0.93072	0	0	0	0	0
AC026790.	113.3871	150.0586	173.8493	156.9332	132.8982	168.9993	116.4062	114.8274	93.38302	180.6115	239.7462	198.7369	136.7352	166.5989	130.0748	163.2415	160.1147	181.1165	275.1665
AC026790.	16.33543	19.00742	12.98874	11.43222	6.64491	1.141887	19.54265	6.482189	23.34575	15.37119	6.023774	2.760235	6.168506	6.515042	9.780058	1.648904	1.026376	16.3824	10.7557
AC026797.	0	0	0	1.039293	8.859879	4.567548	0	0	0	0	0	2.760235	0	1.861441	0	0	0	0	0

AC026798.	0	2.000781	0	1.039293	0	0	0	0.926027	0	0	0	0.920078	1.028084	0	0	0	0	0	0
AC026801.	40.35812	24.00937	28.97488	29.1002	14.3973	13.70264	24.64073	24.0767	1.167288	4.803496	12.04755	19.32164	10.28084	7.445762	25.42815	7.420066	27.71216	20.93307	24.20031
AC026801.	8.648168	11.00429	4.995668	8.314342	5.537425	14.84453	2.549041	1.852054	3.501863	4.803496	0	14.72125	4.112337	12.09936	8.802052	1.648904	1.026376	4.550667	1.792616
AC026801.	300.7641	319.1245	270.7652	254.6267	227.0344	246.6476	241.3092	314.8492	456.4095	260.3495	271.0698	206.0975	261.1334	140.5388	229.8314	204.4641	253.5149	179.2963	198.9804
AC026803.	14.41361	36.01406	38.96621	28.0609	24.36467	35.3985	28.03945	17.59451	38.5205	18.25329	16.86657	31.28266	20.56169	16.75297	24.45015	23.08465	31.81766	18.20267	17.92616
AC026803.	180.6506	159.0621	162.8588	98.73281	120.7159	103.9117	139.3476	78.7123	140.0745	125.8516	210.8321	125.1306	140.8476	174.0447	150.6129	102.232	131.3762	108.3059	131.7573
AC026826.	0	0	0	0	4.42994	0	0	5.556162	0	0	0	0	0	0	0	0	0	0	0
AC026826.	0.960908	0	0	0	0	0	0	0	0	0	0	0	0	0	0	0	0	0	0.896308
AC026877.	31.70995	15.00586	23.97921	23.90373	9.967364	23.97963	21.24201	24.0767	4.669151	12.48909	8.433284	16.56141	32.8987	20.47585	18.58211	7.420066	21.5539	32.7648	20.61508
AC026888.	0	3.001171	0.999134	1.039293	0	0	0	0	0	0	0	0	0	0	0	0.824452	1.026376	0	0
AC026894.	2.882723	2.000781	0.999134	1.039293	5.537425	2.283774	1.69936	0.926027	0	0	12.04755	0	2.056169	5.584322	5.868035	2.473355	13.34289	1.820267	0.896308
AC026904.	6.726353	16.00625	39.96535	2.078586	28.79461	15.98642	23.79105	20.37259	98.05217	35.54587	20.48083	40.48344	46.2638	29.78305	40.09824	37.92478	30.79129	12.74187	16.13354
AC026904.	6.726353	0	0	1.039293	0	3.425661	0	4.630135	0	0	1.204755	1.840156	0	1.861441	0	3.297807	5.131881	0.910133	2.688924
AC026904.	31.70995	7.002733	29.97401	25.98232	12.18233	1.141887	37.38593	5.556162	28.01491	2.882098	16.86657	25.76219	31.87062	22.33729	24.45015	15.66458	16.42202	20.02294	9.859387
AC026904.	5.765445	8.003124	15.98614	1.039293	4.42994	1.141887	10.19616	17.59451	14.00745	8.646293	3.614265	18.40156	9.252759	15.82225	10.75806	2.473355	4.105505	10.9216	17.92616
AC026950.	0	0	0	0	0	1.141887	3.398721	0	0	0	0	0	0	0	5.868035	0	0	0	0
AC026951.	2.882723	6.002343	4.995668	1.039293	6.64491	0	4.248401	0	4.669151	3.842797	8.433284	0.920078	0	1.861441	3.912023	6.595615	1.026376	0.910133	0.896308
AC026954.	44.20175	25.00976	31.97228	38.45383	16.61227	13.70264	33.13753	15.74246	40.85507	1.921399	9.638039	38.64329	25.70211	23.26801	17.6041	17.31349	9.237386	39.13574	29.57816
AC026956.	1.921815	0	0	0	0	0	0	0.926027	3.501863	0	0	0	0	1.861441	0	0	2.052753	0	0.896308
AC026979.	24.9836	11.00429	13.98787	33.25737	9.967364	15.98642	12.7452	16.66849	12.84017	15.37119	15.66181	20.24172	31.87062	28.85233	20.53812	9.06897	23.60665	19.1128	14.34093
AC026979.	9.609075	28.01093	33.97054	16.62868	24.36467	14.84453	28.88913	36.11505	8.171014	24.01748	14.45706	37.72321	13.3651	36.29809	32.27419	8.244518	7.184634	16.3824	10.7557
AC026979.	117.2307	135.0527	139.8787	48.84676	112.9635	150.7291	80.71962	133.3479	314.0004	227.6857	368.655	156.4133	156.2688	129.3701	83.1305	77.49847	133.4289	153.8125	102.1791
AC026979.	117.2307	147.0574	128.8882	177.7191	114.0709	161.0061	85.8177	134.2739	113.2269	171.9652	137.3421	96.60821	105.8927	119.1322	136.9208	110.4765	141.6399	70.08027	77.08248
AC026991.	0.960908	0	7.993069	0	0	4.567548	0	0	0	0	0	0	0	0.93072	0	0	1.026376	0	0
AC026992.	13.45271	6.002343	18.98354	69.63262	16.61227	13.70264	14.44456	33.33697	5.836439	29.78168	13.2523	15.64133	17.47743	26.99089	15.64809	23.9091	16.42202	8.191201	27.78555
AC026992.	117.2307	123.048	88.9229	388.6955	115.1784	149.5872	132.5501	172.241	23.34575	77.81664	67.46627	91.08774	102.8084	190.7977	139.8548	437.7839	244.2775	65.52961	164.9207
AC027013.	14.41361	15.00586	33.97054	8.314342	13.28982	2.283774	39.93497	36.11505	4.669151	8.646293	6.023774	11.96102	21.58977	45.60529	23.47214	23.9091	5.131881	20.93307	7.170464
AC027018.	1.921815	4.001562	0.999134	5.196464	12.18233	3.425661	3.398721	0	7.003726	6.724895	8.433284	1.840156	11.30893	1.861441	3.912023	0.824452	2.052753	2.7304	0.896308
AC027020.	8.648168	12.00469	16.98527	36.37525	27.68712	2.283774	41.63433	30.55889	18.6766	12.48909	10.84279	44.16376	42.15146	11.16864	4.890029	18.13794	32.84404	7.281067	10.7557
AC027020.	306.5295	1313.513	1231.932	1345.884	903.7077	234.0868	806.3465	909.3585	2559.862	1551.529	1620.395	1025.887	994.1576	295.9691	313.9399	334.7274	425.9461	257.5678	259.9293
AC027031.	0	0	0.999134	0	0	0	0.84968	0	0	0	0	0	1.028084	0	0.978006	0	0	0	0
AC027031.	536.1864	557.2175	572.5036	348.1631	754.1972	797.0371	496.2133	626.9203	1534.983	718.603	813.2095	600.8111	623.0191	608.6911	574.0894	339.6742	408.4977	403.1891	394.3755
AC027088.	3.84363	3.001171	0.999134	9.353635	8.859879	11.41887	3.398721	7.408216	15.17474	7.685594	15.66181	3.680313	4.112337	0	3.912023	1.648904	5.131881	2.7304	0
AC027088.	0	3.001171	3.996535	9.353635	1.107485	3.425661	1.69936	0	0	12.48909	15.66181	2.760235	2.056169	6.515042	8.802052	1.648904	1.026376	0	0
AC027088.	12.4918	31.0121	20.98181	32.21808	8.859879	18.27019	9.346483	13.89041	24.51304	46.11356	57.82823	6.440548	15.42127	13.9608	28.36217	13.19123	21.5539	14.56213	16.13354
AC027097.	277.7023	247.0964	293.7453	261.9018	334.4604	242.08	220.9169	366.7067	321.0041	262.2709	334.9219	284.3042	287.8636	287.5926	443.0366	296.8027	307.9129	374.0648	314.6041
AC027097.	217.1651	251.098	276.76	244.2338	295.6985	251.2151	233.6621	335.2218	296.4911	271.8779	351.7884	297.1853	277.5828	269.9089	295.3578	239.9155	217.5918	303.9846	310.1226
AC027104.	19.21815	19.00742	51.95495	68.59332	58.6967	29.68906	39.93497	9.26027	12.84017	24.01748	7.228529	38.64329	34.95487	20.47585	19.56012	23.08465	35.92317	42.77627	35.85232
AC027117.	165.2761	211.0824	234.7964	162.1297	148.403	74.22266	214.9691	158.3506	70.03726	175.808	153.0039	274.1833	318.7062	178.6983	171.151	176.4327	156.0092	223.8928	172.9874
AC027117.	2.882723	1.00039	0	4.157171	0	1.141887	6.797442	7.408216	0	4.803496	0	0	6.168506	5.584322	2.934017	3.297807	10.26376	11.83173	8.963079
AC027130.	32.67086	11.00429	21.98094	33.25737	25.47215	1.141887	18.69297	12.96438	7.003726	7.685594	28.91412	13.80117	18.50552	10.23792	15.64809	19.78684	3.079129	7.281067	8.066772
AC027139.	0	3.001171	5.994802	2.078586	2.21497	5.709435	0.84968	2.778081	1.167288	0.960699	1.204755	7.360626	2.056169	2.792161	0.978006	0.824452	6.158258	2.7304	12.54831
AC027139.	0	0	0	0	0	0	0	0	0	1.921399	0	0	0	0	0	0	0	0	0
AC027141.	0	0	0	0	0	0	0.84968	0	0	0	0	0.920078	0	0	0	0	0	0	0
AC027176.	10.56998	0	13.98787	19.74656	17.71976	4.567548	19.54265	21.29862	16.34203	7.685594	16.86657	22.08188	20.56169	13.03008	11.73607	18.13794	8.21101	9.101334	28.68185
AC027176.	0	4.001562	0	0	0	0	0	0	0	0	0	0	0	0	0	1.648904	2.052753	0	0.896308
AC027216.	0.960908	0	0	0	0	0	0	0	0	0	0	0	0	1.861441	0	0.824452	1.026376	0	3.585232
AC027228.	6.726353	9.003514	3.996535	7.275049	1.107485	7.993209	11.89552	12.96438	4.669151	12.48909	0	6.440548	11.30893	3.722881	7.824047	3.297807	0	0.910133	3.585232
AC027228.	82.63805	164.064	89.92203	96.65423	64.23413	99.34417	59.47762	110.1972	51.36066	113.3625	42.16642	70.84602	86.35909	96.79491	85.08651	120.37	107.7695	85.55254	52.88217
AC027228.	16.33543	43.01679	78.93156	29.1002	18.82724	4.567548	41.63433	29.63287	29.18219	27.86028	48.1902	29.4425	27.75828	39.09025	22.49413	56.88718	34.89679	35.4952	42.12647
AC027229.	0.960908	0	0	4.157171	0	3.425661	1.69936	0	0	0	0	0	6.168506	0.93072	0	0.824452	8.21101	2.7304	0.896308
AC027237.	0	0	0	0	0	0	1.69936	1.852054	0	0	0	0	1.028084	0	0	0	1.026376	0	0.896308
AC027237.	16.33543	10.0039	33.97054	2.078586	28.79461	6.851322	16.14392	12.03835	9.338302	25.93888	20.48083	11.96102	12.33701	14.89152	16.6261	12.36678	11.29014	18.20267	18.82247
AC027237.	66.30262	66.02577	55.95149	25.98232	16.61227	21.69585	39.08529	17.59451	39.68778	34.58517	42.16642	9.200782	29.81445	54.9125	39.12023	35.45143	25.65941	20.93307	20.61508
AC027237.	10.56998	5.001952	8.992203	41.57171	9.967364	6.851322	3.398721	25.00273	5.836439	19.21399	0	16.56141	4.112337	17.68369	3.912023	45.34485	17.4484	4.550667	12.54831
AC027243.	0	6.002343	8.992203	14.5501	2.21497	18.27019	2.549041	12.96438	4.669151	15.37119	15.66181	14.72125	5.140422	5.584322	7.824047	9.06897	9.237386	1.820267	22.4077
AC027243.	76.8726	112.0437	84.92636	178.7584	43.19191	208.9653	123.2036	149.0904	94.55031	90.30573	72.28529	57.96493	74.02207	98.65635	128.1188	163.2415	139.5872	34.58507	110.2459
AC027250.	0	0	0.999134	0	6.64491	1.141887	0.84968	0	0	0	0	0	0	1.861441	0.978006	0	0	0	0.896308
AC027271.	64.3808	99.03865	105.9082	112.2436	116.2859	159.8642	84.11834	69.45203	128.4017	158.5154	142.1611	74.52634	74.02207	63.28898	86.06451	83.26963	119.0596	55.51814	67.2231
AC027277.	8.648168	9.003514	5.994802	14.5501	1.107485	4.567548	3.398721	25.92876	9.338302	24.97818	50.5997	11.96102	18.50552	11.16864	9.780058	6.595615	8.21101	16.3824	4.48154
AC027279.	112.4262	140.0547	123.8926	65.47544	277.9787	126.7495	10.19616	64.82189	832.2762	291.0919	402.3881	56.12477	70.93782	93.07203	127.1408	73.37621	83.13648	120.1376	34.95601
AC027279.	12.4918	5.001952	5.994802	3.117878	0	3.425661	1.69936	21.29862	12.84017	4.803496	3.614265	1.840156	0	12.09936	6.846041	3.297807	2.052753	0.910133	3.585232

AC027279.	0	0	3.996535	0	0	0	0	5.556162	1.167288	0	0	0	2.056169	5.584322	1.956012	0	0	0	0
AC027279.	0	0	5.994802	5.196464	6.64491	0	0	1.852054	3.501863	6.724895	0	0.920078	5.140422	0.93072	0	6.595615	0	2.7304	0
AC027281.	0	0	0	0	0	0	3.398721	3.704108	4.669151	1.921399	2.40951	0	1.028084	1.861441	3.912023	0.824452	0	0	0.896308
AC027281.	4.804538	4.001562	0	4.157171	4.42994	3.425661	1.69936	0.926027	0	3.842797	9.638039	4.600391	8.224675	0.93072	3.912023	1.648904	1.026376	4.550667	0.896308
AC027287.	2.882723	0	1.998267	0	0	0	0.84968	0	0	0	0	0	0	0	0	0.824452	0	0	0
AC027287.	0	0	5.994802	6.235757	0	0	11.04584	7.408216	0	0	0	0	2.056169	4.653602	0.978006	1.648904	2.052753	0	0
AC027288.	8.648168	23.00898	16.98527	21.82515	18.82724	0	20.39233	1.852054	11.67288	6.724895	0	13.80117	14.39318	32.57521	28.36217	14.84013	5.131881	16.3824	1.792616
AC027288.	9.609075	46.01796	42.96275	1.039293	15.50479	6.851322	4.248401	12.96438	74.70641	5.764196	4.81902	11.04094	7.196591	68.8733	50.8563	28.03136	6.158258	24.5736	1.792616
AC027288.	604.4108	671.262	624.4585	179.7976	172.7676	324.2959	735.8231	347.2601	250.9669	62.44545	75.89956	540.0859	415.3461	603.1068	640.5938	233.3199	168.3257	272.1299	72.60094
AC027290.	2128.41	1973.77	2727.635	1478.914	3212.814	1639.75	3410.616	2637.325	862.6256	2169.259	2392.643	3893.771	4154.489	2080.16	2765.8	1210.295	1127.988	1115.824	1383.003
AC027307.	61.49808	57.02225	32.97141	29.1002	62.01916	60.52001	51.83049	36.11505	65.36811	48.99566	18.07132	51.52438	27.75828	25.12945	20.53812	6.595615	6.158258	27.304	19.71877
AC027307.	485.2583	279.1089	276.76	368.9489	446.3164	324.2959	330.5256	358.3725	850.9528	504.3671	598.7632	384.5927	372.1665	351.8123	335.456	373.4767	351.0207	391.3574	325.3598
AC027307.	210.4387	223.0871	215.8129	221.3694	165.0153	212.391	211.5704	188.9095	156.4166	209.4324	168.6657	222.6589	179.9148	245.7102	215.1613	241.5644	269.937	206.6003	260.8256
AC027308.	0	0	0	0	0	0	0	1.852054	0	0	0	0	0	0	0	0	1.026376	0	0
AC027309.	1.921815	0	1.998267	1.039293	0	1.141887	0	0	9.338302	2.882098	0	0	0	0	0	0.824452	0	0.910133	0.896308
AC027309.	99.93438	94.0367	69.93936	74.82908	85.27634	65.08756	60.3273	53.70957	93.38302	88.38433	84.33284	55.20469	75.05016	76.31906	83.1305	56.06272	89.29473	81.00187	49.29694
AC027309.	0	4.001562	0	0	0	6.851322	0.84968	0	0	1.921399	0	0.920078	0	0	2.934017	0	1.026376	3.640534	0.896308
AC027312.	1.921815	5.001952	11.9896	3.117878	9.967364	4.567548	3.398721	7.408216	11.67288	8.646293	12.04755	0.920078	4.112337	3.722881	10.75806	3.297807	6.158258	6.370934	7.170464
AC027313.	4.804538	1.00039	0	0	0	0	0	0	0.926027	0	0	0	1.840156	1.028084	0.93072	0.978006	4.122259	2.052753	2.7304	0.896308
AC027328.	0	0	0	2.078586	0	0	0	0	0	0	0	0.920078	2.056169	0	0	0	0	0	0
AC027335.	0	0	0	0	0	0	3.398721	0	0	0	0	1.840156	0	3.722881	0	0	0	0	0.896308
AC027338.	41.31902	26.01015	49.95668	61.31827	38.76197	39.96605	102.8113	17.59451	8.171014	10.56769	22.89034	44.16376	57.57272	4.653602	15.64809	38.74924	19.50115	26.39387	35.85232
AC027338.	0	0	0	1.039293	0	0	0.84968	0	0	0	0	1.840156	0	0	0	0	0	0	0
AC027343.	0	3.001171	0	0	0	1.141887	0	0	0	0	0	0	0	0	0	0	0	0	0
AC027348.	45.16265	51.01991	53.95322	21.82515	68.66407	50.24303	30.58849	68.526	107.3905	76.85594	90.35662	69.92595	39.06721	66.08114	69.43841	25.55801	55.42432	50.05734	34.0597
AC027348.	0	0	0	0	0	0	0	0	0	0	4.81902	0.920078	1.028084	0	0	0	0	0	0
AC027369.	0	0	0	0	0	1.141887	0.84968	0	0	0	0	0	0	0	0	0	4.105505	0	0
AC027369.	0	0	0	1.039293	0	1.141887	0	0.926027	0	0	0	0	0	0.93072	0.978006	0	0	3.640534	0.896308
AC027373.	101.8562	79.03084	71.93762	81.06484	56.48173	133.6008	74.77186	80.56435	156.4166	121.0481	96.38039	101.2086	87.38717	76.31906	98.77859	85.74299	91.34749	40.956	46.60801
AC027419.	0	0	0	0	0	0	0.84968	0	0	0.960699	0	0	0	0	0	0	1.026376	0	0
AC027449.	0.960908	8.003124	0	0	11.07485	0	4.248401	0	7.003726	0	0	3.680313	0	0	0.978006	0	0	0	0
AC027455.	0.960908	0	0.999134	1.039293	2.21497	0	5.947762	3.704108	0	7.685594	12.04755	2.760235	5.140422	0	0	0	1.026376	0.910133	0
AC027458.	0	0	0	0	0	6.851322	0	0	0	0	0	0	0	0	0	0	0	0	0
AC027514.	38.4363	31.0121	36.96795	29.1002	26.57964	39.96605	36.53625	32.41095	0	31.70308	22.89034	33.12282	20.56169	10.23792	29.34017	23.08465	2.052753	2.7304	10.7557
AC027514.	80.71623	62.02421	141.877	48.84676	124.0383	61.6619	110.4584	226.8766	1.167288	57.64196	40.96167	126.0507	97.66802	124.7165	125.1847	68.4295	23.60665	115.5869	103.9717
AC027544.	18.25724	22.00859	22.98007	33.25737	31.00958	5.709435	28.88913	25.92876	1.167288	14.41049	20.48083	40.48344	14.39318	15.82225	13.69208	15.66458	12.31652	18.20267	25.09662
AC027544.	40.35812	40.01562	95.91683	48.84676	81.95388	44.53359	118.1056	79.63833	1.167288	35.54587	27.70936	104.8889	85.331	67.01186	70.41642	36.27588	14.36927	46.4168	62.74156
AC027544.	32.67086	6.002343	11.9896	1.039293	1.107485	0	4.248401	21.29862	0	18.25329	8.433284	2.760235	0	16.75297	23.47214	0	0	3.640534	18.82247
AC027559.	214.2824	223.0871	346.6994	201.6228	352.1802	189.5532	271.048	164.8328	249.7996	274.76	240.951	280.6239	308.4253	296.8998	306.1158	119.5455	135.4817	288.5123	264.4108
AC027575.	3.84363	0	0.999134	8.314342	0	4.567548	0.84968	1.852054	0	0	0	3.680313	0	3.722881	0	0	0	0.910133	0.896308
AC027575.	78.79442	100.039	158.8623	50.92535	147.2955	103.9117	67.12474	73.15614	224.1192	119.1267	225.2892	87.40743	97.66802	83.76483	63.57038	111.301	153.9564	105.5755	75.28987
AC027575.	1.921815	2.000781	3.996535	2.078586	7.752394	0	2.549041	0.926027	0	7.685594	14.45706	5.520469	9.252759	8.376483	2.934017	1.648904	0	3.640534	0
AC027575.	100.8953	102.0398	145.8735	80.02554	184.95	122.1819	47.58209	150.9424	409.718	194.0612	167.4609	88.32751	122.342	98.65635	101.7126	78.32292	97.50574	50.05734	48.40063
AC027601.	123.9571	163.0636	184.8397	107.0472	202.6697	205.5397	130.8508	199.0958	379.3685	330.4805	599.9679	235.54	169.6339	201.0356	217.1173	143.4546	244.2775	266.6691	293.0927
AC027601.	78.79442	38.01484	27.97574	12.47151	22.1497	19.41208	9.346483	39.81916	63.03354	84.54153	125.2945	46.00391	29.81445	31.64449	36.18622	30.50472	46.18693	22.75334	62.74156
AC027601.	19.21815	33.01288	18.98354	2.078586	26.57964	45.67548	13.59488	26.85478	117.8961	43.23147	103.6089	34.96297	17.47743	28.85233	7.824047	13.19123	27.71216	26.39387	43.91909
AC027601.	15.37452	22.00859	7.993069	8.314342	8.859879	9.135096	3.398721	18.52054	0	9.606993	48.1902	18.40156	15.42127	21.40657	13.69208	8.244518	13.34289	2.7304	12.54831
AC027607.	65.34171	112.0437	131.8856	107.0472	83.06137	78.7902	113.8572	107.4191	87.54658	145.0656	142.1611	76.36649	121.314	135.8852	98.77859	72.55176	66.71446	114.6768	138.0314
AC027612.	58.61536	55.02147	53.95322	120.558	132.8982	29.68906	135.9488	66.67395	67.70269	43.23147	12.04755	115.9299	100.7523	47.46674	34.2302	103.8809	74.92547	50.05734	69.01571
AC027612.	15.37452	84.0328	26.97661	25.98232	69.77155	99.34417	104.5107	54.6356	31.51677	40.34937	12.04755	68.08579	71.96591	20.47585	39.12023	106.3543	107.7695	51.8776	66.32679
AC027612.	0	0	0	0	0	0	0	0	7.003726	0	0	0	0	0	0.978006	0	2.052753	0	0.896308
AC027613.	0.960908	0	0	1.039293	0	0	0	0	0	0	0	0	0	0	0	0	0	0	0
AC027627.	0	0	0	1.039293	0	0	0	0	0	3.842797	0	0	0	0	3.912023	1.648904	0	1.820267	3.585232
AC027644.	218.126	135.0527	196.8293	178.7584	201.5623	95.91851	214.9691	140.7561	73.53913	108.559	102.4042	132.4913	172.7182	200.1049	219.0733	227.5487	237.0929	141.9808	179.2616
AC027644.	26.90541	7.002733	22.98007	17.66798	23.25718	26.2634	30.58849	13.89041	11.67288	20.17468	4.81902	24.84211	41.12337	29.78305	35.20821	28.85581	19.50115	14.56213	13.44462
AC027644.	11.53089	26.01015	11.9896	13.51081	8.859879	5.709435	18.69297	12.03835	15.17474	10.56769	15.66181	18.40156	24.67402	9.307203	12.71408	9.893422	9.237386	13.652	11.652
AC027667.	0.960908	1.00039	3.996535	2.078586	0	0	0.84968	0	0	3.842797	0	0	2.056169	0	0	0	0	0.910133	0.896308
AC027682.	14.41361	25.00976	33.97054	19.74656	23.25718	11.41887	37.38593	36.11505	40.85507	24.97818	28.91412	31.28266	22.61786	13.9608	28.36217	21.43575	12.31652	20.93307	27.78555
AC027682.	47.08447	82.03202	84.92636	39.49313	84.16885	123.3238	35.68657	74.08216	148.2455	73.01314	154.2086	57.04485	42.15146	55.84322	50.8563	59.36053	32.84404	70.99041	49.29694
AC027682.	11.53089	13.00508	14.987	2.078586	22.1497	10.27698	1.69936	18.52054	26.84762	9.606993	19.27608	11.04094	8.224675	12.09936	5.868035	23.9091	9.237386	4.550667	7.170464
AC027682.	136.4489	189.0738	121.8943	65.47544	146.188	235.2287	80.71962	132.4219	340.848	320.8736	430.0975	129.731	135.7071	150.7767	118.3387	88.21635	112.9014	123.7781	155.9576
AC027682.	204.6733	218.0851	285.7522	142.3831	242.5392	347.1336	130.0011	305.5889	49.02608	282.4456	231.3129	210.6979	166.5497	217.7886	196.5792	164.8904	171.4048	239.3651	222.2844

AC027698.	1.921815	6.002343	0.999134	7.275049	5.537425	30.83095	3.398721	11.11232	1.167288	10.56769	0	5.520469	3.084253	15.82225	13.69208	1.648904	2.052753	5.4608	7.170464
AC027701.	0	0	0	0	1.107485	4.567548	0.84968	0	0	0	0	0	1.028084	0	0	0	1.026376	0	0
AC027702.	112.4262	85.03319	78.93156	111.2043	97.45867	94.77662	127.452	121.3095	63.03354	49.95636	30.11887	96.60821	102.8084	99.58707	96.82258	133.5612	139.5872	125.5984	125.4831
AC027763.	1.921815	7.002733	2.997401	9.353635	3.322455	0	10.19616	11.11232	0	11.52839	9.638039	7.360626	8.224675	22.33729	4.890029	15.66458	10.26376	0.910133	11.652
AC027763.	3.84363	5.001952	7.993069	8.314342	8.859879	4.567548	4.248401	4.630135	7.003726	3.842797	4.81902	12.8811	19.5336	12.09936	4.890029	5.771163	1.026376	3.640534	2.688924
AC027779.	0.960908	0	0	0	0	0	0	0	0	0.960699	0	0	0	0	0	0	1.026376	0	0
AC027796.	38.4363	42.0164	95.91683	21.82515	68.66407	52.5268	62.87634	67.59997	17.50932	46.11356	31.32363	62.56532	87.38717	30.71377	39.12023	35.45143	24.63303	31.85467	31.37078
AC027796.	24.02269	26.01015	97.9151	59.23969	75.30897	34.25661	46.73241	54.6356	0	37.46727	13.2523	55.20469	58.60081	42.81313	44.01026	33.80253	42.08143	36.40534	34.95601
AC027796.	29.78813	6.002343	35.96881	21.82515	35.43952	58.23624	36.53625	3.704108	3.501863	48.99566	14.45706	25.76219	46.2638	21.40657	19.56012	37.10033	48.23968	41.86614	26.88924
AC027796.	623.629	537.2097	750.3494	392.8527	728.7251	680.5647	675.4958	678.7778	674.6923	822.3586	1074.641	702.0197	704.2378	611.4832	547.6833	387.4924	467.0012	669.8582	665.0605
AC027796.	80.71623	51.01991	78.93156	133.0295	63.12664	42.24982	100.2623	52.78354	66.5354	90.30573	31.32363	58.88501	67.85357	89.34915	67.4824	61.83389	52.34519	100.1147	102.1791
AC027801.	63.4199	50.01952	114.9004	85.22201	77.52394	47.95925	49.28145	68.526	322.1714	181.5722	130.1135	53.36454	91.49951	71.66546	67.4824	79.97183	84.16285	81.00187	71.70464
AC027801.	1.921815	1.00039	0.999134	0	0	0	2.549041	0	0	0	0	1.840156	1.028084	0	0	0	1.026376	0	0.896308
AC027801.	0.960908	1.00039	1.998267	1.039293	2.21497	0	5.947762	0.926027	2.334575	0.960699	0	3.680313	0	0	0.978006	0	1.026376	0	0.896308
AC027801.	0	4.001562	6.993936	1.039293	7.752394	2.283774	7.647122	3.704108	19.84389	0	0	2.760235	3.084253	0.93072	7.824047	0	0	0	0.896308
AC027801.	5.765445	2.000781	21.98094	5.196464	5.537425	4.567548	7.647122	4.630135	1.167288	1.921399	12.04755	11.04094	10.28084	3.722881	3.912023	9.06897	2.052753	8.191201	8.963079
AC027804.	0	0	0	0	1.107485	0	0.84968	0	0	0	0	0	0	0	0	0	0	0.910133	0.896308
AC027807.	7.68726	14.00547	9.991337	39.49313	16.61227	7.993209	12.7452	16.66849	11.67288	7.685594	14.45706	23.00196	15.42127	5.584322	19.56012	22.2602	19.50115	13.652	28.68185
AC027807.	12.4918	8.003124	17.98441	21.82515	3.322455	13.70264	21.24201	22.22465	17.50932	15.37119	4.81902	15.64133	16.44935	22.33729	22.49413	5.771163	7.184634	12.74187	2.688924
AC027808.	3.84363	14.00547	36.96795	11.43222	34.33203	6.851322	33.13753	24.0767	3.501863	22.09608	9.638039	49.68422	29.81445	16.75297	32.27419	12.36678	7.184634	19.1128	14.34093
AC032011.	63.4199	64.02499	109.9047	43.6503	86.38382	78.7902	89.21643	62.96984	46.69151	56.68126	42.16642	97.52829	62.71315	70.73474	61.61437	42.04704	67.74083	91.01334	77.08248
AC032019.	0	0	0	0	0	0	0	0	0	0	0	2.760235	0	0	0	0	0	0	0
AC032044.	55.73264	122.0476	127.8891	114.3222	50.94431	117.6144	96.01387	83.34243	1.167288	102.7948	86.74235	96.60821	90.47142	74.45762	49.8783	216.0064	113.9278	174.7456	116.52
AC032044.	25.9445	27.01054	65.94282	25.98232	32.11706	27.40529	50.13113	32.41095	7.003726	17.29259	25.29985	46.92399	33.92678	27.92161	44.98827	16.48904	23.60665	32.7648	13.44462
AC034102.	31.70995	13.00508	15.98614	22.86444	14.3973	5.709435	22.09169	30.55889	9.338302	5.764196	7.228529	14.72125	10.28084	18.61441	6.846041	37.10033	30.79129	24.5736	20.61508
AC034102.	9.609075	16.00625	10.99047	8.314342	13.28982	2.283774	17.84329	6.482189	8.171014	7.685594	0	6.440548	9.252759	5.584322	4.890029	5.771163	6.158258	8.191201	7.170464
AC034102.	9.609075	13.00508	11.9896	4.157171	19.93473	1.141887	8.496802	17.59451	16.34203	18.25329	19.27608	5.520469	7.196591	14.89152	15.64809	3.297807	8.21101	15.47227	25.09662
AC034102.	85.52077	79.03084	79.93069	83.14342	73.094	45.67548	109.6088	59.26573	31.51677	77.81664	72.28529	82.80704	90.47142	94.93347	46.94428	70.07841	76.97822	78.27147	166.7133
AC034102.	5.765445	3.001171	0.999134	5.196464	0	0	5.947762	0.926027	0	1.921399	12.04755	2.760235	2.056169	4.653602	0.978006	2.473355	1.026376	0	6.274156
AC034102.	25.9445	26.01015	71.93762	44.68959	18.82724	42.24982	52.68017	38.89314	30.34948	49.95636	30.11887	47.84407	42.15146	15.82225	77.26246	44.5204	41.05505	14.56213	25.99293
AC034105.	0	0	0	0	0	0	0	0	0	0	0	0	0	0.93072	0	0	0	2.7304	0
AC034105.	3.84363	0	0	0	0	0	0.84968	0	0	0	0	0	0	0.93072	3.912023	0	0	0.910133	0
AC034105.	5.765445	1.00039	0.999134	8.314342	4.42994	3.425661	4.248401	7.408216	9.338302	10.56769	0	0.920078	5.140422	0.93072	0	0.824452	3.079129	5.4608	0.896308
AC034105.	0	0	0	1.039293	0	0	0	0	0	0	0	0.920078	0	0	0	0	0	0	0
AC034111.	12.4918	20.00781	59.94802	3.117878	11.07485	9.135096	7.647122	40.74519	0	41.31007	19.27608	6.440548	14.39318	6.515042	21.51613	10.71787	20.52753	20.93307	30.47447
AC034154.	0	2.000781	0.999134	1.039293	4.42994	2.283774	6.797442	0	0	3.842797	1.204755	0	1.028084	3.722881	0	0.824452	1.026376	0	1.792616
AC034187.	0	0	0	4.157171	0	0	0	0	0	0	0	0.920078	0	0	0	0	0	0.910133	0
AC034195.	13.45271	21.0082	24.97834	3.117878	8.859879	12.56076	15.29424	15.74246	9.338302	15.37119	4.81902	12.8811	12.33701	11.16864	29.34017	8.244518	14.36927	3.640534	1.792616
AC034198.	37.47539	41.01601	92.91943	86.2613	56.48173	62.80379	60.3273	68.526	91.04844	151.7905	93.97088	117.77	125.4263	40.02097	69.43841	68.4295	57.47707	54.608	43.02278
AC034199.	20.17906	12.00469	20.98181	16.62868	8.859879	20.55397	21.24201	6.482189	15.17474	19.21399	2.40951	17.48149	11.30893	5.584322	0	18.13794	18.47477	35.4952	18.82247
AC034205.	1.921815	7.002733	0	0	0	1.141887	1.69936	2.778081	0	0	0	0	0	1.861441	1.956012	1.648904	1.026376	0	0
AC034206.	2.882723	9.003514	2.997401	0	0	0	10.19616	0	7.003726	0	2.40951	0.920078	2.056169	3.722881	1.956012	2.473355	2.052753	5.4608	4.48154
AC034207.	1.921815	1.00039	0.999134	2.078586	0	0	0.84968	0.926027	1.167288	0.960699	0	0	2.056169	0	0	0	0	0	0
AC034213.	0	0	0	0	6.64491	0	0.84968	0	0	0	0	0	1.028084	0	0	0	0	0	0
AC034223.	0	0	0	0	0	0	0	0	0	0	0	0	3.084253	0	0	0	0	0	1.792616
AC034223.	0	4.001562	0	1.039293	0	2.283774	0.84968	0	0	0	0	1.840156	1.028084	0	0	0.824452	0	0	0
AC034228.	10.56998	15.00586	28.97488	5.196464	28.79461	6.851322	28.88913	28.70684	42.02236	16.33189	30.11887	6.440548	9.252759	11.16864	10.75806	0.824452	0	10.9216	18.82247
AC034228.	0	0	0	0	0	3.425661	2.549041	0	0	0	0	0	0	0.93072	0	0	0	0	2.688924
AC034228.	1.921815	0	0	4.157171	4.42994	0	3.398721	3.704108	0	4.803496	0	0.920078	0	1.861441	2.934017	0.824452	0	0	1.792616
AC034229.	0	10.0039	0	1.039293	1.107485	13.70264	10.19616	0	3.501863	0.960699	2.40951	5.520469	5.140422	3.722881	7.824047	3.297807	1.026376	5.4608	4.48154
AC034229.	0	2.000781	0	0	0	0	0	0	0	0	0	0	0	0	0.978006	0	0	0	0
AC034229.	37.47539	16.00625	28.97488	17.66798	24.36467	27.40529	21.24201	37.04108	12.84017	40.34937	31.32363	23.00196	24.67402	26.06017	35.20821	25.55801	8.21101	35.4952	16.13354
AC034229.	0	1.00039	5.994802	0	0	0	2.549041	1.852054	0	1.921399	0	0	4.112337	0.93072	0	4.122259	1.026376	0	2.688924
AC034229.	1.921815	0	2.997401	0	1.107485	0	0	0	3.501863	0	0	0	0	0.93072	0	0.824452	0	0.910133	0.896308
AC034231.	133.5661	57.02225	74.93502	36.37525	111.856	47.95925	38.23561	93.52873	95.71759	71.09174	95.17564	105.809	89.44334	86.55699	86.06451	62.65834	75.95184	110.1261	105.7643
AC034232.	0	0	0.999134	0	0	0	5.098081	0	0	0	0	0	0	0	0	3.297807	0	0	0
AC034232.	0	0	0	1.039293	0	0	5.098081	0	14.00745	0	0	0	5.140422	1.861441	0	0	0	0.910133	1.792616
AC034234.	0	0	2.997401	1.039293	1.107485	0	0	0	0	0.960699	0	0.920078	0	0	0	0	0	0	0
AC034236.	65.34171	72.02811	51.95495	31.17878	87.49131	78.7902	29.73881	62.96984	113.2269	88.38433	80.71858	56.12477	37.01104	71.66546	44.01026	64.30724	59.52982	54.608	30.47447
AC034236.	210.4387	287.1121	415.6396	194.3477	411.9844	356.2687	305.0352	272.2519	930.3283	673.4502	712.0101	381.8325	388.6159	425.3392	347.1921	204.4641	226.8292	402.279	388.1013
AC034236.	18.25724	8.003124	9.991337	10.39293	11.07485	3.425661	22.09169	17.59451	73.53913	26.89958	24.0951	10.12086	14.39318	16.75297	19.56012	12.36678	14.36927	9.101334	17.02985
AC034238.	0.960908	0	6.993936	7.275049	4.42994	5.709435	1.69936	30.55889	0	0	1.204755	20.24172	14.39318	0.93072	0	10.71787	6.158258	3.640534	4.48154

AC034243.	27.86632	11.00429	2.997401	0	0	0	0.84968	8.334243	18.6766	22.09608	26.50461	2.760235	1.028084	5.584322	10.75806	1.648904	6.158258	30.0344	12.54831
AC034244.	0	0	0	0	0	0	0	0	0	0	2.40951	0	0	0.93072	0	0	0	0	0.896308
AC034268.	0	0	0	0	0	2.283774	0	0	0	0	0	0.920078	1.028084	0	0.978006	0	0	0.910133	2.688924
AC035139.	6.726353	4.001562	11.9896	4.157171	18.82724	4.567548	6.797442	23.15068	17.50932	1.921399	10.84279	18.40156	31.87062	10.23792	15.64809	4.946711	11.29014	6.370934	15.23724
AC035140.	2.882723	12.00469	6.993936	19.74656	7.752394	3.425661	8.496802	5.556162	0	0.960699	9.638039	3.680313	3.084253	7.445762	15.64809	5.771163	2.052753	9.101334	7.170464
AC036103.	28.82723	37.01445	64.94369	29.1002	39.86946	17.12831	62.02666	49.07943	24.51304	54.75986	10.84279	87.40743	66.82548	37.22881	50.8563	47.81821	37.97592	65.52961	53.77848
AC036108.	4.804538	3.001171	21.98094	8.314342	14.3973	17.12831	24.64073	20.37259	5.836439	12.48909	1.204755	11.04094	14.39318	12.09936	11.73607	9.893422	16.42202	18.20267	12.54831
AC036108.	37.47539	30.01171	48.95755	67.55403	55.37425	22.83774	36.53625	62.04381	38.5205	25.93888	26.50461	75.44642	79.1625	40.95169	59.65836	41.22259	36.94955	59.15867	60.05263
AC036108.	2034.241	2897.131	2663.69	2754.126	1893.799	2708.556	2374.007	2065.04	1320.202	2432.491	1642.081	1577.934	1858.777	2145.31	2335.478	2182.324	2329.874	1323.334	2029.241
AC036108.	0	0	18.98354	16.62868	0	20.55397	6.797442	5.556162	14.00745	19.21399	14.45706	1.840156	2.056169	9.307203	18.58211	6.595615	5.131881	9.101334	5.377848
AC036111.	24.9836	4.001562	6.993936	8.314342	5.537425	0	14.44456	9.26027	3.501863	15.37119	7.228529	13.80117	11.30893	5.584322	4.890029	0.824452	7.184634	6.370934	8.066772
AC036111.	0	0	0.999134	0	0	0	0	0	0	0	0	3.680313	0	0	0.978006	0	0	0	0
AC036111.	0	4.001562	0	0	0	0	0.84968	0	0	0	0	0.920078	1.028084	0	0	0	0	0	0
AC036164.	0	1.00039	0	0	0	0	0	0	0	0.960699	0	0	0	0	0	0	0	0	0
AC036176.	55.73264	66.02577	58.94889	51.96464	68.66407	97.0604	56.92858	78.7123	199.6062	114.3232	163.8467	77.28657	57.57272	75.38834	72.37243	66.7806	93.40024	51.8776	66.32679
AC036176.	3.84363	0	9.991337	0	4.42994	6.851322	1.69936	4.630135	1.167288	2.882098	1.204755	6.440548	7.196591	2.792161	3.912023	0	5.131881	0.910133	6.274156
AC036214.	74.95079	40.01562	37.96708	34.29666	48.72934	29.68906	45.88273	64.82189	51.36066	41.31007	81.92333	53.36454	50.37613	37.22881	48.90029	50.29156	34.89679	42.77627	60.05263
AC036214.	107.6216	108.0422	139.8787	109.1257	68.66407	114.1887	135.9488	123.1616	151.7474	80.69874	72.28529	85.56728	101.7804	101.4485	145.7229	105.5298	115.9805	149.2619	103.9717
AC037198.	40.35812	113.0441	243.7886	41.57171	52.05179	59.37812	120.6546	184.2794	24.51304	180.6115	175.8942	168.3743	122.342	135.8852	215.1613	154.9969	79.03097	222.0726	100.3865
AC037441.	13.45271	2.000781	7.993069	5.196464	8.859879	2.283774	13.59488	8.334243	0	0.960699	4.81902	0.920078	5.140422	0.93072	5.868035	4.946711	9.237386	5.4608	6.274156
AC037441.	0	0	0	4.157171	0	0	0	11.11232	0	0	0	0	1.028084	0	0	0.824452	0	0	0
AC037450.	8.648168	2.000781	2.997401	1.039293	5.537425	1.141887	9.346483	1.852054	1.167288	1.921399	1.204755	6.440548	7.196591	1.861441	3.912023	4.122259	4.105505	4.550667	4.48154
AC037459.	0	0	0	0	0	0	0	0	0	0	0	0	0	0	0	0	0	0	1.792616
AC037459.	565.0136	609.2378	650.436	825.1985	791.8517	703.4024	802.9478	561.1724	545.1234	511.092	395.1596	565.8481	650.7774	533.3027	620.0557	637.3013	611.7202	640.7339	677.6088
AC037459.	699.5407	620.2421	515.553	434.4244	829.5062	477.3088	528.5011	634.3285	619.8298	629.258	851.7617	549.2867	604.5136	543.5407	586.8035	509.5112	643.5379	778.1641	833.5664
AC037471.	0.960908	0	0	1.039293	13.28982	0	0	0	0	0	3.614265	0.920078	2.056169	10.23792	26.40616	0	0	0	0
AC037471.	18.25724	17.00664	8.992203	2.078586	0	3.425661	16.9936	0.926027	0	0	0	8.280704	7.196591	18.61441	14.67009	1.648904	0	0	3.585232
AC037479.	0.960908	1.00039	0	14.5501	3.322455	2.283774	5.947762	5.556162	0	0	0	13.80117	10.28084	0	2.934017	0	6.158258	3.640534	1.792616
AC037486.	0	2.000781	0.999134	0	0	0	0.84968	0	0	0	0	0	2.056169	0	0.978006	0	0	7.281067	1.792616
AC037487.	94.16894	60.02343	97.9151	91.45776	48.72934	33.11472	104.5107	66.67395	122.5652	129.6944	67.46627	80.04681	68.88165	67.94258	117.3607	41.22259	44.13418	114.6768	68.1194
AC037487.	24.02269	18.00703	16.98527	7.275049	9.967364	19.41208	21.24201	18.52054	23.34575	12.48909	27.70936	9.200782	15.42127	12.09936	20.53812	9.06897	17.4484	13.652	8.066772
AC037487.	6.726353	4.001562	8.992203	11.43222	4.42994	7.993209	10.19616	1.852054	11.67288	8.646293	12.04755	6.440548	12.33701	9.307203	2.934017	6.595615	1.026376	5.4608	8.066772
AC037487.	0	0	3.996535	1.039293	0	1.141887	3.398721	1.852054	1.167288	0	1.204755	0	2.056169	1.861441	0.978006	4.122259	1.026376	5.4608	2.688924
AC039056.	49.00628	41.01601	54.95235	21.82515	67.55658	22.83774	51.83049	16.66849	24.51304	73.01314	77.10431	74.52634	66.82548	53.98178	74.32844	11.54233	6.158258	26.39387	36.74863
AC040160.	707.2279	653.255	504.5625	715.0334	496.1532	726.2401	569.2858	675.9997	857.9565	702.2712	666.2294	718.5811	810.1305	495.1432	564.3094	558.9783	660.9863	506.0342	580.8076
AC040160.	24.9836	51.01991	70.93849	31.17878	107.426	45.67548	45.88273	52.78354	245.1304	56.68126	89.15186	43.24368	33.92678	39.09025	48.90029	61.83389	45.16056	65.52961	95.00864
AC040162.	407.4248	313.1222	295.7436	339.8487	551.5275	542.3963	418.8924	456.5313	639.6737	564.8912	634.9058	513.4037	418.4303	497.9354	483.1349	439.4328	368.4691	539.7091	559.2962
AC040162.	0	0	0	0	0	1.141887	3.398721	0	12.84017	0	0	0.920078	1.028084	0	0	5.771163	0	0	0
AC040162.	143.1752	118.0461	147.8718	97.69352	225.9269	181.56	119.8049	113.9013	396.8778	278.6028	255.408	230.9396	242.6279	118.2015	142.7889	128.6145	130.3498	256.6576	239.3142
AC040162.	0	0	0	0	0	0	0	0	2.334575	0	0	0	0	0	0	0	1.026376	0	0
AC040168.	1.921815	0	0.999134	0	8.859879	0	0.84968	11.11232	0	0	0	6.440548	6.168506	0	0	0.824452	1.026376	0.910133	0
AC040169.	0.960908	24.00937	3.996535	7.275049	5.537425	2.283774	1.69936	0.926027	9.338302	4.803496	19.27608	11.04094	14.39318	3.722881	1.956012	7.420066	5.131881	0.910133	8.066772
AC040169.	22.10087	61.02382	31.97228	32.21808	36.547	58.23624	43.33369	36.11505	35.01863	58.60265	37.3474	36.80313	39.06721	26.99089	15.64809	27.20691	49.26606	27.304	33.16339
AC040169.	12.4918	12.00469	30.97314	4.157171	11.07485	15.98642	28.03945	9.26027	19.84389	48.03496	42.16642	32.20274	10.28084	11.16864	21.51613	15.66458	11.29014	17.29253	13.44462
AC040170.	0	9.003514	0.999134	0	0	1.141887	0.84968	0	0	0	0	0	0	0	0	0	0	0.910133	0.896308
AC040173.	22.10087	30.01171	16.98527	11.43222	17.71976	36.54038	34.83689	36.11505	32.68406	8.646293	4.81902	6.440548	6.168506	30.71377	20.53812	9.893422	7.184634	8.191201	5.377848
AC040174.	130.6834	302.1179	124.8917	122.6365	87.49131	31.97284	190.3284	76.86024	32.68406	49.95636	32.52838	81.88696	82.24675	209.4121	192.6671	172.3104	183.7213	106.4856	170.2985
AC040174.	49.96719	125.0488	72.93676	69.63262	55.37425	117.6144	29.73881	79.63833	85.212	79.73804	32.52838	48.76415	57.57272	93.07203	74.32844	93.16306	72.87271	57.33841	55.57109
AC040896.	0	4.001562	0	4.157171	4.42994	9.135096	0.84968	4.630135	15.17474	4.803496	0	2.760235	1.028084	3.722881	1.956012	2.473355	2.052753	0.910133	1.792616
AC040904.	0	1.00039	0.999134	1.039293	2.21497	3.425661	4.248401	3.704108	4.669151	0	0	0.920078	0	0.93072	0.978006	1.648904	3.079129	5.4608	0
AC040914.	2.882723	1.00039	10.99047	7.275049	5.537425	3.425661	0.84968	12.03835	0	1.921399	0	1.840156	8.224675	2.792161	1.956012	8.244518	14.36927	5.4608	6.274156
AC040918.	1.921815	5.001952	5.994802	0	4.42994	4.567548	3.398721	13.89041	15.17474	0.960699	1.204755	5.520469	1.028084	0	8.802052	0.824452	5.131881	0	0
AC040926.	0.960908	5.001952	2.997401	1.039293	0	0	3.398721	0	0	2.882098	0	0	1.028084	0	0	0	0	0.910133	0
AC040934.	28.82723	14.00547	17.98441	5.196464	12.18233	17.12831	11.04584	36.11505	3.501863	10.56769	12.04755	19.32164	33.92678	4.653602	21.51613	15.66458	14.36927	24.5736	17.92616
AC040936.	0.960908	0	0	1.039293	1.107485	1.141887	1.69936	0.926027	0	5.764196	0	0	0	0	4.890029	0.824452	1.026376	0	0
AC040958.	0	0	0	0	0	0	1.69936	0	0	3.842797	0	0	0	0	0	0	0	0	0
AC040963.	0	0	0	0	0	6.851322	0	0	0	3.842797	0	0	0	0	0	0	0	0	0
AC040970.	145.097	117.0457	164.8571	136.1474	147.2955	196.4046	72.22282	178.7232	135.4054	102.7948	130.1135	163.7739	199.4484	108.8943	180.9311	140.9813	91.34749	174.7456	141.6167
AC040975.	0	0	7.993069	0	1.107485	0	3.398721	10.1863	0	0	0	12.8811	5.140422	2.792161	0	4.946711	2.052753	0.910133	3.585232
AC040977.	196.0251	138.0539	189.8354	94.57564	111.856	260.3502	84.96802	157.4246	511.272	243.0569	283.1174	229.0995	238.5156	113.5479	137.8988	131.9123	128.297	169.2848	111.1422
AC040978.	0	0	3.996535	0	1.107485	2.283774	2.549041	5.556162	0	3.842797	1.204755	0.920078	3.084253	0.93072	13.69208	0.824452	1.026376	1.820267	3.585232
AC041039.	0	0	1.998267	0	0	0	0	0	0	0	0	0	0	0	0	0	0	0	0

AC041040.	6.726353	6.002343	8.992203	3.117878	14.3973	13.70264	0.84968	0	29.18219	3.842797	4.81902	4.600391	3.084253	4.653602	4.890029	2.473355	10.26376	4.550667	3.585232
AC041040.	9.609075	5.001952	17.98441	5.196464	19.93473	1.141887	5.947762	3.704108	28.01491	1.921399	9.638039	27.60235	10.28084	24.19873	4.890029	19.78684	5.131881	25.48374	18.82247
AC044781.	62.45899	57.02225	62.94542	63.39686	39.86946	58.23624	49.28145	87.97257	57.1971	78.77734	50.5997	52.44446	75.05016	35.36737	62.59237	45.34485	33.87042	29.12427	39.43755
AC044787.	28.82723	31.0121	16.98527	23.90373	25.47215	11.41887	5.098081	23.15068	61.86625	29.78168	16.86657	30.36258	20.56169	23.26801	28.36217	29.68027	19.50115	30.94454	11.652
AC044797.	233.5005	396.1546	84.92636	0	198.2398	121.04	0.84968	133.3479	299.9929	0.960699	0	0	4.112337	375.0803	440.1026	269.5957	245.3039	0.910133	0
AC044802.	98.97348	87.03397	70.93849	153.8153	78.63143	74.22266	148.694	106.4931	43.18965	82.62014	110.8374	121.4503	110.005	63.28898	94.86656	112.9499	123.1652	71.90054	113.8311
AC044802.	18.25724	12.00469	11.9896	0	11.07485	18.27019	12.7452	12.03835	18.6766	24.97818	31.32363	8.280704	6.168506	11.16864	11.73607	15.66458	19.50115	9.101334	10.7557
AC044810.	0	2.000781	1.998267	0	2.21497	0	5.947762	0.926027	0	3.842797	0	9.200782	4.112337	1.861441	2.934017	2.473355	2.052753	2.7304	7.170464
AC044839.	0.960908	2.000781	0	3.117878	8.859879	0	5.098081	0	7.003726	0	0	3.680313	0	0	0	2.473355	3.079129	0	1.792616
AC044839.	1.921815	1.00039	0	0	0	0	0	0	0	0	0	0.920078	0	0	0	0	0	0	0
AC044839.	0	0	0.999134	0	0	0	0	1.852054	0	0	0	0	0	0	0	0	0	0	0
AC044839.	0	0	0	3.117878	0	2.283774	0.84968	0.926027	0	0	0	0.920078	0	0	0.978006	0	3.079129	0.910133	1.792616
AC044840.	4.804538	3.001171	9.991337	6.235757	28.79461	5.709435	6.797442	4.630135	35.01863	4.803496	15.66181	2.760235	10.28084	10.23792	7.824047	6.595615	11.29014	0.910133	6.274156
AC044849.	0.960908	2.000781	0	5.196464	9.967364	1.141887	0.84968	2.778081	1.167288	0.960699	1.204755	0.920078	2.056169	0.93072	1.956012	3.297807	2.052753	6.370934	1.792616
AC044849.	1.921815	9.003514	0	0	2.21497	0	2.549041	3.704108	2.334575	4.803496	19.27608	0	2.056169	4.653602	1.956012	3.297807	1.026376	0.910133	2.688924
AC044860.	4.804538	23.00898	0.999134	4.157171	6.64491	2.283774	4.248401	4.630135	1.167288	6.724895	12.04755	11.04094	14.39318	2.792161	1.956012	5.771163	7.184634	5.4608	4.48154
AC044860.	5.765445	7.002733	3.996535	5.196464	0	17.12831	16.9936	0.926027	4.669151	10.56769	6.023774	0	5.140422	0.93072	0	0	0	4.550667	1.792616
AC046130.	33.63176	27.01054	27.97574	45.72888	32.11706	29.68906	30.58849	23.15068	18.6766	32.66377	21.68559	35.88305	44.20763	40.02097	23.47214	48.64266	26.68578	19.1128	33.16339
AC046134.	20.17906	20.00781	31.97228	18.70727	5.537425	15.98642	20.39233	48.15341	4.669151	13.44979	4.81902	16.56141	24.67402	39.09025	12.71408	19.78684	26.68578	21.8432	7.170464
AC046136.	3.84363	1.00039	4.995668	7.275049	14.3973	9.135096	5.947762	8.334243	19.84389	44.19217	9.638039	12.8811	12.33701	7.445762	18.58211	9.893422	4.105505	19.1128	12.54831
AC046143.	85.52077	64.02499	94.9177	44.68959	93.02873	78.7902	77.3209	79.63833	136.5727	98.95202	144.5706	72.68618	65.7974	79.11123	104.6466	95.63641	78.0046	99.20454	64.53417
AC046143.	22.10087	42.0164	29.97401	29.1002	24.36467	23.97963	21.24201	34.263	52.52795	23.05678	20.48083	20.24172	19.5336	31.64449	25.42815	35.45143	17.4484	19.1128	13.44462
AC046158.	0	0	3.996535	1.039293	0	0	1.69936	0.926027	0	5.764196	0	0.920078	1.028084	4.653602	6.846041	0.824452	1.026376	0.910133	0
AC046158.	1.921815	0	1.998267	0	1.107485	0	0.84968	0.926027	3.501863	0.960699	2.40951	0.920078	1.028084	1.861441	1.956012	5.771163	1.026376	0	2.688924
AC046158.	12.4918	0	2.997401	9.353635	32.11706	23.97963	17.84329	28.70684	2.334575	13.44979	4.81902	10.12086	5.140422	0.93072	4.890029	11.54233	3.079129	20.93307	26.88924
AC046158.	0	0	10.99047	0	0	0	0.84968	0.926027	4.669151	2.882098	0	5.520469	0	0.93072	0	2.473355	9.237386	0	1.792616
AC046168.	51.88901	54.02108	33.97054	58.2004	13.28982	30.83095	46.73241	55.56162	61.86625	24.01748	67.46627	61.64524	74.02207	40.95169	30.31818	32.97807	70.81996	20.93307	43.02278
AC046168.	11.53089	18.00703	14.987	15.58939	36.547	10.27698	14.44456	17.59451	29.18219	15.37119	26.50461	7.360626	8.224675	10.23792	17.6041	12.36678	8.21101	21.8432	26.88924
AC046176.	100.8953	143.0558	125.8908	80.02554	95.2437	156.4385	82.41898	100.0109	110.8923	84.54153	116.8612	44.16376	86.35909	82.83411	63.57038	85.74299	145.7454	67.34987	91.42341
AC046185.	85.52077	105.041	156.864	124.7151	73.094	114.1887	95.16419	62.96984	71.20455	104.7162	113.247	106.7291	126.4544	80.04195	100.7346	116.2477	108.7959	85.55254	106.6606
AC046185.	195.0642	104.0406	106.9073	110.165	134.0057	140.4521	95.16419	140.7561	148.2455	141.2228	256.6128	141.692	151.1284	131.2316	146.7009	149.2258	151.9037	164.7341	160.4391
AC046195.	77.83351	68.02655	124.8917	78.98625	62.01916	67.37133	96.86355	125.9397	45.52422	90.30573	55.41872	51.52438	101.7804	70.73474	76.28445	46.1693	52.34519	34.58507	69.91202
AC046195.	0	0	0	0	0	0	0	1.852054	0	0	0	0.920078	0	0	0	0	0	0	0
AC048334.	0.960908	0	1.998267	0	1.107485	0	0	0.926027	0	0.960699	0	0	0	1.861441	0	0.824452	2.052753	0	0.896308
AC048337.	0	0	0	0	0	0	0	0	0	1.921399	0	0	0	0.93072	0.978006	0	0	0	0
AC048338.	33.63176	25.00976	20.98181	18.70727	19.93473	35.3985	46.73241	20.37259	7.003726	8.646293	1.204755	17.48149	14.39318	13.03008	23.47214	37.10033	13.34289	23.66347	24.20031
AC048341.	0.960908	4.001562	2.997401	0	0	0	9.346483	0	0	0	0	0.920078	0	3.722881	0	0	0	0.910133	0
AC048341.	3224.806	2233.872	3607.872	3093.975	2675.684	1644.317	3015.515	2338.218	3703.804	2709.172	2954.059	3811.884	3962.237	2777.269	3065.07	2911.964	2876.933	3581.375	3077.025
AC048341.	128.7616	162.0633	268.767	100.8114	229.2494	258.0665	130.8508	226.8766	342.0153	605.2405	775.8621	335.8286	322.8185	272.701	214.1833	138.5079	113.9278	184.7571	156.8539
AC048344.	34.59267	5.001952	45.96015	9.353635	18.82724	5.709435	16.14392	13.89041	4.669151	24.01748	30.11887	24.84211	22.61786	9.307203	17.6041	7.420066	17.4484	24.5736	13.44462
AC048344.	1.921815	0	1.998267	0	0	0	0	0	0	1.921399	0	0	0	0	0	0.824452	1.026376	0	0
AC048344.	30.74904	9.003514	58.94889	8.314342	27.68712	21.69585	16.14392	48.15341	17.50932	14.41049	1.204755	25.76219	28.78636	12.09936	19.56012	17.31349	13.34289	26.39387	25.09662
AC048344.	49.96719	44.01718	81.92896	57.1611	116.2859	85.64153	62.87634	37.04108	239.294	57.64196	39.75691	44.16376	60.65698	60.49682	89.97654	66.7806	39.0023	30.0344	42.12647
AC048347.	0.960908	0	0	0	0	0	0.84968	11.11232	0	0	0	0	0	0	0	0	6.158258	0	0
AC048351.	61.49808	37.01445	52.95408	63.39686	78.63143	51.38492	36.53625	45.37532	38.5205	27.86028	18.07132	38.64329	27.75828	49.32818	30.31818	11.54233	22.58028	50.05734	48.40063
AC048380.	0	1.00039	0	1.039293	0	2.283774	1.69936	0	0	0	0	1.840156	0	0	1.956012	1.648904	0	1.820267	0
AC048380.	86.48168	5.001952	147.8718	35.33595	246.9691	69.65511	0.84968	210.2081	371.1975	207.511	348.1742	47.84407	47.29188	81.90339	114.4267	70.07841	89.29473	126.5085	77.97879
AC048380.	4.804538	3.001171	0	2.078586	0	1.141887	12.7452	0	31.51677	19.21399	1.204755	5.520469	7.196591	0.93072	0.978006	2.473355	5.131881	0.910133	11.652
AC048382.	70.14625	77.03006	45.96015	59.23969	53.15928	53.66869	87.51706	21.29862	36.18592	33.62447	26.50461	56.12477	53.46039	19.54513	29.34017	34.62698	47.21331	78.27147	29.57816
AC048382.	73.98988	39.01523	60.94715	59.23969	58.6967	20.55397	57.77826	70.37805	77.04099	61.48475	25.29985	74.52634	71.96591	51.18962	52.81231	50.29156	50.29244	50.96747	61.84525
AC048382.	0	0	0	0	0	0	0	0	0	0	1.204755	0	0	0	0	0	2.052753	0	0.896308
AC048382.	2.882723	5.001952	3.996535	1.039293	4.42994	5.709435	6.797442	6.482189	1.167288	6.724895	1.204755	2.760235	4.112337	0	2.934017	1.648904	5.131881	3.640534	2.688924
AC048382.	116.2698	93.03631	151.8683	127.833	158.3703	178.1344	145.2953	105.5671	197.2716	134.4979	130.1135	118.6901	142.9037	85.62627	104.6466	101.4076	87.24198	93.74374	116.52
AC048383.	2.882723	3.001171	1.998267	12.47151	12.18233	5.709435	11.04584	0	30.34948	11.52839	18.07132	6.440548	2.056169	2.792161	10.75806	8.244518	11.29014	2.7304	2.688924
AC051618.	0	0	0	0	0	0	2.549041	0	0	0	1.204755	0.920078	0	0	0	0	1.026376	0	0.896308
AC051619.	1.921815	2.000781	2.997401	1.039293	9.967364	0	0	18.52054	7.003726	0.960699	9.638039	11.04094	6.168506	0	0	1.648904	5.131881	0.910133	1.792616
AC051619.	0	0	0	0	0	1.141887	0	4.630135	0	9.606993	4.81902	0.920078	0	0	0	3.297807	0	0	0
AC051619.	266.1714	182.0711	311.7297	138.2259	116.2859	161.0061	64.5757	301.8848	221.7847	190.2185	189.1465	235.54	354.6891	40.95169	57.70234	136.0346	130.3498	81.00187	109.3496
AC051619.	12.4918	9.003514	11.9896	17.66798	24.36467	17.12831	9.346483	16.66849	49.02608	26.89958	46.98544	20.24172	5.140422	13.9608	16.6261	16.48904	8.21101	21.8432	17.92616
AC051619.	367.0667	228.089	211.8163	186.0334	46.51437	99.34417	251.5054	255.5835	389.8741	226.725	204.8083	371.7116	345.4363	10.23792	17.6041	272.0691	414.656	172.0152	181.9505
AC051619.	7.68726	4.001562	16.98527	0	12.18233	0	1.69936	0.926027	1.167288	6.724895	15.66181	3.680313	4.112337	3.722881	7.824047	4.946711	3.079129	8.191201	6.274156
AC051619.	9.609075	3.001171	5.994802	6.235757	1.107485	5.709435	5.947762	8.334243	5.836439	3.842797	3.614265	9.200782	3.084253	3.722881	4.890029	0.824452	2.052753	10.9216	12.54831

AC051619.	0	6.002343	3.996535	0	0	0	0	0	1.167288	0	0	0	0	0.93072	7.824047	0	0	0	0
AC051649.	0	0	0	0	4.42994	0	0	0	7.003726	0	0	0	1.028084	0	0	0	0	0	1.792616
AC051649.	157.5888	133.0519	124.8917	139.2652	313.4182	188.4114	84.11834	359.2985	1484.79	571.6161	1119.217	652.3355	631.2438	183.3519	163.327	126.9656	214.5126	255.7475	230.3511
AC053481.	8.648168	0	4.995668	12.47151	14.3973	10.27698	11.04584	12.03835	0	1.921399	2.40951	13.80117	9.252759	11.16864	7.824047	9.06897	13.34289	9.101334	8.963079
AC053481.	2.882723	14.00547	4.995668	23.90373	4.42994	9.135096	7.647122	4.630135	5.836439	5.764196	15.66181	7.360626	10.28084	17.68369	8.802052	11.54233	12.31652	17.29253	13.44462
AC053503.	24.02269	49.01913	38.96621	46.76817	14.3973	14.84453	41.63433	32.41095	9.338302	21.13538	2.40951	46.00391	30.84253	23.26801	23.47214	37.10033	50.29244	37.31547	39.43755
AC053503.	33.63176	39.01523	32.97141	50.92535	22.1497	59.37812	41.63433	22.22465	26.84762	23.05678	56.62348	22.08188	26.73019	62.35826	35.20821	28.03136	37.97592	25.48374	44.8154
AC053503.	0.960908	2.000781	2.997401	2.078586	0	7.993209	4.248401	5.556162	3.501863	0	0	1.840156	2.056169	0	0	2.473355	3.079129	5.4608	2.688924
AC053503.	21.13997	24.00937	13.98787	46.76817	7.752394	27.40529	22.94137	16.66849	49.02608	36.50657	48.1902	20.24172	17.47743	48.39746	31.29619	18.13794	17.4484	15.47227	35.85232
AC053503.	2.882723	5.001952	0	5.196464	0	1.141887	0.84968	2.778081	7.003726	0.960699	0	0	0	0	1.956012	0	0	0	2.688924
AC053513.	453.5484	397.155	528.5417	698.4047	414.1994	264.9178	658.5022	539.8738	46.69151	276.6814	180.7132	619.2127	473.9469	523.0648	587.7815	394.9124	426.9725	388.627	460.7023
AC053527.	33.63176	32.01249	46.95928	47.80747	35.43952	37.68227	37.38593	41.67122	26.84762	48.99566	49.39495	34.96297	85.331	36.29809	39.12023	39.57369	35.92317	53.69787	29.57816
AC055713.	1223.235	1119.437	1716.512	520.6857	832.8287	1052.82	1012.819	1241.802	1006.202	1221.049	1173.431	1121.575	1030.141	1079.636	1265.54	785.7026	784.1515	1119.464	985.9387
AC055716.	1.921815	0	0	0	0	4.567548	5.098081	3.704108	0	3.842797	0	5.520469	2.056169	0.93072	8.802052	3.297807	4.105505	1.820267	0.896308
AC055717.	48.04538	39.01523	30.97314	57.1611	75.30897	28.54718	45.88273	22.22465	50.19337	20.17468	22.89034	48.76415	47.29188	44.67457	49.8783	70.90286	80.05735	26.39387	43.91909
AC055717.	0	1.00039	1.998267	8.314342	0	5.709435	4.248401	2.778081	1.167288	0	0	0	0	0.93072	1.956012	0.824452	1.026376	0	1.792616
AC055717.	5.765445	0	0	0	26.57964	1.141887	3.398721	0	10.50559	2.882098	3.614265	0.920078	1.028084	1.861441	0	0	2.052753	1.820267	3.585232
AC055720.	0	0	0	10.39293	11.07485	0	1.69936	0	4.669151	1.921399	1.204755	12.8811	1.028084	0.93072	11.73607	5.771163	3.079129	0.910133	0
AC055720.	83.59896	89.03475	94.9177	126.7937	109.641	141.594	78.17058	119.4575	126.0671	164.2796	131.3183	109.4893	110.005	84.69555	94.86656	75.02512	109.8223	67.34987	85.14926
AC055731.	0	0	0	0	0	0	2.549041	0	0	0	1.204755	0	0	0	1.956012	3.297807	0	0	1.792616
AC055733.	0	0	0	0	0	0	0.84968	0	0	3.842797	0	0	1.028084	0	0	0	0	0	0
AC055733.	0	0	0.999134	1.039293	0	2.283774	0.84968	0	0	0	0	1.840156	0	0	0	0	0	3.640534	0.896308
AC055733.	0	0	0	0	0	1.141887	0	0	1.167288	0	9.638039	4.600391	0	0	0	0	0	2.7304	0
AC055733.	9.609075	7.002733	2.997401	2.078586	12.18233	11.41887	3.398721	9.26027	32.68406	18.25329	21.68559	5.520469	17.47743	0.93072	4.890029	2.473355	5.131881	10.01147	0
AC055736.	0	0	0	0	0	0	1.69936	0	0	0	0	0	0	0	0	0	0	0	0
AC055748.	2.882723	0	0	13.51081	0	0	5.098081	3.704108	7.003726	0	0	4.600391	2.056169	0	2.934017	7.420066	6.158258	4.550667	0.896308
AC055758.	0	0	3.996535	0	0	0	2.549041	0	0	0	0	0.920078	1.028084	0.93072	0	0	0	0.910133	0.896308
AC055758.	0	0	0	0	3.322455	6.851322	0.84968	0	0	3.842797	0	3.680313	0	0	0.978006	0	0	0	0
AC055764.	0	3.001171	1.998267	0	0	1.141887	1.69936	0.926027	0	0.960699	8.433284	0	5.140422	2.792161	5.868035	0	0	4.550667	0
AC055764.	73.02897	58.02265	25.97748	53.00393	53.15928	90.20907	57.77826	57.41368	96.88488	122.0088	65.05676	30.36258	45.23571	67.94258	35.20821	61.83389	47.21331	32.7648	44.8154
AC055811.	16.33543	26.01015	17.98441	11.43222	33.22455	11.41887	15.29424	8.334243	0	3.842797	10.84279	57.96493	49.34805	35.36737	27.38416	4.122259	16.42202	5.4608	28.68185
AC055811.	75.91169	52.0203	67.94109	38.45383	50.94431	87.9253	62.02666	73.15614	79.37557	92.22713	104.8137	58.88501	62.71315	33.50593	37.16422	28.85581	44.13418	40.04587	61.84525
AC055811.	0	15.00586	10.99047	15.58939	14.3973	11.41887	17.84329	17.59451	0	32.66377	14.45706	20.24172	14.39318	12.09936	15.64809	21.43575	4.105505	7.281067	10.7557
AC055811.	1714.259	1288.503	2282.021	1650.397	2053.277	849.5639	1671.321	1702.038	987.5254	1880.088	1879.418	2651.665	2645.261	1787.914	1550.139	1667.042	1536.485	1992.282	2480.98
AC055813.	1.921815	9.003514	11.9896	1.039293	12.18233	4.567548	6.797442	0	0	1.921399	0	8.280704	5.140422	13.03008	1.956012	5.771163	0	5.4608	0.896308
AC055822.	22.10087	19.00742	8.992203	15.58939	24.36467	31.97284	10.19616	42.59724	77.04099	41.31007	56.62348	35.88305	28.78636	28.85233	21.51613	26.38246	41.05505	18.20267	34.0597
AC055839.	10.56998	6.002343	4.995668	5.196464	16.61227	1.141887	10.19616	24.0767	8.171014	3.842797	0	23.92203	16.44935	2.792161	0.978006	0.824452	1.026376	0.910133	5.377848
AC055854.	10.56998	1.00039	33.97054	4.157171	13.28982	13.70264	14.44456	21.29862	22.17847	24.01748	38.55216	7.360626	12.33701	27.92161	18.58211	9.06897	8.21101	19.1128	17.92616
AC055855.	152.7843	148.0578	169.8527	109.1257	140.6506	135.8846	130.0011	146.3123	185.5987	170.0438	178.3037	137.0917	197.3922	130.3008	100.7346	112.1254	99.5585	113.7667	104.868
AC055855.	3.84363	24.00937	29.97401	9.353635	19.93473	19.41208	9.346483	6.482189	1.167288	46.11356	28.91412	26.68227	25.70211	18.61441	21.51613	12.36678	2.052753	8.191201	0.896308
AC055874.	32.67086	54.02108	25.97748	32.21808	38.76197	31.97284	25.49041	14.81643	93.38302	27.86028	39.75691	36.80313	30.84253	65.15042	33.2522	79.97183	76.97822	35.4952	20.61508
AC055876.	3.84363	1.00039	0.999134	6.235757	1.107485	4.567548	5.098081	0	5.836439	0	0	2.760235	1.028084	1.861441	1.956012	8.244518	8.21101	0.910133	2.688924
AC055876.	89.3644	142.0554	123.8926	86.2613	129.5757	145.0196	46.73241	109.2712	109.725	88.38433	68.67103	131.5712	83.27483	203.8277	158.4369	108.0032	87.24198	81.00187	108.4533
AC055876.	30.74904	25.00976	43.96188	8.314342	13.28982	34.25661	16.9936	33.33697	52.52795	49.95636	68.67103	24.84211	30.84253	13.03008	7.824047	9.06897	18.47477	4.550667	0
AC055876.	0	0	0	0	0	0	0	3.704108	0	0	9.638039	0	1.028084	0.93072	0	3.297807	1.026376	0	0
AC055878.	0	0	0	1.039293	0	0	0	0	0	0	0	0	0	0	0	0	0	0	0.896308
AC058791.	60.53717	71.02772	77.93243	43.6503	83.06137	77.64832	58.62794	96.30681	164.5876	96.06993	124.0898	69.00587	94.58376	82.83411	121.2727	69.25395	36.94955	37.31547	33.16339
AC058823.	0	0	1.998267	0	0	0	1.69936	3.704108	0	0	0	0	0	0	1.956012	0.824452	0	0.910133	0
AC060234.	1.921815	10.0039	0	9.353635	12.18233	0	2.549041	0.926027	0	0	0	4.600391	1.028084	0.93072	0.978006	3.297807	0	1.820267	1.792616
AC060234.	105.6998	46.01796	80.92983	85.22201	43.19191	49.10114	65.42538	94.45476	57.1971	52.83846	83.12809	67.16571	50.37613	57.70466	43.03226	98.93422	57.47707	112.8565	66.32679
AC060234.	1.921815	0	0	3.117878	0	0	6.797442	7.408216	0	0	0	1.840156	0	0	0	0	0	1.820267	0.896308
AC060764.	0	0	0	0	0	1.141887	0	0	1.167288	0.960699	0	0	0	0	0	0	0	1.820267	0
AC060765.	0	1.00039	0	0	0	6.851322	2.549041	0	0	0	0	0	0	1.861441	0	0	0	0	3.585232
AC060766.	434.3302	234.0914	257.7765	405.3242	508.3356	431.6333	457.128	618.5861	541.6215	822.3586	836.0999	535.4855	562.3622	473.7366	506.607	437.7839	359.2317	453.2464	334.3229
AC060766.	2.882723	4.001562	2.997401	2.078586	0	2.283774	0	3.704108	9.338302	0.960699	4.81902	2.760235	0	2.792161	5.868035	5.771163	2.052753	1.820267	3.585232
AC060766.	3.84363	2.000781	1.998267	23.90373	1.107485	5.709435	12.7452	6.482189	12.84017	17.29259	19.27608	7.360626	6.168506	5.584322	6.846041	20.6113	13.34289	10.9216	8.963079
AC060766.	216.2042	155.0605	132.8848	196.4263	305.6658	318.5865	136.7985	254.6574	524.1122	578.3409	731.2862	259.4621	293.004	260.6017	280.6877	213.533	224.7764	182.0267	156.8539
AC060766.	9.609075	8.003124	0.999134	5.196464	7.752394	7.993209	6.797442	19.44657	26.84762	28.82098	60.23774	17.48149	7.196591	6.515042	9.780058	11.54233	8.21101	22.75334	7.170464
AC060766.	58.61536	40.01562	48.95755	98.73281	151.7254	133.6008	95.16419	165.7588	123.7325	251.7032	268.6603	117.77	131.5948	150.7767	132.0308	108.0032	83.13648	89.19307	82.46033
AC060766.	239.266	173.0675	188.8363	198.5049	354.3952	295.7487	226.8646	414.8601	335.0116	488.9959	440.9403	304.5459	305.3411	327.6135	298.2918	281.1381	227.8555	180.2064	148.7871
AC060771.	0.960908	1.00039	7.993069	1.039293	1.107485	1.141887	5.098081	0	2.334575	0	8.433284	3.680313	3.084253	0	0	2.473355	1.026376	3.640534	4.48154
AC060773.	0	0	5.994802	6.235757	4.42994	1.141887	0	0	0	0	0	0	1.028084	0	0	0	0	3.640534	0

AC060780.	6383.309	3592.402	3948.576	3128.271	3477.503	3818.47	3379.178	4633.839	4341.143	3820.701	3731.126	4600.391	4324.123	3935.085	4017.648	3876.573	3833.515	3378.415	3289.45
AC060780.	40.35812	91.03553	39.96535	58.2004	59.80419	28.54718	71.37314	50.93149	9.338302	33.62447	56.62348	76.36649	80.19058	61.42754	53.79032	56.88718	70.81996	62.79921	76.18618
AC060780.	59.57627	44.01718	45.96015	47.80747	98.56616	41.10793	84.96802	43.52327	23.34575	64.36685	25.29985	94.76806	83.27483	71.66546	55.74633	69.25395	63.63533	84.64241	60.05263
AC060809.	17.29634	26.01015	22.98007	25.98232	12.18233	4.567548	33.13753	11.11232	0	6.724895	0	22.08188	32.8987	18.61441	11.73607	32.97807	9.237386	15.47227	34.95601
AC060814.	0	0	0	0	0	0	0	0	0	0	0	0	0	0	0	0	0	0	1.792616
AC060814.	3.84363	3.001171	13.98787	7.275049	2.21497	4.567548	9.346483	0.926027	0	4.803496	1.204755	7.360626	2.056169	6.515042	7.824047	12.36678	3.079129	8.191201	19.71877
AC060814.	0	3.001171	0	0	1.107485	0	0	0	0	0	0	3.614265	0	1.028084	0.93072	0	0	0	0
AC060814.	3.84363	4.001562	0.999134	3.117878	1.107485	3.425661	0	1.852054	14.00745	13.44979	6.023774	3.680313	7.196591	0	0	0.824452	0	0.910133	5.377848
AC060814.	0.960908	0	0.999134	0	0	1.141887	0	0.926027	0	0	0	0.920078	1.028084	0.93072	0	0.824452	0	0	0.896308
AC060814.	0	0	0	0	0	31.97284	0	0	0	0	0	0	0	0	0	51.94047	101.6112	0	0
AC060834.	0	0	0	0	0	0	0	0.926027	0	0	0	0	0	5.584322	1.956012	0	0	0	0
AC060834.	2.882723	0	0	0	1.107485	0	0	1.852054	0	0	0	1.840156	3.084253	1.861441	3.912023	0	4.105505	0	0
AC061709.	0	0	1.998267	0	0	0	0	0	0	0	0	0	1.028084	0	0	0	0	0	0.896308
AC061709.	0	0	0	1.039293	0	0	0.84968	0	0	0.960699	1.204755	0.920078	0	0	0.978006	0	0	0	0
AC061961.	76.8726	94.0367	144.8744	63.39686	100.7811	97.0604	168.2367	105.5671	15.17474	59.56335	74.6948	108.5692	107.9489	114.4786	68.46041	93.16306	121.1124	70.08027	123.6905
AC061965.	0.960908	0	1.998267	5.196464	1.107485	0	2.549041	3.704108	0	0	0	1.840156	1.028084	0	0.978006	4.122259	5.131881	0.910133	0
AC061975.	57.65445	31.0121	42.96275	97.69352	45.40688	102.7698	79.02026	55.56162	21.01118	47.07426	72.28529	57.04485	60.65698	73.5269	47.92229	65.13169	94.42662	49.1472	75.28987
AC061975.	5.765445	2.000781	1.998267	8.314342	1.107485	0	8.496802	0	0	1.921399	0	8.280704	8.224675	1.861441	3.912023	7.420066	2.052753	4.550667	6.274156
AC061975.	9.609075	24.00937	14.987	24.94303	27.68712	9.135096	52.68017	32.41095	0	0.960699	0	18.40156	22.61786	23.26801	15.64809	28.03136	9.237386	15.47227	38.54124
AC061975.	0	0	3.996535	0	0	0	0	5.556162	0	0	0	0	0	0	0.978006	0	2.052753	0	1.792616
AC061975.	0	0	4.995668	0	0	1.141887	0.84968	0	0	0.960699	0	0	0	0	0	0.824452	0	0	0
AC061975.	19.21815	19.00742	38.96621	2.078586	1.107485	11.41887	12.7452	4.630135	2.334575	4.803496	9.638039	13.80117	26.73019	10.23792	7.824047	27.20691	11.29014	2.7304	21.51139
AC061992.	52.84991	10.0039	47.95842	14.5501	34.33203	31.97284	11.89552	2.778081	42.02236	23.05678	44.57593	48.76415	64.76932	53.98178	46.94428	275.3669	209.3808	383.1662	178.3653
AC061999.	0	4.001562	8.992203	6.235757	6.64491	5.709435	14.44456	0.926027	8.171014	0.960699	3.614265	8.280704	17.47743	14.89152	7.824047	7.420066	15.39564	2.7304	8.066772
AC062004.	0	11.00429	10.99047	1.039293	16.61227	0	2.549041	0	16.34203	17.29259	26.50461	12.8811	12.33701	3.722881	5.868035	16.48904	24.63303	4.550667	11.652
AC062015.	0	0	0	0	0	0	0	0	0	0	0	0	0	0.93072	0	0	1.026376	0	0
AC062016.	0	0	0	1.039293	0	0	0.84968	0	0	0	0	0	0	0	0	0	0	0	0
AC062017.	1.921815	21.0082	11.9896	2.078586	6.64491	3.425661	8.496802	3.704108	31.51677	12.48909	89.15186	1.840156	4.112337	3.722881	6.846041	4.946711	2.052753	11.83173	5.377848
AC062020.	0	0	1.998267	0	0	0	0	0	0	0	0	0	0	0	0	6.595615	2.052753	0	0
AC062021.	3.84363	4.001562	3.996535	4.157171	0	4.567548	2.549041	2.778081	0	6.724895	1.204755	8.280704	1.028084	3.722881	0	6.595615	2.052753	3.640534	3.585232
AC062028.	96.09075	111.0433	52.95408	91.45776	45.40688	67.37133	67.12474	91.67668	46.69151	87.42363	95.17564	135.2515	133.651	151.7074	104.6466	66.7806	88.26836	93.74374	108.4533
AC062028.	3.84363	11.00429	6.993936	5.196464	8.859879	9.135096	4.248401	1.852054	0	23.05678	0	3.680313	0	5.584322	18.58211	13.19123	12.31652	0	5.377848
AC062029.	623.629	591.2307	962.1657	543.5501	514.9805	502.4303	660.2015	695.4463	548.6252	459.2142	457.8069	693.739	535.632	563.0858	661.1319	526.0003	557.3223	621.6211	590.6669
AC062031.	0	5.001952	4.995668	7.275049	4.42994	1.141887	0.84968	18.52054	0	0	0	0	8.224675	0.93072	0	0	0	1.820267	3.585232
AC062037.	0.960908	3.001171	4.995668	11.43222	1.107485	2.283774	5.947762	1.852054	0	17.29259	0	1.840156	3.084253	1.861441	0	8.244518	2.052753	6.370934	2.688924
AC062037.	4.804538	3.001171	9.991337	8.314342	0	10.27698	1.69936	21.29862	2.334575	18.25329	8.433284	18.40156	11.30893	15.82225	3.912023	7.420066	4.105505	7.281067	17.92616
AC062039.	0	0	0	0	0	0	0	0	0	2.882098	0	0	0	0	0.978006	0	0	0	0
AC063919.	85.52077	111.0433	192.8328	181.8762	149.5105	125.6076	231.9627	80.56435	73.53913	184.4543	190.3513	119.6102	114.1174	69.80402	95.84457	46.99375	81.08372	194.7686	164.9207
AC063924.	0	0	0	3.117878	0	0	0	0	0	0	9.638039	0	0	0	0	0.824452	1.026376	0.910133	3.585232
AC063926.	4.804538	1.00039	8.992203	4.157171	0	0	9.346483	7.408216	5.836439	8.646293	7.228529	13.80117	2.056169	9.307203	0	4.122259	11.29014	2.7304	8.066772
AC063926.	0	1.00039	0	0	3.322455	0	0.84968	0	0	2.882098	0	0	0	0	0	0	2.052753	0	0
AC063926.	19.21815	8.003124	13.98787	21.82515	35.43952	6.851322	11.04584	2.778081	1.167288	18.25329	0	18.40156	11.30893	9.307203	12.71408	3.297807	20.52753	5.4608	15.23724
AC063943.	59.57627	76.02967	126.89	69.63262	76.41646	38.82416	94.31451	115.7534	100.3867	95.10923	95.17564	103.9688	71.96591	57.70466	91.93255	35.45143	54.39794	100.1147	81.56402
AC063943.	0	0	0	0	0	0	0	0	0	0	0	4.600391	0	2.792161	0	0	0	0	0
AC063943.	19.21815	7.002733	22.98007	31.17878	17.71976	25.12151	9.346483	5.556162	33.85134	21.13538	27.70936	6.440548	19.5336	22.33729	11.73607	14.01568	10.26376	20.93307	13.44462
AC063944.	70.14625	144.0562	138.8796	108.0864	112.9635	91.35096	96.86355	139.8301	35.01863	103.7555	60.23774	144.4523	114.1174	83.76483	114.4267	112.9499	100.5849	80.09174	139.824
AC063944.	317.0995	362.1413	300.7392	187.0727	184.95	358.5525	231.9627	271.3259	299.9929	303.581	352.9932	194.1365	243.656	201.0356	248.4135	189.6239	182.695	243.0056	292.1964
AC063944.	62.45899	98.03826	30.97314	44.68959	39.86946	79.93209	41.63433	69.45203	102.7213	117.2053	66.26152	41.40352	62.71315	40.02097	38.14223	35.45143	37.97592	38.2256	68.1194
AC063947.	0	5.001952	5.994802	1.039293	0	0	13.59488	0.926027	0	0	7.228529	6.440548	0	0.93072	0	0.824452	2.052753	2.7304	4.48154
AC063947.	0	3.001171	1.998267	4.157171	6.64491	1.141887	0	0.926027	0	0.960699	0	0.920078	0	0.93072	0	7.420066	0	0	1.792616
AC063948.	43.24084	70.02733	40.96448	24.94303	25.47215	33.11472	47.58209	34.263	88.71387	112.4018	110.8374	73.60626	56.54464	45.60529	71.39443	14.84013	44.13418	68.26001	86.04556
AC063950.	24.9836	55.02147	26.97661	61.31827	33.22455	13.70264	39.93497	32.41095	14.00745	40.34937	24.0951	55.20469	44.20763	40.02097	45.96627	19.78684	18.47477	22.75334	79.77141
AC063952.	27.86632	21.0082	72.93676	64.43615	40.97694	46.81737	93.46483	52.78354	66.5354	40.34937	40.96167	39.56336	20.56169	30.71377	56.72434	44.5204	33.87042	53.69787	39.43755
AC063952.	4.804538	0	2.997401	0	0	0	2.549041	0	2.334575	0	1.204755	0	12.33701	0.93072	0.978006	4.122259	3.079129	8.191201	4.48154
AC063952.	0.960908	0	1.998267	0	6.64491	0	0	0	1.167288	0.960699	0	0	2.056169	0	0	1.648904	4.105505	0	0
AC063961.	3.84363	9.003514	1.998267	9.353635	9.967364	5.709435	14.44456	9.26027	0	7.685594	7.228529	7.360626	7.196591	10.23792	15.64809	4.122259	8.21101	9.101334	4.48154
AC063962.	0	11.00429	0	1.039293	0	1.141887	5.098081	5.556162	0	1.921399	1.204755	0	6.168506	3.722881	5.868035	0	7.184634	3.640534	12.54831
AC063965.	123.9571	86.03358	141.877	129.9116	69.77155	77.64832	132.5501	123.1616	70.03726	81.65944	78.30907	126.9708	157.2969	94.93347	119.3167	234.1443	177.5631	189.3078	128.172
AC063976.	0	0	0	0	0	0	0	0	0	0	0	0	1.028084	0	5.868035	0	0	0.910133	0
AC063976.	42.27993	52.0203	149.87	64.43615	165.0153	69.65511	113.8572	178.7232	36.18592	71.09174	66.26152	225.4192	263.1896	80.04195	106.6026	61.83389	68.76721	55.51814	85.14926
AC063976.	0	1.00039	0	0	3.322455	0	0.84968	0	0	0	0	0	2.056169	0	0	0.824452	0	0	0
AC063977.	6.726353	4.001562	2.997401	38.45383	6.64491	1.141887	0.84968	15.74246	2.334575	4.803496	14.45706	13.80117	8.224675	25.12945	4.890029	50.29156	32.84404	42.77627	29.57816

AC063977.	16.33543	9.003514	7.993069	15.58939	17.71976	23.97963	11.04584	43.52327	4.669151	9.606993	6.023774	20.24172	19.5336	49.32818	15.64809	80.79628	29.76491	94.65388	72.60094
AC063977.	0	0	0	0	0	0	0.84968	0	0	0	0	0	0	0	0	0.824452	0	0	0
AC063977.	2.882723	1.00039	3.996535	35.33595	4.42994	26.2634	7.647122	8.334243	22.17847	10.56769	7.228529	10.12086	9.252759	5.584322	3.912023	36.27588	45.16056	28.21414	18.82247
AC063977.	0	0	0.999134	9.353635	0	4.567548	0	3.704108	0	0.960699	2.40951	7.360626	5.140422	0.93072	0	18.96239	11.29014	10.9216	0
AC063979.	5.765445	3.001171	0	8.314342	0	3.425661	4.248401	12.03835	0	0.960699	0	5.520469	5.140422	1.861441	6.846041	9.893422	10.26376	4.550667	12.54831
AC064799.	123.9571	109.0426	96.91597	69.63262	89.70628	92.49285	66.27506	81.49038	68.86998	57.64196	38.55216	101.2086	71.96591	82.83411	67.4824	76.67402	106.7431	123.7781	62.74156
AC064799.	0	0	2.997401	0	0	0	0	0	0	0.960699	0	0	0	0.93072	0.978006	0	0	0	0
AC064801.	10.56998	16.00625	17.98441	11.43222	22.1497	23.97963	10.19616	3.704108	22.17847	6.724895	2.40951	4.600391	16.44935	16.75297	5.868035	3.297807	21.5539	13.652	13.44462
AC064801.	8.648168	3.001171	25.97748	18.70727	36.547	4.567548	46.73241	24.0767	5.836439	6.724895	9.638039	44.16376	53.46039	19.54513	40.09824	3.297807	10.26376	7.281067	38.54124
AC064802.	5.765445	15.00586	6.993936	6.235757	6.64491	0	3.398721	17.59451	5.836439	0.960699	2.40951	0.920078	0	8.376483	21.51613	11.54233	8.21101	5.4608	0.896308
AC064805.	16.33543	7.002733	22.98007	12.47151	28.79461	12.56076	15.29424	38.89314	1.167288	19.21399	26.50461	32.20274	10.28084	12.09936	17.6041	17.31349	7.184634	330.3784	198.9804
AC064805.	10.56998	8.003124	12.98874	12.47151	12.18233	9.135096	16.9936	2.778081	10.50559	8.646293	4.81902	7.360626	10.28084	2.792161	2.934017	16.48904	5.131881	6.370934	19.71877
AC064807.	335.3567	453.1769	357.6899	453.1316	331.138	339.1404	387.4542	351.8903	287.1528	316.0701	383.1121	495.0021	463.6661	436.5078	369.6862	450.1507	383.8647	406.8296	459.806
AC064807.	4.804538	3.001171	0	0	1.107485	1.141887	4.248401	0.926027	29.18219	3.842797	4.81902	5.520469	8.224675	9.307203	3.912023	10.71787	1.026376	10.01147	4.48154
AC064807.	49.96719	78.03045	75.93416	57.1611	63.12664	101.6279	60.3273	84.26846	79.37557	72.05244	103.6089	33.12282	34.95487	73.5269	65.52639	58.53608	75.95184	41.86614	45.71171
AC064834.	1.921815	0	0	0	0	0	0	0	0	0	0	0	0	0	0.978006	1.648904	0	0	0
AC064836.	17.29634	11.00429	3.996535	5.196464	25.47215	15.98642	11.89552	5.556162	25.68033	17.29259	19.27608	22.08188	18.50552	8.376483	9.780058	10.71787	8.21101	6.370934	21.51139
AC064836.	9.609075	16.00625	4.995668	0	9.967364	1.141887	7.647122	12.03835	22.17847	9.606993	2.40951	4.600391	12.33701	8.376483	2.934017	9.06897	0	9.101334	10.7557
AC064836.	100.8953	169.066	169.8527	83.14342	111.856	121.04	120.6546	112.0493	335.0116	217.118	398.7739	175.7349	160.3812	140.5388	159.4149	115.4233	178.5895	138.3403	162.2317
AC064847.	0.960908	1.00039	0	2.078586	6.64491	4.567548	0.84968	0	45.52422	0.960699	14.45706	1.840156	0	0.93072	0.978006	0.824452	2.052753	1.820267	2.688924
AC064850.	16.33543	17.00664	12.98874	3.117878	6.64491	3.425661	7.647122	18.52054	4.669151	13.44979	13.2523	26.68227	12.33701	11.16864	8.802052	17.31349	13.34289	7.281067	17.02985
AC064852.	0	0	0.999134	0	1.107485	0	0.84968	0.926027	0	0	0	0	1.028084	1.861441	0	2.473355	1.026376	0	1.792616
AC064853.	0	0	0	0	0	0	0	0	0	0.960699	0	0.920078	0	0	0	3.297807	1.026376	0	0
AC064853.	2.882723	4.001562	0	0	2.21497	1.141887	0	5.556162	0	0	0	4.600391	10.28084	5.584322	3.912023	0.824452	0	1.820267	1.792616
AC064853.	0	0	0	0	0	0	0	0	0	0	0	0.920078	0	0	0	2.473355	0	0.910133	0
AC064853.	0	0	0	0	0	0	0.84968	0	0	0	0	0	0	0	0	0	0	0.910133	0
AC064862.	0	0	0	0	0	0	0	0	0	0	0	0	2.056169	0.93072	0	0	6.158258	4.550667	0
AC064862.	0	0	0	0	0	4.567548	0	0	0	0	0	0	0	0	0	0	0	0	0
AC064862.	0	0	0.999134	0	0	0	0.84968	0	0	0	0	0	0	0	0	0	0	0	4.48154
AC064871.	1.921815	8.003124	7.993069	2.078586	0	2.283774	10.19616	3.704108	2.334575	0.960699	14.45706	7.360626	3.084253	12.09936	12.71408	1.648904	6.158258	5.4608	3.585232
AC064874.	2.882723	9.003514	0	2.078586	1.107485	1.141887	1.69936	7.408216	2.334575	0	1.204755	1.840156	1.028084	0	0.978006	1.648904	0	0	0
AC064875.	733.1724	595.2323	793.3121	861.5737	634.5889	678.2809	659.3519	585.2491	831.1089	485.1531	532.5017	730.5421	819.3832	786.4587	805.8768	934.1039	807.7581	584.3057	509.9992
AC066595.	7.68726	13.00508	3.996535	0	1.107485	1.141887	0	7.408216	2.334575	8.646293	8.433284	0	1.028084	3.722881	0	0	1.026376	0	0.896308
AC066612.	52.84991	38.01484	36.96795	93.53635	40.97694	39.96605	101.9616	30.55889	18.6766	17.29259	18.07132	69.00587	51.40422	49.32818	31.29619	74.20066	51.31881	52.78774	37.64493
AC066612.	341.1222	484.189	377.6725	667.226	232.5718	541.2544	424.8401	447.2711	178.595	352.5766	220.4701	266.8227	280.667	515.619	418.5865	373.4767	338.7042	91.92348	177.469
AC066613.	367.0667	280.1093	591.4871	332.5737	246.9691	357.4106	283.7932	375.967	358.3573	359.3015	325.2838	324.7876	303.2849	240.1258	291.4457	354.5143	300.7282	370.4243	249.1736
AC066613.	26.90541	23.00898	42.96275	19.74656	27.68712	19.41208	22.94137	39.81916	3.501863	8.646293	18.07132	29.4425	16.44935	18.61441	27.38416	19.78684	17.4484	31.85467	23.30401
AC066615.	0.960908	2.000781	0	0	1.107485	2.283774	1.69936	3.704108	0	0	0	3.680313	3.084253	0	0	0	1.026376	0.910133	0.896308
AC066616.	0	0	0	0	0	0	0	0	0	0	0	0	0	0	0	1.648904	0	0	0
AC066616.	0	0	0	0	0	0	0	0	0	0	0	0	0	0	0	0	0	0	1.792616
AC066692.	0	0	0	4.157171	0	0	0	0	0	0.960699	0	1.840156	2.056169	1.861441	0	0	0	0	0
AC067735.	77.83351	76.02967	110.9038	67.55403	98.56616	44.53359	91.76547	61.11778	120.2306	106.6376	109.6327	92.00782	84.30292	81.90339	65.52639	112.1254	105.7168	183.847	171.1948
AC067747.	16.33543	14.00547	23.97921	15.58939	16.61227	1.141887	19.54265	23.15068	44.35693	52.83846	22.89034	37.72321	17.47743	40.02097	19.56012	22.2602	21.5539	31.85467	13.44462
AC067750.	788.9051	617.2409	1033.104	589.279	605.7943	495.579	882.8178	740.8216	643.1755	748.3847	799.9572	866.7137	1015.747	759.4678	821.5249	498.7934	581.9553	759.9614	654.3048
AC067773.	0	0	7.993069	1.039293	6.64491	1.141887	1.69936	0	0	7.685594	1.204755	3.680313	4.112337	0.93072	0.978006	1.648904	1.026376	2.7304	0
AC067805.	0	0	0	1.039293	0	0	0	0	0	0	0	0.920078	0	0	0	0	0	0	0
AC067817.	18.25724	4.001562	3.996535	1.039293	0	4.567548	9.346483	0.926027	0	0.960699	8.433284	10.12086	3.084253	5.584322	4.890029	18.96239	5.131881	0.910133	7.170464
AC067817.	140.2925	340.1327	347.6985	156.9332	434.1341	203.2559	571.8348	278.7341	110.8923	220.0001	212.0369	308.2262	243.656	313.6527	419.5645	132.7367	141.6399	169.2848	199.8767
AC067838.	203.7124	168.0656	219.8094	206.8193	183.8425	179.2763	166.5373	177.7972	359.5246	331.4412	240.951	161.0137	234.4032	169.3911	252.3255	126.1411	161.1411	92.83361	110.2459
AC067852.	0	2.000781	0	0	1.107485	0	0	0	0	0.960699	12.04755	1.840156	0	0.93072	0.978006	0	0	0.910133	0.896308
AC067852.	0.960908	0	0	0	0	0	0	0	0	0	0	0	1.028084	0.93072	0.978006	0.824452	0	0.910133	0
AC067852.	54.77173	18.00703	80.92983	88.33989	11.07485	20.55397	35.68657	24.0767	19.84389	23.05678	21.68559	42.3236	29.81445	32.57521	22.49413	48.64266	92.37386	38.2256	32.26709
AC067852.	700.5016	489.1909	811.2965	955.1101	561.4949	473.8831	1130.075	734.3394	217.1155	433.2754	332.5123	1019.447	865.647	648.7121	707.0982	1079.207	1029.455	641.6441	698.2239
AC067852.	8.648168	3.001171	0.999134	5.196464	0	6.851322	0	0.926027	11.67288	0	0	192.2964	246.7402	7.445762	1.956012	3.297807	6.158258	4.550667	0.896308
AC067863.	0	1.00039	0	0	0	5.709435	3.398721	0	0	0	6.023774	0	0	0	3.912023	1.648904	0	0.910133	0.896308
AC067863.	0	1.00039	0.999134	3.117878	0	6.851322	5.098081	1.852054	0	0	0	0	2.056169	4.653602	0.978006	0.824452	0	0	0
AC067904.	3.84363	2.000781	3.996535	0	4.42994	11.41887	0.84968	0	10.50559	0	1.204755	1.840156	4.112337	4.653602	3.912023	4.122259	3.079129	2.7304	3.585232
AC067930.	35.55358	39.01523	92.91943	23.90373	64.23413	12.56076	38.23561	85.19449	16.34203	11.52839	16.86657	46.92399	39.06721	42.81313	50.8563	45.34485	56.45069	75.54107	33.16339
AC067930.	35.55358	8.003124	37.96708	9.353635	13.28982	41.10793	20.39233	12.96438	32.68406	11.52839	18.07132	57.04485	25.70211	21.40657	18.58211	36.27588	39.0023	109.216	86.04556
AC067930.	44.20175	44.01718	32.97141	24.94303	32.11706	63.94567	25.49041	45.37532	8.171014	25.93888	36.14265	28.52243	12.33701	27.92161	45.96627	42.8715	39.0023	27.304	41.23017
AC067930.	27.86632	29.01132	40.96448	4.157171	14.3973	29.68906	27.18977	34.263	63.03354	77.81664	116.8612	56.12477	26.73019	53.05106	27.38416	14.01568	42.08143	27.304	27.78555
AC067931.	1.921815	15.00586	14.987	1.039293	19.93473	13.70264	3.398721	6.482189	0	21.13538	31.32363	4.600391	11.30893	18.61441	11.73607	3.297807	5.131881	9.101334	2.688924

AC067931.	54.77173	69.02694	88.9229	87.30059	64.23413	59.37812	50.13113	60.19176	103.8886	122.0088	68.67103	49.68422	85.331	65.15042	87.04252	56.06272	65.68808	120.1376	87.83818
AC067931.	2.882723	1.00039	0	5.196464	0	0	9.346483	7.408216	0	7.685594	0	10.12086	14.39318	3.722881	1.956012	0.824452	8.21101	0.910133	4.48154
AC067940.	0	0	0.999134	8.314342	4.42994	0	5.098081	1.852054	1.167288	0	0	3.680313	0	6.515042	0.978006	1.648904	2.052753	0.910133	0.896308
AC067942.	5.765445	8.003124	9.991337	8.314342	4.42994	9.135096	14.44456	10.1863	2.334575	2.882098	21.68559	11.04094	23.64594	22.33729	29.34017	11.54233	10.26376	15.47227	5.377848
AC067942.	2.882723	5.001952	0	0	1.107485	1.141887	0	0	1.167288	0	0	0.920078	0	0	4.890029	0.824452	0	4.550667	4.48154
AC067942.	0	0	0	0	0	0	1.69936	0	0	0	0	0	0	0	0	0	0	0	0
AC067945.	3.84363	6.002343	3.996535	5.196464	17.71976	0	5.098081	10.1863	0	7.685594	1.204755	2.760235	2.056169	1.861441	2.934017	1.648904	1.026376	0	0
AC067945.	0	0	0.999134	0	0	0	0	2.778081	0	0	0	0	0	0	0	0	0	1.820267	0
AC067945.	9.609075	11.00429	15.98614	10.39293	0	4.567548	9.346483	16.66849	9.338302	7.685594	1.204755	11.04094	9.252759	17.68369	16.6261	4.946711	14.36927	11.83173	17.92616
AC067956.	0.960908	1.00039	0.999134	2.078586	1.107485	1.141887	5.098081	6.482189	0	0.960699	1.204755	1.840156	14.39318	4.653602	0	4.946711	5.131881	0	0.896308
AC067960.	0	0	0	0	0	0	0	0	0	0	0	0	0	5.140422	0	0	0	0	0
AC067968.	1.921815	1.00039	3.996535	0	1.107485	1.141887	4.248401	3.704108	0	1.921399	1.204755	3.680313	7.196591	0.93072	0	0.824452	2.052753	0.910133	2.688924
AC067969.	19.21815	27.01054	46.95928	37.41454	26.57964	34.25661	26.34009	32.41095	50.19337	17.29259	45.78069	49.68422	30.84253	26.06017	20.53812	34.62698	32.84404	20.93307	18.82247
AC067969.	2.882723	0	4.995668	0	8.859879	2.283774	0.84968	0	0	2.882098	4.81902	3.680313	3.084253	0	0	0	5.131881	7.281067	0
AC068014.	2.882723	1.00039	0.999134	2.078586	0	5.709435	2.549041	0	0	0	3.614265	3.680313	1.028084	1.861441	1.956012	0.824452	5.131881	0.910133	7.170464
AC068020.	4.804538	1.00039	0	0	1.107485	7.993209	2.549041	4.630135	1.167288	5.764196	7.228529	3.680313	3.084253	2.792161	1.956012	0.824452	2.052753	5.4608	1.792616
AC068025.	15.37452	14.00547	12.98874	6.235757	4.42994	4.567548	3.398721	0.926027	3.501863	17.29259	13.2523	2.760235	8.224675	11.16864	6.846041	7.420066	9.237386	21.8432	3.585232
AC068025.	3.84363	0	1.998267	1.039293	4.42994	0	1.69936	1.852054	4.669151	0	0	1.840156	2.056169	4.653602	1.956012	2.473355	4.105505	4.550667	4.48154
AC068050.	0	0	0.999134	2.078586	6.64491	1.141887	0.84968	0	1.167288	6.724895	0	0.920078	1.028084	0.93072	0.978006	0	0	0.910133	0
AC068051.	6.726353	3.001171	3.996535	3.117878	0	4.567548	0	3.704108	0	0.960699	0	6.440548	0	5.584322	0	3.297807	2.052753	0.910133	0
AC068057.	0	11.00429	5.994802	4.157171	25.47215	0	0	5.556162	14.00745	0.960699	0	3.680313	8.224675	2.792161	0	8.244518	7.184634	7.281067	4.48154
AC068057.	0	0	0	0	0	0	0	0	0	0	0	0	0	0	0	0	1.026376	1.820267	0
AC068058.	0	0	0	0	0	3.425661	0.84968	1.852054	0	0	0	0	0	0	2.934017	0.824452	0	0	0
AC068075.	0	0	0	4.157171	0	0	0	0	0	0	0	0	0	3.722881	0.978006	0	0	0.910133	0.896308
AC068112.	0.960908	0	0	0	0	1.141887	0	0	0	0	0	0	0	0	0.978006	0	0	0	0
AC068134.	0	0	0	0	3.322455	0	0	3.704108	0	0	0	0	0	0	0	0	0	0	0
AC068134.	119.1525	96.03748	79.93069	60.27898	81.95388	93.63473	88.36674	73.15614	103.8886	77.81664	45.78069	90.16767	78.13441	69.80402	72.37243	122.8433	90.32111	59.15867	34.95601
AC068134.	0	0	0	0	0	0	0	0	1.167288	0	1.204755	0	0	0	0.978006	0	0	0	0.896308
AC068137.	0.960908	1.00039	0	1.039293	0	3.425661	4.248401	0	0	0	2.40951	0	2.056169	0.93072	0	0	1.026376	0.910133	0.896308
AC068138.	0	0	0	0	0	2.283774	0	0	0	0.960699	0	0	0	0	0	0.824452	0	0	0.896308
AC068152.	1107.926	1006.393	1169.986	1468.521	1100.84	800.4628	1222.69	1258.471	799.5921	564.8912	597.5584	1284.429	1220.336	1067.536	914.4354	1155.057	979.1629	1064.856	1214.497
AC068189.	16.33543	11.00429	17.98441	13.51081	23.25718	12.56076	3.398721	21.29862	0	0.960699	6.023774	6.440548	13.3651	20.47585	19.56012	7.420066	8.21101	29.12427	17.92616
AC068189.	2.882723	3.001171	2.997401	0	1.107485	0	0.84968	5.556162	0	0	0	0.920078	5.140422	1.861441	1.956012	0.824452	3.079129	0.910133	5.377848
AC068196.	0	0	4.995668	0	2.21497	1.141887	0	9.26027	21.01118	12.48909	19.27608	0.920078	1.028084	6.515042	7.824047	0	4.105505	4.550667	0
AC068205.	0	0	0	2.078586	1.107485	0	4.248401	0	0	0	0	0	0	0.93072	0	0.824452	1.026376	0.910133	3.585232
AC068205.	406.4639	496.1937	701.3918	219.2908	613.5466	446.4778	309.2836	615.808	245.1304	612.9261	551.7777	552.967	544.8847	521.2034	491.9369	338.8497	385.9175	384.0763	313.7078
AC068213.	0.960908	0	0	1.039293	0	0	0	0	25.68033	0.960699	2.40951	0.920078	0	0	0	1.648904	0	7.281067	0
AC068228.	5.765445	4.001562	1.998267	1.039293	9.967364	1.141887	0.84968	12.96438	0	3.842797	8.433284	7.360626	5.140422	4.653602	1.956012	7.420066	1.026376	5.4608	0
AC068228.	0	2.000781	0	0	0	0	0	0	0	0	0	0	0	0	0	0	0	0	0
AC068234.	2.882723	2.000781	8.992203	4.157171	6.64491	0	5.098081	9.26027	10.50559	6.724895	14.45706	1.840156	8.224675	3.722881	8.802052	13.19123	9.237386	7.281067	1.792616
AC068254.	0.960908	0	2.997401	0	1.107485	0	4.248401	0	5.836439	0.960699	0	0	0	0.93072	3.912023	5.771163	1.026376	0.910133	0
AC068254.	21.13997	16.00625	23.97921	19.74656	11.07485	6.851322	5.098081	22.22465	24.51304	15.37119	8.433284	19.32164	28.78636	19.54513	33.2522	8.244518	10.26376	14.56213	11.652
AC068276.	0.960908	3.001171	0	1.039293	0	0	0	3.704108	0	0	1.204755	0	0	0	0.978006	1.648904	7.184634	0	0
AC068279.	0	5.001952	0	2.078586	0	0	6.797442	0	14.00745	0	1.204755	3.680313	11.30893	0	0	0.824452	2.052753	49.1472	57.36371
AC068282.	172.0024	154.0601	156.864	386.6169	138.4356	207.8234	103.661	150.9424	381.7031	170.0438	197.5798	174.8149	207.673	202.897	198.5352	149.2258	146.7718	158.3632	146.9945
AC068286.	0	0	0.999134	0	0	0	0	0	0	0.960699	0	0	0	0	0	0.824452	0	0	0.896308
AC068298.	0	0	0	0	0	0	0.84968	2.778081	0	0	0	0.920078	0	0	0	0	0	0	0
AC068299.	2.882723	0	2.997401	30.13949	8.859879	5.709435	4.248401	0	0	16.33189	1.204755	6.440548	2.056169	2.792161	6.846041	3.297807	0	2.7304	8.963079
AC068305.	0.960908	0	0	0	0	0	0	0	1.167288	0	0	0	0	0	0	0	0	0	0
AC068305.	8.648168	14.00547	1.998267	11.43222	13.28982	27.40529	6.797442	5.556162	4.669151	11.52839	2.40951	1.840156	2.056169	10.23792	22.49413	4.946711	10.26376	11.83173	2.688924
AC068313.	4.804538	2.000781	6.993936	16.62868	25.47215	12.56076	22.94137	12.03835	7.003726	13.44979	6.023774	15.64133	19.5336	10.23792	6.846041	14.84013	14.36927	7.281067	10.7557
AC068338.	11.53089	11.00429	7.993069	7.275049	17.71976	12.56076	12.7452	11.11232	0	7.685594	9.638039	6.440548	11.30893	9.307203	10.75806	9.893422	12.31652	4.550667	1.792616
AC068338.	93.20803	107.0418	118.8969	108.0864	76.41646	77.64832	113.8572	75.00819	194.9371	112.4018	104.8137	84.6472	52.4323	90.27987	84.1085	102.232	114.9541	62.79921	126.3794
AC068338.	41.31902	57.02225	53.95322	27.02161	49.83682	67.37133	28.03945	46.30135	138.9072	55.72056	79.51382	22.08188	60.65698	25.12945	40.09824	34.62698	47.21331	23.66347	45.71171
AC068389.	0	4.001562	12.98874	4.157171	6.64491	2.283774	5.098081	0.926027	1.167288	5.764196	9.638039	0.920078	0	3.722881	0	0.824452	4.105505	0	1.792616
AC068389.	0.960908	5.001952	0.999134	3.117878	8.859879	6.851322	0	1.852054	0	12.48909	0	6.440548	1.028084	1.861441	0	0.824452	6.158258	0	0
AC068389.	0.960908	0	0.999134	0	0	1.141887	0.84968	0	0	0	0	0.920078	1.028084	0	0.978006	0.824452	0	0	0.896308
AC068389.	0	1.00039	2.997401	3.117878	0	0	0.84968	0	3.501863	0	0	4.600391	1.028084	0	0.978006	1.648904	2.052753	0	0
AC068397.	0	1.00039	0	0	0	4.567548	0	0.926027	17.50932	0	0	0	0	3.722881	3.912023	1.648904	0	0	0.896308
AC068397.	1.921815	1.00039	0	0	0	0	0.84968	0	0	0	3.614265	1.840156	4.112337	2.792161	0	0	1.026376	0	0.896308
AC068408.	0.960908	0	0.999134	1.039293	6.64491	1.141887	2.549041	0	2.334575	2.882098	0	0	0	0	0	0.824452	14.36927	0	3.585232
AC068413.	3.84363	8.003124	9.991337	1.039293	0	3.425661	2.549041	2.778081	0	2.882098	4.81902	5.520469	2.056169	1.861441	2.934017	0	1.026376	0	0

AC068418.	87.44259	100.039	81.92896	80.02554	87.49131	103.9117	90.91579	55.56162	126.0671	67.24895	84.33284	66.24563	74.02207	137.7466	103.6686	54.41382	36.94955	74.63094	108.4533
AC068446.	44.20175	78.03045	108.9056	31.17878	77.52394	19.41208	65.42538	40.74519	22.17847	38.42797	22.89034	103.0488	91.49951	49.32818	80.19648	31.32917	25.65941	28.21414	69.01571
AC068446.	36.51449	44.01718	85.9255	28.0609	26.57964	47.95925	36.53625	36.11505	44.35693	30.74238	6.023774	109.4893	103.8365	23.26801	26.40616	55.23827	78.0046	15.47227	18.82247
AC068446.	9.609075	1.00039	3.996535	11.43222	7.752394	1.141887	0	6.482189	0	0	1.204755	5.520469	0	1.861441	3.912023	2.473355	1.026376	5.4608	0
AC068448.	9.609075	2.000781	3.996535	17.66798	0	15.98642	22.94137	22.22465	0	10.56769	1.204755	0.920078	3.084253	1.861441	0.978006	10.71787	13.34289	2.7304	3.585232
AC068473.	5.765445	2.000781	1.998267	0	1.107485	0	1.69936	0.926027	2.334575	0	0	0.920078	0	0	0.978006	0	0	0	4.48154
AC068473.	0.960908	4.001562	1.998267	1.039293	0	1.141887	0	0	0	0	0	3.680313	0	0	1.956012	0	2.052753	0.910133	5.377848
AC068473.	126.8398	152.0593	132.8848	96.65423	49.83682	62.80379	172.4851	125.9397	68.86998	106.6376	209.6273	236.4601	193.2799	105.1714	92.91055	123.6678	147.7982	45.50667	88.73449
AC068473.	14.41361	18.00703	28.97488	4.157171	1.107485	0	7.647122	6.482189	2.334575	0	0	0.920078	6.168506	11.16864	3.912023	5.771163	2.052753	0	0.896308
AC068473.	877.3086	916.3576	1696.529	1140.104	1472.955	1040.259	922.7527	773.2326	1468.448	1630.307	1519.196	1383.798	1463.992	1152.232	1082.652	946.4707	1000.717	972.0225	943.8123
AC068481.	10.56998	7.002733	19.98267	11.43222	22.1497	4.567548	25.49041	4.630135	2.334575	5.764196	7.228529	11.04094	4.112337	9.307203	13.69208	8.244518	7.184634	9.101334	19.71877
AC068489.	0	0	1.998267	1.039293	0	2.283774	0	0	0	0	0	1.840156	0	0	0.978006	0	2.052753	0	0
AC068490.	0	0	0	0	0	0	0.84968	0	0	0	0	0	0	0	0	0.824452	0	0	0
AC068491.	0	1.00039	0	2.078586	4.42994	1.141887	1.69936	0	0	0	0	0.920078	1.028084	1.861441	0	4.946711	1.026376	0	0.896308
AC068491.	0	0	0	0	0	0	2.549041	0	0	1.921399	0	0.920078	0	0	0	0	1.026376	0	0
AC068491.	0	0	0	0	0	0	0	0	0	0	0	0.920078	0	2.792161	1.956012	0	4.105505	0	0.896308
AC068491.	12.4918	9.003514	9.991337	12.47151	27.68712	28.54718	16.9936	50.00546	60.69896	28.82098	13.2523	15.64133	15.42127	53.05106	43.03226	28.85581	20.52753	35.4952	2.688924
AC068492.	0	1.00039	0.999134	0	2.21497	2.283774	0.84968	0	0	0	0	0.920078	0	0.93072	0	0	1.026376	0	1.792616
AC068506.	0	10.0039	1.998267	3.117878	0	2.283774	2.549041	0.926027	7.003726	5.764196	7.228529	6.440548	3.084253	5.584322	0.978006	4.122259	1.026376	2.7304	5.377848
AC068512.	0	0	0	0	0	0	0	0	0	0	0	0	0	0	0	0	0	5.4608	0
AC068522.	7.68726	5.001952	26.97661	12.47151	21.04221	0	15.29424	23.15068	1.167288	15.37119	12.04755	44.16376	14.39318	13.9608	7.824047	5.771163	3.079129	28.21414	8.963079
AC068531.	0.960908	9.003514	2.997401	1.039293	0	0	0.84968	0.926027	0	6.724895	6.023774	2.760235	3.084253	0	0	1.648904	0	2.7304	2.688924
AC068533.	0	0	0.999134	0	3.322455	0	0	0	0	0	4.81902	0	0	2.792161	1.956012	0	0	0	0
AC068533.	3.84363	11.00429	0	3.117878	6.64491	6.851322	0.84968	2.778081	5.836439	0	1.204755	11.04094	5.140422	1.861441	2.934017	6.595615	1.026376	0	4.48154
AC068533.	5.765445	7.002733	31.97228	10.39293	27.68712	6.851322	11.04584	4.630135	18.6766	16.33189	3.614265	11.96102	10.28084	13.9608	6.846041	9.893422	9.237386	10.01147	16.13354
AC068535.	10.56998	6.002343	0	1.039293	15.50479	5.709435	0	2.778081	2.334575	1.921399	3.614265	0.920078	0	6.515042	1.956012	1.648904	0	10.9216	4.48154
AC068538.	1.921815	0	1.998267	4.157171	0	4.567548	1.69936	2.778081	0	0	0	0.920078	3.084253	0.93072	0.978006	2.473355	7.184634	1.820267	1.792616
AC068544.	0	0	0	0	4.42994	0	0	0	0	0	0	1.840156	0	0	0	3.297807	2.052753	0.910133	0
AC068580.	12.4918	14.00547	32.97141	7.275049	17.71976	10.27698	13.59488	26.85478	26.84762	35.54587	83.12809	36.80313	31.87062	29.78305	12.71408	23.08465	29.76491	23.66347	24.20031
AC068580.	17.29634	0	19.98267	6.235757	1.107485	12.56076	1.69936	3.704108	0	2.882098	1.204755	21.1618	1.028084	10.23792	0.978006	19.78684	25.65941	13.652	17.92616
AC068580.	294.0377	274.107	250.7825	166.2868	413.0919	271.7691	213.2697	173.1671	252.1342	311.2666	450.5783	478.4407	413.2899	499.7968	377.5102	250.6334	354.0998	322.1872	354.0416
AC068580.	438.1738	140.0547	393.6587	259.8232	211.5296	114.1887	361.9638	123.1616	247.465	138.3407	221.6749	279.7038	310.4815	429.0621	485.0909	306.6961	231.961	311.2656	183.7431
AC068587.	983.0084	710.2772	620.462	647.4794	903.7077	1001.435	552.2922	838.9805	632.67	603.3191	544.5492	627.4934	683.6761	775.29	711.9882	643.8969	585.0345	753.5905	521.6512
AC068587.	929.1976	683.2667	525.5443	574.7289	835.0436	936.3473	478.37	746.3778	541.6215	526.4632	502.3828	565.8481	583.9519	765.0521	668.956	591.9564	534.742	688.971	472.3543
AC068587.	45.16265	42.0164	63.94455	72.75049	54.26676	52.5268	70.52346	80.56435	173.9259	73.01314	91.56137	32.20274	48.31997	19.54513	17.6041	37.92478	20.52753	69.17014	25.99293
AC068587.	5.765445	0	9.991337	3.117878	5.537425	5.709435	4.248401	3.704108	21.01118	10.56769	6.023774	3.680313	4.112337	2.792161	1.956012	0	0	0	0.896308
AC068587.	501.5937	940.367	1205.954	1205.58	955.7595	1549.541	736.6728	1312.18	1435.764	1450.656	1597.505	475.6804	515.0703	554.7093	559.4193	539.1915	645.5907	455.0667	545.8515
AC068594.	0	1.00039	0.999134	2.078586	0	0	0	0	0	0.960699	3.614265	0.920078	0	3.722881	1.956012	1.648904	2.052753	7.281067	4.48154
AC068594.	0	0	0	0	0	1.141887	0	0	0	0	0	0	0	0	0	4.122259	0	0.910133	0
AC068599.	0	2.000781	0	0	0	0	0	0	0	0	0	0.920078	0	0	0	0	0	0	0
AC068620.	9.609075	6.002343	17.98441	3.117878	5.537425	9.135096	8.496802	0	4.669151	4.803496	7.228529	4.600391	4.112337	10.23792	4.890029	3.297807	7.184634	2.7304	4.48154
AC068620.	62.45899	129.0504	177.8458	124.7151	117.3934	52.5268	235.3614	124.0876	72.37184	78.77734	53.00921	215.2983	112.0612	175.9061	168.217	112.9499	111.875	121.0477	164.0244
AC068620.	44.20175	70.02733	122.8934	50.92535	78.63143	53.66869	43.33369	97.23284	120.2306	101.8341	79.51382	59.80509	79.1625	91.21059	88.02052	47.81821	19.50115	93.74374	62.74156
AC068620.	64.3808	81.03163	115.8995	62.35757	71.98652	66.22945	93.46483	130.5698	59.53167	23.05678	83.12809	117.77	98.6961	48.39746	122.2507	92.3386	101.6112	77.36134	81.56402
AC068631.	80.71623	70.02733	28.97488	35.33595	32.11706	74.22266	113.0075	36.11505	57.1971	44.19217	14.45706	39.56336	46.2638	39.09025	14.67009	98.93422	56.45069	46.4168	46.60801
AC068631.	0	0	0	2.078586	4.42994	4.567548	1.69936	0.926027	4.669151	2.882098	1.204755	1.840156	2.056169	0.93072	0.978006	0.824452	3.079129	1.820267	1.792616
AC068633.	0	0	0	0	0	0	0.84968	0	0	0	0	0	0	0	0.978006	0.824452	0	0	0
AC068643.	0.960908	0	0.999134	0	0	4.567548	0.84968	1.852054	0	5.764196	0	0	2.056169	0	0.978006	0	0	0	0
AC068643.	3.84363	0	0	0	0	0	0	0	0	0	0	0	0	0	0	0	0	0	0
AC068647.	0	0	4.995668	0	0	0	0.84968	0	0	1.921399	0	0	1.028084	0	0.978006	0	0	0	0
AC068647.	0.960908	0	0	4.157171	0	4.567548	1.69936	0	0	4.803496	0	0.920078	0	0	0	1.648904	0	4.550667	0
AC068657.	5.765445	15.00586	2.997401	13.51081	21.04221	12.56076	6.797442	11.11232	9.338302	1.921399	2.40951	7.360626	4.112337	16.75297	6.846041	9.06897	1.026376	14.56213	2.688924
AC068658.	5.765445	1.00039	4.995668	6.235757	9.967364	0	8.496802	5.556162	3.501863	3.842797	4.81902	3.680313	4.112337	0.93072	2.934017	0.824452	1.026376	0	3.585232
AC068669.	98.97348	147.0574	107.9064	69.63262	122.9308	76.50643	66.27506	100.0109	137.74	137.38	143.3658	70.84602	106.9208	129.3701	67.4824	88.21635	58.50345	74.63094	102.1791
AC068672.	0.960908	0	6.993936	6.235757	0	0	15.29424	1.852054	0	0	0	0	0	0	0.978006	2.473355	0	0	0.896308
AC068672.	0	0	0	2.078586	0	0	0.84968	0	0	0	0	0	0	0	0	0	0	0	0
AC068675.	24.9836	19.00742	15.98614	19.74656	6.64491	18.27019	36.53625	19.44657	15.17474	26.89958	13.2523	27.60235	39.06721	13.9608	20.53812	28.85581	16.42202	17.29253	24.20031
AC068700.	1591.263	2160.843	2547.791	2303.073	2044.417	2192.423	3123.425	2298.399	645.5101	1410.307	998.7418	2066.496	2279.263	1721.833	1795.619	1376.01	1372.265	1197.736	1778.275
AC068707.	1.921815	0	0.999134	2.078586	0	3.425661	9.346483	9.26027	0	0	2.40951	2.760235	0	3.722881	3.912023	4.122259	1.026376	0	2.688924
AC068722.	1.921815	6.002343	8.992203	11.43222	6.64491	2.283774	13.59488	1.852054	1.167288	0	0	1.840156	2.056169	0.93072	4.890029	1.648904	3.079129	8.191201	2.688924
AC068722.	21.13997	15.00586	40.96448	46.76817	46.51437	28.54718	19.54265	50.93149	61.86625	28.82098	78.30907	30.36258	38.03912	8.376483	26.40616	57.71163	39.0023	21.8432	22.4077
AC068724.	11.53089	24.00937	18.98354	5.196464	22.1497	5.709435	8.496802	11.11232	14.00745	5.764196	10.84279	4.600391	12.33701	13.9608	4.890029	4.946711	27.71216	8.191201	7.170464

AC068724.	0	7.002733	10.99047	1.039293	4.42994	14.84453	4.248401	3.704108	10.50559	22.09608	16.86657	10.12086	12.33701	19.54513	2.934017	2.473355	2.052753	2.7304	4.48154
AC068726.	0	1.00039	1.998267	1.039293	2.21497	0	0.84968	0	18.6766	0	0	0	4.112337	0.93072	0.978006	0	0	0.910133	0
AC068733.	0.960908	2.000781	0	5.196464	0	0	2.549041	0	23.34575	0.960699	0	0.920078	0	3.722881	0	0.824452	6.158258	1.820267	0
AC068733.	0.960908	0	17.98441	0	4.42994	0	0.84968	3.704108	10.50559	3.842797	1.204755	0	0	0	0	0	7.184634	0.910133	0.896308
AC068733.	49.00628	60.02343	112.9021	21.82515	29.90209	25.12151	42.48401	44.4493	95.71759	45.15286	39.75691	31.28266	66.82548	39.09025	60.63636	27.20691	9.237386	17.29253	24.20031
AC068756.	0.960908	17.00664	5.994802	13.51081	12.18233	23.97963	16.14392	5.556162	10.50559	1.921399	4.81902	3.680313	1.028084	3.722881	0	3.297807	1.026376	10.9216	2.688924
AC068759.	3.84363	4.001562	0	0	0	1.141887	1.69936	0	0	1.921399	0	5.520469	2.056169	0	2.934017	0	0	2.7304	0.896308
AC068760.	0	0	0	0	2.21497	0	0	0	0	1.921399	0	0	0	0.93072	1.956012	2.473355	0	1.820267	0
AC068768.	425.682	339.1324	349.6968	330.4951	275.7637	343.708	270.1983	391.7094	304.6621	272.8386	248.1795	279.7038	255.993	325.7521	323.7199	359.461	335.625	303.9846	305.641
AC068768.	25.9445	13.00508	23.97921	58.2004	15.50479	3.425661	28.03945	13.89041	10.50559	24.97818	15.66181	22.08188	11.30893	17.68369	14.67009	27.20691	31.81766	11.83173	16.13354
AC068769.	0	1.00039	0	15.58939	0	1.141887	0.84968	0.926027	0	0	0	5.520469	3.084253	3.722881	1.956012	4.946711	1.026376	1.820267	11.652
AC068775.	78.79442	223.0871	37.96708	249.4303	28.79461	47.95925	73.0725	61.11778	12.84017	16.33189	22.89034	57.96493	31.87062	54.9125	33.2522	64.30724	74.92547	292.1528	240.2105
AC068775.	0	0	0	0	1.107485	0	0	0	0	0	0	0	0	0	0	0	0	0.910133	0
AC068787.	25.9445	44.01718	12.98874	102.89	36.547	67.37133	37.38593	15.74246	16.34203	38.42797	19.27608	17.48149	31.87062	13.03008	7.824047	54.41382	40.02867	7.281067	20.61508
AC068790.	19.21815	6.002343	12.98874	11.43222	15.50479	2.283774	14.44456	12.03835	0	0	0	13.80117	16.44935	5.584322	0	8.244518	2.052753	5.4608	10.7557
AC068790.	26.90541	45.01757	33.97054	58.2004	36.547	13.70264	95.16419	39.81916	28.01491	19.21399	45.78069	58.88501	27.75828	24.19873	26.40616	25.55801	56.45069	26.39387	34.0597
AC068790.	26.90541	44.01718	21.98094	38.45383	39.86946	26.2634	43.33369	68.526	11.67288	31.70308	16.86657	49.68422	38.03912	20.47585	35.20821	28.03136	27.71216	23.66347	39.43755
AC068790.	2.882723	9.003514	29.97401	5.196464	23.25718	6.851322	22.94137	10.1863	3.501863	3.842797	14.45706	31.28266	26.73019	20.47585	10.75806	21.43575	12.31652	0.910133	12.54831
AC068790.	28.82723	45.01757	39.96535	35.33595	40.97694	12.56076	58.62794	47.22738	38.5205	25.93888	12.04755	46.92399	50.37613	49.32818	39.12023	43.69595	45.16056	30.0344	36.74863
AC068790.	12.4918	28.01093	12.98874	14.5501	7.752394	7.993209	18.69297	25.00273	0	7.685594	6.023774	20.24172	14.39318	10.23792	2.934017	17.31349	18.47477	19.1128	20.61508
AC068790.	27.86632	27.01054	50.95582	21.82515	22.1497	25.12151	50.98081	25.00273	31.51677	14.41049	24.0951	52.44446	42.15146	19.54513	10.75806	14.01568	9.237386	13.652	22.4077
AC068790.	74.95079	69.02694	125.8908	122.6365	78.63143	66.22945	138.4979	69.45203	30.34948	58.60265	38.55216	91.08774	147.0161	48.39746	111.4927	129.4389	67.74083	81.91201	83.35664
AC068792.	43.24084	21.0082	23.97921	16.62868	22.1497	18.27019	33.98721	14.81643	14.00745	39.38867	32.52838	13.80117	39.06721	37.22881	19.56012	9.893422	17.4484	13.652	10.7557
AC068802.	0.960908	0	0.999134	0	0	1.141887	0	0	0	0	4.81902	0.920078	4.112337	0	0	0	0	0	0
AC068831.	34.59267	21.0082	37.96708	76.90766	36.547	66.22945	28.88913	50.00546	107.3905	54.75986	96.38039	35.88305	40.09529	54.9125	42.05425	47.81821	67.74083	21.8432	27.78555
AC068831.	15.37452	22.00859	10.99047	12.47151	11.07485	9.135096	7.647122	10.1863	4.669151	20.17468	30.11887	4.600391	10.28084	0.93072	3.912023	9.893422	11.29014	17.29253	20.61508
AC068831.	47.08447	73.0285	92.91943	34.29666	83.06137	106.1955	23.79105	120.3835	65.36811	180.6115	250.589	84.6472	80.19058	116.34	56.72434	23.08465	41.05505	35.4952	35.85232
AC068831.	0	2.000781	0	0	0	0	0	0	0	0	0	0	0	0	0	0	0	0	0
AC068831.	0	2.000781	0	0	6.64491	0	0	0	0	0	0	0.920078	0	0	0.978006	0.824452	0	12.74187	0
AC068831.	60.53717	95.03709	66.94196	100.8114	145.0805	119.8981	73.92218	102.789	155.2493	136.4193	101.1994	79.12673	70.93782	88.41843	77.26246	100.5831	132.4025	48.23707	60.94894
AC068858.	12.4918	0	0	10.39293	0	0	0	0	0	0	0	0	0	0	0	0	1.026376	0	0
AC068860.	41.31902	29.01132	54.95235	16.62868	24.36467	21.69585	31.43817	57.41368	42.02236	54.75986	57.82823	48.76415	58.60081	37.22881	68.46041	19.78684	27.71216	24.5736	18.82247
AC068870.	24.9836	25.00976	35.96881	30.13949	9.967364	22.83774	26.34009	28.70684	46.69151	46.11356	0	26.68227	13.3651	22.33729	26.40616	9.06897	18.47477	17.29253	33.16339
AC068870.	183.5333	215.0839	242.7895	151.7367	181.6275	138.1683	192.0277	168.5369	284.8182	182.5329	242.1557	251.1814	314.5938	214.9964	223.9633	115.4233	136.508	253.0171	231.2475
AC068870.	13.45271	17.00664	4.995668	10.39293	18.82724	2.283774	11.89552	16.66849	0	6.724895	0	5.520469	5.140422	6.515042	12.71408	1.648904	2.052753	17.29253	22.4077
AC068875.	0	0	0	0	0	0	2.549041	0	0	0.960699	0	0	0	0	0	0	0	0	0.896308
AC068880.	0	0	0	0	0	0	0.84968	0	0	0	0	0	0	0	0	0.824452	0	0.910133	0.896308
AC068880.	68.22443	61.02382	59.94802	43.6503	38.76197	59.37812	34.83689	61.11778	121.3979	73.01314	55.41872	23.00196	30.84253	52.12034	33.2522	42.04704	49.26606	20.93307	35.85232
AC068888.	7836.201	7395.887	9447.808	5033.295	6719.111	6665.194	8444.972	9338.057	9013.796	8207.254	7463.456	7300.821	7817.554	8370.898	7919.891	3080.976	3464.02	5965.925	5609.991
AC068888.	31.70995	8.003124	48.95755	13.51081	31.00958	7.993209	47.58209	3.704108	17.50932	41.31007	15.66181	24.84211	20.56169	13.9608	20.53812	7.420066	15.39564	18.20267	18.82247
AC068896.	63.4199	44.01718	50.95582	37.41454	66.4491	69.65511	39.08529	55.56162	134.2381	99.91272	126.4993	69.00587	52.4323	44.67457	63.57038	31.32917	65.68808	87.37281	68.1194
AC068898.	0	1.00039	0	3.117878	1.107485	2.283774	0.84968	0	0	0	0	0	1.028084	0	0	0	1.026376	0	0.896308
AC068944.	0	0	0	0	0	0	0.84968	0	0	0.960699	19.27608	1.840156	4.112337	0.93072	0.978006	5.771163	4.105505	4.550667	4.48154
AC068946.	54.77173	55.02147	32.97141	25.98232	54.26676	36.54038	26.34009	63.89587	16.34203	61.48475	22.89034	69.92595	56.54464	37.22881	26.40616	24.73355	24.63303	10.9216	27.78555
AC068985.	0	0	0.999134	2.078586	0	1.141887	0	0.926027	1.167288	0	0	0	0	0	0	0	0	0	0
AC068987.	143.1752	108.0422	180.8432	129.9116	132.8982	133.6008	134.2495	124.0876	137.74	147.9477	144.5706	226.3392	212.8135	173.114	155.5029	86.56744	92.37386	147.4416	207.0471
AC068987.	184.4942	137.0535	236.7947	145.501	167.2302	156.4385	172.4851	261.1396	107.3905	116.2446	98.7899	248.4211	265.2458	186.1441	236.6774	193.7462	189.8796	114.6768	98.59387
AC068987.	9.609075	2.000781	2.997401	1.039293	4.42994	0	2.549041	0	2.334575	0.960699	9.638039	2.760235	0	1.861441	1.956012	0.824452	3.079129	7.281067	7.170464
AC068992.	0	11.00429	3.996535	12.47151	1.107485	106.1955	10.19616	6.482189	17.50932	16.33189	4.81902	9.200782	0	2.792161	8.802052	1.648904	0	3.640534	8.963079
AC068993.	0.960908	0	0	0	1.107485	0	0.84968	1.852054	0	0	0	0.920078	0	2.792161	0.978006	0	0	0.910133	0
AC069023.	0.960908	1.00039	0.999134	12.47151	0	0	8.496802	5.556162	0	0	0	4.600391	6.168506	9.307203	1.956012	9.893422	4.105505	0.910133	3.585232
AC069029.	0	0	0	0	4.42994	0	0.84968	0	4.669151	0	0	0	0	1.861441	0.978006	0	1.026376	0.910133	0
AC069061.	0	1.00039	11.9896	9.353635	8.859879	3.425661	9.346483	1.852054	21.01118	0.960699	7.228529	2.760235	5.140422	0	9.780058	2.473355	3.079129	4.550667	1.792616
AC069061.	0	1.00039	0	0	1.107485	0	0	0	0	0	0	0	0	0	0	0.824452	1.026376	0	0
AC069079.	0	3.001171	2.997401	1.039293	14.3973	1.141887	0	5.556162	3.501863	5.764196	0	0.920078	1.028084	1.861441	6.846041	3.297807	1.026376	2.7304	8.066772
AC069113.	7.68726	4.001562	26.97661	0	3.322455	9.135096	36.53625	5.556162	11.67288	11.52839	3.614265	38.64329	30.84253	20.47585	11.73607	0	0	1.820267	0.896308
AC069113.	0.960908	2.000781	6.993936	2.078586	4.42994	0	10.19616	3.704108	4.669151	9.606993	0	12.8811	10.28084	13.03008	11.73607	0	2.052753	1.820267	1.792616
AC069113.	0	2.000781	22.98007	0	1.107485	10.27698	36.53625	1.852054	2.334575	0.960699	6.023774	25.76219	19.5336	19.54513	17.6041	0	0	3.640534	4.48154
AC069120.	22.10087	37.01445	20.98181	12.47151	19.93473	41.10793	8.496802	60.19176	31.51677	24.97818	22.89034	44.16376	35.98295	27.92161	30.31818	11.54233	31.81766	12.74187	17.02985
AC069120.	406.4639	33.01288	284.7531	44.68959	25.47215	21.69585	36.53625	16.66849	36.18592	13.44979	43.37118	18.40156	18.50552	319.2371	295.3578	26.38246	25.65941	24.5736	30.47447
AC069120.	18.25724	18.00703	23.97921	9.353635	31.00958	3.425661	14.44456	16.66849	3.501863	35.54587	30.11887	18.40156	25.70211	14.89152	16.6261	2.473355	6.158258	11.83173	16.13354
AC069133.	0.960908	0	0	1.039293	0	0	0	0	0	0	0	0	0	0	0	0	0	0.910133	0

AC069148.	5.765445	18.00703	27.97574	10.39293	6.64491	20.55397	7.647122	1.852054	25.68033	35.54587	167.4609	21.1618	3.084253	24.19873	12.71408	10.71787	17.4484	20.93307	41.23017
AC069154.	0	7.002733	0.999134	9.353635	15.50479	11.41887	5.098081	0	0	4.803496	4.81902	6.440548	1.028084	2.792161	7.824047	4.122259	4.105505	4.550667	1.792616
AC069155.	0	0	0	1.039293	8.859879	0	0	3.704108	0	0	0	0	1.028084	0.93072	0	0	0	0	0.896308
AC069185.	87.44259	69.02694	59.94802	71.7112	53.15928	55.95246	84.96802	53.70957	35.01863	43.23147	37.3474	69.00587	55.51656	64.2197	103.6686	48.64266	62.60895	118.3173	77.08248
AC069200.	65.34171	100.039	123.8926	78.98625	122.9308	73.08077	101.9616	87.04654	171.5913	105.6769	114.4517	77.28657	42.15146	60.49682	53.79032	62.65834	73.89909	49.1472	87.83818
AC069208.	31.70995	44.01718	57.94975	21.82515	42.08443	46.81737	34.83689	43.52327	12.84017	17.29259	38.55216	34.96297	35.98295	54.9125	27.38416	36.27588	21.5539	67.34987	51.98586
AC069209.	120.1134	118.0461	84.92636	125.7544	153.9404	166.7155	71.37314	149.0904	213.6137	154.6726	163.8467	79.12673	114.1174	99.58707	100.7346	99.75867	87.24198	77.36134	83.35664
AC069213.	3.84363	23.00898	40.96448	25.98232	12.18233	11.41887	45.88273	25.00273	8.171014	24.01748	8.433284	18.40156	25.70211	38.15953	15.64809	28.85581	24.63303	23.66347	45.71171
AC069213.	0.960908	0	0	0	0	0	0.84968	0	0	0	0	2.760235	0	0	0	0	0	0.910133	0.896308
AC069213.	0.960908	0	0.999134	0	1.107485	2.283774	0	0	7.003726	0	12.04755	0	0	0	0	0.824452	0	0.910133	0.896308
AC069218.	0.960908	0	0.999134	8.314342	0	1.141887	0.84968	0	1.167288	0.960699	1.204755	0	1.028084	0	5.868035	0	1.026376	2.7304	0
AC069222.	76.8726	100.039	143.8752	48.84676	134.0057	82.21586	91.76547	164.8328	113.2269	122.0088	95.17564	106.7291	94.58376	125.6472	122.2507	47.81821	53.37157	78.27147	75.28987
AC069224.	219.0869	373.1456	435.6223	204.7407	355.5027	270.6272	228.564	289.8465	340.848	392.926	390.3406	286.1443	297.1164	345.2972	352.0821	202.8152	217.5918	384.0763	329.8413
AC069228.	7.68726	10.0039	10.99047	11.43222	9.967364	1.141887	13.59488	8.334243	4.669151	0.960699	1.204755	14.72125	19.5336	7.445762	20.53812	9.893422	12.31652	1.820267	6.274156
AC069234.	1.921815	7.002733	4.995668	6.235757	0	6.851322	9.346483	3.704108	1.167288	7.685594	9.638039	2.760235	3.084253	3.722881	2.934017	4.122259	1.026376	5.4608	2.688924
AC069234.	14.41361	23.00898	13.98787	31.17878	24.36467	23.97963	19.54265	23.15068	29.18219	22.09608	31.32363	10.12086	10.28084	21.40657	12.71408	18.96239	11.29014	19.1128	2.688924
AC069234.	49.96719	36.01406	30.97314	59.23969	71.98652	38.82416	52.68017	29.63287	44.35693	24.01748	32.52838	41.40352	35.98295	60.49682	38.14223	28.03136	27.71216	40.04587	58.26002
AC069235.	0	2.000781	0	0	0	0	6.851322	1.69936	0	0	0	0	0.920078	0	0	0	0	1.026376	0	0
AC069236.	4.804538	4.001562	3.996535	4.157171	4.42994	0	0	0	0	0.960699	0	0.920078	12.33701	4.653602	1.956012	1.648904	4.105505	3.640534	0
AC069240.	0	0	0	0	0	1.141887	0	0	0	0.960699	0	0	0	0	0	0	0	0	0
AC069257.	19.21815	10.0039	30.97314	16.62868	31.00958	12.56076	23.79105	13.89041	14.00745	6.724895	6.023774	40.48344	23.64594	16.75297	11.73607	21.43575	45.16056	34.58507	11.652
AC069257.	57.65445	51.01991	40.96448	44.68959	117.3934	99.34417	61.17698	117.6054	8.171014	74.93454	27.70936	84.6472	82.24675	61.42754	55.74633	44.5204	76.97822	66.43974	55.57109
AC069262.	4.804538	3.001171	6.993936	5.196464	0	0	3.398721	3.704108	0	2.882098	0	0.920078	4.112337	0.93072	5.868035	1.648904	0	0	4.48154
AC069271.	0	1.00039	0	1.039293	0	0	0	0	0	0	1.204755	0	1.028084	0	0	0	0	0	0
AC069272.	2.882723	8.003124	0	3.117878	4.42994	4.567548	6.797442	3.704108	0	2.882098	4.81902	0.920078	3.084253	0.93072	2.934017	2.473355	1.026376	0.910133	1.792616
AC069277.	4.804538	0	0	13.51081	4.42994	2.283774	0	5.556162	0	0	2.40951	6.440548	5.140422	0	6.846041	4.122259	0	7.281067	0.896308
AC069277.	0	0	0	2.078586	1.107485	0	0	0	0	0	0	0	1.028084	0	3.912023	0	0	0	5.377848
AC069281.	5.765445	3.001171	19.98267	6.235757	4.42994	6.851322	3.398721	2.778081	4.669151	5.764196	6.023774	10.12086	4.112337	5.584322	10.75806	0.824452	4.105505	3.640534	4.48154
AC069281.	41.31902	61.02382	45.96015	12.47151	67.55658	83.35775	23.79105	44.4493	321.0041	196.9433	680.6865	117.77	85.331	55.84322	38.14223	37.10033	69.79359	181.1165	116.52
AC069282.	983.9693	854.3334	940.1848	896.9097	730.94	900.9488	1114.78	767.6764	476.2534	925.1534	554.1872	828.9905	1054.815	712.001	816.6349	678.5239	787.2306	873.7281	654.3048
AC069285.	0.960908	1.00039	0	0	0	0	3.398721	0	0	0	4.81902	0	1.028084	0.93072	0	0	0	0	0
AC069285.	0.960908	0	0.999134	0	0	0	3.398721	1.852054	0	0	0	0	0	0.93072	0	0	0	0	0
AC069287.	0.960908	0	0	1.039293	2.21497	1.141887	0	2.778081	0	0	0	0	0	0.93072	0.978006	0	0	0.910133	0
AC069287.	0	0	0	0	0	0	0	0	0	0.960699	0	0	0	0	0	0	1.026376	0	0
AC069294.	3.84363	14.00547	6.993936	4.157171	14.3973	19.41208	3.398721	11.11232	18.6766	18.25329	22.89034	11.04094	7.196591	14.89152	6.846041	4.122259	9.237386	1.820267	1.792616
AC069304.	24.02269	43.01679	5.994802	39.49313	2.21497	20.55397	38.23561	25.92876	9.338302	9.606993	0	0.920078	4.112337	18.61441	3.912023	30.50472	20.52753	0.910133	6.274156
AC069304.	0.960908	1.00039	3.996535	0	3.322455	0	0.84968	0	0	4.803496	0	3.680313	0	0	0	0	0	1.820267	0.896308
AC069307.	28.82723	21.0082	36.96795	37.41454	21.04221	13.70264	24.64073	14.81643	58.36439	51.87776	54.21397	10.12086	18.50552	24.19873	17.6041	38.74924	51.31881	15.47227	27.78555
AC069360.	0.960908	2.000781	8.992203	2.078586	0	3.425661	2.549041	1.852054	0	0.960699	18.07132	4.600391	8.224675	1.861441	1.956012	0.824452	0	3.640534	0.896308
AC069366.	0	1.00039	22.98007	4.157171	19.93473	1.141887	6.797442	0	1.167288	0	0	1.840156	1.028084	0.93072	9.780058	0.824452	3.079129	0	4.48154
AC069366.	0	0	0	2.078586	0	0	0.84968	0	0	0	0	2.760235	0	0	0	0.824452	0	0	0
AC069368.	0.960908	3.001171	3.996535	7.275049	5.537425	1.141887	0	0	1.167288	0.960699	0	5.520469	6.168506	6.515042	1.956012	0.824452	0	0	0
AC069431.	20.17906	18.00703	14.987	12.47151	6.64491	3.425661	35.68657	9.26027	5.836439	7.685594	0	11.04094	12.33701	19.54513	12.71408	15.66458	13.34289	16.3824	35.85232
AC069431.	13.45271	4.001562	23.97921	7.275049	1.107485	3.425661	14.44456	14.81643	0	5.764196	7.228529	2.760235	1.028084	0.93072	5.868035	10.71787	6.158258	5.4608	9.859387
AC069437.	0	0	2.997401	2.078586	4.42994	0	1.69936	4.630135	5.836439	5.764196	0	3.680313	1.028084	0	4.890029	0	0	0.910133	0
AC069439.	0	1.00039	0	0	0	0	0.84968	0	0	0.960699	0	0	0	0	0.978006	0.824452	0	0	0
AC069444.	21.13997	4.001562	4.995668	1.039293	13.28982	4.567548	6.797442	0.926027	2.334575	2.882098	2.40951	5.520469	1.028084	4.653602	5.868035	4.122259	8.21101	5.4608	1.792616
AC069499.	36.51449	26.01015	26.97661	37.41454	34.33203	20.55397	45.88273	11.11232	11.67288	16.33189	6.023774	45.08383	54.48847	23.26801	45.96627	64.30724	43.1078	105.5755	75.28987
AC069499.	4.804538	3.001171	4.995668	8.314342	0	0	0	3.704108	0	0	0	0	17.47743	0	9.780058	4.946711	6.158258	5.4608	6.274156
AC069503.	3.84363	2.000781	1.998267	0	1.107485	1.141887	11.89552	0	0	0	7.228529	0.920078	0	0.93072	0.978006	6.595615	1.026376	5.4608	5.377848
AC069503.	34.59267	21.0082	42.96275	14.5501	40.97694	38.82416	26.34009	32.41095	66.5354	58.60265	43.37118	16.56141	17.47743	7.445762	65.52639	21.43575	25.65941	19.1128	16.13354
AC069503.	12.4918	1.00039	0.999134	4.157171	2.21497	0	1.69936	2.778081	0	1.921399	13.2523	4.600391	4.112337	0.93072	1.956012	0	1.026376	4.550667	0
AC069528.	0	1.00039	0	1.039293	0	0	1.69936	0	9.338302	0	0	0.920078	2.056169	0	0.978006	0	0	0	0
AC069528.	76.8726	23.00898	100.9125	91.45776	126.2533	142.7359	64.5757	225.0246	102.7213	134.4979	28.91412	44.16376	69.90974	87.48771	104.6466	186.3261	157.0356	62.79921	59.15632
AC069542.	24.9836	28.01093	3.996535	28.0609	19.93473	27.40529	21.24201	23.15068	16.34203	11.52839	2.40951	38.64329	31.87062	24.19873	35.20821	18.96239	25.65941	43.6864	28.68185
AC069544.	39.39721	53.02069	73.93589	16.62868	98.56616	52.5268	33.98721	87.04654	120.2306	129.6944	238.5415	86.48735	91.49951	87.48771	52.81231	65.13169	45.16056	72.81067	87.83818
AC069544.	274.8196	302.1179	269.7661	325.2986	285.7311	262.634	333.0747	274.104	113.2269	214.2359	210.8321	370.7915	283.7513	298.7612	266.9956	330.6052	232.9874	286.692	342.3896
AC069547.	266.1714	145.0566	200.8259	137.1866	244.7542	142.7359	237.0608	290.7725	180.9296	185.415	260.2271	187.696	277.5828	182.4212	280.6877	112.1254	34.89679	225.7131	136.2388
AC069549.	106.6607	71.02772	101.9116	37.41454	80.8464	105.0536	56.0789	119.4575	408.5507	100.8734	108.4279	53.36454	75.05016	42.81313	41.07624	40.39814	25.65941	59.15867	9.859387
AC072022.	578.4663	568.2218	925.1978	433.3851	585.8595	514.991	923.6024	598.2135	85.212	230.5678	160.2324	1551.252	1179.213	735.269	802.9428	1090.75	1069.484	1260.535	1009.243
AC072022.	36.51449	62.02421	102.9108	16.62868	96.35119	21.69585	173.3348	66.67395	9.338302	59.56335	21.68559	71.7661	98.6961	76.31906	137.8988	28.03136	69.79359	58.24854	60.05263
AC072039.	0	4.001562	3.996535	15.58939	0	1.141887	0.84968	0	0	0	9.638039	0	1.028084	5.584322	3.912023	0.824452	3.079129	3.640534	0

AC072039.	17.29634	15.00586	19.98267	4.157171	24.36467	23.97963	5.098081	19.44657	11.67288	6.724895	0	7.360626	9.252759	13.03008	17.6041	2.473355	12.31652	10.01147	8.963079
AC072052.	31.70995	40.01562	25.97748	60.27898	47.62185	77.64832	44.18337	49.07943	71.20455	83.58083	59.03299	25.76219	56.54464	11.16864	35.20821	24.73355	35.92317	25.48374	29.57816
AC072061.	168.1588	189.0738	200.8259	166.2868	186.0575	183.8438	151.2431	196.3177	215.9482	205.5896	218.0606	195.0566	180.9428	222.4422	170.173	146.7524	182.695	190.2179	227.6622
AC072062.	3.84363	2.000781	14.987	14.5501	11.07485	1.141887	3.398721	1.852054	1.167288	1.921399	0	5.520469	1.028084	0.93072	1.956012	4.946711	6.158258	7.281067	11.652
AC073046.	33.63176	27.01054	41.96361	44.68959	31.00958	33.11472	22.09169	24.0767	11.67288	48.03496	12.04755	46.92399	56.54464	29.78305	24.45015	46.1693	22.58028	46.4168	40.33386
AC073046.	0.960908	1.00039	0	1.039293	0	0	0	6.482189	0	0	0	0.920078	2.056169	0	1.956012	3.297807	1.026376	0.910133	0.896308
AC073050.	26.90541	19.00742	16.98527	14.5501	28.79461	13.70264	22.94137	14.81643	7.003726	1.921399	0	17.48149	18.50552	13.03008	16.6261	28.85581	6.158258	12.74187	10.7557
AC073052.	37.47539	48.01874	137.8804	27.02161	62.01916	34.25661	115.5565	94.45476	43.18965	87.42363	107.2232	50.6043	54.48847	67.94258	58.68035	18.96239	18.47477	30.0344	28.68185
AC073052.	1.921815	0	11.9896	9.353635	1.107485	2.283774	0	0	0	3.842797	22.89034	0	1.028084	0	0.978006	11.54233	1.026376	2.7304	0
AC073062.	0	0	0	4.157171	0	0	4.248401	0	0	0	0	0	0	0	0	0	1.026376	0	0
AC073063.	22.10087	32.01249	36.96795	34.29666	36.547	47.95925	47.58209	70.37805	88.71387	49.95636	63.85201	24.84211	46.2638	48.39746	35.20821	38.74924	29.76491	34.58507	32.26709
AC073065.	0	0	0	0	0	0	0	0	0	0	1.204755	0	1.028084	0	3.912023	0	0	0	0
AC073069.	2.882723	5.001952	14.987	14.5501	4.42994	4.567548	0.84968	17.59451	0	16.33189	1.204755	2.760235	8.224675	0.93072	4.890029	10.71787	6.158258	14.56213	9.859387
AC073071.	0	0	0	0	0	0	0	0	0	0	0	0	0	2.792161	0	0	0	0	0
AC073072.	16.33543	8.003124	17.98441	0	28.79461	1.141887	3.398721	0	18.6766	23.05678	6.023774	9.200782	13.3651	21.40657	3.912023	11.54233	6.158258	22.75334	29.57816
AC073072.	0	1.00039	2.997401	1.039293	2.21497	1.141887	2.549041	0	1.167288	0	2.40951	3.680313	2.056169	0.93072	1.956012	4.122259	0	2.7304	1.792616
AC073073.	39.39721	27.01054	26.97661	16.62868	32.11706	35.3985	23.79105	23.15068	44.35693	44.19217	69.87578	34.04289	34.95487	38.15953	46.94428	19.78684	29.76491	37.31547	10.7557
AC073073.	357.4576	359.1402	414.6405	428.1886	333.353	361.9782	548.0438	393.5615	353.6882	429.4326	301.1887	286.1443	337.2117	323.8907	379.4663	414.6993	465.9748	247.5563	269.7887
AC073082.	4.804538	0	1.998267	1.039293	2.21497	0	5.098081	0	7.003726	12.48909	3.614265	0.920078	5.140422	2.792161	3.912023	0	0	1.820267	0.896308
AC073089.	0	0	0	4.157171	1.107485	1.141887	1.69936	0	2.334575	3.842797	0	1.840156	0	5.584322	2.934017	3.297807	0	2.7304	0
AC073091.	0	1.00039	0	4.157171	0	6.851322	3.398721	0	0	0.960699	0	5.520469	3.084253	7.445762	8.802052	0.824452	0	0.910133	0
AC073091.	0	1.00039	1.998267	0	5.537425	0	0	0	0	1.921399	9.638039	0.920078	1.028084	4.653602	5.868035	0.824452	4.105505	4.550667	7.170464
AC073094.	0	0	0	0	0	0	0	9.26027	0	0	0	0	0	0	0	0	0	0	0
AC073107.	182.5724	188.0734	188.8363	138.2259	178.3051	178.1344	168.2367	185.2054	94.55031	105.6769	101.1994	163.7739	157.2969	125.6472	158.4369	136.0346	133.4289	137.4301	79.77141
AC073107.	21.13997	9.003514	20.98181	31.17878	26.57964	4.567548	24.64073	28.70684	4.669151	37.46727	3.614265	26.68227	48.31997	23.26801	26.40616	17.31349	8.21101	28.21414	25.99293
AC073109.	3.84363	1.00039	1.998267	0	0	2.283774	13.59488	0	4.669151	18.25329	20.48083	3.680313	8.224675	8.376483	8.802052	2.473355	1.026376	0	5.377848
AC073111.	61.49808	67.02616	55.95149	31.17878	49.83682	33.11472	60.3273	65.74792	50.19337	71.09174	87.94711	49.68422	90.47142	83.76483	69.43841	30.50472	35.92317	35.4952	77.97879
AC073111.	0.960908	1.00039	3.996535	1.039293	4.42994	0	3.398721	0	4.669151	0	0	0	0	0	4.890029	0.824452	1.026376	7.281067	0
AC073111.	1968.9	2688.049	2099.18	1492.424	2179.53	3016.865	1596.549	1749.265	3759.834	2809.085	3694.983	2057.295	2014.017	2271.888	2045.988	1963.02	2293.951	1655.533	2186.095
AC073115.	5.765445	1.00039	3.996535	37.41454	0	0	3.398721	0	5.836439	1.921399	0	17.48149	10.28084	14.89152	11.73607	15.66458	4.105505	10.01147	8.066772
AC073115.	18.25724	0	0.999134	50.92535	8.859879	7.993209	11.04584	5.556162	1.167288	15.37119	0	22.08188	13.3651	18.61441	13.69208	33.80253	7.184634	10.01147	13.44462
AC073127.	0	0	0	0	0	0	0.84968	0	0	0	0	2.760235	0	0	0	0.824452	3.079129	0.910133	5.377848
AC073130.	1.921815	8.003124	10.99047	0	1.107485	1.141887	1.69936	2.778081	12.84017	5.764196	3.614265	0.920078	1.028084	7.445762	15.64809	3.297807	3.079129	0	0
AC073130.	63.4199	76.02967	95.91683	53.00393	59.80419	59.37812	135.0992	137.052	40.85507	97.03062	53.00921	103.9688	77.10633	124.7165	116.3827	61.00944	67.74083	43.6864	67.2231
AC073130.	89.3644	35.01367	49.95668	38.45383	91.92125	39.96605	39.93497	72.23011	68.86998	75.89524	50.5997	54.28462	53.46039	48.39746	47.92229	71.72731	83.13648	65.52961	108.4533
AC073133.	21.13997	29.01132	24.97834	9.353635	42.08443	25.12151	11.89552	9.26027	61.86625	25.93888	20.48083	17.48149	21.58977	18.61441	8.802052	1.648904	7.184634	1.820267	4.48154
AC073133.	66.30262	142.0554	173.8493	92.49706	155.0479	43.39171	91.76547	45.37532	102.7213	59.56335	103.6089	83.72712	89.44334	85.62627	67.4824	65.13169	56.45069	32.7648	60.94894
AC073136.	17.29634	25.00976	13.98787	48.84676	26.57964	20.55397	21.24201	29.63287	21.01118	9.606993	16.86657	44.16376	30.84253	40.02097	17.6041	13.19123	22.58028	36.40534	34.0597
AC073136.	0.960908	0	0	0	0	0	1.69936	2.778081	0	0	0	0	0	0	0	0	4.105505	3.640534	0.896308
AC073150.	3.84363	2.000781	2.997401	1.039293	0	1.141887	5.947762	3.704108	14.00745	0.960699	1.204755	9.200782	3.084253	0	0.978006	5.771163	7.184634	3.640534	10.7557
AC073167.	0	2.000781	4.995668	2.078586	15.50479	0	0.84968	0	3.501863	0	0	1.840156	2.056169	1.861441	0	0.824452	1.026376	0.910133	4.48154
AC073172.	0	4.001562	0.999134	4.157171	0	0	5.098081	1.852054	0	2.882098	1.204755	3.680313	1.028084	0.93072	0	3.297807	8.21101	1.820267	0
AC073172.	11.53089	15.00586	3.996535	12.47151	13.28982	14.84453	16.14392	21.29862	4.669151	20.17468	4.81902	17.48149	9.252759	13.03008	4.890029	12.36678	5.131881	1.820267	3.585232
AC073174.	0	3.001171	0	0	1.107485	1.141887	0.84968	0	0	0	0	1.840156	1.028084	0	0	0	0	0.910133	1.792616
AC073176.	0	0	0	6.235757	0	0	0	0	0	0	0	1.840156	0	0	0	0	0	0.910133	0
AC073176.	0	0	0	0	0	0	0	0	0	0	0	0	0	0	0	1.648904	0	0	0
AC073188.	0	0	0.999134	0	0	0	0	0	0	0	0	0	2.056169	0	0	0	0	0	0
AC073195.	19.21815	49.01913	54.95235	23.90373	24.36467	26.2634	13.59488	55.56162	66.5354	74.93454	115.6565	35.88305	17.47743	38.15953	32.27419	23.08465	21.5539	29.12427	22.4077
AC073210.	21.13997	11.00429	38.96621	29.1002	22.1497	4.567548	22.09169	41.67122	17.50932	18.25329	18.07132	34.96297	29.81445	20.47585	34.2302	9.06897	19.50115	14.56213	15.23724
AC073218.	0	0	0	0	0	0	0	0	0	0	0	0	0	1.861441	0	0	0	0	0
AC073218.	0.960908	0	0	0	0	1.141887	0	0	0	3.842797	0	0	0	1.861441	0	0	0	0	0
AC073254.	143.1752	147.0574	171.851	184.9941	151.7254	170.1412	155.4915	125.9397	129.5689	105.6769	169.8704	141.692	163.4654	133.093	133.0088	169.8371	201.1697	158.3632	177.469
AC073257.	4.804538	4.001562	5.994802	3.117878	5.537425	4.567548	3.398721	0	2.334575	3.842797	0	0.920078	1.028084	6.515042	0	11.54233	5.131881	19.1128	27.78555
AC073261.	25.9445	35.01367	9.991337	32.21808	22.1497	25.12151	22.09169	26.85478	5.836439	11.52839	14.45706	25.76219	15.42127	19.54513	17.6041	32.15362	20.52753	26.39387	30.47447
AC073263.	130.6834	24.00937	18.98354	16.62868	44.2994	116.4725	79.86994	88.8986	420.2236	211.3538	268.6603	31.28266	28.78636	102.3792	116.3827	12.36678	25.65941	30.94454	18.82247
AC073263.	0.960908	1.00039	1.998267	12.47151	15.50479	2.283774	2.549041	0	0	0	9.638039	0.920078	6.168506	9.307203	8.802052	5.771163	0	13.652	4.48154
AC073263.	0	0	0	0	0	0	0	1.852054	0	0	0	0	0	0	4.890029	0	0	0.910133	2.688924
AC073264.	4.804538	7.002733	1.998267	11.43222	5.537425	1.141887	2.549041	6.482189	0	10.56769	2.40951	1.840156	1.028084	3.722881	5.868035	0.824452	4.105505	3.640534	5.377848
AC073264.	20.17906	34.01327	8.992203	24.94303	4.42994	20.55397	27.18977	4.630135	0	16.33189	9.638039	11.96102	11.30893	41.88241	46.94428	7.420066	3.079129	9.101334	17.02985
AC073283.	11.53089	23.00898	16.98527	10.39293	23.25718	20.55397	14.44456	24.0767	7.003726	11.52839	34.93789	19.32164	14.39318	12.09936	17.6041	10.71787	16.42202	32.7648	40.33386
AC073283.	41.31902	30.01171	25.97748	119.5187	12.18233	27.40529	66.27506	47.22738	39.68778	24.97818	4.81902	37.72321	42.15146	66.08114	38.14223	56.06272	99.5585	31.85467	45.71171
AC073284.	1.921815	0	0	0	0	1.141887	0	0.926027	0	0	0	2.760235	1.028084	0	0.978006	0	0	0	0

AC073288.	0.960908	1.00039	0	0	0	1.141887	0	0.926027	8.171014	0.960699	0	0	0	0	0	1.648904	1.026376	0	0
AC073288.	6.726353	19.00742	21.98094	2.078586	44.2994	11.41887	11.04584	35.18903	31.51677	1.921399	10.84279	7.360626	16.44935	14.89152	13.69208	6.595615	9.237386	30.0344	8.066772
AC073314.	7.68726	5.001952	8.992203	1.039293	2.21497	6.851322	1.69936	12.03835	0	13.44979	14.45706	0.920078	0	0	1.956012	2.473355	5.131881	11.83173	0
AC073316.	170.0806	194.0757	116.8986	486.389	108.5335	255.7827	183.5309	162.0547	77.04099	84.54153	46.98544	167.4542	178.8867	133.093	120.2947	298.4516	225.8028	62.79921	144.3056
AC073316.	11.53089	7.002733	4.995668	8.314342	2.21497	3.425661	4.248401	14.81643	0	3.842797	1.204755	2.760235	13.3651	8.376483	3.912023	4.946711	5.131881	0	1.792616
AC073316.	48.04538	23.00898	42.96275	25.98232	27.68712	28.54718	15.29424	30.55889	9.338302	7.685594	27.70936	18.40156	27.75828	31.64449	41.07624	4.122259	6.158258	11.83173	15.23724
AC073320.	11.53089	33.01288	16.98527	4.157171	1.107485	12.56076	7.647122	7.408216	7.003726	17.29259	10.84279	8.280704	19.5336	12.09936	16.6261	6.595615	9.237386	13.652	7.170464
AC073321.	0	1.00039	0	0	0	3.425661	1.69936	0.926027	1.167288	0	10.84279	0	0	0	0	0.824452	0	0	1.792616
AC073323.	0	0	0	2.078586	1.107485	2.283774	0.84968	0	0	0	0	0.920078	5.140422	0	1.956012	0	0	0.910133	0
AC073324.	0	1.00039	0	6.235757	0	0	0	0	0	0	0	1.840156	0	0	0	4.946711	6.158258	0	0
AC073324.	2.882723	0	0	4.157171	4.42994	0	2.549041	0	11.67288	0.960699	1.204755	2.760235	2.056169	0.93072	3.912023	13.19123	7.184634	1.820267	0.896308
AC073326.	16.33543	3.001171	8.992203	3.117878	1.107485	3.425661	8.496802	19.44657	24.51304	1.921399	1.204755	10.12086	23.64594	10.23792	19.56012	2.473355	5.131881	6.370934	1.792616
AC073333.	17.29634	17.00664	26.97661	40.53242	8.859879	14.84453	36.53625	38.89314	4.669151	17.29259	15.66181	34.96297	20.56169	19.54513	24.45015	18.13794	19.50115	29.12427	30.47447
AC073335.	1.921815	3.001171	1.998267	3.117878	6.64491	3.425661	0	7.408216	0	8.646293	1.204755	1.840156	3.084253	2.792161	2.934017	9.06897	9.237386	0.910133	3.585232
AC073335.	1156.933	776.303	814.2939	496.7819	1089.765	797.0371	695.8881	641.7367	3008.1	1038.516	1760.147	696.4992	672.3672	1010.762	1026.906	897.0036	976.0838	963.8313	1084.533
AC073336.	1.921815	1.00039	0	7.275049	1.107485	1.141887	4.248401	8.334243	0	0	0	0.920078	0	5.584322	0	0	3.079129	0.910133	0.896308
AC073342.	13.45271	12.00469	17.98441	14.5501	16.61227	22.83774	16.14392	11.11232	23.34575	18.25329	38.55216	19.32164	15.42127	9.307203	8.802052	12.36678	3.079129	17.29253	25.99293
AC073342.	5.765445	0	0	15.58939	0	0	4.248401	4.630135	9.338302	0	0	15.64133	4.112337	15.82225	6.846041	75.84957	16.42202	20.02294	4.48154
AC073343.	190.2597	211.0824	196.8293	254.6267	171.6602	121.04	284.6429	211.1342	72.37184	161.3975	153.0039	174.8149	271.4143	241.0566	158.4369	187.1506	211.4335	114.6768	111.1422
AC073343.	92.24712	125.0488	176.8467	175.6405	171.6602	74.22266	110.4584	93.52873	91.04844	69.17035	143.3658	128.811	116.1735	111.6864	117.3607	111.301	130.3498	48.23707	80.66772
AC073346.	17.29634	3.001171	9.991337	7.275049	1.107485	0	9.346483	13.89041	0	9.606993	6.023774	8.280704	1.028084	13.03008	14.67009	8.244518	8.21101	6.370934	10.7557
AC073346.	14.41361	42.0164	25.97748	36.37525	26.57964	47.95925	26.34009	4.630135	21.01118	48.99566	27.70936	23.92203	33.92678	24.19873	29.34017	29.68027	29.76491	27.304	27.78555
AC073347.	1.921815	0	0	0	0	1.141887	0	0.926027	0	0	0	0	0	0	0	0	0	0	0
AC073348.	0	0	0	0	0	0	0	0	0	0.960699	0	0	0	2.792161	0	0	0	0	0
AC073349.	20.17906	15.00586	12.98874	18.70727	12.18233	25.12151	26.34009	23.15068	10.50559	30.74238	33.73314	16.56141	12.33701	23.26801	17.6041	23.08465	25.65941	23.66347	38.54124
AC073349.	405.503	420.164	626.4568	729.5835	670.0284	391.6672	913.4063	650.071	114.3942	316.0701	180.7132	957.8014	764.8948	735.269	670.912	604.3232	579.9026	440.5046	830.8775
AC073352.	10.56998	31.0121	24.97834	23.90373	9.967364	12.56076	6.797442	35.18903	80.54285	20.17468	59.03299	12.8811	1.028084	13.03008	18.58211	22.2602	19.50115	6.370934	23.30401
AC073358.	0	0	0	0	3.322455	0	0.84968	0	0	0	0	0.920078	8.224675	0	0	0	3.079129	0	0.896308
AC073359.	6.726353	3.001171	9.991337	6.235757	6.64491	1.141887	2.549041	6.482189	12.84017	0.960699	0	8.280704	4.112337	5.584322	14.67009	6.595615	10.26376	2.7304	0
AC073359.	0	0	0	0	0	0	0	0	3.501863	0	0	0	1.028084	0.93072	0	1.648904	4.105505	0	0.896308
AC073365.	17.29634	22.00859	0.999134	48.84676	19.93473	13.70264	52.68017	35.18903	5.836439	9.606993	4.81902	30.36258	29.81445	25.12945	23.47214	25.55801	40.02867	29.12427	25.09662
AC073367.	119.1525	173.0675	145.8735	127.833	98.56616	92.49285	145.2953	141.6821	105.0559	127.773	83.12809	132.4913	124.3982	168.4604	190.7111	79.14738	103.664	93.74374	90.5271
AC073370.	26.90541	18.00703	1.998267	12.47151	6.64491	25.12151	7.647122	5.556162	11.67288	0.960699	0	11.96102	13.3651	12.09936	8.802052	16.48904	7.184634	20.02294	12.54831
AC073389.	140.2925	93.03631	156.864	60.27898	97.45867	30.83095	94.31451	56.48765	73.53913	61.48475	60.23774	100.2885	125.4263	64.2197	83.1305	46.1693	68.76721	131.0592	131.7573
AC073389.	4.804538	7.002733	30.97314	13.51081	8.859879	14.84453	20.39233	15.74246	1.167288	17.29259	14.45706	11.04094	23.64594	3.722881	6.846041	18.13794	12.31652	12.74187	13.44462
AC073389.	159.5106	124.0484	153.8666	80.02554	172.7676	79.93209	100.2623	131.4958	364.1938	162.3582	134.9325	136.1716	105.8927	120.0629	161.371	106.3543	86.21561	112.8565	98.59387
AC073410.	5.765445	36.01406	36.96795	3.117878	23.25718	45.67548	29.73881	38.89314	21.01118	69.17035	73.49005	50.6043	45.23571	26.06017	14.67009	9.893422	12.31652	6.370934	9.859387
AC073410.	4.804538	3.001171	0.999134	9.353635	0	3.425661	0.84968	12.03835	4.669151	7.685594	0	5.520469	5.140422	0	4.890029	4.946711	3.079129	0.910133	4.48154
AC073413.	12.4918	17.00664	25.97748	13.51081	28.79461	6.851322	23.79105	15.74246	22.17847	48.03496	31.32363	24.84211	29.81445	13.9608	27.38416	20.6113	27.71216	24.5736	18.82247
AC073413.	0	2.000781	0	0	0	0	0.84968	0	0	1.921399	14.45706	0.920078	0	0	0	0	5.131881	0	0
AC073415.	6.726353	4.001562	16.98527	4.157171	4.42994	15.98642	15.29424	16.66849	0	4.803496	1.204755	6.440548	14.39318	4.653602	1.956012	5.771163	2.052753	2.7304	8.066772
AC073429.	0	0	5.994802	0	1.107485	0	0.84968	3.704108	1.167288	0	0	0	0	0	3.912023	0.824452	0	0	0
AC073439.	6.726353	6.002343	9.991337	0	9.967364	15.98642	10.19616	5.556162	7.003726	9.606993	20.48083	18.40156	5.140422	4.653602	12.71408	6.595615	10.26376	12.74187	4.48154
AC073464.	7.68726	7.002733	0.999134	10.39293	0	2.283774	5.098081	0	0	4.803496	7.228529	2.760235	2.056169	2.792161	1.956012	3.297807	0	0	1.792616
AC073464.	4.804538	8.003124	2.997401	12.47151	0	10.27698	7.647122	0.926027	1.167288	0.960699	0	6.440548	13.3651	1.861441	2.934017	5.771163	3.079129	0.910133	12.54831
AC073465.	0	0	1.998267	0	1.107485	2.283774	0	0	0	0	0	0	4.112337	0	0	0	0	0	5.377848
AC073465.	0	0	0	0	0	0	0	0	1.167288	0.960699	0	0	1.028084	0	0	0	0	0	0
AC073472.	0.960908	0	0	0	0	0	0	0	0	0.960699	0	0.920078	0	0	0	0	0	0	1.792616
AC073475.	0.960908	0	0	0	0	0	0.84968	0	0	0	0	0	4.112337	0	0.978006	0	1.026376	0	0
AC073476.	74.95079	35.01367	71.93762	56.12181	66.4491	57.09435	90.06611	62.96984	7.003726	45.15286	30.11887	70.84602	98.6961	112.6172	108.5586	58.53608	50.29244	43.6864	50.19325
AC073476.	44.20175	85.03319	152.8675	124.7151	95.2437	85.64153	169.936	145.3862	5.836439	78.77734	87.94711	129.731	125.4263	94.93347	73.35044	117.8966	126.2443	84.64241	121.0016
AC073476.	0	1.00039	0.999134	0	0	0	0	0	0	0	0	0	0	0.93072	0	0.824452	0	0	0
AC073486.	18.25724	9.003514	21.98094	16.62868	17.71976	18.27019	34.83689	17.59451	22.17847	10.56769	12.04755	7.360626	9.252759	21.40657	15.64809	15.66458	21.5539	5.4608	13.44462
AC073487.	249.836	238.0929	333.7106	289.9627	192.7024	188.4114	252.355	234.2848	227.6211	316.0701	221.6749	158.2535	198.4203	266.186	274.8196	230.0221	234.0138	232.9942	164.9207
AC073488.	2.882723	1.00039	0	0	0	0	2.549041	0	14.00745	56.68126	61.4425	11.04094	3.084253	34.43665	29.34017	0.824452	0	0	0
AC073488.	0	0	0	0	0	0	0	0	0	0	7.228529	0.920078	0	0	0.978006	0	0	0	0
AC073488.	0	0	0	0	0	0	0.84968	0	0	4.803496	3.614265	13.80117	1.028084	0	6.846041	0	2.052753	0	0
AC073488.	0.960908	0	0	0	0	0	0	0	0	0	1.204755	4.600391	1.028084	0	0.978006	0	0	0	0
AC073488.	0	0	0	0	0	0	5.098081	0.926027	0	17.29259	8.433284	8.280704	2.056169	3.722881	17.6041	0	0	0	0
AC073488.	0	0	0	0	0	0	1.69936	0	0	15.37119	0	1.840156	0	5.584322	20.53812	0	0	0	0
AC073488.	0	0	0	0	0	0	0.84968	0.926027	0	3.842797	2.40951	0	4.112337	3.722881	1.956012	0	0	0	0
AC073488.	0	0	0	0	0	0	0	0	0	9.606993	8.433284	9.200782	1.028084	13.9608	0	0	0	0	0

AC073488.	4.804538	0	0	0	0	0	1.69936	0	0	26.89958	18.07132	5.520469	1.028084	3.722881	21.51613	0	0	0	0
AC073488.	0	0	0	0	0	0	5.098081	0	0	0	3.614265	0.920078	1.028084	0	0	0	0	0	0
AC073488.	0	0	0	0	0	0	0	0	0	3.842797	16.86657	9.200782	1.028084	1.861441	1.956012	0	0	0	0
AC073508.	0.960908	0	1.998267	1.039293	0	0	0.84968	0	0	0.960699	0	2.760235	0	3.722881	1.956012	0	2.052753	3.640534	0
AC073508.	182.5724	161.0629	243.7886	199.5442	138.4356	187.2695	194.5768	164.8328	419.0563	277.6421	398.7739	200.5771	201.5045	165.6682	182.8871	161.5926	163.1938	198.4091	151.476
AC073517.	11.53089	24.00937	23.97921	21.82515	6.64491	20.55397	15.29424	32.41095	1.167288	13.44979	13.2523	9.200782	28.78636	14.89152	11.73607	17.31349	29.76491	20.93307	31.37078
AC073521.	0	1.00039	0	1.039293	0	0	0	0	0	0	0	0	2.056169	0	0	0	0	0	0
AC073525.	25.9445	29.01132	9.991337	38.45383	13.28982	21.69585	38.23561	37.96711	8.171014	1.921399	0	8.280704	30.84253	16.75297	18.58211	19.78684	12.31652	10.9216	8.066772
AC073529.	34.59267	31.0121	34.96968	43.6503	22.1497	19.41208	48.43177	49.07943	3.501863	31.70308	19.27608	39.56336	33.92678	40.02097	13.69208	32.97807	18.47477	49.1472	90.5271
AC073530.	0	1.00039	0	0	0	0	0	0	0	0.960699	0	0.920078	1.028084	0	0	0.824452	1.026376	0.910133	0
AC073530.	0	0	0	0	0	3.425661	0	0	0	0	0	0.920078	0	0	0	0	0	0	0
AC073534.	182.5724	143.0558	159.8614	124.7151	227.0344	186.1276	193.7271	165.7588	208.9445	208.4717	222.8797	253.9416	255.993	135.8852	145.7229	133.5612	105.7168	162.9139	155.0613
AC073534.	17.29634	13.00508	25.97748	18.70727	29.90209	7.993209	27.18977	18.52054	11.67288	13.44979	10.84279	4.600391	8.224675	13.03008	7.824047	5.771163	12.31652	8.191201	2.688924
AC073539.	0	0	0	0	0	0	0	0	0	4.803496	0	0	0	0	0	0	0	0	0
AC073539.	79.75532	72.02811	51.95495	43.6503	39.86946	54.81058	48.43177	70.37805	51.36066	36.50657	22.89034	56.12477	65.7974	105.1714	92.91055	66.7806	67.74083	70.08027	64.53417
AC073544.	0	4.001562	2.997401	3.117878	1.107485	11.41887	0.84968	5.556162	2.334575	4.803496	0	2.760235	3.084253	3.722881	0	0	1.026376	0.910133	1.792616
AC073544.	0.960908	2.000781	6.993936	0	6.64491	14.84453	5.098081	6.482189	0	0.960699	7.228529	4.600391	3.084253	5.584322	7.824047	5.771163	3.079129	2.7304	6.274156
AC073548.	388.2066	532.2077	568.5071	375.1847	601.3643	629.1797	655.9531	480.608	810.0977	853.1009	1138.493	698.3394	637.4123	683.1487	524.2111	461.693	475.2122	751.7702	674.0236
AC073551.	0	0	0	0	1.107485	0	1.69936	0	0	0	0	0	0	0	0	0	0	0	0
AC073569.	14.41361	1.00039	13.98787	15.58939	9.967364	7.993209	6.797442	3.704108	3.501863	3.842797	3.614265	11.96102	7.196591	15.82225	5.868035	3.297807	2.052753	9.101334	1.792616
AC073569.	0.960908	17.00664	20.98181	7.275049	14.3973	9.135096	34.83689	24.0767	0	8.646293	6.023774	13.80117	21.58977	32.57521	26.40616	9.893422	3.079129	8.191201	8.963079
AC073569.	27.86632	8.003124	37.96708	23.90373	12.18233	27.40529	28.88913	13.89041	32.68406	24.97818	20.48083	10.12086	19.5336	8.376483	4.890029	9.893422	4.105505	14.56213	12.54831
AC073571.	5.765445	0	7.993069	0	0	0	2.549041	0.926027	1.167288	0	6.023774	0.920078	1.028084	0	5.868035	0.824452	8.21101	0.910133	0.896308
AC073573.	7.68726	21.0082	5.994802	1.039293	9.967364	18.27019	6.797442	5.556162	11.67288	14.41049	26.50461	7.360626	5.140422	21.40657	22.49413	12.36678	18.47477	20.02294	7.170464
AC073575.	0.960908	0	0.999134	12.47151	1.107485	1.141887	4.248401	1.852054	0	1.921399	0	9.200782	4.112337	3.722881	0.978006	5.771163	2.052753	0.910133	0
AC073575.	28.82723	20.00781	29.97401	55.08252	34.33203	41.10793	37.38593	43.52327	53.69524	28.82098	21.68559	22.08188	18.50552	34.43665	23.47214	29.68027	57.47707	27.304	38.54124
AC073578.	0.960908	5.001952	2.997401	0	2.21497	0	3.398721	0	0	0	0	3.680313	0	0.93072	0	0	6.158258	5.4608	4.48154
AC073578.	4.804538	3.001171	0.999134	2.078586	1.107485	0	10.19616	5.556162	2.334575	11.52839	3.614265	0	1.028084	4.653602	2.934017	2.473355	6.158258	1.820267	10.7557
AC073578.	0.960908	0	0	0	4.42994	0	0.84968	0	0	0	0	2.760235	0	0.93072	0.978006	0	4.105505	0.910133	0
AC073578.	5.765445	3.001171	8.992203	1.039293	0	4.567548	0	3.704108	0	5.764196	10.84279	4.600391	7.196591	10.23792	0.978006	3.297807	0	3.640534	10.7557
AC073581.	0	0	0	0	1.107485	0	0	0	0	0	0	0	0	0.93072	0	0.824452	0	0	0
AC073583.	1.921815	0	0	0	1.107485	1.141887	0.84968	0.926027	0	3.842797	9.638039	0.920078	1.028084	6.515042	1.956012	0	0	6.370934	1.792616
AC073585.	27.86632	47.01835	34.96968	91.45776	31.00958	58.23624	73.0725	74.08216	16.34203	54.75986	19.27608	55.20469	54.48847	43.74385	51.83431	60.18498	74.92547	59.15867	62.74156
AC073587.	0	0	0	0	0	0	0	0	0	0	0	0	1.028084	0	0	0	2.052753	0	0
AC073591.	1.921815	1.00039	0	4.157171	4.42994	0	1.69936	0	0	0	3.614265	0.920078	2.056169	0	1.956012	0	4.105505	0	2.688924
AC073592.	100.8953	74.02889	128.8882	40.53242	90.81376	53.66869	256.6034	77.78627	78.20828	69.17035	98.7899	92.9279	114.1174	45.60529	70.41642	61.00944	89.29473	29.12427	36.74863
AC073592.	0.960908	1.00039	0	5.196464	0	0	9.346483	4.630135	4.669151	0	0	2.760235	0	1.861441	0	18.13794	5.131881	5.4608	8.066772
AC073592.	0	0	0	0	1.107485	0	0	0	5.836439	1.921399	0	0	0	3.722881	4.890029	4.122259	1.026376	0	1.792616
AC073592.	1.921815	1.00039	0.999134	4.157171	4.42994	1.141887	0.84968	0.926027	5.836439	0.960699	4.81902	5.520469	2.056169	9.307203	3.912023	4.122259	8.21101	2.7304	1.792616
AC073592.	0	0	0	0	0	0	0	0	0	0	0	0	0	0.93072	0	11.54233	0	0.910133	0
AC073592.	0.960908	0	0.999134	0	2.21497	1.141887	3.398721	5.556162	0	5.764196	1.204755	0.920078	1.028084	5.584322	0	4.946711	4.105505	1.820267	1.792616
AC073592.	0.960908	0	7.993069	4.157171	4.42994	4.567548	2.549041	0	0	0.960699	4.81902	0	12.33701	0.93072	3.912023	4.122259	4.105505	0.910133	1.792616
AC073592.	0	0	3.996535	3.117878	1.107485	4.567548	8.496802	0.926027	5.836439	0	0	1.840156	6.168506	4.653602	0	5.771163	9.237386	0.910133	0.896308
AC073592.	0	0	0.999134	0	0	2.283774	0	0	0	1.921399	0	1.840156	0	0	0	0	5.131881	1.820267	0
AC073592.	0	0	0	0	0	0	0	0	1.167288	0.960699	0	0.920078	1.028084	0	0	0	0	0	1.792616
AC073593.	1.921815	0	0	19.74656	0	2.283774	4.248401	2.778081	2.334575	0.960699	0	8.280704	2.056169	0	0	5.771163	12.31652	0	0
AC073594.	0	0	0	0	0	0	0	0	0	0	2.40951	0	1.028084	0	0	0.824452	0	0	0
AC073610.	8.648168	8.003124	9.991337	1.039293	7.752394	18.27019	21.24201	8.334243	5.836439	28.82098	10.84279	8.280704	4.112337	6.515042	4.890029	8.244518	6.158258	5.4608	13.44462
AC073610.	0	0	3.996535	0	0	0	1.69936	0.926027	5.836439	0	4.81902	0.920078	0	0	3.912023	1.648904	3.079129	0.910133	0.896308
AC073611.	270.9759	422.1648	250.7825	259.8232	358.8251	467.0318	329.6759	390.7834	658.3503	465.9391	575.8728	333.9884	356.7453	329.475	320.7859	327.3074	412.6033	410.4702	308.3299
AC073621.	58.61536	74.02889	38.96621	23.90373	121.8233	65.08756	40.78465	62.04381	155.2493	102.7948	89.15186	69.92595	55.51656	70.73474	63.57038	73.37621	65.68808	59.15867	35.85232
AC073621.	0	4.001562	9.991337	5.196464	6.64491	0	1.69936	2.778081	0	0.960699	0	12.8811	6.168506	0	4.890029	7.420066	3.079129	1.820267	8.963079
AC073626.	0	0	0	0	0	0	0	0.926027	0	0	0	0	0	0	0	0	0	2.7304	0
AC073626.	0	0	0	0	0	0	0	0	0	0	0	0	1.028084	0	0	1.648904	0	0	0
AC073636.	10.56998	17.00664	8.992203	8.314342	34.33203	13.70264	8.496802	0	17.50932	16.33189	20.48083	15.64133	10.28084	30.71377	10.75806	3.297807	8.21101	3.640534	0.896308
AC073641.	0.960908	1.00039	8.992203	6.235757	2.21497	1.141887	0	12.03835	4.669151	6.724895	7.228529	7.360626	7.196591	6.515042	1.956012	13.19123	7.184634	11.83173	5.377848
AC073648.	11.53089	2.000781	1.998267	8.314342	1.107485	4.567548	6.797442	9.26027	0	5.764196	4.81902	8.280704	2.056169	0	3.912023	8.244518	6.158258	1.820267	6.274156
AC073648.	168.1588	145.0566	93.91856	73.78979	147.2955	147.3034	92.61515	119.4575	71.20455	111.4411	91.56137	87.40743	75.05016	113.5479	112.4707	112.1254	132.4025	117.4072	103.0754
AC073648.	12.4918	15.00586	7.993069	3.117878	6.64491	3.425661	6.797442	6.482189	37.35321	16.33189	12.04755	8.280704	10.28084	6.515042	6.846041	7.420066	6.158258	6.370934	0
AC073648.	1.921815	1.00039	0	0	0	0	0	0	1.167288	2.882098	16.86657	0.920078	0	0	0	0	1.026376	0	0.896308
AC073651.	9.609075	0	8.992203	17.66798	6.64491	1.141887	9.346483	0	4.669151	0	0	2.760235	5.140422	1.861441	3.912023	11.54233	11.29014	1.820267	1.792616
AC073651.	0.960908	0	0	1.039293	1.107485	0	7.647122	0	4.669151	0	0	1.840156	0	0.93072	3.912023	2.473355	0	0.910133	0.896308

AC073655.	5.765445	3.001171	13.98787	11.43222	3.322455	6.851322	5.947762	22.22465	8.171014	8.646293	1.204755	19.32164	11.30893	1.861441	0	10.71787	9.237386	13.652	7.170464
AC073655.	36.51449	48.01874	81.92896	74.82908	62.01916	29.68906	57.77826	86.12051	159.9184	104.7162	115.6565	115.0098	114.1174	90.27987	83.1305	74.20066	69.79359	136.52	99.49018
AC073834.	0	0	0	0	2.21497	0	0	0	0	0	0	0	0	0	0	0.824452	2.052753	0	0
AC073842.	8.648168	2.000781	3.996535	5.196464	5.537425	3.425661	4.248401	0	0	11.52839	6.023774	0.920078	4.112337	5.584322	3.912023	0.824452	16.42202	0.910133	2.688924
AC073842.	301.725	236.0921	281.7557	430.2672	286.8386	275.1948	342.4211	273.178	372.3648	312.2273	365.0407	285.2243	297.1164	273.6318	304.1598	258.8779	312.0184	276.6806	159.5428
AC073848.	0.960908	5.001952	1.998267	1.039293	8.859879	0	0	0.926027	0	1.921399	0	0	5.140422	6.515042	0.978006	1.648904	0	0	3.585232
AC073850.	43.24084	36.01406	38.96621	12.47151	35.43952	19.41208	66.27506	39.81916	18.6766	104.7162	91.56137	32.20274	48.31997	24.19873	29.34017	3.297807	4.105505	2.7304	1.792616
AC073857.	504.4765	438.171	598.4811	596.554	542.6676	656.585	534.4489	487.0902	679.3615	730.1314	671.0485	444.3978	507.8737	506.3118	445.9707	471.5864	528.5838	571.5638	423.0574
AC073861.	213.3215	184.0718	166.8553	188.112	137.3281	150.7291	150.3934	196.3177	229.9557	183.4936	174.6895	211.618	164.4935	188.9362	173.107	192.0973	179.6158	223.8928	208.8398
AC073862.	7.68726	7.002733	13.98787	11.43222	1.107485	0	16.9936	8.334243	24.51304	23.05678	6.023774	0.920078	10.28084	16.75297	12.71408	24.73355	35.92317	11.83173	13.44462
AC073862.	60.53717	57.02225	75.93416	0	59.80419	17.12831	66.27506	52.78354	19.84389	19.21399	28.91412	58.88501	85.331	71.66546	107.5806	13.19123	6.158258	20.93307	6.274156
AC073862.	4.804538	4.001562	5.994802	0	3.322455	0	26.34009	4.630135	3.501863	12.48909	12.04755	6.440548	1.028084	13.9608	10.75806	8.244518	10.26376	9.101334	0.896308
AC073862.	18.25724	12.00469	30.97314	8.314342	11.07485	10.27698	27.18977	12.96438	16.34203	13.44979	18.07132	9.200782	4.112337	42.81313	47.92229	43.69595	33.87042	38.2256	14.34093
AC073863.	1.921815	0	1.998267	0	0	1.141887	0	0.926027	0	0	0	3.680313	3.084253	0	0	1.648904	0	3.640534	0.896308
AC073869.	3382.395	3296.286	2570.771	1417.595	4032.353	2278.065	2002.696	2374.333	7255.861	4894.763	8745.316	3183.471	3029.765	3457.626	3277.298	2402.453	2628.55	2629.375	2626.182
AC073869.	1.921815	3.001171	0.999134	1.039293	0	0	0	0	0	0	0	0	0	0	0	0	0	0	0
AC073869.	7.68726	7.002733	5.994802	3.117878	7.752394	4.567548	0.84968	4.630135	21.01118	7.685594	14.45706	18.40156	18.50552	10.23792	8.802052	11.54233	2.052753	4.550667	20.61508
AC073869.	19.21815	7.002733	27.97574	17.66798	29.90209	23.97963	24.64073	23.15068	11.67288	20.17468	50.5997	18.40156	27.75828	18.61441	7.824047	21.43575	13.34289	4.550667	17.02985
AC073869.	0	0	0	0	0	0	0	0	0	0	0	0	2.056169	0	0	0	0	0	0.896308
AC073869.	21.13997	43.01679	50.95582	27.02161	28.79461	37.68227	41.63433	20.37259	42.02236	25.93888	33.73314	24.84211	32.8987	26.06017	6.846041	23.08465	20.52753	20.93307	28.68185
AC073869.	7.68726	5.001952	2.997401	14.5501	4.42994	23.97963	6.797442	20.37259	2.334575	12.48909	15.66181	8.280704	5.140422	19.54513	0.978006	6.595615	16.42202	8.191201	18.82247
AC073873.	0	0	0	0	0	0	0	0	0	0	0	0	0	0	0	1.648904	0	0	0
AC073878.	1.921815	11.00429	2.997401	6.235757	8.859879	0	4.248401	10.1863	9.338302	1.921399	1.204755	7.360626	15.42127	0.93072	5.868035	0.824452	4.105505	4.550667	1.792616
AC073878.	0.960908	6.002343	13.98787	0	13.28982	1.141887	1.69936	0.926027	10.50559	0.960699	3.614265	0.920078	4.112337	0.93072	0	0	1.026376	1.820267	0.896308
AC073896.	103.778	74.02889	117.8978	135.1081	155.0479	116.4725	90.91579	108.3452	135.4054	137.38	169.8704	108.5692	137.7633	97.72563	94.86656	117.0722	133.4289	123.7781	141.6167
AC073896.	172.9634	211.0824	316.7254	233.8409	260.259	234.0868	265.9499	250.0273	79.37557	281.4849	213.2416	305.466	289.9198	189.8669	182.8871	173.1349	207.328	207.5104	302.0558
AC073896.	193.1424	299.1167	273.7626	206.8193	475.111	347.1336	176.7335	277.8081	1330.708	543.7558	766.2241	256.7018	239.5437	250.3638	245.4795	247.3355	344.8624	287.6022	183.7431
AC073896.	8.648168	8.003124	10.99047	16.62868	13.28982	15.98642	14.44456	23.15068	18.6766	7.685594	22.89034	13.80117	10.28084	2.792161	9.780058	10.71787	14.36927	8.191201	2.688924
AC073900.	0	1.00039	0	0	0	0	0.84968	0	0	0	0	0	0	0	0	0	0	0.910133	0
AC073909.	0	0	0	1.039293	11.07485	1.141887	0	2.778081	4.669151	0	1.204755	0.920078	0	1.861441	0	0	1.026376	0	1.792616
AC073910.	0.960908	1.00039	4.995668	6.235757	0	0	0.84968	0.926027	0	0	0	0	0	0.93072	0	0	3.079129	0	0.896308
AC073911.	3.84363	3.001171	8.992203	16.62868	2.21497	5.709435	0.84968	7.408216	5.836439	1.921399	0	8.280704	5.140422	7.445762	5.868035	9.893422	16.42202	4.550667	5.377848
AC073911.	0.960908	10.0039	6.993936	9.353635	4.42994	4.567548	11.89552	21.29862	0	4.803496	3.614265	4.600391	11.30893	0	9.780058	9.893422	6.158258	2.7304	0.896308
AC073911.	6.726353	17.00664	10.99047	13.51081	6.64491	11.41887	18.69297	25.00273	7.003726	15.37119	4.81902	14.72125	11.30893	21.40657	23.47214	5.771163	16.42202	27.304	11.652
AC073912.	96.09075	16.00625	21.98094	21.82515	58.6967	4.567548	101.1119	133.3479	9.338302	76.85594	59.03299	32.20274	17.47743	28.85233	27.38416	54.41382	49.26606	20.02294	28.68185
AC073912.	58.61536	6.002343	16.98527	13.51081	36.547	2.283774	72.22282	69.45203	2.334575	28.82098	21.68559	20.24172	12.33701	14.89152	8.802052	45.34485	28.73854	20.93307	18.82247
AC073912.	0	1.00039	1.998267	0	0	5.709435	0	0.926027	0	0	1.204755	0.920078	1.028084	3.722881	5.868035	0	0	0.910133	3.585232
AC073913.	1.921815	4.001562	0.999134	10.39293	7.752394	3.425661	2.549041	2.778081	0	7.685594	2.40951	5.520469	3.084253	0	0.978006	3.297807	1.026376	4.550667	3.585232
AC073916.	6.726353	6.002343	0.999134	8.314342	6.64491	10.27698	0.84968	6.482189	1.167288	1.921399	3.614265	9.200782	10.28084	7.445762	3.912023	0.824452	4.105505	10.9216	8.963079
AC073934.	0.960908	1.00039	3.996535	0	0	1.141887	0	0.926027	7.003726	0.960699	7.228529	3.680313	1.028084	9.307203	0.978006	1.648904	1.026376	1.820267	9.859387
AC073941.	1.921815	0	0.999134	0	0	0	1.69936	0	0	0	0	0.920078	0	4.653602	0	0.824452	0	1.820267	0
AC073941.	0.960908	1.00039	0	0	0	0	0	0	0	0	0	0	0	0	0	0	0	0	0
AC073957.	5.765445	11.00429	3.996535	0	6.64491	5.709435	0.84968	5.556162	5.836439	0	0	0.920078	3.084253	1.861441	0.978006	2.473355	1.026376	5.4608	6.274156
AC073957.	1527.843	1270.496	1644.574	964.4637	1253.673	1274.346	1171.709	1112.158	1197.637	1499.652	1502.329	1631.299	1802.232	1098.25	1143.289	1024.794	1177.254	1074.868	1220.771
AC073964.	4.804538	0	0	0	0	0	1.69936	0	0	0	0	0	0	0	7.824047	0	0	0.910133	0
AC073968.	0.960908	0	3.996535	0	0	0	0	0	0	0	0	0	1.028084	0	3.912023	0	0	0	0
AC073987.	0	0	0	0	0	0	0	1.852054	0	0	0	0	0	0	0	0	0	0	0
AC074011.	45.16265	37.01445	53.95322	33.25737	31.00958	36.54038	22.94137	52.78354	86.37929	65.32755	51.80446	46.92399	39.06721	47.46674	45.96627	82.44518	45.16056	191.128	124.5868
AC074019.	0.960908	1.00039	0	0	0	0	0	0	0	0	0	0	0	0	0	0	0	0	0
AC074029.	0	7.002733	23.97921	1.039293	0	2.283774	10.19616	5.556162	0	13.44979	24.0951	2.760235	1.028084	0	1.956012	1.648904	0	15.47227	10.7557
AC074029.	0	0	0	0	0	0	0	0	0	0.960699	0	0	0	0	3.912023	0	0	0	0
AC074029.	64.3808	78.03045	66.94196	64.43615	35.43952	29.68906	93.46483	49.07943	15.17474	44.19217	34.93789	61.64524	52.4323	38.15953	44.01026	70.07841	48.23968	70.99041	56.4674
AC074029.	49.96719	56.02186	44.96101	85.22201	46.51437	55.95246	93.46483	32.41095	23.34575	75.89524	19.27608	52.44446	53.46039	26.99089	51.83431	46.1693	32.84404	48.23707	80.66772
AC074031.	0	0	0	0	0	0	0	0	0	0	0	3.680313	0	0.93072	1.956012	0	0	0.910133	0
AC074032.	179.6897	175.0683	262.7722	207.8586	241.4317	173.5668	152.0928	137.978	359.5246	180.6115	284.3222	171.1346	154.2127	165.6682	182.8871	104.7054	123.1652	171.1051	158.6465
AC074033.	111.4653	83.03241	78.93156	80.02554	97.45867	84.49964	75.62154	112.9753	115.5615	91.26643	84.33284	71.7661	62.71315	80.04195	70.41642	62.65834	66.71446	76.45121	65.43048
AC074033.	16.33543	6.002343	3.996535	2.078586	7.752394	2.283774	0	13.89041	0	4.803496	2.40951	4.600391	3.084253	6.515042	1.956012	2.473355	3.079129	10.9216	6.274156
AC074043.	0.960908	0	0	1.039293	0	3.425661	1.69936	3.704108	0	0	0	0	3.084253	0	1.956012	1.648904	1.026376	0	3.585232
AC074044.	3.84363	8.003124	0.999134	16.62868	0	1.141887	2.549041	0.926027	9.338302	3.842797	12.04755	1.840156	8.224675	5.584322	0.978006	9.06897	7.184634	6.370934	1.792616
AC074050.	0	0	0	1.039293	0	0	0.84968	0	9.338302	0	0	3.680313	0	0.93072	0	0	0	0	0
AC074050.	0	13.00508	4.995668	0	1.107485	7.993209	0.84968	5.556162	12.84017	3.842797	0	4.600391	5.140422	0.93072	0	0	4.105505	1.820267	3.585232
AC074051.	3.84363	0	0	0	0	0	0	0	2.334575	0	0	0	0	0	0	0	0	0	0

AC074051.	0	0	0	0	0	0	0	0	0	1.921399	0	1.840156	0	0	0	0	0	0	0
AC074051.	0	0	0.999134	0	6.64491	3.425661	0	0	14.00745	8.646293	0	0	0	0	0	4.946711	0	0	3.585232
AC074085.	0	0	7.993069	1.039293	0	0	0	0	0	0	0	0	0	0	0	0.824452	6.158258	0	0
AC074085.	0	0	0	0	0	0	0	0	0	0	0	0.920078	0	0	4.890029	1.648904	1.026376	0	0
AC074099.	5.765445	3.001171	0.999134	0	12.18233	0	7.647122	8.334243	67.70269	5.764196	1.204755	5.520469	13.3651	8.376483	10.75806	8.244518	0	11.83173	17.92616
AC074101.	37.47539	57.02225	54.95235	69.63262	39.86946	15.98642	114.7068	35.18903	0	29.78168	12.04755	88.32751	89.44334	81.90339	75.30645	41.22259	35.92317	66.43974	98.59387
AC074117.	858.0904	814.3178	1109.038	720.2299	1165.074	1051.678	858.177	1333.479	1320.202	1439.127	1403.539	1098.573	1030.141	1133.617	1222.507	1056.947	1198.807	1156.78	878.3818
AC074117.	32.67086	57.02225	14.987	32.21808	35.43952	11.41887	34.83689	11.11232	26.84762	60.52405	15.66181	30.36258	47.29188	22.33729	38.14223	16.48904	25.65941	54.608	29.57816
AC074121.	0.960908	0	0	0	7.752394	0	0.84968	0	0	0	0	0	0	0	0	0	0	5.4608	0
AC074124.	0.960908	2.000781	0	3.117878	1.107485	0	0	6.482189	0	13.44979	6.023774	4.600391	2.056169	0	0	2.473355	0	0	7.170464
AC074131.	8.648168	9.003514	11.9896	16.62868	5.537425	7.993209	5.098081	27.78081	2.334575	7.685594	2.40951	4.600391	2.056169	4.653602	2.934017	8.244518	5.131881	6.370934	8.066772
AC074134.	11.53089	4.001562	0.999134	0	3.322455	1.141887	0.84968	4.630135	0	1.921399	1.204755	5.520469	0	0	0	8.244518	4.105505	0.910133	0
AC074135.	45.16265	52.0203	47.95842	61.31827	21.04221	53.66869	56.92858	38.89314	3.501863	17.29259	16.86657	9.200782	21.58977	60.49682	50.8563	73.37621	59.52982	67.34987	69.01571
AC074135.	5.765445	0	3.996535	1.039293	0	3.425661	3.398721	3.704108	0	0	0	1.840156	6.168506	0.93072	4.890029	0	0	6.370934	0.896308
AC074138.	110.5044	191.0746	225.8042	175.6405	115.1784	140.4521	145.2953	117.6054	56.02981	165.2403	119.2707	215.2983	173.7463	103.31	177.0191	90.6897	149.8509	147.4416	261.7219
AC074140.	0	2.000781	1.998267	0	0	0	0	2.778081	0	0.960699	0	0.920078	0	0	0	0	2.052753	1.820267	0
AC074141.	632.2772	630.246	904.216	523.8036	845.011	599.4907	730.725	769.5285	552.1271	687.8607	698.7578	538.2458	568.5307	436.5078	485.0909	442.7306	450.5792	393.1776	407.8201
AC074194.	67.26353	118.0461	86.92463	139.2652	91.92125	42.24982	148.694	75.93422	14.00745	21.13538	30.11887	115.0098	119.2578	144.2616	91.93255	102.232	124.1915	57.33841	103.0754
AC074194.	46.12356	65.02538	101.9116	85.22201	93.02873	52.5268	176.7335	93.52873	16.34203	51.87776	31.32363	113.1696	93.55568	79.11123	109.5367	41.22259	74.92547	56.42827	93.21603
AC074198.	0	0	0	0	1.107485	0	0	0	0	0	0	0	0	0	0	0	0	0	0.896308
AC074212.	0	3.001171	0.999134	0	3.322455	2.283774	0	0.926027	38.5205	22.09608	83.12809	5.520469	1.028084	0.93072	0	0.824452	1.026376	3.640534	0
AC074276.	0	0	0	0	0	2.283774	0	0	0	0	1.204755	0	0	3.722881	0	4.122259	0	0	3.585232
AC074276.	0	0	0	0	0	0	1.69936	4.630135	0	0	0	0	5.140422	2.792161	1.956012	2.473355	0	0.910133	0
AC074281.	0	1.00039	0.999134	0	0	0	0.84968	0	0	0	0	0	1.028084	1.861441	0.978006	0	0	0	0
AC074286.	35.55358	58.02265	51.95495	88.33989	68.66407	30.83095	61.17698	33.33697	45.52422	40.34937	21.68559	62.56532	79.1625	101.4485	110.5147	19.78684	30.79129	50.05734	43.91909
AC074327.	231.5787	74.02889	141.877	131.9902	111.856	76.50643	90.06611	232.4328	346.6845	81.65944	92.76613	184.0156	123.3701	254.0866	228.8534	350.392	205.2753	526.0571	306.5373
AC074338.	0	1.00039	0	1.039293	0	0	0	0	0	1.921399	3.614265	1.840156	2.056169	2.792161	0.978006	0.824452	3.079129	0.910133	0
AC074348.	4.804538	0	0	0	0	0	0	0	0	0	0	0	0	0	0	0	0	0	0
AC074351.	1.921815	1.00039	13.98787	3.117878	4.42994	3.425661	4.248401	16.66849	21.01118	0.960699	6.023774	1.840156	4.112337	15.82225	5.868035	10.71787	4.105505	5.4608	25.99293
AC074366.	3.84363	4.001562	0.999134	5.196464	0	0	0.84968	0	0	0	0	4.600391	1.028084	0	0	1.648904	1.026376	1.820267	0.896308
AC074367.	10.56998	10.0039	4.995668	3.117878	9.967364	11.41887	4.248401	18.52054	0	0	0	6.440548	6.168506	7.445762	5.868035	14.84013	1.026376	2.7304	2.688924
AC074375.	0	0	0	0	1.107485	0	0	0	0	0	0	0.920078	4.112337	0	0	0	0	0	3.585232
AC074386.	108.5826	106.0414	179.8441	67.55403	108.5335	126.7495	59.47762	110.1972	39.68778	66.28825	72.28529	69.00587	97.66802	2.792161	6.846041	157.4703	127.2707	56.42827	73.49725
AC074387.	26.90541	49.01913	41.96361	30.13949	5.537425	15.98642	14.44456	27.78081	77.04099	53.79916	19.27608	25.76219	22.61786	18.61441	17.6041	28.03136	7.184634	48.23707	23.30401
AC074389.	0	0	0	0	1.107485	0	0	0	0	0	0	0	0	0.93072	0	0	1.026376	0	0.896308
AC074389.	0	0	0	0	6.64491	0	0	5.556162	4.669151	0	0	0	0	0	0	0	0	0.910133	0
AC074389.	0	2.000781	0	0	0	0	0	0	0	0	0	0	0	1.861441	2.934017	1.648904	0	0	0
AC076966.	0.960908	1.00039	0.999134	0	0	1.141887	1.69936	6.482189	0	0	0	3.680313	0	0	0	0	0	0.910133	6.274156
AC078776.	0	0	0	0	0	0	0	2.778081	0	0	0	5.520469	0	0	0.978006	0	0	0	0
AC078777.	11.53089	3.001171	6.993936	15.58939	2.21497	13.70264	10.19616	7.408216	23.34575	11.52839	12.04755	11.96102	8.224675	17.68369	0	14.01568	15.39564	0	4.48154
AC078778.	56.69354	90.03514	103.9099	41.57171	69.77155	92.49285	55.22922	80.56435	52.52795	159.4761	155.4134	69.92595	87.38717	80.04195	91.93255	40.39814	40.02867	118.3173	86.94187
AC078778.	42.27993	15.00586	75.93416	35.33595	32.11706	17.12831	50.13113	34.263	0	22.09608	20.48083	36.80313	25.70211	29.78305	55.74633	35.45143	20.52753	77.36134	36.74863
AC078785.	57.65445	47.01835	87.92376	115.3615	68.66407	55.95246	121.5043	77.78627	44.35693	42.27077	46.98544	98.44837	96.63993	66.08114	63.57038	51.94047	65.68808	80.09174	79.77141
AC078785.	1.921815	1.00039	8.992203	0	1.107485	3.425661	18.69297	4.630135	0	5.764196	0	6.440548	3.084253	0.93072	0	4.122259	12.31652	8.191201	0.896308
AC078785.	0.960908	0	1.998267	2.078586	0	0	3.398721	0	0	1.921399	0	5.520469	1.028084	0.93072	7.824047	0.824452	0	1.820267	0.896308
AC078788.	15.37452	17.00664	18.98354	6.235757	13.28982	10.27698	10.19616	15.74246	22.17847	3.842797	9.638039	14.72125	10.28084	24.19873	29.34017	4.946711	11.29014	18.20267	2.688924
AC078788.	11.53089	9.003514	13.98787	4.157171	6.64491	9.135096	5.947762	10.1863	0	10.56769	4.81902	11.96102	4.112337	12.09936	23.47214	2.473355	2.052753	10.01147	3.585232
AC078789.	0	0	5.994802	2.078586	0	0	0.84968	1.852054	0	0	0	1.840156	1.028084	1.861441	0.978006	0	0	0	0.896308
AC078795.	110.5044	168.0656	245.7869	166.2868	171.6602	163.2898	231.9627	154.6465	121.3979	142.1835	197.5798	239.2203	199.4484	132.1623	159.4149	223.4264	204.2489	124.6883	158.6465
AC078802.	10.56998	29.01132	20.98181	18.70727	12.18233	14.84453	19.54265	15.74246	19.84389	18.25329	14.45706	8.280704	5.140422	29.78305	36.18622	6.595615	22.58028	18.20267	18.82247
AC078814.	310.3731	338.132	247.7851	263.9804	240.3242	294.6068	223.4659	266.6958	339.6807	238.2534	266.2508	170.2145	163.4654	233.6108	265.0396	257.229	305.8601	302.1643	202.5656
AC078817.	4.804538	3.001171	1.998267	4.157171	12.18233	1.141887	1.69936	12.96438	10.50559	3.842797	0	5.520469	0	2.792161	6.846041	4.122259	6.158258	0	5.377848
AC078819.	23.06178	30.01171	16.98527	30.13949	18.82724	12.56076	18.69297	19.44657	0	29.78168	7.228529	21.1618	16.44935	22.33729	24.45015	28.03136	30.79129	24.5736	19.71877
AC078820.	0.960908	15.00586	15.98614	5.196464	9.967364	18.27019	10.19616	24.0767	4.669151	3.842797	1.204755	12.8811	6.168506	12.09936	12.71408	9.06897	10.26376	4.550667	5.377848
AC078828.	2.882723	7.002733	26.97661	5.196464	14.3973	14.84453	24.64073	25.92876	0	16.33189	12.04755	15.64133	43.17954	12.09936	14.67009	14.84013	7.184634	9.101334	18.82247
AC078841.	0	2.000781	0	0	0	0	0	0	0	0	0	0	3.084253	0	0	0	0	0	0
AC078842.	43.24084	43.01679	60.94715	84.18271	22.1497	34.25661	61.17698	37.96711	39.68778	58.60265	86.74235	82.80704	76.07824	26.99089	26.40616	32.15362	69.79359	72.81067	67.2231
AC078842.	0	1.00039	0.999134	0	5.537425	1.141887	0.84968	0	0	0	0	1.840156	6.168506	0	0	0	0	0.910133	5.377848
AC078845.	48.04538	40.01562	11.9896	27.02161	8.859879	10.27698	3.398721	15.74246	0	22.09608	4.81902	23.92203	23.64594	31.64449	47.92229	45.34485	7.184634	42.77627	30.47447
AC078846.	921.5103	920.3592	1504.695	778.4303	1074.26	657.7269	1016.218	1046.411	1164.953	846.376	1008.38	936.6396	997.2418	964.2262	1158.937	898.6525	800.5735	789.9958	874.7966
AC078850.	13.45271	20.00781	10.99047	27.02161	40.97694	25.12151	43.33369	22.22465	26.84762	24.97818	67.46627	19.32164	21.58977	43.74385	59.65836	75.02512	54.39794	50.05734	23.30401
AC078850.	3.84363	9.003514	15.98614	6.235757	6.64491	1.141887	3.398721	12.96438	0	10.56769	6.023774	6.440548	7.196591	14.89152	21.51613	1.648904	5.131881	6.370934	2.688924

AC078851.	1.921815	0	0	0	0	0	0	0	0	0	0	0	0	0	0	0	0	0	0
AC078852.	0	0	1.998267	0	0	0	4.248401	0	18.6766	1.921399	10.84279	0	3.084253	0.93072	0	0.824452	4.105505	6.370934	2.688924
AC078852.	0	0	0.999134	0	0	0	0	0	9.338302	0.960699	7.228529	0.920078	0	2.792161	0	0	0	0	2.688924
AC078857.	0	1.00039	0	0	0	0	0.84968	0	0	0	0	0	0	0	0	0.824452	0	0	0.896308
AC078860.	180.6506	531.2073	706.3875	427.1493	425.2742	412.2212	585.4297	374.1149	156.4166	583.1444	501.178	271.4231	237.4875	343.4358	412.7185	199.5173	247.3567	269.3995	492.0731
AC078864.	0.960908	6.002343	23.97921	4.157171	4.42994	2.283774	3.398721	0	0	1.921399	0	0.920078	2.056169	0.93072	7.824047	2.473355	6.158258	0	1.792616
AC078867.	0	0	0	1.039293	1.107485	0	0	0	0	0	0	0	0	3.722881	3.912023	0	0	0	0
AC078875.	1.921815	5.001952	0.999134	1.039293	0	4.567548	0.84968	7.408216	2.334575	0	1.204755	0.920078	0	0.93072	1.956012	3.297807	1.026376	0.910133	2.688924
AC078880.	0	0	0	7.275049	0	4.567548	0	7.408216	0	0	0	0	0	0.93072	0	8.244518	6.158258	0	0
AC078880.	3.84363	1.00039	0	0	0	0	0	0	0	0	0	0	0	0	0	0	0	0.910133	0
AC078880.	12.4918	14.00547	11.9896	0	0	4.567548	1.69936	0	9.338302	9.606993	2.40951	5.520469	4.112337	1.861441	6.846041	0.824452	2.052753	11.83173	3.585232
AC078880.	0	0	6.993936	1.039293	0	0	0	0	0	0	2.40951	1.840156	2.056169	0	0	0.824452	0	0	0.896308
AC078880.	0	4.001562	15.98614	0	7.752394	4.567548	2.549041	0	0	0	0	5.520469	5.140422	2.792161	3.912023	1.648904	4.105505	7.281067	7.170464
AC078881.	128.7616	141.0551	200.8259	144.4617	184.95	141.594	98.56291	132.4219	182.0969	142.1835	202.3988	152.733	144.9599	213.1349	184.8431	108.0032	126.2443	109.216	145.2019
AC078882.	0.960908	0	0	0	0	0	0	0	0	0	0	0	4.112337	0	0	0	0	0	0
AC078883.	447.7829	546.2132	580.4967	273.334	689.9631	413.3631	517.4553	523.2053	494.93	536.0702	374.6788	615.5323	536.66	592.8688	493.8929	347.0942	280.2007	351.3115	302.9521
AC078883.	5.765445	1.00039	1.998267	0	8.859879	1.141887	0.84968	0.926027	0	0	4.81902	1.840156	0	0	0	0.824452	0	0	0
AC078883.	36.51449	13.00508	17.98441	27.02161	32.11706	25.12151	4.248401	29.63287	37.35321	36.50657	24.0951	21.1618	21.58977	35.36737	33.2522	23.08465	20.52753	30.0344	17.92616
AC078883.	27.86632	35.01367	34.96968	11.43222	52.05179	2.283774	26.34009	29.63287	5.836439	9.606993	15.66181	30.36258	21.58977	21.40657	21.51613	16.48904	4.105505	20.93307	1.792616
AC078886.	0	1.00039	6.993936	3.117878	0	0	2.549041	1.852054	0	7.685594	16.86657	4.600391	1.028084	0	3.912023	0	1.026376	1.820267	0.896308
AC078889.	5.765445	5.001952	6.993936	8.314342	11.07485	2.283774	3.398721	1.852054	2.334575	6.724895	3.614265	2.760235	0	1.861441	5.868035	2.473355	2.052753	11.83173	4.48154
AC078899.	17.29634	14.00547	8.992203	15.58939	3.322455	12.56076	31.43817	12.96438	2.334575	3.842797	2.40951	7.360626	10.28084	10.23792	1.956012	12.36678	9.237386	21.8432	13.44462
AC078899.	0	0	0	0	0	1.141887	0	0.926027	0	0	0	0	1.028084	0	1.956012	2.473355	0	5.4608	0
AC078899.	0	1.00039	0	0	0	2.283774	0	0	0	0	0	0	0	1.861441	0	0	0	0.910133	0.896308
AC078899.	8.648168	7.002733	3.996535	2.078586	6.64491	5.709435	5.947762	7.408216	0	0.960699	0	7.360626	7.196591	5.584322	2.934017	3.297807	3.079129	6.370934	8.963079
AC078899.	0	0	0	2.078586	0	0	0	1.852054	0	0	0	0	0	0	0	0.824452	0	0	0
AC078905.	5.765445	0	2.997401	0	1.107485	1.141887	0	0	0	0.960699	0	1.840156	1.028084	0.93072	0.978006	0	10.26376	0	0
AC078906.	33.63176	4.001562	19.98267	95.61493	23.25718	29.68906	34.83689	28.70684	33.85134	27.86028	49.39495	28.52243	23.64594	25.12945	36.18622	79.97183	63.63533	25.48374	25.99293
AC078909.	5.765445	13.00508	1.998267	4.157171	11.07485	10.27698	8.496802	11.11232	33.85134	50.91706	37.3474	4.600391	15.42127	21.40657	24.45015	18.13794	15.39564	13.652	13.44462
AC078922.	1.921815	0	1.998267	7.275049	11.07485	2.283774	11.04584	7.408216	0	0	0	0.920078	0	0	0	8.244518	10.26376	3.640534	1.792616
AC078923.	5.765445	12.00469	20.98181	18.70727	8.859879	26.2634	7.647122	17.59451	7.003726	22.09608	24.0951	20.24172	9.252759	13.9608	14.67009	5.771163	15.39564	10.9216	9.859387
AC078925.	0	2.000781	0	0	0	0	0.84968	1.852054	0	0.960699	15.66181	1.840156	2.056169	4.653602	1.956012	7.420066	4.105505	1.820267	6.274156
AC078925.	0	0	3.996535	6.235757	0	1.141887	2.549041	0	1.167288	3.842797	0	0.920078	9.252759	0.93072	6.846041	2.473355	1.026376	3.640534	0
AC078925.	0	0	6.993936	0	3.322455	0	0	1.852054	0	17.29259	4.81902	0.920078	2.056169	1.861441	1.956012	11.54233	0	1.820267	4.48154
AC078925.	0	1.00039	12.98874	11.43222	0	5.709435	0.84968	2.778081	25.68033	6.724895	1.204755	7.360626	15.42127	17.68369	4.890029	6.595615	8.21101	15.47227	3.585232
AC078929.	0	6.002343	0	6.235757	2.21497	2.283774	0	0	0	0	0	0	4.112337	0.93072	0	0	0	8.191201	0.896308
AC078942.	0	0	0.999134	4.157171	0	0	0	0	0	4.803496	7.228529	2.760235	0	4.653602	0.978006	23.9091	7.184634	0.910133	2.688924
AC078950.	0.960908	1.00039	1.998267	2.078586	1.107485	0	1.69936	7.408216	0	0	6.023774	0.920078	5.140422	1.861441	0.978006	1.648904	3.079129	14.56213	3.585232
AC078955.	0	1.00039	7.993069	0	0	1.141887	0.84968	2.778081	7.003726	0.960699	0	0.920078	2.056169	1.861441	0	4.122259	0	0.910133	0
AC078960.	0	0	2.997401	3.117878	0	0	1.69936	0	4.669151	1.921399	1.204755	4.600391	1.028084	0.93072	7.824047	1.648904	7.184634	0	0.896308
AC078962.	1.921815	7.002733	0.999134	0	22.1497	6.851322	0.84968	0.926027	14.00745	2.882098	25.29985	6.440548	1.028084	0.93072	0.978006	3.297807	7.184634	1.820267	5.377848
AC078962.	0.960908	0	0.999134	0	0	0	0	0.926027	0	0	0	0	0	0	0	1.648904	1.026376	0	0
AC078962.	0	0	0	1.039293	1.107485	1.141887	3.398721	0	0	0.960699	1.204755	0.920078	0	0.93072	0.978006	0	0	0	0.896308
AC078962.	0	0	0	0	11.07485	0	0	0	0	0	7.228529	0	0	0	0	0	0	0.910133	0
AC078991.	0.960908	1.00039	0	0	0	0	0	0	0	0	0	0	0	0	0	0	0	0	0
AC078993.	108.5826	60.02343	47.95842	96.65423	83.06137	46.81737	83.26866	36.11505	44.35693	57.64196	55.41872	70.84602	58.60081	70.73474	60.63636	79.14738	87.24198	77.36134	72.60094
AC079015.	639.9644	534.2085	543.5287	348.1631	545.9901	372.2552	276.9958	646.3669	657.183	389.0832	384.3168	336.7486	356.7453	837.6483	834.239	203.6396	252.4886	226.6232	235.729
AC079031.	0	0	0	0	0	0	0	0	0	0	0	0	0	0	0	0	3.079129	0	0
AC079031.	0	0	2.997401	0	0	0	0.84968	0	0	9.606993	0	0.920078	6.168506	0	0	0	6.158258	0	0.896308
AC079035.	0	2.000781	16.98527	9.353635	1.107485	11.41887	15.29424	1.852054	11.67288	8.646293	14.45706	0.920078	1.028084	6.515042	0	6.595615	0	13.652	0
AC079054.	0.960908	0	0	0	0	1.141887	1.69936	0	1.167288	0.960699	0	0	0	0	0	0	0	2.7304	0
AC079061.	216.2042	211.0824	181.8423	542.5108	186.0575	227.2355	524.2527	187.9835	60.69896	160.4368	122.885	303.6258	240.5717	262.4631	292.4237	432.0128	459.8166	319.4568	429.3315
AC079062.	1.921815	0	10.99047	8.314342	4.42994	161.0061	0	0	0	0.960699	1.204755	0	0	0	4.890029	2.473355	3.079129	0	3.585232
AC079070.	1.921815	0	0	0	0	0	0	0.926027	0	0	0	0	0	0	0	0.824452	0	0	2.688924
AC079075.	0	5.001952	0	0	0	2.283774	1.69936	0	0	0	14.45706	0	1.028084	0.93072	0	0	1.026376	3.640534	0
AC079075.	9.609075	10.0039	12.98874	1.039293	0	5.709435	2.549041	11.11232	8.171014	2.882098	9.638039	1.840156	23.64594	5.584322	7.824047	11.54233	1.026376	6.370934	7.170464
AC079080.	0	0	0	12.47151	6.64491	0	11.89552	12.03835	0	3.842797	0	0	3.084253	4.653602	0	0.824452	9.237386	0	0
AC079089.	60.53717	82.03202	63.94455	86.2613	50.94431	83.35775	104.5107	49.07943	87.54658	97.99132	91.56137	37.72321	27.75828	70.73474	71.39443	94.81196	81.08372	34.58507	75.28987
AC079089.	0	0	0	1.039293	0	0	5.098081	0.926027	0	0	0	0.920078	5.140422	0	1.956012	0	1.026376	0	0
AC079098.	5.765445	0	1.998267	3.117878	0	0	4.248401	1.852054	0	0	0	0.920078	1.028084	0.93072	0.978006	0.824452	6.158258	0	0.896308
AC079117.	0.960908	0	0	0	0	0	0	0.926027	0	0.960699	0	0.920078	0	0.93072	0	6.595615	7.184634	0	1.792616
AC079140.	0.960908	1.00039	3.996535	0	0	5.709435	0.84968	8.334243	0	0.960699	0	9.200782	5.140422	3.722881	0.978006	4.946711	6.158258	7.281067	7.170464

AC079140.	2.882723	4.001562	5.994802	4.157171	4.42994	3.425661	2.549041	3.704108	17.50932	12.48909	7.228529	1.840156	6.168506	5.584322	8.802052	4.122259	3.079129	2.7304	6.274156
AC079140.	172.9634	110.0429	209.8181	117.4401	126.2533	140.4521	111.3081	141.6821	61.86625	150.8298	125.2945	159.1735	164.4935	178.6983	186.7991	141.8057	110.8486	144.7112	118.3126
AC079140.	15.37452	7.002733	29.97401	7.275049	3.322455	13.70264	10.19616	8.334243	5.836439	0.960699	0	8.280704	17.47743	7.445762	11.73607	8.244518	14.36927	19.1128	14.34093
AC079140.	13.45271	17.00664	14.987	16.62868	7.752394	23.97963	8.496802	13.89041	7.003726	22.09608	15.66181	26.68227	5.140422	16.75297	22.49413	15.66458	5.131881	19.1128	8.066772
AC079145.	50.9281	121.0472	129.8874	128.8723	126.2533	98.20228	206.4723	112.9753	79.37557	97.03062	55.41872	87.40743	63.74123	122.8551	132.0308	57.71163	49.26606	67.34987	114.7274
AC079148.	109.5435	96.03748	99.91337	48.84676	81.95388	97.0604	74.77186	87.04654	148.2455	165.2403	108.4279	110.4094	98.6961	76.31906	92.91055	59.36053	61.58258	87.37281	73.49725
AC079154.	19.21815	14.00547	3.996535	14.5501	0	5.709435	23.79105	7.408216	0	5.764196	7.228529	17.48149	9.252759	0.93072	10.75806	7.420066	6.158258	7.281067	25.99293
AC079160.	0.960908	0	0	0	6.64491	0	1.69936	0	0	0	0	0	0	0	0	0	0	0	0
AC079168.	3.84363	0	0	0	0	0	0	0	0	0	0	0	0	0	0	0	0	0	0
AC079174.	8.648168	6.002343	7.993069	7.275049	6.64491	3.425661	4.248401	2.778081	5.836439	14.41049	1.204755	6.440548	11.30893	6.515042	14.67009	8.244518	20.52753	5.4608	8.963079
AC079174.	396.8548	550.2147	690.4014	411.5599	455.1763	524.1261	433.3369	448.1971	1299.191	675.3716	850.5569	484.8812	528.4354	505.3811	495.849	382.5456	323.3085	465.9883	504.6214
AC079193.	5.765445	2.000781	3.996535	13.51081	5.537425	3.425661	4.248401	16.66849	14.00745	0	2.40951	0.920078	9.252759	5.584322	3.912023	17.31349	16.42202	1.820267	7.170464
AC079193.	89.3644	38.01484	60.94715	110.165	26.57964	26.2634	73.0725	50.93149	60.69896	86.46293	19.27608	82.80704	75.05016	49.32818	32.27419	58.53608	56.45069	101.0248	37.64493
AC079203.	39.39721	66.02577	102.9108	27.02161	19.93473	101.6279	57.77826	62.04381	30.34948	78.77734	101.1994	46.92399	48.31997	69.80402	53.79032	34.62698	21.5539	36.40534	41.23017
AC079203.	0	3.001171	0	1.039293	0	0	1.69936	0	0	0	0	0.920078	0	0.93072	0.978006	0.824452	0	0.910133	0.896308
AC079209.	57.65445	5.001952	21.98094	29.1002	19.93473	20.55397	4.248401	9.26027	4.669151	23.05678	6.023774	23.00196	2.056169	20.47585	6.846041	57.71163	35.92317	31.85467	15.23724
AC079209.	0	2.000781	21.98094	0	0	3.425661	9.346483	4.630135	0	7.685594	0	8.280704	7.196591	1.861441	0.978006	0	2.052753	0	0
AC079210.	22.10087	14.00547	10.99047	8.314342	3.322455	4.567548	5.098081	21.29862	95.71759	17.29259	22.89034	19.32164	23.64594	6.515042	20.53812	60.18498	62.60895	43.6864	28.68185
AC079226.	0	2.000781	0	2.078586	2.21497	0	0	4.630135	0	0	0	0.920078	0	0	0	0	0	0	0
AC079226.	0.960908	0	0	0	0	0	0	0	0	0	0	0.920078	0	0	0	0	1.026376	0	0
AC079228.	24.02269	32.01249	31.97228	33.25737	38.76197	37.68227	12.7452	26.85478	29.18219	26.89958	50.5997	19.32164	25.70211	21.40657	26.40616	33.80253	26.68578	23.66347	26.88924
AC079230.	2.882723	10.0039	25.97748	10.39293	1.107485	23.97963	11.89552	35.18903	15.17474	28.82098	24.0951	11.04094	28.78636	13.9608	10.75806	9.893422	15.39564	1.820267	35.85232
AC079238.	0	4.001562	3.996535	0	0	0	0	0	0	0	0	0	0	0	0	0	1.026376	0	0.896308
AC079250.	16.33543	7.002733	3.996535	10.39293	15.50479	6.851322	2.549041	7.408216	26.84762	13.44979	45.78069	24.84211	10.28084	6.515042	22.49413	18.13794	25.65941	16.3824	11.652
AC079296.	41.31902	26.01015	25.97748	36.37525	19.93473	25.12151	62.02666	53.70957	0	25.93888	12.04755	33.12282	50.37613	20.47585	32.27419	28.85581	55.42432	26.39387	52.88217
AC079298.	0	1.00039	0	1.039293	1.107485	6.851322	1.69936	0.926027	1.167288	0	0	12.8811	0	3.722881	0.978006	0.824452	1.026376	4.550667	0
AC079298.	0	0	0	0	0	0	0	2.778081	0	0	0	0	1.028084	0.93072	0	0	1.026376	0.910133	0
AC079298.	22.10087	24.00937	36.96795	17.66798	15.50479	21.69585	26.34009	27.78081	4.669151	16.33189	10.84279	17.48149	24.67402	48.39746	80.19648	44.5204	19.50115	26.39387	21.51139
AC079305.	13.45271	30.01171	19.98267	16.62868	5.537425	6.851322	16.9936	15.74246	22.17847	22.09608	36.14265	16.56141	12.33701	7.445762	20.53812	9.893422	1.026376	12.74187	31.37078
AC079305.	4.804538	5.001952	2.997401	0	0	5.709435	3.398721	2.778081	4.669151	2.882098	2.40951	2.760235	8.224675	9.307203	8.802052	2.473355	1.026376	0.910133	4.48154
AC079313.	12.4918	43.01679	33.97054	33.25737	47.62185	22.83774	33.13753	30.55889	0	3.842797	15.66181	45.08383	26.73019	14.89152	32.27419	30.50472	46.18693	24.5736	12.54831
AC079313.	1.921815	2.000781	11.9896	5.196464	2.21497	4.567548	5.947762	9.26027	0	6.724895	0	4.600391	3.084253	0.93072	0.978006	7.420066	8.21101	7.281067	5.377848
AC079315.	245.9923	270.1054	209.8181	246.3124	261.3664	487.5858	174.1844	237.9889	768.0753	495.7208	712.0101	242.9007	230.2909	228.9572	225.9193	253.1067	357.1789	261.2083	342.3896
AC079316.	0	0	0	0	0	1.141887	0	0.926027	18.6766	0	7.228529	2.760235	4.112337	0	0.978006	7.420066	0	0	0
AC079316.	0	6.002343	3.996535	2.078586	3.322455	1.141887	5.947762	0.926027	2.334575	1.921399	0	0.920078	13.3651	7.445762	13.69208	5.771163	3.079129	7.281067	1.792616
AC079322.	103.778	92.03592	111.903	46.76817	78.63143	61.6619	51.83049	83.34243	137.74	102.7948	120.4755	70.84602	65.7974	86.55699	75.30645	94.81196	84.16285	81.91201	75.28987
AC079325.	2.882723	0	1.998267	1.039293	3.322455	0	3.398721	0.926027	0	0.960699	0	1.840156	0	0	0.978006	0.824452	0	1.820267	1.792616
AC079325.	1.921815	2.000781	7.993069	0	6.64491	2.283774	15.29424	0	5.836439	0.960699	19.27608	7.360626	2.056169	9.307203	5.868035	22.2602	11.29014	228.4435	128.172
AC079328.	0	0	0.999134	0	1.107485	2.283774	0	0	0	1.921399	0	0.920078	1.028084	0	2.934017	0	0	2.7304	0.896308
AC079328.	40.35812	91.03553	93.91856	75.86837	74.20149	45.67548	65.42538	97.23284	172.7586	73.97384	101.1994	39.56336	28.78636	67.94258	53.79032	54.41382	91.34749	93.74374	123.6905
AC079331.	3.84363	1.00039	1.998267	0	0	3.425661	0	0	0	6.724895	0	8.280704	1.028084	0.93072	0	4.946711	0	8.191201	0
AC079331.	24.02269	21.0082	20.98181	11.43222	17.71976	22.83774	14.44456	37.96711	57.1971	12.48909	30.11887	11.04094	5.140422	37.22881	18.58211	26.38246	15.39564	51.8776	18.82247
AC079336.	0	0	0	4.157171	0	1.141887	0	0	7.003726	0.960699	0	0.920078	0	0	0.978006	0	0	0	0
AC079336.	3.84363	6.002343	19.98267	8.314342	19.93473	7.993209	5.098081	5.556162	23.34575	14.41049	8.433284	20.24172	18.50552	26.06017	15.64809	12.36678	1.026376	16.3824	6.274156
AC079336.	0	1.00039	0	0	0	0	0	0	0	0	0	0.920078	1.028084	0.93072	0	0	0	0	0
AC079336.	0	0	0.999134	0	0	0	0	0	1.167288	0	0	0.920078	0	0	0	0	1.026376	0	0
AC079336.	39.39721	29.01132	75.93416	53.00393	25.47215	13.70264	34.83689	54.6356	29.18219	68.20965	110.8374	71.7661	39.06721	26.99089	46.94428	53.58937	49.26606	34.58507	60.94894
AC079336.	0.960908	3.001171	6.993936	1.039293	0	0	1.69936	10.1863	8.171014	0	1.204755	0.920078	0	0	6.846041	3.297807	3.079129	7.281067	0.896308
AC079336.	7.68726	9.003514	0.999134	1.039293	0	2.283774	11.89552	4.630135	0	7.685594	1.204755	22.08188	9.252759	4.653602	2.934017	15.66458	2.052753	4.550667	3.585232
AC079340.	5.765445	2.000781	3.996535	8.314342	2.21497	3.425661	15.29424	0.926027	0	1.921399	0	0	1.028084	5.584322	0.978006	9.893422	8.21101	12.74187	8.066772
AC079340.	8.648168	7.002733	14.987	9.353635	4.42994	6.851322	0.84968	0.926027	22.17847	0.960699	12.04755	8.280704	8.224675	2.792161	0.978006	9.06897	6.158258	0	6.274156
AC079341.	1.921815	0	0.999134	9.353635	2.21497	2.283774	4.248401	0.926027	1.167288	0.960699	0	0	6.168506	4.653602	11.73607	1.648904	6.158258	4.550667	1.792616
AC079349.	0	0	0.999134	0	0	0	0	0	0	0	0	0	0	1.861441	0	0	0	0	0
AC079354.	35.55358	37.01445	33.97054	29.1002	55.37425	28.54718	37.38593	27.78081	22.17847	33.62447	30.11887	57.04485	40.09529	27.92161	38.14223	23.9091	35.92317	26.39387	37.64493
AC079354.	0	0	0	0	2.21497	0	0	0	0	0	3.614265	0	0	0	0	0	0	0	0
AC079354.	13.45271	34.01327	29.97401	23.90373	25.47215	45.67548	22.94137	20.37259	37.35321	46.11356	85.5376	22.08188	16.44935	26.99089	19.56012	18.96239	27.71216	20.02294	22.4077
AC079355.	5.765445	0	0	0	0	0	3.398721	0	2.334575	0	0	1.840156	4.112337	2.792161	3.912023	0	0	3.640534	1.792616
AC079360.	0.960908	0	0.999134	0	0	0	2.549041	0.926027	0	0.960699	0	2.760235	1.028084	0	0.978006	1.648904	0	8.191201	4.48154
AC079362.	0	0	0	1.039293	0	0	0	0	0	0	0	0.920078	3.084253	0	0	0	0	0	0
AC079363.	0	0	0	0	0	0	0	0	0	0	0	0	1.028084	0	0	0	5.131881	0	0
AC079380.	0	1.00039	2.997401	1.039293	11.07485	3.425661	3.398721	0.926027	0	9.606993	6.023774	6.440548	6.168506	0.93072	6.846041	1.648904	7.184634	3.640534	0.896308
AC079384.	0	1.00039	0	1.039293	2.21497	0	0	0	0	0	0	0	0	0	0	0	0	0	0

AC079385.	20.17906	18.00703	21.98094	7.275049	15.50479	21.69585	18.69297	24.0767	4.669151	20.17468	14.45706	23.00196	18.50552	11.16864	19.56012	31.32917	9.237386	17.29253	11.652
AC079385.	12.4918	5.001952	1.998267	0	0	5.709435	0.84968	0.926027	1.167288	3.842797	0	1.840156	1.028084	5.584322	11.73607	3.297807	9.237386	0	3.585232
AC079385.	24.9836	14.00547	15.98614	19.74656	9.967364	34.25661	30.58849	12.03835	43.18965	28.82098	31.32363	8.280704	5.140422	21.40657	17.6041	12.36678	25.65941	20.02294	15.23724
AC079395.	0	0	0	0	0	0	0.84968	0	0	0	0	0	0	0	0	0	0	0	8.963079
AC079395.	0	0	2.997401	0	0	0	0	0	0	0	0	0	0	0	0	0	0	0	0.896308
AC079395.	0.960908	0	2.997401	0	0	0	0	0	0	0	0	0	2.056169	0	0	0	0	0	0
AC079411.	0	0	0	5.196464	0	0	1.69936	3.704108	0	1.921399	0	0	0	0	0.978006	0	1.026376	0	0.896308
AC079414.	268.0932	163.0636	187.8371	312.8271	260.259	244.3638	168.2367	123.1616	503.101	317.0308	286.7317	208.8578	207.673	219.65	230.8094	182.2039	138.5608	132.8795	112.0385
AC079416.	7.68726	15.00586	17.98441	16.62868	26.57964	9.135096	21.24201	26.85478	1.167288	14.41049	10.84279	21.1618	19.5336	14.89152	25.42815	24.73355	18.47477	40.04587	34.95601
AC079416.	13.45271	17.00664	9.991337	14.5501	28.79461	9.135096	31.43817	29.63287	11.67288	3.842797	15.66181	28.52243	8.224675	13.03008	17.6041	5.771163	1.026376	7.281067	9.859387
AC079416.	0	2.000781	4.995668	1.039293	1.107485	3.425661	4.248401	2.778081	2.334575	0.960699	13.2523	1.840156	2.056169	0.93072	5.868035	1.648904	1.026376	0	1.792616
AC079447.	69.18534	90.03514	114.9004	90.41847	125.1458	105.0536	84.96802	98.15887	84.04472	122.9695	116.8612	62.56532	143.9318	100.5178	92.91055	78.32292	71.84634	71.90054	56.4674
AC079448.	0.960908	1.00039	3.996535	1.039293	1.107485	13.70264	0.84968	1.852054	1.167288	0.960699	1.204755	6.440548	1.028084	0	1.956012	1.648904	0	0	3.585232
AC079456.	0	0	0	0	0	0	0	0	0	0	0	0	0	0	0	0	0	1.820267	0
AC079465.	38.4363	31.0121	27.97574	25.98232	24.36467	30.83095	16.14392	16.66849	31.51677	23.05678	71.08054	21.1618	31.87062	13.03008	20.53812	32.15362	34.89679	4.550667	30.47447
AC079466.	0	0	0	0	0	0	0	4.630135	0	0.960699	1.204755	5.520469	4.112337	0	0	0	4.105505	0	0
AC079467.	39.39721	79.03084	45.96015	136.1474	245.8617	0	73.0725	56.48765	11.67288	22.09608	18.07132	19.32164	7.196591	27.92161	38.14223	52.76492	16.42202	11.83173	10.7557
AC079584.	0	0	0	0	0	0	0	0.926027	0	0	0	0	0	0	0	0	0	0	0.896308
AC079584.	59.57627	72.02811	92.91943	11.43222	33.22455	36.54038	32.28785	25.92876	43.18965	84.54153	72.28529	74.52634	59.62889	78.18051	87.04252	17.31349	3.079129	34.58507	70.80833
AC079594.	4.804538	1.00039	0.999134	2.078586	3.322455	4.567548	11.04584	3.704108	3.501863	1.921399	0	7.360626	7.196591	0	1.956012	0.824452	4.105505	0	0
AC079598.	0.960908	0	0	0	0	4.567548	0	0.926027	0	1.921399	0	0	0	0	2.934017	0	1.026376	0	0
AC079598.	5.765445	0	0	0	1.107485	4.567548	0	0.926027	1.167288	0	4.81902	0	1.028084	0	0.978006	0.824452	1.026376	0	2.688924
AC079598.	0.960908	0	0	0	0	0	0	0	0	0	0	0.920078	0	0	0	0	0	0.910133	0
AC079600.	0	0	0	0	0	1.141887	0.84968	0.926027	1.167288	0	0	0	0	0	0	0.824452	0	0	0
AC079600.	0.960908	0	6.993936	3.117878	0	1.141887	0.84968	3.704108	3.501863	5.764196	7.228529	0	1.028084	0	0.978006	0.824452	0	0	0.896308
AC079601.	3.84363	12.00469	19.98267	6.235757	13.28982	35.3985	9.346483	0.926027	37.35321	15.37119	8.433284	20.24172	8.224675	0.93072	12.71408	14.84013	9.237386	4.550667	6.274156
AC079601.	19.21815	11.00429	27.97574	30.13949	36.547	6.851322	50.13113	20.37259	8.171014	19.21399	21.68559	34.04289	27.75828	31.64449	35.20821	15.66458	5.131881	26.39387	18.82247
AC079602.	1.921815	3.001171	0	0	0	0	2.549041	0	0	0	0	0.920078	5.140422	0.93072	0	0	0	0	1.792616
AC079602.	0	0	0.999134	0	0	0	0	0	11.67288	0	0	0	1.028084	0	0	0	0	0	0
AC079602.	0	0	5.994802	0	0	0	0	0.926027	0	0	0	4.600391	0	6.515042	0	0.824452	0	0	0.896308
AC079610.	39.39721	44.01718	41.96361	69.63262	15.50479	54.81058	142.7463	59.26573	66.5354	17.29259	24.0951	38.64329	38.03912	47.46674	36.18622	50.29156	80.05735	50.05734	86.94187
AC079610.	83.59896	116.0453	136.8813	68.59332	153.9404	87.9253	79.86994	113.9013	74.70641	67.24895	27.70936	115.9299	100.7523	124.7165	102.6906	70.90286	48.23968	164.7341	155.0613
AC079610.	21.13997	23.00898	10.99047	25.98232	12.18233	15.98642	24.64073	13.89041	31.51677	8.646293	21.68559	10.12086	13.3651	6.515042	19.56012	13.19123	22.58028	10.9216	17.92616
AC079612.	0	0	0	0	0	0	0	0	0	0	0	0	0	0	0	0	4.105505	0	0
AC079630.	268.0932	281.1097	418.637	302.4342	240.3242	207.8234	368.7612	284.2903	226.4538	244.9783	215.6511	307.3061	320.7623	266.186	359.9061	203.6396	225.8028	474.1795	338.8044
AC079684.	52.84991	41.01601	28.97488	51.96464	84.16885	42.24982	33.98721	39.81916	79.37557	61.48475	46.98544	38.64329	50.37613	30.71377	50.8563	65.95615	25.65941	44.59654	24.20031
AC079741.	0.960908	5.001952	19.98267	4.157171	13.28982	1.141887	3.398721	10.1863	0	10.56769	7.228529	7.360626	4.112337	2.792161	17.6041	0.824452	1.026376	2.7304	6.274156
AC079753.	0	2.000781	0	1.039293	0	0	1.69936	3.704108	1.167288	0	0	2.760235	0	0	0.978006	0	1.026376	2.7304	0
AC079760.	57.65445	42.0164	26.97661	56.12181	34.33203	51.38492	34.83689	42.59724	37.35321	38.42797	25.29985	43.24368	26.73019	57.70466	36.18622	54.41382	50.29244	19.1128	36.74863
AC079760.	24.9836	10.0039	43.96188	31.17878	3.322455	4.567548	25.49041	6.482189	8.171014	15.37119	19.27608	24.84211	14.39318	24.19873	27.38416	33.80253	26.68578	27.304	40.33386
AC079766.	4.804538	3.001171	10.99047	7.275049	21.04221	11.41887	0	10.1863	44.35693	18.25329	38.55216	3.680313	7.196591	4.653602	8.802052	12.36678	3.079129	5.4608	12.54831
AC079768.	0.960908	1.00039	0	0	4.42994	0	0.84968	0	0	0.960699	0	0	0	0	0	0	0	0	0
AC079776.	0	0	0	0	0	0	0.84968	0	9.338302	0	2.40951	0.920078	1.028084	0	0	0.824452	0	0.910133	0.896308
AC079776.	40.35812	28.01093	63.94455	34.29666	50.94431	28.54718	30.58849	13.89041	8.171014	4.803496	10.84279	45.08383	49.34805	36.29809	36.18622	32.97807	35.92317	57.33841	51.98586
AC079776.	1.921815	0	0	1.039293	0	0	0.84968	3.704108	0	0	0	0	0	0	0	0	0	0	0
AC079777.	29.78813	9.003514	30.97314	21.82515	6.64491	15.98642	25.49041	13.89041	21.01118	44.19217	28.91412	45.08383	58.60081	30.71377	17.6041	23.08465	18.47477	29.12427	13.44462
AC079779.	0.960908	6.002343	0	0	0	1.141887	0	0	0	0	9.638039	1.840156	0	0	0.978006	1.648904	6.158258	0	0
AC079779.	26.90541	37.01445	78.93156	40.53242	71.98652	15.98642	60.3273	114.8274	38.5205	39.38867	13.2523	70.84602	37.01104	47.46674	42.05425	42.04704	13.34289	30.0344	20.61508
AC079779.	0	1.00039	0.999134	0	3.322455	2.283774	0	5.556162	0	0	0	5.520469	1.028084	3.722881	0	9.893422	0	0	0
AC079780.	0	0	0	0	0	0	0	0	1.167288	0	0	0	0	0	0	0	0	1.820267	3.585232
AC079781.	14.41361	8.003124	9.991337	8.314342	19.93473	12.56076	4.248401	27.78081	3.501863	16.33189	15.66181	11.04094	20.56169	22.33729	18.58211	4.946711	1.026376	10.9216	10.7557
AC079781.	1.921815	5.001952	14.987	1.039293	3.322455	4.567548	1.69936	5.556162	21.01118	3.842797	0	2.760235	7.196591	5.584322	3.912023	6.595615	0	0	5.377848
AC079781.	10.56998	4.001562	7.993069	1.039293	4.42994	0	4.248401	6.482189	17.50932	7.685594	6.023774	10.12086	27.75828	3.722881	4.890029	3.297807	5.131881	0.910133	8.963079
AC079781.	100.8953	139.0543	230.7999	133.0295	190.4874	216.9585	98.56291	255.5835	315.1677	154.6726	230.1082	265.9026	262.1615	247.5716	232.7654	230.0221	168.3257	172.9253	150.5797
AC079789.	0	0	0	0	13.28982	0	0	0	0	0	0	0	0	0.93072	0	0	0	0	0
AC079790.	4.804538	0	0	0	4.42994	0	0.84968	0	7.003726	0	0	0	0	0	0	0.824452	0	0	0
AC079793.	8.648168	17.00664	25.97748	3.117878	19.93473	27.40529	12.7452	43.52327	51.36066	63.40615	44.57593	27.60235	35.98295	24.19873	19.56012	4.946711	2.052753	10.01147	17.92616
AC079795.	12.4918	9.003514	8.992203	12.47151	13.28982	9.135096	12.7452	7.408216	0	4.803496	0	13.80117	3.084253	11.16864	7.824047	0	2.052753	10.01147	2.688924
AC079799.	0	0	0.999134	1.039293	2.21497	0	0	0	0	0	0	0	1.028084	0	0	0	0	0	0
AC079804.	1.921815	0	0.999134	0	0	0	0	0	3.501863	0	0	0	0	2.792161	0	0	0	0	0
AC079804.	0.960908	5.001952	5.994802	5.196464	2.21497	5.709435	5.098081	3.704108	4.669151	1.921399	2.40951	2.760235	4.112337	2.792161	4.890029	6.595615	2.052753	5.4608	2.688924
AC079807.	0	0	0	1.039293	0	0	0.84968	0	0	0.960699	0	0	4.112337	5.584322	0	0	0	0	0

AC079807.	315.1777	317.1238	450.6093	173.5619	290.161	268.3434	296.5384	299.1067	257.9706	341.0482	487.9257	282.464	268.33	261.5324	277.7537	150.0502	252.4886	234.8144	279.6481
AC079834.	6.726353	9.003514	11.9896	2.078586	2.21497	5.709435	1.69936	0	2.334575	7.685594	30.11887	10.12086	3.084253	0.93072	6.846041	4.122259	8.21101	4.550667	2.688924
AC079848.	69.18534	97.03787	105.9082	70.67191	108.5335	106.1955	78.17058	69.45203	28.01491	78.77734	53.00921	80.96688	83.27483	72.59618	115.4047	107.1787	115.9805	61.88907	86.04556
AC079858.	0.960908	1.00039	0.999134	0	0	0	0	4.630135	0	0	0	8.280704	5.140422	0.93072	0	0	0	0	5.377848
AC079866.	1.921815	5.001952	11.9896	1.039293	6.64491	5.709435	6.797442	10.1863	0	1.921399	7.228529	10.12086	7.196591	5.584322	3.912023	15.66458	2.052753	1.820267	0.896308
AC079866.	0	2.000781	5.994802	0	0	1.141887	2.549041	11.11232	2.334575	0	0	2.760235	7.196591	0	3.912023	0	0	1.820267	3.585232
AC079880.	65.34171	45.01757	57.94975	83.14342	24.36467	53.66869	71.37314	43.52327	133.0708	97.03062	95.17564	41.40352	47.29188	57.70466	66.5044	63.48279	78.0046	51.8776	69.01571
AC079880.	16.33543	5.001952	55.95149	19.74656	44.2994	23.97963	30.58849	23.15068	24.51304	48.03496	18.07132	19.32164	32.8987	19.54513	34.2302	9.06897	12.31652	4.550667	16.13354
AC079882.	0	6.002343	0.999134	2.078586	0	3.425661	5.947762	0.926027	1.167288	3.842797	3.614265	0.920078	2.056169	0	3.912023	0.824452	1.026376	1.820267	0.896308
AC079905.	0	1.00039	0	0	4.42994	0	0	3.704108	7.003726	0	0	0.920078	0	0	2.934017	0	0	0	0
AC079905.	0.960908	0	0	0	4.42994	0	0	0	0	0	0	0.920078	0	0	0	0	0	2.7304	2.688924
AC079906.	24.02269	24.00937	15.98614	17.66798	27.68712	12.56076	6.797442	30.55889	54.86252	11.52839	21.68559	32.20274	32.8987	12.09936	36.18622	37.10033	19.50115	20.02294	1.792616
AC079907.	38.4363	50.01952	52.95408	35.33595	56.48173	31.97284	53.52986	39.81916	26.84762	35.54587	51.80446	75.44642	61.68506	28.85233	56.72434	32.97807	28.73854	53.69787	20.61508
AC079907.	1.921815	11.00429	6.993936	6.235757	2.21497	10.27698	7.647122	7.408216	5.836439	24.97818	21.68559	11.04094	9.252759	0.93072	9.780058	4.122259	7.184634	17.29253	13.44462
AC079915.	0	3.001171	1.998267	6.235757	12.18233	6.851322	1.69936	13.89041	32.68406	1.921399	27.70936	6.440548	6.168506	6.515042	2.934017	0	0	3.640534	4.48154
AC079917.	0.960908	4.001562	0	11.43222	0	9.135096	7.647122	1.852054	0	2.882098	1.204755	1.840156	3.084253	0.93072	0.978006	4.946711	2.052753	2.7304	0
AC079921.	64.3808	130.0508	95.91683	86.2613	156.1554	73.08077	110.4584	113.9013	10.50559	92.22713	51.80446	116.8499	115.1454	62.35826	101.7126	57.71163	52.34519	68.26001	61.84525
AC079921.	149.9016	99.03865	224.8051	177.7191	126.2533	82.21586	93.46483	162.0547	24.51304	94.14853	113.247	244.7408	200.4765	137.7466	128.1188	151.6991	108.7959	180.2064	179.2616
AC079922.	1.921815	3.001171	1.998267	5.196464	0	2.283774	0	2.778081	5.836439	0	3.614265	7.360626	0	2.792161	0	1.648904	0	1.820267	3.585232
AC079922.	604.4108	459.1792	895.2238	760.7623	634.5889	502.4303	363.6631	756.5641	789.0865	1003.931	1186.684	361.5907	485.2558	701.7631	653.3079	1127.85	1059.22	420.4816	455.3244
AC079942.	2.882723	0	0	0	0	1.141887	2.549041	0	0	0	0	0	3.084253	3.722881	0	0	0	3.640534	0.896308
AC079943.	0	0	0	0	0	0	0	0	0	0	0	0	0	0	0	0.824452	1.026376	0	0
AC079944.	0.960908	0	0	0	4.42994	5.709435	0	0	0	0.960699	0	0	1.028084	5.584322	0.978006	1.648904	3.079129	0	0.896308
AC079944.	41.31902	54.02108	43.96188	35.33595	34.33203	21.69585	45.03305	50.93149	0	50.91706	36.14265	36.80313	61.68506	54.9125	46.94428	70.90286	69.79359	33.67494	58.26002
AC079949.	37.47539	28.01093	21.98094	33.25737	11.07485	30.83095	31.43817	14.81643	16.34203	21.13538	36.14265	23.92203	21.58977	54.9125	21.51613	41.22259	31.81766	40.956	71.70464
AC079949.	671.6744	492.1921	620.462	444.8173	322.2781	552.6733	484.3177	401.8957	344.3499	485.1531	332.5123	327.5479	392.7282	564.9472	626.9017	309.9939	373.601	468.7187	470.5617
AC079950.	25.9445	35.01367	26.97661	22.86444	17.71976	51.38492	62.87634	33.33697	46.69151	49.95636	16.86657	32.20274	38.03912	35.36737	33.2522	44.5204	17.4484	38.2256	46.60801
AC079987.	0.960908	2.000781	1.998267	1.039293	1.107485	1.141887	0	0	0	0	4.81902	4.600391	0	0	0	0	1.026376	0	0
AC080013.	763.9215	729.2846	621.4611	651.6366	328.923	628.0379	615.1685	454.6793	412.0526	687.8607	539.7302	562.1678	554.1375	954.919	963.3357	977.7999	922.7123	736.2979	683.883
AC080013.	6.726353	8.003124	5.994802	8.314342	12.18233	10.27698	2.549041	7.408216	11.67288	5.764196	20.48083	9.200782	4.112337	11.16864	18.58211	10.71787	15.39564	3.640534	5.377848
AC080013.	106.6607	173.0675	108.9056	99.77211	120.7159	121.04	78.17058	83.34243	197.2716	129.6944	162.6419	95.68814	121.314	101.4485	144.7449	145.1035	137.5344	88.28294	103.0754
AC080013.	210.4387	186.0726	206.8207	311.7878	233.6793	181.56	291.4403	331.5177	74.70641	387.1618	197.5798	222.6589	203.5607	312.722	341.324	535.0692	563.4806	276.6806	320.8782
AC080013.	9.609075	12.00469	21.98094	6.235757	12.18233	9.135096	20.39233	24.0767	51.36066	26.89958	24.0951	15.64133	13.3651	14.89152	15.64809	9.893422	17.4484	25.48374	13.44462
AC080013.	30.74904	26.01015	62.94542	24.94303	62.01916	25.12151	83.26866	67.59997	57.1971	57.64196	74.6948	61.64524	57.57272	59.5661	80.19648	112.1254	149.8509	50.05734	86.94187
AC080014.	0	0	1.998267	0	0	0	0	0	0	0.960699	0	0	0	0	0	0.824452	0	0	0
AC080023.	19.21815	7.002733	16.98527	17.66798	7.752394	0	8.496802	10.1863	2.334575	1.921399	2.40951	10.12086	2.056169	10.23792	10.75806	8.244518	5.131881	24.5736	17.92616
AC080023.	17.29634	14.00547	26.97661	9.353635	5.537425	18.27019	16.14392	20.37259	3.501863	20.17468	9.638039	13.80117	6.168506	3.722881	3.912023	46.1693	42.08143	50.05734	23.30401
AC080037.	0.960908	0	2.997401	2.078586	2.21497	5.709435	1.69936	0	0	7.685594	4.81902	0.920078	1.028084	3.722881	0	1.648904	4.105505	5.4608	1.792616
AC080038.	180.6506	156.0609	146.8726	119.5187	338.8904	59.37812	68.8241	193.5397	639.6737	319.9129	356.6074	196.8967	200.4765	107.0328	89.97654	98.93422	61.58258	21.8432	35.85232
AC080038.	3.84363	6.002343	9.991337	9.353635	11.07485	0	6.797442	8.334243	28.01491	3.842797	15.66181	10.12086	2.056169	6.515042	0.978006	0.824452	4.105505	0.910133	3.585232
AC080038.	53.81082	45.01757	70.93849	117.4401	84.16885	30.83095	61.17698	163.9068	240.4613	103.7555	149.3896	128.811	121.314	56.77394	50.8563	44.5204	106.7431	58.24854	50.19325
AC080075.	0	0	0	0	0	0	0	0	0.926027	0	0	0	0	1.028084	0.93072	0	3.297807	1.026376	0.910133	0.896308
AC080078.	0	1.00039	0.999134	0	0	0	0.84968	0	0	0	0	0	0	0.93072	2.934017	0	1.026376	0	0
AC080078.	20.17906	37.01445	19.98267	33.25737	34.33203	42.24982	43.33369	17.59451	33.85134	7.685594	32.52838	17.48149	18.50552	14.89152	24.45015	22.2602	25.65941	9.101334	17.92616
AC080078.	3.84363	15.00586	13.98787	31.17878	3.322455	9.135096	14.44456	4.630135	16.34203	7.685594	14.45706	1.840156	3.084253	4.653602	13.69208	1.648904	6.158258	2.7304	9.859387
AC080080.	124.918	245.0957	340.7046	138.2259	225.9269	158.7223	213.2697	251.8794	186.766	257.4674	220.4701	241.9806	202.5326	212.2042	244.5015	178.0816	119.0596	234.8144	241.1068
AC080100.	1356.801	1444.564	1890.361	1505.935	1493.997	1132.752	1089.29	1448.306	596.484	1379.564	1287.883	1115.135	1290.246	1268.572	1378.988	1304.283	1071.537	1139.487	1106.044
AC080112.	45.16265	58.02265	50.95582	40.53242	49.83682	46.81737	71.37314	18.52054	53.69524	53.79916	69.87578	56.12477	46.2638	39.09025	44.01026	46.1693	55.42432	50.05734	50.19325
AC080112.	232.5396	201.0785	231.799	263.9804	205.9922	216.9585	251.5054	200.0218	298.8257	260.3495	262.6366	279.7038	338.2398	236.403	266.9956	303.3983	379.7592	306.715	468.7691
AC080112.	248.8751	291.1136	210.8172	298.277	251.3991	312.877	354.3167	186.1314	287.1528	341.0482	268.6603	276.9435	254.9649	254.0866	262.1056	394.088	456.7374	409.56	350.4564
AC080125.	1.921815	0	0.999134	0	0	6.851322	1.69936	5.556162	0	0	6.023774	0	2.056169	0	5.868035	0.824452	0	0.910133	1.792616
AC080128.	3.84363	2.000781	6.993936	10.39293	24.36467	6.851322	11.89552	1.852054	22.17847	0.960699	9.638039	16.56141	15.42127	10.23792	17.6041	4.946711	4.105505	5.4608	11.652
AC080128.	53.81082	99.03865	319.7228	107.0472	281.3012	110.763	17.84329	137.052	39.68778	195.9826	102.4042	91.08774	82.24675	59.5661	70.41642	113.7744	119.0596	42.77627	91.42341
AC080129.	0	0	0	0	2.21497	4.567548	4.248401	0	0	0	0	0	0	0	0	0	0	0	0
AC080129.	27.86632	20.00781	49.95668	18.70727	38.76197	22.83774	28.88913	24.0767	54.86252	30.74238	75.89956	31.28266	20.56169	27.92161	16.6261	11.54233	28.73854	45.50667	35.85232
AC080162.	165.2761	145.0566	154.8657	159.0118	205.9922	113.0468	176.7335	144.4602	101.554	170.0438	137.3421	237.3802	172.7182	120.0629	137.8988	143.4546	121.1124	132.8795	139.824
AC080188.	0	0	0	0	1.107485	0	0	0.926027	4.669151	0	0	0	1.028084	0	0	0	1.026376	0.910133	0
AC080188.	7.68726	6.002343	3.996535	0	4.42994	4.567548	16.14392	5.556162	8.171014	17.29259	9.638039	7.360626	13.3651	19.54513	7.824047	4.946711	1.026376	7.281067	11.652
AC080188.	10.56998	10.0039	24.97834	1.039293	18.82724	11.41887	26.34009	17.59451	7.003726	21.13538	26.50461	11.96102	12.33701	18.61441	13.69208	5.771163	19.50115	2.7304	6.274156
AC082651.	29.78813	5.001952	0	12.47151	1.107485	3.425661	1.69936	16.66849	5.836439	0.960699	0	6.440548	3.084253	9.307203	8.802052	6.595615	10.26376	6.370934	6.274156
AC082651.	0	0	0	0	0	0	0	0	0	0	0	0	3.084253	0	0	0	0	0	0
AC082651.	123.9571	132.0515	175.8475	134.0688	109.641	173.5668	125.7527	153.7205	395.7105	215.1966	330.1028	111.3295	142.9037	151.7074	152.5689	75.02512	126.2443	112.8565	107.557

AC082651.	0	0	1.998267	0	0	0	0	0	0	0	0	0	1.028084	0	0	0	1.026376	0	0.896308
AC083795.	1.921815	5.001952	0.999134	5.196464	9.967364	3.425661	1.69936	5.556162	7.003726	0.960699	2.40951	1.840156	3.084253	7.445762	4.890029	7.420066	6.158258	3.640534	6.274156
AC083795.	0	0	0	2.078586	0	0	0.84968	0	0	0	0	0	0	0	0.978006	0.824452	0	0	0
AC083798.	4.804538	12.00469	13.98787	12.47151	7.752394	25.12151	7.647122	32.41095	0	11.52839	12.04755	23.92203	23.64594	14.89152	19.56012	4.122259	11.29014	15.47227	27.78555
AC083798.	367.0667	328.1281	492.5729	277.4912	305.6658	215.8166	454.5789	370.4108	157.5838	229.6071	192.7608	456.3588	357.7734	358.3273	370.6642	311.6428	285.3326	434.1136	306.5373
AC083799.	709.1498	340.1327	385.6656	828.3163	386.5122	437.3427	333.9243	289.8465	625.6662	354.498	297.5745	457.2789	463.6661	467.2216	493.8929	825.2763	636.3533	702.623	338.8044
AC083801.	4.804538	4.001562	1.998267	1.039293	5.537425	1.141887	2.549041	3.704108	14.00745	0	0	7.360626	10.28084	0	1.956012	3.297807	0	0	12.54831
AC083805.	88.40349	125.0488	306.734	215.1336	167.2302	98.20228	186.9297	115.7534	144.7437	131.6158	221.6749	228.1794	225.1505	136.8159	165.283	117.8966	114.9541	362.2331	419.4721
AC083805.	9.609075	3.001171	4.995668	9.353635	14.3973	3.425661	8.496802	1.852054	14.00745	3.842797	0	7.360626	21.58977	2.792161	12.71408	14.84013	6.158258	15.47227	1.792616
AC083805.	0	0	0.999134	0	0	0	0	0	0	0	0	0	0	0	0	0	0	4.550667	0
AC083806.	2.882723	0	0	0	0	1.141887	0.84968	0	0	0	0	0	0	0	0	0	6.158258	0.910133	2.688924
AC083806.	13.45271	16.00625	24.97834	60.27898	14.3973	14.84453	39.93497	47.22738	4.669151	22.09608	20.48083	38.64329	42.15146	25.12945	42.05425	50.29156	50.29244	10.01147	32.26709
AC083806.	1.921815	0	4.995668	0	4.42994	3.425661	2.549041	0.926027	0	7.685594	10.84279	0	1.028084	7.445762	1.956012	1.648904	2.052753	0	0.896308
AC083809.	11.53089	24.00937	7.993069	4.157171	32.11706	22.83774	13.59488	15.74246	72.37184	22.09608	12.04755	13.80117	10.28084	13.9608	13.69208	23.9091	22.58028	9.101334	10.7557
AC083822.	8.648168	1.00039	0	2.078586	3.322455	0	0.84968	0.926027	0	4.803496	0	0	1.028084	4.653602	0.978006	2.473355	0	0.910133	1.792616
AC083829.	34.59267	31.0121	34.96968	32.21808	46.51437	42.24982	60.3273	28.70684	60.69896	34.58517	21.68559	46.00391	69.90974	33.50593	55.74633	19.78684	25.65941	41.86614	34.0597
AC083836.	0	0	0	0	0	7.993209	0	0	0	0	0	0	1.028084	0	2.934017	0.824452	0	0	0
AC083837.	50.9281	11.00429	30.97314	39.49313	16.61227	18.27019	44.18337	11.11232	2.334575	23.05678	36.14265	21.1618	38.03912	25.12945	40.09824	37.92478	56.45069	70.99041	83.35664
AC083841.	84.55986	62.02421	73.93589	96.65423	40.97694	119.8981	100.2623	94.45476	17.50932	80.69874	24.0951	91.08774	95.61185	99.58707	74.32844	145.928	88.26836	56.42827	69.01571
AC083841.	0.960908	0	0	0	1.107485	0	0.84968	0	0	0	0	1.840156	1.028084	0	2.934017	0	0	0.910133	0.896308
AC083841.	0	0	0	0	1.107485	0	0	0	0	0	0	0	0	0	0	0	0	0.910133	0
AC083843.	36.51449	23.00898	56.95062	37.41454	27.68712	7.993209	19.54265	29.63287	12.84017	0.960699	0	11.96102	13.3651	17.68369	23.47214	5.771163	13.34289	31.85467	24.20031
AC083843.	1598.95	1909.745	3124.291	1523.603	2392.167	2439.071	2854.076	2623.435	1546.656	2579.477	2113.14	2548.617	2364.594	2388.228	2712.01	1242.449	1164.937	2285.345	2042.686
AC083843.	411.2684	747.2917	694.3979	469.7603	687.7481	823.3005	635.5608	897.3202	782.0828	725.3279	724.0577	499.6025	417.4023	594.7303	599.5176	580.4141	698.9622	306.715	239.3142
AC083855.	0	0	0	0	0	0	0	0	9.338302	0	0	5.520469	0	0	0	0	1.026376	0	0
AC083862.	5.765445	7.002733	17.98441	5.196464	14.3973	4.567548	3.398721	8.334243	26.84762	3.842797	25.29985	4.600391	5.140422	0	4.890029	1.648904	10.26376	10.9216	1.792616
AC083862.	52.84991	35.01367	63.94455	57.1611	46.51437	47.95925	49.28145	50.93149	74.70641	58.60265	92.76613	93.84798	30.84253	55.84322	42.05425	82.44518	87.24198	82.82214	33.16339
AC083863.	2.882723	0	0.999134	0	0	3.425661	0.84968	0	0	1.921399	2.40951	4.600391	1.028084	0	0.978006	5.771163	4.105505	0.910133	3.585232
AC083864.	0	0	0	1.039293	0	1.141887	0.84968	0	7.003726	0	0	0.920078	0	0.93072	0	0	0	0	0.896308
AC083864.	3.84363	4.001562	0	6.235757	4.42994	0	8.496802	0	0	0	0	1.840156	4.112337	0	0	13.19123	10.26376	5.4608	3.585232
AC083864.	0	0	0	0	0	0	0	0	0	0	0	0	0	0	1.956012	0	0	0	0
AC083865.	5.765445	2.000781	4.995668	0	0	1.141887	0	2.778081	3.501863	8.646293	0	4.600391	1.028084	4.653602	1.956012	1.648904	4.105505	0.910133	5.377848
AC083867.	0	2.000781	0.999134	0	0	4.567548	0	0.926027	3.501863	0	2.40951	0.920078	3.084253	1.861441	3.912023	3.297807	1.026376	4.550667	0.896308
AC083873.	15.37452	13.00508	15.98614	15.58939	15.50479	13.70264	16.9936	11.11232	2.334575	10.56769	4.81902	7.360626	7.196591	13.9608	5.868035	14.01568	20.52753	13.652	13.44462
AC083875.	0	0	0	0	0	0	3.398721	0	0	0.960699	0	0	0	3.722881	0	0	0	0	0
AC083880.	5.765445	20.00781	5.994802	5.196464	4.42994	18.27019	10.19616	6.482189	18.6766	35.54587	21.68559	12.8811	3.084253	4.653602	5.868035	19.78684	9.237386	0.910133	8.963079
AC083899.	462.1965	561.219	497.5686	398.0491	507.2281	493.2952	344.1205	462.0875	458.7441	475.5461	465.0354	444.3978	395.8125	520.2726	463.5748	398.2102	412.6033	413.2006	448.154
AC083899.	0	0	0	0	0	0	0	0	7.003726	0	0	0	0	0	0	0	0	0	0
AC083899.	0	0	0	0	0	0	0	0	0	0	0	0	0	0	0	0	1.026376	4.550667	0.896308
AC083900.	6.726353	16.00625	39.96535	35.33595	26.57964	9.135096	35.68657	30.55889	9.338302	9.606993	2.40951	13.80117	8.224675	17.68369	31.29619	45.34485	17.4484	7.281067	8.066772
AC083902.	1.921815	3.001171	0.999134	1.039293	8.859879	1.141887	0.84968	0	2.334575	0.960699	3.614265	0	0	0.93072	1.956012	0	0	0	5.377848
AC083902.	18.25724	16.00625	8.992203	5.196464	15.50479	14.84453	2.549041	0	1.167288	5.764196	0	10.12086	1.028084	12.09936	25.42815	2.473355	10.26376	7.281067	0.896308
AC083904.	0.960908	2.000781	0.999134	3.117878	1.107485	0	0	1.852054	1.167288	0	1.204755	0	1.028084	0	0	0	2.052753	0	0.896308
AC083906.	0	1.00039	6.993936	0	1.107485	0	0.84968	0	1.167288	0	0	1.840156	1.028084	0.93072	3.912023	0	0	0	0
AC083906.	0.960908	0	1.998267	1.039293	3.322455	1.141887	5.098081	4.630135	1.167288	1.921399	1.204755	0	0	0.93072	0	4.946711	0	2.7304	2.688924
AC083906.	0	0	0	0	0	0	0.84968	0	0	0	0	0.920078	0	0	0.978006	0	0	0	0.896308
AC083923.	15.37452	6.002343	25.97748	17.66798	7.752394	9.135096	5.098081	10.1863	14.00745	0.960699	0	4.600391	18.50552	17.68369	21.51613	12.36678	16.42202	12.74187	0.896308
AC083949.	5.765445	2.000781	0	30.13949	0	18.27019	5.947762	7.408216	9.338302	9.606993	0	5.520469	14.39318	7.445762	0	6.595615	6.158258	8.191201	5.377848
AC083964.	95.12985	106.0414	113.9012	50.92535	76.41646	111.9049	95.16419	98.15887	198.4389	159.4761	121.6802	62.56532	72.99399	103.31	96.82258	96.46086	87.24198	55.51814	86.04556
AC083964.	58.61536	51.01991	44.96101	17.66798	57.58922	27.40529	59.47762	40.74519	51.36066	27.86028	40.96167	67.16571	38.03912	38.15953	26.40616	27.20691	30.79129	42.77627	21.51139
AC083967.	0	0	0	0	0	9.135096	0	0	1.167288	2.882098	0	0	2.056169	0	1.956012	0	0	0	0
AC083973.	55.73264	16.00625	13.98787	43.6503	16.61227	1.141887	15.29424	31.48492	52.52795	9.606993	4.81902	12.8811	11.30893	22.33729	19.56012	37.10033	45.16056	66.43974	16.13354
AC083982.	16.33543	1.00039	28.97488	1.039293	11.07485	22.83774	29.73881	17.59451	2.334575	1.921399	0	3.680313	1.028084	4.653602	2.934017	15.66458	14.36927	2.7304	0
AC084017.	0	0	0	0	1.107485	0	0	0	0	0	4.81902	0	1.028084	0	0	0	0	0	0
AC084018.	807.1623	705.2753	1169.986	499.8998	544.8826	815.3073	732.4244	721.3751	891.8078	896.3324	1168.612	885.1153	802.9339	1041.476	1144.267	634.0035	690.7512	609.7894	760.0691
AC084018.	47.08447	57.02225	65.94282	41.57171	35.43952	17.12831	68.8241	35.18903	28.01491	53.79916	49.39495	53.36454	46.2638	43.74385	69.43841	51.11601	46.18693	45.50667	55.57109
AC084024.	1.921815	4.001562	3.996535	5.196464	3.322455	0	3.398721	0	1.167288	1.921399	0	0	0	0	0	2.473355	4.105505	3.640534	5.377848
AC084024.	1.921815	6.002343	5.994802	8.314342	0	1.141887	0.84968	9.26027	0	0	0	3.680313	1.028084	0.93072	0.978006	2.473355	3.079129	4.550667	1.792616
AC084024.	0	0	2.997401	1.039293	1.107485	1.141887	1.69936	0	3.501863	0	0	3.680313	0	0.93072	0.978006	0.824452	1.026376	0	0
AC084026.	0.960908	6.002343	1.998267	6.235757	2.21497	2.283774	1.69936	2.778081	0	0.960699	2.40951	9.200782	2.056169	0	3.912023	6.595615	8.21101	3.640534	1.792616
AC084026.	24.9836	12.00469	29.97401	40.53242	39.86946	5.709435	5.098081	10.1863	54.86252	26.89958	13.2523	23.92203	23.64594	30.71377	22.49413	14.01568	46.18693	23.66347	32.26709
AC084026.	0	0	0	0	0	0	0	3.704108	0	0	0	0	1.028084	0	0	0	0	0	0

AC084032.	0	0	0	2.078586	0	0	0	0.926027	0	0	1.204755	2.760235	1.028084	0	0	0	0	3.640534	1.792616
AC084033.	8.648168	7.002733	9.991337	13.51081	12.18233	11.41887	8.496802	8.334243	4.669151	12.48909	16.86657	5.520469	19.5336	8.376483	7.824047	1.648904	3.079129	9.101334	2.688924
AC084033.	0	2.000781	0	0	0	1.141887	0	0	1.167288	0	0	0	1.028084	0	0	0	0	0	0
AC084033.	1337.583	2048.8	1560.647	1502.817	1423.118	1732.243	1487.79	1466.827	2521.342	1928.123	1674.609	1174.02	1186.409	1653.89	1701.73	1535.954	1725.338	1344.267	1146.378
AC084035.	45.16265	40.01562	34.96968	16.62868	37.65449	66.22945	15.29424	27.78081	10.50559	61.48475	63.85201	7.360626	27.75828	29.78305	28.36217	14.01568	16.42202	25.48374	21.51139
AC084036.	233.5005	351.137	359.6881	233.8409	385.4048	340.2823	281.2442	205.578	804.2612	490.9173	584.3061	230.9396	250.8526	328.5443	307.0938	157.4703	216.5654	252.107	207.9434
AC084048.	0	3.001171	0	1.039293	0	0	0	0	0	0	0	0.920078	1.028084	3.722881	0.978006	6.595615	1.026376	0	6.274156
AC084064.	122.0353	18.00703	9.991337	24.94303	14.3973	13.70264	10.19616	10.1863	21.01118	26.89958	4.81902	19.32164	20.56169	22.33729	17.6041	24.73355	13.34289	16.3824	14.34093
AC084082.	1.921815	2.000781	6.993936	4.157171	6.64491	1.141887	7.647122	0	4.669151	0	0	2.760235	9.252759	0.93072	4.890029	0.824452	8.21101	2.7304	12.54831
AC084083.	3.84363	1.00039	9.991337	10.39293	2.21497	5.709435	15.29424	2.778081	1.167288	4.803496	27.70936	11.96102	13.3651	11.16864	0.978006	4.946711	3.079129	2.7304	9.859387
AC084116.	0	5.001952	0	0	0	0	5.947762	10.1863	0	1.921399	0	0.920078	2.056169	1.861441	0.978006	0	0	1.820267	4.48154
AC084116.	0	0	0	0	0	0	0	0	0	0	0	1.840156	0	0	0	0	0	0	0
AC084117.	48.04538	22.00859	124.8917	12.47151	76.41646	51.38492	50.98081	100.0109	32.68406	62.44545	59.03299	48.76415	76.07824	113.5479	82.15249	29.68027	47.21331	91.92348	70.80833
AC084121.	0	0	0.999134	2.078586	1.107485	0	5.098081	0	0	3.842797	0	1.840156	4.112337	0	1.956012	0	0	0	1.792616
AC084121.	0	2.000781	0	0	0	3.425661	0	0	3.501863	4.803496	12.04755	0.920078	0	0	0.978006	0.824452	2.052753	0.910133	0
AC084121.	0	0	0	0	0	0	0	0	0	0	1.204755	0	0	0.93072	0.978006	0.824452	0	0	0
AC084125.	112.4262	136.0531	127.8891	82.10413	151.7254	172.4249	93.46483	104.6411	259.1379	197.904	363.836	268.6628	222.0662	174.0447	159.4149	78.32292	105.7168	188.3976	186.4321
AC084125.	2.882723	0	0	14.5501	0	1.141887	1.69936	0	3.501863	3.842797	1.204755	3.680313	3.084253	0	3.912023	4.122259	1.026376	2.7304	6.274156
AC084149.	0	0	0.999134	0	0	0	0.84968	0	0	0	0	0	0	0	0	0	0	0	0.896308
AC084149.	0	0	0.999134	0	0	0	0	0	0	0	0	0	0	0	0	0	1.026376	0	0
AC084198.	0	1.00039	0	0	0	1.141887	1.69936	0	0	2.882098	0	0	0	0	0	0	0	0.910133	0.896308
AC084200.	1.921815	3.001171	0	1.039293	0	0	0	5.556162	4.669151	1.921399	0	7.360626	0	3.722881	0	0	5.131881	0	0
AC084211.	0	0	8.992203	0	5.537425	0	0	5.556162	0	0.960699	4.81902	0	0	6.515042	0	0	0	0	0
AC084251.	2.882723	1.00039	10.99047	0	0	0	0	0.926027	0	7.685594	4.81902	0.920078	1.028084	8.376483	0	0.824452	1.026376	0	5.377848
AC084262.	0	0	5.994802	0	1.107485	0	0.84968	5.556162	0	0	10.84279	3.680313	0	0	3.912023	0.824452	2.052753	0.910133	3.585232
AC084290.	0	8.003124	0.999134	3.117878	1.107485	0	0	3.704108	0	0	2.40951	0	0	5.584322	11.73607	0	0	1.820267	1.792616
AC084291.	0	0	0	0	0	0	0.84968	0	0	0	0	0	0	0	0	0	1.026376	0	0
AC084337.	350.7312	325.1269	424.6318	377.2633	519.4104	210.1072	563.338	353.7423	224.1192	309.3452	316.8505	489.4816	538.7162	316.4449	380.4443	292.6804	356.1526	328.5582	345.9749
AC084346.	7.68726	0	3.996535	0	0	1.141887	0	0	0	0	4.81902	0.920078	0	0.93072	0	0	0	0.910133	0
AC084346.	7.68726	7.002733	15.98614	2.078586	0	4.567548	4.248401	4.630135	21.01118	9.606993	15.66181	3.680313	9.252759	5.584322	3.912023	14.01568	7.184634	2.7304	4.48154
AC084357.	5.765445	5.001952	12.98874	13.51081	14.3973	3.425661	5.098081	5.556162	11.67288	7.685594	8.433284	9.200782	14.39318	4.653602	6.846041	14.84013	14.36927	11.83173	17.02985
AC084357.	0	0	0	4.157171	6.64491	0	0	0	1.167288	0	0	0	0	3.722881	1.956012	3.297807	0	0.910133	0
AC084361.	0	0	0	0	3.322455	0	1.69936	0	0	0	0	0	0	0	0	0	0	0	0
AC084368.	1.921815	0	0	0	0	0	0	0.926027	0	0	0	0	0	0	0	0	1.026376	0	1.792616
AC084373.	0	0	0	0	2.21497	0	0	0	0	0	0	0	0	0	0	0	0	0	0
AC084398.	0.960908	0	4.995668	0	0	0	0.84968	0	0	0.960699	0	0	0	0	0	1.648904	0	0	0.896308
AC084398.	2.882723	2.000781	10.99047	4.157171	6.64491	2.283774	4.248401	9.26027	10.50559	0.960699	4.81902	2.760235	1.028084	2.792161	0.978006	6.595615	4.105505	7.281067	8.066772
AC084706.	1.921815	2.000781	1.998267	5.196464	5.537425	3.425661	0	9.26027	11.67288	0	1.204755	4.600391	0	0	3.912023	3.297807	4.105505	0	0
AC084754.	11.53089	4.001562	4.995668	4.157171	3.322455	10.27698	12.7452	2.778081	3.501863	1.921399	1.204755	6.440548	10.28084	7.445762	11.73607	9.893422	6.158258	0	3.585232
AC084757.	9.609075	12.00469	5.994802	11.43222	11.07485	1.141887	16.14392	12.03835	5.836439	0.960699	2.40951	2.760235	9.252759	12.09936	7.824047	1.648904	10.26376	11.83173	7.170464
AC084757.	2.882723	1.00039	0.999134	6.235757	8.859879	2.283774	0	1.852054	5.836439	2.882098	3.614265	0.920078	2.056169	1.861441	9.780058	0	4.105505	6.370934	0
AC084757.	64.3808	31.0121	92.91943	65.47544	52.05179	54.81058	39.93497	43.52327	92.21573	59.56335	27.70936	57.04485	38.03912	65.15042	46.94428	56.06272	34.89679	73.72081	60.05263
AC084757.	8.648168	9.003514	13.98787	5.196464	37.65449	15.98642	9.346483	12.96438	1.167288	13.44979	7.228529	4.600391	2.056169	5.584322	5.868035	4.946711	5.131881	11.83173	7.170464
AC084759.	0	0	0	0	0	1.141887	0	0	0	0	0	0	0	0	0	0.824452	0	0	0.896308
AC084759.	0	1.00039	0	0	0	0	0	0	0	0	0	0	0	0	0	0	1.026376	0	0
AC084768.	0	0	0	0	0	0	3.398721	0.926027	2.334575	0	0	0	0	0	0	0.824452	0	0	0
AC084781.	5.765445	5.001952	11.9896	1.039293	0	1.141887	5.098081	5.556162	30.34948	1.921399	12.04755	0	2.056169	0.93072	0.978006	0.824452	1.026376	9.101334	1.792616
AC084781.	0	0	0	0	0	0	0.84968	0	3.501863	0	0	0	0	0	0	0	0	0	0
AC084782.	0	0	1.998267	3.117878	0	0	2.549041	0	0	1.921399	0	1.840156	0	0	0	0.824452	0	0.910133	5.377848
AC084782.	49.96719	40.01562	52.95408	33.25737	31.00958	34.25661	63.72602	61.11778	39.68778	31.70308	9.638039	69.92595	78.13441	41.88241	44.98827	35.45143	19.50115	48.23707	51.08955
AC084782.	15.37452	45.01757	32.97141	10.39293	39.86946	20.55397	31.43817	39.81916	28.01491	27.86028	14.45706	29.4425	22.61786	29.78305	22.49413	12.36678	19.50115	18.20267	12.54831
AC084783.	7.68726	0	3.996535	0	5.537425	7.993209	3.398721	0.926027	0	0	9.638039	0.920078	0	0	5.868035	2.473355	1.026376	1.820267	0.896308
AC084809.	10.56998	7.002733	13.98787	18.70727	4.42994	17.12831	24.64073	8.334243	5.836439	7.685594	13.2523	15.64133	15.42127	30.71377	16.6261	22.2602	9.237386	7.281067	12.54831
AC084809.	12.4918	10.0039	7.993069	8.314342	7.752394	12.56076	5.098081	0	10.50559	2.882098	12.04755	7.360626	10.28084	18.61441	15.64809	5.771163	5.131881	9.101334	0.896308
AC084816.	0.960908	2.000781	1.998267	1.039293	2.21497	7.993209	1.69936	0	4.669151	0.960699	0	3.680313	7.196591	20.47585	1.956012	0	0	10.9216	4.48154
AC084819.	0.960908	0	0	0	0	0	0	0	0	0.960699	0	0	0	0	1.956012	0	5.131881	5.4608	0
AC084824.	558.2873	442.1726	716.3788	404.2849	648.9862	468.1737	481.7687	545.4299	817.1014	728.21	681.8913	591.6103	678.5357	491.4203	467.4868	295.1538	335.625	417.7512	346.8712
AC084824.	5.765445	8.003124	4.995668	2.078586	14.3973	7.993209	4.248401	8.334243	15.17474	8.646293	14.45706	9.200782	16.44935	6.515042	6.846041	5.771163	1.026376	8.191201	5.377848
AC084824.	307.4904	251.098	356.6907	272.2947	354.3952	259.2084	344.1205	314.8492	179.7623	340.0875	278.2984	310.9864	380.3912	303.4148	284.5997	226.7243	231.961	285.7819	258.1367
AC084824.	171.0415	189.0738	161.8597	243.1945	153.9404	155.2966	296.5384	238.915	52.52795	185.415	92.76613	221.7389	238.5156	192.6591	232.7654	197.044	182.695	202.0496	162.2317
AC084824.	172.9634	136.0531	211.8163	59.23969	271.3338	167.8574	127.452	148.1643	448.2385	236.332	293.9602	210.6979	239.5437	147.0538	146.7009	83.26963	101.6112	129.2389	91.42341
AC084838.	1.921815	0	0	0	0	0	0	0	0	0	0	0	0	1.028084	0.93072	0	0	0	0

AC084855.	8.648168	20.00781	6.993936	16.62868	17.71976	14.84453	16.14392	24.0767	9.338302	54.75986	61.4425	23.92203	24.67402	38.15953	12.71408	3.297807	4.105505	3.640534	6.274156
AC084855.	57.65445	49.01913	21.98094	18.70727	23.25718	22.83774	29.73881	23.15068	11.67288	51.87776	71.08054	39.56336	70.93782	23.26801	35.20821	26.38246	22.58028	20.02294	25.99293
AC084859.	9.609075	16.00625	18.98354	22.86444	18.82724	3.425661	19.54265	12.96438	5.836439	23.05678	10.84279	34.04289	23.64594	14.89152	17.6041	11.54233	2.052753	10.01147	3.585232
AC084864.	13.45271	8.003124	2.997401	11.43222	1.107485	13.70264	8.496802	2.778081	31.51677	20.17468	14.45706	8.280704	2.056169	10.23792	5.868035	8.244518	6.158258	10.9216	8.963079
AC084866.	6.726353	15.00586	21.98094	10.39293	11.07485	1.141887	11.89552	11.11232	4.669151	24.01748	6.023774	10.12086	26.73019	14.89152	5.868035	4.946711	3.079129	1.820267	13.44462
AC084866.	0	0	0	3.117878	0	0	6.797442	8.334243	0	0	7.228529	0	0	0	0	0.824452	2.052753	0	0
AC084871.	42.27993	45.01757	34.96968	37.41454	40.97694	22.83774	40.78465	29.63287	178.595	33.62447	9.638039	54.28462	77.10633	37.22881	43.03226	51.11601	33.87042	33.67494	27.78555
AC084871.	0	0	0	0	0	0	0	0	0	0	0	0.920078	0	0	0	0	0	3.640534	0
AC084876.	22.10087	30.01171	50.95582	22.86444	32.11706	44.53359	29.73881	33.33697	32.68406	52.83846	48.1902	27.60235	23.64594	42.81313	42.05425	17.31349	16.42202	18.20267	25.09662
AC084876.	46.12356	51.01991	48.95755	45.72888	54.26676	54.81058	60.3273	100.0109	36.18592	24.97818	21.68559	41.40352	35.98295	53.05106	49.8783	45.34485	50.29244	31.85467	58.26002
AC084879.	1.921815	4.001562	0	3.117878	6.64491	4.567548	6.797442	13.89041	1.167288	0	1.204755	4.600391	14.39318	0	0.978006	14.84013	9.237386	4.550667	2.688924
AC084879.	0	0	0	0	0	0	0	0	0	0	0	0	0	1.861441	0	0	0	0	0
AC084880.	189.2988	422.1648	248.7843	481.1926	224.8194	307.1676	455.4286	261.1396	305.8294	235.3713	225.2892	293.505	356.7453	346.228	306.1158	314.9406	435.1835	214.7915	430.2278
AC084880.	0	0	0	3.117878	0	0	2.549041	0	2.334575	0	0	0	0	0	0.978006	0	0	0	0
AC084880.	0	7.002733	10.99047	5.196464	8.859879	2.283774	11.89552	0.926027	2.334575	9.606993	15.66181	10.12086	13.3651	5.584322	1.956012	19.78684	4.105505	8.191201	1.792616
AC084880.	0.960908	4.001562	0.999134	1.039293	15.50479	0	5.947762	0	4.669151	0	0	0	9.252759	6.515042	3.912023	4.122259	0	5.4608	12.54831
AC084882.	4.804538	2.000781	0	0	0	0	0.84968	0	0	0.960699	0	0.920078	1.028084	0	0.978006	0.824452	0	0.910133	0.896308
AC087045.	1.921815	1.00039	7.993069	6.235757	9.967364	3.425661	1.69936	11.11232	25.68033	15.37119	21.68559	7.360626	1.028084	5.584322	0.978006	10.71787	3.079129	6.370934	7.170464
AC087045.	1.921815	4.001562	49.95668	0	8.859879	10.27698	5.098081	3.704108	0	1.921399	10.84279	11.96102	15.42127	10.23792	15.64809	5.771163	1.026376	22.75334	23.30401
AC087071.	20.17906	9.003514	10.99047	13.51081	4.42994	20.55397	21.24201	4.630135	0	2.882098	4.81902	14.72125	13.3651	18.61441	14.67009	9.06897	18.47477	37.31547	14.34093
AC087071.	12.4918	21.0082	14.987	23.90373	25.47215	4.567548	25.49041	19.44657	5.836439	16.33189	13.2523	12.8811	14.39318	14.89152	18.58211	23.9091	12.31652	19.1128	12.54831
AC087072.	0	0	0.999134	0	0	0	0.84968	0.926027	0	0	0	0	1.028084	3.722881	1.956012	2.473355	1.026376	0	0
AC087072.	1.921815	4.001562	15.98614	1.039293	0	0	5.098081	0	18.6766	12.48909	7.228529	4.600391	4.112337	3.722881	4.890029	3.297807	4.105505	1.820267	0.896308
AC087163.	0	0	1.998267	0	3.322455	0	0	0	0	0	0	0	0	0	0	0	0	0	0
AC087163.	122.0353	140.0547	140.8778	170.444	89.70628	107.3374	171.6354	116.6794	0	76.85594	75.89956	152.733	137.7633	77.24979	98.77859	157.4703	136.508	80.09174	140.7203
AC087163.	49.96719	31.0121	45.96015	21.82515	57.58922	19.41208	31.43817	32.41095	26.84762	53.79916	62.64725	67.16571	63.74123	60.49682	42.05425	32.15362	21.5539	35.4952	43.02278
AC087164.	49.96719	70.02733	78.93156	74.82908	59.80419	55.95246	67.97442	88.8986	128.4017	54.75986	49.39495	80.04681	80.19058	67.01186	54.76833	38.74924	68.76721	74.63094	80.66772
AC087164.	0	4.001562	0.999134	2.078586	0	2.283774	7.647122	0	12.84017	0	4.81902	0.920078	1.028084	0	0	2.473355	5.131881	3.640534	0
AC087190.	331.5131	416.1624	241.7903	155.8939	254.7215	285.4718	263.4009	248.1752	107.3905	186.3757	131.3183	313.7467	203.5607	372.2881	318.8299	333.903	246.3303	330.3784	150.5797
AC087190.	0	0	0	0	0	0	1.69936	0	0	0	0	0.920078	0	0	0.978006	0	0	0	0
AC087203.	0	2.000781	0	5.196464	1.107485	0	2.549041	0	2.334575	0	0	0.920078	0	0	0	4.122259	1.026376	5.4608	2.688924
AC087203.	0.960908	0	3.996535	6.235757	0	0	11.04584	4.630135	3.501863	4.803496	0	10.12086	0	0.93072	6.846041	4.946711	5.131881	9.101334	8.963079
AC087203.	37.47539	73.0285	72.93676	50.92535	107.426	55.95246	51.83049	131.4958	207.7772	84.54153	151.7991	85.56728	93.55568	88.41843	122.2507	46.1693	63.63533	58.24854	53.77848
AC087222.	117.2307	77.03006	106.9073	84.18271	66.4491	44.53359	129.1514	94.45476	28.01491	99.91272	50.5997	97.52829	100.7523	89.34915	112.4707	56.88718	46.18693	62.79921	90.5271
AC087235.	0	0	0	0	0	0	0	1.852054	0	0	0	0	0	0	0	0	0	0	0
AC087239.	18.25724	16.00625	37.96708	0	36.547	14.84453	9.346483	13.89041	116.7288	39.38867	139.7516	22.08188	16.44935	15.82225	18.58211	7.420066	1.026376	10.9216	15.23724
AC087241.	3.84363	0	0	0	0	1.141887	0.84968	0	1.167288	0.960699	4.81902	0	0	0	0.978006	4.946711	0	1.820267	5.377848
AC087241.	0	0	0	1.039293	4.42994	1.141887	0.84968	0.926027	0	7.685594	0	0.920078	0	0.93072	0.978006	0.824452	3.079129	3.640534	0.896308
AC087241.	10.56998	2.000781	6.993936	2.078586	2.21497	1.141887	9.346483	14.81643	11.67288	1.921399	1.204755	4.600391	5.140422	9.307203	0.978006	4.122259	6.158258	0.910133	4.48154
AC087242.	0	1.00039	0	3.117878	0	0	0	1.852054	0	0	0	0	0	0	0.978006	0	1.026376	0	3.585232
AC087257.	20.17906	11.00429	27.97574	10.39293	1.107485	3.425661	11.89552	5.556162	5.836439	0	20.48083	7.360626	3.084253	0.93072	6.846041	10.71787	12.31652	16.3824	5.377848
AC087257.	46.12356	28.01093	40.96448	20.78586	27.68712	13.70264	41.63433	8.334243	24.51304	55.72056	48.1902	21.1618	28.78636	21.40657	22.49413	4.122259	5.131881	20.93307	9.859387
AC087258.	0.960908	1.00039	0	5.196464	0	0	0	0	7.003726	0	1.204755	0	1.028084	0.93072	0	0.824452	1.026376	0.910133	0
AC087260.	0	2.000781	3.996535	0	0	4.567548	0	0	0	2.882098	0	0.920078	0	0	4.890029	0	0	0	0
AC087269.	3.84363	0	0.999134	4.157171	0	7.993209	0	0	0	0	0	10.12086	0	1.861441	0	9.893422	3.079129	2.7304	1.792616
AC087269.	0	0	0	0	0	0	0	0	0	0	0	1.840156	0	0	0	0	0	0	0
AC087273.	0	0	0	0	0	0	0	0	0	0	0	0	0	0	0	0	2.052753	0	0
AC087273.	282.5068	139.0543	169.8527	277.4912	214.8521	208.9653	79.02026	182.4273	235.7921	234.4106	184.3275	207.0176	151.1284	152.6381	157.4589	14.01568	7.184634	276.6806	121.8979
AC087276.	21.13997	19.00742	22.98007	15.58939	11.07485	18.27019	22.94137	31.48492	21.01118	12.48909	19.27608	46.92399	25.70211	36.29809	45.96627	10.71787	25.65941	8.191201	11.652
AC087276.	33.63176	22.00859	44.96101	56.12181	52.05179	46.81737	91.76547	46.30135	35.01863	43.23147	26.50461	56.12477	67.85357	49.32818	45.96627	54.41382	30.79129	44.59654	56.4674
AC087276.	16.33543	17.00664	20.98181	39.49313	24.36467	5.709435	42.48401	12.96438	11.67288	14.41049	19.27608	12.8811	18.50552	34.43665	9.780058	11.54233	12.31652	18.20267	19.71877
AC087276.	0	0	4.995668	0	9.967364	0	0.84968	3.704108	0	1.921399	0	0.920078	2.056169	0	0.978006	0.824452	1.026376	0	0.896308
AC087277.	0	0	0	1.039293	0	0	0	1.852054	0	0	0	3.680313	0	1.861441	0	6.595615	6.158258	1.820267	0
AC087277.	0	1.00039	3.996535	1.039293	0	3.425661	0	0	0	0	0	1.840156	2.056169	1.861441	0	2.473355	0	0.910133	1.792616
AC087280.	0	0	0	0	8.859879	0	0.84968	0	0	0	0	0	0	0	0.978006	0	0	0	0.896308
AC087280.	0.960908	0	1.998267	11.43222	0	11.41887	8.496802	7.408216	2.334575	5.764196	8.433284	0.920078	5.140422	8.376483	7.824047	18.96239	21.5539	1.820267	2.688924
AC087283.	0	0	0	0	4.42994	0	0	0	0	0	0	0	0	1.861441	0	0	0	0	0
AC087284.	6.726353	0	4.995668	8.314342	21.04221	7.993209	3.398721	11.11232	24.51304	19.21399	8.433284	5.520469	7.196591	8.376483	10.75806	10.71787	4.105505	14.56213	0.896308
AC087286.	0.960908	5.001952	2.997401	2.078586	0	2.283774	1.69936	7.408216	9.338302	4.803496	0	0.920078	0	1.861441	2.934017	0	7.184634	1.820267	5.377848
AC087286.	90.32531	118.0461	139.8787	173.5619	102.9961	93.63473	230.2633	94.45476	16.34203	16.33189	7.228529	172.9747	135.7071	134.9544	123.2287	145.1035	99.5585	117.4072	125.4831
AC087286.	19.21815	30.01171	13.98787	14.5501	4.42994	5.709435	6.797442	8.334243	16.34203	0.960699	7.228529	5.520469	20.56169	12.09936	25.42815	12.36678	3.079129	9.101334	13.44462
AC087289.	40.35812	56.02186	38.96621	32.21808	40.97694	31.97284	39.93497	25.00273	9.338302	53.79916	78.30907	43.24368	49.34805	50.2589	57.70234	32.97807	20.52753	50.05734	41.23017

AC087289.	56.69354	78.03045	58.94889	25.98232	131.7907	49.10114	33.98721	62.96984	226.4538	140.2621	361.4265	69.00587	40.09529	80.97267	57.70234	31.32917	64.6617	84.64241	79.77141
AC087289.	22.10087	25.00976	16.98527	6.235757	6.64491	35.3985	14.44456	47.22738	14.00745	13.44979	20.48083	14.72125	8.224675	40.02097	33.2522	12.36678	11.29014	23.66347	19.71877
AC087289.	87.44259	98.03826	90.92116	51.96464	118.5009	116.4725	62.87634	77.78627	158.7511	125.8516	262.6366	99.36845	123.3701	92.14131	80.19648	90.6897	88.26836	180.2064	122.7942
AC087294.	83.59896	141.0551	111.903	77.94696	94.13622	49.10114	76.47122	119.4575	40.85507	85.50223	75.89956	107.6492	88.41526	96.79491	117.3607	74.20066	72.87271	86.46267	103.0754
AC087301.	44.20175	41.01601	61.94629	61.31827	40.97694	35.3985	57.77826	60.19176	18.6766	35.54587	37.3474	74.52634	55.51656	40.95169	44.98827	58.53608	55.42432	51.8776	45.71171
AC087311.	0	5.001952	3.996535	1.039293	6.64491	0	0	0	0	0	0	0	0	0	1.956012	3.297807	1.026376	0.910133	0
AC087311.	0	0	0	0	0	1.141887	0	0	0	0	0	0.920078	0	0	0	0	0	0	0.896308
AC087318.	0.960908	0	0	0	0	0	0	0	0	0	0	0	1.028084	0	0	0	0	0	0
AC087343.	5.765445	16.00625	6.993936	3.117878	4.42994	5.709435	2.549041	15.74246	18.6766	20.17468	13.2523	5.520469	7.196591	12.09936	11.73607	16.48904	6.158258	12.74187	3.585232
AC087354.	4.804538	0	0	1.039293	0	0	1.69936	0	0	0	1.204755	0.920078	2.056169	1.861441	1.956012	3.297807	3.079129	0.910133	2.688924
AC087359.	12.4918	7.002733	1.998267	3.117878	23.25718	5.709435	11.04584	15.74246	26.84762	8.646293	12.04755	11.04094	1.028084	3.722881	22.49413	3.297807	4.105505	12.74187	6.274156
AC087362.	8.648168	2.000781	11.9896	15.58939	17.71976	1.141887	4.248401	0	9.338302	0.960699	9.638039	3.680313	8.224675	4.653602	0.978006	1.648904	4.105505	0.910133	4.48154
AC087362.	26.90541	22.00859	37.96708	20.78586	31.00958	15.98642	28.03945	27.78081	1.167288	34.58517	19.27608	30.36258	25.70211	26.99089	25.42815	23.08465	21.5539	20.02294	8.963079
AC087369.	0	0	3.996535	0	0	0	0	0	0	0	0	0.920078	0	0	0	0	0	0	0
AC087373.	0	0	0	0	0	0	0.84968	0	0	0	0	0	0	0	0	0	0	0	0.896308
AC087379.	15.37452	44.01718	20.98181	37.41454	22.1497	12.56076	55.22922	26.85478	11.67288	28.82098	34.93789	29.4425	30.84253	2.792161	28.36217	25.55801	19.50115	10.01147	5.377848
AC087379.	10.56998	17.00664	11.9896	41.57171	15.50479	12.56076	34.83689	14.81643	45.52422	31.70308	32.52838	11.96102	13.3651	12.09936	9.780058	18.13794	15.39564	5.4608	8.066772
AC087380.	16.33543	2.000781	21.98094	2.078586	9.967364	19.41208	26.34009	6.482189	18.6766	0.960699	4.81902	18.40156	15.42127	7.445762	8.802052	0	0	10.01147	9.859387
AC087385.	41.31902	11.00429	16.98527	16.62868	11.07485	13.70264	37.38593	31.48492	9.338302	6.724895	2.40951	31.28266	25.70211	36.29809	13.69208	37.92478	30.79129	25.48374	27.78555
AC087385.	0	4.001562	9.991337	2.078586	3.322455	1.141887	6.797442	3.704108	1.167288	0	4.81902	1.840156	0	0.93072	0	0	0	0.910133	0
AC087385.	0	0	5.994802	0	0	0	0	0	0	0	0	0	0	0	0	0	0	0	0
AC087386.	0	0	4.995668	0	4.42994	0	1.69936	1.852054	0	0	0	2.760235	1.028084	0	0	0	1.026376	0	0
AC087388.	115.3089	101.0394	128.8882	47.80747	160.5853	146.1615	96.86355	148.1643	233.4575	177.7294	242.1557	138.0117	144.9599	118.2015	168.217	85.74299	96.47937	158.3632	139.824
AC087392.	2.882723	2.000781	4.995668	7.275049	1.107485	4.567548	1.69936	5.556162	2.334575	0	1.204755	7.360626	5.140422	8.376483	5.868035	10.71787	17.4484	8.191201	0
AC087392.	19.21815	19.00742	8.992203	23.90373	15.50479	50.24303	27.18977	17.59451	11.67288	2.882098	22.89034	17.48149	10.28084	30.71377	28.36217	24.73355	15.39564	32.7648	19.71877
AC087392.	65.34171	62.02421	47.95842	98.73281	52.05179	70.79699	134.2495	43.52327	59.53167	56.68126	53.00921	59.80509	45.23571	53.05106	58.68035	83.26963	37.97592	87.37281	79.77141
AC087392.	16.33543	23.00898	18.98354	0	13.28982	17.12831	14.44456	9.26027	7.003726	7.685594	0	3.680313	6.168506	5.584322	10.75806	12.36678	18.47477	8.191201	1.792616
AC087392.	9.609075	5.001952	3.996535	4.157171	6.64491	6.851322	8.496802	13.89041	52.52795	35.54587	24.0951	16.56141	7.196591	24.19873	3.912023	18.13794	6.158258	10.9216	9.859387
AC087393.	0	3.001171	0	0	0	0	0.84968	0	0	0	0	0	0	0	0	0	0	0	0
AC087430.	4.804538	0	3.996535	0	1.107485	0	4.248401	0	4.669151	0	4.81902	2.760235	1.028084	3.722881	0	0	0	0	0
AC087439.	0	0	0	2.078586	0	0	0.84968	0	0	0	0	1.840156	0	0	0	0	0	0	0
AC087439.	0.960908	0	2.997401	0	0	13.70264	0	0	0	5.764196	0	0	0	0	0	1.648904	2.052753	3.640534	0.896308
AC087441.	0	0	0	0	0	1.141887	0.84968	0	0	0	0	0	1.028084	0	0.978006	0	0	0	0
AC087442.	1.921815	0	0.999134	0	1.107485	1.141887	5.947762	0.926027	2.334575	0.960699	2.40951	3.680313	1.028084	1.861441	7.824047	2.473355	3.079129	4.550667	0
AC087457.	19.21815	19.00742	41.96361	36.37525	2.21497	19.41208	33.13753	31.48492	16.34203	19.21399	10.84279	17.48149	19.5336	19.54513	17.6041	51.94047	64.6617	9.101334	36.74863
AC087463.	1.921815	10.0039	0	2.078586	0	0	5.947762	0	10.50559	13.44979	0	1.840156	0	0	0.978006	3.297807	1.026376	1.820267	0.896308
AC087463.	0	0	0	5.196464	0	0	0	0	0	1.921399	0	0	6.168506	0	0	0.824452	2.052753	0	0
AC087463.	24.9836	7.002733	4.995668	14.5501	2.21497	11.41887	5.947762	7.408216	2.334575	11.52839	8.433284	7.360626	6.168506	6.515042	1.956012	4.946711	6.158258	3.640534	9.859387
AC087465.	0	0	0	0	3.322455	1.141887	0	0	0	0	0	0	0	0	0	0.824452	0	0.910133	0.896308
AC087468.	0	1.00039	0	4.157171	0	1.141887	0	6.482189	3.501863	5.764196	4.81902	0.920078	1.028084	3.722881	3.912023	0	0	0	0.896308
AC087473.	0	4.001562	0	0	0	1.141887	6.797442	3.704108	0	0	0	0	1.028084	0.93072	4.890029	9.06897	0	0.910133	0.896308
AC087477.	0	5.001952	12.98874	3.117878	6.64491	0	2.549041	3.704108	11.67288	0	1.204755	1.840156	8.224675	0	0.978006	0.824452	2.052753	0.910133	0.896308
AC087477.	2998.992	2433.95	2935.455	1445.656	2279.204	1758.506	3186.301	2429.895	3096.814	2846.552	2982.973	2728.952	3163.416	2148.102	2506.629	1345.505	1464.639	1832.099	1815.92
AC087477.	0	0	0	0	2.21497	2.283774	0	0	0	1.921399	0	10.12086	0	0.93072	0	0	1.026376	0	0
AC087477.	8.648168	0	10.99047	4.157171	6.64491	0	7.647122	0.926027	11.67288	3.842797	8.433284	11.04094	4.112337	0.93072	3.912023	0	0	2.7304	0.896308
AC087477.	80.71623	64.02499	101.9116	44.68959	63.12664	102.7698	98.56291	100.0109	84.04472	87.42363	50.5997	115.0098	118.2297	57.70466	79.21847	54.41382	53.37157	68.26001	53.77848
AC087477.	154.7061	132.0515	176.8467	127.833	131.7907	62.80379	266.7996	175.0191	33.85134	92.22713	21.68559	272.3432	224.1224	156.361	145.7229	125.3167	88.26836	144.7112	120.1053
AC087481.	16.33543	8.003124	2.997401	5.196464	1.107485	0	16.14392	3.704108	4.669151	2.882098	16.86657	7.360626	5.140422	23.26801	7.824047	14.01568	5.131881	11.83173	19.71877
AC087481.	10.56998	4.001562	0.999134	15.58939	27.68712	1.141887	9.346483	3.704108	1.167288	2.882098	4.81902	14.72125	4.112337	9.307203	7.824047	23.08465	10.26376	0	6.274156
AC087481.	2269.664	2394.935	3047.358	2385.177	3122	2725.684	2542.243	2442.859	2848.182	3177.993	3987.739	3187.151	2877.608	2485.954	2515.431	2885.581	2950.832	2570.217	3091.366
AC087482.	10.56998	19.00742	10.99047	12.47151	7.752394	10.27698	7.647122	0.926027	44.35693	8.646293	9.638039	16.56141	14.39318	19.54513	8.802052	14.01568	6.158258	14.56213	14.34093
AC087491.	10.56998	5.001952	2.997401	13.51081	12.18233	20.55397	16.14392	4.630135	16.34203	1.921399	4.81902	3.680313	4.112337	8.376483	0	0.824452	13.34289	4.550667	7.170464
AC087498.	0	2.000781	4.995668	0	0	0	1.69936	0	0	6.724895	0	2.760235	0	0	0	0.824452	8.21101	0	4.48154
AC087498.	0.960908	0	1.998267	1.039293	0	0	0.84968	0	3.501863	0.960699	7.228529	1.840156	1.028084	5.584322	0	1.648904	4.105505	0	0
AC087499.	376.6758	459.1792	314.7271	393.892	284.6236	423.6401	406.9968	286.1424	88.71387	206.5503	195.1703	465.5596	440.0201	452.3301	385.3343	490.5488	538.8475	366.7838	715.2537
AC087499.	7.68726	0	2.997401	0	0	1.141887	8.496802	0	5.836439	0.960699	1.204755	11.96102	4.112337	3.722881	0.978006	2.473355	0	1.820267	5.377848
AC087499.	5.765445	4.001562	1.998267	5.196464	0	18.27019	1.69936	0.926027	9.338302	1.921399	9.638039	4.600391	3.084253	0	0	1.648904	2.052753	1.820267	8.963079
AC087499.	0	0	1.998267	1.039293	0	4.567548	0	0	0	0	0	0	0	3.722881	0.978006	4.122259	11.29014	2.7304	14.34093
AC087499.	0	3.001171	4.995668	8.314342	3.322455	15.98642	9.346483	6.482189	0	10.56769	7.228529	7.360626	4.112337	12.09936	16.6261	14.84013	8.21101	6.370934	8.066772
AC087499.	0	0	0	0	0	0	0	0	0	0	0	0	0	0.93072	0	0	1.026376	0	0
AC087500.	442.9784	497.194	529.5408	292.0413	483.9709	430.4914	458.8273	542.6518	456.4095	415.0221	515.6351	502.3627	492.4524	528.6491	481.1789	327.3074	316.1239	389.5371	356.7306
AC087500.	142.2143	94.0367	77.93243	63.39686	64.23413	69.65511	85.8177	98.15887	89.88116	113.3625	103.6089	87.40743	100.7523	82.83411	72.37243	57.71163	48.23968	89.19307	61.84525

AC087501.	12.4918	0	3.996535	0	0	19.41208	0	28.70684	17.50932	44.19217	28.91412	7.360626	1.028084	3.722881	1.956012	6.595615	0	38.2256	23.30401
AC087501.	0	0	0	0	0	0	0	0.926027	0	0	0	0	0	0	0	0	0	0.910133	0.896308
AC087501.	1.921815	0	0	0	0	0	0.84968	0	0	1.921399	14.45706	0.920078	1.028084	0.93072	0	0	0	4.550667	2.688924
AC087501.	0	0	0	0	0	1.141887	0.84968	1.852054	0	0	0	1.840156	1.028084	0	0	0	0	0	3.585232
AC087501.	204.6733	258.1007	215.8129	279.5698	318.9557	227.2355	263.4009	409.3039	201.9408	233.4499	160.2324	222.6589	235.4313	256.8788	288.5117	259.7023	239.1457	245.736	186.4321
AC087516.	0	0	0	0	0	0	0	0	0	0	0	0	0	0	0.978006	0	1.026376	0.910133	0
AC087516.	0.960908	0	0	0	0	0	3.398721	3.704108	0	0	0	0	1.028084	2.792161	0	2.473355	1.026376	0.910133	3.585232
AC087521.	94.16894	38.01484	34.96968	31.17878	16.61227	9.135096	36.53625	34.263	124.8998	2.882098	32.52838	36.80313	30.84253	33.50593	31.29619	32.15362	31.81766	25.48374	28.68185
AC087521.	38.4363	48.01874	65.94282	136.1474	118.5009	36.54038	144.4456	48.15341	15.17474	45.15286	25.29985	71.7661	64.76932	32.57521	44.01026	49.46711	52.34519	70.99041	146.9945
AC087521.	73.02897	52.0203	61.94629	55.08252	32.11706	95.91851	39.93497	41.67122	77.04099	96.06993	89.15186	47.84407	45.23571	39.09025	33.2522	78.32292	41.05505	30.0344	33.16339
AC087525.	13.45271	7.002733	20.98181	16.62868	5.537425	15.98642	11.04584	14.81643	0	3.842797	4.81902	4.600391	9.252759	7.445762	0.978006	38.74924	16.42202	10.9216	18.82247
AC087565.	0	0	1.998267	0	0	1.141887	5.098081	1.852054	0	5.764196	0	1.840156	0	0.93072	0.978006	3.297807	0	4.550667	0
AC087565.	0	4.001562	1.998267	0	0	1.141887	5.947762	0	0	0	12.04755	3.680313	6.168506	0	0	0	0	0	3.585232
AC087565.	24.02269	49.01913	32.97141	39.49313	42.08443	19.41208	21.24201	17.59451	42.02236	28.82098	59.03299	26.68227	30.84253	53.98178	44.98827	26.38246	16.42202	21.8432	16.13354
AC087575.	0	0	0	0	0	0	3.398721	0	2.334575	0	2.40951	1.840156	0	3.722881	0	2.473355	1.026376	0.910133	0.896308
AC087588.	0	0	0	0	0	0	0	0	0	0	0	0	1.028084	0.93072	0	0	0	0	0
AC087588.	44.20175	9.003514	55.95149	10.39293	47.62185	47.95925	16.9936	67.59997	66.5354	30.74238	44.57593	23.00196	28.78636	41.88241	64.54838	23.9091	16.42202	36.40534	25.99293
AC087612.	3.84363	14.00547	0.999134	6.235757	0	2.283774	0.84968	0	11.67288	0	1.204755	3.680313	2.056169	0	3.912023	1.648904	0	1.820267	8.963079
AC087620.	3.84363	2.000781	2.997401	10.39293	0	0	7.647122	6.482189	0	0	7.228529	6.440548	1.028084	8.376483	3.912023	4.946711	8.21101	9.101334	7.170464
AC087623.	45.16265	65.02538	102.9108	122.6365	67.55658	78.7902	73.0725	60.19176	107.3905	66.28825	54.21397	40.48344	50.37613	29.78305	54.76833	108.8276	103.664	34.58507	47.50432
AC087623.	59.57627	72.02811	106.9073	62.35757	90.81376	67.37133	45.03305	87.97257	162.253	163.3189	189.1465	108.5692	163.4654	94.00275	57.70234	55.23827	67.74083	100.1147	86.94187
AC087623.	42.27993	32.01249	20.98181	12.47151	13.28982	21.69585	16.14392	5.556162	137.74	32.66377	18.07132	33.12282	29.81445	39.09025	32.27419	47.81821	59.52982	34.58507	48.40063
AC087623.	12.4918	16.00625	29.97401	39.49313	38.76197	17.12831	23.79105	17.59451	16.34203	25.93888	27.70936	19.32164	20.56169	30.71377	16.6261	28.03136	18.47477	10.01147	16.13354
AC087627.	24.02269	28.01093	16.98527	92.49706	37.65449	35.3985	22.94137	27.78081	0	16.33189	15.66181	57.96493	46.2638	21.40657	28.36217	61.00944	49.26606	27.304	73.49725
AC087632.	0	6.002343	0.999134	3.117878	0	7.993209	5.098081	0	8.171014	1.921399	2.40951	5.520469	2.056169	3.722881	8.802052	0	2.052753	0	6.274156
AC087633.	4.804538	12.00469	2.997401	16.62868	9.967364	9.135096	4.248401	13.89041	14.00745	11.52839	9.638039	13.80117	3.084253	6.515042	25.42815	1.648904	4.105505	0.910133	8.066772
AC087636.	0	1.00039	0.999134	0	1.107485	0	0.84968	0.926027	0	0	0	0.920078	1.028084	0	0	0.824452	0	0	0
AC087639.	13.45271	5.001952	9.991337	11.43222	2.21497	12.56076	5.098081	5.556162	15.17474	2.882098	4.81902	11.04094	2.056169	1.861441	6.846041	3.297807	4.105505	3.640534	4.48154
AC087641.	0	0	0	2.078586	0	0	5.098081	0.926027	0	0	0	0.920078	1.028084	0	0	0	1.026376	0.910133	3.585232
AC087645.	0	10.0039	6.993936	0	0	1.141887	0	0.926027	9.338302	0	0	0.920078	1.028084	0	0.978006	0	6.158258	0.910133	3.585232
AC087645.	44.20175	4.001562	20.98181	33.25737	24.36467	37.68227	11.89552	18.52054	17.50932	8.646293	22.89034	57.96493	29.81445	23.26801	20.53812	60.18498	64.6617	136.52	96.80126
AC087645.	0	1.00039	0	0	0	0	0	0	0	0	0	0	0	0	0	0	0	3.640534	3.585232
AC087645.	0.960908	2.000781	0	1.039293	5.537425	1.141887	0	0	2.334575	0.960699	0	0	0	0	0	2.473355	1.026376	2.7304	1.792616
AC087650.	0	0	0.999134	0	0	0	0	0	1.167288	0	1.204755	0	0	0.93072	0	0	0	0.910133	0
AC087667.	25.9445	34.01327	18.98354	57.1611	45.40688	23.97963	56.92858	44.4493	25.68033	27.86028	10.84279	27.60235	31.87062	17.68369	14.67009	46.1693	49.26606	10.9216	27.78555
AC087672.	0	0	0	1.039293	0	0	0	0	1.167288	0	0	0	0	0	0	0.824452	4.105505	0	0
AC087672.	0.960908	1.00039	0	2.078586	0	0	0	2.778081	0	0	0	0.920078	0	0	1.956012	0	5.131881	10.01147	0.896308
AC087672.	24.9836	25.00976	15.98614	39.49313	26.57964	18.27019	8.496802	12.96438	64.20083	19.21399	24.0951	34.04289	29.81445	36.29809	46.94428	63.48279	46.18693	41.86614	45.71171
AC087683.	14.41361	20.00781	15.98614	11.43222	15.50479	19.41208	5.098081	15.74246	9.338302	15.37119	2.40951	16.56141	21.58977	8.376483	37.16422	6.595615	13.34289	11.83173	24.20031
AC087683.	13.45271	26.01015	45.96015	44.68959	31.00958	25.12151	35.68657	29.63287	21.01118	21.13538	9.638039	21.1618	21.58977	30.71377	33.2522	13.19123	4.105505	15.47227	16.13354
AC087685.	0	0	0	0	0	0	0.84968	0	3.501863	0	0	0	0	3.722881	0	0	0	0	0
AC087699.	5.765445	1.00039	13.98787	0	1.107485	5.709435	10.19616	0	18.6766	2.882098	0	2.760235	2.056169	0.93072	10.75806	5.771163	5.131881	1.820267	4.48154
AC087721.	2.882723	0	0.999134	4.157171	1.107485	2.283774	5.947762	3.704108	0	3.842797	1.204755	6.440548	0	3.722881	0.978006	9.06897	5.131881	13.652	8.066772
AC087741.	643.808	624.2436	747.352	365.8311	936.9322	1264.069	307.5842	737.1175	1628.366	1466.027	2045.674	1353.435	1538.014	802.2809	744.2624	1150.11	1332.236	657.1163	667.7494
AC087741.	22.10087	32.01249	39.96535	12.47151	29.90209	15.98642	16.9936	41.67122	15.17474	27.86028	46.98544	30.36258	35.98295	49.32818	50.8563	19.78684	39.0023	30.0344	18.82247
AC087741.	28.82723	76.02967	66.94196	7.275049	31.00958	89.06719	44.18337	65.74792	29.18219	99.91272	113.247	97.52829	49.34805	75.38834	69.43841	65.95615	99.5585	84.64241	109.3496
AC087742.	35.55358	26.01015	35.96881	37.41454	43.19191	17.12831	38.23561	20.37259	24.51304	22.09608	9.638039	23.00196	43.17954	13.9608	12.71408	9.06897	26.68578	35.4952	12.54831
AC087742.	31.70995	61.02382	70.93849	18.70727	38.76197	29.68906	47.58209	52.78354	113.2269	66.28825	119.2707	39.56336	47.29188	77.24979	55.74633	20.6113	31.81766	61.88907	45.71171
AC087749.	19.21815	4.001562	6.993936	3.117878	12.18233	1.141887	8.496802	7.408216	1.167288	3.842797	0	10.12086	22.61786	18.61441	22.49413	18.13794	7.184634	10.01147	8.066772
AC087749.	3.84363	0	5.994802	5.196464	0	0	0.84968	1.852054	23.34575	0.960699	0	2.760235	7.196591	0	3.912023	1.648904	4.105505	0	0.896308
AC087752.	0	1.00039	0	0	3.322455	0	4.248401	0	0	1.921399	0	0.920078	0	0	0	0	5.131881	7.281067	0.896308
AC087752.	0	2.000781	4.995668	12.47151	2.21497	4.567548	11.89552	12.96438	0	0	0	14.72125	22.61786	1.861441	6.846041	0.824452	0	3.640534	0.896308
AC087752.	30.74904	36.01406	65.94282	31.17878	96.35119	31.97284	49.28145	57.41368	84.04472	46.11356	36.14265	92.9279	75.05016	66.08114	78.24047	50.29156	40.02867	89.19307	55.57109
AC087752.	61.49808	53.02069	59.94802	73.78979	83.06137	47.95925	50.13113	29.63287	54.86252	51.87776	42.16642	66.24563	62.71315	67.01186	45.96627	66.7806	50.29244	109.216	69.91202
AC087761.	0	12.00469	12.98874	9.353635	2.21497	7.993209	1.69936	2.778081	3.501863	7.685594	0	3.680313	10.28084	4.653602	17.6041	17.31349	6.158258	5.4608	4.48154
AC087762.	0.960908	2.000781	8.992203	1.039293	4.42994	1.141887	8.496802	2.778081	0	0	0	8.280704	7.196591	0	4.890029	0.824452	1.026376	2.7304	1.792616
AC087762.	0	0	0	8.314342	17.71976	0	0.84968	5.556162	4.669151	0	0	12.04755	0	0.93072	1.956012	4.946711	1.026376	6.370934	3.585232
AC087763.	5.765445	0	0	8.314342	2.21497	10.27698	6.797442	3.704108	0	0	4.81902	3.680313	7.196591	4.653602	2.934017	7.420066	3.079129	7.281067	3.585232
AC087783.	0	0	0	0	4.42994	0	0	0	0	0	0	0	0	0	0	0.824452	0	3.640534	0
AC087783.	0	0	0.999134	1.039293	1.107485	0	0	0.926027	0	0	0	0.920078	4.112337	0	0	0.824452	0	3.640534	0
AC087808.	0.960908	0	0	0	0	0	0	14.81643	0	0	0	0	0	0	0	0	0	0	0
AC087821.	0.960908	2.000781	0	0	0	0	0	0	0	0.960699	0	0	0	0	0	0.824452	0	0.910133	0.896308

AC087854.	14.41361	14.00547	26.97661	1.039293	32.11706	9.135096	4.248401	28.70684	9.338302	55.72056	51.80446	13.80117	18.50552	5.584322	8.802052	14.01568	9.237386	16.3824	8.963079
AC087855.	6.726353	4.001562	7.993069	12.47151	1.107485	3.425661	6.797442	3.704108	1.167288	4.803496	0	7.360626	7.196591	9.307203	7.824047	11.54233	1.026376	0	3.585232
AC087857.	13.45271	0	1.998267	11.43222	18.82724	4.567548	0.84968	7.408216	0	6.724895	3.614265	1.840156	3.084253	2.792161	0.978006	1.648904	1.026376	0	1.792616
AC087878.	0.960908	9.003514	4.995668	2.078586	0	5.709435	3.398721	0	0	1.921399	4.81902	0.920078	0	1.861441	11.73607	0.824452	2.052753	12.74187	5.377848
AC087884.	5.765445	20.00781	15.98614	2.078586	12.18233	3.425661	9.346483	10.1863	0	8.646293	1.204755	15.64133	23.64594	0.93072	13.69208	3.297807	9.237386	3.640534	3.585232
AC087885.	6.726353	8.003124	0	1.039293	0	0	2.549041	5.556162	0	6.724895	2.40951	1.840156	0	0	0	0	2.052753	1.820267	3.585232
AC087893.	7.68726	5.001952	4.995668	14.5501	19.93473	20.55397	15.29424	9.26027	40.85507	9.606993	7.228529	14.72125	22.61786	14.89152	7.824047	5.771163	11.29014	1.820267	14.34093
AC087894.	0	0	2.997401	0	0	1.141887	0	2.778081	0	0	0	0.920078	0	0	0	0	0	0	0
AC087897.	0	0	0	9.353635	7.752394	0	0	2.778081	2.334575	7.685594	0	0	0	3.722881	1.956012	0	0	0	0.896308
AC089983.	0.960908	2.000781	0	0	1.107485	0	0.84968	0	0	7.685594	1.204755	6.440548	6.168506	0.93072	0	6.595615	1.026376	0	3.585232
AC089984.	11.53089	6.002343	0.999134	15.58939	11.07485	2.283774	0.84968	3.704108	2.334575	16.33189	13.2523	5.520469	0	7.445762	5.868035	9.06897	17.4484	8.191201	10.7557
AC089984.	0	0	0	4.157171	0	0	0	0	0	0	0	0	0	0	0	0.824452	0	0	0
AC089987.	25.9445	12.00469	37.96708	40.53242	14.3973	36.54038	17.84329	24.0767	25.68033	25.93888	13.2523	41.40352	40.09529	13.03008	17.6041	17.31349	22.58028	28.21414	34.95601
AC089987.	0	4.001562	0	0	0	0	0	0	0	0	0	0	0	0	0	0	0	0	0
AC089987.	4.804538	1.00039	3.996535	0	0	10.27698	5.947762	3.704108	0	5.764196	1.204755	3.680313	1.028084	3.722881	3.912023	2.473355	3.079129	1.820267	7.170464
AC089998.	27.86632	46.01796	15.98614	10.39293	8.859879	12.56076	11.89552	16.66849	8.171014	49.95636	65.05676	7.360626	14.39318	15.82225	14.67009	9.06897	17.4484	4.550667	21.51139
AC089998.	4.804538	0	0	1.039293	0	0	0.84968	1.852054	0	0	0	0.920078	0	0.93072	0	0	1.026376	0	2.688924
AC089998.	10.56998	18.00703	25.97748	14.5501	19.93473	2.283774	30.58849	37.04108	0	3.842797	4.81902	13.80117	8.224675	11.16864	22.49413	5.771163	9.237386	3.640534	20.61508
AC089998.	15.37452	28.01093	0.999134	6.235757	1.107485	9.135096	14.44456	17.59451	8.171014	6.724895	16.86657	4.600391	3.084253	12.09936	16.6261	4.946711	11.29014	10.9216	6.274156
AC089999.	14.41361	51.01991	76.93329	12.47151	37.65449	38.82416	20.39233	41.67122	57.1971	59.56335	21.68559	23.92203	22.61786	59.5661	36.18622	24.73355	19.50115	35.4952	19.71877
AC090001.	32.67086	15.00586	21.98094	21.82515	13.28982	5.709435	11.04584	1.852054	7.003726	15.37119	22.89034	13.80117	5.140422	39.09025	17.6041	14.84013	1.026376	12.74187	33.16339
AC090004.	151.8234	158.0617	196.8293	93.53635	218.1745	161.0061	118.9552	169.4629	140.0745	255.546	246.9747	184.9357	184.0271	149.846	125.1847	106.3543	157.0356	125.5984	123.6905
AC090013.	1.921815	7.002733	3.996535	6.235757	12.18233	2.283774	0	16.66849	0	1.921399	2.40951	4.600391	3.084253	2.792161	2.934017	5.771163	6.158258	7.281067	14.34093
AC090017.	1.921815	0	0.999134	4.157171	0	0	3.398721	0	0	0	4.81902	1.840156	0	11.16864	6.846041	15.66458	9.237386	0	0.896308
AC090018.	0	0	0.999134	5.196464	1.107485	0	0	0	0	0	0	0	0	2.792161	0.978006	0	0	0.910133	3.585232
AC090023.	0	0	0	0	0	0	2.283774	0	0	0	0	0	0	0.93072	0	0	0	0.910133	0.896308
AC090044.	52.84991	63.0246	167.8545	106.0079	138.4356	54.81058	127.452	125.9397	217.1155	220.9608	169.8704	134.3314	106.9208	77.24979	127.1408	82.44518	64.6617	75.54107	77.08248
AC090049.	133.5661	113.0441	66.94196	15.58939	96.35119	22.83774	124.0533	25.00273	12.84017	11.52839	1.204755	92.9279	82.24675	42.81313	50.8563	40.39814	60.5562	75.54107	75.28987
AC090049.	0.960908	0	0.999134	0	0	0	0.84968	3.704108	0	0	0	1.840156	1.028084	0.93072	0	3.297807	3.079129	0.910133	0
AC090058.	0	0	0	0	0	1.141887	2.549041	7.408216	0	0	0	3.680313	1.028084	0	0.978006	0.824452	0	0	0.896308
AC090061.	35.55358	26.01015	32.97141	27.02161	28.79461	63.94567	8.496802	14.81643	44.35693	26.89958	27.70936	27.60235	18.50552	18.61441	11.73607	21.43575	29.76491	14.56213	23.30401
AC090071.	0	0	0.999134	1.039293	1.107485	0	0.84968	0	0	0	0	3.680313	1.028084	0	0	0	0	1.820267	1.792616
AC090092.	30.74904	31.0121	40.96448	25.98232	36.547	35.3985	43.33369	13.89041	21.01118	21.13538	25.29985	28.52243	31.87062	34.43665	19.56012	18.96239	36.94955	16.3824	15.23724
AC090096.	0	0	0.999134	0	0	0	0	0.84968	0	0	0	0	0	0	0	0	0	0	0
AC090099.	0	0	0	0	0	0	0	0	0	0	0	0	0	0.93072	0	0	1.026376	0.910133	0
AC090099.	0.960908	0	0	1.039293	0	0	0	0	16.34203	0	0	0	0	0	0	4.122259	3.079129	0	0
AC090099.	0	1.00039	0	0	0	0	0	0	0	0	0	0	0	0.93072	0.978006	0.824452	0	0	0.896308
AC090099.	0.960908	1.00039	0.999134	34.29666	9.967364	17.12831	19.54265	13.89041	0	3.842797	1.204755	11.96102	6.168506	2.792161	1.956012	23.08465	12.31652	0.910133	2.688924
AC090103.	0.960908	1.00039	0	0	0	0	0	0	8.334243	2.334575	0	7.228529	0	1.861441	0.978006	0.824452	1.026376	0	0
AC090109.	0	0	0	0	0	0	1.69936	0	0	0	0	0	0	0	0	0	0	0	0
AC090114.	1.921815	2.000781	0.999134	2.078586	0	1.141887	4.248401	2.778081	4.669151	0	3.614265	2.760235	1.028084	13.03008	4.890029	4.122259	1.026376	5.4608	1.792616
AC090114.	2956.712	2407.94	2601.744	2098.332	2192.82	2571.53	2551.59	2116.898	1752.099	2409.434	2095.069	3070.301	2983.501	2812.637	2650.396	2024.854	2034.278	3049.857	2416.446
AC090114.	34.59267	25.00976	42.96275	61.31827	25.47215	22.83774	75.62154	48.15341	17.50932	35.54587	27.70936	78.20665	84.30292	32.57521	53.79032	66.7806	71.84634	60.97894	64.53417
AC090115.	37.47539	34.01327	28.97488	13.51081	38.76197	28.54718	33.13753	37.04108	42.02236	16.33189	22.89034	42.3236	44.20763	17.68369	35.20821	27.20691	34.89679	28.21414	40.33386
AC090115.	0	0	0	0	0	0	0	0	0	0	0	0.920078	0	0	0	0	0	0	3.585232
AC090115.	40.35812	34.01327	38.96621	9.353635	17.71976	50.24303	8.496802	19.44657	33.85134	12.48909	71.08054	25.76219	25.70211	18.61441	27.38416	10.71787	33.87042	23.66347	34.0597
AC090115.	0.960908	8.003124	0	0	1.107485	0	0	0	0	0	0	0.920078	1.028084	1.861441	0.978006	1.648904	0	0	0
AC090116.	74.95079	109.0426	119.896	22.86444	147.2955	119.8981	61.17698	86.12051	58.36439	133.5372	183.1227	37.72321	89.44334	61.42754	58.68035	14.01568	5.131881	2.7304	12.54831
AC090124.	13.45271	43.01679	29.97401	15.58939	13.28982	25.12151	54.37954	22.22465	9.338302	10.56769	4.81902	15.64133	18.50552	60.49682	27.38416	28.03136	24.63303	23.66347	53.77848
AC090124.	5.765445	5.001952	0	3.117878	1.107485	1.141887	6.797442	0.926027	29.18219	4.803496	3.614265	10.12086	3.084253	2.792161	3.912023	39.57369	34.89679	17.29253	25.09662
AC090124.	0	5.001952	0.999134	0	0	0	0	1.852054	0	5.764196	0	0	1.028084	3.722881	13.69208	0	0	0	1.792616
AC090125.	0	1.00039	0	0	0	0	5.098081	0	0	0	0	0	0	0	0	0	0	0	0
AC090136.	15.37452	0	0	5.196464	1.107485	1.141887	2.549041	14.81643	0	0.960699	3.614265	4.600391	0	0	0.978006	0.824452	0	0	0.896308
AC090136.	22.10087	2.000781	4.995668	1.039293	8.859879	6.851322	2.549041	12.03835	24.51304	0	1.204755	11.96102	18.50552	6.515042	3.912023	3.297807	7.184634	27.304	13.44462
AC090142.	0	0	3.996535	0	0	0	0	0	0	0	0	0	0	0	0	0	0	0	0
AC090150.	0	0	0	0	0	0	0.84968	0	0	0	0	0.920078	0	0	0	0	0	0	0
AC090151.	0	0	0	0	0	0	0	0	0	0.960699	0	0	0	0	0	0.824452	0	0	0
AC090152.	368.0276	274.107	363.6847	160.0511	342.2128	373.3971	170.7857	325.9615	412.0526	456.3321	598.7632	412.195	425.6269	263.3938	307.0938	155.8214	238.1193	313.996	288.6112
AC090159.	0	1.00039	0	4.157171	0	0	1.69936	0	0	0.960699	0	0.920078	0	0.93072	0	0.824452	0	1.820267	0
AC090164.	14.41361	1.00039	2.997401	6.235757	7.752394	3.425661	0.84968	3.704108	5.836439	0	6.023774	1.840156	0	2.792161	5.868035	0.824452	6.158258	4.550667	11.652
AC090164.	0	0	0.999134	0	0	4.567548	0.84968	0	0	0	1.204755	0	2.056169	0	3.912023	0.824452	1.026376	0	0
AC090164.	0	2.000781	0	0	0	0	0	0	0	0	0	0.920078	0	0	0	0	0	10.01147	3.585232

AC090181.	69.18534	57.02225	74.93502	57.1611	63.12664	17.12831	81.5693	92.6027	42.02236	65.32755	19.27608	94.76806	66.82548	95.86419	71.39443	61.00944	56.45069	38.2256	57.36371
AC090181.	61.49808	35.01367	53.95322	139.2652	66.4491	11.41887	124.903	59.26573	28.01491	11.52839	42.16642	105.809	102.8084	52.12034	49.8783	90.6897	64.6617	50.05734	55.57109
AC090186.	24.9836	31.0121	36.96795	37.41454	26.57964	11.41887	27.18977	46.30135	1.167288	1.921399	4.81902	34.04289	41.12337	30.71377	22.49413	23.9091	24.63303	29.12427	25.09662
AC090192.	0	0	0	2.078586	0	0	0	0	0	0	0	3.680313	3.084253	0.93072	0.978006	4.122259	0	0.910133	1.792616
AC090192.	44.20175	46.01796	41.96361	49.88605	12.18233	3.425661	94.31451	16.66849	3.501863	11.52839	7.228529	25.76219	20.56169	65.15042	38.14223	23.9091	42.08143	38.2256	20.61508
AC090193.	0	0	0	0	0	0	0	0	0	0	0	1.840156	2.056169	0	5.868035	3.297807	0	0	0
AC090197.	82.63805	50.01952	69.93936	136.1474	129.5757	92.49285	79.86994	128.7178	33.85134	71.09174	104.8137	117.77	113.0893	90.27987	123.2287	138.5079	153.9564	101.9349	84.25295
AC090198.	1047.389	1284.501	1478.718	973.8173	1256.995	1367.981	1070.597	1401.079	675.8596	1291.18	1096.327	1251.306	1102.106	1148.509	1200.991	799.7183	889.8682	1170.432	976.9757
AC090200.	0	1.00039	0.999134	1.039293	0	1.141887	12.7452	0.926027	0	1.921399	2.40951	5.520469	2.056169	4.653602	4.890029	9.893422	7.184634	2.7304	5.377848
AC090204.	1015.679	1228.479	902.2177	1167.126	996.7364	1107.63	1064.649	1073.265	861.4583	916.5071	824.0523	943.0802	1093.882	967.9491	920.3035	1052.001	1008.928	1039.372	1030.754
AC090206.	0	20.00781	13.98787	27.02161	23.25718	10.27698	15.29424	2.778081	12.84017	6.724895	16.86657	10.12086	23.64594	18.61441	22.49413	4.946711	5.131881	23.66347	31.37078
AC090220.	0.960908	0	0	4.157171	1.107485	0	0	0	4.669151	0	0	0	0	0	0	1.648904	0	8.191201	0
AC090227.	0.960908	0	1.998267	0	1.107485	0	0	0	9.338302	0.960699	0	0.920078	0	0	0	0	0	0	0
AC090229.	82.63805	73.0285	133.8839	91.45776	36.547	63.94567	101.1119	36.11505	40.85507	65.32755	89.15186	27.60235	37.01104	50.2589	46.94428	87.39189	114.9541	62.79921	56.4674
AC090236.	14.41361	15.00586	30.97314	3.117878	27.68712	0	22.09169	22.22465	0	0	4.81902	34.96297	23.64594	33.50593	33.2522	14.01568	7.184634	7.281067	8.963079
AC090241.	1.921815	0	0.999134	5.196464	0	0	0	0	0	0.960699	0	5.520469	0	0.93072	5.868035	0	2.052753	0	0
AC090241.	48.04538	71.02772	66.94196	104.9686	45.40688	49.10114	90.91579	83.34243	84.04472	49.95636	55.41872	42.3236	65.7974	58.63538	112.4707	72.55176	74.92547	83.73227	94.11233
AC090241.	152.7843	137.0535	103.9099	242.1552	158.3703	190.6951	238.7601	167.6109	177.4277	129.6944	149.3896	153.6531	198.4203	101.4485	132.0308	172.3104	170.3785	98.29441	179.2616
AC090246.	13.45271	15.00586	25.97748	14.5501	7.752394	19.41208	18.69297	5.556162	36.18592	13.44979	32.52838	16.56141	12.33701	33.50593	19.56012	14.84013	17.4484	26.39387	9.859387
AC090255.	0	0	0	0	0	0	0.84968	0	1.167288	0	0	0	0	0	0	0	0	0	0
AC090260.	51.88901	46.01796	110.9038	67.55403	54.26676	63.94567	75.62154	34.263	28.01491	89.34503	46.98544	86.48735	76.07824	42.81313	53.79032	48.64266	71.84634	83.73227	101.2828
AC090281.	3.84363	1.00039	2.997401	2.078586	0	0	0.84968	0	2.334575	0	0	0	0	0	0	6.595615	7.184634	5.4608	0.896308
AC090282.	0	8.003124	0.999134	6.235757	1.107485	3.425661	11.89552	3.704108	0	5.764196	6.023774	2.760235	1.028084	0	0	0	0	0	0
AC090282.	6.726353	8.003124	8.992203	7.275049	5.537425	0	2.549041	7.408216	2.334575	7.685594	1.204755	5.520469	6.168506	0.93072	0	11.54233	7.184634	10.9216	8.066772
AC090283.	0	0	0.999134	0	1.107485	0	0	0	0	0	0	0.920078	2.056169	0	0	0	0	0.910133	0
AC090286.	6.726353	18.00703	2.997401	5.196464	8.859879	15.98642	5.098081	9.26027	4.669151	16.33189	13.2523	11.96102	9.252759	5.584322	15.64809	5.771163	9.237386	4.550667	4.48154
AC090311.	0	0	0.999134	0	0	0	0	0	0	0	0	0	0	0	0	0.824452	0	0	0
AC090330.	2.882723	2.000781	0	3.117878	0	0	1.69936	5.556162	10.50559	0	3.614265	0	1.028084	9.307203	0	1.648904	1.026376	0.910133	0.896308
AC090337.	10.56998	10.0039	9.991337	10.39293	35.43952	21.69585	3.398721	3.704108	5.836439	17.29259	30.11887	14.72125	24.67402	3.722881	8.802052	17.31349	20.52753	20.93307	3.585232
AC090340.	1.921815	1.00039	0	0	0	2.283774	0.84968	4.630135	0	0.960699	0	0	1.028084	0.93072	0	1.648904	0	0.910133	6.274156
AC090358.	67.26353	18.00703	12.98874	4.157171	13.28982	15.98642	28.88913	35.18903	21.01118	22.09608	13.2523	32.20274	47.29188	30.71377	51.83431	4.122259	22.58028	4.550667	4.48154
AC090360.	298.8422	484.189	280.7566	308.67	281.3012	441.9103	313.532	399.1177	817.1014	622.5331	965.0087	270.503	274.4985	408.5862	338.39	284.4359	307.9129	213.8814	320.8782
AC090371.	0.960908	0	1.998267	0	0	0	0	0.926027	0	0	0	0.920078	0	0	0	0	0	0	0
AC090373.	0	0	0	0	0	0	1.69936	0	0	0	0	0	0	0	0	0	4.105505	0	0
AC090376.	0	0	0	0	0	0	0.84968	0.926027	0	0	0	0	0	0	0	0	0	0	0.896308
AC090377.	2.882723	5.001952	0	0	1.107485	0	16.9936	0	0	1.921399	1.204755	0	1.028084	0	0	0	0	0.910133	3.585232
AC090386.	0.960908	0	0	0	0	0	0.84968	0	0	0	0	0	0	3.722881	0.978006	0.824452	0	0.910133	0
AC090386.	0	0	0	0	0	0	0	0	0	3.842797	0	0	0	0	0	0	0	0	0
AC090398.	0	0	0	0	0	0	0.84968	0	0	1.921399	0	0	0	0	0	0.824452	0	0	0
AC090403.	0	0	1.998267	0	2.21497	1.141887	0.84968	0	0	0.960699	1.204755	0	5.140422	0	4.890029	0	0	2.7304	0
AC090409.	101.8562	53.02069	53.95322	37.41454	109.641	87.9253	45.03305	89.82462	81.71014	86.46293	83.12809	80.04681	59.62889	93.07203	105.6246	62.65834	95.45299	75.54107	53.77848
AC090409.	1.921815	0	0.999134	4.157171	8.859879	4.567548	1.69936	2.778081	0	5.764196	0	3.680313	2.056169	1.861441	0	0	4.105505	6.370934	2.688924
AC090424.	0	0	0	1.039293	0	4.567548	0	0	0	6.724895	0	0	0	0	0	0	0	0.910133	0
AC090425.	48.04538	44.01718	35.96881	31.17878	26.57964	45.67548	58.62794	53.70957	17.50932	20.17468	40.96167	37.72321	35.98295	71.66546	38.14223	33.80253	34.89679	40.956	33.16339
AC090425.	65.34171	76.02967	72.93676	90.41847	77.52394	54.81058	78.17058	107.4191	39.68778	78.77734	43.37118	57.96493	56.54464	47.46674	56.72434	49.46711	52.34519	38.2256	43.91909
AC090453.	0	0	0	0	0	0	0	0	0	0	0	0.920078	0	0	0.978006	0	0	0	0
AC090458.	3.84363	9.003514	4.995668	19.74656	5.537425	25.12151	18.69297	12.03835	0	5.764196	0	2.760235	0	0	0.978006	1.648904	5.131881	0.910133	2.688924
AC090498.	146.0579	71.02772	60.94715	83.14342	101.8886	73.08077	60.3273	74.08216	81.71014	45.15286	60.23774	80.04681	93.55568	49.32818	61.61437	92.3386	101.6112	61.88907	44.8154
AC090502.	0	1.00039	2.997401	0	0	0	0	0	0	6.724895	0	0	0	0.93072	0.978006	1.648904	0	0	0.896308
AC090502.	0	1.00039	0	0	0	0	0	0	1.167288	0	0	0	0	0	0	0.824452	0	0	0
AC090505.	0	0	0	0	0	0	0	0	4.669151	0	0	0.920078	0	0	0	0	0	3.640534	0
AC090506.	0	0	0	0	0	1.141887	4.248401	0	0	0	0	0	0	0	0	0	0	0	0
AC090510.	174.8852	148.0578	168.8536	102.89	186.0575	196.4046	113.8572	161.1287	287.1528	240.1748	295.1649	199.657	223.0943	137.7466	137.8988	105.5298	96.47937	150.172	152.3724
AC090515.	3.84363	6.002343	7.993069	1.039293	5.537425	5.709435	8.496802	9.26027	19.84389	0	1.204755	0.920078	2.056169	7.445762	0	3.297807	4.105505	0.910133	0
AC090515.	202.7515	199.0777	264.7704	152.776	137.3281	167.8574	208.1717	163.9068	273.1453	227.6857	234.9272	191.3763	200.4765	214.9964	240.5894	162.417	205.2753	303.0744	360.3158
AC090515.	1.921815	0	0.999134	0	6.64491	1.141887	0	0	0	0	0	0.920078	0	1.861441	0	0	0	0.910133	1.792616
AC090515.	19.21815	8.003124	22.98007	12.47151	14.3973	22.83774	24.64073	22.22465	9.338302	26.89958	13.2523	11.96102	15.42127	9.307203	1.956012	16.48904	22.58028	10.9216	23.30401
AC090515.	4.804538	1.00039	13.98787	4.157171	29.90209	4.567548	11.89552	8.334243	1.167288	2.882098	1.204755	3.680313	16.44935	12.09936	5.868035	6.595615	4.105505	3.640534	10.7557
AC090517.	0.960908	0	0	4.157171	6.64491	1.141887	2.549041	5.556162	0	3.842797	0	0	5.140422	0	3.912023	0	0	4.550667	1.792616
AC090517.	57.65445	41.01601	104.909	64.43615	84.16885	42.24982	58.62794	55.56162	26.84762	48.99566	85.5376	64.40548	50.37613	93.07203	89.97654	70.07841	53.37157	55.51814	49.29694
AC090517.	1.921815	0	0.999134	3.117878	11.07485	4.567548	2.549041	0	0	1.921399	0	1.840156	9.252759	1.861441	0.978006	1.648904	3.079129	0	0.896308
AC090518.	6.726353	3.001171	1.998267	3.117878	1.107485	6.851322	4.248401	5.556162	3.501863	1.921399	3.614265	8.280704	1.028084	6.515042	2.934017	6.595615	4.105505	3.640534	1.792616

AC090519.	3.84363	21.0082	12.98874	6.235757	1.107485	5.709435	1.69936	0.926027	1.167288	12.48909	8.433284	2.760235	15.42127	3.722881	5.868035	2.473355	2.052753	5.4608	6.274156
AC090519.	9.609075	1.00039	1.998267	6.235757	5.537425	2.283774	2.549041	11.11232	1.167288	7.685594	1.204755	0.920078	11.30893	1.861441	0.978006	4.946711	5.131881	4.550667	1.792616
AC090525.	0	0	0	0	0	0	0	0	7.003726	0	0	0	0	0	0	0	0	0	0
AC090525.	0.960908	0	0	0	0	0	1.69936	0	0	0	0	0	0	0	0	0	0	0	0
AC090527.	0	0	2.997401	0	0	0	0	0	0	3.842797	1.204755	0.920078	0	0	0.978006	0	0	0	1.792616
AC090527.	28.82723	71.02772	67.94109	17.66798	25.47215	21.69585	37.38593	65.74792	16.34203	61.48475	33.73314	41.40352	28.78636	27.92161	20.53812	28.85581	29.76491	50.05734	41.23017
AC090531.	1.921815	2.000781	4.995668	0	1.107485	1.141887	0.84968	13.89041	5.836439	3.842797	0	4.600391	3.084253	1.861441	4.890029	20.6113	2.052753	0	0
AC090539.	0	0	0	0	0	0	0	0	9.338302	0	0	0	0	0	0	0	0	0	0
AC090541.	0	0	1.998267	5.196464	0	10.27698	2.549041	0	8.171014	1.921399	0	0	1.028084	0.93072	1.956012	4.946711	1.026376	0	0.896308
AC090543.	4.804538	0	3.996535	0	0	0	0.84968	9.26027	0	0	0	0	0	7.445762	1.956012	0.824452	2.052753	5.4608	0.896308
AC090543.	5.765445	9.003514	3.996535	0	2.21497	2.283774	1.69936	2.778081	5.836439	2.882098	7.228529	7.360626	0	5.584322	3.912023	13.19123	11.29014	7.281067	0.896308
AC090559.	2524.304	1049.41	1276.893	2250.069	967.9418	1146.455	1168.31	1677.035	692.2016	564.8912	528.8874	1737.108	1152.483	1377.466	1211.749	2464.287	1610.384	2955.203	2286.482
AC090559.	0	0	0	13.51081	2.21497	0	0	0	16.34203	0.960699	0	3.680313	4.112337	0.93072	0.978006	1.648904	1.026376	1.820267	2.688924
AC090568.	55.73264	59.02304	22.98007	34.29666	19.93473	38.82416	52.68017	12.96438	3.501863	10.56769	2.40951	58.88501	71.96591	28.85233	37.16422	33.80253	39.0023	36.40534	57.36371
AC090572.	0	0	0	0	0	0	0.84968	0	5.836439	0	0	0	0	0	0	0	0	0	0.896308
AC090578.	13.45271	18.00703	9.991337	22.86444	31.00958	28.54718	30.58849	12.96438	5.836439	17.29259	13.2523	15.64133	21.58977	9.307203	14.67009	3.297807	2.052753	10.9216	6.274156
AC090579.	221.0087	101.0394	146.8726	96.65423	118.5009	79.93209	99.41259	131.4958	79.37557	90.30573	89.15186	103.0488	125.4263	131.2316	129.0968	89.0408	59.52982	53.69787	60.94894
AC090582.	0	1.00039	1.998267	17.66798	4.42994	0	1.69936	7.408216	0	0	2.40951	0.920078	1.028084	5.584322	0	4.122259	9.237386	1.820267	7.170464
AC090587.	8.648168	19.00742	16.98527	6.235757	24.36467	17.12831	17.84329	28.70684	28.01491	12.48909	28.91412	30.36258	17.47743	21.40657	4.890029	19.78684	36.94955	36.40534	12.54831
AC090587.	14.41361	12.00469	17.98441	8.314342	13.28982	18.27019	12.7452	7.408216	14.00745	15.37119	31.32363	14.72125	14.39318	27.92161	7.824047	13.19123	15.39564	27.304	19.71877
AC090589.	21.13997	7.002733	26.97661	3.117878	18.82724	3.425661	14.44456	28.70684	33.85134	87.42363	87.94711	6.440548	6.168506	9.307203	0	7.420066	16.42202	2.7304	7.170464
AC090589.	10.56998	19.00742	19.98267	13.51081	16.61227	9.135096	18.69297	11.11232	3.501863	3.842797	7.228529	8.280704	2.056169	3.722881	14.67009	7.420066	8.21101	1.820267	17.02985
AC090589.	655.3389	469.1831	635.449	510.2928	640.1263	503.5722	618.5672	651.923	443.5693	494.7601	352.9932	642.2146	660.0302	598.4532	624.9457	511.9846	459.8166	618.8907	417.6795
AC090592.	0	1.00039	0.999134	0	0	1.141887	0	0	0	0	1.204755	0	6.168506	3.722881	0	0	0	0.910133	0
AC090602.	9.609075	14.00547	0.999134	1.039293	8.859879	3.425661	2.549041	4.630135	8.171014	4.803496	14.45706	8.280704	10.28084	5.584322	0.978006	6.595615	2.052753	4.550667	2.688924
AC090607.	38.4363	54.02108	57.94975	69.63262	79.73891	35.3985	103.661	61.11778	44.35693	29.78168	28.91412	96.60821	77.10633	43.74385	70.41642	52.76492	44.13418	50.05734	69.91202
AC090607.	275.7805	242.0945	288.7496	330.4951	455.1763	283.188	297.3881	392.6355	324.506	250.7425	171.0752	378.1522	459.5537	467.2216	467.4868	262.1757	215.539	265.759	230.3511
AC090607.	0.960908	1.00039	0	0	0	0	0	0	0	0	0	0.920078	0	3.722881	0	3.297807	0	0	0.896308
AC090607.	0	0	0	0	0	0	0	0	0	2.882098	0	0	0	0	0	0	0	0.910133	0
AC090615.	8.648168	0	0	0	0	15.98642	0	3.704108	0	11.52839	6.023774	6.440548	1.028084	0.93072	1.956012	0	0	0	0.896308
AC090616.	0	1.00039	0.999134	6.235757	0	0	5.098081	1.852054	0	0.960699	0	5.520469	0	0	0.978006	0.824452	4.105505	3.640534	0.896308
AC090616.	5.765445	4.001562	6.993936	8.314342	6.64491	1.141887	1.69936	0	14.00745	2.882098	0	1.840156	2.056169	0	1.956012	3.297807	7.184634	0.910133	0.896308
AC090616.	0	6.002343	3.996535	1.039293	1.107485	3.425661	0	7.408216	4.669151	0	1.204755	2.760235	1.028084	5.584322	0	0.824452	1.026376	3.640534	8.066772
AC090616.	0.960908	6.002343	0.999134	0	7.752394	2.283774	0.84968	5.556162	0	2.882098	0	4.600391	6.168506	0.93072	1.956012	2.473355	0	0	0
AC090616.	12.4918	3.001171	1.998267	9.353635	0	11.41887	4.248401	0	5.836439	3.842797	1.204755	15.64133	9.252759	0	19.56012	8.244518	3.079129	10.01147	8.066772
AC090616.	97.05166	142.0554	186.838	61.31827	147.2955	133.6008	156.3412	238.915	157.5838	298.7775	445.7593	230.0196	187.1114	154.4996	150.6129	201.9907	193.9851	155.6328	196.2914
AC090616.	719.7197	726.2835	985.1458	469.7603	612.4392	741.0847	680.5939	672.2956	587.1457	829.0835	883.0853	632.0937	674.4233	732.4769	755.9985	436.9595	479.3177	520.5963	538.6811
AC090617.	13.45271	5.001952	4.995668	0	0	0	0.84968	0	23.34575	5.764196	4.81902	0.920078	0	0.93072	0	0	5.131881	8.191201	8.066772
AC090617.	15.37452	21.0082	25.97748	11.43222	13.28982	3.425661	11.04584	31.48492	2.334575	21.13538	16.86657	21.1618	20.56169	18.61441	22.49413	20.6113	16.42202	17.29253	18.82247
AC090617.	1.921815	2.000781	0	0	1.107485	0	0.84968	4.630135	0	0	1.204755	0.920078	3.084253	0.93072	0	0	0	1.820267	0.896308
AC090617.	1.921815	4.001562	5.994802	1.039293	4.42994	0	0.84968	4.630135	22.17847	0	0	0.920078	1.028084	0.93072	9.780058	0	8.21101	4.550667	5.377848
AC090617.	12.4918	0	19.98267	8.314342	16.61227	11.41887	15.29424	0.926027	0	7.685594	0	3.680313	16.44935	9.307203	6.846041	10.71787	3.079129	12.74187	8.066772
AC090617.	159.5106	178.0695	166.8553	205.78	160.5853	175.8506	167.387	182.4273	288.3201	231.5285	302.3935	161.9338	169.6339	210.3428	158.4369	193.7462	171.4048	198.4091	162.2317
AC090617.	0	1.00039	1.998267	0	0	2.283774	0.84968	11.11232	3.501863	0	1.204755	4.600391	1.028084	0	1.956012	3.297807	3.079129	4.550667	1.792616
AC090617.	0.960908	1.00039	3.996535	3.117878	1.107485	1.141887	0.84968	0	3.501863	0	0	1.840156	0	3.722881	0	0	0	0	0
AC090617.	0	0	0	0	0	0	2.549041	0.926027	0	0	0	0.920078	2.056169	0.93072	0	0	2.052753	0.910133	0
AC090618.	22.10087	30.01171	46.95928	40.53242	27.68712	18.27019	100.2623	25.92876	9.338302	37.46727	44.57593	22.08188	16.44935	20.47585	29.34017	14.01568	9.237386	26.39387	51.98586
AC090625.	0	0	0	4.157171	0	0	0	0	1.167288	0	0	0	1.028084	0	0	0	1.026376	3.640534	9.859387
AC090630.	9.609075	15.00586	21.98094	4.157171	16.61227	0	0	11.11232	4.669151	7.685594	10.84279	8.280704	3.084253	16.75297	34.2302	11.54233	2.052753	18.20267	7.170464
AC090643.	0.960908	1.00039	0	0	0	0	0	0	1.167288	2.882098	0	0.920078	0	2.792161	1.956012	0.824452	0	0	0.896308
AC090643.	0	0	0	0	0	0	0	0	0	0	0	0	0	0	2.934017	0	0	0	0
AC090651.	3.84363	0	0.999134	5.196464	7.752394	3.425661	0.84968	0.926027	0	0	4.81902	0	2.056169	0	0.978006	0	0	0	3.585232
AC090666.	0.960908	0	2.997401	23.90373	0	4.567548	8.496802	0.926027	21.01118	4.803496	24.0951	11.96102	11.30893	8.376483	6.846041	8.244518	14.36927	17.29253	13.44462
AC090673.	211.3997	314.1226	207.8198	108.0864	190.4874	211.2491	109.6088	142.6082	165.7549	412.14	277.0936	223.579	248.7964	159.1532	246.4575	38.74924	65.68808	135.6099	131.7573
AC090673.	0	2.000781	0.999134	0	2.21497	0	0	1.852054	7.003726	2.882098	2.40951	1.840156	3.084253	9.307203	0	0.824452	0	0	0
AC090680.	0	0	1.998267	0	0	0	0	0	0	0	0	0	0	0	0	0	0	0	0
AC090686.	0	2.000781	5.994802	4.157171	0	0	0	0	3.501863	0	0	2.760235	1.028084	5.584322	0.978006	0	2.052753	1.820267	1.792616
AC090692.	5.765445	0	0	0	0	0	0	0	0	0	0	0	0	0	0	0	0	0	0
AC090695.	37.47539	62.02421	99.91337	75.86837	75.30897	45.67548	87.51706	117.6054	0	94.14853	65.05676	119.6102	84.30292	120.9936	106.6026	74.20066	36.94955	73.72081	84.25295
AC090699.	0	0	0	2.078586	3.322455	0	0	0	0	0.960699	7.228529	0	1.028084	0.93072	0.978006	0.824452	1.026376	0.910133	0
AC090707.	4.804538	2.000781	0	0	0	5.709435	0.84968	0	0	0	0	2.760235	0	0	0.978006	4.122259	2.052753	0.910133	0
AC090709.	0.960908	0	18.98354	5.196464	3.322455	4.567548	7.647122	7.408216	0	0.960699	0	8.280704	3.084253	7.445762	0	3.297807	6.158258	0.910133	4.48154

AC090735.	0	0	0	21.82515	0	0	0	40.74519	0	8.646293	15.66181	0	0	0.93072	0.978006	0	3.079129	0	0.896308
AC090739.	40.35812	55.02147	104.909	40.53242	125.1458	77.64832	81.5693	148.1643	85.212	134.4979	118.066	94.76806	122.342	113.5479	97.80058	89.86525	66.71446	117.4072	76.18618
AC090740.	0	0	0	1.039293	0	0	0	0	0	0	0	0	0	0	0	0.824452	0	0	0
AC090751.	0	0	0.999134	0	0	0	5.098081	8.334243	0	0.960699	0	0	3.084253	0	0.978006	0	0	0	0
AC090771.	9.609075	0	0	0	2.21497	0	0.84968	0	0	0.960699	0	0.920078	6.168506	0	0	0	0	10.01147	1.792616
AC090771.	41.31902	27.01054	0	0	23.25718	1.141887	33.13753	7.408216	0	4.803496	16.86657	12.8811	18.50552	0.93072	4.890029	18.13794	18.47477	12.74187	15.23724
AC090772.	39.39721	33.01288	48.95755	48.84676	37.65449	31.97284	66.27506	38.89314	23.34575	55.72056	40.96167	63.4854	51.40422	27.92161	58.68035	26.38246	45.16056	60.97894	63.63786
AC090772.	0	0	7.993069	0	0	1.141887	0	6.482189	0	1.921399	0	1.840156	2.056169	2.792161	1.956012	1.648904	0	11.83173	1.792616
AC090772.	136.4489	197.0769	166.8553	152.776	214.8521	258.0665	158.8902	227.8027	242.7958	257.4674	142.1611	115.0098	135.7071	201.0356	162.349	157.4703	213.4863	91.92348	133.5499
AC090772.	73.02897	151.059	122.8934	77.94696	169.4452	153.0129	90.06611	122.2356	236.9594	140.2621	106.0184	85.56728	56.54464	92.14131	120.2947	87.39189	101.6112	74.63094	54.67478
AC090774.	0	0	0	0	0	0	0	1.69936	0	0	0	0	0	0	0	0	0	0	0
AC090774.	20.17906	32.01249	19.98267	16.62868	27.68712	15.98642	19.54265	26.85478	0	14.41049	30.11887	19.32164	29.81445	16.75297	11.73607	16.48904	30.79129	8.191201	21.51139
AC090774.	21.13997	11.00429	7.993069	9.353635	102.9961	14.84453	35.68657	9.26027	15.17474	52.83846	86.74235	60.72516	54.48847	35.36737	29.34017	4.946711	3.079129	8.191201	8.066772
AC090791.	3.84363	0	0.999134	5.196464	1.107485	1.141887	10.19616	0	7.003726	2.882098	2.40951	3.680313	2.056169	0	0.978006	13.19123	10.26376	9.101334	2.688924
AC090796.	0	0	0	11.43222	0	0	0.84968	3.704108	2.334575	2.882098	0	0	3.084253	0	9.780058	18.96239	14.36927	8.191201	3.585232
AC090802.	0.960908	0	0	0	0	0	0	0	1.167288	1.921399	0	0	2.056169	0	0	0	0	1.820267	0
AC090804.	4.804538	20.00781	16.98527	5.196464	9.967364	21.69585	17.84329	15.74246	38.5205	34.58517	37.3474	11.96102	41.12337	12.09936	4.890029	16.48904	5.131881	4.550667	13.44462
AC090820.	0	0	0	0	0	0	0	0	0	0	0	1.840156	0	0	0	0	0	0	0
AC090825.	245.9923	154.0601	254.7791	141.3438	406.447	188.4114	118.9552	179.6492	173.9259	274.76	350.5837	255.7817	254.9649	222.4422	252.3255	143.4546	176.5367	71.90054	52.88217
AC090826.	8.648168	5.001952	35.96881	31.17878	39.86946	17.12831	43.33369	38.89314	7.003726	11.52839	16.86657	70.84602	40.09529	27.92161	31.29619	50.29156	35.92317	20.02294	39.43755
AC090826.	52.84991	36.01406	94.9177	69.63262	70.87903	22.83774	44.18337	132.4219	31.51677	48.99566	37.3474	126.0507	114.1174	102.3792	127.1408	95.63641	111.875	119.2275	67.2231
AC090826.	0	0	0	0	0	0	0	0	0	0	0	0.920078	0	0	0	0	0	3.640534	0
AC090833.	66.30262	40.01562	10.99047	56.12181	27.68712	7.993209	37.38593	7.408216	14.00745	46.11356	18.07132	27.60235	16.44935	23.26801	30.31818	109.6521	102.6376	36.40534	52.88217
AC090835.	0	0	0	0	0	0	0	0	2.334575	0	0	0	0	0	0	0	1.026376	0	0
AC090844.	0	0	0	1.039293	0	1.141887	0	0	0	0	0	1.840156	0	0	0	0.824452	0	0	0
AC090844.	67.26353	81.03163	149.87	47.80747	131.7907	49.10114	87.51706	84.26846	72.37184	134.4979	89.15186	123.2905	67.85357	122.8551	88.99853	30.50472	60.5562	138.3403	141.6167
AC090844.	11.53089	1.00039	0	1.039293	7.752394	4.567548	0.84968	1.852054	3.501863	3.842797	0	1.840156	8.224675	0	1.956012	3.297807	0	1.820267	0.896308
AC090877.	24.9836	53.02069	37.96708	24.94303	23.25718	43.39171	27.18977	51.85751	81.71014	68.20965	81.92333	24.84211	44.20763	44.67457	64.54838	45.34485	27.71216	35.4952	34.95601
AC090877.	0.960908	0	0	2.078586	0	0	0	0	0	0	0	0	0	0.93072	0.978006	2.473355	0	0	5.377848
AC090897.	216.2042	441.1722	297.7418	336.7309	397.5871	322.0121	302.4862	340.7779	287.1528	325.677	297.5745	239.2203	265.2458	403.0019	405.8724	326.4829	318.1766	354.952	308.3299
AC090897.	1.921815	0	1.998267	0	0	0	3.398721	0	0	0	0	1.840156	2.056169	0	0.978006	1.648904	4.105505	5.4608	1.792616
AC090907.	51.88901	28.01093	25.97748	22.86444	54.26676	42.24982	23.79105	23.15068	101.554	54.75986	80.71858	32.20274	43.17954	27.92161	37.16422	32.15362	37.97592	21.8432	41.23017
AC090907.	54.77173	45.01757	39.96535	35.33595	66.4491	49.10114	33.13753	39.81916	108.5578	98.95202	57.82823	43.24368	33.92678	51.18962	41.07624	35.45143	46.18693	30.0344	26.88924
AC090912.	3.84363	12.00469	1.998267	6.235757	6.64491	1.141887	4.248401	5.556162	0	4.803496	21.68559	8.280704	6.168506	6.515042	2.934017	2.473355	4.105505	1.820267	11.652
AC090912.	2.882723	16.00625	0.999134	15.58939	0	4.567548	1.69936	0	0	4.803496	4.81902	3.680313	1.028084	0	2.934017	4.122259	1.026376	0	0
AC090912.	7.68726	1.00039	5.994802	6.235757	0	4.567548	0.84968	0	3.501863	5.764196	4.81902	2.760235	3.084253	0	2.934017	0.824452	5.131881	7.281067	0
AC090921.	18.25724	47.01835	26.97661	31.17878	24.36467	43.39171	34.83689	42.59724	89.88116	27.86028	28.91412	25.76219	16.44935	38.15953	43.03226	33.80253	47.21331	48.23707	29.57816
AC090922.	52.84991	32.01249	36.96795	73.78979	40.97694	31.97284	66.27506	40.74519	39.68778	44.19217	3.614265	57.96493	31.87062	49.32818	60.63636	50.29156	32.84404	57.33841	34.95601
AC090948.	23.06178	56.02186	36.96795	35.33595	21.04221	50.24303	25.49041	57.41368	114.3942	69.17035	31.32363	59.80509	46.2638	52.12034	63.57038	46.1693	46.18693	43.6864	54.67478
AC090948.	74.95079	52.0203	97.9151	107.0472	63.12664	62.80379	86.66738	68.526	23.34575	63.40615	74.6948	74.52634	54.48847	77.24979	35.20821	106.3543	66.71446	39.13574	48.40063
AC090948.	49.00628	18.00703	59.94802	87.30059	75.30897	55.95246	49.28145	48.15341	150.5801	62.44545	75.89956	72.68618	45.23571	45.60529	56.72434	78.32292	62.60895	53.69787	62.74156
AC090950.	24.9836	9.003514	49.95668	3.117878	9.967364	22.83774	24.64073	17.59451	21.01118	27.86028	24.0951	11.04094	25.70211	12.09936	39.12023	15.66458	27.71216	24.5736	22.4077
AC090950.	3.84363	15.00586	5.994802	8.314342	17.71976	10.27698	20.39233	16.66849	0	21.13538	16.86657	11.96102	7.196591	15.82225	13.69208	2.473355	12.31652	10.9216	1.792616
AC090952.	0	2.000781	1.998267	0	3.322455	0	3.398721	3.704108	1.167288	3.842797	13.2523	10.12086	6.168506	5.584322	1.956012	0.824452	3.079129	9.101334	8.066772
AC090952.	0	0	0	0	0	0	3.398721	0	0	0	4.81902	0	0	0	0	1.648904	0	0.910133	0.896308
AC090953.	0.960908	1.00039	0.999134	7.275049	4.42994	6.851322	0.84968	3.704108	0	0	0	0.920078	0	0	3.912023	0.824452	0	0	0
AC090954.	94.16894	87.03397	113.9012	84.18271	115.1784	37.68227	94.31451	74.08216	213.6137	29.78168	9.638039	127.8909	163.4654	52.12034	65.52639	38.74924	13.34289	38.2256	10.7557
AC090957.	0	0	0	0	0	0	0	0	0	0	0	6.440548	0	0	0	0	1.026376	0.910133	0
AC090960.	0	0	0	0	6.64491	0	0	0	0	0	0	0	1.028084	0.93072	0	0.824452	0	0	0
AC090970.	0	4.001562	0.999134	1.039293	2.21497	2.283774	4.248401	5.556162	8.171014	0.960699	7.228529	3.680313	1.028084	0.93072	0.978006	0	4.105505	3.640534	1.792616
AC090970.	59.57627	43.01679	36.96795	31.17878	9.967364	30.83095	18.69297	32.41095	31.51677	46.11356	10.84279	40.48344	49.34805	53.98178	46.94428	59.36053	18.47477	80.09174	59.15632
AC090970.	57.65445	88.03436	84.92636	68.59332	55.37425	57.09435	50.13113	49.07943	165.7549	99.91272	98.7899	71.7661	67.85357	71.66546	51.83431	62.65834	63.63533	99.20454	77.08248
AC090971.	1.921815	2.000781	16.98527	6.235757	9.967364	3.425661	3.398721	12.03835	14.00745	3.842797	3.614265	15.64133	14.39318	2.792161	8.802052	5.771163	1.026376	1.820267	3.585232
AC090971.	58.61536	63.0246	65.94282	74.82908	43.19191	42.24982	64.5757	106.4931	86.37929	97.99132	75.89956	69.92595	48.31997	64.2197	55.74633	76.67402	50.29244	39.13574	29.57816
AC090971.	0.960908	0	9.991337	8.314342	0	4.567548	0.84968	0	1.167288	0	0	3.680313	0	12.09936	0.978006	0	3.079129	0	0.896308
AC090971.	0	1.00039	7.993069	11.43222	0	2.283774	4.248401	1.852054	2.334575	11.52839	0	2.760235	2.056169	1.861441	0.978006	0.824452	0	2.7304	0.896308
AC090983.	20.17906	5.001952	1.998267	14.5501	0	15.98642	11.04584	6.482189	0	2.882098	4.81902	2.760235	2.056169	13.9608	10.75806	5.771163	6.158258	2.7304	2.688924
AC090985.	0.960908	2.000781	3.996535	0	0	0	0.84968	0	0	0	0	3.680313	5.140422	0.93072	0	0	9.237386	0	5.377848
AC090987.	1.921815	0	0	0	0	0	0	0	0	0	0	0	0	0	0	0	0	0	0
AC090993.	0	0	0.999134	1.039293	0	0	0.84968	0	0	0	0	0	0	0.93072	0	0	1.026376	0	1.792616
AC091013.	2.882723	9.003514	10.99047	3.117878	1.107485	2.283774	7.647122	11.11232	37.35321	13.44979	1.204755	2.760235	4.112337	0	0	4.122259	3.079129	3.640534	3.585232
AC091042.	0	1.00039	0	1.039293	4.42994	0	0	0	0	0	0	0	0	0	0.978006	0.824452	0	0	0

AC091044.	0	0	0	0	0	0	0.84968	0	0	3.842797	0	0	0	1.861441	0	0	1.026376	0	1.792616
AC091045.	38.4363	25.00976	48.95755	66.51474	53.15928	39.96605	28.88913	32.41095	33.85134	24.97818	30.11887	54.28462	40.09529	47.46674	53.79032	62.65834	58.50345	22.75334	34.0597
AC091046.	0	0	0	1.039293	0	0	0.84968	0	0	0	0	0.920078	1.028084	0.93072	0	0.824452	0	0	2.688924
AC091046.	0	0	1.998267	0	0	0	0.84968	0	0	4.803496	0	6.440548	0	0	0	0.824452	1.026376	0	0.896308
AC091046.	31.70995	19.00742	26.97661	11.43222	48.72934	13.70264	22.09169	25.92876	56.02981	71.09174	138.5468	9.200782	18.50552	26.99089	21.51613	18.13794	2.052753	25.48374	5.377848
AC091047.	0	1.00039	6.993936	1.039293	1.107485	4.567548	0.84968	0.926027	0	0	1.204755	0.920078	0	0	1.956012	0	0	0	0
AC091053.	0	4.001562	4.995668	6.235757	11.07485	1.141887	6.797442	0	7.003726	0	0	11.04094	12.33701	5.584322	5.868035	6.595615	3.079129	7.281067	10.7557
AC091057.	56.69354	72.02811	113.9012	62.35757	44.2994	23.97963	57.77826	91.67668	33.85134	69.17035	44.57593	80.04681	81.21867	61.42754	88.02052	42.04704	62.60895	78.27147	58.26002
AC091057.	80.71623	94.0367	38.96621	69.63262	52.05179	62.80379	42.48401	75.93422	68.86998	106.6376	93.97088	57.04485	58.60081	58.63538	59.65836	48.64266	74.92547	40.956	49.29694
AC091057.	18.25724	29.01132	11.9896	18.70727	31.00958	10.27698	1.69936	17.59451	23.34575	15.37119	6.023774	20.24172	19.5336	14.89152	14.67009	28.85581	21.5539	58.24854	23.30401
AC091059.	0	0	0	0	0	0	1.69936	0	0	0	4.81902	3.680313	1.028084	3.722881	0	0	0	0	0.896308
AC091059.	0	0	0.999134	3.117878	0	0	0.84968	1.852054	0	0.960699	0	0.920078	11.30893	3.722881	0	0	0	0	0
AC091060.	32.67086	46.01796	64.94369	56.12181	48.72934	36.54038	34.83689	37.96711	16.34203	62.44545	63.85201	49.68422	27.75828	20.47585	52.81231	52.76492	36.94955	62.79921	61.84525
AC091073.	0.960908	5.001952	12.98874	0	0	2.283774	0.84968	5.556162	0	1.921399	1.204755	4.600391	2.056169	6.515042	0.978006	4.122259	2.052753	7.281067	0.896308
AC091076.	0	0	0	0	0	0	0	0	1.167288	0	8.433284	0	0	0	0	0	0	0	0
AC091078.	172.0024	231.0902	276.76	110.165	228.1419	81.07398	201.3742	176.8712	67.70269	223.8429	91.56137	87.40743	112.0612	139.608	214.1833	214.3575	186.8005	64.61947	103.9717
AC091078.	0	0	0	0	0	0	1.69936	0.926027	0	0	0	0	0	0	0.978006	0	0	0	0
AC091078.	0	4.001562	0	0	0	0	0	0	0	0	0	4.600391	1.028084	0	0	0	0	0	0
AC091096.	2.882723	2.000781	0	2.078586	0	0	2.549041	0	1.167288	0	0	0.920078	3.084253	0	0	0	2.052753	0	3.585232
AC091096.	4.804538	2.000781	0.999134	7.275049	0	4.567548	5.098081	4.630135	0	1.921399	1.204755	1.840156	1.028084	3.722881	0.978006	2.473355	7.184634	0.910133	6.274156
AC091100.	48.04538	58.02265	58.94889	62.35757	33.22455	14.84453	62.02666	69.45203	84.04472	23.05678	20.48083	54.28462	68.88165	65.15042	47.92229	42.8715	30.79129	8.191201	40.33386
AC091114.	0.960908	5.001952	1.998267	0	1.107485	1.141887	0.84968	0	0	0	12.04755	4.600391	2.056169	0	0	0.824452	1.026376	1.820267	3.585232
AC091117.	0	3.001171	0	0	0	0	3.398721	0	0	1.921399	0	0	0	0	0	0	1.026376	0	0.896308
AC091132.	4.804538	3.001171	7.993069	4.157171	13.28982	13.70264	6.797442	2.778081	43.18965	2.882098	0	17.48149	13.3651	20.47585	6.846041	13.19123	16.42202	0.910133	6.274156
AC091132.	59.57627	45.01757	129.8874	53.00393	49.83682	25.12151	87.51706	36.11505	33.85134	57.64196	59.03299	69.00587	87.38717	71.66546	64.54838	64.30724	43.1078	40.956	58.26002
AC091132.	0	0	0	0	0	0	0	0	0	0	0	3.614265	0.920078	0	0	0	0	0	0
AC091132.	31.70995	37.01445	40.96448	15.58939	22.1497	31.97284	15.29424	26.85478	42.02236	15.37119	20.48083	39.56336	29.81445	48.39746	44.01026	46.1693	63.63533	40.956	24.20031
AC091132.	12.4918	11.00429	13.98787	19.74656	58.6967	13.70264	5.947762	26.85478	71.20455	3.842797	13.2523	27.60235	38.03912	33.50593	19.56012	32.97807	42.08143	30.0344	32.26709
AC091132.	8.648168	10.0039	13.98787	4.157171	6.64491	2.283774	16.14392	4.630135	0	3.842797	2.40951	6.440548	13.3651	2.792161	2.934017	8.244518	10.26376	10.01147	5.377848
AC091133.	6.726353	9.003514	0.999134	2.078586	2.21497	2.283774	6.797442	1.852054	8.171014	13.44979	2.40951	11.04094	8.224675	3.722881	1.956012	13.19123	2.052753	10.01147	11.652
AC091138.	0	9.003514	4.995668	11.43222	27.68712	12.56076	6.797442	2.778081	8.171014	3.842797	9.638039	14.72125	8.224675	18.61441	15.64809	2.473355	3.079129	6.370934	5.377848
AC091144.	0	0	0	0	2.21497	4.567548	0.84968	5.556162	0	0	0	0	1.028084	1.861441	4.890029	0	0	0	0
AC091147.	20.17906	11.00429	26.97661	12.47151	13.28982	12.56076	13.59488	25.00273	23.34575	8.646293	19.27608	24.84211	15.42127	15.82225	27.38416	8.244518	14.36927	32.7648	9.859387
AC091151.	0	0	0	0	1.107485	0	0	0	0	0.960699	3.614265	1.840156	1.028084	0	0	0	0	0	0
AC091152.	11.53089	11.00429	13.98787	29.1002	4.42994	28.54718	14.44456	0	32.68406	36.50657	14.45706	10.12086	19.5336	7.445762	18.58211	6.595615	8.21101	21.8432	9.859387
AC091152.	44.20175	52.0203	41.96361	98.73281	52.05179	47.95925	73.0725	58.3397	5.836439	69.17035	30.11887	70.84602	72.99399	68.8733	40.09824	65.95615	45.16056	48.23707	44.8154
AC091152.	0	0	0	0	0	0	0	0	0	0	1.204755	0	2.056169	0	0	0	0	0	0
AC091152.	119.1525	92.03592	171.851	179.7976	64.23413	35.3985	175.0341	96.30681	70.03726	55.72056	15.66181	92.9279	110.005	106.1021	115.4047	98.93422	98.53212	100.1147	112.9348
AC091153.	20.17906	12.00469	16.98527	4.157171	8.859879	17.12831	20.39233	5.556162	40.85507	18.25329	40.96167	11.96102	16.44935	10.23792	17.6041	8.244518	19.50115	9.101334	11.652
AC091153.	8.648168	15.00586	4.995668	1.039293	11.07485	10.27698	3.398721	9.26027	21.01118	9.606993	22.89034	7.360626	12.33701	10.23792	15.64809	2.473355	0	3.640534	8.066772
AC091153.	5.765445	2.000781	10.99047	0	1.107485	0	9.346483	6.482189	3.501863	1.921399	16.86657	4.600391	7.196591	3.722881	5.868035	3.297807	3.079129	10.01147	1.792616
AC091153.	0	0	0	0	0	0	0	0	4.669151	0	0	1.840156	0	0	0	0.824452	1.026376	0	0.896308
AC091163.	0	1.00039	0	2.078586	0	0	1.69936	0	0	0	0	4.600391	1.028084	0	0	0	6.158258	3.640534	0.896308
AC091163.	0	0	0	0	0	0	0.84968	0	0	0	0	0	0	0	0.978006	0	0	0	0
AC091167.	50.9281	58.02265	181.8423	186.0334	143.973	39.96605	167.387	128.7178	18.6766	75.89524	68.67103	112.2495	158.325	51.18962	72.37243	215.1819	217.5918	174.7456	316.3967
AC091167.	0	1.00039	0.999134	0	0	0	1.69936	4.630135	3.501863	0	0	4.600391	4.112337	1.861441	1.956012	1.648904	0	0.910133	2.688924
AC091167.	0	1.00039	2.997401	0	0	0	0	0.926027	0	0.960699	2.40951	0	2.056169	0	0	1.648904	0	0	1.792616
AC091167.	74.95079	111.0433	51.95495	145.501	126.2533	166.7155	146.145	50.93149	44.35693	156.594	110.8374	95.68814	115.1454	118.2015	127.1408	96.46086	72.87271	91.01334	113.8311
AC091167.	4.804538	3.001171	9.991337	13.51081	13.28982	14.84453	18.69297	10.1863	0	12.48909	10.84279	11.04094	11.30893	3.722881	14.67009	10.71787	7.184634	10.01147	10.7557
AC091167.	4.804538	6.002343	0.999134	9.353635	9.967364	12.56076	7.647122	0	0	6.724895	18.07132	0.920078	3.084253	3.722881	11.73607	4.946711	19.50115	15.47227	7.170464
AC091167.	5.765445	7.002733	0.999134	5.196464	4.42994	0	0.84968	6.482189	2.334575	6.724895	0	3.680313	2.056169	0	0	6.595615	4.105505	0.910133	6.274156
AC091170.	0	0	0	1.039293	0	0	0	3.704108	0	0	0	0	0	0	0	0	0	0	0
AC091173.	4.804538	5.001952	0	0	5.537425	0	7.647122	0	0	3.842797	0	2.760235	2.056169	0	0	0	7.184634	13.652	0
AC091175.	0.960908	0	4.995668	3.117878	6.64491	3.425661	1.69936	2.778081	0	5.764196	0	0.920078	2.056169	0	5.868035	0	0	0.910133	0.896308
AC091178.	135.488	72.02811	124.8917	96.65423	90.81376	34.25661	104.5107	158.3506	71.20455	109.5197	67.46627	153.6531	130.5667	137.7466	181.9091	94.81196	162.1674	144.7112	116.52
AC091178.	0	0	0	0	2.21497	0	0	0	0	0	0	0	0	0	0	0	0	0	0
AC091179.	88.40349	73.0285	56.95062	48.84676	108.5335	97.0604	67.12474	83.34243	26.84762	58.60265	66.26152	85.56728	52.4323	83.76483	84.1085	40.39814	49.26606	94.65388	113.8311
AC091179.	88.40349	70.02733	53.95322	47.80747	106.3186	95.91851	67.12474	73.15614	26.84762	58.60265	66.26152	84.6472	52.4323	82.83411	82.15249	39.57369	47.21331	94.65388	113.8311
AC091180.	0	0	2.997401	4.157171	6.64491	2.283774	0.84968	0	0	0	0	0	4.112337	0	5.868035	0	0	0	0
AC091180.	186.4161	252.0984	331.7124	151.7367	224.8194	206.6815	280.3945	213.9122	91.04844	136.4193	107.2232	86.48735	145.988	274.5625	280.6877	145.1035	138.5608	143.8011	196.2914
AC091180.	67.26353	184.0718	207.8198	65.47544	139.5431	166.7155	94.31451	158.3506	130.7362	140.2621	101.1994	46.00391	34.95487	132.1623	166.261	64.30724	73.89909	49.1472	75.28987
AC091180.	97.05166	177.0691	321.721	82.10413	150.6179	154.1547	124.0533	133.3479	102.7213	113.3625	118.066	61.64524	56.54464	169.3911	266.0176	63.48279	83.13648	73.72081	102.1791

AC091181.	50.9281	63.0246	62.94542	47.80747	81.95388	51.38492	73.0725	81.49038	82.87743	38.42797	102.4042	40.48344	51.40422	77.24979	96.82258	65.13169	55.42432	40.956	85.14926
AC091182.	13.45271	47.01835	113.9012	7.275049	42.08443	4.567548	0	53.70957	78.20828	7.685594	13.2523	14.72125	27.75828	35.36737	22.49413	33.80253	30.79129	10.01147	8.963079
AC091182.	46.12356	4.001562	0.999134	3.117878	0	0	37.38593	6.482189	1.167288	9.606993	0	23.00196	20.56169	12.09936	12.71408	11.54233	30.79129	27.304	24.20031
AC091185.	552.5218	456.178	688.4031	367.9096	518.3029	422.4982	466.4745	609.3258	157.5838	569.6947	538.5254	501.4426	519.1826	398.3483	431.3006	245.6866	275.0688	346.7608	300.2632
AC091198.	0	0	0	0	0	0	0	1.852054	0	0	0	5.520469	2.056169	0	0.978006	0	6.158258	3.640534	0.896308
AC091212.	8.648168	7.002733	14.987	14.5501	1.107485	12.56076	4.248401	5.556162	1.167288	1.921399	4.81902	23.92203	20.56169	20.47585	12.71408	2.473355	6.158258	22.75334	2.688924
AC091230.	38.4363	56.02186	39.96535	54.04322	32.11706	97.0604	54.37954	37.04108	105.0559	43.23147	96.38039	46.92399	31.87062	67.94258	104.6466	60.18498	81.08372	57.33841	112.0385
AC091231.	1.921815	0	0	1.039293	0	0	2.549041	0.926027	1.167288	0	0	0.920078	1.028084	0.93072	0.978006	3.297807	1.026376	3.640534	0
AC091271.	164.3152	138.0539	247.7851	177.7191	246.9691	176.9925	200.5245	147.2383	389.8741	244.9783	257.8175	200.5771	168.6058	191.7284	177.0191	149.2258	129.3234	141.9808	135.3425
AC091286.	0	0	0	1.039293	0	0	0.84968	0	0	0	0	0	0	7.445762	0	0.824452	0	0	0
AC091304.	0	1.00039	0	1.039293	0	0	1.69936	0	0	0	0	0	0	0	0	0	0	0	0.896308
AC091304.	1.921815	1.00039	0	0	0	3.425661	0.84968	3.704108	0	0	4.81902	0	2.056169	2.792161	1.956012	0.824452	2.052753	0	3.585232
AC091304.	2.882723	1.00039	2.997401	3.117878	0	3.425661	0	0	0	3.842797	0	0	9.252759	5.584322	1.956012	11.54233	0	0.910133	5.377848
AC091305.	27.86632	30.01171	18.98354	57.1611	27.68712	42.24982	29.73881	37.04108	7.003726	28.82098	50.5997	24.84211	39.06721	34.43665	23.47214	71.72731	34.89679	44.59654	51.98586
AC091390.	36.51449	37.01445	31.97228	13.51081	24.36467	9.135096	20.39233	30.55889	35.01863	54.75986	50.5997	31.28266	38.03912	35.36737	32.27419	25.55801	15.39564	28.21414	43.02278
AC091390.	0.960908	2.000781	0	0	8.859879	1.141887	0	0	5.836439	10.56769	4.81902	1.840156	8.224675	9.307203	1.956012	0	4.105505	1.820267	0
AC091390.	92.24712	108.0422	182.8415	84.18271	84.16885	68.51322	158.8902	112.0493	96.88488	110.4804	110.8374	126.9708	110.005	130.3008	121.2727	77.49847	89.29473	91.92348	96.80126
AC091390.	13.45271	13.00508	22.98007	10.39293	8.859879	6.851322	14.44456	10.1863	22.17847	20.17468	12.04755	7.360626	11.30893	12.09936	14.67009	7.420066	9.237386	11.83173	12.54831
AC091393.	0	0	0	0	0	0	0	0	0	0	0	0	4.112337	0	0	0	0	0	0
AC091429.	3.84363	5.001952	0.999134	0	3.322455	5.709435	3.398721	1.852054	4.669151	5.764196	3.614265	3.680313	2.056169	4.653602	7.824047	2.473355	4.105505	1.820267	5.377848
AC091435.	0	1.00039	2.997401	0	3.322455	2.283774	0	0	0	0	0	0	7.196591	0.93072	0	0	2.052753	0.910133	0.896308
AC091435.	26.90541	18.00703	60.94715	17.66798	44.2994	78.7902	21.24201	35.18903	16.34203	22.09608	24.0951	27.60235	38.03912	29.78305	35.20821	43.69595	17.4484	34.58507	23.30401
AC091488.	18.25724	18.00703	12.98874	6.235757	33.22455	7.993209	15.29424	12.03835	50.19337	20.17468	19.27608	23.92203	12.33701	4.653602	4.890029	6.595615	16.42202	16.3824	16.13354
AC091492.	8.648168	1.00039	8.992203	2.078586	0	1.141887	4.248401	4.630135	2.334575	0	1.204755	2.760235	0	0.93072	0.978006	2.473355	2.052753	2.7304	0.896308
AC091516.	18.25724	39.01523	42.96275	55.08252	31.00958	18.27019	39.93497	32.41095	0	39.38867	12.04755	46.00391	28.78636	18.61441	45.96627	34.62698	27.71216	15.47227	34.0597
AC091534.	1.921815	18.00703	0.999134	5.196464	0	1.141887	16.14392	2.778081	2.334575	2.882098	0	2.760235	3.084253	1.861441	5.868035	4.946711	4.105505	10.01147	4.48154
AC091544.	0.960908	24.00937	18.98354	0	0	7.993209	1.69936	6.482189	0	7.685594	4.81902	15.64133	4.112337	4.653602	9.780058	2.473355	5.131881	2.7304	7.170464
AC091544.	35.55358	28.01093	38.96621	71.7112	26.57964	17.12831	23.79105	25.92876	0	28.82098	7.228529	46.00391	34.95487	40.02097	42.05425	35.45143	31.81766	35.4952	38.54124
AC091544.	55.73264	70.02733	76.93329	24.94303	29.90209	41.10793	32.28785	32.41095	80.54285	52.83846	112.0422	41.40352	63.74123	79.11123	38.14223	27.20691	65.68808	57.33841	56.4674
AC091544.	0.960908	2.000781	0.999134	2.078586	4.42994	0	3.398721	3.704108	0	3.842797	0	5.520469	5.140422	0	9.780058	2.473355	5.131881	0.910133	3.585232
AC091544.	110.5044	134.0523	81.92896	117.4401	89.70628	75.36454	88.36674	43.52327	156.4166	103.7555	106.0184	58.88501	60.65698	81.90339	54.76833	65.13169	47.21331	54.608	43.02278
AC091544.	24.02269	16.00625	11.9896	40.53242	8.859879	2.283774	10.19616	14.81643	14.00745	10.56769	19.27608	11.96102	3.084253	10.23792	17.6041	10.71787	9.237386	10.9216	30.47447
AC091544.	0	0	4.995668	0	0	0	0	0.926027	0	0	1.204755	0.920078	1.028084	0	0	0.824452	0	0.910133	0
AC091563.	558.2873	412.1609	431.6257	719.1906	378.7598	416.7888	558.2399	561.1724	257.9706	391.0046	378.293	432.4368	433.8516	552.8479	556.4853	411.4015	322.2821	377.7054	259.9293
AC091564.	22.10087	13.00508	6.993936	16.62868	8.859879	17.12831	11.04584	21.29862	21.01118	7.685594	8.433284	10.12086	15.42127	5.584322	22.49413	10.71787	8.21101	9.101334	28.68185
AC091564.	3.84363	3.001171	0	2.078586	0	2.283774	4.248401	0	1.167288	0	7.228529	2.760235	0	0	0	0.824452	0	6.370934	0.896308
AC091564.	0	0	3.996535	1.039293	4.42994	4.567548	1.69936	0	0	0	2.40951	0.920078	1.028084	0	4.890029	5.771163	0	3.640534	0
AC091564.	0	0	0	0	0	0	0.84968	2.778081	2.334575	4.803496	0	0	2.056169	0	1.956012	4.122259	3.079129	3.640534	11.652
AC091588.	0	0	0	0	0	0	0.84968	5.556162	0	0	0	0.920078	0	0.93072	0	0	4.105505	0	0.896308
AC091588.	0	0	0	0	0	0	0	0	0	0	0	0	0	0	0	0	0	2.7304	0
AC091588.	7.68726	2.000781	0.999134	0	3.322455	6.851322	0	0	0	6.724895	0	0	1.028084	0	0	1.648904	0	3.640534	2.688924
AC091607.	3.84363	0	0	0	0	0	0	0	11.67288	0	0	0	0	0	0.978006	0.824452	0	0	0
AC091614.	0.960908	1.00039	4.995668	3.117878	1.107485	4.567548	5.098081	0.926027	14.00745	1.921399	7.228529	5.520469	5.140422	4.653602	1.956012	5.771163	1.026376	4.550667	3.585232
AC091675.	0	0	0	0	0	0	0	0	0	0	3.614265	0	0	0	0	0	0	0	0
AC091685.	0	0	4.995668	0	0	2.283774	0	0.926027	1.167288	0	0	0	8.224675	0.93072	0	0	0	0	0
AC091705.	0	0	0	0	0	0	0	0	0	0	0	0	0	0	3.912023	0	1.026376	0	0
AC091729.	4.804538	2.000781	4.995668	4.157171	12.18233	3.425661	1.69936	3.704108	1.167288	3.842797	10.84279	1.840156	1.028084	7.445762	17.6041	4.122259	8.21101	7.281067	0.896308
AC091729.	0	0	0	0	0	0	0	3.704108	0	1.921399	1.204755	0	0	0	0.978006	0	1.026376	0.910133	0.896308
AC091729.	1070.451	562.2194	1193.965	1107.886	761.9496	528.6937	813.9937	475.9779	1148.611	1098.079	1216.802	633.9339	696.0131	651.5042	768.7126	1053.649	1005.849	1381.583	1524.62
AC091768.	20.17906	15.00586	53.95322	8.314342	13.28982	15.98642	10.19616	9.26027	11.67288	5.764196	3.614265	23.92203	27.75828	24.19873	31.29619	8.244518	15.39564	3.640534	13.44462
AC091769.	0	0	0	1.039293	0	1.141887	3.398721	0	0	0	0	0	1.028084	0	0	0	0	0	0
AC091769.	1.921815	0	3.996535	0	1.107485	0	2.549041	4.630135	0	0.960699	0	2.760235	3.084253	0.93072	0	0.824452	0	1.820267	0.896308
AC091799.	0	2.000781	2.997401	9.353635	0	0	5.947762	7.408216	1.167288	14.41049	3.614265	1.840156	2.056169	2.792161	3.912023	0	4.105505	1.820267	4.48154
AC091804.	0	4.001562	0	1.039293	0	0	0	3.704108	0	0.960699	4.81902	0	0	2.792161	2.934017	0	1.026376	2.7304	1.792616
AC091805.	0	5.001952	0.999134	0	7.752394	0	1.69936	1.852054	0	1.921399	4.81902	11.04094	4.112337	4.653602	1.956012	0.824452	0	0	0.896308
AC091806.	196.0251	61.02382	161.8597	39.49313	83.06137	21.69585	89.21643	82.41641	120.2306	77.81664	54.21397	112.2495	118.2297	70.73474	76.28445	23.08465	19.50115	77.36134	43.02278
AC091807.	0	0	0	0	0	0	0	0	0	0.960699	0	0	0	0	0	0	0	0.910133	1.792616
AC091812.	0	0	0	0	0	0	0	0	0	0	0	0	0	0	0	0	0	0	3.585232
AC091812.	1.921815	0	1.998267	0	0	1.141887	0	2.778081	0	0.960699	3.614265	0.920078	0	3.722881	0.978006	0	0	0	0
AC091812.	1.921815	0	2.997401	0	1.107485	1.141887	0	0.926027	0	0.960699	3.614265	0.920078	0	0	0.978006	0	0	0	0
AC091814.	2.882723	2.000781	11.9896	7.275049	0	0	1.69936	4.630135	0	0	0	3.680313	1.028084	0	2.934017	9.06897	6.158258	0.910133	9.859387
AC091819.	0	0	0	0	0	0	0	0	0	0	0	0	4.112337	0	0	0	0	0	0

AC091820.	0	0	0.999134	0	0	0	0.84968	0	1.167288	0	0	0	0	0	0	0	1.026376	0	4.48154
AC091820.	0	0	0	0	0	0	0	0	0	3.842797	0	0	0	0	0	0	1.026376	0	0
AC091825.	3729.282	3638.42	2463.864	5090.456	2989.102	5581.544	3634.932	2765.117	3037.283	3808.212	3369.699	3306.761	3402.959	4076.555	4117.405	4171.726	4453.447	3144.511	4169.625
AC091826.	0	0	0.999134	0	0	0	0	0	0	10.56769	0	0	0	0	0	0	0	0.910133	0
AC091826.	11.53089	16.00625	20.98181	3.117878	15.50479	13.70264	16.14392	22.22465	29.18219	10.56769	2.40951	6.440548	9.252759	1.861441	12.71408	7.420066	16.42202	23.66347	15.23724
AC091839.	4.804538	2.000781	3.996535	12.47151	4.42994	0	5.947762	2.778081	4.669151	5.764196	6.023774	6.440548	8.224675	2.792161	0.978006	1.648904	7.184634	11.83173	1.792616
AC091849.	40.35812	41.01601	73.93589	41.57171	43.19191	85.64153	65.42538	50.00546	46.69151	22.09608	15.66181	119.6102	136.7352	64.2197	56.72434	70.90286	55.42432	30.0344	34.0597
AC091849.	74.95079	25.00976	38.96621	21.82515	32.11706	12.56076	59.47762	35.18903	35.01863	11.52839	25.29985	40.48344	42.15146	26.06017	13.69208	46.99375	31.81766	45.50667	43.02278
AC091860.	2.882723	3.001171	18.98354	17.66798	15.50479	1.141887	16.14392	1.852054	0	0	4.81902	2.760235	10.28084	7.445762	1.956012	7.420066	1.026376	1.820267	1.792616
AC091862.	0	1.00039	0.999134	2.078586	0	0	0	0	0	0	0	0	3.084253	0	0	0	5.131881	0.910133	1.792616
AC091868.	4.804538	7.002733	9.991337	3.117878	15.50479	2.283774	10.19616	18.52054	21.01118	7.685594	21.68559	11.04094	13.3651	5.584322	2.934017	14.01568	13.34289	2.7304	12.54831
AC091868.	13.45271	37.01445	68.94022	13.51081	45.40688	30.83095	22.94137	60.19176	0	49.95636	71.08054	34.96297	20.56169	13.9608	32.27419	1.648904	0	10.01147	8.963079
AC091874.	0	12.00469	0	2.078586	0	1.141887	0	0.926027	1.167288	0	0	5.520469	2.056169	2.792161	6.846041	0	0	0	3.585232
AC091885.	9.609075	0	0	0	0	1.141887	0	0.926027	0	0	0	0	0	0	0	0	0	0	0
AC091885.	0.960908	0	5.994802	0	0	0	0.84968	0	0	0	0	0.920078	0	0	0	0.824452	0	0.910133	0
AC091887.	19.21815	20.00781	43.96188	21.82515	23.25718	43.39171	28.88913	11.11232	8.171014	21.13538	27.70936	25.76219	27.75828	22.33729	15.64809	20.6113	31.81766	32.7648	28.68185
AC091901.	7.68726	6.002343	0.999134	7.275049	14.3973	4.567548	14.44456	0.926027	4.669151	5.764196	12.04755	6.440548	13.3651	15.82225	10.75806	8.244518	5.131881	13.652	5.377848
AC091905.	0	0	0	0	0	0	0	0	0	0	0	0	0	0	0	0	0	3.640534	0
AC091905.	0	0	3.996535	4.157171	1.107485	1.141887	5.098081	0	0	0	0	1.840156	1.028084	0	0	0.824452	0	0	0
AC091905.	0	1.00039	0	3.117878	0	0	6.797442	0	0	0	2.40951	0	0	0	0	0.824452	6.158258	0	1.792616
AC091906.	0	3.001171	0.999134	0	0	1.141887	1.69936	0.926027	0	2.882098	0	1.840156	1.028084	5.584322	0	0	1.026376	0.910133	0
AC091917.	0	0	0	0	0	0	0	0	0	0	0	0	1.028084	0	0	0	1.026376	0	0
AC091917.	0	0	0	0	0	0	0	0	0	0	0	0	0	0	0.978006	0	0	0	1.792616
AC091925.	22.10087	21.0082	33.97054	11.43222	0	1.141887	18.69297	16.66849	32.68406	24.01748	7.228529	11.04094	12.33701	14.89152	8.802052	20.6113	9.237386	13.652	12.54831
AC091925.	0	0	0	2.078586	2.21497	0	1.69936	0	0	0	0	0.920078	1.028084	0.93072	4.890029	3.297807	0	0	0
AC091931.	0	0	0.999134	0	9.967364	2.283774	0	0	0	0	0	0.920078	0	12.09936	1.956012	0	1.026376	0	0
AC091932.	10.56998	1.00039	4.995668	0	25.47215	17.12831	2.549041	16.66849	82.87743	39.38867	118.066	16.56141	3.084253	1.861441	4.890029	10.71787	9.237386	7.281067	0.896308
AC091932.	0	0	0	0	1.107485	0	0	0	0	0	0	0	1.028084	0	0	0	0	0	0
AC091932.	0	0	0	0	0	0	0	0	0	0	9.638039	0	0	0	0	0	0	0	0
AC091939.	0	0	2.997401	1.039293	0	0	0	0	0	0	0	0	0	0	0.978006	0	1.026376	0.910133	0
AC091940.	280.585	221.0863	592.4863	139.2652	377.6524	179.2763	328.8263	515.7971	124.8998	275.7207	266.2508	490.4017	492.4524	238.2644	320.7859	61.83389	60.5562	170.1949	291.3001
AC091946.	12.4918	17.00664	52.95408	17.66798	42.08443	22.83774	37.38593	45.37532	22.17847	24.01748	44.57593	2.760235	10.28084	16.75297	21.51613	7.420066	2.052753	20.93307	20.61508
AC091946.	12.4918	7.002733	53.95322	25.98232	16.61227	15.98642	8.496802	7.408216	23.34575	14.41049	10.84279	26.68227	12.33701	36.29809	13.69208	2.473355	2.052753	9.101334	6.274156
AC091948.	256.5623	278.1085	356.6907	197.4656	241.4317	147.3034	313.532	298.1807	183.2642	167.1617	180.7132	216.2184	241.5998	241.9873	196.5792	202.8152	166.273	223.8928	221.3881
AC091948.	0	0	0.999134	0	0	0	0.84968	0	4.669151	1.921399	0	0.920078	0	0	0	0	0	0	0
AC091951.	0	7.002733	0	23.90373	1.107485	14.84453	16.9936	7.408216	2.334575	7.685594	0	11.04094	12.33701	6.515042	10.75806	11.54233	19.50115	0.910133	7.170464
AC091951.	0.960908	11.00429	0	2.078586	27.68712	1.141887	5.947762	0.926027	3.501863	9.606993	9.638039	2.760235	5.140422	4.653602	5.868035	17.31349	7.184634	5.4608	8.066772
AC091951.	0	0	0	0	0	0	0	0	0	0	0	0	0	0	1.956012	0	0	0	0
AC091959.	0.960908	3.001171	11.9896	7.275049	7.752394	7.993209	5.098081	1.852054	0	2.882098	0	6.440548	8.224675	0	6.846041	9.893422	0	9.101334	11.652
AC091959.	0.960908	2.000781	4.995668	5.196464	1.107485	3.425661	2.549041	0	0	18.25329	6.023774	12.8811	28.78636	1.861441	2.934017	3.297807	4.105505	6.370934	3.585232
AC091959.	1.921815	2.000781	0	0	0	1.141887	3.398721	0.926027	0	0.960699	0	4.600391	2.056169	1.861441	1.956012	1.648904	0	3.640534	0
AC091962.	0	0	0	0	0	0	0	0	7.003726	0	0	0	0	0	0	6.595615	0	0	0
AC091965.	28.82723	28.01093	37.96708	27.02161	47.62185	11.41887	22.94137	46.30135	10.50559	22.09608	16.86657	25.76219	28.78636	26.99089	24.45015	22.2602	14.36927	10.01147	16.13354
AC091965.	0	2.000781	2.997401	0	19.93473	2.283774	0	0.926027	0	0.960699	1.204755	0.920078	0	0	0	0.824452	1.026376	0	0
AC091965.	0	0	0	0	0	0	0	0	0	0	0	0	0	0	0	0	2.052753	0	0
AC091965.	21.13997	43.01679	30.97314	9.353635	67.55658	33.11472	20.39233	28.70684	43.18965	33.62447	46.98544	3.680313	5.140422	17.68369	32.27419	15.66458	8.21101	7.281067	16.13354
AC091965.	5.765445	12.00469	4.995668	6.235757	16.61227	18.27019	11.04584	5.556162	0	0	12.04755	6.440548	12.33701	4.653602	8.802052	14.01568	4.105505	5.4608	14.34093
AC091976.	1.921815	9.003514	3.996535	3.117878	16.61227	12.56076	12.7452	10.1863	0	7.685594	2.40951	29.4425	19.5336	21.40657	25.42815	31.32917	20.52753	32.7648	23.30401
AC091979.	0.960908	4.001562	3.996535	9.353635	0	13.70264	11.04584	12.96438	1.167288	3.842797	2.40951	4.600391	1.028084	0.93072	0.978006	10.71787	5.131881	5.4608	12.54831
AC091979.	0.960908	0	0.999134	1.039293	0	0	0.84968	0	0	0	0	0	0	0	0.978006	0	0	0.910133	0.896308
AC091980.	436.252	654.2553	478.585	374.1454	227.0344	438.4846	583.7303	529.6875	156.4166	370.8299	268.6603	413.1151	385.5316	619.8597	488.0249	430.3639	450.5792	443.235	595.1485
AC091982.	147.0189	155.0605	196.8293	84.18271	148.403	147.3034	58.62794	107.4191	296.4911	179.6508	246.9747	125.1306	99.72418	156.361	127.1408	84.91854	86.21561	131.9693	108.4533
AC091982.	83.59896	24.00937	78.93156	72.75049	47.62185	58.23624	38.23561	52.78354	23.34575	77.81664	46.98544	49.68422	62.71315	81.90339	78.24047	46.99375	25.65941	50.05734	82.46033
AC091982.	4.804538	1.00039	3.996535	2.078586	2.21497	2.283774	5.098081	0	0	0.960699	12.04755	1.840156	2.056169	3.722881	0.978006	1.648904	0	8.191201	0.896308
AC091982.	301.725	287.1121	279.7574	277.4912	235.8943	302.6001	122.354	232.4328	203.1081	251.7032	249.3843	268.6628	311.5096	263.3938	297.3138	206.9374	212.4599	313.0859	338.8044
AC091987.	0.960908	0	0	0	0	0	1.69936	0	0	0	0	0	0	3.722881	1.956012	0	0	0	0
AC091996.	0	0	0	0	5.537425	0	5.098081	1.852054	0	0.960699	0	4.600391	1.028084	0.93072	0	0	0	0.910133	0
AC092001.	0.960908	0	0	0	0	0	0	0	0	0	0	0	0	0	0	0	0	0.910133	0
AC092013.	0	0	0	0	0	0	0	3.704108	0	0	0	0.920078	0	0	0	0	0	0	0
AC092017.	1.921815	0	0	1.039293	1.107485	0	0	0	1.167288	0	2.40951	0	0	0	0.978006	1.648904	0	0	0.896308
AC092017.	0	0	0	0	0	0	0	0	2.334575	2.882098	2.40951	1.840156	0	0	0	0.824452	3.079129	2.7304	0
AC092017.	4.804538	4.001562	0	1.039293	1.107485	0	4.248401	0.926027	1.167288	1.921399	1.204755	1.840156	0	0	0.978006	3.297807	4.105505	0	0.896308

AC092028.	1.921815	7.002733	0	1.039293	1.107485	5.709435	4.248401	24.0767	4.669151	1.921399	0	0	4.112337	0	8.802052	0.824452	1.026376	13.652	11.652
AC092032.	16.33543	32.01249	10.99047	13.51081	19.93473	5.709435	32.28785	22.22465	7.003726	30.74238	21.68559	21.1618	16.44935	19.54513	7.824047	17.31349	14.36927	12.74187	9.859387
AC092042.	36.51449	42.0164	87.92376	84.18271	69.77155	76.50643	75.62154	50.00546	33.85134	92.22713	75.89956	44.16376	54.48847	31.64449	27.38416	38.74924	70.81996	33.67494	65.43048
AC092042.	0.960908	7.002733	7.993069	1.039293	4.42994	2.283774	5.947762	1.852054	4.669151	0	15.66181	0.920078	3.084253	0.93072	1.956012	2.473355	10.26376	0.910133	5.377848
AC092042.	390.1285	399.1558	512.5556	659.9509	732.0475	536.6869	730.725	553.7642	561.4654	609.0833	646.9534	495.0021	524.323	542.6099	472.3768	367.7055	447.5	653.4758	639.9639
AC092045.	25.9445	6.002343	29.97401	23.90373	26.57964	31.97284	71.37314	19.44657	57.1971	18.25329	7.228529	23.00196	34.95487	52.12034	16.6261	37.10033	6.158258	56.42827	17.92616
AC092053.	6.726353	1.00039	0	0	1.107485	2.283774	3.398721	3.704108	0	0.960699	0	6.440548	1.028084	0.93072	4.890029	0	0	6.370934	5.377848
AC092053.	641.8862	499.1948	269.7661	285.8055	511.658	192.9789	782.5555	442.6409	117.8961	169.0831	162.6419	341.349	903.6862	545.4021	632.7698	417.9971	320.2294	367.6939	444.5687
AC092066.	0	0	0	0	0	0	0	0	0	0	0	0	0	1.861441	0	0	0	0	0
AC092067.	1.921815	0	0	0	0	0	0	3.704108	0	0.960699	0	0.920078	0	3.722881	0	0	0	0	0
AC092068.	0.960908	0	10.99047	14.5501	0	0	3.398721	0	0	0.960699	8.433284	0.920078	2.056169	0.93072	2.934017	20.6113	20.52753	18.20267	17.92616
AC092068.	0.960908	0	1.998267	1.039293	11.07485	1.141887	3.398721	7.408216	2.334575	5.764196	8.433284	3.680313	12.33701	0.93072	9.780058	4.946711	0	6.370934	5.377848
AC092068.	2.882723	1.00039	0	9.353635	8.859879	2.283774	7.647122	0.926027	3.501863	19.21399	1.204755	2.760235	10.28084	3.722881	0	1.648904	2.052753	0	8.963079
AC092070.	2059.225	2251.879	3098.313	1658.711	1996.795	1666.013	2019.69	2042.816	929.161	1333.451	1179.455	2925.849	2787.137	1799.082	1890.485	4014.256	3414.754	2412.764	1923.477
AC092070.	0	0	0	1.039293	0	0	0.84968	0	1.167288	0	0	3.680313	1.028084	0	0	0	5.131881	0.910133	0
AC092071.	2.882723	4.001562	3.996535	2.078586	8.859879	10.27698	4.248401	9.26027	7.003726	5.764196	0	4.600391	0	9.307203	0	5.771163	5.131881	4.550667	3.585232
AC092078.	0	0	0	0	0	0	1.69936	0	0	0	0	1.840156	0	0	0	0	1.026376	0	0
AC092078.	0	0	2.997401	0	0	0	0	0	0	0	0	0	0	0	0	0	0	0.910133	0
AC092100.	0	0	0	0	1.107485	0	0	0	0	0	0	0.920078	1.028084	0.93072	0	0.824452	0	0	0
AC092106.	0	0	0	0	1.107485	0	1.69936	0	0	0	0	0	1.028084	0	0.978006	0	0	0	0
AC092106.	4.804538	0	0.999134	0	1.107485	5.709435	0	1.852054	11.67288	0.960699	2.40951	4.600391	4.112337	1.861441	15.64809	3.297807	1.026376	1.820267	8.963079
AC092106.	0	6.002343	0	0	4.42994	0	5.098081	0	0	0	1.204755	1.840156	2.056169	2.792161	3.912023	1.648904	0	0.910133	0.896308
AC092111.	741.8206	1132.442	1734.496	827.277	967.9418	1082.509	1095.238	887.1339	2104.62	887.6861	1056.57	1025.887	1146.314	997.7322	1053.312	897.828	890.8946	930.1564	909.7526
AC092111.	70.14625	43.01679	88.9229	44.68959	54.26676	37.68227	79.02026	100.0109	28.01491	18.25329	39.75691	97.52829	111.0331	49.32818	48.90029	40.39814	70.81996	26.39387	60.05263
AC092112.	50.9281	45.01757	62.94542	47.80747	38.76197	15.98642	6.797442	32.41095	37.35321	27.86028	54.21397	32.20274	44.20763	35.36737	36.18622	28.85581	32.84404	33.67494	34.95601
AC092114.	0.960908	19.00742	1.998267	6.235757	0	3.425661	3.398721	2.778081	0	0	0	6.440548	2.056169	10.23792	4.890029	17.31349	21.5539	10.01147	8.066772
AC092115.	23.06178	9.003514	15.98614	15.58939	13.28982	12.56076	18.69297	26.85478	8.171014	10.56769	21.68559	19.32164	9.252759	6.515042	24.45015	37.92478	27.71216	18.20267	18.82247
AC092115.	19.21815	21.0082	20.98181	7.275049	15.50479	26.2634	16.14392	25.00273	57.1971	28.82098	16.86657	26.68227	27.75828	16.75297	40.09824	23.08465	41.05505	12.74187	20.61508
AC092117.	269.0541	216.0843	483.5807	201.6228	173.8751	162.148	217.5181	50.00546	261.4725	103.7555	143.3658	325.7077	344.4083	242.918	218.0953	131.9123	140.6135	243.0056	229.4548
AC092117.	26.90541	25.00976	33.97054	48.84676	52.05179	10.27698	56.92858	49.07943	7.003726	21.13538	19.27608	84.6472	67.85357	29.78305	18.58211	52.76492	42.08143	49.1472	18.82247
AC092118.	0.960908	2.000781	3.996535	1.039293	7.752394	9.135096	5.947762	3.704108	0	5.764196	0	0.920078	4.112337	5.584322	6.846041	17.31349	14.36927	4.550667	19.71877
AC092118.	19.21815	10.0039	10.99047	19.74656	34.33203	6.851322	8.496802	17.59451	21.01118	17.29259	22.89034	14.72125	8.224675	26.99089	22.49413	14.01568	18.47477	26.39387	17.92616
AC092119.	3.84363	0	6.993936	8.314342	4.42994	0	0.84968	0	2.334575	0	0	1.840156	0	3.722881	4.890029	3.297807	10.26376	0	0.896308
AC092119.	317.0995	519.2026	706.3875	247.3517	471.7886	432.7752	429.0885	593.5833	812.4323	704.1926	971.0324	535.4855	399.9248	445.815	369.6862	300.9249	339.7305	479.6403	401.546
AC092119.	10.56998	20.00781	6.993936	11.43222	3.322455	2.283774	10.19616	15.74246	36.18592	4.803496	15.66181	14.72125	16.44935	10.23792	22.49413	14.01568	12.31652	10.9216	9.859387
AC092120.	9.609075	3.001171	0.999134	10.39293	0	9.135096	2.549041	2.778081	5.836439	10.56769	2.40951	4.600391	8.224675	3.722881	3.912023	10.71787	9.237386	5.4608	0.896308
AC092120.	0	5.001952	0	0	9.967364	0	3.398721	1.852054	0	0	7.228529	1.840156	6.168506	3.722881	0.978006	4.122259	2.052753	0	1.792616
AC092120.	0.960908	0	7.993069	6.235757	6.64491	0	0	0	0	1.167288	9.606993	1.204755	0	1.028084	3.722881	0.978006	4.122259	4.105505	0
AC092123.	187.377	171.0668	284.7531	178.7584	119.6084	223.8099	224.3156	264.8437	63.03354	173.8866	139.7516	274.1833	280.667	209.4121	213.2053	164.8904	140.6135	240.2752	202.5656
AC092123.	1134.832	334.1304	1339.838	2162.768	1157.322	962.6107	988.1781	857.501	337.3462	852.1402	813.2095	1614.737	1671.665	964.2262	1006.368	1567.283	1547.775	1018.439	880.1744
AC092125.	0.960908	1.00039	7.993069	13.51081	0	0	4.248401	0	0	0	0	0	10.28084	0.93072	1.956012	1.648904	0	5.4608	0
AC092127.	6.726353	1.00039	3.996535	0	4.42994	2.283774	4.248401	3.704108	5.836439	0.960699	19.27608	3.680313	15.42127	4.653602	1.956012	14.01568	16.42202	6.370934	5.377848
AC092127.	14.41361	4.001562	5.994802	10.39293	6.64491	0	3.398721	6.482189	7.003726	3.842797	1.204755	6.440548	17.47743	10.23792	1.956012	6.595615	6.158258	7.281067	7.170464
AC092128.	0.960908	4.001562	0.999134	1.039293	0	2.283774	4.248401	0	1.167288	0	1.204755	10.12086	4.112337	0	3.912023	0	4.105505	0.910133	3.585232
AC092131.	389.1675	117.0457	555.5183	70.67191	548.205	68.51322	98.56291	434.3067	37.35321	91.26643	98.7899	69.92595	97.66802	408.5862	488.0249	972.0287	681.5138	719.9155	732.2836
AC092134.	29.78813	39.01523	54.95235	42.611	32.11706	53.66869	47.58209	25.92876	93.38302	73.01314	114.4517	22.08188	22.61786	40.95169	48.90029	14.01568	16.42202	26.39387	31.37078
AC092134.	0	8.003124	11.9896	0	1.107485	3.425661	5.098081	2.778081	0	0	0	2.760235	2.056169	3.722881	0	0	0	0	0.896308
AC092135.	6.726353	6.002343	5.994802	1.039293	5.537425	0	1.69936	3.704108	0	0	6.023774	4.600391	12.33701	9.307203	0	9.06897	6.158258	1.820267	14.34093
AC092135.	1.921815	8.003124	0	0	7.752394	0	0.84968	0	16.34203	0.960699	0	5.520469	4.112337	2.792161	0	0.824452	0	4.550667	1.792616
AC092135.	5.765445	2.000781	4.995668	8.314342	39.86946	6.851322	8.496802	2.778081	11.67288	5.764196	1.204755	11.96102	13.3651	13.9608	3.912023	12.36678	8.21101	33.67494	6.274156
AC092139.	0.960908	11.00429	0	5.196464	1.107485	4.567548	3.398721	6.482189	8.171014	0	1.204755	1.840156	3.084253	4.653602	4.890029	7.420066	2.052753	1.820267	4.48154
AC092139.	3.84363	2.000781	6.993936	0	3.322455	0	7.647122	5.556162	0	1.921399	1.204755	0	6.168506	0.93072	0.978006	0	1.026376	0.910133	6.274156
AC092139.	6.726353	8.003124	17.98441	20.78586	1.107485	3.425661	5.098081	0.926027	1.167288	6.724895	7.228529	28.52243	27.75828	10.23792	8.802052	14.01568	2.052753	7.281067	3.585232
AC092140.	0	0	0	0	0	0	0.84968	1.852054	0	0	1.204755	1.840156	0	0	0	0.824452	0	0	0
AC092140.	132.6052	183.0715	212.8155	162.1297	184.95	214.6748	174.1844	148.1643	466.9151	169.0831	145.7753	120.5302	133.651	148.9152	123.2287	71.72731	82.1101	80.09174	81.56402
AC092142.	0	1.00039	5.994802	0	0	1.141887	0	1.852054	7.003726	3.842797	1.204755	0.920078	0	0.93072	0	4.946711	1.026376	6.370934	5.377848
AC092142.	0	0	0	0	0	0	0	0	0	0	0	0	0	2.792161	0	2.473355	1.026376	0	0
AC092143.	0	0	0.999134	0	0	0	0	0	0	0	0	0	1.028084	0	0	0.824452	0	0	0
AC092143.	16.33543	15.00586	17.98441	16.62868	13.28982	19.41208	14.44456	14.81643	32.68406	23.05678	14.45706	23.92203	19.5336	29.78305	16.6261	13.19123	18.47477	23.66347	37.64493
AC092145.	23.06178	26.01015	4.995668	43.6503	43.19191	11.41887	57.77826	17.59451	7.003726	6.724895	24.0951	59.80509	67.85357	14.89152	44.01026	8.244518	16.42202	8.191201	20.61508
AC092155.	44.20175	32.01249	54.95235	27.02161	42.08443	19.41208	50.98081	46.30135	57.1971	33.62447	38.55216	33.12282	50.37613	66.08114	57.70234	48.64266	45.16056	56.42827	37.64493
AC092155.	10.56998	11.00429	0	3.117878	7.752394	13.70264	4.248401	4.630135	3.501863	5.764196	4.81902	6.440548	9.252759	9.307203	6.846041	5.771163	14.36927	4.550667	3.585232

AC092159.	0	1.00039	4.995668	0	6.64491	0	0.84968	3.704108	0	7.685594	1.204755	0.920078	2.056169	5.584322	0.978006	0.824452	0	0	0.896308
AC092159.	20.17906	17.00664	17.98441	29.1002	23.25718	19.41208	26.34009	9.26027	8.171014	23.05678	22.89034	11.96102	7.196591	11.16864	33.2522	29.68027	37.97592	14.56213	22.4077
AC092159.	0	3.001171	20.98181	0	2.21497	6.851322	0	0	0	0.960699	2.40951	0.920078	0	1.861441	4.890029	0	7.184634	10.01147	3.585232
AC092159.	10.56998	32.01249	0.999134	17.66798	0	0	0	1.852054	0	0	9.638039	0	2.056169	7.445762	8.802052	0.824452	7.184634	0.910133	5.377848
AC092162.	0.960908	6.002343	4.995668	3.117878	2.21497	19.41208	4.248401	27.78081	0	21.13538	44.57593	2.760235	11.30893	7.445762	0.978006	0	0	0.910133	1.792616
AC092162.	135.488	229.0894	147.8718	111.2043	122.9308	137.0264	123.2036	104.6411	99.21946	139.3014	149.3896	113.1696	80.19058	67.94258	93.88856	63.48279	63.63533	48.23707	90.5271
AC092162.	513.1246	1157.452	696.3962	902.1061	814.0014	931.7798	446.0821	525.9834	890.6405	988.5595	1178.25	322.0274	410.2057	231.7494	265.0396	306.6961	395.1549	220.2523	379.1383
AC092164.	351.6922	544.2124	465.5963	276.4519	419.7368	546.9639	355.1663	353.7423	457.5768	442.8824	451.7831	269.5829	279.6389	468.1523	506.607	444.3795	415.6824	345.8507	412.3017
AC092167.	0	0	1.998267	10.39293	9.967364	1.141887	0.84968	3.704108	0	0	2.40951	5.520469	4.112337	4.653602	1.956012	2.473355	1.026376	5.4608	0
AC092168.	0.960908	1.00039	3.996535	0	0	2.283774	3.398721	3.704108	0	0	0	0	3.084253	6.515042	0.978006	3.297807	0	8.191201	5.377848
AC092171.	33.63176	50.01952	42.96275	19.74656	56.48173	23.97963	48.43177	56.48765	39.68778	31.70308	28.91412	46.00391	35.98295	50.2589	26.40616	42.8715	33.87042	44.59654	38.54124
AC092171.	1303.952	984.3842	685.4057	1097.493	1232.631	1053.962	708.6333	872.3175	1610.857	1177.817	1837.251	1100.414	1095.938	1059.16	943.7756	1244.098	1446.164	1006.608	1235.112
AC092171.	18.25724	10.0039	1.998267	9.353635	11.07485	2.283774	38.23561	0	5.836439	21.13538	50.5997	29.4425	12.33701	13.9608	7.824047	2.473355	7.184634	11.83173	19.71877
AC092171.	94.16894	57.02225	66.94196	14.5501	87.49131	83.35775	37.38593	87.97257	218.2828	153.7119	261.4318	77.28657	80.19058	113.5479	79.21847	54.41382	50.29244	70.08027	74.39356
AC092171.	69.18534	71.02772	62.94542	50.92535	52.05179	59.37812	41.63433	68.526	89.88116	71.09174	114.4517	53.36454	102.8084	90.27987	69.43841	47.81821	68.76721	73.72081	106.6606
AC092175.	0	0	1.998267	0	0	0	0	0	0	0	6.023774	0	0	0	0	0	0	0	0
AC092183.	0.960908	0	0	0	0	0	0	0	0	0	0	0	0	0	0.978006	0	0	0	0
AC092184.	7.68726	0	4.995668	4.157171	17.71976	1.141887	5.098081	13.89041	25.68033	2.882098	1.204755	6.440548	3.084253	0.93072	7.824047	4.122259	8.21101	0	0.896308
AC092198.	9.609075	4.001562	15.98614	5.196464	2.21497	5.709435	9.346483	11.11232	7.003726	0.960699	4.81902	12.8811	5.140422	4.653602	9.780058	6.595615	9.237386	6.370934	11.652
AC092264.	1.921815	1.00039	0	0	0	0	0	0	0	0	0	0.920078	0	0	0.978006	0	2.052753	1.820267	0
AC092278.	2.882723	2.000781	7.993069	0	3.322455	1.141887	2.549041	8.334243	0	6.724895	8.433284	0.920078	8.224675	2.792161	2.934017	0	0	3.640534	0.896308
AC092279.	658.2217	609.2378	1142.01	755.5658	648.9862	721.6726	945.6941	778.7887	562.6327	1140.35	836.0999	599.891	667.2268	1025.654	1009.302	637.3013	626.0895	398.6384	449.0503
AC092279.	664.948	491.1917	755.345	402.2063	468.4661	483.0182	614.3188	464.8656	470.417	776.245	708.3959	437.9572	465.7222	512.8269	621.0337	469.9375	370.5218	470.539	451.7392
AC092287.	8.648168	7.002733	0	1.039293	0	4.567548	2.549041	6.482189	7.003726	6.724895	14.45706	11.04094	10.28084	4.653602	0	5.771163	10.26376	5.4608	11.652
AC092295.	26.90541	8.003124	16.98527	5.196464	13.28982	5.709435	6.797442	15.74246	2.334575	14.41049	3.614265	14.72125	15.42127	12.09936	17.6041	9.893422	17.4484	10.9216	8.963079
AC092295.	316.1386	195.0761	255.7782	280.609	200.4548	207.8234	241.3092	218.5424	47.8588	150.8298	206.0131	244.7408	214.8696	308.0684	176.041	338.0253	349.9943	211.151	209.7361
AC092296.	11.53089	7.002733	3.996535	18.70727	4.42994	15.98642	9.346483	10.1863	0	0.960699	4.81902	8.280704	22.61786	17.68369	21.51613	18.13794	9.237386	3.640534	9.859387
AC092296.	21.13997	34.01327	35.96881	19.74656	9.967364	22.83774	41.63433	34.263	31.51677	42.27077	38.55216	26.68227	35.98295	48.39746	56.72434	53.58937	49.26606	26.39387	37.64493
AC092296.	5.765445	6.002343	3.996535	3.117878	0	6.851322	2.549041	0	0	0	15.66181	1.840156	1.028084	0	7.824047	9.06897	0	1.820267	0.896308
AC092301.	58.61536	23.00898	59.94802	76.90766	66.4491	77.64832	67.97442	58.3397	63.03354	68.20965	101.1994	77.28657	91.49951	62.35826	61.61437	79.14738	68.76721	69.17014	59.15632
AC092316.	0	3.001171	1.998267	2.078586	1.107485	1.141887	6.797442	1.852054	0	0	1.204755	1.840156	4.112337	0.93072	1.956012	0.824452	1.026376	5.4608	3.585232
AC092326.	0	6.002343	1.998267	0	8.859879	0	0	0	0	2.882098	3.614265	4.600391	1.028084	2.792161	0	0	0	0	0.896308
AC092329.	0	1.00039	0	0	0	0	0	0	0	3.842797	0	0	0	0	0	0.824452	0	0.910133	5.377848
AC092329.	694.7361	682.2663	766.3355	798.1768	511.658	592.6394	791.0523	823.238	361.8592	537.0309	451.7831	798.6279	867.7032	702.6938	762.8445	843.4142	603.5092	563.3726	558.3999
AC092332.	0	0	8.992203	0	0	1.141887	0	1.852054	9.338302	3.842797	1.204755	1.840156	0	0	3.912023	0.824452	1.026376	4.550667	0
AC092335.	106.6607	124.0484	58.94889	171.4833	34.33203	73.08077	62.87634	59.26573	63.03354	127.773	84.33284	90.16767	105.8927	147.0538	94.86656	136.859	82.1101	47.32694	121.0016
AC092336.	0.960908	0	0.999134	0	0	2.283774	0	0	2.334575	5.764196	2.40951	0	0	3.722881	5.868035	0	0	1.820267	0
AC092338.	18.25724	14.00547	22.98007	21.82515	18.82724	18.27019	24.64073	11.11232	46.69151	25.93888	24.0951	36.80313	13.3651	16.75297	26.40616	38.74924	18.47477	16.3824	23.30401
AC092338.	10.56998	16.00625	4.995668	13.51081	4.42994	2.283774	13.59488	4.630135	7.003726	5.764196	10.84279	11.04094	3.084253	10.23792	10.75806	9.893422	4.105505	10.9216	16.13354
AC092343.	0.960908	0	0	0	0	1.141887	2.549041	2.778081	1.167288	0	8.433284	0	0	0	0.978006	1.648904	0	7.281067	18.82247
AC092354.	3.84363	11.00429	9.991337	8.314342	26.57964	1.141887	2.549041	13.89041	9.338302	20.17468	7.228529	5.520469	7.196591	6.515042	21.51613	9.06897	5.131881	5.4608	9.859387
AC092354.	43.24084	46.01796	42.96275	63.39686	37.65449	53.66869	41.63433	32.41095	53.69524	55.72056	28.91412	13.80117	47.29188	40.02097	49.8783	23.9091	52.34519	41.86614	21.51139
AC092364.	2.882723	1.00039	1.998267	1.039293	3.322455	9.135096	1.69936	0	1.167288	0	0	6.440548	1.028084	2.792161	0	4.122259	3.079129	6.370934	4.48154
AC092364.	14.41361	12.00469	22.98007	19.74656	13.28982	10.27698	43.33369	15.74246	4.669151	3.842797	20.48083	23.92203	25.70211	9.307203	21.51613	16.48904	16.42202	13.652	19.71877
AC092368.	116.2698	124.0484	79.93069	101.8507	178.3051	82.21586	84.11834	110.1972	208.9445	111.4411	274.6841	146.2924	125.4263	76.31906	88.99853	112.1254	159.0883	178.3861	150.5797
AC092373.	0	3.001171	0	2.078586	1.107485	0	0.84968	0	1.167288	0	1.204755	0	9.252759	0	0	0	4.105505	0	0
AC092375.	0	0	0.999134	1.039293	5.537425	1.141887	8.496802	3.704108	0	1.921399	0	0.920078	5.140422	1.861441	0.978006	0	1.026376	0	0.896308
AC092375.	540.9909	384.1499	524.5452	363.7525	541.5601	387.0997	135.0992	504.6847	617.4952	319.9129	287.9364	325.7077	336.1836	420.6856	391.2023	210.2352	139.5872	344.9406	250.9662
AC092375.	2.882723	2.000781	0	0	0	0	1.141887	1.69936	0	0	0	0	1.028084	0	0	0	0	0	0
AC092376.	19.21815	31.0121	22.98007	36.37525	11.07485	19.41208	45.03305	15.74246	7.003726	13.44979	8.433284	25.76219	45.23571	22.33729	25.42815	46.1693	22.58028	15.47227	24.20031
AC092376.	1901.636	2865.118	2081.195	2104.568	1177.256	1981.174	2554.988	1750.191	1413.585	1999.215	1546.905	1369.996	1563.716	2421.734	2115.427	2553.327	2388.378	1468.955	1644.725
AC092378.	13.45271	20.00781	21.98094	10.39293	22.1497	30.83095	14.44456	27.78081	0	4.803496	10.84279	12.8811	23.64594	37.22881	29.34017	23.08465	17.4484	2.7304	18.82247
AC092378.	8.648168	3.001171	2.997401	10.39293	0	5.709435	1.69936	0	0	0.960699	0	3.680313	4.112337	0.93072	9.780058	2.473355	5.131881	0	2.688924
AC092384.	24.02269	15.00586	3.996535	21.82515	66.4491	19.41208	11.89552	10.1863	7.003726	34.58517	19.27608	19.32164	18.50552	24.19873	18.58211	4.122259	11.29014	27.304	25.99293
AC092384.	14.41361	27.01054	8.992203	10.39293	66.4491	41.10793	13.59488	53.70957	17.50932	59.56335	49.39495	52.44446	27.75828	15.82225	25.42815	22.2602	46.18693	18.20267	43.91909
AC092384.	9.609075	6.002343	10.99047	11.43222	16.61227	6.851322	13.59488	11.11232	7.003726	12.48909	10.84279	11.04094	12.33701	18.61441	15.64809	4.946711	8.21101	10.9216	26.88924
AC092418.	0	0	0	0	0	0	0	0	0	0	1.204755	0	0	0	5.868035	3.297807	0	0	0
AC092422.	0	2.000781	3.996535	1.039293	0	1.141887	2.549041	0	0	0	0	0	8.224675	7.445762	2.934017	0	8.21101	0	0
AC092423.	4.804538	3.001171	0	0	0	4.567548	14.44456	7.408216	0	0	0	1.840156	1.028084	1.861441	2.934017	0	2.052753	0.910133	5.377848
AC092427.	0.960908	0	0.999134	1.039293	0	0	0	0	0	1.921399	7.228529	0.920078	1.028084	0	0	0.824452	1.026376	0	0
AC092431.	11.53089	18.00703	10.99047	19.74656	6.64491	13.70264	14.44456	13.89041	2.334575	13.44979	7.228529	22.08188	13.3651	3.722881	11.73607	11.54233	12.31652	54.608	25.99293
AC092431.	9.609075	0	3.996535	7.275049	6.64491	1.141887	2.549041	1.852054	7.003726	6.724895	4.81902	1.840156	8.224675	0.93072	0	0.824452	0	5.4608	0.896308

AC092435.	1.921815	7.002733	3.996535	10.39293	0	6.851322	13.59488	1.852054	1.167288	0	0	2.760235	8.224675	5.584322	0.978006	9.06897	10.26376	7.281067	7.170464
AC092435.	0.960908	0	0	0	0	1.141887	0	0	0	1.921399	0	0.920078	0	0	0	0	0	0.910133	0
AC092435.	0	0	0	5.196464	0	0	0	0	0	0	0	0	1.028084	0	0	3.297807	0	0	1.792616
AC092435.	0	0	0	1.039293	8.859879	6.851322	5.947762	3.704108	0	0	0	0.920078	0	0.93072	0.978006	0	2.052753	0	0
AC092436.	1.921815	0	0	0	0	0	0	0	0	0	2.40951	0	0	0	0	0	2.052753	0	0.896308
AC092436.	0	0	0	0	0	0	0	0	0	0	0	0	0	0	0	0	1.026376	0	0.896308
AC092436.	1.921815	7.002733	0.999134	2.078586	1.107485	2.283774	4.248401	0	0	11.52839	0	14.72125	11.30893	9.307203	6.846041	10.71787	7.184634	11.83173	2.688924
AC092437.	56.69354	75.02928	63.94455	50.92535	31.00958	37.68227	66.27506	68.526	29.18219	65.32755	39.75691	66.24563	62.71315	88.41843	45.96627	32.15362	59.52982	58.24854	18.82247
AC092440.	0	0	0	0	0	0	0	0	0	0	0	0	0	0	1.956012	0	0	0	0
AC092447.	0	0	0	0	0	0	0	0	0	0	0	0	0	2.792161	0	0	0	0	0
AC092447.	0	1.00039	3.996535	1.039293	0	1.141887	0.84968	1.852054	0	0	1.204755	1.840156	2.056169	2.792161	2.934017	0	0	0.910133	2.688924
AC092447.	26.90541	11.00429	0.999134	1.039293	12.18233	0	31.43817	7.408216	16.34203	3.842797	7.228529	31.28266	35.98295	9.307203	4.890029	1.648904	0	10.01147	10.7557
AC092447.	7.68726	23.00898	0.999134	8.314342	0	10.27698	186.9297	12.96438	14.00745	4.803496	0	13.80117	21.58977	11.16864	1.956012	8.244518	8.21101	13.652	17.92616
AC092451.	0	10.0039	0	0	0	0	0	0	0	0	0	0	1.028084	0	0.978006	0	0	0	0
AC092451.	0	0	0	0	0	0	0.84968	0	9.338302	0	0	0	0	0	0	0.824452	0	0	0
AC092468.	0	1.00039	0	7.275049	1.107485	0	0	0	1.167288	0.960699	1.204755	0.920078	3.084253	3.722881	2.934017	0.824452	0	0.910133	5.377848
AC092471.	9.609075	13.00508	12.98874	14.5501	16.61227	14.84453	20.39233	7.408216	5.836439	3.842797	14.45706	10.12086	24.67402	12.09936	3.912023	14.84013	9.237386	10.9216	16.13354
AC092471.	0.960908	0	0	0	0	0	0	0	0	0	1.204755	0	0	0	0	0	1.026376	3.640534	0
AC092474.	0	0	0	0	0	0	0	0	0	0	0	0	4.112337	0.93072	0	0	0	0	0.896308
AC092474.	1.921815	1.00039	3.996535	4.157171	3.322455	7.993209	0	0	1.167288	0	0	0.920078	3.084253	1.861441	0	4.122259	4.105505	0	0
AC092484.	12.4918	0	0	1.039293	2.21497	0	0	0	0	0	0	0	0	5.584322	14.67009	19.78684	0	9.101334	0
AC092490.	281.5459	158.0617	275.7609	447.9352	275.7637	191.837	194.5768	408.3779	227.6211	97.03062	140.9563	66.24563	106.9208	262.4631	165.283	186.3261	126.2443	111.0363	123.6905
AC092490.	8.648168	2.000781	6.993936	9.353635	2.21497	1.141887	5.098081	13.89041	2.334575	6.724895	0	0	0	0.93072	7.824047	0	0	0.910133	0.896308
AC092490.	0.960908	0	0	4.157171	0	0	0	0	1.167288	0	0	0.920078	0	0.93072	0	1.648904	0	0	0
AC092506.	62.45899	14.00547	0	0	57.58922	0	5.098081	0	0	49.95636	86.74235	1.840156	10.28084	0.93072	6.846041	2.473355	6.158258	0	7.170464
AC092535.	9.609075	5.001952	8.992203	4.157171	4.42994	7.993209	4.248401	8.334243	9.338302	4.803496	14.45706	11.96102	6.168506	13.03008	7.824047	1.648904	8.21101	7.281067	2.688924
AC092535.	17.29634	5.001952	28.97488	33.25737	25.47215	15.98642	21.24201	4.630135	12.84017	16.33189	4.81902	8.280704	7.196591	19.54513	20.53812	10.71787	14.36927	10.9216	10.7557
AC092535.	0	1.00039	0	0	0	0	1.69936	0.926027	0	0	0	1.840156	0	1.861441	0.978006	0	5.131881	0	0
AC092535.	71.10716	55.02147	68.94022	24.94303	89.70628	34.25661	65.42538	57.41368	219.4501	195.9826	355.4027	146.2924	102.8084	60.49682	65.52639	55.23827	61.58258	41.86614	34.95601
AC092542.	11.53089	25.00976	7.993069	4.157171	17.71976	2.283774	19.54265	12.96438	3.501863	27.86028	24.0951	8.280704	13.3651	19.54513	23.47214	9.06897	6.158258	7.281067	1.792616
AC092546.	0.960908	0	0.999134	0	0	5.709435	0	13.89041	0	0	0	0.920078	1.028084	0	0	0.824452	0	0	0
AC092552.	0	0	1.998267	0	1.107485	0	0	0	0	7.685594	0	0	0	7.445762	0	1.648904	0	0.910133	0.896308
AC092567.	1.921815	0	0	0	0	0	0.84968	0.926027	0	0	0	0	1.028084	0	0.978006	0	0	0	0
AC092569.	0	2.000781	0	1.039293	0	0	0	0	0	0	1.204755	0	0	0	0	0	0	0	0
AC092570.	2.882723	5.001952	0	3.117878	2.21497	0	0.84968	0	0	2.882098	0	0	1.028084	0	3.912023	0	0	0.910133	0.896308
AC092573.	2.882723	0	0.999134	0	0	0	0.84968	0.926027	0	0	0	0.920078	4.112337	6.515042	0	13.19123	5.131881	0.910133	0
AC092574.	52.84991	75.02928	84.92636	76.90766	109.641	93.63473	74.77186	77.78627	163.4203	87.42363	102.4042	58.88501	47.29188	65.15042	64.54838	65.13169	65.68808	60.97894	70.80833
AC092574.	54.77173	39.01523	28.97488	75.86837	31.00958	35.3985	69.67378	52.78354	1.167288	25.93888	24.0951	47.84407	40.09529	45.60529	44.01026	28.03136	62.60895	39.13574	40.33386
AC092580.	3.84363	0	0	0	16.61227	4.567548	0.84968	0	9.338302	9.606993	12.04755	3.680313	3.084253	0.93072	0.978006	0	0	0.910133	0.896308
AC092580.	0	0	0	0	0	0	0	0	0	0	0	3.680313	0	0	1.956012	0	4.105505	0	0
AC092587.	0.960908	5.001952	2.997401	0	3.322455	0	2.549041	3.704108	0	5.764196	7.228529	3.680313	5.140422	0	5.868035	4.946711	4.105505	6.370934	1.792616
AC092590.	0	2.000781	0	1.039293	0	0	0	0	0	0	0	0	0	0	3.912023	0	4.105505	0	0.896308
AC092597.	23.06178	27.01054	13.98787	9.353635	27.68712	11.41887	11.04584	11.11232	30.34948	20.17468	25.29985	26.68227	8.224675	21.40657	31.29619	31.32917	23.60665	17.29253	22.4077
AC092598.	2.882723	5.001952	8.992203	12.47151	11.07485	6.851322	7.647122	2.778081	7.003726	2.882098	6.023774	13.80117	9.252759	13.03008	17.6041	0.824452	3.079129	6.370934	6.274156
AC092608.	0	0	0	0	4.42994	0	0	0	0	0	0	0	0	0	0	0	0	0	0
AC092608.	0	1.00039	0	1.039293	0	4.567548	0.84968	0	0	0	0	0	0	0	0	2.473355	0	0	0
AC092610.	11.53089	13.00508	8.992203	13.51081	15.50479	18.27019	10.19616	6.482189	14.00745	23.05678	12.04755	11.04094	9.252759	15.82225	13.69208	18.96239	7.184634	11.83173	4.48154
AC092611.	170.0806	139.0543	216.812	113.2829	78.63143	78.7902	140.1972	121.3095	25.68033	96.06993	48.1902	129.731	154.2127	128.4394	139.8548	114.5988	74.92547	62.79921	109.3496
AC092611.	89.3644	55.02147	76.93329	65.47544	75.30897	37.68227	24.64073	33.33697	14.00745	30.74238	46.98544	49.68422	80.19058	56.77394	46.94428	20.6113	30.79129	50.05734	82.46033
AC092611.	131.6443	159.0621	164.8571	129.9116	153.9404	132.4589	49.28145	115.7534	182.0969	213.2752	192.7608	84.6472	181.9709	149.846	148.6569	93.98751	88.26836	81.91201	107.557
AC092612.	2.882723	5.001952	1.998267	0	0	4.567548	2.549041	0	0	0	0	7.360626	5.140422	9.307203	0.978006	4.122259	0	1.820267	0
AC092614.	63.4199	33.01288	34.96968	59.23969	19.93473	42.24982	41.63433	23.15068	12.84017	35.54587	26.50461	54.28462	48.31997	40.95169	47.92229	42.04704	33.87042	36.40534	26.88924
AC092620.	0.960908	2.000781	5.994802	3.117878	0	6.851322	6.797442	3.704108	22.17847	0.960699	9.638039	10.12086	2.056169	5.584322	8.802052	5.771163	5.131881	11.83173	13.44462
AC092620.	2.882723	10.0039	1.998267	6.235757	0	2.283774	0.84968	0	2.334575	2.882098	0	0.920078	1.028084	0.93072	0	0	0	3.640534	1.792616
AC092620.	14.41361	5.001952	0	5.196464	0	1.141887	5.947762	4.630135	0	11.52839	1.204755	12.8811	10.28084	4.653602	11.73607	11.54233	10.26376	0.910133	3.585232
AC092625.	0	1.00039	0	0	0	0	0.84968	0	0	0	0	0	0	0	0	0	0	0	0
AC092628.	0	0	0	0	0	0	0	0	0	0	0	0	0	0	0	0	0	1.820267	0
AC092628.	0	1.00039	0	0	0	0	0	0	0	0	0	0.920078	0	0	0.978006	0	0	1.820267	0
AC092634.	0.960908	0	0	0	0	0	0.84968	0	0	0	0	0	0	0	0	0	0	0	1.792616
AC092634.	186.4161	56.02186	7.993069	56.12181	19.93473	18.27019	107.9094	32.41095	39.68778	6.724895	25.29985	87.40743	91.49951	52.12034	80.19648	31.32917	26.68578	57.33841	42.12647
AC092634.	60.53717	54.02108	42.96275	42.611	25.47215	25.12151	87.51706	57.41368	46.69151	67.24895	75.89956	81.88696	79.1625	66.08114	74.32844	61.83389	62.60895	71.90054	87.83818
AC092634.	170.0806	129.0504	104.909	73.78979	71.98652	39.96605	107.9094	125.9397	24.51304	53.79916	18.07132	98.44837	99.72418	120.9936	150.6129	100.5831	49.26606	165.6443	112.9348

AC092634.	13.45271	3.001171	4.995668	6.235757	5.537425	3.425661	19.54265	0	3.501863	3.842797	9.638039	8.280704	12.33701	12.09936	7.824047	2.473355	2.052753	8.191201	11.652
AC092634.	0.960908	1.00039	0	0	0	0	0.84968	0	2.334575	0	0	0	0	0	0	0.824452	0	0	1.792616
AC092634.	0	0	0	0	0	0	0	0	0	0	0	0	0	0	0	0.824452	0	1.820267	0
AC092634.	0	0	0	0	0	0	0.84968	0	0	0	0	0.920078	1.028084	0	0.978006	0	1.026376	0	0
AC092636.	101.8562	73.0285	134.883	73.78979	53.15928	55.95246	101.9616	94.45476	168.0894	126.8123	112.0422	87.40743	110.005	83.76483	125.1847	56.88718	43.1078	79.18161	127.2757
AC092641.	125.8789	112.0437	235.7955	170.444	227.0344	77.64832	298.2378	147.2383	15.17474	187.3364	137.3421	241.0605	197.3922	168.4604	182.8871	98.10977	63.63533	95.56401	119.209
AC092645.	117.2307	111.0433	158.8623	267.0982	222.6045	166.7155	165.6876	324.1095	16.34203	141.2228	138.5468	242.9007	338.2398	144.2616	156.4809	140.9813	151.9037	88.28294	75.28987
AC092647.	65.34171	69.02694	29.97401	58.2004	47.62185	60.52001	36.53625	61.11778	21.01118	36.50657	31.32363	36.80313	35.98295	28.85233	34.2302	53.58937	52.34519	43.6864	49.29694
AC092647.	0.960908	0	0	0	0	0	0	0.926027	0	0	0	0	0	0.93072	0	0	0	0.910133	0
AC092647.	4.804538	6.002343	7.993069	0	15.50479	7.993209	11.89552	0.926027	3.501863	5.764196	12.04755	10.12086	10.28084	9.307203	7.824047	15.66458	20.52753	6.370934	22.4077
AC092648.	0	0	0	0	0	0	0	0	0	0	0	1.840156	0	0	0	0	0	0	0
AC092650.	25.9445	9.003514	8.992203	27.02161	8.859879	6.851322	6.797442	22.22465	32.68406	6.724895	31.32363	18.40156	19.5336	9.307203	18.58211	9.06897	4.105505	6.370934	6.274156
AC092650.	0	1.00039	0	0	0	0	0	0	0	1.921399	0	0	1.028084	0	0	0	0	0	0
AC092651.	1.921815	3.001171	0	1.039293	0	4.567548	0	2.778081	0	6.724895	27.70936	3.680313	9.252759	0.93072	2.934017	0	4.105505	0	0.896308
AC092652.	7.68726	30.01171	10.99047	0	1.107485	9.135096	2.549041	10.1863	16.34203	11.52839	1.204755	2.760235	15.42127	5.584322	3.912023	0.824452	1.026376	1.820267	9.859387
AC092652.	7.68726	21.0082	25.97748	5.196464	38.76197	18.27019	9.346483	41.67122	11.67288	23.05678	4.81902	19.32164	18.50552	9.307203	5.868035	3.297807	3.079129	4.550667	8.066772
AC092652.	293.0768	417.1628	471.5911	106.0079	281.3012	232.9449	274.4467	341.704	393.376	410.2186	368.655	358.8305	355.7172	269.9089	363.8182	94.81196	82.1101	213.8814	190.9136
AC092653.	126.8398	101.0394	138.8796	144.4617	107.426	81.07398	151.2431	79.63833	33.85134	80.69874	48.1902	114.0897	136.7352	74.45762	99.75659	89.86525	90.32111	146.5315	120.1053
AC092653.	1.921815	9.003514	13.98787	11.43222	3.322455	2.283774	8.496802	6.482189	3.501863	6.724895	2.40951	11.96102	13.3651	0.93072	0.978006	10.71787	4.105505	12.74187	8.963079
AC092656.	18.25724	22.00859	51.95495	44.68959	19.93473	28.54718	49.28145	47.22738	50.19337	30.74238	32.52838	23.92203	11.30893	46.53602	60.63636	45.34485	7.184634	57.33841	55.57109
AC092658.	18.25724	3.001171	5.994802	15.58939	6.64491	5.709435	12.7452	14.81643	0	5.764196	12.04755	13.80117	0	7.445762	0.978006	1.648904	6.158258	10.9216	7.170464
AC092666.	406.4639	528.2062	316.7254	523.8036	454.0688	212.391	229.4137	121.3095	248.6323	692.6642	1145.722	585.1698	677.5076	90.27987	104.6466	570.5207	719.4898	722.6459	724.2168
AC092666.	2.882723	0	0	0	0	1.141887	0.84968	2.778081	0	12.48909	10.84279	0.920078	0	1.861441	0	3.297807	7.184634	4.550667	0.896308
AC092667.	41.31902	85.03319	70.93849	83.14342	86.38382	73.08077	92.61515	134.2739	98.05217	113.3625	54.21397	51.52438	61.68506	46.53602	88.99853	89.86525	64.6617	75.54107	106.6606
AC092670.	18.25724	10.0039	8.992203	4.157171	9.967364	11.41887	2.549041	7.408216	1.167288	4.803496	6.023774	9.200782	6.168506	8.376483	3.912023	8.244518	11.29014	15.47227	2.688924
AC092674.	0.960908	8.003124	0	0	0	0	0	1.852054	0	0	0	0	1.028084	0	0	0	0	0	5.377848
AC092675.	0	0	3.996535	0	2.21497	0	1.69936	0	0	1.921399	0	0	1.028084	0	0.978006	0	1.026376	0.910133	0
AC092681.	57.65445	206.0804	69.93936	109.1257	73.094	86.78341	94.31451	149.0904	37.35321	51.87776	33.73314	86.48735	103.8365	134.9544	135.9428	146.7524	106.7431	62.79921	108.4533
AC092681.	13.45271	1.00039	13.98787	33.25737	25.47215	14.84453	5.098081	25.92876	5.836439	1.921399	3.614265	4.600391	9.252759	21.40657	30.31818	19.78684	10.26376	13.652	8.066772
AC092681.	6.726353	16.00625	12.98874	35.33595	11.07485	12.56076	16.9936	4.630135	23.34575	19.21399	18.07132	16.56141	12.33701	26.99089	15.64809	12.36678	11.29014	3.640534	15.23724
AC092683.	117.2307	157.0613	99.91337	122.6365	188.2724	95.91851	244.7079	246.3232	93.38302	225.7643	215.6511	103.9688	92.52759	58.63538	56.72434	156.6458	133.4289	197.499	256.3441
AC092683.	10.56998	11.00429	17.98441	3.117878	0	5.709435	1.69936	0	11.67288	0	0	6.440548	11.30893	28.85233	8.802052	6.595615	26.68578	6.370934	4.48154
AC092685.	0.960908	6.002343	5.994802	0	5.537425	1.141887	4.248401	0	0	0	0	0.920078	2.056169	7.445762	0	0	0	0	0
AC092687.	0	0	0	0	0	0	0	0	0	0	0	0	0	0	0	0	0	2.7304	0
AC092687.	0.960908	2.000781	0	0	0	0	0.84968	0	2.334575	1.921399	0	0	0	0	0	0	1.026376	6.370934	0
AC092687.	24.02269	14.00547	32.97141	25.98232	15.50479	12.56076	13.59488	12.03835	65.36811	52.83846	57.82823	48.76415	32.8987	19.54513	10.75806	30.50472	44.13418	28.21414	20.61508
AC092691.	93.20803	120.0469	48.95755	61.31827	102.9961	95.91851	94.31451	70.37805	46.69151	114.3232	232.5177	126.9708	131.5948	103.31	74.32844	98.10977	154.9828	143.8011	251.8625
AC092691.	0.960908	9.003514	1.998267	5.196464	8.859879	10.27698	14.44456	0	14.00745	6.724895	1.204755	7.360626	0	3.722881	5.868035	1.648904	2.052753	14.56213	8.066772
AC092691.	0	0	0	0	0	0	0	0	0	0	0	0.920078	0	0	0	0.824452	0	0	0
AC092692.	61.49808	50.01952	32.97141	77.94696	24.36467	20.55397	67.12474	25.92876	9.338302	34.58517	32.52838	32.20274	40.09529	33.50593	39.12023	34.62698	28.73854	29.12427	35.85232
AC092701.	0	0	0	0	0	0	0	0	0	0.960699	0	0	1.028084	0	0	0.824452	0	0	0
AC092709.	0	0	0	0	0	4.567548	0	0	0	0	0	1.840156	0	0	0	0	5.131881	0	0
AC092718.	7.68726	8.003124	17.98441	3.117878	12.18233	14.84453	9.346483	12.96438	24.51304	19.21399	10.84279	8.280704	14.39318	13.03008	16.6261	12.36678	26.68578	8.191201	17.92616
AC092718.	9.609075	3.001171	0.999134	7.275049	16.61227	1.141887	14.44456	9.26027	4.669151	0.960699	7.228529	8.280704	9.252759	12.09936	6.846041	3.297807	7.184634	31.85467	8.963079
AC092718.	331.5131	295.1152	272.7635	348.1631	305.6658	292.3231	285.4926	303.7369	210.1118	255.546	204.8083	332.1482	316.65	339.7129	286.5557	326.4829	376.6801	292.1528	337.0118
AC092718.	238.3051	300.1171	296.7427	248.391	272.4413	315.1608	169.936	259.2876	793.7557	393.8867	478.2877	170.2145	237.4875	251.2945	272.8636	318.2384	331.5195	384.9864	291.3001
AC092718.	283.4677	267.1042	243.7886	252.5481	221.497	261.4921	248.9563	290.7725	49.02608	200.7861	114.4517	288.9046	303.2849	392.764	376.5322	269.5957	318.1766	283.9616	312.8115
AC092718.	27.86632	6.002343	7.993069	17.66798	7.752394	0	16.9936	12.03835	0	19.21399	8.433284	27.60235	18.50552	22.33729	7.824047	7.420066	13.34289	3.640534	16.13354
AC092718.	149.9016	111.0433	130.8865	205.78	184.95	137.0264	161.4392	90.75065	121.3979	198.8647	115.6565	125.1306	169.6339	100.5178	150.6129	161.5926	138.5608	133.7896	126.3794
AC092718.	42.27993	92.03592	70.93849	106.0079	65.34161	51.38492	70.52346	87.04654	23.34575	108.559	61.4425	54.28462	31.87062	59.5661	45.96627	61.00944	45.16056	54.608	56.4674
AC092720.	98.97348	72.02811	68.94022	125.7544	56.48173	65.08756	104.5107	93.52873	52.52795	49.95636	78.30907	156.4133	130.5667	142.4002	106.6026	119.5455	120.086	129.2389	121.8979
AC092720.	51.88901	87.03397	60.94715	63.39686	42.08443	60.52001	60.3273	73.15614	84.04472	70.13105	95.17564	57.04485	77.10633	52.12034	57.70234	37.10033	97.50574	45.50667	77.97879
AC092723.	0.960908	6.002343	0	0	0	0	0.84968	0.926027	7.003726	0.960699	0	0.920078	0	0	0.978006	1.648904	0	0	0
AC092724.	0	0	0	0	0	0	0	5.556162	4.669151	0	0	0	0	0	3.912023	0	2.052753	0	0
AC092725.	12.4918	4.001562	4.995668	2.078586	9.967364	4.567548	3.398721	2.778081	0	2.882098	0	5.520469	12.33701	7.445762	6.846041	7.420066	4.105505	4.550667	5.377848
AC092745.	3.84363	2.000781	13.98787	9.353635	0	9.135096	9.346483	7.408216	0	2.882098	0	1.840156	9.252759	2.792161	7.824047	1.648904	1.026376	14.56213	8.066772
AC092745.	24.9836	28.01093	34.96968	40.53242	65.34161	47.95925	17.84329	12.03835	94.55031	39.38867	91.56137	39.56336	30.84253	22.33729	29.34017	32.97807	14.36927	50.05734	30.47447
AC092745.	5.765445	7.002733	9.991337	5.196464	6.64491	4.567548	1.69936	11.11232	2.334575	5.764196	4.81902	6.440548	16.44935	0.93072	0.978006	0.824452	1.026376	3.640534	3.585232
AC092746.	0	8.003124	0	0	0	0	5.947762	2.778081	0	0.960699	0	0	0	0	3.912023	0	0	0.910133	0.896308
AC092747.	0	3.001171	5.994802	3.117878	0	12.56076	0.84968	2.778081	0	4.803496	1.204755	8.280704	2.056169	0.93072	4.890029	1.648904	0	2.7304	2.688924
AC092747.	144.1361	123.048	94.9177	122.6365	157.2629	106.1955	94.31451	165.7588	84.04472	66.28825	44.57593	140.772	138.7914	106.1021	102.6906	135.2101	101.6112	94.65388	99.49018
AC092747.	0	4.001562	3.996535	0	0	4.567548	0	0	2.334575	0	0	0	0	0	0	0	0	0	0

AC092747.	3882.066	3041.187	3107.306	4158.21	2901.61	3567.255	3136.17	2743.818	3329.105	3487.338	2582.994	2545.856	2804.614	2735.387	2776.559	2338.145	2634.708	2292.626	2330.401
AC092754.	5.765445	11.00429	3.996535	0	4.42994	5.709435	2.549041	3.704108	0	0.960699	1.204755	10.12086	4.112337	7.445762	4.890029	0.824452	3.079129	9.101334	5.377848
AC092755.	6.726353	13.00508	11.9896	8.314342	8.859879	9.135096	22.09169	19.44657	0	24.01748	7.228529	28.52243	9.252759	15.82225	16.6261	13.19123	15.39564	10.01147	8.963079
AC092755.	1.921815	3.001171	1.998267	1.039293	13.28982	3.425661	9.346483	12.96438	4.669151	3.842797	20.48083	11.96102	7.196591	12.09936	11.73607	22.2602	15.39564	13.652	7.170464
AC092756.	22.10087	2.000781	25.97748	3.117878	45.40688	11.41887	16.9936	4.630135	18.6766	5.764196	2.40951	13.80117	5.140422	7.445762	8.802052	4.122259	7.184634	17.29253	7.170464
AC092757.	2.882723	1.00039	0	0	4.42994	1.141887	0	4.630135	1.167288	1.921399	4.81902	0	1.028084	0	1.956012	0.824452	0	0	2.688924
AC092757.	135.488	84.0328	149.87	70.67191	114.0709	110.763	300.7868	52.78354	171.5913	78.77734	72.28529	215.2983	259.0773	80.97267	109.5367	84.09409	61.58258	249.3766	257.2404
AC092757.	24.02269	24.00937	12.98874	49.88605	48.72934	30.83095	78.17058	19.44657	168.0894	50.91706	62.64725	44.16376	81.21867	32.57521	16.6261	20.6113	8.21101	72.81067	95.90495
AC092769.	0.960908	2.000781	0	0	1.107485	3.425661	5.098081	0	8.171014	0.960699	0	0.920078	1.028084	3.722881	2.934017	10.71787	1.026376	0.910133	5.377848
AC092794.	143.1752	191.0746	234.7964	226.5658	177.1976	137.0264	223.4659	207.4301	126.0671	155.6333	79.51382	224.4991	230.2909	167.5297	188.7551	163.2415	150.8773	201.1395	186.4321
AC092794.	7.68726	8.003124	9.991337	14.5501	18.82724	5.709435	16.9936	20.37259	23.34575	22.09608	34.93789	11.96102	7.196591	3.722881	12.71408	7.420066	7.184634	11.83173	12.54831
AC092798.	0.960908	8.003124	0.999134	5.196464	4.42994	5.709435	39.08529	1.852054	2.334575	0	0	16.56141	3.084253	5.584322	6.846041	0.824452	3.079129	6.370934	11.652
AC092800.	4.804538	6.002343	1.998267	3.117878	0	0	5.947762	0	0	10.56769	1.204755	4.600391	0	7.445762	0	4.122259	1.026376	0.910133	1.792616
AC092801.	1.921815	2.000781	2.997401	3.117878	0	4.567548	1.69936	0	0	1.921399	9.638039	5.520469	3.084253	1.861441	0	4.122259	2.052753	0	3.585232
AC092802.	93.20803	133.0519	190.8345	87.30059	73.094	81.07398	77.3209	178.7232	92.21573	124.8909	206.0131	101.2086	96.63993	80.04195	84.1085	84.91854	101.6112	91.01334	112.9348
AC092802.	153.7452	163.0636	200.8259	331.5344	186.0575	182.7019	225.1653	280.5862	91.04844	212.3145	146.9801	174.8149	201.5045	161.9453	177.9971	227.5487	181.6686	162.9139	189.121
AC092802.	4.804538	6.002343	2.997401	3.117878	2.21497	1.141887	7.647122	2.778081	14.00745	1.921399	1.204755	7.360626	7.196591	2.792161	8.802052	0.824452	8.21101	3.640534	9.859387
AC092803.	2.882723	0	3.996535	0	0	0	0.84968	0	0	5.764196	0	0.920078	0	0	1.956012	1.648904	3.079129	3.640534	0
AC092803.	159.5106	148.0578	168.8536	141.3438	176.0901	165.5736	147.8444	127.7917	213.6137	232.4892	418.0499	130.6511	143.9318	198.2434	180.9311	123.6678	84.16285	160.1835	161.3354
AC092807.	0	2.000781	0.999134	0	0	2.283774	3.398721	0	0	9.606993	0	0	7.196591	0	0.978006	2.473355	0	5.4608	2.688924
AC092807.	18.25724	16.00625	43.96188	28.0609	7.752394	13.70264	39.93497	16.66849	77.04099	30.74238	28.91412	17.48149	34.95487	23.26801	37.16422	7.420066	11.29014	34.58507	22.4077
AC092807.	25.9445	55.02147	47.95842	28.0609	17.71976	4.567548	65.42538	18.52054	29.18219	7.685594	16.86657	34.04289	38.03912	54.9125	46.94428	30.50472	9.237386	37.31547	37.64493
AC092809.	0	1.00039	0	0	0	0	1.69936	7.408216	0	0	0	1.840156	0	0	7.824047	0.824452	0	3.640534	0
AC092809.	128.7616	48.01874	114.9004	28.0609	76.41646	111.9049	21.24201	74.08216	46.69151	45.15286	48.1902	107.6492	82.24675	65.15042	51.83431	51.94047	58.50345	46.4168	48.40063
AC092809.	2.882723	3.001171	11.9896	7.275049	17.71976	11.41887	4.248401	1.852054	24.51304	15.37119	12.04755	11.96102	6.168506	11.16864	2.934017	9.893422	8.21101	14.56213	9.859387
AC092809.	37.47539	41.01601	55.95149	18.70727	43.19191	86.78341	45.03305	90.75065	182.0969	87.42363	130.1135	58.88501	58.60081	53.05106	51.83431	26.38246	34.89679	30.94454	19.71877
AC092810.	7.68726	1.00039	0	0	0	0	0.84968	1.852054	4.669151	7.685594	0	4.600391	0	1.861441	4.890029	4.946711	1.026376	0.910133	0
AC092810.	0	1.00039	2.997401	0	0	1.141887	0	6.482189	1.167288	0	0	0.920078	0	0	0	3.297807	3.079129	5.4608	4.48154
AC092811.	1.921815	4.001562	0	4.157171	6.64491	3.425661	0	5.556162	2.334575	0	2.40951	0	1.028084	1.861441	5.868035	0	0	0	3.585232
AC092811.	4.804538	4.001562	0	6.235757	0	2.283774	11.04584	0.926027	0	0.960699	0	0.920078	1.028084	3.722881	3.912023	3.297807	9.237386	7.281067	8.066772
AC092813.	5.765445	0	0	1.039293	3.322455	1.141887	1.69936	10.1863	2.334575	0	0	3.680313	2.056169	0.93072	0	3.297807	0	5.4608	1.792616
AC092813.	1.921815	6.002343	2.997401	1.039293	0	0	7.647122	0.926027	0	0.960699	0	1.840156	3.084253	0.93072	0	0	0	1.820267	3.585232
AC092814.	8.648168	28.01093	23.97921	36.37525	23.25718	36.54038	9.346483	33.33697	52.52795	25.93888	20.48083	5.520469	10.28084	3.722881	20.53812	23.08465	19.50115	10.01147	11.652
AC092818.	16.33543	60.02343	17.98441	12.47151	32.11706	45.67548	24.64073	12.96438	14.00745	30.74238	63.85201	11.04094	10.28084	20.47585	9.780058	10.71787	13.34289	3.640534	11.652
AC092819.	0	1.00039	0	0	0	0	0	0	0	3.842797	2.40951	0.920078	5.140422	3.722881	0	0	2.052753	0.910133	0.896308
AC092821.	112.4262	2.000781	140.8778	13.51081	105.2111	108.4793	45.03305	112.0493	145.911	46.11356	48.1902	106.7291	99.72418	29.78305	18.58211	112.9499	84.16285	212.9712	187.3284
AC092821.	0	5.001952	0	0	0	1.141887	1.69936	0.926027	0	0	0	1.840156	0	0	0	0.824452	0	0.910133	4.48154
AC092821.	59.57627	28.01093	25.97748	66.51474	40.97694	51.38492	37.38593	75.93422	130.7362	74.93454	77.10431	52.44446	33.92678	111.6864	85.08651	100.5831	68.76721	37.31547	57.36371
AC092824.	0	0	3.996535	0	0	0	0	0	0	0	0	0	0	0	0	0	0	0	0
AC092828.	35.55358	27.01054	24.97834	82.10413	31.00958	21.69585	56.0789	30.55889	73.53913	22.09608	54.21397	28.52243	41.12337	38.15953	44.01026	35.45143	39.0023	26.39387	60.94894
AC092832.	0	0	0	0	0	0	0	0	0	0	0	0	0	0	0	0	0	0	5.377848
AC092832.	0	0	0	0	0	0	0	3.704108	0	0	0	0	0	0	0	0	4.105505	0.910133	0
AC092834.	0	0	0	0	1.107485	9.135096	5.098081	0	0	0	0	4.600391	0	4.653602	0.978006	1.648904	1.026376	0.910133	1.792616
AC092835.	109.5435	140.0547	176.8467	155.8939	168.3377	74.22266	202.2239	149.0904	65.36811	67.24895	67.46627	231.8597	194.3079	133.093	157.4589	145.928	107.7695	121.9579	132.6536
AC092839.	1.921815	0	2.997401	0	1.107485	0	1.69936	0	3.501863	3.842797	2.40951	1.840156	2.056169	0.93072	3.912023	0	1.026376	0	0
AC092839.	0	0	5.994802	3.117878	0	1.141887	1.69936	0	1.167288	0	0	1.840156	5.140422	3.722881	10.75806	0.824452	4.105505	3.640534	0
AC092849.	9.609075	2.000781	4.995668	9.353635	3.322455	2.283774	3.398721	3.704108	2.334575	4.803496	13.2523	1.840156	2.056169	4.653602	8.802052	13.19123	7.184634	5.4608	5.377848
AC092849.	2.882723	2.000781	1.998267	4.157171	0	0	0	0	0	0.960699	0	0	0	0.93072	0	0	1.026376	0.910133	0.896308
AC092850.	3.84363	0	0	0	0	0	0	0	0	0.960699	0	0	0	0	1.956012	0.824452	0	0	0
AC092850.	3.84363	3.001171	0	0	1.107485	0	5.098081	0	0	0	0	2.760235	1.028084	0	0.978006	2.473355	4.105505	0.910133	0
AC092851.	3.84363	8.003124	0	9.353635	1.107485	1.141887	6.797442	10.1863	0	0.960699	0	0.920078	1.028084	3.722881	1.956012	3.297807	3.079129	0.910133	4.48154
AC092865.	0	2.000781	0.999134	0	0	0	0.84968	0.926027	4.669151	0	0	0	2.056169	0	1.956012	0	0	0.910133	0.896308
AC092865.	2.882723	3.001171	1.998267	0	0	1.141887	0.84968	10.1863	7.003726	0	0	3.680313	2.056169	0	3.912023	1.648904	4.105505	4.550667	3.585232
AC092865.	0	1.00039	0	0	1.107485	2.283774	5.098081	0.926027	0	0.960699	0	0.920078	0	0.93072	3.912023	0.824452	0	0.910133	0
AC092868.	15.37452	0	11.9896	0	0	4.567548	5.947762	0	1.167288	0	1.204755	4.600391	3.084253	0	4.890029	0	1.026376	0.910133	0
AC092868.	0.960908	1.00039	0	0	2.21497	0	0	1.852054	4.669151	0	0	0.920078	1.028084	0	1.956012	0.824452	4.105505	0.910133	2.688924
AC092868.	0	4.001562	0	0	0	0	0.84968	0	0	0.960699	0	0	0	0	0	0	0	0.910133	0
AC092881.	28.82723	1.00039	6.993936	57.1611	12.18233	9.135096	60.3273	35.18903	15.17474	4.803496	0	35.88305	6.168506	23.26801	12.71408	11.54233	14.36927	28.21414	25.09662
AC092892.	0	0	0.999134	0	0	0	1.69936	0.926027	0	0	0	0	0	0	0	5.771163	1.026376	8.191201	1.792616
AC092894.	0	1.00039	0	0	0	2.283774	0	0	0	0	0	0	0	0	0	2.473355	0	0	0
AC092902.	40.35812	39.01523	54.95235	18.70727	17.71976	38.82416	33.98721	18.52054	40.85507	47.07426	49.39495	30.36258	30.84253	15.82225	23.47214	41.22259	23.60665	20.93307	9.859387
AC092902.	774.4915	722.2819	844.2679	509.2535	780.7769	899.807	365.3625	821.386	1434.597	904.9787	1218.007	782.9866	795.7373	823.6875	758.9325	710.6775	706.1469	680.7798	743.0393

AC092902.	0.960908	9.003514	10.99047	1.039293	13.28982	3.425661	1.69936	1.852054	4.669151	2.882098	4.81902	7.360626	4.112337	7.445762	6.846041	7.420066	6.158258	3.640534	11.652
AC092902.	121.0743	110.0429	260.7739	50.92535	150.6179	102.7698	100.2623	185.2054	71.20455	187.3364	126.4993	179.4153	194.3079	116.34	144.7449	111.301	118.0333	89.19307	77.97879
AC092903.	0	1.00039	0	0	0	2.283774	0.84968	3.704108	4.669151	0	0	5.520469	1.028084	0	0	0	18.47477	3.640534	0
AC092903.	39.39721	23.00898	19.98267	22.86444	29.90209	13.70264	6.797442	25.00273	53.69524	31.70308	30.11887	28.52243	24.67402	15.82225	42.05425	20.6113	11.29014	17.29253	18.82247
AC092910.	0	0	8.992203	1.039293	0	2.283774	0.84968	3.704108	14.00745	0	0	5.520469	1.028084	3.722881	0	0.824452	7.184634	0	0
AC092910.	0	0	0	0	0	0	3.398721	0.926027	0	0	0	0	0	0	0	0	0	0	0
AC092910.	256.5623	368.1437	441.6171	207.8586	334.4604	341.4242	333.9243	410.23	247.465	329.5198	320.4648	391.9533	341.324	297.8305	381.4223	213.533	238.1193	335.8392	331.6339
AC092916.	0	0	0	0	4.42994	0	0	1.852054	0	0	0	0.920078	1.028084	0	0	0	0	0	1.792616
AC092919.	1.921815	3.001171	9.991337	12.47151	3.322455	1.141887	5.947762	0.926027	3.501863	4.803496	18.07132	2.760235	7.196591	1.861441	8.802052	4.946711	5.131881	2.7304	6.274156
AC092919.	245.9923	294.1148	235.7955	265.0197	241.4317	263.7759	230.2633	297.2547	224.1192	311.2666	371.0645	200.5771	147.0161	313.6527	311.0059	206.9374	300.7282	190.2179	295.7816
AC092920.	12.4918	9.003514	0.999134	2.078586	0	6.851322	9.346483	7.408216	0	4.803496	8.433284	1.840156	7.196591	6.515042	5.868035	3.297807	16.42202	16.3824	6.274156
AC092924.	5.765445	11.00429	8.992203	4.157171	15.50479	10.27698	21.24201	21.29862	10.50559	6.724895	0	4.600391	9.252759	13.9608	21.51613	9.893422	8.21101	3.640534	12.54831
AC092933.	3.84363	1.00039	0	8.314342	0	0	0	0.84968	2.778081	0	4.803496	0	0.920078	4.112337	0	0.978006	4.946711	0	0.910133	0
AC092941.	0	0	0	0	0	0	3.398721	0	3.501863	0	0	0	0	9.307203	0	0	0	0	0
AC092943.	0.960908	0	0.999134	0	0	4.567548	0.84968	11.11232	8.171014	0	0	5.520469	1.028084	0	0	0.824452	0	0	1.792616
AC092944.	102.8171	90.03514	143.8752	145.501	98.56616	93.63473	90.91579	93.52873	92.21573	81.65944	56.62348	92.9279	74.02207	70.73474	69.43841	86.56744	65.68808	79.18161	86.94187
AC092944.	5.765445	2.000781	0.999134	0	1.107485	0	0	0	0	3.842797	0	0	0	0	0	3.297807	0	0	0
AC092952.	0	0	0	0	0	1.141887	0	0	0	0	0	0.920078	0	0	0	0	0	0	3.585232
AC092953.	7.68726	5.001952	4.995668	3.117878	2.21497	1.141887	4.248401	14.81643	9.338302	3.842797	8.433284	4.600391	3.084253	3.722881	3.912023	2.473355	1.026376	8.191201	0.896308
AC092953.	128.7616	158.0617	209.8181	218.2515	126.2533	150.7291	194.5768	137.978	79.37557	142.1835	201.1941	113.1696	176.8305	140.5388	144.7449	117.0722	171.4048	200.2294	131.7573
AC092954.	0	5.001952	0.999134	1.039293	6.64491	10.27698	9.346483	0.926027	11.67288	9.606993	6.023774	1.840156	4.112337	13.03008	2.934017	7.420066	4.105505	5.4608	15.23724
AC092954.	0	1.00039	1.998267	0	0	4.567548	0.84968	0	9.338302	0.960699	2.40951	0	1.028084	0	1.956012	0.824452	0	0	0
AC092957.	9.609075	7.002733	0.999134	5.196464	0	1.141887	0.84968	0	23.34575	2.882098	7.228529	0.920078	0	2.792161	5.868035	1.648904	2.052753	3.640534	0.896308
AC092958.	84.55986	18.00703	32.97141	48.84676	39.86946	45.67548	21.24201	37.96711	121.3979	20.17468	67.46627	91.08774	99.72418	67.94258	70.41642	25.55801	43.1078	46.4168	22.4077
AC092960.	0	0	0	0	0	0	0	0	0	3.842797	0	0	0	0	0	0	0	0	0
AC092964.	11.53089	6.002343	19.98267	6.235757	29.90209	19.41208	2.549041	24.0767	17.50932	9.606993	28.91412	17.48149	26.73019	12.09936	15.64809	2.473355	0	7.281067	1.792616
AC092965.	0	0	0	0	0	0	0	0	0	0	0	0.920078	3.084253	0	0	0.824452	3.079129	0.910133	0
AC092967.	0.960908	1.00039	5.994802	0	0	0	0	0	0	3.842797	0	0	0	4.653602	0	0	0	0	0.896308
AC092978.	10.56998	10.0039	17.98441	1.039293	4.42994	4.567548	7.647122	6.482189	4.669151	9.606993	2.40951	7.360626	10.28084	5.584322	6.846041	4.946711	2.052753	0.910133	1.792616
AC092979.	0	0	0	0	0	1.141887	0.84968	0	2.334575	0	0	0	0	0	0	0.824452	0	0	0
AC092989.	0.960908	0	0	0	0	0	0	0	0	1.921399	0	0	0	0	0	0	0	0	0
AC092991.	0	0	0	0	0	0	4.248401	0	0	0	0	0	0	0	0	0	0	0	0
AC092999.	178.7288	50.01952	67.94109	143.4224	52.05179	81.07398	75.62154	147.2383	43.18965	110.4804	71.08054	153.6531	153.1846	94.93347	119.3167	94.81196	91.34749	237.5448	160.4391
AC093001.	0	0	0	0	0	1.141887	0	0	0	0	0	0	0	0	1.956012	0	0	0	0
AC093010.	3.84363	4.001562	0	0	0	0	0	0.926027	0	0	0	0	1.028084	7.445762	0	0.824452	0	0	0
AC093010.	3.84363	11.00429	20.98181	3.117878	12.18233	3.425661	13.59488	7.408216	10.50559	1.921399	20.48083	20.24172	26.73019	9.307203	19.56012	4.946711	12.31652	21.8432	2.688924
AC093012.	24.9836	24.00937	22.98007	14.5501	22.1497	22.83774	68.8241	45.37532	0	20.17468	1.204755	29.4425	19.5336	44.67457	44.01026	23.08465	35.92317	43.6864	39.43755
AC093014.	2.882723	5.001952	11.9896	1.039293	6.64491	4.567548	1.69936	6.482189	4.669151	8.646293	12.04755	2.760235	1.028084	1.861441	4.890029	10.71787	1.026376	10.01147	1.792616
AC093019.	0	6.002343	1.998267	0	8.859879	4.567548	2.549041	0	22.17847	0	4.81902	0.920078	9.252759	0	1.956012	2.473355	0	0	0.896308
AC093019.	0	0	0	1.039293	0	1.141887	2.549041	0.926027	0	0.960699	0	0.920078	0	0	0	3.297807	0	0	1.792616
AC093025.	0	0	0	0	0	0	1.69936	0	0	0	0	0	0	0	0	0	1.026376	0	0
AC093028.	0	0	0	1.039293	0	1.141887	0	3.704108	37.35321	0	8.433284	1.840156	3.084253	0	0	0.824452	0	0.910133	0.896308
AC093063.	0	8.003124	0	0	19.93473	10.27698	0	0	0	0.960699	8.433284	0	0	0	3.912023	0	2.052753	1.820267	4.48154
AC093083.	1.921815	0	0	0	0	0	0	0	0	0	0	2.760235	0	0	0	0	0	0	0
AC093084.	3.84363	0	5.994802	0	0	0	1.69936	0	0	0	1.204755	0	0	0	0	0	0	0	0
AC093107.	2.882723	26.01015	12.98874	6.235757	8.859879	12.56076	21.24201	0	14.00745	13.44979	37.3474	7.360626	3.084253	14.89152	0	17.31349	8.21101	2.7304	6.274156
AC093107.	6.726353	15.00586	12.98874	23.90373	1.107485	14.84453	23.79105	11.11232	0	45.15286	21.68559	24.84211	8.224675	10.23792	13.69208	10.71787	18.47477	7.281067	9.859387
AC093110.	3348.763	3365.313	4652.965	3059.678	4147.531	3085.379	4599.319	3762.448	2460.643	4747.776	4302.18	4534.146	4955.367	3297.542	3740.872	2084.214	2187.208	2785.918	3271.524
AC093117.	15.37452	9.003514	32.97141	15.58939	1.107485	11.41887	22.94137	12.96438	10.50559	13.44979	9.638039	7.360626	13.3651	9.307203	1.956012	12.36678	16.42202	22.75334	11.652
AC093142.	4.804538	0	10.99047	2.078586	2.21497	2.283774	0	1.852054	0	18.25329	3.614265	0	4.112337	19.54513	0.978006	1.648904	1.026376	7.281067	0.896308
AC093151.	0	0	0	0	0	0	0	0	2.334575	0	0	0.920078	2.056169	0	1.956012	0	0	0	0
AC093151.	22.10087	58.02265	53.95322	19.74656	27.68712	41.10793	22.94137	57.41368	131.9035	69.17035	59.03299	45.08383	38.03912	58.63538	48.90029	5.771163	23.60665	23.66347	18.82247
AC093151.	11.53089	16.00625	16.98527	12.47151	7.752394	19.41208	11.89552	11.11232	17.50932	31.70308	31.32363	8.280704	4.112337	6.515042	20.53812	10.71787	6.158258	19.1128	16.13354
AC093152.	13.45271	7.002733	4.995668	9.353635	3.322455	4.567548	15.29424	12.03835	9.338302	7.685594	3.614265	11.04094	4.112337	7.445762	6.846041	11.54233	8.21101	6.370934	8.963079
AC093155.	0	0	0	1.039293	0	1.141887	0	0	0	0	9.638039	2.760235	1.028084	0	0.978006	0	1.026376	0	0.896308
AC093155.	0	0	0	0	2.21497	0	0.84968	0.926027	1.167288	2.882098	6.023774	3.680313	2.056169	0	2.934017	0	0	0	0
AC093155.	0	3.001171	0	0	0	3.425661	0	0	0	0.960699	0	0.920078	0	0	0	0	0	0	0
AC093156.	0	0	3.996535	0	0	0	0	0	0	0	0	0	1.028084	0	0	0	0	0	0
AC093157.	683.2053	914.3569	685.4057	792.9804	840.5811	826.7262	621.9659	1300.142	1690.233	863.6686	832.4856	801.3881	745.3612	1089.873	1065.048	1070.963	887.8155	778.1641	743.9356
AC093157.	35.55358	49.01913	47.95842	27.02161	52.05179	17.12831	49.28145	68.526	24.51304	29.78168	30.11887	54.28462	71.96591	41.88241	43.03226	65.95615	54.39794	68.26001	50.19325
AC093158.	41.31902	29.01132	69.93936	37.41454	52.05179	67.37133	51.83049	49.07943	38.5205	27.86028	44.57593	57.96493	105.8927	40.95169	46.94428	24.73355	22.58028	35.4952	32.26709
AC093159.	24.9836	29.01132	18.98354	20.78586	48.72934	27.40529	16.9936	4.630135	91.04844	36.50657	63.85201	11.96102	15.42127	12.09936	14.67009	16.48904	17.4484	13.652	14.34093

AC093159.	0	0	0	1.039293	4.42994	2.283774	0	0	0	0.960699	0	0	0	0	0	0	0	0	0
AC093162.	4.804538	3.001171	1.998267	3.117878	6.64491	5.709435	3.398721	7.408216	4.669151	1.921399	6.023774	4.600391	4.112337	0.93072	5.868035	8.244518	1.026376	13.652	4.48154
AC093162.	169.1197	139.0543	95.91683	171.4833	126.2533	135.8846	96.86355	446.345	63.03354	280.5242	291.5507	172.9747	194.3079	187.0748	186.7991	224.2509	154.9828	167.4645	218.6991
AC093166.	0	0	0	8.314342	0	0	0	0	0	0	0	0	0	0	0.978006	0.824452	0	0	1.792616
AC093166.	0.960908	1.00039	1.998267	0	0	0	2.549041	0	0	0	0	0	0	0	4.890029	0	0	0.910133	0
AC093166.	0	0	4.995668	0	0	4.567548	0	0	0	0	4.81902	1.840156	1.028084	0	5.868035	0.824452	1.026376	7.281067	0.896308
AC093206.	1.921815	1.00039	9.991337	0	0	3.425661	2.549041	0.926027	4.669151	4.803496	0	0.920078	2.056169	0	0	0	1.026376	0	0.896308
AC093214.	0.960908	0	5.994802	5.196464	0	0	5.947762	6.482189	1.167288	3.842797	7.228529	7.360626	1.028084	0.93072	0	7.420066	7.184634	1.820267	4.48154
AC093225.	4.804538	6.002343	7.993069	0	0	2.283774	0.84968	10.1863	0	17.29259	0	4.600391	6.168506	11.16864	8.802052	0	0	0	0
AC093227.	910.9403	779.3042	896.2229	702.5619	684.4257	977.4553	800.3988	882.5038	555.629	720.5244	548.1635	862.1133	996.2138	779.9436	1067.982	708.2041	680.4875	762.6918	639.0676
AC093227.	13.45271	28.01093	10.99047	6.235757	7.752394	28.54718	13.59488	12.96438	43.18965	29.78168	38.55216	10.12086	6.168506	17.68369	9.780058	14.01568	21.5539	9.101334	22.4077
AC093227.	3.84363	4.001562	1.998267	0	0	2.283774	1.69936	0	5.836439	0	19.27608	0	0	5.584322	3.912023	0	7.184634	4.550667	2.688924
AC093248.	0	4.001562	0	0	0	0	0.84968	9.26027	0	0	2.40951	0	1.028084	0	0	0	1.026376	0	0
AC093249.	0	0	0	0	0	0	0	0	0	0	2.40951	0.920078	0	0	0	0	0	0	0.896308
AC093249.	28.82723	27.01054	50.95582	27.02161	40.97694	46.81737	44.18337	62.04381	33.85134	27.86028	98.7899	45.08383	17.47743	43.74385	41.07624	28.85581	20.52753	43.6864	47.50432
AC093249.	0.960908	2.000781	3.996535	0	9.967364	1.141887	4.248401	0.926027	14.00745	1.921399	0	3.680313	4.112337	1.861441	2.934017	0.824452	14.36927	11.83173	19.71877
AC093249.	6.726353	9.003514	0.999134	7.275049	4.42994	1.141887	0	3.704108	4.669151	0.960699	3.614265	11.04094	4.112337	0	3.912023	6.595615	1.026376	1.820267	4.48154
AC093249.	0.960908	0	4.995668	0	0	5.709435	0	0	0	0	0	0	0	0	0	0	0	0	0
AC093249.	35.55358	53.02069	69.93936	27.02161	55.37425	21.69585	38.23561	24.0767	208.9445	109.5197	278.2984	45.08383	39.06721	57.70466	55.74633	15.66458	27.71216	25.48374	43.91909
AC093259.	0	0	0	0	0	0	0.84968	0	0	0	0	0	0	0	0	0	0	0.910133	0
AC093267.	0	1.00039	12.98874	0	0	0	4.248401	3.704108	0	3.842797	1.204755	0.920078	2.056169	6.515042	0.978006	5.771163	2.052753	0	0
AC093270.	2.882723	0	1.998267	0	0	4.567548	3.398721	0	2.334575	0.960699	4.81902	5.520469	0	1.861441	0	4.122259	5.131881	0.910133	5.377848
AC093270.	0	0	0.999134	0	1.107485	0	0	0	1.167288	0	0	0	0	0	1.956012	0	0	0	0
AC093274.	16.33543	5.001952	18.98354	4.157171	0	11.41887	28.03945	1.852054	7.003726	16.33189	7.228529	5.520469	10.28084	18.61441	6.846041	10.71787	7.184634	3.640534	2.688924
AC093277.	0	0	0	8.314342	0	0	3.398721	3.704108	0	0	0	0.920078	0	0	0.978006	1.648904	0	2.7304	1.792616
AC093278.	5.765445	9.003514	34.96968	11.43222	8.859879	19.41208	18.69297	31.48492	0	9.606993	1.204755	19.32164	18.50552	3.722881	2.934017	11.54233	7.184634	10.9216	10.7557
AC093278.	758.156	1021.399	1146.006	818.9627	1144.032	1484.453	631.3124	973.2544	773.9118	1043.319	1157.769	716.7409	1002.382	1468.677	1579.479	688.4173	758.492	779.0742	551.2294
AC093281.	0	0	0	4.157171	0	0	0	0	0	0	0	0	3.084253	0	0	4.122259	0	0	1.792616
AC093281.	0	0	0	0	0	0	0	0	0	0	0	2.760235	3.084253	3.722881	0	6.595615	0	0.910133	0.896308
AC093283.	0	2.000781	0	0	0	1.141887	1.69936	0	0	0.960699	0	4.600391	0	1.861441	0	0	0	0.910133	0
AC093286.	0	0	0	0	0	0	0.84968	1.852054	0	0	1.204755	0.920078	1.028084	0	0	0.824452	0	0	0.896308
AC093292.	0.960908	2.000781	0.999134	0	0	0	2.549041	0.926027	0	0	0	0	1.028084	0	0	0	4.105505	2.7304	0
AC093297.	1.921815	2.000781	4.995668	3.117878	5.537425	2.283774	1.69936	3.704108	9.338302	2.882098	2.40951	10.12086	0	2.792161	3.912023	9.893422	2.052753	9.101334	3.585232
AC093297.	1449.049	1563.61	1104.043	1607.786	948.0071	892.9556	1560.863	1116.789	587.1457	761.8345	449.3736	1150.098	1136.033	1131.756	1216.639	1301.809	1402.03	982.034	1143.689
AC093297.	3760.031	2983.164	3547.924	4240.315	3009.037	4050.273	3023.162	3414.262	3660.614	3570.919	3364.88	2821.88	3200.427	3240.768	3345.758	2848.481	2859.484	2842.347	2567.922
AC093305.	0	1.00039	1.998267	0	0	0	0	0	0	0	0	0	0	0	3.912023	0	0	1.820267	0
AC093311.	2.882723	4.001562	20.98181	6.235757	1.107485	4.567548	0	13.89041	9.338302	6.724895	24.0951	9.200782	5.140422	15.82225	6.846041	5.771163	8.21101	5.4608	0
AC093323.	1999.649	2441.953	2145.14	1677.419	2679.006	2281.49	2317.928	2368.777	3222.881	2552.578	2682.989	1917.443	1961.585	2266.304	2156.503	2168.308	2137.942	2030.508	1588.258
AC093323.	6.726353	12.00469	13.98787	8.314342	0	3.425661	5.098081	6.482189	2.334575	10.56769	14.45706	14.72125	6.168506	5.584322	6.846041	9.893422	10.26376	2.7304	15.23724
AC093323.	5.765445	3.001171	5.994802	1.039293	5.537425	1.141887	2.549041	1.852054	1.167288	0.960699	4.81902	7.360626	9.252759	11.16864	7.824047	0.824452	2.052753	2.7304	1.792616
AC093326.	33.63176	20.00781	30.97314	67.55403	29.90209	9.135096	1.69936	3.704108	1.167288	4.803496	0	24.84211	4.112337	16.75297	18.58211	4.122259	2.052753	6.370934	1.792616
AC093326.	0	0	2.997401	0	1.107485	0	0.84968	3.704108	0	0	0	0.920078	0	0.93072	4.890029	0	0	0	0.896308
AC093330.	16.33543	17.00664	31.97228	86.2613	63.12664	38.82416	34.83689	18.52054	206.6099	56.68126	62.64725	24.84211	57.57272	6.515042	14.67009	21.43575	22.58028	18.20267	28.68185
AC093330.	278.6632	169.066	820.2887	261.9018	516.088	203.2559	687.3913	267.6218	189.1006	209.4324	231.3129	537.3257	422.5427	193.5898	196.5792	82.44518	103.664	118.3173	173.8837
AC093331.	0	0	0	0	0	0	0	0	0	0	0	0.920078	0	0	0	0	1.026376	0	0
AC093334.	0.960908	1.00039	3.996535	0	2.21497	0	1.69936	2.778081	0	0	0	3.680313	2.056169	0	0	0.824452	2.052753	1.820267	3.585232
AC093367.	3.84363	11.00429	4.995668	11.43222	21.04221	13.70264	0.84968	10.1863	0	3.842797	39.75691	9.200782	8.224675	11.16864	7.824047	4.946711	13.34289	2.7304	6.274156
AC093382.	8.648168	22.00859	19.98267	5.196464	7.752394	9.135096	11.89552	21.29862	5.836439	7.685594	8.433284	3.680313	0	22.33729	10.75806	2.473355	1.026376	0	1.792616
AC093388.	36.51449	51.01991	30.97314	36.37525	32.11706	35.3985	31.43817	52.78354	84.04472	48.03496	44.57593	30.36258	25.70211	35.36737	20.53812	51.94047	36.94955	75.54107	55.57109
AC093390.	9.609075	12.00469	18.98354	9.353635	2.21497	15.98642	10.19616	12.96438	29.18219	3.842797	1.204755	27.60235	6.168506	15.82225	11.73607	17.31349	24.63303	6.370934	17.02985
AC093390.	11.53089	22.00859	8.992203	6.235757	17.71976	13.70264	2.549041	11.11232	3.501863	13.44979	18.07132	8.280704	7.196591	5.584322	3.912023	10.71787	20.52753	10.9216	9.859387
AC093392.	0	0	0	0	0	2.283774	0.84968	0	0	0	0	0.920078	2.056169	0	0	0	1.026376	1.820267	2.688924
AC093392.	0	0	0	0	0	0	0	0	0	0	0	0	0	0	0	3.297807	0	0	0
AC093414.	0	0	0	0	0	0	1.69936	0	0	0	0	0	0	0	0	0	1.026376	0	0.896308
AC093422.	0	0	0	2.078586	0	0	0.84968	0	0	0	0	0.920078	1.028084	5.584322	0	0	0	0.910133	0
AC093422.	2.882723	1.00039	6.993936	1.039293	3.322455	0	0	9.26027	7.003726	4.803496	0	1.840156	1.028084	5.584322	3.912023	0	0	0	0.896308
AC093422.	0	0	4.995668	5.196464	0	15.98642	0.84968	0	0	3.842797	0	0.920078	1.028084	8.376483	0	3.297807	4.105505	6.370934	11.652
AC093423.	2.882723	0	0	0	18.82724	0	2.549041	0	11.67288	0	1.204755	11.96102	2.056169	0.93072	0.978006	0	0	3.640534	1.792616
AC093423.	120.1134	127.0496	208.8189	98.73281	131.7907	89.06719	156.3412	168.5369	64.20083	108.559	97.58515	132.4913	159.3531	136.8159	114.4267	79.14738	119.0596	126.5085	115.6237
AC093424.	34.59267	69.02694	29.97401	39.49313	35.43952	26.2634	72.22282	41.67122	102.7213	97.03062	97.58515	26.68227	60.65698	45.60529	43.03226	64.30724	65.68808	53.69787	40.33386
AC093426.	0	1.00039	0	0	0	0	0	0	4.669151	0	1.204755	0	0	0	0	0	0	0	0
AC093433.	0.960908	0	0	1.039293	0	0	0.84968	0	0	0.960699	0	2.760235	0	0	0	0	0	5.4608	0

AC093458.	18.25724	31.0121	10.99047	11.43222	27.68712	5.709435	11.04584	29.63287	3.501863	17.29259	16.86657	20.24172	20.56169	3.722881	6.846041	14.84013	16.42202	9.101334	26.88924
AC093458.	166.237	314.1226	236.7947	240.0766	198.2398	191.837	144.4456	217.6164	378.2012	302.6203	512.0208	182.1755	214.8696	161.9453	213.2053	137.6835	152.9301	233.9043	409.6127
AC093459.	0	0	0	0	0	0	0	0	0	0	0	0	0	0	0	0	0	0	3.585232
AC093462.	0.960908	21.0082	29.97401	33.25737	15.50479	28.54718	9.346483	1.852054	16.34203	22.09608	22.89034	33.12282	24.67402	26.99089	14.67009	31.32917	13.34289	30.0344	26.88924
AC093484.	30.74904	25.00976	10.99047	33.25737	23.25718	20.55397	14.44456	16.66849	12.84017	10.56769	24.0951	22.08188	26.73019	21.40657	11.73607	34.62698	28.73854	29.12427	30.47447
AC093484.	16.33543	13.00508	6.993936	2.078586	17.71976	21.69585	5.947762	9.26027	0	11.52839	24.0951	33.12282	14.39318	18.61441	18.58211	5.771163	15.39564	11.83173	30.47447
AC093484.	11.53089	8.003124	0	0	2.21497	1.141887	2.549041	0	5.836439	4.803496	0	0.920078	5.140422	2.792161	3.912023	5.771163	6.158258	9.101334	2.688924
AC093484.	47.08447	54.02108	42.96275	44.68959	90.81376	46.81737	42.48401	57.41368	14.00745	39.38867	85.5376	88.32751	92.52759	84.69555	65.52639	57.71163	72.87271	101.9349	54.67478
AC093495.	829.2632	579.2261	1088.057	757.6444	837.2586	556.099	932.9489	811.1997	605.8223	776.245	895.1329	1014.846	1053.786	904.6601	915.4135	850.8343	777.9932	1033.001	1114.111
AC093496.	0.960908	1.00039	0	0	0	11.41887	0	0.926027	25.68033	0	0	0.920078	1.028084	0	0	10.71787	5.131881	0.910133	0.896308
AC093503.	8.648168	25.00976	50.95582	5.196464	42.08443	17.12831	18.69297	57.41368	18.6766	13.44979	39.75691	23.92203	14.39318	32.57521	18.58211	9.893422	7.184634	27.304	25.99293
AC093503.	52.84991	60.02343	48.95755	42.611	68.66407	61.6619	30.58849	70.37805	47.8588	83.58083	148.1848	54.28462	45.23571	38.15953	47.92229	59.36053	100.5849	38.2256	42.12647
AC093510.	27.86632	36.01406	40.96448	36.37525	34.33203	42.24982	28.03945	26.85478	63.03354	19.21399	50.5997	33.12282	51.40422	29.78305	34.2302	15.66458	39.0023	32.7648	43.02278
AC093510.	50.9281	31.0121	25.97748	82.10413	37.65449	73.08077	33.13753	77.78627	56.02981	67.24895	65.05676	26.68227	26.73019	31.64449	51.83431	42.8715	48.23968	27.304	34.0597
AC093515.	0	1.00039	0	4.157171	0	0	2.549041	0	0	0	0	0	0	0.93072	0	0	0	0	0
AC093520.	0	1.00039	0.999134	4.157171	6.64491	2.283774	0.84968	0	0	0	0	2.760235	1.028084	0	0	6.595615	8.21101	5.4608	4.48154
AC093520.	15.37452	20.00781	26.97661	10.39293	18.82724	25.12151	6.797442	23.15068	29.18219	7.685594	3.614265	15.64133	20.56169	0	7.824047	4.122259	4.105505	2.7304	0.896308
AC093523.	11.53089	6.002343	2.997401	6.235757	1.107485	3.425661	4.248401	11.11232	7.003726	0	0	4.600391	2.056169	5.584322	0	13.19123	9.237386	10.9216	6.274156
AC093524.	0.960908	0	3.996535	0	4.42994	0	1.69936	5.556162	0	0.960699	0	0	0	3.722881	0	2.473355	6.158258	0.910133	0
AC093525.	24.02269	45.01757	49.95668	48.84676	141.7581	29.68906	56.0789	14.81643	75.8737	47.07426	89.15186	108.5692	64.76932	56.77394	37.16422	45.34485	62.60895	31.85467	43.02278
AC093525.	0	0	0	0	0	2.283774	0	0	0	0	0	0	0	0	0	0	0	0	0
AC093525.	0.960908	0	0	0	5.537425	1.141887	0	2.778081	0	0	0	0	0	0	0.978006	0	0	0	0
AC093525.	4.804538	0	5.994802	11.43222	4.42994	11.41887	10.19616	6.482189	5.836439	1.921399	9.638039	15.64133	13.3651	0.93072	3.912023	6.595615	7.184634	3.640534	6.274156
AC093525.	0	0	0	0	3.322455	0	0	0	0	0	0	0	0	0	0	0	0	0	0
AC093525.	0.960908	77.03006	12.98874	1.039293	0	54.81058	6.797442	52.78354	342.0153	164.2796	145.7753	32.20274	17.47743	3.722881	2.934017	3.297807	2.052753	5.4608	0.896308
AC093525.	6.726353	1.00039	19.98267	8.314342	8.859879	3.425661	11.89552	12.03835	1.167288	11.52839	0	34.96297	26.73019	2.792161	1.956012	3.297807	6.158258	4.550667	4.48154
AC093525.	276.7414	184.0718	190.8345	302.4342	119.6084	219.2423	191.1781	185.2054	169.2567	277.6421	249.3843	250.2613	226.1786	168.4604	162.349	380.0723	251.4622	502.3936	402.4423
AC093525.	41.31902	34.01327	47.95842	15.58939	40.97694	25.12151	24.64073	34.263	44.35693	39.38867	12.04755	68.08579	52.4323	13.9608	21.51613	50.29156	45.16056	23.66347	25.09662
AC093535.	185.4552	168.0656	303.7366	162.1297	201.5623	174.7087	268.499	197.2438	273.1453	194.0612	193.9655	275.1034	260.1053	280.1468	330.566	144.2791	135.4817	261.2083	220.4918
AC093535.	4025.242	2685.048	4460.133	3133.468	3324.67	4070.827	3120.026	3529.089	5431.39	4326.029	4425.065	3891.011	4314.87	3401.783	3504.195	2297.747	2421.222	3565.903	3369.222
AC093536.	1.921815	0	0	1.039293	2.21497	0	0	0	0	0	2.40951	0.920078	0	0	0	0.824452	1.026376	0.910133	0.896308
AC093536.	0.960908	4.001562	0	10.39293	2.21497	0	0.84968	0	0	0	0	0	0	0	0	0	0	0	0.896308
AC093567.	11.53089	7.002733	13.98787	20.78586	13.28982	5.709435	15.29424	2.778081	2.334575	11.52839	1.204755	28.52243	6.168506	10.23792	16.6261	41.22259	19.50115	28.21414	20.61508
AC093578.	0	0	0	0	2.21497	1.141887	3.398721	0	1.167288	0	0	4.600391	1.028084	0	0.978006	0	0	0	0.896308
AC093579.	1.921815	0	0	0	0	2.283774	0	2.778081	0	5.764196	0	0.920078	0	9.307203	0.978006	0	0	0	0
AC093580.	0	0	0	2.078586	6.64491	0	0	0	0	0	0	0	0	0	0	0	0	0	0
AC093582.	12.4918	2.000781	6.993936	7.275049	0	14.84453	13.59488	6.482189	1.167288	9.606993	0	21.1618	9.252759	10.23792	6.846041	4.946711	13.34289	28.21414	28.68185
AC093583.	0.960908	7.002733	0	0	1.107485	1.141887	1.69936	6.482189	1.167288	3.842797	0	10.12086	4.112337	0	0.978006	0	1.026376	5.4608	5.377848
AC093585.	0	4.001562	0	0	0	0	0	3.704108	1.167288	0	1.204755	5.520469	3.084253	4.653602	0.978006	8.244518	1.026376	4.550667	0.896308
AC093591.	13.45271	12.00469	9.991337	3.117878	6.64491	4.567548	7.647122	2.778081	3.501863	2.882098	3.614265	8.280704	8.224675	9.307203	3.912023	8.244518	0	7.281067	3.585232
AC093591.	17.29634	25.00976	23.97921	7.275049	33.22455	43.39171	33.98721	13.89041	53.69524	26.89958	30.11887	14.72125	13.3651	22.33729	9.780058	17.31349	28.73854	16.3824	17.92616
AC093591.	13.45271	18.00703	4.995668	15.58939	19.93473	10.27698	17.84329	11.11232	1.167288	4.803496	1.204755	19.32164	11.30893	10.23792	9.780058	16.48904	4.105505	14.56213	17.92616
AC093599.	14.41361	9.003514	11.9896	6.235757	3.322455	9.135096	17.84329	30.55889	5.836439	10.56769	12.04755	6.440548	15.42127	5.584322	4.890029	4.946711	5.131881	0	2.688924
AC093607.	0	1.00039	1.998267	3.117878	1.107485	2.283774	3.398721	1.852054	1.167288	0.960699	0	2.760235	6.168506	2.792161	5.868035	2.473355	0	0	2.688924
AC093609.	196.986	241.0941	522.5469	150.6975	237.0018	261.4921	342.4211	266.6958	259.1379	364.105	356.6074	255.7817	295.0602	332.2671	366.7522	64.30724	69.79359	85.55254	126.3794
AC093616.	277.7023	306.1195	253.78	268.1375	326.7081	283.188	332.225	230.5807	243.9631	215.1966	204.8083	422.3159	377.307	354.6044	350.1261	321.5362	289.4381	425.0323	397.0644
AC093620.	3.84363	6.002343	8.992203	1.039293	1.107485	7.993209	0.84968	0	21.01118	8.646293	59.03299	6.440548	10.28084	0.93072	0.978006	0.824452	0	4.550667	2.688924
AC093627.	0	1.00039	0.999134	4.157171	0	2.283774	0.84968	0	7.003726	0.960699	0	6.440548	1.028084	0	0	11.54233	19.50115	0	10.7557
AC093627.	0	1.00039	0	0	11.07485	0	0.84968	0	0	0	0	0.920078	1.028084	0	0	0.824452	0	0	0.896308
AC093627.	4.804538	2.000781	1.998267	2.078586	2.21497	4.567548	0	0.926027	5.836439	7.685594	0	11.96102	7.196591	6.515042	6.846041	4.946711	4.105505	4.550667	1.792616
AC093627.	471.8056	462.1804	438.6197	525.8821	598.0419	559.5246	284.6429	697.2984	571.971	415.9828	514.4303	343.1892	305.3411	419.7549	415.6525	612.5677	443.3945	672.5886	406.9238
AC093627.	183.5333	155.0605	204.8224	121.5973	153.9404	76.50643	135.9488	158.3506	68.86998	121.0481	81.92333	142.6121	125.4263	193.5898	156.4809	170.6615	137.5344	153.8125	138.9277
AC093627.	12.4918	21.0082	36.96795	24.94303	1.107485	18.27019	9.346483	11.11232	5.836439	0.960699	16.86657	1.840156	4.112337	27.92161	27.38416	80.79628	95.45299	25.48374	18.82247
AC093627.	249.836	185.0722	85.9255	184.9941	170.5527	100.4861	44.18337	42.59724	26.84762	52.83846	73.49005	132.4913	77.10633	145.1924	123.2287	448.5018	254.5413	244.8259	190.9136
AC093635.	77.83351	102.0398	63.94455	65.47544	44.2994	93.63473	101.9616	100.9369	228.7884	142.1835	196.375	99.36845	75.05016	84.69555	67.4824	50.29156	76.97822	103.7552	105.7643
AC093639.	0	0	0	0	0	0	1.69936	0	0	0	0	0	0	0	0	0	0	0	0
AC093642.	0	0	3.996535	16.62868	2.21497	0	10.19616	21.29862	0	5.764196	4.81902	23.00196	3.084253	6.515042	10.75806	9.06897	12.31652	2.7304	1.792616
AC093642.	902.2922	496.1937	909.2116	470.7996	983.4466	389.3835	559.9393	1145.495	971.1834	799.3018	581.8966	493.1619	521.2388	526.7877	496.827	367.7055	524.4783	420.4816	329.8413
AC093663.	1.921815	6.002343	0.999134	7.275049	9.967364	3.425661	2.549041	5.556162	9.338302	0.960699	2.40951	1.840156	5.140422	7.445762	5.868035	7.420066	6.158258	5.4608	7.170464
AC093668.	19.21815	33.01288	42.96275	15.58939	19.93473	15.98642	33.13753	29.63287	39.68778	41.31007	55.41872	20.24172	18.50552	38.15953	44.01026	9.06897	10.26376	13.652	27.78555
AC093671.	3.84363	11.00429	7.993069	11.43222	1.107485	7.993209	9.346483	5.556162	14.00745	13.44979	1.204755	4.600391	5.140422	7.445762	11.73607	14.01568	16.42202	8.191201	16.13354
AC093673.	759.1169	786.3069	612.4689	946.7957	594.7194	841.5707	884.5171	454.6793	1435.764	728.21	797.5477	798.6279	806.0181	652.4349	691.4501	1084.154	1162.884	840.9633	833.5664

AC093673.	67.26353	84.0328	35.96881	38.45383	64.23413	77.64832	65.42538	77.78627	64.20083	93.18783	45.78069	45.08383	38.03912	98.65635	60.63636	46.1693	73.89909	72.81067	79.77141
AC093677.	21.13997	49.01913	67.94109	51.96464	8.859879	42.24982	20.39233	45.37532	21.01118	34.58517	20.48083	39.56336	33.92678	38.15953	53.79032	34.62698	44.13418	37.31547	31.37078
AC093690.	66.30262	79.03084	98.91423	84.18271	50.94431	70.79699	106.21	93.52873	65.36811	81.65944	116.8612	113.1696	132.6229	83.76483	126.1628	80.79628	85.18923	67.34987	53.77848
AC093698.	0.960908	3.001171	0	6.235757	7.752394	0	0	0	1.167288	0.960699	1.204755	2.760235	1.028084	2.792161	0.978006	2.473355	0	0.910133	1.792616
AC093698.	0	4.001562	0	0	0	0	1.69936	0	9.338302	0	0	0	1.028084	0	1.956012	0.824452	4.105505	0	2.688924
AC093700.	16.33543	20.00781	35.96881	11.43222	14.3973	11.41887	18.69297	33.33697	1.167288	22.09608	33.73314	14.72125	16.44935	0.93072	0.978006	6.595615	9.237386	41.86614	34.0597
AC093702.	0	0	0	0	0	0	0	0	0	0	4.81902	0	0	0	0	0	0	0	0.896308
AC093719.	6.726353	13.00508	0.999134	21.82515	31.00958	3.425661	9.346483	1.852054	0	24.97818	15.66181	4.600391	6.168506	4.653602	20.53812	5.771163	15.39564	13.652	10.7557
AC093722.	0	0	0	0	0	0	1.69936	0	0	0	0	0	0	0	0	0	0	0	0
AC093724.	134.5271	136.0531	144.8744	114.3222	187.165	66.22945	71.37314	161.1287	50.19337	74.93454	132.523	194.1365	198.4203	201.0356	217.1173	159.1192	206.3016	231.1739	262.6182
AC093726.	824.4587	764.2983	708.3858	646.4401	827.2912	722.8145	680.5939	901.0243	811.265	783.9306	639.7248	685.4583	687.7884	737.1305	863.5791	782.4048	721.5425	703.5331	553.022
AC093726.	190.2597	152.0593	209.8181	120.558	204.8847	187.2695	143.596	194.4657	302.3275	271.8779	312.0315	168.3743	173.7463	176.8369	156.4809	123.6678	124.1915	139.2504	160.4391
AC093726.	1.921815	0	0	1.039293	0	0	1.69936	0	0	0	0	0	0	0	0	1.648904	10.26376	1.820267	0
AC093732.	9.609075	8.003124	14.987	5.196464	27.68712	11.41887	11.04584	13.89041	2.334575	6.724895	0	18.40156	35.98295	21.40657	18.58211	26.38246	12.31652	20.93307	14.34093
AC093732.	4.804538	17.00664	12.98874	6.235757	21.04221	4.567548	14.44456	20.37259	17.50932	15.37119	8.433284	13.80117	9.252759	17.68369	2.934017	10.71787	8.21101	8.191201	0.896308
AC093734.	5.765445	2.000781	7.993069	0	2.21497	0	0.84968	0.926027	3.501863	4.803496	4.81902	3.680313	4.112337	0	0.978006	0.824452	4.105505	4.550667	1.792616
AC093752.	3063.373	2923.141	3883.633	2399.727	3103.173	2442.496	3561.86	3853.198	2727.951	2982.01	2479.386	2820.96	3202.483	2764.239	3177.541	2204.584	1923.429	2817.773	2507.87
AC093752.	16.33543	5.001952	10.99047	10.39293	21.04221	11.41887	13.59488	11.11232	0	6.724895	0	20.24172	11.30893	18.61441	17.6041	9.893422	14.36927	10.01147	9.859387
AC093755.	0	0	0	0	0	0	0	0.84968	0.926027	0	0	0	0	0	0	0	0	0	0
AC093762.	0	0	0	0	0	0	0	0	0	0	0	0	0	1.861441	0	0	0	0	0
AC093762.	0	0	0	0	0	0	0	0	2.334575	0	0	0	0	0	0	0	0	0	0
AC093763.	0	0	0	0	0	0	0	0	7.003726	0	0	0	0	0	0	0	0	0	0
AC093765.	4.804538	3.001171	0	1.039293	1.107485	1.141887	0.84968	0	2.334575	0	0	4.600391	0	3.722881	4.890029	0	0	1.820267	0
AC093766.	0	0	2.997401	0	0	0	2.549041	5.556162	0	0	0	0	3.084253	0	5.868035	0	0	3.640534	0
AC093768.	151.8234	178.0695	201.825	275.4126	145.0805	167.8574	250.6557	225.9506	66.5354	135.4586	104.8137	179.4153	151.1284	161.9453	195.6012	232.4954	325.3613	168.3747	155.0613
AC093770.	3.84363	6.002343	0.999134	0	3.322455	0	0	0	1.167288	0	0	0.920078	1.028084	0.93072	0	0	2.052753	0.910133	0
AC093772.	0.960908	12.00469	3.996535	9.353635	5.537425	0	8.496802	5.556162	4.669151	7.685594	9.638039	2.760235	10.28084	1.861441	0.978006	3.297807	2.052753	5.4608	0
AC093775.	19.21815	12.00469	26.97661	12.47151	9.967364	33.11472	12.7452	48.15341	0	17.29259	7.228529	11.04094	14.39318	16.75297	19.56012	12.36678	26.68578	16.3824	22.4077
AC093783.	0.960908	5.001952	2.997401	0	0	0	8.496802	0	0	0	0	1.840156	8.224675	0	1.956012	1.648904	3.079129	7.281067	6.274156
AC093787.	10.56998	4.001562	11.9896	0	14.3973	0	2.549041	16.66849	0	2.882098	9.638039	1.840156	0	7.445762	3.912023	3.297807	7.184634	7.281067	17.02985
AC093788.	258.4841	196.0765	272.7635	222.4087	389.8347	208.9653	296.5384	408.3779	330.3424	272.8386	177.099	239.2203	215.8977	283.8697	319.8079	249.8089	131.3762	323.0974	195.3951
AC093789.	1.921815	0	1.998267	0	0	1.141887	0	0	0	0	0	0	0	2.792161	0.978006	0	0	0	0
AC093797.	104.7389	113.0441	140.8778	73.78979	168.3377	93.63473	208.1717	242.6191	63.03354	115.2839	156.6181	96.60821	98.6961	160.0839	194.6232	58.53608	67.74083	78.27147	102.1791
AC093799.	0	0	0	0	0	0	0	0	0	0	7.228529	0	6.168506	0	0	0	0	0	0
AC093799.	65.34171	47.01835	90.92116	68.59332	124.0383	76.50643	140.1972	86.12051	157.5838	138.3407	179.5085	64.40548	76.07824	74.45762	60.63636	26.38246	41.05505	67.34987	78.8751
AC093801.	6.726353	4.001562	5.994802	5.196464	3.322455	3.425661	4.248401	4.630135	0	0	18.07132	1.840156	5.140422	0.93072	4.890029	1.648904	6.158258	10.9216	5.377848
AC093801.	0	0	0	0	0	0	0	0	0	0	0	0	0	2.792161	0	0	0	0	0
AC093802.	14.41361	5.001952	3.996535	25.98232	3.322455	7.993209	18.69297	12.96438	4.669151	43.23147	9.638039	1.840156	2.056169	4.653602	2.934017	0.824452	7.184634	25.48374	18.82247
AC093802.	0	0	0	4.157171	0	0	0	0	0	0	0	0	0	0	0	0	0	0.910133	0.896308
AC093809.	2.882723	1.00039	108.9056	1.039293	4.42994	0	2.549041	2.778081	7.003726	2.882098	2.40951	2.760235	4.112337	0	0.978006	0.824452	2.052753	2.7304	0.896308
AC093813.	0	0	0	0	0	0	0	0	0	0	0	0	1.028084	1.861441	0	0	0	0	0
AC093816.	0.960908	1.00039	0	1.039293	3.322455	6.851322	0	0.926027	0	0.960699	0	1.840156	0	1.861441	0.978006	0.824452	0	0	0
AC093817.	0	1.00039	0.999134	1.039293	0	1.141887	0	2.778081	0	1.921399	3.614265	0	4.112337	3.722881	0	0.824452	0	4.550667	0.896308
AC093821.	0	0	0	2.078586	0	0	0.84968	0	0	0	3.614265	0	0	0	0	0	0	0.910133	0
AC093826.	0	0	3.996535	0	0	0	0	0	0	0	0	0	0	0	0	0	0	0	0
AC093826.	9.609075	1.00039	8.992203	0	2.21497	1.141887	0	7.408216	0	4.803496	3.614265	1.840156	1.028084	0.93072	0	1.648904	0	2.7304	0
AC093827.	0	0	5.994802	0	0	1.141887	0.84968	3.704108	0	0	0	0	1.028084	1.861441	0	2.473355	3.079129	0	0
AC093827.	0.960908	0	0	1.039293	1.107485	0	0	0	1.167288	0	0	0	0	0	0	0	0	0	0.896308
AC093827.	22.10087	67.02616	110.9038	41.57171	56.48173	95.91851	53.52986	38.89314	152.9147	103.7555	162.6419	63.4854	59.62889	66.08114	95.84457	65.95615	75.95184	69.17014	31.37078
AC093831.	0	0	3.996535	0	0	0	0	0	3.704108	0	0	0	0	0	0	0	0	0	0
AC093835.	16.33543	40.01562	50.95582	11.43222	5.537425	3.425661	33.13753	23.15068	2.334575	10.56769	21.68559	9.200782	29.81445	25.12945	10.75806	17.31349	12.31652	25.48374	9.859387
AC093840.	0	1.00039	0	1.039293	0	2.283774	0.84968	0.926027	0	2.882098	0	0	3.084253	0	1.956012	0	0	0.910133	0
AC093843.	0	0	0	1.039293	0	4.567548	3.398721	0	0	0.960699	0	0	0	6.515042	0	1.648904	1.026376	0	1.792616
AC093844.	5.765445	1.00039	0.999134	4.157171	0	2.283774	3.398721	1.852054	4.669151	0.960699	0	0	4.112337	6.515042	1.956012	0	1.026376	5.4608	3.585232
AC093849.	18.25724	41.01601	27.97574	32.21808	75.30897	33.11472	61.17698	61.11778	12.84017	18.25329	0	31.28266	19.5336	12.09936	16.6261	17.31349	11.29014	44.59654	48.40063
AC093850.	0.960908	0	3.996535	1.039293	0	0	0.84968	0	0	0.960699	14.45706	0	2.056169	0	0	0	0	0	0
AC093853.	0	4.001562	0	0	0	0	0	0	0	0	0	0	0	0	0	0	0	0	0
AC093864.	9.609075	32.01249	32.97141	39.49313	4.42994	11.41887	22.94137	20.37259	40.85507	6.724895	22.89034	13.80117	27.75828	15.82225	20.53812	27.20691	20.52753	22.75334	33.16339
AC093865.	0	1.00039	13.98787	2.078586	0	9.135096	4.248401	11.11232	0	0	12.04755	0	6.168506	0	4.890029	1.648904	0	0	5.377848
AC093879.	0	7.002733	0.999134	7.275049	1.107485	1.141887	2.549041	0	0	2.882098	1.204755	0.920078	5.140422	4.653602	6.846041	0.824452	14.36927	10.01147	5.377848
AC093879.	0.960908	0	1.998267	1.039293	0	0	0.84968	0	0	0.960699	0	0	0	0.93072	0.978006	0.824452	3.079129	0.910133	1.792616
AC093890.	73.98988	60.02343	68.94022	42.611	84.16885	62.80379	62.87634	62.04381	77.04099	65.32755	45.78069	80.04681	71.96591	97.72563	92.91055	87.39189	45.16056	130.1491	71.70464

AC093895.	31.70995	27.01054	23.97921	46.76817	14.3973	1.141887	29.73881	21.29862	10.50559	14.41049	7.228529	19.32164	11.30893	20.47585	74.32844	60.18498	22.58028	78.27147	16.13354
AC093899.	7.68726	38.01484	2.997401	4.157171	7.752394	13.70264	8.496802	34.263	29.18219	24.01748	25.29985	6.440548	23.64594	8.376483	16.6261	12.36678	6.158258	8.191201	6.274156
AC093901.	15.37452	26.01015	12.98874	16.62868	26.57964	19.41208	16.14392	9.26027	46.69151	21.13538	18.07132	4.600391	19.5336	12.09936	23.47214	22.2602	18.47477	19.1128	20.61508
AC093903.	0	0	0	3.117878	0	0	4.248401	0	0	1.921399	0	0	4.112337	0.93072	3.912023	0	1.026376	0	0
AC093904.	0	0	0	0	0	1.141887	0	0.926027	0	0	0	0.920078	1.028084	0	0	0	1.026376	0	2.688924
AC093904.	0	0	0	0	0	0	0	0	0	0	0	3.680313	0	0	0	3.297807	4.105505	0	0
AC093908.	886.9177	1061.414	669.4196	2422.591	574.7847	737.659	1096.937	738.0435	311.6658	632.1401	609.606	813.3492	796.7654	887.9072	1037.664	1770.923	1598.068	567.0131	858.663
AC093909.	78.79442	39.01523	91.9203	25.98232	89.70628	82.21586	29.73881	104.6411	0	70.13105	26.50461	28.52243	16.44935	18.61441	26.40616	27.20691	35.92317	10.9216	7.170464
AC093909.	0	0	0	0	0	1.141887	0	0	0	0	0	0	3.084253	0	0	0	0	3.640534	0.896308
AC093909.	0.960908	0	0	0	0	0	0	0	0	1.921399	0	0.920078	0	0	0	3.297807	0	0.910133	0.896308
AC093909.	0	0	0	0	6.64491	0	0.84968	0	0	0	0	3.680313	9.252759	0	0.978006	0	0	0.910133	0.896308
AC093909.	2.882723	0	0	0	0	12.56076	0	0	0	8.646293	0	5.520469	15.42127	5.584322	0	0.824452	0	0.910133	0.896308
AC093909.	414.1511	493.1925	699.3936	219.2908	408.6619	698.8348	367.0619	357.4464	552.1271	698.4284	789.1144	349.6297	405.0652	381.5953	478.2448	410.577	388.9966	63.70934	96.80126
AC093915.	0.960908	0	0.999134	2.078586	0	0	7.647122	0	0	3.842797	0	1.840156	1.028084	0.93072	12.71408	2.473355	8.21101	0	0.896308
AC094019.	22.10087	35.01367	71.93762	45.72888	56.48173	4.567548	38.23561	62.04381	1.167288	22.09608	13.2523	74.52634	34.95487	26.99089	36.18622	15.66458	16.42202	27.304	69.01571
AC094085.	0	2.000781	14.987	3.117878	14.3973	9.135096	1.69936	9.26027	0	1.921399	6.023774	2.760235	5.140422	0	4.890029	4.122259	7.184634	0.910133	8.066772
AC094103.	0.960908	1.00039	1.998267	0	0	0	0	0.926027	0	0	0	0.920078	1.028084	4.653602	0	2.473355	1.026376	2.7304	2.688924
AC094104.	0	0	1.998267	0	0	0	0	0	0	0	0	0	0	0	0	0	0	0	0
AC095030.	0	0	1.998267	0	0	0	3.398721	0	2.334575	0	4.81902	2.760235	1.028084	0	0	3.297807	0	1.820267	1.792616
AC095031.	83.59896	298.1164	187.8371	286.8448	116.2859	304.8838	224.3156	145.3862	277.8145	316.0701	313.2363	127.8909	117.2016	214.9964	184.8431	198.6929	211.4335	129.2389	164.9207
AC095032.	182.5724	259.1011	185.8389	316.9843	130.6832	208.9653	313.532	248.1752	63.03354	146.987	93.97088	211.618	165.5216	235.4722	219.0733	261.3512	335.625	202.0496	209.7361
AC095032.	0	4.001562	5.994802	0	0	1.141887	1.69936	0	0	0	0	0.920078	1.028084	0	0.978006	0.824452	0	1.820267	0
AC095033.	0.960908	0	10.99047	6.235757	21.04221	10.27698	11.89552	13.89041	0	12.48909	20.48083	7.360626	9.252759	0.93072	4.890029	0.824452	7.184634	6.370934	10.7557
AC095038.	0	0	0	1.039293	0	0	0	0	0	2.882098	0	0.920078	0	3.722881	0	0	0	0	0
AC095038.	1.921815	4.001562	1.998267	1.039293	1.107485	18.27019	5.947762	0.926027	5.836439	2.882098	0	2.760235	0	0.93072	0.978006	4.946711	4.105505	0.910133	0.896308
AC095040.	0	0	0.999134	0	1.107485	0	2.549041	0	0	0	4.81902	1.840156	1.028084	0	0.978006	0	1.026376	0	0
AC095041.	0.960908	2.000781	0.999134	0	2.21497	0	0.84968	0	1.167288	0	3.614265	0.920078	0	1.861441	0	0	1.026376	0	1.792616
AC095050.	0.960908	0	0	0	0	0	0	0.926027	0	0	0	0	6.168506	0.93072	0	0.824452	0	0.910133	0
AC095052.	0	0	6.993936	0	1.107485	5.709435	0	0.926027	0	3.842797	0	0	0	0	0	0	0	0	0
AC095055.	172.0024	167.0652	264.7704	373.1061	209.3147	146.1615	198.8252	139.8301	145.911	175.808	57.82823	76.36649	140.8476	219.65	217.1173	204.4641	188.8532	192.0382	150.5797
AC095056.	0	0	0	0	0	0	3.398721	0	1.167288	0	0	0	0	0	0.978006	0	0	0	0
AC095057.	0.960908	0	3.996535	2.078586	0	0	0	1.852054	0	0	1.204755	8.280704	0	0.93072	1.956012	0	0	0	0.896308
AC095057.	0.960908	0	0	0	0	1.141887	0	0	0	0	0	0	0	0	0	0	0	0.910133	0
AC095057.	209.4778	216.0843	245.7869	283.7269	256.9365	224.9517	199.6749	220.3944	545.1234	257.4674	250.589	240.1404	258.0492	198.2434	224.9413	186.3261	193.9851	202.9598	161.3354
AC095060.	14.41361	24.00937	9.991337	5.196464	3.322455	4.567548	5.947762	8.334243	14.00745	7.685594	6.023774	11.96102	14.39318	8.376483	2.934017	14.84013	4.105505	2.7304	8.066772
AC095350.	3.84363	0	0	0	0	4.567548	0	0	0	0	0	2.760235	0	0	0	0	0	0	0
AC095350.	160.4716	97.03787	84.92636	120.558	87.49131	83.35775	200.5245	120.3835	7.003726	45.15286	26.50461	182.1755	160.3812	151.7074	132.0308	136.859	119.0596	99.20454	135.3425
AC096531.	0	0	0	0	0	0	0	0	1.167288	0	0	0	0	0	0.978006	0.824452	0	0	0
AC096533.	6.726353	5.001952	0	9.353635	21.04221	1.141887	10.19616	3.704108	0	2.882098	1.204755	16.56141	6.168506	8.376483	0.978006	3.297807	9.237386	13.652	4.48154
AC096536.	0	6.002343	1.998267	2.078586	5.537425	3.425661	4.248401	0.926027	10.50559	2.882098	2.40951	1.840156	10.28084	3.722881	0	0	1.026376	5.4608	11.652
AC096536.	0.960908	2.000781	0	1.039293	0	0	0.84968	0	0	0	0	4.600391	0	0.93072	0.978006	0.824452	2.052753	1.820267	0
AC096536.	0	0	0	1.039293	0	0	0.84968	0	0	0	0	0	0	0	0.978006	0	0	0	0
AC096537.	53.81082	11.00429	40.96448	14.5501	36.547	46.81737	16.14392	7.408216	150.5801	78.77734	60.23774	42.3236	54.48847	25.12945	33.2522	9.893422	18.47477	11.83173	25.09662
AC096537.	0	1.00039	3.996535	0	0	0	0	0	0	0	0	0	0	0	0	4.946711	2.052753	0.910133	1.792616
AC096540.	24.02269	26.01015	51.95495	23.90373	40.97694	22.83774	37.38593	54.6356	73.53913	45.15286	37.3474	11.04094	42.15146	46.53602	31.29619	28.85581	19.50115	29.12427	29.57816
AC096541.	12.4918	6.002343	14.987	12.47151	24.36467	20.55397	22.09169	8.334243	37.35321	12.48909	28.91412	21.1618	21.58977	8.376483	20.53812	3.297807	1.026376	30.0344	11.652
AC096543.	8.648168	11.00429	12.98874	5.196464	26.57964	4.567548	13.59488	6.482189	7.003726	10.56769	4.81902	7.360626	7.196591	5.584322	2.934017	4.122259	5.131881	13.652	9.859387
AC096543.	7.68726	1.00039	2.997401	1.039293	7.752394	7.993209	3.398721	8.334243	2.334575	1.921399	2.40951	3.680313	6.168506	0.93072	0.978006	9.893422	4.105505	1.820267	0
AC096558.	21.13997	19.00742	14.987	8.314342	6.64491	30.83095	12.7452	45.37532	105.0559	31.70308	38.55216	14.72125	29.81445	22.33729	17.6041	14.01568	20.52753	38.2256	12.54831
AC096558.	0.960908	0	0.999134	2.078586	0	0	3.398721	1.852054	5.836439	0	7.228529	2.760235	0	1.861441	0.978006	3.297807	5.131881	0	5.377848
AC096559.	0	0	0	0	0	0	0	0	0	0	0	0	0	0.93072	0	0	0	0.910133	0
AC096564.	52.84991	50.01952	42.96275	34.29666	47.62185	60.52001	30.58849	74.08216	40.85507	48.99566	38.55216	40.48344	47.29188	32.57521	44.01026	35.45143	47.21331	61.88907	24.20031
AC096564.	54.77173	64.02499	88.9229	136.1474	94.13622	68.51322	160.5896	82.41641	72.37184	102.7948	63.85201	138.9318	137.7633	80.97267	103.6686	109.6521	80.05735	101.0248	86.04556
AC096570.	0.960908	0	0	0	0	10.27698	0.84968	0	0	0	0	0	1.028084	4.653602	0	0.824452	0	0	0
AC096571.	42.27993	47.01835	18.98354	51.96464	33.22455	51.38492	23.79105	49.07943	7.003726	21.13538	8.433284	33.12282	53.46039	31.64449	37.16422	14.84013	30.79129	38.2256	53.77848
AC096576.	0.960908	0	0.999134	4.157171	0	0	0	0	0	1.921399	0	0	1.028084	0	0	3.297807	2.052753	0	0
AC096576.	1.921815	1.00039	0.999134	0	0	0	0.84968	0	0	2.882098	3.614265	5.520469	4.112337	1.861441	3.912023	4.122259	2.052753	5.4608	2.688924
AC096576.	0.960908	1.00039	3.996535	15.58939	6.64491	5.709435	7.647122	1.852054	0	5.764196	8.433284	3.680313	4.112337	1.861441	0.978006	9.06897	1.026376	0	0.896308
AC096577.	0	1.00039	0	0	0	2.283774	0	0	0	0	0	0.920078	0	0	0	0	6.158258	1.820267	0
AC096582.	0.960908	1.00039	0	2.078586	0	0	2.549041	1.852054	0	7.685594	7.228529	11.04094	1.028084	0	0	4.946711	4.105505	0	0
AC096582.	0	0	0	0	0	0	0	0	0	0	3.614265	0	0	0	0.978006	0	0	0	0
AC096582.	113.3871	201.0785	85.9255	73.78979	13.28982	134.7427	96.01387	109.2712	106.2232	105.6769	115.6565	116.8499	155.2407	134.9544	100.7346	46.99375	42.08143	121.9579	173.8837

AC096586.	70.14625	93.03631	135.8822	90.41847	83.06137	74.22266	123.2036	171.315	133.0708	135.4586	91.56137	69.00587	81.21867	166.5989	130.0748	106.3543	106.7431	81.91201	71.70464
AC096631.	0	0	0	1.039293	2.21497	0	0	0	0	0	1.204755	0	1.028084	0	0	0.824452	0	0	0
AC096631.	0	0	0	0	0	0	0	0	0	0	0	0	0	0	1.956012	0	0	0.910133	0
AC096632.	0	0	0	0	0	0	0	0	0	7.685594	0	0	0	0.93072	0.978006	0	0	3.640534	0
AC096633.	0.960908	0	0.999134	0	0	0	0	0.926027	0	6.724895	0	0	0	0	0	0	0	0	0
AC096637.	0	0	0	0	0	0	0	0	14.00745	0	0	0.920078	0	0	0.978006	0	0	0	0
AC096637.	6.726353	0	0	0	1.107485	0	5.098081	1.852054	0	0	4.81902	0	0	0.93072	3.912023	1.648904	0	3.640534	1.792616
AC096638.	1.921815	0	7.993069	4.157171	0	1.141887	0	6.482189	0	0.960699	0	3.680313	4.112337	0	1.956012	1.648904	0	1.820267	4.48154
AC096638.	1.921815	0	0	2.078586	0	0	0.84968	0	0	0	0	0	0	0	0	0	0	0	0
AC096639.	1.921815	0	0.999134	0	0	1.141887	7.647122	0	0	0	0	0	3.084253	1.861441	0.978006	0.824452	1.026376	0	0
AC096642.	286.3504	245.0957	262.7722	133.0295	334.4604	202.114	192.0277	277.8081	333.8443	333.3626	340.9456	256.7018	276.5547	271.7703	245.4795	206.9374	190.906	180.2064	224.9733
AC096649.	0.960908	2.000781	0	0	1.107485	0	0.84968	0	2.334575	0.960699	4.81902	0	1.028084	0	0.978006	0	1.026376	0.910133	1.792616
AC096656.	0	0	0	1.039293	0	0	0	0	0	0	0	0	1.028084	0	0	0.824452	0	0	0
AC096659.	0	0	5.994802	0	0	0	0	0	0	0	0	0	0	0	0	1.648904	0	0.910133	0
AC096664.	0	0	0	2.078586	0	0	0	0.926027	0	0	0	0	0	0	0	3.297807	0	0	0
AC096664.	0	0	0	0	0	0	0	0	0	0	0	0	0	0.93072	1.956012	0	0	0	0.896308
AC096666.	0	4.001562	0	0	0	0	0.84968	0	0	0	0	0	0	0	0	0	0	0	0.896308
AC096667.	172.9634	71.02772	79.93069	108.0864	67.55658	164.4317	93.46483	104.6411	123.7325	78.77734	54.21397	162.8538	98.6961	107.0328	98.77859	186.3261	138.5608	334.019	159.5428
AC096669.	0	0	0	0	2.21497	0	5.947762	0	0	0.960699	1.204755	0	0	0	1.956012	1.648904	0	0	0
AC096677.	259.445	379.148	413.6413	308.67	313.4182	486.4439	288.0416	423.1944	609.3242	541.8344	586.7156	310.0664	357.7734	430.9235	422.4985	311.6428	331.5195	318.5467	310.1226
AC096708.	4.804538	4.001562	1.998267	0	8.859879	0	3.398721	2.778081	0	0	0	0	1.028084	0.93072	0	0	3.079129	7.281067	0
AC096708.	0	0	1.998267	0	0	0	0	0	0	0	0	0	0	0	0.978006	0	0	0.910133	0
AC096708.	2.882723	3.001171	18.98354	10.39293	4.42994	2.283774	6.797442	5.556162	0	0	0	5.520469	6.168506	5.584322	2.934017	10.71787	4.105505	2.7304	2.688924
AC096708.	5.765445	8.003124	17.98441	9.353635	4.42994	1.141887	14.44456	5.556162	19.84389	16.33189	14.45706	7.360626	7.196591	27.92161	15.64809	8.244518	10.26376	1.820267	7.170464
AC096711.	0	0	3.996535	0	0	1.141887	0.84968	0.926027	0	0	0	5.520469	0	0	0	0	0	0	0
AC096711.	20.17906	11.00429	15.98614	1.039293	2.21497	1.141887	1.69936	2.778081	10.50559	7.685594	26.50461	5.520469	3.084253	6.515042	17.6041	51.11601	78.0046	0	0.896308
AC096711.	2.882723	0	3.996535	0	4.42994	27.40529	2.549041	0.926027	11.67288	0	0	11.04094	6.168506	11.16864	1.956012	0	1.026376	5.4608	4.48154
AC096713.	0	0	3.996535	0	4.42994	1.141887	0	0	11.67288	2.882098	0	0.920078	1.028084	7.445762	0	0.824452	2.052753	0	0
AC096719.	92.24712	116.0453	93.91856	106.0079	66.4491	44.53359	18.69297	52.78354	32.68406	26.89958	24.0951	14.72125	12.33701	67.94258	91.93255	34.62698	31.81766	29.12427	59.15632
AC096720.	5.765445	7.002733	6.993936	20.78586	11.07485	13.70264	21.24201	24.0767	4.669151	3.842797	7.228529	22.08188	16.44935	17.68369	16.6261	23.08465	4.105505	4.550667	9.859387
AC096720.	114.348	183.0715	125.8908	171.4833	102.9961	206.6815	129.1514	106.4931	203.1081	195.9826	138.5468	120.5302	135.7071	158.2225	111.4927	149.2258	136.508	137.4301	125.4831
AC096732.	0	0	0	0	0	0	0	0.926027	0	1.921399	0	0	0	0	0	0	0	0	0
AC096733.	1.921815	0	0	0	4.42994	0	0	0	0	0	0	0.920078	1.028084	1.861441	0.978006	3.297807	0	0	0
AC096733.	116.2698	202.0789	149.87	198.5049	186.0575	158.7223	152.9424	147.2383	200.7735	124.8909	136.1373	132.4913	142.9037	140.5388	139.8548	174.7838	169.3521	144.7112	162.2317
AC096734.	0	2.000781	0.999134	1.039293	1.107485	4.567548	2.549041	0	0	0.960699	1.204755	11.04094	7.196591	0	1.956012	0.824452	0	0.910133	4.48154
AC096734.	6.726353	5.001952	0.999134	10.39293	0	9.135096	15.29424	10.1863	23.34575	18.25329	9.638039	5.520469	2.056169	15.82225	1.956012	7.420066	7.184634	4.550667	17.02985
AC096741.	1.921815	0	0	0	0	0	0	0	1.167288	0.960699	0	0	0	0	0	0.824452	0	0	0
AC096741.	0	2.000781	0	0	0	0	2.549041	1.852054	0	0	0	0	2.056169	3.722881	0	0.824452	1.026376	0.910133	0.896308
AC096743.	0	2.000781	0	0	2.21497	0	0.84968	0	0	0	0	0	0	0	0.978006	0	0	0	2.688924
AC096745.	0	0	0	0	0	0	4.248401	0	15.17474	0	0	0.920078	0	0	0.978006	0.824452	4.105505	0	0.896308
AC096745.	97.05166	50.01952	113.9012	51.96464	53.15928	38.82416	96.01387	92.6027	66.5354	47.07426	44.57593	82.80704	63.74123	94.00275	131.0528	90.6897	106.7431	74.63094	104.868
AC096746.	0	1.00039	0	1.039293	0	0	0	0	0	0	0	0.920078	0	0	0	3.297807	3.079129	0	0.896308
AC096751.	0	2.000781	5.994802	7.275049	7.752394	4.567548	5.947762	2.778081	1.167288	3.842797	0	7.360626	1.028084	5.584322	6.846041	1.648904	6.158258	5.4608	8.963079
AC096751.	2.882723	25.00976	11.9896	5.196464	1.107485	10.27698	11.89552	0.926027	25.68033	4.803496	16.86657	7.360626	12.33701	8.376483	6.846041	0	6.158258	7.281067	12.54831
AC096759.	0.960908	0	0	0	0	0	0	0.926027	0	0	0	0.920078	0	0	0	0	0	0	0
AC096759.	0	0	0.999134	0	0	0	0	0.926027	0	0	0	0.920078	1.028084	0	0	0	0	0	0
AC096773.	0	0	1.998267	0	0	0	0	0	0	5.764196	0	1.840156	0	0	0	0	0	0	0.896308
AC096775.	0	0	0	0	4.42994	0	0	0	0	0	0	1.840156	0	0	0	0	0	3.640534	0
AC096861.	4.804538	6.002343	0.999134	0	0	0	0	0	0	0	0	3.680313	0	0	0	0	0	0.910133	0
AC096887.	46.12356	56.02186	68.94022	88.33989	63.12664	60.52001	44.18337	105.5671	87.54658	69.17035	53.00921	82.80704	78.13441	43.74385	60.63636	73.37621	74.92547	60.97894	73.49725
AC096887.	41.31902	84.0328	57.94975	39.49313	53.15928	63.94567	27.18977	74.08216	99.21946	125.8516	33.73314	58.88501	75.05016	95.86419	50.8563	61.83389	84.16285	56.42827	79.77141
AC096888.	8.648168	3.001171	3.996535	8.314342	0	1.141887	0	0	0	0	0	0	0	0.93072	0.978006	0.824452	0	0	0.896308
AC096915.	0	4.001562	0	0	1.107485	0	0	0	0	0	1.204755	0	0	0.93072	0.978006	0	0	0	0
AC096919.	48.04538	66.02577	30.97314	17.66798	50.94431	45.67548	29.73881	40.74519	59.53167	58.60265	86.74235	57.96493	50.37613	67.94258	50.8563	44.5204	75.95184	83.73227	44.8154
AC096920.	0	1.00039	3.996535	0	0	0	0.84968	0.926027	0	3.842797	0	1.840156	0	0	0	0	10.26376	0.910133	0
AC096921.	6.726353	2.000781	0	10.39293	12.18233	11.41887	1.69936	0	0	16.33189	0	5.520469	0	0.93072	3.912023	4.946711	3.079129	1.820267	0.896308
AC096921.	492.9456	399.1558	553.52	583.0432	396.4796	400.8023	635.5608	398.1916	815.9341	406.3758	587.9204	317.427	298.1445	442.0921	517.3651	535.8937	559.3751	620.711	305.641
AC096947.	11.53089	4.001562	13.98787	7.275049	2.21497	9.135096	16.14392	5.556162	21.01118	6.724895	7.228529	10.12086	3.084253	4.653602	5.868035	9.893422	2.052753	3.640534	4.48154
AC096992.	4.804538	1.00039	0.999134	1.039293	0	5.709435	1.69936	10.1863	2.334575	6.724895	0	3.680313	1.028084	2.792161	7.824047	4.946711	1.026376	0	7.170464
AC096992.	662.0653	366.1429	414.6405	428.1886	365.47	347.1336	349.2186	495.4245	384.0377	382.3583	360.2217	421.3958	431.7954	403.0019	363.8182	526.0003	506.0035	377.7054	278.7518
AC096996.	5.765445	0	0	0	0	0	0	0	0	0.960699	0	0	0	0	0	0	0	0	0
AC096996.	0	0	0	0	1.107485	0	0	0	0	0	0	0	0	0	0	0	1.026376	0	0

AC097059.	7.68726	1.00039	0	3.117878	0	0	3.398721	8.334243	3.501863	3.842797	0	3.680313	6.168506	4.653602	3.912023	0	3.079129	10.01147	2.688924
AC097059.	1.921815	3.001171	15.98614	2.078586	2.21497	6.851322	0.84968	0.926027	0	4.803496	0	4.600391	3.084253	10.23792	6.846041	6.595615	4.105505	8.191201	0
AC097063.	0	0	0	0	6.64491	0	0.84968	0	0	1.921399	0	2.760235	4.112337	0	0	0.824452	1.026376	0	0
AC097065.	0	0	0	0	3.322455	0	0	0.926027	0	0	0	0	1.028084	0	0	0	0	0	0
AC097065.	0	0	0	0	2.21497	4.567548	0	0	0	0	0	0	0	0	0.978006	0.824452	0	0	0
AC097103.	0	0	0	0	0	4.567548	0	0	0	0	1.204755	0	0	0	0.978006	0	0	0	0.896308
AC097103.	301.725	331.1292	396.6561	342.9666	282.4087	210.1072	265.9499	461.1615	264.9743	253.6246	286.7317	344.1093	357.7734	286.6619	253.3035	284.4359	224.7764	319.4568	340.597
AC097110.	0	0	0.999134	6.235757	0	1.141887	0	1.852054	2.334575	0.960699	0	0.920078	0	1.861441	0.978006	0	0	0	0
AC097173.	0.960908	0	0.999134	0	0	0	0	0	0	0	0	0.920078	2.056169	2.792161	0	0	0	1.820267	8.066772
AC097263.	0.960908	4.001562	5.994802	2.078586	3.322455	6.851322	0.84968	6.482189	12.84017	7.685594	20.48083	5.520469	6.168506	3.722881	1.956012	0.824452	5.131881	5.4608	0
AC097347.	20.17906	7.002733	15.98614	6.235757	19.93473	12.56076	6.797442	7.408216	22.17847	5.764196	8.433284	14.72125	16.44935	3.722881	3.912023	16.48904	11.29014	17.29253	17.92616
AC097358.	0	0	0	0	0	4.567548	0	0	0	0	0	0	5.140422	0	0	0	0	0	0
AC097358.	0	4.001562	0.999134	0	0	2.283774	0	1.852054	0	0	0	2.760235	4.112337	4.653602	0	0	1.026376	0.910133	7.170464
AC097359.	22.10087	11.00429	27.97574	4.157171	35.43952	18.27019	9.346483	42.59724	39.68778	35.54587	63.85201	18.40156	21.58977	12.09936	22.49413	13.19123	7.184634	22.75334	6.274156
AC097359.	119.1525	120.0469	159.8614	148.6189	74.20149	102.7698	150.3934	113.9013	119.0633	94.14853	128.9088	104.8889	100.7523	160.0839	87.04252	165.7148	130.3498	169.2848	144.3056
AC097359.	22.10087	32.01249	21.98094	4.157171	26.57964	45.67548	6.797442	45.37532	92.21573	29.78168	83.12809	15.64133	14.39318	15.82225	24.45015	9.893422	8.21101	12.74187	11.652
AC097372.	606.3327	548.214	478.585	503.0177	626.8365	393.951	572.6845	266.6958	707.3764	464.9784	552.9825	762.7449	806.0181	342.5051	377.5102	680.9972	818.0219	515.1355	669.542
AC097372.	0.960908	1.00039	0	0	1.107485	0	0.84968	0	0	0	0	0	0	0	0	0	4.105505	0	0
AC097372.	39.39721	21.0082	11.9896	6.235757	18.82724	15.98642	28.03945	26.85478	29.18219	12.48909	21.68559	18.40156	11.30893	32.57521	21.51613	19.78684	18.47477	6.370934	4.48154
AC097372.	3.84363	0	2.997401	1.039293	0	11.41887	1.69936	2.778081	18.6766	0.960699	1.204755	1.840156	3.084253	5.584322	0	0	1.026376	0.910133	8.066772
AC097374.	21.13997	18.00703	13.98787	22.86444	11.07485	17.12831	36.53625	20.37259	1.167288	16.33189	7.228529	9.200782	20.56169	13.9608	15.64809	7.420066	7.184634	7.281067	20.61508
AC097374.	17.29634	5.001952	4.995668	14.5501	15.50479	13.70264	10.19616	21.29862	4.669151	6.724895	3.614265	24.84211	20.56169	7.445762	5.868035	12.36678	37.97592	10.01147	25.09662
AC097375.	4.804538	1.00039	0	0	11.07485	2.283774	0.84968	0	0	0.960699	0	13.80117	1.028084	0	0.978006	0	5.131881	0	0
AC097375.	43.24084	43.01679	8.992203	5.196464	25.47215	11.41887	39.08529	0	50.19337	15.37119	4.81902	4.600391	24.67402	13.03008	11.73607	32.15362	25.65941	16.3824	7.170464
AC097376.	12.4918	7.002733	3.996535	5.196464	12.18233	10.27698	5.098081	3.704108	11.67288	3.842797	6.023774	5.520469	9.252759	3.722881	1.956012	0.824452	4.105505	3.640534	7.170464
AC097376.	831.185	799.312	1048.091	694.2476	974.5867	743.3684	830.9873	984.3667	1066.901	1229.695	1009.585	1041.529	1031.169	855.332	1024.95	737.8844	650.7225	898.3017	877.4855
AC097381.	0.960908	1.00039	0	1.039293	0	0	5.098081	0	4.669151	3.842797	9.638039	0.920078	3.084253	3.722881	0	4.946711	0	0	1.792616
AC097381.	3.84363	6.002343	0.999134	0	2.21497	0	1.69936	0	0	0	0	0	3.084253	0.93072	0	0.824452	4.105505	0.910133	0
AC097381.	39.39721	63.0246	45.96015	33.25737	17.71976	75.36454	37.38593	81.49038	16.34203	60.52405	71.08054	47.84407	76.07824	38.15953	54.76833	36.27588	70.81996	70.08027	27.78555
AC097382.	10.56998	7.002733	20.98181	24.94303	11.07485	1.141887	23.79105	10.1863	0	3.842797	1.204755	30.36258	15.42127	16.75297	20.53812	27.20691	11.29014	40.956	17.92616
AC097382.	4.804538	8.003124	17.98441	25.98232	17.71976	9.135096	28.88913	15.74246	0	24.01748	20.48083	15.64133	37.01104	10.23792	14.67009	37.92478	24.63303	37.31547	32.26709
AC097451.	0	0	0	0	2.21497	0	0	0	0	0	0	0	0	0	0	0	0	0	2.688924
AC097460.	140.2925	98.03826	55.95149	80.02554	17.71976	53.66869	138.4979	38.89314	45.52422	70.13105	50.5997	68.08579	33.92678	107.0328	77.26246	72.55176	70.81996	95.56401	145.2019
AC097460.	1.921815	2.000781	0	2.078586	2.21497	1.141887	0.84968	0	1.167288	0.960699	0	0	1.028084	0	0	3.297807	0	16.3824	8.963079
AC097460.	0	4.001562	8.992203	24.94303	6.64491	0	7.647122	4.630135	0	5.764196	8.433284	0.920078	6.168506	12.09936	1.956012	9.06897	9.237386	1.820267	3.585232
AC097461.	489.1019	808.3155	694.3979	666.1867	757.5197	353.985	1094.388	363.9286	465.7478	311.2666	279.5031	379.9923	432.8235	560.2936	568.2214	588.6586	567.5861	689.8811	710.7722
AC097467.	26.90541	21.0082	19.98267	22.86444	8.859879	2.283774	23.79105	30.55889	1.167288	12.48909	12.04755	26.68227	17.47743	13.03008	26.40616	9.06897	6.158258	6.370934	8.066772
AC097468.	23.06178	31.0121	42.96275	24.94303	5.537425	15.98642	9.346483	21.29862	21.01118	40.34937	39.75691	3.680313	11.30893	12.09936	6.846041	4.122259	1.026376	23.66347	10.7557
AC097468.	45.16265	46.01796	72.93676	99.77211	17.71976	55.95246	49.28145	44.4493	45.52422	44.19217	36.14265	36.80313	35.98295	25.12945	33.2522	62.65834	68.76721	48.23707	61.84525
AC097468.	154.7061	170.0664	157.8631	135.1081	255.829	178.1344	141.8966	168.5369	323.3387	224.8036	275.8889	157.3334	176.8305	172.1833	140.8328	158.2948	148.8246	135.6099	107.557
AC097474.	0	0	0	0	3.322455	0	0	0	0	0	0	0	1.028084	0	0	0	0	0	0
AC097478.	12.4918	7.002733	25.97748	1.039293	11.07485	18.27019	13.59488	2.778081	11.67288	33.62447	0	8.280704	8.224675	6.515042	8.802052	2.473355	22.58028	0.910133	3.585232
AC097478.	0.960908	0	5.994802	4.157171	0	0	5.947762	0	0	0	14.45706	6.440548	7.196591	4.653602	3.912023	0.824452	0	0	0
AC097478.	0	0	1.998267	5.196464	1.107485	0	7.647122	0	5.836439	2.882098	0	0	0	0	0	4.946711	1.026376	0.910133	0
AC097478.	4.804538	7.002733	17.98441	4.157171	46.51437	9.135096	3.398721	11.11232	16.34203	12.48909	18.07132	16.56141	3.084253	0.93072	8.802052	2.473355	0	2.7304	0.896308
AC097480.	0	0	0	0	0	0	2.283774	3.398721	18.52054	0	0	0	11.96102	17.47743	1.861441	0	1.648904	2.052753	0	0.896308
AC097484.	2.882723	2.000781	0	16.62868	1.107485	6.851322	4.248401	7.408216	0	2.882098	19.27608	3.680313	1.028084	0	0.978006	3.297807	1.026376	3.640534	7.170464
AC097487.	0.960908	0	0	0	0	0	0	0	0	0	0	0.920078	0	0	0	0	0	0	4.48154
AC097488.	0	3.001171	0.999134	0	0	5.709435	0.84968	8.334243	0	10.56769	12.04755	4.600391	0	2.792161	1.956012	0	1.026376	0	1.792616
AC097493.	22.10087	12.00469	5.994802	14.5501	19.93473	7.993209	10.19616	29.63287	37.35321	28.82098	28.91412	11.04094	19.5336	6.515042	6.846041	7.420066	12.31652	2.7304	5.377848
AC097493.	167.1979	141.0551	105.9082	73.78979	146.188	138.1683	110.4584	116.6794	71.20455	109.5197	87.94711	98.44837	79.1625	114.4786	114.4267	116.2477	136.508	119.2275	101.2828
AC097493.	11.53089	2.000781	1.998267	8.314342	1.107485	4.567548	6.797442	9.26027	0	5.764196	4.81902	8.280704	2.056169	0	3.912023	4.946711	6.158258	1.820267	6.274156
AC097493.	7.68726	4.001562	0	4.157171	0	4.567548	0.84968	0	8.171014	16.33189	132.523	0.920078	6.168506	0	0	0	4.105505	0	6.274156
AC097493.	2.882723	5.001952	0.999134	0	6.64491	4.567548	2.549041	5.556162	7.003726	5.764196	7.228529	4.600391	4.112337	3.722881	2.934017	7.420066	4.105505	1.820267	4.48154
AC097494.	0	0	0	0	0	0	0	0	0	0	0	0	4.112337	0	0	0	0	0	0
AC097495.	0	0	0	0	0	0	0	0	0	0	0	0	0	0	0	0	4.105505	0	0
AC097504.	0	0	4.995668	0	0	1.141887	2.549041	2.778081	0	0	0	1.840156	1.028084	0	3.912023	3.297807	2.052753	0	1.792616
AC097504.	49.96719	21.0082	37.96708	34.29666	35.43952	60.52001	17.84329	27.78081	51.36066	20.17468	28.91412	37.72321	30.84253	52.12034	65.52639	41.22259	30.79129	77.36134	48.40063
AC097505.	24.02269	22.00859	26.97661	48.84676	23.25718	12.56076	5.947762	32.41095	19.84389	18.25329	43.37118	7.360626	20.56169	9.307203	11.73607	20.6113	31.81766	2.7304	14.34093
AC097512.	0	1.00039	0	0	0	0	0	0	0	0	0	3.680313	0	0.93072	1.956012	0.824452	0	0	0
AC097515.	2.882723	6.002343	10.99047	3.117878	13.28982	6.851322	6.797442	2.778081	10.50559	7.685594	2.40951	11.04094	14.39318	6.515042	11.73607	6.595615	4.105505	7.281067	3.585232
AC097520.	0	0	0	0	0	0	3.398721	0	0	0	0	0	0	0	0	0	0	0	0

AC097522.	23.06178	3.001171	25.97748	3.117878	1.107485	21.69585	12.7452	12.03835	16.34203	3.842797	1.204755	11.96102	0	6.515042	2.934017	3.297807	1.026376	11.83173	5.377848
AC097523.	8.648168	8.003124	14.987	2.078586	7.752394	3.425661	2.549041	11.11232	2.334575	9.606993	10.84279	4.600391	18.50552	26.99089	7.824047	4.122259	17.4484	2.7304	8.066772
AC097523.	0.960908	0	2.997401	1.039293	1.107485	1.141887	0	0	0	6.724895	1.204755	0	1.028084	0.93072	4.890029	0	0	0	0
AC097527.	0.960908	0	0	0	0	0	0	0	0	0	0	1.840156	0	0	0	1.648904	2.052753	0	0
AC097532.	33.63176	18.00703	31.97228	16.62868	33.22455	12.56076	23.79105	38.89314	17.50932	7.685594	12.04755	15.64133	19.5336	26.06017	31.29619	13.19123	12.31652	10.01147	10.7557
AC097533.	0	2.000781	1.998267	1.039293	3.322455	4.567548	4.248401	1.852054	2.334575	1.921399	0	3.680313	1.028084	0	1.956012	3.297807	3.079129	2.7304	3.585232
AC097534.	91.28622	101.0394	74.93502	81.06484	86.38382	102.7698	143.596	99.08489	75.8737	96.06993	62.64725	133.4113	143.9318	80.97267	94.86656	69.25395	68.76721	173.8355	125.4831
AC097534.	1727.712	1865.728	1765.469	1119.318	1984.613	1889.823	1431.711	1655.736	1815.132	1586.114	1572.205	1703.985	1840.271	1547.788	1578.501	1256.465	1341.474	2050.531	1508.486
AC097537.	8.648168	12.00469	0.999134	1.039293	28.79461	15.98642	2.549041	22.22465	89.88116	49.95636	74.6948	2.760235	1.028084	18.61441	17.6041	0	2.052753	3.640534	1.792616
AC097634.	108.5826	91.03553	183.8406	40.53242	88.59879	111.9049	77.3209	233.3588	121.3979	157.5547	98.7899	102.1287	94.58376	107.0328	157.4589	42.04704	32.84404	78.27147	64.53417
AC097634.	0	0	0	0	0	0	1.69936	0	0	0	0	0	1.028084	0	0.978006	0	0	0	0
AC097634.	11.53089	13.00508	19.98267	10.39293	12.18233	14.84453	11.89552	4.630135	2.334575	0	0	23.00196	15.42127	8.376483	2.934017	3.297807	4.105505	8.191201	11.652
AC097635.	0.960908	4.001562	15.98614	6.235757	2.21497	5.709435	7.647122	3.704108	24.51304	5.764196	6.023774	11.04094	9.252759	2.792161	1.956012	4.946711	5.131881	11.83173	6.274156
AC097637.	26.90541	3.001171	6.993936	8.314342	16.61227	22.83774	26.34009	4.630135	45.52422	27.86028	36.14265	29.4425	13.3651	12.09936	6.846041	18.96239	11.29014	16.3824	4.48154
AC097638.	0.960908	0	0	0	2.21497	0	0	2.778081	0	0.960699	1.204755	0	1.028084	1.861441	0	0	2.052753	1.820267	0
AC097639.	104.7389	78.03045	138.8796	102.89	99.67364	129.0332	66.27506	80.56435	108.5578	75.89524	69.87578	118.6901	133.651	179.629	157.4589	235.7932	193.9851	288.5123	178.3653
AC097639.	0	0	0	0	0	0	0	0	0	0.960699	0	1.840156	0	0	0	4.122259	0	0.910133	0
AC097641.	1.921815	0	0.999134	6.235757	1.107485	0	3.398721	1.852054	0	1.921399	0	4.600391	4.112337	4.653602	0.978006	1.648904	0	0.910133	0.896308
AC097641.	0	0	0	0	0	0	0	0	0	0	1.204755	0	0	0	0.978006	0	0	0	0
AC097641.	73.98988	41.01601	53.95322	68.59332	44.2994	42.24982	132.5501	85.19449	87.54658	63.40615	56.62348	64.40548	86.35909	59.5661	73.35044	75.02512	55.42432	71.90054	105.7643
AC097652.	0	0	5.994802	30.13949	8.859879	7.993209	0	23.15068	191.4352	0	0	4.600391	7.196591	9.307203	0.978006	8.244518	13.34289	14.56213	5.377848
AC097658.	7.68726	0	0	4.157171	0	1.141887	0	0	7.003726	10.56769	3.614265	4.600391	3.084253	2.792161	0	0	2.052753	2.7304	0.896308
AC097658.	29.78813	25.00976	44.96101	17.66798	2.21497	13.70264	21.24201	21.29862	32.68406	19.21399	12.04755	23.92203	19.5336	17.68369	27.38416	31.32917	32.84404	24.5736	12.54831
AC097662.	304.6077	267.1042	327.7158	354.3988	223.712	350.5593	366.2122	327.8136	155.2493	223.8429	136.1373	218.0585	262.1615	283.8697	254.2815	251.4578	275.0688	291.2427	214.2176
AC097713.	0	0	0	0	0	0	0	0	0	0	0	1.840156	0	0	0	0.824452	0	0	0
AC097713.	1.921815	0	0	0	0	0	0	0	0	0	0	0	0	0	0	0	0	2.7304	0
AC097714.	0	2.000781	0	0	13.28982	0	0.84968	9.26027	3.501863	0	0	0	0	0	0	2.473355	0	0	0.896308
AC097716.	7.68726	1.00039	2.997401	0	0	0	5.947762	6.482189	0	2.882098	0	0.920078	0	1.861441	0	0	0	0	0
AC097717.	19.21815	31.0121	20.98181	8.314342	19.93473	21.69585	4.248401	19.44657	14.00745	25.93888	8.433284	18.40156	25.70211	9.307203	9.780058	6.595615	23.60665	18.20267	8.066772
AC097721.	0	0	1.998267	0	6.64491	0	1.69936	1.852054	0	1.921399	0	0	1.028084	0	0.978006	0.824452	1.026376	0.910133	0
AC097724.	0	6.002343	5.994802	0	0	0	3.398721	5.556162	0	3.842797	2.40951	0.920078	5.140422	0.93072	0	8.244518	1.026376	6.370934	1.792616
AC098476.	294.9986	152.0593	122.8934	121.5973	173.8751	247.7895	91.76547	255.5835	339.6807	211.3538	106.0184	144.4523	138.7914	127.5087	129.0968	74.20066	84.16285	119.2275	74.39356
AC098479.	16.33543	13.00508	20.98181	28.0609	18.82724	9.135096	33.13753	5.556162	32.68406	24.01748	9.638039	23.92203	19.5336	20.47585	12.71408	14.84013	19.50115	10.01147	23.30401
AC098483.	6.726353	4.001562	4.995668	5.196464	2.21497	0	0.84968	6.482189	0	2.882098	0	0	0	0	1.956012	0	1.026376	0	0.896308
AC098484.	291.155	247.0964	328.715	509.2535	345.5353	417.9306	440.984	292.6245	198.4389	155.6333	157.8229	264.0625	346.4644	423.4777	430.3226	449.3262	323.3085	395.908	396.1681
AC098484.	110.5044	159.0621	139.8787	142.3831	108.5335	138.1683	113.0075	122.2356	189.1006	179.6508	251.7938	128.811	88.41526	80.97267	98.77859	77.49847	63.63533	72.81067	73.49725
AC098484.	0	0	0	2.078586	0	0	0.84968	0	0	0	0	0	0	1.861441	0	0.824452	4.105505	0	0.896308
AC098487.	158.5497	229.0894	249.7834	155.8939	342.2128	214.6748	89.21643	161.1287	249.7996	309.3452	404.7976	155.4932	132.6229	192.6591	140.8328	161.5926	254.5413	150.172	134.4462
AC098583.	7.68726	7.002733	2.997401	0	2.21497	3.425661	3.398721	0	0	0	1.204755	4.600391	0	2.792161	3.912023	4.946711	11.29014	5.4608	6.274156
AC098590.	0	0	0	0	0	0	0	0	0	0	1.204755	0	0	0	0	0	1.026376	0	0
AC098591.	0	0	0	0	7.752394	1.141887	2.549041	6.482189	2.334575	5.764196	4.81902	1.840156	0	3.722881	7.824047	5.771163	4.105505	6.370934	2.688924
AC098591.	2.882723	3.001171	0	1.039293	0	1.141887	0.84968	0	5.836439	0.960699	0	0	1.028084	4.653602	0	0.824452	6.158258	0.910133	0.896308
AC098613.	336.3176	181.0707	426.6301	307.6307	269.1188	565.2341	130.8508	518.5751	219.4501	275.7207	232.5177	467.3997	306.3691	304.3455	370.6642	630.7056	419.7879	810.0187	538.6811
AC098614.	804.2796	565.2206	622.4603	593.4362	652.3086	369.9714	613.4691	573.2107	561.4654	363.1443	416.8452	507.8832	495.5367	679.4258	633.7478	699.1352	617.8785	935.6172	739.4541
AC098614.	0	1.00039	0	0	0	0	0	0	0	0	1.204755	0	0	0	0	0	0	0	0
AC098614.	36.51449	47.01835	45.96015	23.90373	42.08443	26.2634	23.79105	24.0767	106.2232	54.75986	56.62348	24.84211	41.12337	44.67457	76.28445	14.01568	18.47477	42.77627	35.85232
AC098617.	7.68726	12.00469	10.99047	9.353635	19.93473	9.135096	28.88913	16.66849	16.34203	5.764196	2.40951	27.60235	19.5336	13.9608	18.58211	20.6113	14.36927	13.652	14.34093
AC098650.	164.3152	150.0586	128.8882	172.5226	125.1458	191.837	160.5896	141.6821	166.9221	135.4586	112.0422	115.0098	154.2127	228.0265	196.5792	164.0659	177.5631	73.72081	134.4462
AC098679.	2424.37	1700.664	2727.635	2025.582	2487.411	1672.864	1485.241	1544.613	1115.927	2271.093	2228.797	2372.882	2570.211	685.0101	736.4384	1474.12	1518.01	1829.368	1855.357
AC098679.	493.9065	530.2069	765.3364	412.5992	734.2625	508.1397	270.1983	432.4546	280.1491	625.4152	437.326	641.2945	724.7995	82.83411	80.19648	319.8873	438.2627	166.5544	277.8555
AC098679.	0	0	7.993069	4.157171	0	2.283774	0	0	0	0.960699	13.2523	0.920078	14.39318	5.584322	1.956012	2.473355	1.026376	0.910133	0
AC098679.	42.27993	17.00664	30.97314	35.33595	58.6967	25.12151	27.18977	11.11232	35.01863	20.17468	60.23774	23.92203	23.64594	18.61441	22.49413	15.66458	24.63303	15.47227	11.652
AC098680.	0	0	0	0	0	0	0	0	0	0	0	0.920078	4.112337	0	0	0	0	0	0
AC098798.	0	0	0.999134	0	0	0	0.84968	0	0	0	9.638039	0.920078	1.028084	0.93072	0	3.297807	0	0	0.896308
AC098818.	4.804538	0	0	1.039293	0	1.141887	0.84968	0	0	0	0	0.920078	2.056169	0	0.978006	8.244518	5.131881	11.83173	8.066772
AC098818.	4.804538	7.002733	4.995668	3.117878	21.04221	6.851322	5.098081	7.408216	14.00745	9.606993	0	5.520469	5.140422	8.376483	6.846041	4.946711	7.184634	12.74187	5.377848
AC098820.	66.30262	85.03319	80.92983	28.0609	50.94431	57.09435	56.0789	109.2712	88.71387	29.78168	50.5997	74.52634	91.49951	59.5661	58.68035	64.30724	54.39794	58.24854	50.19325
AC098820.	0	0	0.999134	0	0	2.283774	3.398721	1.852054	0	0	1.204755	0.920078	1.028084	3.722881	5.868035	0.824452	2.052753	0.910133	0
AC098820.	6.726353	7.002733	5.994802	3.117878	23.25718	6.851322	9.346483	10.1863	25.68033	7.685594	12.04755	7.360626	24.67402	6.515042	11.73607	7.420066	26.68578	10.9216	12.54831
AC098824.	28.82723	39.01523	41.96361	39.49313	15.50479	13.70264	31.43817	34.263	5.836439	22.09608	16.86657	17.48149	35.98295	42.81313	50.8563	46.99375	56.45069	30.0344	38.54124
AC098826.	0	1.00039	0	1.039293	2.21497	0	0	5.556162	22.17847	4.803496	4.81902	2.760235	0	0	2.934017	0.824452	5.131881	0	1.792616
AC098827.	13.45271	0	10.99047	6.235757	4.42994	3.425661	5.098081	0	0	3.842797	0	8.280704	6.168506	4.653602	3.912023	5.771163	6.158258	8.191201	2.688924

AC098828.	3.84363	0	0	1.039293	0	0	0	0	0	0	0	0	0	0	0	0	0	0	0	0
AC098828.	16.33543	13.00508	14.987	18.70727	12.18233	10.27698	23.79105	11.11232	37.35321	52.83846	39.75691	29.4425	9.252759	36.29809	19.56012	9.893422	11.29014	25.48374	7.170464
AC098828.	5.765445	0	0	1.039293	4.42994	4.567548	0	0	0	0	0	0	0	0	0	0	0	0	0
AC098829.	11.53089	16.00625	31.97228	20.78586	23.25718	15.98642	37.38593	18.52054	0	5.764196	10.84279	28.52243	14.39318	20.47585	28.36217	23.9091	17.4484	11.83173	10.7557
AC098847.	0	1.00039	0	0	0	0	0	0.926027	0	2.882098	1.204755	0	1.028084	0	0	0	0	0	0
AC098847.	0	2.000781	0	0	0	0	0.84968	0	0	0	6.023774	0	0	0.93072	0	9.893422	8.21101	0	4.48154
AC098848.	0	3.001171	4.995668	4.157171	2.21497	9.135096	3.398721	5.556162	2.334575	0.960699	1.204755	5.520469	6.168506	0	0	1.648904	5.131881	0.910133	0
AC098848.	0	1.00039	0.999134	1.039293	0	0	0	0	0	0	0	0	0	0	0	0.978006	0	3.079129	0	0
AC098850.	2.882723	2.000781	0.999134	0	0	0	1.69936	0	2.334575	0.960699	0	5.520469	0	0	0	4.122259	1.026376	0.910133	5.377848
AC098850.	0	19.00742	0	23.90373	2.21497	0	0.84968	0	2.334575	1.921399	0	35.88305	22.61786	10.23792	18.58211	17.31349	16.42202	4.550667	7.170464
AC098850.	1.921815	0	10.99047	1.039293	0	0	0.84968	0	0	0	0	4.600391	1.028084	7.445762	0	0.824452	0	1.820267	0
AC098850.	19.21815	347.1355	9.991337	297.2377	17.71976	7.993209	11.04584	8.334243	45.52422	4.803496	0	448.9982	273.4704	188.9362	296.3358	400.6836	405.4186	97.38428	135.3425
AC098851.	106.6607	70.02733	174.8484	106.0079	102.9961	105.0536	169.936	129.6438	53.69524	162.3582	112.0422	178.4952	189.1675	94.93347	125.1847	75.84957	122.1388	98.29441	99.49018
AC098859.	0.960908	2.000781	0	0	0	1.141887	0	0	0	0.960699	0	1.840156	0	0.93072	0	0.824452	4.105505	0.910133	0.896308
AC098859.	1.921815	0	11.9896	0	2.21497	1.141887	2.549041	1.852054	0	2.882098	0	1.840156	3.084253	0	3.912023	0.824452	1.026376	0	0
AC098864.	361.3012	394.1538	384.6665	247.3517	375.4374	206.6815	316.9307	396.3396	262.6397	327.5984	444.5545	235.54	280.667	329.475	214.1833	342.1475	251.4622	227.5334	307.4336
AC098869.	0	0	0	0	0	0	0	0	0	0	0	0	0	0	0	2.473355	0	0	0
AC098869.	21.13997	5.001952	1.998267	21.82515	9.967364	13.70264	20.39233	12.03835	0	6.724895	8.433284	20.24172	16.44935	2.792161	27.38416	70.07841	28.73854	32.7648	30.47447
AC098872.	4.804538	0	0	0	0	0	0	0	0	0.960699	0	0	2.056169	0	0	0	0	0	0
AC098934.	65.34171	45.01757	45.96015	114.3222	119.6084	114.1887	60.3273	63.89587	136.5727	94.14853	62.64725	88.32751	104.8646	32.57521	58.68035	55.23827	104.6904	42.77627	60.05263
AC098934.	33.63176	61.02382	54.95235	70.67191	58.6967	42.24982	64.5757	40.74519	22.17847	47.07426	45.78069	36.80313	46.2638	45.60529	67.4824	43.69595	48.23968	40.956	59.15632
AC098934.	5.765445	9.003514	18.98354	4.157171	7.752394	17.12831	17.84329	14.81643	7.003726	2.882098	6.023774	11.04094	7.196591	4.653602	16.6261	6.595615	14.36927	16.3824	13.44462
AC098934.	0	0	0.999134	0	4.42994	0	0	0	4.669151	0	0	0	0	0	0	0.824452	0	0	0
AC098935.	0.960908	0	0	1.039293	0	0	0	0	0	0.960699	1.204755	0	1.028084	5.584322	0	3.297807	0	0	0
AC098935.	3.84363	1.00039	2.997401	0	0	1.141887	0	0	0	0	0	0.920078	0	0	3.912023	1.648904	0	0.910133	0
AC098936.	22.10087	12.00469	23.97921	31.17878	35.43952	5.709435	7.647122	16.66849	10.50559	12.48909	6.023774	22.08188	21.58977	8.376483	6.846041	17.31349	8.21101	1.820267	28.68185
AC098976.	2.882723	1.00039	0.999134	0	0	0	0	0.926027	9.338302	0	0	0	0	0	0	0	2.052753	3.640534	0
AC098976.	13.45271	5.001952	5.994802	8.314342	8.859879	4.567548	5.098081	5.556162	11.67288	7.685594	24.0951	5.520469	6.168506	3.722881	7.824047	15.66458	5.131881	18.20267	11.652
AC099060.	87.44259	131.0511	100.9125	28.0609	38.76197	36.54038	38.23561	71.30408	80.54285	37.46727	59.03299	79.12673	102.8084	64.2197	82.15249	56.88718	54.39794	61.88907	51.98586
AC099062.	16.33543	29.01132	29.97401	14.5501	1.107485	10.27698	17.84329	16.66849	26.84762	20.17468	21.68559	34.96297	37.01104	26.99089	18.58211	12.36678	11.29014	24.5736	34.95601
AC099063.	0	0	0.999134	1.039293	0	0	3.398721	0	0	0	0	0	1.028084	0	0	0	0	0.910133	0
AC099063.	0	1.00039	1.998267	1.039293	0	0	0	0	0	0.960699	0	0.920078	0	4.653602	4.890029	1.648904	1.026376	0	0.896308
AC099063.	66.30262	88.03436	102.9108	55.08252	29.90209	50.24303	61.17698	84.26846	114.3942	80.69874	101.1994	85.56728	81.21867	100.5178	74.32844	115.4233	83.13648	104.6653	60.05263
AC099066.	17.29634	6.002343	8.992203	8.314342	6.64491	6.851322	18.69297	29.63287	37.35321	15.37119	4.81902	16.56141	7.196591	7.445762	4.890029	14.84013	6.158258	0	2.688924
AC099066.	4.804538	7.002733	5.994802	2.078586	16.61227	1.141887	0	0.926027	15.17474	9.606993	26.50461	5.520469	3.084253	0.93072	4.890029	5.771163	0	1.820267	0.896308
AC099066.	1.921815	1.00039	0	0	1.107485	2.283774	4.248401	0	0	0	1.204755	5.520469	0	0	0	0.824452	1.026376	3.640534	6.274156
AC099313.	0	0	0	0	0	1.141887	0	0	0	3.842797	0	0	0	0	0	0	0	0	0
AC099314.	0.960908	3.001171	6.993936	6.235757	7.752394	0	3.398721	1.852054	0	3.842797	7.228529	4.600391	9.252759	0	1.956012	2.473355	3.079129	2.7304	1.792616
AC099328.	0	0	0	4.157171	1.107485	0	0	0	4.669151	3.842797	0	0	0	0	0	0.824452	1.026376	0	0
AC099329.	13.45271	6.002343	22.98007	11.43222	0	0	11.04584	6.482189	1.167288	0	0	7.360626	4.112337	5.584322	21.51613	3.297807	22.58028	0	1.792616
AC099329.	1114.653	564.2202	1605.608	448.9745	604.6868	468.1737	764.7122	708.4107	1629.534	633.1008	495.1543	770.1055	844.0573	917.6902	937.9076	616.69	561.4278	374.0648	374.6567
AC099335.	4.804538	4.001562	0.999134	2.078586	3.322455	5.709435	4.248401	4.630135	3.501863	4.803496	8.433284	1.840156	4.112337	6.515042	5.868035	4.946711	1.026376	2.7304	0.896308
AC099336.	0.960908	13.00508	0.999134	1.039293	5.537425	9.135096	8.496802	4.630135	14.00745	8.646293	4.81902	3.680313	8.224675	9.307203	0.978006	4.122259	12.31652	0	9.859387
AC099336.	37.47539	28.01093	7.993069	18.70727	34.33203	17.12831	8.496802	17.59451	11.67288	32.66377	28.91412	11.04094	10.28084	27.92161	20.53812	22.2602	32.84404	21.8432	17.92616
AC099340.	3.84363	0	1.998267	1.039293	0	0	1.69936	3.704108	0	1.921399	2.40951	0.920078	0	0.93072	0.978006	0.824452	0	2.7304	0
AC099342.	0	0	0	0	0	0	0	0	1.167288	0.960699	0	0	0	0.93072	0	0	0	0	0
AC099343.	178.7288	225.0878	215.8129	296.1984	223.712	456.7548	280.3945	285.2163	325.6733	419.8256	568.6443	216.2184	221.0381	295.0383	310.0278	334.7274	286.359	164.7341	208.8398
AC099343.	893.644	844.3295	1285.885	1138.026	942.4697	927.2122	1158.964	791.7531	1157.949	980.8739	1073.437	1129.856	1021.916	1310.454	1178.497	1055.298	950.4244	1271.456	1175.956
AC099344.	0.960908	1.00039	0.999134	11.43222	4.42994	9.135096	0.84968	3.704108	0	0	0	1.840156	0	0	3.912023	0	0	1.820267	0
AC099344.	7.68726	8.003124	0	11.43222	0	6.851322	2.549041	5.556162	0	0.960699	0	2.760235	4.112337	16.75297	0.978006	0.824452	1.026376	0.910133	8.963079
AC099344.	0.960908	3.001171	0	3.117878	0	0	0.84968	0	0	2.882098	16.86657	3.680313	1.028084	0	0	0	4.105505	0.910133	0
AC099398.	0	0	4.995668	0	0	0	0.84968	0.926027	0	0	0	0	1.028084	2.792161	1.956012	0	0	0.910133	0
AC099482.	0	0	0	1.039293	0	0	0	0	0	0	0	3.680313	0	0	5.868035	0	0	0.910133	0
AC099487.	0	0	0	1.039293	1.107485	0	0	0	0	0	0	0.920078	2.056169	0	0	0.824452	0	0	0
AC099489.	145.097	84.0328	109.9047	107.0472	124.0383	91.35096	175.0341	66.67395	454.0749	229.6071	320.4648	122.3704	125.4263	115.4093	44.01026	84.09409	81.08372	231.1739	146.9945
AC099489.	3.84363	6.002343	3.996535	0	7.752394	6.851322	6.797442	7.408216	0	13.44979	0	6.440548	15.42127	0	10.75806	1.648904	3.079129	8.191201	5.377848
AC099489.	7.68726	1.00039	1.998267	1.039293	1.107485	1.141887	11.04584	5.556162	0	2.882098	0	4.600391	3.084253	9.307203	0.978006	8.244518	3.079129	1.820267	4.48154
AC099494.	13.45271	2.000781	0	2.078586	2.21497	6.851322	3.398721	0	14.00745	0	4.81902	1.840156	1.028084	0.93072	0	0	4.105505	5.4608	0
AC099494.	47.08447	20.00781	1.998267	7.275049	34.33203	39.96605	25.49041	48.15341	77.04099	28.82098	18.07132	28.52243	47.29188	59.5661	47.92229	16.48904	8.21101	13.652	17.02985
AC099494.	0.960908	0	9.991337	0	0	0	8.496802	0	0	0	0	1.840156	2.056169	0	0.978006	0	0	0	0
AC099499.	0	0	9.991337	0	0	0	11.89552	0	0	0	0	2.760235	1.028084	0	0	0	0	0	0
AC099506.	0	6.002343	0	9.353635	0	1.141887	2.549041	3.704108	4.669151	0	0	0	0	3.722881	4.890029	0	0	0	0.896308

AC099508.	5.765445	0	3.996535	6.235757	0	2.283774	1.69936	0	0	0	0	0	1.028084	0	0	3.297807	5.131881	3.640534	4.48154
AC099509.	0	0	0	1.039293	0	0	0	0	0	0	0	0	0	0	0.978006	13.19123	22.58028	23.66347	7.170464
AC099509.	0	0	0	5.196464	0	0	0	0	0	0	0	0	0	0	0	0	0	0	0
AC099513.	16.33543	25.00976	14.987	24.94303	7.752394	20.55397	8.496802	11.11232	0	19.21399	21.68559	6.440548	5.140422	15.82225	4.890029	18.13794	8.21101	6.370934	6.274156
AC099518.	75.91169	74.02889	59.94802	61.31827	32.11706	55.95246	57.77826	111.1232	53.69524	53.79916	55.41872	78.20665	59.62889	73.5269	30.31818	84.09409	51.31881	100.1147	48.40063
AC099518.	31.70995	40.01562	31.97228	21.82515	39.86946	70.79699	19.54265	33.33697	30.34948	38.42797	24.0951	22.08188	32.8987	10.23792	19.56012	24.73355	37.97592	25.48374	19.71877
AC099518.	7.68726	1.00039	1.998267	0	0	1.141887	3.398721	0	0	0.960699	0	1.840156	4.112337	0	7.824047	1.648904	1.026376	0.910133	1.792616
AC099518.	15.37452	21.0082	19.98267	20.78586	31.00958	20.55397	15.29424	19.44657	9.338302	13.44979	21.68559	11.04094	6.168506	11.16864	12.71408	14.01568	15.39564	15.47227	7.170464
AC099518.	41.31902	23.00898	46.95928	41.57171	29.90209	27.40529	35.68657	37.96711	89.88116	66.28825	60.23774	51.52438	57.57272	68.8733	27.38416	23.08465	39.0023	38.2256	27.78555
AC099520.	9.609075	2.000781	2.997401	2.078586	0	2.283774	5.098081	8.334243	0	0	0	1.840156	4.112337	4.653602	0	0	1.026376	7.281067	0.896308
AC099521.	0	0	0	0	0	0	0	0.926027	0	0	0	0.920078	0	0	0	0	0	0	0
AC099521.	76.8726	93.03631	71.93762	77.94696	59.80419	71.93888	123.2036	89.82462	75.8737	76.85594	86.74235	53.36454	97.66802	93.07203	66.5044	74.20066	129.3234	81.00187	161.3354
AC099521.	12.4918	5.001952	11.9896	11.43222	5.537425	10.27698	9.346483	15.74246	4.669151	0.960699	18.07132	21.1618	7.196591	0.93072	8.802052	9.06897	1.026376	14.56213	13.44462
AC099522.	9.609075	11.00429	8.992203	5.196464	6.64491	5.709435	2.549041	7.408216	2.334575	20.17468	9.638039	9.200782	4.112337	9.307203	4.890029	6.595615	6.158258	11.83173	10.7557
AC099522.	147.0189	98.03826	139.8787	50.92535	109.641	103.9117	111.3081	106.4931	213.6137	145.0656	310.8268	110.4094	118.2297	155.4303	136.9208	96.46086	103.664	172.9253	106.6606
AC099524.	139.3316	84.0328	140.8778	77.94696	116.2859	91.35096	29.73881	176.8712	321.0041	198.8647	238.5415	146.2924	138.7914	86.55699	94.86656	104.7054	93.40024	115.5869	130.861
AC099535.	0	7.002733	1.998267	0	1.107485	4.567548	9.346483	0.926027	0	0	12.04755	0.920078	1.028084	7.445762	0.978006	1.648904	4.105505	1.820267	0.896308
AC099541.	0	0	0	0	0	0	0	0	0	0	7.228529	0	0	0	0.978006	1.648904	0	0	0
AC099542.	1.921815	8.003124	29.97401	20.78586	16.61227	10.27698	19.54265	37.04108	16.34203	30.74238	8.433284	14.72125	12.33701	2.792161	16.6261	24.73355	18.47477	7.281067	24.20031
AC099542.	0	0	0	0	0	0	1.69936	0	0	0	0	0	3.084253	0	0	0	0	0	0
AC099548.	0	1.00039	0	1.039293	0	1.141887	1.69936	0	0	0	0	0.920078	2.056169	0	3.912023	0	0	0	0
AC099548.	77.83351	107.0418	78.93156	84.18271	85.27634	75.36454	88.36674	90.75065	84.04472	79.73804	98.7899	107.6492	97.66802	127.5087	114.4267	123.6678	102.6376	151.9923	116.52
AC099550.	0.960908	22.00859	10.99047	2.078586	1.107485	4.567548	11.89552	3.704108	7.003726	4.803496	0	4.600391	4.112337	2.792161	2.934017	4.122259	1.026376	9.101334	10.7557
AC099552.	0	0	0	0	0	0	0	0	0	0	2.40951	0	0	0	0	0	0	0	0
AC099552.	0	0	0	0	2.21497	0	0.84968	0	0	0	0	0	0	0	0	0	0	0	0
AC099552.	1.921815	0	1.998267	0	1.107485	1.141887	0.84968	0	0	0	12.04755	0.920078	1.028084	0.93072	0.978006	4.946711	3.079129	0	0
AC099554.	1.921815	1.00039	0	0	0	7.993209	5.098081	0	7.003726	0.960699	0	0	0	1.861441	3.912023	0.824452	1.026376	3.640534	0
AC099560.	3.84363	0	0	0	1.107485	0	0.84968	0	0	1.921399	0	5.520469	0	0	0	1.648904	7.184634	10.01147	1.792616
AC099560.	45.16265	62.02421	37.96708	18.70727	36.547	44.53359	15.29424	36.11505	37.35321	35.54587	39.75691	25.76219	17.47743	52.12034	31.29619	704.9063	755.4129	38.2256	22.4077
AC099566.	0	1.00039	0.999134	0	0	0	0	0	0	0	0	0	0	1.861441	0	0	0	0	0
AC099567.	0	0	0	0	0	0	0	2.778081	0	0	0	0	0	0	0	0.824452	0	0	0
AC099568.	54.77173	56.02186	62.94542	70.67191	21.04221	27.40529	19.54265	81.49038	164.5876	89.34503	79.51382	23.00196	12.33701	46.53602	41.07624	66.7806	74.92547	12.74187	11.652
AC099568.	23.06178	35.01367	45.96015	12.47151	14.3973	39.96605	9.346483	10.1863	38.5205	30.74238	55.41872	23.00196	32.8987	12.09936	32.27419	10.71787	27.71216	36.40534	32.26709
AC099654.	0	0	0	0	0	0	0	0	0	0	0	0	0	0	0	0	0	0	3.585232
AC099654.	0.960908	0	0	4.157171	0	1.141887	7.647122	0	0	0	0	0	1.028084	0	1.956012	0	0	0	0
AC099654.	0.960908	0	0.999134	0	0	0	4.248401	0.926027	0	2.882098	0	0	0	0	0	0.824452	0	0	2.688924
AC099654.	0	0	0	0	0	0	0.84968	0	0	0	0	0.920078	0	0	0	0	0	0	0
AC099654.	0	0	0	0	0	0	5.098081	0	0	0	0	3.680313	0	0	0	0	0	0	0
AC099654.	0	0	0	0	0	0	0.84968	0	0	0	0	0.920078	0	0	0	0	0	0	0
AC099654.	41.31902	30.01171	5.994802	17.66798	3.322455	1.141887	33.98721	0.926027	4.669151	0	9.638039	26.68227	28.78636	11.16864	12.71408	0.824452	0	6.370934	6.274156
AC099654.	1.921815	7.002733	0	0	0	0	3.398721	0.926027	0	0	0	0.920078	0	1.861441	0	0	0	0	0
AC099654.	0	0	0	0	0	0	0.84968	0.926027	0	0	0	0	0	0	0	0	0	0	0
AC099667.	8.648168	17.00664	44.96101	35.33595	18.82724	17.12831	56.92858	16.66849	8.171014	20.17468	16.86657	26.68227	27.75828	28.85233	47.92229	30.50472	26.68578	17.29253	18.82247
AC099669.	22.10087	21.0082	55.95149	35.33595	37.65449	42.24982	35.68657	10.1863	16.34203	32.66377	32.52838	60.72516	29.81445	35.36737	41.07624	31.32917	22.58028	40.956	22.4077
AC099673.	0	1.00039	0	0	0	0	1.69936	0	0	0	0	0	5.140422	0	0.978006	0	0	0	1.792616
AC099676.	390.1285	256.1	298.741	345.0452	270.2263	232.9449	278.6951	213.9122	452.9076	314.1487	275.8889	244.7408	259.0773	274.5625	303.1818	405.6303	376.6801	629.8123	385.4124
AC099677.	179.6897	214.0836	414.6405	234.8802	277.9787	155.2966	424.8401	231.5068	7.003726	64.36685	62.64725	352.39	377.307	162.8761	171.151	193.7462	257.6204	223.8928	353.1453
AC099677.	5.765445	20.00781	11.9896	8.314342	8.859879	6.851322	8.496802	6.482189	0	8.646293	1.204755	6.440548	14.39318	12.09936	6.846041	5.771163	1.026376	2.7304	8.963079
AC099677.	0	0	0	4.157171	0	0	0	0	0	0.960699	0	0	0	0	0	1.648904	0	0	0
AC099681.	15.37452	25.00976	17.98441	27.02161	6.64491	13.70264	20.39233	24.0767	10.50559	0	31.32363	7.360626	9.252759	25.12945	3.912023	32.97807	13.34289	33.67494	24.20031
AC099681.	2.882723	22.00859	2.997401	9.353635	6.64491	2.283774	6.797442	13.89041	39.68778	4.803496	6.023774	17.48149	13.3651	6.515042	7.824047	12.36678	19.50115	13.652	23.30401
AC099681.	34.59267	14.00547	24.97834	38.45383	11.07485	33.11472	21.24201	32.41095	22.17847	6.724895	16.86657	35.88305	14.39318	38.15953	30.31818	28.03136	24.63303	26.39387	31.37078
AC099684.	0	5.001952	0	1.039293	7.752394	1.141887	1.69936	0	14.00745	11.52839	8.433284	1.840156	4.112337	1.861441	1.956012	11.54233	1.026376	0	0.896308
AC099684.	3.84363	8.003124	0.999134	3.117878	23.25718	2.283774	1.69936	4.630135	9.338302	15.37119	9.638039	6.440548	1.028084	1.861441	17.6041	1.648904	2.052753	13.652	2.688924
AC099684.	0	1.00039	0	0	0	0	0	0	0	0	0	0	1.028084	0	0	0	0	0	0
AC099687.	80.71623	74.02889	26.97661	68.59332	23.25718	22.83774	60.3273	12.03835	1.167288	12.48909	6.023774	15.64133	21.58977	41.88241	29.34017	116.2477	73.89909	5.4608	43.91909
AC099689.	0	4.001562	0	1.039293	1.107485	0	0	0	18.6766	7.685594	16.86657	0.920078	0	0.93072	5.868035	6.595615	0	2.7304	0
AC099753.	4.804538	2.000781	0	6.235757	0	9.135096	0.84968	0	0	0.960699	4.81902	0	0	0	3.912023	2.473355	1.026376	0	0.896308
AC099754.	0	5.001952	8.992203	0	2.21497	2.283774	1.69936	3.704108	4.669151	12.48909	4.81902	5.520469	7.196591	2.792161	3.912023	4.946711	2.052753	0.910133	3.585232
AC099777.	0	0	0	1.039293	0	0	4.248401	3.704108	1.167288	1.921399	0	0.920078	0	8.376483	0	13.19123	0	0.910133	1.792616
AC099778.	379.5585	419.1636	687.404	267.0982	468.4661	331.1472	268.499	555.6162	731.8894	666.7253	858.9902	507.8832	399.9248	449.5379	501.717	343.7964	374.6273	454.1566	417.6795
AC099778.	6.726353	8.003124	8.992203	6.235757	8.859879	6.851322	3.398721	9.26027	0	9.606993	0	11.96102	6.168506	5.584322	0.978006	2.473355	5.131881	5.4608	8.963079

AC099782.	0	0	0	0	0	1.141887	3.398721	0.926027	0	0	0	0	1.028084	0	0	0	0	0	0
AC099789.	0	1.00039	0	0	0	0	0	0	0	0	0	0	1.028084	0	0	0	0	0	0.896308
AC099791.	0	0	0	0	0	1.141887	0	0	1.167288	3.842797	0	3.680313	1.028084	0	0	0	0	0.910133	0
AC099791.	47.08447	66.02577	64.94369	16.62868	95.2437	54.81058	50.13113	76.86024	84.04472	93.18783	133.7278	64.40548	77.10633	54.9125	69.43841	32.97807	44.13418	38.2256	30.47447
AC099792.	503.5155	1044.408	1200.959	544.5894	578.1071	771.9156	1056.153	735.2655	451.7404	1569.783	1714.366	696.4992	858.4505	470.9445	567.2434	475.7087	578.8762	339.4798	559.2962
AC099793.	2.882723	2.000781	0	0	1.107485	0	0	0	0	0	0	0.920078	1.028084	0	0.978006	4.122259	0	0	0
AC099794.	19.21815	19.00742	4.995668	39.49313	23.25718	19.41208	20.39233	37.96711	8.171014	18.25329	34.93789	31.28266	38.03912	15.82225	24.45015	14.01568	19.50115	31.85467	27.78555
AC099794.	0	5.001952	2.997401	0	0	6.851322	0	0	10.50559	0	1.204755	1.840156	3.084253	1.861441	0.978006	0.824452	1.026376	0	5.377848
AC099795.	30.74904	26.01015	84.92636	7.275049	17.71976	27.40529	22.09169	23.15068	30.34948	19.21399	3.614265	32.20274	47.29188	29.78305	24.45015	33.80253	15.39564	20.93307	21.51139
AC099795.	4.804538	20.00781	17.98441	1.039293	8.859879	6.851322	5.947762	14.81643	19.84389	11.52839	4.81902	13.80117	10.28084	8.376483	8.802052	8.244518	7.184634	1.820267	4.48154
AC099796.	0	0	0	0	0	0	0	0	0	0	0	0	0	0	0	0.824452	0	0.910133	0
AC099796.	0	0	0	0	0	0	0	0	7.003726	0	0	0	0	0	0	0	0	0	0
AC099804.	10.56998	4.001562	11.9896	11.43222	8.859879	5.709435	6.797442	3.704108	7.003726	0.960699	1.204755	10.12086	4.112337	0.93072	3.912023	7.420066	0	6.370934	3.585232
AC099805.	3.84363	4.001562	6.993936	17.66798	19.93473	7.993209	5.098081	3.704108	1.167288	6.724895	28.91412	11.04094	17.47743	3.722881	13.69208	1.648904	0	13.652	2.688924
AC099811.	16.33543	6.002343	20.98181	23.90373	17.71976	6.851322	38.23561	25.92876	7.003726	26.89958	6.023774	14.72125	20.56169	17.68369	17.6041	28.03136	13.34289	17.29253	11.652
AC099811.	5.765445	10.0039	10.99047	32.21808	6.64491	7.993209	11.89552	7.408216	4.669151	2.882098	25.29985	15.64133	13.3651	13.03008	15.64809	23.9091	10.26376	23.66347	34.95601
AC099811.	7.68726	16.00625	13.98787	29.1002	2.21497	5.709435	24.64073	26.85478	0	20.17468	20.48083	25.76219	17.47743	12.09936	24.45015	28.03136	6.158258	22.75334	8.066772
AC099811.	0.960908	9.003514	8.992203	11.43222	8.859879	5.709435	24.64073	5.556162	9.338302	3.842797	1.204755	29.4425	5.140422	10.23792	12.71408	16.48904	10.26376	1.820267	8.963079
AC099811.	16.33543	18.00703	29.97401	36.37525	38.76197	2.283774	25.49041	23.15068	11.67288	7.685594	26.50461	31.28266	25.70211	16.75297	10.75806	21.43575	31.81766	11.83173	6.274156
AC099850.	162.3934	129.0504	241.7903	118.4794	117.3934	142.7359	141.8966	149.0904	127.2344	114.3232	161.4372	165.6141	150.1003	151.7074	174.085	105.5298	117.0069	164.7341	173.8837
AC099850.	94.16894	61.02382	79.93069	106.0079	75.30897	52.5268	71.37314	93.52873	1.167288	31.70308	30.11887	64.40548	61.68506	106.1021	88.02052	218.4797	112.9014	382.256	206.1508
AC100756.	0.960908	0	0	0	0	0	0	1.69936	3.704108	0	0	0	0	0.93072	0	0	0	0	0
AC100756.	57.65445	63.0246	22.98007	144.4617	32.11706	38.82416	38.23561	30.55889	28.01491	20.17468	31.32363	37.72321	44.20763	34.43665	49.8783	33.80253	34.89679	47.32694	47.50432
AC100756.	5.765445	1.00039	1.998267	1.039293	5.537425	0	0.84968	4.630135	4.669151	5.764196	3.614265	2.760235	3.084253	0.93072	0	1.648904	1.026376	0.910133	0
AC100757.	0	3.001171	7.993069	5.196464	2.21497	3.425661	0	0	0	0	0	4.600391	0	0	0	0.824452	4.105505	0.910133	1.792616
AC100757.	2.882723	30.01171	31.97228	5.196464	8.859879	14.84453	12.7452	8.334243	15.17474	0	0	27.60235	12.33701	6.515042	7.824047	20.6113	1.026376	10.9216	6.274156
AC100757.	10.56998	9.003514	9.991337	14.5501	6.64491	11.41887	11.89552	0	1.167288	6.724895	9.638039	23.92203	12.33701	17.68369	17.6041	15.66458	7.184634	21.8432	12.54831
AC100763.	0	0	0	0	0	1.141887	0	0	0	0	0	0.920078	2.056169	0	0	0.824452	0	0	0
AC100767.	0	0	2.997401	0	0	0	0.84968	0	0	0	0	0.920078	0	0	0	0	0	0	0
AC100768.	0	0	0	0	3.322455	0	0	0	0	0	0	0	0	0	0.978006	0	0	0	0.896308
AC100771.	3.84363	5.001952	1.998267	0	1.107485	0	0.84968	0	12.84017	1.921399	9.638039	4.600391	6.168506	0.93072	0.978006	0.824452	1.026376	0.910133	0.896308
AC100771.	68.22443	68.02655	121.8943	45.72888	33.22455	41.10793	76.47122	52.78354	133.0708	53.79916	39.75691	51.52438	60.65698	92.14131	35.20821	32.15362	41.05505	32.7648	53.77848
AC100773.	0	0	1.998267	0	0	0	0	0	0	0	0	0	0	0	0	0	0	0	0
AC100774.	18.25724	31.0121	36.96795	22.86444	24.36467	6.851322	28.03945	28.70684	0	5.764196	7.228529	32.20274	18.50552	23.26801	33.2522	14.84013	6.158258	10.01147	10.7557
AC100778.	0	0	0	0	0	0	1.69936	0	0	0	0	0	0	0	0	0	0	0	0
AC100778.	80.71623	54.02108	76.93329	71.7112	33.22455	63.94567	77.3209	121.3095	25.68033	69.17035	49.39495	70.84602	71.96591	67.01186	66.5044	100.5831	55.42432	94.65388	82.46033
AC100778.	0	0	0	0	2.21497	0	0	0	0	0	0	0	0	0	3.912023	0.824452	0	0	0
AC100778.	19.21815	19.00742	28.97488	6.235757	3.322455	13.70264	19.54265	9.26027	35.01863	2.882098	20.48083	16.56141	17.47743	16.75297	11.73607	15.66458	21.5539	31.85467	13.44462
AC100781.	0	0	0	0	0	0	0	0	0	0	0	0	4.112337	0	0	0	0	0	0
AC100782.	4.804538	2.000781	25.97748	9.353635	0	12.56076	25.49041	11.11232	0	6.724895	0	4.600391	9.252759	8.376483	13.69208	4.946711	5.131881	11.83173	5.377848
AC100784.	0	0	0	8.314342	0	0	0	0	0	0	0	0	1.028084	0	0	0	0	0	0
AC100786.	2.882723	18.00703	7.993069	0	3.322455	5.709435	5.098081	6.482189	10.50559	0	0	0.920078	5.140422	0.93072	5.868035	1.648904	3.079129	6.370934	6.274156
AC100788.	60.53717	14.00547	44.96101	11.43222	32.11706	12.56076	61.17698	47.22738	29.18219	29.78168	26.50461	55.20469	76.07824	21.40657	22.49413	11.54233	26.68578	6.370934	20.61508
AC100788.	23.06178	22.00859	39.96535	33.25737	87.49131	38.82416	67.97442	53.70957	49.02608	115.2839	108.4279	78.20665	96.63993	15.82225	35.20821	47.81821	58.50345	31.85467	45.71171
AC100791.	0	0	0	0	0	0	3.398721	0	0	0	9.638039	0.920078	0	0.93072	0	0	0	0	0
AC100791.	2.882723	3.001171	1.998267	0	0	3.425661	2.549041	0	14.00745	0	0	3.680313	2.056169	0	2.934017	6.595615	0	0.910133	0
AC100793.	0	13.00508	18.98354	0	2.21497	2.283774	5.947762	1.852054	7.003726	3.842797	0	4.600391	6.168506	7.445762	4.890029	0.824452	9.237386	1.820267	15.23724
AC100793.	76.8726	79.03084	71.93762	75.86837	104.1036	49.10114	45.88273	67.59997	142.4091	101.8341	84.33284	78.20665	85.331	57.70466	40.09824	69.25395	92.37386	61.88907	58.26002
AC100793.	416.073	351.137	434.6231	347.1238	369.9	372.2552	398.5	373.1889	186.766	335.284	275.8889	394.7136	454.4133	414.1705	337.412	386.6679	300.7282	261.2083	335.2192
AC100793.	334.3958	211.0824	316.7254	159.0118	269.1188	295.7487	196.2761	362.0766	961.8451	411.1793	375.8835	223.579	212.8135	276.4239	243.5235	79.14738	146.7718	100.1147	48.40063
AC100797.	0	9.003514	7.993069	3.117878	0	6.851322	8.496802	12.96438	8.171014	10.56769	28.91412	9.200782	11.30893	26.99089	10.75806	22.2602	22.58028	0	0
AC100797.	0	3.001171	0.999134	1.039293	4.42994	0	0.84968	3.704108	1.167288	7.685594	21.68559	0.920078	1.028084	0	8.802052	4.946711	1.026376	0.910133	0.896308
AC100800.	15.37452	2.000781	24.97834	0	0	1.141887	1.69936	0	7.003726	1.921399	0	0	0	3.722881	2.934017	3.297807	0	0.910133	1.792616
AC100801.	0	0	3.996535	0	0	2.283774	0	0	0	0	4.81902	0	5.140422	1.861441	0.978006	0	0	0.910133	0
AC100802.	3.84363	2.000781	1.998267	0	4.42994	0	2.549041	9.26027	2.334575	2.882098	0	2.760235	1.028084	1.861441	3.912023	0	0	0	0
AC100803.	8.648168	20.00781	12.98874	8.314342	7.752394	15.98642	4.248401	12.96438	44.35693	5.764196	15.66181	14.72125	9.252759	8.376483	13.69208	23.08465	13.34289	8.191201	32.26709
AC100803.	68.22443	113.0441	49.95668	22.86444	169.4452	89.06719	16.14392	45.37532	180.9296	135.4586	255.408	141.692	98.6961	96.79491	62.59237	44.5204	97.50574	46.4168	71.70464
AC100803.	121.0743	126.0492	135.8822	102.89	198.2398	108.4793	163.1386	111.1232	631.5027	158.5154	281.9126	294.425	319.7342	172.1833	149.6349	120.37	137.5344	506.0342	562.8814
AC100807.	0	0	0	0	0	0	5.098081	0	0	0	0	0	0	0	0	0	0	0	0
AC100810.	1569.162	860.3358	1610.603	1053.843	1601.423	1034.55	740.9212	921.3969	2108.122	1466.027	1657.743	1318.472	1368.38	1482.637	1377.032	1186.386	1303.498	1099.441	1054.954
AC100810.	0	0	0	0	0	0	0	3.704108	0	0	0	0	0	0	0	0	0	0	0
AC100810.	13.45271	38.01484	44.96101	57.1611	13.28982	29.68906	35.68657	28.70684	47.8588	33.62447	30.11887	33.12282	23.64594	33.50593	20.53812	39.57369	25.65941	32.7648	27.78555

AC100810.	0	4.001562	0	0	0	0	0	0	0	0	0	0	0	0	0	0	0	0	0
AC100812.	1.921815	16.00625	12.98874	9.353635	0	10.27698	3.398721	13.89041	11.67288	4.803496	8.433284	8.280704	6.168506	0.93072	6.846041	7.420066	13.34289	5.4608	11.652
AC100814.	158.5497	224.0875	195.8302	203.7014	199.3473	195.2627	191.1781	228.7287	85.212	227.6857	95.17564	192.2964	245.7122	162.8761	188.7551	189.6239	169.3521	186.5774	95.90495
AC100818.	0	0	0	3.117878	0	0	0	2.778081	0	0	4.81902	0	0	0	0	0.824452	0	0	0
AC100821.	214.2824	241.0941	324.7184	163.169	277.9787	148.4453	330.5256	265.7698	12.84017	220.0001	114.4517	299.9455	310.4815	196.382	306.1158	108.8276	113.9278	193.8584	175.6764
AC100821.	12.4918	9.003514	17.98441	25.98232	19.93473	10.27698	38.23561	19.44657	32.68406	6.724895	13.2523	12.8811	23.64594	20.47585	14.67009	25.55801	17.4484	6.370934	32.26709
AC100823.	140.2925	97.03787	168.8536	22.86444	155.0479	71.93888	64.5757	408.3779	206.6099	85.50223	109.6327	125.1306	140.8476	174.0447	179.9531	90.6897	69.79359	48.23707	77.97879
AC100823.	0	6.002343	0	1.039293	0	1.141887	0	2.778081	1.167288	0	0	0	0	0	0.978006	0	4.105505	5.4608	0
AC100826.	11.53089	13.00508	25.97748	11.43222	11.07485	9.135096	9.346483	28.70684	51.36066	14.41049	25.29985	18.40156	16.44935	10.23792	5.868035	2.473355	8.21101	9.101334	12.54831
AC100827.	0	1.00039	9.991337	2.078586	0	0	2.549041	0.926027	1.167288	1.921399	0	0.920078	1.028084	2.792161	0.978006	0	2.052753	0	0
AC100827.	27.86632	24.00937	73.93589	71.7112	69.77155	44.53359	39.93497	35.18903	102.7213	38.42797	72.28529	49.68422	32.8987	27.92161	43.03226	32.97807	39.0023	30.0344	61.84525
AC100827.	22.10087	11.00429	46.95928	35.33595	6.64491	30.83095	48.43177	32.41095	52.52795	32.66377	16.86657	50.6043	46.2638	54.9125	28.36217	24.73355	31.81766	20.02294	13.44462
AC100827.	17.29634	6.002343	17.98441	14.5501	17.71976	17.12831	35.68657	31.48492	7.003726	10.56769	2.40951	25.76219	14.39318	17.68369	7.824047	18.96239	31.81766	29.12427	22.4077
AC100830.	51.88901	56.02186	71.93762	48.84676	25.47215	39.96605	95.16419	57.41368	26.84762	18.25329	30.11887	70.84602	34.95487	42.81313	33.2522	33.80253	71.84634	73.72081	40.33386
AC100830.	91.28622	69.02694	55.95149	104.9686	23.25718	68.51322	73.0725	40.74519	30.34948	33.62447	19.27608	81.88696	74.02207	53.05106	96.82258	98.10977	46.18693	93.74374	87.83818
AC100832.	6.726353	0	0.999134	5.196464	0	0	0.84968	0	0	0	0	3.680313	0	0.93072	0	0	1.026376	0	0
AC100832.	38.4363	47.01835	30.97314	67.55403	65.34161	57.09435	23.79105	37.04108	67.70269	45.15286	59.03299	35.88305	17.47743	27.92161	24.45015	19.78684	20.52753	30.0344	34.0597
AC100835.	0.960908	0	14.987	2.078586	11.07485	0	2.549041	5.556162	0	8.646293	4.81902	7.360626	11.30893	0	0.978006	8.244518	0	2.7304	12.54831
AC100839.	0	1.00039	1.998267	2.078586	3.322455	1.141887	4.248401	2.778081	0	2.882098	0	14.72125	5.140422	2.792161	0	1.648904	3.079129	3.640534	1.792616
AC100843.	0	0	0	0	0	0	0.84968	0	0	0	0	0	1.028084	1.861441	0.978006	3.297807	0	0.910133	0
AC100843.	5.765445	0	1.998267	0	1.107485	0	1.69936	7.408216	10.50559	0	0	13.80117	1.028084	7.445762	7.824047	0.824452	0	0	0
AC100847.	24.9836	15.00586	19.98267	22.86444	27.68712	19.41208	23.79105	34.263	67.70269	52.83846	13.2523	9.200782	56.54464	20.47585	16.6261	22.2602	27.71216	36.40534	30.47447
AC100848.	0	0	0	0	0	0	3.398721	0	0	0	0	0	0	0	0.978006	0	0	0	0
AC100849.	98.97348	41.01601	11.9896	139.2652	4.42994	11.41887	9.346483	42.59724	10.50559	0.960699	14.45706	29.4425	10.28084	59.5661	11.73607	190.4484	46.18693	91.01334	38.54124
AC100849.	0	0	0	1.039293	0	0	0	0	1.167288	3.842797	0	0	0	3.722881	5.868035	0	2.052753	0	0
AC100850.	8.648168	0	0	0	0	0	0	4.630135	0	0	0	0	0	0.93072	0	0	0	0	0
AC100854.	115.3089	92.03592	156.864	70.67191	106.3186	6.851322	145.2953	14.81643	150.5801	149.8691	108.4279	92.00782	64.76932	57.70466	76.28445	81.62073	37.97592	86.46267	76.18618
AC100858.	0	0	0	0	0	0	1.69936	0	0	0	0	0	0	0	0	0.824452	0	0	0
AC100858.	224.8524	70.02733	88.9229	194.3477	21.04221	91.35096	74.77186	85.19449	110.8923	9.606993	46.98544	172.0546	78.13441	130.3008	88.99853	464.1664	203.2225	205.6902	85.14926
AC100860.	0.960908	0	1.998267	9.353635	0	6.851322	6.797442	0.926027	0	0.960699	15.66181	5.520469	5.140422	0.93072	18.58211	4.122259	10.26376	16.3824	21.51139
AC100861.	20.17906	20.00781	39.96535	7.275049	35.43952	50.24303	15.29424	32.41095	11.67288	18.25329	24.0951	19.32164	23.64594	49.32818	46.94428	12.36678	4.105505	70.08027	25.99293
AC100863.	0.960908	0	0	0	1.107485	0	0.84968	0	0	4.803496	0	0	3.084253	0.93072	0	2.473355	1.026376	0	0
AC100868.	0	1.00039	0	0	3.322455	4.567548	1.69936	4.630135	0	0	0	2.760235	0	0	0	0	2.052753	0	1.792616
AC100871.	0	0	0.999134	0	0	0	0	0	0	0	0	0	1.028084	0	0	0	0	0	0
AC100871.	10.56998	39.01523	15.98614	63.39686	29.90209	26.2634	32.28785	12.96438	1.167288	7.685594	2.40951	27.60235	25.70211	43.74385	43.03226	39.57369	45.16056	24.5736	26.88924
AC100872.	0	0	0	0	0	0	0	0.926027	0	0	0	3.680313	1.028084	0	0.978006	0	1.026376	4.550667	0
AC102797.	1.921815	1.00039	0.999134	0	0	0	0.84968	0	0	0	0	0	0	0	0	0	0	0	0.896308
AC102941.	0	0	0	8.314342	4.42994	0	0	0	16.34203	3.842797	0	6.440548	3.084253	0	0	4.122259	9.237386	0	10.7557
AC102945.	2.882723	9.003514	3.996535	4.157171	1.107485	1.141887	2.549041	2.778081	18.6766	0	4.81902	4.600391	1.028084	0.93072	11.73607	4.122259	6.158258	5.4608	0.896308
AC102945.	120.1134	146.057	228.8016	109.1257	183.8425	116.4725	182.6813	182.4273	51.36066	95.10923	75.89956	253.9416	213.8415	215.9271	244.5015	201.1662	206.3016	219.3422	196.2914
AC102953.	13.45271	6.002343	8.992203	11.43222	0	5.709435	0.84968	3.704108	19.84389	10.56769	14.45706	8.280704	7.196591	1.861441	8.802052	3.297807	11.29014	16.3824	7.170464
AC102953.	679.3616	544.2124	408.6457	480.1533	507.2281	649.7337	343.2708	433.3807	925.6592	623.4938	958.9849	464.6395	552.0813	510.0347	455.7507	643.8969	839.5758	480.5504	656.0974
AC103409.	0	0	0	0	0	0	3.398721	1.852054	0	0	0	1.840156	1.028084	0	0	0	0	0	0
AC103563.	111.4653	130.0508	80.92983	65.47544	121.8233	137.0264	79.02026	91.67668	235.7921	150.8298	186.737	146.2924	140.8476	89.34915	69.43841	29.68027	27.71216	105.5755	146.9945
AC103563.	17.29634	9.003514	30.97314	16.62868	25.47215	12.56076	20.39233	12.96438	24.51304	11.52839	21.68559	24.84211	38.03912	19.54513	25.42815	4.946711	8.21101	18.20267	26.88924
AC103563.	0	0	0	0	11.07485	0	0.84968	0	0	2.882098	0	0	2.056169	0.93072	0	0	0	0	3.585232
AC103564.	0	0	0	0	0	0	0	3.704108	0	0	2.40951	0.920078	0	0	0	0	0	0	0
AC103564.	0.960908	0	0	0	1.107485	0	1.69936	0	2.334575	0	0	0	0	5.584322	0	0	0	0	0
AC103564.	7.68726	3.001171	6.993936	1.039293	1.107485	0	1.69936	0.926027	12.84017	4.803496	2.40951	0	0	0.93072	0.978006	4.122259	1.026376	3.640534	0
AC103591.	0.960908	0	0	4.157171	1.107485	0	0	0	1.167288	0	0	2.760235	0	0	2.934017	0	0	0	0.896308
AC103591.	32.67086	28.01093	28.97488	12.47151	9.967364	20.55397	32.28785	42.59724	36.18592	25.93888	19.27608	28.52243	10.28084	14.89152	19.56012	33.80253	45.16056	61.88907	43.02278
AC103591.	128.7616	73.0285	106.9073	123.6758	188.2724	115.3306	184.3806	105.5671	99.21946	47.07426	30.11887	141.692	107.9489	127.5087	145.7229	103.0565	83.13648	117.4072	145.2019
AC103681.	1.921815	10.0039	15.98614	16.62868	23.25718	5.709435	16.14392	8.334243	1.167288	16.33189	7.228529	25.76219	29.81445	10.23792	8.802052	11.54233	8.21101	4.550667	3.585232
AC103691.	122.9962	92.03592	62.94542	186.0334	132.8982	42.24982	99.41259	38.89314	552.1271	166.201	180.7132	148.1326	115.1454	61.42754	65.52639	65.95615	73.89909	127.4187	130.861
AC103691.	2.882723	3.001171	3.996535	1.039293	6.64491	0	6.797442	0	2.334575	5.764196	7.228529	1.840156	2.056169	8.376483	8.802052	1.648904	2.052753	0.910133	5.377848
AC103702.	4.804538	15.00586	25.97748	16.62868	15.50479	9.135096	29.73881	22.22465	15.17474	18.25329	3.614265	19.32164	45.23571	30.71377	23.47214	23.9091	33.87042	20.93307	18.82247
AC103702.	150.8625	86.03358	56.95062	44.68959	97.45867	125.6076	163.9883	125.9397	254.4687	99.91272	125.2945	82.80704	99.72418	80.97267	93.88856	29.68027	72.87271	65.52961	98.59387
AC103703.	12.4918	8.003124	3.996535	4.157171	15.50479	2.283774	10.19616	2.778081	45.52422	30.74238	19.27608	0.920078	21.58977	5.584322	4.890029	6.595615	5.131881	0	0.896308
AC103706.	158.5497	206.0804	296.7427	114.3222	210.4221	269.4853	163.9883	222.2465	525.2795	366.9871	519.2494	155.4932	177.8586	194.5205	223.9633	162.417	154.9828	250.2867	166.7133
AC103706.	0	0	0	0	0	2.283774	0	3.704108	0	0	7.228529	3.680313	2.056169	0	0	3.297807	2.052753	1.820267	0
AC103706.	0	0	0	0	0	0	0	0.926027	0	0.960699	0	0	0	0.93072	0	0.824452	5.131881	0	0
AC103718.	0	4.001562	0	0	0	0	0.84968	0	0	0	0	1.840156	0	0	0.978006	0	0	0	0

AC103719.	227.7351	69.02694	181.8423	157.9725	29.90209	105.0536	83.26866	175.9451	22.17847	62.44545	49.39495	117.77	132.6229	101.4485	66.5044	30.50472	30.79129	53.69787	80.66772
AC103724.	0.960908	0	0.999134	0	0	1.141887	0	0	0	0	0	0	0	0	0.978006	0	0	0	0
AC103724.	1.921815	1.00039	1.998267	0	1.107485	23.97963	7.647122	0	1.167288	24.01748	12.04755	4.600391	4.112337	20.47585	4.890029	18.96239	9.237386	2.7304	37.64493
AC103724.	0	6.002343	3.996535	1.039293	0	4.567548	0.84968	5.556162	1.167288	0	0	9.200782	1.028084	4.653602	9.780058	0.824452	0	2.7304	8.963079
AC103724.	3.84363	10.0039	1.998267	4.157171	13.28982	10.27698	12.7452	12.96438	18.6766	16.33189	0	0.920078	3.084253	0	2.934017	6.595615	3.079129	4.550667	1.792616
AC103726.	5.765445	9.003514	2.997401	7.275049	0	5.709435	9.346483	2.778081	4.669151	4.803496	0	1.840156	0	3.722881	0.978006	7.420066	8.21101	10.01147	7.170464
AC103736.	55.73264	110.0429	90.92116	67.55403	160.5853	63.94567	60.3273	142.6082	172.7586	99.91272	231.3129	92.00782	65.7974	71.66546	47.92229	93.16306	128.297	119.2275	61.84525
AC103739.	30.74904	25.00976	65.94282	39.49313	60.91167	17.12831	33.98721	40.74519	23.34575	29.78168	20.48083	38.64329	42.15146	17.68369	20.53812	33.80253	39.0023	38.2256	38.54124
AC103739.	33.63176	54.02108	63.94455	61.31827	57.58922	25.12151	62.02666	38.89314	30.34948	59.56335	27.70936	47.84407	55.51656	54.9125	39.12023	43.69595	48.23968	50.96747	61.84525
AC103740.	192.1815	232.0906	514.5538	268.1375	307.8808	168.9993	486.0171	341.704	92.21573	317.0308	209.6273	414.0352	335.1555	280.1468	256.2375	126.1411	189.8796	130.1491	264.4108
AC103740.	0	0	0	0	0	0	11.04584	0	0	0	1.204755	0	1.028084	0	0	0	0	3.640534	0
AC103746.	182.5724	104.0406	148.8709	88.33989	93.02873	115.3306	113.8572	103.715	343.1826	132.5765	146.9801	151.8129	157.2969	116.34	116.3827	90.6897	136.508	123.7781	120.1053
AC103760.	100.8953	102.0398	78.93156	128.8723	73.094	78.7902	116.4062	70.37805	91.04844	112.4018	107.2232	81.88696	70.93782	90.27987	93.88856	107.1787	49.26606	66.43974	86.94187
AC103764.	0	1.00039	3.996535	0	0	0	0	0	0	0	0	1.840156	0	0	0	0	0	0	3.585232
AC103769.	0	3.001171	0	6.235757	0	5.709435	4.248401	0	0	34.58517	87.94711	0.920078	0	0	3.912023	0.824452	0	0.910133	0.896308
AC103770.	700.5016	188.0734	195.8302	3035.774	781.8844	1227.529	679.7442	257.4355	148.2455	368.9085	285.5269	340.4289	306.3691	918.6209	784.3607	985.2199	541.9267	57.33841	247.381
AC103783.	0	0	0	4.157171	0	0	0	0	4.669151	0.960699	0	0	0	0	0	0.824452	1.026376	0	0
AC103792.	15.37452	0	24.97834	0	5.537425	26.2634	20.39233	2.778081	9.338302	16.33189	0	0	0	0	0	0	1.026376	0.910133	0
AC103794.	0	0	5.994802	1.039293	0	0	1.69936	1.852054	4.669151	0	0	1.840156	1.028084	0	1.956012	0.824452	1.026376	0.910133	0.896308
AC103796.	1.921815	8.003124	7.993069	13.51081	1.107485	1.141887	5.947762	9.26027	12.84017	1.921399	10.84279	3.680313	4.112337	16.75297	2.934017	2.473355	4.105505	2.7304	6.274156
AC103808.	0	1.00039	0	0	0	0	0	0	0	3.842797	0	0	0	0	0	1.648904	0	0.910133	1.792616
AC103808.	9.609075	0	0	9.353635	6.64491	5.709435	0.84968	0.926027	0	5.764196	7.228529	0	1.028084	9.307203	1.956012	0	0	17.29253	1.792616
AC103808.	0.960908	0	0	6.235757	34.33203	3.425661	2.549041	0	0	0	1.204755	0.920078	0	9.307203	5.868035	3.297807	9.237386	4.550667	2.688924
AC103808.	0	0	0.999134	0	0	0	0.84968	0	0	0	0	0	6.168506	0	3.912023	0	0	0.910133	0
AC103808.	0	1.00039	1.998267	0	4.42994	1.141887	3.398721	0	0	0	2.40951	3.680313	0	3.722881	1.956012	0	0	4.550667	0
AC103808.	0.960908	0	3.996535	11.43222	16.61227	0	7.647122	0	25.68033	7.685594	7.228529	6.440548	1.028084	0.93072	9.780058	9.893422	3.079129	1.820267	2.688924
AC103809.	1.921815	1.00039	9.991337	0	2.21497	7.993209	2.549041	3.704108	0	5.764196	0	6.440548	15.42127	1.861441	6.846041	8.244518	4.105505	17.29253	9.859387
AC103810.	66.30262	88.03436	69.93936	87.30059	73.094	45.67548	66.27506	90.75065	84.04472	99.91272	84.33284	68.08579	93.55568	75.38834	74.32844	63.48279	57.47707	60.97894	105.7643
AC103810.	0	0	0	0	0	0	0	0	0	0	0	0	0	0	0	0	3.079129	0.910133	0
AC103810.	52.84991	69.02694	30.97314	40.53242	187.165	31.97284	30.58849	90.75065	246.2977	24.97818	13.2523	146.2924	195.336	61.42754	56.72434	116.2477	130.3498	43.6864	37.64493
AC103810.	0	0	0	0	0	1.141887	0	0.926027	4.669151	0	9.638039	0	1.028084	3.722881	0.978006	0	1.026376	0.910133	1.792616
AC103810.	0	4.001562	4.995668	0	3.322455	6.851322	1.69936	5.556162	4.669151	0.960699	4.81902	8.280704	9.252759	0.93072	5.868035	1.648904	6.158258	1.820267	13.44462
AC103810.	40.35812	46.01796	39.96535	48.84676	56.48173	19.41208	57.77826	42.59724	18.6766	34.58517	14.45706	96.60821	81.21867	37.22881	25.42815	74.20066	104.6904	35.4952	46.60801
AC103810.	0	0	0	0	0	0	0.84968	1.852054	0	0	0	0	1.028084	0	2.934017	0	0	0	0
AC103810.	0	0	0	0	0	0	0	0	7.003726	0	0	0.920078	0	0	0	0	0	4.550667	0
AC103831.	0	0	0	0	0	0	0	0	0	2.882098	0	0	0	0	0	0	0	0	0
AC103834.	0	0	0	0	0	0	0	0	0	0	0	1.840156	0	0	0	0	0	0	0
AC103843.	0	0	0	0	0	0	3.398721	0	0	0	0	2.760235	0	1.861441	0	0	1.026376	11.83173	1.792616
AC103843.	0	0	0	4.157171	0	0	5.098081	0	0	0	9.638039	1.840156	1.028084	0.93072	0	0	0	0.910133	0.896308
AC103851.	0	0	0	8.314342	0	0	0	0	0	0	0	0.920078	0	0	0	0	0	0	0
AC103853.	0.960908	4.001562	7.993069	21.82515	18.82724	2.283774	11.04584	8.334243	11.67288	0.960699	1.204755	7.360626	7.196591	15.82225	7.824047	8.244518	18.47477	3.640534	9.859387
AC103853.	4.804538	13.00508	11.9896	4.157171	21.04221	1.141887	7.647122	0	2.334575	7.685594	7.228529	1.840156	4.112337	3.722881	1.956012	0	0	0	0.896308
AC103855.	0	0	0	0	0	0	1.69936	0	0	0	0	0	0	0	0	0	0	0.910133	0
AC103858.	44.20175	23.00898	57.94975	37.41454	17.71976	34.25661	53.52986	37.96711	33.85134	10.56769	0	21.1618	21.58977	13.03008	12.71408	26.38246	13.34289	29.12427	56.4674
AC103858.	26.90541	25.00976	31.97228	32.21808	11.07485	39.96605	32.28785	21.29862	18.6766	45.15286	61.4425	34.04289	25.70211	31.64449	19.56012	17.31349	18.47477	35.4952	52.88217
AC103876.	9.609075	3.001171	4.995668	1.039293	5.537425	22.83774	5.947762	2.778081	10.50559	15.37119	7.228529	1.840156	1.028084	4.653602	13.69208	10.71787	3.079129	3.640534	11.652
AC103879.	0	2.000781	3.996535	2.078586	4.42994	1.141887	0.84968	3.704108	9.338302	0	0	2.760235	4.112337	0.93072	5.868035	1.648904	0	0	1.792616
AC103923.	97.05166	88.03436	196.8293	134.0688	153.9404	78.7902	171.6354	117.6054	16.34203	107.5983	75.89956	159.1735	149.0722	124.7165	97.80058	117.8966	135.4817	202.9598	264.4108
AC103925.	3.84363	4.001562	0	6.235757	2.21497	3.425661	2.549041	0	0	0	0	0.920078	3.084253	0.93072	1.956012	3.297807	1.026376	7.281067	16.13354
AC103949.	68.22443	45.01757	49.95668	50.92535	16.61227	31.97284	39.93497	25.92876	124.8998	79.73804	31.32363	42.3236	45.23571	49.32818	45.96627	32.97807	7.184634	43.6864	31.37078
AC103952.	61.49808	104.0406	35.96881	49.88605	27.68712	30.83095	87.51706	45.37532	117.8961	67.24895	115.6565	76.36649	63.74123	51.18962	88.99853	64.30724	81.08372	72.81067	78.8751
AC103957.	0.960908	0	0	1.039293	0	2.283774	0	0	0	0.960699	3.614265	0	0	0.93072	0	2.473355	0	0	0
AC103974.	367.0667	163.0636	222.8068	242.1552	244.7542	266.0597	219.2175	192.6136	352.5209	216.1573	330.1028	220.8188	210.7573	154.4996	222.9853	197.8684	253.5149	321.2771	311.9152
AC103975.	5.765445	7.002733	3.996535	8.314342	9.967364	7.993209	0.84968	19.44657	15.17474	23.05678	45.78069	3.680313	7.196591	3.722881	10.75806	0	10.26376	5.4608	11.652
AC103982.	0.960908	2.000781	0	5.196464	4.42994	0	1.69936	0.926027	0	6.724895	0	5.520469	2.056169	0.93072	0	7.420066	0	4.550667	0.896308
AC103987.	0.960908	0	3.996535	0	0	0	9.346483	0	0	3.842797	0	4.600391	5.140422	0	0	0.824452	0	0	4.48154
AC103988.	1.921815	0	0	0	0	1.141887	0	0	0	0	0	0.920078	2.056169	0	0	0	0	0	0
AC103988.	0	0	0	0	0	0	0.84968	1.852054	0	0	0	0.920078	0	0	0	4.122259	0	0.910133	0.896308
AC103993.	1.921815	0	0	0	0	0	0	0	0	0	0	6.440548	0	0	0	0	0	0	0
AC103996.	9.609075	18.00703	62.94542	73.78979	37.65449	31.97284	78.17058	12.96438	0	21.13538	26.50461	33.12282	26.73019	16.75297	41.07624	24.73355	23.60665	20.02294	35.85232
AC103996.	6.726353	1.00039	14.987	0	11.07485	9.135096	3.398721	8.334243	14.00745	7.685594	51.80446	11.04094	15.42127	7.445762	8.802052	0	3.079129	6.370934	5.377848
AC104002.	0	0	5.994802	0	0	0	1.69936	0	0	0	0	0	0	0	0	2.473355	0	0	0

AC104002.	0	0	3.996535	1.039293	0	4.567548	2.549041	1.852054	1.167288	0	0	1.840156	0	0.93072	0.978006	1.648904	0	0	3.585232
AC104009.	176.807	157.0613	116.8986	348.1631	107.426	204.3978	224.3156	124.0876	78.20828	94.14853	45.78069	171.1346	148.0441	146.1231	150.6129	231.671	247.3567	128.3288	190.0173
AC104012.	1.921815	1.00039	0	0	0	0	0	0	0	0	0	0	0	0.93072	0	0	0	0	0
AC104024.	0	1.00039	0.999134	0	1.107485	1.141887	0.84968	0	0	0	0	0	0	0.93072	0.978006	0	0	0	0
AC104024.	39.39721	4.001562	21.98094	3.117878	8.859879	2.283774	1.69936	0	22.17847	21.13538	21.68559	20.24172	14.39318	12.09936	19.56012	9.893422	5.131881	0	3.585232
AC104027.	0	0	0.999134	0	0	0	0	0	0	0	1.204755	0.920078	0	0	0	0	0	0	0
AC104031.	8.648168	0	10.99047	1.039293	4.42994	0	5.947762	0.926027	4.669151	0.960699	0	14.72125	5.140422	9.307203	7.824047	7.420066	20.52753	9.101334	7.170464
AC104036.	0	1.00039	2.997401	0	17.71976	1.141887	4.248401	1.852054	4.669151	0	1.204755	0	0	0.93072	0.978006	0	1.026376	0	1.792616
AC104041.	11.53089	6.002343	7.993069	0	11.07485	3.425661	51.83049	5.556162	1.167288	4.803496	10.84279	0.920078	5.140422	2.792161	2.934017	1.648904	1.026376	4.550667	6.274156
AC104042.	0	6.002343	4.995668	1.039293	0	0	0.84968	0	0	0	0	0	4.112337	0	0	0	11.29014	0	0.896308
AC104046.	6.726353	4.001562	37.96708	1.039293	4.42994	7.993209	8.496802	25.92876	80.54285	51.87776	83.12809	0.920078	16.44935	0.93072	6.846041	4.946711	7.184634	12.74187	10.7557
AC104051.	0	0	0	0	0	0	2.549041	0	0	0	0	0	0	0	0	0	0	0	0
AC104051.	227.7351	245.0957	217.8111	90.41847	180.52	211.2491	77.3209	165.7588	1027.213	168.1224	362.6312	273.2632	314.5938	122.8551	178.9751	71.72731	92.37386	108.3059	122.7942
AC104057.	0	0	0	0	0	0	1.69936	0	0	0	0	0	0	0.93072	0	0	0	0	0
AC104058.	6.726353	1.00039	4.995668	3.117878	0	0	2.549041	6.482189	0	5.764196	0	4.600391	6.168506	0	2.934017	14.84013	6.158258	0	3.585232
AC104063.	0	1.00039	3.996535	0	3.322455	0	1.69936	0	0	8.646293	0	3.680313	10.28084	3.722881	0.978006	0.824452	4.105505	0	3.585232
AC104066.	48.04538	21.0082	33.97054	33.25737	29.90209	19.41208	49.28145	34.263	10.50559	22.09608	8.433284	28.52243	51.40422	24.19873	32.27419	28.03136	10.26376	23.66347	26.88924
AC104066.	15.37452	4.001562	9.991337	3.117878	4.42994	5.709435	4.248401	11.11232	0	0.960699	0	11.04094	16.44935	7.445762	0.978006	1.648904	2.052753	1.820267	3.585232
AC104072.	1466.345	2003.782	1314.86	1980.892	1029.961	2882.123	1471.646	1341.813	986.3581	1811.879	1087.894	1191.501	1420.813	1193.183	1415.174	2008.365	2127.678	705.3534	1073.777
AC104073.	0	2.000781	2.997401	1.039293	9.967364	2.283774	0.84968	2.778081	0	7.685594	14.45706	2.760235	7.196591	0	2.934017	4.946711	1.026376	4.550667	0.896308
AC104073.	0.960908	8.003124	6.993936	16.62868	3.322455	7.993209	5.098081	12.96438	2.334575	13.44979	18.07132	5.520469	16.44935	22.33729	4.890029	13.19123	8.21101	7.281067	17.02985
AC104073.	0	0	0	6.235757	0	11.41887	2.549041	0	0	0	0	0.920078	4.112337	0	0	0	3.079129	3.640534	0.896308
AC104076.	8.648168	2.000781	1.998267	0	2.21497	0	0.84968	4.630135	3.501863	0.960699	0	3.680313	4.112337	7.445762	0.978006	0	1.026376	8.191201	4.48154
AC104078.	34.59267	41.01601	20.98181	39.49313	38.76197	62.80379	47.58209	57.41368	52.52795	44.19217	42.16642	39.56336	37.01104	48.39746	51.83431	15.66458	30.79129	23.66347	29.57816
AC104078.	0	0	0	0	0	0	0	0	0	0	0	0.920078	0	0	0	0	0	0.910133	0
AC104083.	953.2203	625.244	746.3528	531.0786	656.7386	367.6876	366.2122	667.6655	519.443	731.0921	473.4687	589.7701	730.968	548.1943	671.89	516.9313	617.8785	391.3574	297.5742
AC104088.	7.68726	0	3.996535	10.39293	16.61227	6.851322	10.19616	5.556162	0	0	0	6.440548	9.252759	5.584322	9.780058	4.122259	2.052753	6.370934	6.274156
AC104090.	0	0	0.999134	21.82515	0	5.709435	0.84968	12.96438	11.67288	17.29259	1.204755	10.12086	16.44935	0.93072	0	0	9.237386	0	0
AC104109.	0.960908	0	0.999134	0	3.322455	1.141887	1.69936	0	0	0	0	0	1.028084	0	0.978006	0	0	0.910133	0.896308
AC104109.	117.2307	209.0816	140.8778	147.5796	131.7907	118.7562	179.2825	98.15887	142.4091	105.6769	79.51382	176.655	165.5216	132.1623	161.371	137.6835	145.7454	155.6328	212.425
AC104109.	29.78813	53.02069	79.93069	33.25737	94.13622	76.50643	77.3209	95.38078	3.501863	59.56335	39.75691	67.16571	113.0893	44.67457	73.35044	52.76492	43.1078	36.40534	25.99293
AC104113.	38.4363	41.01601	26.97661	41.57171	27.68712	42.24982	20.39233	58.3397	33.85134	93.18783	40.96167	17.48149	38.03912	43.74385	47.92229	47.81821	60.5562	48.23707	33.16339
AC104115.	0	2.000781	1.998267	6.235757	4.42994	5.709435	0	5.556162	0	9.606993	1.204755	1.840156	5.140422	15.82225	1.956012	14.01568	3.079129	0.910133	0.896308
AC104115.	5.765445	10.0039	8.992203	16.62868	32.11706	2.283774	24.64073	18.52054	1.167288	16.33189	4.81902	29.4425	21.58977	11.16864	14.67009	13.19123	7.184634	3.640534	11.652
AC104117.	21.13997	6.002343	11.9896	16.62868	6.64491	2.283774	11.89552	12.96438	0	25.93888	3.614265	11.04094	5.140422	8.376483	4.890029	18.96239	14.36927	14.56213	15.23724
AC104117.	49.96719	88.03436	120.8952	72.75049	66.4491	91.35096	80.71962	101.863	144.7437	85.50223	127.704	79.12673	68.88165	86.55699	89.97654	63.48279	72.87271	53.69787	54.67478
AC104117.	2.882723	2.000781	0	0	4.42994	2.283774	0.84968	0	0	3.842797	0	0	0	0	3.912023	1.648904	6.158258	0.910133	0.896308
AC104117.	9.609075	8.003124	21.98094	5.196464	1.107485	20.55397	4.248401	7.408216	44.35693	43.23147	27.70936	15.64133	3.084253	13.9608	8.802052	15.66458	5.131881	11.83173	15.23724
AC104118.	135.488	120.0469	184.8397	236.9588	126.2533	99.34417	130.0011	108.3452	268.4762	171.0045	231.3129	127.8909	105.8927	83.76483	92.91055	171.486	198.0906	205.6902	170.2985
AC104123.	17.29634	12.00469	1.998267	33.25737	2.21497	29.68906	36.53625	10.1863	2.334575	12.48909	1.204755	15.64133	17.47743	18.61441	6.846041	32.97807	19.50115	17.29253	8.963079
AC104126.	0	2.000781	0	0	0	0	0.84968	1.852054	0	0	0	0	1.028084	0	0	0.824452	0	0	0
AC104129.	1.921815	1.00039	9.991337	0	8.859879	1.141887	5.098081	0	0	6.724895	1.204755	4.600391	11.30893	13.03008	6.846041	1.648904	2.052753	2.7304	11.652
AC104134.	0	1.00039	1.998267	7.275049	1.107485	1.141887	0.84968	1.852054	1.167288	3.842797	3.614265	0	0	0	0.978006	2.473355	0	3.640534	0.896308
AC104135.	0	16.00625	31.97228	29.1002	6.64491	26.2634	43.33369	38.89314	0	0	0	26.68227	39.06721	5.584322	26.40616	43.69595	46.18693	18.20267	34.0597
AC104136.	0	0	3.996535	0	0	0	0	0	0	0	0	0	0	0	0	0	0	0	0
AC104137.	184.4942	221.0863	277.7592	236.9588	225.9269	227.2355	167.387	192.6136	25.68033	227.6857	189.1465	137.0917	167.5778	174.0447	193.6452	191.2728	165.2466	157.4531	165.817
AC104162.	120.1134	105.041	298.741	117.4401	134.0057	108.4793	157.1908	112.0493	137.74	56.68126	62.64725	138.9318	150.1003	170.3218	112.4707	96.46086	89.29473	149.2619	155.9576
AC104164.	0.960908	0	0	0	0	0	0	0	0	0.960699	4.81902	0	0	0	0	0	1.026376	0	0
AC104164.	16.33543	24.00937	15.98614	10.39293	33.22455	26.2634	7.647122	8.334243	81.71014	48.99566	56.62348	5.520469	4.112337	13.9608	11.73607	7.420066	21.5539	11.83173	15.23724
AC104164.	5.765445	0	4.995668	0	0	0	0	0	0	1.921399	0	0	4.112337	0	0	0	0	0.910133	0
AC104164.	16.33543	27.01054	15.98614	9.353635	32.11706	19.41208	5.947762	7.408216	78.20828	41.31007	55.41872	6.440548	5.140422	13.9608	10.75806	8.244518	20.52753	10.01147	16.13354
AC104170.	91.28622	37.01445	37.96708	48.84676	33.22455	50.24303	56.92858	51.85751	31.51677	52.83846	22.89034	80.04681	85.331	70.73474	69.43841	55.23827	100.5849	87.37281	57.36371
AC104170.	22.10087	17.00664	17.98441	25.98232	6.64491	27.40529	18.69297	22.22465	22.17847	20.17468	24.0951	12.8811	6.168506	13.9608	16.6261	14.84013	15.39564	10.9216	9.859387
AC104184.	92.24712	115.0449	122.8934	86.2613	145.0805	103.9117	109.6088	129.6438	162.253	145.0656	109.6327	87.40743	81.21867	115.4093	96.82258	100.5831	111.875	111.9464	148.7871
AC104187.	3.84363	0	0	0	4.42994	0	0	0	0	0	0	0	0	0	0	0	0	0	0
AC104211.	68.22443	59.02304	103.9099	88.33989	237.0018	86.78341	146.9947	130.5698	45.52422	57.64196	22.89034	156.4133	167.5778	142.4002	113.4487	18.13794	21.5539	48.23707	21.51139
AC104211.	1.921815	3.001171	0	0	0	0	0	0	0	0	14.45706	0	0	0	0.978006	0	0	0	0
AC104212.	0	2.000781	1.998267	0	0	0	3.398721	0	0	0	0	1.840156	1.028084	3.722881	0	1.648904	3.079129	8.191201	1.792616
AC104212.	0.960908	1.00039	0	0	0	0	0	0.926027	0	0	1.204755	0	1.028084	0	0	0.824452	0	0	0
AC104212.	0.960908	0	0	0	6.64491	0	1.69936	0	3.501863	0	0	0	0	0	0	0	0	0	1.792616
AC104232.	6.726353	8.003124	27.97574	7.275049	11.07485	11.41887	16.9936	12.96438	3.501863	3.842797	8.433284	28.52243	13.3651	10.23792	7.824047	7.420066	9.237386	8.191201	17.02985
AC104232.	0.960908	0	0	0	0	0	0	2.778081	4.669151	0	0	1.840156	1.028084	1.861441	0	1.648904	8.21101	0	0

AC104233.	5.765445	6.002343	6.993936	2.078586	4.42994	4.567548	6.797442	4.630135	0	13.44979	6.023774	4.600391	8.224675	4.653602	0.978006	3.297807	1.026376	2.7304	0.896308
AC104233.	1.921815	3.001171	2.997401	2.078586	3.322455	1.141887	0	1.852054	0	2.882098	6.023774	2.760235	1.028084	1.861441	1.956012	0	1.026376	3.640534	1.792616
AC104237.	0.960908	7.002733	3.996535	10.39293	0	0	2.549041	2.778081	0	4.803496	12.04755	6.440548	17.47743	8.376483	4.890029	0.824452	3.079129	3.640534	2.688924
AC104237.	0.960908	4.001562	2.997401	1.039293	0	0	2.549041	5.556162	0	1.921399	1.204755	11.04094	1.028084	0	7.824047	0	0	3.640534	0.896308
AC104237.	0.960908	1.00039	0	0	0	4.567548	0	3.704108	3.501863	4.803496	3.614265	10.12086	7.196591	6.515042	4.890029	1.648904	0	1.820267	12.54831
AC104241.	4.804538	1.00039	0	5.196464	0	0	0	0.926027	0	2.882098	4.81902	0.920078	1.028084	0.93072	0.978006	0	0	0	1.792616
AC104241.	0	6.002343	0	0	0	1.141887	0	0	0	0.960699	0	2.760235	0	0	0	0	1.026376	0.910133	0.896308
AC104248.	0	6.002343	0.999134	0	5.537425	3.425661	0.84968	0	0	19.21399	0	9.200782	6.168506	0	10.75806	5.771163	0	6.370934	1.792616
AC104257.	0	2.000781	0	8.314342	3.322455	1.141887	1.69936	0	11.67288	0	0	0.920078	4.112337	0	1.956012	13.19123	6.158258	8.191201	4.48154
AC104260.	66.30262	56.02186	88.9229	67.55403	63.12664	54.81058	85.8177	95.38078	74.70641	66.28825	32.52838	82.80704	65.7974	68.8733	43.03226	79.14738	46.18693	78.27147	59.15632
AC104260.	769.6869	616.2405	1037.101	722.3085	750.8748	495.579	661.9009	668.5915	324.506	633.1008	609.606	1000.125	933.5006	709.2089	914.4354	621.6367	548.0849	731.7473	708.9796
AC104261.	2.882723	3.001171	4.995668	7.275049	0	6.851322	1.69936	0	4.669151	5.764196	0	0.920078	0	3.722881	0.978006	4.122259	6.158258	0.910133	5.377848
AC104297.	0	1.00039	5.994802	2.078586	0	10.27698	0.84968	3.704108	0	7.685594	1.204755	2.760235	3.084253	0.93072	1.956012	0.824452	3.079129	0.910133	1.792616
AC104304.	0.960908	0	0	0	0	0	0	0	0	0	0	0	0	0	0	3.297807	1.026376	0	0
AC104306.	9.609075	12.00469	3.996535	0	1.107485	3.425661	7.647122	0	0	0	4.81902	9.200782	0	0	0	0	20.52753	6.370934	7.170464
AC104307.	0	0	0	0	0	0	1.69936	0	0	0	0	0	4.112337	0	0	0	0	0	1.792616
AC104316.	2.882723	2.000781	5.994802	5.196464	3.322455	0	2.549041	0	2.334575	2.882098	0	2.760235	7.196591	1.861441	0.978006	2.473355	0	6.370934	3.585232
AC104316.	0	2.000781	3.996535	0	1.107485	1.141887	0.84968	3.704108	1.167288	0	0	0	1.028084	0.93072	0.978006	5.771163	0	0	0.896308
AC104332.	0.960908	0	0	2.078586	4.42994	0	0.84968	0	7.003726	0	0	0.920078	8.224675	0.93072	3.912023	7.420066	0	1.820267	0.896308
AC104332.	0	0	1.998267	0	0	0	0.84968	0	0	0	0	0	0	0	0	0	0	0	0
AC104333.	8.648168	3.001171	1.998267	12.47151	4.42994	12.56076	10.19616	6.482189	3.501863	6.724895	12.04755	10.12086	7.196591	24.19873	11.73607	5.771163	4.105505	10.9216	10.7557
AC104333.	17.29634	25.00976	45.96015	30.13949	25.47215	25.12151	8.496802	32.41095	28.01491	33.62447	42.16642	17.48149	15.42127	17.68369	12.71408	16.48904	27.71216	18.20267	19.71877
AC104333.	0	0	0	0	0	0	0	0	0	0	0	0	1.028084	3.722881	0	0	0	0	0
AC104339.	0	0	0.999134	0	2.21497	1.141887	0	0	0	0	0	0	0	0.93072	1.956012	0	1.026376	0.910133	0
AC104350.	0	0	1.998267	2.078586	0	1.141887	1.69936	0	0	0	0	3.680313	0	0	0.978006	4.122259	5.131881	0	0.896308
AC104365.	43.24084	42.0164	41.96361	23.90373	35.43952	29.68906	40.78465	42.59724	14.00745	25.93888	7.228529	27.60235	21.58977	19.54513	39.12023	37.10033	49.26606	7.281067	13.44462
AC104365.	7.68726	1.00039	8.992203	27.02161	2.21497	21.69585	4.248401	4.630135	36.18592	2.882098	19.27608	7.360626	8.224675	5.584322	5.868035	27.20691	7.184634	5.4608	12.54831
AC104371.	10.56998	11.00429	13.98787	1.039293	4.42994	4.567548	3.398721	13.89041	14.00745	7.685594	10.84279	18.40156	8.224675	1.861441	9.780058	0.824452	10.26376	0.910133	1.792616
AC104383.	0	0	0	0	0	0	0	0	11.67288	0	0	0	0	0	0	0.824452	0	0	0
AC104389.	0	0	0	0	0	0	0	5.556162	0	0	0	0	1.028084	0	0	0.824452	0	0	0
AC104390.	0	2.000781	0	0	1.107485	1.141887	0	0	0	1.921399	1.204755	0	0	0	0	0	2.052753	0.910133	0
AC104393.	0	3.001171	0	0	0	0	3.398721	0	0	0	0	0.920078	0	0	0.978006	0.824452	1.026376	0	0
AC104394.	0	4.001562	0	0	0	0	0	0	0	0	0	1.840156	0	0	0	0	0	0	0
AC104407.	0	0	0	1.039293	0	0	0	0	0	0	0	0	0	0	0	0	0	0.910133	0
AC104411.	9.609075	8.003124	5.994802	6.235757	3.322455	9.135096	5.098081	15.74246	0	11.52839	6.023774	3.680313	3.084253	2.792161	0.978006	10.71787	7.184634	5.4608	7.170464
AC104417.	0	0	3.996535	0	0	0	0	0	0	0	0	0	0	0	0	0	0	0	0
AC104435.	0	0	0	1.039293	0	1.141887	0	0	0	0.960699	0	0	0	0	0	0	0	0	0
AC104435.	0.960908	10.0039	0	0	4.42994	0	4.248401	0	0	0.960699	1.204755	0	0	0.93072	0	1.648904	1.026376	6.370934	2.688924
AC104445.	0	0	0	0	1.107485	0	0	0	0	0	0	0	1.028084	0	0.978006	0	0	0.910133	7.170464
AC104446.	87.44259	81.03163	65.94282	63.39686	46.51437	47.95925	56.92858	50.93149	38.5205	75.89524	65.05676	87.40743	91.49951	58.63538	69.43841	74.20066	61.58258	63.70934	69.91202
AC104447.	442.9784	342.1335	379.6708	595.5148	408.6619	327.7216	390.8529	436.1587	156.4166	242.0962	204.8083	335.8286	367.0261	394.6254	321.7639	465.8153	410.5505	439.5944	287.7149
AC104451.	0	0	0	0	1.107485	0	0	1.852054	0	0	0	0	0	0	0	0	0	0	0
AC104452.	89.3644	54.02108	53.95322	62.35757	67.55658	84.49964	79.02026	70.37805	155.2493	126.8123	179.5085	105.809	124.3982	81.90339	86.06451	81.62073	73.89909	71.90054	76.18618
AC104454.	0.960908	0	0	0	6.64491	0	0	0	0	0.960699	0	0	0	0	0	0	0	0	0
AC104457.	0	0	0	0	0	0	3.398721	0.926027	0	0	0	0	0	0	0	0	0	0.910133	0
AC104457.	0	0	2.997401	0	0	0	0	0	0	0	0	0	0	0.93072	0	0	0	0	0
AC104458.	18.25724	4.001562	35.96881	27.02161	25.47215	17.12831	14.44456	7.408216	18.6766	10.56769	3.614265	28.52243	31.87062	13.9608	13.69208	11.54233	9.237386	10.9216	3.585232
AC104461.	17.29634	13.00508	3.996535	3.117878	15.50479	4.567548	2.549041	10.1863	5.836439	5.764196	0	9.200782	9.252759	1.861441	8.802052	1.648904	1.026376	2.7304	0.896308
AC104462.	10.56998	7.002733	21.98094	19.74656	25.47215	7.993209	12.7452	11.11232	9.338302	16.33189	9.638039	5.520469	13.3651	7.445762	11.73607	18.13794	16.42202	3.640534	12.54831
AC104462.	3.84363	2.000781	4.995668	8.314342	1.107485	0	4.248401	0	0	6.724895	0	1.840156	2.056169	3.722881	0.978006	0	2.052753	0	3.585232
AC104463.	8.648168	1.00039	0.999134	1.039293	2.21497	5.709435	5.098081	12.96438	0	5.764196	12.04755	0	2.056169	0	0.978006	2.473355	15.39564	0.910133	3.585232
AC104463.	41.31902	35.01367	25.97748	15.58939	55.37425	44.53359	20.39233	53.70957	115.5615	63.40615	67.46627	51.52438	39.06721	27.92161	35.20821	67.60505	59.52982	34.58507	23.30401
AC104472.	0	4.001562	0	0	0	0	0	1.852054	8.171014	0.960699	4.81902	0.920078	1.028084	1.861441	5.868035	0	0	0.910133	6.274156
AC104472.	52.84991	21.0082	18.98354	25.98232	17.71976	6.851322	43.33369	3.704108	9.338302	22.09608	12.04755	10.12086	11.30893	10.23792	30.31818	12.36678	13.34289	24.5736	12.54831
AC104521.	14.41361	13.00508	6.993936	5.196464	6.64491	15.98642	11.04584	3.704108	12.84017	21.13538	16.86657	10.12086	14.39318	12.09936	10.75806	22.2602	12.31652	22.75334	18.82247
AC104532.	294.0377	187.073	245.7869	152.776	173.8751	170.1412	263.4009	160.2027	88.71387	128.7337	97.58515	195.0566	185.0552	178.6983	182.8871	144.2791	113.9278	218.432	173.8837
AC104534.	88.40349	105.041	172.8501	54.04322	159.4778	113.0468	134.2495	125.9397	21.01118	140.2621	125.2945	96.60821	84.30292	111.6864	114.4267	71.72731	60.5562	63.70934	71.70464
AC104561.	36.51449	11.00429	10.99047	8.314342	1.107485	4.567548	23.79105	30.55889	0	0	16.86657	5.520469	13.3651	22.33729	6.846041	16.48904	11.29014	25.48374	28.68185
AC104561.	0	5.001952	5.994802	1.039293	2.21497	6.851322	5.947762	12.03835	17.50932	11.52839	14.45706	11.96102	8.224675	6.515042	13.69208	1.648904	4.105505	8.191201	20.61508
AC104561.	18.25724	9.003514	17.98441	4.157171	1.107485	14.84453	7.647122	23.15068	14.00745	10.56769	19.27608	7.360626	10.28084	4.653602	4.890029	8.244518	8.21101	8.191201	20.61508
AC104563.	0.960908	9.003514	17.98441	8.314342	12.18233	15.98642	6.797442	16.66849	22.17847	7.685594	15.66181	15.64133	11.30893	11.16864	18.58211	12.36678	8.21101	16.3824	8.066772
AC104564.	19.21815	1.00039	4.995668	3.117878	2.21497	12.56076	1.69936	13.89041	40.85507	10.56769	2.40951	4.600391	8.224675	16.75297	0.978006	3.297807	0	1.820267	1.792616

AC104564.	15.37452	17.00664	26.97661	31.17878	22.1497	10.27698	29.73881	31.48492	4.669151	8.646293	28.91412	29.4425	20.56169	20.47585	45.96627	23.9091	26.68578	19.1128	20.61508
AC104564.	42.27993	52.0203	52.95408	74.82908	47.62185	99.34417	84.11834	54.6356	31.51677	100.8734	60.23774	76.36649	70.93782	56.77394	54.76833	66.7806	70.81996	45.50667	37.64493
AC104564.	156.6279	196.0765	157.8631	176.6798	171.6602	293.465	173.3348	194.4657	144.7437	230.5678	193.9655	244.7408	272.4424	164.7375	153.5469	164.8904	125.2179	208.4206	173.8837
AC104564.	3.84363	1.00039	3.996535	0	0	2.283774	0	0.926027	0	1.921399	0	1.840156	1.028084	2.792161	0.978006	0.824452	0	0	7.170464
AC104564.	86.48168	127.0496	211.8163	47.80747	304.5584	132.4589	203.0736	151.8684	475.0861	154.6726	150.5944	133.4113	138.7914	175.9061	312.9619	88.21635	66.71446	75.54107	97.69757
AC104574.	0	0	2.997401	1.039293	0	0	1.69936	0	0	0	0	2.760235	9.252759	8.376483	17.6041	18.96239	8.21101	1.820267	3.585232
AC104574.	1.921815	1.00039	0.999134	10.39293	1.107485	12.56076	5.947762	9.26027	3.501863	17.29259	9.638039	14.72125	16.44935	25.12945	18.58211	107.1787	85.18923	13.652	12.54831
AC104581.	1199.213	1031.403	1230.933	906.2633	1127.42	1292.616	1392.626	1273.287	1501.132	1213.363	1258.969	1042.449	1137.061	1189.461	1263.584	880.5146	826.2329	1392.504	1140.104
AC104581.	1.921815	0	4.995668	3.117878	0	1.141887	2.549041	0	0	2.882098	3.614265	4.600391	3.084253	4.653602	0.978006	3.297807	9.237386	1.820267	0.896308
AC104581.	14.41361	33.01288	40.96448	40.53242	5.537425	46.81737	54.37954	35.18903	39.68778	8.646293	10.84279	17.48149	13.3651	32.57521	39.12023	46.99375	49.26606	29.12427	26.88924
AC104581.	295.9595	339.1324	393.6587	452.0924	337.7829	220.3842	439.2847	371.3368	250.9669	370.8299	230.1082	406.6746	415.3461	352.743	317.8519	380.0723	445.4473	373.1547	411.4053
AC104590.	55.73264	74.02889	87.92376	31.17878	18.82724	49.10114	71.37314	40.74519	56.02981	41.31007	21.68559	58.88501	53.46039	28.85233	51.83431	23.9091	27.71216	59.15867	69.01571
AC104596.	143.1752	86.03358	115.8995	91.45776	88.59879	67.37133	147.8444	94.45476	92.21573	66.28825	83.12809	104.8889	91.49951	107.0328	102.6906	87.39189	90.32111	84.64241	62.74156
AC104596.	0	0	0	0	4.42994	0	5.098081	0	0	0	0	0.920078	0	0	3.912023	0	0	0	0
AC104619.	0	1.00039	0	0	0	0	0	0	0	0	1.204755	0	3.084253	0	0.978006	0	1.026376	0	0.896308
AC104619.	12.4918	7.002733	10.99047	5.196464	18.82724	6.851322	13.59488	13.89041	5.836439	5.764196	4.81902	8.280704	11.30893	22.33729	16.6261	5.771163	8.21101	20.02294	2.688924
AC104623.	0	0	0	0	0	0	0	0	0	0.960699	0	0.920078	0	0	0	1.648904	0	0	0
AC104623.	111.4653	187.073	131.8856	304.5128	34.33203	219.2423	316.9307	164.8328	32.68406	163.3189	77.10431	170.2145	261.1334	131.2316	120.2947	235.7932	287.3854	266.6691	235.729
AC104634.	5.765445	7.002733	3.996535	0	0	1.141887	0	19.44657	2.334575	2.882098	1.204755	11.04094	1.028084	11.16864	13.69208	4.946711	2.052753	0.910133	7.170464
AC104651.	0	0	0	3.117878	0	0	2.549041	0	0	3.842797	0	0.920078	1.028084	0	0	4.946711	0	3.640534	0.896308
AC104651.	14.41361	6.002343	4.995668	13.51081	14.3973	3.425661	10.19616	12.03835	0	13.44979	0	8.280704	7.196591	0.93072	5.868035	6.595615	5.131881	4.550667	0
AC104653.	41.31902	31.0121	52.95408	49.88605	57.58922	44.53359	36.53625	42.59724	74.70641	61.48475	30.11887	39.56336	28.78636	51.18962	49.8783	70.07841	44.13418	47.32694	46.60801
AC104653.	17.29634	7.002733	13.98787	15.58939	14.3973	5.709435	8.496802	21.29862	28.01491	4.803496	3.614265	13.80117	4.112337	17.68369	15.64809	14.01568	12.31652	6.370934	8.066772
AC104662.	3.84363	11.00429	12.98874	0	2.21497	7.993209	5.947762	11.11232	9.338302	0	0	6.440548	9.252759	9.307203	10.75806	0	1.026376	4.550667	7.170464
AC104662.	534.2646	620.2421	550.5226	371.0275	383.1898	395.0929	727.3263	633.4025	636.1718	303.581	331.3076	526.2847	563.3902	322.9599	260.1495	178.906	203.2225	363.1432	371.9678
AC104663.	0	0	0	0	0	0	0	1.69936	0	0	0	0	0	0	0	0	0	0	0
AC104667.	21.13997	55.02147	69.93936	106.0079	22.1497	54.81058	56.92858	56.48765	40.85507	24.01748	30.11887	28.52243	60.65698	88.41843	64.54838	48.64266	49.26606	90.10321	123.6905
AC104667.	1.921815	2.000781	6.993936	5.196464	0	2.283774	14.44456	1.852054	4.669151	4.803496	0	16.56141	5.140422	3.722881	7.824047	3.297807	3.079129	18.20267	6.274156
AC104685.	1.921815	0	0	0	1.107485	1.141887	0.84968	2.778081	2.334575	0	0	10.12086	5.140422	0	1.956012	0	0	2.7304	7.170464
AC104687.	5.765445	3.001171	0.999134	4.157171	0	9.135096	1.69936	0	0	1.921399	0	0	0	0	0	0.824452	6.158258	1.820267	0
AC104688.	0	2.000781	4.995668	0	0	4.567548	0.84968	0	0	5.764196	0	0	0	0	0	0	0	0	0
AC104692.	22.10087	0	10.99047	0	9.967364	0	20.39233	8.334243	0	0	0	0	2.056169	13.9608	7.824047	1.648904	2.052753	0	0
AC104692.	6.726353	0	4.995668	0	7.752394	0	9.346483	6.482189	0	0	0	0	0	5.584322	0	0	0	0.910133	0
AC104695.	0	1.00039	0.999134	0	0	0	0	0	3.501863	0	0	0.920078	0	0	0	0	0	0	0
AC104695.	62.45899	103.0402	97.9151	28.0609	38.76197	70.79699	79.02026	31.48492	95.71759	150.8298	113.247	93.84798	96.63993	105.1714	67.4824	40.39814	29.76491	151.0821	155.9576
AC104695.	85.52077	132.0515	464.5972	5.196464	198.2398	62.80379	142.7463	162.9808	66.5354	207.511	452.9878	143.5322	223.0943	204.7585	259.1715	23.08465	19.50115	32.7648	25.09662
AC104698.	0	4.001562	9.991337	0	0	0	0	0	0	0	0	0	0	0	0	0.824452	1.026376	0	0
AC104699.	1.921815	0	4.995668	0	1.107485	2.283774	0.84968	4.630135	0	0	4.81902	2.760235	4.112337	1.861441	6.846041	11.54233	0	9.101334	1.792616
AC104758.	21.13997	30.01171	20.98181	17.66798	7.752394	2.283774	0.84968	0.926027	14.00745	11.52839	9.638039	22.08188	19.5336	9.307203	0.978006	8.244518	12.31652	0.910133	2.688924
AC104758.	3.84363	9.003514	16.98527	1.039293	25.47215	0	5.098081	2.778081	33.85134	6.724895	15.66181	11.96102	4.112337	2.792161	5.868035	1.648904	24.63303	20.02294	8.066772
AC104758.	2.882723	264.1031	187.8371	297.2377	235.8943	1.141887	15.29424	70.37805	23.34575	66.28825	60.23774	58.88501	90.47142	23.26801	10.75806	231.671	239.1457	14.56213	17.02985
AC104758.	0.960908	3.001171	2.997401	4.157171	11.07485	0	2.549041	7.408216	9.338302	2.882098	7.228529	0.920078	6.168506	3.722881	0	4.122259	9.237386	0	0
AC104759.	0	1.00039	0	0	0	0	0	0	0	0.960699	0	0.920078	0	0	7.824047	4.946711	3.079129	0	0
AC104763.	0.960908	4.001562	4.995668	3.117878	15.50479	3.425661	6.797442	17.59451	8.171014	21.13538	10.84279	6.440548	7.196591	7.445762	8.802052	14.01568	18.47477	5.4608	3.585232
AC104763.	0	5.001952	5.994802	6.235757	1.107485	1.141887	3.398721	5.556162	0	4.803496	1.204755	2.760235	1.028084	0	0.978006	1.648904	0	0	0
AC104771.	0	2.000781	0.999134	1.039293	4.42994	1.141887	5.098081	0	0	7.685594	7.228529	3.680313	3.084253	5.584322	2.934017	1.648904	4.105505	1.820267	3.585232
AC104772.	0	0	0	0	0	0	3.398721	5.556162	11.67288	0	0	0	0	0	0.978006	1.648904	0	0	0
AC104777.	1.921815	2.000781	4.995668	0	0	0	0	0	0	0	0	0	1.028084	0	0.978006	0.824452	1.026376	9.101334	0.896308
AC104777.	0	0	0	0	0	0	0.84968	0	0	0	0	1.840156	0	0	0	0	0	0	0
AC104779.	0.960908	5.001952	4.995668	4.157171	2.21497	2.283774	16.14392	10.1863	0	17.29259	1.204755	12.8811	13.3651	7.445762	14.67009	5.771163	7.184634	3.640534	1.792616
AC104781.	0	0	1.998267	2.078586	4.42994	18.27019	3.398721	0.926027	2.334575	0	1.204755	2.760235	1.028084	0.93072	1.956012	0	1.026376	0	0.896308
AC104785.	46.12356	35.01367	44.96101	49.88605	44.2994	93.63473	27.18977	13.89041	92.21573	33.62447	109.6327	44.16376	28.78636	29.78305	52.81231	29.68027	36.94955	29.12427	51.98586
AC104791.	2.882723	2.000781	0.999134	11.43222	3.322455	2.283774	5.098081	0.926027	5.836439	10.56769	4.81902	5.520469	9.252759	6.515042	1.956012	8.244518	1.026376	7.281067	3.585232
AC104791.	11.53089	2.000781	7.993069	7.275049	15.50479	7.993209	5.947762	1.852054	14.00745	15.37119	9.638039	10.12086	14.39318	7.445762	4.890029	13.19123	14.36927	1.820267	1.792616
AC104793.	23.06178	43.01679	20.98181	7.275049	13.28982	12.56076	63.72602	51.85751	3.501863	22.09608	9.638039	34.96297	34.95487	32.57521	33.2522	5.771163	11.29014	36.40534	36.74863
AC104794.	4.804538	1.00039	2.997401	1.039293	3.322455	1.141887	3.398721	3.704108	0	1.921399	2.40951	8.280704	2.056169	10.23792	3.912023	0.824452	1.026376	2.7304	8.066772
AC104794.	1320.287	1213.474	1135.016	1292.88	1120.775	1167.009	1617.791	921.3969	2013.571	1331.529	1624.01	1325.833	1513.34	1224.828	1377.032	745.3045	771.8349	1071.227	852.3889
AC104794.	2.882723	1.00039	0	1.039293	0	2.283774	1.69936	1.852054	0	0.960699	0	0	4.112337	0	0	0.824452	1.026376	1.820267	0
AC104803.	0	0	0	0	0	0	0	0.926027	0	0	0	0	0	1.861441	0	0	0	0	0
AC104806.	1.921815	3.001171	7.993069	7.275049	4.42994	3.425661	6.797442	8.334243	0	0.960699	14.45706	3.680313	3.084253	3.722881	1.956012	2.473355	1.026376	7.281067	10.7557
AC104806.	104.7389	133.0519	166.8553	71.7112	107.426	65.08756	115.5565	122.2356	140.0745	48.99566	55.41872	93.84798	122.342	115.4093	102.6906	108.8276	126.2443	163.824	161.3354
AC104809.	35.55358	20.00781	10.99047	16.62868	80.8464	22.83774	4.248401	34.263	39.68778	4.803496	10.84279	41.40352	24.67402	134.0237	171.151	337.2008	357.1789	156.5429	71.70464

AC104809.	34.59267	10.0039	22.98007	13.51081	45.40688	5.709435	16.14392	19.44657	32.68406	77.81664	31.32363	37.72321	22.61786	40.02097	23.47214	122.0189	109.8223	61.88907	57.36371
AC104819.	9.609075	27.01054	46.95928	4.157171	4.42994	12.56076	18.69297	28.70684	9.338302	18.25329	14.45706	13.80117	8.224675	14.89152	24.45015	6.595615	1.026376	0.910133	0
AC104819.	1.921815	0	0.999134	0	0	0	0.84968	2.778081	0	2.882098	0	0	2.056169	0	0	0	0	0	0
AC104819.	4.804538	4.001562	14.987	10.39293	7.752394	1.141887	4.248401	12.03835	0	0	1.204755	8.280704	4.112337	8.376483	4.890029	4.122259	1.026376	2.7304	11.652
AC104823.	0	1.00039	0	0	0	0	0	0	0	0	0	0	0	0	0	0	1.026376	0	0
AC104825.	1886.261	1739.679	1648.571	1553.743	1434.193	2005.154	1443.607	1500.164	2053.259	1847.425	2056.517	1409.56	1641.851	1619.453	1616.644	1629.941	1834.134	1720.152	1717.326
AC104827.	13.45271	15.00586	14.987	1.039293	8.859879	11.41887	25.49041	9.26027	36.18592	6.724895	0	11.96102	17.47743	18.61441	10.75806	27.20691	22.58028	10.9216	8.066772
AC104831.	6.726353	10.0039	14.987	1.039293	9.967364	2.283774	6.797442	5.556162	2.334575	7.685594	0	0.920078	1.028084	9.307203	2.934017	11.54233	1.026376	2.7304	5.377848
AC104836.	0	0	0	0	0	0	0.84968	0	0	0.960699	0	0	1.028084	0.93072	0	3.297807	0	0	0.896308
AC104836.	384.363	333.13	460.6006	246.3124	386.5122	456.7548	252.355	314.8492	339.6807	336.2447	384.3168	220.8188	252.9088	308.0684	339.368	275.3669	312.0184	160.1835	207.9434
AC104837.	1.921815	0	0	1.039293	0	0	0.84968	0	3.501863	0	0	0.920078	5.140422	9.307203	0.978006	0	1.026376	0.910133	1.792616
AC104837.	8.648168	50.01952	39.96535	68.59332	19.93473	17.12831	59.47762	24.0767	8.171014	18.25329	37.3474	52.44446	38.03912	27.92161	30.31818	22.2602	27.71216	14.56213	34.95601
AC104843.	4.804538	6.002343	10.99047	2.078586	7.752394	7.993209	0.84968	16.66849	17.50932	9.606993	15.66181	10.12086	4.112337	11.16864	8.802052	13.19123	8.21101	18.20267	6.274156
AC104849.	0.960908	0	0	3.117878	4.42994	1.141887	0	0	0	0.960699	0	0.920078	4.112337	0	0.978006	0.824452	0	0	0
AC104850.	1.921815	1.00039	3.996535	1.039293	2.21497	2.283774	1.69936	1.852054	7.003726	0.960699	0	1.840156	0	1.861441	12.71408	0	0	0	0
AC104852.	12.4918	2.000781	5.994802	1.039293	9.967364	11.41887	12.7452	3.704108	0	22.09608	26.50461	6.440548	7.196591	0	0.978006	11.54233	15.39564	0.910133	7.170464
AC104938.	226.7742	275.1074	259.7748	351.281	169.4452	180.4181	257.4531	187.0575	240.4613	237.2927	216.8559	166.5342	199.4484	238.2644	250.3695	245.6866	237.0929	192.0382	215.1139
AC104958.	3.84363	0	0	0	0	1.141887	0	0	14.00745	0.960699	1.204755	0.920078	1.028084	1.861441	0.978006	2.473355	1.026376	4.550667	0.896308
AC104958.	5.765445	19.00742	5.994802	12.47151	29.90209	6.851322	8.496802	17.59451	37.35321	13.44979	21.68559	19.32164	21.58977	4.653602	19.56012	3.297807	9.237386	11.83173	19.71877
AC104961.	0	0	0	0	0	0	1.69936	0	1.167288	0	0	0	0	1.861441	3.912023	0	0	0	0
AC104961.	0	0	0	0	0	4.567548	0.84968	0	0	5.764196	0	0	0	0	0.978006	0	0	0	3.585232
AC104964.	0.960908	10.0039	7.993069	19.74656	9.967364	1.141887	11.89552	0.926027	19.84389	26.89958	10.84279	19.32164	26.73019	6.515042	6.846041	20.6113	8.21101	10.9216	6.274156
AC104964.	61.49808	42.0164	29.97401	17.66798	28.79461	38.82416	45.88273	86.12051	35.01863	35.54587	55.41872	52.44446	23.64594	54.9125	53.79032	45.34485	57.47707	37.31547	39.43755
AC104964.	433.3693	489.1909	664.4239	346.0845	536.0227	224.9517	453.7292	363.0026	459.9114	648.472	704.7816	460.0391	445.1605	457.9144	384.3563	390.7902	413.6296	343.1203	333.4266
AC104966.	7.68726	1.00039	0	2.078586	0	1.141887	0	0.926027	5.836439	0	0	5.520469	2.056169	3.722881	3.912023	0.824452	2.052753	14.56213	1.792616
AC104971.	30.74904	65.02538	63.94455	32.21808	47.62185	73.08077	39.08529	5.556162	79.37557	66.28825	51.80446	36.80313	43.17954	81.90339	70.41642	32.15362	44.13418	59.15867	82.46033
AC104971.	0	0	0	2.078586	0	0	0	0	0	0	0	0	2.056169	0.93072	0	0	0	0	0
AC104971.	0	0	0	0	0	0	1.69936	0.926027	0	0	0	0.920078	0	3.722881	0.978006	2.473355	0	0	4.48154
AC104978.	0	0	0	1.039293	8.859879	10.27698	0.84968	5.556162	0	0	0	0	1.028084	0.93072	1.956012	0	1.026376	0	3.585232
AC104982.	0	1.00039	0.999134	0	0	1.141887	0.84968	4.630135	0	0	0	0.920078	0	0.93072	3.912023	0	0	0	0.896308
AC104982.	1.921815	1.00039	0.999134	0	0	0	0.84968	1.852054	2.334575	0.960699	0	0	0	0	0.978006	1.648904	1.026376	6.370934	0
AC104982.	0.960908	0	0	1.039293	0	0	0	0.926027	0	0.960699	0	0	0	0	0	0.824452	0	0	0
AC104984.	0	0	11.9896	0	5.537425	1.141887	7.647122	11.11232	0	1.921399	1.204755	1.840156	2.056169	4.653602	7.824047	2.473355	5.131881	0.910133	0
AC104984.	10.56998	10.0039	10.99047	24.94303	16.61227	9.135096	9.346483	26.85478	7.003726	29.78168	12.04755	20.24172	11.30893	8.376483	7.824047	27.20691	20.52753	12.74187	17.02985
AC104984.	4.804538	0	3.996535	3.117878	6.64491	0	1.69936	0	0	0	0	1.840156	8.224675	2.792161	1.956012	0.824452	1.026376	2.7304	6.274156
AC104984.	12.4918	12.00469	6.993936	5.196464	0	19.41208	16.14392	12.03835	0	9.606993	22.89034	7.360626	6.168506	11.16864	7.824047	0.824452	13.34289	8.191201	7.170464
AC104984.	0	2.000781	2.997401	4.157171	0	1.141887	6.797442	3.704108	0	0.960699	0	8.280704	12.33701	1.861441	11.73607	3.297807	0	5.4608	9.859387
AC104985.	0	0	2.997401	0	3.322455	0	0	0	0	0	0	1.840156	3.084253	0	0	2.473355	1.026376	0	1.792616
AC104985.	0	1.00039	0	1.039293	0	1.141887	0	0	0	1.921399	0	0	1.028084	0	0	0	0	0	0.896308
AC104985.	0	1.00039	0	0	0	0	0	0	0	0	0	0	1.028084	0	0.978006	0	0	0.910133	0
AC104986.	47.08447	46.01796	88.9229	37.41454	66.4491	38.82416	74.77186	44.4493	3.501863	44.19217	19.27608	105.809	44.20763	32.57521	61.61437	63.48279	35.92317	54.608	64.53417
AC104986.	184.4942	301.1175	221.8077	115.3615	229.2494	219.2423	175.0341	224.0985	459.9114	400.6116	520.4541	213.4581	259.0773	168.4604	200.4912	150.8747	215.539	225.7131	180.1579
AC104996.	0.960908	0	0.999134	0	0	0	1.69936	0	0	0	0	0	5.140422	3.722881	0	0.824452	0	1.820267	0
AC104996.	7.68726	3.001171	4.995668	15.58939	2.21497	1.141887	8.496802	1.852054	8.171014	10.56769	1.204755	15.64133	11.30893	4.653602	4.890029	5.771163	5.131881	12.74187	11.652
AC104997.	0	0	0	0	0	0	0	0	0	0	0	0	0	0	0	3.297807	0	1.820267	0
AC105001.	51.88901	39.01523	50.95582	71.7112	81.95388	50.24303	59.47762	53.70957	19.84389	62.44545	75.89956	84.6472	64.76932	85.62627	62.59237	46.1693	65.68808	94.65388	101.2828
AC105020.	311.334	250.0976	299.7401	202.6621	423.0592	389.3835	279.5448	248.1752	346.6845	429.4326	342.1504	312.8266	356.7453	208.4813	203.4252	237.4421	222.7236	274.8603	208.8398
AC105020.	0.960908	2.000781	8.992203	10.39293	0	4.567548	0	0	2.334575	8.646293	2.40951	3.680313	1.028084	2.792161	5.868035	14.84013	10.26376	2.7304	0
AC105020.	127.8007	77.03006	103.9099	71.7112	86.38382	116.4725	100.2623	96.30681	91.04844	120.0874	165.0514	105.809	134.6791	99.58707	95.84457	46.99375	78.0046	50.96747	73.49725
AC105020.	61.49808	86.03358	71.93762	45.72888	97.45867	66.22945	51.83049	106.4931	72.37184	60.52405	106.0184	79.12673	74.02207	93.07203	70.41642	49.46711	61.58258	40.956	60.05263
AC105021.	0	1.00039	3.996535	0	3.322455	0	0	0	0	0	0	0	1.028084	0	0.978006	0.824452	0	7.281067	2.688924
AC105031.	0.960908	1.00039	0	0	0	0	0.84968	0	0	0	0	0	0	3.722881	0	0	0	0	0
AC105036.	0.960908	2.000781	1.998267	2.078586	0	4.567548	10.19616	3.704108	19.84389	9.606993	1.204755	0	0	13.9608	0	3.297807	0	0.910133	3.585232
AC105036.	0	2.000781	0	0	0	0	0	0	2.334575	0	1.204755	0	1.028084	0	0	0	0	0	0
AC105036.	42.27993	46.01796	80.92983	58.2004	28.79461	39.96605	76.47122	91.67668	18.6766	53.79916	27.70936	54.28462	76.07824	69.80402	43.03226	51.94047	45.16056	48.23707	39.43755
AC105046.	38.4363	13.00508	24.97834	33.25737	18.82724	21.69585	278.6951	0	52.52795	15.37119	19.27608	37.72321	37.01104	31.64449	35.20821	7.420066	7.184634	35.4952	43.02278
AC105052.	13.45271	37.01445	47.95842	33.25737	23.25718	13.70264	19.54265	37.96711	38.5205	45.15286	55.41872	15.64133	15.42127	34.43665	38.14223	9.06897	15.39564	14.56213	23.30401
AC105052.	23.06178	57.02225	53.95322	30.13949	62.01916	51.38492	38.23561	31.48492	148.2455	59.56335	72.28529	41.40352	51.40422	39.09025	62.59237	28.03136	28.73854	27.304	24.20031
AC105052.	119.1525	152.0593	192.8328	231.7623	369.9	235.2287	287.1919	196.3177	99.21946	300.6989	234.9272	142.6121	156.2688	275.4932	305.1378	153.348	134.4553	178.3861	168.5059
AC105053.	50.9281	43.01679	76.93329	48.84676	43.19191	19.41208	83.26866	14.81643	24.51304	21.13538	14.45706	13.80117	35.98295	47.46674	51.83431	28.85581	13.34289	72.81067	74.39356
AC105074.	57.65445	40.01562	42.96275	30.13949	40.97694	30.83095	19.54265	30.55889	95.71759	133.5372	189.1465	34.04289	37.01104	39.09025	35.20821	36.27588	45.16056	41.86614	17.92616
AC105081.	0	0	0	1.039293	0	0	0	0	0	0	0	0	0	0	0	0	0	0.910133	0

AC105094.	0.960908	0	0	0	0	0	0	0	1.167288	0	0	0	0	0	0	0	0	0.910133	0.896308
AC105101.	6.726353	12.00469	6.993936	12.47151	22.1497	10.27698	5.098081	19.44657	8.171014	8.646293	6.023774	13.80117	1.028084	0.93072	5.868035	2.473355	12.31652	7.281067	0.896308
AC105105.	1.921815	1.00039	0	0	0	0	1.69936	0	0	0	0	0	0	0	0	0	0	0.910133	0
AC105105.	0	0	0	1.039293	0	0	0	0	0	4.803496	0	0	0	0	1.956012	6.595615	1.026376	4.550667	0
AC105105.	0.960908	4.001562	2.997401	6.235757	0	0	0	18.52054	0	0	4.81902	4.600391	0	0	0	0.824452	3.079129	0	5.377848
AC105105.	0	0	0	0	0	0	0	0	0	0.960699	0	0	0	0	0	0.824452	1.026376	0	0
AC105105.	8.648168	0	0.999134	2.078586	0	0	5.098081	0	0	0	0	9.200782	6.168506	4.653602	6.846041	4.122259	5.131881	3.640534	2.688924
AC105114.	0.960908	1.00039	0.999134	0	11.07485	1.141887	11.89552	0.926027	0	6.724895	8.433284	1.840156	4.112337	0	4.890029	0	0	0	0
AC105118.	129.7225	68.02655	47.95842	82.10413	130.6832	143.8778	44.18337	103.715	135.4054	95.10923	185.5323	166.5342	190.1956	60.49682	57.70234	129.4389	146.7718	229.3536	276.9592
AC105129.	11.53089	5.001952	0.999134	3.117878	8.859879	0	8.496802	14.81643	4.669151	2.882098	14.45706	6.440548	11.30893	10.23792	10.75806	14.84013	25.65941	7.281067	31.37078
AC105129.	0	1.00039	0	0	0	5.709435	0	7.408216	0	1.921399	0	0	0	0	0	0	0	0	0
AC105129.	22.10087	20.00781	5.994802	29.1002	14.3973	4.567548	54.37954	27.78081	0	5.764196	0	31.28266	9.252759	14.89152	15.64809	16.48904	13.34289	7.281067	15.23724
AC105137.	7.68726	2.000781	1.998267	2.078586	0	3.425661	0.84968	0.926027	0	0	0	0.920078	4.112337	5.584322	0	0	0	0.910133	2.688924
AC105137.	1.921815	0	1.998267	0	1.107485	0	0	1.852054	0	10.56769	14.45706	0.920078	0	1.861441	0	3.297807	2.052753	1.820267	1.792616
AC105137.	11.53089	15.00586	32.97141	3.117878	17.71976	17.12831	5.098081	37.04108	40.85507	24.97818	71.08054	7.360626	21.58977	14.89152	18.58211	11.54233	2.052753	31.85467	4.48154
AC105150.	0	1.00039	0	0	0	0	0	0	0	0	4.81902	0	0	0	0	0	0	0	0
AC105180.	0	1.00039	0.999134	4.157171	0	4.567548	0	0	0	0	0	3.680313	0	5.584322	0	0	0	0	0.896308
AC105185.	0	1.00039	0	0	0	1.141887	0	0	0	0	0	0	0	0	0	0.824452	0	0	0
AC105202.	183.5333	204.0796	174.8484	322.1808	156.1554	220.3842	298.2378	278.7341	149.4128	195.9826	125.2945	167.4542	216.9258	125.6472	121.2727	236.6177	253.5149	176.5659	176.5727
AC105206.	0	0	3.996535	4.157171	1.107485	1.141887	3.398721	5.556162	0	0	0	0.920078	1.028084	5.584322	1.956012	2.473355	0	1.820267	8.066772
AC105206.	274.8196	290.1132	322.7202	336.7309	244.7542	351.7012	315.2314	325.9615	117.8961	309.3452	148.1848	326.6278	254.9649	279.2161	287.5337	284.4359	331.5195	330.3784	301.1595
AC105219.	174.8852	155.0605	184.8397	171.4833	218.1745	163.2898	113.8572	180.5753	151.7474	122.0088	246.9747	196.8967	190.1956	142.4002	168.217	158.2948	159.0883	157.4531	179.2616
AC105219.	0	0	0	0	1.107485	1.141887	0	0	1.167288	0	6.023774	0	0	0	0	0	0	0	0.896308
AC105219.	0	0	0	0	0	0	0.84968	0	0	0	0	0	0	0	0.978006	0.824452	0	0	0
AC105219.	2.882723	13.00508	9.991337	10.39293	2.21497	5.709435	1.69936	26.85478	9.338302	11.52839	1.204755	2.760235	9.252759	11.16864	5.868035	9.893422	5.131881	18.20267	18.82247
AC105227.	2.882723	0	0	0	0	0	0	0	0	0	0	0	0	0	0	0	0	0	0
AC105233.	0	2.000781	0.999134	7.275049	1.107485	0	0.84968	0	5.836439	1.921399	0	1.840156	1.028084	0.93072	1.956012	0.824452	0	2.7304	0
AC105233.	0	0	0	0	0	0	0.84968	0	0	1.921399	0	0	0	0	0	0.824452	0	0.910133	0
AC105233.	0	0	0	0	0	0	0	0	0	0	0	1.840156	0	0	0	0	0	0	0
AC105233.	0	8.003124	3.996535	2.078586	3.322455	6.851322	0	3.704108	10.50559	5.764196	8.433284	1.840156	0	0.93072	0.978006	1.648904	1.026376	1.820267	4.48154
AC105233.	70.14625	238.0929	212.8155	199.5442	150.6179	283.188	96.86355	208.3561	147.0783	80.69874	110.8374	149.9728	124.3982	192.6591	184.8431	124.4922	133.4289	192.0382	170.2985
AC105235.	93.20803	21.0082	142.8761	20.78586	36.547	69.65511	37.38593	95.38078	112.0596	63.40615	120.4755	26.68227	17.47743	26.06017	48.90029	18.13794	34.89679	65.52961	44.8154
AC105243.	0	2.000781	7.993069	1.039293	3.322455	0	0	3.704108	0	1.921399	0	0	2.056169	1.861441	1.956012	0	0	3.640534	5.377848
AC105250.	6.726353	12.00469	0.999134	9.353635	2.21497	7.993209	3.398721	1.852054	17.50932	8.646293	6.023774	3.680313	5.140422	8.376483	7.824047	6.595615	5.131881	11.83173	1.792616
AC105265.	0	0	0	2.078586	8.859879	0	0	0	0	2.882098	0	0	3.084253	0	0	0	0	0	0
AC105271.	15.37452	17.00664	17.98441	49.88605	55.37425	43.39171	16.14392	25.00273	43.18965	57.64196	44.57593	28.52243	16.44935	60.49682	31.29619	14.01568	7.184634	10.9216	16.13354
AC105275.	2.882723	0	0.999134	0	6.64491	0	2.549041	0	1.167288	9.606993	0	0.920078	0	3.722881	6.846041	0	1.026376	12.74187	3.585232
AC105277.	157.5888	187.073	209.8181	126.7937	167.2302	181.56	149.5437	186.1314	381.7031	154.6726	202.3988	148.1326	175.8024	155.4303	177.0191	124.4922	120.086	163.824	107.557
AC105285.	302.6859	202.0789	242.7895	174.6012	186.0575	269.4853	224.3156	319.4793	241.6286	233.4499	279.5031	233.6999	259.0773	226.165	241.5674	184.6772	133.4289	125.5984	164.0244
AC105339.	45.16265	56.02186	56.95062	58.2004	24.36467	66.22945	45.88273	47.22738	35.01863	57.64196	34.93789	68.08579	69.90974	15.82225	56.72434	36.27588	50.29244	40.956	51.98586
AC105339.	97.05166	117.0457	103.9099	123.6758	48.72934	93.63473	141.8966	121.3095	85.212	127.773	78.30907	120.5302	134.6791	80.97267	96.82258	77.49847	86.21561	149.2619	115.6237
AC105339.	13.45271	20.00781	10.99047	1.039293	11.07485	10.27698	28.03945	13.89041	8.171014	8.646293	24.0951	9.200782	25.70211	16.75297	20.53812	5.771163	12.31652	10.01147	12.54831
AC105339.	49.00628	46.01796	24.97834	18.70727	42.08443	43.39171	39.08529	41.67122	70.03726	44.19217	46.98544	33.12282	38.03912	27.92161	42.05425	25.55801	23.60665	52.78774	34.0597
AC105343.	0	0	0.999134	0	0	0	0	0	0	0	0	0	1.028084	0	0	0	0	0	0
AC105345.	559.2482	613.2393	581.4958	373.1061	717.6502	548.1058	557.3902	423.1944	726.053	755.1096	979.4657	745.2634	698.0693	307.1377	311.0059	394.9124	445.4473	897.3915	740.3504
AC105345.	0.960908	4.001562	2.997401	6.235757	1.107485	2.283774	0.84968	0	0	0	0	0	1.028084	14.89152	1.956012	0	0	0	3.585232
AC105362.	0	0	0.999134	0	0	0	0	0	0	0.960699	0	0	0	0	0	0	0	0	0
AC105383.	645.7299	789.3081	769.3329	682.8153	812.8939	706.8281	623.6653	799.1613	311.6658	657.1183	742.129	630.2536	850.2258	671.9801	680.6921	706.5552	609.6675	521.5064	543.1626
AC105384.	1.921815	1.00039	3.996535	7.275049	0	0	0.84968	3.704108	14.00745	10.56769	9.638039	1.840156	2.056169	0.93072	1.956012	4.946711	10.26376	0.910133	3.585232
AC105384.	0.960908	10.0039	3.996535	2.078586	15.50479	14.84453	3.398721	8.334243	14.00745	8.646293	15.66181	7.360626	2.056169	11.16864	14.67009	1.648904	4.105505	19.1128	8.963079
AC105389.	0	0	0.999134	1.039293	4.42994	0	0	0	0	1.921399	0	0	3.084253	0	0	0	5.131881	0	0.896308
AC105389.	37.47539	21.0082	46.95928	27.02161	13.28982	15.98642	42.48401	14.81643	3.501863	25.93888	19.27608	29.4425	40.09529	35.36737	28.36217	29.68027	37.97592	29.12427	25.99293
AC105389.	0	0	3.996535	2.078586	0	0	3.398721	0	4.669151	0	0	1.840156	2.056169	0	0	0	0	0	4.48154
AC105390.	0	0	0	2.078586	0	0	0	0	5.836439	0	0	0	1.028084	1.861441	3.912023	0.824452	1.026376	0	0.896308
AC105393.	2.882723	0	4.995668	0	0	1.141887	0.84968	3.704108	0	0	0	2.760235	0	0	0	0.824452	0	0	0
AC105393.	0	0	0	0	0	0	0.84968	0	0	0	0	0	1.028084	0	0.978006	1.648904	1.026376	0.910133	0
AC105398.	74.95079	66.02577	26.97661	98.73281	148.403	89.06719	35.68657	67.59997	73.53913	40.34937	59.03299	92.00782	123.3701	57.70466	97.80058	64.30724	50.29244	68.26001	90.5271
AC105399.	0	8.003124	0	0	0	0	3.398721	0	0	0	0	0	0	0	0	0	0	0	0
AC105402.	0	4.001562	1.998267	1.039293	1.107485	10.27698	15.29424	1.852054	0	0.960699	0	7.360626	1.028084	6.515042	8.802052	0.824452	1.026376	11.83173	3.585232
AC105402.	0	3.001171	9.991337	4.157171	0	2.283774	3.398721	5.556162	1.167288	1.921399	0	0	0	0	0	0.824452	0	3.640534	0
AC105402.	6.726353	7.002733	10.99047	15.58939	5.537425	2.283774	12.7452	0.926027	5.836439	13.44979	0	16.56141	11.30893	2.792161	2.934017	14.01568	9.237386	9.101334	2.688924
AC105411.	39.39721	23.00898	37.96708	27.02161	9.967364	39.96605	16.14392	29.63287	68.86998	21.13538	34.93789	25.76219	27.75828	16.75297	13.69208	14.01568	26.68578	20.02294	19.71877

AC105415.	0.960908	0	3.996535	0	1.107485	0	0.84968	0	1.167288	0	0	2.760235	3.084253	4.653602	0.978006	1.648904	0	1.820267	4.48154
AC105429.	200.8297	134.0523	163.8579	106.0079	179.4126	75.36454	157.1908	54.6356	199.6062	219.0394	345.7646	122.3704	156.2688	194.5205	228.8534	73.37621	74.92547	259.388	199.8767
AC105429.	3.84363	6.002343	5.994802	10.39293	4.42994	0	5.947762	0	9.338302	0.960699	4.81902	0.920078	0	0	0	0	0	0	4.48154
AC105430.	4.804538	1.00039	0.999134	5.196464	0	6.851322	0	0	0	0	2.40951	6.440548	4.112337	0.93072	1.956012	0	4.105505	2.7304	0
AC105446.	18.25724	35.01367	12.98874	10.39293	17.71976	36.54038	16.14392	29.63287	59.53167	24.97818	63.85201	34.04289	17.47743	19.54513	20.53812	13.19123	13.34289	50.96747	39.43755
AC105460.	0	0	0	0	0	0	0	0	0	0	7.228529	0	0	0	0	0	0	0	0
AC105749.	98.01257	113.0441	102.9108	130.9509	66.4491	99.34417	70.52346	60.19176	129.5689	114.3232	101.1994	91.08774	51.40422	58.63538	59.65836	113.7744	102.6376	69.17014	81.56402
AC105760.	0	0	4.995668	1.039293	8.859879	5.709435	2.549041	7.408216	0	3.842797	6.023774	3.680313	1.028084	0.93072	0.978006	2.473355	1.026376	0	2.688924
AC105760.	188.3379	127.0496	236.7947	269.1768	186.0575	155.2966	211.5704	162.0547	101.554	132.5765	78.30907	196.8967	167.5778	148.9152	160.393	165.7148	171.4048	152.9024	140.7203
AC105910.	0	0	0	0	1.107485	0	3.398721	0	0	0	3.614265	0	0	0	0	0	2.052753	0	0.896308
AC105914.	0	0	0	1.039293	0	0	0	6.482189	0	0	2.40951	0.920078	0	0	0	0.824452	0	0	0
AC105917.	3.84363	0	0	0	0	0	0.84968	0	5.836439	7.685594	4.81902	0	0	0	0	0	0	0	0
AC105924.	0	0	0	0	0	0	0	0	0	0	0	0	0	0	0	0	4.105505	0	0
AC105935.	35.55358	26.01015	13.98787	18.70727	16.61227	5.709435	15.29424	32.41095	11.67288	9.606993	24.0951	31.28266	13.3651	10.23792	20.53812	13.19123	36.94955	28.21414	21.51139
AC105935.	0	0	0	0	0	0	0	0	2.334575	0	0	0	1.028084	0	0.978006	0	0	0	0
AC105940.	0	0	0	0	4.42994	0	0	0	0	0	0	0	1.028084	0.93072	0	0	0	0	0.896308
AC105940.	0	0	0.999134	0	0	0	0	0	0	0	0	0	1.028084	0	0.978006	0	0	0	0
AC105942.	1246.297	888.3467	1774.461	1080.864	1593.671	1029.982	974.5832	1039.928	1609.69	1930.045	2481.795	1134.456	1143.23	1417.487	1424.954	1089.101	1056.141	1645.521	1516.553
AC105999.	0	1.00039	0	1.039293	0	0	4.248401	0	0	0	0	3.680313	0	0.93072	0.978006	0	0	1.820267	3.585232
AC106017.	8.648168	1.00039	1.998267	0	0	4.567548	0	0	0	2.882098	7.228529	0	0	0	0.978006	4.946711	7.184634	1.820267	0.896308
AC106017.	12.4918	3.001171	7.993069	2.078586	3.322455	5.709435	3.398721	3.704108	9.338302	8.646293	9.638039	11.04094	8.224675	4.653602	9.780058	4.122259	7.184634	5.4608	6.274156
AC106028.	0.960908	0	5.994802	0	0	1.141887	0	2.778081	0	3.842797	0	0	4.112337	0	0	1.648904	10.26376	0	0
AC106028.	4.804538	12.00469	8.992203	10.39293	17.71976	3.425661	3.398721	12.96438	17.50932	15.37119	13.2523	14.72125	18.50552	10.23792	17.6041	18.13794	3.079129	14.56213	15.23724
AC106028.	129.7225	146.057	90.92116	55.08252	184.95	134.7427	74.77186	108.3452	128.4017	124.8909	106.0184	95.68814	116.1735	134.9544	124.2067	108.0032	123.1652	149.2619	99.49018
AC106028.	0.960908	2.000781	1.998267	4.157171	1.107485	2.283774	0.84968	4.630135	0	9.606993	0	4.600391	4.112337	0	2.934017	0	5.131881	1.820267	0
AC106028.	5.765445	0	9.991337	3.117878	0	3.425661	5.098081	4.630135	8.171014	1.921399	9.638039	0.920078	3.084253	3.722881	0	0.824452	1.026376	5.4608	1.792616
AC106037.	63.4199	142.0554	149.87	108.0864	109.641	139.3102	103.661	168.5369	19.84389	213.2752	106.0184	126.9708	151.1284	133.093	123.2287	88.21635	100.5849	126.5085	69.91202
AC106037.	28.82723	23.00898	62.94542	38.45383	47.62185	18.27019	41.63433	25.92876	11.67288	40.34937	31.32363	99.36845	38.03912	41.88241	27.38416	16.48904	21.5539	26.39387	20.61508
AC106038.	114.348	85.03319	185.8389	89.37918	104.1036	59.37812	122.354	128.7178	71.20455	139.3014	119.2707	183.0956	163.4654	198.2434	152.5689	87.39189	91.34749	144.7112	143.4093
AC106038.	0	4.001562	0	0	0	0	0	0	0	0	0	0	0	0	0	0	0	0	0
AC106047.	102.8171	109.0426	139.8787	10.39293	63.12664	108.4793	65.42538	116.6794	32.68406	158.5154	114.4517	92.9279	120.2859	106.1021	97.80058	61.00944	81.08372	137.4301	122.7942
AC106052.	33.63176	29.01132	10.99047	19.74656	2.21497	37.68227	11.04584	31.48492	15.17474	33.62447	6.023774	18.40156	39.06721	16.75297	32.27419	7.420066	14.36927	24.5736	8.066772
AC106053.	0	0	0	0	0	0	0	0	0	0	0	0	0	0	0	1.648904	0	0	0
AC106706.	0	0	0	0	0	0	0	0	0	0	0	5.520469	0	0	0	0.824452	0	0	0
AC106707.	0.960908	0	0	8.314342	0	0	5.098081	1.852054	0	1.921399	0	0	0	0	0	0	0	0	0.896308
AC106707.	0	0	0	0	0	0	0.84968	0.926027	3.501863	0	0	0	0	0	0	0	1.026376	0	4.48154
AC106712.	0.960908	0	0	0	1.107485	0	0	0	0	0	0	3.680313	0	0	0	0.824452	0	2.7304	0
AC106729.	2.882723	0	0	0	13.28982	0	0	0	0	0	0	0	0	1.861441	0.978006	1.648904	1.026376	0	0
AC106730.	1.921815	1.00039	1.998267	0	4.42994	0	0.84968	8.334243	0	5.764196	1.204755	2.760235	4.112337	4.653602	1.956012	10.71787	10.26376	6.370934	2.688924
AC106730.	0	0	0	0	0	1.141887	0	0.926027	0	0	0	0	4.112337	0	0	0	0	0	3.585232
AC106736.	0	0	0	0	0	0	0	0	0	0	0	0	0	0	0	1.648904	0	0	0
AC106738.	0	0	0	0	0	0	0	0	1.167288	0	0	0	0	0	0	0	0	0	3.585232
AC106739.	0	1.00039	0	0	0	0	0	0	0	0	0	0	0	0.93072	0	0	1.026376	0	5.377848
AC106745.	27.86632	11.00429	23.97921	8.314342	16.61227	13.70264	15.29424	18.52054	15.17474	18.25329	26.50461	16.56141	13.3651	35.36737	30.31818	14.01568	11.29014	10.01147	7.170464
AC106748.	0.960908	0	0.999134	0	0	0	4.248401	0.926027	0	0	0	5.520469	4.112337	0	0	0.824452	0	0	0.896308
AC106754.	0	0	23.97921	0	0	0	52.68017	0	0	0	0	2.760235	6.168506	0	0	0	0	0	0
AC106760.	0	21.0082	6.993936	7.275049	0	6.851322	4.248401	7.408216	1.167288	7.685594	21.68559	7.360626	12.33701	17.68369	8.802052	4.946711	5.131881	4.550667	15.23724
AC106772.	15.37452	33.01288	39.96535	28.0609	18.82724	29.68906	9.346483	35.18903	39.68778	32.66377	12.04755	30.36258	31.87062	18.61441	15.64809	6.595615	15.39564	7.281067	28.68185
AC106772.	0.960908	4.001562	14.987	0	14.3973	26.2634	7.647122	0	19.84389	21.13538	54.21397	26.68227	16.44935	8.376483	8.802052	0.824452	4.105505	9.101334	15.23724
AC106779.	141.2534	132.0515	130.8865	108.0864	60.91167	122.1819	73.0725	128.7178	120.2306	72.05244	165.0514	115.0098	128.5105	124.7165	140.8328	126.1411	122.1388	107.3957	113.8311
AC106782.	4.804538	5.001952	0.999134	1.039293	23.25718	6.851322	2.549041	0.926027	15.17474	10.56769	15.66181	0	3.084253	2.792161	1.956012	2.473355	3.079129	6.370934	7.170464
AC106782.	217.1651	188.0734	197.8285	222.4087	140.6506	172.4249	186.08	205.578	152.9147	172.9259	216.8559	286.1443	217.9539	250.3638	223.9633	231.671	188.8532	284.8718	293.0927
AC106782.	0	0	0	1.039293	0	0	0.84968	0	0	0	0	0.920078	0	0	0.978006	0	0	0.910133	0
AC106782.	16.33543	4.001562	0	0	0	9.135096	0	1.852054	0	0	0	1.840156	1.028084	0	1.956012	0	2.052753	0	3.585232
AC106782.	19.21815	45.01757	35.96881	19.74656	31.00958	28.54718	8.496802	48.15341	50.19337	57.64196	62.64725	32.20274	14.39318	35.36737	38.14223	39.57369	16.42202	36.40534	30.47447
AC106782.	61.49808	76.02967	98.91423	60.27898	74.20149	123.3238	62.02666	84.26846	82.87743	122.0088	140.9563	76.36649	88.41526	90.27987	74.32844	46.1693	37.97592	70.08027	60.94894
AC106785.	3.84363	0	0	0	3.322455	2.283774	0	0.926027	0	4.803496	0	0	1.028084	0	0	1.648904	0	0	1.792616
AC106785.	0	0	0	0	0	0	0	11.11232	0	0	0	0	0	0	0	0	0	0	0
AC106785.	1.921815	0	0	1.039293	1.107485	0	0.84968	0	0	0	0	0	1.028084	0	1.956012	0.824452	0	3.640534	0
AC106786.	27.86632	7.002733	15.98614	10.39293	18.82724	5.709435	30.58849	7.408216	1.167288	4.803496	6.023774	33.12282	16.44935	18.61441	8.802052	21.43575	17.4484	19.1128	14.34093
AC106786.	24.02269	13.00508	22.98007	24.94303	12.18233	13.70264	20.39233	5.556162	3.501863	5.764196	22.89034	16.56141	37.01104	15.82225	18.58211	21.43575	23.60665	16.3824	25.09662
AC106788.	68.22443	41.01601	38.96621	46.76817	40.97694	26.2634	27.18977	34.263	42.02236	57.64196	75.89956	32.20274	32.8987	40.95169	18.58211	50.29156	59.52982	23.66347	24.20031

AC106788.	0	0	0	0	2.21497	0	0	0	0	0	0	0	1.028084	0	0	0	2.052753	3.640534	0.896308
AC106789.	1.921815	5.001952	0.999134	0	1.107485	2.283774	5.098081	11.11232	0	1.921399	1.204755	0	0	3.722881	3.912023	12.36678	1.026376	3.640534	7.170464
AC106791.	230.6178	255.0996	407.6465	255.666	243.6467	171.2831	412.0949	371.3368	152.9147	236.332	139.7516	264.0625	240.5717	290.3847	311.9839	159.9437	157.0356	171.1051	172.0911
AC106791.	4.804538	26.01015	18.98354	6.235757	17.71976	14.84453	9.346483	28.70684	35.01863	26.89958	7.228529	4.600391	18.50552	15.82225	16.6261	8.244518	7.184634	7.281067	15.23724
AC106795.	311.334	756.2952	661.4265	516.5285	693.2856	746.7941	332.225	664.8874	1505.801	1488.123	2009.531	323.8675	383.4755	590.0767	614.1877	331.4296	336.6514	273.9502	253.6551
AC106795.	137.4098	192.075	156.864	123.6758	227.0344	172.4249	227.7143	119.4575	199.6062	254.5853	316.8505	108.5692	150.1003	156.361	127.1408	88.21635	138.5608	63.70934	99.49018
AC106795.	6.726353	3.001171	0.999134	6.235757	1.107485	17.12831	8.496802	3.704108	3.501863	6.724895	3.614265	1.840156	11.30893	0.93072	9.780058	4.946711	1.026376	0.910133	5.377848
AC106795.	5.765445	11.00429	17.98441	29.1002	12.18233	17.12831	19.54265	6.482189	0	24.01748	16.86657	10.12086	11.30893	13.03008	19.56012	9.893422	8.21101	4.550667	5.377848
AC106796.	0	0	2.997401	1.039293	12.18233	4.567548	7.647122	1.852054	0	0	0	1.840156	0	0	0.978006	4.946711	2.052753	0	0
AC106798.	29.78813	66.02577	179.8441	145.501	114.0709	15.98642	67.97442	225.9506	56.02981	11.52839	25.29985	57.96493	50.37613	161.9453	135.9428	18.96239	11.29014	35.4952	6.274156
AC106799.	0	0	0.999134	0	0	0	0	0	0	0	0	0	3.084253	0	0	2.473355	0	0	0
AC106800.	5.765445	18.00703	3.996535	8.314342	4.42994	5.709435	8.496802	1.852054	2.334575	12.48909	18.07132	4.600391	6.168506	2.792161	7.824047	7.420066	2.052753	2.7304	6.274156
AC106800.	0	4.001562	5.994802	4.157171	6.64491	14.84453	5.947762	12.03835	36.18592	10.56769	9.638039	1.840156	12.33701	13.03008	5.868035	6.595615	7.184634	4.550667	5.377848
AC106800.	0	0	3.996535	0	0	0	0.84968	0	1.167288	0	0	0	0	0	0	0.824452	0	0	0.896308
AC106800.	5.765445	1.00039	0	4.157171	3.322455	6.851322	0.84968	2.778081	14.00745	2.882098	16.86657	2.760235	1.028084	3.722881	2.934017	2.473355	6.158258	1.820267	0.896308
AC106801.	0.960908	2.000781	0	0	6.64491	0	0.84968	2.778081	7.003726	1.921399	0	3.680313	8.224675	1.861441	1.956012	5.771163	6.158258	1.820267	3.585232
AC106818.	117.2307	117.0457	312.7288	104.9686	149.5105	44.53359	153.7921	199.0958	18.6766	75.89524	57.82823	268.6628	182.999	129.3701	165.283	74.20066	53.37157	91.01334	139.824
AC106820.	8.648168	5.001952	3.996535	8.314342	1.107485	1.141887	0.84968	9.26027	26.84762	2.882098	7.228529	12.8811	1.028084	4.653602	4.890029	2.473355	6.158258	2.7304	9.859387
AC106820.	4.804538	19.00742	29.97401	17.66798	15.50479	13.70264	2.549041	4.630135	59.53167	24.01748	28.91412	6.440548	22.61786	13.9608	24.45015	11.54233	4.105505	15.47227	13.44462
AC106820.	16.33543	42.0164	29.97401	18.70727	29.90209	41.10793	23.79105	37.04108	106.2232	48.03496	36.14265	32.20274	40.09529	44.67457	30.31818	14.01568	30.79129	40.04587	34.0597
AC106820.	189.2988	130.0508	119.896	248.391	124.0383	168.9993	153.7921	185.2054	73.53913	149.8691	109.6327	161.0137	167.5778	162.8761	188.7551	204.4641	177.5631	100.1147	130.861
AC106820.	33.63176	27.01054	35.96881	14.5501	6.64491	21.69585	28.88913	31.48492	23.34575	19.21399	12.04755	35.88305	28.78636	15.82225	21.51613	15.66458	17.4484	46.4168	56.4674
AC106822.	0.960908	0	0	0	0	0	0	0	0	0	0	0.920078	4.112337	0.93072	0	0	0	0	0
AC106827.	0	0	0	0	0	0	0.84968	0	0	0	0	0	4.112337	0	0	0	0	0	0
AC106858.	36.51449	25.00976	69.93936	41.57171	63.12664	51.38492	36.53625	83.34243	86.37929	73.01314	84.33284	31.28266	35.98295	49.32818	28.36217	13.19123	18.47477	14.56213	11.652
AC106864.	12.4918	10.0039	16.98527	27.02161	17.71976	11.41887	33.13753	31.48492	2.334575	12.48909	2.40951	16.56141	3.084253	15.82225	23.47214	16.48904	11.29014	21.8432	25.99293
AC106864.	11.53089	14.00547	10.99047	16.62868	8.859879	7.993209	14.44456	5.556162	8.171014	11.52839	22.89034	2.760235	6.168506	13.9608	1.956012	9.06897	5.131881	2.7304	5.377848
AC106865.	262.3278	140.0547	278.7583	373.1061	339.9979	1189.846	213.2697	324.1095	283.6509	239.2141	106.0184	405.7545	261.1334	1882.847	2048.922	487.251	225.8028	1260.535	775.3064
AC106869.	161.4325	192.075	333.7106	148.6189	202.6697	382.5321	129.1514	303.7369	109.725	239.2141	238.5415	207.0176	240.5717	142.4002	208.3152	198.6929	197.0642	185.6672	190.9136
AC106870.	0	0	0	0	0	0	0	0	2.334575	0	2.40951	0.920078	0	0	0	0	0	0	0
AC106871.	0	4.001562	0	0	0	0	0	0	0	0	0	0	0	3.722881	0	0	0	0	0
AC106872.	16.33543	1.00039	4.995668	0	1.107485	6.851322	0.84968	8.334243	9.338302	4.803496	12.04755	5.520469	8.224675	7.445762	0.978006	0	5.131881	1.820267	3.585232
AC106872.	0	0	0	0	0	0	0.84968	0	0	0	0	1.840156	0	0	0	0	0	0	0
AC106872.	1.921815	5.001952	0	4.157171	11.07485	1.141887	0.84968	0.926027	15.17474	4.803496	3.614265	0.920078	1.028084	1.861441	2.934017	5.771163	6.158258	0.910133	2.688924
AC106872.	28.82723	23.00898	43.96188	15.58939	7.752394	11.41887	17.84329	6.482189	0	10.56769	1.204755	13.80117	21.58977	18.61441	8.802052	10.71787	5.131881	17.29253	23.30401
AC106872.	286.3504	152.0593	401.6517	248.391	394.2646	385.9578	322.0288	488.9423	287.1528	385.2404	390.3406	344.1093	303.2849	284.8004	271.8856	305.8716	349.9943	202.0496	174.7801
AC106872.	40.35812	0	0	0	0	1.141887	0	0	0	0	0	0	0	0	0	0.824452	2.052753	0.910133	0.896308
AC106872.	7.68726	5.001952	0	4.157171	8.859879	5.709435	0.84968	0	1.167288	0	3.614265	6.440548	1.028084	0	0.978006	0	1.026376	4.550667	3.585232
AC106872.	0	0	0	0	0	0	0	0	0	0	1.204755	0	0	0	0.978006	0	0	0	0
AC106872.	0	1.00039	12.98874	0	11.07485	6.851322	0.84968	0	0	0.960699	0	4.600391	1.028084	4.653602	3.912023	0.824452	1.026376	0	0
AC106873.	0	5.001952	1.998267	0	9.967364	1.141887	5.947762	0	0	2.882098	15.66181	0.920078	1.028084	18.61441	2.934017	0	1.026376	3.640534	1.792616
AC106875.	0	0	0	0	0	0	0	0	0	0	0	0.920078	4.112337	3.722881	0	0	0	0	0.896308
AC106875.	0	0	0	0	0	0	3.398721	0	0	0	0	0	0	0	0	0	0	0	0
AC106876.	0.960908	0	0	0	9.967364	0	0	0	0	0	0	0	1.028084	0.93072	0	0	0	5.4608	0.896308
AC106881.	206.5951	200.0781	182.8415	232.8016	153.9404	210.1072	254.9041	268.5478	224.1192	159.4761	154.2086	177.5751	199.4484	267.1167	236.6774	205.2885	237.0929	232.084	317.293
AC106882.	0	0	0	0	0	0	0	0	0	0.960699	0	0.920078	1.028084	0	1.956012	3.297807	0	0	0
AC106886.	0	0	0.999134	0	0	3.425661	0	0.926027	4.669151	3.842797	2.40951	0.920078	1.028084	0	0	0	0	0	0
AC106886.	11.53089	5.001952	2.997401	1.039293	5.537425	14.84453	22.94137	6.482189	67.70269	31.70308	12.04755	19.32164	20.56169	10.23792	9.780058	1.648904	20.52753	8.191201	4.48154
AC106886.	110.5044	105.041	62.94542	82.10413	129.5757	99.34417	89.21643	173.1671	273.1453	167.1617	192.7608	47.84407	77.10633	85.62627	81.17448	89.86525	104.6904	121.9579	103.9717
AC106886.	72.06806	166.0648	141.877	165.2476	106.3186	167.8574	79.86994	112.0493	324.506	112.4018	99.99465	112.2495	86.35909	108.8943	115.4047	140.1568	158.0619	88.28294	96.80126
AC106894.	2.882723	2.000781	1.998267	0	0	1.141887	0	0.926027	0	2.882098	0	0.920078	1.028084	0.93072	0	0	1.026376	2.7304	0
AC106895.	3.84363	0	0	0	0	0	0	9.26027	0	0	4.81902	0	0	0	0	0	0	0	0
AC106895.	21.13997	10.0039	37.96708	5.196464	27.68712	7.993209	25.49041	27.78081	2.334575	7.685594	14.45706	4.600391	12.33701	11.16864	18.58211	9.893422	2.052753	15.47227	20.61508
AC106897.	4.804538	18.00703	1.998267	0	1.107485	9.135096	9.346483	2.778081	3.501863	6.724895	3.614265	3.680313	7.196591	5.584322	2.934017	0.824452	14.36927	2.7304	8.963079
AC106900.	0.960908	0	2.997401	5.196464	0	0	1.69936	0.926027	0	3.842797	0	1.840156	1.028084	2.792161	0	0.824452	2.052753	0	2.688924
AC107015.	0	0	0.999134	0	0	0	0.84968	0	0	0	0	0	0	0.93072	0	0	0	0	0
AC107021.	1.921815	11.00429	29.97401	18.70727	23.25718	1.141887	16.9936	4.630135	2.334575	15.37119	2.40951	17.48149	33.92678	19.54513	20.53812	10.71787	11.29014	9.101334	6.274156
AC107021.	101.8562	85.03319	156.864	96.65423	90.81376	134.7427	130.8508	160.2027	95.71759	192.1399	159.0276	129.731	124.3982	85.62627	154.5249	53.58937	53.37157	31.85467	57.36371
AC107027.	17.29634	31.0121	23.97921	32.21808	68.66407	17.12831	32.28785	26.85478	37.35321	45.15286	13.2523	30.36258	4.112337	20.47585	19.56012	29.68027	25.65941	12.74187	22.4077
AC107027.	0.960908	10.0039	3.996535	7.275049	3.322455	1.141887	3.398721	6.482189	0	5.764196	6.023774	7.360626	3.084253	2.792161	4.890029	9.893422	10.26376	2.7304	10.7557
AC107027.	835.0286	781.3049	799.3069	1025.782	962.4044	838.1451	1040.009	789.9011	609.3242	619.651	543.3444	829.9106	905.7423	896.2837	830.3269	850.8343	771.8349	1037.552	1060.332
AC107029.	5.765445	12.00469	22.98007	13.51081	15.50479	11.41887	17.84329	32.41095	1.167288	3.842797	6.023774	11.96102	17.47743	12.09936	17.6041	10.71787	10.26376	13.652	23.30401

AC107029.	3.84363	1.00039	22.98007	3.117878	4.42994	1.141887	16.14392	3.704108	0	1.921399	12.04755	11.04094	3.084253	1.861441	11.73607	4.122259	4.105505	5.4608	8.963079
AC107031.	0	5.001952	1.998267	4.157171	0	0	1.69936	1.852054	11.67288	0	1.204755	5.520469	1.028084	0	3.912023	1.648904	0	6.370934	10.7557
AC107032.	767.7651	1028.401	686.4048	366.8703	626.8365	737.659	657.6525	545.4299	194.9371	454.4107	321.6696	792.1874	503.7613	848.8169	676.78	901.9503	464.9484	786.3553	377.3456
AC107032.	9.609075	11.00429	21.98094	6.235757	12.18233	11.41887	12.7452	12.03835	1.167288	5.764196	19.27608	13.80117	18.50552	23.26801	31.29619	12.36678	16.42202	10.01147	12.54831
AC107057.	3.84363	4.001562	0.999134	14.5501	0	3.425661	0.84968	9.26027	0	0.960699	7.228529	4.600391	7.196591	0.93072	5.868035	8.244518	6.158258	8.191201	3.585232
AC107067.	413.1902	221.0863	51.95495	129.9116	253.614	235.2287	190.3284	203.7259	274.3126	74.93454	25.29985	152.733	193.2799	331.3364	401.9604	180.555	170.3785	260.2982	259.033
AC107068.	1012.797	1088.425	1067.075	1310.548	764.1646	1028.84	1168.31	988.0708	817.1014	801.2232	1003.561	886.9554	1010.607	992.1478	873.3592	1062.718	1107.46	1033.001	998.4871
AC107068.	0.960908	0	2.997401	0	0	0	9.346483	12.03835	5.836439	1.921399	1.204755	0	0	0.93072	0	1.648904	3.079129	0.910133	4.48154
AC107072.	2.882723	9.003514	7.993069	3.117878	7.752394	2.283774	8.496802	8.334243	2.334575	6.724895	2.40951	12.8811	3.084253	2.792161	7.824047	1.648904	7.184634	9.101334	8.963079
AC107072.	15.37452	47.01835	30.97314	7.275049	7.752394	20.55397	28.88913	70.37805	17.50932	19.21399	21.68559	14.72125	20.56169	19.54513	24.45015	18.13794	22.58028	23.66347	34.0597
AC107074.	3.84363	9.003514	0.999134	5.196464	5.537425	1.141887	4.248401	1.852054	9.338302	5.764196	0	4.600391	10.28084	0.93072	1.956012	7.420066	7.184634	2.7304	1.792616
AC107075.	17.29634	22.00859	22.98007	7.275049	18.82724	27.40529	5.098081	13.89041	94.55031	17.29259	33.73314	4.600391	6.168506	21.40657	18.58211	14.01568	36.94955	3.640534	13.44462
AC107079.	7.68726	0	5.994802	7.275049	5.537425	0	2.549041	0	0	5.764196	0	1.840156	2.056169	0	0	6.595615	8.21101	1.820267	4.48154
AC107081.	20.17906	29.01132	2.997401	5.196464	11.07485	2.283774	16.14392	6.482189	28.01491	8.646293	12.04755	11.04094	12.33701	4.653602	8.802052	9.893422	7.184634	8.191201	9.859387
AC107081.	26.90541	71.02772	93.91856	55.08252	83.06137	38.82416	113.0075	42.59724	36.18592	67.24895	55.41872	64.40548	55.51656	54.9125	67.4824	55.23827	32.84404	48.23707	42.12647
AC107081.	3.84363	1.00039	7.993069	9.353635	0	0	5.947762	0.926027	14.00745	5.764196	19.27608	15.64133	5.140422	1.861441	4.890029	0	15.39564	0.910133	1.792616
AC107083.	3.84363	20.00781	12.98874	7.275049	22.1497	5.709435	16.9936	22.22465	0	14.41049	0	23.92203	26.73019	23.26801	28.36217	11.54233	5.131881	23.66347	26.88924
AC107214.	26.90541	14.00547	22.98007	29.1002	16.61227	27.40529	50.13113	22.22465	19.84389	17.29259	14.45706	25.76219	29.81445	7.445762	25.42815	26.38246	19.50115	13.652	11.652
AC107214.	90.32531	93.03631	53.95322	36.37525	46.51437	86.78341	41.63433	31.48492	81.71014	76.85594	85.5376	37.72321	27.75828	58.63538	70.41642	47.81821	62.60895	42.77627	37.64493
AC107220.	0	0	0	0	0	0	0	0	0	3.842797	0	0	0	0	0	0	3.079129	3.640534	0
AC107222.	14.41361	15.00586	49.95668	23.90373	15.50479	17.12831	33.13753	16.66849	51.36066	12.48909	56.62348	7.360626	4.112337	22.33729	25.42815	4.122259	10.26376	17.29253	30.47447
AC107241.	3.84363	6.002343	14.987	1.039293	4.42994	2.283774	0.84968	11.11232	1.167288	0	9.638039	0	1.028084	4.653602	7.824047	0.824452	1.026376	5.4608	5.377848
AC107294.	14.41361	17.00664	14.987	13.51081	26.57964	15.98642	9.346483	25.00273	79.37557	22.09608	59.03299	22.08188	19.5336	13.03008	14.67009	9.06897	29.76491	26.39387	8.963079
AC107294.	171.0415	148.0578	176.8467	148.6189	180.52	121.04	77.3209	93.52873	280.1491	164.2796	260.2271	196.8967	199.4484	117.2708	119.3167	127.79	161.1411	124.6883	178.3653
AC107294.	78.79442	97.03787	87.92376	66.51474	99.67364	49.10114	45.88273	60.19176	224.1192	123.9302	151.7991	94.76806	99.72418	67.94258	39.12023	81.62073	100.5849	100.1147	74.39356
AC107302.	9.609075	2.000781	0	7.275049	0	0	3.398721	3.704108	0	0	0	0	0	0	0	0	0	0	0
AC107308.	0.960908	0	0.999134	0	0	0	1.69936	0	0	8.646293	1.204755	3.680313	1.028084	0	0	0.824452	0	0	0
AC107373.	12.4918	16.00625	9.991337	13.51081	4.42994	7.993209	16.14392	7.408216	11.67288	1.921399	14.45706	5.520469	6.168506	20.47585	18.58211	24.73355	4.105505	12.74187	15.23724
AC107375.	1299.147	1974.771	2156.13	866.7702	1969.108	1474.176	1592.301	1471.457	1619.028	1892.578	2183.016	1502.488	1744.659	1899.6	2040.12	886.2857	1029.455	1362.47	1555.991
AC107393.	0	0	0	2.078586	0	0	0.84968	0	0	0	0	0	1.028084	0	0.978006	0	0	0	0
AC107396.	0	4.001562	0	0	0	6.851322	0.84968	0	0	0	7.228529	1.840156	0	0	2.934017	4.122259	0	0.910133	0
AC107398.	0	0	0	0	0	0	1.69936	0	0	0	2.40951	0.920078	1.028084	1.861441	0	0.824452	1.026376	0	0.896308
AC107398.	41.31902	44.01718	68.94022	60.27898	48.72934	34.25661	92.61515	36.11505	3.501863	31.70308	24.0951	59.80509	58.60081	36.29809	44.98827	24.73355	29.76491	35.4952	35.85232
AC107398.	110.5044	195.0761	375.6743	178.7584	122.9308	69.65511	104.5107	79.63833	43.18965	51.87776	53.00921	112.2495	131.5948	100.5178	140.8328	89.86525	50.29244	156.5429	287.7149
AC107398.	4.804538	10.0039	5.994802	9.353635	1.107485	4.567548	18.69297	9.26027	0	0	0	1.840156	7.196591	0.93072	5.868035	0	5.131881	21.8432	29.57816
AC107419.	6.726353	1.00039	6.993936	4.157171	1.107485	11.41887	13.59488	0	0	0	0	5.520469	7.196591	1.861441	0	1.648904	0	3.640534	0.896308
AC107421.	0	0	3.996535	0	0	0	0	0	0	0	0	0	0	0	0	0	0	0	0
AC107464.	11.53089	53.02069	4.995668	16.62868	5.537425	19.41208	20.39233	19.44657	67.70269	38.42797	62.64725	8.280704	21.58977	27.92161	21.51613	5.771163	25.65941	18.20267	38.54124
AC107464.	7.68726	1.00039	0.999134	0	1.107485	4.567548	2.549041	0.926027	2.334575	6.724895	21.68559	1.840156	2.056169	0	0	0.824452	2.052753	6.370934	4.48154
AC107464.	49.00628	107.0418	66.94196	73.78979	65.34161	75.36454	86.66738	48.15341	185.5987	102.7948	225.2892	63.4854	80.19058	63.28898	83.1305	68.4295	54.39794	102.8451	84.25295
AC107626.	0	0	0	3.117878	0	0	0	0	0	0	0	0	0	0	0	0	0	0	0
AC107871.	353.614	399.1558	528.5417	494.7034	493.9383	347.1336	672.9468	568.5806	158.7511	369.8692	265.0461	597.1308	516.0984	372.2881	423.4765	537.5426	431.078	668.9481	458.9097
AC107871.	12.4918	39.01523	22.98007	1.039293	27.68712	7.993209	45.88273	19.44657	96.88488	45.15286	24.0951	28.52243	34.95487	8.376483	8.802052	4.946711	17.4484	36.40534	31.37078
AC107882.	0	0	0	0	0	0	0	0	0	0	0	0	1.028084	0	0.978006	0	0	0	0
AC107884.	955.1421	910.3553	1076.067	1416.556	650.0936	759.3549	1146.219	847.3147	766.908	519.7383	418.0499	1331.353	1483.526	903.7294	868.4692	751.0756	612.7466	779.9843	909.7526
AC107884.	0	0	0	0	0	0	1.69936	0	0	8.646293	4.81902	0.920078	0	0	0	0	0	0.910133	3.585232
AC107886.	5.765445	11.00429	6.993936	20.78586	12.18233	31.97284	22.94137	0.926027	2.334575	13.44979	33.73314	8.280704	7.196591	12.09936	13.69208	16.48904	25.65941	10.01147	26.88924
AC107892.	8.648168	25.00976	22.98007	41.57171	17.71976	94.77662	18.69297	39.81916	18.6766	36.50657	20.48083	8.280704	9.252759	30.71377	22.49413	18.13794	21.5539	9.101334	8.066772
AC107896.	0	1.00039	0	0	0	1.141887	0.84968	0	0	0	0	4.600391	0	0.93072	1.956012	3.297807	0	0	2.688924
AC107905.	0	0	0	1.039293	0	2.283774	0.84968	0	0	0	0	0	0	0	0.978006	0	1.026376	0.910133	0
AC107909.	7.68726	0	1.998267	1.039293	0	1.141887	2.549041	5.556162	0	4.803496	14.45706	0.920078	1.028084	0.93072	0	4.122259	1.026376	0	0
AC107909.	16.33543	7.002733	15.98614	4.157171	0	2.283774	5.098081	1.852054	14.00745	10.56769	16.86657	6.440548	5.140422	5.584322	7.824047	15.66458	3.079129	6.370934	12.54831
AC107918.	0	0	0	0	1.107485	0	1.69936	0	0	0	0	0.920078	0	0.93072	0	0	0	0.910133	0
AC107918.	0	2.000781	0.999134	3.117878	8.859879	2.283774	2.549041	0	0	0.960699	0	1.840156	1.028084	0	0.978006	0.824452	1.026376	0	0
AC107918.	477.571	484.189	405.6483	1045.529	344.4278	347.1336	319.4798	421.3423	289.4874	397.7295	333.7171	335.8286	353.661	367.6345	356.9721	690.0662	671.2501	305.8048	405.1312
AC107926.	2.882723	0	3.996535	0	0	0	2.549041	0	2.334575	0	1.204755	5.520469	0	0.93072	0.978006	0	1.026376	4.550667	0.896308
AC107952.	219.0869	321.1253	235.7955	216.1729	282.4087	393.951	181.8316	223.1725	598.8186	437.1182	354.1979	108.5692	174.7743	201.0356	204.4032	179.7305	208.3544	152.9024	154.165
AC107953.	0	0	0	12.47151	0	0	0	0.926027	0	0	0	0	0	0	5.868035	0.824452	4.105505	2.7304	11.652
AC107954.	4.804538	0	0.999134	3.117878	1.107485	4.567548	3.398721	12.96438	0	0.960699	0	0	0	0.93072	0	1.648904	3.079129	3.640534	11.652
AC107956.	12.4918	7.002733	10.99047	11.43222	1.107485	4.567548	8.496802	0.926027	5.836439	1.921399	12.04755	6.440548	6.168506	13.03008	11.73607	1.648904	4.105505	11.83173	1.792616
AC107956.	0	0	0.999134	0	4.42994	0	0	0	0	0	0	0	0	0	0	0	0	0.910133	0
AC107959.	179.6897	98.03826	79.93069	163.169	180.52	70.79699	144.4456	98.15887	187.9333	79.73804	151.7991	114.0897	160.3812	102.3792	133.0088	122.0189	90.32111	131.9693	147.8908

AC107959.	0.960908	0	0	4.157171	0	0	0	0	0	3.842797	0	0.920078	1.028084	3.722881	0	3.297807	2.052753	0.910133	3.585232
AC107959.	0	0	0.999134	2.078586	0	0	0.84968	0	0	0	0	0	1.028084	0.93072	0	0.824452	1.026376	8.191201	2.688924
AC107973.	1.921815	4.001562	3.996535	0	1.107485	0	3.398721	1.852054	0	0	0	0	0	0.93072	7.824047	0	1.026376	2.7304	0
AC107977.	0	0	0	0	0	0	0	0	0	0	0	0	4.112337	0	0	0	0	0	0
AC107980.	0	1.00039	0.999134	1.039293	0	1.141887	0	0	0	5.764196	0	0	0	0.93072	0	0	1.026376	0	0.896308
AC107982.	1.921815	3.001171	0	0	1.107485	0	0	6.482189	4.669151	0	0	0	2.056169	0	0	0.824452	2.052753	0	1.792616
AC107982.	9.609075	2.000781	0.999134	8.314342	2.21497	6.851322	12.7452	2.778081	12.84017	5.764196	9.638039	8.280704	5.140422	1.861441	1.956012	1.648904	3.079129	4.550667	3.585232
AC107982.	98.97348	105.041	81.92896	113.2829	108.5335	161.0061	102.8113	97.23284	158.7511	168.1224	125.2945	96.60821	94.58376	83.76483	101.7126	111.301	127.2707	70.08027	97.69757
AC107983.	5.765445	2.000781	1.998267	3.117878	6.64491	6.851322	6.797442	0.926027	24.51304	16.33189	46.98544	1.840156	0	5.584322	7.824047	1.648904	0	12.74187	8.963079
AC107983.	235.4223	8.003124	804.3026	733.7407	1048.788	744.5103	1246.481	1171.424	413.2199	779.1271	408.4119	549.2867	456.4695	728.754	904.6554	6.595615	9.237386	378.6155	362.1084
AC107993.	7.68726	15.00586	11.9896	7.275049	57.58922	11.41887	7.647122	37.04108	89.88116	23.05678	33.73314	17.48149	9.252759	32.57521	18.58211	9.893422	3.079129	4.550667	0.896308
AC108002.	691.8534	643.2511	642.4429	1201.422	460.7137	340.2823	1231.187	502.8327	190.2679	178.6901	114.4517	633.0138	611.7102	644.9892	602.4516	568.8718	568.6124	638.9137	1006.554
AC108002.	0.960908	0	0.999134	0	0	2.283774	4.248401	0	0	2.882098	0	2.760235	0	1.861441	0	0	3.079129	0.910133	3.585232
AC108010.	240.2269	185.0722	277.7592	330.4951	198.2398	139.3102	342.4211	260.2136	75.8737	199.8254	154.2086	530.8851	441.0482	244.7794	241.5674	328.9563	361.2844	390.4472	346.8712
AC108022.	0	1.00039	0.999134	1.039293	0	1.141887	0	0	0	0	0	0.920078	4.112337	0.93072	0	0	0	0.910133	0
AC108024.	0	0	0.999134	0	2.21497	0	0	0	0	0	0	1.840156	2.056169	0	0	0	0	0	0
AC108025.	23.06178	28.01093	23.97921	27.02161	25.47215	38.82416	31.43817	20.37259	4.669151	20.17468	9.638039	33.12282	41.12337	31.64449	11.73607	40.39814	40.02867	21.8432	43.02278
AC108025.	8.648168	12.00469	10.99047	22.86444	1.107485	13.70264	16.14392	2.778081	0	1.921399	1.204755	7.360626	6.168506	7.445762	7.824047	7.420066	17.4484	2.7304	4.48154
AC108032.	0	1.00039	1.998267	0	2.21497	1.141887	0	6.482189	0	0.960699	10.84279	1.840156	0	0.93072	0	0	1.026376	2.7304	0
AC108039.	0	3.001171	10.99047	4.157171	0	2.283774	8.496802	5.556162	4.669151	0.960699	15.66181	0.920078	2.056169	1.861441	0	2.473355	1.026376	1.820267	0.896308
AC108039.	0	1.00039	6.993936	8.314342	0	0	5.098081	0	9.338302	0.960699	1.204755	1.840156	7.196591	7.445762	1.956012	0	4.105505	0.910133	0
AC108047.	25.9445	27.01054	19.98267	30.13949	5.537425	20.55397	43.33369	30.55889	10.50559	36.50657	10.84279	29.4425	27.75828	13.03008	38.14223	24.73355	36.94955	47.32694	38.54124
AC108051.	0	0	0	0	0	0	0.84968	0.926027	5.836439	0	0	0	1.028084	0	0.978006	0	0	0.910133	0
AC108058.	90.32531	53.02069	124.8917	136.1474	68.66407	99.34417	104.5107	60.19176	32.68406	44.19217	56.62348	72.68618	79.1625	71.66546	69.43841	133.5612	108.7959	46.4168	45.71171
AC108059.	0	0	0	0	0	0	0	0	0	2.882098	0	0	0	0	0	0	0	0	0
AC108059.	0.960908	0	0	0	0	0	0	0	0	0	1.204755	0	0	0	0	0.824452	0	1.820267	0.896308
AC108062.	313.2559	251.098	283.754	196.4263	274.6563	262.634	293.1397	584.3231	764.5735	498.6029	448.1688	346.8695	376.2789	379.7339	416.6305	253.1067	240.172	356.7723	233.9364
AC108063.	8.648168	14.00547	38.96621	8.314342	14.3973	12.56076	25.49041	48.15341	5.836439	10.56769	22.89034	29.4425	12.33701	18.61441	19.56012	11.54233	3.079129	10.9216	18.82247
AC108066.	0.960908	0	2.997401	0	1.107485	1.141887	0	0.926027	0	0	0	0.920078	0	0.93072	0	4.122259	0	0.910133	0
AC108067.	2.882723	0	0.999134	0	0	0	0.84968	0	0	0	0	1.840156	0	1.861441	0	0	0	0	0
AC108073.	9.609075	3.001171	1.998267	1.039293	0	6.851322	0.84968	7.408216	1.167288	0	1.204755	0	0	0.93072	0	0.824452	1.026376	0	3.585232
AC108097.	0	2.000781	1.998267	4.157171	1.107485	4.567548	8.496802	2.778081	15.17474	6.724895	0	2.760235	7.196591	1.861441	8.802052	3.297807	2.052753	0.910133	4.48154
AC108102.	0	2.000781	9.991337	1.039293	0	0	0.84968	4.630135	0	0.960699	0	0.920078	1.028084	0	3.912023	1.648904	0	3.640534	0.896308
AC108120.	0	0	0	4.157171	0	1.141887	0	0	0	0	7.228529	0	0	0	0	0	0	0.910133	0
AC108120.	0	0	0	4.157171	0	0	0	0	0	0	0	0	1.028084	1.861441	0	0.824452	0	5.4608	0
AC108125.	0	0	0	0	0	0	0	0	0	0	0	0	0	3.722881	0	0	0	0	0
AC108134.	37.47539	21.0082	29.97401	7.275049	39.86946	45.67548	45.03305	31.48492	123.7325	135.4586	255.408	23.00196	17.47743	48.39746	51.83431	6.595615	23.60665	10.01147	40.33386
AC108134.	97.05166	100.039	63.94455	93.53635	44.2994	50.24303	100.2623	35.18903	57.1971	63.40615	72.28529	42.3236	53.46039	92.14131	70.41642	73.37621	55.42432	66.43974	56.4674
AC108134.	413.1902	267.1042	212.8155	373.1061	260.259	369.9714	234.5117	198.1698	667.6886	565.8519	652.9771	375.3919	389.644	295.9691	316.8739	337.2008	275.0688	573.3841	357.6269
AC108134.	26.90541	21.0082	29.97401	10.39293	32.11706	39.96605	13.59488	23.15068	40.85507	23.05678	24.0951	45.08383	68.88165	46.53602	21.51613	16.48904	41.05505	36.40534	32.26709
AC108136.	0	4.001562	0.999134	2.078586	0	0	0.84968	0.926027	5.836439	0	3.614265	0.920078	2.056169	10.23792	9.780058	54.41382	25.65941	29.12427	16.13354
AC108145.	0	0	0	0	0	0	0	1.852054	0	0	0	2.760235	0	0	0	0	0	0	0
AC108156.	21.13997	23.00898	17.98441	38.45383	2.21497	12.56076	33.98721	14.81643	12.84017	7.685594	1.204755	18.40156	13.3651	34.43665	28.36217	18.13794	13.34289	13.652	38.54124
AC108157.	0	0	7.993069	1.039293	13.28982	5.709435	1.69936	0.926027	0	0	0	0.920078	0	0	0.978006	0	0	0.910133	0.896308
AC108159.	0.960908	1.00039	4.995668	1.039293	5.537425	1.141887	6.797442	4.630135	8.171014	15.37119	0	6.440548	2.056169	0	1.956012	0	2.052753	4.550667	3.585232
AC108161.	14.41361	7.002733	1.998267	0	14.3973	12.56076	19.54265	12.96438	1.167288	5.764196	9.638039	0.920078	7.196591	11.16864	8.802052	9.06897	10.26376	5.4608	5.377848
AC108174.	6.726353	2.000781	7.993069	1.039293	0	13.70264	12.7452	0.926027	0	1.921399	0	7.360626	10.28084	0	0	0.824452	0	1.820267	4.48154
AC108199.	0	0	11.9896	0	0	0	0	0	0	0	0	0	0	0	0	0	2.052753	0	0
AC108206.	0	0	0	0	0	0	0	0.926027	0	0	0	0	1.028084	0	0	0	0	13.652	0.896308
AC108210.	26.90541	7.002733	25.97748	1.039293	24.36467	6.851322	4.248401	5.556162	0	28.82098	27.70936	26.68227	22.61786	5.584322	12.71408	4.946711	1.026376	12.74187	17.02985
AC108210.	0	0	0	0	0	0	0	0	0	0	0	1.840156	0	0	0	0	0	2.7304	0
AC108215.	11.53089	18.00703	17.98441	1.039293	9.967364	4.567548	16.9936	25.92876	4.669151	4.803496	20.48083	8.280704	14.39318	17.68369	10.75806	10.71787	10.26376	14.56213	12.54831
AC108448.	70.14625	12.00469	12.98874	0	14.3973	2.283774	1.69936	0	28.01491	23.05678	7.228529	34.04289	38.03912	12.09936	8.802052	14.01568	21.5539	0	0
AC108448.	3.84363	12.00469	13.98787	41.57171	55.37425	59.37812	5.947762	49.07943	60.69896	59.56335	60.23774	30.36258	19.5336	24.19873	26.40616	74.20066	45.16056	6.370934	17.92616
AC108449.	8.648168	9.003514	9.991337	25.98232	1.107485	4.567548	13.59488	6.482189	5.836439	7.685594	16.86657	15.64133	6.168506	3.722881	11.73607	20.6113	20.52753	10.01147	12.54831
AC108449.	884.9958	777.3034	1044.095	1648.318	872.6981	573.2273	1466.548	965.8462	151.7474	343.9303	173.4847	1338.714	1251.179	893.4915	1038.642	1333.139	1204.966	921.055	1020.895
AC108451.	93.20803	75.02928	58.94889	14.5501	33.22455	21.69585	22.09169	102.789	14.00745	43.23147	38.55216	22.08188	14.39318	90.27987	70.41642	22.2602	13.34289	13.652	12.54831
AC108451.	21.13997	10.0039	8.992203	5.196464	7.752394	1.141887	13.59488	6.482189	9.338302	12.48909	8.433284	15.64133	14.39318	5.584322	8.802052	15.66458	13.34289	15.47227	10.7557
AC108462.	0	1.00039	0	0	6.64491	0	0	0	0	0	1.204755	0	0	3.722881	0	4.946711	0	3.640534	4.48154
AC108463.	32.67086	52.0203	18.98354	75.86837	36.547	30.83095	37.38593	40.74519	59.53167	27.86028	25.29985	56.12477	29.81445	45.60529	34.2302	65.95615	52.34519	121.0477	53.77848
AC108463.	4.804538	31.0121	13.98787	22.86444	25.47215	11.41887	16.14392	30.55889	32.68406	33.62447	3.614265	40.48344	30.84253	40.95169	14.67009	42.04704	32.84404	45.50667	39.43755
AC108463.	45.16265	25.00976	37.96708	20.78586	19.93473	1.141887	32.28785	26.85478	5.836439	7.685594	16.86657	66.24563	47.29188	30.71377	37.16422	76.67402	41.05505	61.88907	77.97879

AC108471.	3.84363	4.001562	0	1.039293	2.21497	3.425661	1.69936	0	26.84762	0	1.204755	6.440548	4.112337	0.93072	0.978006	0	8.21101	0.910133	7.170464
AC108471.	260.4059	343.1339	444.6145	305.5521	317.8482	307.1676	399.3497	359.2985	375.8666	304.5417	256.6128	302.7057	422.5427	287.5926	353.0601	253.1067	368.4691	338.5696	393.4792
AC108472.	7.68726	16.00625	2.997401	9.353635	16.61227	1.141887	37.38593	21.29862	24.51304	1.921399	24.0951	14.72125	24.67402	14.89152	19.56012	3.297807	8.21101	6.370934	10.7557
AC108474.	38.4363	6.002343	43.96188	22.86444	16.61227	27.40529	32.28785	37.96711	136.5727	19.21399	49.39495	23.92203	21.58977	50.2589	64.54838	28.85581	24.63303	37.31547	25.99293
AC108475.	88.40349	73.0285	113.9012	83.14342	181.6275	44.53359	84.96802	120.3835	73.53913	76.85594	91.56137	130.6511	113.0893	135.8852	126.1628	75.02512	87.24198	97.38428	99.49018
AC108477.	13.45271	18.00703	9.991337	15.58939	25.47215	6.851322	22.09169	17.59451	9.338302	16.33189	14.45706	38.64329	37.01104	45.60529	21.51613	10.71787	8.21101	9.101334	0.896308
AC108479.	1.921815	3.001171	1.998267	2.078586	4.42994	0	0	3.704108	1.167288	3.842797	3.614265	5.520469	2.056169	0	0	2.473355	4.105505	0	0.896308
AC108479.	15.37452	0	0	1.039293	0	1.141887	10.19616	4.630135	0	0	6.023774	0.920078	0	0.93072	0	0	1.026376	1.820267	1.792616
AC108482.	5.765445	1.00039	0	1.039293	3.322455	2.283774	4.248401	0	3.501863	0	0	0.920078	1.028084	0.93072	0	0	2.052753	0.910133	8.066772
AC108488.	1011.836	848.3311	1091.054	614.222	1158.429	975.1715	678.0448	1037.15	1072.737	1378.603	1452.934	876.8346	1030.141	787.3894	809.7888	582.063	572.7179	899.2118	856.8704
AC108515.	0	0	0	3.117878	0	1.141887	0.84968	0	0	0	0	0	4.112337	0	1.956012	2.473355	1.026376	0	0
AC108516.	0.960908	2.000781	0	4.157171	0	1.141887	0	0	4.669151	7.685594	0	2.760235	3.084253	3.722881	4.890029	1.648904	1.026376	0	0
AC108516.	3.84363	3.001171	3.996535	3.117878	1.107485	2.283774	10.19616	3.704108	0	2.882098	0	5.520469	1.028084	4.653602	3.912023	1.648904	0	3.640534	7.170464
AC108667.	0	0	0.999134	0	0	0	1.69936	1.852054	0	0	0	0.920078	2.056169	0	0	0	1.026376	0	0
AC108673.	34.59267	32.01249	45.96015	34.29666	52.05179	39.96605	43.33369	53.70957	36.18592	37.46727	62.64725	26.68227	53.46039	50.2589	38.14223	52.76492	70.81996	45.50667	60.94894
AC108673.	167.1979	410.1601	273.7626	186.0334	384.2973	417.9306	170.7857	209.2821	677.0269	490.9173	721.6482	186.7759	192.2518	215.9271	194.6232	179.7305	226.8292	177.476	192.7062
AC108676.	83.59896	114.0445	88.9229	82.10413	134.0057	85.64153	130.0011	87.04654	25.68033	73.01314	60.23774	116.8499	83.27483	105.1714	96.82258	81.62073	91.34749	105.5755	99.49018
AC108681.	3.84363	0	0	0	0	0	0	0	0	0	0	0	4.112337	0	0	0.824452	0	0	4.48154
AC108688.	0	0	0	0	0	0	0	0.926027	0	0.960699	0	0	0	1.861441	0	0.824452	0	0	0
AC108693.	0	1.00039	0	4.157171	0	1.141887	6.797442	2.778081	10.50559	0.960699	1.204755	2.760235	5.140422	3.722881	0.978006	4.946711	7.184634	4.550667	7.170464
AC108693.	27.86632	21.0082	24.97834	59.23969	21.04221	21.69585	49.28145	50.93149	0	9.606993	1.204755	26.68227	16.44935	40.95169	35.20821	30.50472	23.60665	25.48374	41.23017
AC108704.	9.609075	18.00703	6.993936	13.51081	3.322455	4.567548	13.59488	24.0767	25.68033	27.86028	26.50461	16.56141	9.252759	25.12945	11.73607	14.01568	25.65941	15.47227	8.963079
AC108704.	39.39721	87.03397	105.9082	150.6975	78.63143	82.21586	98.56291	62.04381	0	45.15286	19.27608	115.9299	99.72418	65.15042	92.91055	138.5079	117.0069	91.92348	111.1422
AC108706.	0.960908	0	0	0	0	0	0	0	0	0	0	0	1.028084	0	0	0	0	0	0
AC108718.	14.41361	4.001562	0.999134	13.51081	4.42994	3.425661	6.797442	2.778081	0	0	0	10.12086	6.168506	3.722881	0	1.648904	3.079129	12.74187	11.652
AC108721.	0	0	0	0	0	0	1.141887	4.248401	0	0	0.960699	0	0.920078	0	0	0	4.946711	0	0.910133	0.896308
AC108724.	0	4.001562	2.997401	2.078586	1.107485	2.283774	1.69936	1.852054	12.84017	1.921399	2.40951	0.920078	0	1.861441	0	3.297807	0	0	5.377848
AC108727.	28.82723	59.02304	124.8917	41.57171	79.73891	47.95925	114.7068	119.4575	14.00745	53.79916	60.23774	79.12673	62.71315	67.94258	87.04252	36.27588	31.81766	52.78774	50.19325
AC108729.	0	2.000781	1.998267	8.314342	0	0	0.84968	1.852054	0	0	0	0	0	0.93072	0.978006	0.824452	0	0	1.792616
AC108729.	14.41361	23.00898	11.9896	8.314342	14.3973	6.851322	13.59488	7.408216	42.02236	17.29259	16.86657	5.520469	7.196591	5.584322	8.802052	4.946711	10.26376	10.9216	7.170464
AC108734.	0.960908	0	4.995668	1.039293	1.107485	0	3.398721	0	3.501863	0.960699	7.228529	0.920078	8.224675	5.584322	2.934017	2.473355	14.36927	0	4.48154
AC108738.	0	0	0	0	0	0	0	0	0	0.960699	0	0	0	4.653602	0	0	0	0	0
AC108748.	0	0	0	1.039293	0	0	1.69936	0	0	0	0	0.920078	1.028084	0	0	1.648904	0	0	0
AC108750.	4.804538	0	8.992203	12.47151	22.1497	0	5.098081	0	4.669151	12.48909	1.204755	11.96102	7.196591	4.653602	6.846041	1.648904	4.105505	0.910133	0.896308
AC108751.	0	0	0	0	1.107485	0	0	0	0	0	0	0.920078	0	0	0	0	0	0	0
AC108751.	1.921815	4.001562	0	0	0	0	1.69936	5.556162	2.334575	1.921399	4.81902	1.840156	3.084253	5.584322	1.956012	0.824452	1.026376	7.281067	5.377848
AC108751.	0	0	0	0	0	0	0.84968	0	0	0	0	7.360626	4.112337	0	0	0	0	0	0
AC108860.	0	0	0	2.078586	0	0	0	0	0	0	0	0	0	0.93072	0	0	0	0	0
AC108861.	8.648168	4.001562	19.98267	2.078586	3.322455	2.283774	15.29424	8.334243	0	5.764196	4.81902	9.200782	9.252759	9.307203	5.868035	0.824452	1.026376	1.820267	0.896308
AC108865.	52.84991	13.00508	18.98354	1.039293	1.107485	17.12831	2.549041	16.66849	18.6766	29.78168	31.32363	50.6043	46.2638	60.49682	49.8783	19.78684	29.76491	23.66347	21.51139
AC108865.	28.82723	0	2.997401	6.235757	2.21497	4.567548	1.69936	12.96438	1.167288	5.764196	6.023774	11.04094	12.33701	13.9608	23.47214	1.648904	1.026376	2.7304	3.585232
AC108866.	69.18534	91.03553	87.92376	35.33595	75.30897	42.24982	84.11834	46.30135	105.0559	70.13105	44.57593	94.76806	70.93782	59.5661	82.15249	34.62698	23.60665	54.608	86.04556
AC108938.	0	0	2.997401	0	6.64491	4.567548	0	0	0	0	0	0.920078	2.056169	0.93072	0.978006	0.824452	5.131881	1.820267	1.792616
AC108941.	3.84363	1.00039	0	0	5.537425	0	1.69936	0	0	0.960699	1.204755	0.920078	0	0	0	1.648904	2.052753	1.820267	2.688924
AC109309.	4796.85	4709.838	2860.52	5302.472	1939.206	5808.779	4163.433	3155.9	5009.999	4231.88	5985.222	5014.426	4851.53	5588.975	4768.756	5663.984	6568.808	3534.958	6266.089
AC109309.	0	1.00039	0	1.039293	0	0	0.84968	0	0	1.921399	0	0	1.028084	0	0	0	0	0	0.896308
AC109315.	0	0	0	0	0	0	0	0	0	1.921399	0	0	0	0	0	0.824452	0	0	0
AC109322.	77.83351	85.03319	122.8934	66.51474	115.1784	110.763	47.58209	64.82189	249.7996	117.2053	220.4701	86.48735	111.0331	101.4485	83.1305	66.7806	113.9278	96.47414	105.7643
AC109326.	98.97348	103.0402	41.96361	146.5403	56.48173	62.80379	98.56291	58.3397	31.51677	152.7512	156.6181	102.1287	94.58376	59.5661	28.36217	74.20066	61.58258	131.9693	157.7502
AC109327.	0	4.001562	0	0	0	2.283774	0.84968	0	1.167288	0.960699	0	0	1.028084	0.93072	0.978006	0.824452	0	0	0
AC109327.	5.765445	1.00039	0	0	0	0	1.69936	3.704108	0	1.921399	0	9.200782	1.028084	0	0.978006	0.824452	0	0	0
AC109329.	0	4.001562	2.997401	0	0	0	0	0	0	0	0	0	0	0	0	0	0	0	1.792616
AC109329.	0	12.00469	0	0	0	6.851322	5.098081	5.556162	23.34575	5.764196	0	1.840156	12.33701	2.792161	2.934017	0	2.052753	0	0
AC109347.	322.8649	467.1823	445.6136	413.6385	351.0727	326.5797	443.5331	469.4957	261.4725	440.961	406.0024	423.236	401.981	457.9144	374.5762	391.6146	367.4427	496.0227	461.5986
AC109347.	8.648168	3.001171	0.999134	14.5501	46.51437	21.69585	4.248401	4.630135	15.17474	23.05678	9.638039	7.360626	9.252759	3.722881	4.890029	25.55801	5.131881	22.75334	5.377848
AC109361.	14.41361	18.00703	18.98354	21.82515	28.79461	10.27698	19.54265	20.37259	21.01118	26.89958	18.07132	6.440548	2.056169	7.445762	12.71408	2.473355	1.026376	17.29253	0.896308
AC109361.	0.960908	2.000781	7.993069	2.078586	0	5.709435	0	0	10.50559	0	0	0	4.112337	0.93072	1.956012	0	0	3.640534	0
AC109446.	0.960908	0	1.998267	0	0	1.141887	5.098081	0	0	0	0	0	0	0	0	0	0	0	0
AC109446.	2.882723	8.003124	1.998267	4.157171	3.322455	1.141887	2.549041	3.704108	23.34575	1.921399	0	0	1.028084	0.93072	2.934017	0	5.131881	0	0
AC109446.	0	9.003514	9.991337	0	17.71976	0	3.398721	9.26027	1.167288	0.960699	0	6.440548	1.028084	3.722881	0	0	2.052753	1.820267	4.48154
AC109449.	52.84991	65.02538	41.96361	39.49313	48.72934	43.39171	39.08529	72.23011	49.02608	29.78168	43.37118	61.64524	54.48847	56.77394	55.74633	65.13169	44.13418	50.96747	56.4674
AC109454.	15.37452	1.00039	9.991337	3.117878	7.752394	13.70264	8.496802	0.926027	11.67288	8.646293	7.228529	1.840156	9.252759	11.16864	12.71408	3.297807	7.184634	9.101334	5.377848

AC109454.	132.6052	141.0551	133.8839	249.4303	111.856	154.1547	203.0736	148.1643	105.0559	83.58083	107.2232	139.8519	117.2016	205.6892	189.7331	150.0502	138.5608	144.7112	172.0911
AC109454.	0.960908	6.002343	0.999134	11.43222	1.107485	3.425661	0	0	14.00745	9.606993	9.638039	5.520469	1.028084	2.792161	0	2.473355	0	0.910133	3.585232
AC109454.	58.61536	85.03319	60.94715	41.57171	37.65449	39.96605	38.23561	71.30408	31.51677	40.34937	60.23774	36.80313	62.71315	56.77394	65.52639	37.92478	40.02867	48.23707	44.8154
AC109458.	0.960908	0	0	0	0	0	0.84968	0	1.167288	0	0	0	0	0	0	0	0	0	0
AC109460.	128.7616	113.0441	126.89	85.22201	114.0709	107.3374	108.7591	134.2739	149.4128	157.5547	128.9088	139.8519	120.2859	84.69555	128.1188	98.93422	145.7454	127.4187	123.6905
AC109460.	618.8244	667.2604	822.287	821.0413	685.5332	616.619	723.9276	829.7202	266.1416	674.4109	390.3406	850.1523	765.9229	673.8415	791.2067	638.1257	645.5907	721.7358	574.5334
AC109460.	157.5888	166.0648	204.8224	95.61493	222.6045	212.391	127.452	162.0547	402.7143	332.4019	459.0116	237.3802	197.3922	137.7466	172.129	167.3637	214.5126	190.2179	211.5287
AC109462.	0	0	0	3.117878	0	0	0	0	0	0	0	0	0	0	0	0	0	0	0
AC109462.	0.960908	0	0	2.078586	0	9.135096	0	0	0	0	0	0	0	7.445762	0	0.824452	0	0	0
AC109462.	5.765445	0	0	4.157171	0	0	0	0	0	4.803496	10.84279	0.920078	0	0	4.890029	0	0	0	0
AC109462.	3.84363	0	0	4.157171	4.42994	4.567548	0	3.704108	0	0	1.204755	0	0	0	3.912023	0	2.052753	0.910133	5.377848
AC109464.	0	0	0	0	0	0	0	0	0	0	0	0	0	0	0	0	0	0	3.585232
AC109466.	23.06178	22.00859	10.99047	57.1611	7.752394	13.70264	78.17058	7.408216	11.67288	2.882098	9.638039	19.32164	6.168506	32.57521	29.34017	41.22259	26.68578	31.85467	63.63786
AC109471.	0	0	0	0	0	0	0	3.704108	7.003726	0	0	0	0	0	0	0	0	0	0
AC109471.	8.648168	0	0	0	0	1.141887	3.398721	0	0	1.921399	0	0	0	0	0	0	0	0	0
AC109479.	3.84363	0	0	2.078586	0	1.141887	0	0	0	0	0	0.920078	0	1.861441	0	0.824452	2.052753	0	1.792616
AC109479.	0	0	0	0	0	0	0	0	0	0	0	0	0	0	2.934017	0	0	0	0
AC109481.	0	0	0.999134	0	0	0	0	3.704108	0	0.960699	1.204755	3.680313	1.028084	0.93072	0	0	4.105505	0.910133	0
AC109486.	0	0	0	0	0	1.141887	0	0	0	0	0	0	0	0	0	0	0	0	3.585232
AC109492.	0	0	0	0	0	0	0	0	0	0	0	0.920078	3.084253	0	0	4.946711	0	0	0
AC109583.	20.17906	15.00586	12.98874	37.41454	18.82724	18.27019	22.09169	9.26027	15.17474	7.685594	14.45706	23.00196	11.30893	5.584322	25.42815	23.9091	19.50115	13.652	13.44462
AC109583.	11.53089	4.001562	0.999134	0	8.859879	6.851322	7.647122	16.66849	0	1.921399	0	6.440548	15.42127	10.23792	4.890029	0	2.052753	2.7304	0
AC109587.	219.0869	239.0933	316.7254	348.1631	242.5392	285.4718	257.4531	335.2218	172.7586	204.6289	168.6657	272.3432	281.6951	222.4422	291.4457	281.1381	211.4335	287.6022	239.3142
AC109597.	0	1.00039	2.997401	0	1.107485	5.709435	3.398721	4.630135	0	0	0	0.920078	0	0	0.978006	4.122259	0	0.910133	0.896308
AC109597.	13.45271	10.0039	8.992203	3.117878	1.107485	2.283774	8.496802	0	26.84762	7.685594	20.48083	4.600391	2.056169	6.515042	0	2.473355	6.158258	8.191201	8.066772
AC109630.	5.765445	0	3.996535	0	0	0	0	0	0	0	0	0	1.028084	0.93072	0	10.71787	3.079129	8.191201	15.23724
AC109631.	17.29634	27.01054	11.9896	32.21808	7.752394	19.41208	49.28145	9.26027	0	11.52839	0	28.52243	39.06721	22.33729	28.36217	9.06897	6.158258	17.29253	27.78555
AC109635.	0	0	0	0	4.42994	4.567548	1.69936	0	0	0	0	0	0	0	0	0	0	0	0
AC109635.	0	0	3.996535	0	0	0	3.398721	0	0	0	0	0	0	0	0	0	0	0	0
AC109635.	23.06178	35.01367	23.97921	28.0609	22.1497	47.95925	59.47762	28.70684	3.501863	25.93888	26.50461	18.40156	14.39318	16.75297	22.49413	36.27588	31.81766	27.304	25.09662
AC109635.	0.960908	0	0	0	0	0	1.69936	0.926027	0	0	0	0	0	0	0	0	0	0	1.792616
AC109635.	0.960908	1.00039	0.999134	1.039293	1.107485	0	1.69936	0	0	0	0	0	0	0	0.978006	0	1.026376	0	0.896308
AC109779.	9.609075	8.003124	0	8.314342	0	2.283774	0.84968	6.482189	0	0.960699	0	5.520469	0	4.653602	1.956012	1.648904	11.29014	4.550667	8.066772
AC109780.	0	0	0	0	0	0	0	0	0	0	0	0	0	0	0	0	0	0	1.792616
AC109780.	0	3.001171	0	0	1.107485	0	3.398721	0	0	0	0	0	0	0	0	0	0	0	0
AC109809.	0	5.001952	14.987	21.82515	11.07485	1.141887	5.098081	5.556162	11.67288	0.960699	4.81902	3.680313	5.140422	2.792161	5.868035	5.771163	1.026376	0.910133	3.585232
AC109811.	8.648168	2.000781	21.98094	7.275049	0	15.98642	5.947762	12.96438	1.167288	7.685594	22.89034	11.96102	8.224675	16.75297	2.934017	16.48904	22.58028	6.370934	6.274156
AC109811.	0	0	0	1.039293	1.107485	0	0.84968	0	0	0	0	0	1.028084	0	4.890029	0	0	0	0.896308
AC109826.	701.4625	654.2553	474.5885	948.8743	435.2416	484.1601	519.1546	632.4765	689.867	531.2667	480.6972	774.7059	655.9178	591.0074	609.2976	1898.713	1637.07	1529.024	1046.888
AC109830.	0	0	0	4.157171	0	0	0	0	0	0	0	0	0	0	0	0	0	3.640534	0
AC109927.	9.609075	7.002733	11.9896	4.157171	5.537425	2.283774	3.398721	10.1863	1.167288	3.842797	4.81902	2.760235	4.112337	10.23792	15.64809	9.06897	3.079129	7.281067	3.585232
AC109992.	6.726353	10.0039	11.9896	23.90373	22.1497	17.12831	33.13753	33.33697	3.501863	14.41049	19.27608	20.24172	12.33701	21.40657	18.58211	15.66458	16.42202	11.83173	18.82247
AC110015.	18.25724	49.01913	22.98007	24.94303	19.93473	33.11472	33.98721	15.74246	7.003726	81.65944	44.57593	37.72321	26.73019	17.68369	39.12023	11.54233	23.60665	20.02294	28.68185
AC110023.	10.56998	25.00976	38.96621	4.157171	25.47215	5.709435	28.03945	16.66849	18.6766	14.41049	15.66181	11.04094	3.084253	17.68369	7.824047	21.43575	24.63303	1.820267	1.792616
AC110048.	0	4.001562	21.98094	4.157171	0	0	6.797442	10.1863	0	10.56769	0	20.24172	16.44935	10.23792	21.51613	10.71787	8.21101	0.910133	2.688924
AC110048.	99.93438	113.0441	103.9099	55.08252	127.3608	98.20228	108.7591	91.67668	290.6546	140.2621	97.58515	173.8948	148.0441	122.8551	122.2507	59.36053	68.76721	91.92348	81.56402
AC110053.	9.609075	6.002343	1.998267	2.078586	1.107485	0	6.797442	0	0	0	0	0	5.140422	0.93072	0	0.824452	4.105505	0	0
AC110056.	0.960908	0	0	0	0	0	0	0	0	0	0	0.920078	0	0.93072	0	0.824452	5.131881	0	0
AC110058.	2.882723	5.001952	0	1.039293	0	1.141887	5.947762	7.408216	2.334575	8.646293	0	1.840156	0	0	0	10.71787	8.21101	29.12427	6.274156
AC110079.	2780.866	2010.785	2645.706	1759.523	2576.01	2492.739	2167.534	2768.821	4853.582	2665.94	4015.448	2846.722	2713.115	2519.46	2618.122	2038.045	2228.263	3118.117	3224.02
AC110079.	68.22443	165.0644	116.8986	67.55403	52.05179	55.95246	115.5565	92.6027	15.17474	41.31007	61.4425	83.72712	81.21867	82.83411	77.26246	46.1693	44.13418	88.28294	107.557
AC110285.	877.3086	963.376	841.2705	807.5305	1139.602	631.4635	989.0278	651.923	605.8223	989.5202	1410.768	621.9729	688.8165	696.1788	680.6921	368.53	655.8544	374.975	576.326
AC110285.	987.8129	795.3104	905.2151	274.3733	1240.383	1175.002	1278.769	705.6326	1886.337	1772.49	3590.17	1181.38	1222.392	1589.67	1358.45	614.2166	870.3671	795.4566	1189.401
AC110285.	6.726353	16.00625	8.992203	20.78586	5.537425	5.709435	29.73881	10.1863	1.167288	17.29259	32.52838	13.80117	9.252759	4.653602	4.890029	4.122259	25.65941	0.910133	14.34093
AC110285.	3.84363	4.001562	1.998267	0	0	1.141887	0	0.926027	14.00745	0	10.84279	0.920078	6.168506	1.861441	0	0	0	0	5.377848
AC110285.	5.765445	8.003124	10.99047	2.078586	12.18233	1.141887	1.69936	6.482189	4.669151	3.842797	18.07132	0.920078	3.084253	0	1.956012	0.824452	0	8.191201	5.377848
AC110285.	1.921815	8.003124	1.998267	20.78586	16.61227	12.56076	2.549041	8.334243	9.338302	11.52839	3.614265	0.920078	6.168506	1.861441	21.51613	0	4.105505	3.640534	8.066772
AC110288.	0.960908	0	5.994802	13.51081	0	1.141887	5.947762	0	0	0	0	2.760235	0	6.515042	2.934017	9.06897	0	0	7.170464
AC110296.	0.960908	2.000781	0.999134	1.039293	0	6.851322	6.797442	1.852054	4.669151	1.921399	0	3.680313	7.196591	1.861441	2.934017	3.297807	11.29014	0	10.7557
AC110373.	87.44259	79.03084	115.8995	125.7544	80.8464	33.11472	448.6312	59.26573	18.6766	24.01748	32.52838	84.6472	77.10633	63.28898	82.15249	123.6678	143.6927	88.28294	112.0385
AC110491.	680.3225	1027.401	754.3459	1338.609	612.4392	1220.677	903.2101	648.2189	269.6435	636.9436	371.0645	517.084	635.3561	708.2782	684.6041	773.3358	715.3842	483.2808	589.7706
AC110491.	0	1.00039	0.999134	0	4.42994	10.27698	1.69936	0.926027	2.334575	5.764196	0	0	1.028084	0	0	0.824452	0	2.7304	0.896308

AC110491.	0.960908	0	2.997401	0	0	6.851322	0.84968	5.556162	4.669151	0	1.204755	2.760235	0	0.93072	0	0	0	6.370934	0
AC110597.	121.0743	123.048	132.8848	138.2259	158.3703	123.3238	209.0213	177.7972	105.0559	100.8734	85.5376	147.2125	139.8195	141.4695	144.7449	128.6145	102.6376	121.9579	144.3056
AC110602.	1.921815	0	0.999134	0	0	0	0	0	0	0.960699	1.204755	0	0	0	0	0.824452	3.079129	2.7304	0
AC110603.	0	0	0	2.078586	0	0	0	0.926027	0	0	0	0	1.028084	0	0	0	0	0	0
AC110609.	4.804538	1.00039	26.97661	2.078586	2.21497	9.135096	0.84968	0.926027	4.669151	7.685594	1.204755	6.440548	6.168506	17.68369	2.934017	7.420066	2.052753	8.191201	8.066772
AC110615.	5.765445	1.00039	0.999134	0	0	0	0.84968	0	0	0.960699	1.204755	2.760235	3.084253	0	0	0.824452	2.052753	1.820267	1.792616
AC110620.	0	0	2.997401	0	0	1.141887	0.84968	0	0	0	0	0	0	0	0	0	0	0	7.170464
AC110716.	0	0	0	0	0	0	0	0	0	0	0	0.920078	0	0	0	0	0	0	3.585232
AC110716.	0	0	0.999134	0	0	0	0	0	0	0.960699	0	0	0	0	0	0	0	0	0
AC110741.	29.78813	14.00547	13.98787	8.314342	28.79461	10.27698	12.7452	42.59724	26.84762	24.01748	24.0951	17.48149	38.03912	22.33729	14.67009	0.824452	20.52753	6.370934	3.585232
AC110743.	0	0	0	0	0	0	0	0	0	0.960699	0	0.920078	0	0	0	0	1.026376	0	3.585232
AC110749.	1.921815	8.003124	0	1.039293	0	4.567548	0	3.704108	5.836439	0	2.40951	0.920078	2.056169	0	3.912023	0.824452	0	0	0.896308
AC110751.	0	2.000781	0.999134	6.235757	13.28982	0	5.098081	0.926027	5.836439	1.921399	3.614265	3.680313	6.168506	0	10.75806	5.771163	0	1.820267	4.48154
AC110753.	0.960908	9.003514	4.995668	0	1.107485	0	0	0.84968	7.408216	0	0.960699	0	0	0	3.722881	0	0.824452	3.079129	0.910133	0.896308
AC110754.	4.804538	7.002733	7.993069	6.235757	0	6.851322	7.647122	6.482189	4.669151	0	0	0.920078	0	0	0.978006	5.771163	3.079129	0	0.896308
AC110760.	0	0	0	0	0	0	0	0	0	0	0	0	1.028084	0	0	0	0	0	0.896308
AC110760.	3.84363	1.00039	12.98874	2.078586	0	1.141887	6.797442	0	0	0	0	0	0	1.861441	1.956012	1.648904	3.079129	1.820267	3.585232
AC110768.	0	0	5.994802	1.039293	0	0	0	0	0	0	0	0	1.028084	0.93072	5.868035	3.297807	0	1.820267	0.896308
AC110768.	0	0	0	0	0	0	0	0	1.167288	0	0	0.920078	0	3.722881	0	0.824452	0	0	1.792616
AC110769.	4.804538	0	0	6.235757	3.322455	3.425661	3.398721	1.852054	0	1.921399	1.204755	2.760235	1.028084	2.792161	0.978006	8.244518	2.052753	5.4608	0
AC110769.	72.06806	77.03006	89.92203	89.37918	76.41646	22.83774	78.17058	77.78627	78.20828	48.03496	73.49005	99.36845	79.1625	81.90339	121.2727	83.26963	54.39794	59.15867	42.12647
AC110769.	0	0	0	0	0	0	0	0	0	0	0	0	1.028084	0.93072	0	13.19123	2.052753	0.910133	0.896308
AC110772.	2.882723	0	1.998267	4.157171	0	9.135096	1.69936	0.926027	0	11.52839	19.27608	4.600391	13.3651	1.861441	0	2.473355	0	6.370934	3.585232
AC110774.	30.74904	38.01484	51.95495	108.0864	36.547	92.49285	80.71962	51.85751	16.34203	39.38867	27.70936	85.56728	33.92678	28.85233	43.03226	83.26963	82.1101	70.99041	87.83818
AC110792.	6.726353	30.01171	34.96968	47.80747	26.57964	25.12151	33.98721	35.18903	10.50559	33.62447	8.433284	15.64133	21.58977	16.75297	37.16422	24.73355	20.52753	18.20267	29.57816
AC110792.	10.56998	1.00039	0.999134	15.58939	7.752394	0	9.346483	0	0	0.960699	0	0.920078	1.028084	0	1.956012	9.893422	7.184634	5.4608	4.48154
AC110792.	44.20175	38.01484	78.93156	36.37525	32.11706	26.2634	48.43177	38.89314	16.34203	58.60265	40.96167	40.48344	57.57272	62.35826	37.16422	22.2602	15.39564	43.6864	25.09662
AC110794.	5.765445	4.001562	0	0	0	0	0	0	0	0	4.81902	0	0	1.861441	3.912023	0	0	0	0
AC110799.	1.921815	0	2.997401	5.196464	0	1.141887	0.84968	0	18.6766	1.921399	6.023774	2.760235	3.084253	11.16864	1.956012	6.595615	10.26376	10.9216	6.274156
AC110801.	0	0	0	0	0	0	0	0	0	0	0	0	0	0	0.978006	2.473355	0	0.910133	0
AC110813.	22.10087	28.01093	10.99047	40.53242	35.43952	35.3985	19.54265	13.89041	19.84389	27.86028	42.16642	3.680313	3.084253	14.89152	18.58211	18.96239	21.5539	0	13.44462
AC110921.	0	0	0	0	0	0	0	3.704108	0	0.960699	0	4.600391	4.112337	0	0	4.946711	2.052753	0.910133	0.896308
AC110926.	1.921815	3.001171	4.995668	3.117878	19.93473	3.425661	0.84968	11.11232	0	5.764196	2.40951	2.760235	2.056169	6.515042	14.67009	0.824452	0	4.550667	1.792616
AC110994.	0	2.000781	0	1.039293	0	0	0	0	0	0	0	0	0	0	0	0	0	0.910133	0
AC110994.	13.45271	6.002343	10.99047	4.157171	15.50479	23.97963	16.14392	11.11232	21.01118	2.882098	18.07132	9.200782	2.056169	4.653602	5.868035	6.595615	9.237386	10.01147	3.585232
AC110995.	757.1951	157.0613	136.8813	1218.051	262.4739	216.9585	205.6226	304.6629	247.465	218.0787	193.9655	621.0528	328.987	349.0201	360.8841	1612.628	728.7271	1100.351	446.3614
AC110998.	17.29634	38.01484	26.97661	6.235757	40.97694	7.993209	18.69297	41.67122	16.34203	26.89958	10.84279	46.00391	25.70211	31.64449	37.16422	26.38246	18.47477	17.29253	35.85232
AC111000.	0	0	0	10.39293	15.50479	13.70264	4.248401	0	0	10.56769	0	12.8811	9.252759	0	0	0	0	0	0
AC111000.	0	0	0	23.90373	32.11706	27.40529	0.84968	0	0	19.21399	8.433284	23.00196	24.67402	0	0	0	0	0	0
AC111000.	0	0	0	2.078586	0	0	0	0	0	0	0	0	0	0	0	0	0	0	0
AC111149.	1.921815	0	0	0	0	0	0	0	0	0	0	0.920078	0	0	0	0	0	0	0
AC111152.	0	0	0.999134	6.235757	0	0	0	0	0	0	0	0	0	0.93072	0	0	0	0	0
AC111152.	5.765445	7.002733	5.994802	12.47151	0	0	0	3.704108	0	0	4.81902	0	0	5.584322	14.67009	9.893422	5.131881	0.910133	0
AC111152.	2.882723	3.001171	4.995668	0	5.537425	1.141887	0	6.482189	0	0.960699	0	0.920078	1.028084	1.861441	2.934017	5.771163	4.105505	18.20267	12.54831
AC111170.	14.41361	6.002343	22.98007	13.51081	16.61227	0	11.89552	16.66849	0	6.724895	22.89034	9.200782	18.50552	6.515042	4.890029	5.771163	6.158258	5.4608	2.688924
AC111170.	8.648168	0	1.998267	4.157171	0	2.283774	4.248401	15.74246	3.501863	0	0	19.32164	6.168506	7.445762	0.978006	0	1.026376	8.191201	0.896308
AC111170.	68.22443	67.02616	41.96361	59.23969	100.7811	90.20907	90.06611	62.96984	226.4538	73.01314	101.1994	103.9688	113.0893	71.66546	65.52639	102.232	40.02867	62.79921	78.8751
AC111182.	3.84363	7.002733	15.98614	3.117878	11.07485	12.56076	6.797442	19.44657	9.338302	21.13538	26.50461	2.760235	18.50552	11.16864	20.53812	6.595615	17.4484	0.910133	7.170464
AC111186.	10.56998	5.001952	2.997401	1.039293	1.107485	6.851322	5.098081	2.778081	5.836439	1.921399	2.40951	6.440548	2.056169	7.445762	20.53812	9.893422	11.29014	10.9216	7.170464
AC111188.	5.765445	10.0039	18.98354	6.235757	15.50479	6.851322	8.496802	7.408216	0	23.05678	20.48083	8.280704	4.112337	13.9608	12.71408	7.420066	1.026376	23.66347	9.859387
AC111188.	0	2.000781	3.996535	0	1.107485	0	0	0	3.501863	0.960699	1.204755	0	8.224675	7.445762	0	0.824452	0	0	0
AC111193.	0	0	0	0	0	0	0	0	7.003726	0	0	0.920078	1.028084	2.792161	6.846041	0.824452	0	0	0
AC111194.	0	1.00039	0	1.039293	0	1.141887	0	0	0	0	0	0	0	0	0.978006	0.824452	0	0.910133	0.896308
AC111194.	6.726353	7.002733	4.995668	1.039293	9.967364	1.141887	31.43817	16.66849	0	6.724895	20.48083	9.200782	8.224675	5.584322	16.6261	11.54233	5.131881	2.7304	17.02985
AC111200.	3.84363	2.000781	2.997401	3.117878	1.107485	2.283774	1.69936	0.926027	3.501863	3.842797	13.2523	2.760235	5.140422	1.861441	1.956012	2.473355	4.105505	2.7304	1.792616
AC112173.	4.804538	0	5.994802	0	1.107485	1.141887	0	9.26027	0	1.921399	7.228529	0.920078	2.056169	0	0	0	0	0.910133	0
AC112176.	0	0	0.999134	0	0	1.141887	0	0.926027	0	0	0	0	0	0	0	0	0	1.820267	0.896308
AC112178.	0	0	0	0	0	0	0	0	0	0	0	0	0	0	0	1.648904	0	0	0
AC112184.	82.63805	88.03436	106.9073	83.14342	86.38382	73.08077	51.83049	93.52873	315.1677	110.4804	292.7554	64.40548	69.90974	74.45762	105.6246	65.95615	87.24198	48.23707	52.88217
AC112187.	0	3.001171	0	2.078586	0	0	0	2.778081	0	0	0	0	0	0.93072	0	0.824452	1.026376	0	0.896308
AC112187.	3.84363	5.001952	15.98614	3.117878	18.82724	15.98642	4.248401	23.15068	28.01491	6.724895	39.75691	10.12086	10.28084	12.09936	8.802052	9.06897	3.079129	7.281067	4.48154
AC112191.	1.921815	0	5.994802	15.58939	0	2.283774	4.248401	5.556162	0	0	0	8.280704	1.028084	2.792161	0	7.420066	5.131881	2.7304	3.585232

AC112191.	8.648168	15.00586	5.994802	6.235757	4.42994	10.27698	6.797442	6.482189	9.338302	17.29259	3.614265	11.04094	7.196591	13.9608	4.890029	8.244518	5.131881	4.550667	7.170464
AC112192.	0	0	0	0	4.42994	2.283774	0.84968	0	0	3.842797	0	2.760235	0	2.792161	0.978006	4.946711	0	0	0
AC112196.	0	0	5.994802	0	0	0	0.84968	0	0	1.921399	0	0.920078	0	0	0.978006	1.648904	1.026376	0	0.896308
AC112198.	2.882723	3.001171	0	2.078586	0	1.141887	1.69936	7.408216	0	0	6.023774	10.12086	5.140422	3.722881	0.978006	18.13794	15.39564	8.191201	7.170464
AC112200.	0.960908	1.00039	5.994802	1.039293	1.107485	2.283774	0	0	0	0	1.204755	0.920078	0	1.861441	0.978006	1.648904	2.052753	1.820267	0.896308
AC112206.	0	0	0	0	0	0	0.84968	0	1.167288	5.764196	0	0	0	0.93072	0	0	1.026376	0	0
AC112206.	123.9571	211.0824	376.6734	201.6228	238.1093	93.63473	157.1908	293.5506	215.9482	97.03062	113.247	137.0917	133.651	295.0383	303.1818	103.8809	93.40024	51.8776	48.40063
AC112206.	29.78813	75.02928	168.8536	88.33989	160.5853	9.135096	11.04584	133.3479	405.0488	72.05244	69.87578	39.56336	41.12337	265.2553	303.1818	24.73355	10.26376	14.56213	13.44462
AC112206.	14.41361	55.02147	165.8562	47.80747	39.86946	33.11472	20.39233	84.26846	70.03726	82.62014	53.00921	46.00391	45.23571	155.4303	133.9868	14.84013	18.47477	15.47227	19.71877
AC112219.	0.960908	1.00039	0	10.39293	8.859879	0	5.098081	0	23.34575	3.842797	1.204755	0	0	0	2.934017	0.824452	1.026376	0	0.896308
AC112220.	0	0	1.998267	2.078586	0	1.141887	1.69936	0	0	0	0	0	0	0	0	0	0	0	0
AC112220.	139.3316	141.0551	143.8752	242.1552	116.2859	103.9117	193.7271	103.715	35.01863	80.69874	34.93789	134.3314	115.1454	149.846	147.6789	123.6678	128.297	107.3957	155.0613
AC112220.	59.57627	130.0508	59.94802	72.75049	97.45867	62.80379	52.68017	75.93422	88.71387	96.06993	57.82823	56.12477	43.17954	86.55699	97.80058	40.39814	61.58258	64.61947	56.4674
AC112220.	420.8775	440.1718	312.7288	473.9175	274.6563	318.5865	410.3956	351.8903	100.3867	178.6901	183.1227	362.5108	284.7794	337.8515	446.9487	394.9124	433.1308	334.019	310.1226
AC112225.	0	0	0	0	0	2.21497	0	2.549041	0	0	0	0	0	0	0	0	0	0	0
AC112229.	7.68726	4.001562	0.999134	1.039293	2.21497	0	1.69936	3.704108	0	0.960699	1.204755	9.200782	3.084253	3.722881	7.824047	14.84013	20.52753	0	1.792616
AC112229.	3.84363	0	0	0	0	0	0	0	0	0	0	1.840156	0	0	1.956012	0.824452	0	0	0
AC112229.	5.765445	2.000781	0	1.039293	3.322455	2.283774	4.248401	0	0	0	1.204755	1.840156	14.39318	0	9.780058	5.771163	5.131881	10.01147	4.48154
AC112236.	0.960908	13.00508	14.987	4.157171	2.21497	4.567548	20.39233	0.926027	0	0	0	3.680313	2.056169	0	0	6.595615	6.158258	12.74187	6.274156
AC112236.	23.06178	46.01796	57.94975	45.72888	18.82724	29.68906	49.28145	22.22465	12.84017	17.29259	18.07132	30.36258	32.8987	43.74385	24.45015	49.46711	34.89679	49.1472	50.19325
AC112243.	0	0	0	1.039293	0	0	0.84968	0	0	0	0	0	0	0	0	0.824452	0	0	0
AC112247.	0	0	1.998267	0	1.107485	0	0	0	0	0	1.204755	0	0	0	0	0	1.026376	0	0.896308
AC112250.	0	0	0	0	0	0	0	0	0	0	0	0	0	3.722881	0	0	0	0	0
AC112250.	42.27993	52.0203	52.95408	83.14342	69.77155	22.83774	62.87634	19.44657	85.212	21.13538	13.2523	28.52243	14.39318	25.12945	29.34017	58.53608	58.50345	38.2256	3.585232
AC112255.	0	0	0	0	0	0	0.84968	0.926027	0	3.842797	0	0	1.028084	0.93072	0.978006	1.648904	0	1.820267	0
AC112484.	55.73264	83.03241	69.93936	136.1474	69.77155	81.07398	109.6088	87.04654	74.70641	98.95202	86.74235	70.84602	105.8927	130.3008	92.91055	85.74299	84.16285	86.46267	102.1791
AC112484.	118.1916	115.0449	173.8493	102.89	166.1227	127.8913	191.1781	145.3862	163.4203	104.7162	97.58515	96.60821	98.6961	109.825	116.3827	156.6458	150.8773	118.3173	136.2388
AC112484.	10.56998	3.001171	10.99047	2.078586	13.28982	5.709435	16.14392	20.37259	16.34203	16.33189	9.638039	4.600391	18.50552	12.09936	16.6261	17.31349	10.26376	10.9216	12.54831
AC112484.	30.74904	67.02616	83.92723	37.41454	88.59879	89.06719	68.8241	50.93149	194.9371	60.52405	85.5376	50.6043	57.57272	53.05106	52.81231	89.86525	125.2179	59.15867	50.19325
AC112484.	24.9836	18.00703	18.98354	21.82515	24.36467	14.84453	25.49041	18.52054	44.35693	22.09608	19.27608	17.48149	33.92678	25.12945	22.49413	7.420066	24.63303	13.652	17.92616
AC112487.	11.53089	21.0082	7.993069	39.49313	27.68712	13.70264	92.61515	10.1863	37.35321	20.17468	3.614265	10.12086	17.47743	55.84322	44.98827	24.73355	25.65941	7.281067	9.859387
AC112487.	0	8.003124	0	1.039293	0	0	0	0	0	0	0	0	1.028084	0	0	0.824452	1.026376	2.7304	0
AC112491.	0	0	0	0	0	0	0	0	0	0	0	0	0	0	0	0	0	1.820267	0
AC112492.	0	0	0	0	0	0	0	0	0	0	0	0	0	0	0	0	2.052753	0	0
AC112492.	0	1.00039	0	3.117878	0	12.56076	3.398721	0.926027	0	0	0	0	2.056169	4.653602	2.934017	4.946711	1.026376	3.640534	0
AC112493.	0.960908	1.00039	0	1.039293	2.21497	7.993209	4.248401	0	0	0	0	6.440548	3.084253	0	0	0	0	3.640534	0
AC112495.	14.41361	3.001171	1.998267	12.47151	0	1.141887	5.947762	5.556162	30.34948	4.803496	0	2.760235	3.084253	5.584322	10.75806	7.420066	8.21101	0	0
AC112496.	68.22443	61.02382	94.9177	74.82908	36.547	45.67548	107.0597	99.08489	7.003726	19.21399	43.37118	101.2086	63.74123	61.42754	86.06451	77.49847	83.13648	177.476	124.5868
AC112497.	11.53089	17.00664	20.98181	2.078586	17.71976	29.68906	11.89552	29.63287	128.4017	45.15286	175.8942	28.52243	17.47743	18.61441	5.868035	8.244518	23.60665	22.75334	8.066772
AC112503.	74.95079	59.02304	74.93502	87.30059	94.13622	60.52001	78.17058	53.70957	63.03354	91.26643	65.05676	67.16571	62.71315	48.39746	48.90029	65.13169	68.76721	46.4168	54.67478
AC112504.	5.765445	0	9.991337	0	25.47215	10.27698	4.248401	10.1863	5.836439	4.803496	1.204755	4.600391	4.112337	6.515042	2.934017	1.648904	3.079129	3.640534	10.7557
AC112512.	4.804538	7.002733	3.996535	6.235757	11.07485	7.993209	5.947762	10.1863	17.50932	15.37119	9.638039	17.48149	9.252759	9.307203	6.846041	0	1.026376	4.550667	0
AC112518.	0	0	0	2.078586	4.42994	0	0	0	0	0	0	0	0	0	0	0	0	0	0
AC112656.	0.960908	0	0	0	0	0	1.69936	0.926027	0	0	0	0	0	0	0	4.946711	0	6.370934	0
AC112673.	1.921815	0	4.995668	0	0	0	0	0.926027	11.67288	0.960699	0	3.680313	0	0	0	0	0	0.910133	0
AC112693.	10.56998	8.003124	18.98354	2.078586	1.107485	2.283774	12.7452	13.89041	5.836439	9.606993	9.638039	10.12086	3.084253	7.445762	4.890029	5.771163	5.131881	0.910133	6.274156
AC112693.	0	0	0.999134	0	0	0	0	2.549041	0	0	0	0	0.920078	0	0	0	0	0	0
AC112693.	3.84363	6.002343	6.993936	3.117878	9.967364	3.425661	3.398721	2.778081	11.67288	0	12.04755	0	8.224675	4.653602	0	7.420066	1.026376	20.02294	8.963079
AC112694.	52.84991	98.03826	123.8926	58.2004	78.63143	103.9117	107.9094	197.2438	11.67288	97.99132	71.08054	126.9708	115.1454	92.14131	77.26246	121.1944	67.74083	121.9579	77.08248
AC112694.	118.1916	106.0414	169.8527	148.6189	160.5853	158.7223	153.7921	197.2438	126.0671	186.3757	168.6657	126.9708	131.5948	124.7165	163.327	129.4389	122.1388	174.7456	100.3865
AC112695.	0	4.001562	0	1.039293	0	0	0	0	0	0	0	0	0	0.93072	0	0	0	0	0
AC112702.	0	0	0	0	0	0	0	2.778081	0	0	0	0	0	0	0	0	1.026376	0	0.896308
AC112715.	0	0	0	8.314342	14.3973	0	2.549041	1.852054	2.334575	2.882098	4.81902	1.840156	0	0.93072	6.846041	0	4.105505	0.910133	5.377848
AC112719.	0	2.000781	4.995668	0	0	1.141887	8.496802	6.482189	0	2.882098	0	0	0	2.792161	0	3.297807	1.026376	0	1.792616
AC112721.	0	0	0	0	0	0	0	0	0	0	0	0	0	0	0	4.946711	0	0	0
AC112722.	24.9836	43.01679	37.96708	84.18271	26.57964	94.77662	24.64073	66.67395	71.20455	81.65944	85.5376	55.20469	45.23571	58.63538	50.8563	60.18498	62.60895	18.20267	36.74863
AC112777.	0	4.001562	0	0	21.04221	10.27698	4.248401	0.926027	0	13.44979	31.32363	3.680313	2.056169	0	0.978006	6.595615	1.026376	24.5736	18.82247
AC112907.	4.804538	12.00469	2.997401	2.078586	2.21497	7.993209	5.947762	8.334243	4.669151	1.921399	1.204755	5.520469	9.252759	5.584322	9.780058	8.244518	10.26376	10.9216	18.82247
AC112907.	19.21815	31.0121	28.97488	15.58939	8.859879	34.25661	24.64073	33.33697	23.34575	24.97818	43.37118	37.72321	27.75828	21.40657	32.27419	23.9091	26.68578	66.43974	73.49725
AC112907.	270.9759	184.0718	192.8328	405.3242	139.5431	79.93209	280.3945	163.9068	91.04844	146.987	98.7899	159.1735	201.5045	233.6108	211.2493	368.53	364.3636	449.6059	544.9552
AC113133.	2.882723	8.003124	6.993936	17.66798	4.42994	17.12831	7.647122	22.22465	35.01863	0	12.04755	9.200782	13.3651	3.722881	7.824047	10.71787	10.26376	1.820267	2.688924
AC113137.	1.921815	0	1.998267	0	0	1.141887	0.84968	0.926027	0	0	0	0	1.028084	0.93072	0	0	3.079129	0	0.896308

AC113139.	15.37452	23.00898	24.97834	12.47151	45.40688	12.56076	28.03945	33.33697	23.34575	57.64196	50.5997	30.36258	31.87062	30.71377	41.07624	16.48904	10.26376	18.20267	27.78555
AC113143.	2.882723	10.0039	5.994802	8.314342	54.26676	15.98642	3.398721	6.482189	81.71014	23.05678	59.03299	1.840156	10.28084	19.54513	12.71408	4.122259	2.052753	10.9216	1.792616
AC113143.	0.960908	13.00508	11.9896	1.039293	4.42994	7.993209	2.549041	3.704108	9.338302	20.17468	14.45706	0.920078	5.140422	0.93072	1.956012	0	6.158258	4.550667	1.792616
AC113145.	0	0	0	0	0	1.141887	0.84968	0	9.338302	0	0	3.680313	0	2.792161	0	0	0	0	0
AC113146.	25.9445	9.003514	25.97748	1.039293	4.42994	1.141887	17.84329	3.704108	0	8.646293	10.84279	2.760235	7.196591	5.584322	14.67009	4.946711	5.131881	0.910133	16.13354
AC113146.	13.45271	0	0	0	11.07485	0	0.84968	0	1.167288	0	0	2.760235	0	1.861441	0.978006	0.824452	5.131881	5.4608	0.896308
AC113155.	0	2.000781	0	2.078586	0	0	0.84968	0	0	4.803496	6.023774	0.920078	0	0	0	12.36678	0	0	0
AC113167.	9.609075	0	0	0	0	3.425661	0	0	9.338302	0.960699	0	0.920078	2.056169	0.93072	2.934017	2.473355	0	0	0
AC113189.	0	0	0	0	0	0	0	0	0	0	0	0	0	0	0	0	0	1.820267	0
AC113189.	0	0	0	0	5.537425	0	1.69936	3.704108	0	0	0	0	0	0	0	0	0	0	0
AC113189.	0	0	0	0	0	0	5.098081	3.704108	0	0	0	0	0	0	0	0.824452	1.026376	8.191201	0.896308
AC113191.	345.9267	267.1042	292.7462	678.6582	266.9039	372.2552	546.3444	394.4875	21.01118	139.3014	48.1902	451.7584	417.4023	355.5352	338.39	568.0473	394.1285	345.8507	395.2718
AC113192.	0	3.001171	0	0	0	0	5.098081	1.852054	7.003726	0	0	1.840156	0	0	0	2.473355	1.026376	0.910133	0
AC113192.	0.960908	3.001171	0.999134	0	2.21497	4.567548	3.398721	1.852054	0	8.646293	0	0.920078	1.028084	2.792161	1.956012	1.648904	0	0.910133	1.792616
AC113192.	0.960908	2.000781	0	1.039293	0	1.141887	1.69936	0	0	7.685594	0	0.920078	0	0.93072	0.978006	1.648904	1.026376	0	3.585232
AC113194.	5.765445	7.002733	0	1.039293	0	1.141887	0	3.704108	0	0	0	0.920078	4.112337	2.792161	1.956012	0	1.026376	0.910133	0.896308
AC113208.	13.45271	3.001171	6.993936	11.43222	16.61227	12.56076	10.19616	3.704108	25.68033	7.685594	1.204755	1.840156	8.224675	0.93072	7.824047	0	6.158258	6.370934	4.48154
AC113208.	4.804538	2.000781	1.998267	3.117878	1.107485	1.141887	0	0.926027	23.34575	4.803496	1.204755	1.840156	1.028084	0.93072	0.978006	3.297807	1.026376	2.7304	2.688924
AC113208.	35.55358	53.02069	48.95755	42.611	66.4491	34.25661	60.3273	59.26573	19.84389	61.48475	89.15186	44.16376	69.90974	29.78305	14.67009	11.54233	23.60665	40.04587	86.94187
AC113340.	0	4.001562	0	0	0	0	0	5.556162	0	0	0	0	2.056169	0	0	0	1.026376	0	0
AC113346.	10.56998	10.0039	4.995668	7.275049	8.859879	4.567548	0.84968	7.408216	5.836439	9.606993	4.81902	1.840156	2.056169	7.445762	0	10.71787	6.158258	0	3.585232
AC113346.	0.960908	0	3.996535	0	0	0	4.248401	0	0	0	0	0.920078	2.056169	0.93072	0	5.771163	1.026376	0.910133	2.688924
AC113347.	16.33543	23.00898	7.993069	3.117878	0	20.55397	1.69936	16.66849	8.171014	13.44979	1.204755	4.600391	3.084253	18.61441	4.890029	0.824452	12.31652	5.4608	0
AC113349.	0	2.000781	0	0	0	0	0	0	0	0	0	0	0	0	0	0	0	0	0
AC113352.	0	0	0	5.196464	0	0	0.84968	0	0	0	0	0	0	0	0	0	0	0	0
AC113355.	0.960908	0	0	0	0	0	1.69936	0	0	0	0	0	0	0	0	0	0	0	0
AC113361.	21.13997	41.01601	29.97401	0	22.1497	12.56076	9.346483	20.37259	49.02608	24.01748	26.50461	13.80117	15.42127	15.82225	10.75806	2.473355	15.39564	8.191201	9.859387
AC113368.	0.960908	4.001562	4.995668	15.58939	6.64491	0	11.89552	5.556162	7.003726	5.764196	0	9.200782	2.056169	12.09936	4.890029	1.648904	4.105505	5.4608	3.585232
AC113378.	5.765445	0	1.998267	0	0	3.425661	0.84968	0	0	0	0	1.840156	0	0	0	0	2.052753	1.820267	0
AC113382.	24.02269	39.01523	41.96361	14.5501	13.28982	26.2634	29.73881	37.96711	42.02236	59.56335	51.80446	31.28266	40.09529	67.94258	45.96627	15.66458	3.079129	19.1128	29.57816
AC113382.	7.68726	6.002343	12.98874	21.82515	11.07485	1.141887	12.7452	5.556162	0	3.842797	9.638039	15.64133	10.28084	0	26.40616	8.244518	4.105505	9.101334	3.585232
AC113383.	783.1396	781.3049	881.2359	874.0452	836.1511	905.5164	995.8252	873.2435	741.2277	810.8302	790.3192	748.0236	846.1134	690.5945	762.8445	743.6556	833.4175	626.1718	723.3205
AC113385.	0	6.002343	3.996535	0	0	0	0	1.852054	0	7.685594	0	0	1.028084	0	0	1.648904	4.105505	0	4.48154
AC113385.	23.06178	14.00547	16.98527	10.39293	12.18233	19.41208	14.44456	51.85751	11.67288	16.33189	3.614265	40.48344	37.01104	7.445762	15.64809	4.122259	8.21101	15.47227	39.43755
AC113386.	8.648168	0	4.995668	0	0	1.141887	0	4.630135	0	1.921399	1.204755	0	5.140422	0	1.956012	4.946711	5.131881	4.550667	3.585232
AC113391.	0	0	0	0	0	0	0.84968	0	0	0	0	0	0	0	0	0	0	1.820267	0
AC113398.	2.882723	4.001562	0.999134	0	1.107485	2.283774	0.84968	4.630135	16.34203	4.803496	8.433284	1.840156	2.056169	5.584322	4.890029	4.122259	2.052753	2.7304	3.585232
AC113403.	0.960908	0	0	0	0	0	0	0	0	0	0	0	0	3.722881	0	0	0	0	0
AC113404.	3.84363	1.00039	7.993069	1.039293	4.42994	0	5.098081	0.926027	0	3.842797	6.023774	4.600391	1.028084	0.93072	2.934017	0	0	0.910133	0
AC113404.	23.06178	37.01445	42.96275	31.17878	23.25718	35.3985	37.38593	31.48492	9.338302	30.74238	10.84279	23.00196	15.42127	19.54513	34.2302	35.45143	34.89679	36.40534	34.95601
AC113410.	14.41361	27.01054	16.98527	40.53242	74.20149	26.2634	29.73881	62.04381	32.68406	17.29259	24.0951	38.64329	52.4323	41.88241	32.27419	18.96239	29.76491	64.61947	34.95601
AC113410.	0	0	0	0	0	1.141887	0	0	1.167288	0	0	0	0	0	0	0	0	0.910133	0
AC113410.	0	0	0	1.039293	0	0	0	0	5.836439	1.921399	0	0	1.028084	0	0	2.473355	0	0	0
AC113412.	0	0	0.999134	0	0	1.141887	0	1.852054	0	0	0	0	0	0	0	0.824452	0	0	0
AC113418.	0	0	0	0	0	0	0	3.704108	0	0	0	0	0	0	0.978006	0	0	0	0.896308
AC113423.	0.960908	0	0	0	2.21497	4.567548	0	0	0	0	0	0	0	0	0	0	2.052753	0	0
AC113554.	1.921815	2.000781	5.994802	1.039293	4.42994	5.709435	16.14392	0.926027	1.167288	4.803496	2.40951	4.600391	3.084253	11.16864	1.956012	6.595615	16.42202	10.9216	5.377848
AC113554.	0	4.001562	0	1.039293	0	0	1.69936	0	0	0.960699	12.04755	0	0	1.861441	0	0	0	0	0
AC113607.	0	0	0	3.117878	0	0	0	0.926027	0	6.724895	0	3.680313	1.028084	0	0.978006	3.297807	0	0.910133	3.585232
AC113608.	0	6.002343	0.999134	0	0	0	0	0.926027	0	0	0	0	0	0	0	0	0	0	0
AC113615.	9.609075	15.00586	10.99047	11.43222	9.967364	1.141887	9.346483	0	0	6.724895	0	4.600391	3.084253	7.445762	6.846041	4.946711	5.131881	11.83173	15.23724
AC113935.	137.4098	141.0551	132.8848	129.9116	114.0709	119.8981	73.0725	95.38078	151.7474	108.559	107.2232	104.8889	85.331	105.1714	109.5367	118.7211	144.7191	122.868	87.83818
AC114271.	19.21815	7.002733	17.98441	12.47151	26.57964	22.83774	13.59488	28.70684	50.19337	49.95636	34.93789	21.1618	19.5336	7.445762	18.58211	14.01568	30.79129	16.3824	20.61508
AC114284.	16.33543	15.00586	10.99047	11.43222	4.42994	26.2634	28.03945	10.1863	0	5.764196	6.023774	26.68227	23.64594	13.9608	28.36217	9.06897	1.026376	16.3824	21.51139
AC114296.	2.882723	6.002343	0.999134	1.039293	0	1.141887	5.947762	4.630135	1.167288	10.56769	1.204755	3.680313	3.084253	5.584322	4.890029	4.946711	6.158258	2.7304	3.585232
AC114316.	3.84363	1.00039	0	0	0	0	0.84968	0	0	0	0	1.840156	0	5.584322	0	0.824452	0	0.910133	0
AC114316.	10.56998	19.00742	19.98267	5.196464	11.07485	6.851322	8.496802	18.52054	0	6.724895	2.40951	13.80117	11.30893	13.03008	10.75806	8.244518	6.158258	3.640534	1.792616
AC114321.	0	0	1.998267	0	1.107485	1.141887	2.549041	0	0	0	0	0	0	0	0.978006	0	1.026376	0	3.585232
AC114324.	2.882723	0	0	0	0	0	0	0	0	0	0	0.920078	0	4.653602	0	0	0	0	0
AC114341.	9.609075	15.00586	8.992203	18.70727	9.967364	9.135096	21.24201	21.29862	9.338302	15.37119	27.70936	23.92203	14.39318	21.40657	16.6261	34.62698	24.63303	9.101334	27.78555
AC114400.	0	7.002733	1.998267	1.039293	1.107485	0	0	1.852054	0	0	0	1.840156	0	0.93072	8.802052	0	0	0	0.896308
AC114401.	0	0	0	1.039293	0	1.141887	0	0	0	0	1.204755	0	0	0	0	0.824452	1.026376	0	0

AC114402.	33.63176	77.03006	85.9255	51.96464	44.2994	30.83095	83.26866	59.26573	0	41.31007	54.21397	49.68422	61.68506	86.55699	79.21847	30.50472	11.29014	75.54107	53.77848
AC114402.	1.921815	3.001171	3.996535	0	5.537425	0	0.84968	3.704108	0	0.960699	4.81902	5.520469	0	0.93072	1.956012	6.595615	0	5.4608	7.170464
AC114480.	24.02269	20.00781	8.992203	15.58939	14.3973	12.56076	23.79105	5.556162	49.02608	23.05678	21.68559	11.96102	41.12337	14.89152	14.67009	7.420066	14.36927	26.39387	19.71877
AC114482.	0	0	0	4.157171	0	0	0	0	0	0	1.204755	0	0	0	1.956012	0	0	0	0
AC114485.	0	0	0.999134	0	0	0	0	0	0	0	0	0	0	0	0	0	0	0	0.896308
AC114488.	8.648168	4.001562	3.996535	2.078586	5.537425	2.283774	5.947762	12.96438	4.669151	12.48909	25.29985	15.64133	5.140422	3.722881	17.6041	4.122259	13.34289	1.820267	6.274156
AC114488.	540.9909	994.3881	1355.824	861.5737	1755.364	513.8492	763.0129	888.0599	1321.37	1227.774	1670.995	718.5811	822.4675	510.0347	610.2756	346.2698	504.9771	363.1432	500.1398
AC114488.	5.765445	5.001952	20.98181	8.314342	27.68712	3.425661	6.797442	10.1863	5.836439	2.882098	19.27608	9.200782	9.252759	7.445762	8.802052	5.771163	7.184634	14.56213	6.274156
AC114489.	11.53089	16.00625	10.99047	29.1002	8.859879	13.70264	48.43177	17.59451	21.01118	9.606993	2.40951	20.24172	13.3651	23.26801	19.56012	11.54233	24.63303	31.85467	27.78555
AC114490.	1204.978	942.3678	1193.965	1448.774	1146.247	773.0575	1385.828	893.6161	1405.414	916.5071	1097.532	1468.445	1561.66	973.5334	930.0835	1008.305	1109.513	1535.395	1182.23
AC114490.	0	0	0	0	0	0	0.84968	0	0	0.960699	0	0.920078	2.056169	0	0	0	0	0.910133	0
AC114491.	6.726353	28.01093	11.9896	10.39293	8.859879	23.97963	11.89552	2.778081	30.34948	18.25329	9.638039	15.64133	9.252759	16.75297	13.69208	18.13794	16.42202	14.56213	10.7557
AC114495.	193.1424	194.0757	289.7488	190.1906	234.7868	168.9993	264.2506	273.178	63.03354	226.725	296.3697	221.7389	253.9368	147.0538	208.3152	141.8057	131.3762	225.7131	168.5059
AC114498.	0	0	0	0	0	0	0	0	0	0	0	0	0	0	0	0	0	1.820267	0.896308
AC114501.	0	3.001171	1.998267	2.078586	1.107485	1.141887	0.84968	2.778081	5.836439	0	0	3.680313	0	2.792161	1.956012	4.946711	6.158258	0	3.585232
AC114501.	13.45271	21.0082	8.992203	51.96464	29.90209	4.567548	25.49041	13.89041	39.68778	31.70308	15.66181	27.60235	30.84253	20.47585	28.36217	21.43575	24.63303	8.191201	15.23724
AC114501.	0	1.00039	0	0	0	0	6.797442	0	0	0	0	0	0	0	0	0	0	0	0
AC114546.	11.53089	6.002343	0	24.94303	8.859879	2.283774	5.947762	0.926027	7.003726	1.921399	16.86657	3.680313	10.28084	11.16864	6.846041	5.771163	2.052753	9.101334	6.274156
AC114546.	2.882723	0	1.998267	3.117878	1.107485	0	5.947762	9.26027	0	0	0	0.920078	2.056169	1.861441	6.846041	1.648904	0	0.910133	0
AC114546.	0	0	0	0	0	11.41887	0	0.926027	0	0	1.204755	4.600391	0	0	0	0.824452	1.026376	0	0
AC114550.	0	0	0	2.078586	0	1.141887	0.84968	0	0	0	0	0	0	0	0	1.648904	1.026376	0	1.792616
AC114550.	0	0	0	0	0	0	0	0	0	0	0	0.920078	0	0	0	0.824452	1.026376	0	0
AC114550.	0.960908	0	2.997401	0	0	0	0.84968	0	0	0	0	0	0	0	0	0.824452	0	0	0
AC114684.	3.84363	0	0	0	0	2.283774	0.84968	0	0	0.960699	2.40951	0	2.056169	5.584322	5.868035	0	1.026376	0	0
AC114689.	0	0	0.999134	0	0	0	2.549041	0	0	0	0	0.920078	0	0	0	0	0	3.640534	0
AC114689.	0	0	0	0	0	0	0	0	0	0	0	0	4.112337	0	0	0.824452	0	0	0
AC114689.	1.921815	5.001952	7.993069	7.275049	0	1.141887	13.59488	0	0	3.842797	3.614265	1.840156	7.196591	0.93072	1.956012	8.244518	16.42202	0.910133	8.963079
AC114728.	10.56998	17.00664	5.994802	5.196464	15.50479	17.12831	5.098081	8.334243	7.003726	6.724895	12.04755	7.360626	11.30893	12.09936	5.868035	11.54233	4.105505	9.101334	2.688924
AC114730.	0	0	0	0	0	0	0	0	0	0	0	1.840156	0	0	0	0.824452	0	0	0
AC114730.	6.726353	8.003124	8.992203	6.235757	26.57964	4.567548	5.098081	6.482189	10.50559	6.724895	6.023774	10.12086	9.252759	7.445762	6.846041	5.771163	5.131881	8.191201	4.48154
AC114730.	13.45271	13.00508	4.995668	1.039293	4.42994	21.69585	0.84968	13.89041	46.69151	37.46727	71.08054	34.04289	6.168506	6.515042	16.6261	1.648904	12.31652	25.48374	15.23724
AC114737.	1.921815	7.002733	3.996535	5.196464	9.967364	5.709435	2.549041	1.852054	2.334575	1.921399	10.84279	1.840156	2.056169	2.792161	1.956012	9.06897	2.052753	0	4.48154
AC114737.	0	2.000781	0	1.039293	0	0	0	8.334243	0	0	0	1.840156	0	0	0.978006	0	1.026376	0.910133	0
AC114744.	37.47539	34.01327	32.97141	10.39293	17.71976	43.39171	28.88913	30.55889	15.17474	18.25329	38.55216	46.00391	28.78636	49.32818	58.68035	37.10033	5.131881	51.8776	24.20031
AC114752.	0	0	0.999134	0	2.21497	1.141887	3.398721	0	0	0	0	0	1.028084	0	0	0.824452	0	0	0
AC114755.	0	0	0.999134	0	0	0	0	0	0	0	1.204755	0	0	0	0	0.824452	0	0	0
AC114755.	0	3.001171	0	2.078586	0	0	0.84968	1.852054	0	0	0	0.920078	0	0	0.978006	0.824452	0	0.910133	0
AC114760.	0	0	0	0	0	0	0	0	0	0	0	0	5.140422	0	0	0	0	0	0
AC114760.	59.57627	33.01288	75.93416	49.88605	102.9961	44.53359	96.01387	114.8274	7.003726	4.803496	12.04755	84.6472	112.0612	71.66546	68.46041	18.13794	4.105505	70.08027	35.85232
AC114763.	13.45271	31.0121	34.96968	11.43222	22.1497	21.69585	29.73881	27.78081	29.18219	16.33189	25.29985	21.1618	35.98295	44.67457	42.05425	12.36678	13.34289	31.85467	35.85232
AC114763.	0	0	1.998267	0	0	0	0	0	0	0.960699	0	0	0	0	0	0.824452	0	0	0
AC114774.	0.960908	0	0	0	0	4.567548	2.549041	5.556162	0	0.960699	0	0.920078	0	0	0	0.824452	1.026376	2.7304	0.896308
AC114776.	0	0	0.999134	0	0	0	0	0	0	0	0	0	1.028084	0	0	0	2.052753	0	0
AC114781.	3.84363	0	0.999134	0	1.107485	0	1.69936	0.926027	0	11.52839	1.204755	4.600391	4.112337	0	7.824047	9.06897	6.158258	1.820267	8.066772
AC114781.	10.56998	7.002733	7.993069	8.314342	14.3973	5.709435	5.098081	17.59451	11.67288	11.52839	1.204755	7.360626	13.3651	5.584322	15.64809	23.08465	8.21101	19.1128	14.34093
AC114781.	5.765445	9.003514	14.987	12.47151	3.322455	10.27698	11.04584	22.22465	1.167288	9.606993	9.638039	8.280704	12.33701	11.16864	16.6261	23.9091	11.29014	15.47227	18.82247
AC114781.	0	0	0	2.078586	0	0	0	0	0	0	0	0	4.112337	0	0	0.824452	0	0	0
AC114801.	0.960908	4.001562	3.996535	5.196464	0	0	4.248401	0.926027	0	0	0	0.920078	4.112337	0	0.978006	0.824452	0	0.910133	11.652
AC114803.	15.37452	7.002733	4.995668	6.235757	5.537425	7.993209	12.7452	19.44657	16.34203	12.48909	19.27608	19.32164	3.084253	7.445762	11.73607	20.6113	25.65941	6.370934	15.23724
AC114808.	0	0	0.999134	1.039293	0	0	0	0	0	0	0	2.760235	0	0	0.978006	0.824452	1.026376	0.910133	0
AC114808.	2.882723	2.000781	0	1.039293	3.322455	5.709435	10.19616	0.926027	0	5.764196	0	4.600391	6.168506	9.307203	7.824047	0	1.026376	7.281067	4.48154
AC114810.	74.95079	129.0504	77.93243	81.06484	128.4683	119.8981	76.47122	123.1616	121.3979	157.5547	184.3275	90.16767	89.44334	89.34915	67.4824	80.79628	106.7431	41.86614	49.29694
AC114811.	377.6367	503.1964	577.4993	312.8271	437.4565	385.9578	518.3049	587.1011	299.9929	493.7994	268.6603	487.6415	485.2558	602.176	545.7272	427.8905	521.3991	583.3955	564.674
AC114812.	0	0	0	0	0	0	0	0	11.67288	0	0	0	0	0	0	0	0	0	0
AC114814.	0	0	0	0	0	1.141887	0	0	0	0	0	0	0	0	0	0	0	3.640534	0
AC114814.	6.726353	0	0	0	0	0	0	3.704108	0	0.960699	2.40951	0.920078	6.168506	0	0.978006	4.946711	4.105505	6.370934	2.688924
AC114876.	0.960908	0	0.999134	0	0	1.141887	0.84968	0	0	0	4.81902	0	0	0.93072	0	0.824452	1.026376	0.910133	0
AC114877.	7.68726	11.00429	38.96621	13.51081	44.2994	23.97963	32.28785	49.07943	7.003726	24.01748	32.52838	16.56141	21.58977	13.9608	14.67009	19.78684	10.26376	29.12427	31.37078
AC114878.	0	1.00039	4.995668	1.039293	0	1.141887	1.69936	8.334243	0	1.921399	0	4.600391	2.056169	0.93072	0	0.824452	0	0	0
AC114878.	15.37452	13.00508	17.98441	10.39293	23.25718	4.567548	16.9936	29.63287	4.669151	0	14.45706	23.00196	20.56169	18.61441	9.780058	21.43575	14.36927	20.02294	6.274156
AC114912.	0	0	0	0	0	4.567548	0.84968	0	0	0	0	0	0	0	0	0.824452	0	0	0
AC114939.	26.90541	25.00976	58.94889	68.59332	29.90209	21.69585	92.61515	33.33697	21.01118	28.82098	12.04755	46.00391	29.81445	33.50593	50.8563	53.58937	28.73854	53.69787	70.80833

AC114947.	0.960908	1.00039	0.999134	1.039293	5.537425	0	0.84968	0	3.501863	2.882098	4.81902	3.680313	6.168506	0.93072	0	0	0	2.7304	3.585232
AC114947.	1627.777	4294.676	3527.941	3704.039	2954.77	2402.53	1707.008	3134.602	1538.485	3791.88	2715.517	1989.209	2091.124	2701.881	2730.592	2492.318	3010.362	1842.11	2326.815
AC114956.	68.22443	62.02421	66.94196	87.30059	29.90209	47.95925	46.73241	59.26573	66.5354	63.40615	60.23774	61.64524	88.41526	53.98178	94.86656	46.1693	39.0023	51.8776	65.43048
AC114956.	25.9445	32.01249	18.98354	20.78586	17.71976	6.851322	14.44456	16.66849	31.51677	20.17468	24.0951	28.52243	44.20763	21.40657	12.71408	21.43575	18.47477	18.20267	31.37078
AC114956.	98.01257	85.03319	91.9203	82.10413	67.55658	99.34417	105.3603	98.15887	28.01491	98.95202	78.30907	96.60821	121.314	89.34915	77.26246	92.3386	101.6112	141.9808	138.0314
AC114964.	0	4.001562	0.999134	0	0	0	0	0	0	0	0	0	0	0	0	0	0	0	0
AC114967.	0	0	0	0	0	1.141887	0.84968	12.96438	0	0	0	0.920078	4.112337	0	4.890029	1.648904	0	0	0
AC114980.	278.6632	261.1019	324.7184	205.78	361.0401	203.2559	254.9041	265.7698	211.2791	287.2491	236.132	288.9046	322.8185	329.475	320.7859	75.84957	166.273	205.6902	164.9207
AC114982.	8.648168	22.00859	11.9896	3.117878	12.18233	2.283774	25.49041	10.1863	0	0	14.45706	10.12086	22.61786	14.89152	23.47214	1.648904	3.079129	8.191201	17.02985
AC114982.	0	0	0.999134	10.39293	6.64491	0	11.89552	0	0	0	0	0	0	3.722881	2.934017	0	1.026376	0	0
AC115085.	3.84363	4.001562	7.993069	58.2004	9.967364	5.709435	31.43817	8.334243	1.167288	3.842797	4.81902	26.68227	16.44935	22.33729	12.71408	26.38246	15.39564	27.304	31.37078
AC115099.	1.921815	7.002733	3.996535	1.039293	6.64491	1.141887	5.947762	3.704108	7.003726	1.921399	3.614265	2.760235	4.112337	3.722881	0.978006	0.824452	8.21101	6.370934	7.170464
AC115102.	4.804538	11.00429	18.98354	4.157171	7.752394	9.135096	24.64073	19.44657	5.836439	15.37119	9.638039	12.8811	19.5336	25.12945	22.49413	20.6113	1.026376	28.21414	14.34093
AC115220.	2.882723	1.00039	0.999134	0	7.752394	2.283774	1.69936	4.630135	11.67288	0.960699	0	2.760235	2.056169	0.93072	0	0	5.131881	7.281067	2.688924
AC115223.	87.44259	77.03006	70.93849	53.00393	37.65449	60.52001	56.92858	62.04381	80.54285	45.15286	42.16642	48.76415	68.88165	92.14131	82.15249	72.55176	99.5585	89.19307	43.91909
AC115284.	170.0806	201.0785	162.8588	155.8939	296.806	235.2287	134.2495	131.4958	226.4538	183.4936	193.9655	147.2125	159.3531	79.11123	108.5586	132.7367	151.9037	134.6997	140.7203
AC115285.	20.17906	26.01015	31.97228	30.13949	7.752394	20.55397	6.797442	5.556162	4.669151	14.41049	13.2523	11.04094	14.39318	17.68369	8.802052	29.68027	17.4484	16.3824	22.4077
AC115522.	136.4489	192.075	155.8649	141.3438	85.27634	135.8846	141.0469	101.863	73.53913	94.14853	92.76613	121.4503	99.72418	139.608	123.2287	169.0126	161.1411	116.4971	143.4093
AC115618.	45.16265	39.01523	19.98267	23.90373	67.55658	29.68906	41.63433	24.0767	45.52422	67.24895	126.4993	59.80509	46.2638	48.39746	25.42815	25.55801	28.73854	30.0344	44.8154
AC115618.	936.8848	1008.394	1152.001	1041.371	1058.756	1007.144	1131.774	1027.89	569.6364	1060.612	1045.727	1122.495	1195.662	1058.229	897.8093	962.1353	926.8178	1045.743	960.8421
AC115618.	492.9456	575.2245	634.4499	569.5324	630.1589	586.9299	546.3444	648.2189	911.6571	825.2407	820.4381	608.1717	712.4625	613.3447	549.6393	460.8686	497.7925	576.1145	517.1697
AC115621.	1.921815	10.0039	19.98267	2.078586	6.64491	1.141887	28.88913	5.556162	9.338302	31.70308	18.07132	8.280704	7.196591	6.515042	4.890029	3.297807	5.131881	3.640534	0
AC115622.	4.804538	6.002343	10.99047	6.235757	13.28982	6.851322	5.947762	9.26027	9.338302	6.724895	7.228529	10.12086	6.168506	13.03008	3.912023	6.595615	2.052753	7.281067	11.652
AC115837.	70.14625	34.01327	37.96708	39.49313	42.08443	36.54038	45.88273	43.52327	54.86252	33.62447	59.03299	25.76219	32.8987	33.50593	32.27419	39.57369	53.37157	36.40534	35.85232
AC115837.	0	1.00039	4.995668	5.196464	0	1.141887	1.69936	0	0	0	0	0	3.084253	8.376483	0.978006	4.122259	0	0.910133	0.896308
AC115989.	84.55986	97.03787	82.92809	61.31827	166.1227	139.3102	82.41898	133.3479	93.38302	25.93888	54.21397	76.36649	90.47142	33.50593	17.6041	89.0408	48.23968	98.29441	112.0385
AC115989.	4.804538	20.00781	20.98181	23.90373	8.859879	4.567548	23.79105	20.37259	0	5.764196	2.40951	35.88305	22.61786	26.99089	11.73607	29.68027	17.4484	26.39387	18.82247
AC115990.	0.960908	0	0	1.039293	0	1.141887	0.84968	5.556162	0	0.960699	0	2.760235	1.028084	0.93072	0.978006	0	0	5.4608	0.896308
AC116003.	3.84363	1.00039	0.999134	0	0	1.141887	0	0	0	0	0	1.840156	1.028084	0.93072	0.978006	0	4.105505	2.7304	2.688924
AC116003.	0.960908	0	0	8.314342	0	1.141887	0.84968	0	0	0	4.81902	0.920078	2.056169	0	0.978006	0.824452	0	0	0
AC116003.	3.84363	8.003124	2.997401	0	2.21497	4.567548	11.04584	3.704108	7.003726	5.764196	10.84279	0.920078	9.252759	0.93072	0.978006	4.122259	3.079129	0.910133	0.896308
AC116021.	10.56998	1.00039	2.997401	1.039293	0	2.283774	3.398721	0	1.167288	0.960699	13.2523	7.360626	11.30893	6.515042	5.868035	8.244518	8.21101	1.820267	8.066772
AC116025.	12.4918	1.00039	16.98527	6.235757	8.859879	14.84453	18.69297	7.408216	25.68033	0.960699	4.81902	11.96102	2.056169	13.9608	8.802052	30.50472	26.68578	14.56213	2.688924
AC116025.	0	4.001562	0	3.117878	0	1.141887	3.398721	0	0	0.960699	1.204755	0.920078	2.056169	3.722881	0.978006	2.473355	3.079129	5.4608	1.792616
AC116035.	0	0	0	0	0	0	0	3.704108	0	0	0	0	0	0.93072	0	0	2.052753	0	0
AC116036.	14.41361	3.001171	15.98614	4.157171	0	27.40529	16.9936	16.66849	11.67288	9.606993	8.433284	3.680313	6.168506	14.89152	11.73607	5.771163	11.29014	10.01147	7.170464
AC116036.	38.4363	24.00937	29.97401	42.611	21.04221	25.12151	11.04584	48.15341	23.34575	41.31007	56.62348	20.24172	27.75828	22.33729	18.58211	20.6113	30.79129	12.74187	22.4077
AC116038.	0	0	0	8.314342	0	0	0	0	0	2.882098	0	0	0	0	0.978006	0	0	0.910133	0
AC116049.	3.84363	0	0	0	0	0	0	0	0	0.960699	9.638039	2.760235	0	0	0	2.473355	1.026376	1.820267	0
AC116050.	614.9808	924.3608	882.235	1055.921	258.044	578.9367	1022.165	902.8764	1177.793	693.6249	810.8	1151.938	1191.55	742.7148	744.2624	871.4456	1157.752	527.8774	579.0149
AC116050.	3.84363	18.00703	1.998267	6.235757	1.107485	3.425661	13.59488	22.22465	8.171014	1.921399	1.204755	17.48149	17.47743	0	10.75806	16.48904	1.026376	1.820267	4.48154
AC116096.	2.882723	7.002733	3.996535	35.33595	1.107485	28.54718	9.346483	4.630135	0	11.52839	21.68559	3.680313	5.140422	5.584322	3.912023	12.36678	16.42202	7.281067	5.377848
AC116158.	134.5271	157.0613	169.8527	408.4421	132.8982	124.4657	217.5181	163.9068	70.03726	46.11356	72.28529	191.3763	132.6229	145.1924	129.0968	176.4327	187.8269	127.4187	186.4321
AC116158.	4.804538	4.001562	3.996535	17.66798	8.859879	13.70264	5.947762	3.704108	7.003726	0	0	7.360626	16.44935	11.16864	3.912023	23.9091	9.237386	8.191201	14.34093
AC116165.	0.960908	0	0	0	0	0	1.69936	3.704108	0	0	0	0	0	0.93072	0	0	0	0	0
AC116312.	15.37452	6.002343	12.98874	10.39293	5.537425	2.283774	10.19616	12.96438	24.51304	3.842797	12.04755	10.12086	11.30893	4.653602	7.824047	3.297807	11.29014	0.910133	0
AC116337.	0	0	0	0	0	0	0	0	0	0	0	0	0	0	0	0	3.079129	0	0
AC116337.	0.960908	0	0	0	0	2.283774	1.69936	0.926027	0	2.882098	0	0	1.028084	3.722881	0	1.648904	0	0.910133	0.896308
AC116337.	9.609075	1.00039	8.992203	5.196464	21.04221	6.851322	8.496802	7.408216	22.17847	8.646293	19.27608	4.600391	14.39318	7.445762	6.846041	9.06897	12.31652	10.9216	5.377848
AC116345.	3492.899	884.3451	1851.395	1731.462	2113.081	714.8213	1585.503	2125.232	2712.777	1596.682	1609.553	2243.151	2181.595	1853.995	1918.847	1608.506	1602.173	1338.806	707.187
AC116345.	0	5.001952	0	0	0	0	3.398721	0	0	0.960699	9.638039	0	0	0.93072	0	4.946711	7.184634	0	0.896308
AC116345.	0.960908	10.0039	17.98441	15.58939	4.42994	21.69585	5.947762	16.66849	18.6766	26.89958	8.433284	2.760235	1.028084	17.68369	7.824047	15.66458	33.87042	4.550667	8.066772
AC116347.	19.21815	120.0469	144.8744	41.57171	39.86946	109.6212	13.59488	141.6821	108.5578	184.4543	220.4701	22.08188	57.57272	20.47585	36.18622	27.20691	41.05505	221.1624	103.9717
AC116348.	1.921815	3.001171	0	4.157171	8.859879	1.141887	0	0.926027	8.171014	13.44979	12.04755	0.920078	2.056169	4.653602	1.956012	0.824452	3.079129	5.4608	10.7557
AC116348.	1.921815	4.001562	10.99047	6.235757	4.42994	5.709435	5.098081	0.926027	1.167288	0.960699	1.204755	3.680313	4.112337	4.653602	3.912023	1.648904	2.052753	5.4608	7.170464
AC116348.	0	0	0	1.039293	0	0	0.84968	0	4.669151	0	0	4.600391	0	0.93072	0	0.824452	0	0	0
AC116348.	1.921815	0	0	0	0	0	0	0	2.334575	0	0	0	0	0	0	0	0	0	0
AC116351.	230.6178	509.1987	432.6249	224.4872	290.161	309.4514	311.8326	246.3232	254.4687	330.4805	289.1412	242.9007	316.65	172.1833	233.7434	329.7807	332.5459	235.7246	314.6041
AC116351.	25.9445	44.01718	103.9099	4.157171	70.87903	54.81058	14.44456	33.33697	135.4054	59.56335	78.30907	25.76219	33.92678	32.57521	29.34017	45.34485	63.63533	70.08027	86.94187
AC116353.	16.33543	10.0039	3.996535	1.039293	0	15.98642	4.248401	5.556162	21.01118	0.960699	0	8.280704	17.47743	5.584322	12.71408	5.771163	0	0	7.170464
AC116353.	0	0	0.999134	0	0	0	0	0	0	0	0	0	0	0	0	0	1.026376	9.101334	0.896308
AC116353.	0	0	0	0	0	0	0.84968	2.778081	0	0	0	0.920078	0	3.722881	0	3.297807	0	0	0

AC116353.	126.8398	34.01327	69.93936	75.86837	85.27634	41.10793	20.39233	65.74792	109.725	98.95202	81.92333	99.36845	86.35909	134.9544	123.2287	176.4327	199.117	275.7704	207.0471
AC116366.	1475.954	1277.499	1680.543	1260.662	1449.698	1238.947	1581.255	1619.621	568.4691	1667.774	1389.082	1654.301	1808.4	1344.891	1447.449	1168.248	1125.935	1542.676	1428.715
AC116366.	5.765445	0	0	10.39293	1.107485	2.283774	0	0	11.67288	0.960699	0	5.520469	6.168506	3.722881	1.956012	4.122259	1.026376	10.01147	4.48154
AC116366.	25.9445	24.00937	28.97488	29.1002	8.859879	9.135096	23.79105	22.22465	4.669151	13.44979	14.45706	29.4425	35.98295	13.9608	23.47214	15.66458	12.31652	33.67494	34.0597
AC116407.	56.69354	100.039	68.94022	85.22201	35.43952	92.49285	124.903	67.59997	40.85507	104.7162	134.9325	49.68422	76.07824	80.04195	60.63636	79.97183	72.87271	70.99041	103.0754
AC116407.	220.0478	260.1015	297.7418	83.14342	261.3664	259.2084	183.5309	225.9506	525.2795	437.1182	620.4488	342.2691	230.2909	281.0775	240.5894	197.044	260.6996	382.256	248.2773
AC116407.	1.921815	3.001171	4.995668	1.039293	9.967364	2.283774	0.84968	7.408216	4.669151	0	22.89034	7.360626	1.028084	5.584322	0.978006	1.648904	1.026376	1.820267	0.896308
AC116407.	45.16265	18.00703	20.98181	30.13949	48.72934	31.97284	30.58849	40.74519	52.52795	31.70308	68.67103	37.72321	17.47743	26.06017	29.34017	20.6113	15.39564	48.23707	35.85232
AC116424.	1.921815	1.00039	2.997401	8.314342	0	0	5.098081	0.926027	0	2.882098	0	10.12086	4.112337	2.792161	1.956012	12.36678	5.131881	11.83173	2.688924
AC116447.	46.12356	85.03319	73.93589	55.08252	60.91167	135.8846	90.91579	64.82189	57.1971	93.18783	81.92333	112.2495	77.10633	69.80402	80.19648	70.90286	83.13648	72.81067	70.80833
AC116456.	0	0	7.993069	0	0	0	0	0.926027	0	0	0	0	0	0.93072	0	0	0	0.910133	1.792616
AC116456.	8.648168	0	3.996535	0	15.50479	5.709435	10.19616	0.926027	1.167288	0	1.204755	3.680313	1.028084	2.792161	3.912023	4.946711	0	1.820267	0.896308
AC116533.	491.0237	362.1413	342.7028	234.8802	414.1994	423.6401	307.5842	413.9341	541.6215	403.4937	352.9932	315.5868	352.6329	460.7066	431.3006	369.3544	435.1835	564.2827	281.4407
AC116535.	332.474	253.0988	395.6569	326.3379	291.2685	232.9449	267.6493	312.9971	295.3238	260.3495	181.918	299.0254	341.324	312.722	319.8079	293.5049	236.0665	335.8392	345.9749
AC116535.	3.84363	0	0	1.039293	0	0	0	0.926027	0	2.882098	0	1.840156	0	0	0	4.946711	3.079129	2.7304	2.688924
AC116552.	37.47539	61.02382	67.94109	33.25737	28.79461	28.54718	61.17698	44.4493	47.8588	38.42797	44.57593	57.04485	47.29188	74.45762	81.17448	42.8715	51.31881	63.70934	67.2231
AC116562.	0	0	0	0	0	1.141887	1.69936	0	0	0	0	0.920078	0	0	0	3.297807	1.026376	0	0
AC116562.	0	0	0	0	0	0	0	0	0	0	4.81902	0.920078	1.028084	0	0	0.824452	0	0	0
AC116562.	32.67086	25.00976	64.94369	47.80747	37.65449	65.08756	21.24201	90.75065	35.01863	148.9084	175.8942	26.68227	24.67402	22.33729	45.96627	23.9091	33.87042	37.31547	22.4077
AC116563.	2.882723	12.00469	16.98527	7.275049	1.107485	3.425661	27.18977	8.334243	4.669151	5.764196	1.204755	3.680313	6.168506	23.26801	9.780058	6.595615	15.39564	8.191201	4.48154
AC116565.	290.1941	417.1628	275.7609	320.1022	330.0305	481.8763	285.4926	293.5506	653.6811	451.5286	648.1581	342.2691	252.9088	335.99	325.6759	295.9782	348.9679	196.5888	316.3967
AC116565.	660.1435	679.2651	550.5226	750.3694	324.4931	691.9835	783.4052	544.5039	318.6696	351.6159	420.4595	667.0567	664.1425	700.8324	651.3519	704.0819	629.1686	665.3075	815.6402
AC116609.	53.81082	17.00664	5.994802	10.39293	4.42994	20.55397	43.33369	5.556162	31.51677	41.31007	12.04755	5.520469	8.224675	40.95169	29.34017	9.893422	21.5539	15.47227	17.92616
AC116609.	5.765445	3.001171	3.996535	7.275049	3.322455	3.425661	3.398721	1.852054	1.167288	5.764196	0	4.600391	6.168506	6.515042	0.978006	0.824452	0	8.191201	4.48154
AC116612.	0	1.00039	0	0	0	0	0	0	0	0	0	0.920078	0	0	0	0	0	0	0.896308
AC116614.	142.2143	67.02616	158.8623	101.8507	270.2263	148.4453	110.4584	130.5698	385.205	186.3757	284.3222	268.6628	245.7122	146.1231	134.9648	75.02512	91.34749	131.9693	77.97879
AC116616.	2.882723	3.001171	0.999134	6.235757	0	9.135096	11.04584	1.852054	5.836439	0	1.204755	6.440548	0	3.722881	7.824047	6.595615	4.105505	0	7.170464
AC116618.	72.06806	68.02655	61.94629	62.35757	79.73891	54.81058	103.661	25.92876	21.01118	59.56335	56.62348	79.12673	101.7804	17.68369	15.64809	39.57369	52.34519	62.79921	61.84525
AC116631.	0.960908	3.001171	7.993069	6.235757	5.537425	6.851322	2.549041	12.96438	9.338302	1.921399	0	0	5.140422	3.722881	4.890029	0.824452	4.105505	4.550667	11.652
AC116634.	0	0	0	0	0	0	0	0	0	0	0	1.840156	0	0	0	0	0	0.910133	0.896308
AC116651.	20.17906	43.01679	12.98874	25.98232	66.4491	25.12151	18.69297	31.48492	109.725	42.27077	25.29985	42.3236	34.95487	59.5661	39.12023	17.31349	11.29014	21.8432	9.859387
AC116667.	958.9857	850.3319	962.1657	658.9116	1000.059	1109.914	633.8615	1172.35	824.1051	976.0704	742.129	1286.269	1172.016	692.4559	811.7448	636.4768	533.7157	832.7721	651.6159
AC116667.	33.63176	13.00508	23.97921	58.2004	32.11706	62.80379	12.7452	60.19176	31.51677	54.75986	43.37118	41.40352	22.61786	42.81313	51.83431	33.80253	47.21331	56.42827	36.74863
AC116903.	7.68726	10.0039	20.98181	12.47151	19.93473	5.709435	14.44456	6.482189	0	3.842797	10.84279	31.28266	31.87062	13.9608	16.6261	10.71787	9.237386	11.83173	11.652
AC116913.	432.4084	430.1679	523.546	319.0629	481.7559	601.7745	360.2644	598.2135	547.4579	543.7558	562.6205	457.2789	448.2448	453.2608	445.9707	229.1976	262.7523	513.3152	342.3896
AC116914.	0	0	1.998267	2.078586	1.107485	1.141887	4.248401	0.926027	0	0	0	0	0	3.722881	0.978006	3.297807	2.052753	1.820267	0
AC116914.	370.9103	277.1082	253.78	258.7839	322.2781	350.5593	200.5245	364.8547	172.7586	258.4281	244.5652	335.8286	284.7794	239.1951	347.1921	302.5738	227.8555	242.0955	163.128
AC117373.	0	0	0	0	0	0	1.141887	0	0	0	0	0	0	1.028084	0	0	0	1.026376	0	0
AC117377.	0.960908	8.003124	2.997401	6.235757	4.42994	5.709435	5.098081	6.482189	12.84017	11.52839	13.2523	2.760235	2.056169	5.584322	2.934017	0	0	0	0.896308
AC117382.	1.921815	8.003124	12.98874	5.196464	7.752394	5.709435	1.69936	8.334243	12.84017	4.803496	37.3474	2.760235	8.224675	6.515042	5.868035	5.771163	5.131881	1.820267	2.688924
AC117382.	15.37452	12.00469	18.98354	18.70727	26.57964	0	12.7452	28.70684	10.50559	29.78168	16.86657	23.92203	18.50552	22.33729	26.40616	10.71787	23.60665	11.83173	31.37078
AC117383.	12.4918	11.00429	3.996535	4.157171	12.18233	9.135096	24.64073	6.482189	0	0	2.40951	11.96102	14.39318	5.584322	1.956012	10.71787	15.39564	15.47227	13.44462
AC117383.	13.45271	8.003124	7.993069	15.58939	2.21497	4.567548	11.89552	6.482189	4.669151	3.842797	2.40951	9.200782	5.140422	18.61441	3.912023	3.297807	9.237386	9.101334	3.585232
AC117386.	4.804538	0	0	1.039293	0	0	3.398721	0	0	0.960699	0	0	1.028084	0	0.978006	2.473355	1.026376	2.7304	0
AC117386.	9.609075	2.000781	8.992203	4.157171	1.107485	3.425661	2.549041	5.556162	11.67288	4.803496	0	5.520469	7.196591	1.861441	5.868035	9.06897	10.26376	1.820267	4.48154
AC117392.	0.960908	0	0	6.235757	1.107485	0	0.84968	0	0	0	0	3.680313	0	0.93072	0	0	0	0	0.896308
AC117394.	5.765445	0	0.999134	6.235757	1.107485	0	0.84968	0	0	10.56769	7.228529	3.680313	2.056169	2.792161	1.956012	1.648904	0	1.820267	7.170464
AC117395.	0	10.0039	4.995668	6.235757	9.967364	2.283774	1.69936	0	0	8.646293	13.2523	1.840156	2.056169	0.93072	0.978006	4.946711	12.31652	0.910133	0.896308
AC117395.	67.26353	62.02421	74.93502	97.69352	49.83682	70.79699	50.98081	47.22738	59.53167	72.05244	86.74235	74.52634	83.27483	57.70466	67.4824	89.0408	78.0046	54.608	60.05263
AC117395.	14.41361	9.003514	41.96361	16.62868	17.71976	10.27698	20.39233	9.26027	17.50932	21.13538	8.433284	19.32164	22.61786	17.68369	8.802052	15.66458	17.4484	22.75334	32.26709
AC117402.	21.13997	25.00976	52.95408	58.2004	48.72934	13.70264	55.22922	49.07943	26.84762	36.50657	13.2523	33.12282	58.60081	26.99089	23.47214	28.03136	36.94955	50.96747	73.49725
AC117409.	0.960908	6.002343	5.994802	6.235757	0	6.851322	3.398721	5.556162	1.167288	0.960699	2.40951	0	1.028084	3.722881	0	3.297807	2.052753	10.9216	2.688924
AC117422.	0.960908	1.00039	0	0	3.322455	0	1.69936	1.852054	2.334575	0	0	0.920078	1.028084	2.792161	0	1.648904	0	0	0.896308
AC117430.	0	0	0	0	0	0	0	0	0	0	0	1.840156	0	0	0	0	0	0	0
AC117440.	0	8.003124	0.999134	0	0	0	3.398721	0.926027	0	0	0	0	0	0	3.912023	0	0	0	3.585232
AC117457.	0.960908	0	0	0	0	0	0.84968	0	0	0	0	0	0	0	0	1.648904	0	0	0
AC117462.	1.921815	1.00039	14.987	0	0	3.425661	5.098081	7.408216	5.836439	0	0	0	1.028084	7.445762	5.868035	0	0	0.910133	0.896308
AC117465.	0	0	0	0	0	0	0	0	0	1.921399	0	1.840156	0	0	0	0	0	0	0
AC117489.	214.2824	148.0578	287.7505	151.7367	112.9635	107.3374	220.9169	179.6492	249.7996	221.9215	184.3275	172.0546	211.7854	250.3638	223.9633	105.5298	129.3234	174.7456	128.172
AC117490.	3.84363	3.001171	4.995668	2.078586	4.42994	1.141887	0	0	7.003726	1.921399	3.614265	0.920078	1.028084	7.445762	5.868035	2.473355	2.052753	10.01147	0.896308
AC117490.	85.52077	64.02499	55.95149	44.68959	66.4491	68.51322	53.52986	49.07943	137.74	55.72056	80.71858	47.84407	68.88165	48.39746	44.98827	37.10033	28.73854	40.04587	34.95601
AC117498.	17.29634	7.002733	10.99047	4.157171	22.1497	17.12831	10.19616	14.81643	28.01491	14.41049	24.0951	12.8811	8.224675	0.93072	7.824047	6.595615	20.52753	4.550667	3.585232

AC117498.	16.33543	24.00937	13.98787	14.5501	1.107485	11.41887	10.19616	8.334243	16.34203	16.33189	3.614265	32.20274	19.5336	1.861441	11.73607	7.420066	7.184634	19.1128	18.82247
AC117500.	0	1.00039	0.999134	0	0	2.283774	2.549041	0	0	0	0	0	6.168506	0	0	0	0	3.640534	0
AC117500.	27.86632	5.001952	21.98094	0	5.537425	18.27019	15.29424	15.74246	22.17847	25.93888	16.86657	10.12086	18.50552	12.09936	4.890029	4.122259	7.184634	29.12427	33.16339
AC117500.	13.45271	9.003514	10.99047	11.43222	65.34161	6.851322	29.73881	9.26027	16.34203	11.52839	4.81902	26.68227	18.50552	15.82225	17.6041	4.122259	19.50115	10.9216	19.71877
AC117500.	0.960908	1.00039	1.998267	0	9.967364	0	3.398721	0	0	0	2.40951	0.920078	3.084253	0	0	0	0	0	1.792616
AC117500.	0	0	0	0	0	0	0	0	0	0	1.204755	0	2.056169	0	0	0	0	0	0
AC117500.	1.921815	15.00586	4.995668	6.235757	0	0	2.549041	17.59451	0	0	4.81902	11.96102	18.50552	1.861441	12.71408	3.297807	2.052753	1.820267	6.274156
AC117503.	0	1.00039	0	1.039293	1.107485	0	1.69936	0	0	0	0	0.920078	1.028084	0	0	0	0	0.910133	0.896308
AC117503.	49.00628	93.03631	117.8978	138.2259	105.2111	75.36454	130.0011	88.8986	22.17847	72.05244	49.39495	172.0546	185.0552	108.8943	107.5806	122.0189	143.6927	114.6768	93.21603
AC117503.	20.17906	33.01288	39.96535	39.49313	35.43952	22.83774	68.8241	38.89314	28.01491	34.58517	36.14265	53.36454	49.34805	36.29809	48.90029	30.50472	36.94955	44.59654	32.26709
AC117503.	101.8562	93.03631	124.8917	118.4794	112.9635	69.65511	148.694	105.5671	28.01491	86.46293	62.64725	127.8909	119.2578	71.66546	108.5586	116.2477	63.63533	114.6768	147.8908
AC117505.	0	0	3.996535	4.157171	0	1.141887	1.69936	0	0	0	0	0	1.028084	0	0	0	0	0	0
AC117513.	6.726353	9.003514	17.98441	9.353635	19.93473	18.27019	24.64073	12.03835	0	4.803496	9.638039	32.20274	39.06721	34.43665	39.12023	6.595615	12.31652	33.67494	25.09662
AC117522.	0	2.000781	0	0	0	0	0	0	0	0	0	0	0	0	0	0	0	0	0
AC117529.	0	0	0	2.078586	0	4.567548	3.398721	0	0	0	9.638039	4.600391	0	3.722881	0	0.824452	3.079129	0.910133	0
AC117529.	1.921815	19.00742	7.993069	9.353635	24.36467	1.141887	9.346483	0	10.50559	9.606993	8.433284	3.680313	1.028084	7.445762	8.802052	10.71787	1.026376	0.910133	2.688924
AC117532.	0	0	0	1.039293	4.42994	1.141887	1.69936	0	1.167288	0	0	4.600391	2.056169	1.861441	11.73607	4.946711	2.052753	0	6.274156
AC117834.	9.609075	19.00742	13.98787	6.235757	19.93473	12.56076	14.44456	15.74246	2.334575	10.56769	4.81902	23.92203	26.73019	13.03008	21.51613	14.84013	3.079129	15.47227	7.170464
AC117944.	2.882723	6.002343	8.992203	1.039293	2.21497	2.283774	8.496802	3.704108	1.167288	0	0	0	0	5.140422	0.93072	9.780058	1.648904	1.026376	0.910133	3.585232
AC117945.	0.960908	6.002343	0	0	0	1.141887	1.69936	0	2.334575	0.960699	0	0.920078	0	0	0	0	3.079129	1.820267	0.896308
AC117945.	0	0	3.996535	0	0	0	0	0	0	0	0	0	0	0	0	0	0	0	0
AC117947.	0	0	0	0	2.21497	1.141887	0	2.778081	0	0	0	2.760235	0	0	0	0.824452	0	2.7304	1.792616
AC118282.	9.609075	14.00547	4.995668	0	25.47215	5.709435	4.248401	0.926027	8.171014	1.921399	0	14.72125	16.44935	10.23792	8.802052	5.771163	6.158258	7.281067	4.48154
AC118282.	3.84363	0	0.999134	4.157171	3.322455	0	0.84968	0.926027	0	0	0	0	3.084253	0	0	0	0	0	0
AC118282.	0	0	0	0	0	0	0	0	0	0	0	0	0	0	0	0	3.079129	0	0
AC118282.	0	0	0	0	5.537425	0	0	0	0	0	0	0.920078	0	0.93072	0	0	0	0.910133	0.896308
AC118344.	131.6443	201.0785	197.8285	189.1513	222.6045	154.1547	237.0608	244.4711	29.18219	184.4543	120.4755	253.9416	273.4704	166.5989	219.0733	181.3794	156.0092	172.0152	230.3511
AC118344.	2166.846	1472.575	2017.251	2517.167	1396.538	1513	2119.103	1678.887	561.4654	1586.114	1290.292	1697.544	2162.061	1379.327	1530.579	2160.064	2319.61	1914.921	1800.683
AC118345.	0	0	0	0	1.107485	0	0.84968	0	0	0	0	3.680313	4.112337	0	0.978006	0.824452	0	0	0
AC118465.	13.45271	6.002343	21.98094	12.47151	9.967364	2.283774	38.23561	12.96438	1.167288	11.52839	1.204755	0.920078	16.44935	5.584322	6.846041	29.68027	18.47477	14.56213	17.02985
AC118469.	0	1.00039	0	0	0	0	0	0	0	0	0	0	0	0	0	0	0	0.910133	0
AC118553.	24.02269	7.002733	27.97574	10.39293	22.1497	23.97963	12.7452	11.11232	30.34948	5.764196	25.29985	13.80117	33.92678	11.16864	12.71408	20.6113	11.29014	18.20267	25.99293
AC118553.	112.4262	244.0953	366.6821	83.14342	203.7772	173.5668	318.6301	281.5122	5.836439	244.9783	191.556	201.4971	159.3531	176.8369	154.5249	97.28532	70.81996	110.1261	161.3354
AC118555.	0.960908	4.001562	0	1.039293	0	0	0.84968	2.778081	1.167288	0	2.40951	1.840156	0	5.584322	1.956012	0.824452	6.158258	1.820267	0
AC118658.	15.37452	15.00586	20.98181	22.86444	8.859879	29.68906	13.59488	24.0767	4.669151	39.38867	54.21397	16.56141	24.67402	20.47585	24.45015	21.43575	3.079129	20.02294	12.54831
AC118658.	16.33543	1.00039	6.993936	5.196464	2.21497	5.709435	5.098081	20.37259	29.18219	0	4.81902	0	6.168506	2.792161	6.846041	4.122259	0	10.01147	5.377848
AC118754.	18.25724	14.00547	5.994802	5.196464	33.22455	35.3985	10.19616	18.52054	17.50932	22.09608	43.37118	40.48344	16.44935	13.03008	14.67009	9.893422	24.63303	33.67494	42.12647
AC118754.	0	0	0.999134	4.157171	0	0	0.84968	0	0	0	0	0.920078	6.168506	0	0	0.824452	2.052753	1.820267	0
AC118755.	0	3.001171	0	0	4.42994	0	0	0	1.167288	0.960699	0	0.920078	1.028084	0.93072	0	0	0	0	1.792616
AC118755.	0.960908	4.001562	1.998267	0	11.07485	0	0.84968	3.704108	0	1.921399	0	0	0	0	0	0	1.026376	0	0
AC118757.	2.882723	3.001171	8.992203	9.353635	16.61227	12.56076	0	4.630135	4.669151	19.21399	15.66181	2.760235	2.056169	0	5.868035	5.771163	7.184634	6.370934	5.377848
AC118758.	27.86632	15.00586	19.98267	18.70727	16.61227	17.12831	14.44456	25.00273	18.6766	11.52839	13.2523	34.04289	20.56169	20.47585	15.64809	21.43575	41.05505	9.101334	14.34093
AC118758.	93.20803	51.01991	119.896	45.72888	77.52394	93.63473	68.8241	78.7123	220.6174	107.5983	218.0606	107.6492	90.47142	73.5269	81.17448	77.49847	103.664	88.28294	83.35664
AC118942.	7.68726	1.00039	0	2.078586	6.64491	0	0.84968	3.704108	0	3.842797	0	0	2.056169	0	4.890029	0	0	0	0
AC119044.	21.13997	28.01093	48.95755	46.76817	29.90209	60.52001	18.69297	61.11778	129.5689	38.42797	77.10431	70.84602	46.2638	51.18962	46.94428	71.72731	39.0023	74.63094	51.98586
AC119150.	0	2.000781	0	0	3.322455	0	0.84968	0	0	0.960699	0	0	2.056169	0	3.912023	0	0	0	0
AC119396.	98.97348	90.03514	76.93329	30.13949	75.30897	77.64832	67.12474	153.7205	51.36066	70.13105	44.57593	96.60821	82.24675	41.88241	89.97654	40.39814	27.71216	86.46267	59.15632
AC119396.	68.22443	28.01093	33.97054	49.88605	34.33203	20.55397	33.13753	43.52327	24.51304	14.41049	22.89034	58.88501	47.29188	19.54513	37.16422	14.01568	27.71216	18.20267	14.34093
AC119403.	53.81082	117.0457	78.93156	174.6012	94.13622	110.763	68.8241	74.08216	187.9333	146.0263	161.4372	58.88501	92.52759	63.28898	75.30645	101.4076	115.9805	54.608	52.88217
AC119403.	4.804538	6.002343	0	14.5501	2.21497	5.709435	3.398721	0	0	7.685594	6.023774	6.440548	1.028084	0	3.912023	11.54233	0	11.83173	4.48154
AC119424.	88.40349	61.02382	80.92983	48.84676	102.9961	22.83774	155.4915	37.96711	23.34575	25.93888	16.86657	41.40352	93.55568	148.9152	108.5586	78.32292	73.89909	83.73227	89.63079
AC119427.	8.648168	1.00039	8.992203	0	1.107485	9.135096	0	12.96438	7.003726	4.803496	6.023774	0.920078	0	0.93072	5.868035	0	8.21101	5.4608	0.896308
AC119428.	0.960908	0	0	0	0	0	0	0	1.167288	0	0	0	0	0	0	0	0	0	0.896308
AC119428.	112.4262	208.0812	110.9038	149.6582	167.2302	145.0196	50.13113	248.1752	60.69896	83.58083	66.26152	149.9728	123.3701	97.72563	105.6246	182.2039	93.40024	222.9827	127.2757
AC119501.	0	0	0	0	0	0	0	0	0	0	0	0	1.028084	0	0	0	0	0	0.896308
AC119618.	0	0	0	0	0	0	0	0	0	1.921399	0	0	0	0	0	0.824452	1.026376	0	0
AC119674.	49.00628	28.01093	92.91943	28.0609	32.11706	10.27698	28.88913	53.70957	72.37184	54.75986	116.8612	33.12282	31.87062	40.95169	44.98827	17.31349	28.73854	49.1472	39.43755
AC119674.	24.02269	30.01171	47.95842	10.39293	43.19191	41.10793	38.23561	34.263	29.18219	43.23147	30.11887	18.40156	21.58977	25.12945	19.56012	6.595615	31.81766	31.85467	18.82247
AC119677.	0	1.00039	0	0	0	1.141887	0	0	4.669151	0	0	0	0	0	0	0	0	0	0
AC119744.	0	1.00039	3.996535	2.078586	1.107485	3.425661	6.797442	11.11232	0	21.13538	4.81902	0	9.252759	0	7.824047	0	0	0	0.896308
AC119749.	0	0	0	0	0	0	0	0	0	0	0	0	0	0	0.978006	4.122259	0	0	0.896308
AC119751.	0.960908	2.000781	0	0	8.859879	0	0	0	0	0.960699	1.204755	0	2.056169	0.93072	0	0	0	0	0

AC119751.	12.4918	7.002733	7.993069	14.5501	27.68712	3.425661	10.19616	27.78081	8.171014	2.882098	3.614265	28.52243	8.224675	19.54513	13.69208	11.54233	9.237386	15.47227	11.652
AC119751.	9.609075	2.000781	9.991337	0	4.42994	0	1.69936	0	0	0	0	3.680313	10.28084	6.515042	0	1.648904	0	0.910133	1.792616
AC119751.	3.84363	0	0	0	0	0	0	0	0	0	0	0	0	0	0	0	0	0.910133	0
AC119751.	1.921815	27.01054	50.95582	18.70727	89.70628	34.25661	33.13753	4.630135	0	9.606993	2.40951	94.76806	65.7974	57.70466	43.03226	53.58937	82.1101	64.61947	25.99293
AC119800.	6.726353	4.001562	0	6.235757	7.752394	7.993209	11.89552	1.852054	14.00745	8.646293	19.27608	0.920078	8.224675	7.445762	8.802052	5.771163	2.052753	12.74187	0
AC119868.	2.882723	1.00039	0.999134	0	0	0	1.69936	0	10.50559	3.842797	1.204755	1.840156	0	14.89152	1.956012	0.824452	0	0	0.896308
AC119868.	13.45271	14.00547	27.97574	1.039293	14.3973	31.97284	16.14392	19.44657	109.725	30.74238	44.57593	6.440548	5.140422	10.23792	1.956012	0.824452	0	0	3.585232
AC120024.	24.9836	13.00508	7.993069	6.235757	4.42994	7.993209	10.19616	0.926027	8.171014	9.606993	39.75691	6.440548	9.252759	0.93072	4.890029	2.473355	10.26376	3.640534	14.34093
AC120024.	0	0	0	0	0	0	0.84968	0	0	0	0	0	0	0.93072	0	0	0	0.910133	0
AC120024.	0	0	0	0	0	0	0	3.704108	0	0	0	0	0	0	0	0	0	0	0
AC120024.	3.84363	2.000781	0	2.078586	0	0	5.098081	3.704108	0	0	1.204755	1.840156	4.112337	0	3.912023	3.297807	0	5.4608	3.585232
AC120036.	1.921815	7.002733	0.999134	2.078586	3.322455	3.425661	5.098081	0.926027	4.669151	5.764196	4.81902	9.200782	2.056169	7.445762	4.890029	9.06897	4.105505	3.640534	4.48154
AC120036.	0	2.000781	0	0	0	2.283774	5.098081	0	1.167288	11.52839	1.204755	7.360626	1.028084	1.861441	0.978006	0	0	0.910133	3.585232
AC120036.	5.765445	4.001562	25.97748	0	0	10.27698	4.248401	6.482189	5.836439	21.13538	49.39495	4.600391	2.056169	10.23792	2.934017	3.297807	13.34289	5.4608	6.274156
AC120036.	44.20175	56.02186	28.97488	42.611	33.22455	51.38492	58.62794	57.41368	30.34948	57.64196	46.98544	69.00587	32.8987	53.05106	28.36217	47.81821	23.60665	40.04587	45.71171
AC120036.	980.1257	1099.429	997.1354	1251.308	1000.059	1498.156	1237.984	1019.556	981.689	1117.293	1155.36	862.1133	1031.169	903.7294	816.6349	998.4112	1165.963	558.8219	855.9741
AC120042.	4.804538	11.00429	3.996535	13.51081	11.07485	4.567548	11.04584	3.704108	9.338302	10.56769	3.614265	7.360626	15.42127	12.09936	7.824047	10.71787	15.39564	7.281067	8.066772
AC120042.	3.84363	6.002343	1.998267	7.275049	15.50479	11.41887	8.496802	8.334243	0	9.606993	1.204755	9.200782	5.140422	9.307203	3.912023	3.297807	5.131881	10.01147	2.688924
AC120045.	0	2.000781	0	1.039293	1.107485	14.84453	3.398721	2.778081	0	4.803496	0	1.840156	0	3.722881	5.868035	1.648904	6.158258	0	6.274156
AC120049.	436.252	406.1585	455.605	495.7427	233.6793	417.9306	529.3508	280.5862	239.294	271.8779	299.984	424.1561	409.1776	402.0712	351.1041	553.2072	536.7948	363.1432	441.8798
AC120053.	468.9229	522.2038	663.4248	318.0236	557.0649	639.4567	387.4542	566.7285	869.6294	716.6816	767.4289	472.0001	473.9469	416.9627	390.2243	355.3387	424.9198	518.776	480.4211
AC120057.	19.21815	20.00781	14.987	11.43222	6.64491	15.98642	7.647122	11.11232	28.01491	10.56769	24.0951	34.04289	20.56169	30.71377	13.69208	90.6897	49.26606	51.8776	34.95601
AC120057.	0.960908	6.002343	0	0	0	1.141887	0.84968	1.852054	0	0	0	9.200782	14.39318	0.93072	0	0	0	0	0
AC120057.	28.82723	37.01445	25.97748	34.29666	11.07485	49.10114	30.58849	6.482189	44.35693	31.70308	40.96167	34.04289	18.50552	33.50593	35.20821	32.97807	48.23968	37.31547	41.23017
AC120057.	19.21815	13.00508	6.993936	13.51081	12.18233	10.27698	2.549041	15.74246	75.8737	38.42797	65.05676	36.80313	35.98295	26.99089	21.51613	9.893422	27.71216	30.0344	36.74863
AC120114.	185.4552	127.0496	197.8285	142.3831	93.02873	129.0332	151.2431	105.5671	53.69524	85.50223	63.85201	103.9688	138.7914	153.5689	140.8328	145.1035	136.508	144.7112	120.1053
AC120114.	855.2077	1168.456	1499.7	814.8055	1130.742	969.4621	1121.578	1472.383	305.8294	1043.319	745.7433	1114.215	1059.955	1149.44	1222.507	746.1289	684.593	796.3667	590.6669
AC120114.	1101.2	1517.592	2222.073	979.0138	1482.922	1362.271	1248.18	1566.838	954.8414	1820.525	1628.829	1311.111	1316.976	1643.652	1879.727	1121.254	1157.752	891.0206	737.6614
AC120193.	88.40349	109.0426	92.91943	164.2083	36.547	66.22945	197.1258	87.04654	32.68406	64.36685	18.07132	50.6043	77.10633	122.8551	95.84457	123.6678	99.5585	85.55254	121.0016
AC120338.	1.921815	1.00039	0	0	4.42994	3.425661	0	1.852054	0	0	1.204755	10.12086	2.056169	0.93072	0	2.473355	7.184634	22.75334	33.16339
AC120349.	1.921815	0	1.998267	5.196464	14.3973	15.98642	22.09169	21.29862	12.84017	17.29259	39.75691	23.00196	15.42127	64.2197	63.57038	4.946711	13.34289	38.2256	34.0597
AC120349.	0	0	0	0	0	0	0	0	0	0	1.204755	1.840156	0	0	0	0	0	0	0
AC120349.	23.06178	34.01327	29.97401	27.02161	44.2994	20.55397	50.98081	37.96711	14.00745	2.882098	14.45706	39.56336	55.51656	29.78305	18.58211	30.50472	4.105505	21.8432	17.92616
AC120498.	0	0	0.999134	0	0	0	0	6.482189	1.167288	3.842797	0	0	2.056169	0	0	0	0	0	0.896308
AC120498.	15.37452	4.001562	0.999134	3.117878	0	7.993209	4.248401	1.852054	24.51304	1.921399	1.204755	0.920078	1.028084	1.861441	2.934017	4.946711	21.5539	0.910133	3.585232
AC120498.	0	2.000781	5.994802	6.235757	24.36467	12.56076	0	0	15.17474	7.685594	24.0951	20.24172	20.56169	20.47585	0.978006	4.946711	1.026376	24.5736	12.54831
AC120498.	0	0	0	0	1.107485	0	0	0	14.00745	0	0	0	0	0	0	9.893422	1.026376	0	0
AC120498.	0.960908	5.001952	0	0	0	0	0	0	0	0.960699	0	0	0	0	0	0	0	0.910133	1.792616
AC120498.	0	0	0	0	0	0	0	0	0	1.921399	0	5.520469	2.056169	0.93072	0.978006	0	0	0	5.377848
AC120498.	0	6.002343	0	0	0	0	0	0	4.669151	5.764196	0	0	0	0.93072	0	0	4.105505	0	2.688924
AC120498.	40.35812	8.003124	34.96968	17.66798	8.859879	35.3985	31.43817	87.97257	32.68406	59.56335	55.41872	47.84407	40.09529	48.39746	51.83431	21.43575	13.34289	22.75334	25.99293
AC121158.	1.921815	1.00039	0.999134	0	0	0	0.84968	3.704108	0	0	0	0	1.028084	0.93072	0	0	2.052753	0.910133	0.896308
AC121247.	79.75532	24.00937	86.92463	102.89	31.00958	74.22266	78.17058	161.1287	113.2269	143.1442	92.76613	99.36845	121.314	92.14131	98.77859	89.86525	74.92547	73.72081	77.08248
AC121247.	2.882723	5.001952	0.999134	11.43222	1.107485	2.283774	3.398721	0.926027	0	1.921399	1.204755	1.840156	2.056169	17.68369	0.978006	3.297807	11.29014	0.910133	6.274156
AC121251.	0	8.003124	6.993936	0	0	2.283774	0.84968	7.408216	15.17474	2.882098	8.433284	2.760235	11.30893	3.722881	1.956012	0.824452	2.052753	2.7304	0.896308
AC121320.	0.960908	0	0	0	1.107485	0	3.398721	0	4.669151	3.842797	0	9.200782	0	3.722881	0	3.297807	1.026376	1.820267	0
AC121333.	0	0	0	2.078586	0	0	0	0	0	0	0	0	0	0	0	0	0	0	0
AC121334.	0.960908	0	0.999134	0	0	1.141887	0.84968	3.704108	1.167288	3.842797	0	0.920078	1.028084	9.307203	4.890029	0	0	0	0
AC121338.	0.960908	0	0	0	0	0	0	0	0	0	9.638039	0	0	0	0	6.595615	0	0	0.896308
AC121338.	4.804538	6.002343	20.98181	3.117878	21.04221	5.709435	13.59488	1.852054	16.34203	15.37119	2.40951	2.760235	1.028084	3.722881	13.69208	11.54233	5.131881	5.4608	7.170464
AC121342.	0	0	0	0	1.107485	0	0.84968	0.926027	0	0	0	0.920078	0	4.653602	0.978006	0	4.105505	0	1.792616
AC121493.	636.1208	916.3576	531.5391	760.7623	691.0706	810.7398	662.7506	592.6573	1546.656	836.769	793.9335	731.4622	772.0914	560.2936	627.8797	628.2323	867.2879	747.2195	714.3574
AC121757.	37.47539	34.01327	48.95755	46.76817	54.26676	38.82416	59.47762	36.11505	8.171014	7.685594	14.45706	55.20469	74.02207	46.53602	78.24047	76.67402	57.47707	89.19307	71.70464
AC121758.	0	2.000781	0	0	9.967364	7.993209	6.797442	0	0	0	6.023774	0	2.056169	0	5.868035	3.297807	3.079129	0.910133	0.896308
AC121761.	238.3051	86.03358	191.8337	133.0295	97.45867	129.0332	238.7601	90.75065	262.6397	174.8473	146.9801	176.655	169.6339	147.0538	118.3387	196.2195	139.5872	341.3	270.685
AC121761.	233.5005	155.0605	246.786	124.7151	155.0479	116.4725	186.08	138.9041	345.5172	236.332	224.0844	180.3353	214.8696	218.7193	192.6671	145.928	157.0356	149.2619	155.0613
AC121764.	0	8.003124	1.998267	0	0	11.41887	2.549041	8.334243	0	0	0	5.520469	2.056169	3.722881	4.890029	0	3.079129	0.910133	0
AC122108.	0	0	0	1.039293	0	0	0	0	0	0.960699	0	0	0	0	0	0	0	0	7.170464
AC122129.	3066.256	2787.088	4231.331	2722.947	3673.527	2025.708	3609.442	2850.311	952.5068	2865.766	2487.819	3937.935	4041.4	2676.752	3107.125	1972.913	2150.258	2945.192	3133.493
AC122133.	0	0	0	0	0	0	0	0	0	0	0	0	0	0	0	0	2.052753	0	0.896308
AC122134.	0	0	0	0	0	0	0	0	4.669151	3.842797	0	0	0	0	0	0	0	0	0
AC122136.	0	0	0	0	0	0	0	0	0	0	0	0	0	0	0	0	2.052753	0	0

AC122138.	0	0	0.999134	0	0	0	0	0	0	0	0	0	0	0	0	5.771163	1.026376	0	0
AC122683.	6.726353	7.002733	12.98874	12.47151	23.25718	5.709435	8.496802	6.482189	0	12.48909	0	8.280704	5.140422	2.792161	0.978006	4.122259	8.21101	7.281067	4.48154
AC122688.	540.9909	1310.511	1035.102	863.6523	1515.039	1618.054	595.6258	1445.528	1132.269	2865.766	2998.635	698.3394	719.6591	979.1178	848.9091	928.3328	1305.551	743.579	1072.881
AC122688.	0.960908	0	0.999134	0	0	1.141887	0	0	0	0	0	1.840156	1.028084	1.861441	1.956012	2.473355	0	3.640534	0.896308
AC122694.	2.882723	0	0	0	0	0	0	0	0	0	0	0	0	0	0	0	0	0	0
AC122707.	0	4.001562	7.993069	4.157171	6.64491	0	1.69936	11.11232	4.669151	0.960699	7.228529	0	0	0.93072	0.978006	2.473355	1.026376	2.7304	4.48154
AC122710.	0.960908	0	0	0	0	0	0	0	9.338302	0	9.638039	0	0	0	0	0	0	0.910133	0
AC122710.	39.39721	67.02616	31.97228	66.51474	13.28982	28.54718	66.27506	37.04108	7.003726	8.646293	16.86657	34.04289	34.95487	45.60529	29.34017	72.55176	23.60665	29.12427	61.84525
AC122710.	4.804538	15.00586	12.98874	14.5501	13.28982	1.141887	1.69936	8.334243	4.669151	14.41049	4.81902	6.440548	9.252759	0.93072	21.51613	17.31349	19.50115	3.640534	14.34093
AC122713.	2.882723	0	0.999134	0	1.107485	0	0.84968	0	0	0	0	1.840156	3.084253	0	0	3.297807	0	0.910133	0.896308
AC122713.	88.40349	25.00976	65.94282	63.39686	13.28982	34.25661	9.346483	55.56162	22.17847	3.842797	12.04755	92.9279	103.8365	66.08114	31.29619	69.25395	62.60895	86.46267	58.26002
AC122714.	0	0	0	0	0	1.141887	0	0	0	0	0	0.920078	0	0	0	0	0	0	0
AC122714.	3.84363	0	9.991337	0	0	4.567548	26.34009	0	22.17847	0.960699	18.07132	6.440548	0	0	0	4.122259	1.026376	0.910133	5.377848
AC122718.	13.45271	26.01015	24.97834	16.62868	34.33203	5.709435	25.49041	17.59451	102.7213	12.48909	36.14265	16.56141	13.3651	20.47585	16.6261	14.84013	36.94955	15.47227	12.54831
AC122718.	16.33543	9.003514	29.97401	23.90373	19.93473	3.425661	34.83689	4.630135	4.669151	1.921399	21.68559	8.280704	21.58977	10.23792	12.71408	9.06897	27.71216	25.48374	6.274156
AC122719.	0	1.00039	0	0	0	0	0	0	0	0	0	0	2.056169	0	0	0	0	0	0
AC122719.	1.921815	0	0	3.117878	1.107485	2.283774	0	0	0	0	1.204755	0	0	0	0	0.824452	2.052753	0	1.792616
AC123567.	0	0	0	0	0	1.141887	0	0	0	7.685594	0	0	1.028084	0	0	6.595615	1.026376	0	0
AC123567.	13.45271	0	6.993936	9.353635	5.537425	15.98642	13.59488	1.852054	7.003726	8.646293	0	3.680313	6.168506	2.792161	11.73607	8.244518	4.105505	2.7304	1.792616
AC123595.	36.51449	29.01132	46.95928	31.17878	36.547	18.27019	43.33369	21.29862	5.836439	22.09608	10.84279	24.84211	20.56169	57.70466	30.31818	32.97807	32.84404	37.31547	36.74863
AC123767.	0	0	0.999134	10.39293	11.07485	3.425661	3.398721	5.556162	4.669151	0	0	2.760235	4.112337	0.93072	1.956012	0	4.105505	0.910133	1.792616
AC123768.	3.84363	2.000781	0.999134	3.117878	0	0	1.69936	0	0	1.921399	0	2.760235	1.028084	0.93072	0	4.946711	7.184634	2.7304	3.585232
AC123768.	78.79442	85.03319	85.9255	63.39686	86.38382	55.95246	70.52346	89.82462	43.18965	90.30573	95.17564	79.12673	66.82548	106.1021	79.21847	44.5204	60.5562	92.83361	77.97879
AC123768.	87.44259	43.01679	72.93676	64.43615	58.6967	59.37812	71.37314	47.22738	28.01491	75.89524	27.70936	69.00587	71.96591	56.77394	63.57038	34.62698	63.63533	68.26001	69.01571
AC123768.	15.37452	1.00039	0	16.62868	32.11706	3.425661	39.08529	8.334243	22.17847	2.882098	3.614265	23.92203	28.78636	1.861441	0	28.03136	20.52753	22.75334	44.8154
AC123769.	15.37452	32.01249	52.95408	51.96464	19.93473	29.68906	33.98721	27.78081	14.00745	11.52839	7.228529	30.36258	20.56169	26.06017	19.56012	29.68027	39.0023	23.66347	21.51139
AC123769.	0	0	0	0	0	0	5.098081	0	0	0	0	6.440548	4.112337	0	7.824047	0	0	0	5.377848
AC123777.	0	4.001562	5.994802	0	0	0	4.248401	0	0	0	0	0	0	0	5.868035	0	0	1.820267	0
AC123786.	1.921815	6.002343	2.997401	0	6.64491	2.283774	3.398721	0	0	3.842797	1.204755	3.680313	3.084253	1.861441	4.890029	0	3.079129	3.640534	2.688924
AC123786.	0	0	0	0	0	0	0	0	16.34203	0.960699	0	0.920078	0	0	0	0	0	0	0
AC123788.	13.45271	7.002733	3.996535	22.86444	5.537425	14.84453	7.647122	7.408216	5.836439	21.13538	0	2.760235	8.224675	18.61441	8.802052	32.15362	12.31652	0.910133	0.896308
AC123788.	0.960908	1.00039	0.999134	0	0	1.141887	2.549041	0.926027	0	1.921399	1.204755	0	1.028084	0	0.978006	0	0	0	0.896308
AC123905.	0	2.000781	0	0	6.64491	2.283774	0.84968	0	0	0	0	0.920078	1.028084	0.93072	3.912023	6.595615	0	1.820267	1.792616
AC123912.	59.57627	43.01679	183.8406	77.94696	53.15928	93.63473	58.62794	60.19176	31.51677	43.23147	49.39495	157.3334	130.5667	85.62627	152.5689	65.13169	61.58258	68.26001	90.5271
AC123912.	5.765445	4.001562	7.993069	4.157171	1.107485	2.283774	1.69936	0	1.167288	5.764196	0	2.760235	3.084253	4.653602	0	3.297807	1.026376	0.910133	1.792616
AC123912.	24.02269	18.00703	29.97401	22.86444	0	37.68227	21.24201	3.704108	16.34203	19.21399	20.48083	23.00196	25.70211	22.33729	20.53812	13.19123	19.50115	11.83173	16.13354
AC123912.	41.31902	35.01367	115.8995	43.6503	7.752394	52.5268	75.62154	16.66849	101.554	65.32755	96.38039	69.92595	79.1625	53.98178	89.97654	28.03136	39.0023	7.281067	41.23017
AC123912.	0	0	0	0	0	5.709435	0	0	11.67288	0	0	0	0	0	0	0	0	0	0.896308
AC124016.	98.97348	165.0644	82.92809	75.86837	130.6832	118.7562	74.77186	92.6027	232.2903	149.8691	197.5798	84.6472	78.13441	89.34915	88.99853	43.69595	92.37386	65.52961	119.209
AC124016.	161.4325	186.0726	200.8259	245.2731	222.6045	219.2423	213.2697	97.23284	240.4613	300.6989	146.9801	126.9708	214.8696	204.7585	226.8974	164.0659	188.8532	150.172	112.9348
AC124017.	7.68726	14.00547	1.998267	48.84676	24.36467	5.709435	0	12.96438	0	0	1.204755	12.8811	5.140422	2.792161	3.912023	1.648904	0	2.7304	4.48154
AC124045.	580.3881	696.2717	720.3754	878.2024	719.8652	713.6794	733.274	559.3203	940.8339	880.0005	936.0945	610.0119	648.7212	671.0493	718.8343	676.875	650.7225	594.3171	563.7777
AC124067.	0.960908	0	0	0	0	0	0	0	0	0	0	0	0	0	0	0.824452	0	0.910133	0
AC124067.	24.9836	5.001952	25.97748	12.47151	1.107485	13.70264	2.549041	25.00273	4.669151	19.21399	1.204755	24.84211	21.58977	22.33729	6.846041	37.10033	18.47477	30.94454	17.92616
AC124067.	0	1.00039	0	0	0	0	0	0	0	0	0	0	1.028084	0	0	0.824452	0	3.640534	0
AC124067.	7.68726	10.0039	19.98267	18.70727	3.322455	7.993209	14.44456	0.926027	25.68033	3.842797	0	8.280704	15.42127	8.376483	16.6261	4.122259	3.079129	21.8432	16.13354
AC124068.	9.609075	6.002343	19.98267	11.43222	5.537425	20.55397	8.496802	4.630135	8.171014	21.13538	61.4425	10.12086	26.73019	10.23792	14.67009	9.893422	8.21101	22.75334	18.82247
AC124069.	13.45271	1.00039	5.994802	1.039293	0	0	4.248401	0	1.167288	0	0	1.840156	1.028084	5.584322	0.978006	3.297807	5.131881	0.910133	5.377848
AC124242.	229.6569	147.0574	194.8311	235.9195	150.6179	156.4385	196.2761	169.4629	128.4017	124.8909	143.3658	111.3295	138.7914	163.8068	188.7551	165.7148	172.4312	211.151	169.4022
AC124242.	15.37452	30.01171	11.9896	25.98232	9.967364	5.709435	29.73881	28.70684	22.17847	11.52839	1.204755	23.00196	3.084253	16.75297	18.58211	32.15362	20.52753	15.47227	14.34093
AC124248.	12.4918	1.00039	8.992203	27.02161	11.07485	7.993209	2.549041	8.334243	16.34203	28.82098	25.29985	3.680313	14.39318	14.89152	16.6261	27.20691	30.79129	52.78774	8.066772
AC124254.	8.648168	2.000781	0.999134	5.196464	0	0	4.248401	4.630135	0	0	4.81902	7.360626	2.056169	0.93072	0	4.122259	10.26376	3.640534	11.652
AC124276.	0	0	0	0	1.107485	0	0	0	0	0	0	0	0	0	0	0.824452	0	0	0
AC124283.	77.83351	68.02655	73.93589	75.86837	127.3608	75.36454	157.1908	108.3452	50.19337	90.30573	81.92333	116.8499	106.9208	117.2708	105.6246	103.0565	85.18923	58.24854	62.74156
AC124283.	40.35812	39.01523	85.9255	57.1611	65.34161	28.54718	97.71323	90.75065	25.68033	75.89524	38.55216	62.56532	63.74123	69.80402	71.39443	70.90286	44.13418	97.38428	71.70464
AC124283.	40.35812	56.02186	57.94975	68.59332	79.73891	29.68906	100.2623	37.96711	21.01118	36.50657	57.82823	66.24563	75.05016	26.99089	47.92229	42.04704	59.52982	61.88907	84.25295
AC124283.	36.51449	29.01132	35.96881	18.70727	43.19191	34.25661	32.28785	41.67122	18.6766	28.82098	42.16642	38.64329	31.87062	34.43665	54.76833	18.96239	15.39564	90.10321	58.26002
AC124287.	53.81082	24.00937	79.93069	25.98232	29.90209	26.2634	64.5757	57.41368	7.003726	26.89958	38.55216	23.00196	34.95487	51.18962	51.83431	59.36053	48.23968	70.99041	41.23017
AC124293.	0	0	0.999134	0	0	0	0	0	0	0	0	0.920078	0	0	0	0	0	0	0
AC124303.	17.29634	9.003514	16.98527	15.58939	5.537425	6.851322	21.24201	0	1.167288	13.44979	14.45706	11.96102	19.5336	12.09936	2.934017	11.54233	2.052753	4.550667	14.34093
AC124303.	54.77173	26.01015	68.94022	38.45383	37.65449	22.83774	175.8838	38.89314	14.00745	9.606993	18.07132	84.6472	76.07824	35.36737	48.90029	41.22259	55.42432	29.12427	55.57109
AC124312.	630.3553	630.246	342.7028	1417.595	427.4892	344.8499	1429.162	463.9395	46.69151	143.1442	55.41872	794.0275	565.4464	716.6546	674.824	847.5365	668.1709	578.8449	938.4344

AC124312.	15.37452	27.01054	35.96881	13.51081	19.93473	11.41887	39.08529	36.11505	10.50559	12.48909	8.433284	26.68227	26.73019	18.61441	41.07624	27.20691	7.184634	21.8432	26.88924
AC124312.	114.348	151.059	101.9116	239.0373	143.973	76.50643	242.1589	146.3123	32.68406	38.42797	30.11887	153.6531	101.7804	147.9845	146.7009	122.0189	106.7431	76.45121	150.5797
AC124312.	9.609075	6.002343	21.98094	28.0609	16.61227	6.851322	16.14392	12.96438	4.669151	8.646293	22.89034	21.1618	5.140422	10.23792	2.934017	17.31349	12.31652	10.9216	16.13354
AC124319.	56.69354	96.03748	113.9012	44.68959	120.7159	109.6212	72.22282	119.4575	191.4352	80.69874	61.4425	117.77	66.82548	100.5178	140.8328	32.97807	39.0023	60.97894	50.19325
AC124319.	320.9431	406.1585	471.5911	355.4381	478.4335	353.985	375.5587	453.7532	602.3205	490.9173	740.9242	471.0801	451.329	371.3574	306.1158	488.8999	542.953	308.5352	421.2647
AC124319.	24.9836	26.01015	49.95668	21.82515	39.86946	35.3985	19.54265	27.78081	21.01118	47.07426	37.3474	14.72125	19.5336	26.06017	15.64809	18.96239	43.1078	14.56213	18.82247
AC124319.	24.9836	54.02108	65.94282	60.27898	125.1458	85.64153	53.52986	101.863	50.19337	83.58083	108.4279	78.20665	54.48847	36.29809	31.29619	56.06272	90.32111	46.4168	25.99293
AC124657.	28.82723	95.03709	51.95495	41.57171	25.47215	3.425661	16.14392	7.408216	14.00745	48.99566	59.03299	5.520469	16.44935	33.50593	21.51613	9.06897	11.29014	27.304	43.91909
AC124784.	0.960908	3.001171	9.991337	7.275049	7.752394	1.141887	6.797442	1.852054	1.167288	0.960699	10.84279	0.920078	12.33701	1.861441	4.890029	4.946711	2.052753	1.820267	1.792616
AC124798.	495.8283	589.23	553.52	548.7466	1070.938	802.7466	355.1663	424.1204	792.5884	976.0704	1026.451	424.1561	391.7001	627.3055	625.9237	703.2574	719.4898	628.9022	584.3928
AC124804.	44.20175	12.00469	25.97748	20.78586	26.57964	20.55397	24.64073	11.11232	53.69524	35.54587	49.39495	58.88501	46.2638	22.33729	34.2302	43.69595	47.21331	30.94454	65.43048
AC124854.	27.86632	26.01015	37.96708	24.94303	33.22455	17.12831	13.59488	45.37532	5.836439	24.01748	8.433284	15.64133	16.44935	34.43665	22.49413	54.41382	48.23968	28.21414	17.02985
AC124856.	1.921815	13.00508	2.997401	4.157171	5.537425	1.141887	0	0	0	0.960699	0	0.920078	1.028084	0.93072	7.824047	1.648904	0	0	0
AC124861.	0	0	1.998267	0	0	6.851322	1.69936	0	1.167288	0	2.40951	2.760235	0	0	0.978006	3.297807	3.079129	5.4608	3.585232
AC124893.	0	0	0	0	1.107485	0	0	0	0	0	0	0	0	1.861441	0	0	0	0	0
AC124916.	0.960908	0	0	5.196464	1.107485	1.141887	0	0	1.167288	0	0	0	0	0	0.978006	1.648904	2.052753	0	0.896308
AC124916.	2.882723	0	0	0	0	1.141887	2.549041	0	12.84017	7.685594	0	0	0	0.93072	0	0	1.026376	0	1.792616
AC124944.	18.25724	5.001952	15.98614	1.039293	9.967364	19.41208	0	15.74246	26.84762	0	20.48083	1.840156	6.168506	13.03008	5.868035	14.84013	15.39564	12.74187	9.859387
AC124944.	228.696	252.0984	333.7106	171.4833	272.4413	334.5729	262.5512	346.3341	386.3722	200.7861	154.2086	265.9026	321.7904	308.0684	374.5762	225.0753	184.7477	319.4568	310.1226
AC124944.	830.2241	765.2987	1211.949	525.8821	746.4448	765.0643	889.6152	966.7722	531.1159	484.1924	485.5162	1052.569	1037.337	998.6629	1061.136	712.3264	692.804	1353.368	1332.81
AC124947.	122.9962	127.0496	55.95149	110.165	84.16885	106.1955	197.9755	75.00819	80.54225	121.0481	98.7899	194.1365	184.0271	139.608	177.0191	134.3856	122.1388	124.6883	222.2844
AC124947.	24.02269	52.0203	17.98441	19.74656	21.04221	59.37812	39.08529	6.482189	43.18965	29.78168	14.45706	70.84602	64.76932	59.5661	52.81231	23.9091	31.81766	24.5736	51.08955
AC125232.	445.8611	411.1605	582.4949	279.5698	249.1841	464.748	424.8401	609.3258	143.5764	645.5899	703.5768	504.2029	498.6209	301.5534	317.8519	511.9846	500.8716	272.1299	303.8484
AC125232.	23.06178	15.00586	25.97748	23.90373	12.18233	10.27698	21.24201	19.44657	1.167288	21.13538	8.433284	17.48149	24.67402	13.9608	18.58211	15.66458	7.184634	17.29253	26.88924
AC125238.	3.84363	0	0	0	0	0	0	0	0	0	0	0.920078	0	5.584322	0	0	0	0.910133	0
AC125238.	0.960908	0	0	0	0	0	0	0	0	0	7.228529	5.520469	0	0	0	0	0	0	0
AC125257.	3331.466	3620.413	2932.457	3558.538	2386.63	3564.971	3241.53	3168.865	3438.83	3516.159	2793.827	2531.135	3086.309	3012.742	3330.11	3197.224	3213.584	2558.385	2424.513
AC125336.	0	0	0	1.039293	0	0	0.84968	0	9.338302	0	0	0.920078	0	0	0	0	0	0	0
AC125421.	0	0	0	0	0	0	0	0	0	0	0	0.920078	0	0	0	0	2.052753	0	0
AC125437.	104.7389	132.0515	111.903	78.98625	120.7159	78.7902	137.6482	190.7616	218.2828	76.85594	77.10431	90.16767	110.005	150.7767	166.261	83.26963	57.47707	89.19307	70.80833
AC125437.	3.84363	2.000781	5.994802	0	0	2.283774	1.69936	0	5.836439	3.842797	13.2523	4.600391	1.028084	0.93072	0	14.01568	5.131881	5.4608	3.585232
AC125494.	35.55358	76.02967	99.91337	27.02161	81.95388	97.0604	67.97442	139.8301	28.01491	119.1267	83.12809	94.76806	77.10633	62.35826	91.93255	43.69595	23.60665	72.81067	40.33386
AC125494.	69.18534	140.0547	105.9082	133.0295	212.6371	236.3706	169.0864	142.6082	308.164	150.8298	228.9034	97.52829	170.662	154.4996	175.063	54.41382	102.6376	74.63094	80.66772
AC125494.	0.960908	1.00039	1.998267	2.078586	16.61227	0	5.098081	1.852054	0	2.882098	15.66181	4.600391	4.112337	2.792161	6.846041	2.473355	4.105505	0	4.48154
AC125603.	0	0	0.999134	4.157171	0	1.141887	0	0	0	0	2.40951	3.680313	0	0.93072	1.956012	0.824452	2.052753	0.910133	0
AC125603.	7.68726	0	4.995668	4.157171	1.107485	0	0.84968	0	9.338302	0.960699	2.40951	0.920078	0	6.515042	0	3.297807	0	0.910133	0
AC125603.	4.804538	10.0039	23.97921	3.117878	8.859879	9.135096	11.04584	19.44657	7.003726	9.606993	0	8.280704	19.5336	9.307203	21.51613	6.595615	23.60665	29.12427	25.09662
AC125603.	21.13997	6.002343	8.992203	20.78586	18.82724	18.27019	28.88913	42.59724	4.669151	8.646293	10.84279	18.40156	15.42127	12.09936	24.45015	19.78684	3.079129	20.02294	30.47447
AC125604.	1.921815	3.001171	3.996535	2.078586	14.3973	9.135096	0.84968	6.482189	2.334575	8.646293	22.89034	5.520469	6.168506	2.792161	1.956012	0	10.26376	4.550667	1.792616
AC125611.	3.84363	4.001562	17.98441	0	38.76197	25.12151	4.248401	9.26027	74.70641	48.03496	33.73314	7.360626	0	9.307203	4.890029	8.244518	4.105505	1.820267	6.274156
AC125611.	7.68726	0	20.98181	7.275049	2.21497	0	9.346483	11.11232	12.84017	7.685594	21.68559	8.280704	2.056169	4.653602	7.824047	17.31349	13.34289	10.01147	4.48154
AC125611.	209.4778	192.075	119.896	258.7839	193.8099	267.2016	126.6024	181.5013	171.5913	164.2796	218.0606	244.7408	231.319	202.897	174.085	224.2509	280.2007	269.3995	293.989
AC125612.	0	0	0	0	0	0	0	2.778081	0	5.764196	3.614265	3.680313	4.112337	5.584322	0	0.824452	0	0.910133	0
AC125613.	31.70995	41.01601	36.96795	10.39293	50.94431	25.12151	55.22922	48.15341	5.836439	19.21399	15.66181	35.88305	30.84253	49.32818	62.59237	14.84013	13.34289	34.58507	50.19325
AC125616.	98.01257	168.0656	109.9047	85.22201	99.67364	132.4589	82.41898	127.7917	217.1155	130.6551	144.5706	105.809	113.0893	141.4695	164.305	57.71163	168.3257	71.90054	130.861
AC125618.	27.86632	30.01171	18.98354	17.66798	12.18233	14.84453	27.18977	9.26027	25.68033	17.29259	19.27608	10.12086	15.42127	23.26801	16.6261	9.893422	6.158258	8.191201	8.066772
AC125634.	0	1.00039	0	1.039293	6.64491	0	0	0	0	6.724895	0	4.600391	1.028084	0	0.978006	1.648904	2.052753	0.910133	0.896308
AC125793.	0	0	0	1.039293	0	0	3.398721	0	0	0	0	0	0	0.93072	0	1.648904	0	0	0
AC125807.	0	1.00039	3.996535	0	1.107485	0	2.549041	0	0	0	2.40951	0	0	0.93072	0	0.824452	1.026376	0	1.792616
AC125807.	1724.829	633.2471	919.203	1213.894	1536.082	1131.61	583.7303	641.7367	642.0083	1045.241	1451.73	1302.831	1303.611	1336.514	1239.133	1975.387	1888.532	2428.236	2086.605
AC126118.	150.8625	162.0633	202.8241	85.22201	198.2398	178.1344	113.8572	241.6931	243.9631	282.4456	206.0131	197.8168	171.6901	174.0447	200.4912	83.26963	73.89909	142.8909	124.5868
AC126120.	0	0	0	0	0	0	0	0	9.338302	0	0	0	6.168506	0	0	0.824452	0	0	0
AC126124.	20.17906	2.000781	1.998267	3.117878	3.322455	5.709435	5.098081	3.704108	2.334575	0	2.40951	11.96102	8.224675	6.515042	0.978006	0.824452	1.026376	3.640534	0
AC126124.	0.960908	2.000781	3.996535	4.157171	0	1.141887	5.098081	3.704108	2.334575	0	0	5.520469	6.168506	2.792161	0	4.122259	0	2.7304	9.859387
AC126175.	17.29634	8.003124	8.992203	1.039293	8.859879	5.709435	18.69297	9.26027	8.171014	15.37119	9.638039	14.72125	26.73019	14.89152	14.67009	13.19123	2.052753	11.83173	10.7557
AC126177.	0	0	0	0	4.42994	0	0.84968	0	0	0	0	0	0	0.93072	0	0	0	0.910133	0.896308
AC126177.	26.90541	17.00664	13.98787	46.76817	25.47215	10.27698	24.64073	10.1863	14.00745	14.41049	12.04755	32.20274	16.44935	10.23792	11.73607	112.9499	52.34519	28.21414	8.963079
AC126177.	0	0	0	0	0	0	3.398721	0	0	0	0	0	0	0.93072	0	0	0	0	0
AC126177.	32.67086	9.003514	19.98267	60.27898	13.28982	21.69585	17.84329	29.63287	8.171014	32.66377	33.73314	45.08383	41.12337	36.29809	14.67009	77.49847	68.76721	31.85467	42.12647
AC126177.	0.960908	0	0	0	0	0	0	0	0	0	0	7.360626	1.028084	7.445762	0	0	1.026376	0	0.896308
AC126177.	291.155	254.0992	139.8787	375.1847	157.2629	358.5525	338.1727	204.652	51.36066	118.166	74.6948	233.6999	260.1053	211.2735	226.8974	362.7588	331.5195	195.6787	333.4266
AC126177.	148.9407	226.0882	72.93676	260.8625	94.13622	182.7019	192.0277	143.5342	7.003726	65.32755	61.4425	150.8928	147.0161	129.3701	106.6026	235.7932	189.8796	113.7667	198.9804

AC126177.	32.67086	31.0121	29.97401	80.02554	37.65449	50.24303	46.73241	64.82189	53.69524	31.70308	4.81902	50.6043	57.57272	23.26801	28.36217	122.8433	61.58258	50.96747	22.4077
AC126178.	1.921815	0	0.999134	0	1.107485	6.851322	0	23.15068	9.338302	9.606993	1.204755	1.840156	4.112337	0.93072	1.956012	0.824452	4.105505	0	0
AC126182.	3.84363	10.0039	2.997401	6.235757	16.61227	1.141887	24.64073	1.852054	0	8.646293	0	1.840156	4.112337	2.792161	2.934017	5.771163	4.105505	1.820267	0.896308
AC126283.	33.63176	56.02186	69.93936	25.98232	17.71976	19.41208	31.43817	32.41095	36.18592	24.97818	55.41872	21.1618	34.95487	62.35826	29.34017	29.68027	52.34519	27.304	34.0597
AC126323.	1.921815	6.002343	13.98787	11.43222	28.79461	1.141887	23.79105	11.11232	7.003726	1.921399	27.70936	12.8811	9.252759	0.93072	18.58211	16.48904	16.42202	1.820267	25.09662
AC126323.	0	1.00039	0	0	0	0	0	0	0	0	7.228529	0	0	0	0	0	0	0	0
AC126323.	0	0	0	0	0	0	0	0	0	0	0	0	0	0	0	0	3.079129	0	0.896308
AC126323.	0	0	0.999134	0	8.859879	0	0.84968	0	0	0	0	0	1.028084	0	0.978006	0.824452	2.052753	0	0.896308
AC126365.	18.25724	13.00508	14.987	5.196464	29.90209	10.27698	2.549041	25.00273	9.338302	28.82098	22.89034	9.200782	17.47743	8.376483	10.75806	13.19123	26.68578	9.101334	15.23724
AC126389.	1.921815	5.001952	1.998267	16.62868	1.107485	0	11.89552	2.778081	1.167288	0.960699	2.40951	0.920078	4.112337	7.445762	7.824047	2.473355	7.184634	5.4608	2.688924
AC126407.	35.55358	46.01796	35.96881	25.98232	93.02873	41.10793	46.73241	34.263	25.68033	20.17468	44.57593	39.56336	25.70211	16.75297	48.90029	25.55801	11.29014	50.96747	59.15632
AC126474.	27.86632	57.02225	38.96621	44.68959	48.72934	73.08077	68.8241	45.37532	5.836439	49.95636	57.82823	54.28462	40.09529	54.9125	55.74633	79.14738	65.68808	65.52961	64.53417
AC126474.	1563.397	1541.602	1465.729	1926.849	1445.268	1932.073	1558.314	1409.413	1624.865	1823.407	1495.101	1336.874	1365.296	1443.547	1397.57	1732.173	1800.264	1589.093	1438.574
AC126544.	173.9243	139.0543	137.8804	56.12181	17.71976	110.763	43.33369	164.8328	30.34948	46.11356	73.49005	44.16376	18.50552	102.3792	134.9648	100.5831	113.9278	113.7667	110.2459
AC126544.	196.986	131.0511	262.7722	111.2043	68.66407	154.1547	15.29424	314.8492	85.212	79.73804	91.56137	77.28657	61.68506	220.5807	196.5792	159.9437	161.1411	116.4971	168.5059
AC126564.	4.804538	4.001562	0.999134	9.353635	4.42994	2.283774	8.496802	0.926027	0	1.921399	0	0	6.168506	2.792161	0.978006	4.122259	4.105505	0	0.896308
AC126603.	3.84363	6.002343	0	7.275049	5.537425	3.425661	0.84968	0	0	1.921399	1.204755	3.680313	1.028084	0.93072	0.978006	0.824452	0	0	1.792616
AC126614.	2.882723	0	5.994802	5.196464	3.322455	0	1.69936	1.852054	1.167288	0	2.40951	1.840156	3.084253	1.861441	7.824047	2.473355	10.26376	15.47227	5.377848
AC126615.	0.960908	0	0.999134	0	0	1.141887	2.549041	0.926027	4.669151	1.921399	4.81902	0	2.056169	0.93072	0	3.297807	1.026376	0.910133	0
AC126615.	1.921815	4.001562	0.999134	0	0	0	0.84968	0	1.167288	5.764196	0	5.520469	4.112337	0.93072	0.978006	1.648904	1.026376	0	3.585232
AC126696.	4.804538	0	2.997401	0	2.21497	0	0	5.556162	7.003726	0	0	0	0	5.584322	0	2.473355	0	0	1.792616
AC126696.	11.53089	0	5.994802	0	4.42994	1.141887	1.69936	1.852054	3.501863	0.960699	7.228529	6.440548	0	10.23792	5.868035	0	0	7.281067	9.859387
AC126696.	7.68726	2.000781	8.992203	4.157171	2.21497	1.141887	10.19616	0.926027	0	1.921399	9.638039	0.920078	5.140422	5.584322	5.868035	7.420066	1.026376	0	8.963079
AC126755.	444.9002	434.1695	273.7626	209.9371	454.0688	403.0861	312.6823	381.5231	596.484	592.7514	1009.585	433.3568	320.7623	369.496	321.7639	403.9814	455.7111	394.0878	561.9851
AC126755.	19.21815	15.00586	31.97228	8.314342	28.79461	26.2634	9.346483	59.26573	30.34948	24.01748	42.16642	14.72125	9.252759	19.54513	5.868035	34.62698	29.76491	10.9216	13.44462
AC126755.	1.921815	2.000781	4.995668	2.078586	5.537425	7.993209	2.549041	6.482189	1.167288	0	3.614265	12.8811	6.168506	13.03008	6.846041	14.01568	9.237386	3.640534	9.859387
AC126755.	0.960908	0	0	0	0	0	0	1.852054	0	0	0	0	0	0	0	0	0	0	1.792616
AC126763.	0.960908	2.000781	0.999134	0	0	0	4.248401	0	9.338302	3.842797	0	1.840156	3.084253	0	2.934017	2.473355	0	0.910133	2.688924
AC126768.	44.20175	38.01484	81.92896	27.02161	56.48173	92.49285	85.8177	52.78354	198.4389	13.44979	49.39495	10.12086	17.47743	87.48771	76.28445	3.297807	0	7.281067	12.54831
AC126768.	34.59267	61.02382	57.94975	8.314342	35.43952	13.70264	25.49041	101.863	23.34575	18.25329	34.93789	52.44446	50.37613	13.03008	20.53812	23.9091	17.4484	18.20267	13.44462
AC126768.	19.21815	16.00625	8.992203	0	0	3.425661	0	11.11232	2.334575	2.882098	0	23.00196	31.87062	0.93072	0.978006	0	1.026376	0	0
AC126773.	0.960908	16.00625	10.99047	7.275049	11.07485	15.98642	1.69936	18.52054	17.50932	26.89958	73.49005	5.520469	2.056169	5.584322	4.890029	6.595615	4.105505	12.74187	17.02985
AC126773.	5.765445	6.002343	8.992203	11.43222	1.107485	21.69585	6.797442	11.11232	44.35693	12.48909	31.32363	7.360626	2.056169	19.54513	32.27419	24.73355	14.36927	15.47227	31.37078
AC126773.	0	0	9.991337	1.039293	0	0	4.248401	0	0	3.842797	0	8.280704	0	4.653602	3.912023	2.473355	0	1.820267	0
AC126773.	108.5826	100.039	123.8926	140.3045	73.094	69.65511	116.4062	130.5698	121.3979	118.166	97.58515	82.80704	132.6229	109.825	122.2507	67.60505	108.7959	121.0477	148.7871
AC126915.	0.960908	0	0	0	0	0	0.84968	5.556162	0	0	0	0	0	0	0	0	0	0	0
AC127002.	14.41361	2.000781	12.98874	5.196464	0	9.135096	5.947762	7.408216	4.669151	0	0	1.840156	1.028084	0	0	0.824452	1.026376	8.191201	5.377848
AC127024.	14.41361	1.00039	19.98267	12.47151	6.64491	3.425661	4.248401	0	1.167288	6.724895	0	1.840156	5.140422	0.93072	6.846041	0.824452	10.26376	2.7304	2.688924
AC127024.	44.20175	21.0082	30.97314	49.88605	50.94431	21.69585	25.49041	15.74246	63.03354	67.24895	25.29985	58.88501	24.67402	32.57521	24.45015	37.10033	47.21331	47.32694	64.53417
AC127024.	9.609075	15.00586	16.98527	29.1002	24.36467	7.993209	9.346483	15.74246	1.167288	11.52839	8.433284	26.68227	30.84253	11.16864	13.69208	34.62698	17.4484	19.1128	23.30401
AC127024.	181.6115	195.0761	183.8406	157.9725	156.1554	189.5532	241.3092	224.0985	143.5764	256.5067	224.0844	149.9728	187.1114	199.1741	211.2493	126.9656	135.4817	210.2408	133.5499
AC127024.	369.9494	310.121	307.7332	352.3202	292.376	342.5661	339.8721	356.5204	158.7511	364.105	238.5415	307.3061	352.6329	370.4267	311.9839	366.0566	356.1526	334.019	288.6112
AC127024.	98.97348	72.02811	71.93762	35.33595	85.27634	116.4725	42.48401	64.82189	317.5023	124.8909	189.1465	51.52438	100.7523	46.53602	51.83431	42.04704	81.08372	40.956	37.64493
AC127024.	0	2.000781	0	5.196464	9.967364	2.283774	0	0.926027	0	1.921399	2.40951	1.840156	1.028084	0	2.934017	2.473355	4.105505	0.910133	1.792616
AC127024.	83.59896	81.03163	114.9004	76.90766	87.49131	50.24303	123.2036	98.15887	14.00745	62.44545	19.27608	127.8909	134.6791	119.1322	114.4267	136.859	58.50345	130.1491	122.7942
AC127029.	0.960908	0	0	0	2.21497	0	0	0	0	0	0	0.920078	1.028084	0	0	0.824452	0	0.910133	0
AC127029.	5.765445	3.001171	6.993936	5.196464	8.859879	10.27698	5.098081	6.482189	0	0	12.04755	1.840156	4.112337	19.54513	21.51613	15.66458	20.52753	1.820267	4.48154
AC127070.	48.04538	50.01952	77.93243	36.37525	52.05179	36.54038	22.94137	93.52873	38.5205	54.75986	26.50461	64.40548	57.57272	40.95169	51.83431	46.99375	67.74083	44.59654	25.99293
AC127070.	39.39721	50.01952	50.95582	36.37525	60.91167	47.95925	51.83049	45.37532	81.71014	56.68126	51.80446	23.00196	65.7974	55.84322	43.03226	46.1693	58.50345	46.4168	51.98586
AC127070.	292.1159	363.1417	365.6829	541.4715	261.3664	415.6469	300.7868	357.4464	311.6658	201.7468	203.6036	305.466	341.324	343.4358	354.0381	511.1601	519.3464	412.2904	375.553
AC127070.	0.960908	0	5.994802	1.039293	2.21497	1.141887	3.398721	0	0	10.56769	0	0.920078	0	1.861441	2.934017	0	0	3.640534	0
AC127070.	178.7288	163.0636	243.7886	349.2024	134.0057	131.317	171.6354	225.9506	112.0596	221.9215	149.3896	169.2944	151.1284	107.0328	182.8871	268.7713	222.7236	243.0056	166.7133
AC127071.	0	0	6.993936	0	0	0	0	0	0	0	0	0	2.056169	0	0	0	0	0	0
AC127071.	0	1.00039	0	0	0	0	0	0	0	0	0	0	0	0	0	0	0	0.910133	0
AC127164.	15.37452	36.01406	20.98181	20.78586	31.00958	14.84453	47.58209	32.41095	19.84389	24.01748	28.91412	23.92203	12.33701	37.22881	17.6041	16.48904	20.52753	1.820267	22.4077
AC127381.	1.921815	0	0	0	0	0	0	0.926027	0	0	0	0	1.028084	3.722881	0	0	0	0	0
AC127455.	0.960908	2.000781	0.999134	3.117878	2.21497	1.141887	1.69936	0	0	0.960699	3.614265	1.840156	0	1.861441	5.868035	1.648904	1.026376	0.910133	1.792616
AC127459.	24.02269	33.01288	6.993936	22.86444	29.90209	23.97963	34.83689	42.59724	26.84762	29.78168	25.29985	13.80117	14.39318	36.29809	35.20821	21.43575	7.184634	19.1128	66.32679
AC127496.	107.6216	59.02304	80.92983	143.4224	43.19191	137.0264	62.02666	36.11505	51.36066	98.95202	142.1611	191.3763	170.662	186.1441	118.3387	133.5612	169.3521	122.868	170.2985
AC127496.	50.9281	15.00586	33.97054	22.86444	11.07485	35.3985	45.03305	22.22465	14.00745	19.21399	27.70936	32.20274	44.20763	20.47585	18.58211	19.78684	27.71216	10.01147	37.64493
AC127496.	0	0	0	12.47151	0	0	0.84968	0.926027	0	0	0	0	5.140422	0	0.978006	0.824452	3.079129	0	0
AC127496.	0	0	0.999134	0	0	0	0.84968	0	0	0	2.40951	6.440548	0	0	0	0	0	0	0.896308

AC127496.	253.6796	122.0476	114.9004	102.89	86.38382	141.594	137.6482	99.08489	175.0932	162.3582	167.4609	151.8129	216.9258	117.2708	103.6686	113.7744	98.53212	83.73227	112.9348
AC127496.	181.6115	117.0457	70.93849	146.5403	98.56616	145.0196	107.0597	106.4931	98.05217	116.2446	116.8612	81.88696	87.38717	97.72563	93.88856	157.4703	166.273	91.92348	83.35664
AC127502.	401.6593	451.1761	230.7999	658.9116	228.1419	660.0107	298.2378	479.682	164.5876	463.057	324.0791	370.7915	316.65	321.0985	356.9721	574.6429	532.6893	597.0475	587.978
AC127502.	2374.403	2007.784	1151.002	1967.381	2007.87	2986.035	1723.152	2341.922	2601.884	2328.735	2173.378	2089.498	2051.028	2109.943	2258.215	2771.807	2656.262	3403.899	2285.585
AC127502.	39.39721	19.00742	11.9896	20.78586	27.68712	29.68906	2.549041	43.52327	108.5578	29.78168	27.70936	37.72321	38.03912	12.09936	27.38416	47.81821	33.87042	15.47227	16.13354
AC127521.	54.77173	34.01327	35.96881	16.62868	7.752394	39.96605	29.73881	86.12051	33.85134	24.97818	40.96167	58.88501	31.87062	24.19873	45.96627	20.6113	43.1078	49.1472	61.84525
AC127522.	4.804538	13.00508	0.999134	7.275049	13.28982	1.141887	5.098081	0	7.003726	6.724895	3.614265	1.840156	4.112337	3.722881	10.75806	14.01568	19.50115	8.191201	12.54831
AC127526.	5.765445	0	1.998267	1.039293	0	0	0.84968	0	0	0	0	0.920078	2.056169	0	0	0	0	0	0
AC127526.	0.960908	2.000781	2.997401	0	0	0	1.69936	0	1.167288	6.724895	4.81902	0.920078	0	5.584322	0	0.824452	1.026376	0.910133	1.792616
AC127526.	0	0	0	1.039293	0	0	0	0	1.167288	7.685594	0	2.760235	0	0	0	3.297807	0	0	0
AC127526.	6.726353	7.002733	14.987	15.58939	13.28982	7.993209	4.248401	1.852054	9.338302	0	6.023774	7.360626	9.252759	5.584322	1.956012	3.297807	2.052753	15.47227	3.585232
AC127537.	2.882723	4.001562	7.993069	0	0	2.283774	2.549041	2.778081	0	0	0	1.840156	5.140422	0.93072	0	0.824452	0	5.4608	2.688924
AC127537.	3.84363	1.00039	6.993936	0	2.21497	0	0	2.778081	0	0	1.204755	0.920078	2.056169	3.722881	0	4.122259	1.026376	0.910133	2.688924
AC127537.	16.33543	41.01601	35.96881	30.13949	23.25718	25.12151	24.64073	8.334243	60.69896	19.21399	21.68559	22.08188	30.84253	15.82225	39.12023	36.27588	13.34289	40.04587	27.78555
AC127540.	11.53089	4.001562	20.98181	23.90373	7.752394	4.567548	37.38593	33.33697	0	6.724895	33.73314	15.64133	26.73019	5.584322	20.53812	9.893422	20.52753	21.8432	17.92616
AC127894.	0	0	0	0	0	0	0	0	0	0	0	0	0	0	0	0	0	1.820267	0
AC128657.	3.84363	0	0	1.039293	0	0	0.84968	0	0	0	0	0	0	0	0	0	1.026376	1.820267	0
AC128685.	0	0	1.998267	0	1.107485	0	0.84968	0	0	0	0	0	1.028084	0	0	0	0	0	0
AC128687.	0	2.000781	0	11.43222	8.859879	0	5.098081	3.704108	0	4.803496	0	4.600391	7.196591	4.653602	1.956012	4.946711	5.131881	0	3.585232
AC128687.	0	10.0039	11.9896	0	0	0	3.398721	12.03835	5.836439	7.685594	4.81902	16.56141	4.112337	0.93072	7.824047	8.244518	4.105505	3.640534	0.896308
AC128688.	1.921815	0	0	0	0	0	0	0	0	0	0	0.920078	1.028084	0	0	0	0	0	0
AC128688.	164.3152	214.0836	141.877	247.3517	146.188	149.5872	202.2239	162.0547	119.0633	181.5722	109.6327	166.5342	165.5216	249.433	217.1173	317.414	290.4645	210.2408	164.9207
AC128689.	97.05166	161.0629	129.8874	221.3694	89.70628	181.56	166.5373	150.0164	110.8923	63.40615	28.91412	135.2515	98.6961	213.1349	180.9311	178.906	192.9587	77.36134	198.0841
AC128707.	22.10087	5.001952	5.994802	2.078586	25.47215	7.993209	19.54265	3.704108	0	5.764196	1.204755	40.48344	20.56169	22.33729	22.49413	4.946711	11.29014	3.640534	6.274156
AC128709.	0	0	3.996535	10.39293	0	3.425661	3.398721	3.704108	0	0	0	1.840156	7.196591	0	0	3.297807	0	0	1.792616
AC128709.	10.56998	4.001562	3.996535	33.25737	8.859879	5.709435	16.9936	31.48492	0	12.48909	0	14.72125	20.56169	3.722881	19.56012	15.66458	14.36927	7.281067	18.82247
AC128709.	9.609075	13.00508	16.98527	66.51474	15.50479	3.425661	1.69936	23.15068	7.003726	9.606993	0	7.360626	18.50552	17.68369	9.780058	26.38246	1.026376	1.820267	35.85232
AC128714.	4.804538	0	0	1.039293	0	0	0	0	0	0	0	1.840156	0	0	0	3.297807	0	0.910133	0
AC129102.	0.960908	0	0.999134	0	0	1.141887	1.69936	0	0	0	0	0	0	0	0.978006	0	0	0	0.896308
AC129492.	4.804538	3.001171	7.993069	11.43222	7.752394	14.84453	11.89552	9.26027	1.167288	6.724895	10.84279	18.40156	4.112337	13.9608	9.780058	5.771163	9.237386	1.820267	13.44462
AC129492.	0.960908	0	6.993936	1.039293	1.107485	0	0.84968	0	0	0	0	0	8.224675	7.445762	0	3.297807	0	4.550667	4.48154
AC129492.	16.33543	15.00586	26.97661	10.39293	16.61227	26.2634	6.797442	15.74246	14.00745	18.25329	15.66181	6.440548	14.39318	5.584322	6.846041	15.66458	2.052753	7.281067	0.896308
AC129492.	7.68726	5.001952	11.9896	18.70727	5.537425	4.567548	7.647122	11.11232	36.18592	4.803496	2.40951	8.280704	8.224675	10.23792	3.912023	14.84013	7.184634	8.191201	4.48154
AC129492.	5.765445	6.002343	12.98874	0	4.42994	5.709435	3.398721	7.408216	7.003726	10.56769	1.204755	4.600391	3.084253	0	4.890029	18.96239	2.052753	2.7304	16.13354
AC129502.	2.882723	7.002733	11.9896	7.275049	4.42994	1.141887	7.647122	4.630135	29.18219	2.882098	1.204755	15.64133	7.196591	26.06017	30.31818	10.71787	1.026376	7.281067	0.896308
AC129507.	1008.953	973.3799	999.1337	582.004	841.6885	884.9624	774.0587	966.7722	911.6517	977.0311	1272.221	481.2009	490.3962	633.8205	739.3724	590.3075	568.6124	675.319	697.3276
AC129507.	5.765445	2.000781	14.987	0	1.107485	2.283774	1.69936	0	15.17474	0.960699	1.204755	4.600391	7.196591	5.584322	4.890029	4.946711	1.026376	0	0.896308
AC129507.	3.84363	4.001562	4.995668	9.353635	6.64491	2.283774	8.496802	14.81643	11.67288	0	0	10.12086	3.084253	13.9608	11.73607	0.824452	1.026376	0.910133	5.377848
AC129507.	0	1.00039	0.999134	0	2.21497	0	2.549041	4.630135	0	0	0	0.920078	20.56169	0.93072	4.890029	0	0	3.640534	2.688924
AC129510.	35.55358	54.02108	64.94369	36.37525	44.2994	58.23624	40.78465	35.18903	59.53167	42.27077	74.6948	71.7661	59.62889	42.81313	44.98827	37.10033	48.23968	77.36134	78.8751
AC129510.	6.726353	6.002343	3.996535	0	16.61227	0	4.248401	5.556162	1.167288	7.685594	3.614265	9.200782	9.252759	11.16864	1.956012	0	0	0.910133	6.274156
AC129778.	0	1.00039	0.999134	1.039293	0	2.283774	7.647122	1.852054	2.334575	0.960699	1.204755	0	0	0	0.978006	0.824452	0	0	0
AC129807.	0	0	0	0	0	0	1.69936	0.926027	0	0.960699	0	0	0	0.93072	0.978006	0	0	0	0
AC129908.	0	0	0	0	8.859879	3.425661	0.84968	6.482189	28.01491	0	0	0	4.112337	0	0.978006	0	3.079129	0	1.792616
AC129915.	0.960908	2.000781	0	4.157171	0	28.54718	14.44456	2.778081	0	0.960699	0	6.440548	4.112337	7.445762	3.912023	0.824452	7.184634	6.370934	0.896308
AC129926.	0	0	3.996535	0	0	0	2.549041	0	0	0	0	0	0	0	0	0	0	0	0
AC130324.	25.9445	25.00976	38.96621	12.47151	47.62185	33.11472	14.44456	33.33697	33.85134	35.54587	27.70936	14.72125	14.39318	6.515042	21.51613	28.03136	28.73854	21.8432	39.43755
AC130324.	3.84363	6.002343	16.98527	2.078586	6.64491	10.27698	3.398721	12.96438	5.836439	0.960699	4.81902	6.440548	11.30893	26.06017	5.868035	10.71787	14.36927	3.640534	3.585232
AC130343.	0.960908	2.000781	0	0	9.967364	1.141887	0	0	11.67288	3.842797	1.204755	0.920078	1.028084	0	0	0.824452	0	6.370934	0.896308
AC130352.	22.10087	26.01015	26.97661	40.53242	46.51437	27.40529	15.29424	35.18903	82.87743	24.01748	59.03299	48.76415	41.12337	38.15953	20.53812	20.6113	45.16056	8.191201	22.4077
AC130360.	0	0	0	0	0	1.141887	0	0	0	0	1.204755	0	0	0	0	0	0	0	0
AC130364.	0	1.00039	0	1.039293	0	0	0.84968	0.926027	0	0.960699	0	0.920078	0	0	1.956012	1.648904	1.026376	0	0
AC130364.	0	0	0	0	0	0	0	0	0	0	0	0	0	0	0.978006	0	0	0.910133	0.896308
AC130365.	0.960908	0	0	0	0	0	0	0	0	0	0	0	0	0	0	0	1.026376	0.910133	0.896308
AC130366.	0.960908	0	7.993069	0	0	0	0.84968	0	0	0	0	2.760235	2.056169	0	0	0	0	0	0
AC130371.	7.68726	10.0039	4.995668	5.196464	0	12.56076	0	10.1863	0	0.960699	0	7.360626	2.056169	7.445762	0.978006	8.244518	4.105505	10.01147	2.688924
AC130371.	55.73264	31.0121	25.97748	17.66798	46.51437	39.96605	29.73881	25.00273	133.0708	53.79916	75.89956	65.32555	88.41526	26.06017	37.16422	24.73355	59.52982	83.73227	79.77141
AC130404.	2.882723	0	0	2.078586	1.107485	0	0.84968	5.556162	0	0	0	0.920078	1.028084	0	0	0.824452	1.026376	0	0.896308
AC130415.	0.960908	0	1.998267	0	4.42994	0	0	0	0	0	0	0.920078	0	0	0	0	0	0	0
AC130448.	0.960908	7.002733	0	0	0	1.141887	0.84968	0.926027	0	0	0	1.840156	1.028084	0.93072	0	0	1.026376	0	0
AC130448.	22.10087	10.0039	15.98614	6.235757	18.82724	0	15.29424	5.556162	0	1.921399	0	16.56141	6.168506	7.445762	0	1.648904	0	2.7304	17.02985
AC130454.	0	6.002343	0	0	0	0	0	0	0	0	0	1.840156	0	0	0	0	0	0	0

AC130456.	22.10087	40.01562	27.97574	16.62868	19.93473	22.83774	35.68657	12.96438	22.17847	24.97818	9.638039	19.32164	19.5336	40.02097	27.38416	16.48904	40.02867	26.39387	16.13354
AC130456.	0	0	0	0	0	0	0	0	0	0	0	0	0	0	0	0	0	1.820267	0
AC130456.	0	1.00039	5.994802	0	0	0	0	1.852054	0	0	2.40951	0.920078	1.028084	0	2.934017	0	0	0	0.896308
AC130462.	18.25724	6.002343	24.97834	15.58939	3.322455	5.709435	10.19616	22.22465	10.50559	10.56769	24.0951	4.600391	9.252759	14.89152	11.73607	6.595615	10.26376	20.93307	18.82247
AC130466.	271.9368	280.1093	204.8224	418.835	230.3569	199.8302	418.8924	231.5068	347.8517	170.0438	203.6036	257.6219	297.1164	358.3273	254.2815	341.3231	334.5987	255.7475	269.7887
AC130469.	56.69354	57.02225	34.96968	62.35757	28.79461	55.95246	56.92858	75.93422	128.4017	55.72056	68.67103	83.72712	80.19058	80.97267	57.70234	28.03136	25.65941	86.46267	51.08955
AC130469.	96.09075	46.01796	35.96881	140.3045	76.41646	62.80379	49.28145	59.26573	696.8708	95.10923	225.2892	26.68227	30.84253	73.5269	40.09824	142.6302	188.8532	262.1184	113.8311
AC130472.	1.921815	0	0	0	0	0	0.84968	0	0	2.882098	3.614265	0	3.084253	0	0.978006	0	1.026376	0.910133	0
AC130509.	0	1.00039	0	2.078586	5.537425	1.141887	0.84968	0.926027	0	0	0	0	0	2.792161	0.978006	0	0	1.820267	2.688924
AC130650.	0	0	5.994802	6.235757	34.33203	2.283774	0.84968	8.334243	4.669151	5.764196	0	3.680313	3.084253	0	1.956012	17.31349	2.052753	5.4608	2.688924
AC130650.	253.6796	163.0636	148.8709	58.2004	222.6045	123.3238	190.3284	230.5807	147.0783	130.6551	162.6419	190.4562	259.0773	165.6682	202.4472	102.232	92.37386	102.8451	83.35664
AC130651.	0	3.001171	0.999134	0	0	0	0.84968	0	1.167288	1.921399	0	0	0	0	0	0	0	0	0.896308
AC130651.	8.648168	24.00937	4.995668	17.66798	18.82724	19.41208	13.59488	10.1863	0	6.724895	12.04755	13.80117	3.084253	2.792161	17.6041	16.48904	22.58028	14.56213	7.170464
AC130686.	12.4918	7.002733	13.98787	18.70727	19.93473	2.283774	7.647122	5.556162	39.68778	23.05678	53.00921	13.80117	25.70211	13.9608	15.64809	9.06897	2.052753	10.9216	24.20031
AC130689.	59.57627	63.0246	60.94715	48.84676	26.57964	41.10793	73.92218	51.85751	10.50559	34.58517	22.89034	51.52438	52.4323	46.53602	59.65836	79.97183	54.39794	39.13574	34.95601
AC130709.	0	0	0.999134	4.157171	0	1.141887	5.098081	0	0	0	0	0.920078	0	0	0	0	0	0	0
AC130710.	0	0	1.998267	0	0	0	0	1.852054	0	0	0	0.920078	0	5.584322	0	0	0	0.910133	0
AC130895.	0.960908	0	1.998267	5.196464	1.107485	0	0.84968	0.926027	4.669151	0.960699	0	0.920078	6.168506	0.93072	1.956012	1.648904	3.079129	6.370934	0
AC131009.	53.81082	45.01757	35.96881	32.21808	26.57964	50.24303	54.37954	49.07943	67.70269	55.72056	102.4042	44.16376	43.17954	61.42754	44.98827	51.11601	58.50345	34.58507	40.33386
AC131009.	14.41361	6.002343	11.9896	10.39293	9.967364	30.83095	3.398721	7.408216	10.50559	14.41049	24.0951	10.12086	10.28084	17.68369	2.934017	15.66458	12.31652	20.02294	3.585232
AC131009.	130.6834	89.03475	125.8908	181.8762	127.3608	99.34417	110.4584	124.0876	117.8961	122.0088	93.97088	139.8519	141.8756	124.7165	144.7449	161.5926	140.6135	156.5429	156.8539
AC131009.	79.75532	133.0519	176.8467	65.47544	54.26676	74.22266	78.17058	110.1972	10.50559	111.4411	134.9325	112.2495	98.6961	98.65635	84.1085	61.00944	83.13648	87.37281	118.3126
AC131011.	16.33543	32.01249	33.97054	23.90373	25.47215	13.70264	35.68657	54.6356	5.836439	13.44979	9.638039	35.88305	21.58977	37.22881	17.6041	22.2602	19.50115	19.1128	39.43755
AC131025.	24.02269	25.00976	49.95668	34.29666	56.48173	30.83095	31.43817	56.48765	63.03354	79.73804	95.17564	26.68227	21.58977	34.43665	31.29619	28.03136	55.42432	29.12427	29.57816
AC131097.	10.56998	21.0082	57.94975	4.157171	39.86946	22.83774	50.98081	30.55889	19.84389	32.66377	14.45706	30.36258	27.75828	43.74385	22.49413	27.20691	20.52753	33.67494	21.51139
AC131097.	20.17906	30.01171	21.98094	2.078586	7.752394	45.67548	16.9936	50.93149	49.02608	34.58517	43.37118	23.00196	33.92678	53.98178	45.96627	18.96239	35.92317	36.40534	27.78555
AC131097.	122.0353	60.02343	291.747	372.0668	444.1015	388.2416	126.6024	118.5315	464.5805	50.91706	124.0898	219.8987	244.6841	177.7676	185.8211	65.13169	113.9278	39.13574	86.04556
AC131097.	12.4918	14.00547	19.98267	8.314342	6.64491	14.84453	33.13753	22.22465	7.003726	2.882098	8.433284	11.04094	11.30893	26.99089	15.64809	33.80253	16.42202	10.9216	25.09662
AC131157.	0	1.00039	0	0	0	0	1.69936	7.408216	0	0.960699	0	0	0	0.93072	0.978006	0	1.026376	0	0.896308
AC131159.	12.4918	11.00429	8.992203	6.235757	14.3973	13.70264	5.947762	7.408216	9.338302	12.48909	4.81902	16.56141	16.44935	13.03008	9.780058	9.06897	16.42202	4.550667	5.377848
AC131159.	64.3808	55.02147	58.94889	65.47544	70.87903	78.7902	64.5757	45.37532	143.5764	87.42363	121.6802	70.84602	62.71315	45.60529	68.46041	55.23827	80.05735	60.06881	67.2231
AC131160.	0.960908	1.00039	0	1.039293	6.64491	0	0.84968	4.630135	0	0	0	2.760235	0	0	0	2.473355	6.158258	0	0.896308
AC131182.	2.882723	4.001562	0	17.66798	13.28982	1.141887	4.248401	0.926027	1.167288	0.960699	4.81902	1.840156	1.028084	3.722881	0	2.473355	3.079129	4.550667	13.44462
AC131206.	70.14625	98.03826	70.93849	55.08252	44.2994	59.37812	103.661	41.67122	53.69524	74.93454	67.46627	102.1287	103.8365	54.9125	89.97654	65.95615	37.97592	50.05734	112.9348
AC131210.	0	4.001562	0	0	0	0	0	0.926027	0	0	0	0	0	0	0	0	2.052753	0	0
AC131211.	0.960908	0	0	0	0	0	3.398721	0	0	0	0	0	0	0	0	0	0	0	0
AC131212.	7.68726	18.00703	8.992203	2.078586	19.93473	13.70264	16.14392	12.96438	67.70269	39.38867	36.14265	6.440548	11.30893	13.03008	7.824047	14.84013	41.05505	14.56213	10.7557
AC131212.	313.2559	349.1363	341.7037	126.7937	352.1802	326.5797	266.7996	396.3396	128.4017	404.4544	385.5216	365.2711	456.4695	361.1195	340.346	220.1286	227.8555	252.107	286.8185
AC131212.	137.4098	179.0699	81.92896	68.59332	141.7581	125.6076	100.2623	141.6821	162.253	217.118	321.6696	204.2574	160.3812	94.00275	137.8988	75.84957	112.9014	148.3517	233.9364
AC131212.	0	0	0	0	0	4.567548	0.84968	0	0	1.921399	0	0.920078	0	0	0	0.824452	2.052753	0	6.274156
AC131233.	21.13997	33.01288	26.97661	15.58939	60.91167	39.96605	25.49041	22.22465	127.2344	64.36685	66.26152	23.00196	22.61786	40.95169	38.14223	27.20691	27.71216	22.75334	42.12647
AC131235.	53.81082	55.02147	58.94889	42.611	43.19191	51.38492	43.33369	51.85751	45.52422	22.09608	50.5997	55.20469	58.60081	30.71377	37.16422	34.62698	50.29244	56.42827	34.95601
AC131235.	1.921815	0	11.9896	3.117878	1.107485	1.141887	0.84968	3.704108	0	7.685594	4.81902	0	2.056169	3.722881	4.890029	4.122259	3.079129	2.7304	8.963079
AC131235.	24.9836	11.00429	22.98007	19.74656	32.11706	23.97963	41.63433	23.15068	28.01491	40.34937	22.89034	14.72125	21.58977	15.82225	14.67009	20.6113	17.4484	37.31547	26.88924
AC131235.	6.726353	36.01406	32.97141	38.45383	16.61227	12.56076	27.18977	57.41368	4.669151	25.93888	6.023774	21.1618	13.3651	40.02097	28.36217	29.68027	20.52753	9.101334	22.4077
AC131254.	4.804538	0	0	0	4.42994	0	0	1.852054	1.167288	9.606993	0	0	0	0.93072	0	9.893422	8.21101	0.910133	2.688924
AC131274.	0	12.00469	5.994802	0	0	0	5.947762	0	0	0	0	0	2.056169	0	0	0	0	0	0
AC131280.	4.804538	3.001171	2.997401	10.39293	26.57964	9.135096	30.58849	0.926027	1.167288	12.48909	13.2523	18.40156	7.196591	0	6.846041	6.595615	1.026376	8.191201	0.896308
AC131281.	5.765445	3.001171	13.98787	6.235757	16.61227	15.98642	11.89552	22.22465	18.6766	32.66377	24.0951	15.64133	21.58977	1.861441	21.51613	9.06897	7.184634	16.3824	18.82247
AC131281.	0	0	0	0	0	1.141887	0	0	0	0	0	0	0	0	0.978006	0	0	0	0
AC131391.	4.804538	0	6.993936	4.157171	7.752394	0	11.04584	0.926027	0	0	0	13.80117	9.252759	4.653602	2.934017	0.824452	4.105505	1.820267	0.896308
AC131392.	63.4199	88.03436	70.93849	34.29666	42.08443	14.84453	5.098081	81.49038	56.02981	35.54587	13.2523	42.3236	55.51656	53.05106	57.70234	70.07841	61.58258	43.6864	25.99293
AC131392.	28.82723	6.002343	19.98267	16.62868	39.86946	27.40529	0	35.18903	137.74	24.97818	33.73314	49.68422	47.29188	78.18051	66.5044	97.28532	65.68808	103.7552	57.36371
AC131532.	0	5.001952	0	0	0	0	1.69936	0	0	0	0	0	0	0	6.846041	0	0	0	0.896308
AC131571.	0.960908	6.002343	19.98267	7.275049	15.50479	21.69585	4.248401	20.37259	9.338302	20.17468	18.07132	5.520469	1.028084	9.307203	13.69208	0.824452	3.079129	0.910133	3.585232
AC131649.	4.804538	4.001562	3.996535	0	0	1.141887	0.84968	0.926027	0	0	1.204755	7.360626	6.168506	0	0	0	1.026376	3.640534	5.377848
AC131649.	1138.675	1428.558	1756.477	1001.878	1209.374	847.2802	1278.769	1362.186	646.6774	1293.101	1255.355	1548.492	1671.665	947.4733	1079.718	1028.091	1013.033	1552.688	1944.092
AC131888.	7.68726	23.00898	20.98181	7.275049	2.21497	27.40529	11.89552	47.22738	0	6.724895	12.04755	17.48149	28.78636	9.307203	21.51613	16.48904	15.39564	8.191201	10.7557
AC131902.	33.63176	39.01523	166.8553	61.31827	116.2859	43.39171	15.29424	115.7534	67.70269	120.0874	118.066	88.32751	88.41526	43.74385	44.98827	51.94047	51.31881	26.39387	34.0597
AC131902.	21.13997	81.03163	127.8891	83.14342	198.2398	100.4861	16.14392	124.0876	245.1304	161.3975	151.7991	103.0488	124.3982	38.15953	68.46041	63.48279	97.50574	40.04587	26.88924
AC131902.	11.53089	33.01288	99.91337	56.12181	108.5335	66.22945	31.43817	112.0493	185.5987	95.10923	96.38039	85.56728	65.7974	32.57521	33.2522	33.80253	41.05505	30.0344	25.09662
AC131934.	40.35812	43.01679	68.94022	53.00393	68.66407	27.40529	60.3273	35.18903	115.5615	31.70308	19.27608	74.52634	89.44334	32.57521	44.01026	36.27588	52.34519	33.67494	36.74863

AC131935.	0	0	0	0	0	0	0	0	4.669151	0	0	0	0	0	0	0	0	0	0
AC131953.	34.59267	73.0285	105.9082	33.25737	139.5431	21.69585	81.5693	53.70957	46.69151	62.44545	34.93789	79.12673	64.76932	78.18051	84.1085	29.68027	39.0023	36.40534	39.43755
AC131956.	24.02269	16.00625	22.98007	28.0609	6.64491	2.283774	58.62794	14.81643	18.6766	7.685594	1.204755	2.760235	3.084253	40.95169	44.98827	51.94047	9.237386	9.101334	6.274156
AC131971.	32.67086	26.01015	38.96621	39.49313	37.65449	19.41208	69.67378	34.263	8.171014	14.41049	7.228529	31.28266	26.73019	28.85233	22.49413	47.81821	32.84404	40.04587	34.95601
AC132008.	126.8398	129.0504	122.8934	93.53635	182.735	141.594	111.3081	74.08216	193.7698	122.9695	133.7278	184.0156	145.988	68.8733	63.57038	63.48279	64.6617	60.06881	77.97879
AC132008.	7597.896	6715.621	8280.82	5544.627	6504.259	7181.327	9736.486	8454.627	7227.846	6909.349	5924.984	7219.854	6868.632	8599.856	9445.58	6189.984	5985.826	5767.515	5585.791
AC132068.	0	2.000781	1.998267	8.314342	0	1.141887	2.549041	0	3.501863	0	0	1.840156	16.44935	0	3.912023	0	2.052753	0.910133	1.792616
AC132154.	3.84363	1.00039	0	0	0	0	3.398721	6.482189	0	0	2.40951	7.360626	3.084253	11.16864	1.956012	4.122259	1.026376	1.820267	1.792616
AC132186.	0	5.001952	1.998267	0	0	5.709435	6.797442	9.26027	0	0.960699	0	2.760235	4.112337	0.93072	4.890029	0	0	6.370934	3.585232
AC132192.	30.74904	42.0164	25.97748	30.13949	57.58922	30.83095	70.52346	64.82189	17.50932	37.46727	45.78069	58.88501	38.03912	49.32818	52.81231	24.73355	22.58028	46.4168	40.33386
AC132192.	129.7225	234.0914	334.7098	88.33989	259.1515	219.2423	173.3348	283.3643	462.2459	456.3321	540.9349	229.0995	148.0441	221.5114	247.4355	136.859	148.8246	193.8584	198.0841
AC132216.	1.921815	6.002343	0	11.43222	12.18233	0	0	15.74246	4.669151	3.842797	4.81902	6.440548	1.028084	5.584322	0.978006	8.244518	1.026376	1.820267	2.688924
AC132219.	14.41361	29.01132	2.997401	9.353635	3.322455	6.851322	16.9936	12.03835	2.334575	11.52839	10.84279	23.00196	13.3651	13.03008	18.58211	14.84013	20.52753	9.101334	9.859387
AC132219.	147.0189	200.0781	160.8605	322.1808	259.1515	246.6476	258.3028	181.5013	50.19337	104.7162	81.92333	181.2554	179.9148	195.4513	202.4472	205.2885	186.8005	143.8011	114.7274
AC132803.	0.960908	0	4.995668	0	2.21497	0	2.549041	1.852054	0	0.960699	0	0	1.028084	0	0	0.824452	0	0	0.896308
AC132807.	5.765445	0	0	0	0	0	0	0.926027	0	0	0	0	2.056169	3.722881	0	0	0	0	0
AC132812.	980.1257	959.3744	929.1943	844.945	1142.924	1097.353	542.9457	970.4763	2497.996	1365.154	1566.181	944.0003	1023.972	847.8862	880.2052	1113.834	1191.623	967.4718	832.6701
AC132812.	33.63176	1.00039	1.998267	4.157171	0	0	25.49041	12.96438	3.501863	0.960699	0	0.920078	0	6.515042	7.824047	7.420066	10.26376	7.281067	3.585232
AC132825.	5.765445	0	0	0	1.107485	0	0	0	0	0	0	0	0	0	0	0	0	0.910133	0
AC132825.	3.84363	0	0	0	0	0	0	0	0	3.842797	0	0	0	0	0	0	0	0	0
AC132872.	1098.317	1129.441	1396.789	529	1518.362	1069.948	832.6866	1088.082	2048.59	1402.621	1957.727	1007.486	1039.393	1299.286	1258.693	685.9439	801.5999	1507.181	1262.002
AC132872.	86.48168	44.01718	101.9116	63.39686	48.72934	25.12151	36.53625	37.04108	141.2418	69.17035	151.7991	94.76806	87.38717	93.07203	77.26246	83.26963	115.9805	174.7456	152.3724
AC132872.	40.35812	86.03358	90.92116	35.33595	106.3186	116.4725	45.88273	40.74519	107.3905	76.85594	236.132	96.60821	103.8365	93.07203	84.1085	46.1693	40.02867	117.4072	100.3865
AC132872.	236.3833	283.1105	388.663	219.2908	207.0997	235.2287	265.1002	325.9615	57.1971	325.677	242.1557	299.9455	228.2347	308.0684	324.6979	327.3074	404.3922	501.4835	406.9238
AC132938.	19.21815	40.01562	52.95408	19.74656	19.93473	43.39171	16.9936	53.70957	49.02608	47.07426	43.37118	34.96297	46.2638	28.85233	21.51613	33.80253	44.13418	35.4952	12.54831
AC132938.	14.41361	10.0039	6.993936	9.353635	26.57964	10.27698	9.346483	1.852054	39.68778	10.56769	19.27608	12.8811	14.39318	4.653602	8.802052	4.122259	5.131881	19.1128	9.859387
AC132938.	49.00628	35.01367	48.95755	84.18271	38.76197	51.38492	31.43817	25.92876	94.55031	69.17035	30.11887	38.64329	54.48847	87.48771	46.94428	50.29156	61.58258	96.47414	80.66772
AC132938.	147.0189	170.0664	208.8189	239.0373	159.4778	166.7155	283.7932	161.1287	108.5578	153.7119	150.5944	274.1833	263.1896	132.1623	137.8988	201.9907	195.0115	216.6118	242.8995
AC132938.	583.2709	771.301	940.1848	486.389	747.5523	373.3971	1036.61	838.9805	248.6323	669.6074	655.3867	811.509	851.2539	676.6337	747.1964	575.4674	608.6411	976.5732	1106.044
AC132942.	329.5913	290.1132	368.6803	191.2299	266.9039	264.9178	265.1002	238.915	354.8555	246.8997	227.6987	197.8168	278.6109	260.6017	260.1495	179.7305	187.8269	132.8795	181.0542
AC133041.	1.921815	10.0039	0.999134	1.039293	3.322455	5.709435	6.797442	1.852054	0	13.44979	42.16642	11.96102	2.056169	1.861441	2.934017	4.122259	2.052753	6.370934	5.377848
AC133065.	14.41361	4.001562	19.98267	2.078586	15.50479	3.425661	18.69297	18.52054	2.334575	1.921399	24.0951	14.72125	27.75828	4.653602	6.846041	10.71787	4.105505	10.9216	10.7557
AC133065.	365.1449	270.1054	312.7288	328.4165	507.2281	540.1126	217.5181	438.9368	489.0936	328.5591	568.6443	847.3921	756.6701	403.0019	395.1144	509.5112	459.8166	876.4585	978.7683
AC133065.	16.33543	15.00586	20.98181	24.94303	6.64491	25.12151	36.53625	15.74246	5.836439	7.685594	24.0951	17.48149	11.30893	7.445762	17.6041	13.19123	8.21101	14.56213	13.44462
AC133065.	508.3201	374.146	434.6231	577.8468	537.1302	392.8091	394.2516	597.2874	333.8443	334.3233	307.2125	706.6201	717.6029	536.0949	609.2976	742.8311	643.5379	960.1908	955.4643
AC133065.	1668.135	696.2717	1155.998	1010.193	1913.734	2006.295	516.6056	1959.473	2740.792	1656.246	1986.641	3280.999	3380.341	1336.514	1469.943	1942.409	1512.879	4005.497	3020.558
AC133106.	0	2.000781	5.994802	2.078586	0	1.141887	3.398721	0	4.669151	10.56769	0	2.760235	3.084253	0.93072	0	0	3.079129	2.7304	0
AC133134.	0	0	0.999134	0	0	0	0	0	1.167288	0.960699	3.614265	1.840156	6.168506	0	0	0.824452	1.026376	0	1.792616
AC133141.	0	0	0	0	0	0	0	0	0	0	4.81902	0	0	0	0	0	0	0	0
AC133435.	52.84991	55.02147	46.95928	16.62868	52.05179	59.37812	28.03945	38.89314	101.554	33.62447	54.21397	35.88305	24.67402	40.95169	37.16422	18.13794	21.5539	30.0344	27.78555
AC133473.	29.78813	9.003514	36.96795	27.02161	26.57964	12.56076	27.18977	15.74246	0	15.37119	27.70936	12.8811	21.58977	15.82225	24.45015	12.36678	22.58028	16.3824	28.68185
AC133485.	0	0	5.994802	0	0	0	0	0	0	0	0	0	0	0	0	0	0	0	0
AC133485.	0	1.00039	0	0	0	0	0	0	0	0	0	0	0	0	0	0	0	2.7304	0.896308
AC133528.	49.00628	36.01406	26.97661	48.84676	64.23413	60.52001	27.18977	19.44657	73.53913	14.41049	43.37118	23.00196	19.5336	26.99089	24.45015	47.81821	28.73854	15.47227	6.274156
AC133539.	4.804538	0	0	0	0	0	1.141887	0	0	0	0	0	1.028084	4.653602	0	0.824452	0	0	0
AC133540.	13.45271	19.00742	0.999134	9.353635	6.64491	6.851322	6.797442	8.334243	49.02608	14.41049	43.37118	19.32164	10.28084	2.792161	0	4.122259	4.105505	11.83173	38.54124
AC133548.	1.921815	0	0.999134	0	0	0	0.84968	0	0	0	0	0	0	0	0	0	1.026376	0	0
AC133548.	0.960908	0	0.999134	0	2.21497	0	2.549041	2.778081	0	0	0	4.600391	0	0	3.912023	0	0	0	0.896308
AC133550.	116.2698	117.0457	168.8536	140.3045	95.2437	115.3306	135.0992	120.3835	88.71387	167.1617	107.2232	108.5692	126.4544	135.8852	133.9868	140.1568	211.4335	167.4645	171.1948
AC133552.	25.9445	14.00547	23.97921	30.13949	13.28982	14.84453	63.72602	57.41368	3.501863	10.56769	18.07132	45.08383	35.98295	17.68369	29.34017	14.84013	21.5539	18.20267	26.88924
AC133552.	149.9016	227.0886	231.799	201.6228	242.5392	183.8438	158.8902	176.8712	304.6621	353.5373	432.507	246.581	214.8696	190.7977	226.8974	145.1035	248.3831	237.5448	284.1296
AC133552.	79.75532	108.0422	182.8415	30.13949	119.6084	68.51322	73.92218	137.978	366.5283	209.4324	115.6565	115.0098	177.8586	100.5178	87.04252	61.00944	118.0333	93.74374	94.11233
AC133552.	210.4387	218.0851	412.6422	323.2201	306.7733	129.0332	451.1802	259.2876	68.86998	224.8036	259.0223	381.8325	280.667	227.0958	227.8754	190.4484	269.937	212.0611	368.3826
AC133552.	141.2534	115.0449	345.7002	259.8232	171.6602	101.6279	478.37	169.4629	9.338302	134.4979	101.1994	284.3042	306.3691	155.4303	111.4927	191.2728	160.1147	164.7341	278.7518
AC133552.	27.86632	41.01601	40.96448	5.196464	17.71976	22.83774	14.44456	22.22465	54.86252	30.74238	137.3421	27.60235	29.81445	16.75297	29.34017	32.97807	39.0023	23.66347	34.0597
AC133555.	0	1.00039	7.993069	0	3.322455	1.141887	1.69936	2.778081	0	0	0	0	0	4.653602	0	1.648904	1.026376	1.820267	0.896308
AC133555.	1.921815	0	0	0	0	1.141887	0	0	0	2.882098	0	2.760235	0	0	0	0	0	0.910133	0
AC133555.	1.921815	1.00039	2.997401	0	0	2.283774	0.84968	0	0	1.921399	2.40951	0	4.112337	0.93072	3.912023	0	3.079129	5.4608	1.792616
AC133561.	0	13.00508	4.995668	7.275049	11.07485	5.709435	8.496802	4.630135	0	12.48909	12.04755	0.920078	5.140422	1.861441	4.890029	5.771163	5.131881	2.7304	2.688924
AC133561.	0	0	0	0	0	0	0	0.926027	0	0	0	0	1.028084	0	0	0	0	0	0
AC133561.	0	1.00039	0	0	0	0	0.84968	0	0	0	0	0	0	0	0	0	0	0	0
AC133637.	1.921815	0	0	0	0	0	0	0	0	3.842797	0	0	0	0	0	3.297807	0	0	0

AC133644.	0.960908	0	0	0	0	1.141887	0	0	0	0	0	0	0	0	1.956012	0	1.026376	0	0.896308
AC133644.	6.726353	0	2.997401	0	1.107485	9.135096	0.84968	0	28.01491	0	0	0.920078	0	0	0	0	2.052753	0.910133	0.896308
AC133785.	76.8726	134.0523	171.851	51.96464	127.3608	78.7902	71.37314	100.9369	179.7623	126.8123	210.8321	38.64329	59.62889	123.7858	86.06451	84.09409	96.47937	67.34987	87.83818
AC133794.	18.25724	27.01054	9.991337	20.78586	11.07485	18.27019	23.79105	14.81643	4.669151	24.01748	4.81902	14.72125	4.112337	15.82225	11.73607	31.32917	21.5539	12.74187	8.066772
AC133919.	0	0	0	4.157171	0	0	1.69936	2.778081	0	0	0	0	1.028084	0	3.912023	5.771163	3.079129	2.7304	0
AC133919.	0	42.0164	1.998267	1.039293	2.21497	0	0	0.926027	0	276.6814	787.9097	3.680313	6.168506	3.722881	0.978006	7.420066	5.131881	113.7667	121.8979
AC133919.	1.921815	6.002343	0	2.078586	0	0	0.84968	0	0	14.41049	21.68559	0.920078	2.056169	0	0	1.648904	0	5.4608	16.13354
AC133961.	16.33543	34.01327	33.97054	20.78586	3.322455	17.12831	21.24201	6.482189	23.34575	28.82098	37.3474	46.92399	27.75828	49.32818	6.846041	24.73355	41.05505	30.94454	59.15632
AC133963.	1.921815	1.00039	0	2.078586	6.64491	4.567548	9.346483	10.1863	7.003726	7.685594	43.37118	14.72125	4.112337	0.93072	1.956012	4.122259	1.026376	5.4608	3.585232
AC133963.	9.609075	1.00039	11.9896	8.314342	8.859879	0	3.398721	10.1863	0	0.960699	0	11.96102	15.42127	1.861441	7.824047	8.244518	5.131881	4.550667	1.792616
AC133965.	0	0	0	0	0	2.283774	0	0	0	0.960699	1.204755	0.920078	0	0	0	0	0	0	0
AC134026.	980.1257	1137.444	814.2939	1299.116	632.3739	1391.96	1564.261	905.6544	563.8	718.603	403.5929	808.7488	912.9389	960.5034	793.1627	1081.681	1059.22	753.5905	1118.592
AC134043.	4.804538	0	0	0	0	0	4.248401	0	0	0.960699	0	0.920078	1.028084	1.861441	0	6.595615	0	1.820267	1.792616
AC134043.	270.9759	257.1003	280.7566	366.8703	241.4317	196.4046	282.0938	280.5862	185.5987	308.3845	239.7462	230.0196	228.2347	411.3784	338.39	256.4045	325.3613	324.9176	260.8256
AC134050.	0	0	0	9.353635	0	0	0	0	0	0	0	0	1.028084	0	3.912023	0.824452	1.026376	0	4.48154
AC134312.	118.1916	153.0597	80.92983	56.12181	145.0805	39.96605	186.08	54.6356	79.37557	143.1442	146.9801	48.76415	38.03912	152.6381	128.1188	132.7367	218.6181	156.5429	338.8044
AC134312.	15.37452	14.00547	15.98614	4.157171	7.752394	2.283774	11.89552	3.704108	7.003726	12.48909	12.04755	6.440548	6.168506	11.16864	13.69208	7.420066	9.237386	4.550667	17.92616
AC134312.	35.55358	26.01015	32.97141	20.78586	23.25718	12.56076	28.88913	14.81643	23.34575	13.44979	33.73314	23.00196	7.196591	37.22881	44.98827	26.38246	22.58028	25.48374	69.91202
AC134312.	0	0	0	0	0	0	0	0	7.003726	0	0	0	0	0	3.912023	0	1.026376	0	0.896308
AC134312.	0	2.000781	0	5.196464	0	0	2.549041	0	0	0.960699	0	0	0	3.722881	0	0	0	0	0
AC134349.	1.921815	0	8.992203	1.039293	2.21497	0	0.84968	3.704108	0	0	7.228529	4.600391	3.084253	0	6.846041	3.297807	0	0.910133	6.274156
AC134349.	5.765445	10.0039	7.993069	8.314342	14.3973	12.56076	13.59488	1.852054	14.00745	17.29259	4.81902	19.32164	13.3651	17.68369	6.846041	5.771163	6.158258	9.101334	12.54831
AC134407.	31.70995	39.01523	54.95235	56.12181	39.86946	34.25661	32.28785	50.00546	52.52795	33.62447	43.37118	13.80117	32.8987	46.53602	23.47214	4.946711	17.4484	10.01147	9.859387
AC134407.	36.51449	53.02069	59.94802	25.98232	40.97694	33.11472	43.33369	37.04108	17.50932	56.68126	74.6948	41.40352	26.73019	40.95169	29.34017	17.31349	21.5539	5.4608	18.82247
AC134407.	94.16894	121.0472	131.8856	91.45776	91.92125	53.66869	175.8838	170.389	49.02608	78.77734	46.98544	132.4913	102.8084	91.21059	163.327	80.79628	101.4076	76.45121	67.2231
AC134511.	1.921815	0	0	3.117878	0	0	0	1.852054	0	0	0	1.840156	2.056169	0	0.978006	0.824452	2.052753	0.910133	2.688924
AC134669.	53.81082	18.00703	10.99047	23.90373	39.86946	14.84453	39.08529	19.44657	21.01118	31.70308	46.98544	11.96102	30.84253	34.43665	36.18622	42.04704	54.39794	30.0344	50.19325
AC134669.	4.804538	2.000781	0.999134	0	3.322455	0	0	4.630135	0	1.921399	0	0.920078	1.028084	0	0	2.473355	7.184634	0.910133	0
AC134682.	68.22443	64.02499	46.95928	130.9509	34.33203	54.81058	117.2559	16.66849	96.88488	95.10923	113.247	147.2125	188.1394	101.4485	48.90029	101.4076	153.9564	128.3288	223.1807
AC134684.	0	0	0	0	0	0	0.84968	0	0	1.921399	0	0	0	0	0	0.824452	0	0.910133	0
AC134684.	3.84363	31.0121	17.98441	4.157171	26.57964	28.54718	2.549041	9.26027	42.02236	25.93888	83.12809	8.280704	4.112337	8.376483	6.846041	7.420066	3.079129	3.640534	19.71877
AC134698.	5.765445	0	0	0	0	0	0	0	0	0	0	0	0	0.93072	0	0	0	1.820267	0.896308
AC134772.	1.921815	2.000781	0.999134	0	0	0	4.248401	3.704108	0	1.921399	0	0.920078	2.056169	0.93072	0	4.122259	3.079129	5.4608	5.377848
AC134772.	24.02269	15.00586	16.98527	22.86444	27.68712	21.69585	26.34009	26.85478	15.17474	24.97818	44.57593	23.00196	12.33701	17.68369	29.34017	23.08465	28.73854	39.13574	15.23724
AC134775.	41.31902	16.00625	55.95149	46.76817	25.47215	5.709435	39.08529	42.59724	4.669151	18.25329	10.84279	45.08383	25.70211	30.71377	35.20821	38.74924	20.52753	46.4168	20.61508
AC134878.	40.35812	46.01796	15.98614	2.078586	48.72934	1.141887	1.69936	5.556162	0	37.46727	132.523	0.920078	0	0	0	0	1.026376	2.7304	5.377848
AC134915.	0	0	0	0	0	0	0	0	0	0	0	0.920078	0	0	0	0	4.105505	0	0
AC134980.	0	0	3.996535	0	3.322455	0	0	2.778081	0	0	0	0	4.112337	0	0	1.648904	0	1.820267	0
AC134981.	0	2.000781	0	0	0	0	0	0	0	1.921399	0	0	0	0.93072	1.956012	0.824452	0	1.820267	1.792616
AC135012.	0.960908	1.00039	0.999134	0	1.107485	6.851322	0	0	0	0	0	0	1.028084	0.93072	0.978006	0	0	0	0
AC135048.	0	1.00039	5.994802	0	0	0	3.398721	1.852054	0	1.921399	0	1.840156	3.084253	0	0	0	2.052753	1.820267	0
AC135048.	29.78813	52.0203	27.97574	3.117878	39.86946	29.68906	20.39233	15.74246	22.17847	47.07426	30.11887	42.3236	28.78636	20.47585	25.42815	42.04704	33.87042	26.39387	17.92616
AC135048.	39.39721	29.01132	17.98441	54.04322	76.41646	92.49285	33.98721	32.41095	77.04099	53.79916	92.76613	70.84602	61.68506	57.70466	44.98827	84.09409	70.81996	101.9349	98.59387
AC135050.	84.55986	107.0418	76.93329	118.4794	95.2437	102.7698	89.21643	43.52327	96.88488	70.13105	104.8137	98.44837	79.1625	95.86419	86.06451	103.0565	137.5344	68.26001	93.21603
AC135050.	180.6506	92.03592	86.92463	66.51474	163.9078	146.1615	73.92218	251.8794	284.8182	269.9565	409.6167	108.5692	87.38717	117.2708	97.80058	84.09409	130.3498	112.8565	114.7274
AC135050.	25.9445	28.01093	31.97228	27.02161	15.50479	31.97284	35.68657	22.22465	32.68406	40.34937	34.93789	51.52438	39.06721	28.85233	21.51613	37.92478	20.52753	12.74187	25.99293
AC135050.	438.1738	370.1445	459.6015	359.5953	234.7868	298.0325	464.7751	411.156	165.7549	214.2359	219.2654	297.1853	266.2739	313.6527	336.434	330.6052	260.6996	433.2235	363.0047
AC135050.	2428.213	2148.839	2508.825	2300.994	2209.432	2254.085	2255.901	2417.857	2429.126	1997.294	1745.69	2184.266	2164.118	2278.403	2295.38	2212.004	1991.17	2454.63	1509.383
AC135050.	24.9836	14.00547	19.98267	14.5501	33.22455	14.84453	5.947762	17.59451	66.5354	16.33189	18.07132	24.84211	11.30893	6.515042	16.6261	6.595615	4.105505	20.02294	18.82247
AC135068.	0	0	0	0	0	0	0	0	1.167288	2.882098	0	0	0	0	0	0	0	0	0
AC135068.	0	0	0	0	0	0	1.69936	0	0	0	0	0	0	0	0	0	0	0	1.792616
AC135068.	0	1.00039	0.999134	6.235757	0	1.141887	0	0	0	0	3.614265	0	0	0	0	0	0	0.910133	1.792616
AC135068.	1.921815	0	0	0	0	0	0	0	0	0	0	0	1.028084	0.93072	1.956012	0	0	2.7304	0
AC135068.	0	0	0	0	0	0	0	2.778081	0	1.921399	0	0	2.056169	0	0	0	0	0	0
AC135068.	0	0	0	0	2.21497	0	0	0	0	0	0	0	0	0	0	0	0	0	0
AC135068.	378.5976	368.1437	313.728	217.2122	571.4622	511.5654	189.4787	386.1533	1391.407	622.5331	854.1712	365.2711	336.1836	470.0138	471.3988	328.1318	441.3418	434.1336	294.8853
AC135068.	0.960908	0	0	0	0	0	0	0	0	0	0	1.840156	0	0	0	0	0	0	0
AC135068.	0	2.000781	0.999134	4.157171	0	1.141887	0.84968	1.852054	5.836439	0.960699	0	0	1.028084	1.861441	1.956012	202.8152	5.131881	0.910133	3.585232
AC135068.	0.960908	0	0	0	4.42994	0	1.69936	5.556162	0	0	4.81902	1.840156	5.140422	7.445762	2.934017	3.297807	4.105505	9.101334	0.896308
AC135178.	57.65445	42.0164	32.97141	50.92535	44.2994	27.40529	46.73241	37.04108	61.86625	37.46727	21.68559	31.28266	28.78636	51.18962	33.2522	27.20691	59.52982	46.4168	41.23017
AC135178.	49.00628	40.01562	49.95668	49.88605	24.36467	45.67548	58.62794	29.63287	7.003726	19.21399	2.40951	61.64524	68.88165	40.02097	39.12023	72.55176	53.37157	52.78774	40.33386
AC135178.	329.5913	223.0871	390.6613	227.6051	181.6275	199.8302	328.8263	263.9177	112.0596	229.6071	168.6657	366.1911	313.5657	211.2735	199.5132	300.1005	288.4117	238.455	284.1296

AC135178.	0	0	0	1.039293	0	0	0	0	0	0	0	0	0	0	0	0.824452	0	0	3.585232
AC135178.	59.57627	59.02304	71.93762	84.18271	44.2994	73.08077	76.47122	59.26573	193.7698	89.34503	121.6802	71.7661	87.38717	62.35826	84.1085	68.4295	53.37157	68.26001	53.77848
AC135178.	1.921815	0	2.997401	4.157171	14.3973	5.709435	2.549041	0.926027	0	8.646293	0	0.920078	1.028084	3.722881	0.978006	1.648904	4.105505	5.4608	2.688924
AC135279.	49.96719	63.0246	63.94455	89.37918	68.66407	53.66869	146.9947	83.34243	42.02236	44.19217	22.89034	116.8499	93.55568	57.70466	84.1085	84.09409	79.03097	110.1261	72.60094
AC135279.	0	9.003514	4.995668	5.196464	4.42994	10.27698	5.098081	1.852054	1.167288	0	0	2.760235	0	5.584322	4.890029	3.297807	9.237386	3.640534	8.066772
AC135279.	2.882723	12.00469	8.992203	2.078586	3.322455	17.12831	7.647122	14.81643	0	0.960699	16.86657	5.520469	13.3651	6.515042	10.75806	8.244518	12.31652	10.01147	4.48154
AC135352.	23.06178	22.00859	16.98527	11.43222	6.64491	14.84453	22.94137	10.1863	2.334575	28.82098	1.204755	10.12086	12.33701	11.16864	10.75806	0.824452	8.21101	56.42827	25.99293
AC135371.	0.960908	0	0	1.039293	0	1.141887	0	0.926027	0	0	0	0	5.140422	0	0	0	0	0	1.792616
AC135506.	344.0049	238.0929	236.7947	249.4303	314.5257	331.1472	309.2836	191.6876	536.9524	399.6509	390.3406	352.39	363.9419	274.5625	249.3915	214.3575	213.4863	411.3803	371.9678
AC135507.	13.45271	28.01093	10.99047	28.0609	12.18233	22.83774	11.04584	35.18903	31.51677	35.54587	39.75691	8.280704	9.252759	13.9608	7.824047	18.96239	11.29014	18.20267	12.54831
AC135507.	0	0	0	1.039293	0	1.141887	0	0	0	0	0	0.920078	0	0	0	0.824452	0	0	0
AC135584.	5.765445	1.00039	0	1.039293	4.42994	1.141887	1.69936	0.926027	0	3.842797	0	1.840156	3.084253	3.722881	7.824047	2.473355	0	0	6.274156
AC135586.	0.960908	3.001171	10.99047	0	1.107485	1.141887	0.84968	3.704108	0	3.842797	7.228529	0.920078	8.224675	0	4.890029	0	0	2.7304	0
AC135586.	0	0	0	0	2.21497	0	0	0	0	0	0	0	0	0	0	0	0	0	0
AC135628.	0	0	0	1.039293	2.21497	0	0	0	0	0	0	0	1.028084	0	0	0	0	0.910133	0.896308
AC135721.	0.960908	7.002733	0	0	3.322455	6.851322	2.549041	0	8.171014	0	2.40951	0.920078	0	0	0.978006	0.824452	4.105505	3.640534	15.23724
AC135721.	83.59896	28.01093	22.98007	24.94303	28.79461	36.54038	36.53625	25.92876	24.51304	31.70308	68.67103	55.20469	44.20763	30.71377	34.2302	42.8715	66.71446	28.21414	20.61508
AC135731.	82.63805	67.02616	4.995668	89.37918	16.61227	78.7902	33.98721	42.59724	0	51.87776	16.86657	86.48735	130.5667	115.4093	69.43841	112.1254	114.9541	72.81067	133.5499
AC135731.	8.648168	5.001952	0.999134	15.58939	15.50479	23.97963	10.19616	2.778081	21.01118	18.25329	33.73314	8.280704	7.196591	2.792161	4.890029	4.122259	19.50115	1.820267	12.54831
AC135776.	1.921815	0	0	0	1.107485	0	0	0	0	0.960699	2.40951	0.920078	0	0	0	0.824452	0	0	0.896308
AC135782.	137.4098	286.1117	204.8224	166.2868	126.2533	336.8567	426.5395	174.0931	291.8219	319.9129	410.8214	209.7778	294.0321	216.8578	187.7771	154.9969	171.4048	222.9827	289.5075
AC135782.	10.56998	8.003124	1.998267	5.196464	11.07485	14.84453	9.346483	39.81916	4.669151	19.21399	13.2523	25.76219	8.224675	20.47585	14.67009	14.84013	31.81766	11.83173	23.30401
AC135803.	111.4653	228.089	183.8406	228.6444	191.5949	129.0332	210.7207	50.00546	191.4352	148.9084	36.14265	171.1346	171.6901	125.6472	133.9868	67.60505	69.79359	22.75334	24.20031
AC135895.	0	1.00039	0	0	0	0	0	0	0	0	0	0	0	0	0	0	1.026376	0	0
AC135977.	21.13997	12.00469	11.9896	19.74656	8.859879	19.41208	28.03945	22.22465	32.68406	22.09608	24.0951	7.360626	15.42127	11.16864	23.47214	23.08465	30.79129	10.01147	18.82247
AC135983.	30.74904	39.01523	18.98354	43.6503	36.547	7.993209	33.13753	26.85478	31.51677	15.37119	13.2523	12.8811	15.42127	13.03008	15.64809	14.01568	19.50115	13.652	20.61508
AC135983.	1450.97	1423.556	1556.65	1160.89	1809.63	808.456	2121.652	1145.495	745.8969	1130.743	786.7049	1680.983	1701.48	1533.827	1850.387	1368.59	1228.572	1815.716	1574.813
AC135983.	24.9836	3.001171	8.992203	40.53242	29.90209	95.91851	33.13753	95.38078	7.003726	32.66377	45.78069	9.200782	4.112337	2.792161	4.890029	65.13169	39.0023	10.9216	8.963079
AC135983.	54.77173	3.001171	0.999134	30.13949	52.05179	60.52001	37.38593	88.8986	157.5838	35.54587	22.89034	21.1618	16.44935	18.61441	10.75806	42.04704	36.94955	9.101334	4.48154
AC135983.	0	2.000781	0.999134	1.039293	6.64491	1.141887	1.69936	9.26027	1.167288	0	0	0	3.084253	0.93072	0.978006	2.473355	0	2.7304	0
AC136285.	4.804538	1.00039	10.99047	2.078586	3.322455	3.425661	10.19616	4.630135	12.84017	0	14.45706	3.680313	3.084253	12.09936	1.956012	4.122259	3.079129	1.820267	0.896308
AC136285.	9.609075	2.000781	3.996535	1.039293	9.967364	4.567548	6.797442	0	4.669151	14.41049	2.40951	3.680313	0	7.445762	1.956012	8.244518	9.237386	0.910133	0
AC136424.	3.84363	6.002343	1.998267	7.275049	0	0	1.69936	3.704108	3.501863	11.52839	15.66181	1.840156	6.168506	0.93072	3.912023	2.473355	6.158258	0.910133	2.688924
AC136424.	5.765445	12.00469	5.994802	4.157171	15.50479	9.135096	4.248401	10.1863	8.171014	12.48909	8.433284	7.360626	18.50552	1.861441	0	1.648904	11.29014	5.4608	0
AC136428.	0	1.00039	0	0	0	0	0.84968	0	0	0	0	0	0	0	0	0	0	0	0
AC136428.	0	4.001562	0	0	0	0	0	0	0	0	0	0	1.028084	0	0	0	0	0	0
AC136428.	5.765445	0	0	0	0	1.141887	0	0	0	0	0	0	1.028084	0.93072	0.978006	0.824452	0	0	0
AC136443.	0	0	1.998267	0	0	0	0	0.926027	0	0	0	0	0	1.861441	0	0	0	0	0
AC136443.	3.84363	4.001562	4.995668	9.353635	4.42994	9.135096	7.647122	1.852054	7.003726	0.960699	4.81902	10.12086	2.056169	19.54513	15.64809	1.648904	6.158258	9.101334	4.48154
AC136443.	1.921815	0	0	3.117878	3.322455	3.425661	0	1.852054	2.334575	0	0	1.840156	4.112337	3.722881	4.890029	0	1.026376	0	1.792616
AC136443.	0.960908	1.00039	2.997401	0	0	1.141887	0	0	0	0.960699	0	0.920078	2.056169	0	0	0	0	0	0
AC136469.	22.10087	15.00586	7.993069	14.5501	6.64491	18.27019	5.947762	16.66849	22.17847	22.09608	22.89034	16.56141	24.67402	20.47585	10.75806	20.6113	18.47477	47.32694	31.37078
AC136469.	5.765445	0	3.996535	1.039293	3.322455	2.283774	2.549041	0	4.669151	0	0	2.760235	6.168506	2.792161	1.956012	4.946711	6.158258	0	5.377848
AC136475.	408.3857	316.1234	460.6006	273.334	199.3473	303.7419	308.4339	372.2629	791.4211	336.2447	438.5308	308.2262	308.4253	268.9782	251.3475	264.649	346.9152	323.0974	389.894
AC136475.	79.75532	157.0613	224.8051	171.4833	172.7676	141.594	214.9691	292.6245	77.04099	177.7294	132.523	229.0995	191.2237	201.9663	152.5689	225.0753	201.1697	427.7627	364.7973
AC136475.	147.0189	115.0449	178.8449	60.27898	203.7772	89.06719	96.01387	167.6109	472.7515	250.7425	544.5492	276.9435	262.1615	198.2434	187.7771	155.8214	154.9828	463.2579	447.2577
AC136475.	26.90541	18.00703	8.992203	12.47151	40.97694	13.70264	8.496802	23.15068	75.8737	61.48475	153.0039	23.92203	27.75828	9.307203	9.780058	16.48904	28.73854	43.6864	52.88217
AC136475.	0	0	0	0	3.322455	0	0.84968	0	0	0	2.40951	0	0	0	0	0	3.079129	0	2.688924
AC136475.	25.9445	23.00898	106.9073	40.53242	76.41646	55.95246	39.93497	20.37259	54.86252	48.03496	46.98544	75.44642	55.51656	67.94258	107.5806	51.94047	43.1078	141.0707	132.6536
AC136475.	3.84363	1.00039	0	9.353635	0	0	8.496802	4.630135	0	0	0	0	0	0	0	0	0	0.910133	0.896308
AC136475.	24.9836	16.00625	87.92376	24.94303	44.2994	6.851322	26.34009	36.11505	25.68033	18.25329	26.50461	64.40548	54.48847	37.22881	32.27419	23.08465	58.50345	72.81067	60.05263
AC136475.	11.53089	21.0082	20.98181	9.353635	9.967364	1.141887	5.947762	8.334243	10.50559	6.724895	4.81902	20.24172	16.44935	4.653602	13.69208	3.297807	14.36927	7.281067	10.7557
AC136475.	10.56998	18.00703	40.96448	30.13949	65.34161	36.54038	13.59488	6.482189	176.2604	54.75986	31.32363	26.68227	43.17954	39.09025	49.8783	10.71787	20.52753	35.4952	54.67478
AC136489.	3.84363	5.001952	12.98874	11.43222	3.322455	15.98642	17.84329	17.59451	16.34203	3.842797	4.81902	11.04094	8.224675	13.03008	6.846041	4.946711	10.26376	18.20267	3.585232
AC136601.	0.960908	0	0.999134	0	0	0	0	0	0	4.803496	0	0	2.056169	0	3.912023	0	0	0.910133	1.792616
AC136601.	0	0	0	0	4.42994	0	0	5.556162	1.167288	0	0	0	0	0.93072	0	0	0	0.910133	0
AC136603.	0	0	0	0	0	0	0	0	9.338302	0	0	0	4.112337	0	0	0	0	0	0
AC136604.	0	2.000781	0	1.039293	1.107485	0	2.549041	0	0	0	0	0.920078	2.056169	0	0	0	0	0	0
AC136604.	172.9634	114.0445	150.8692	193.3085	170.5527	139.3102	140.1972	124.0876	224.1192	178.6901	146.9801	192.2964	206.645	176.8369	147.6789	151.6991	146.7718	221.1624	179.2616
AC136604.	31.70995	12.00469	11.9896	7.275049	26.57964	17.12831	33.98721	25.92876	22.17847	17.29259	53.00921	41.40352	28.78636	11.16864	27.38416	20.6113	25.65941	26.39387	12.54831
AC136618.	0	0	0	0	0	0	0	0	0	0.960699	9.638039	0	0	0	0	0	0	0	0
AC136618.	0.960908	0	0.999134	0	0	4.567548	0	0	0	0	7.228529	0	0	0	0	0	0	0	0

AC136619.	0	0	0	0	0	0	0	0	0	0	0	0	0	0	0	0	1.026376	0.910133	0
AC136621.	34.59267	23.00898	16.98527	3.117878	4.42994	18.27019	9.346483	18.52054	10.50559	23.05678	13.2523	8.280704	23.64594	40.95169	16.6261	0	14.36927	0	0.896308
AC136624.	0	0	0	0	1.107485	0	0	0	0	0.960699	0	0.920078	0	0.93072	0	0	0	0.910133	0
AC136624.	0.960908	1.00039	10.99047	1.039293	5.537425	0	0.84968	0	0	0	1.204755	1.840156	1.028084	0.93072	0	3.297807	9.237386	0	3.585232
AC136624.	0	0	0	1.039293	4.42994	0	0	0	0	0	0	0	1.028084	0	0	0	0	0	0
AC136628.	0	0	0	1.039293	0	0	0	0	0	0.960699	0	0	1.028084	0	0	8.244518	1.026376	0	0
AC136628.	0	0	0.999134	1.039293	1.107485	1.141887	1.69936	0	0	0.960699	0	0	4.112337	0	0	0	0	1.820267	0
AC136628.	84.55986	92.03592	95.91683	156.9332	152.8329	161.0061	82.41898	149.0904	168.0894	183.4936	199.9893	110.4094	95.61185	167.5297	142.7889	253.9312	192.9587	213.8814	139.824
AC136628.	0.960908	3.001171	1.998267	1.039293	4.42994	0	0.84968	14.81643	0	2.882098	13.2523	1.840156	1.028084	0.93072	0.978006	0.824452	2.052753	2.7304	0.896308
AC136632.	53.81082	102.0398	68.94022	38.45383	97.45867	89.06719	33.13753	92.6027	175.0932	90.30573	159.0276	81.88696	64.76932	67.01186	66.5044	55.23827	54.39794	58.24854	57.36371
AC136632.	6.726353	10.0039	7.993069	2.078586	17.71976	11.41887	4.248401	37.96711	11.67288	13.44979	7.228529	19.32164	1.028084	7.445762	8.802052	4.946711	2.052753	1.820267	4.48154
AC136759.	0	12.00469	5.994802	6.235757	4.42994	9.135096	5.947762	1.852054	14.00745	0	0	0	0	0	0	0	2.052753	0.910133	0.896308
AC136944.	0.960908	0	1.998267	3.117878	0	1.141887	0	0	0	1.921399	0	0	1.028084	0	0	0	0	0	0
AC136944.	1.921815	0	0	4.157171	0	5.709435	0	0	3.501863	1.921399	0	1.840156	0	0.93072	0	0.824452	0	2.7304	0
AC136944.	0	0	0	0	1.107485	2.283774	0	0	0	0	3.614265	0	0	0	0	0	0	0	0
AC137055.	17.29634	3.001171	0	4.157171	2.21497	4.567548	0.84968	1.852054	12.84017	0.960699	0	0.920078	3.084253	0.93072	3.912023	3.297807	1.026376	4.550667	7.170464
AC137056.	11.53089	6.002343	15.98614	21.82515	9.967364	19.41208	26.34009	37.96711	7.003726	18.25329	22.89034	20.24172	15.42127	12.09936	4.890029	5.771163	8.21101	9.101334	18.82247
AC137056.	0	0	3.996535	0	0	0	0	0	0	0	0	0	0	0	0	0	0	0	0
AC137499.	0.960908	0	0	2.078586	0	0	0.84968	0	0	0	0	0	0	0	0	0	0	0	0
AC137527.	0	4.001562	0	3.117878	6.64491	3.425661	1.69936	0	4.669151	4.803496	0	3.680313	1.028084	0	0	0	0	0	0
AC137527.	0	0	0	0	0	0	0	2.778081	0	0	0	0	1.028084	0	0	0	0	0	0
AC137579.	0	0	0	0	0	0	0	0	0	0	0	0	1.028084	0.93072	0.978006	0	0	0	0
AC137579.	0.960908	0	1.998267	0	0	0	0.84968	7.408216	3.501863	0	7.228529	1.840156	9.252759	1.861441	7.824047	0	0	0	0.896308
AC137590.	0.960908	4.001562	0	1.039293	0	0	1.69936	0	3.501863	0	1.204755	1.840156	0	0	7.824047	3.297807	0	1.820267	0
AC137627.	0	1.00039	0	0	1.107485	0	0.84968	0	3.501863	3.842797	12.04755	4.600391	0	0.93072	0.978006	0.824452	0	0	0.896308
AC137630.	29.78813	31.0121	19.98267	20.78586	33.22455	13.70264	17.84329	38.89314	44.35693	22.09608	32.52838	28.52243	27.75828	33.50593	19.56012	41.22259	16.42202	36.40534	60.05263
AC137630.	56.69354	21.0082	64.94369	41.57171	35.43952	42.24982	56.92858	34.263	43.18965	60.52405	4.81902	40.48344	23.64594	39.09025	30.31818	45.34485	40.02867	63.70934	39.43755
AC137630.	0.960908	2.000781	5.994802	3.117878	11.07485	1.141887	0	5.556162	14.00745	1.921399	3.614265	0.920078	2.056169	0.93072	2.934017	0	0	0	1.792616
AC137695.	35.55358	6.002343	4.995668	1.039293	5.537425	11.41887	12.7452	8.334243	7.003726	4.803496	2.40951	13.80117	11.30893	6.515042	3.912023	11.54233	4.105505	0.910133	7.170464
AC137695.	6.726353	9.003514	22.98007	20.78586	13.28982	10.27698	49.28145	7.408216	38.5205	14.41049	7.228529	11.96102	7.196591	8.376483	11.73607	14.01568	10.26376	3.640534	1.792616
AC137723.	139.3316	240.0937	283.754	143.4224	263.5814	239.7963	177.5832	236.1369	254.4687	223.8429	352.9932	86.48735	113.0893	176.8369	188.7551	117.8966	105.7168	144.7112	116.52
AC137723.	28.82723	21.0082	13.98787	16.62868	37.65449	19.41208	11.89552	17.59451	58.36439	24.01748	26.50461	9.200782	28.78636	18.61441	17.6041	9.893422	12.31652	8.191201	11.652
AC137761.	6.726353	24.00937	6.993936	5.196464	12.18233	3.425661	14.44456	5.556162	2.334575	4.803496	4.81902	6.440548	14.39318	10.23792	14.67009	5.771163	8.21101	4.550667	5.377848
AC137767.	252.7187	172.0672	174.8484	129.9116	151.7254	125.6076	128.3017	155.5725	570.8037	261.3102	365.0407	194.1365	195.336	155.4303	137.8988	166.5393	172.4312	314.9062	262.6182
AC137770.	1.921815	12.00469	1.998267	4.157171	9.967364	1.141887	6.797442	12.03835	0	9.606993	9.638039	6.440548	13.3651	3.722881	7.824047	2.473355	2.052753	10.01147	2.688924
AC137794.	0	10.0039	4.995668	0	21.04221	0	0	7.408216	0	0.960699	6.023774	0.920078	0	0	0	0	0	3.640534	2.688924
AC137800.	0	0	0	0	0	0	0	0	0	1.921399	0	0	0	0	0	0	0	0	0
AC137803.	0	1.00039	0.999134	0	1.107485	1.141887	0	0	0	1.921399	0	0	0	5.584322	0	0	0	0	0.896308
AC137810.	1.921815	0	0	1.039293	0	1.141887	4.248401	0.926027	0	0.960699	0	2.760235	2.056169	0	0	0	0	0	0
AC137834.	0	0	2.997401	3.117878	6.64491	0	0.84968	0	9.338302	6.724895	0	6.440548	1.028084	0.93072	0	9.893422	7.184634	4.550667	0
AC137894.	24.02269	8.003124	8.992203	0	11.07485	7.993209	12.7452	19.44657	0	15.37119	25.29985	7.360626	8.224675	1.861441	9.780058	3.297807	4.105505	9.101334	8.963079
AC137932.	107.6216	95.03709	116.8986	145.501	129.5757	107.3374	127.452	130.5698	93.38302	117.2053	148.1848	114.0897	168.6058	80.97267	171.151	124.4922	127.2707	135.6099	157.7502
AC137932.	84.55986	129.0504	80.92983	96.65423	56.48173	109.6212	72.22282	75.93422	119.0633	80.69874	125.2945	75.44642	69.90974	106.1021	97.80058	87.39189	123.1652	87.37281	81.56402
AC137932.	384.363	442.1726	537.5339	445.8566	421.9518	465.8899	450.3305	530.6135	463.4132	713.7995	671.0485	406.6746	382.4474	445.815	364.7962	328.1318	358.2053	650.7454	574.5334
AC137936.	0	4.001562	0	0	0	0	0	0	0	1.167288	0.960699	0	5.520469	2.056169	2.792161	3.912023	0.824452	2.052753	1.820267	0
AC137936.	52.84991	19.00742	43.96188	34.29666	28.79461	41.10793	22.94137	42.59724	22.17847	46.11356	8.433284	38.64329	24.67402	23.26801	21.51613	27.20691	28.73854	39.13574	25.99293
AC138024.	0	0	0	0	0	0	0	0.84968	0	0	0	0	0.920078	0	0	0	0.824452	0	0	0
AC138028.	24.02269	33.01288	17.98441	7.275049	16.61227	21.69585	11.04584	33.33697	58.36439	33.62447	97.58515	12.8811	27.75828	21.40657	22.49413	11.54233	22.58028	9.101334	34.95601
AC138028.	43.24084	93.03631	77.93243	67.55403	81.95388	58.23624	45.88273	44.4493	171.5913	87.42363	260.2271	24.84211	60.65698	68.8733	53.79032	38.74924	85.18923	94.65388	111.1422
AC138028.	375.7148	367.1433	367.6812	360.6346	466.2512	403.0861	420.5917	436.1587	394.5433	456.3321	478.2877	368.9514	363.9419	550.0557	474.3328	367.7055	401.3131	586.1259	443.6724
AC138028.	0	2.000781	0	0	0	1.141887	1.69936	0.926027	0	0	8.433284	2.760235	1.028084	2.792161	0.978006	0.824452	6.158258	2.7304	7.170464
AC138028.	91.28622	52.0203	59.94802	58.2004	65.34161	62.80379	46.73241	27.78081	92.21573	50.91706	137.3421	32.20274	86.35909	43.74385	63.57038	67.60505	52.34519	80.09174	103.0754
AC138035.	90.32531	300.1171	179.8441	136.1474	141.7581	52.5268	160.5896	198.1698	114.3942	507.2492	575.8728	207.0176	179.9148	158.2225	134.9648	176.4327	149.8509	376.7952	380.9309
AC138035.	0	0	0	0	0	0	0	0	0	0	3.614265	0	0	0	0	0	0	0	0
AC138058.	0.960908	0	0	0	0	0	0	0	2.334575	0	6.023774	2.760235	1.028084	0.93072	1.956012	0.824452	0	0	0
AC138089.	0	0	0	2.078586	0	0	0	0	0	3.842797	0	0	0	0.93072	0	0	0	0	0
AC138123.	30.74904	63.0246	38.96621	25.98232	28.79461	36.54038	30.58849	25.00273	50.19337	31.70308	36.14265	29.4425	31.87062	36.29809	46.94428	40.39814	27.71216	40.04587	16.13354
AC138123.	12.4918	18.00703	35.96881	4.157171	23.25718	29.68906	45.88273	0	7.003726	48.99566	24.0951	70.84602	47.29188	39.09025	23.47214	45.34485	34.89679	38.2256	60.94894
AC138123.	0	0	7.993069	2.078586	0	0	10.19616	0.926027	0	3.842797	1.204755	0	9.252759	0	1.956012	2.473355	1.026376	2.7304	0
AC138150.	77.83351	85.03319	144.8744	162.1297	84.16885	37.68227	144.4456	79.63833	18.6766	65.32755	46.98544	126.9708	115.1454	97.72563	109.5367	111.301	96.47937	107.3957	86.04556
AC138150.	98.97348	138.0539	176.8467	48.84676	197.1323	119.8981	86.66738	98.15887	261.4725	128.7337	195.1703	158.2535	190.1956	153.5689	140.8328	99.75867	134.4553	204.78	185.5357
AC138207.	135.488	72.02811	82.92809	95.61493	43.19191	37.68227	71.37314	75.93422	66.5354	57.64196	77.10431	103.0488	98.6961	111.6864	69.43841	135.2101	64.6617	143.8011	79.77141

AC138207.	71.10716	70.02733	42.96275	65.47544	46.51437	55.95246	42.48401	58.3397	80.54285	86.46293	16.86657	44.16376	60.65698	51.18962	50.8563	35.45143	46.18693	70.08027	41.23017
AC138207.	14.41361	15.00586	11.9896	14.5501	6.64491	12.56076	20.39233	12.03835	39.68778	14.41049	34.93789	16.56141	9.252759	15.82225	12.71408	18.13794	4.105505	21.8432	20.61508
AC138207.	824.4587	382.1491	392.6595	797.1376	439.6715	424.782	543.7954	507.4628	470.417	556.2449	530.0921	822.5499	614.7945	560.2936	590.7155	1431.248	952.4772	1172.252	618.4525
AC138207.	452.5874	143.0558	79.93069	527.9607	173.8751	191.837	187.7793	284.2903	289.4874	269.9565	207.2178	398.3939	294.0321	203.8277	185.8211	666.1571	413.6296	526.0571	219.5954
AC138207.	270.9759	206.0804	264.7704	333.613	179.4126	165.5736	168.2367	334.2958	189.1006	160.4368	104.8137	283.3841	181.9709	166.5989	187.7771	540.8404	329.4668	322.1872	391.6866
AC138207.	19.21815	32.01249	22.98007	78.98625	53.15928	31.97284	39.08529	60.19176	2.334575	16.33189	37.3474	46.92399	34.95487	26.99089	25.42815	47.81821	43.1078	70.99041	53.77848
AC138207.	608.2545	434.1695	526.5434	406.3635	436.3491	441.9103	667.8487	534.3176	592.9822	625.4152	524.0684	567.6883	501.7052	539.8178	565.2874	441.0817	419.7879	491.472	500.1398
AC138230.	26.90541	37.01445	55.95149	40.53242	50.94431	44.53359	30.58849	57.41368	53.69524	81.65944	65.05676	69.00587	63.74123	54.9125	82.15249	43.69595	47.21331	58.24854	57.36371
AC138304.	1.921815	0	0.999134	1.039293	3.322455	0	1.69936	0	0	0	0	3.680313	2.056169	0	0	0	0	5.4608	0
AC138305.	218.126	352.1374	220.8085	323.2201	170.5527	285.4718	146.9947	229.6547	114.3942	141.2228	45.78069	200.5771	223.0943	373.2188	253.3035	359.461	341.7833	131.0592	380.9309
AC138305.	0	0	0	0	0	0	4.248401	0	9.338302	1.921399	1.204755	0	0	0.93072	0	0.824452	0	0	3.585232
AC138305.	0	1.00039	0.999134	0	0	1.141887	0.84968	0	0	0	1.204755	0.920078	0	0	0	0.824452	0	0	1.792616
AC138331.	0	1.00039	1.998267	0	0	3.425661	1.69936	0	7.003726	0	7.228529	3.680313	2.056169	2.792161	1.956012	1.648904	6.158258	0	3.585232
AC138356.	81.67714	86.03358	91.9203	95.61493	109.641	54.81058	116.4062	61.11778	103.8886	71.09174	104.8137	58.88501	78.13441	86.55699	72.37243	111.301	125.2179	130.1491	115.6237
AC138356.	239.266	304.1187	248.7843	323.2201	234.7868	167.8574	220.9169	330.5917	190.2679	283.4063	255.408	181.2554	233.3752	216.8578	237.6554	183.0283	200.1434	179.2963	187.3284
AC138356.	0	0	0	0	0	0	0	0	0	6.724895	7.228529	1.840156	1.028084	0	0	4.946711	0	0	2.688924
AC138360.	0	2.000781	1.998267	1.039293	0	1.141887	0	0	0	0	0	0	3.084253	0	0	0	1.026376	0	1.792616
AC138392.	174.8852	82.03202	68.94022	74.82908	56.48173	116.4725	215.8188	121.3095	18.6766	45.15286	25.29985	90.16767	47.29188	61.42754	98.77859	97.28532	98.53212	96.47414	72.60094
AC138393.	2338.849	1848.722	2356.956	2321.78	1712.172	1139.603	1814.067	2109.49	1687.898	1711.966	1495.101	2304.796	2497.217	1715.318	1832.783	2094.932	2087.649	2194.332	1638.451
AC138393.	27.86632	20.00781	24.97834	12.47151	26.57964	19.41208	44.18337	25.92876	18.6766	24.97818	21.68559	24.84211	33.92678	11.16864	12.71408	18.13794	13.34289	22.75334	21.51139
AC138393.	34.59267	49.01913	101.9116	60.27898	75.30897	49.10114	53.52986	88.8986	3.501863	62.44545	45.78069	136.1716	94.58336	101.4485	75.30645	49.46711	42.08143	74.63094	61.84525
AC138409.	14.41361	4.001562	38.96621	22.86444	38.76197	28.54718	12.7452	17.59451	35.01863	39.38867	42.16642	61.64524	49.34805	42.81313	22.49413	18.96239	44.13418	33.67494	39.43755
AC138409.	540.9909	405.1581	643.4421	456.2495	496.1532	406.5118	480.919	538.9477	331.5097	429.4326	418.0499	547.4465	588.0643	507.2426	738.3944	483.9532	427.9989	513.3152	492.0731
AC138430.	56.69354	170.0664	69.93936	81.06484	126.2533	114.1887	139.3476	97.23284	187.9333	200.7861	143.3658	54.28462	51.40422	129.3701	123.2287	75.02512	94.42662	69.17014	127.2757
AC138466.	4.804538	6.002343	0.999134	0	15.50479	6.851322	3.398721	0	5.836439	11.52839	27.70936	0	10.28084	11.16864	5.868035	7.420066	13.34289	10.01147	10.7557
AC138466.	0	0	7.993069	0	1.107485	4.567548	0	3.704108	0	0	1.204755	0	0	0	0	0	0	0.910133	0.896308
AC138466.	7.68726	1.00039	3.996535	14.5501	0	1.141887	5.947762	0.926027	5.836439	1.921399	12.04755	4.600391	4.112337	9.307203	0	0.824452	0	10.01147	7.170464
AC138466.	16.33543	10.0039	2.997401	7.275049	5.537425	17.12831	11.89552	27.78081	1.167288	47.07426	53.00921	28.52243	22.61786	7.445762	20.53812	24.73355	27.71216	21.8432	22.4077
AC138470.	140.2925	111.0433	91.9203	155.8939	162.8003	163.2898	90.06611	66.67395	148.2455	148.9084	153.0039	80.04681	127.4825	148.9152	111.4927	93.98751	85.18923	111.0363	51.08955
AC138473.	14.41361	5.001952	6.993936	39.49313	7.752394	11.41887	18.69297	10.1863	11.67288	26.89958	0	23.92203	17.47743	18.61441	3.912023	14.01568	10.26376	14.56213	5.377848
AC138474.	0.960908	3.001171	0	1.039293	0	0	0	0	0	0	6.023774	3.680313	0	0	0.978006	0	0	0	0
AC138512.	0	0	2.997401	1.039293	1.107485	0	0.84968	3.704108	0	0	0	0	1.028084	0	0	0.824452	0	2.7304	1.792616
AC138517.	3.84363	0	0	0	0	0	0	0	9.338302	0	0	0	0	0	0	0	0	0	0
AC138517.	0	0	0	0	0	0	3.398721	0	0	0.960699	0	0	0	0	0	0	5.131881	0	1.792616
AC138625.	0	2.000781	0	0	0	0	1.69936	0	7.003726	0.960699	0	0	1.028084	0.93072	0	0	4.105505	0.910133	1.792616
AC138625.	0	0	0	0	0	0	0	3.704108	0	0	0	0.920078	2.056169	0	0	1.648904	4.105505	0	0
AC138627.	32.67086	19.00742	54.95235	16.62868	23.25718	25.12151	41.63433	17.59451	36.18592	11.52839	14.45706	49.68422	39.06721	26.06017	30.31818	62.65834	36.94955	57.33841	53.77848
AC138627.	0	0	0	1.039293	0	0	1.69936	0	0	0	0	0.920078	1.028084	0	2.934017	0	0	1.820267	5.377848
AC138645.	10.56998	9.003514	15.98614	8.314342	16.61227	4.567548	7.647122	4.630135	5.836439	10.56769	4.81902	11.04094	9.252759	7.445762	7.824047	1.648904	1.026376	2.7304	3.585232
AC138646.	9.609075	16.00625	9.991337	7.275049	8.859879	10.27698	11.89552	4.630135	7.003726	1.921399	3.614265	11.96102	5.140422	10.23792	3.912023	4.946711	9.237386	12.74187	14.34093
AC138649.	835.0286	1151.449	876.2402	1661.829	748.6598	1365.697	1223.54	904.7284	960.6778	1211.442	1032.475	778.3862	780.316	1034.961	941.8196	1389.201	1410.241	679.8697	1009.243
AC138649.	48.04538	20.00781	18.98354	15.58939	27.68712	63.94567	39.08529	63.89587	50.19337	51.87776	120.4755	58.88501	51.40422	26.06017	27.38416	21.43575	20.52753	30.94454	31.37078
AC138649.	6.726353	2.000781	0	0	2.21497	0	0.84968	0	0	0.960699	2.40951	0	0	0	0	0	0	0	0
AC138649.	0	1.00039	0	1.039293	0	0	0	0.926027	0	1.921399	0	0	0	0	0	0	0	0	1.792616
AC138649.	2.882723	17.00664	5.994802	4.157171	1.107485	3.425661	11.89552	3.704108	1.167288	13.44979	6.023774	11.04094	7.196591	9.307203	6.846041	1.648904	8.21101	0.910133	6.274156
AC138696.	19.21815	9.003514	13.98787	10.39293	15.50479	13.70264	2.549041	2.778081	52.52795	18.25329	24.0951	8.280704	9.252759	14.89152	3.912023	6.595615	15.39564	4.550667	9.859387
AC138761.	0	0	0	0	0	1.141887	0	0	0	0	0	0	0	6.515042	0	0	0	0	0.896308
AC138761.	0	0	0	0	0	0	0	0	0	0	0	0	0	0.93072	0	0	0	0	4.48154
AC138761.	47.08447	84.0328	26.97661	31.17878	56.48173	23.97963	58.62794	43.52327	7.003726	7.685594	9.638039	28.52243	13.3651	14.89152	16.6261	10.71787	12.31652	33.67494	55.57109
AC138781.	0	0	0	1.039293	1.107485	0	0	0	0	0	0	0	0	0	0	0	0	0.910133	0
AC138811.	13.45271	14.00547	11.9896	12.47151	11.07485	9.135096	16.9936	1.852054	8.171014	16.33189	21.68559	25.76219	14.39318	16.75297	17.6041	10.71787	4.105505	24.5736	11.652
AC138819.	0.960908	12.00469	0	9.353635	2.21497	5.709435	5.098081	9.26027	15.17474	4.803496	3.614265	3.680313	3.084253	1.861441	5.868035	0	4.105505	0.910133	1.792616
AC138832.	0	0	0	0	2.21497	2.283774	0	4.630135	0	0	0	0	0	0	0	0	4.105505	0	0
AC138866.	65.34171	68.02655	108.9056	90.41847	100.7811	81.07398	102.8113	120.3835	70.03726	103.7555	50.5997	138.9318	150.1003	107.0328	149.6349	53.58937	80.05735	143.8011	207.9434
AC138866.	46.12356	47.01835	43.96188	36.37525	99.67364	49.10114	59.47762	106.4931	14.00745	48.03496	46.98544	77.28657	79.1625	58.63538	52.81231	46.99375	56.45069	92.83361	111.1422
AC138869.	0	0	0	0	0	0	0	0	0	0	4.81902	0	0	0	0	0	0	0	0
AC138894.	27.86632	4.001562	15.98614	4.157171	16.61227	20.55397	21.24201	4.630135	16.34203	6.724895	6.023774	22.08188	44.20763	5.584322	9.780058	6.595615	6.158258	7.281067	10.7557
AC138894.	0	0	1.998267	2.078586	0	4.567548	10.19616	5.556162	0	6.724895	4.81902	0.920078	0	0.93072	0	3.297807	11.29014	0.910133	22.4077
AC138894.	0	0	0	0	0	0	0	0	0	0	0	0	0	0	0	0	0	2.7304	0
AC138904.	7.68726	7.002733	29.97401	18.70727	9.967364	26.2634	40.78465	12.96438	18.6766	13.44979	18.07132	12.8811	2.056169	4.653602	22.49413	34.62698	32.84404	21.8432	38.54124
AC138904.	10.56998	29.01132	23.97921	28.0609	9.967364	50.24303	33.13753	21.29862	14.00745	33.62447	110.8374	21.1618	12.33701	31.64449	28.36217	17.31349	46.18693	33.67494	48.40063
AC138907.	0.960908	8.003124	0.999134	0	6.64491	5.709435	3.398721	8.334243	4.669151	4.803496	4.81902	3.680313	3.084253	1.861441	4.890029	2.473355	2.052753	3.640534	2.688924

AC138907.	0	0	0	1.039293	0	0	0	0	1.167288	0.960699	0	0	0	0	0.978006	0	0	0	0
AC138907.	0	0	0	0	0	0	0	0	0	0	0	0	0	0	0	0	2.052753	0	0
AC138907.	1.921815	1.00039	0	0	3.322455	0	0.84968	0	0	0	0	0.920078	1.028084	0	0	0.824452	0	0.910133	0
AC138907.	0	1.00039	0	0	0	0	0	0	0	0	0	4.600391	1.028084	0	0.978006	0	0	1.820267	0.896308
AC138932.	113.3871	100.039	108.9056	60.27898	121.8233	79.93209	30.58849	100.9369	130.7362	178.6901	414.4357	104.8889	149.0722	105.1714	70.41642	48.64266	80.05735	141.9808	96.80126
AC138932.	0	20.00781	16.98527	14.5501	18.82724	2.283774	9.346483	1.852054	9.338302	5.764196	0	11.04094	8.224675	20.47585	17.6041	4.946711	10.26376	10.01147	8.066772
AC138932.	0.960908	16.00625	20.98181	13.51081	67.55658	34.25661	11.04584	0	45.52422	92.22713	232.5177	44.16376	60.65698	71.66546	30.31818	12.36678	15.39564	27.304	16.13354
AC138932.	0	3.001171	0	2.078586	0	0	2.549041	0	0	0	0	0	0	0	1.956012	0	0	4.550667	7.170464
AC138932.	24.02269	50.01952	114.9004	76.90766	110.7485	44.53359	48.43177	33.33697	40.85507	70.13105	103.6089	84.6472	70.93782	74.45762	79.21847	28.03136	28.73854	53.69787	75.28987
AC138951.	5.765445	18.00703	170.8519	94.57564	36.547	10.27698	20.39233	13.89041	33.85134	23.05678	30.11887	291.6648	285.8075	114.4786	159.4149	37.92478	22.58028	14.56213	12.54831
AC138951.	1.921815	8.003124	1.998267	10.39293	0	6.851322	0	3.704108	1.167288	3.842797	7.228529	3.680313	6.168506	3.722881	7.824047	9.893422	0	0	3.585232
AC138956.	81.67714	63.0246	80.92983	56.12181	60.91167	70.79699	55.22922	74.08216	78.20828	65.32755	61.4425	54.28462	59.62889	60.49682	58.68035	86.56744	85.18923	80.09174	62.74156
AC138956.	167.1979	131.0511	242.7895	156.9332	197.1323	178.1344	210.7207	220.3944	54.86252	164.2796	133.7278	202.4172	276.5547	125.6472	171.151	132.7367	157.0356	168.3747	179.2616
AC138965.	2.882723	0	5.994802	0	4.42994	0	5.098081	0	0	0	0	8.280704	4.112337	0	0	3.297807	0	0	0.896308
AC138965.	0.960908	1.00039	1.998267	0	0	0	0	0	0.926027	0	0	0	0.920078	0	3.722881	0	3.297807	7.184634	0	3.585232
AC138969.	251.7578	63.0246	457.6032	222.4087	261.3664	254.6408	62.02666	234.2848	473.9188	582.1837	619.244	305.466	317.6781	402.0712	387.2903	128.6145	170.3785	166.5544	138.0314
AC138969.	682.2443	548.214	334.7098	232.8016	472.8961	444.194	270.1983	359.2985	464.5805	572.5768	863.8092	984.4837	764.8948	547.2635	455.7507	374.3011	498.8189	649.8353	738.5578
AC138969.	0	0	0	2.078586	2.21497	0	0	0	0	0	3.614265	0	0	0	0	0	0	0	0
AC138969.	0	1.00039	0	1.039293	0	0	0	0	0	0	0	0	0	0	0	0	0	0.910133	3.585232
AC139085.	0	0	0	0	0	0	0	0	0	0	0	0	4.112337	0	0	0	0	0	0
AC139099.	2.882723	1.00039	4.995668	6.235757	2.21497	0	3.398721	3.704108	3.501863	10.56769	20.48083	13.80117	1.028084	2.792161	0	3.297807	3.079129	5.4608	3.585232
AC139099.	1.921815	12.00469	2.997401	5.196464	0	6.851322	1.69936	0	0	2.882098	2.40951	9.200782	4.112337	7.445762	0	0	2.052753	1.820267	0
AC139099.	0	4.001562	0.999134	0	0	0	0	0	3.704108	0	2.882098	0	0	0	0	0.978006	0.824452	1.026376	1.820267	5.377848
AC139099.	0	0	0	0	0	0	0.84968	3.704108	2.334575	0	0	0	0	0	0	0	0	0.910133	0
AC139100.	70.14625	174.0679	129.8874	145.501	99.67364	103.9117	178.4329	88.8986	241.6286	127.773	127.704	134.3314	127.4825	183.3519	192.6671	86.56744	58.50345	59.15867	68.1194
AC139100.	153.7452	84.0328	134.883	139.2652	183.8425	191.837	91.76547	181.5013	331.5097	185.415	215.6511	155.4932	103.8365	128.4394	107.5806	140.9813	113.9278	121.0477	108.4533
AC139103.	5.765445	7.002733	13.98787	5.196464	0	11.41887	3.398721	1.852054	10.50559	6.724895	28.91412	10.12086	10.28084	13.9608	9.780058	9.893422	17.4484	19.1128	12.54831
AC139149.	16.33543	2.000781	0	25.98232	5.537425	10.27698	27.18977	21.29862	9.338302	9.606993	31.32363	17.48149	38.03912	7.445762	4.890029	22.2602	16.42202	20.93307	19.71877
AC139256.	343.044	173.0675	277.7592	169.4047	335.5679	365.4038	348.3689	193.5397	110.8923	443.8431	337.3314	126.0507	153.1846	501.6582	537.9032	166.5393	198.0906	138.3403	138.0314
AC139272.	3.84363	0	0.999134	0	6.64491	1.141887	2.549041	2.778081	3.501863	4.803496	3.614265	2.760235	4.112337	2.792161	6.846041	1.648904	2.052753	4.550667	3.585232
AC139426.	24.02269	78.03045	18.98354	31.17878	25.47215	34.25661	59.47762	40.74519	12.84017	77.81664	81.92333	76.36649	55.51656	40.95169	42.05425	136.0346	145.7454	50.05734	51.08955
AC139426.	0	6.002343	0.999134	6.235757	1.107485	7.993209	0.84968	6.482189	0	10.56769	51.80446	12.8811	10.28084	13.03008	11.73607	4.122259	0	0.910133	11.652
AC139451.	4.804538	0	0	0	0	0	0	0.926027	0	0	0	0	1.028084	0	0	0.824452	6.158258	0	0
AC139453.	23.06178	34.01327	26.97661	13.51081	60.91167	36.54038	22.09169	22.22465	127.2344	62.44545	63.85201	25.76219	22.61786	40.95169	37.16422	27.20691	27.71216	22.75334	35.85232
AC139453.	10.56998	65.02538	35.96881	50.92535	33.22455	25.12151	56.0789	20.37259	30.34948	1.921399	14.45706	80.04681	72.99399	61.42754	57.70234	28.03136	53.37157	60.06881	108.4533
AC139453.	21.13997	163.0636	178.8449	80.02554	127.3608	106.1955	141.0469	105.5671	192.6025	110.4804	68.67103	126.9708	181.9709	149.846	162.349	84.91854	82.1101	147.4416	197.1877
AC139491.	10.56998	10.0039	5.994802	15.58939	4.42994	25.12151	22.94137	0.926027	23.34575	22.09608	32.52838	11.96102	20.56169	12.09936	19.56012	11.54233	9.237386	20.93307	14.34093
AC139491.	4.804538	39.01523	22.98007	28.0609	40.97694	42.24982	28.03945	18.52054	59.53167	49.95636	32.52838	32.20274	35.98295	35.36737	35.20821	26.38246	32.84404	28.21414	11.652
AC139491.	0	2.000781	0	2.078586	0	2.283774	0	1.852054	0	0	0	0	0	0	0	0	1.026376	0	0
AC139491.	1.921815	2.000781	0	0	8.859879	4.567548	0.84968	0	0	2.882098	2.40951	0	0	0	0	0.824452	0	1.820267	0
AC139491.	0	1.00039	0	2.078586	0	5.709435	0	0	0	0	0	0	2.056169	0	0.978006	0	0	0	0
AC139493.	0.960908	2.000781	0	2.078586	0	0	0.84968	7.408216	0	0	1.204755	0.920078	0	3.722881	0.978006	0	1.026376	0	0
AC139493.	18.25724	28.01093	33.97054	9.353635	29.90209	35.3985	73.0725	27.78081	23.34575	48.03496	51.80446	49.68422	31.87062	56.77394	28.36217	23.9091	51.31881	29.12427	53.77848
AC139495.	42.27993	35.01367	64.94369	46.76817	37.65449	34.25661	9.346483	64.82189	24.51304	31.70308	22.89034	78.20665	32.8987	67.01186	110.5147	137.6835	51.31881	130.1491	83.35664
AC139495.	854.2468	421.1644	528.5417	374.1454	569.2472	588.0718	33.13753	550.0601	2684.762	651.3541	583.1014	1296.39	821.4394	826.4796	933.0176	3788.356	3133.527	2385.46	1977.255
AC139530.	62.45899	57.02225	66.94196	34.29666	136.2206	75.36454	51.83049	75.00819	263.807	193.1005	306.0077	89.24759	93.55568	70.73474	78.24047	45.34485	42.08143	103.7552	68.1194
AC139697.	0	0	0	4.157171	0	0	0	0	0	0	0	0	0	0	0	0.824452	0	0	0
AC139700.	0	1.00039	0.999134	0	4.42994	0	3.398721	3.704108	0	0	0	8.280704	3.084253	0	0	0.824452	0	3.640534	0.896308
AC139712.	0	1.00039	0	0	1.107485	0	0	0	0	0	0	0	0	0	0	0	0	0	0
AC139713.	73.98988	116.0453	91.9203	130.9509	102.9961	87.9253	160.5896	89.82462	123.7325	80.69874	99.99465	113.1696	115.1454	184.2826	149.6349	131.9123	122.1388	97.38428	118.3126
AC139718.	0	0	8.992203	6.235757	0	1.141887	0	0	0	0	0	0	0	0	0	0.824452	2.052753	0	1.792616
AC139718.	0	0	0	0	3.322455	1.141887	0	0	0	0	0	0.920078	0	0	0	0	0	0	0
AC139720.	4.804538	0	0	6.235757	0	0	0.84968	0	12.84017	0	8.433284	4.600391	3.084253	1.861441	0	1.648904	0	11.83173	5.377848
AC139720.	0	0	0	0	0	0	1.69936	0	0	0	0	0	0	0.93072	4.890029	3.297807	0	0	0
AC139722.	0	1.00039	0	0	3.322455	0	0.84968	0	0	0	0	0.920078	1.028084	0	0	0	0	0	0
AC139749.	43.24084	15.00586	13.98787	44.68959	19.93473	25.12151	24.64073	31.48492	46.69151	33.62447	36.14265	27.60235	15.42127	38.15953	28.36217	33.80253	52.34519	68.26001	36.74863
AC139768.	433.3693	528.2062	521.5478	676.5796	539.3452	543.5382	666.1493	493.5724	751.7333	667.686	643.3391	420.4758	459.5537	489.5589	458.6847	512.809	533.7157	381.3459	419.4721
AC139769.	293.0768	321.1253	604.4759	425.0707	396.4796	412.2212	492.8145	362.0766	137.74	213.2752	161.4372	372.6317	354.6891	265.2553	310.0278	290.207	281.2271	275.7704	236.6253
AC139769.	13.45271	34.01327	10.99047	29.1002	17.71976	21.69585	53.52986	15.74246	21.01118	10.56769	19.27608	5.520469	9.252759	51.18962	8.802052	18.13794	23.60665	17.29253	11.652
AC139769.	0.960908	5.001952	1.998267	5.196464	12.18233	14.84453	0.84968	1.852054	2.334575	1.921399	0	9.200782	7.196591	1.861441	8.802052	4.946711	6.158258	5.4608	0
AC139783.	0.960908	0	0	4.157171	0	0	0.84968	0.926027	0	0	0	2.760235	1.028084	0	0	0	1.026376	0	6.274156
AC139783.	0	0	0.999134	0	0	0	5.098081	0	0	3.842797	8.433284	0	0	0.93072	2.934017	0.824452	0	0.910133	0

AC139792.	0	2.000781	3.996535	1.039293	0	1.141887	0.84968	0	7.003726	0.960699	0	4.600391	0	0.93072	0.978006	4.122259	4.105505	5.4608	0
AC139792.	3.84363	2.000781	0	0	9.967364	0	9.346483	2.778081	0	2.882098	3.614265	2.760235	3.084253	3.722881	14.67009	6.595615	4.105505	13.652	5.377848
AC139792.	0	0	0	0	0	0	0	0.926027	2.334575	0	0	0.920078	1.028084	1.861441	0	0	0	0	0
AC139795.	260.4059	351.137	447.6119	408.4421	405.3395	452.1873	633.0118	468.5697	183.2642	199.8254	157.8229	587.93	481.1435	486.7667	468.4648	302.5738	329.4668	392.2675	487.5915
AC139795.	473.7274	596.2327	551.5218	595.5148	589.182	567.5178	553.9915	683.408	520.6103	506.2885	484.3115	574.1288	560.306	464.4294	467.4868	517.7557	615.8258	535.1584	648.927
AC139795.	106.6607	147.0574	109.9047	77.94696	73.094	90.20907	73.0725	89.82462	186.766	134.4979	186.737	165.6141	120.2859	101.4485	126.1628	58.53608	44.13418	80.09174	89.63079
AC139887.	32.67086	25.00976	27.97574	17.66798	31.00958	17.12831	33.13753	38.89314	17.50932	39.38867	62.64725	72.68618	47.29188	57.70466	59.65836	31.32917	27.71216	60.06881	61.84525
AC139887.	418.9557	455.1776	615.4663	275.4126	495.0458	344.8499	457.9776	678.7778	197.2716	405.4151	293.9602	551.1269	559.2779	589.146	641.5718	371.8278	394.1285	506.9443	579.9112
AC139887.	0	0	3.996535	0	0	0	0	3.704108	0	0	0	0.920078	0	0	0	0	1.026376	3.640534	0.896308
AC139887.	161.4325	165.0644	266.7687	129.9116	203.7772	77.64832	203.0736	235.2109	141.2418	210.3931	232.5177	186.7759	178.8867	208.4813	222.0073	136.0346	150.8773	257.5678	260.8256
AC140076.	0	0	0	0	1.107485	0	0	0	0	0	0	3.680313	1.028084	0.93072	0	0.824452	0	0	0
AC140113.	1.921815	0	0	0	0	0	0	0	0	0	0	0	0	0	0	0	0	0	0
AC140118.	10.56998	5.001952	12.98874	1.039293	4.42994	0	8.496802	0.926027	0	6.724895	1.204755	3.680313	6.168506	4.653602	0.978006	3.297807	0	20.02294	7.170464
AC140125.	12.4918	6.002343	9.991337	2.078586	35.43952	139.3102	26.34009	41.67122	70.03726	73.97384	42.16642	16.56141	15.42127	101.4485	88.99853	87.39189	52.34519	21.8432	25.99293
AC140125.	0	0	5.994802	0	0	0	0	0	0	0	0	0	0	0	0	0	0	0	0
AC140134.	458.3529	334.1304	456.6041	576.8075	300.1284	300.3163	322.0288	590.8052	324.506	383.319	357.8122	602.6512	619.9349	574.2544	711.0102	597.7276	449.5528	1249.613	864.0409
AC140479.	0	0	0	0	0	0	0	0	0	0	0	0.920078	1.028084	0	0	0.824452	1.026376	0	0
AC140479.	44.20175	34.01327	13.98787	14.5501	85.27634	63.94567	14.44456	71.30408	68.86998	15.37119	15.66181	56.12477	33.92678	22.33729	27.38416	122.0189	91.34749	23.66347	2.688924
AC140479.	0.960908	0	0	6.235757	3.322455	0	3.398721	0	0	0	2.40951	5.520469	2.056169	0	0.978006	4.122259	0	0	0
AC140479.	18.25724	13.00508	38.96621	27.02161	26.57964	38.82416	11.89552	17.59451	93.38302	24.97818	44.57593	29.4425	22.61786	53.05106	53.79032	48.64266	50.29244	50.96747	13.44462
AC140479.	2.882723	1.00039	0	1.039293	1.107485	0	0.84968	3.704108	0	0.960699	0	3.680313	0	0	0	5.771163	2.052753	0.910133	0.896308
AC140479.	4.804538	1.00039	0.999134	0	2.21497	2.283774	0	0	1.167288	0	0	0.920078	3.084253	0	0.978006	0	3.079129	9.101334	0.896308
AC140481.	0.960908	1.00039	0	5.196464	3.322455	3.425661	3.398721	0.926027	4.669151	1.921399	0	3.680313	4.112337	5.584322	2.934017	1.648904	1.026376	5.4608	1.792616
AC140481.	0	0	2.997401	0	0	0	0	0	0	0	0	0	0	0	0	0	0	0	0
AC140481.	2.882723	0	0	0	0	2.283774	2.549041	0	0	0	0	1.840156	0	0.93072	0	0.824452	0	0.910133	0
AC140725.	0.960908	4.001562	13.98787	3.117878	11.07485	17.12831	5.098081	5.556162	23.34575	0	2.40951	4.600391	11.30893	9.307203	0	19.78684	8.21101	4.550667	6.274156
AC140847.	0	4.001562	0	0	0	0	0	0	0	0	0	0	0	0	0	0	0	0	0
AC140847.	0	2.000781	5.994802	3.117878	1.107485	2.283774	8.496802	1.852054	7.003726	3.842797	0	3.680313	0	6.515042	1.956012	4.122259	0	3.640534	4.48154
AC140912.	7.68726	33.01288	9.991337	21.82515	8.859879	12.56076	29.73881	15.74246	36.18592	16.33189	1.204755	8.280704	16.44935	14.89152	23.47214	50.29156	56.45069	32.7648	43.02278
AC141002.	64.3808	53.02069	48.95755	68.59332	47.62185	51.38492	78.17058	66.67395	10.50559	62.44545	46.98544	70.84602	58.60081	44.67457	44.01026	60.18498	39.0023	31.85467	59.15632
AC141273.	0	0	0	0	0	0	0	0	0	0	0	0	0	0	0	0	0	0	1.792616
AC141424.	6.726353	1.00039	0	2.078586	0	0	2.549041	0	11.67288	0	0	5.520469	0	1.861441	4.890029	8.244518	4.105505	3.640534	0
AC141557.	380.5194	123.048	109.9047	403.2456	259.1515	345.9918	48.43177	214.8383	239.294	514.9348	575.8728	299.9455	415.3461	363.9116	397.0704	211.0597	220.6709	500.5734	412.3017
AC141557.	84.55986	65.02538	9.991337	44.68959	24.36467	75.36454	23.79105	68.526	32.68406	157.5547	199.9893	57.96493	77.10633	53.05106	60.63636	26.38246	21.5539	92.83361	82.46033
AC141586.	342.0831	244.0953	260.7739	312.8271	321.1706	255.7827	358.5651	272.2519	124.8998	284.367	368.655	348.7097	318.7062	294.1076	311.0059	301.7494	315.0975	274.8603	334.3229
AC141586.	70.14625	39.01523	13.98787	33.25737	64.23413	23.97963	18.69297	24.0767	32.68406	55.72056	31.32363	13.80117	6.168506	26.99089	45.96627	39.57369	40.02867	39.13574	27.78555
AC141586.	1.921815	6.002343	6.993936	23.90373	4.42994	10.27698	5.098081	45.37532	0	1.921399	13.2523	13.80117	14.39318	9.307203	5.868035	4.122259	5.131881	8.191201	8.963079
AC141586.	32.67086	70.02733	93.91856	31.17878	71.98652	34.25661	84.96802	50.00546	43.18965	48.03496	57.82823	116.8499	78.13441	53.05106	72.37243	42.04704	54.39794	29.12427	47.50432
AC141928.	378.5976	689.269	562.5123	327.3772	361.0401	735.3752	494.5139	563.0244	728.3875	680.1751	1083.075	529.045	481.1435	567.7394	500.739	355.3387	467.0012	499.6632	675.8162
AC141930.	0	0	0	5.196464	0	5.709435	2.549041	9.26027	0	0.960699	7.228529	0	3.084253	19.54513	0.978006	1.648904	5.131881	0.910133	1.792616
AC141930.	0.960908	1.00039	0.999134	7.275049	3.322455	0	0.84968	3.704108	0	0	0	0	0	0	0	0	1.026376	0	0
AC141930.	0.960908	3.001171	0	0	1.107485	4.567548	5.947762	3.704108	9.338302	10.56769	0	0	4.112337	3.722881	0	4.946711	1.026376	0.910133	1.792616
AC142086.	0	1.00039	0.999134	0	0	0	0	0	0	0	0	0	0	0	0	0	0	2.7304	0
AC142086.	0	0	0	0	1.107485	0	4.248401	0	0	0	0	0	0	3.722881	0	0	0	0.910133	0
AC142086.	63.4199	49.01913	31.97228	15.58939	37.65449	25.12151	12.7452	12.96438	38.5205	14.41049	31.32363	36.80313	46.2638	29.78305	35.20821	28.03136	18.47477	18.20267	56.4674
AC142381.	16.33543	45.01757	12.98874	7.275049	16.61227	13.70264	17.84329	5.556162	10.50559	14.41049	14.45706	31.28266	32.8987	48.39746	14.67009	20.6113	23.60665	28.21414	28.68185
AC142381.	37.47539	39.01523	16.98527	11.43222	28.79461	42.24982	26.34009	39.81916	3.501863	9.606993	56.62348	31.28266	34.95487	18.61441	34.2302	29.68027	26.68578	30.94454	43.91909
AC142381.	8.648168	23.00898	6.993936	2.078586	18.82724	9.135096	12.7452	0	8.171014	5.764196	4.81902	12.8811	15.42127	13.9608	15.64809	0.824452	4.105505	5.4608	4.48154
AC142384.	0.960908	0	0	0	0	0	0	0	0	0	0	2.760235	1.028084	0.93072	0	0	0	0	0
AC142472.	200.8297	146.057	230.7999	204.7407	172.7676	199.8302	164.838	141.6821	430.7292	216.1573	377.0883	167.4542	180.9428	218.7193	206.3592	209.4108	222.7236	202.9598	198.9804
AC143336.	0	0	0	0	0	0	0	0	0	0	0	0	0	0	0	4.946711	0	0	0
AC144450.	8.648168	2.000781	0	4.157171	6.64491	1.141887	0.84968	0	0	0	0	0	2.056169	0.93072	3.912023	3.297807	8.21101	0.910133	0
AC144521.	0	0	0.999134	0	0	0	0.84968	0.926027	0	0	0	0.920078	0	0	0.978006	1.648904	0	0	0.896308
AC144522.	1.921815	0	0	0	2.21497	0	0	0	0	0	0	0.920078	0	0	0	0.824452	0	0	0
AC144530.	60.53717	41.01601	39.96535	36.37525	56.48173	50.24303	50.98081	39.81916	23.34575	10.56769	31.32363	31.28266	23.64594	48.39746	36.18622	47.81821	36.94955	52.78774	70.80833
AC144548.	1.921815	1.00039	0	0	7.752394	2.283774	0	1.852054	0	8.646293	7.228529	0	0	0	1.956012	0	4.105505	5.4608	5.377848
AC144548.	45.16265	51.01991	73.93589	43.6503	70.87903	53.66869	41.63433	71.30408	38.5205	54.75986	62.64725	57.04485	65.7974	49.32818	55.74633	51.94047	43.1078	77.36134	63.63786
AC144568.	4.804538	19.00742	22.98007	17.66798	15.50479	21.69585	19.54265	10.1863	51.36066	31.70308	22.89034	14.72125	10.28084	8.376483	22.49413	21.43575	23.60665	17.29253	9.859387
AC144568.	1.921815	2.000781	1.998267	8.314342	15.50479	0	7.647122	0.926027	9.338302	3.842797	2.40951	9.200782	3.084253	2.792161	4.890029	5.771163	15.39564	12.74187	10.7557
AC144652.	397.8157	463.1808	631.4525	327.3772	296.806	444.194	282.9435	310.2191	653.6811	588.9086	722.8529	419.5557	480.1154	391.8332	322.7419	368.53	580.929	542.4395	639.9639
AC144831.	686.088	235.0918	263.7713	239.0373	280.1937	182.7019	180.1322	199.0958	484.4244	342.0089	374.6788	436.1171	493.4805	276.4239	275.7976	196.2195	260.6996	488.7416	495.6583
AC144833.	0	0	12.98874	8.314342	5.537425	7.993209	2.549041	0	0	0.960699	0	2.760235	3.084253	13.9608	5.868035	0.824452	0	0	7.170464

AC144836.	0	0	0.999134	6.235757	0	1.141887	0.84968	0.926027	0	0	1.204755	4.600391	0	4.653602	0	0	0	0.910133	0
AC145098.	133.5661	92.03592	50.95582	123.6758	70.87903	150.7291	103.661	116.6794	128.4017	82.62014	144.5706	138.9318	125.4263	135.8852	123.2287	278.6647	227.8555	374.975	350.4564
AC145098.	296.9204	201.0785	166.8553	90.41847	232.5718	389.3835	180.1322	289.8465	255.636	161.3975	168.6657	291.6648	226.1786	188.9362	222.0073	177.2571	236.0665	105.5755	127.2757
AC145124.	262.3278	193.0754	168.8536	269.1768	394.2646	227.2355	192.0277	538.0217	242.7958	150.8298	165.0514	283.3841	312.5376	208.4813	151.5909	426.2416	427.9989	296.7035	413.198
AC145138.	820.615	700.2733	632.4516	684.8939	826.1838	709.1118	544.645	1049.189	755.2352	800.2625	828.8714	767.3452	832.7483	1008.901	1026.906	1113.834	893.9737	1640.971	1253.935
AC145141.	0	0	0	0	0	0	0	0	0	0	0	0	4.112337	0	0	0	0	0	0.896308
AC145146.	0.960908	0	0	0	1.107485	0	0	0	5.836439	0.960699	1.204755	0	1.028084	0	0	1.648904	1.026376	0	0
AC145207.	22.10087	12.00469	4.995668	12.47151	0	23.97963	22.09169	10.1863	3.501863	8.646293	7.228529	12.8811	13.3651	6.515042	16.6261	12.36678	17.4484	11.83173	26.88924
AC145207.	194.1033	218.0851	143.8752	133.0295	197.1323	221.5261	204.7729	176.8712	291.8219	220.0001	245.77	164.694	202.5326	251.2945	233.7434	145.1035	175.5103	201.1395	201.6693
AC145207.	0	0	0	3.117878	0	0	0	0	0	0.960699	0	0	1.028084	0	0	0	0	0.910133	0
AC145207.	19.21815	34.01327	16.98527	37.41454	45.40688	28.54718	41.63433	25.92876	14.00745	10.56769	25.29985	37.72321	45.23571	46.53602	18.58211	87.39189	88.26836	40.04587	49.29694
AC145207.	5.765445	25.00976	44.96101	9.353635	29.90209	36.54038	28.88913	24.0767	46.69151	34.58517	62.64725	39.56336	28.78636	47.46674	53.79032	20.6113	18.47477	37.31547	20.61508
AC145285.	25.9445	19.00742	49.95668	29.1002	33.22455	36.54038	16.14392	27.78081	67.70269	23.05678	48.1902	29.4425	11.30893	51.18962	19.56012	37.10033	31.81766	27.304	34.95601
AC145285.	218.126	228.089	254.7791	141.3438	222.6045	248.9314	197.1258	217.6164	133.0708	390.0439	334.9219	176.655	155.2407	235.4722	232.7654	144.2791	196.0379	347.671	331.6339
AC145285.	0	15.00586	37.96708	6.235757	24.36467	17.12831	15.29424	1.852054	49.02608	39.38867	101.1994	44.16376	22.61786	29.78305	12.71408	9.893422	16.42202	0	0.896308
AC145285.	53.81082	22.00859	71.93762	48.84676	25.47215	22.83774	36.53625	53.70957	22.17847	39.38867	39.75691	58.88501	45.23571	15.82225	26.40616	40.39814	41.05505	33.67494	62.74156
AC145285.	0	0	0	0	2.21497	0	0	0.926027	4.669151	0	0	0.920078	1.028084	0	0	0	0	0.910133	0
AC145285.	0	5.001952	4.995668	5.196464	0	4.567548	17.84329	0	0	3.842797	0	21.1618	18.50552	6.515042	3.912023	1.648904	1.026376	18.20267	8.963079
AC145285.	37.47539	50.01952	58.94889	31.17878	37.65449	61.6619	44.18337	31.48492	54.86252	70.13105	84.33284	40.48344	34.95487	47.46674	41.07624	55.23827	49.26606	81.00187	103.9717
AC145343.	7.68726	21.0082	21.98094	3.117878	27.68712	9.135096	6.797442	8.334243	72.37184	17.29259	25.29985	19.32164	8.224675	19.54513	17.6041	9.06897	15.39564	15.47227	10.7557
AC145350.	5.765445	8.003124	3.996535	1.039293	2.21497	0	0.84968	1.852054	0	3.842797	8.433284	9.200782	3.084253	8.376483	0	0	2.052753	4.550667	3.585232
AC145350.	0.960908	0	0	0	0	0	0	0.84968	0	0	2.882098	6.023774	0	0	0	0	0	0	0
AC145422.	62.45899	58.02265	36.96795	57.1611	57.58922	45.67548	39.08529	55.56162	37.35321	27.86028	67.46627	49.68422	49.34805	63.28898	62.59237	22.2602	59.52982	59.15867	55.57109
AC145423.	11.53089	4.001562	1.998267	0	1.107485	6.851322	0	9.26027	1.167288	10.56769	4.81902	0.920078	4.112337	0.93072	9.780058	4.946711	5.131881	10.01147	8.963079
AC145423.	31.70995	28.01093	5.994802	29.1002	35.43952	19.41208	21.24201	12.03835	33.85134	30.74238	39.75691	27.60235	19.5336	32.57521	23.47214	26.38246	21.5539	11.83173	19.71877
AC145423.	78.79442	64.02499	130.8865	62.35757	121.8233	81.07398	93.46483	120.3835	136.5727	174.8473	222.8797	127.8909	120.2859	96.79491	94.86656	56.88718	42.08143	111.9464	160.4391
AC145676.	56.69354	60.02343	43.96188	41.57171	44.2994	49.10114	30.58849	9.26027	45.52422	43.23147	55.41872	61.64524	68.88165	28.85233	38.14223	91.51415	49.26606	130.1491	179.2616
AC146507.	0.960908	0	0.999134	0	0	0	0	0	1.167288	0	0	3.680313	1.028084	3.722881	0.978006	0.824452	0	0.910133	0
AC146507.	0.960908	3.001171	9.991337	5.196464	15.50479	1.141887	16.14392	12.03835	9.338302	4.803496	9.638039	17.48149	12.33701	1.861441	23.47214	11.54233	11.29014	3.640534	2.688924
AC146944.	4.804538	3.001171	0.999134	0	0	4.567548	6.797442	0	4.669151	1.921399	0	0	0	0.93072	0	4.946711	1.026376	0.910133	0
AC146944.	15.37452	45.01757	23.97921	37.41454	36.547	49.10114	10.19616	72.23011	25.68033	40.34937	28.91412	42.3236	39.06721	69.80402	129.0968	234.1443	89.29473	62.79921	56.4674
AC146944.	21.13997	48.01874	0	0	2.21497	4.567548	3.398721	0	43.18965	0.960699	0	0	3.084253	0.93072	0	13.19123	18.47477	0.910133	0
AC146944.	241.1878	196.0765	12.98874	11.43222	12.18233	109.6212	9.346483	69.45203	150.5801	6.724895	2.40951	5.520469	11.30893	137.7466	150.6129	219.3042	222.7236	2.7304	8.963079
AC146949.	27.86632	22.00859	42.96275	42.611	27.68712	14.84453	50.98081	37.96711	2.334575	31.70308	37.3474	57.96493	72.99399	17.68369	13.69208	16.48904	13.34289	39.13574	71.70464
AC147055.	21.13997	0	0	0	0	0	0	0	0	0	0	0	0	0	2.934017	4.122259	1.026376	0	0
AC147067.	166.237	108.0422	87.92376	56.12181	194.9173	100.4861	65.42538	116.6794	395.7105	199.8254	177.099	65.32555	82.24675	87.48771	74.32844	89.0408	149.8509	66.43974	91.42341
AC147067.	3.84363	2.000781	2.997401	4.157171	11.07485	1.141887	0.84968	0.926027	17.50932	0.960699	8.433284	6.440548	5.140422	8.376483	5.868035	4.122259	7.184634	5.4608	3.585232
AC147651.	1.921815	2.000781	2.997401	2.078586	8.859879	2.283774	1.69936	3.704108	0	0	4.81902	10.12086	1.028084	2.792161	14.67009	3.297807	6.158258	2.7304	0.896308
AC147651.	1.921815	13.00508	9.991337	3.117878	6.64491	6.851322	0.84968	0.926027	0	0	4.81902	4.600391	2.056169	9.307203	3.912023	8.244518	10.26376	0	0
AC147651.	73.02897	54.02108	101.9116	80.02554	89.70628	107.3374	31.43817	48.15341	284.8182	79.73804	106.0184	117.77	77.10633	105.1714	88.02052	165.7148	96.47937	218.432	114.7274
AC148476.	6.726353	24.00937	1.998267	6.235757	18.82724	18.27019	10.19616	12.96438	36.18592	19.21399	9.638039	14.72125	12.33701	13.9608	3.912023	6.595615	7.184634	19.1128	8.963079
AC148477.	33.63176	26.01015	34.96968	25.98232	43.19191	38.82416	35.68657	26.85478	49.02608	33.62447	62.64725	30.36258	51.40422	30.71377	29.34017	32.97807	36.94955	29.12427	40.33386
AC148477.	0	1.00039	0	0	0	1.141887	0.84968	0	0	4.803496	7.228529	0	0	4.653602	1.956012	4.946711	0	9.101334	6.274156
AC148477.	29.78813	18.00703	5.994802	12.47151	5.537425	3.425661	24.64073	5.556162	0	8.646293	21.68559	6.440548	22.61786	4.653602	1.956012	0.824452	13.34289	7.281067	11.652
AC148477.	4.804538	2.000781	0.999134	3.117878	5.537425	0	5.947762	10.1863	1.167288	9.606993	13.2523	7.360626	9.252759	1.861441	1.956012	0.824452	10.26376	1.820267	7.170464
AC148477.	11.53089	18.00703	35.96881	19.74656	28.79461	34.25661	28.88913	38.89314	17.50932	27.86028	13.2523	27.60235	37.01104	40.95169	30.31818	13.19123	23.60665	30.0344	31.37078
AC152007.	0	0	0	0	0	1.141887	0.84968	0	0	0	0	0	0	0	0	0	0	0	0
AC152010.	0	0	0	0	0	0	0	0.926027	0	0	0	0	0	0	0	0	0	0	7.170464
AC156455.	42.27993	36.01406	51.95495	69.63262	101.8886	49.10114	66.27506	44.4493	30.34948	42.27077	54.21397	114.0897	80.19058	105.1714	115.4047	105.5298	76.97822	95.56401	73.49725
AC159540.	0	0	0	0	0	0	0	1.69936	1.852054	0	0	0	0	0	0	0	0	0	0
AC159540.	14.41361	21.0082	16.98527	14.5501	33.22455	19.41208	26.34009	19.44657	32.68406	18.25329	20.48083	29.4425	12.33701	11.16864	13.69208	10.71787	9.237386	13.652	13.44462
AC183088.	0.960908	0	0	0	0	0	0	0	4.669151	0.960699	7.228529	3.680313	1.028084	0	0	0	0	0	0
AC187653.	0.960908	0	0	0	0	4.567548	0.84968	0	7.003726	0	0	5.520469	1.028084	4.653602	0.978006	0	0	2.7304	2.688924
AC209007.	1.921815	1.00039	0.999134	0	0	0	0.84968	0	0	0	0	0	0	0	0	0	0	0	0
AC211433.	25.9445	25.00976	55.95149	55.08252	63.12664	38.82416	113.8572	97.23284	32.68406	43.23147	21.68559	79.12673	56.54464	39.09025	56.72434	48.64266	35.92317	54.608	57.36371
AC211433.	0.960908	2.000781	2.997401	0	1.107485	11.41887	4.248401	0.926027	14.00745	5.764196	4.81902	6.440548	1.028084	0	3.912023	0	3.079129	0	7.170464
AC211433.	0.960908	0	0	0	0	4.567548	0	0	4.669151	0	0	0	0	0	0.978006	0	0	0	2.688924
AC211469.	0	0	0	0	0	0	0	0	9.338302	0	0	0.920078	0	0	0	0	0	0	0
AC211469.	0	1.00039	0	0	0	0	1.69936	1.852054	0	0	0	0	1.028084	0	0	0	0	0	0
AC211476.	8.648168	12.00469	12.98874	0	1.107485	6.851322	12.7452	5.556162	5.836439	4.803496	6.023774	5.520469	8.224675	4.653602	3.912023	7.420066	1.026376	1.820267	3.585232
AC211476.	35.55358	38.01484	34.96968	34.29666	69.77155	49.10114	29.73881	80.56435	24.51304	63.40615	50.5997	48.76415	49.34805	53.05106	83.1305	58.53608	56.45069	28.21414	69.01571
AC211476.	0.960908	2.000781	0	1.039293	11.07485	0	2.549041	3.704108	3.501863	2.882098	6.023774	4.600391	6.168506	9.307203	2.934017	1.648904	0	0	0

AC211476.	0.960908	2.000781	0	1.039293	11.07485	0	2.549041	3.704108	3.501863	2.882098	6.023774	4.600391	6.168506	9.307203	2.934017	1.648904	0	0	0
AC211485.	2.882723	4.001562	0	5.196464	0	1.141887	0.84968	6.482189	0	3.842797	0	3.680313	1.028084	0.93072	0.978006	0	1.026376	0	0.896308
AC211486.	10.56998	1.00039	7.993069	0	4.42994	6.851322	15.29424	2.778081	5.836439	7.685594	4.81902	5.520469	2.056169	5.584322	3.912023	8.244518	3.079129	5.4608	2.688924
AC211486.	0	0	0	0	0	0	0.84968	0	0	0	0	0	0	0	0.978006	0	0	0	0
AC211486.	14.41361	17.00664	23.97921	18.70727	19.93473	22.83774	11.04584	21.29862	18.6766	23.05678	16.86657	14.72125	19.5336	26.06017	16.6261	16.48904	18.47477	19.1128	14.34093
AC211486.	0	0	0	0	0	0	0.84968	0	0	0	0	2.760235	1.028084	0	0.978006	0	6.158258	2.7304	0
AC215217.	0.960908	3.001171	1.998267	3.117878	0	0	0	0	0	3.842797	3.614265	0	0	0	0	0	0	0	0
AC215219.	1676.784	971.3791	850.2627	402.2063	3509.62	1846.431	960.9884	2092.821	7867.519	3137.644	7659.832	1709.505	1589.418	2309.117	2009.802	718.922	853.945	2572.947	2557.167
AC215522.	0	0	0	0	0	2.283774	0	0	0	7.685594	0	0	0	0.93072	1.956012	1.648904	0	0	1.792616
AC215522.	18.25724	13.00508	22.98007	63.39686	38.76197	21.69585	16.9936	20.37259	17.50932	0	21.68559	33.12282	18.50552	38.15953	38.14223	124.4922	58.50345	293.9731	185.5357
AC217774.	0	0	0	0	0	0	0	0	0	0	3.614265	0	0	0	0	0.824452	0	0.910133	0
AC217777.	1742.125	1240.484	963.1649	1866.57	827.2912	1098.495	1682.367	1233.468	32.68406	464.0177	307.2125	1539.291	1518.481	1504.975	1211.749	1425.477	1435.9	1253.254	1557.783
AC226101.	3.84363	0	0	0	0	1.141887	1.69936	0	0	0	0	0	0	0	0	0.824452	2.052753	2.7304	0
AC226101.	33.63176	26.01015	34.96968	29.1002	15.50479	37.68227	30.58849	32.41095	52.52795	30.74238	30.11887	39.56336	19.5336	48.39746	60.63636	15.66458	51.31881	30.0344	40.33386
AC226118.	29.78813	8.003124	27.97574	30.13949	16.61227	26.2634	45.03305	23.15068	0	14.41049	44.57593	24.84211	8.224675	17.68369	14.67009	21.43575	16.42202	52.78774	34.0597
AC231533.	12.4918	9.003514	1.998267	14.5501	13.28982	11.41887	11.89552	15.74246	26.84762	21.13538	26.50461	21.1618	5.140422	16.75297	16.6261	11.54233	8.21101	17.29253	16.13354
AC231533.	37.47539	38.01484	30.97314	22.86444	42.08443	31.97284	14.44456	40.74519	148.2455	55.72056	31.32363	31.28266	35.98295	40.02097	46.94428	28.85581	36.94955	36.40534	19.71877
AC231657.	15.37452	30.01171	39.96535	32.21808	16.61227	31.97284	28.03945	16.66849	4.669151	13.44979	36.14265	19.32164	24.67402	52.12034	17.6041	32.97807	21.5539	31.85467	24.20031
AC231657.	11.53089	29.01132	37.96708	34.29666	44.2994	10.27698	34.83689	59.26573	0	19.21399	16.86657	48.76415	50.37613	25.12945	33.2522	18.13794	21.5539	30.94454	30.47447
AC231759.	0.960908	0	0	0	0	3.425661	0	0	0	0	12.04755	0	0	0.93072	0	0	0	0	0.896308
AC231981.	46.12356	40.01562	58.94889	32.21808	66.4491	92.49285	29.73881	64.82189	26.84762	98.95202	69.87578	25.76219	50.37613	36.29809	44.98827	43.69595	44.13418	15.47227	30.47447
AC232271.	835.9896	823.3213	1303.869	654.7544	1035.498	776.4832	969.4852	1014.926	1481.288	1304.63	1346.916	1141.817	1247.066	966.0877	875.3152	598.552	480.3441	1068.497	1146.378
AC233263.	0	0	0	0	0	0	0.84968	0	0	0.960699	0	0	0	0	0	0	0	0	1.792616
AC233263.	9.609075	22.00859	19.98267	12.47151	6.64491	9.135096	16.9936	16.66849	18.6766	43.23147	14.45706	14.72125	12.33701	3.722881	19.56012	11.54233	2.052753	7.281067	16.13354
AC233266.	1.921815	6.002343	3.996535	10.39293	3.322455	3.425661	20.39233	2.778081	7.003726	12.48909	3.614265	13.80117	10.28084	8.376483	16.6261	14.01568	4.105505	11.83173	9.859387
AC233266.	19.21815	24.00937	35.96881	33.25737	37.65449	35.3985	100.2623	39.81916	53.69524	7.685594	1.204755	59.80509	79.1625	53.05106	42.05425	65.13169	95.45299	37.31547	32.26709
AC233279.	0	4.001562	0	0	0	0	0.84968	0	2.334575	2.882098	0	0.920078	0	3.722881	0.978006	1.648904	0	0	0
AC233280.	3.84363	23.00898	25.97748	5.196464	38.76197	22.83774	25.49041	34.263	57.1971	93.18783	107.2232	34.04289	26.73019	28.85233	24.45015	41.22259	61.58258	13.652	25.09662
AC233280.	15.37452	13.00508	58.94889	9.353635	15.50479	29.68906	44.18337	30.55889	3.501863	24.01748	22.89034	22.08188	30.84253	27.92161	35.20821	17.31349	28.73854	13.652	17.02985
AC233280.	24.9836	37.01445	46.95928	38.45383	76.41646	63.94567	73.92218	100.9369	116.7288	99.91272	79.51382	100.2885	90.47142	40.02097	76.28445	138.5079	145.7454	59.15867	60.94894
AC233290.	0	4.001562	0.999134	0	0	1.141887	0.84968	0	0	0	0	0	0	0	0	0	0	0.910133	0
AC233300.	165.2761	97.03787	228.8016	251.5089	194.9173	86.78341	239.6098	182.4273	14.00745	132.5765	50.5997	261.3022	319.7342	216.8578	240.5894	154.9969	110.8486	178.3861	143.4093
AC233699.	139.3316	170.0664	198.8276	167.3261	128.4683	176.9925	209.871	137.978	52.52795	132.5765	73.49005	138.0117	173.7463	137.7466	196.5792	135.2101	199.117	118.3173	133.5499
AC233701.	0.960908	0	3.996535	0	2.21497	0	0	3.704108	0	1.921399	0	0.920078	0	3.722881	0	0	0	0	4.48154
AC233702.	5.765445	2.000781	13.98787	12.47151	13.28982	19.41208	7.647122	5.556162	32.68406	12.48909	20.48083	16.56141	21.58977	3.722881	8.802052	4.946711	7.184634	3.640534	8.963079
AC233702.	10.56998	2.000781	5.994802	0	0	0	0	0	0	0	0	6.440548	2.056169	0	0	0	0	1.820267	0
AC233702.	10.56998	3.001171	10.99047	11.43222	0	0	5.947762	0	4.669151	3.842797	20.48083	14.72125	2.056169	0	2.934017	0	5.131881	17.29253	8.963079
AC233702.	7.68726	28.01093	0.999134	1.039293	2.21497	1.141887	4.248401	0	1.167288	4.803496	0	12.8811	3.084253	0	2.934017	0	0	0.910133	2.688924
AC233702.	33.63176	10.0039	7.993069	28.0609	7.752394	5.709435	21.24201	4.630135	11.67288	0.960699	0	25.76219	16.44935	4.653602	14.67009	8.244518	12.31652	11.83173	14.34093
AC233702.	6.726353	10.0039	0.999134	3.117878	8.859879	10.27698	4.248401	3.704108	19.84389	1.921399	1.204755	2.760235	3.084253	7.445762	5.868035	0	10.26376	5.4608	4.48154
AC233702.	0.960908	1.00039	3.996535	0	0	0	1.69936	2.778081	1.167288	0	1.204755	3.680313	3.084253	7.445762	1.956012	4.122259	4.105505	0.910133	0.896308
AC233702.	1.921815	1.00039	0.999134	0	1.107485	0	1.69936	0	0	3.842797	0	1.840156	0	2.792161	0.978006	0.824452	3.079129	0	0
AC233723.	5.765445	24.00937	1.998267	4.157171	24.36467	13.70264	6.797442	17.59451	46.69151	52.83846	57.82823	26.68227	16.44935	5.584322	4.890029	7.420066	3.079129	4.550667	9.859387
AC233723.	1166.542	1328.519	1161.992	1172.322	998.9514	886.1043	1405.371	942.6955	1378.567	1386.289	1316.797	1519.969	1597.643	1430.517	1394.636	1128.675	1320.946	1639.15	1676.096
AC233724.	0	1.00039	0	0	0	0	0	0	9.338302	0	0	0	0	0	0	0	0	0	0
AC233724.	0	0	0	0	0	0	0	0	3.501863	0	0	0	0	0	0	0	0	0	0
AC233724.	0.960908	0	0	0	0	0	0	0	3.501863	0	0	0	0	0	0	0	0	0	0
AC233728.	42.27993	53.02069	52.95408	55.08252	45.40688	46.81737	74.77186	50.00546	119.0633	71.09174	66.26152	37.72321	45.23571	69.80402	46.94428	46.99375	42.08143	34.58507	49.29694
AC233728.	0	1.00039	0	1.039293	0	0	0	0	0	0	0	0	0	0	0	0	0	0	0
AC233964.	0.960908	0	0	0	1.107485	0	0	0	0	0	0	0	0	0	0	0.824452	0	0	0
AC233968.	17.29634	30.01171	59.94802	48.84676	63.12664	39.96605	29.73881	32.41095	37.35321	29.78168	49.39495	46.92399	57.57272	38.15953	39.12023	25.55801	58.50345	33.67494	18.82247
AC233976.	10.56998	16.00625	7.993069	4.157171	25.47215	5.709435	9.346483	12.03835	9.338302	10.56769	39.75691	16.56141	13.3651	4.653602	17.6041	13.19123	8.21101	7.281067	14.34093
AC233981.	0.960908	1.00039	0	1.039293	0	2.283774	0.84968	0	0	0	2.40951	0	1.028084	0	3.912023	0	0	0	0
AC233982.	0	0	0	0	0	1.141887	0	0.926027	0	0	0	0.920078	0	0.93072	3.912023	0	0	0.910133	0
AC233992.	44.20175	41.01601	21.98094	31.17878	66.4491	39.96605	22.94137	68.526	191.4352	93.18783	231.3129	50.6043	46.2638	40.02097	34.2302	42.04704	59.52982	37.31547	27.78555
AC233992.	0	2.000781	3.996535	0	0	0	0.84968	0	0	0	0	0.920078	1.028084	0	0	0.824452	0	2.7304	6.274156
AC234582.	955.1421	1413.552	1372.81	897.949	1382.141	1242.373	1000.923	1198.279	1986.724	1672.577	1964.955	981.7235	1089.769	1116.864	1409.306	1081.681	1162.884	918.3246	1024.48
AC234644.	1.921815	1.00039	5.994802	0	4.42994	0	1.69936	0.926027	0	2.882098	0	3.680313	8.224675	0	0	4.946711	4.105505	4.550667	5.377848
AC234771.	12.4918	19.00742	34.96968	12.47151	5.537425	18.27019	58.62794	34.263	39.68778	3.842797	0	30.36258	23.64594	19.54513	5.868035	45.34485	40.02867	55.51814	40.33386
AC234771.	0	0	0	0	0	0	0	0	0	0	0	0	0	0	0	0	8.21101	0	0
AC234772.	0.960908	2.000781	0	0	0	0	0.84968	0	0	0	0	0	1.028084	1.861441	0	2.473355	0	0.910133	0.896308
AC234772.	2.882723	2.000781	0	11.43222	6.64491	2.283774	7.647122	0.926027	12.84017	0.960699	6.023774	0.920078	0	0	3.912023	18.96239	25.65941	4.550667	0.896308

AC234775.	0	0	0	1.039293	0	0	0	0	0	0.960699	0	2.760235	1.028084	3.722881	0	0	2.052753	0	0
AC234775.	6.726353	0	6.993936	27.02161	2.21497	1.141887	11.04584	1.852054	1.167288	5.764196	0	2.760235	8.224675	5.584322	3.912023	7.420066	9.237386	1.820267	13.44462
AC234775.	326.7086	487.1901	533.5374	1001.878	439.6715	221.5261	1184.454	327.8136	144.7437	146.0263	116.8612	630.2536	532.5477	545.4021	508.563	546.6116	545.0058	484.191	622.0377
AC234778.	0	0	0	0	0	0	0.84968	0.926027	2.334575	0	0	0	3.084253	0	0	0	1.026376	0	0
AC234778.	1.921815	4.001562	2.997401	3.117878	5.537425	0	9.346483	4.630135	5.836439	8.646293	0	18.40156	18.50552	14.89152	6.846041	9.893422	7.184634	11.83173	16.13354
AC234781.	25.9445	21.0082	8.992203	8.314342	50.94431	5.709435	11.89552	13.89041	10.50559	11.52839	4.81902	16.56141	19.5336	20.47585	21.51613	9.893422	12.31652	30.0344	12.54831
AC234781.	0	7.002733	0.999134	0	2.21497	7.993209	0.84968	5.556162	0	0.960699	1.204755	6.440548	9.252759	0.93072	7.824047	0	1.026376	10.9216	14.34093
AC234781.	6.726353	2.000781	7.993069	1.039293	1.107485	2.283774	7.647122	23.15068	0	1.921399	0	4.600391	2.056169	2.792161	2.934017	12.36678	9.237386	2.7304	1.792616
AC234781.	1.921815	3.001171	0	2.078586	2.21497	3.425661	1.69936	6.482189	0	3.842797	0	2.760235	2.056169	5.584322	10.75806	8.244518	3.079129	14.56213	0
AC234781.	3.84363	0	3.996535	0	0	0	3.398721	0.926027	0	0	4.81902	5.520469	2.056169	0.93072	0	0	0	2.7304	1.792616
AC234782.	50.9281	41.01601	49.95668	108.0864	18.82724	26.2634	31.43817	27.78081	7.003726	26.89958	12.04755	31.28266	34.95487	62.35826	67.4824	93.16306	70.81996	40.04587	95.00864
AC234782.	86.48168	42.0164	119.896	81.06484	37.65449	30.83095	96.01387	54.6356	64.20083	50.91706	30.11887	81.88696	114.1174	113.5479	77.26246	79.97183	104.6904	124.6883	109.3496
AC234782.	3.84363	1.00039	5.994802	1.039293	6.64491	7.993209	1.69936	3.704108	16.34203	3.842797	1.204755	0	3.084253	0.93072	1.956012	6.595615	12.31652	9.101334	2.688924
AC234782.	28.82723	49.01913	50.95582	28.0609	35.43952	28.54718	56.0789	90.75065	33.85134	27.86028	20.48083	80.04681	74.02207	50.2589	88.02052	61.83389	39.0023	52.78774	48.40063
AC234782.	19.21815	8.003124	14.987	23.90373	5.537425	17.12831	32.28785	25.00273	9.338302	15.37119	26.50461	29.4425	49.34805	16.75297	21.51613	18.96239	6.158258	32.7648	14.34093
AC234783.	11.53089	21.0082	25.97748	18.70727	19.93473	41.10793	7.647122	11.11232	39.68778	50.91706	45.78069	20.24172	16.44935	9.307203	6.846041	18.13794	45.16056	16.3824	20.61508
AC234917.	1.921815	13.00508	11.9896	15.58939	13.28982	25.12151	27.18977	8.334243	19.84389	29.78168	25.29985	12.8811	8.224675	16.75297	11.73607	17.31349	12.31652	12.74187	4.48154
AC235097.	0	0	0	0	0	0	0	0	0	0	0	0	0	0	0.978006	0	0	1.820267	2.688924
AC236430.	0	0	3.996535	0	0	0	0	0	0	0	0	0	0	0	0	0	0	0	0
AC236972.	0	7.002733	0	6.235757	1.107485	1.141887	3.398721	3.704108	0	1.921399	0	2.760235	8.224675	0.93072	0	4.946711	0	0	0.896308
AC236972.	12.4918	47.01835	18.98354	39.49313	21.04221	43.39171	33.98721	19.44657	17.50932	46.11356	42.16642	14.72125	18.50552	21.40657	17.6041	20.6113	23.60665	17.29253	29.57816
AC237221.	344.9658	984.3842	33.97054	450.0138	259.1515	620.0446	848.8306	262.9917	60.69896	57.64196	53.00921	384.5927	359.8295	513.7576	358.9281	70.07841	23.60665	41.86614	56.4674
AC237221.	92.24712	76.02967	51.95495	44.68959	24.36467	58.23624	115.5565	26.85478	9.338302	8.646293	0	84.6472	97.66802	39.09025	26.40616	4.946711	1.026376	94.65388	87.83818
AC239367.	12.4918	7.002733	29.97401	12.47151	4.42994	14.84453	6.797442	15.74246	16.34203	16.33189	21.68559	11.96102	23.64594	5.584322	3.912023	11.54233	4.105505	12.74187	0.896308
AC239585.	1.921815	5.001952	2.997401	2.078586	1.107485	0	0	2.778081	2.334575	0	0	1.840156	0	0	0	0.824452	0	0.910133	0
AC239585.	63.4199	105.041	113.9012	123.6758	65.34161	103.9117	62.87634	87.04654	54.86252	100.8734	104.8137	90.16767	98.6961	73.5269	76.28445	37.92478	65.68808	31.85467	40.33386
AC239600.	17.29634	0	2.997401	11.43222	7.752394	2.283774	15.29424	6.482189	1.167288	0	6.023774	9.200782	9.252759	4.653602	7.824047	18.96239	4.105505	4.550667	3.585232
AC239600.	0	0	0	0	0	0	0	0	0	0	0	0	1.028084	0	0	0	1.026376	0	0
AC239727.	0	0	0	0	0	0	0	0	0	0	0	0	0	0	0.978006	0.824452	0	0	0.896308
AC239798.	9.609075	5.001952	3.996535	3.117878	17.71976	1.141887	2.549041	0	17.50932	5.764196	7.228529	3.680313	2.056169	10.23792	18.58211	48.64266	21.5539	5.4608	4.48154
AC239798.	163.3543	132.0515	253.78	333.613	189.3799	93.63473	197.1258	172.241	158.7511	155.6333	92.76613	405.7545	251.8807	288.5233	289.4897	824.4518	508.0562	726.2865	458.9097
AC239798.	0	0	0	0	0	0	0.84968	0	0	0	0	0	0	0.93072	0	0.824452	0	0	0
AC239799.	6.726353	0	3.996535	1.039293	4.42994	0	5.098081	8.334243	0	0	0	0	2.056169	1.861441	3.912023	4.122259	3.079129	3.640534	0.896308
AC239799.	4.804538	2.000781	57.94975	3.117878	3.322455	4.567548	6.797442	33.33697	2.334575	11.52839	0	11.96102	2.056169	6.515042	9.780058	23.08465	32.84404	12.74187	12.54831
AC239800.	1.921815	0	3.996535	3.117878	0	2.283774	0	0	0	1.921399	0	2.760235	0	0	3.912023	3.297807	1.026376	0	1.792616
AC239800.	79.75532	98.03826	115.8995	67.55403	110.7485	148.4453	37.38593	79.63833	154.082	145.0656	84.33284	115.0098	58.60081	112.6172	136.9208	145.1035	118.0333	139.2504	80.66772
AC239800.	0	4.001562	0	5.196464	2.21497	1.141887	0	0.926027	1.167288	0	2.40951	1.840156	1.028084	0	0.978006	0.824452	2.052753	1.820267	0.896308
AC239800.	0	2.000781	0	0	0	0	0	0	0	0	0	0	0	0	0	0	2.052753	0	0
AC239801.	0	0	0	0	0	0	0	0	0	0	0	9.200782	0	0	0	0	0	0.910133	0
AC239802.	0	0	0	0	0	0	0.84968	0	0	0	0	0	0	0.93072	0	0	0	0	0
AC239802.	0.960908	17.00664	21.98094	9.353635	15.50479	4.567548	18.69297	17.59451	0	18.25329	20.48083	14.72125	17.47743	18.61441	26.40616	14.84013	3.079129	5.4608	7.170464
AC239803.	2.882723	9.003514	4.995668	6.235757	7.752394	15.98642	20.39233	12.96438	0	0	0	11.04094	8.224675	12.09936	6.846041	2.473355	8.21101	8.191201	0.896308
AC239803.	0	0	0.999134	0	2.21497	0	3.398721	0.926027	1.167288	0.960699	0	0	0	0	1.956012	2.473355	1.026376	2.7304	3.585232
AC239803.	16.33543	36.01406	19.98267	40.53242	19.93473	28.54718	32.28785	20.37259	89.88116	9.606993	65.05676	31.28266	13.3651	22.33729	13.69208	39.57369	29.76491	13.652	14.34093
AC239804.	11.53089	21.0082	11.9896	38.45383	23.25718	12.56076	33.98721	53.70957	33.85134	5.764196	14.45706	21.1618	20.56169	8.376483	28.36217	5.771163	3.079129	20.93307	14.34093
AC239804.	0.960908	1.00039	0	0	1.107485	0	0.84968	0	0	0	0	1.840156	0	0	0	2.473355	13.34289	6.370934	0.896308
AC239809.	0	0	0	4.157171	0	0	0	0	3.501863	0	0	0	0	0	0	3.297807	0	0	0
AC239809.	17.29634	1.00039	5.994802	9.353635	8.859879	0	8.496802	3.704108	0	0	6.023774	7.360626	8.224675	18.61441	19.56012	48.64266	43.1078	6.370934	2.688924
AC239809.	293.0768	377.1472	242.7895	399.0884	249.1841	262.634	435.0363	225.9506	172.7586	202.7075	165.0514	320.1872	346.4644	370.4267	309.0498	371.8278	406.445	248.4664	360.3158
AC239859.	0.960908	0	5.994802	5.196464	12.18233	2.283774	5.947762	2.778081	3.501863	7.685594	2.40951	7.360626	5.140422	3.722881	2.934017	7.420066	2.052753	3.640534	13.44462
AC239859.	1.921815	22.00859	7.993069	12.47151	6.64491	1.141887	32.28785	6.482189	5.836439	4.803496	6.023774	11.04094	6.168506	13.9608	8.802052	4.946711	3.079129	6.370934	6.274156
AC239859.	0.960908	6.002343	1.998267	0	4.42994	0	4.248401	0	0	0	0	4.600391	1.028084	3.722881	3.912023	0.824452	1.026376	3.640534	1.792616
AC239859.	0	0	0	1.039293	0	2.283774	2.549041	0	0	1.921399	0	3.680313	0	0.93072	0.978006	0	0	0	3.585232
AC239859.	0	0	0.999134	1.039293	0	0	0	0	0	0	0	0	1.028084	0	0	0	0	0	0
AC239859.	122.0353	102.0398	188.8363	192.2692	140.6506	140.4521	164.838	165.7588	130.7362	121.0481	118.066	179.4153	130.5667	181.4905	133.9868	117.0722	180.6422	116.4971	117.4163
AC239860.	42.27993	81.03163	38.96621	28.0609	25.47215	85.64153	46.73241	29.63287	18.6766	26.89958	42.16642	36.80313	33.92678	16.75297	44.01026	29.68027	25.65941	21.8432	24.20031
AC239860.	22.10087	7.002733	7.993069	7.275049	5.537425	0	7.647122	0	0	7.685594	1.204755	3.680313	0	7.445762	2.934017	0.824452	6.158258	1.820267	0
AC239868.	13.45271	8.003124	8.992203	10.39293	14.3973	15.98642	7.647122	18.52054	5.836439	18.25329	8.433284	22.08188	20.56169	19.54513	14.67009	8.244518	4.105505	17.29253	17.92616
AC239868.	1377.941	1269.495	1901.351	944.7171	1305.725	2058.822	1184.454	1739.079	1049.392	1728.298	1405.949	1853.958	1705.592	1068.467	1241.089	1359.521	1166.99	1690.118	1407.203
AC239868.	0	0	0	0	0	0	0	0	0	0	0	0	0	0	0	0	0	1.820267	0
AC240504.	0.960908	0	0	0	0	0	0.84968	0	0	1.921399	0	0.920078	0	0	0	0.824452	0	0	0.896308
AC240565.	0	5.001952	0	8.314342	0	2.283774	34.83689	0	0	5.764196	2.40951	0	6.168506	3.722881	1.956012	4.122259	3.079129	9.101334	3.585232

AC240565.	5.765445	40.01562	23.97921	46.76817	40.97694	25.12151	110.4584	2.778081	43.18965	136.4193	186.737	23.92203	17.47743	43.74385	19.56012	84.91854	49.26606	119.2275	89.63079
AC241377.	32.67086	41.01601	27.97574	37.41454	182.735	25.12151	23.79105	53.70957	37.35321	27.86028	21.68559	55.20469	32.8987	158.2225	186.7991	295.9782	223.75	60.06881	25.99293
AC241377.	5.765445	16.00625	18.98354	25.98232	91.92125	33.11472	29.73881	0	9.338302	19.21399	26.50461	16.56141	19.5336	12.09936	17.6041	8.244518	15.39564	17.29253	19.71877
AC241520.	0	1.00039	0	0	14.3973	0	1.69936	0	0	0	0	0.920078	4.112337	3.722881	8.802052	0	1.026376	0.910133	1.792616
AC241584.	0.960908	5.001952	0	6.235757	0	7.993209	1.69936	0	0	0.960699	1.204755	3.680313	8.224675	0	0.978006	0.824452	1.026376	0.910133	0.896308
AC241584.	0	0	0	0	5.537425	0	2.549041	5.556162	1.167288	0	0	0.920078	0	0.93072	0.978006	1.648904	1.026376	0	0.896308
AC241585.	0.960908	9.003514	4.995668	12.47151	16.61227	6.851322	7.647122	7.408216	19.84389	15.37119	39.75691	12.8811	9.252759	16.75297	7.824047	9.893422	11.29014	23.66347	9.859387
AC241585.	564.0527	480.1874	392.6595	511.332	445.2089	702.2605	536.9979	525.0573	808.9304	577.3803	554.1872	680.8579	736.1084	482.1131	452.8167	636.4768	590.1663	562.4625	459.806
AC241585.	0	0	0	0	0	1.141887	0	0.926027	0	0.960699	2.40951	0	0	1.861441	0	1.648904	0	0.910133	0.896308
AC241585.	8.648168	0	0	1.039293	4.42994	3.425661	2.549041	0	10.50559	0	3.614265	2.760235	0	11.16864	1.956012	18.13794	14.36927	0	0.896308
AC241644.	0	0	0	0	4.42994	1.141887	0	0	0	0	0	0	0	0	2.934017	5.771163	0	0.910133	0
AC241952.	6071.975	7516.934	14669.28	8057.637	10977.39	8726.3	15266.2	8374.989	5494.423	8791.359	8423.646	10290.15	10736.29	8705.027	9789.838	4891.473	5065.167	6381.856	9116.348
AC242022.	0	0	0	0	0	1.141887	0	2.778081	0	6.724895	2.40951	2.760235	0	0.93072	0	0	4.105505	1.820267	0.896308
AC242376.	15.37452	1.00039	0.999134	4.157171	1.107485	1.141887	1.69936	0	0	0	0	19.32164	24.67402	2.792161	3.912023	5.771163	3.079129	17.29253	3.585232
AC242376.	1430.791	826.3225	491.5738	1203.501	450.7464	920.3609	994.9756	1046.411	240.4613	537.9916	519.2494	986.3239	991.0733	992.1478	1095.367	845.8876	929.8969	1017.529	950.0864
AC242426.	9.609075	9.003514	8.992203	19.74656	29.90209	2.283774	13.59488	17.59451	14.00745	13.44979	18.07132	16.56141	15.42127	34.43665	38.14223	18.13794	12.31652	5.4608	6.274156
AC242426.	380.5194	376.1468	396.6561	545.6287	281.3012	364.262	436.7356	337.9999	415.5544	393.8867	397.5691	370.7915	432.8235	371.3574	338.39	435.3106	465.9748	398.6384	371.9678
AC242498.	0.960908	2.000781	0.999134	0	1.107485	0	0	0.926027	0	0	0	0	3.084253	0	0.978006	0	0	1.820267	0.896308
AC242842.	14.41361	25.00976	27.97574	9.353635	33.22455	23.97963	15.29424	17.59451	50.19337	29.78168	118.066	23.92203	29.81445	18.61441	16.6261	9.893422	14.36927	31.85467	26.88924
AC242843.	45.16265	64.02499	47.95842	60.27898	42.08443	41.10793	98.56291	50.00546	29.18219	49.95636	27.70936	54.28462	61.68506	62.35826	56.72434	58.53608	63.63533	47.32694	52.88217
AC242852.	0	0	4.995668	3.117878	0	0	4.248401	1.852054	0	1.921399	1.204755	0.920078	3.084253	5.584322	4.890029	1.648904	2.052753	1.820267	6.274156
AC242988.	1.921815	15.00586	13.98787	7.275049	1.107485	11.41887	6.797442	5.556162	1.167288	16.33189	12.04755	0.920078	5.140422	16.75297	5.868035	1.648904	4.105505	8.191201	12.54831
AC243312.	0	3.001171	0	0	0	2.283774	0	0	0	0	0	0.920078	0	0.93072	0	1.648904	4.105505	0	0.896308
AC243428.	0	0	0	10.39293	1.107485	0	1.69936	0.926027	1.167288	0.960699	0	11.96102	1.028084	1.861441	0.978006	9.06897	0	6.370934	1.792616
AC243547.	0.960908	16.00625	9.991337	4.157171	6.64491	6.851322	19.54265	3.704108	16.34203	14.41049	6.023774	10.12086	2.056169	13.9608	20.53812	6.595615	10.26376	16.3824	4.48154
AC243562.	135.488	169.066	119.896	108.0864	218.1745	116.4725	192.8774	137.978	21.01118	185.415	299.984	232.7798	214.8696	161.0146	104.6466	149.2258	184.7477	205.6902	332.5302
AC243562.	1362.567	1140.445	1181.975	1393.692	1293.542	1083.651	1495.437	952.8818	916.3209	1237.381	1442.092	1603.696	1679.89	1210.867	1307.594	1285.32	1377.397	1575.441	1746.008
AC243562.	30.74904	48.01874	31.97228	54.04322	52.05179	44.53359	44.18337	37.96711	25.68033	43.23147	48.1902	35.88305	50.37613	49.32818	52.81231	36.27588	32.84404	35.4952	73.49725
AC243571.	0	0	0	4.157171	0	0	0	0	1.167288	0	0	0	0	0	0	4.122259	0	0	0
AC243571.	21.13997	25.00976	6.993936	21.82515	12.18233	13.70264	30.58849	0.926027	8.171014	15.37119	4.81902	9.200782	7.196591	13.03008	22.49413	24.73355	23.60665	8.191201	17.92616
AC243585.	0	0	0	1.039293	3.322455	0	0	0	0	0	0	3.680313	1.028084	1.861441	4.890029	0.824452	0	0.910133	0
AC243591.	0	0	0	0	0	0	0	13.89041	14.00745	0.960699	4.81902	0	0	0	0	0	6.158258	0	5.377848
AC243654.	0	0	0	0	1.107485	0	4.248401	6.482189	0	0.960699	0	1.840156	5.140422	6.515042	6.846041	0	2.052753	1.820267	0.896308
AC243654.	12.4918	23.00898	11.9896	23.90373	13.28982	9.135096	8.496802	50.93149	16.34203	13.44979	15.66181	16.56141	18.50552	26.99089	12.71408	12.36678	5.131881	44.59654	27.78555
AC243654.	22.10087	20.00781	10.99047	9.353635	7.752394	19.41208	24.64073	2.778081	0	24.01748	6.023774	8.280704	23.64594	9.307203	5.868035	14.01568	13.34289	5.4608	9.859387
AC243725.	2.882723	1.00039	3.996535	3.117878	14.3973	3.425661	5.098081	6.482189	1.167288	3.842797	8.433284	6.440548	14.39318	5.584322	2.934017	4.122259	1.026376	3.640534	2.688924
AC243732.	53.81082	67.02616	66.94196	53.00393	78.63143	55.95246	67.97442	91.67668	128.4017	116.2446	179.5085	108.5692	72.99399	86.55699	98.77859	75.02512	72.87271	95.56401	70.80833
AC243756.	1.921815	0	1.998267	3.117878	6.64491	6.851322	0	7.408216	14.00745	0.960699	14.45706	1.840156	0	8.376483	3.912023	4.946711	9.237386	0	0
AC243756.	50.9281	70.02733	82.92809	66.51474	94.13622	57.09435	56.0789	64.82189	25.68033	26.89958	18.07132	23.92203	30.84253	115.4093	91.93255	138.5079	117.0069	36.40534	15.23724
AC243772.	0	0	0	3.117878	0	0	0	0	0	0	0	0	1.028084	0	0	0	0	0	0
AC243772.	248.8751	62.02421	101.9116	127.833	67.55658	76.50643	103.661	105.5671	8.171014	96.06993	60.23774	141.692	69.90974	118.2015	75.30645	391.6146	346.9152	339.4798	186.4321
AC243772.	93.20803	31.0121	79.93069	110.165	151.7254	34.25661	52.68017	107.4191	58.36439	51.87776	49.39495	125.1306	71.96591	147.0538	201.4692	352.0409	235.0402	202.0496	108.4533
AC243773.	36.51449	0	0	0	0	0	0	0	0	3.842797	0	0	0	0	0	1.648904	1.026376	0	0.896308
AC243773.	1.921815	0	0	1.039293	1.107485	0	0	0	0	0.960699	0	3.680313	3.084253	0	0.978006	2.473355	7.184634	0	0
AC243829.	17.29634	5.001952	38.96621	41.57171	0	38.82416	36.53625	21.29862	0	21.13538	7.228529	75.44642	35.98295	15.82225	2.934017	27.20691	22.58028	0	0
AC243829.	4.804538	6.002343	4.995668	3.117878	4.42994	1.141887	2.549041	4.630135	3.501863	3.842797	3.614265	10.12086	1.028084	1.861441	2.934017	3.297807	3.079129	6.370934	3.585232
[truncated: 981,810 more chars]
